# An Observation Medicine Curriculum for Emergency Medicine Education

**DOI:** 10.21980/J87P92

**Published:** 2021-04-19

**Authors:** Danie Ty, Marney Gruber, Noah Klein, Jeremy Branzetti, Matthew Brown, Matthew McCarty, Tracy Svetcov, Barie Miller, Maurice Hinson, Raj Machhar, Sharon Uralil, Catherine Capo, Yitzchak Weinberger, Melanie Raffoul, Robert Femia, Christopher Caspers

**Affiliations:** * New York University Langone Medical Center, Ronald O. Perelman Department of Emergency Medicine, New York, NY

## Abstract

**Audience and type of curriculum:**

This curriculum, designed and implemented at the Ronald O. Perelman Department of Emergency Medicine at NYU Langone Health, primarily targets third- and fourth-year emergency medicine (EM) residents, and is an immersive observation medicine rotation that can be integrated into existing emergency medicine residency training.

**Length of curriculum:**

The curriculum is designed for a dedicated rotation of two weeks for senior residents and can be expanded to 4 weeks.

**Introduction:**

Observation medicine is an extension of emergency medicine and is increasingly playing a role in the delivery of acute healthcare, with over half of all observation units (OUs) in the nation being led by emergency medicine.^1^ Despite this, many emergency medicine residencies have yet to establish a formal observation medicine curriculum. In a 2002 study by Mace and Shah, only 10% of emergency medicine residencies had a dedicated observation medicine rotation, despite 85% of emergency medicine residency directors believing this was an important part of emergency medicine training.^2^ The first description of a model longitudinal observation medicine curriculum did not appear until 2016.^3^ In order to prepare our graduates for the evolving demands of the EM workplace, we must provide diverse educational experiences that train and showcase the expanding skill set of future emergency physicians.

**Educational Goals:**

The primary goal of this observation medicine curriculum is to train current EM residents in short-term acute care beyond the initial ED visit. This entails caring for patients from the time of their arrival to the OU to the point when a final disposition from the OU is determined, be it inpatient admission or discharge to home.

**Educational Methods:**

The educational strategies used in this curriculum include experiential learning through supervised direct patient care, independent learning based on prescribed literature, and didactic teaching.

**Research Methods:**

Education content was evaluated by the learners through pre- and post-rotation surveys, as well as written attending evaluations describing the progress of the learners during the rotation.

**Results:**

All residents reported increases in the confidence of their abilities to perform observation care.

**Discussion:**

Observation medicine is an increasingly vital aspect of emergency medicine, but education in observation medicine has not developed in tandem with its implementation. A lack of observation medicine training represents a missed opportunity for each trainee to gain a robust understanding of the interface between inpatient and outpatient care, and how to arrive at the most appropriate disposition for ED patients. Considering the wide breadth of clinical conditions managed in OUs and the variability of OU management at various learning sites, the curriculum must be tailored to the specific unit to maximize effectiveness of the learning experience.

**Topics:**

Observation medicine, curriculum, education, clinical rotation.

## USER GUIDE

**Table t1-jetem-6-2-c1:** 

**List of Resources:**	
Abstract	1
User Guide	3
Didactics and Hands on Curriculum Chart	9
Appendix A: Pre- and Post-Rotation Survey Results	37
Appendix B: Attending End-of-Shift Resident Evaluation	41
Observation Curriculum Lectures	46


[Table t1-jetem-6-2-c1]

**Learner Audience:**

Senior Residents

**Length of Curriculum:**

This curriculum is designed to last 2 weeks and can be expanded to 4 weeks if a longer duration is desired.

**Topics:**

Observation medicine, curriculum, education, clinical rotation.

**Objectives:**

By the end of this curriculum:

1. Residents will gain a fundamental understanding of observation care. Gain familiarity with respiratory pathophysiology and interventions unique to pediatric populations.
2. Residents will learn the unique systems-based practice (SBP) differences of observation medicine relevant to emergency medicine.
3. Residents will learn how to complete required documentation for observation care.

### Brief introduction

Observation medicine is an increasingly vital aspect of emergency medicine, but education in observation medicine has not developed in tandem with its implementation. More than half of all observation units are led by the emergency department, though many residencies have yet to establish a formal observation medicine curriculum.^1^ In a 2002 study by Mace and Shah, only 10% of emergency medicine residencies had a dedicated observation medicine rotation, despite 85% of emergency medicine residency directors believing this was an important part of emergency medicine training.^2^ The first description of a model longitudinal observation medicine curriculum did not appear until 2016. EM residents require training in observation medicine to be appropriately prepared for the workforce.

### Problem identification, general and targeted needs assessment

Most current EM residents are not trained in observation medicine and lack the necessary skills to successfully manage patients dispositioned to an observation unit. These graduates face a competitive disadvantage in the job market relative to their peers who have had formal training, since most observation units are administered by the hospital’s emergency department. Additionally, a lack of observation medicine training represents a missed opportunity for each trainee to gain a robust understanding of the interface between inpatient and outpatient care, and how to arrive at the most appropriate disposition for ED patients.

In response, we present an observation medicine curriculum that was developed and implemented at the Ronald O. Perelman Department of Emergency Medicine at NYU Langone Health (New York City, New York). The study setting represents a large, urban, academic health system. Observation medicine is administered by the emergency department at NYU Langone Health via a traditional Type 1, protocol-driven, dedicated unit.^4^ This curriculum synthesizes design aspects of Kern’s “Curriculum Development for Medical Education: a Six Step Approach” and Wheatley et al’s “A model longitudinal observation medicine curriculum for an emergency medicine residency.”^3^,^5^ This curriculum is differentiated from Wheatley’s approach in that this curriculum features a rotational training experience of 2–4 weeks, similar to how an emergency medicine resident would rotate through an intensive care unit. For some programs, this may offer the advantage of an initial, immersive training experience in a finite period as opposed to a longitudinal experience over the full duration of emergency medicine training. Additionally, this could serve as a new rotation for programs looking to replace other rotations of lesser value. For those residents who desire ongoing observation unit shifts after the rotation is completed, future OU shifts can be intermixed with ED shifts.

### Goals of the curriculum

The primary goal of this observation medicine curriculum is to train current EM residents in short-term acute care beyond the initial ED visit. This entails caring for patients from the time of their arrival to the OU to the point when a final disposition from the OU is determined, be it inpatient admission or discharge to home. The secondary goal of this curriculum is to equip graduating senior residents with the skills necessary to care for ED patients requiring observation care.

### Objectives of the curriculum

The objectives of the observation medicine curriculum and the individualized sub-goals are presented below:

Objective 1: Residents will gain a fundamental understanding of observation care.

Residents will learn how to apply patient selection criteria to identify ED patients appropriate for OU care, perform initial OU arrival assessments, create an OU-appropriate management plan, and apply evidence-based, protocolized care to OU patients based on protocol-specific inclusion and exclusion criteria. Residents will also learn how to tailor ongoing patient care according to reassessments, select the appropriate disposition plan based on predetermined OU disposition goals, and coordinate post-discharge plans with a multi-disciplinary team of social workers, physical and occupational therapists, care managers and home health service liaisons to execute the disposition plan.

Residents will learn how to manage the following OU clinical conditions using evidence-based, protocolized care. The selected conditions were derived from a consensus decision by the authors based on our internally used observation protocols.

1. Acute decompensated heart failure
2. Acute kidney injury
3. Abdominal pain
4. Alcohol withdrawal
5. Allergic reaction
6. Asthma
7. Atrial Arrhythmia
8. Back Pain
9. Cellulitis
10. Chest pain
11. Coronary catheterization
12. Coronary CT angiography
13. Colitis
14. Dehydration
15. Delirium
16. Deep vein thrombosis
17. Fever
18. Gastrointestinal bleed
19. Geriatric
20. Headache
21. Head injury
22. Hyperglycemia
23. Hypoglycemia
24. Kidney stone
25. Metabolic derangement
26. Ovarian torsion
27. Pancreatitis
28. Peripheral vertigo
29. Pulmonary Embolism (PE)
30. Pneumonia
31. Rib fracture
32. Seizure
33. Sickle cell pain
34. Syncope
35. Transfusion
36. Transient Ischemic Attack (TIA)
37. Urinary tract infection

Objective 2: Residents will learn the unique systems-based practice (SBP) differences of observation medicine relevant to emergency medicine.

The observation medicine rotation is structured to provide residents the opportunity to learn the key skills necessary to deliver the various stages of observation care. The resident will accept OU patient placement from the ED, complete the initial evaluation of new OU patients, and implement initial management steps such as protocol implementation and care coordination. Residents will learn ongoing management through periodic reassessments and actively managing the patient towards disposition endpoints. The resident will learn the nuances of effective disposition planning such as evaluating and resolving clinical and/or psychosocial discharge barriers through interdisciplinary collaboration with case managers, social workers, and home service liaisons. Residents will also learn distinguishing operational characteristics of observation units in the lectures entitled “Overview of Observation Medicine,” “Systems-based Practices Relevant to Observation Medicine,” and “Observation Documentation.”

Shift timing and length will depend on the logistics of each observation unit. For example, a larger unit with more robust provider staffing may allow for resident rotation at different shift times. In the presented curriculum, resident shifts are divided into two eight-hour shifts occurring over a twenty-four period (0800–1600; 1601–2359), each with unique workflows and complementary aspects of patient care. Actual shift duration and timing should be tailored to the individual observation unit staffing at the clinical site.

During the shift occurring from 0800–1600, the resident will be assigned to a teaching OU attending and participate in multidisciplinary handoff from the overnight team followed by morning rounds. The OU attending will identify which patients provide valuable clinical experiences and assign them to the resident.

During morning rounds, the resident will formulate a management plan that simultaneously addresses diagnostics and therapeutic strategies, coordinates care with consultants and allied health professionals, evaluates clinical progression, and develops an effective sign out at shift change. Specifically, residents will learn how to conduct efficient, team-based care during rounding. Residents will interface with nursing, social work, pharmacy, and case management and will participate in team-based rounds to comprehensively address the needs of their assigned patients. Each resident will develop an understanding of the social determinants of health and how they impact disposition planning and outpatient care.

For the 1600–2359 shift, the resident will begin by attending sign out from the morning OU team. The resident will reassess these patients with the evening OU attending and progress the patient’s management throughout the evening. During the remainder of the shift, the resident will mainly focus on accepting new patients from the ED. For each new patient, the resident will perform a verbal handoff on new patient placements from ED providers and summarize the intended observation care plan at the conclusion of the handoff conversation. They will formulate a complete management plan following the initial OU evaluation based on the verbal hand off from the ED, chart review, clinical evaluation, and discussion with the observation attending. Residents will use the electronic medical record (EMR) to document and place orders via order sets specific to observation care consistent with evidence-based clinical protocols. The resident will then execute the initial plan per protocol. The resident will engage in direct patient care supervised by a dedicated attending at all times.

Objective 3: Residents will learn how to complete required documentation for observation care.

The resident will learn how to complete the required documentation for observation care, such as the initial observation history and physical (H&P) with attention to OU anticipated care, the history of present illness (HPI), the complete past medical, surgical, social and family histories, a 10-point review of systems, code status documentation, medication reconciliation, and goals of care. The H&P will describe the intended care plan, the indication for observation, as well as clinical end points required for disposition.

Residents will learn to complete a daily progress note for patients following morning rounding that documents the need for ongoing observation care and relevant clinical events in a standard SOAP (subjective, objective, asessment, plan) note format. Residents will learn to complete an observation discharge narrative, which includes a summary of the observation hospital course, a final physical exam, and post-discharge plan, including follow-up information, discharge instructions, and discharge medication reconciliation.

### Educational Strategies

The resident will complete a rotation of at least two weeks and the rotation may be expanded to 4 weeks depending on the desired duration and intensity of the learning experience. After this introductory rotation, future OU shifts can be scheduled among ED shifts based on the interest of the residency program. The OU rotation should accommodate the number of residents that can be adequately supervised by the attending physician at a single time. Generally, this number will be 1–2 residents, but will also depend on the clinical complexity and size of the unit. All rotations should be coordinated by educational and operational leadership to ensure an effective learning experience. The resident will work five weekday 8-hour shifts each week. Residents should not be on back-up call during the rotation or have other service obligations in order to maximize the learning experience.

Prior to beginning the OU rotation, residents will be provided with literature to establish an initial knowledge base for the successful practice of observation medicine. The reading list represents a compilation of relevant peer-reviewed articles focusing on the management of common conditions seen in the OU, such as: chest pain, syncope, atrial fibrillation, transient ischemic attack (TIA), heart failure, infectious processes (ie, pneumonia, cellulitis, urinary tract infection), conditions associated with acute pain (ie, back pain, sickle cell crisis, headache), chronic obstructive pulmonary disease (COPD), asthma, alcohol withdrawal, and other conditions commonly managed in OUs. Suggested readings are included in this manuscript following the references. The resident should initially select relevant readings from this list throughout the rotation as they relate to patient care.

During the beginning of the rotation, the resident should manage patients that represent a relatively simple level of observation complexity (ie, chest pain, cellulitis, syncope, etc.), which will transition to more complex observation management (ie, heart failure, TIA, seizure, mild alcohol withdrawal, non-op fracture, complex psychosocial cases, a diagnostically ambiguous case, a clinically evolving patient, etc.) as the rotation progresses. The selection of appropriate patients should be at the discretion of the supervising attending.

Residents will receive regular, focused didactic teaching in the OU that is relevant to clinical conditions that they are managing. Each teaching will take approximately 10 to 15 minutes during the clinical shift and will review the evidence base for the protocolized OU care of a patient in the OU. Teaching will take the form of didactic presentations prepared in advance and correspond to the clinical protocols used most frequently in the OU. Approximately 1–2 teachings can occur per shift. These focused didactics should relate to patients the resident is currently caring for to facilitate rapid translation of knowledge to clinical care. The number of lecture examples provided in the curriculum are vast and should be prioritized based on relevance to patient care at that time. Additionally, the resident should conduct independent study and is expected to read relevant literature beyond shift times. Residents are also encouraged to participate in ongoing quality improvement projects in the OU. Residents may identify an area for future study and/or research in the OU. Of note, they will continue to attend the dedicated weekly EM didactic conference per their core curriculum and mandated by the Accreditation Council for Graduate Medical Education (ACGME) Emergency Medicine program requirements.^6^,^7^

Residents will be evaluated on professionalism, communication skills, history, physical exam, ability to formulate and execute a plan of care, and ability to work as an integral part of a multidisciplinary team. Residents will be evaluated using the standard evaluation system currently in use at their residency program. OU attendings will also complete an evaluation of the resident at the end of each shift and provide direct formative verbal feedback to the resident. Resident evaluation content will be based on a selection of the EM Milestones identified by Wheatley et al, that have been determined by the authors as relevant to their observation medicine rotation.^5^ At the end of the rotation, the rotation directors will review all evaluations and provide summative feedback of the resident to the residency program director.

### Results and tips for successful implementation: Implementation

An online pre-rotation survey should be implemented to assess the resident’s perceptions of his or her comfort practicing observation medicine, understanding of the role of observation medicine in acute care, and the role of observation medicine in the management of ED patients. This survey can be conducted both before and after the rotation to compare responses and evaluate efficacy of the rotation towards meeting the above objectives. As an example, this specific curriculum has been piloted with three senior EM residents thus far. All residents reported increases in the confidence of their abilities to perform observation care. De-identified survey results and comments from the residents are included in the appendix.

Considering the wide breadth of clinical conditions managed in OUs and the variability of OU management at various learning sites, the curriculum must be tailored to the specific unit to maximize effectiveness of the learning experience. It is key for representatives from both the educational and clinical operations teams to collaborate in the development and implementation of the OU experience, as well as having an ongoing process for collecting resident feedback of the rotation in the OU. This will allow optimization of the learning experience as the rotation matures and/or the OU evolves clinically.

### Evaluation and Feedback

In the development and optimization of this curriculum, seeking direct, face-to-face feedback from the residents was extremely helpful in collecting rich, detailed information about the rotation. The curriculum was modified based on learner feedback: the prepared materials for didactic teaching were expanded, the number of clinical preceptors were increased, the resident’s role as an advanced practice provider (APP) supervisor was created, and the curriculum was adjusted. This also provided an opportunity for residents to increase their patient care load if they believe it to be useful towards reaching their educational goals.

### Appendices

- Appendix A Pre- and Post-Rotation Survey Results
- Appendix B Attending End-of-Shift Resident Evaluation

### Associated Contents

1. Observation Curriculum Cellulitis Lecture
2. Observation Curriculum Chest Pain Lecture
3. Observation Curriculum DVT and PE Lecture
4. Observation Curriculum GI Bleed Lecture
5. Observation Curriculum Headache Lecture
6. Observation Curriculum Sickle Cell Lecture
7. Observation Curriculum asthma Lecture
8. Observation Curriculum Abdominal Pain Lecture
9. Observation Curriculum Atrial Fibrillation Lecture
10. Observation Curriculum Dehydration Lecture
11. Observation Curriculum Heart Failure Lecture
12. Observation Curriculum Nephrolithiasis Lecture
13. Observation Curriculum Ovarian Torsion Lecture
14. Observation Curriculum Overview of Obs Med Lecture
15. Observation Curriculum Pancreatitis Lecture
16. Observation Curriculum Pneumonia Lecture
17. Observation Curriculum Rib Fracture Lecture
18. Observation Curriculum Transfusion Lecture
19. Observation Curriculum UTI Lecture
20. Observation Curriculum Vertigo Lecture
21. Observation Curriculum Allergic Reaction Lecture
22. Observation Curriculum Back Pain Lecture
23. Observation Curriculum Hyper & Hypoglycemia Lecture
24. Observation Curriculum Geriatric Frailty Lecture
25. Observation Curriculum Management of Alcohol Withdrawal Lecture
26. Observation Curriculum Seizures Lecture
27. Observation Curriculum TIA Lecture

References/Further Readings1

WilerJL
RossMA
GindeAA

National study of emergency department observation services
Acad Emerg Med
2011
18
959
965
2188363810.1111/j.1553-2712.2011.01151.x2

MaceSE
ShahJ

Observation medicine in emergency medicine residency programs
Acad Emerg Med
2002
9
169
71
1182584510.1111/j.1553-2712.2002.tb00236.x3

ThomasP
KernD
HughesM
ChenB

Curriculum Development for Medical Education: A Six-Step Approach
3rd ed
Baltimore, MD
Johns Hopkins University Press
2015
4

RossM
HockenberryJ
MutterR

Protocol-Driven Emergency Department Observation Units Offer Savings, Shorter Stays, And Reduced Admissions
Health Affairs
December
2013
10.1377/hlthaff.2013.0662243013995

WheatleyM
BaughC
OsborneA
ClarkC
ShayneP
RossM

A model longitudinal observation medicine curriculum for an emergency medicine residency
Acad Emerg Med
2016
23
4
482
92
2680666410.1111/acem.129096
Accreditation Council for Graduate Medical Education
ACGME Program Requirements for Graduate Medical Education
www.acgme.org/Portals/0/PFAssets/ProgramRequirements/110_emergency_medicine_2017-0701.pdf Revised February 6, 2017 Effective July 1, 2017
Accessed August 28, 2018
7
Accreditation Council for Graduate Medical Education
Emergency Medicine Milestones: Revised February 2021
Accessed March 13, 2021
Effective July 1, 2021https://www.acgme.org/Portals/0/PDFs/Milestones/EmergencyMedicineMilestones2.0.pdf?ver=2021-02-24-104718-043


### Background

- Wilkerson RG, Baugh C, Mattu A. Observation Medicine; Emergency Medicine Clinics of North America; vol 35-3; 1^st^ edition; July 2017.

### Atrial Arrhythmia

- Lloyd-Jones D, Adams RJ, Brown TM, et al. Heart disease and stroke statistics--2010 update: a report from the American Heart Association. *Circulation*. 2010;121(7): e46–e215.
- Gage BF, Waterman AD, Shannon W, Boechler M, Rich MW, Radford MJ; Validation of clinical classification schemes for predicting stroke: results from the National Registry of Atrial Fibrillation. *JAMA*. 2001;285(22):2864–2870
- Friberg L, Rosenqvist M, Lip GY. Evaluation of risk stratification schemes for ischaemic stroke and bleeding in 182 678 patients with atrial fibrillation: the Swedish Atrial Fibrillation cohort study. *Eur Heart J*. 2012 Jun;33(12):1500–10. Epub 2012 Jan 13. PMID: 22246443. doi:10.1093/eurheartj/ehr488
- Pisters R, Lane DA, Nieuwlaat R, de Vos CB, Crijns HJ, Lip GY. A novel user-friendly score (HAS-BLED) to assess 1-year risk of major bleeding in patients with atrial fibrillation: The Euro Heart Survey. *Chest*. 2010; 138(5):1093–1100.
- Al-Khatib SM, Lapointe NA, Chatterjee R, et al. Treatment of Atrial Fibrillation [Internet]. Rockville (MD): Agency for Healthcare Research and Quality (US); 2013 Jun. (Comparative Effectiveness Reviews, No. 119.) Available from: https://www.ncbi.nlm.nih.gov/books/NBK153118/
- January CT, Wann LS, Alpert JS, et al. 2014 AHA/ACC/HRS guideline for the management of patients with atrial fibrillation: a report of the American College of Cardiology/American Heart Association Task Force on Practice Guidelines and the Heart Rhythm Society. *J Am Coll Cardiol*. 2014; 64(21):e1–76.

### Chest Pain

- Kline JA, Zeitouni RA, Hernandez-Nino J, Jones AE. Randomized trial of computerized quantitative pretest probability in low-risk chest pain patients: effect on safety and resource use. *Ann Emerg Med* 2009; 53:727–35.
- Than M, Herbert M, Flaws D, et al. What is an acceptable risk of major adverse cardiac event in chest pain patients soon after discharge from the emergency department? *Int J Cardiol*. 2013; 166:752–4
- Foy AJ, Liu G, Davidson WR Jr, Sciamanna C, Leslie DL, Comparative effectiveness of diagnostic testing strategies in emergency department patients with chest pain: an analysis of downstream testing, interventions, and outcomes; *JAMA Intern Med*. 2015 Mar;175(3):428–36
- Asher E, Reuveni H, Shlomo N. Clinical outcomes and cost effectiveness of accelerated diagnostic protocol in a chest pain center compared with routine care of patients with chest pain; PLoS One. 2015 Jan 26;10(1):e0117287
- Six AJ, Cullen L, Backus BE, et al. The HEART score for the assessment of patients with chest pain in the emergency department: a multinational validation study. *Crit Pathw Cardiol*. 2013; 12(3):121–126.
- Mahler SA, Riley RF, Hiestand BC, et al. The HEART Pathway Randomized Trial: Identifying Emergency Department Patients with Acute Chest Pain for Early Discharge. Circulation Cardiovascular quality and outcomes. 2015;8(2):195–203. doi:10.1161/CIRCOUTCOMES.114.001384
- Than M, Flaws D, Sanders S, et al. Development and validation of the Emergency Department Assessment of Chest pain Score and 2 h accelerated diagnostic protocol. *Emerg Med Australas*. 2014 Feb;26(1):34–44.
- Cullen L, Greenslade JH, Than M, et al. The new Vancouver Chest Pain Rule using troponin as the only biomarker: an external validation study. *Am J Emerg Med*. 2014 Feb;32(2):129–34.

### Acute Congestive Heart Failure

- Felker GM, Lee KL, Bull DA et al. Diuretic Strategies in Patients with Acute Decompensated Heart Failure. *N Engl J Med*. 2011; 364:797–805.
- Carubelli V, Metra M, Lund LH. Negotiating renal dysfunction when treating patients with heart failure. *Expert Review of Cardiovascular Therapy*. 2018;16(2):113–122.
- Collins SP, **Pang PS**, Fonarow GC, Yancy CW, Bonow RO, Gheorghiade M. Is Hospital Admission for Heart Failure Really Necessary? The Role of the ED and Observation Unit in Preventing Hospitalization and Rehospitalization. *J Am Coll Cardiol*. 2013 Jan 15; 61(2): 121–126.

### Geriatric

- Southerland LT, Vargas AJ, Nagaraj L, Gure TR, Caterino JM. An Emergency Department Observation Unit Is a Feasible Setting for Multidisciplinary Geriatric Assessments in Compliance with the Geriatric Emergency Department Guidelines. *Acad Emerg Med*. 2018 Jan;25(1):76–82.

### Syncope

- Patel PR, Quinn JV. Syncope: a review of emergency department management and disposition. *Clin Exp Emerg Med*. 2015 Jun; 2(2): 67–74.
- Kessler C, Tristano JM, De Lorenzo R. The emergency department approach to syncope: evidence-based guidelines and prediction rules; *Emerg Med Clin North Am*. 2010 Aug;28(3):487–500.

### Transient Ischemic Attack / Non-Debilitating Stroke

- Sehatzadeh S. Is Transient Ischemic Attack a Medical Emergency? An Evidence-Based Analysis. *Ont Health Technol Assess Ser*. 2015 Feb 1;15(3):1–45. PMID: 26355823; PMCID: PMC4558772.
- Nahab F, Leach G, Kingston C. Impact of an emergency department observation unit transient ischemic attack protocol on length of stay and cost. *J Stroke Cerebrovasc Dis*. 2012 Nov;21(8):673–8.
- Ross MA, Compton S, Medado P, Fitzgerald M, Kilanowski P, O’Neil BJ. An emergency department diagnostic protocol for patients with transient ischemic attack: a randomized controlled trial; *Ann Emerg Med*. 2007 Aug;50(2):109–19.
- Ong ME, Chan YH, Lin WP, Chung WL. Validating the ABCD^2^ Score for predicting stroke risk after transient ischemic attack in the ED. *Am J Emerg Med*. 2010 Jan;28(1):44–8.

## Figures and Tables

**Table t2-jetem-6-2-c1:** 

Topic	Recommended Educational Strategy	Educational Content	Objectives	Learners	Timing, Resources Needed (Space, Instructors, Equipment, citations of JETem pubs or other literature)	Recommended Assessment, Milestones Addressed (based on EM Milestones 2.0)^7^
Overview of Observation Medicine	Brief lecture describing an overview of Observation Medicine	-Definition of observation medicine -Benefits of observation care -Distinction between an ED, observation, and inpatient care -Observation service delivery models -Benefits of protocolized care	Learners will understand: -Characteristics and benefits of a dedicated, type 1 observation unit -Appropriate patient selection for observation care -How to incorporate evidence-based clinical protocols into observation care	PGY-3, PGY-4	20 minutes (lecture) Instructors: 1 Equipment: PowerPoint (and projector/screen)	Milestone: -SBP 4: Physician Role in the Healthcare System (all milestones) Assessment: End of rotation written assessment
Abdominal Pain	Brief lecture on the management of abdominal pain	-Epidemiology of abdominal pain -Differential diagnosis of abdominal pain -Appropriate diagnostic testing for patients presenting abdominal pain -Evidence supporting observation care for abdominal pain -Indications for consultation (ie, GI, Surgery, Gyn, etc.) -Pharmacological agents for treating abdominal pain	Learners will understand: -Benefits of protocolized observation care for patients with abdominal pain -How to reassess patients with negative or equivocal initial ED diagnostic studies -Indications and contraindications for subsequent testing -Which pharmacologic agents to manage abdominal pain and when to use each	PGY-3, PGY-4	20 minutes (lecture) Instructors: 1 Equipment: PowerPoint (and projector/screen)	Milestones: -MK 2: Treatment and Clinical Reasoning (all milestones) -SBP 3: System Navigation for Patient-Centered Care (milestones spanning levels 1–4, excluding community health themed milestones) -PBLI 1: Evidence-Based and Informed Practice (all milestones) Assessment: End of rotation written assessment
Alcohol withdrawal	Brief lecture on the management of alcohol withdrawal	-Epidemiology of alcohol withdrawal -Detecting etiology of alcohol withdrawal -Appropriate risk stratification for patients with alcohol withdrawal -Evidence supporting pharmacologic agents to manage withdrawal -Indications for consultation (ie, toxicology, poison control center, etc.) -Parameters for disposition end points	Learners will understand: -Appropriate patient selection for OU management of alcohol withdrawal -Benefits of protocolized observation care for patients with alcohol withdrawal -How to protocolize symptom-triggered therapy to manage withdrawal -How frequently to reassess patients and determine response to therapy -Indications and contraindications to pharmacologic agents commonly use to manage withdrawal -Effective disposition planning and outpatient follow up	PGY-3, PGY-4	20 minutes (lecture) Instructors: 1 Equipment: PowerPoint (and projector/screen)	Milestones: -MK 2: Treatment and Clinical Reasoning (all milestones) -SBP 3: System Navigation for Patient-Centered Care (milestones spanning levels 1–4, excluding community health themed milestones) -PBLI 1: Evidence-Based and Informed Practice (all milestones) Assessment: End of rotation written assessment
Allergic Reaction	Brief lecture on the management of allergic reactions	-Epidemiology of allergic reactions -Defining different types of allergic reactions -Appropriate risk stratification for patients with an acute allergic reaction -Evidence supporting pharmacologic agents to manage the reaction -Parameters for disposition end points	Learners will understand: -Appropriate patient selection for OU management of allergic reactions -How frequently to reassess patients and determine response to therapy -Indications and contraindications to pharmacologic agents commonly used to manage the reaction -Effective disposition planning and outpatient follow up	PGY-3, PGY-4	20 minutes (lecture) Instructors: 1 Equipment: PowerPoint (and projector/screen)	Milestones: -MK 2: Treatment and Clinical Reasoning (all milestones) -SBP 3: System Navigation for Patient-Centered Care (milestones spanning levels 1–4, excluding community health themed milestones) -PBLI 1: Evidence-Based and Informed Practice (all milestones) Assessment: End of rotation written assessment
Asthma	Brief lecture on the management of asthma	-Epidemiology of asthma -Detecting etiology of asthma exacerbations -Appropriate risk stratification for patients’ acute asthma exacerbations -Evidence supporting pharmacologic agents to asthma -Indications for admission to a monitored setting -Parameters for disposition end points	Learners will understand: -Appropriate patient selection for OU management of asthma exacerbations -Benefits of protocolized observation care for patients with asthma exacerbations -How to protocolize symptom-triggered therapy to asthma exacerbations -How frequently to reassess patients and determine response to therapy -Indications and contraindications to pharmacologic agents commonly used for asthma -Effective disposition planning and outpatient follow up	PGY-3, PGY-4	20 minutes (lecture) Instructors: 1 Equipment: PowerPoint (and projector/screen)	Milestones: -MK 2: Treatment and Clinical Reasoning (all milestones) -SBP 3: System Navigation for Patient-Centered Care (milestones spanning levels 1–4, excluding community health themed milestones) -PBLI 1: Evidence-Based and Informed Practice (all milestones) Assessment: End of rotation written assessment
Atrial Fibrillation	Brief lecture on the management of atrial fibrillation	-Epidemiology -Reversible and non-reversible causes of atrial fibrillation -Characterizing atrial fibrillation -Determining stroke risk -Risks and benefits of different anticoagulant medications -Rate versus rhythm control	Learners will understand: -The epidemiology of atrial fibrillation -How to address reversible and non-reversible causes of atrial fibrillation -How to characterize atrial fibrillation -Risks and benefits of different anticoagulant medications -How to select rate and rhythm control -When to utilize a cardiology consult	PGY-3, PGY-4	20 minutes (lecture) Instructors: 1 Equipment: PowerPoint (and projector/screen)	Milestones: -MK 2: Treatment and Clinical Reasoning (all milestones) -SBP 3: System Navigation for Patient-Centered Care (milestones spanning levels 1–4, excluding community health themed milestones) -PBLI 1: Evidence-Based and Informed Practice (all milestones) Assessment: End of rotation written assessment
Back Pain	Brief lecture on the management of back pain	-Epidemiology of back pain -Detecting emergent etiologies of back pain -Indications for diagnostic imaging -Evidence supporting pharmacologic agents to treat pain -Indications for physical therapy -Parameters for disposition end points	Learners will understand: -Appropriate patient selection for OU management of back pain -Benefits of pharmacologic therapy for symptom control -How frequently to reassess patients and determine response to therapy -Effective disposition planning and outpatient follow up	PGY-3, PGY-4	20 minutes (lecture) Instructors: 1 Equipment: PowerPoint (and projector/screen)	Milestones: -MK 2: Treatment and Clinical Reasoning (all milestones) -SBP 3: System Navigation for Patient-Centered Care (milestones spanning levels 1–4, excluding community health themed milestones) -PBLI 1: Evidence-Based and Informed Practice (all milestones) Assessment: End of rotation written assessment
Cellulitis	Brief lecture on management of cellulitis	-Causes of cellulitis -Typical presentations of cellulitis -Appropriate treatment regimens -Potential complications associated with cellulitis -Differential diagnosis for cellulitis	Learners will understand: -Typical bacterial causes of cellulitis, as well as unique scenarios -Common antimicrobial treatments regimens -How to identify complications -How to arrange a discharge and follow up plan	PGY-3, PGY-4	20 minutes (lecture) Instructors: 1 Equipment: PowerPoint (and projector/screen)	Milestones: -MK 2: Treatment and Clinical Reasoning (all milestones) -SBP 3: System Navigation for Patient-Centered Care (milestones spanning levels 1–4, excluding community health themed milestones) -PBLI 1: Evidence-Based and Informed Practice (all milestones) Assessment: End of rotation written assessment
Chest Pain	Brief lecture on managing patients presenting to the ED with chest pain	-Epidemiology of chest pain -Differential diagnosis of chest pain -Appropriate diagnostic testing for patients presenting with chest pain -Evidence supporting observation care for chest pain patients -Risk stratification tools for chest pain (HEART, TIMI, EDACS, Vancouver Chest Pain) -Absolute and relative contraindication s for stress testing -Pharmacological agents for treating chest pain	Learners will understand: -Benefits of protocolized observation care for chest pain patients -How to incorporate risk stratification tools for chest pain -Indications and contraindications for noninvasive testing -Which pharmacologic agents to manage chest pain and when to use each	PGY-3, PGY-4	20 minutes (lecture) Instructors: 1 Equipment: PowerPoint (and projector/screen)	Milestones: -MK 2: Treatment and Clinical Reasoning (all milestones) -SBP 3: System Navigation for Patient-Centered Care (milestones spanning levels 1–4, excluding community health themed milestones) -PBLI 1: Evidence-Based and Informed Practice (all milestones) Assessment: End of rotation written assessment
Decompensated Heart Failure	Brief lecture on managing patients presenting with acute decompensated heart failure	-Definition and classification of heart failure -Causes of heart failure -Diagnostic tests for heart failure -Treatment for heart failure -Evidence for diuretic strategies in acute decompensated heart failure	By the end of this lecture, learners will understand: -The pathophysiology of heart failure -What diagnostic tests are appropriate for evaluating heart failure in the OU -The clinical management protocol for heart failure in the OU -When it is appropriate to use high-dose diuretics	PGY-3, PGY-4	20 minutes (lecture) Instructors: 1 Equipment: PowerPoint (and projector/screen)	Milestones: -MK 2: Treatment and Clinical Reasoning (all milestones) -SBP 3: System Navigation for Patient-Centered Care (milestones spanning levels 1–4, excluding community health themed milestones) -PBLI 1: Evidence-Based and Informed Practice (all milestones) Assessment: End of rotation written assessment
Deep Vein Thrombosis (DVT) and Pulmonary Embolism (PE)	Brief lecture on the diagnosis and management of DVT and PE	-Risk calculation for DVT and PE (Well’s criteria) -Diagnostic strategies -Differentiating massive, submassive, and small to moderate PEs -Anticoagulation strategies -Indications for IVC filter	By the end of this lecture, learners will understand: -Preventative strategies for DVT and PE -Differential diagnoses for DVT and PE -Risk and benefits of lab and/or imaging studies to diagnose DVT and PE -Management strategies for massive, submassive, and small to moderate PEs -Outpatient management strategies of DVT and PE	PGY-3, PGY-4	20 minutes (lecture) Instructors: 1 Equipment: PowerPoint (and projector/screen)	Milestones: -MK 2: Treatment and Clinical Reasoning (all milestones) -SBP 3: System Navigation for Patient-Centered Care (milestones spanning levels 1–4, excluding community health themed milestones) -PBLI 1: Evidence-Based and Informed Practice (all milestones) Assessment: End of rotation written assessment
Dehydration	Brief lecture on the management of dehydration	-Epidemiology of dehydration -Detecting etiology of dehydration -Indications for admission to manage complications of dehydration -Parameters for disposition end points	Learners will understand: -Appropriate patient selection for OU management of dehydration -Benefits of protocolized observation care for patients with dehydration -How to protocolize fluid resuscitation and fluid loss prevention in dehydrated patients -How to evaluate for underlying causes of dehydration -How frequently to reassess patients and determine response to therapy -Indications and contraindications pharmacologic agents commonly used to manage dehydration -Effective disposition planning and outpatient follow up	PGY-3, PGY-4	20 minutes (lecture) Instructors: 1 Equipment: PowerPoint (and projector/screen)	Milestones: -MK 2: Treatment and Clinical Reasoning (all milestones) -SBP 3: System Navigation for Patient-Centered Care (milestones spanning levels 1–4, excluding community health themed milestones) -PBLI 1: Evidence-Based and Informed Practice (all milestones) Assessment: End of rotation written assessment
Geriatric Frailty	Brief lecture on the management of geriatric patients	-Definition of geriatric frailty -Unique clinical conditions and psychosocial challenges associated with geriatric patients -Parameters for disposition end points	Learners will understand: -Appropriate patient selection for OU management -Benefits of protocolized observation care for patients with frailty -Indications for social work, case management, physical therapy and home services -How frequently to reassess patients and determine response to therapy -Indications and contraindications to pharmacologic agents in geriatric patients (ie, BEERS criteria) -Effective disposition planning and outpatient follow up	PGY-3, PGY-4	20 minutes (lecture) Instructors: 1 Equipment: PowerPoint (and projector/screen)	Milestones: -MK 2: Treatment and Clinical Reasoning (all milestones) -SBP 3: System Navigation for Patient-Centered Care (milestones spanning levels 1–4, excluding community health themed milestones) -PBLI 1: Evidence-Based and Informed Practice (all milestones) Assessment: End of rotation written assessment
Gastrointestinal Bleed	Brief lecture on the management of GI bleeding	-Epidemiology of asthma -Detecting etiology of asthma exacerbations -Appropriate risk stratification for patients with acute asthma exacerbations -Evidence supporting pharmacologic agents to asthma -Indications for admission to a monitored setting -Parameters for disposition end points	Learners will understand: -Appropriate patient selection for OU management of asthma exacerbations -Benefits of protocolized observation care for patients with asthma exacerbations -How to protocolize symptom-triggered therapy to asthma exacerbations -How frequently to reassess patients and determine response to therapy -Indications and contraindications to pharmacologic agents commonly used to asthma -Effective disposition planning and outpatient follow up	PGY-3, PGY-4	20 minutes (lecture) Instructors: 1 Equipment: PowerPoint (and projector/screen	Milestones: -MK 2: Treatment and Clinical Reasoning (all milestones) -SBP 3: System Navigation for Patient-Centered Care (milestones spanning levels 1–4, excluding community health themed milestones) -PBLI 1: Evidence-Based and Informed Practice (all milestones) Assessment: End of rotation written assessment
Headache	Brief lecture on the evaluation and management of headache	-Etiologies of headaches (primary versus secondary) -Diagnostic approach to headaches -Evidence supporting pharmacologic agents for headaches -Disposition endpoints	By the end of this lecture, learners will understand: -How to conduct a systematic approach to evaluating the etiology of a headache -Key aspects of history and physical as they relate to the potential cause of the headache -What diagnostic tests are indicated when evaluating a headache -Medications that are indicated to treat various primary headache disorders	PGY-3, PGY-4	20 minutes (lecture) Instructors: 1 Equipment: PowerPoint (and projector/screen)	Milestones: -MK 2: Treatment and Clinical Reasoning (all milestones) -SBP 3: System Navigation for Patient-Centered Care (milestones spanning levels 1–4, excluding community health themed milestones) -PBLI 1: Evidence-Based and Informed Practice (all milestones) Assessment: End of rotation written assessment
Hyper-/Hypoglycemia	Brief lecture on the evaluation and management of dysglycemia	-Etiologies of abnormal dysglycemia -Management of dysglycemia -Disposition endpoints	By the end of this lecture, learners will understand: -How to conduct a systematic approach to evaluating the etiology of abnormal blood glucose homeostasis -Key aspects of history and physical as they relate to the potential cause of dysglycemia -What agents are used to manage hyperglycemia -How to prevent hypoglycemia	PGY-3, PGY-4	20 minutes (lecture) Instructors: 1 Equipment: PowerPoint (and projector/screen)	Milestones: -MK 2: Treatment and Clinical Reasoning (all milestones) -SBP 3: System Navigation for Patient-Centered Care (milestones spanning levels 1–4, excluding community health themed milestones) -PBLI 1: Evidence-Based and Informed Practice (all milestones) Assessment: End of rotation written assessment
Nephrolithiasis	Brief lecture on the evaluation and management of nephrolithiasis	-Management of renal colic -Management of simultaneous urinary tract infection -Disposition endpoints	By the end of this lecture, learners will understand: -Diagnostic modalities to evaluate renal colic -Evidence supporting different analgesic agents -What lab tests are indicated when evaluating potential complications of stones -When an urgent procedural intervention is indicated -When it is appropriate to disposition the patient	PGY-3, PGY-4	20 minutes (lecture) Instructors: 1 Equipment: PowerPoint (and projector/screen)	Milestones: -MK 2: Treatment and Clinical Reasoning (all milestones) -SBP 3: System Navigation for Patient-Centered Care (milestones spanning levels 1–4, excluding community health themed milestones) -PBLI 1: Evidence-Based and Informed Practice (all milestones) Assessment: End of rotation written assessment
Ovarian Torsion	Brief lecture on the evaluation and management of potential	-Risk factors for ovarian torsion -Diagnostic approach to ovarian torsion -Disposition endpoints	By the end of this lecture, learners will understand: -How to conduct a systematic approach to evaluating the cause of pelvic pain -Key aspects of history and physical as they relate to the potential cause of pelvic pain -What tests are indicated when evaluating for possible ovarian torsion, and subsequent testing if the initial results are equivocal -When it is appropriate to disposition the patient	PGY-3, PGY-4	20 minutes (lecture) Instructors: 1 Equipment: PowerPoint (and projector/screen)	Milestones: -MK 2: Treatment and Clinical Reasoning (all milestones) -SBP 3: System Navigation for Patient-Centered Care (milestones spanning levels 1–4, excluding community health themed milestones) -PBLI 1: Evidence-Based and Informed Practice (all milestones) Assessment: End of rotation written assessment
Pancreatitis	Brief lecture on the evaluation and management of pancreatitis	-Etiologies of pancreatitis -Diagnostic approach to cause of pancreatitis -Risk stratification of pancreatitis -Disposition endpoints	By the end of this lecture, learners will understand: -How to conduct a systematic approach to evaluating the etiology of pancreatitis -Key aspects of history and physical as they relate to the potential cause of pancreatitis -What tests are indicated when evaluating the hepatobiliary tract -Fluid, diet, and pain management strategies -When it is appropriate to disposition the patient	PGY-3, PGY-4	20 minutes (lecture) Instructors: 1 Equipment: PowerPoint (and projector/screen)	Milestones: -MK 2: Treatment and Clinical Reasoning (all milestones) -SBP 3: System Navigation for Patient-Centered Care (milestones spanning levels 1–4, excluding community health themed milestones) -PBLI 1: Evidence-Based and Informed Practice (all milestones) Assessment: End of rotation written assessment
Pneumonia	Brief lecture on the evaluation and management of pneumonia	-Risk stratification of pneumonia -Management of pneumonia -Disposition endpoints	By the end of this lecture, learners will understand: -What tests are indicated when managing pneumonia -How to classify pneumonia -The evidence supporting various antibiotic regimens for pneumonia -Adjunctive therapies to manage symptoms of pneumonia -When it is appropriate to disposition the patient	PGY-3, PGY-4	20 minutes (lecture) Instructors: 1 Equipment: PowerPoint (and projector/screen)	Milestones: -MK 2: Treatment and Clinical Reasoning (all milestones) -SBP 3: System Navigation for Patient-Centered Care (milestones spanning levels 1–4, excluding community health themed milestones) -PBLI 1: Evidence-Based and Informed Practice (all milestones) Assessment: End of rotation written assessment
Rib Fracture	Brief lecture on understanding, diagnosing, and managing rib fractures	-Etiology of rib fractures -Diagnostic tests for TIA -How to manage symptoms related to rib fractures -Disposition end points	By the end of this lecture, learners will understand: -Clinical manifestations of rib fractures -Diagnostic tests for evaluating rib fractures -Methods for pain control -Adjunctive therapies for symptom control -When it is appropriate to disposition the patient	PGY-3, PGY-4	20 minutes (lecture) Instructors: 1 Equipment: PowerPoint (and projector/screen)	Milestones: -MK 2: Treatment and Clinical Reasoning (all milestones) -SBP 3: System Navigation for Patient-Centered Care (milestones spanning levels 1–4, excluding community health themed milestones) -PBLI 1: Evidence-Based and Informed Practice (all milestones) Assessment: End of rotation written assessment
Seizure	Brief lecture on the evaluation and management of uncomplicated seizures	-Risk stratification of seizure disorders -Management of seizures -Disposition endpoints	By the end of this lecture, learners will understand: -How to classify seizure disorders -What diagnostic tests are indicated when managing seizure disorders -Safety precautions and rescue medications for seizures -The evidence supporting various antiepileptic drugs, and their side effects -How to collaborate with the neurology service in OU patient management -When it is appropriate to disposition the patient	PGY-3, PGY-4	20 minutes (lecture) Instructors: 1 Equipment: PowerPoint (and projector/screen)	Milestones: -MK 2: Treatment and Clinical Reasoning (all milestones) -SBP 3: System Navigation for Patient-Centered Care (milestones spanning levels 1–4, excluding community health themed milestones) -PBLI 1: Evidence-Based and Informed Practice (all milestones) Assessment: End of rotation written assessment
Sickle Cell Pain Crisis	Brief lecture on the evaluation and management of sickle cell disease (SSD) pain crisis	-Risk stratification of SSD -Pain management associated with SSD -Disposition endpoints	By the end of this lecture, learners will understand: -Pathophysiology and complications of SSD -What tests are indicated when evaluating SSD -The evidence supporting various therapies for managing SSD pain crisis -When it is appropriate to disposition the patient	PGY-3, PGY-4	20 minutes (lecture) Instructors: 1 Equipment: PowerPoint (and projector/screen)	Milestones: -MK 2: Treatment and Clinical Reasoning (all milestones) -SBP 3: System Navigation for Patient-Centered Care (milestones spanning levels 1–4, excluding community health themed milestones) -PBLI 1: Evidence-Based and Informed Practice (all milestones) Assessment: End of rotation written assessment
Transfusion	Brief lecture on the evaluation and management of anemia	-Risk stratification of anemia -Management of symptomatic anemia -Disposition endpoints	By the end of this lecture, learners will understand: -What tests are indicated when evaluating anemia in the OU -How to classify anemia -The evidence supporting transfusion thresholds in various patient populations -Adjunctive therapies to manage symptoms of anemia -How to manage complications related to transfusions -When it is appropriate to disposition the patient	PGY-3, PGY-4	20 minutes (lecture) Instructors: 1 Equipment: PowerPoint (and projector/screen)	Milestones: -MK 2: Treatment and Clinical Reasoning (all milestones) -SBP 3: System Navigation for Patient-Centered Care (milestones spanning levels 1–4, excluding community health themed milestones) -PBLI 1: Evidence-Based and Informed Practice (all milestones) Assessment: End of rotation written assessment
Transient Ischemic Attack (TIA)	Brief lecture on understanding, diagnosing, and managing TIA	-Etiology of TIA -Risk factors for TIA -Spectrum of TIA presentations -Differential diagnosis for TIA -Diagnostic tests for TIA -When to utilize a neurology consult	By the end of this lecture, learners will understand: -The definition of TIA -Clinical manifestations of TIA -Risk stratification of TIA -Diagnostic tests for evaluating TIA -Evidence-based OU management of TIA -Medications indicated at discharge to prevent future TIA/stroke	PGY-3, PGY-4	20 minutes (lecture) Instructors: 1 Equipment: PowerPoint (and projector/screen)	Milestones: -MK 2: Treatment and Clinical Reasoning (all milestones) -SBP 3: System Navigation for Patient-Centered Care (milestones spanning levels 1–4, excluding community health themed milestones) -PBLI 1: Evidence-Based and Informed Practice (all milestones) Assessment: End of rotation written assessment
Urinary Tract Infection (UTI)	Brief lecture on the evaluation and management of urinary tract infection	-Risk stratification of urinary tract infection -Management of urinary tract infection -Disposition endpoints	By the end of this lecture, learners will understand: -What tests are indicated when managing UTIs -How to identify patients with complicated UTIs -The evidence supporting various antibiotic regimens for UTIs -Adjunctive therapies to manage symptoms of UTIs -When it is appropriate to disposition the patient	PGY-3, PGY-4	20 minutes (lecture) Instructors: 1 Equipment: PowerPoint (and projector/screen)	Milestones: -MK 2: Treatment and Clinical Reasoning (all milestones) -SBP 3: System Navigation for Patient-Centered Care (milestones spanning levels 1–4, excluding community health themed milestones) -PBLI 1: Evidence-Based and Informed Practice (all milestones) Assessment: End of rotation written assessment
Vertigo (peripheral)	Brief lecture on the evaluation and management of vertigo	-Etiologies of vertigo -Differentiating peripheral from central vertigo -Symptom control for vertigo -Disposition endpoints	By the end of this lecture, learners will understand: -How to conduct a systematic approach to differentiating central from peripheral vertigo -Key aspects of history and physical as they relate to the potential cause of vertigo -Which medications are indicated when managing vertigo -What diagnostic tests are indicated when evaluating vertigo -When it is appropriate to consult neurology	PGY-3, PGY-4	20 minutes (lecture) Instructors: 1 Equipment: PowerPoint (and projector/screen)	Milestones: -MK 2: Treatment and Clinical Reasoning (all milestones) -SBP 3: System Navigation for Patient-Centered Care (milestones spanning levels 1–4, excluding community health themed milestones) -PBLI 1: Evidence-Based and Informed Practice (all milestones) Assessment: End of rotation written assessment
Systems-based Practices Relevant to Observation Medicine	Hands-on teaching during rounds regarding systems base-based practices used in the observation unit	-Critical assessment of OU protocol performance -How to propose new OU protocols -Applying evidence-based risk stratification -How to efficiently incorporate relevant consult services	By end of the rotation, learners will understand how to: -Critically assess OU protocol performance based on provider, unit, or institutional feedback -Propose a new OU clinical protocol that incorporates personal, local, or published data. -Apply evidenced-based risk stratification to determine appropriate use of diagnostic testing and clinical end-points. -Efficiently incorporate relevant consult services with targeted clinical question as it relates to observation care and disposition	PGY-3, PGY-4	1 hour (Lecture and Hands-on learning) Instructors: 1	Milestones: -SBP 2: Quality Improvement (all milestones) Assessment: Attendings review learner’s assessment of protocol performance, new clinical protocols, risk stratification, and engagement with consult services, and provide continuous feedback.
Observation Documentation	Hands-on Teaching during rounds regarding documentation specific to observation medicine	-OU arrival notes -reassessment notes -discharge narratives -arrival, transfer, and discharge medication reconciliations	By end of the rotation, learners will understand how to: -Write a complete OU arrival note that accurately assesses the patient’s observation care needs and complies with observation billing and coding regulations -Write reassessment note(s) that reflect timely and accurate reassessments based on the specific patient needs -Write accurate, informative, and complete discharge narratives that describe the observation course, final physical exam, disposition, and post-discharge follow-up plan -Write accurate, informative, and complete arrival, transfer and discharge medication reconciliations	PGY-3, PGY-4	Continuous over the two-week rotation (Hands-on learning) Instructors: 2	Milestones: -SBP 4 (Physician Role in Health Care Systems): -“Demonstrates efficient integration of information technology required for medical practice (eg, electronic health record, documentation required for billing and coding)” -“Identifies basic knowledge domains required for medical practice (eg, information technology, legal, billing, coding, financial, and personnel aspects)” Assessment: Attendings review learner’s documentation and provide attestation if done correctly and feedback when done incorrectly.

## DIDACTIC AND HANDS-ON CURRICULUM

[Table t2-jetem-6-2-c1]

## Appendix A Pre- and Post-Rotation Survey Results

**Table t3-jetem-6-2-c1:**

Multiple Choice Questions	Pre-Rotation Survey Responses	Post-Rotation Survey Responses
Q1: Which residency program are you currently a part of? -Emergency Medicine -Internal Medicine -Family Medicine -Surgical Specialty	Learner 1: Emergency Medicine Learner 2: Emergency Medicine Learner 3: Emergency Medicine	Learner 1: Emergency Medicine Learner 2: Emergency Medicine Learner 3: Emergency Medicine
Q2: Please select your current level of training: -PGY-1 -PGY-2 -PGY-3 -PGY-4 -PGY-5 -PGY-6 -PGY-7 -PGY-8	Learner 1: PGY-3 Learner 2: PGY-4 Learner 3: PGY-4	Learner 1: PGY-3 Learner 2: PGY-4 Learner 3: PGY-4
Q3 - Have you ever completed an observation medicine rotation? -Yes -No	Learner 1: No Learner 2: No Learner 3: No	Learner 1: Yes Learner 2: Yes Learner 3: Yes
Q4: Rate your current ability to define the role and purpose of the observation unit. -Not at all confident -Somewhat confident -Fairly confident -Very confident	Learner 1: Somewhat confident Learner 2: Fairly confident Learner 3: Fairly confident	Learner 1: Very confident Learner 2: Very confident Learner 3: Very confident
Q5: Please rate your current ability to accurately triage patients from the ED to the observation unit, the short stay unit, or the medicine service. -Not at all confident -Somewhat confident -Fairly confident -Very confident	Learner 1: Fairly confident Learner 2: Fairly confident Learner 3: Fairly confident	Learner 1: Fairly confident Learner 2: Very confident Learner 3: Very confident
Q6: Please rate your current ability to complete an initial assessment of observation unit patients. -Not at all confident -Somewhat confident -Fairly confident -Very confident	Learner 1: Somewhat confident Learner 2: Fairly confident Learner 3: Fairly confident	Learner 1: Very confident Learner 2: Very confident Learner 3: Very confident
Q7: Please rate your current ability to establish a management plan for an observation unit patient. -Not at all confident -Somewhat confident -Fairly confident -Very confident	Learner 1: Somewhat confident Learner 2: Somewhat confident Learner 3: Fairly confident	Learner 1: Fairly confident Learner 2: Very confident Learner 3: Very confident
Q8: Please rate your current ability to set up a safe discharge plan for a complex patient (ie, palliative, non-weight bearing, severely demented, difficult family). -Not at all confident -Somewhat confident -Fairly confident -Very confident	Learner 1: Somewhat confident Learner 2: Somewhat confident Learner 3: Somewhat confident	Learner 1: Fairly confident Learner 2: Very confident Learner 3: Very confident

**Table t4-jetem-6-2-c1:**

Pre-Rotation Written Question 1: What do you hope to gain from a rotation in observation medicine?
**Learner 1:** Develop a better understanding of the role of the observation unit and the approach to a diagnostic work up of observation unit patients. Identify patient safety related issues in the observation unit and develop plan of action for improvement.
**Learner 2:** Learn the treatment pathways and end goals for discharge.
**Learner 3:** The ability to effectively assess and manage patients in an observation unit with a wide variety of diagnoses.
**Post-Rotation Written Question 1:** **Do you feel that the observation medicine elective was useful for you, in the context of your emergency medicine training?**
**Learner 1:** Yes, this was a very useful rotation. Gave insight into the capabilities of the observation unit for further diagnostic testing and management of patients. Helped provide a better understanding of what it means to perform a cardiac rule out, will better enable me to make ER management decisions.
**Learner 2:** Yes.
**Learner 3:** Yes, very useful.
**Post-Rotation Written Question 2:** **Would you recommend the observation medicine elective to your fellow residents? Why or why not?**
**Learner 1:** Yes. Many residents will work in hospitals where they will be responsible for running observation units. This rotation could replace in part or all of the internal medicine rotation in residency as it teaches more relevant topics in the initial management of those admitted/observed in the hospital. Gives an important understanding of what happens to patients after placement in the hospital. Allows for senior residents to have a degree of personal responsibility for patients when running a side.
**Learner 2:** Yes if they want to see what obs is about. The staff and providers were great. Very welcoming. Very informative.
**Learner 3:** Yes, definitely. It is important to have an understanding of the management of patients beyond the ER. The rotation exposes one to internal medicine skills that we do not see in the ER, such as management of CHF, workup of angina, etc, that are important concepts for ER doctors to grasp. The rotation also allows for a chance for residents to work on formulating more of a differential diagnosis and management plan by being able to spend more time with a single patient and more time thinking about that patient than the time we have in the ER. Working in an observation unit is also required for many ER jobs and exposure in residency seems important if that is to be a part of one’s career.
**Post-Rotation Written Question 3:** **Do you have any comments or feedback for the observation medicine elective? Anything you wanted more of? Structure? Teaching or other?**
**Learner 1:** Overall a strong rotation. I appreciated the mix of shift times that allowed for both the management of patients already in the unit and admission of new patients. I appreciated being given the responsibility to run the unit for the day. Maybe for further senior residents have them perform screening of all new admissions in a particular evening.
**Learner 2:** The preceptor role was great.
**Learner 3:** I really enjoyed it. I enjoyed the mix of roles I was assigned, including taking care of patients already admitted to the observation unit, accepting new patients to the observation unit from the ER, and deciding on the workup and management plan for new observation unit patients. I also really enjoyed working one on one with the attending and hearing their thought process and seeing how they manage the patients, deciding who to send home, who to admit, who needs further workup and to what extent, etc. The lectures are also helpful and productive and I would recommend trying to have one every day on the rotation.

[Table t3-jetem-6-2-c1] [Table t4-jetem-6-2-c1]

## Appendix B Attending End-of-Shift Resident Evaluation

[Fig f1-jetem-6-2-c1]

### Observation Curriculum Cellulitis Lecture

Please see associated PowerPoint file

### Observation Curriculum Chest Pain Lecture

Please see associated PowerPoint file

### Observation Curriculum DVT and PE Lecture

Please see associated PowerPoint file

### Observation Curriculum GI Bleed Lecture

Please see associated PowerPoint file

### Observation Curriculum Headache Lecture

Please see associated PowerPoint file

### Observation Curriculum Sickle Cell Lecture

Please see associated PowerPoint file

### Observation Curriculum Asthma Lecture

Please see associated PowerPoint file

### Observation Curriculum Abdominal Pain Lecture

Please see associated PowerPoint file

### Observation Curriculum Atrial Fibrillation Lecture

Please see associated PowerPoint file

### Observation Curriculum Dehydration Lecture

Please see associated PowerPoint file

### Observation Curriculum Heart Failure Lecture

Please see associated PowerPoint file

### Observation Curriculum Nephrolithiasis Lecture

Please see associated PowerPoint file

### Observation Curriculum Ovarian Torsion Lecture

Please see associated PowerPoint file

### Observation Curriculum Overview of Obs Lecture

Please see associated PowerPoint file

### Observation Curriculum Pancreatitis Lecture

Please see associated PowerPoint file

### Observation Curriculum Pneumonia Lecture

Please see associated PowerPoint file

### Observation Curriculum Rib Fractures Lecture

Please see associated PowerPoint file

### Observation Curriculum Transfusion Lecture

Please see associated PowerPoint file

### Observation Curriculum UTI Lecture

Please see associated PowerPoint file

### Observation Curriculum Vertigo Lecture

Please see associated PowerPoint file

### Observation Curriculum Allergic Reaction Lecture

Please see associated PowerPoint file

### Observation Curriculum Back Pain Lecture

Please see associated PowerPoint file

### Observation Curriculum Hyper & Hypoglycemia Lecture

Please see associated PowerPoint file

### Observation Curriculum Geriatric Frailty Lecture

Please see associated PowerPoint file

### Observation Curriculum Management of Alcohol Withdrawal Lecture

Please see associated PowerPoint file

### Observation Curriculum Seizures Lecture

Please see associated PowerPoint file

### Observation Curriculum TIA Lecture

Please see associated PowerPoint file
