# Supplementary material for: An Observation Medicine Curriculum for Emergency Medicine Education
Source: J Educ Teach Emerg Med. 2021 Apr 19;6(2):C1–C72. doi: 10.21980/J87P92 (PMC10332786; doi:10.21980/J87P92)
Supplement: Supplementary file 1 — Please see associated PowerPoint file [file jetem-6-2-c1-supp1.pptx]

## Slide 1
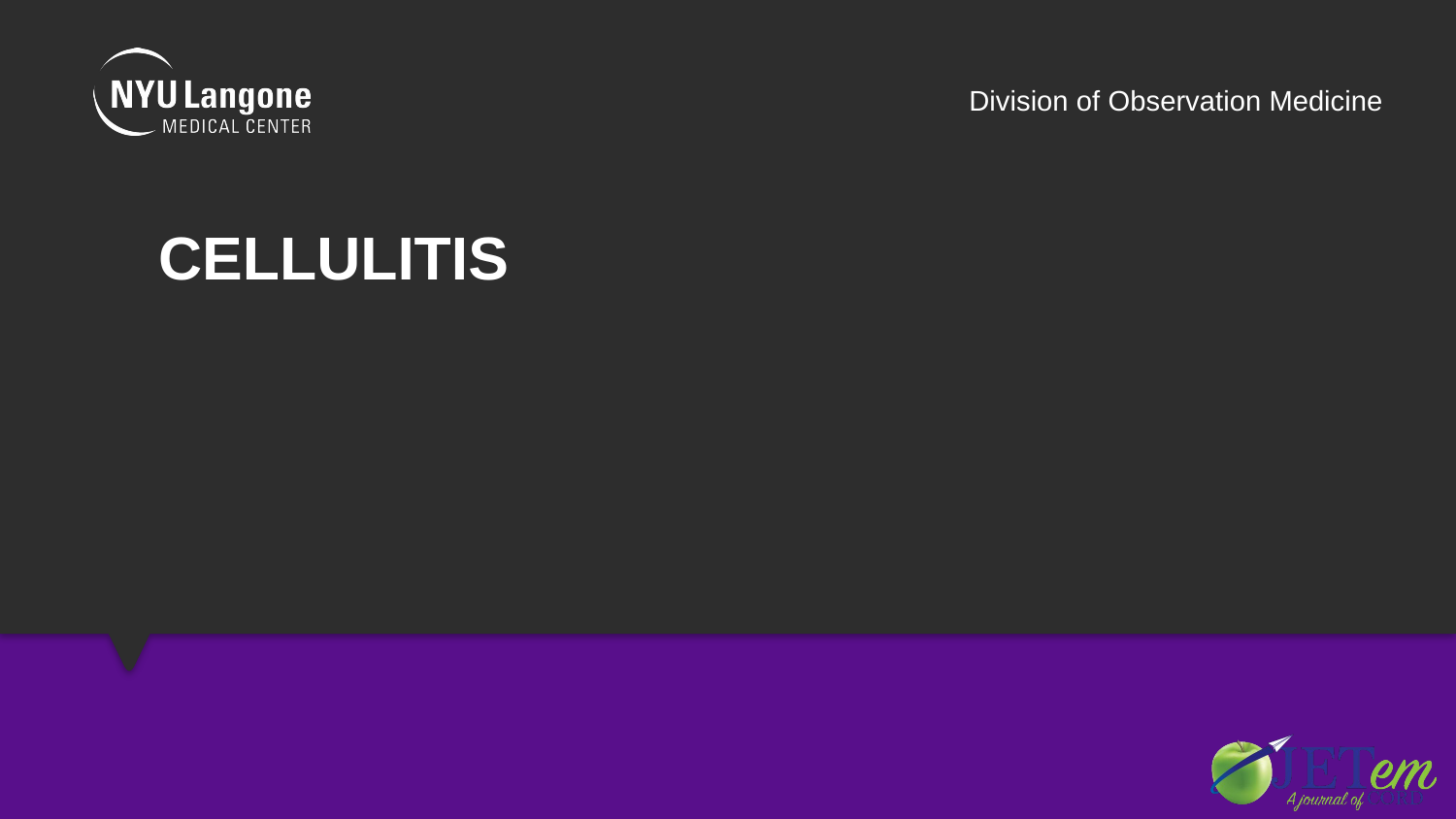

Division of Observation Medicine
# CELLULITIS

## Slide 2
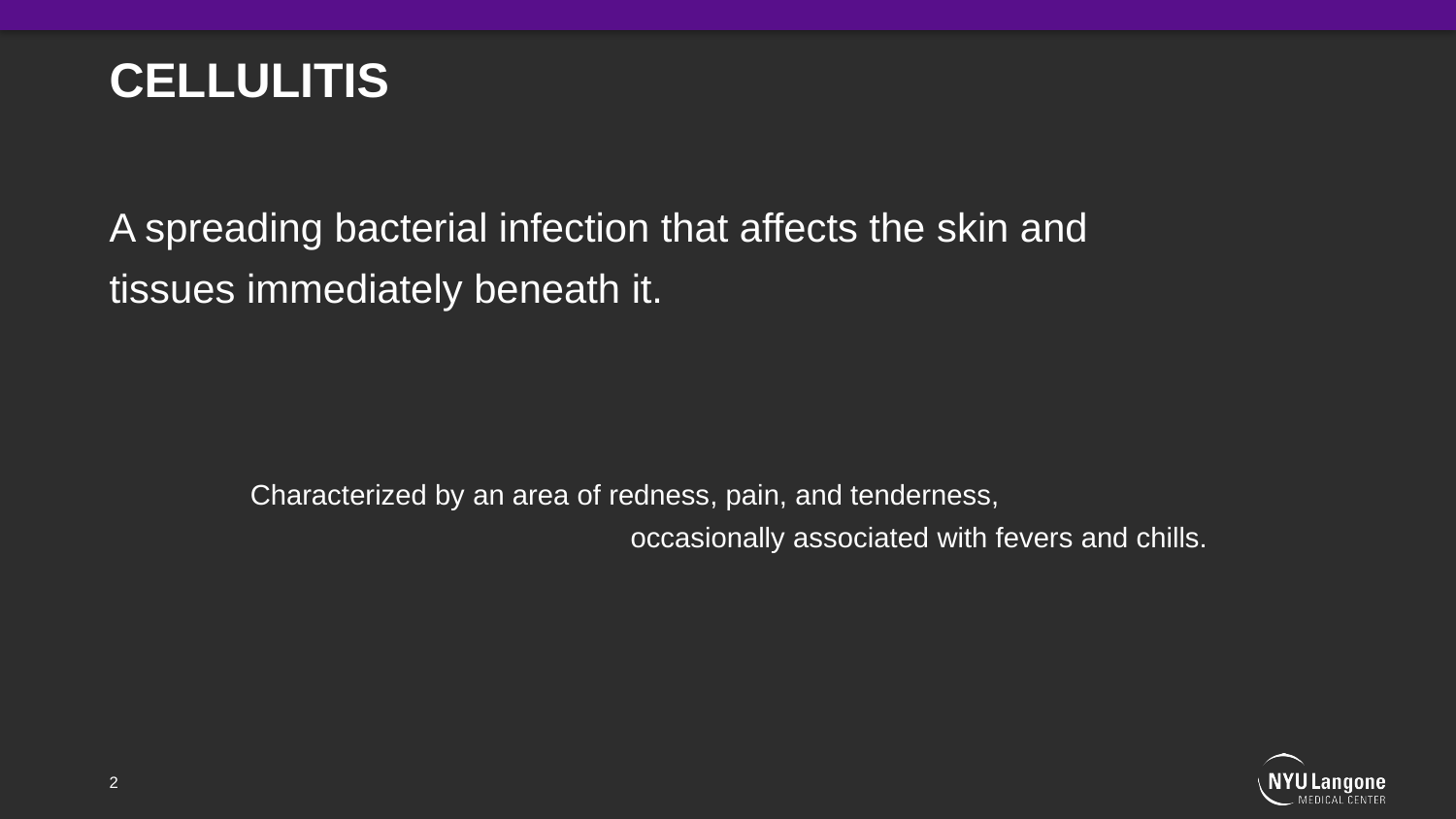

# CELLULITIS
A spreading bacterial infection that affects the skin and tissues immediately beneath it.
Characterized by an area of redness, pain, and tenderness, occasionally associated with fevers and chills.
2

## Slide 3
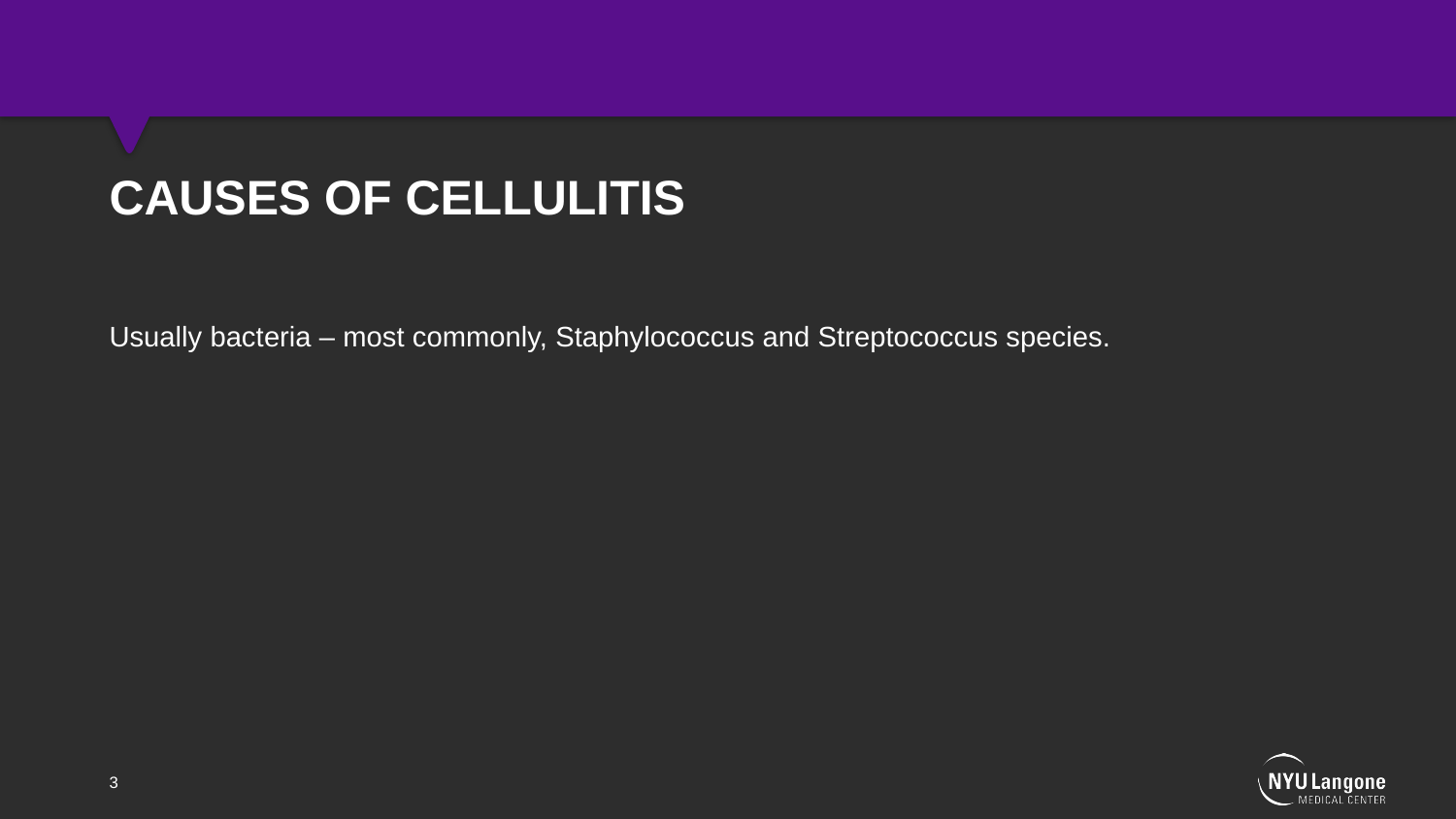

# CAUSES OF CELLULITIS
Usually bacteria – most commonly, Staphylococcus and Streptococcus species.
3

## Slide 4
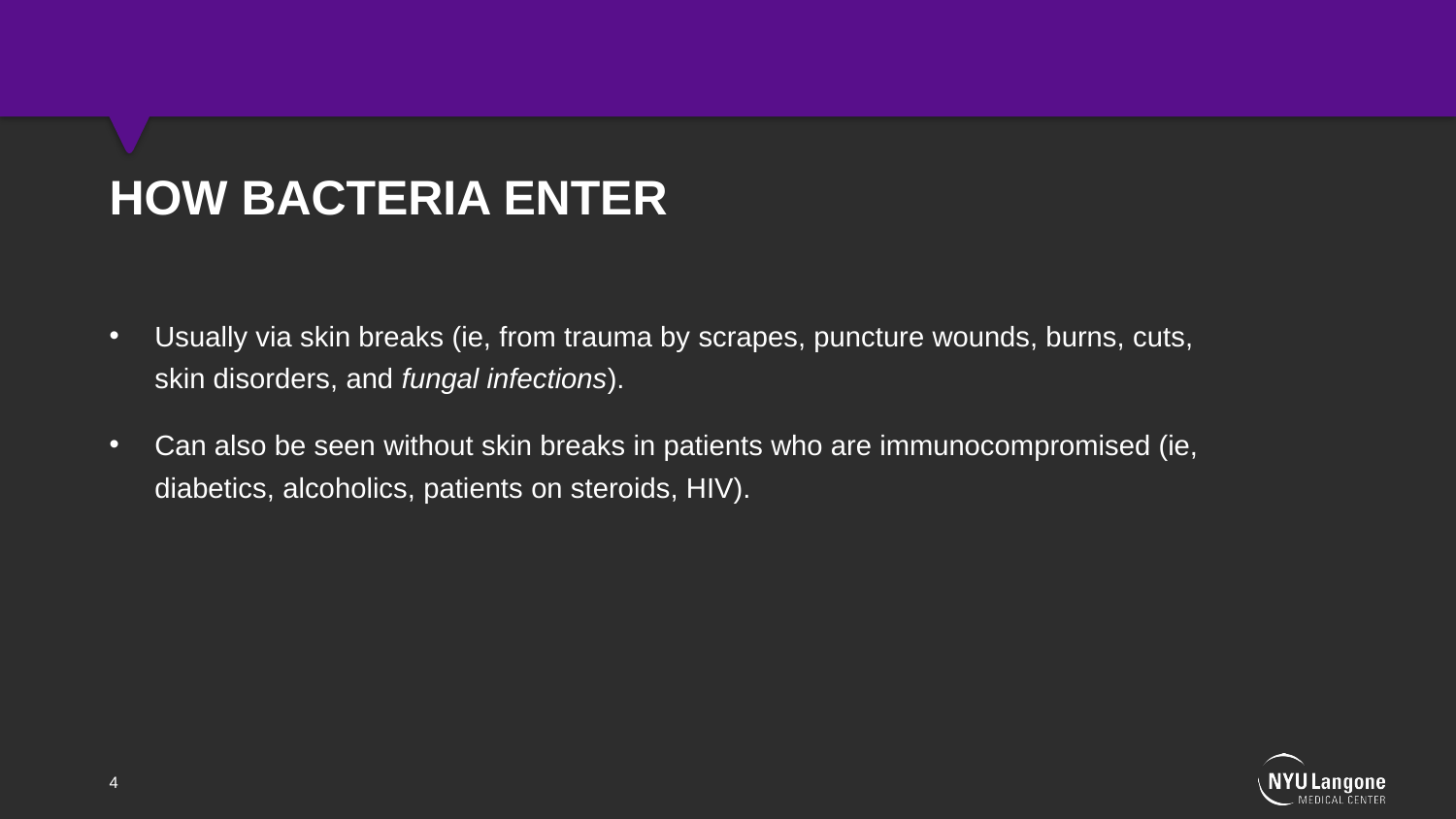

# HOW BACTERIA ENTER
Usually via skin breaks (ie, from trauma by scrapes, puncture wounds, burns, cuts, skin disorders, and fungal infections).
Can also be seen without skin breaks in patients who are immunocompromised (ie, diabetics, alcoholics, patients on steroids, HIV).
4

## Slide 5
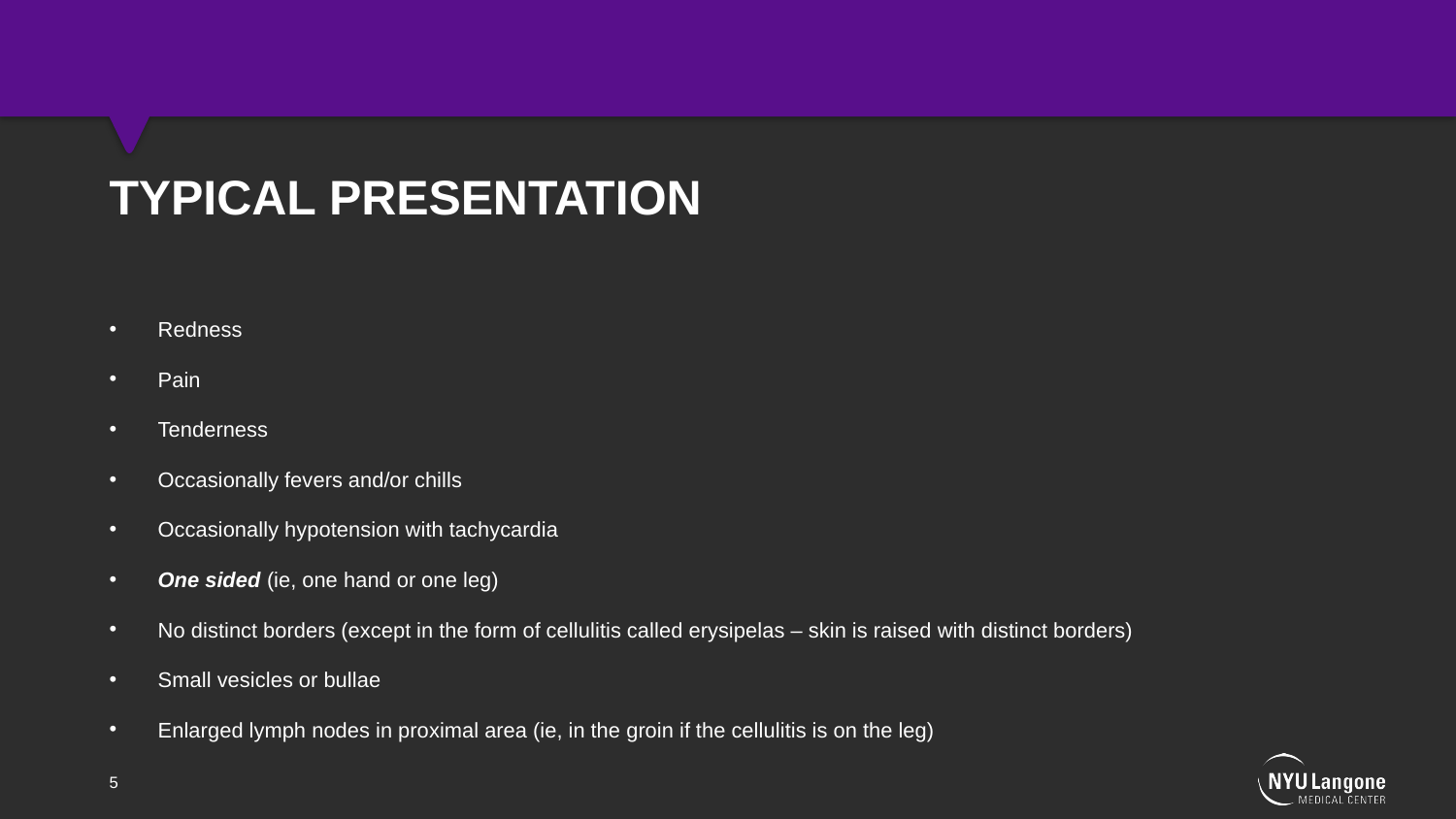

# TYPICAL PRESENTATION
Redness
Pain
Tenderness
Occasionally fevers and/or chills
Occasionally hypotension with tachycardia
One sided (ie, one hand or one leg)
No distinct borders (except in the form of cellulitis called erysipelas – skin is raised with distinct borders)
Small vesicles or bullae
Enlarged lymph nodes in proximal area (ie, in the groin if the cellulitis is on the leg)
5

## Slide 6
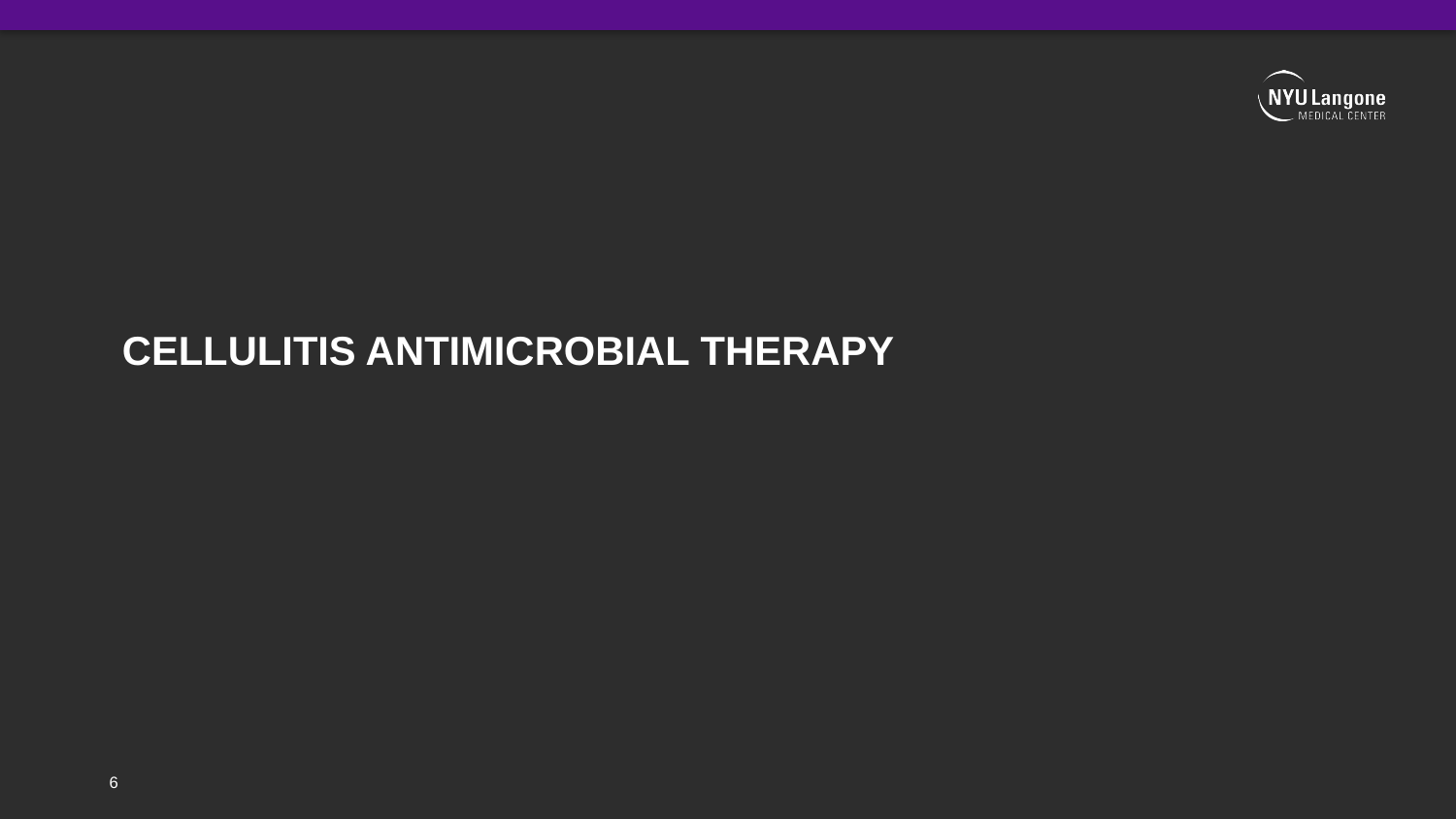

# CELLULITIS ANTIMICROBIAL THERAPY
6

## Slide 7
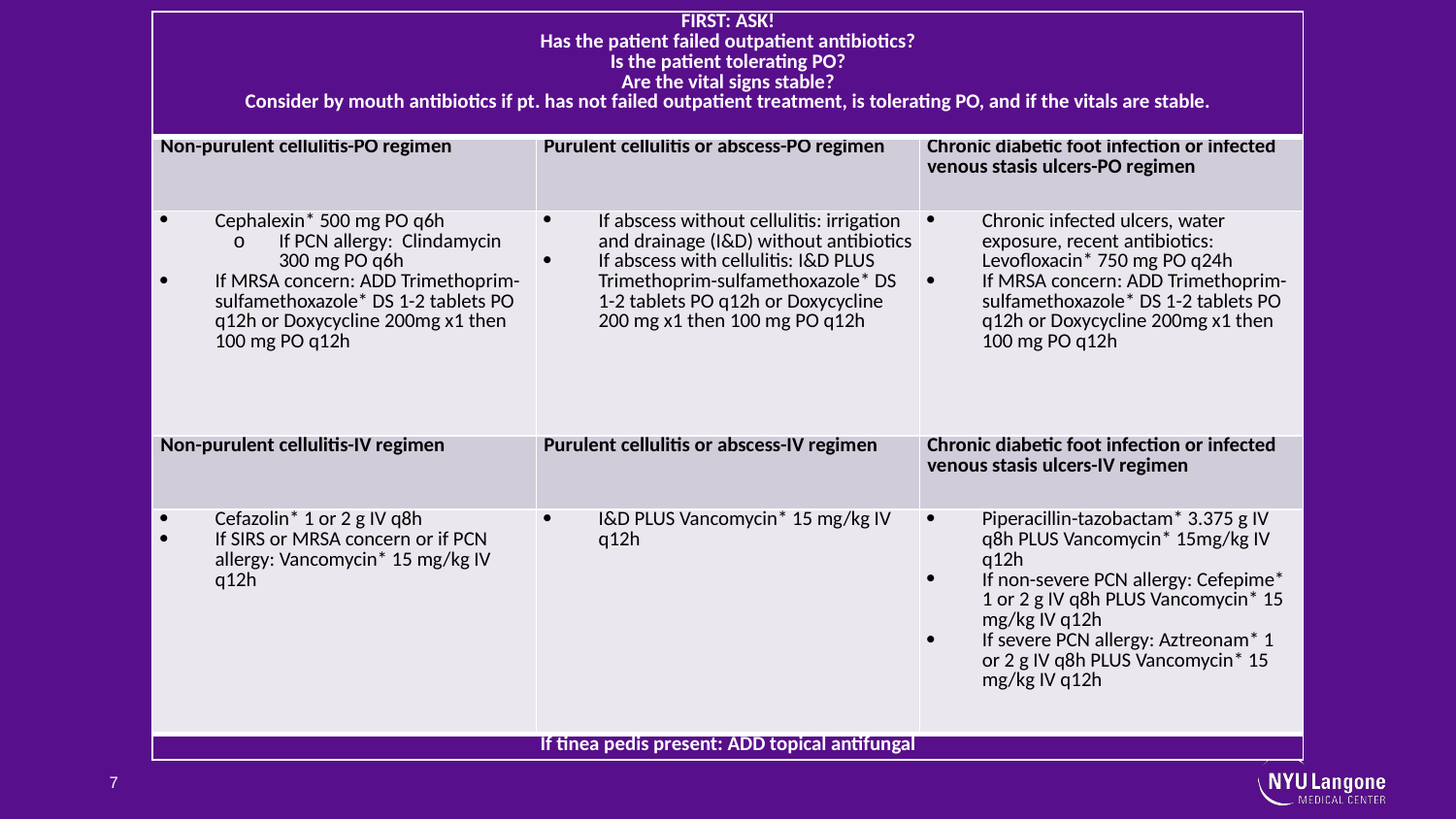

| FIRST: ASK! Has the patient failed outpatient antibiotics? Is the patient tolerating PO? Are the vital signs stable? Consider by mouth antibiotics if pt. has not failed outpatient treatment, is tolerating PO, and if the vitals are stable. | | |
| --- | --- | --- |
| Non-purulent cellulitis-PO regimen | Purulent cellulitis or abscess-PO regimen | Chronic diabetic foot infection or infected venous stasis ulcers-PO regimen |
| Cephalexin\* 500 mg PO q6h If PCN allergy: Clindamycin 300 mg PO q6h If MRSA concern: ADD Trimethoprim-sulfamethoxazole\* DS 1-2 tablets PO q12h or Doxycycline 200mg x1 then 100 mg PO q12h | If abscess without cellulitis: irrigation and drainage (I&D) without antibiotics If abscess with cellulitis: I&D PLUS Trimethoprim-sulfamethoxazole\* DS 1-2 tablets PO q12h or Doxycycline 200 mg x1 then 100 mg PO q12h | Chronic infected ulcers, water exposure, recent antibiotics: Levofloxacin\* 750 mg PO q24h If MRSA concern: ADD Trimethoprim-sulfamethoxazole\* DS 1-2 tablets PO q12h or Doxycycline 200mg x1 then 100 mg PO q12h |
| Non-purulent cellulitis-IV regimen | Purulent cellulitis or abscess-IV regimen | Chronic diabetic foot infection or infected venous stasis ulcers-IV regimen |
| Cefazolin\* 1 or 2 g IV q8h If SIRS or MRSA concern or if PCN allergy: Vancomycin\* 15 mg/kg IV q12h | I&D PLUS Vancomycin\* 15 mg/kg IV q12h | Piperacillin-tazobactam\* 3.375 g IV q8h PLUS Vancomycin\* 15mg/kg IV q12h If non-severe PCN allergy: Cefepime\* 1 or 2 g IV q8h PLUS Vancomycin\* 15 mg/kg IV q12h If severe PCN allergy: Aztreonam\* 1 or 2 g IV q8h PLUS Vancomycin\* 15 mg/kg IV q12h |
| If tinea pedis present: ADD topical antifungal | | |
7

## Slide 8
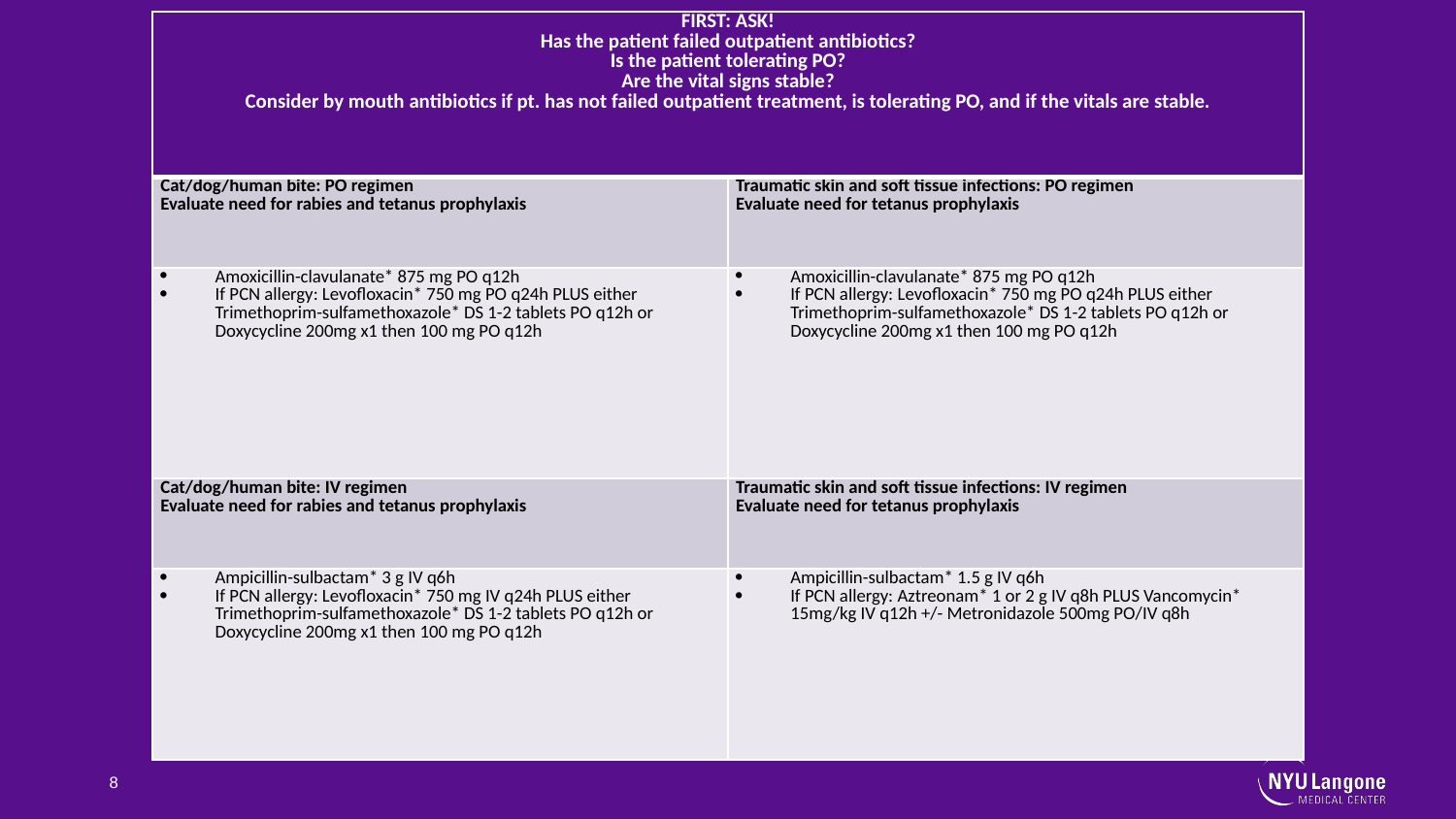

| FIRST: ASK! Has the patient failed outpatient antibiotics? Is the patient tolerating PO? Are the vital signs stable? Consider by mouth antibiotics if pt. has not failed outpatient treatment, is tolerating PO, and if the vitals are stable. | |
| --- | --- |
| Cat/dog/human bite: PO regimen Evaluate need for rabies and tetanus prophylaxis | Traumatic skin and soft tissue infections: PO regimen Evaluate need for tetanus prophylaxis |
| Amoxicillin-clavulanate\* 875 mg PO q12h If PCN allergy: Levofloxacin\* 750 mg PO q24h PLUS either Trimethoprim-sulfamethoxazole\* DS 1-2 tablets PO q12h or Doxycycline 200mg x1 then 100 mg PO q12h | Amoxicillin-clavulanate\* 875 mg PO q12h If PCN allergy: Levofloxacin\* 750 mg PO q24h PLUS either Trimethoprim-sulfamethoxazole\* DS 1-2 tablets PO q12h or Doxycycline 200mg x1 then 100 mg PO q12h |
| Cat/dog/human bite: IV regimen Evaluate need for rabies and tetanus prophylaxis | Traumatic skin and soft tissue infections: IV regimen Evaluate need for tetanus prophylaxis |
| Ampicillin-sulbactam\* 3 g IV q6h If PCN allergy: Levofloxacin\* 750 mg IV q24h PLUS either Trimethoprim-sulfamethoxazole\* DS 1-2 tablets PO q12h or Doxycycline 200mg x1 then 100 mg PO q12h | Ampicillin-sulbactam\* 1.5 g IV q6h If PCN allergy: Aztreonam\* 1 or 2 g IV q8h PLUS Vancomycin\* 15mg/kg IV q12h +/- Metronidazole 500mg PO/IV q8h |
8

## Slide 9
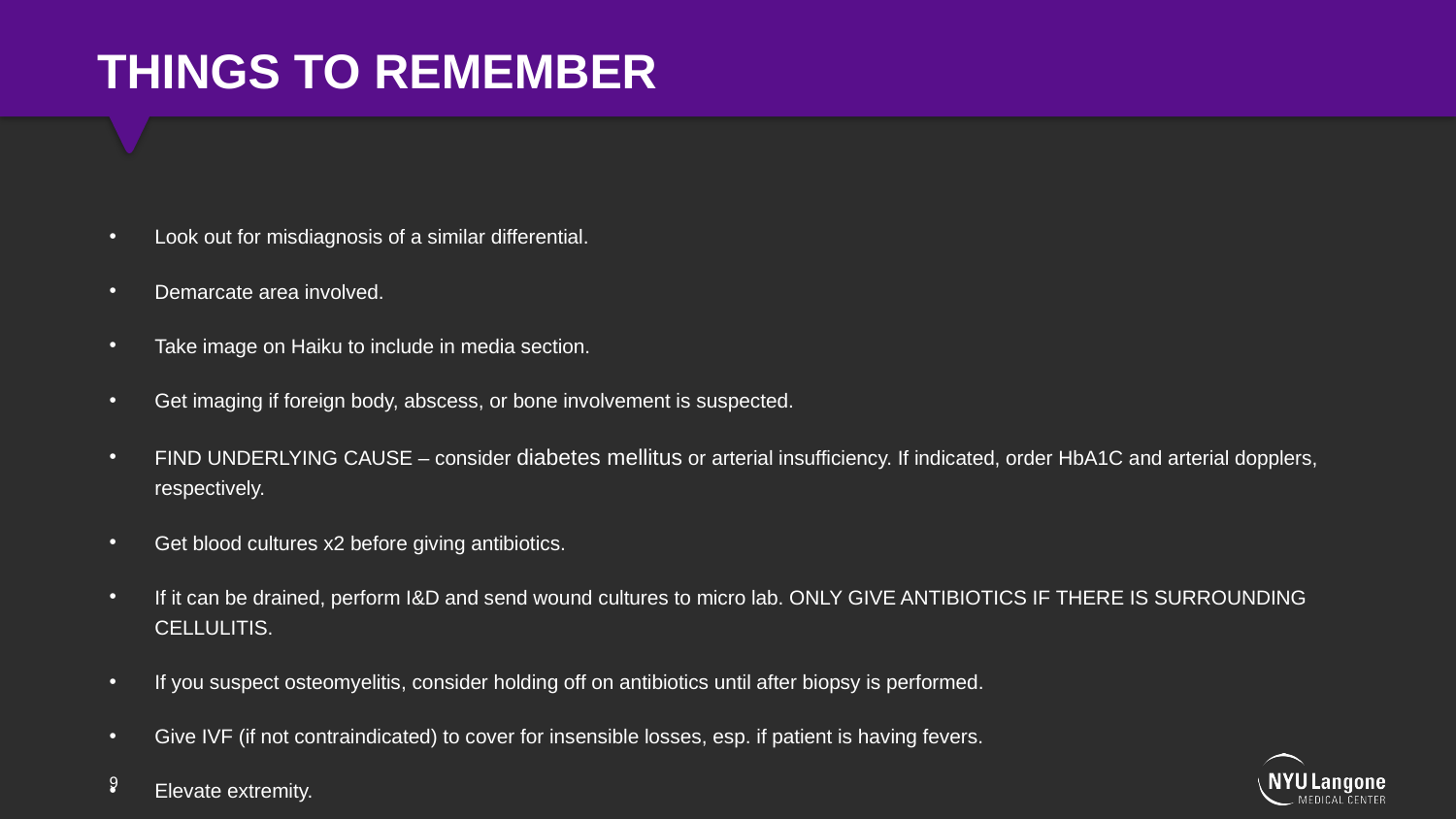

# THINGS TO REMEMBER
Look out for misdiagnosis of a similar differential.
Demarcate area involved.
Take image on Haiku to include in media section.
Get imaging if foreign body, abscess, or bone involvement is suspected.
FIND UNDERLYING CAUSE – consider diabetes mellitus or arterial insufficiency. If indicated, order HbA1C and arterial dopplers, respectively.
Get blood cultures x2 before giving antibiotics.
If it can be drained, perform I&D and send wound cultures to micro lab. ONLY GIVE ANTIBIOTICS IF THERE IS SURROUNDING CELLULITIS.
If you suspect osteomyelitis, consider holding off on antibiotics until after biopsy is performed.
Give IVF (if not contraindicated) to cover for insensible losses, esp. if patient is having fevers.
Elevate extremity.
9

## Slide 10
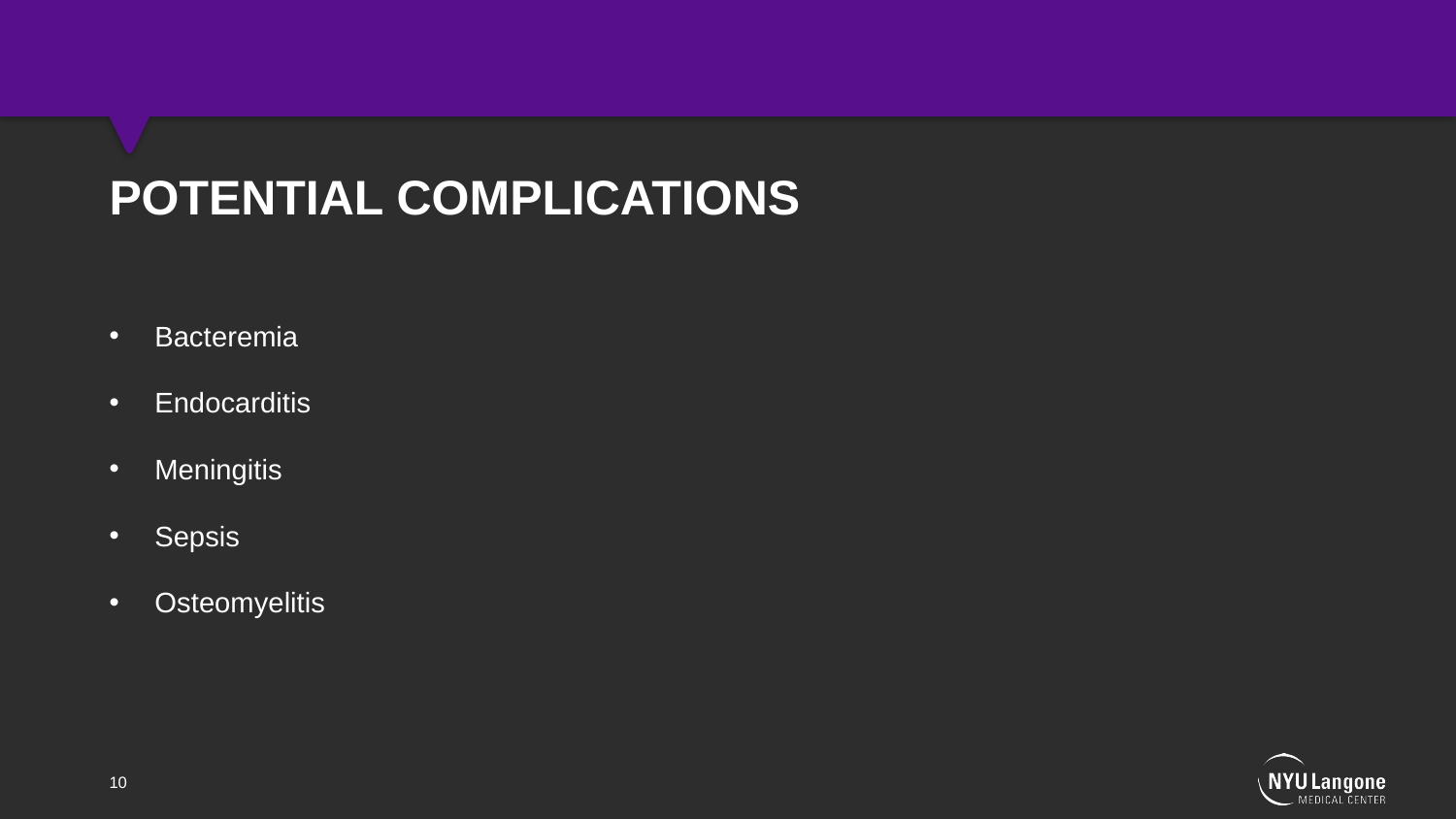

# POTENTIAL COMPLICATIONS
Bacteremia
Endocarditis
Meningitis
Sepsis
Osteomyelitis
10

## Slide 11
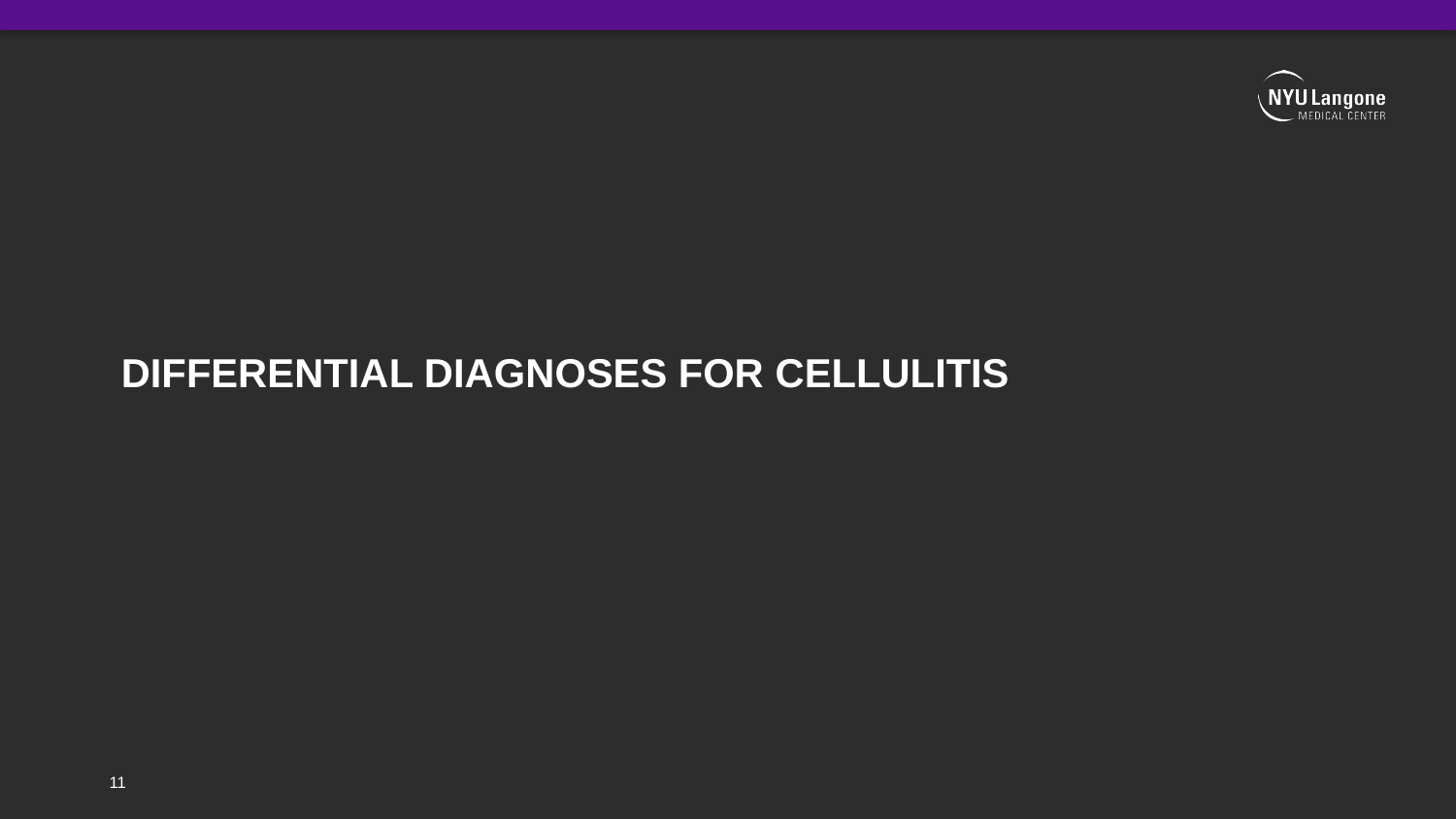

# DIFFERENTIAL DIAGNOSES FOR CELLULITIS
11

## Slide 12
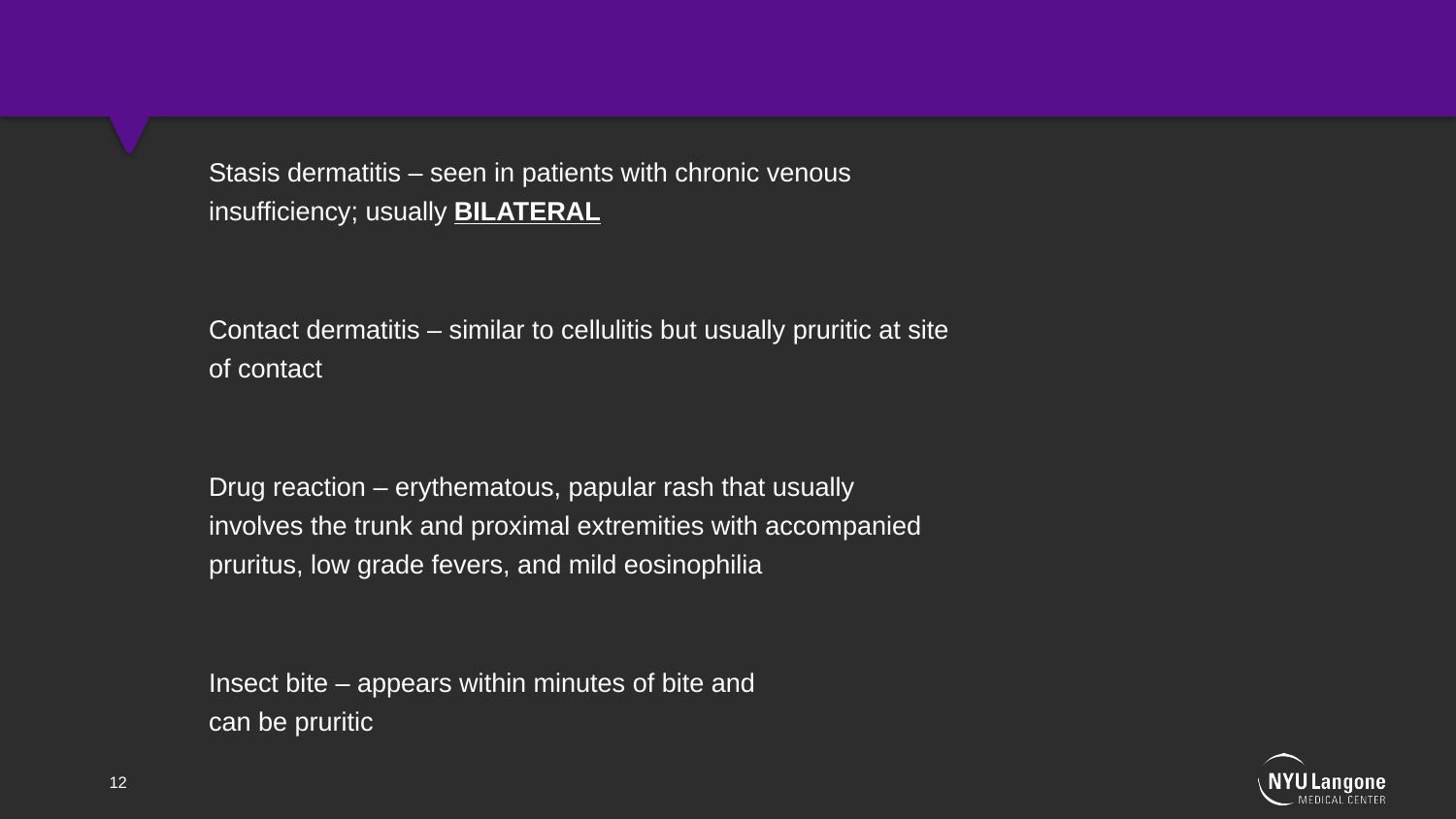

Stasis dermatitis – seen in patients with chronic venous
insufficiency; usually BILATERAL
Contact dermatitis – similar to cellulitis but usually pruritic at site
of contact
Drug reaction – erythematous, papular rash that usually
involves the trunk and proximal extremities with accompanied
pruritus, low grade fevers, and mild eosinophilia
Insect bite – appears within minutes of bite and
can be pruritic
12

## Slide 13
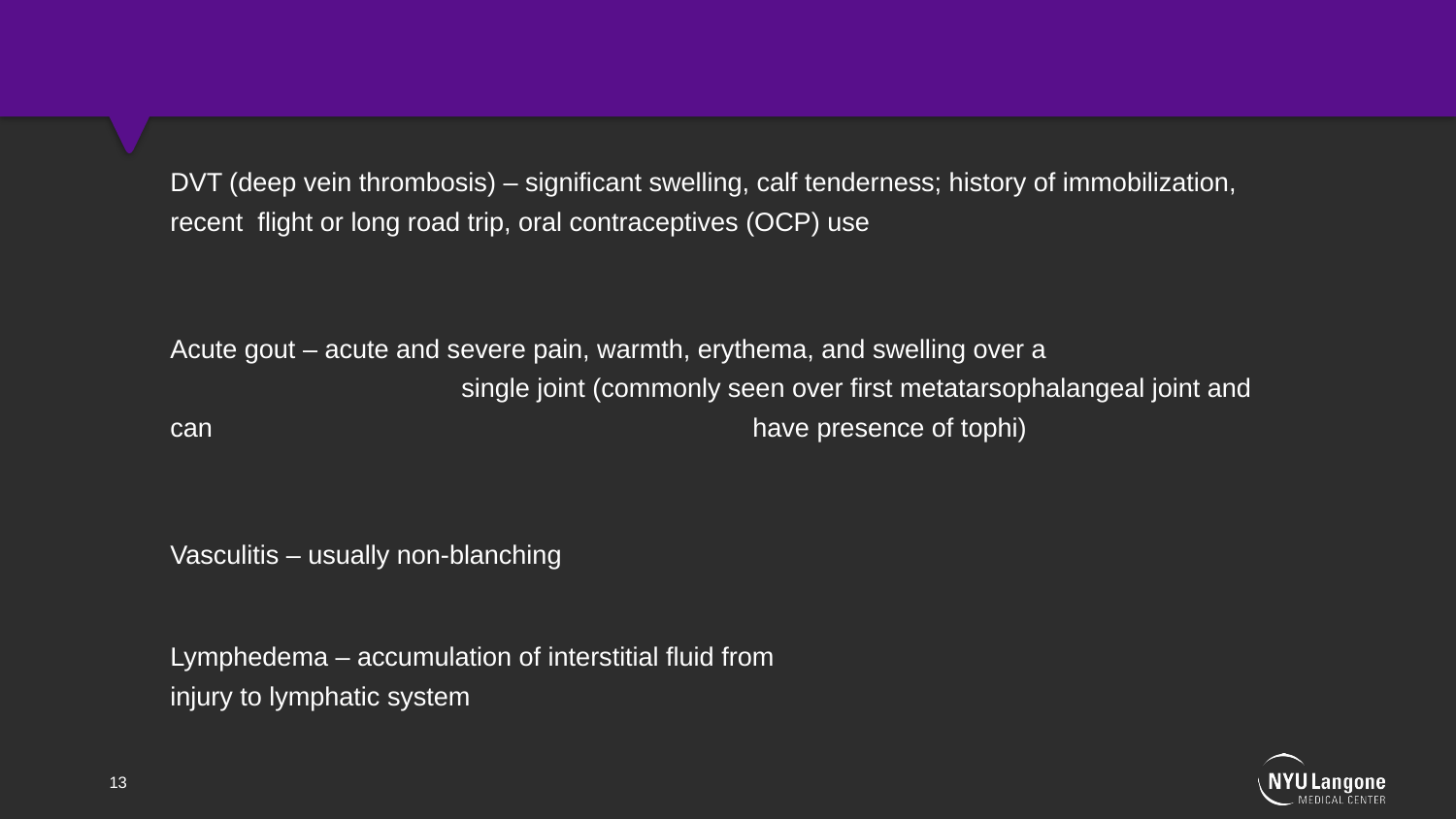

DVT (deep vein thrombosis) – significant swelling, calf tenderness; history of immobilization, recent flight or long road trip, oral contraceptives (OCP) use
Acute gout – acute and severe pain, warmth, erythema, and swelling over a 			single joint (commonly seen over first metatarsophalangeal joint and can 				have presence of tophi)
Vasculitis – usually non-blanching
Lymphedema – accumulation of interstitial fluid from
injury to lymphatic system
13

## Slide 14
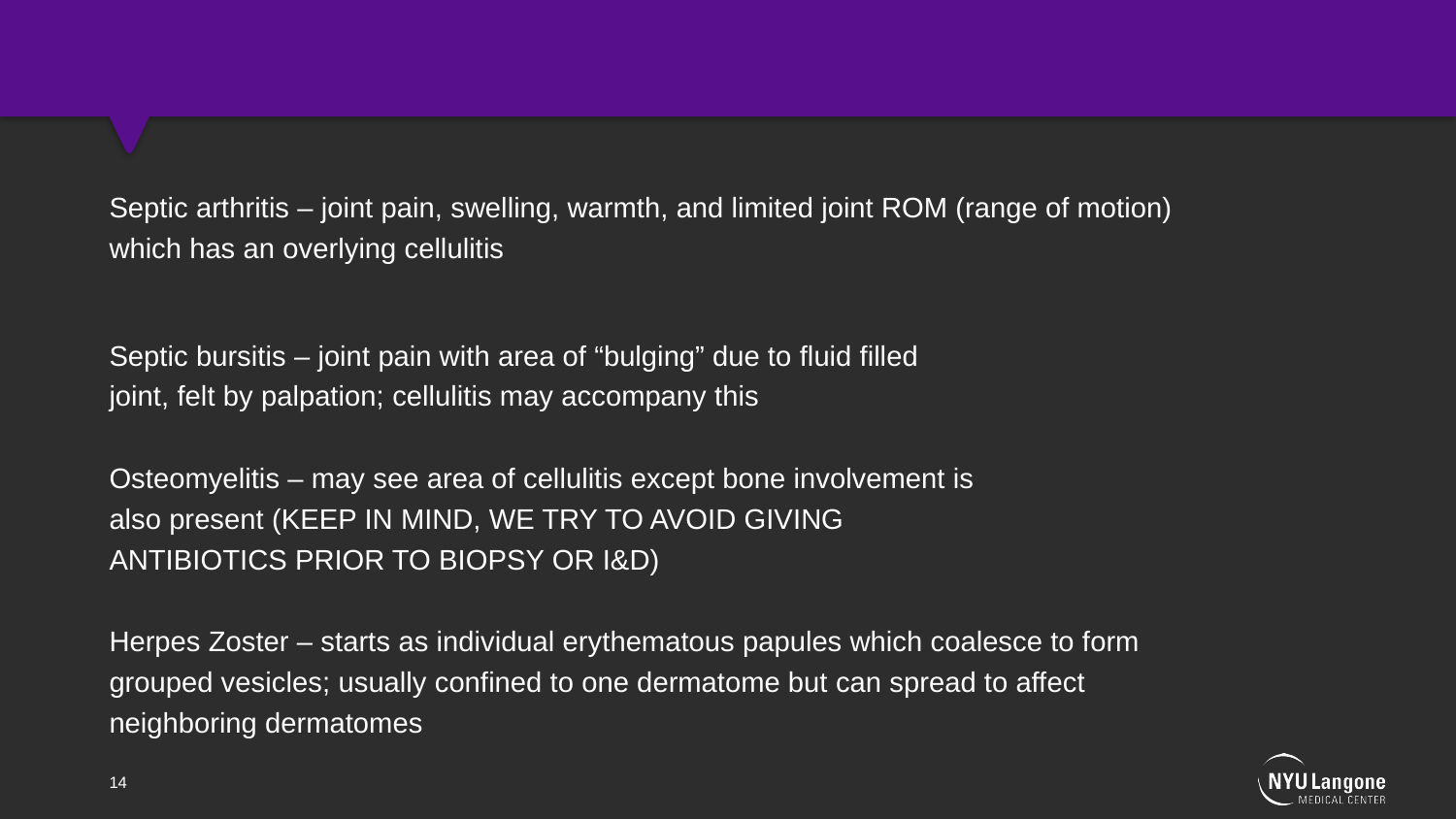

Septic arthritis – joint pain, swelling, warmth, and limited joint ROM (range of motion)
which has an overlying cellulitis
Septic bursitis – joint pain with area of “bulging” due to fluid filled
joint, felt by palpation; cellulitis may accompany this
Osteomyelitis – may see area of cellulitis except bone involvement is
also present (KEEP IN MIND, WE TRY TO AVOID GIVING
ANTIBIOTICS PRIOR TO BIOPSY OR I&D)
Herpes Zoster – starts as individual erythematous papules which coalesce to form grouped vesicles; usually confined to one dermatome but can spread to affect neighboring dermatomes
14

## Slide 15
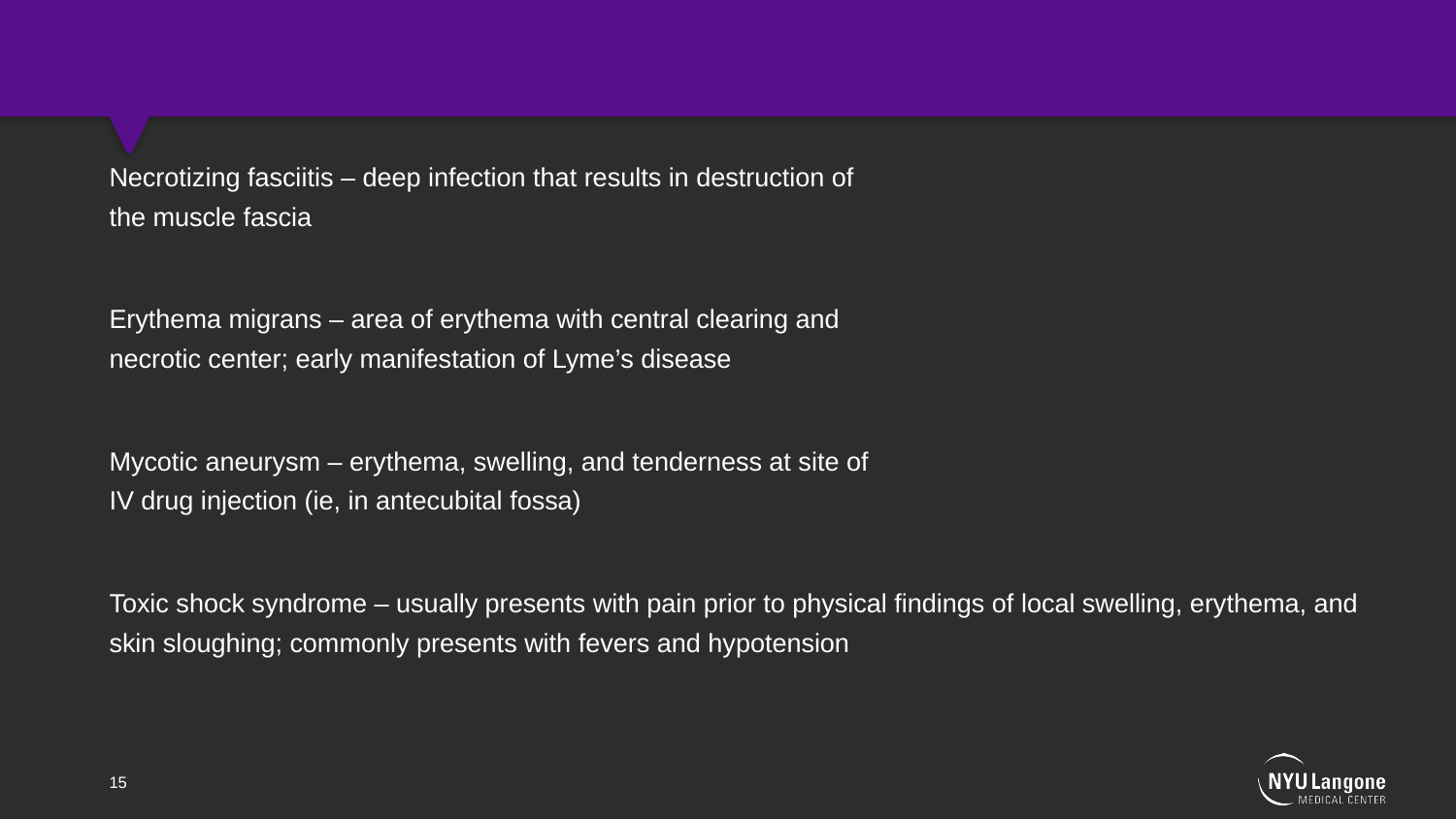

Necrotizing fasciitis – deep infection that results in destruction of
the muscle fascia
Erythema migrans – area of erythema with central clearing and
necrotic center; early manifestation of Lyme’s disease
Mycotic aneurysm – erythema, swelling, and tenderness at site of
IV drug injection (ie, in antecubital fossa)
Toxic shock syndrome – usually presents with pain prior to physical findings of local swelling, erythema, and
skin sloughing; commonly presents with fevers and hypotension
15

## Slide 16
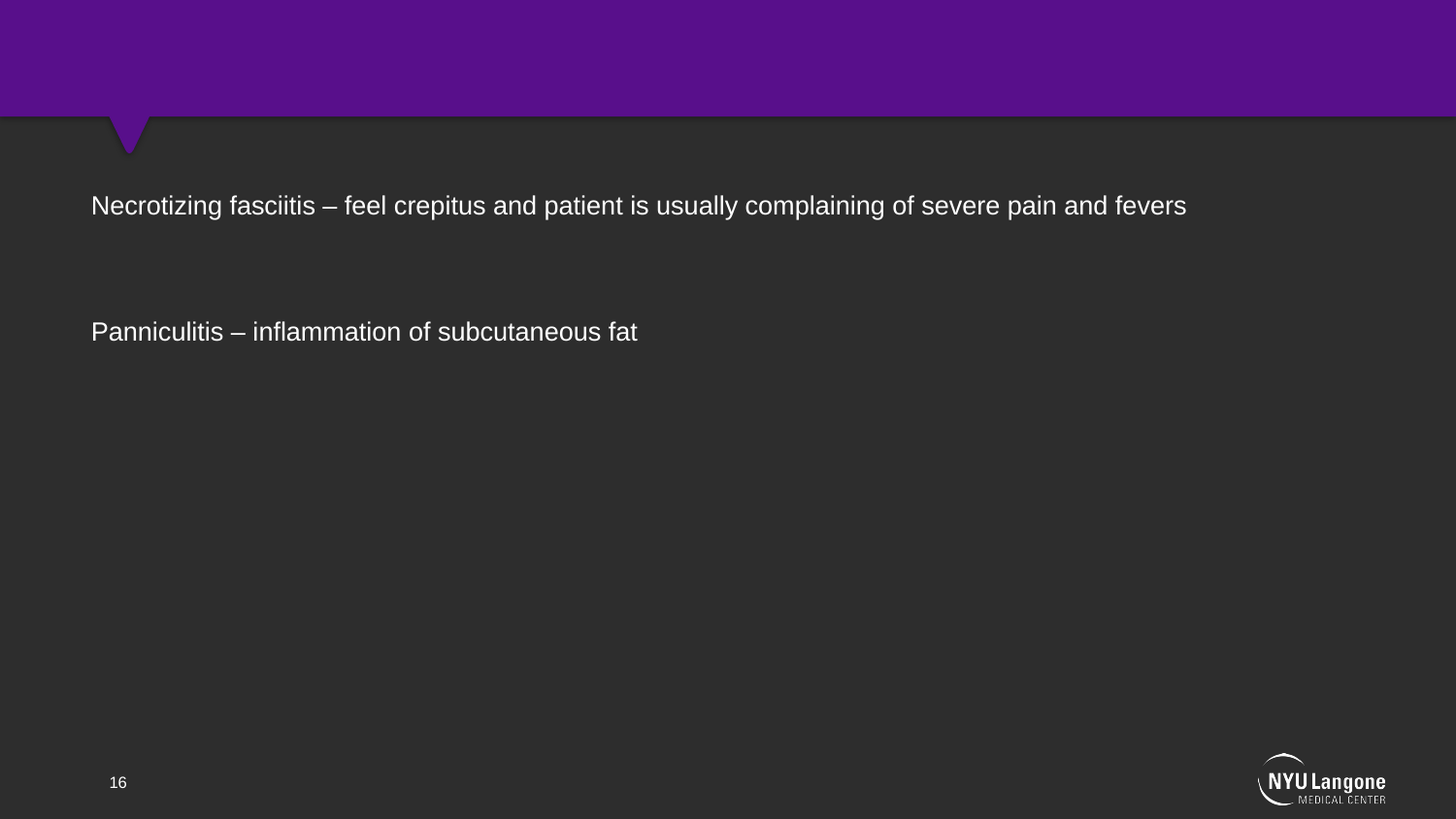

Necrotizing fasciitis – feel crepitus and patient is usually complaining of severe pain and fevers
Panniculitis – inflammation of subcutaneous fat
16

## Slide 17
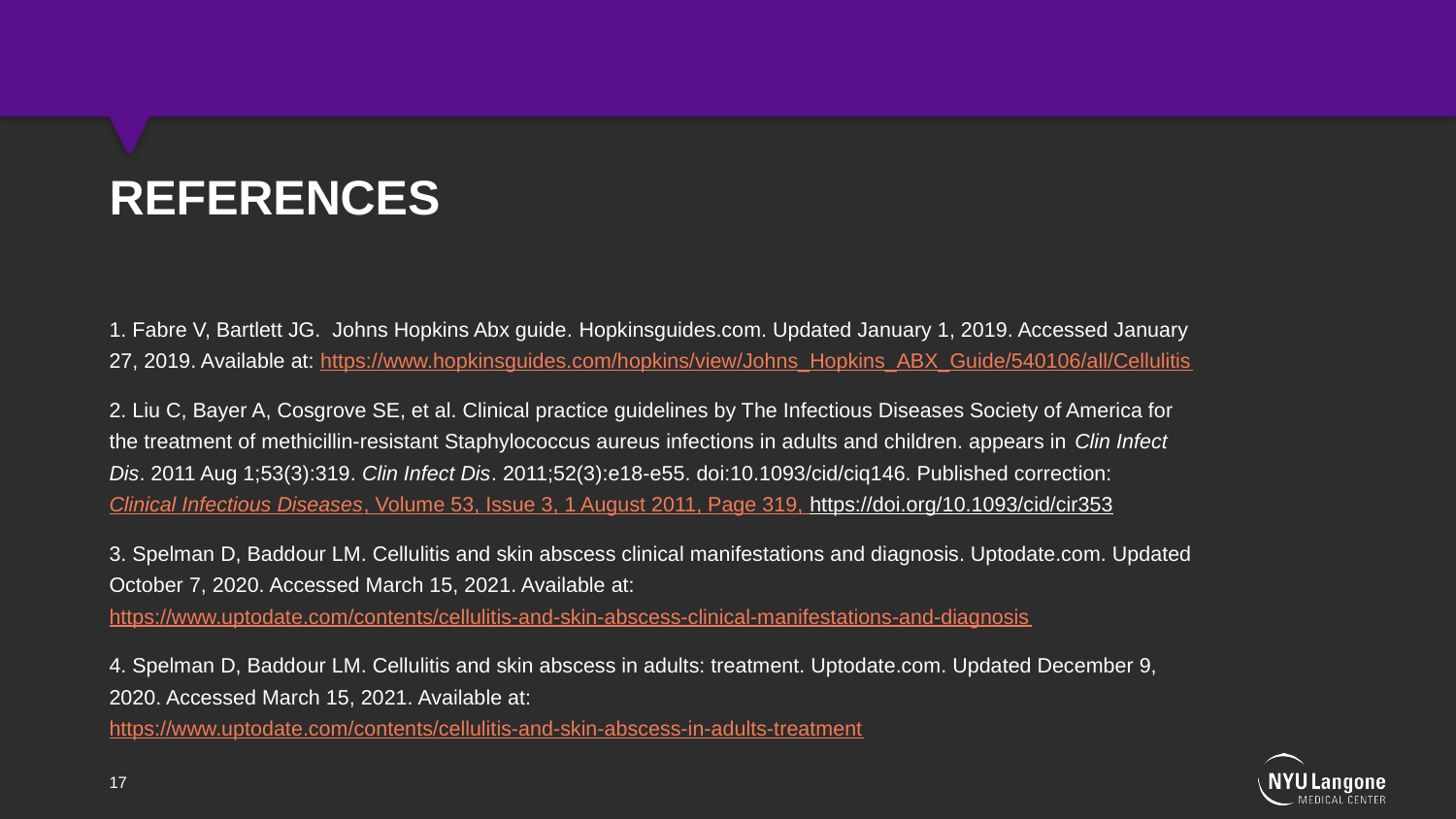

# REFERENCES
1. Fabre V, Bartlett JG.  Johns Hopkins Abx guide. Hopkinsguides.com. Updated January 1, 2019. Accessed January 27, 2019. Available at: https://www.hopkinsguides.com/hopkins/view/Johns_Hopkins_ABX_Guide/540106/all/Cellulitis
2. Liu C, Bayer A, Cosgrove SE, et al. Clinical practice guidelines by The Infectious Diseases Society of America for the treatment of methicillin-resistant Staphylococcus aureus infections in adults and children. appears in Clin Infect Dis. 2011 Aug 1;53(3):319. Clin Infect Dis. 2011;52(3):e18-e55. doi:10.1093/cid/ciq146. Published correction: Clinical Infectious Diseases, Volume 53, Issue 3, 1 August 2011, Page 319, https://doi.org/10.1093/cid/cir353
3. Spelman D, Baddour LM. Cellulitis and skin abscess clinical manifestations and diagnosis. Uptodate.com. Updated October 7, 2020. Accessed March 15, 2021. Available at: https://www.uptodate.com/contents/cellulitis-and-skin-abscess-clinical-manifestations-and-diagnosis
4. Spelman D, Baddour LM. Cellulitis and skin abscess in adults: treatment. Uptodate.com. Updated December 9, 2020. Accessed March 15, 2021. Available at: https://www.uptodate.com/contents/cellulitis-and-skin-abscess-in-adults-treatment
17
