# Supplementary material for: An Observation Medicine Curriculum for Emergency Medicine Education
Source: J Educ Teach Emerg Med. 2021 Apr 19;6(2):C1–C72. doi: 10.21980/J87P92 (PMC10332786; doi:10.21980/J87P92)
Supplement: Supplementary file 2 — Please see associated PowerPoint file [file jetem-6-2-c1-supp2.pptx]

## Slide 1
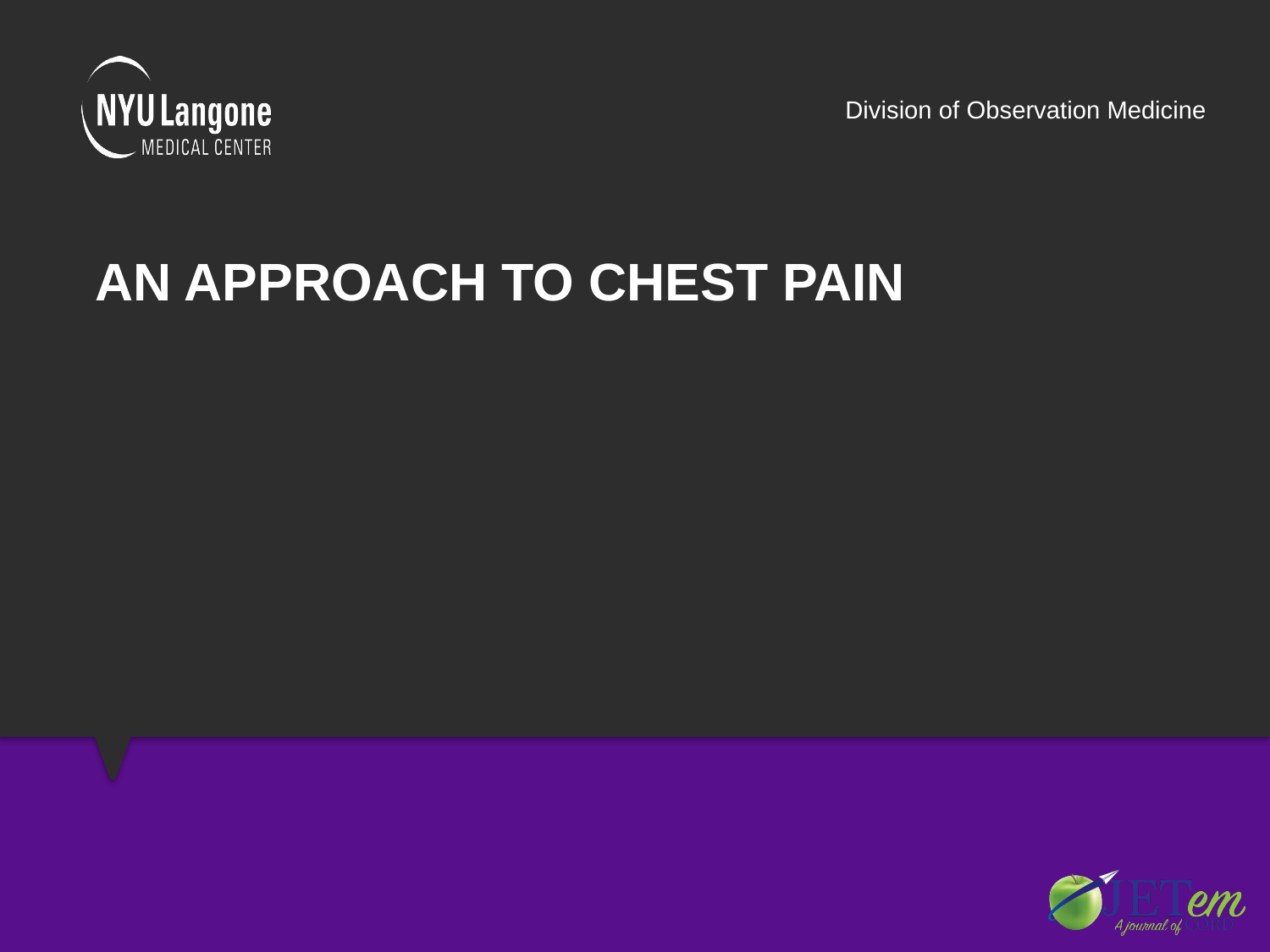

Division of Observation Medicine
# An Approach to Chest Pain

## Slide 2
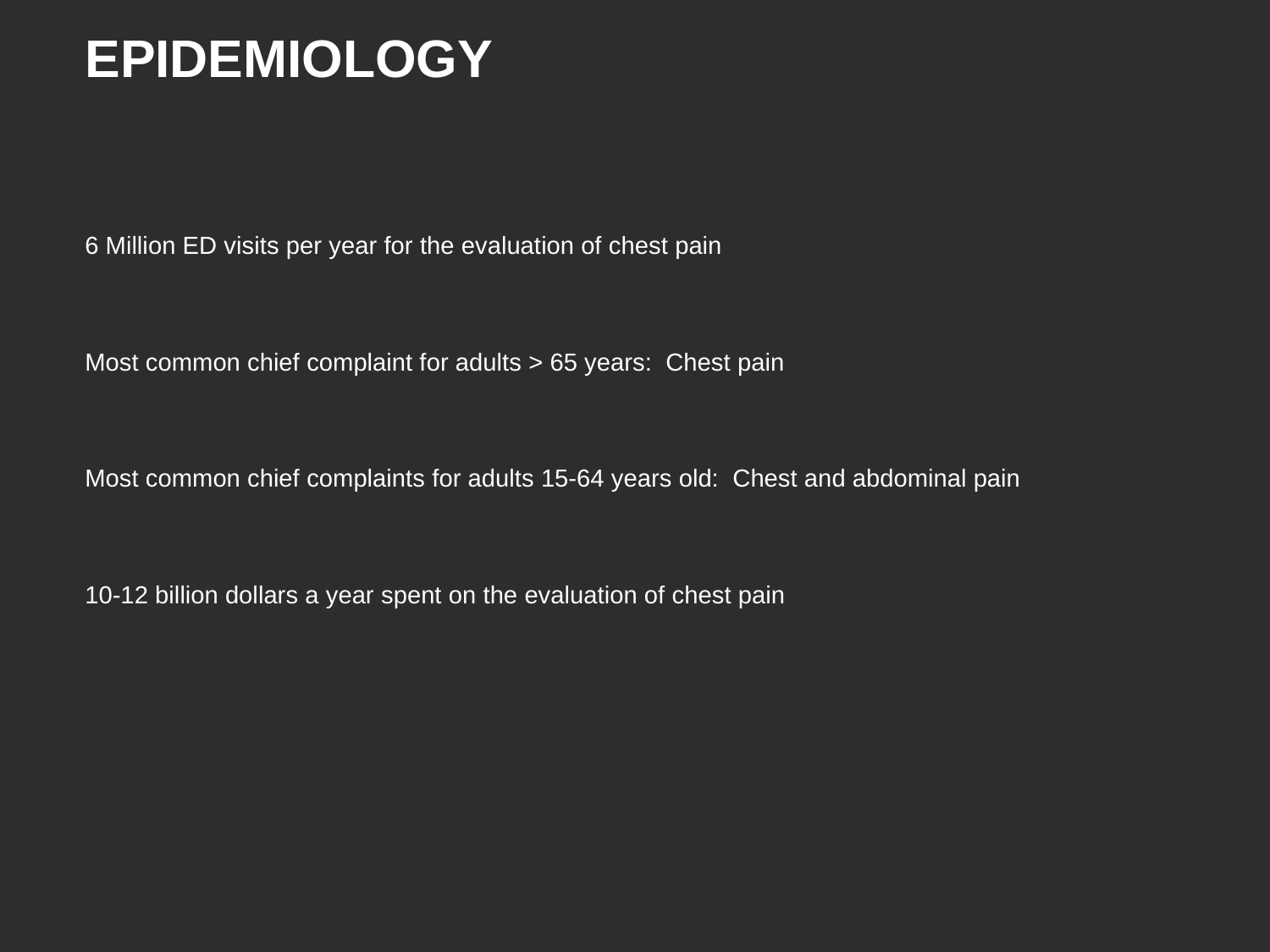

# Epidemiology
6 Million ED visits per year for the evaluation of chest pain
Most common chief complaint for adults > 65 years: Chest pain
Most common chief complaints for adults 15-64 years old: Chest and abdominal pain
10-12 billion dollars a year spent on the evaluation of chest pain

## Slide 3
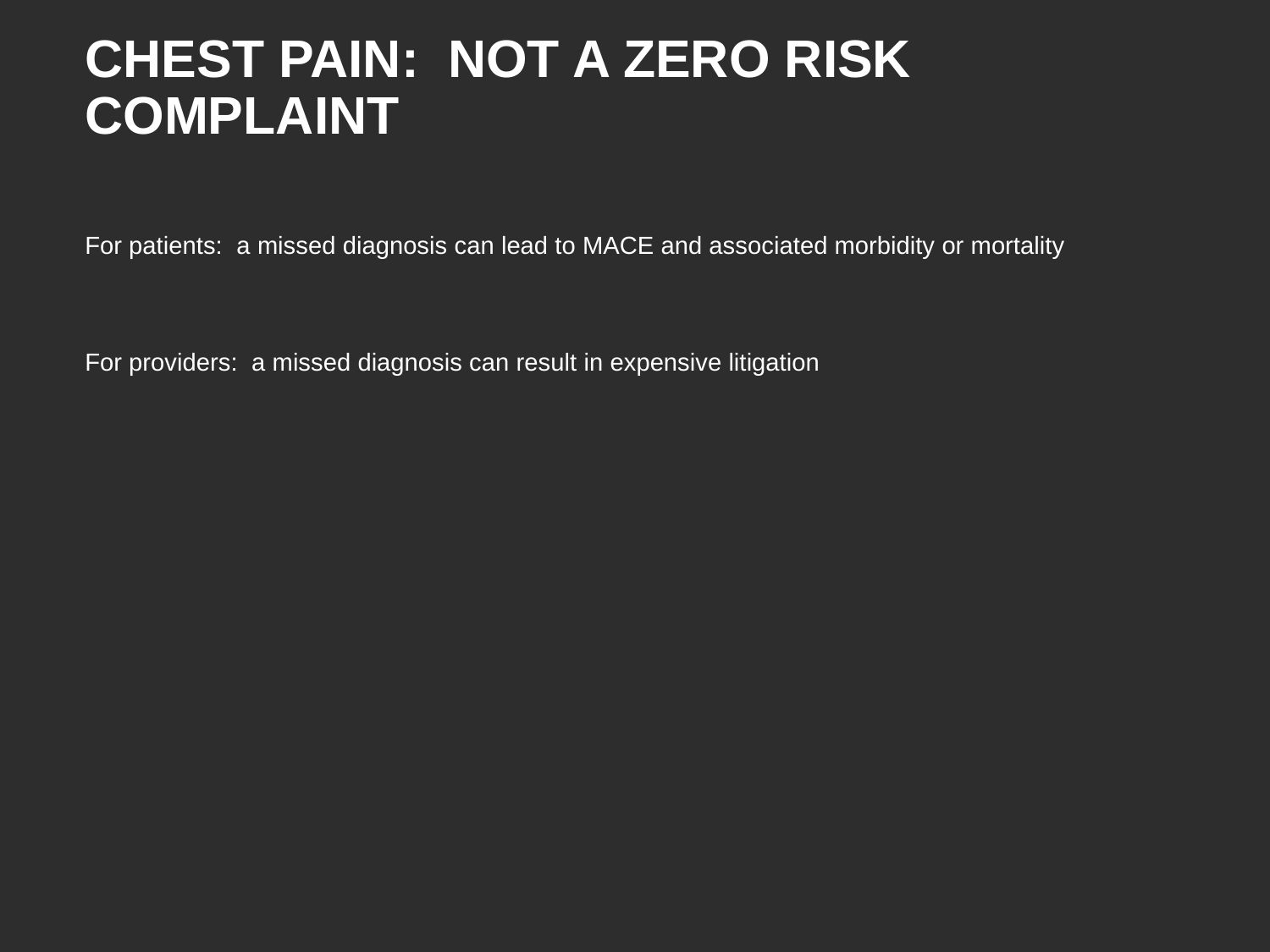

# Chest Pain: Not a zero risk complaint
For patients: a missed diagnosis can lead to MACE and associated morbidity or mortality
For providers: a missed diagnosis can result in expensive litigation

## Slide 4
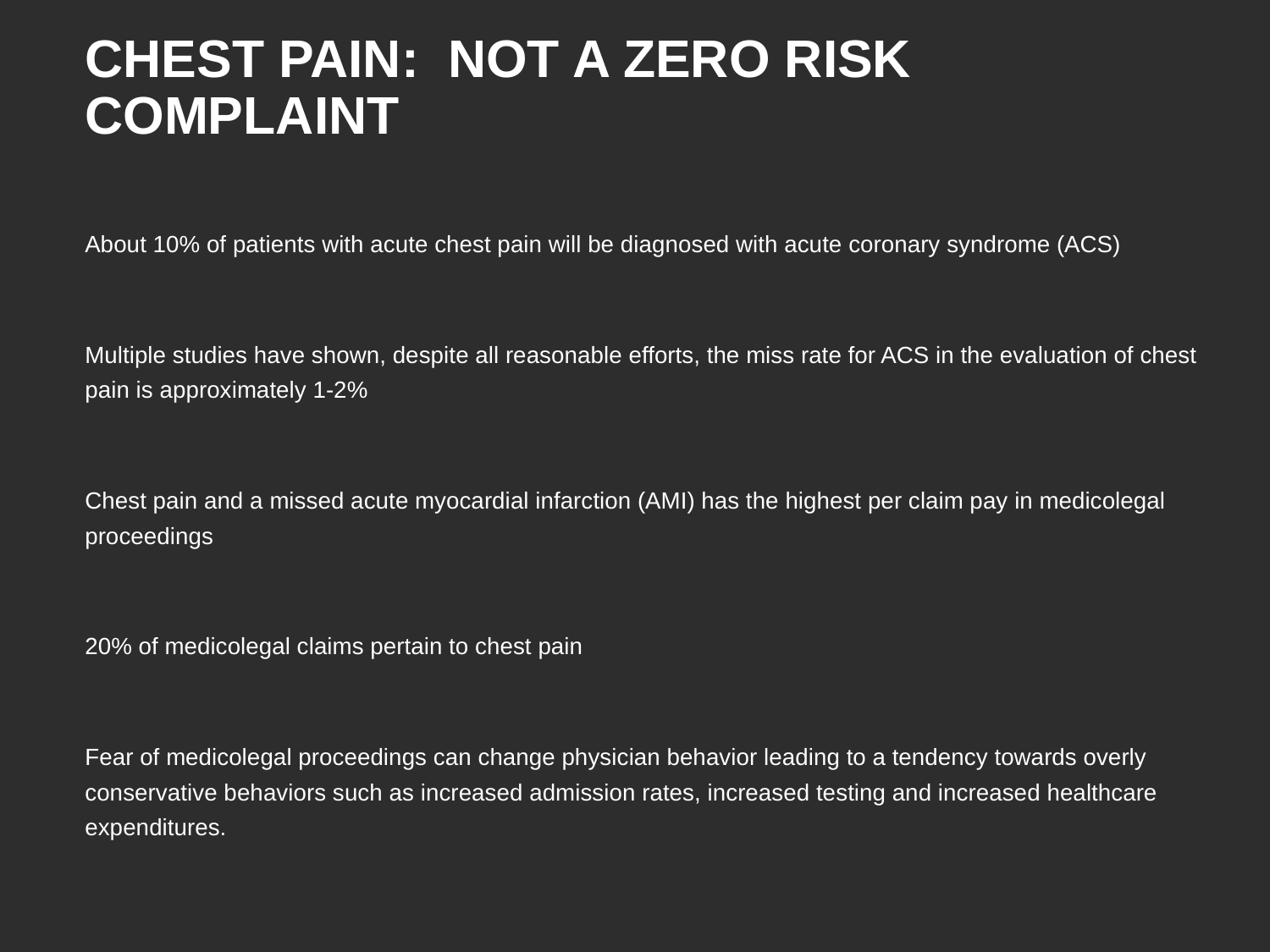

# Chest Pain: Not a zero risk complaint
About 10% of patients with acute chest pain will be diagnosed with acute coronary syndrome (ACS)
Multiple studies have shown, despite all reasonable efforts, the miss rate for ACS in the evaluation of chest pain is approximately 1-2%
Chest pain and a missed acute myocardial infarction (AMI) has the highest per claim pay in medicolegal proceedings
20% of medicolegal claims pertain to chest pain
Fear of medicolegal proceedings can change physician behavior leading to a tendency towards overly conservative behaviors such as increased admission rates, increased testing and increased healthcare expenditures.

## Slide 5
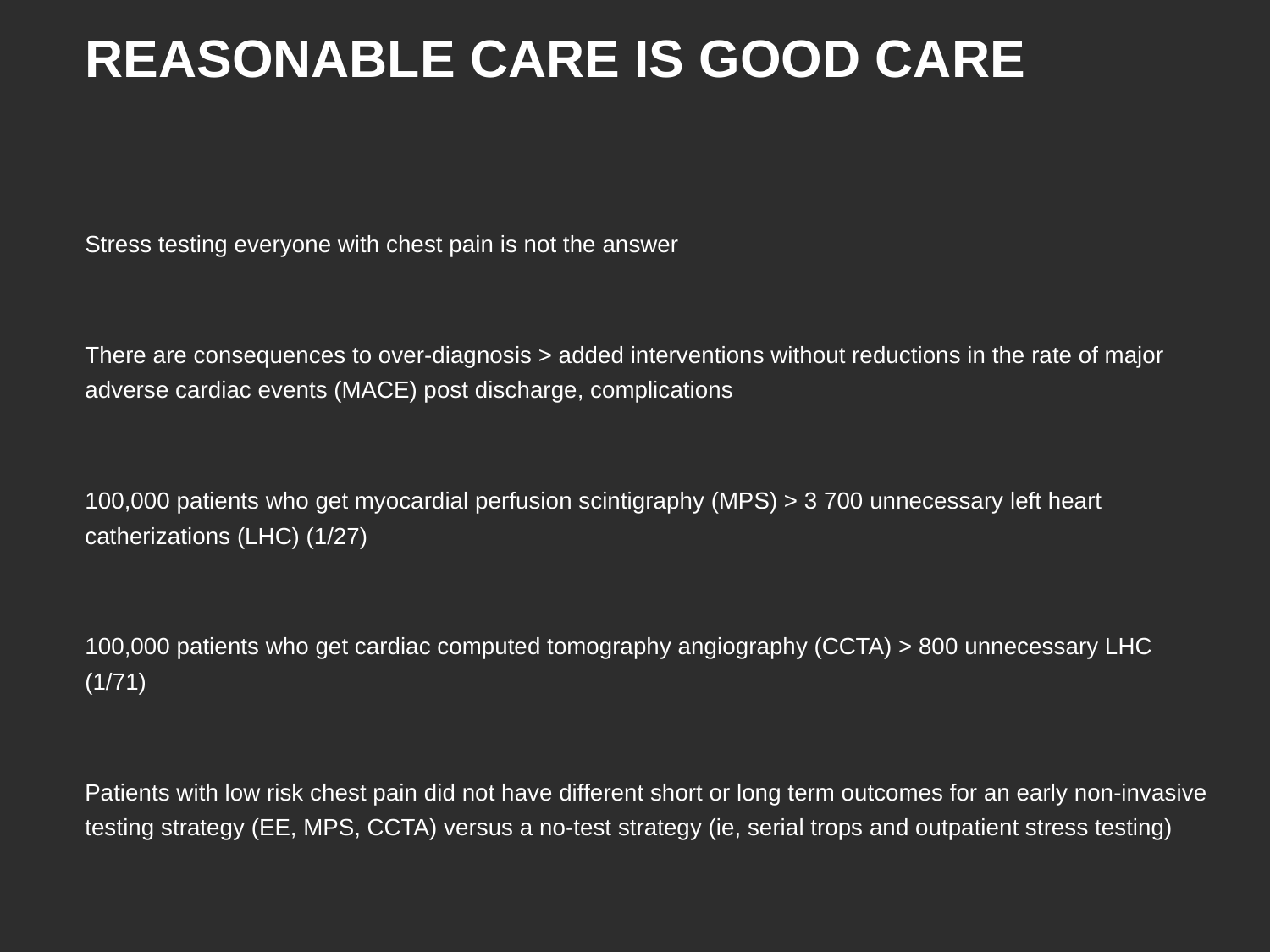

# Reasonable care is good care
Stress testing everyone with chest pain is not the answer
There are consequences to over-diagnosis > added interventions without reductions in the rate of major adverse cardiac events (MACE) post discharge, complications
100,000 patients who get myocardial perfusion scintigraphy (MPS) > 3 700 unnecessary left heart catherizations (LHC) (1/27)
100,000 patients who get cardiac computed tomography angiography (CCTA) > 800 unnecessary LHC (1/71)
Patients with low risk chest pain did not have different short or long term outcomes for an early non-invasive testing strategy (EE, MPS, CCTA) versus a no-test strategy (ie, serial trops and outpatient stress testing)

## Slide 6
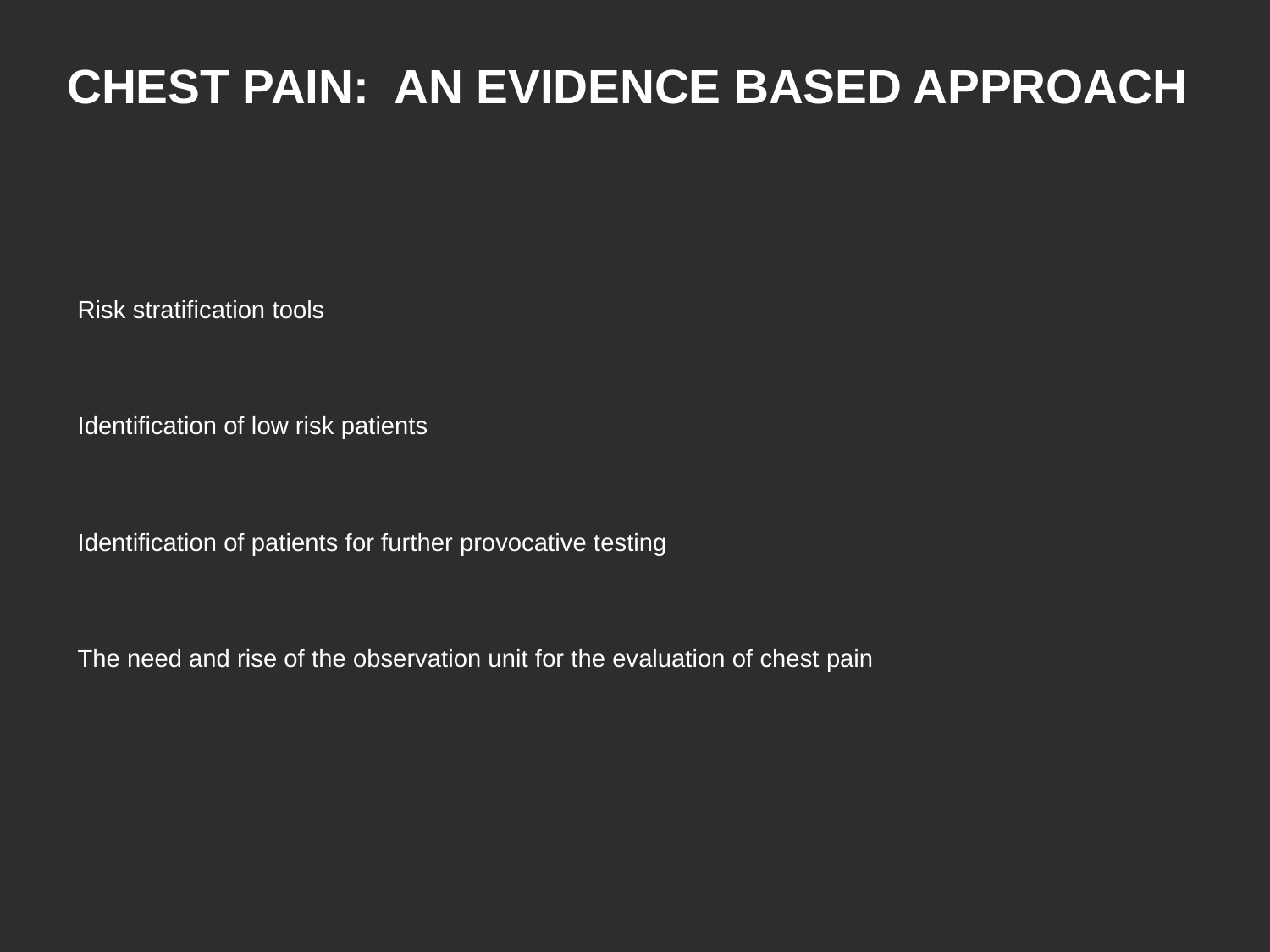

# Chest Pain: An evidence based approach
Risk stratification tools
Identification of low risk patients
Identification of patients for further provocative testing
The need and rise of the observation unit for the evaluation of chest pain

## Slide 7
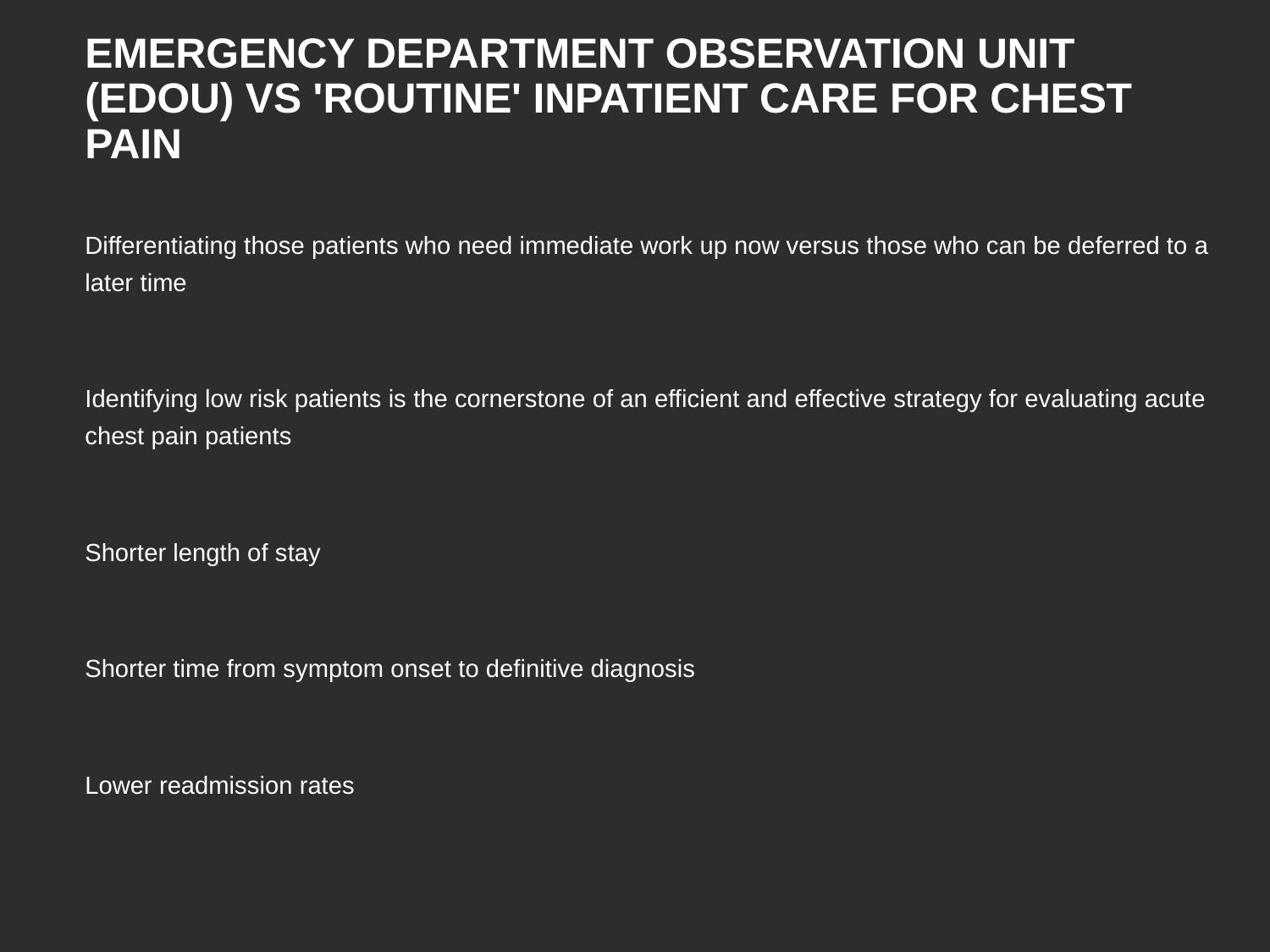

# Emergency department observation unit (EDOU) vs 'routine' inpatient care for chest pain
Differentiating those patients who need immediate work up now versus those who can be deferred to a later time
Identifying low risk patients is the cornerstone of an efficient and effective strategy for evaluating acute chest pain patients
Shorter length of stay
Shorter time from symptom onset to definitive diagnosis
Lower readmission rates

## Slide 8
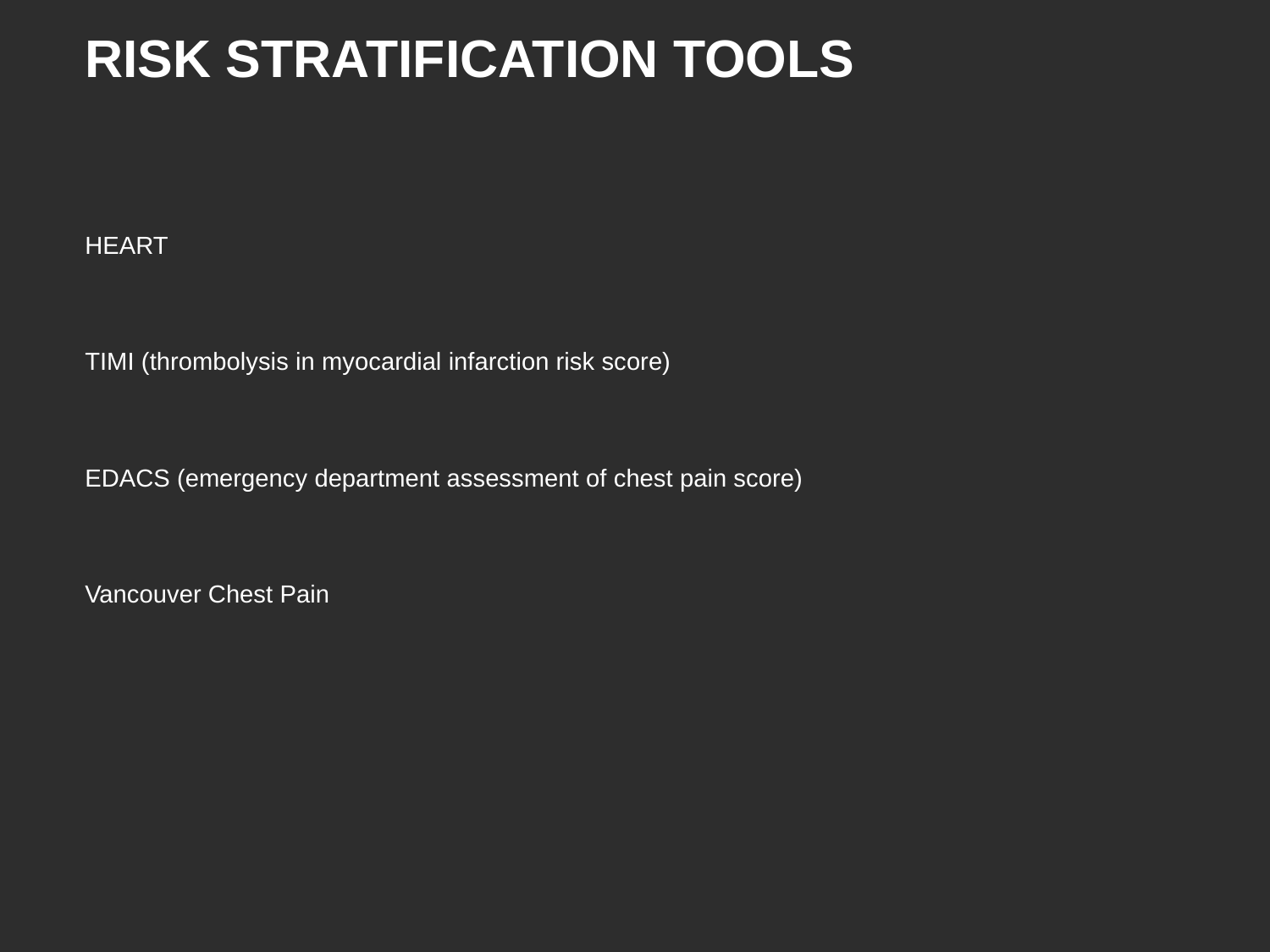

# Risk stratification tools
HEART
TIMI (thrombolysis in myocardial infarction risk score)
EDACS (emergency department assessment of chest pain score)
Vancouver Chest Pain

## Slide 9
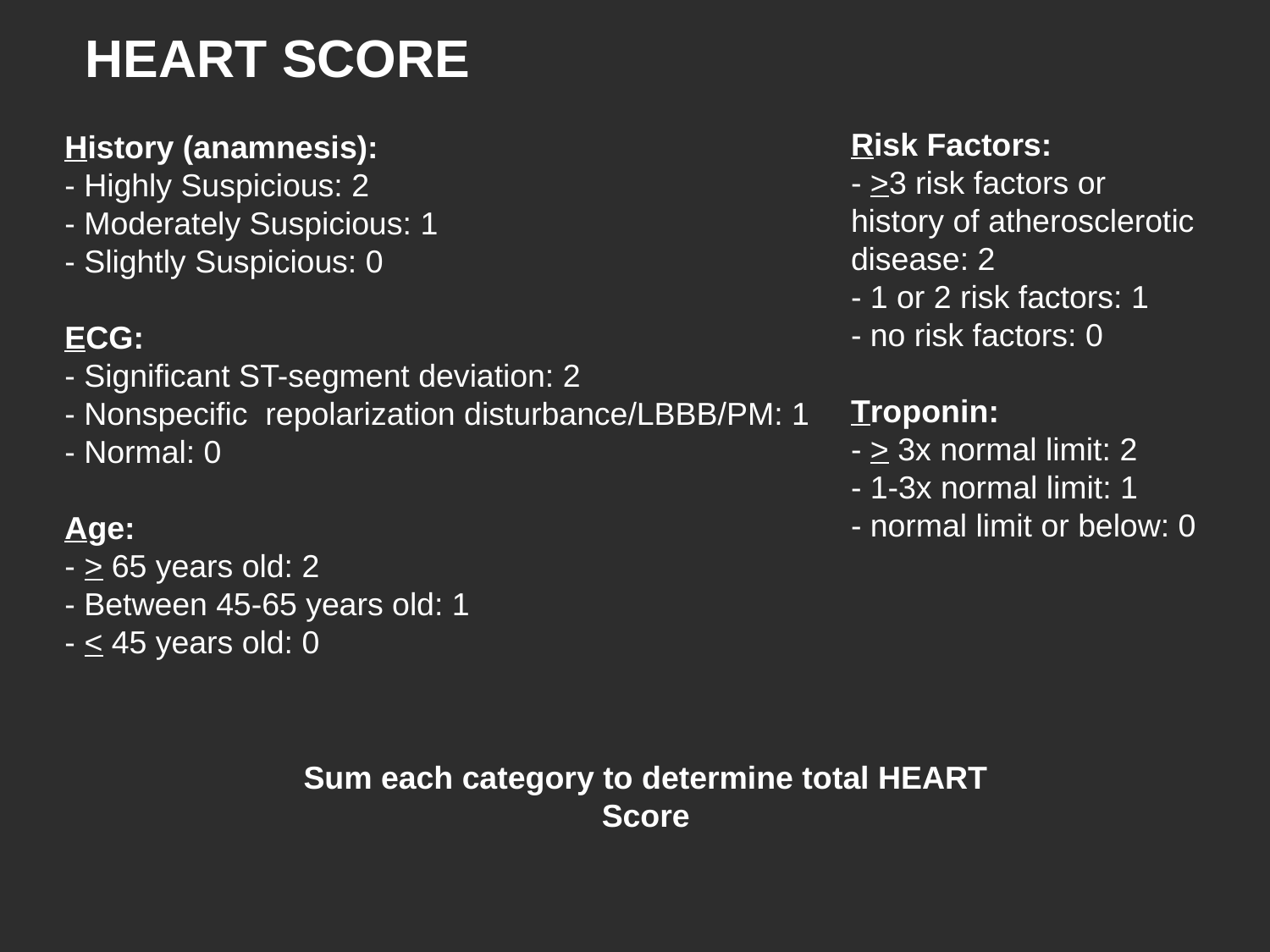

# HEART score
Risk Factors:
- >3 risk factors or history of atherosclerotic disease: 2
- 1 or 2 risk factors: 1
- no risk factors: 0
Troponin:
- > 3x normal limit: 2
- 1-3x normal limit: 1
- normal limit or below: 0
History (anamnesis):
- Highly Suspicious: 2
- Moderately Suspicious: 1
- Slightly Suspicious: 0
ECG:
- Significant ST-segment deviation: 2
- Nonspecific  repolarization disturbance/LBBB/PM: 1
- Normal: 0
Age:
- > 65 years old: 2
- Between 45-65 years old: 1
- < 45 years old: 0
Sum each category to determine total HEART Score

## Slide 10
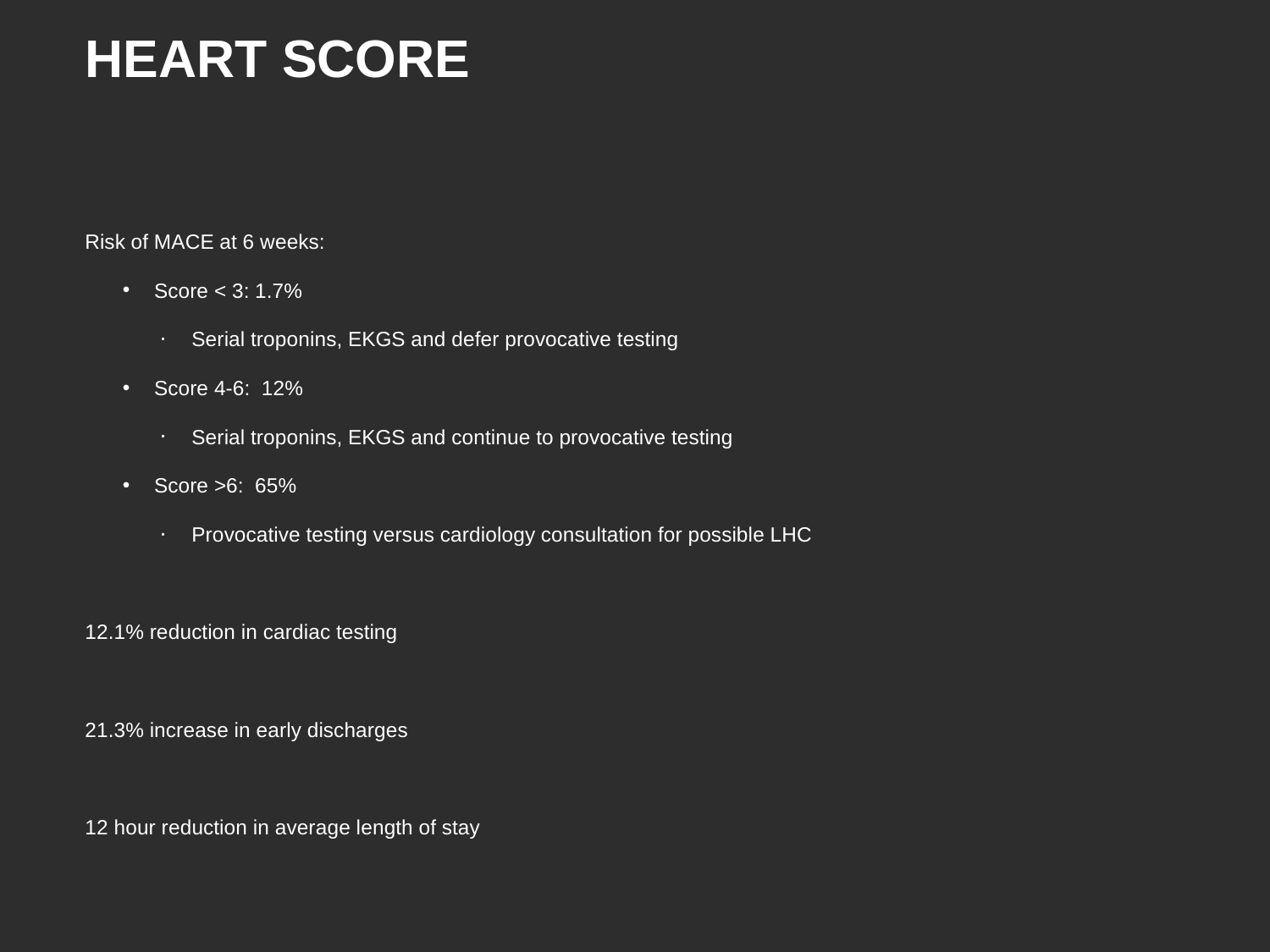

# HEART score
Risk of MACE at 6 weeks:
Score < 3: 1.7%
Serial troponins, EKGS and defer provocative testing
Score 4-6: 12%
Serial troponins, EKGS and continue to provocative testing
Score >6: 65%
Provocative testing versus cardiology consultation for possible LHC
12.1% reduction in cardiac testing
21.3% increase in early discharges
12 hour reduction in average length of stay

## Slide 11
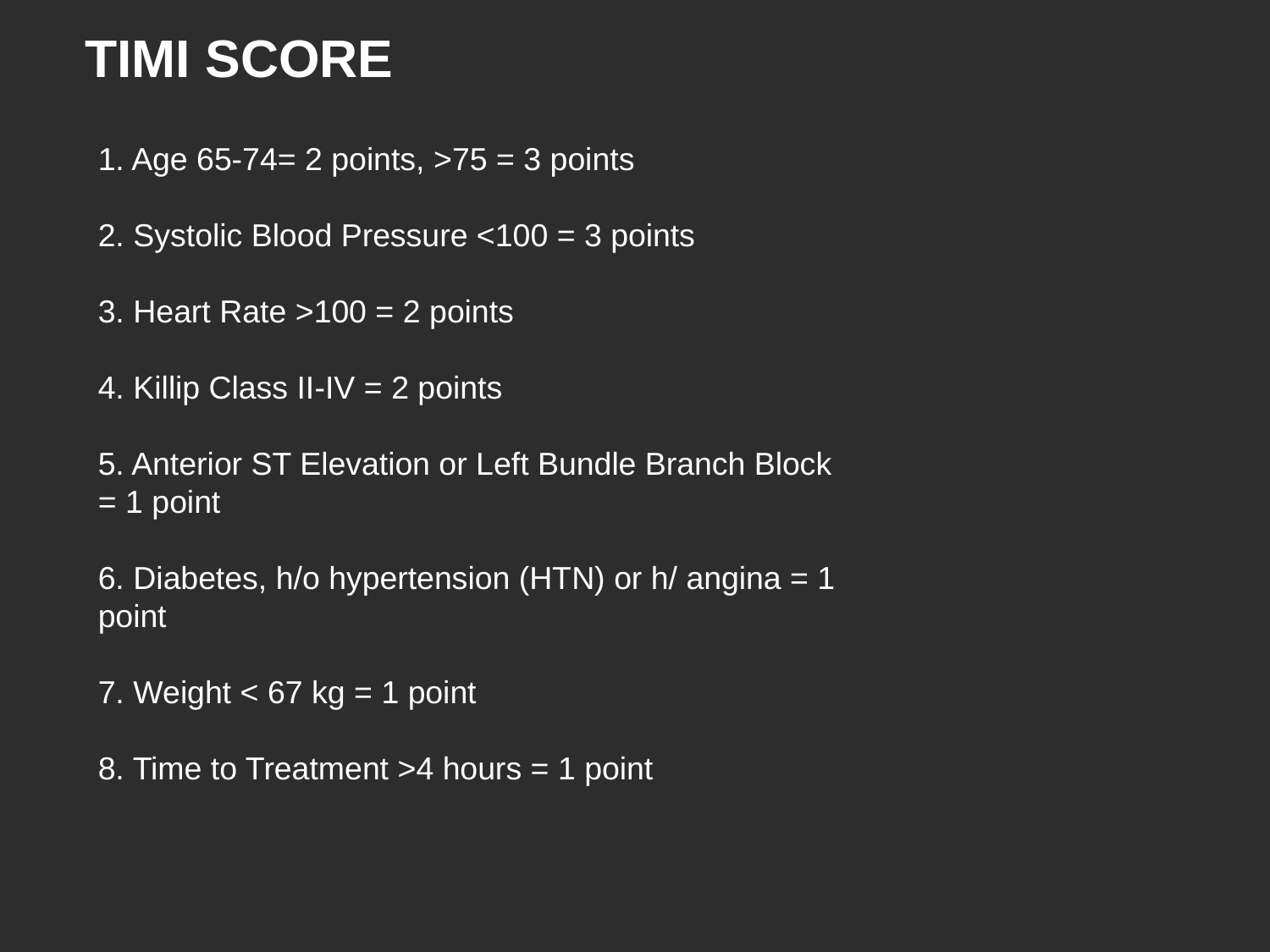

# TIMI Score
1. Age 65-74= 2 points, >75 = 3 points
2. Systolic Blood Pressure <100 = 3 points
3. Heart Rate >100 = 2 points
4. Killip Class II-IV = 2 points
5. Anterior ST Elevation or Left Bundle Branch Block = 1 point
6. Diabetes, h/o hypertension (HTN) or h/ angina = 1 point
7. Weight < 67 kg = 1 point
8. Time to Treatment >4 hours = 1 point

## Slide 12
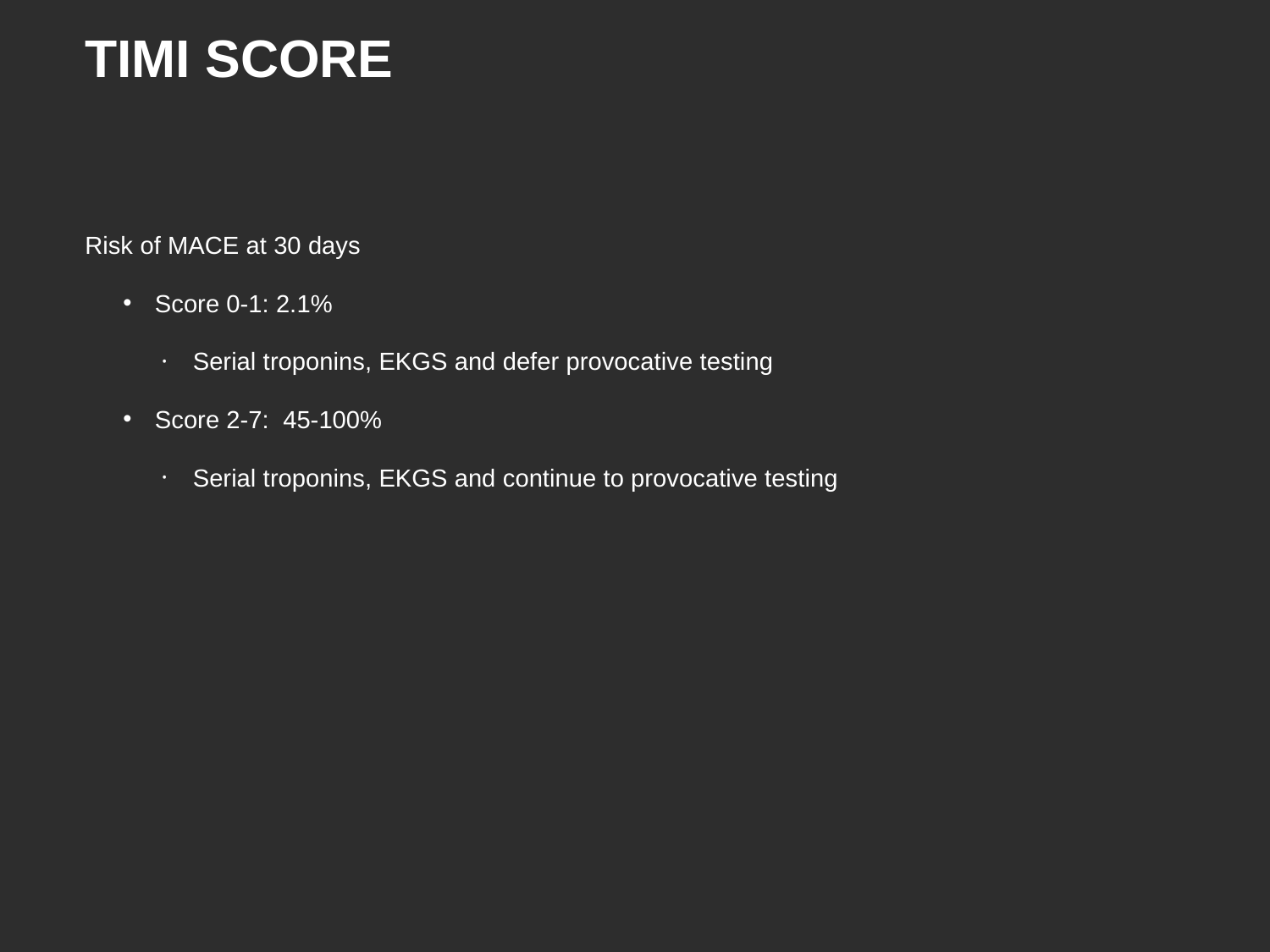

# TIMI Score
Risk of MACE at 30 days
Score 0-1: 2.1%
Serial troponins, EKGS and defer provocative testing
Score 2-7: 45-100%
Serial troponins, EKGS and continue to provocative testing

## Slide 13
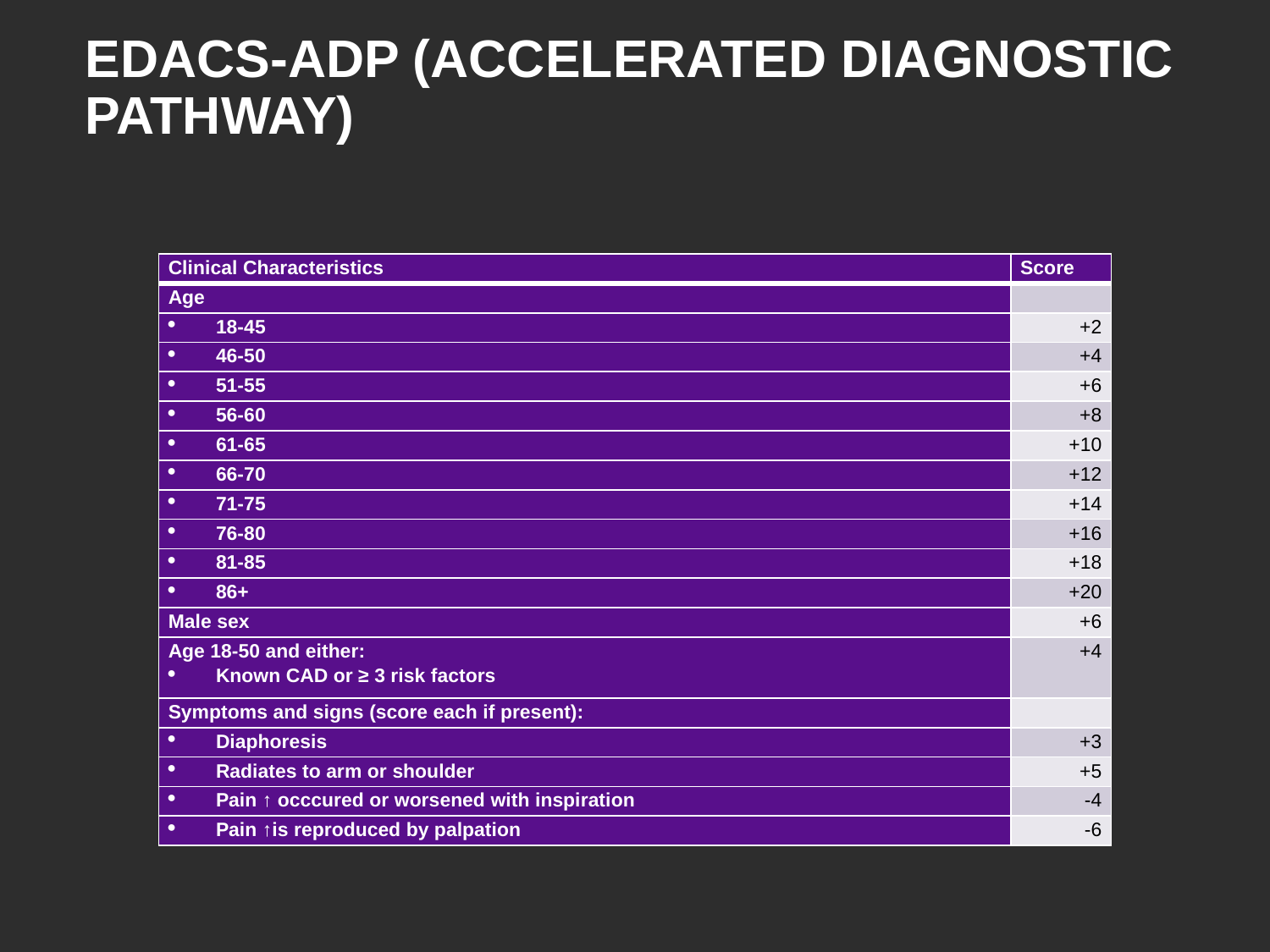

# EDACS-ADP (accelerated diagnostic pathway)
| Clinical Characteristics | Score |
| --- | --- |
| Age | |
| 18-45 | +2 |
| 46-50 | +4 |
| 51-55 | +6 |
| 56-60 | +8 |
| 61-65 | +10 |
| 66-70 | +12 |
| 71-75 | +14 |
| 76-80 | +16 |
| 81-85 | +18 |
| 86+ | +20 |
| Male sex | +6 |
| Age 18-50 and either: Known CAD or ≥ 3 risk factors | +4 |
| Symptoms and signs (score each if present): | |
| Diaphoresis | +3 |
| Radiates to arm or shoulder | +5 |
| Pain ↑ occcured or worsened with inspiration | -4 |
| Pain ↑is reproduced by palpation | -6 |

## Slide 14
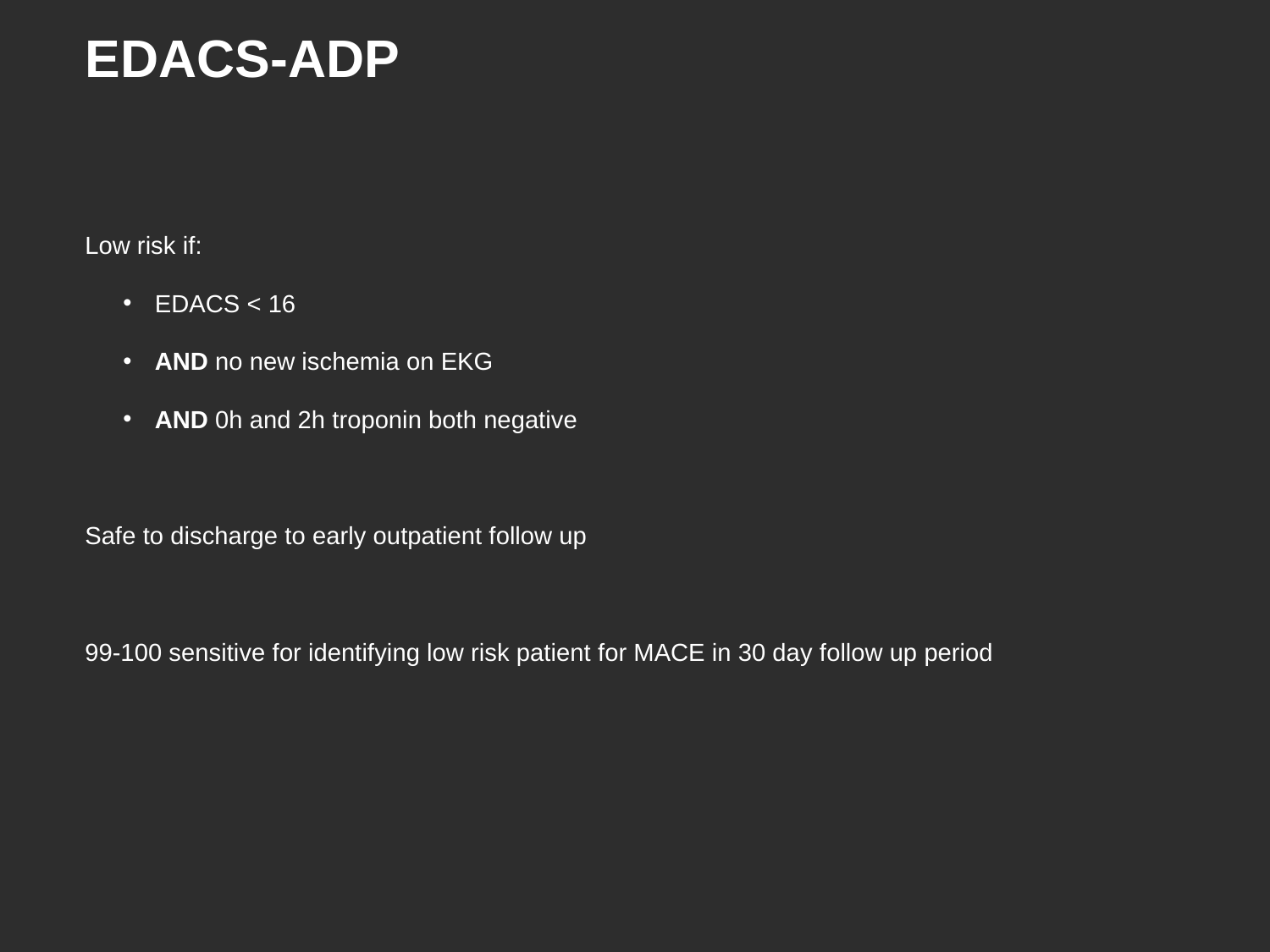

# EDACS-ADP
Low risk if:
EDACS < 16
AND no new ischemia on EKG
AND 0h and 2h troponin both negative
Safe to discharge to early outpatient follow up
99-100 sensitive for identifying low risk patient for MACE in 30 day follow up period

## Slide 15
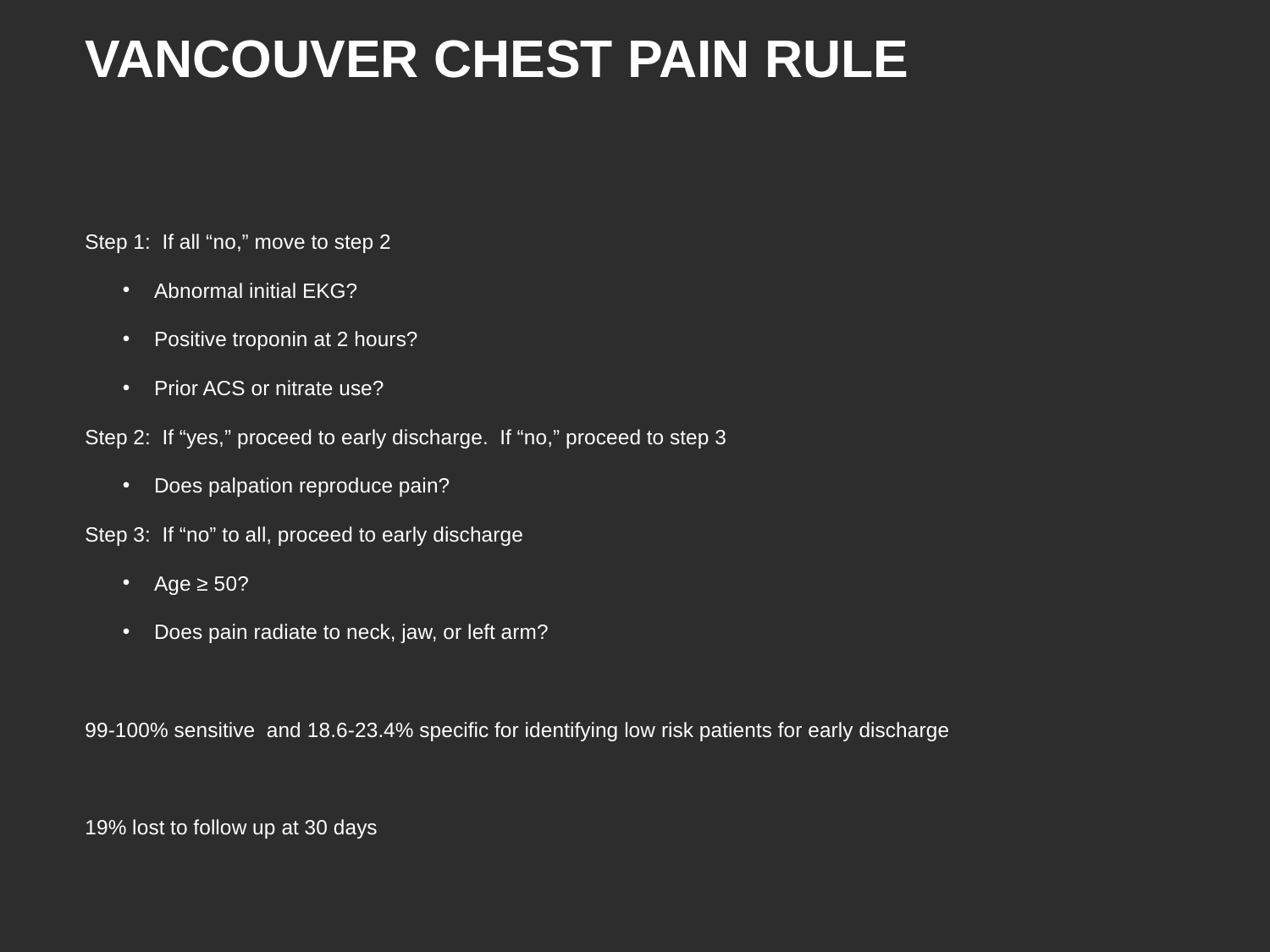

# Vancouver Chest Pain Rule
Step 1: If all “no,” move to step 2
Abnormal initial EKG?
Positive troponin at 2 hours?
Prior ACS or nitrate use?
Step 2: If “yes,” proceed to early discharge. If “no,” proceed to step 3
Does palpation reproduce pain?
Step 3: If “no” to all, proceed to early discharge
Age ≥ 50?
Does pain radiate to neck, jaw, or left arm?
99-100% sensitive and 18.6-23.4% specific for identifying low risk patients for early discharge
19% lost to follow up at 30 days

## Slide 16
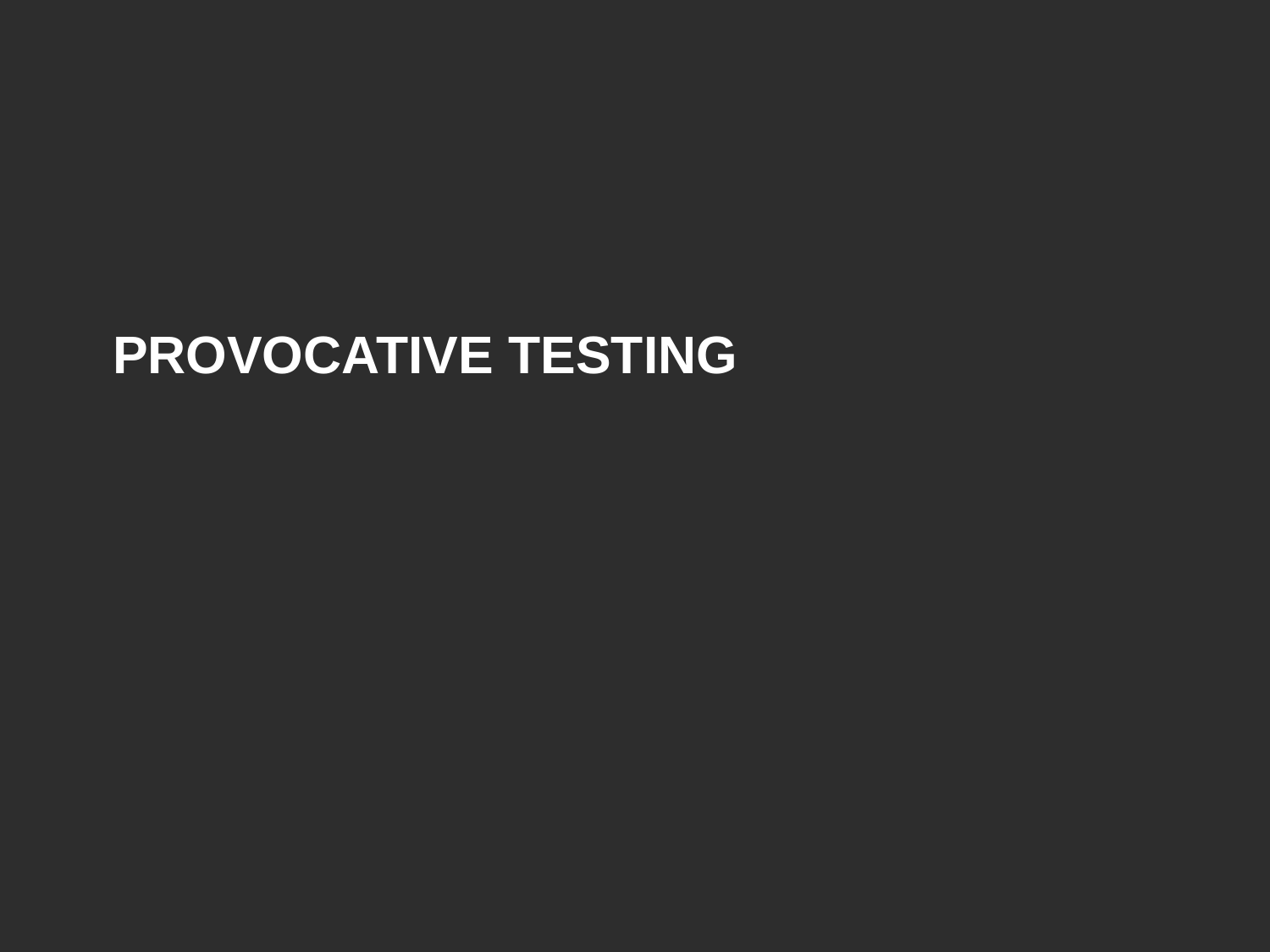

# Provocative Testing

## Slide 17
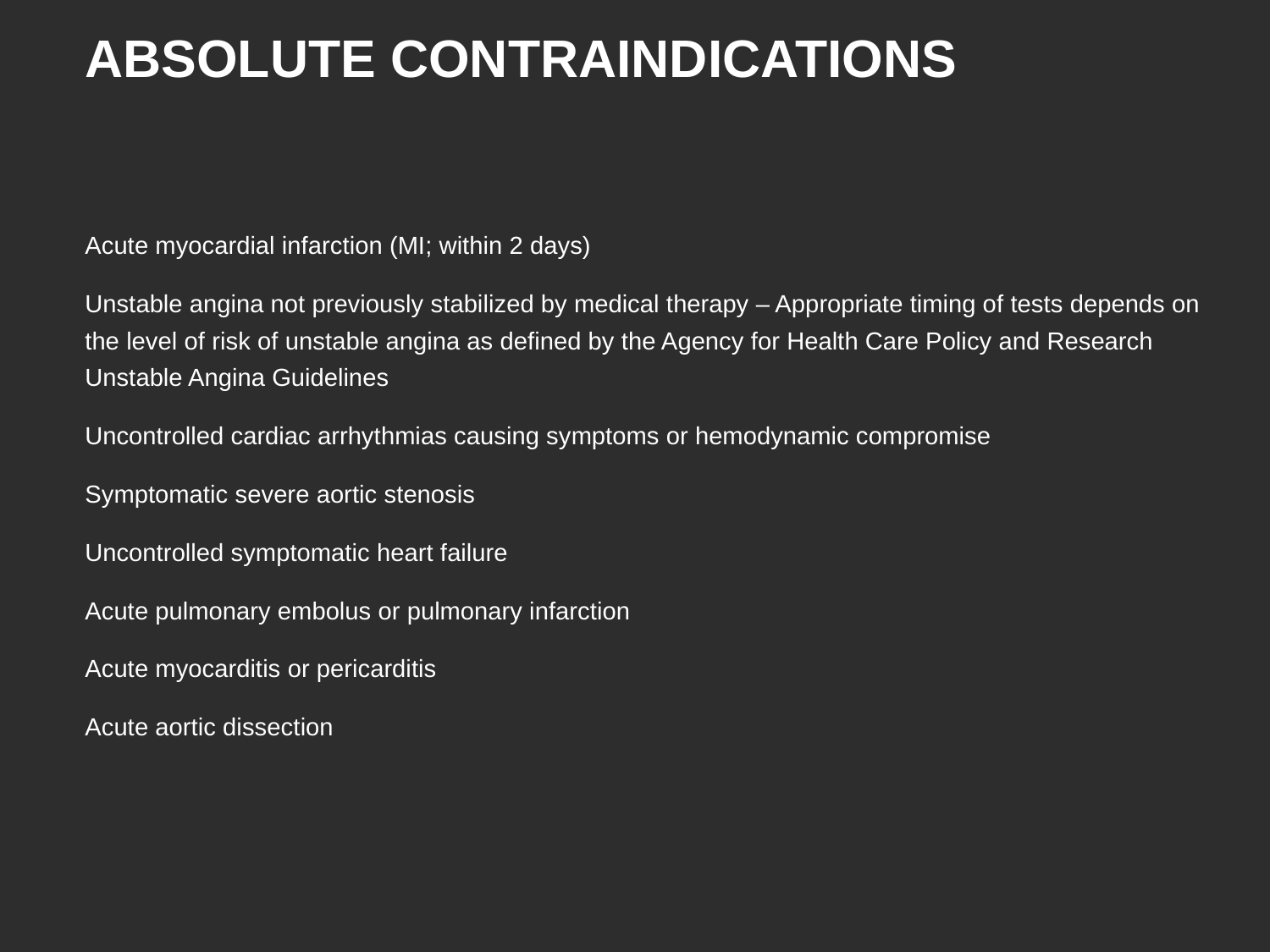

# Absolute Contraindications
Acute myocardial infarction (MI; within 2 days)
Unstable angina not previously stabilized by medical therapy – Appropriate timing of tests depends on the level of risk of unstable angina as defined by the Agency for Health Care Policy and Research Unstable Angina Guidelines
Uncontrolled cardiac arrhythmias causing symptoms or hemodynamic compromise
Symptomatic severe aortic stenosis
Uncontrolled symptomatic heart failure
Acute pulmonary embolus or pulmonary infarction
Acute myocarditis or pericarditis
Acute aortic dissection

## Slide 18
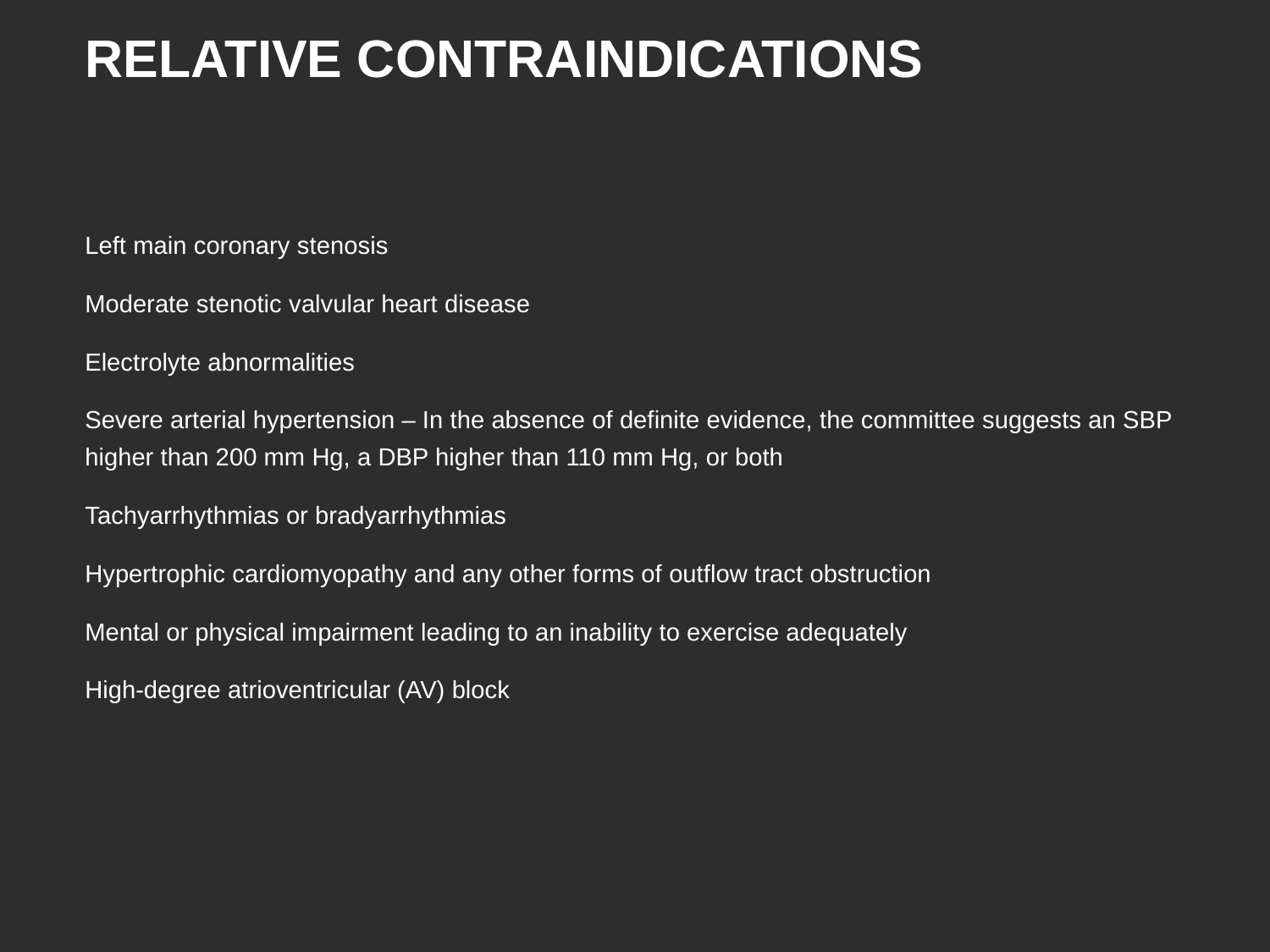

# Relative Contraindications
Left main coronary stenosis
Moderate stenotic valvular heart disease
Electrolyte abnormalities
Severe arterial hypertension – In the absence of definite evidence, the committee suggests an SBP higher than 200 mm Hg, a DBP higher than 110 mm Hg, or both
Tachyarrhythmias or bradyarrhythmias
Hypertrophic cardiomyopathy and any other forms of outflow tract obstruction
Mental or physical impairment leading to an inability to exercise adequately
High-degree atrioventricular (AV) block

## Slide 19
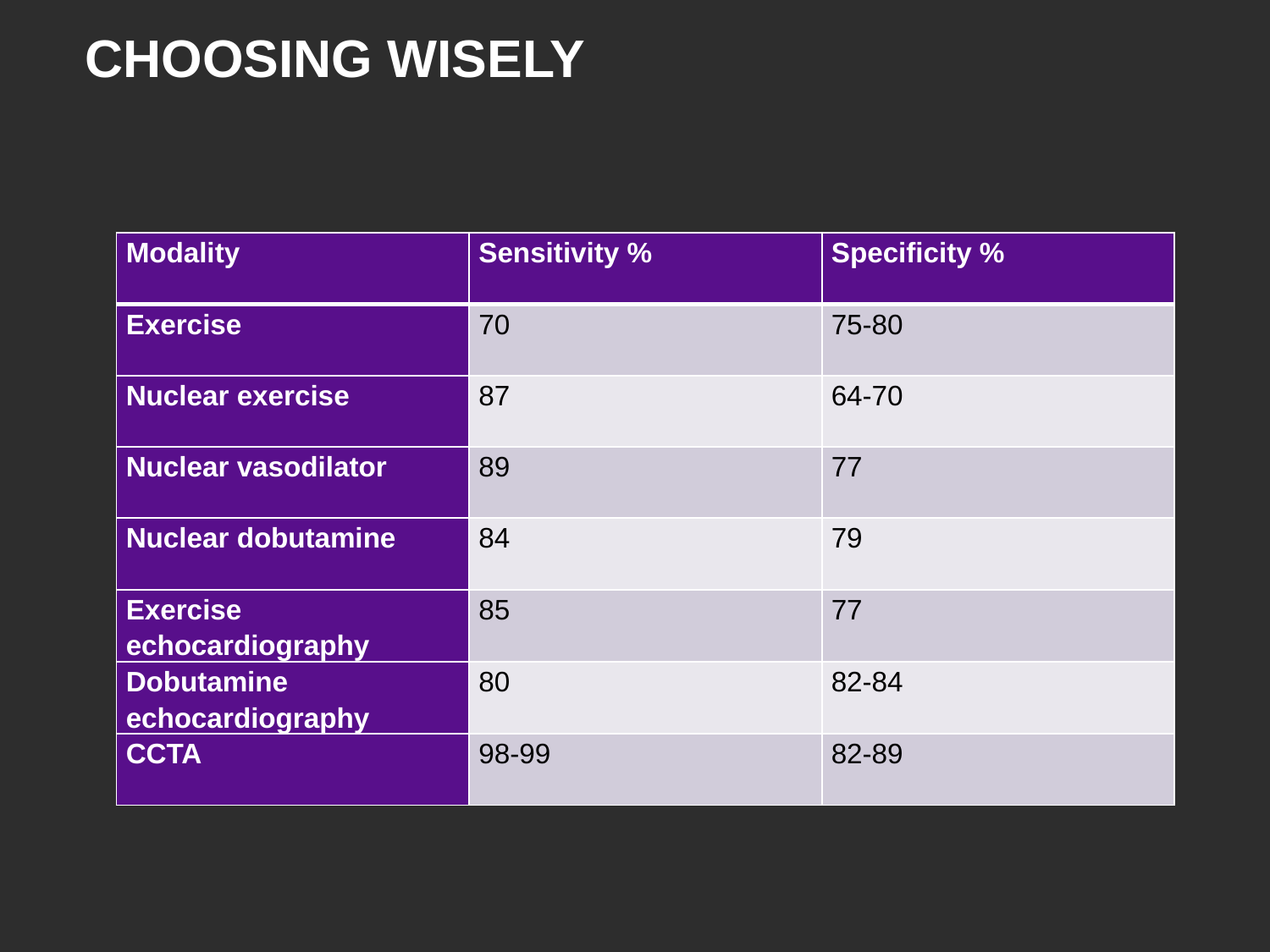

# Choosing wisely
| Modality | Sensitivity % | Specificity % |
| --- | --- | --- |
| Exercise | 70 | 75-80 |
| Nuclear exercise | 87 | 64-70 |
| Nuclear vasodilator | 89 | 77 |
| Nuclear dobutamine | 84 | 79 |
| Exercise echocardiography | 85 | 77 |
| Dobutamine echocardiography | 80 | 82-84 |
| CCTA | 98-99 | 82-89 |

## Slide 20
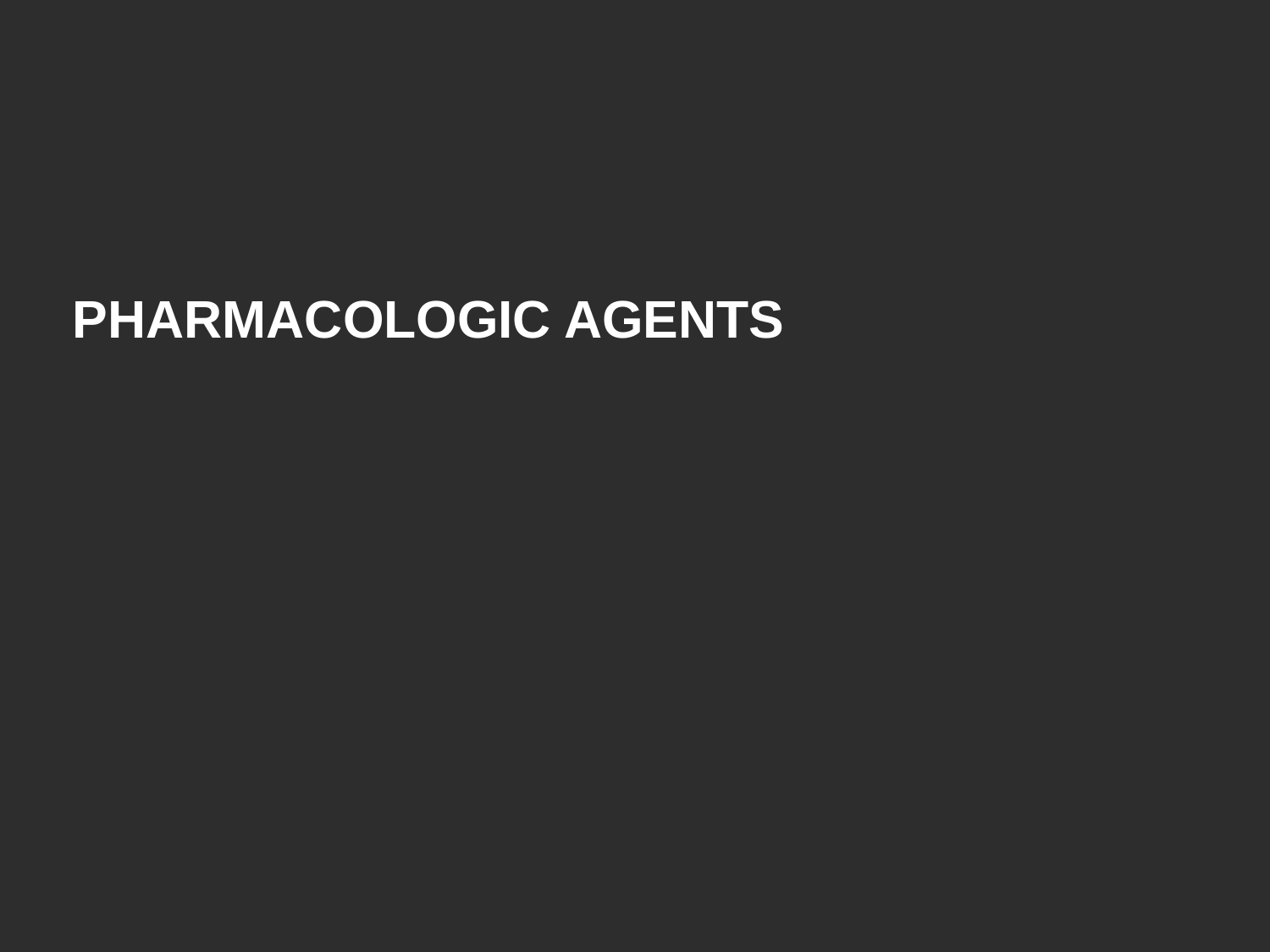

# Pharmacologic agents

## Slide 21
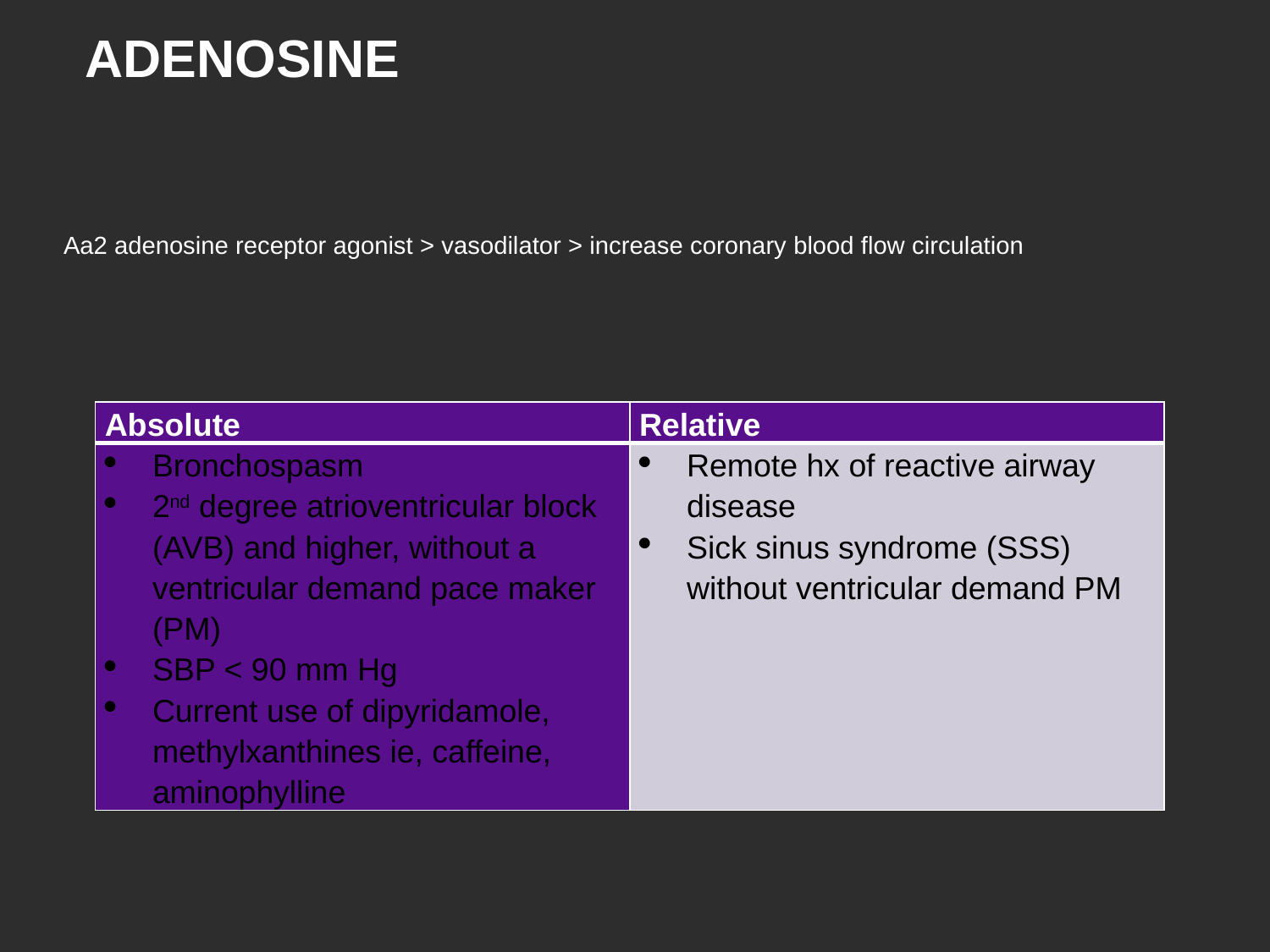

# Adenosine
Aa2 adenosine receptor agonist > vasodilator > increase coronary blood flow circulation
| Absolute | Relative |
| --- | --- |
| Bronchospasm 2nd degree atrioventricular block (AVB) and higher, without a ventricular demand pace maker (PM) SBP < 90 mm Hg Current use of dipyridamole, methylxanthines ie, caffeine, aminophylline | Remote hx of reactive airway disease Sick sinus syndrome (SSS) without ventricular demand PM |

## Slide 22
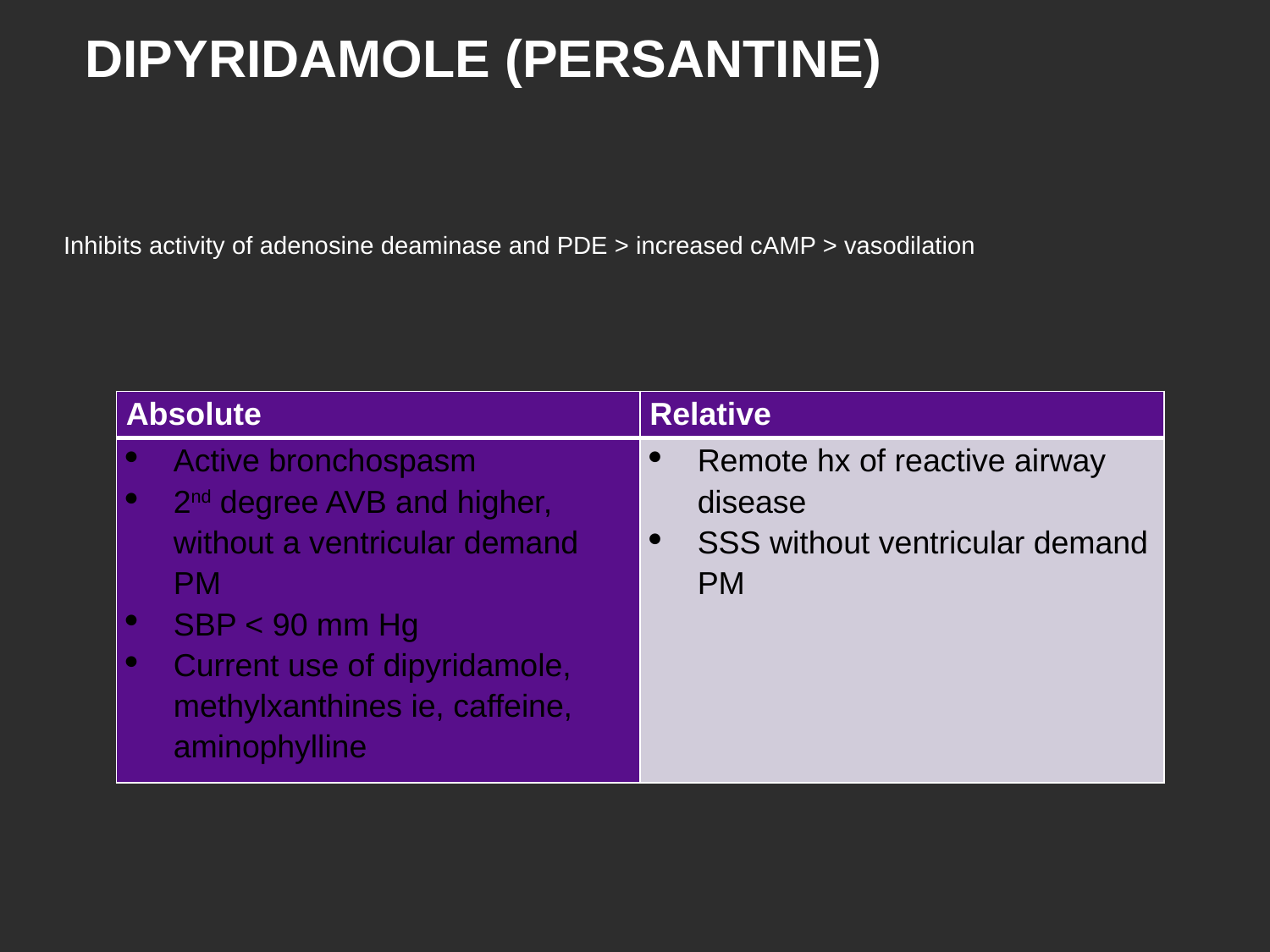

# Dipyridamole (Persantine)
Inhibits activity of adenosine deaminase and PDE > increased cAMP > vasodilation
| Absolute | Relative |
| --- | --- |
| Active bronchospasm 2nd degree AVB and higher, without a ventricular demand PM SBP < 90 mm Hg Current use of dipyridamole, methylxanthines ie, caffeine, aminophylline | Remote hx of reactive airway disease SSS without ventricular demand PM |

## Slide 23
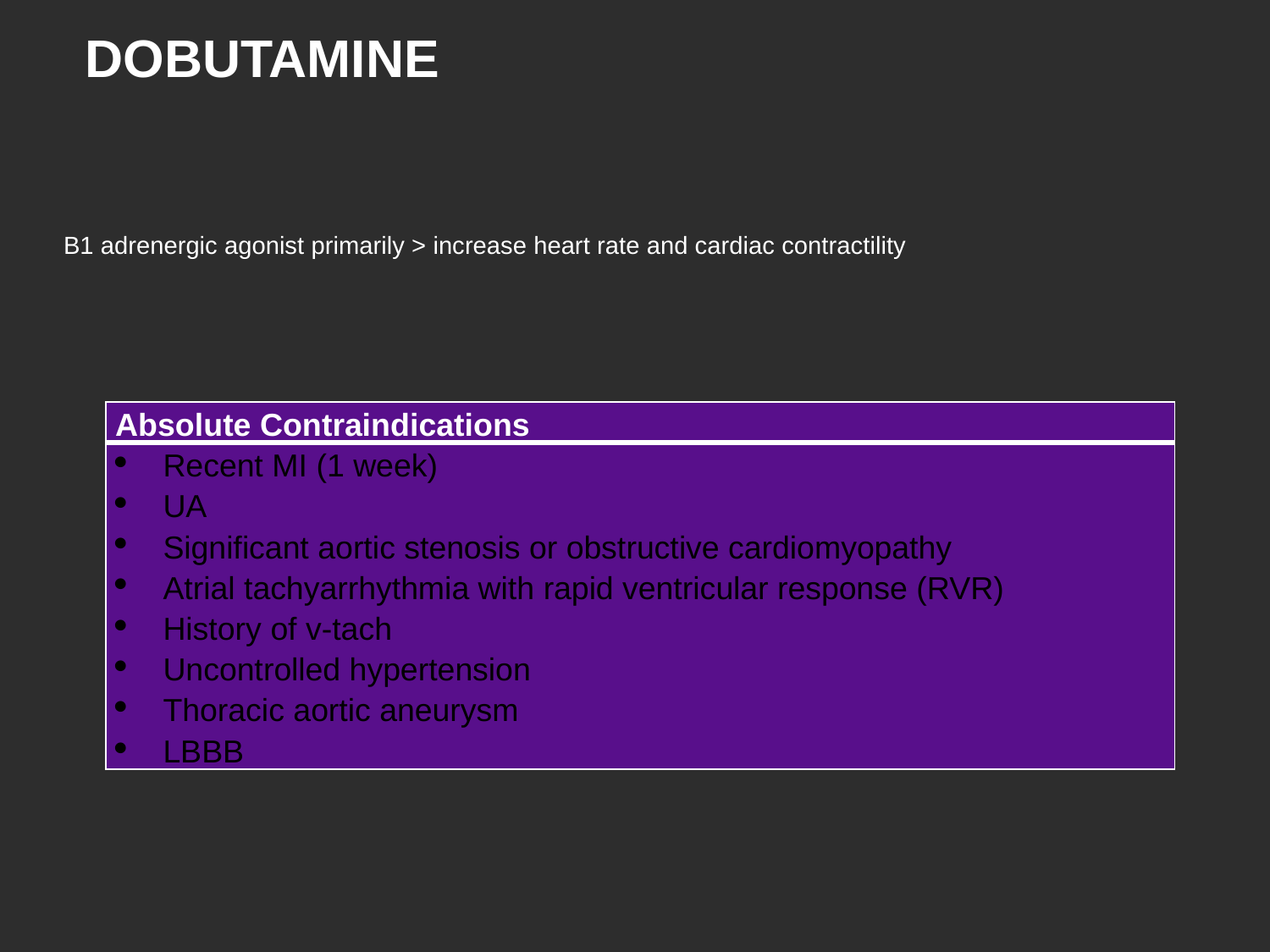

# Dobutamine
B1 adrenergic agonist primarily > increase heart rate and cardiac contractility
| Absolute Contraindications |
| --- |
| Recent MI (1 week) UA Significant aortic stenosis or obstructive cardiomyopathy Atrial tachyarrhythmia with rapid ventricular response (RVR) History of v-tach Uncontrolled hypertension Thoracic aortic aneurysm LBBB |

## Slide 24
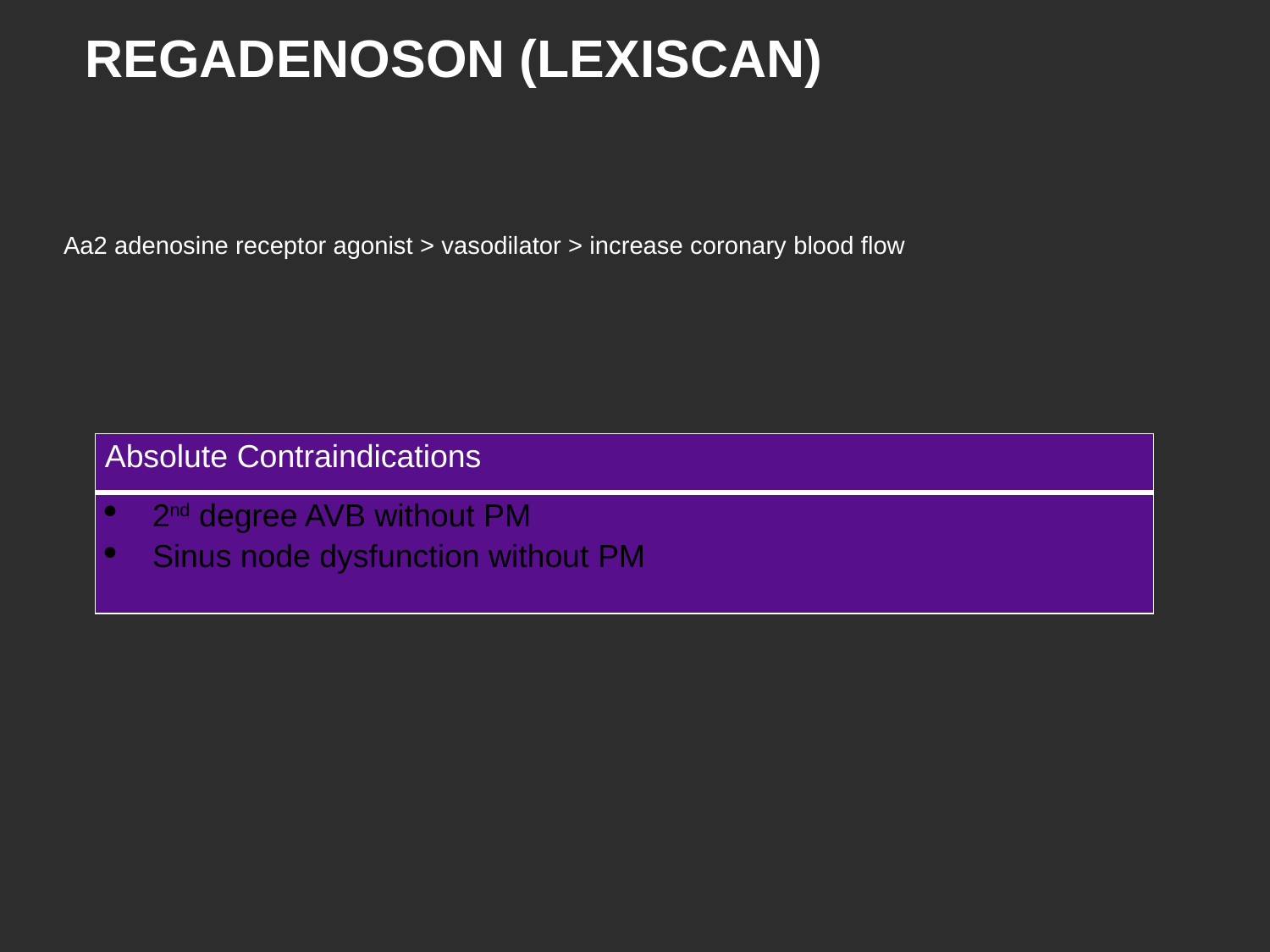

# Regadenoson (Lexiscan)
Aa2 adenosine receptor agonist > vasodilator > increase coronary blood flow
| Absolute Contraindications |
| --- |
| 2nd degree AVB without PM Sinus node dysfunction without PM |

## Slide 25
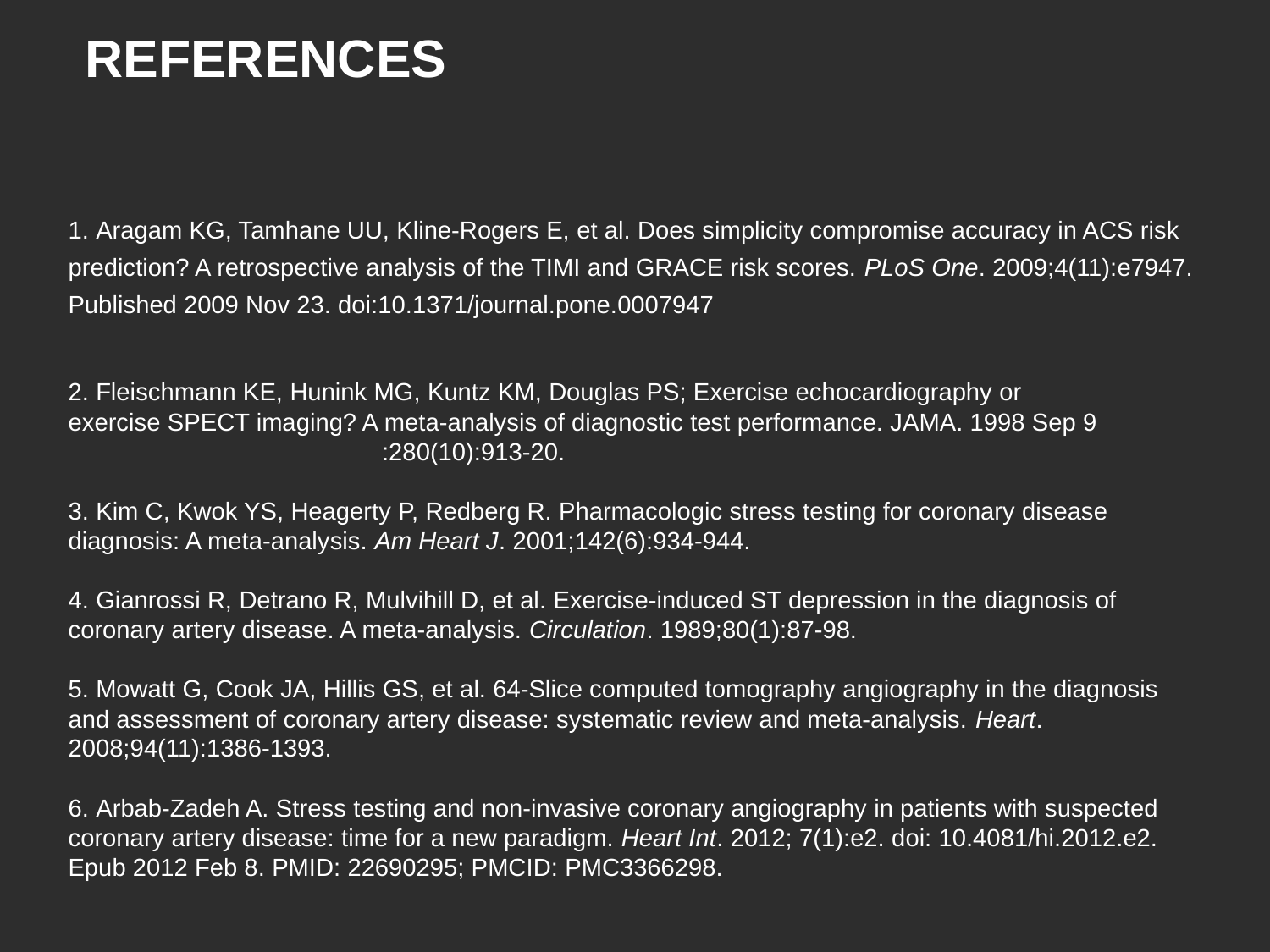

# References
1. Aragam KG, Tamhane UU, Kline-Rogers E, et al. Does simplicity compromise accuracy in ACS risk prediction? A retrospective analysis of the TIMI and GRACE risk scores. PLoS One. 2009;4(11):e7947. Published 2009 Nov 23. doi:10.1371/journal.pone.0007947
2. Fleischmann KE, Hunink MG, Kuntz KM, Douglas PS; Exercise echocardiography or exercise SPECT imaging? A meta-analysis of diagnostic test performance. JAMA. 1998 Sep 9 :280(10):913-20.
3. Kim C, Kwok YS, Heagerty P, Redberg R. Pharmacologic stress testing for coronary disease diagnosis: A meta-analysis. Am Heart J. 2001;142(6):934-944.
4. Gianrossi R, Detrano R, Mulvihill D, et al. Exercise-induced ST depression in the diagnosis of coronary artery disease. A meta-analysis. Circulation. 1989;80(1):87-98.
5. Mowatt G, Cook JA, Hillis GS, et al. 64-Slice computed tomography angiography in the diagnosis and assessment of coronary artery disease: systematic review and meta-analysis. Heart. 2008;94(11):1386-1393.
6. Arbab-Zadeh A. Stress testing and non-invasive coronary angiography in patients with suspected coronary artery disease: time for a new paradigm. Heart Int. 2012; 7(1):e2. doi: 10.4081/hi.2012.e2. Epub 2012 Feb 8. PMID: 22690295; PMCID: PMC3366298.
