# Supplementary material for: An Observation Medicine Curriculum for Emergency Medicine Education
Source: J Educ Teach Emerg Med. 2021 Apr 19;6(2):C1–C72. doi: 10.21980/J87P92 (PMC10332786; doi:10.21980/J87P92)
Supplement: Supplementary file 3 — Please see associated PowerPoint file [file jetem-6-2-c1-supp3.pptx]

## Slide 1
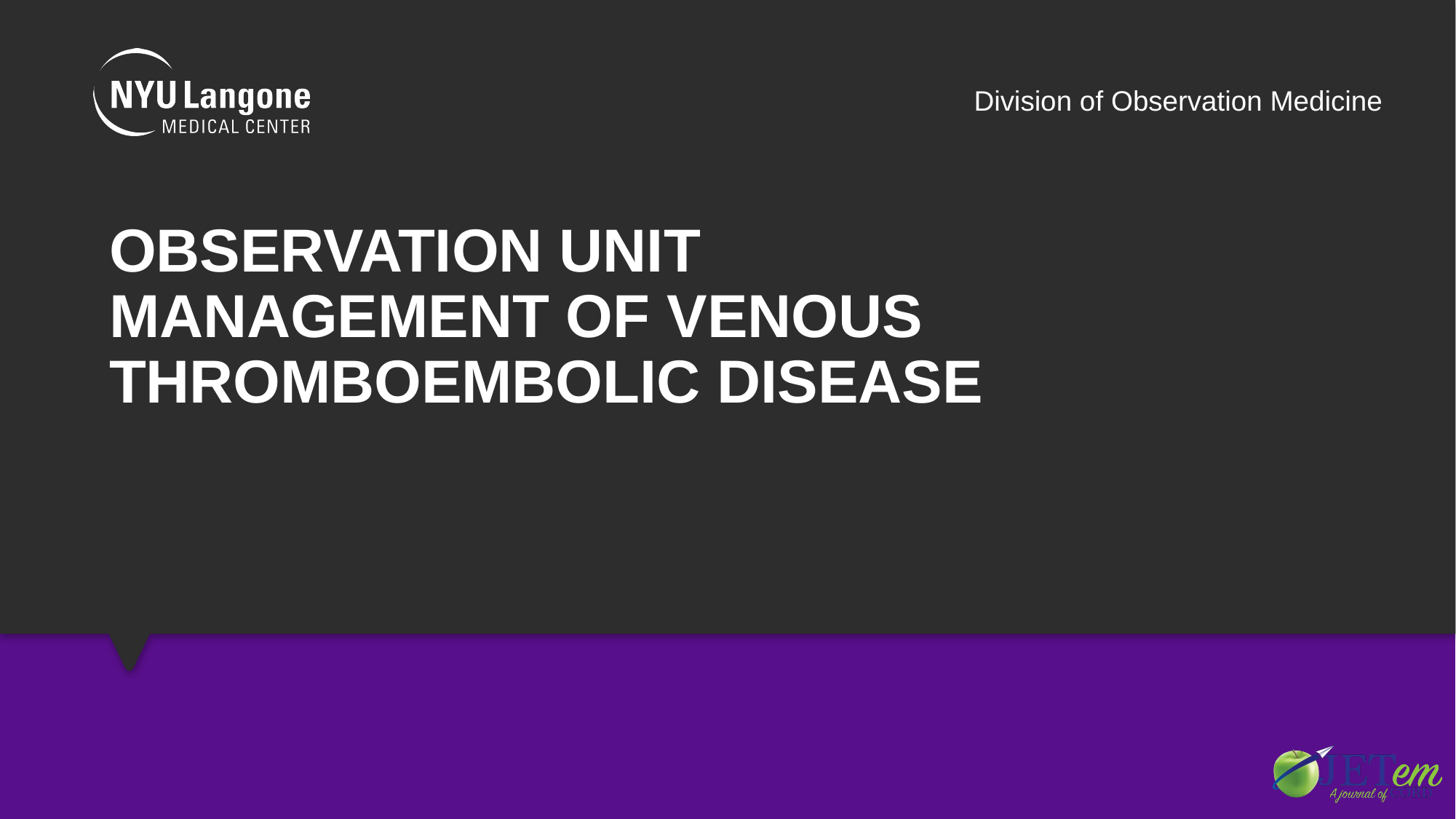

Division of Observation Medicine
# Observation unit Management of Venous Thromboembolic disease

## Slide 2
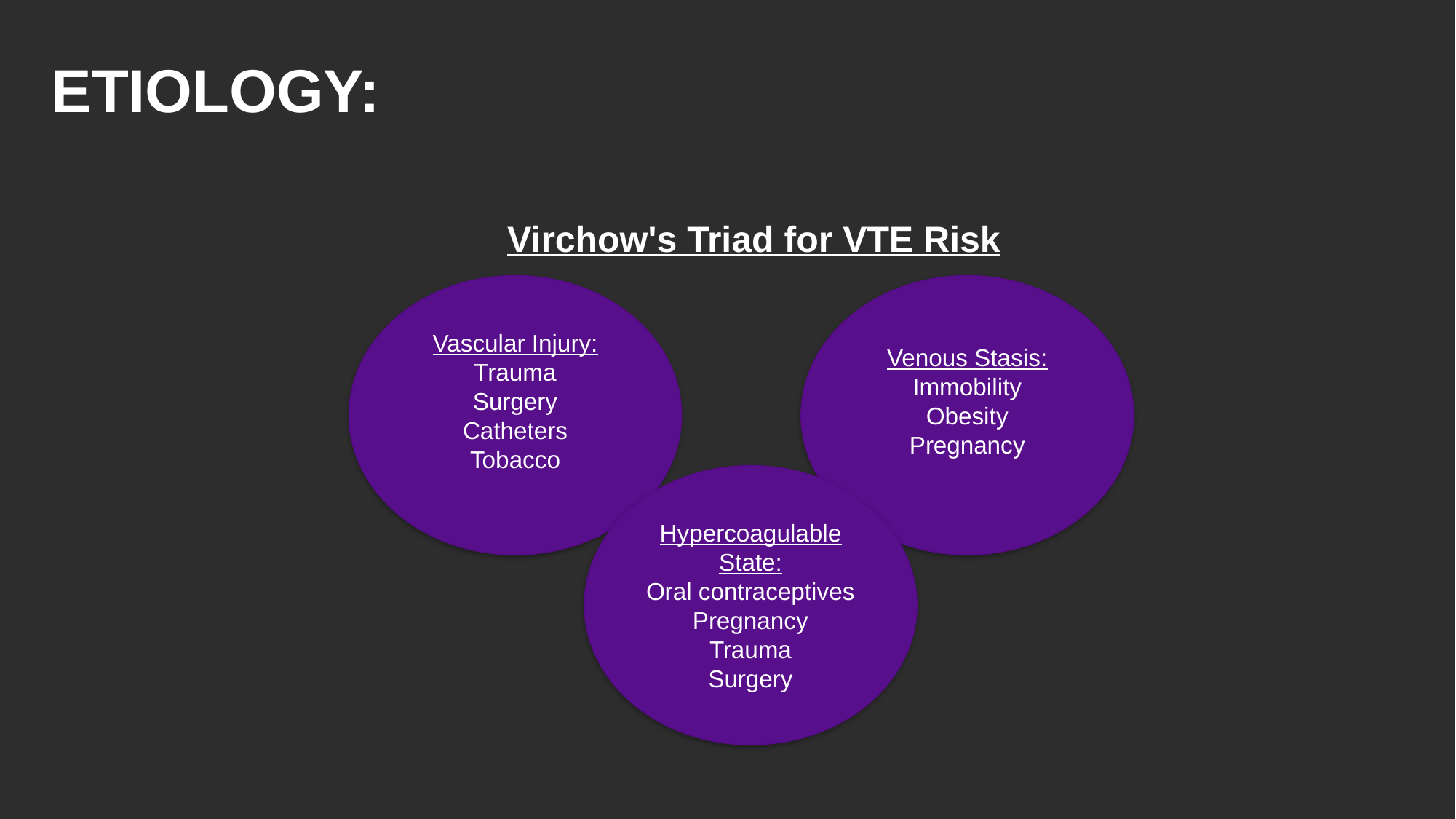

# ETIOLOGY:
Virchow's Triad for VTE Risk
Vascular Injury:
Trauma
Surgery
Catheters
Tobacco
Venous Stasis:
Immobility
Obesity
Pregnancy
Hypercoagulable State:
Oral contraceptives
Pregnancy
Trauma
Surgery

## Slide 3
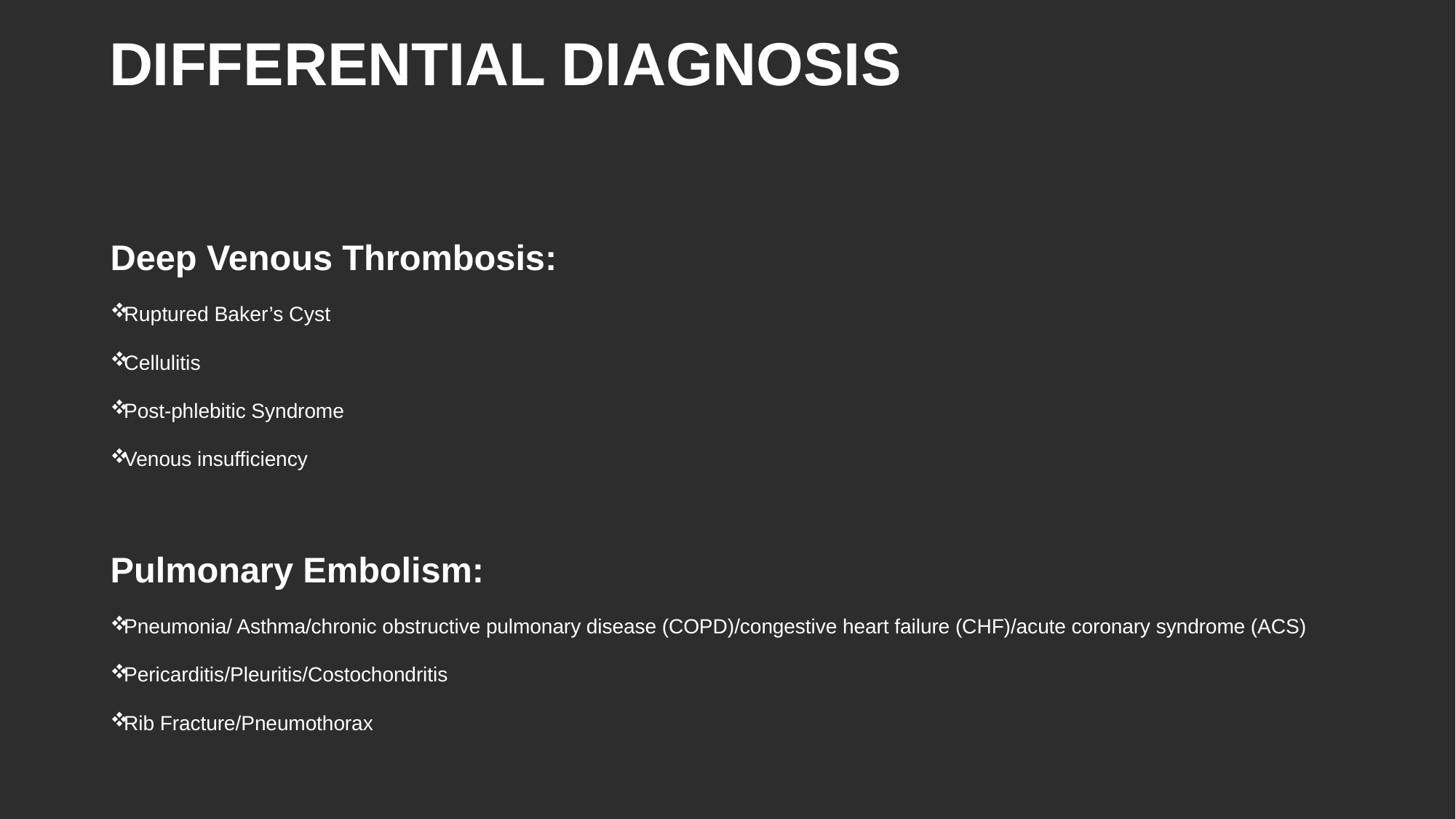

# DIFFERENTIAL DIAGNOSIS
Deep Venous Thrombosis:
Ruptured Baker’s Cyst
Cellulitis
Post-phlebitic Syndrome
Venous insufficiency
Pulmonary Embolism:
Pneumonia/ Asthma/chronic obstructive pulmonary disease (COPD)/congestive heart failure (CHF)/acute coronary syndrome (ACS)
Pericarditis/Pleuritis/Costochondritis
Rib Fracture/Pneumothorax

## Slide 4
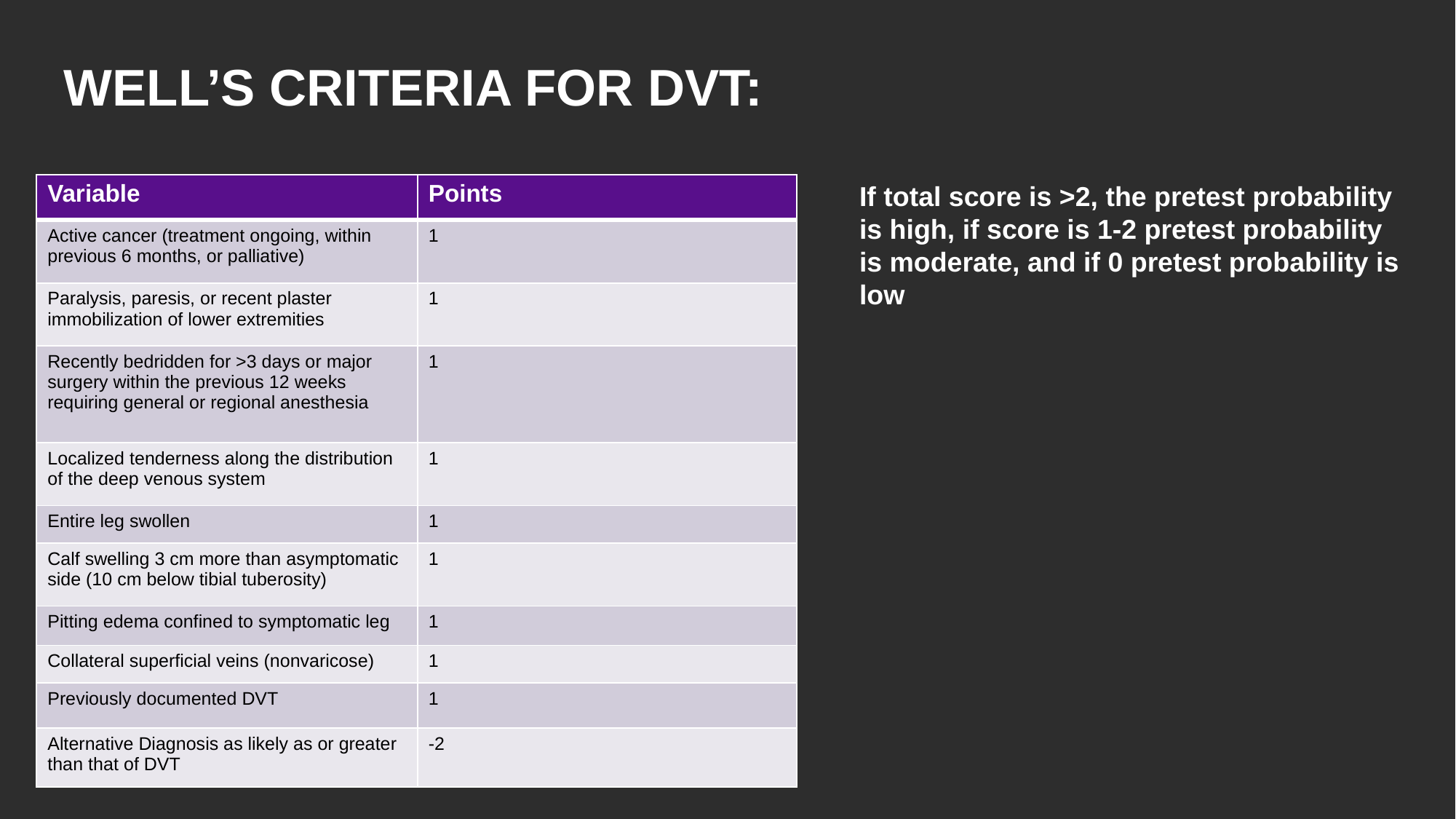

# WELL’S CRITERIA FOR DVT:
If total score is >2, the pretest probability is high, if score is 1-2 pretest probability is moderate, and if 0 pretest probability is low
| Variable | Points |
| --- | --- |
| Active cancer (treatment ongoing, within previous 6 months, or palliative) | 1 |
| Paralysis, paresis, or recent plaster immobilization of lower extremities | 1 |
| Recently bedridden for >3 days or major surgery within the previous 12 weeks requiring general or regional anesthesia | 1 |
| Localized tenderness along the distribution of the deep venous system | 1 |
| Entire leg swollen | 1 |
| Calf swelling 3 cm more than asymptomatic side (10 cm below tibial tuberosity) | 1 |
| Pitting edema confined to symptomatic leg | 1 |
| Collateral superficial veins (nonvaricose) | 1 |
| Previously documented DVT | 1 |
| Alternative Diagnosis as likely as or greater than that of DVT | -2 |

## Slide 5
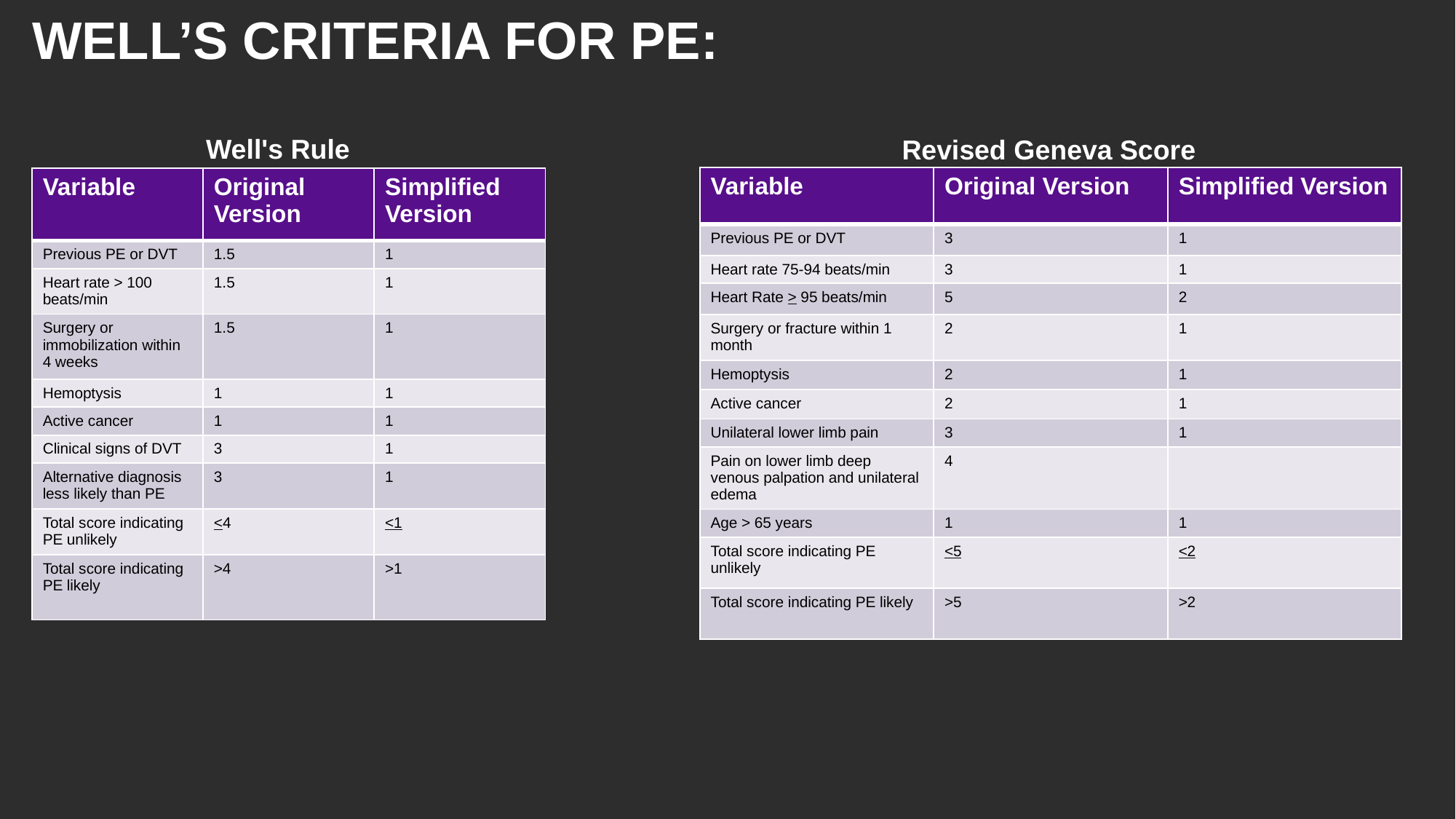

# WELL’S CRITERIA FOR PE:
Well's Rule
Revised Geneva Score
| Variable | Original Version | Simplified Version |
| --- | --- | --- |
| Previous PE or DVT | 3 | 1 |
| Heart rate 75-94 beats/min | 3 | 1 |
| Heart Rate > 95 beats/min | 5 | 2 |
| Surgery or fracture within 1 month | 2 | 1 |
| Hemoptysis | 2 | 1 |
| Active cancer | 2 | 1 |
| Unilateral lower limb pain | 3 | 1 |
| Pain on lower limb deep venous palpation and unilateral edema | 4 | |
| Age > 65 years | 1 | 1 |
| Total score indicating PE unlikely | <5 | <2 |
| Total score indicating PE likely | >5 | >2 |
| Variable | Original Version | Simplified Version |
| --- | --- | --- |
| Previous PE or DVT | 1.5 | 1 |
| Heart rate > 100 beats/min | 1.5 | 1 |
| Surgery or immobilization within 4 weeks | 1.5 | 1 |
| Hemoptysis | 1 | 1 |
| Active cancer | 1 | 1 |
| Clinical signs of DVT | 3 | 1 |
| Alternative diagnosis less likely than PE | 3 | 1 |
| Total score indicating PE unlikely | <4 | <1 |
| Total score indicating PE likely | >4 | >1 |

## Slide 6
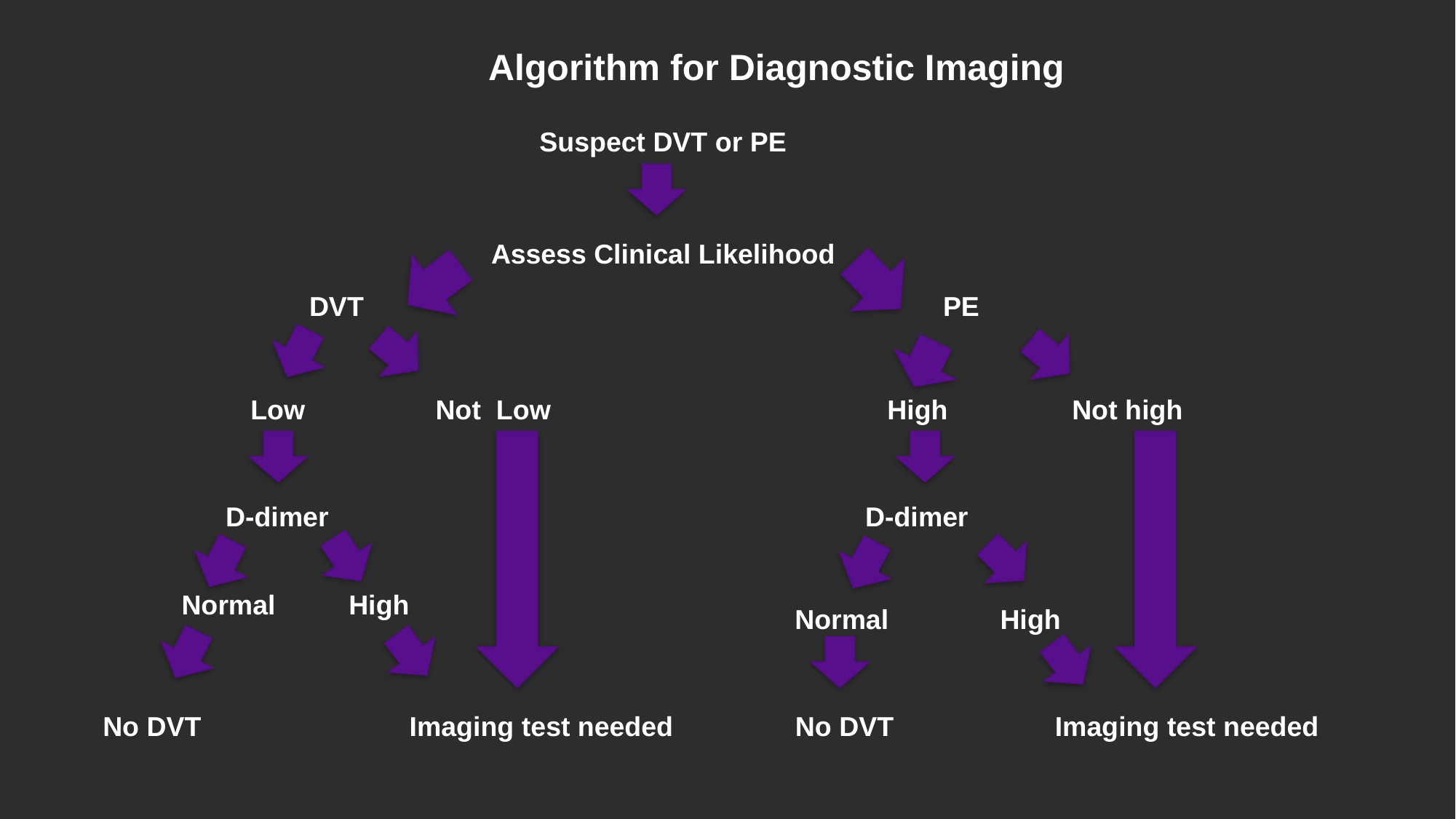

Algorithm for Diagnostic Imaging
Suspect DVT or PE
Assess Clinical Likelihood
DVT
PE
Low
Not  Low
High
Not high
D-dimer
D-dimer
High
Normal
Normal
High
No DVT
Imaging test needed
Imaging test needed
No DVT

## Slide 7
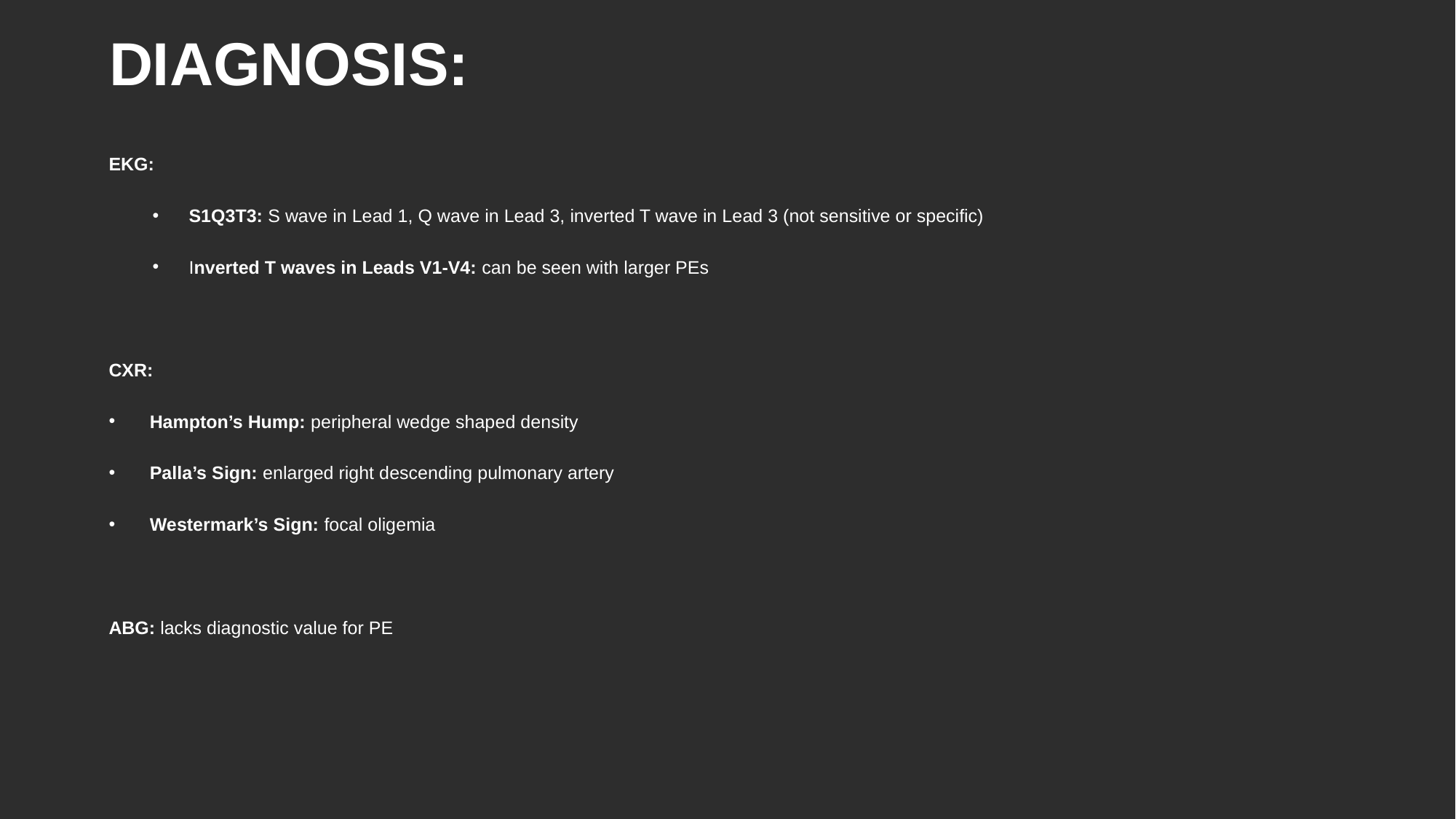

# DIAGNOSIS:
EKG:
S1Q3T3: S wave in Lead 1, Q wave in Lead 3, inverted T wave in Lead 3 (not sensitive or specific)
Inverted T waves in Leads V1-V4: can be seen with larger PEs
CXR:
Hampton’s Hump: peripheral wedge shaped density
Palla’s Sign: enlarged right descending pulmonary artery
Westermark’s Sign: focal oligemia
ABG: lacks diagnostic value for PE

## Slide 8
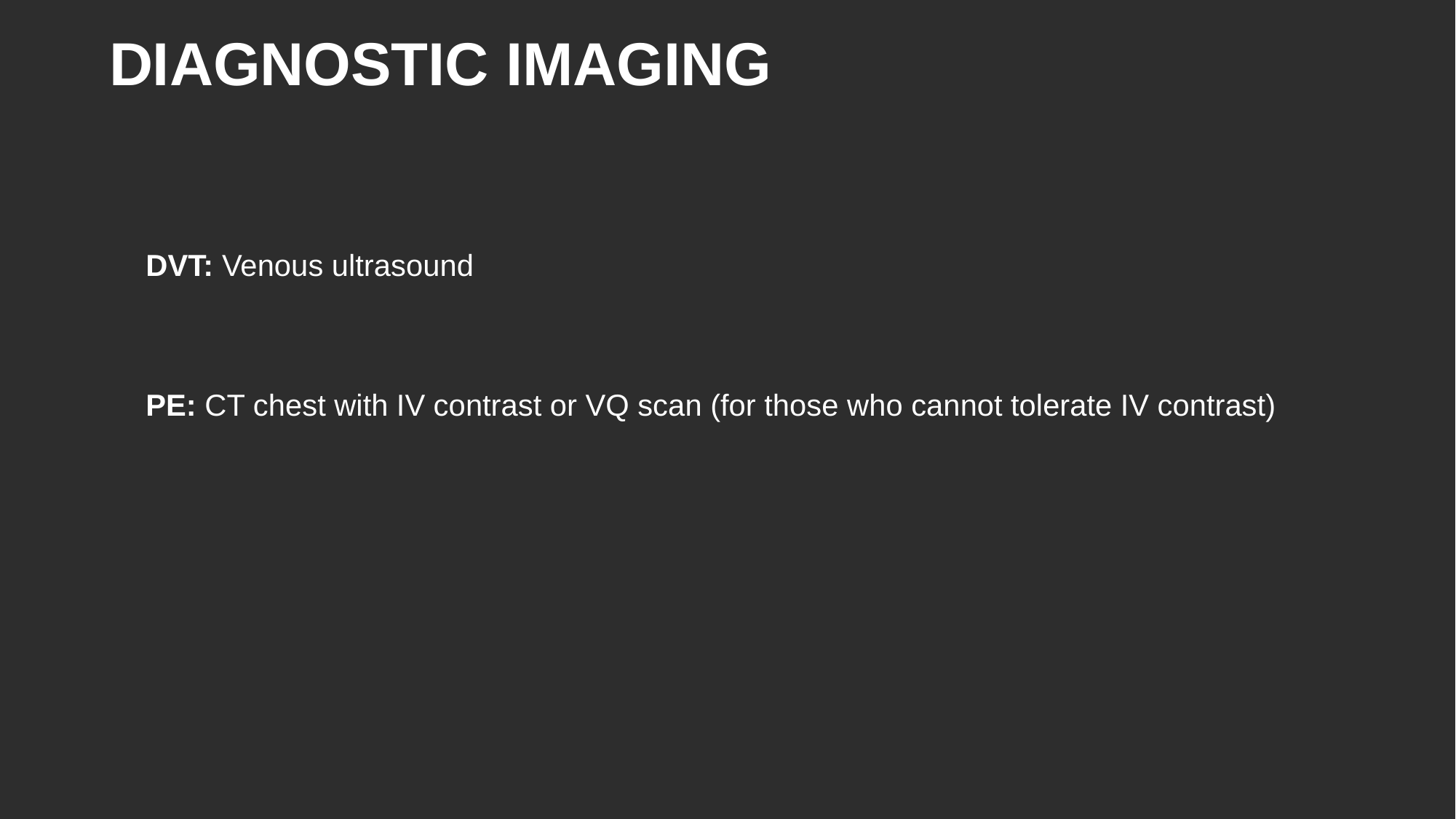

# DIAGNOStic IMAGING
DVT: Venous ultrasound
PE: CT chest with IV contrast or VQ scan (for those who cannot tolerate IV contrast)

## Slide 9
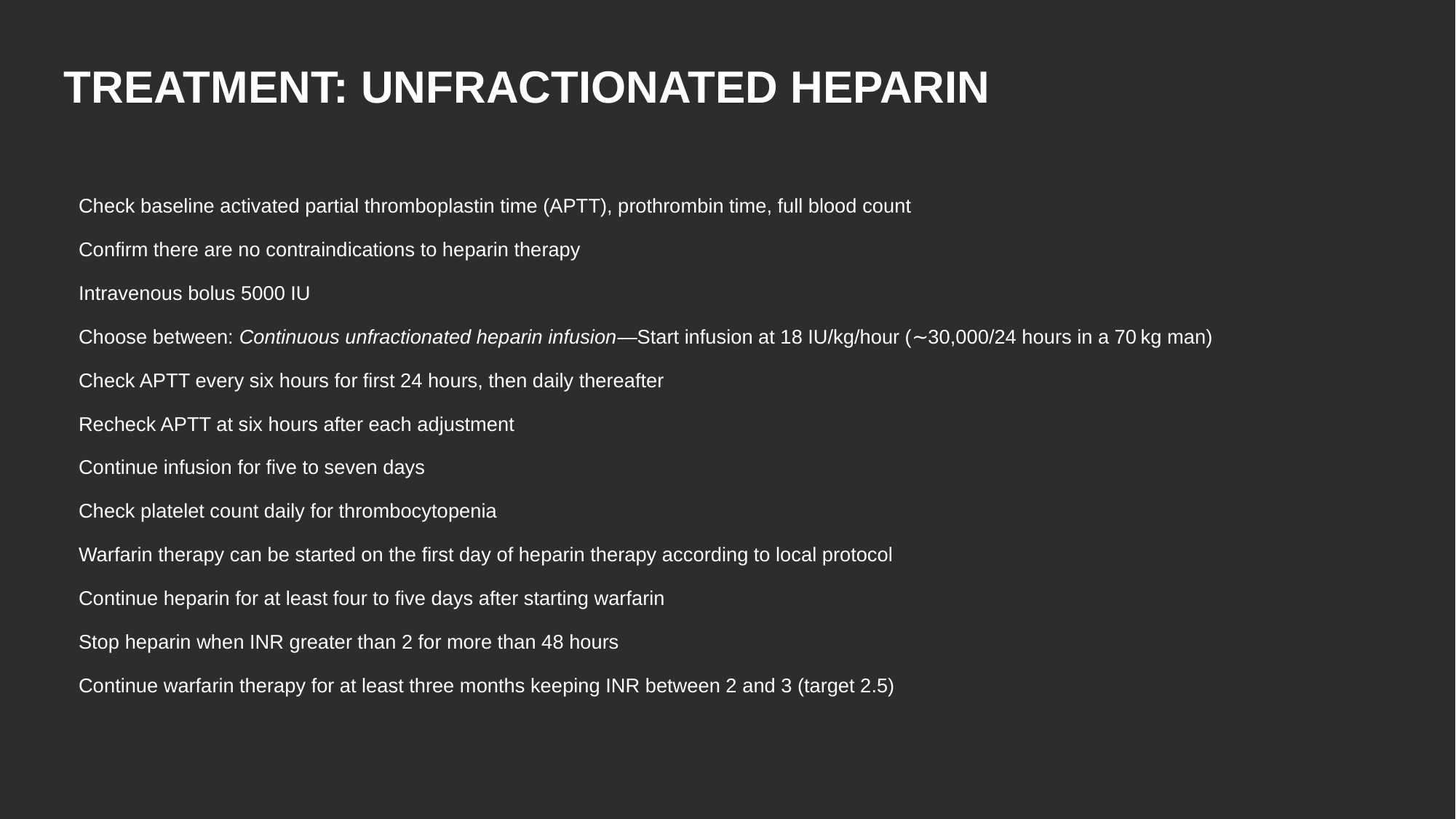

# TREATMENT: UNFRACTIONATED HEPARIN
Check baseline activated partial thromboplastin time (APTT), prothrombin time, full blood count
Confirm there are no contraindications to heparin therapy
Intravenous bolus 5000 IU
Choose between: Continuous unfractionated heparin infusion—Start infusion at 18 IU/kg/hour (∼30,000/24 hours in a 70 kg man)
Check APTT every six hours for first 24 hours, then daily thereafter
Recheck APTT at six hours after each adjustment
Continue infusion for five to seven days
Check platelet count daily for thrombocytopenia
Warfarin therapy can be started on the first day of heparin therapy according to local protocol
Continue heparin for at least four to five days after starting warfarin
Stop heparin when INR greater than 2 for more than 48 hours
Continue warfarin therapy for at least three months keeping INR between 2 and 3 (target 2.5)

## Slide 10
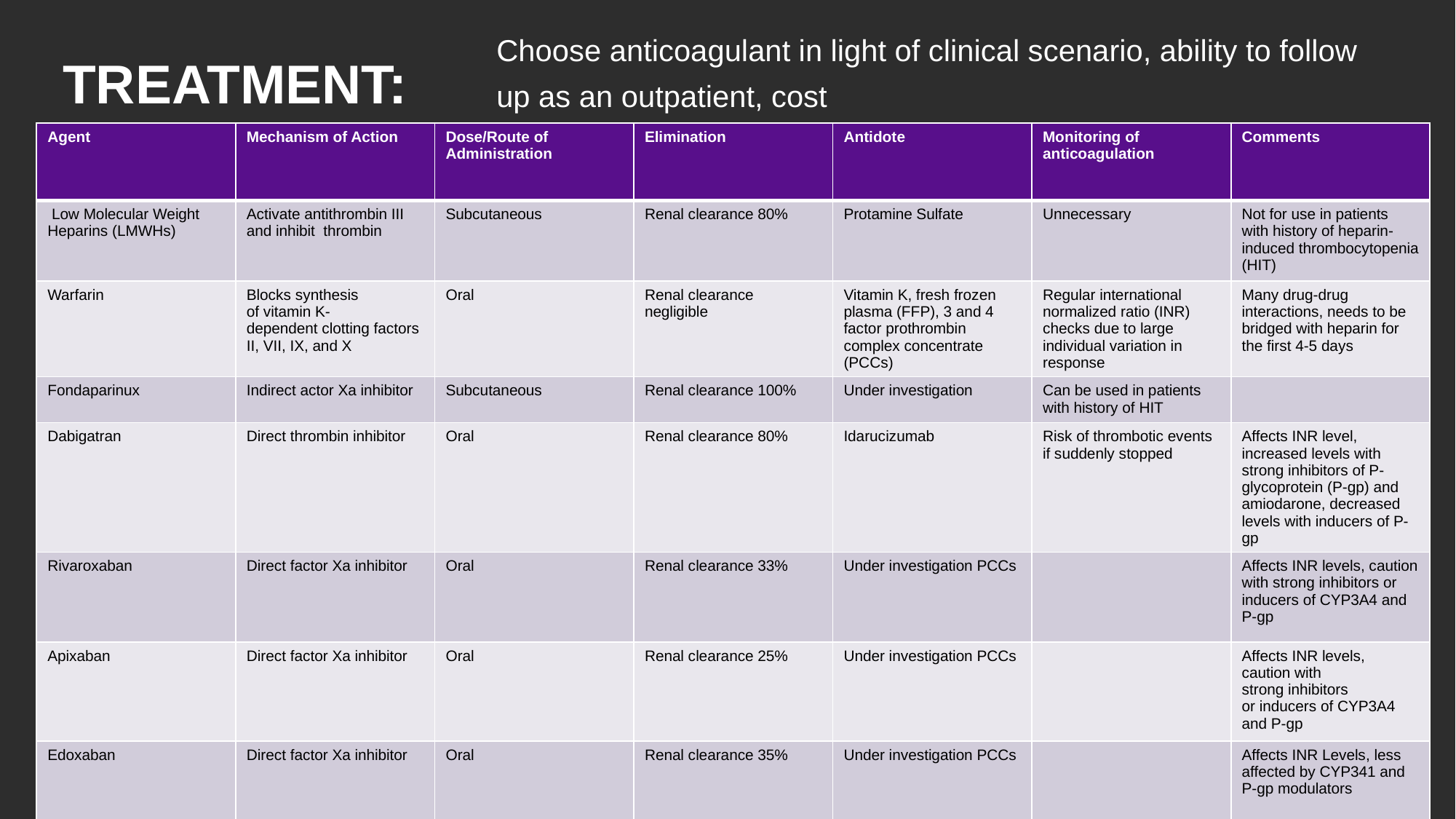

Choose anticoagulant in light of clinical scenario, ability to follow up as an outpatient, cost
# TREATMENT:
| Agent | Mechanism of Action | Dose/Route of Administration | Elimination | Antidote | Monitoring of anticoagulation | Comments |
| --- | --- | --- | --- | --- | --- | --- |
| Low Molecular Weight Heparins (LMWHs) | Activate antithrombin III and inhibit  thrombin | Subcutaneous | Renal clearance 80% | Protamine Sulfate | Unnecessary | Not for use in patients with history of heparin-induced thrombocytopenia (HIT) |
| Warfarin | Blocks synthesis of vitamin K-dependent clotting factors II, VII, IX, and X | Oral | Renal clearance negligible | Vitamin K, fresh frozen plasma (FFP), 3 and 4 factor prothrombin complex concentrate (PCCs) | Regular international normalized ratio (INR) checks due to large individual variation in response | Many drug-drug interactions, needs to be bridged with heparin for the first 4-5 days |
| Fondaparinux | Indirect actor Xa inhibitor | Subcutaneous | Renal clearance 100% | Under investigation | Can be used in patients with history of HIT | |
| Dabigatran | Direct thrombin inhibitor | Oral | Renal clearance 80% | Idarucizumab | Risk of thrombotic events if suddenly stopped | Affects INR level, increased levels with strong inhibitors of P-glycoprotein (P-gp) and amiodarone, decreased levels with inducers of P-gp |
| Rivaroxaban | Direct factor Xa inhibitor | Oral | Renal clearance 33% | Under investigation PCCs | | Affects INR levels, caution with strong inhibitors or inducers of CYP3A4 and P-gp |
| Apixaban | Direct factor Xa inhibitor | Oral | Renal clearance 25% | Under investigation PCCs | | Affects INR levels, caution with strong inhibitors or inducers of CYP3A4 and P-gp |
| Edoxaban | Direct factor Xa inhibitor | Oral | Renal clearance 35% | Under investigation PCCs | | Affects INR Levels, less affected by CYP341 and P-gp modulators |

## Slide 11
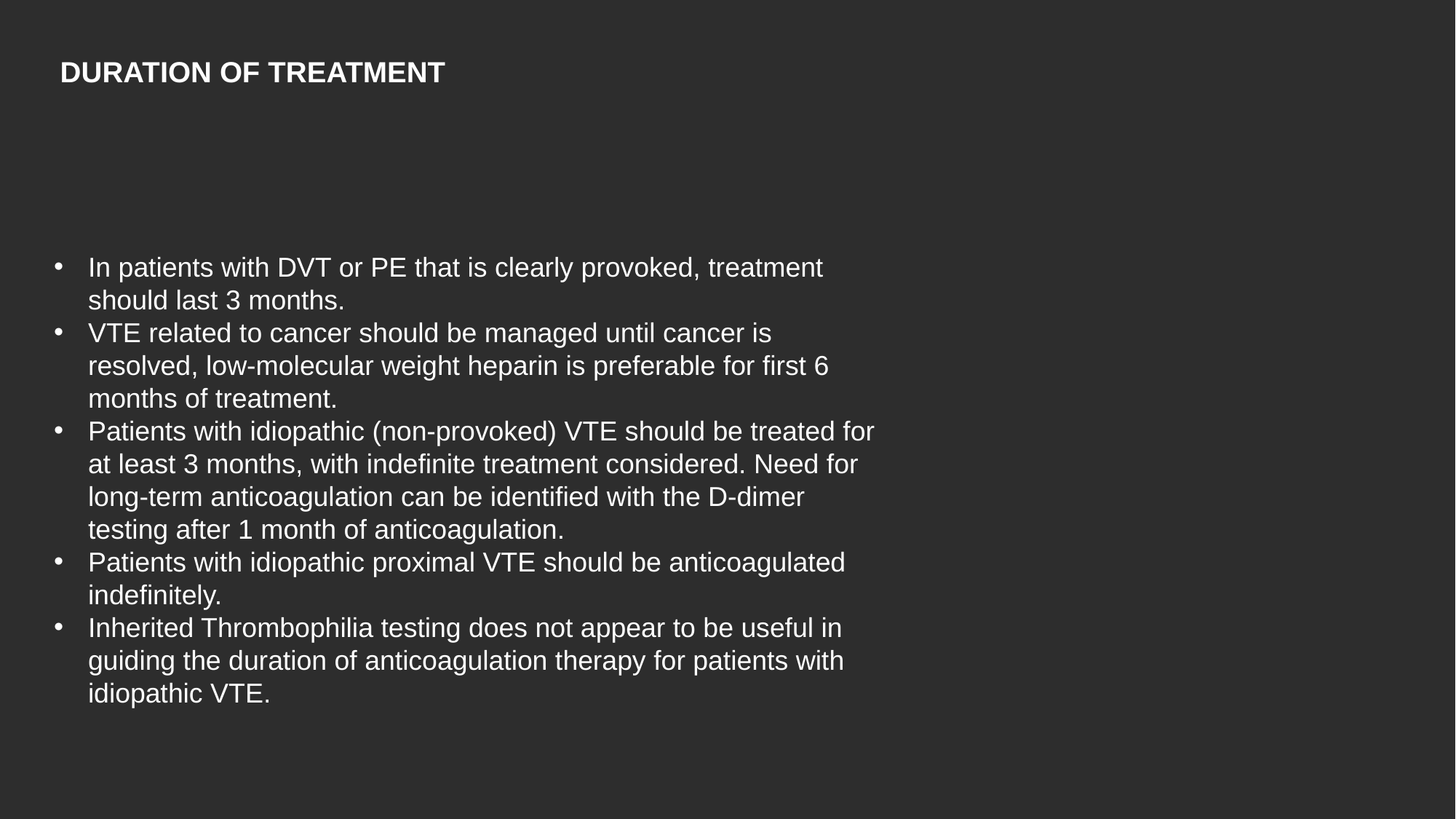

# DURATION OF TREATMENT
In patients with DVT or PE that is clearly provoked, treatment should last 3 months.
VTE related to cancer should be managed until cancer is resolved, low-molecular weight heparin is preferable for first 6 months of treatment.
Patients with idiopathic (non-provoked) VTE should be treated for at least 3 months, with indefinite treatment considered. Need for long-term anticoagulation can be identified with the D-dimer testing after 1 month of anticoagulation.
Patients with idiopathic proximal VTE should be anticoagulated indefinitely.
Inherited Thrombophilia testing does not appear to be useful in guiding the duration of anticoagulation therapy for patients with idiopathic VTE.

## Slide 12
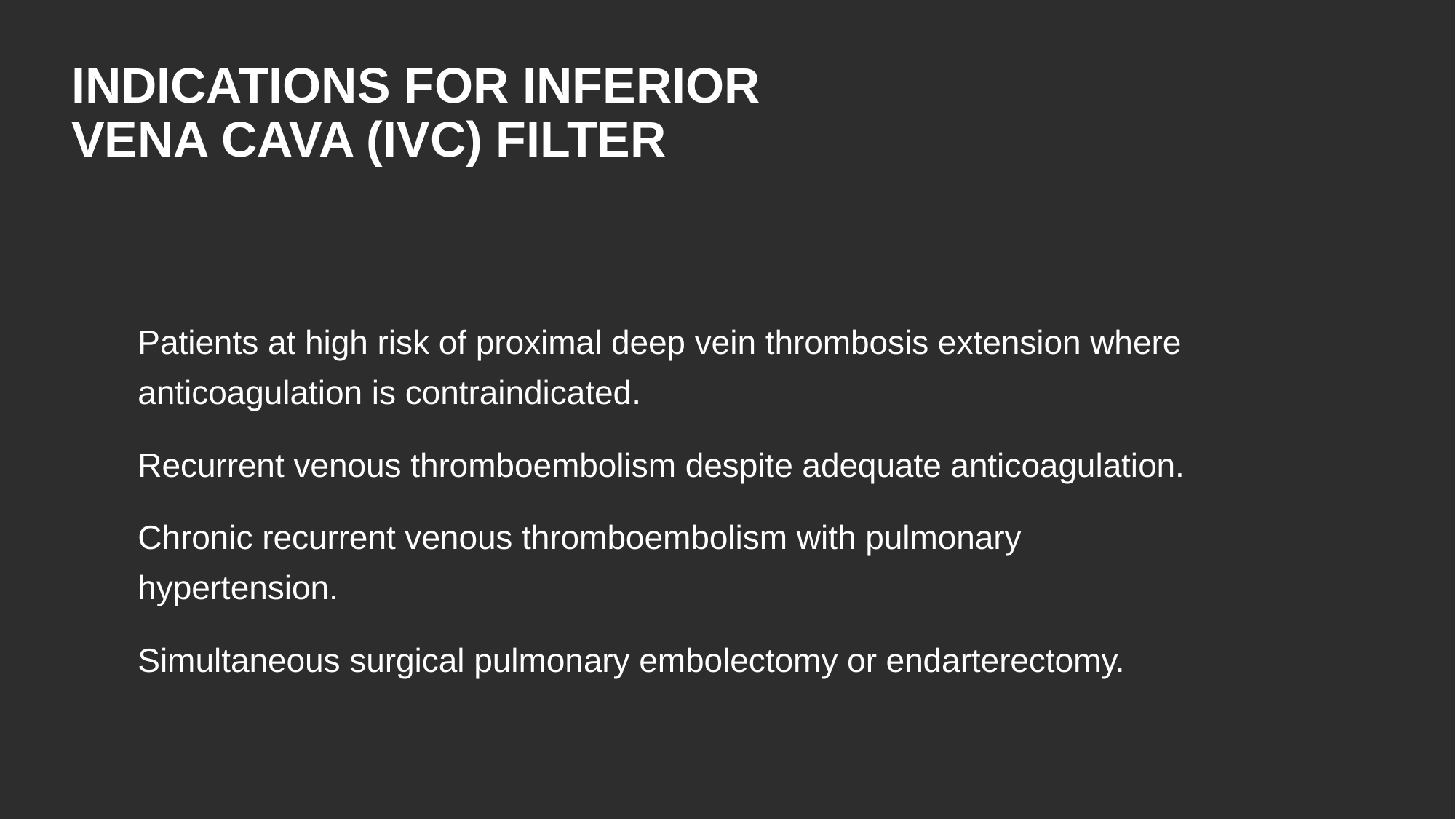

# INDICATIONS FOR Inferior vena cava (IVC) FILTER
Patients at high risk of proximal deep vein thrombosis extension where anticoagulation is contraindicated.
Recurrent venous thromboembolism despite adequate anticoagulation.
Chronic recurrent venous thromboembolism with pulmonary hypertension.
Simultaneous surgical pulmonary embolectomy or endarterectomy.

## Slide 13
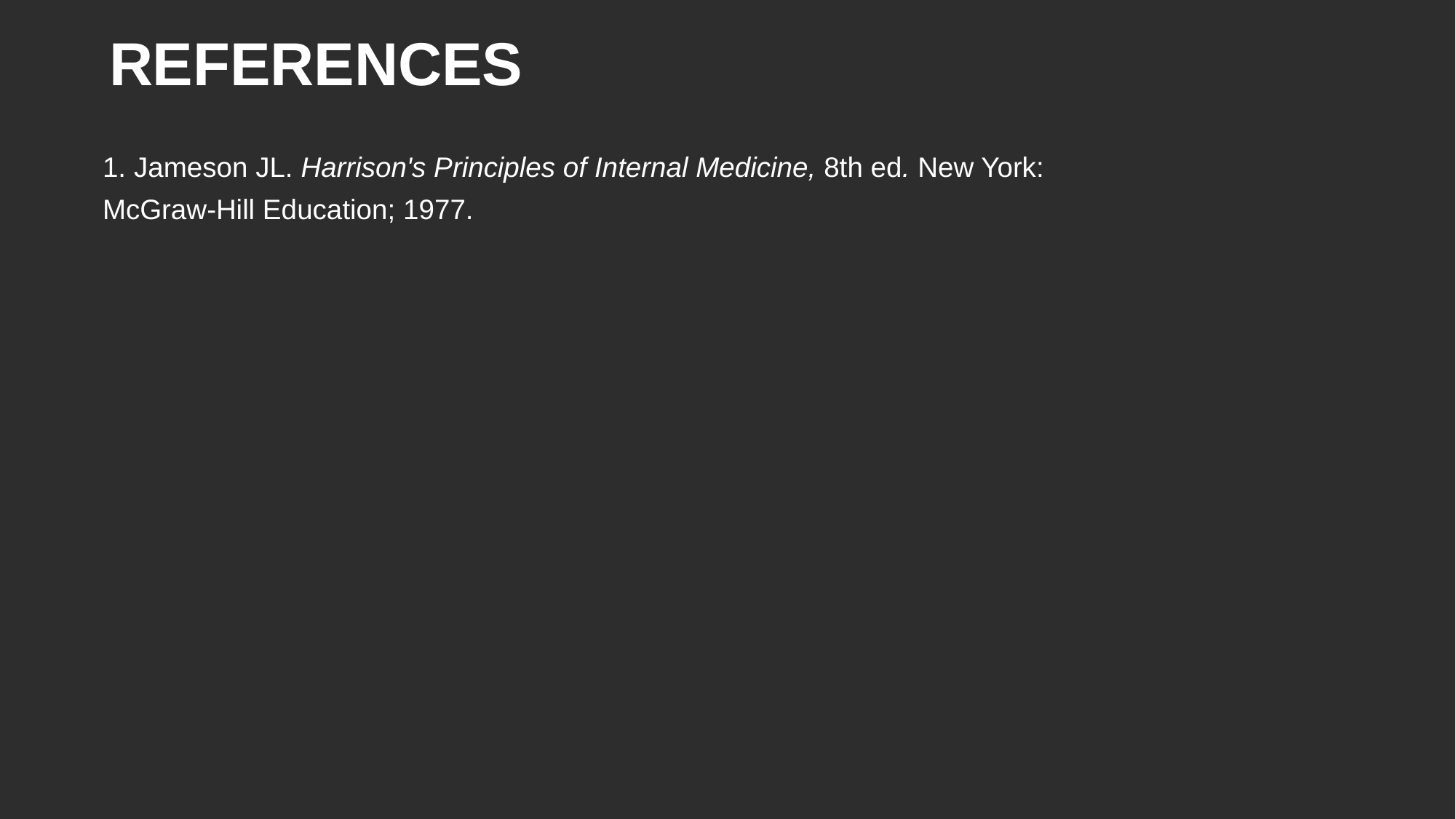

# References
1. Jameson JL. Harrison's Principles of Internal Medicine, 8th ed. New York: McGraw-Hill Education; 1977.
