# Supplementary material for: An Observation Medicine Curriculum for Emergency Medicine Education
Source: J Educ Teach Emerg Med. 2021 Apr 19;6(2):C1–C72. doi: 10.21980/J87P92 (PMC10332786; doi:10.21980/J87P92)
Supplement: Supplementary file 4 — Please see associated PowerPoint file [file jetem-6-2-c1-supp4.pptx]

## Slide 1
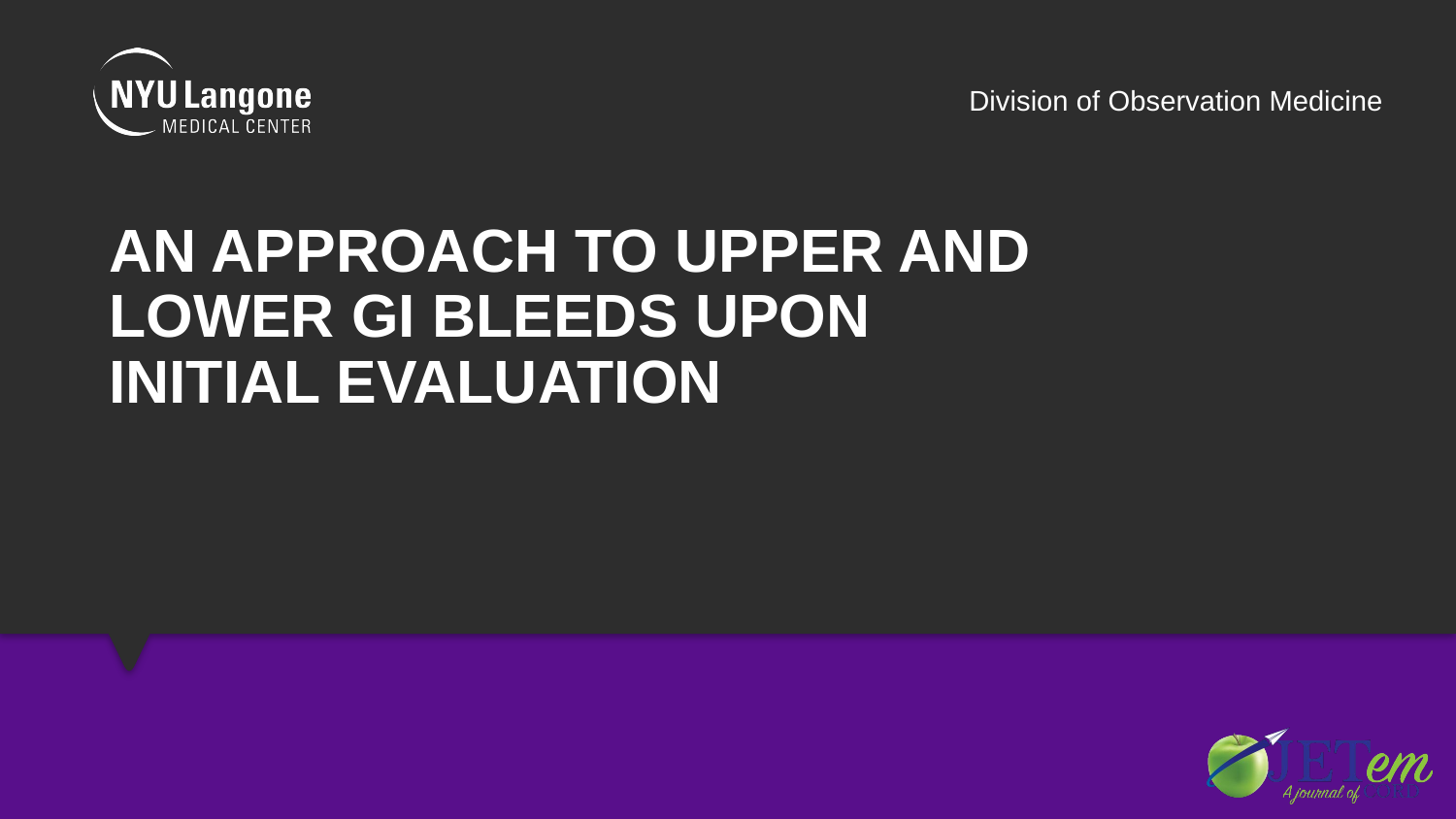

Division of Observation Medicine
# AN APPROACH TO UPPER AND LOWER GI BLEEDS UPON INITIAL EVALUATION

## Slide 2
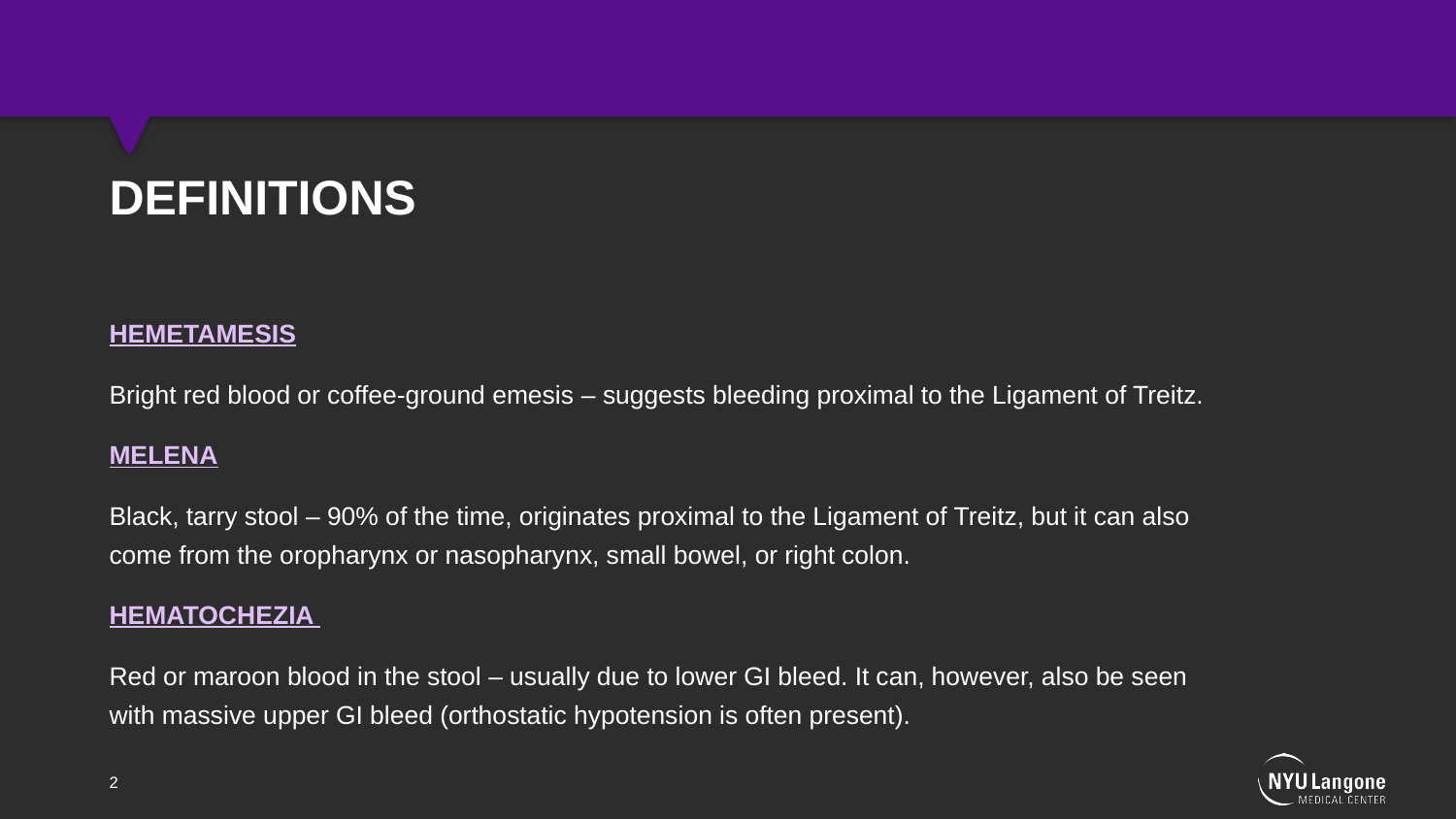

# DEFINITIONS
HEMETAMESIS
Bright red blood or coffee-ground emesis – suggests bleeding proximal to the Ligament of Treitz.
MELENA
Black, tarry stool – 90% of the time, originates proximal to the Ligament of Treitz, but it can also come from the oropharynx or nasopharynx, small bowel, or right colon.
HEMATOCHEZIA
Red or maroon blood in the stool – usually due to lower GI bleed. It can, however, also be seen with massive upper GI bleed (orthostatic hypotension is often present).
2

## Slide 3
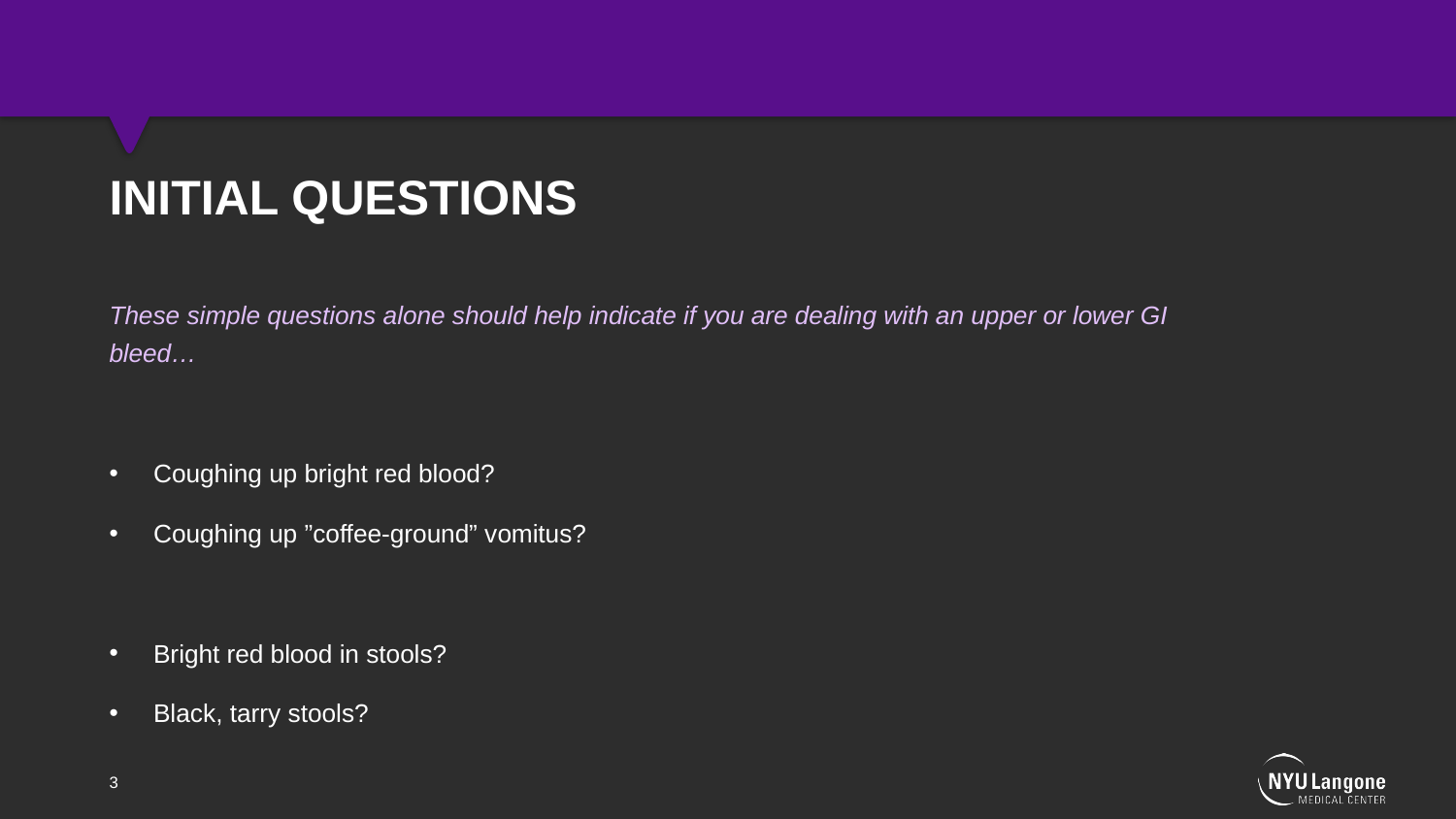

# INITIAL QUESTIONS
These simple questions alone should help indicate if you are dealing with an upper or lower GI bleed…
Coughing up bright red blood?
Coughing up ”coffee-ground” vomitus?
Bright red blood in stools?
Black, tarry stools?
3

## Slide 4
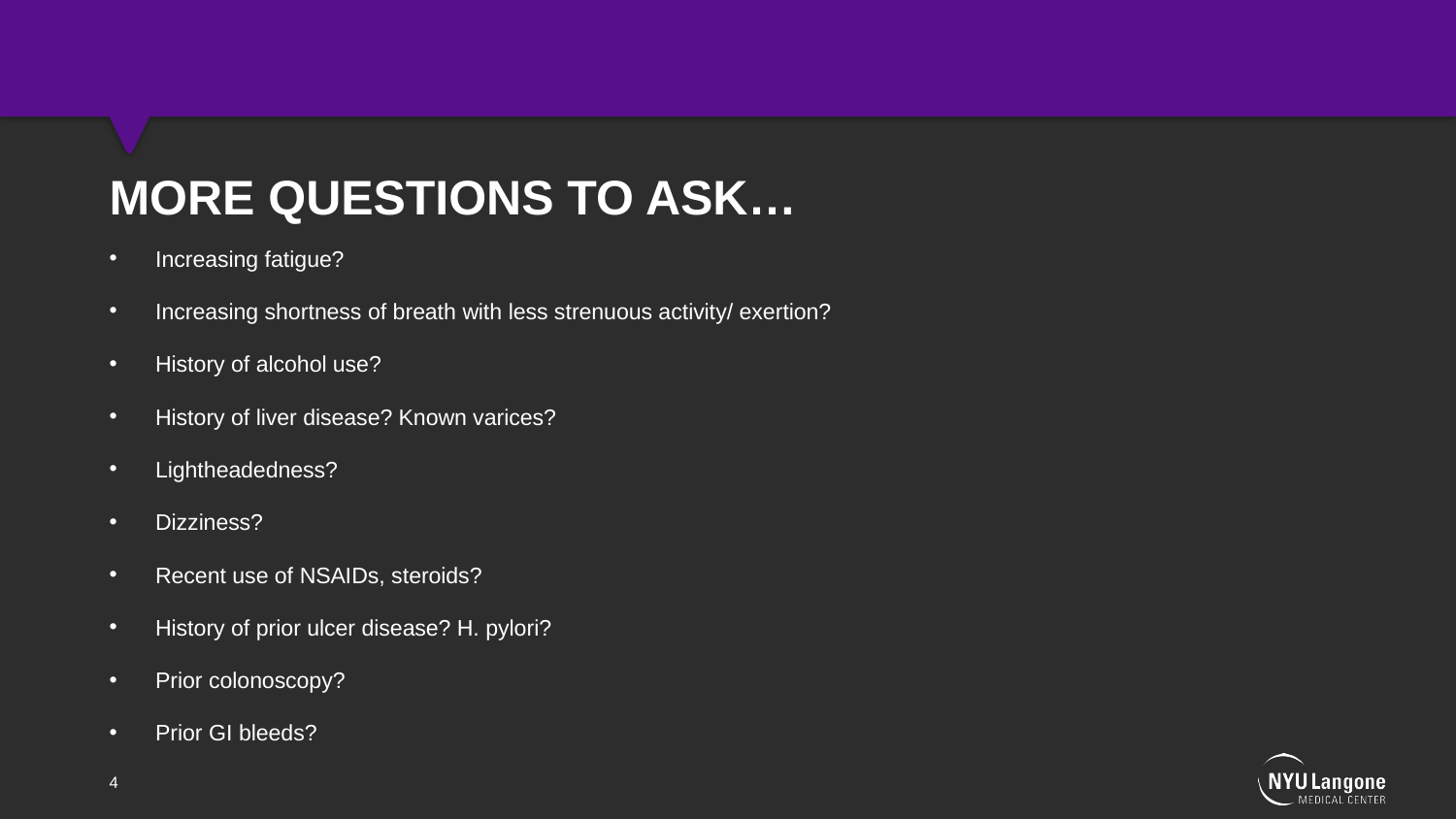

# MORE QUESTIONS TO ASK…
Increasing fatigue?
Increasing shortness of breath with less strenuous activity/ exertion?
History of alcohol use?
History of liver disease? Known varices?
Lightheadedness?
Dizziness?
Recent use of NSAIDs, steroids?
History of prior ulcer disease? H. pylori?
Prior colonoscopy?
Prior GI bleeds?
4

## Slide 5
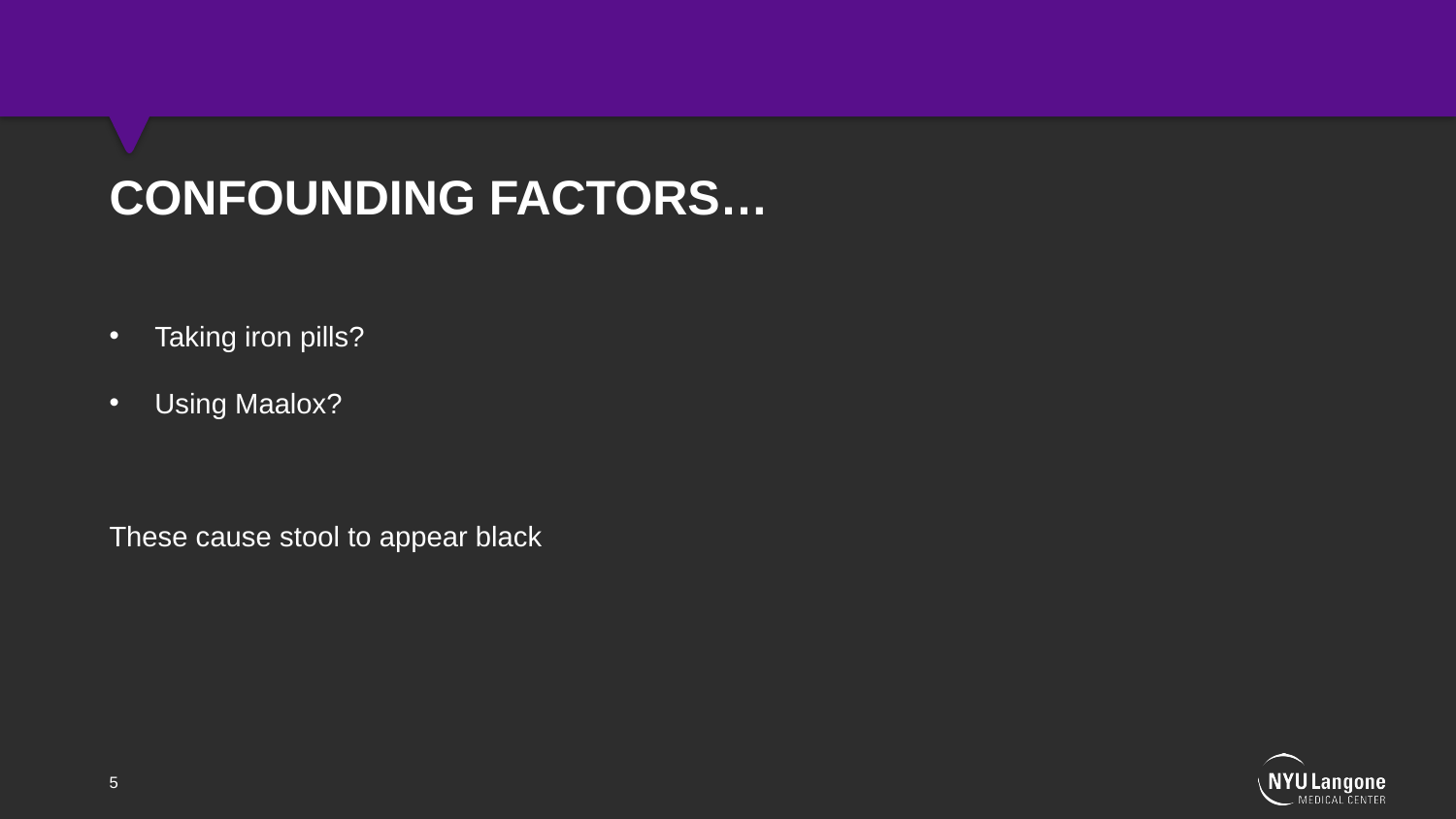

# CONFOUNDING FACTORS…
Taking iron pills?
Using Maalox?
These cause stool to appear black
5

## Slide 6
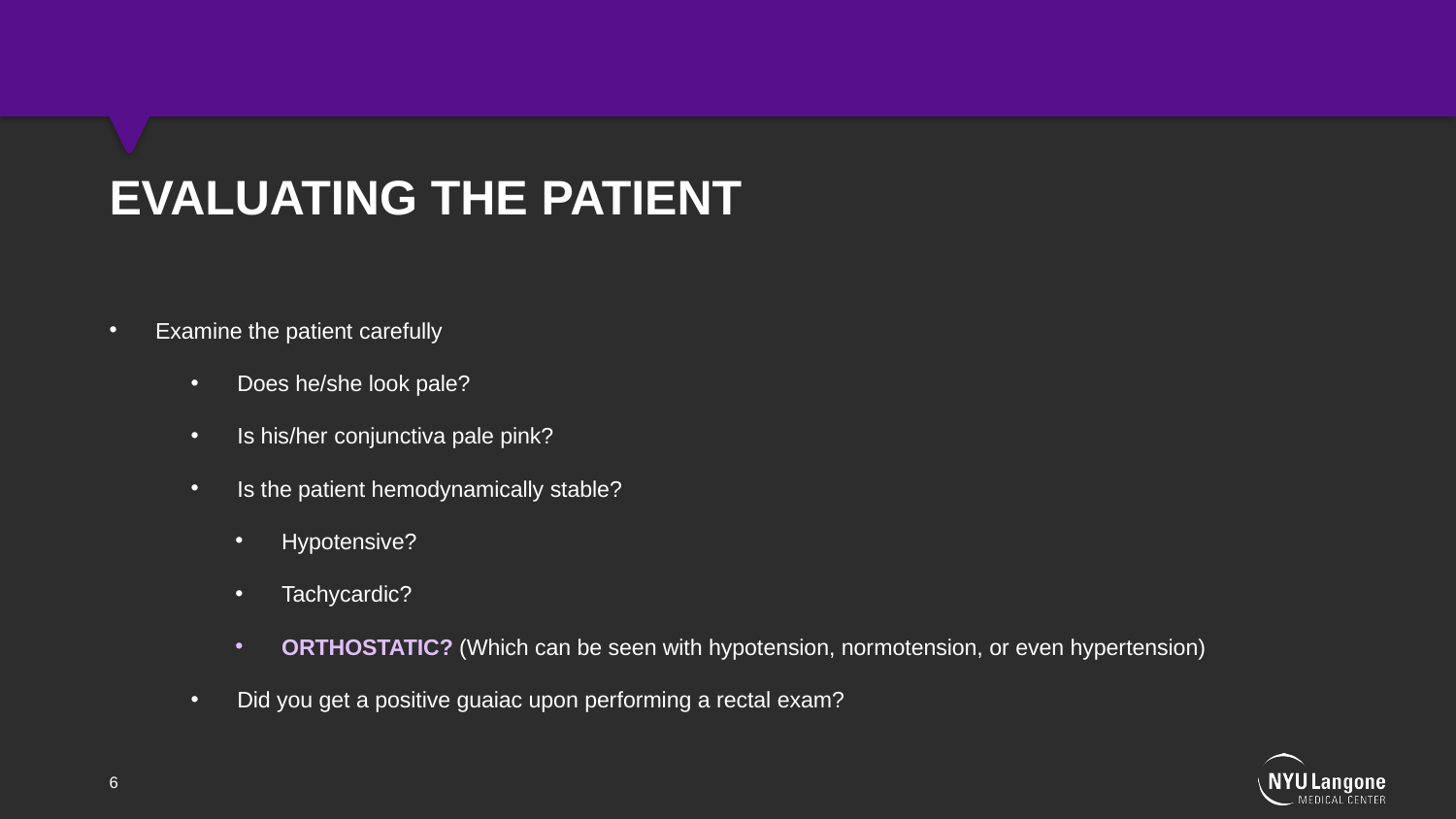

# EVALUATING THE PATIENT
Examine the patient carefully
Does he/she look pale?
Is his/her conjunctiva pale pink?
Is the patient hemodynamically stable?
Hypotensive?
Tachycardic?
ORTHOSTATIC? (Which can be seen with hypotension, normotension, or even hypertension)
Did you get a positive guaiac upon performing a rectal exam?
6

## Slide 7
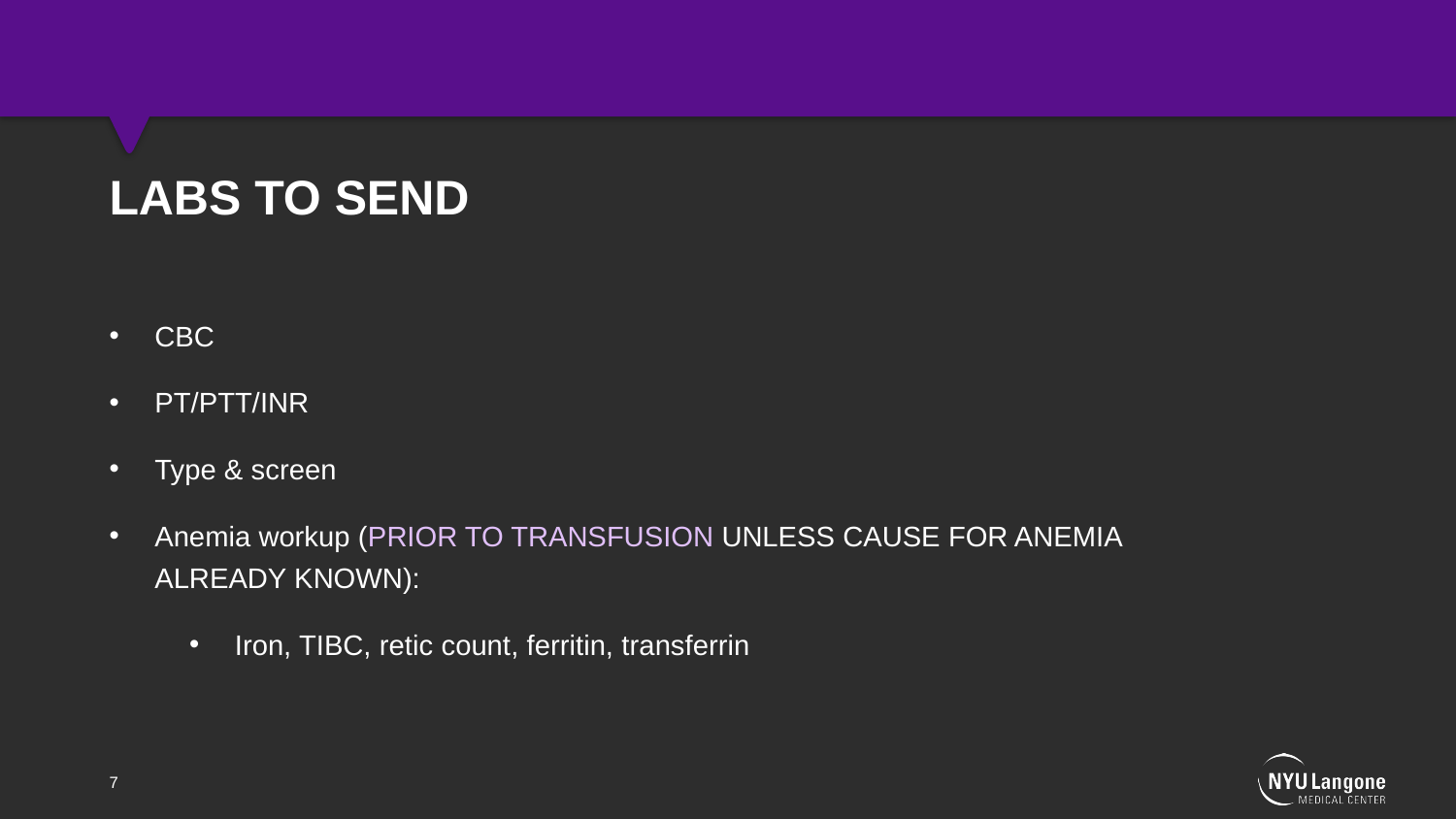

# LABS TO SEND
CBC
PT/PTT/INR
Type & screen
Anemia workup (PRIOR TO TRANSFUSION UNLESS CAUSE FOR ANEMIA ALREADY KNOWN):
Iron, TIBC, retic count, ferritin, transferrin
7

## Slide 8
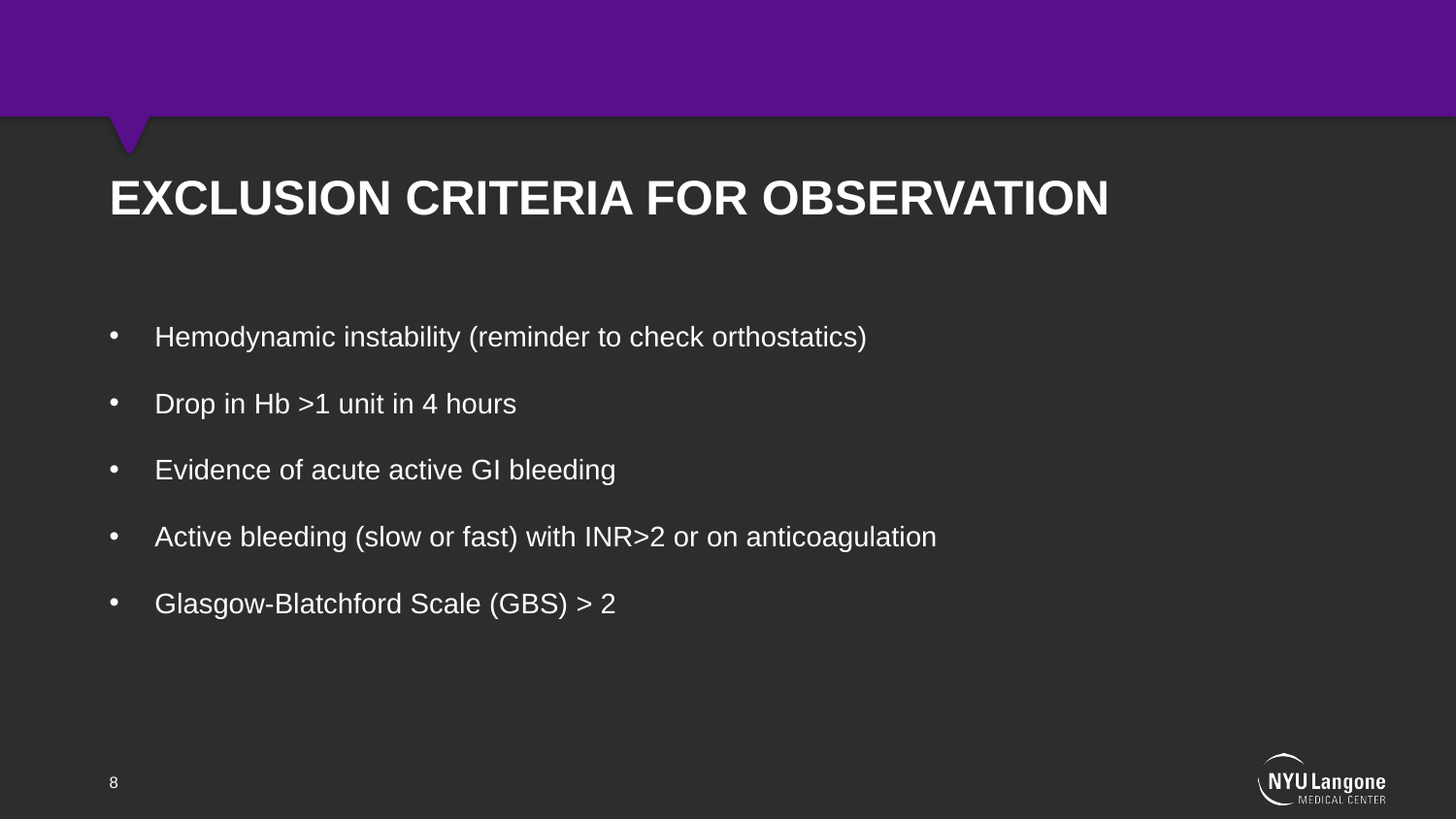

# EXCLUSION CRITERIA FOR OBSERVATION
Hemodynamic instability (reminder to check orthostatics)
Drop in Hb >1 unit in 4 hours
Evidence of acute active GI bleeding
Active bleeding (slow or fast) with INR>2 or on anticoagulation
Glasgow-Blatchford Scale (GBS) > 2
8

## Slide 9
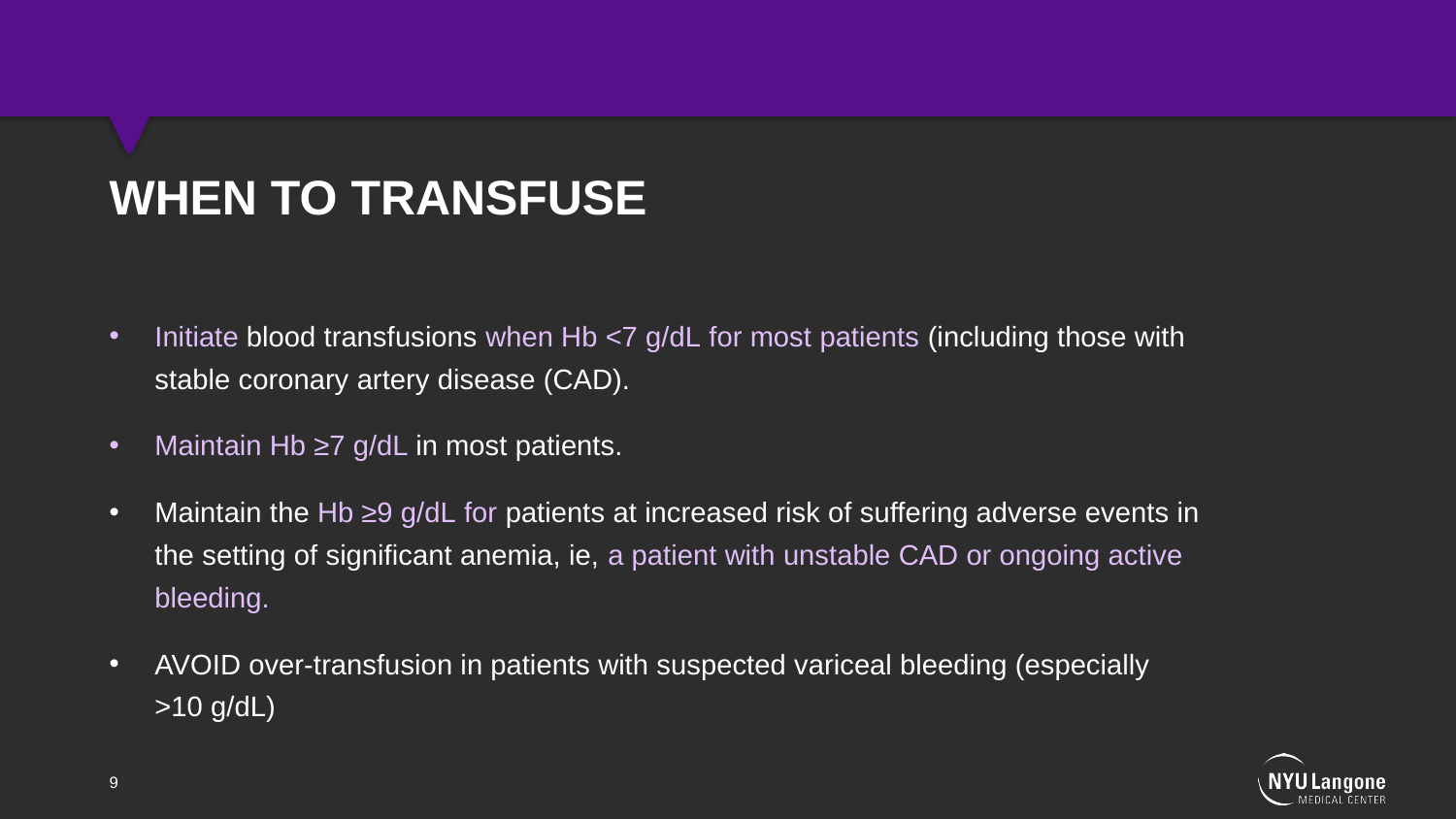

# WHEN TO TRANSFUSE
Initiate blood transfusions when Hb <7 g/dL for most patients (including those with stable coronary artery disease (CAD).
Maintain Hb ≥7 g/dL in most patients.
Maintain the Hb ≥9 g/dL for patients at increased risk of suffering adverse events in the setting of significant anemia, ie, a patient with unstable CAD or ongoing active bleeding.
AVOID over-transfusion in patients with suspected variceal bleeding (especially >10 g/dL)
9

## Slide 10
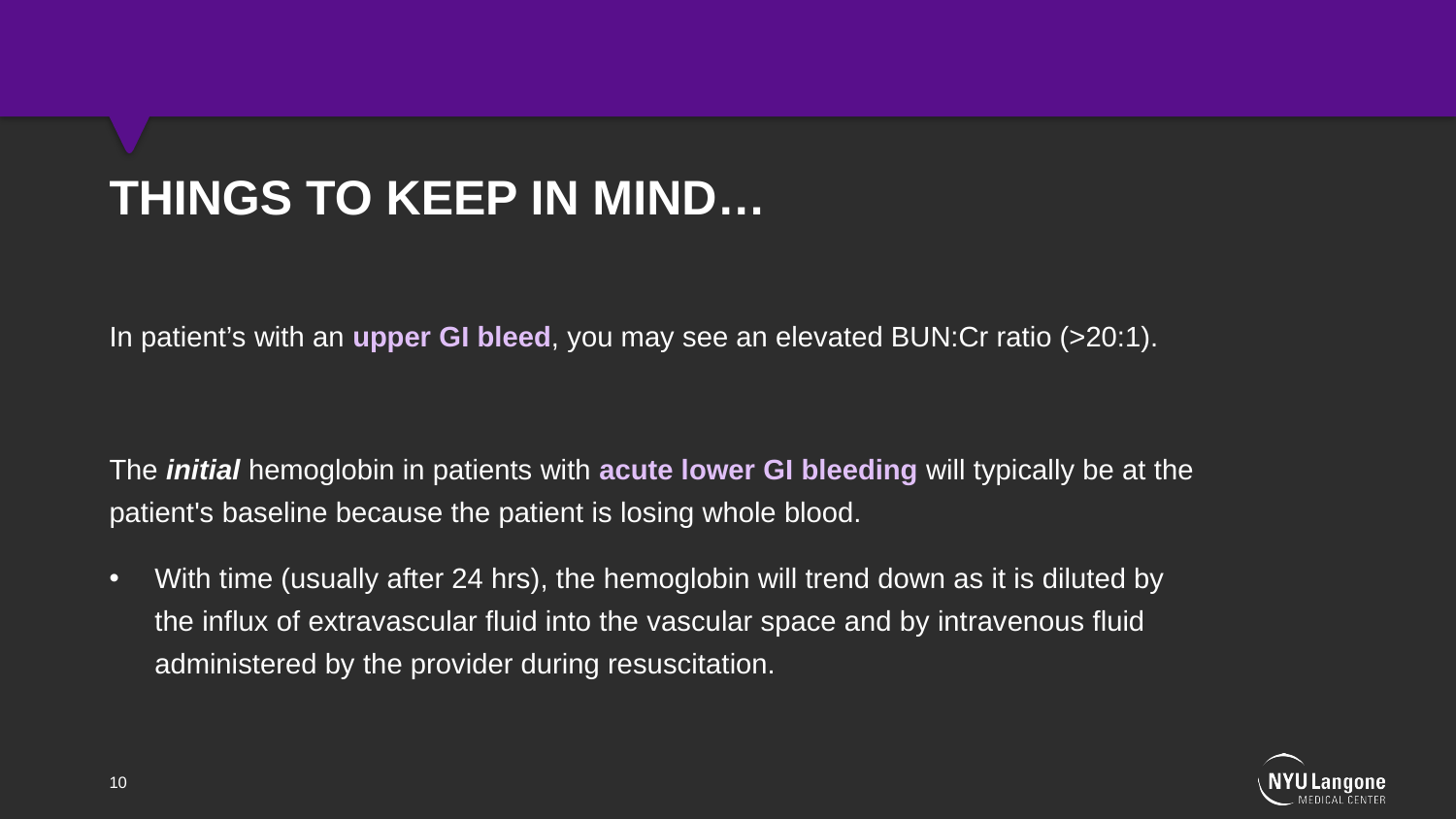

# THINGS TO KEEP IN MIND…
In patient’s with an upper GI bleed, you may see an elevated BUN:Cr ratio (>20:1).
The initial hemoglobin in patients with acute lower GI bleeding will typically be at the patient's baseline because the patient is losing whole blood.
With time (usually after 24 hrs), the hemoglobin will trend down as it is diluted by the influx of extravascular fluid into the vascular space and by intravenous fluid administered by the provider during resuscitation.
10

## Slide 11
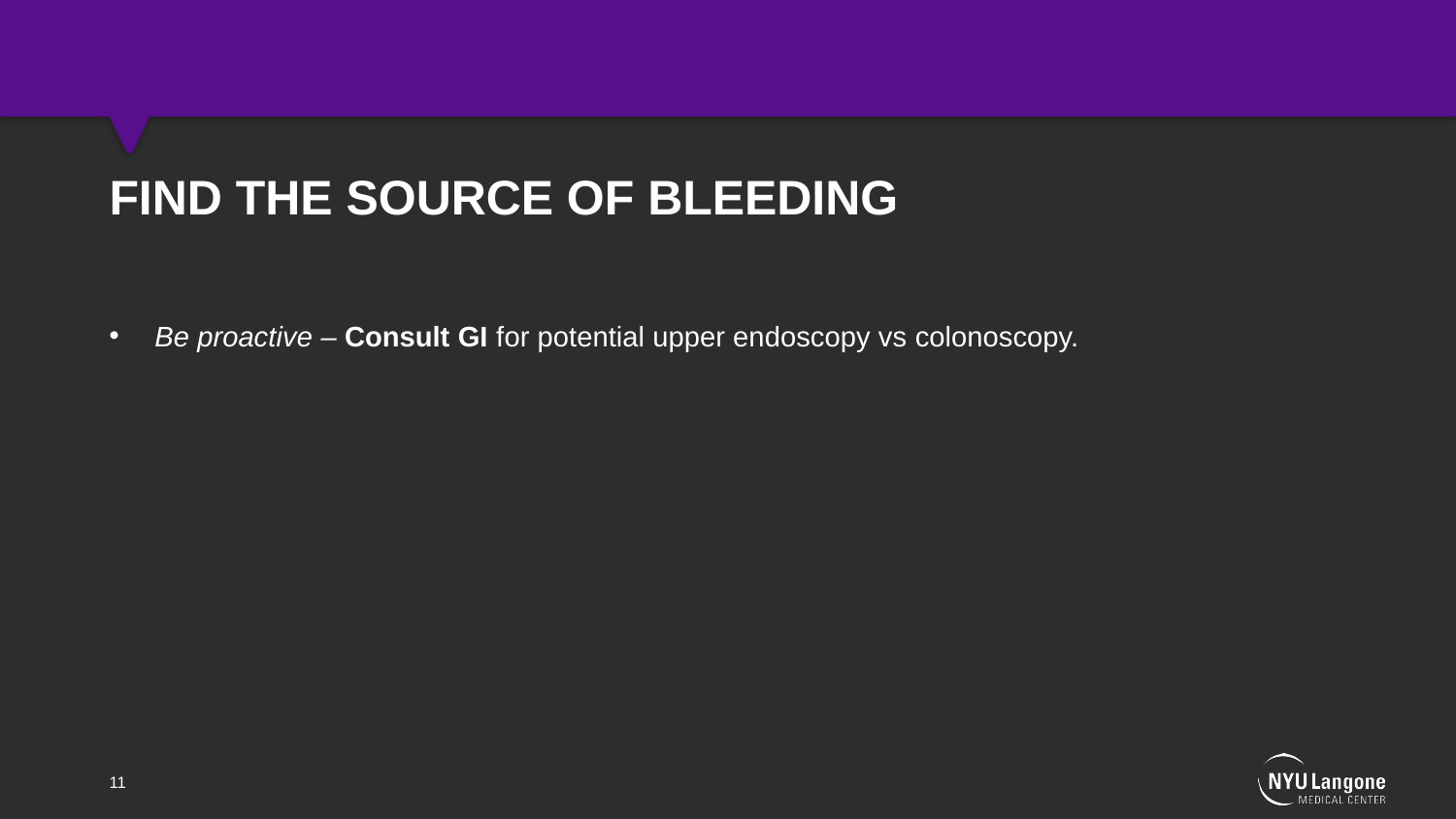

# FIND THE SOURCE OF BLEEDING
Be proactive – Consult GI for potential upper endoscopy vs colonoscopy.
11

## Slide 12
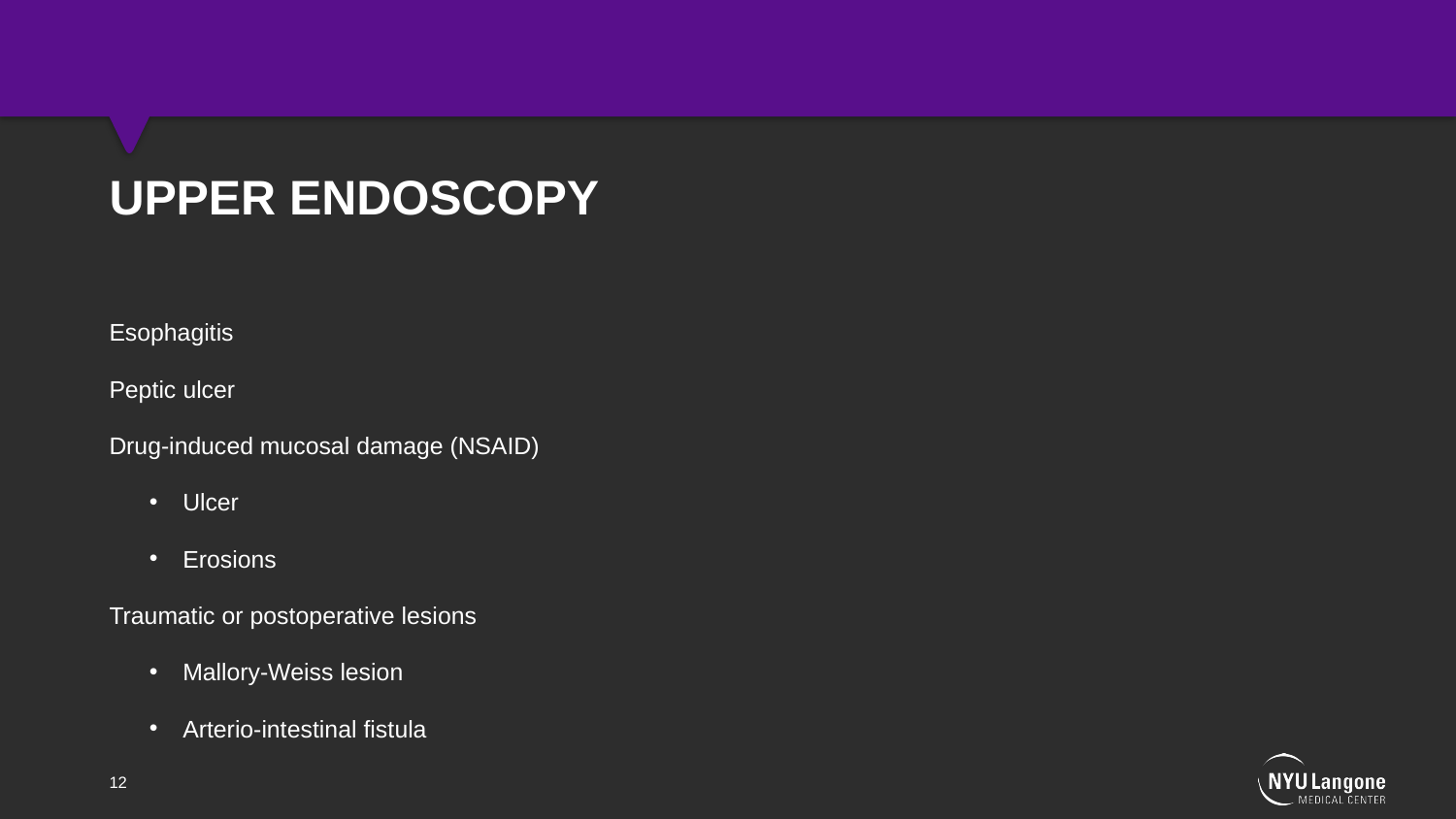

# UPPER ENDOSCOPY
Esophagitis
Peptic ulcer
Drug-induced mucosal damage (NSAID)
Ulcer
Erosions
Traumatic or postoperative lesions
Mallory-Weiss lesion
Arterio-intestinal fistula
12

## Slide 13
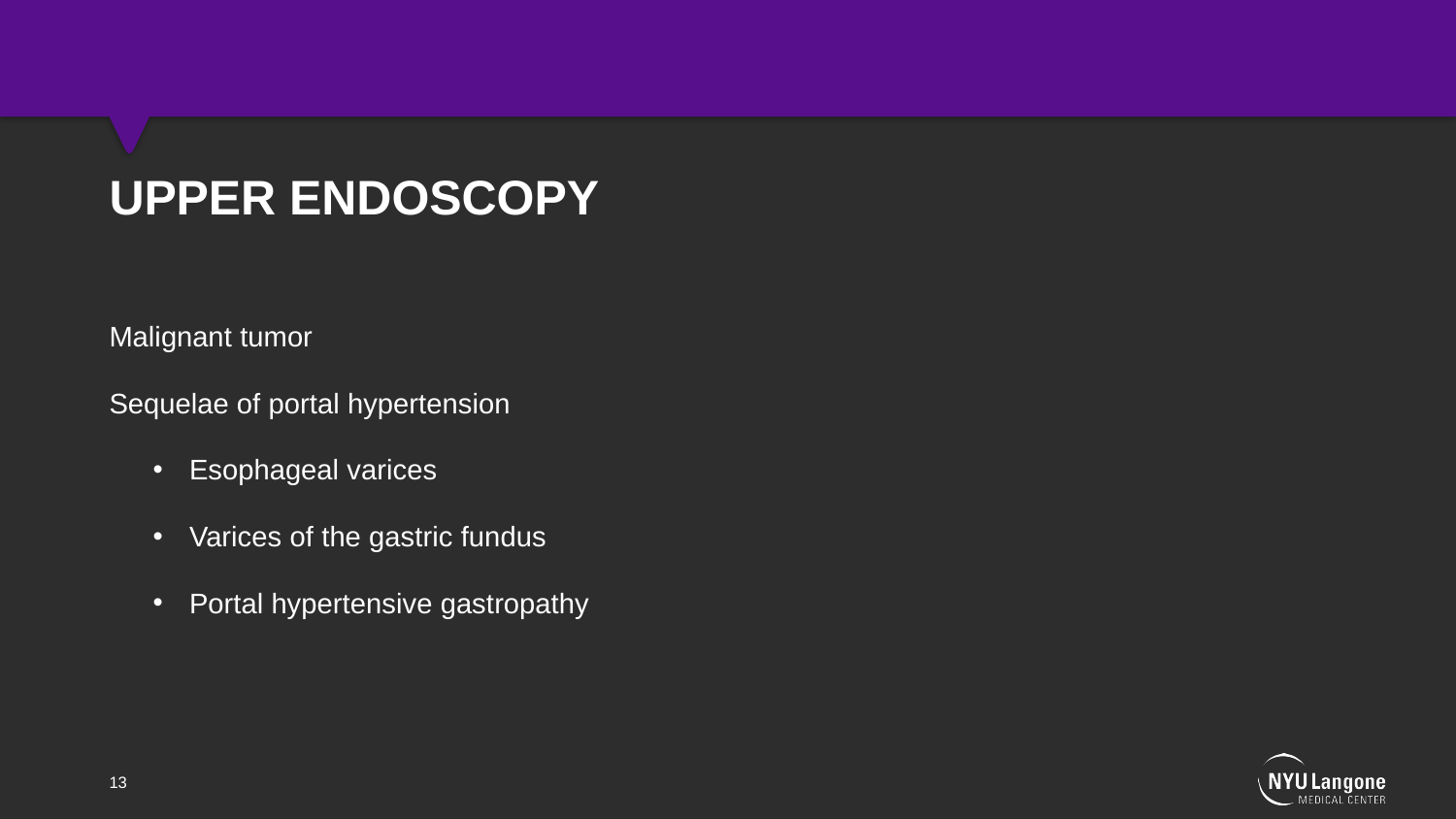

# UPPER ENDOSCOPY
Malignant tumor
Sequelae of portal hypertension
Esophageal varices
Varices of the gastric fundus
Portal hypertensive gastropathy
13

## Slide 14
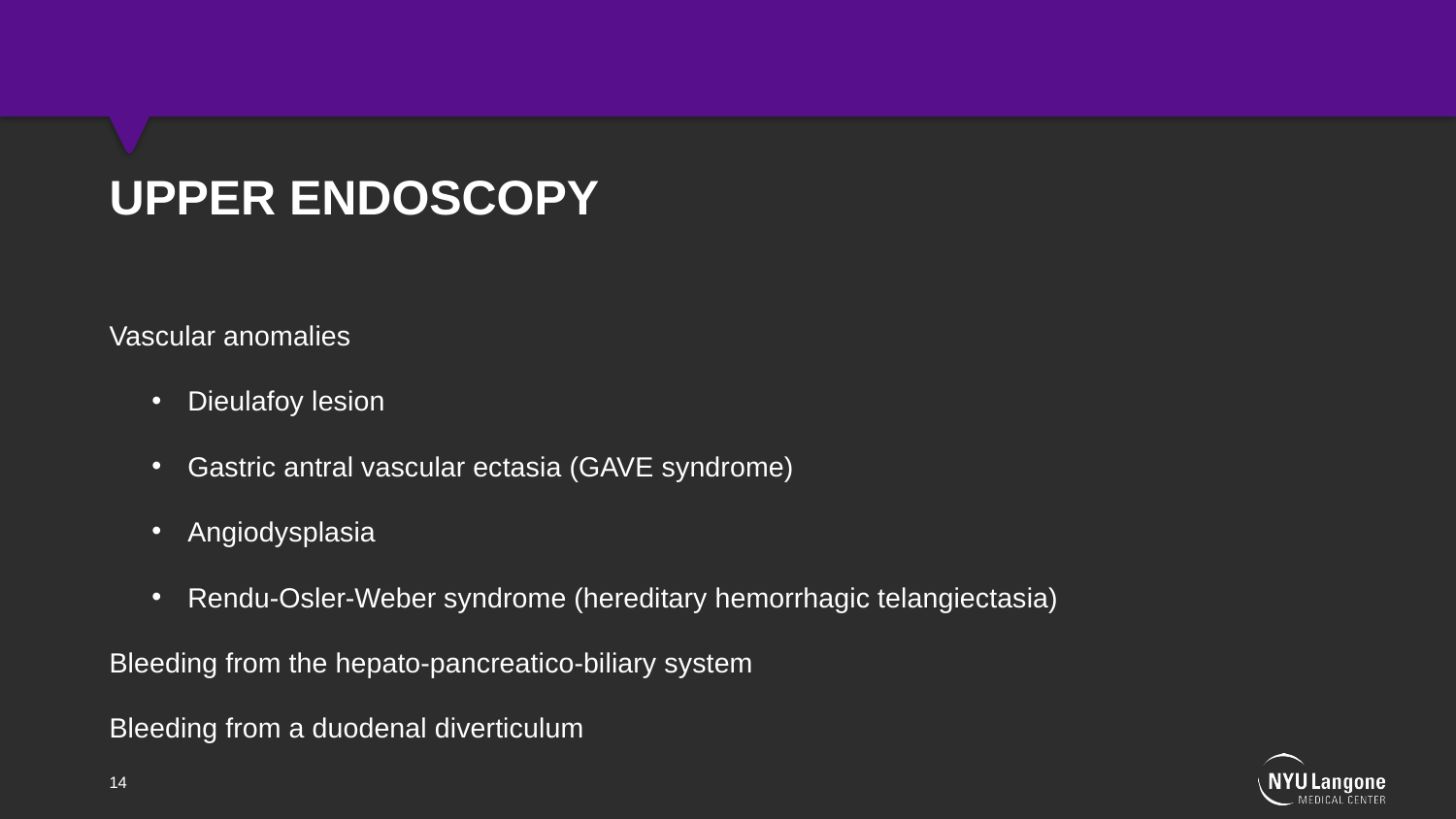

# UPPER ENDOSCOPY
Vascular anomalies
Dieulafoy lesion
Gastric antral vascular ectasia (GAVE syndrome)
Angiodysplasia
Rendu-Osler-Weber syndrome (hereditary hemorrhagic telangiectasia)
Bleeding from the hepato-pancreatico-biliary system
Bleeding from a duodenal diverticulum
14

## Slide 15
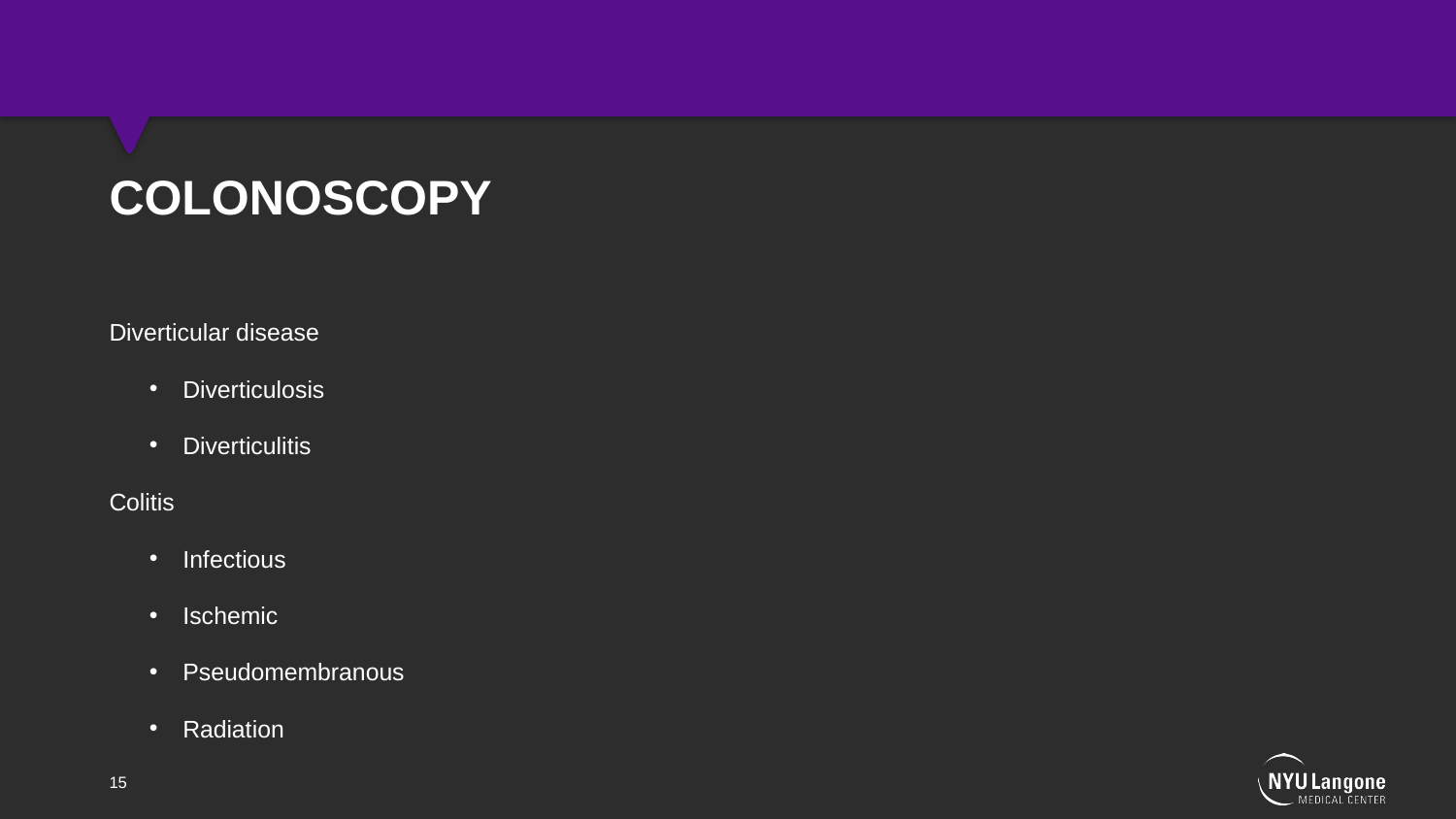

# COLONOSCOPY
Diverticular disease
Diverticulosis
Diverticulitis
Colitis
Infectious
Ischemic
Pseudomembranous
Radiation
15

## Slide 16
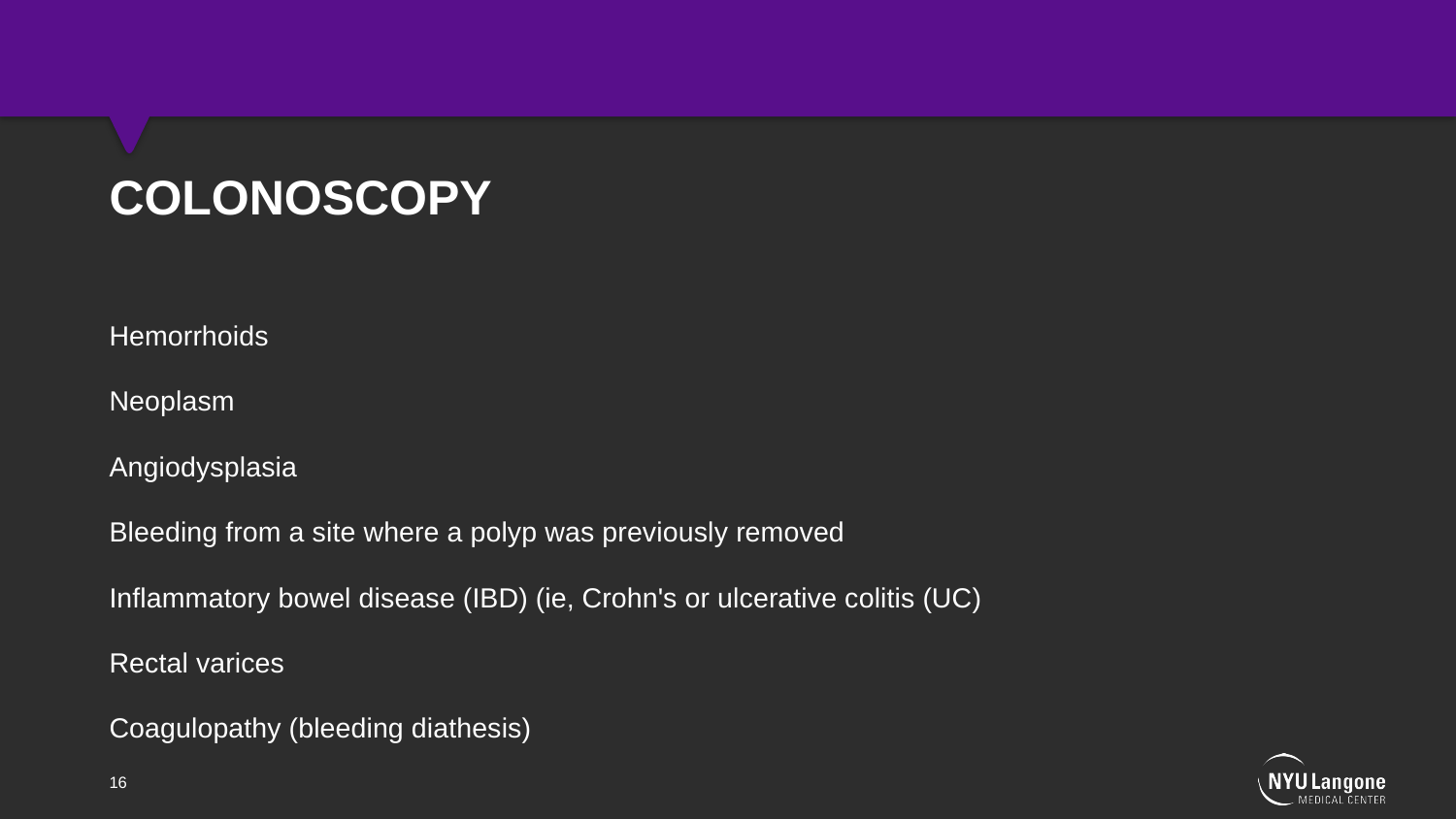

# COLONOSCOPY
Hemorrhoids
Neoplasm
Angiodysplasia
Bleeding from a site where a polyp was previously removed
Inflammatory bowel disease (IBD) (ie, Crohn's or ulcerative colitis (UC)
Rectal varices
Coagulopathy (bleeding diathesis)
16

## Slide 17
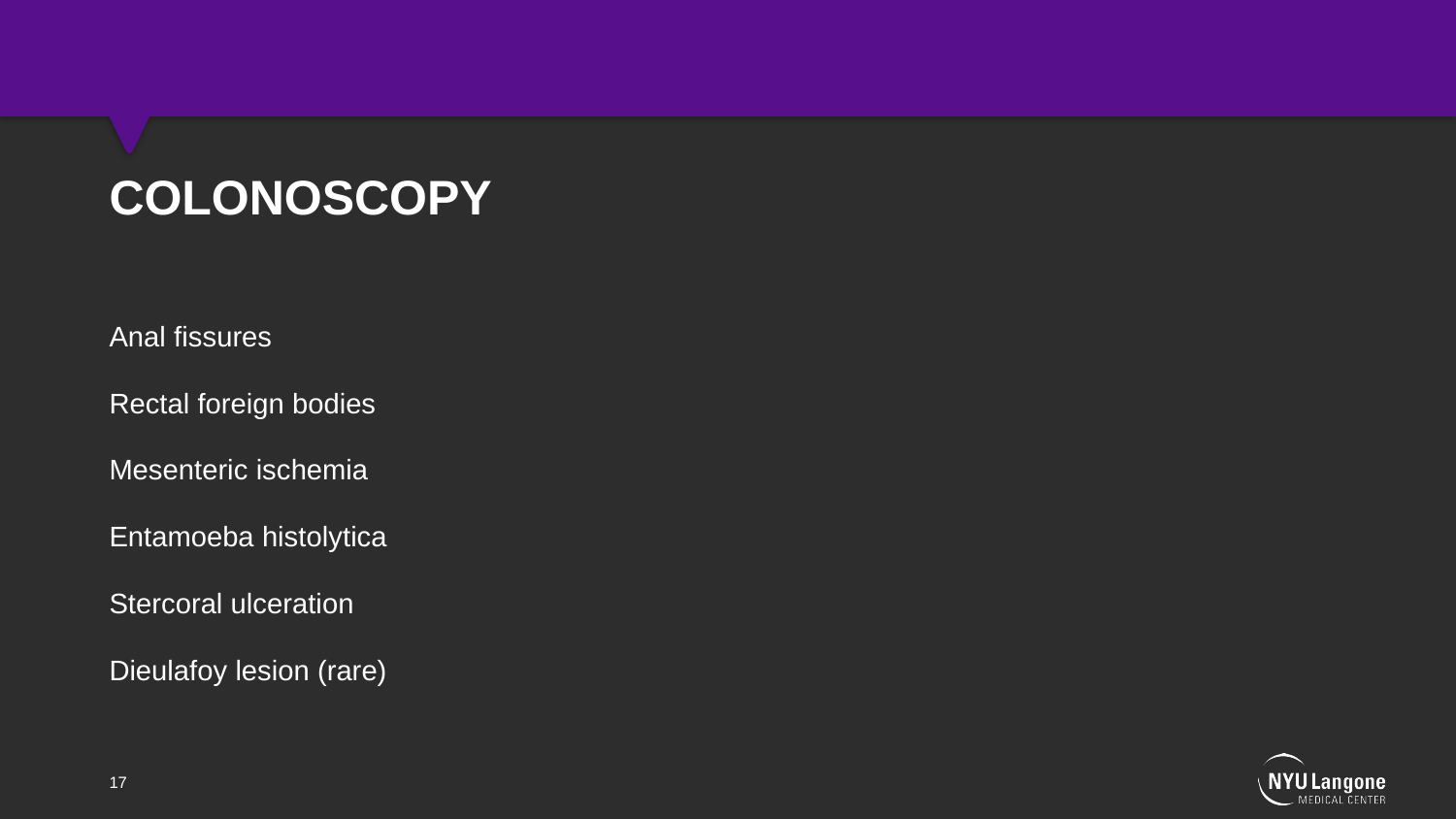

# COLONOSCOPY
Anal fissures
Rectal foreign bodies
Mesenteric ischemia
Entamoeba histolytica
Stercoral ulceration
Dieulafoy lesion (rare)
17

## Slide 18
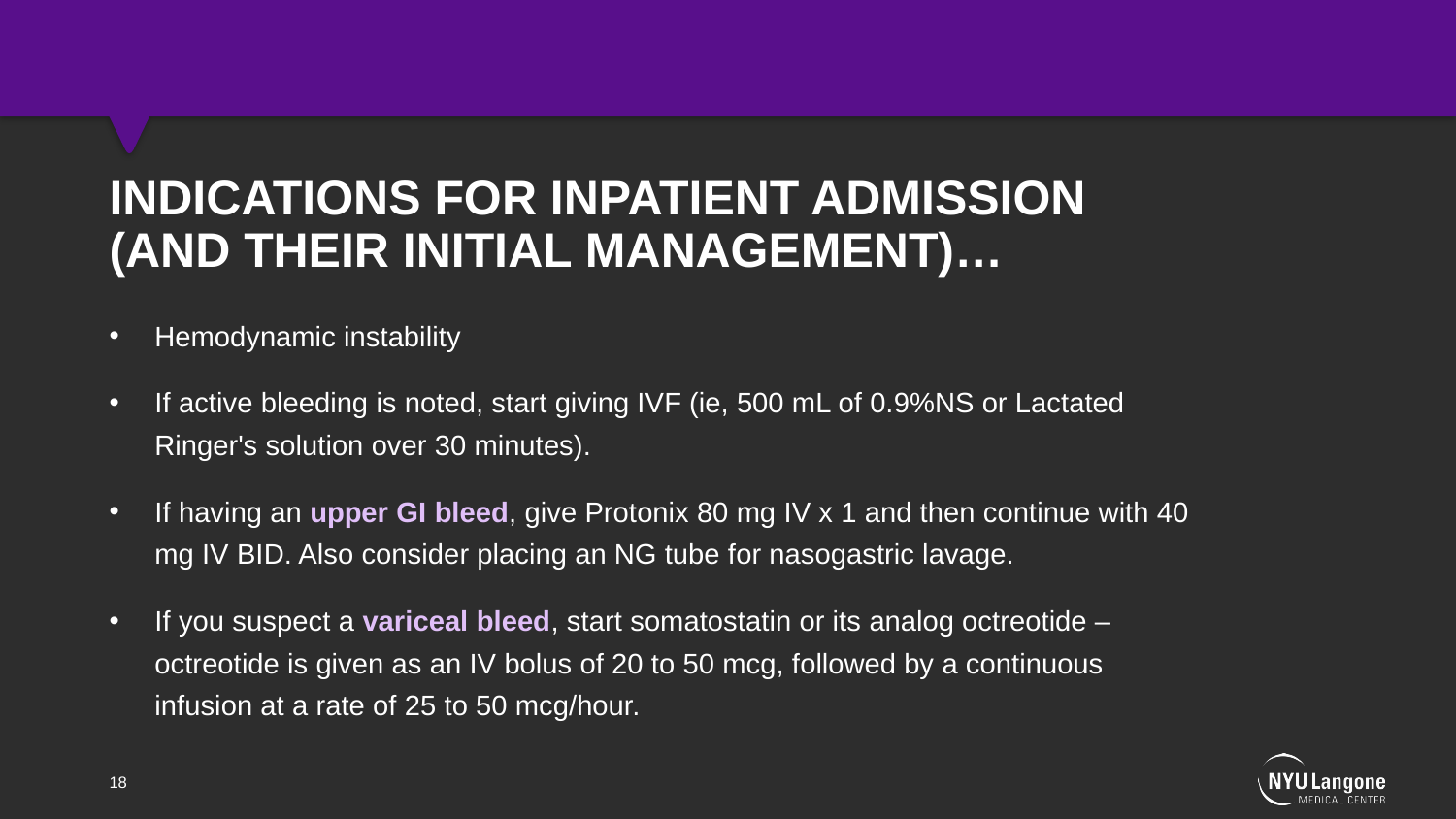

# INDICATIONS FOR INPATIENT ADMISSION (AND THEIR INITIAL MANAGEMENT)…
Hemodynamic instability
If active bleeding is noted, start giving IVF (ie, 500 mL of 0.9%NS or Lactated Ringer's solution over 30 minutes).
If having an upper GI bleed, give Protonix 80 mg IV x 1 and then continue with 40 mg IV BID. Also consider placing an NG tube for nasogastric lavage.
If you suspect a variceal bleed, start somatostatin or its analog octreotide – octreotide is given as an IV bolus of 20 to 50 mcg, followed by a continuous infusion at a rate of 25 to 50 mcg/hour.
18

## Slide 19
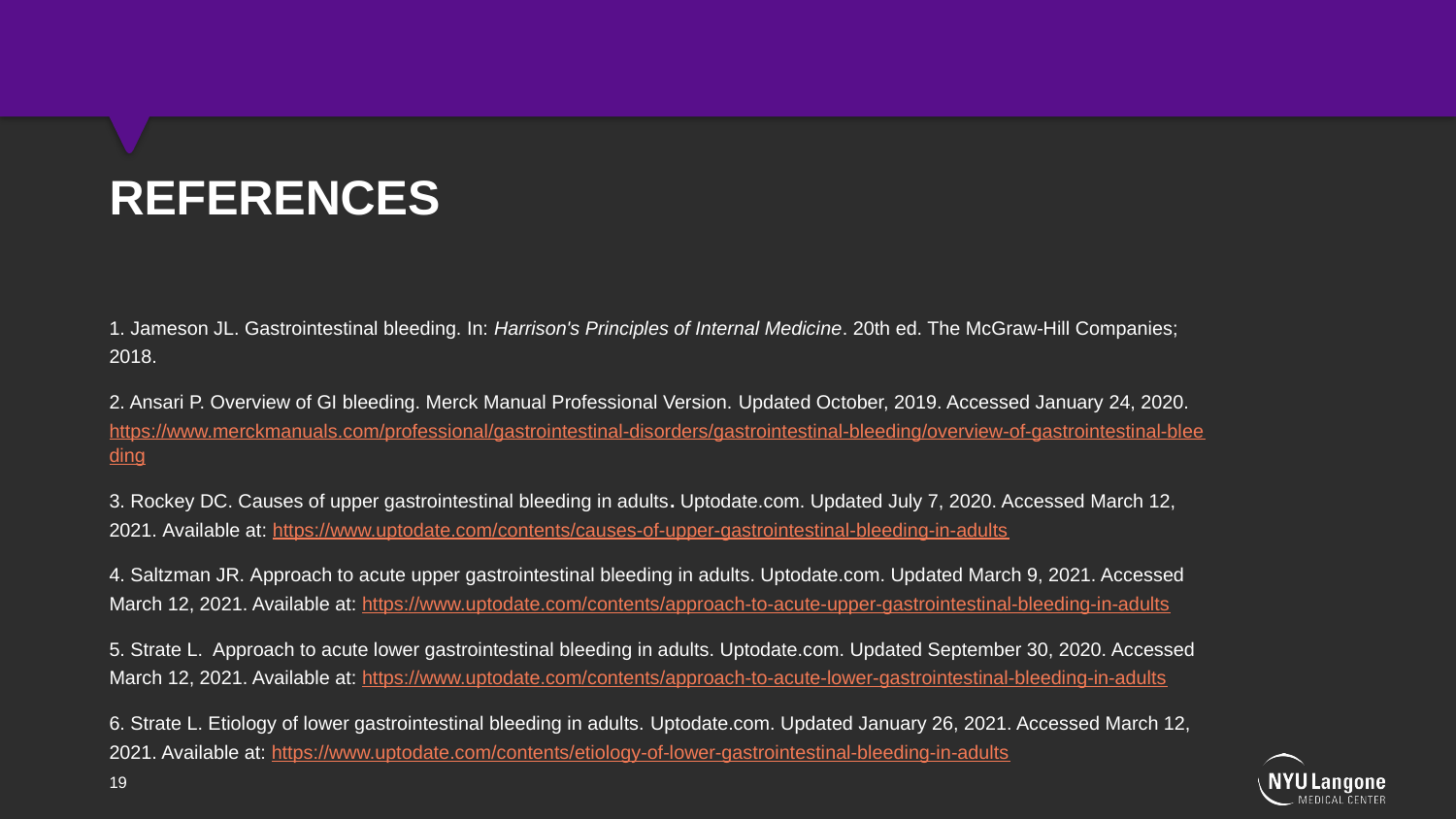

# REFERENCES
1. Jameson JL. Gastrointestinal bleeding. In: Harrison's Principles of Internal Medicine. 20th ed. The McGraw-Hill Companies; 2018.
2. Ansari P. Overview of GI bleeding. Merck Manual Professional Version. Updated October, 2019. Accessed January 24, 2020. https://www.merckmanuals.com/professional/gastrointestinal-disorders/gastrointestinal-bleeding/overview-of-gastrointestinal-bleeding
3. Rockey DC. Causes of upper gastrointestinal bleeding in adults. Uptodate.com. Updated July 7, 2020. Accessed March 12, 2021. Available at: https://www.uptodate.com/contents/causes-of-upper-gastrointestinal-bleeding-in-adults
4. Saltzman JR. Approach to acute upper gastrointestinal bleeding in adults. Uptodate.com. Updated March 9, 2021. Accessed March 12, 2021. Available at: https://www.uptodate.com/contents/approach-to-acute-upper-gastrointestinal-bleeding-in-adults
5. Strate L. Approach to acute lower gastrointestinal bleeding in adults. Uptodate.com. Updated September 30, 2020. Accessed March 12, 2021. Available at: https://www.uptodate.com/contents/approach-to-acute-lower-gastrointestinal-bleeding-in-adults
6. Strate L. Etiology of lower gastrointestinal bleeding in adults. Uptodate.com. Updated January 26, 2021. Accessed March 12, 2021. Available at: https://www.uptodate.com/contents/etiology-of-lower-gastrointestinal-bleeding-in-adults
19
