# Supplementary material for: An Observation Medicine Curriculum for Emergency Medicine Education
Source: J Educ Teach Emerg Med. 2021 Apr 19;6(2):C1–C72. doi: 10.21980/J87P92 (PMC10332786; doi:10.21980/J87P92)
Supplement: Supplementary file 5 — Please see associated PowerPoint file [file jetem-6-2-c1-supp5.pptx]

## Slide 1
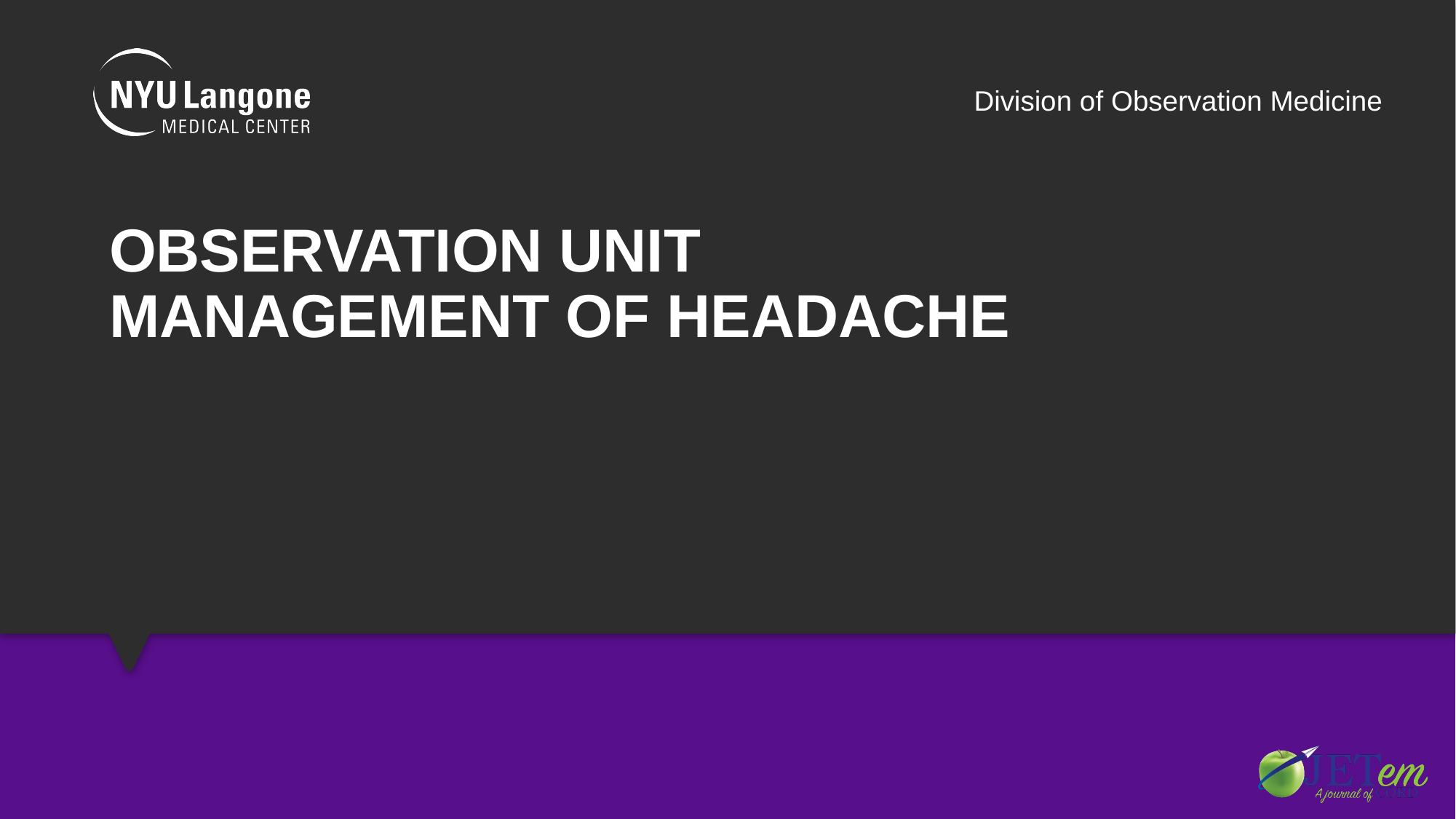

Division of Observation Medicine
# Observation Unit Management of Headache

## Slide 2
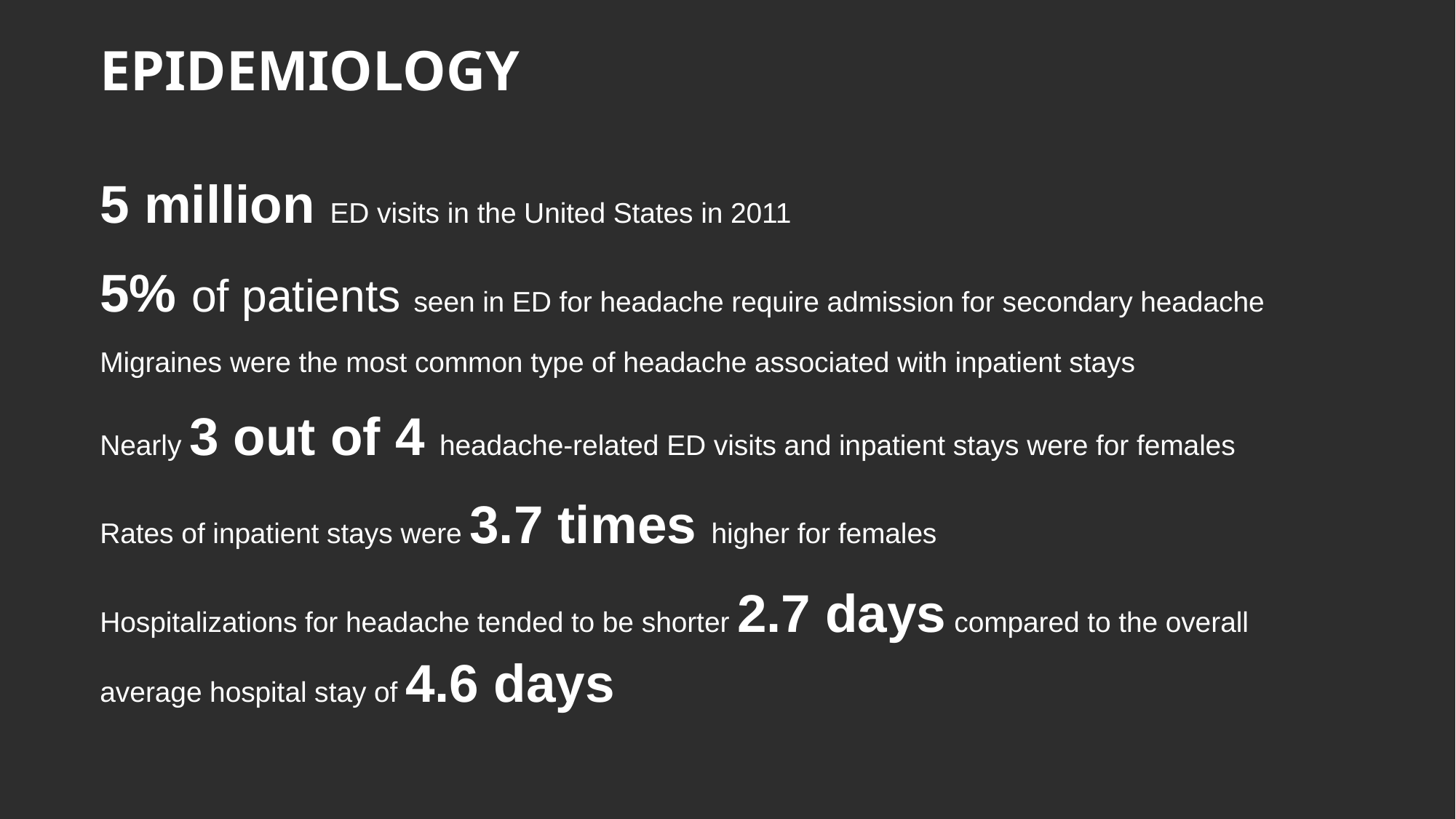

# Epidemiology
5 million ED visits in the United States in 2011
5% of patients seen in ED for headache require admission for secondary headache
Migraines were the most common type of headache associated with inpatient stays
Nearly 3 out of 4 headache-related ED visits and inpatient stays were for females
Rates of inpatient stays were 3.7 times higher for females
Hospitalizations for headache tended to be shorter 2.7 days compared to the overall average hospital stay of 4.6 days

## Slide 3
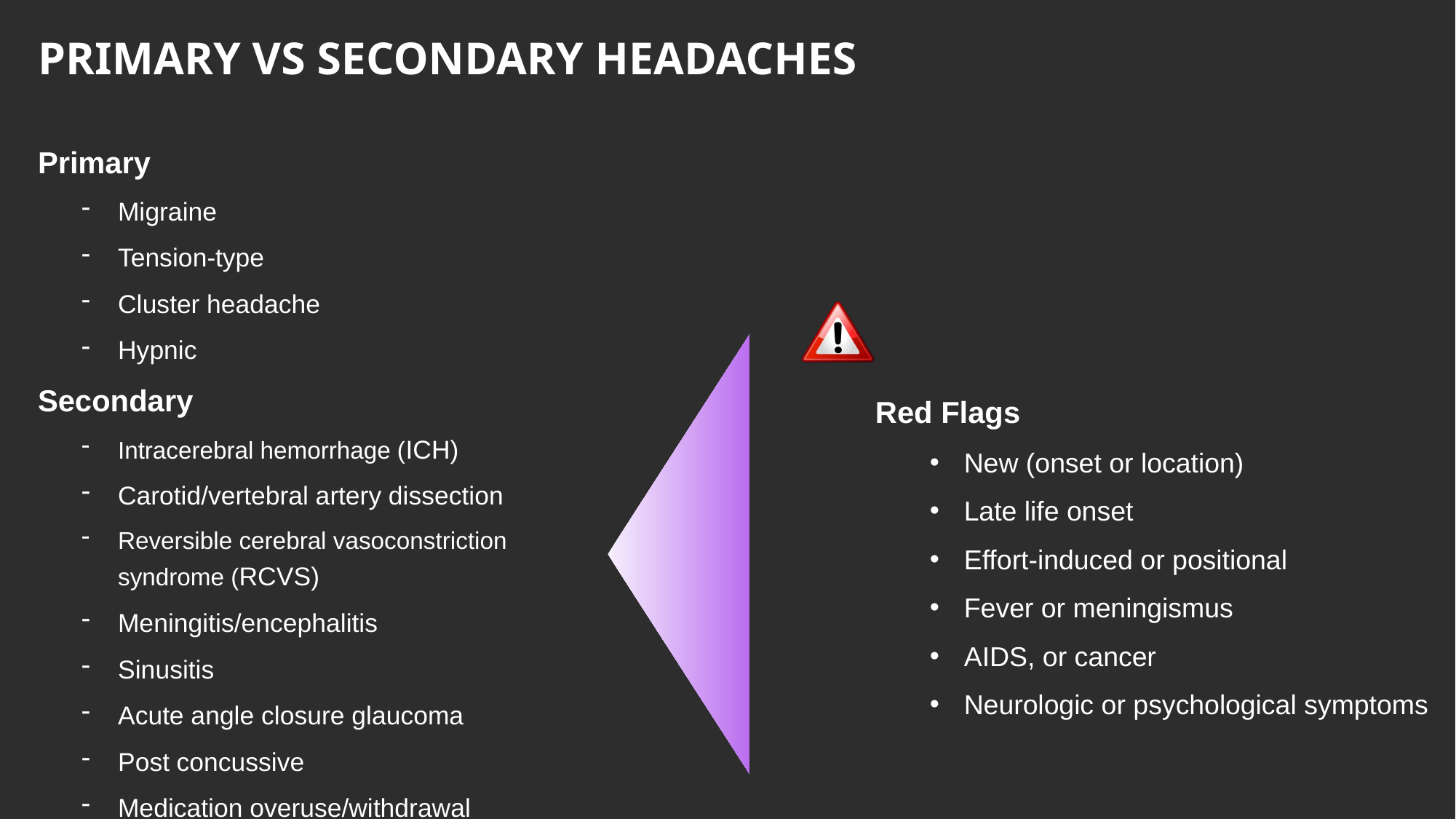

# Primary vs Secondary Headaches
Primary
Migraine
Tension-type
Cluster headache
Hypnic
Secondary
Intracerebral hemorrhage (ICH)
Carotid/vertebral artery dissection
Reversible cerebral vasoconstriction syndrome (RCVS)
Meningitis/encephalitis
Sinusitis
Acute angle closure glaucoma
Post concussive
Medication overuse/withdrawal
Red Flags
New (onset or location)
Late life onset
Effort-induced or positional
Fever or meningismus
AIDS, or cancer
Neurologic or psychological symptoms

## Slide 4
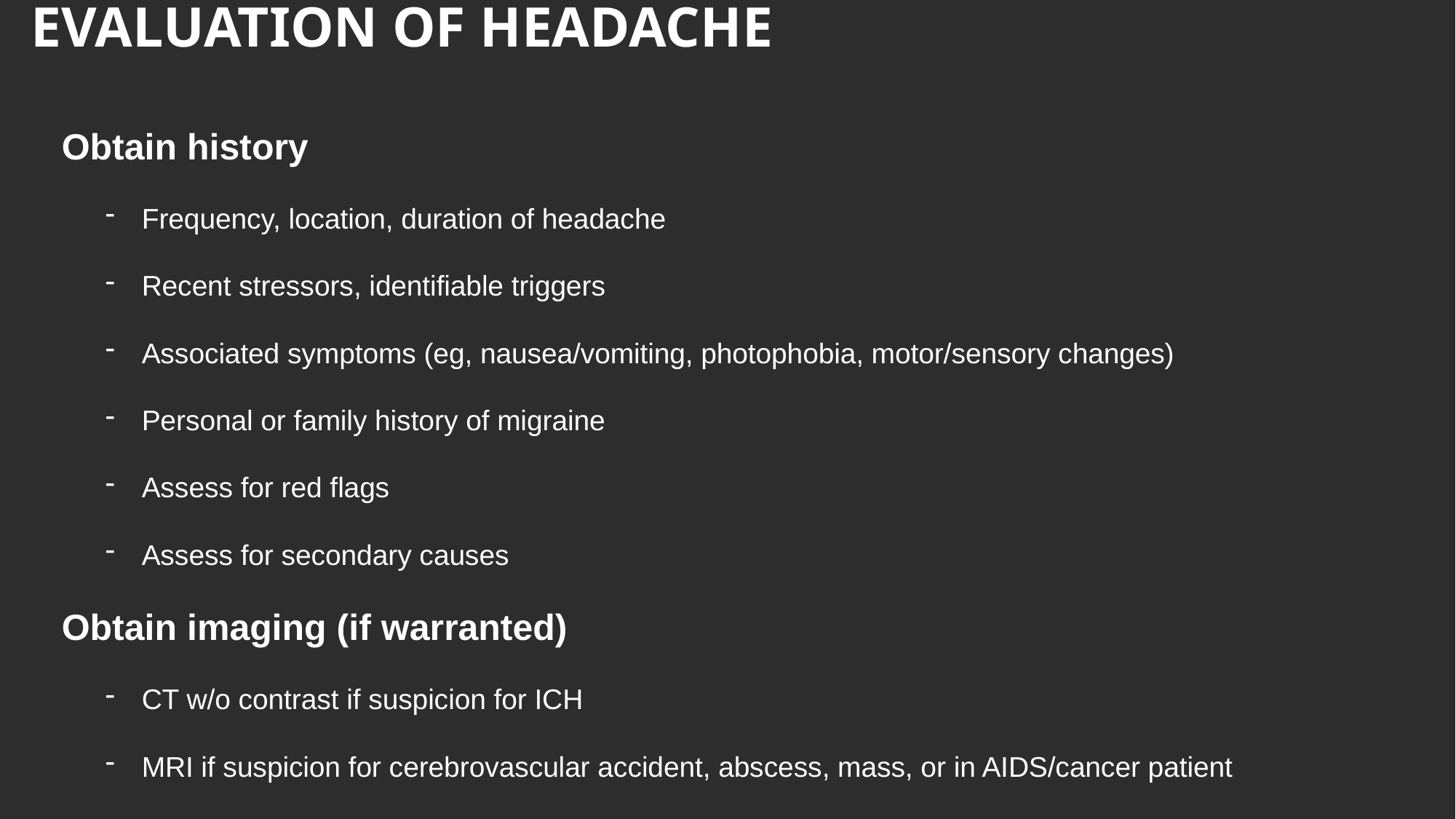

# Evaluation of Headache
Obtain history
Frequency, location, duration of headache
Recent stressors, identifiable triggers
Associated symptoms (eg, nausea/vomiting, photophobia, motor/sensory changes)
Personal or family history of migraine
Assess for red flags
Assess for secondary causes
Obtain imaging (if warranted)
CT w/o contrast if suspicion for ICH
MRI if suspicion for cerebrovascular accident, abscess, mass, or in AIDS/cancer patient

## Slide 5
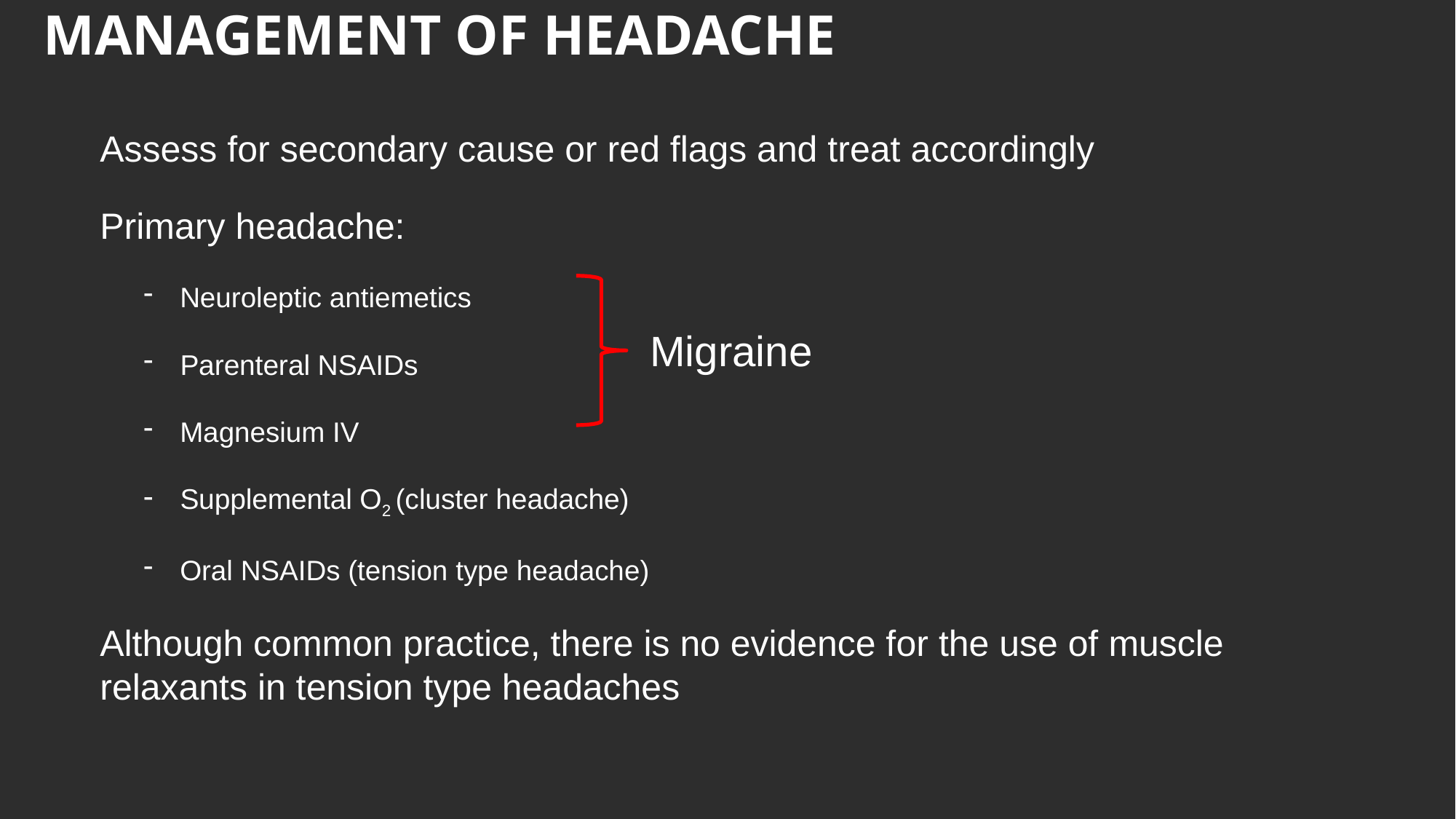

# Management of Headache
Assess for secondary cause or red flags and treat accordingly
Primary headache:
Neuroleptic antiemetics
Parenteral NSAIDs
Magnesium IV
Supplemental O2 (cluster headache)
Oral NSAIDs (tension type headache)
Although common practice, there is no evidence for the use of muscle relaxants in tension type headaches
Migraine

## Slide 6
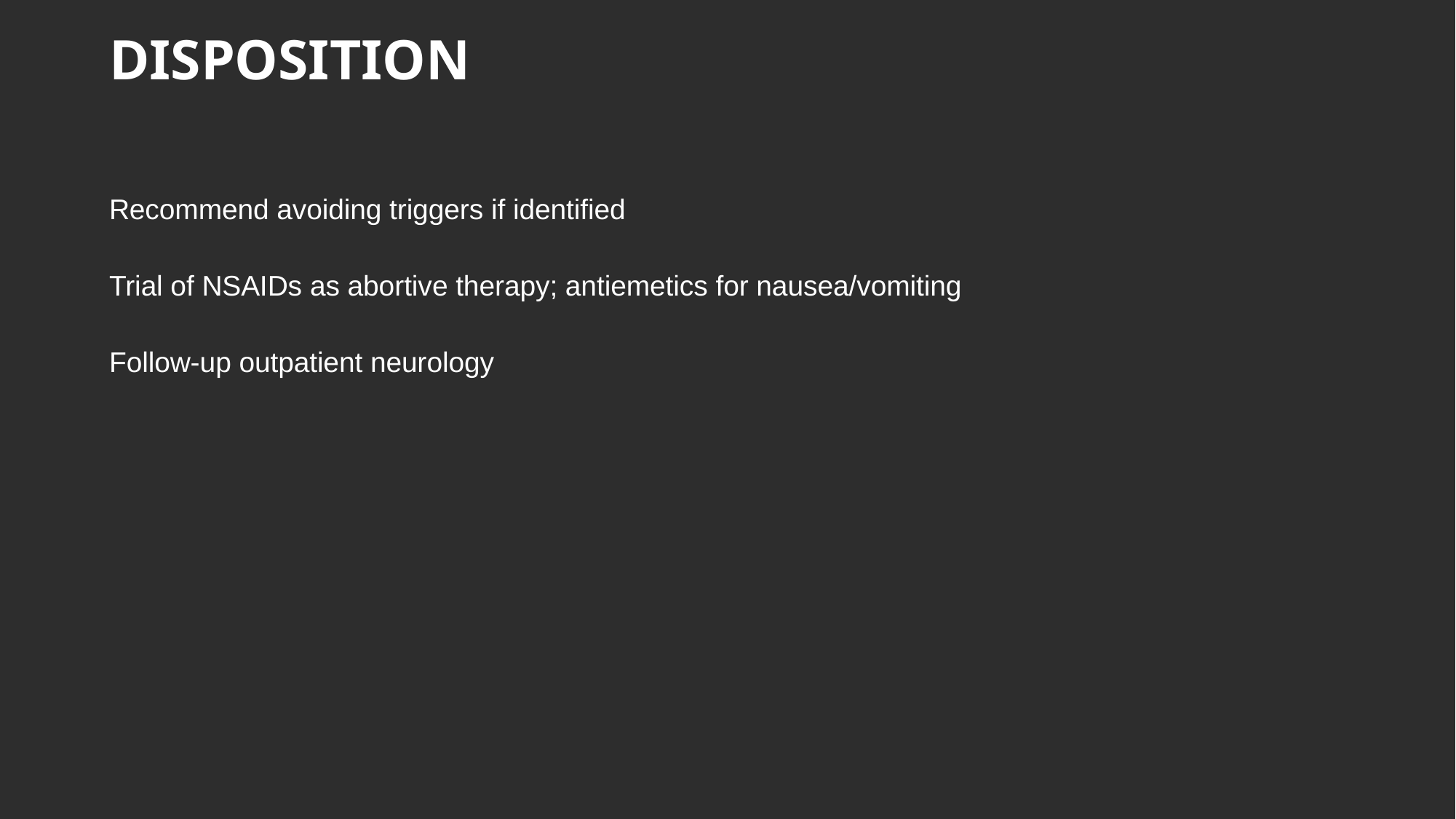

# Disposition
Recommend avoiding triggers if identified
Trial of NSAIDs as abortive therapy; antiemetics for nausea/vomiting
Follow-up outpatient neurology

## Slide 7
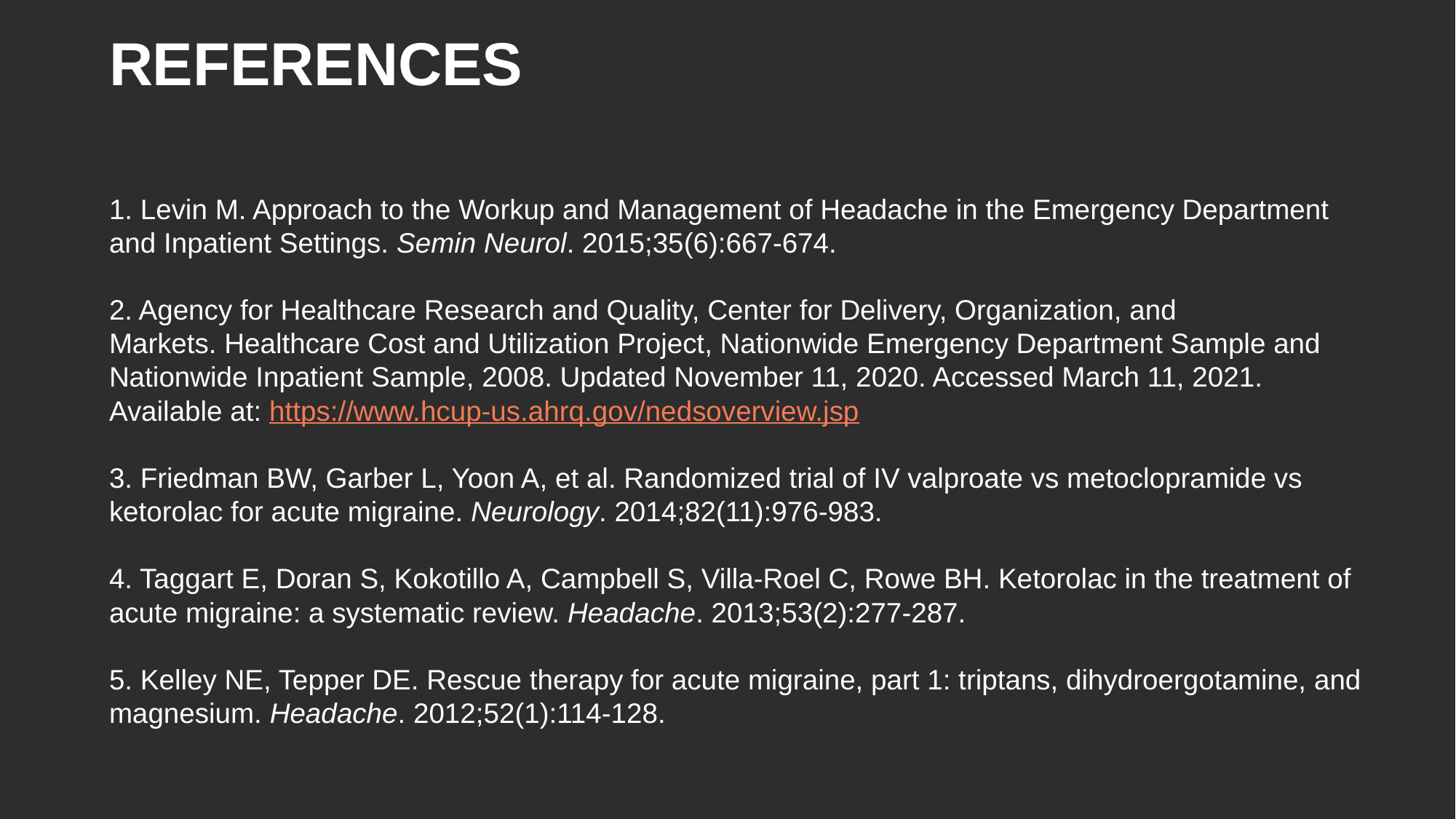

# References
1. Levin M. Approach to the Workup and Management of Headache in the Emergency Department and Inpatient Settings. Semin Neurol. 2015;35(6):667-674.
2. Agency for Healthcare Research and Quality, Center for Delivery, Organization, and Markets. Healthcare Cost and Utilization Project, Nationwide Emergency Department Sample and Nationwide Inpatient Sample, 2008. Updated November 11, 2020. Accessed March 11, 2021. Available at: https://www.hcup-us.ahrq.gov/nedsoverview.jsp
3. Friedman BW, Garber L, Yoon A, et al. Randomized trial of IV valproate vs metoclopramide vs ketorolac for acute migraine. Neurology. 2014;82(11):976-983.
4. Taggart E, Doran S, Kokotillo A, Campbell S, Villa-Roel C, Rowe BH. Ketorolac in the treatment of acute migraine: a systematic review. Headache. 2013;53(2):277-287.
5. Kelley NE, Tepper DE. Rescue therapy for acute migraine, part 1: triptans, dihydroergotamine, and magnesium. Headache. 2012;52(1):114-128.
