# Supplementary material for: An Observation Medicine Curriculum for Emergency Medicine Education
Source: J Educ Teach Emerg Med. 2021 Apr 19;6(2):C1–C72. doi: 10.21980/J87P92 (PMC10332786; doi:10.21980/J87P92)
Supplement: Supplementary file 7 — Please see associated PowerPoint file [file jetem-6-2-c1-supp7.pptx]

## Slide 1
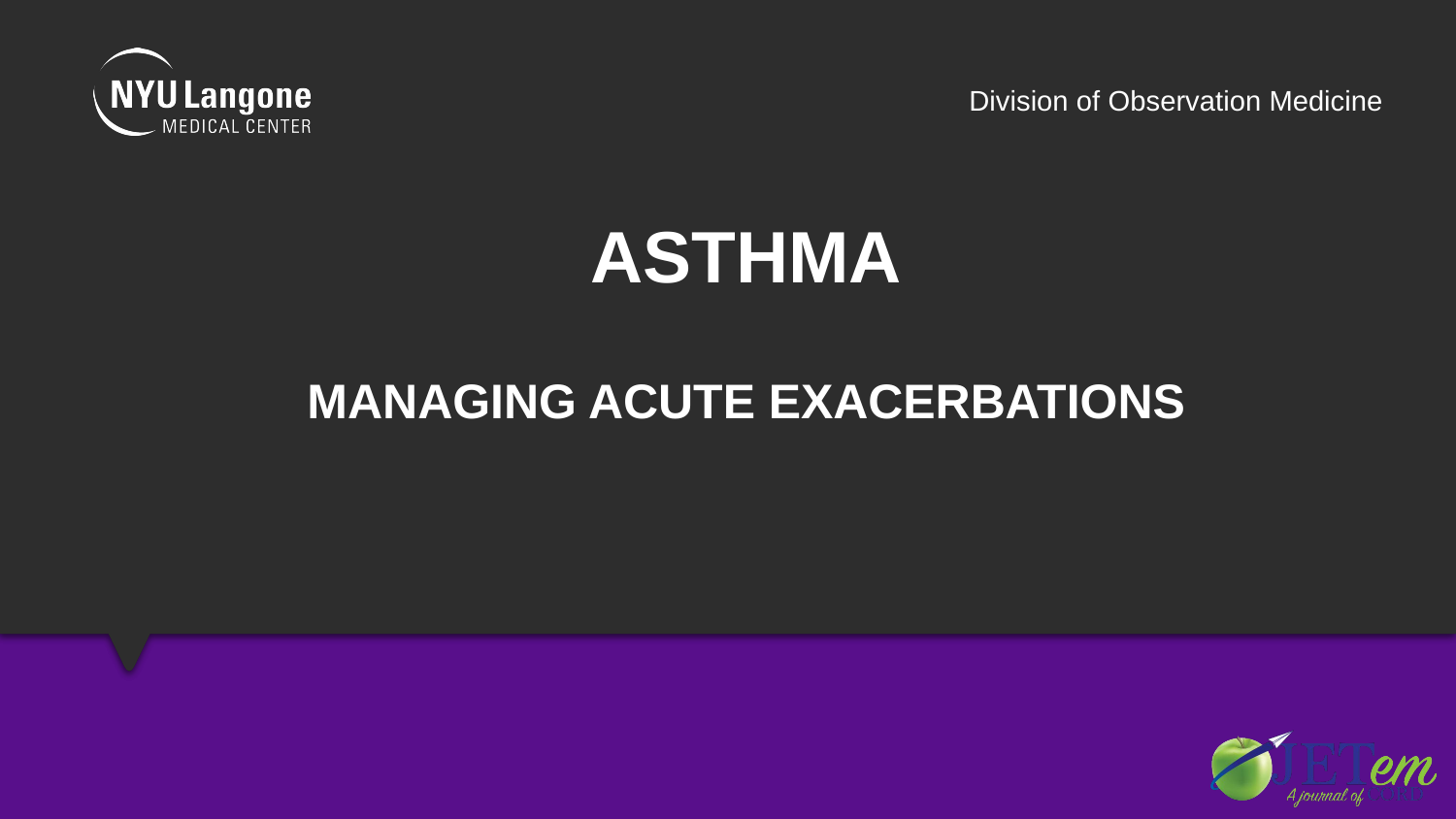

Division of Observation Medicine
# ASTHMA Managing ACUTE EXACERBATIONS

## Slide 2
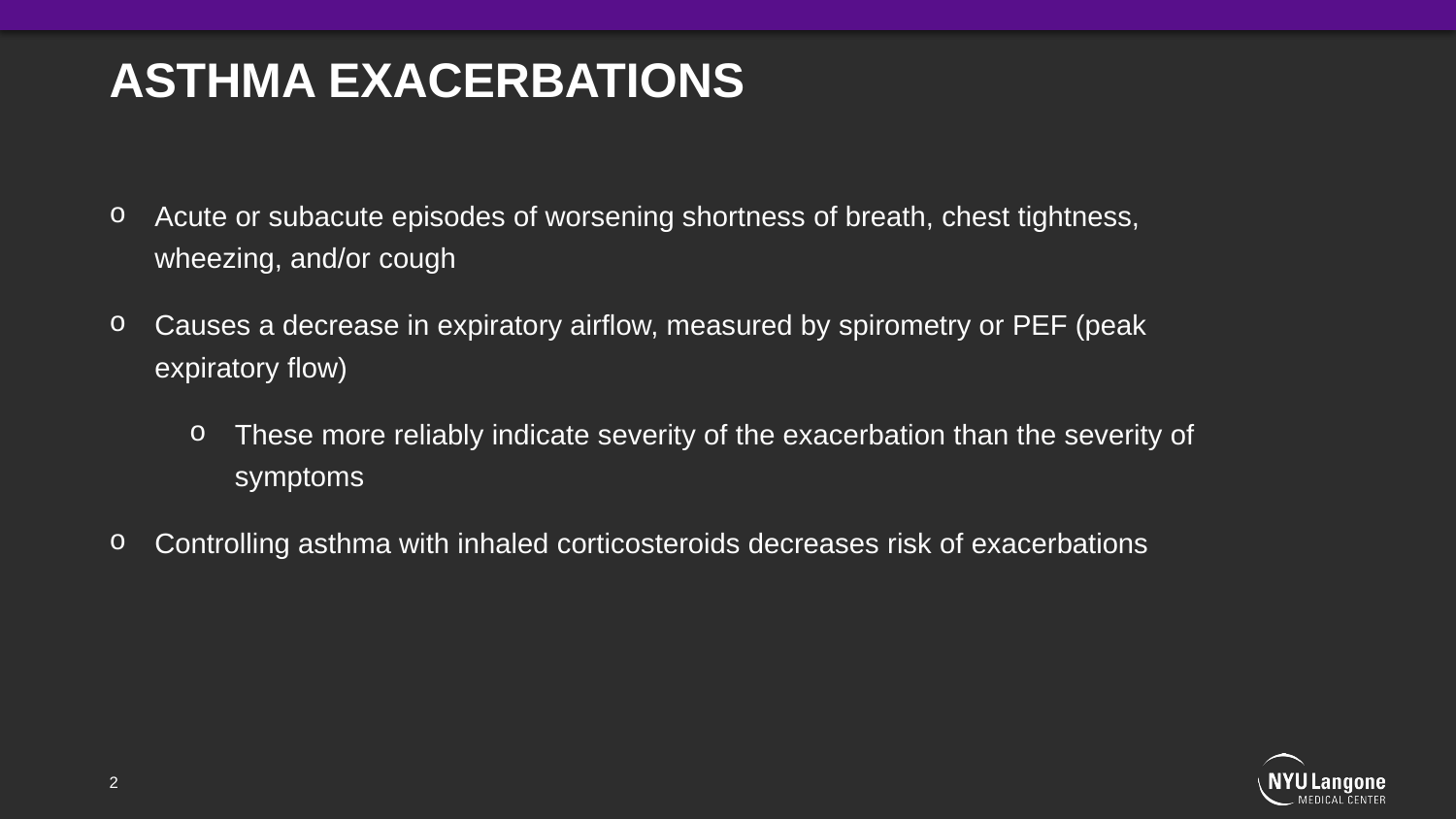

# ASTHMA EXACERBATIONS
Acute or subacute episodes of worsening shortness of breath, chest tightness, wheezing, and/or cough
Causes a decrease in expiratory airflow, measured by spirometry or PEF (peak expiratory flow)
These more reliably indicate severity of the exacerbation than the severity of symptoms
Controlling asthma with inhaled corticosteroids decreases risk of exacerbations
2

## Slide 3
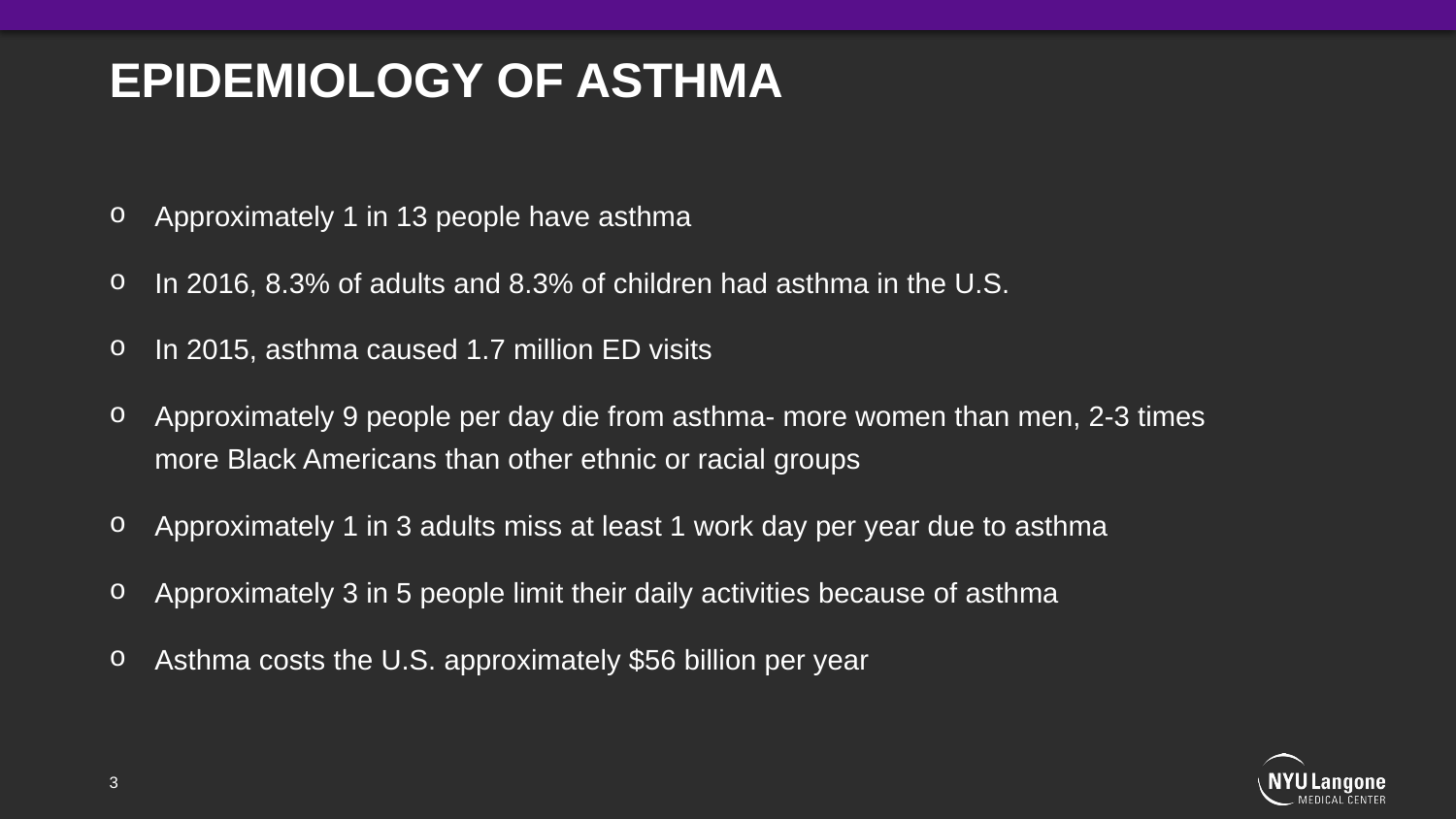

# EPIDEMIOLOGY OF ASTHMA
Approximately 1 in 13 people have asthma
In 2016, 8.3% of adults and 8.3% of children had asthma in the U.S.
In 2015, asthma caused 1.7 million ED visits
Approximately 9 people per day die from asthma- more women than men, 2-3 times more Black Americans than other ethnic or racial groups
Approximately 1 in 3 adults miss at least 1 work day per year due to asthma
Approximately 3 in 5 people limit their daily activities because of asthma
Asthma costs the U.S. approximately $56 billion per year
3

## Slide 4
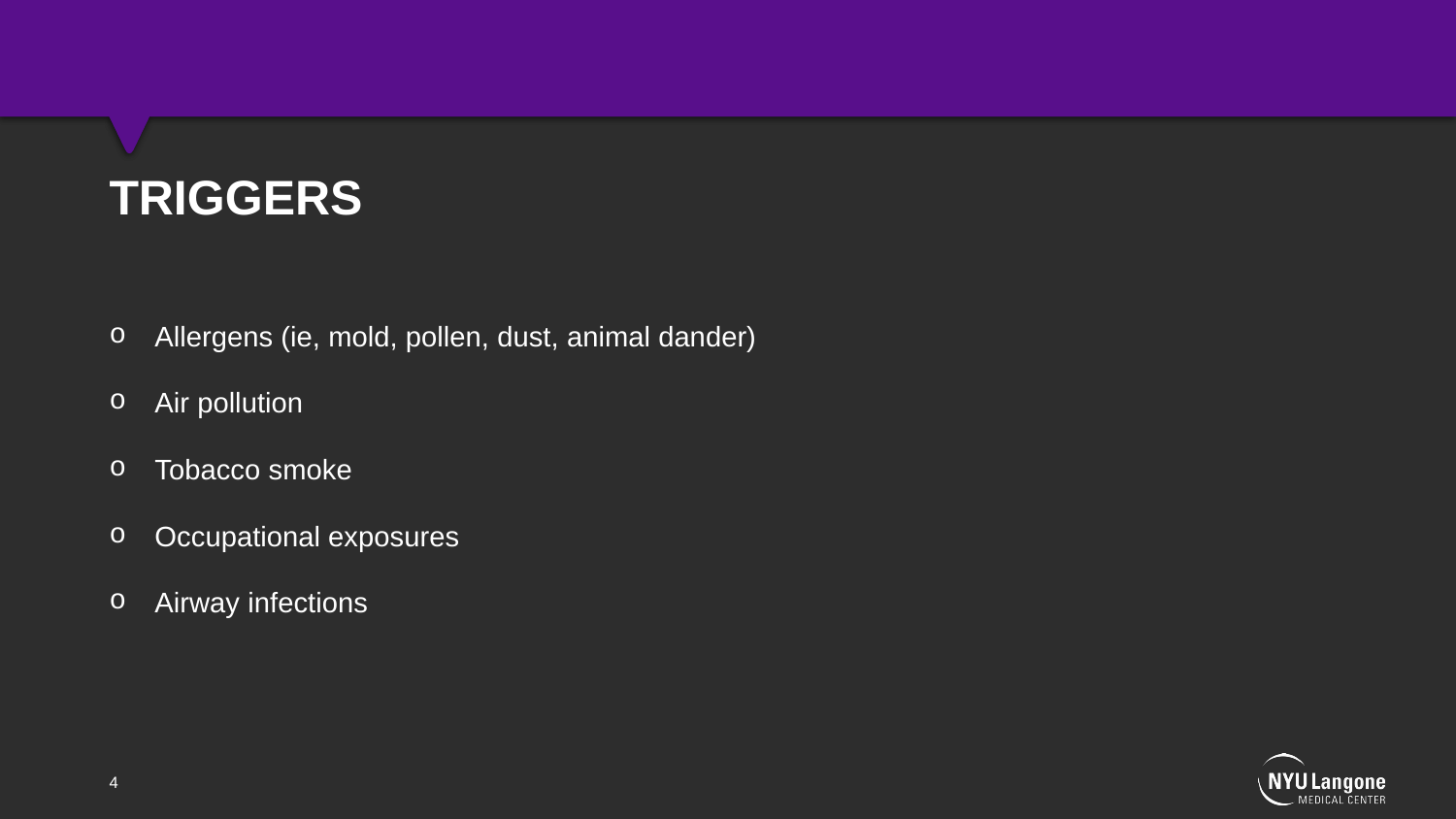

# TRIGGERS
Allergens (ie, mold, pollen, dust, animal dander)
Air pollution
Tobacco smoke
Occupational exposures
Airway infections
4

## Slide 5
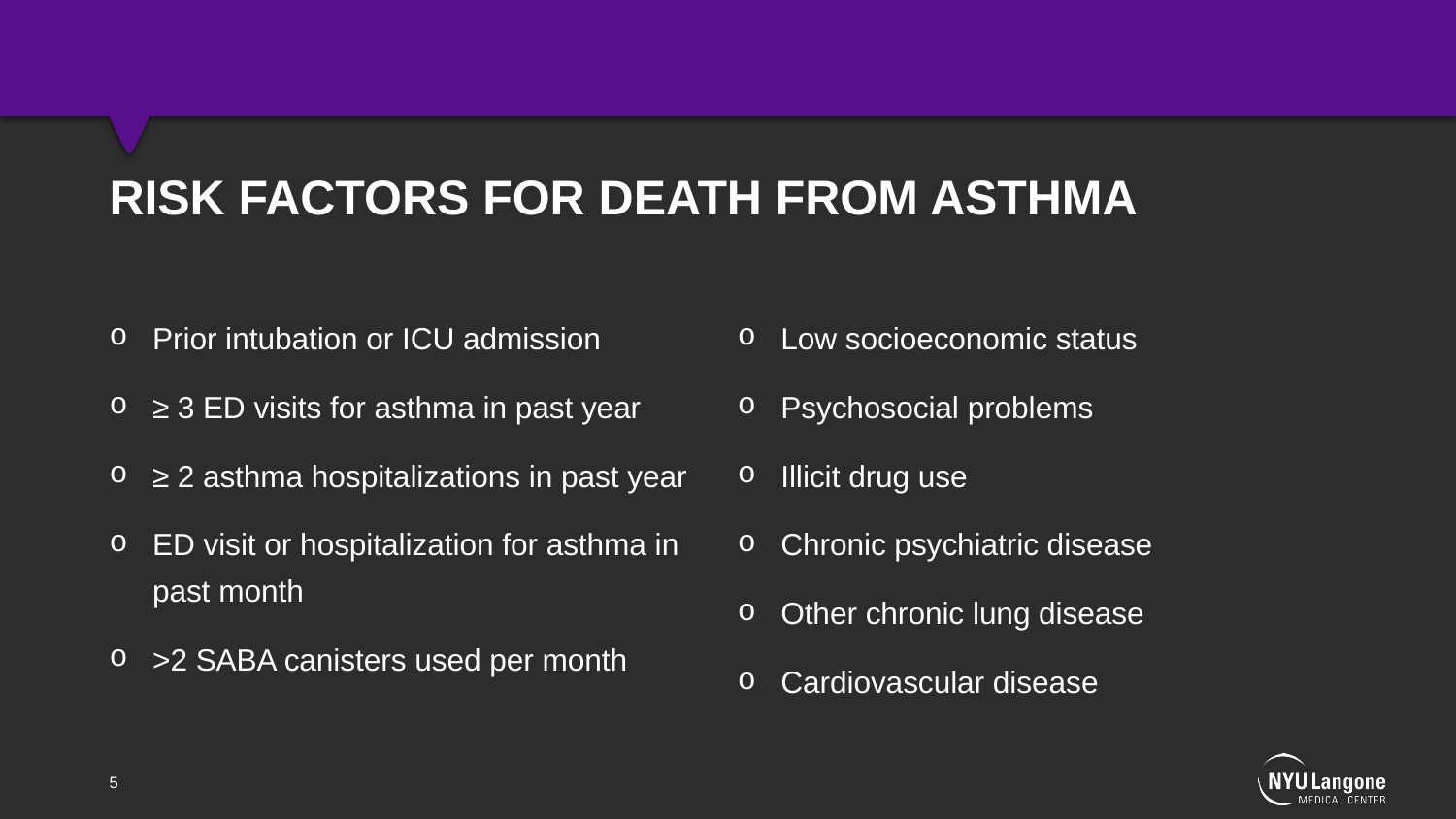

# RISK FACTORS FOR DEATH FROM ASTHMA
Prior intubation or ICU admission
≥ 3 ED visits for asthma in past year
≥ 2 asthma hospitalizations in past year
ED visit or hospitalization for asthma in past month
>2 SABA canisters used per month
Low socioeconomic status
Psychosocial problems
Illicit drug use
Chronic psychiatric disease
Other chronic lung disease
Cardiovascular disease
5

## Slide 6
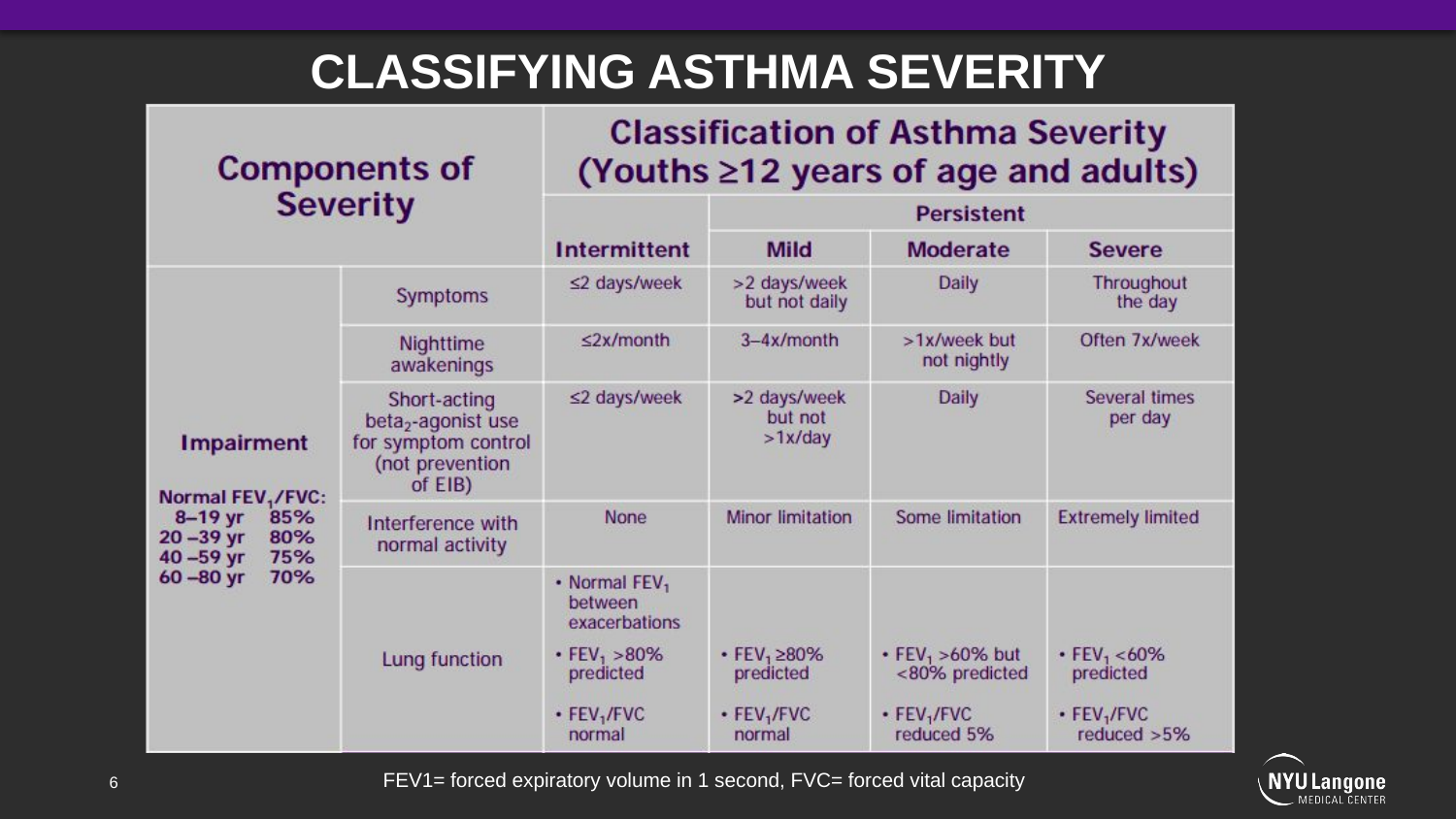

# CLASSIFYING ASTHMA SEVERITY
6
FEV1= forced expiratory volume in 1 second, FVC= forced vital capacity

## Slide 7
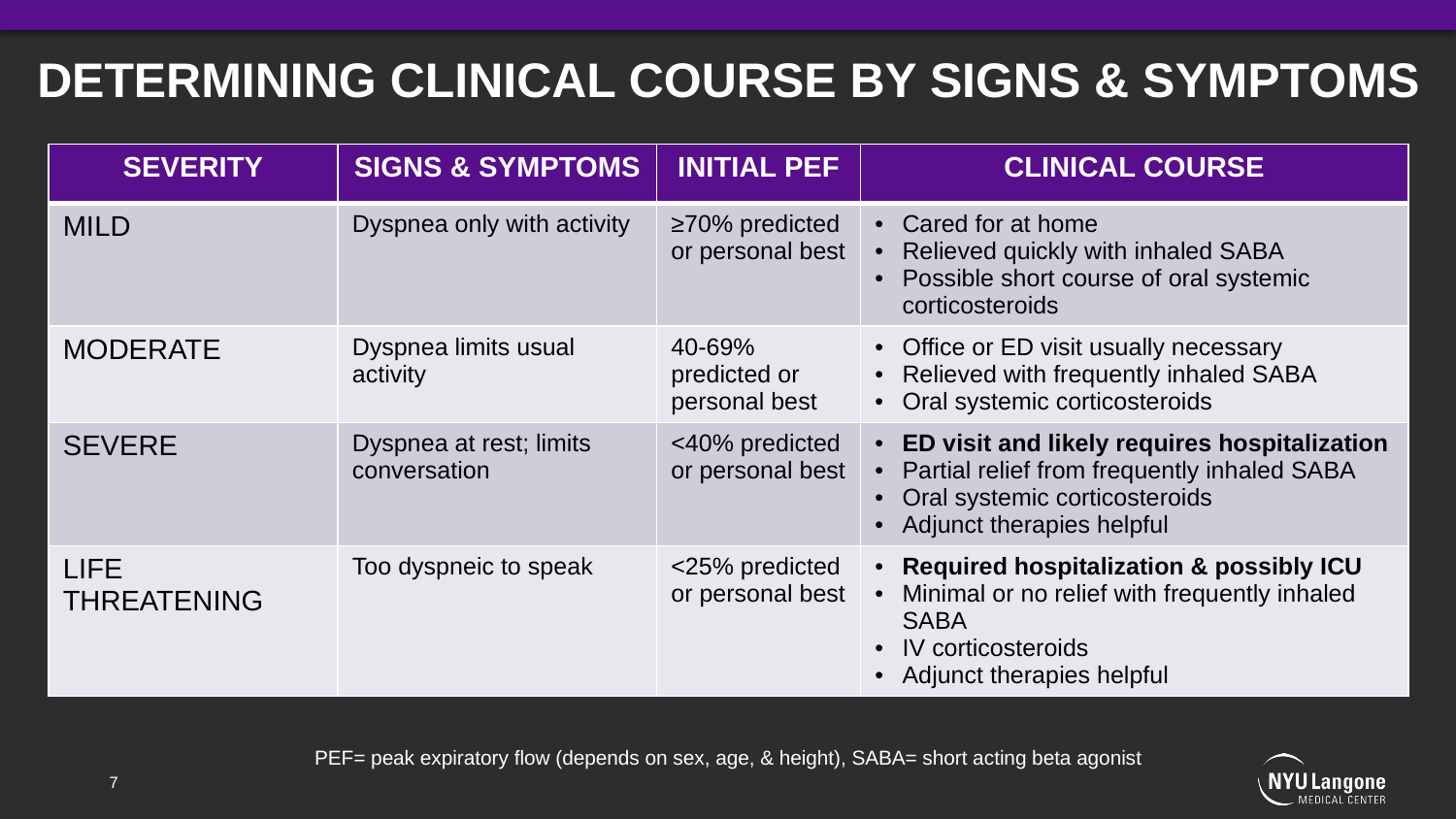

# DETERMINING CLINICAL COURSE BY SIGNS & SYMPTOMS
| SEVERITY | SIGNS & SYMPTOMS | INITIAL PEF | CLINICAL COURSE |
| --- | --- | --- | --- |
| MILD | Dyspnea only with activity | ≥70% predicted or personal best | Cared for at home Relieved quickly with inhaled SABA Possible short course of oral systemic corticosteroids |
| MODERATE | Dyspnea limits usual activity | 40-69% predicted or personal best | Office or ED visit usually necessary Relieved with frequently inhaled SABA Oral systemic corticosteroids |
| SEVERE | Dyspnea at rest; limits conversation | <40% predicted or personal best | ED visit and likely requires hospitalization Partial relief from frequently inhaled SABA Oral systemic corticosteroids Adjunct therapies helpful |
| LIFE THREATENING | Too dyspneic to speak | <25% predicted or personal best | Required hospitalization & possibly ICU Minimal or no relief with frequently inhaled SABA IV corticosteroids Adjunct therapies helpful |
PEF= peak expiratory flow (depends on sex, age, & height), SABA= short acting beta agonist
7

## Slide 8
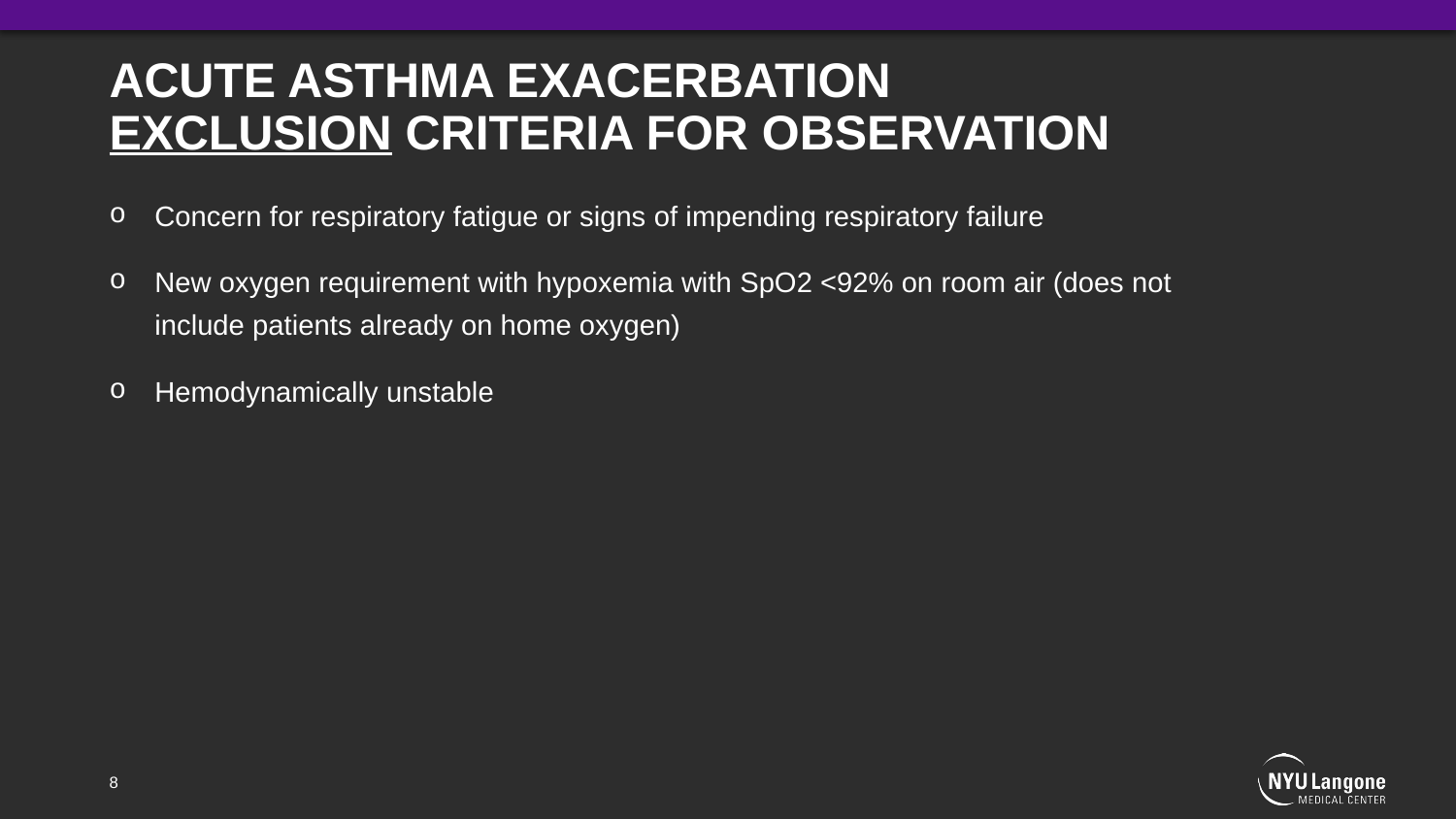

# ACUTE ASTHMA EXACERBATION EXCLUSION CRITERIA FOR OBSERVATION
Concern for respiratory fatigue or signs of impending respiratory failure
New oxygen requirement with hypoxemia with SpO2 <92% on room air (does not include patients already on home oxygen)
Hemodynamically unstable
8

## Slide 9
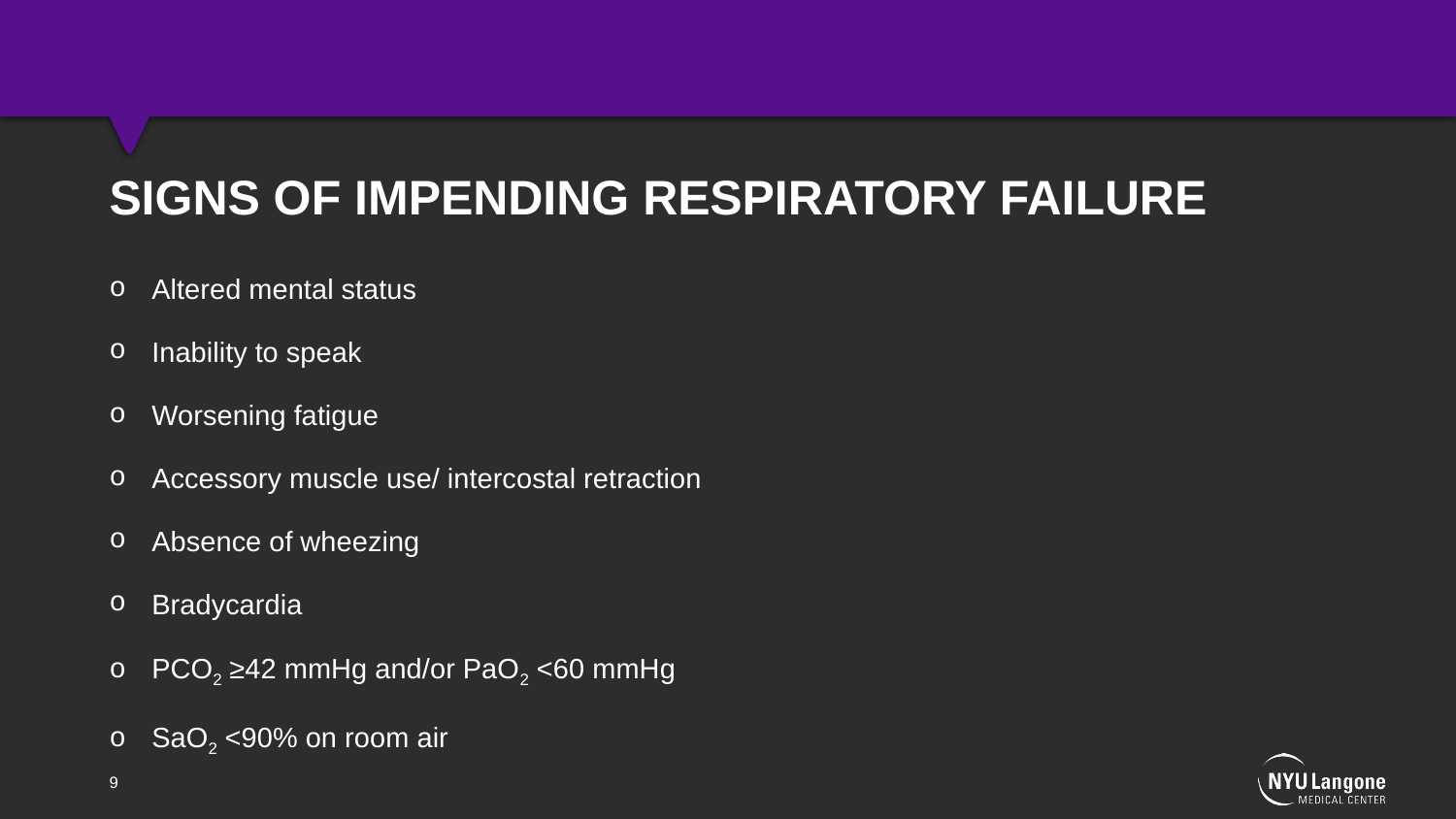

# SIGNS OF IMPENDING RESPIRATORY FAILURE
Altered mental status
Inability to speak
Worsening fatigue
Accessory muscle use/ intercostal retraction
Absence of wheezing
Bradycardia
PCO2 ≥42 mmHg and/or PaO2 <60 mmHg
SaO2 <90% on room air
9

## Slide 10
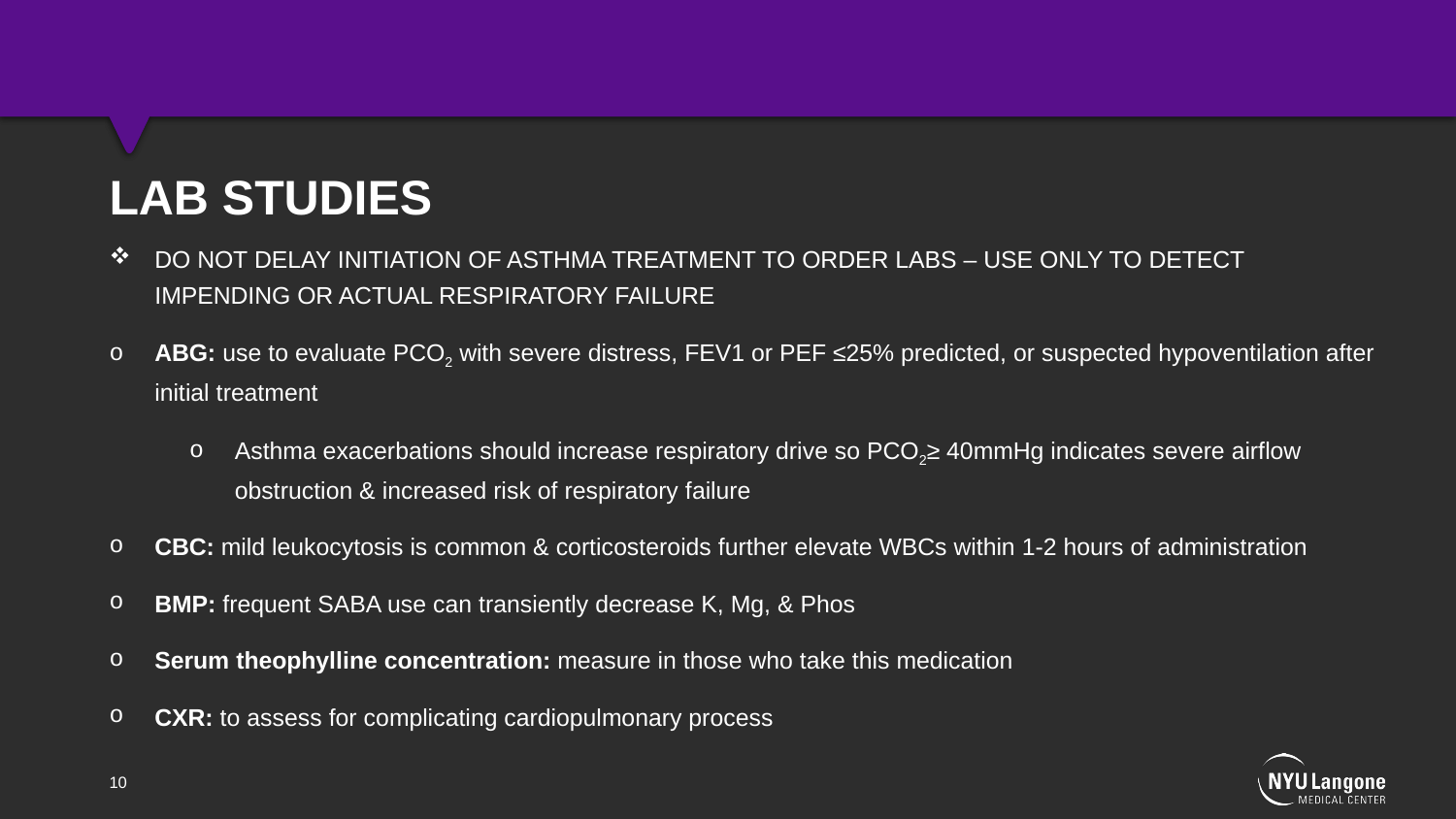

# LAB STUDIES
DO NOT DELAY INITIATION OF ASTHMA TREATMENT TO ORDER LABS – USE ONLY TO DETECT IMPENDING OR ACTUAL RESPIRATORY FAILURE
ABG: use to evaluate PCO2 with severe distress, FEV1 or PEF ≤25% predicted, or suspected hypoventilation after initial treatment
Asthma exacerbations should increase respiratory drive so PCO2≥ 40mmHg indicates severe airflow obstruction & increased risk of respiratory failure
CBC: mild leukocytosis is common & corticosteroids further elevate WBCs within 1-2 hours of administration
BMP: frequent SABA use can transiently decrease K, Mg, & Phos
Serum theophylline concentration: measure in those who take this medication
CXR: to assess for complicating cardiopulmonary process
10

## Slide 11
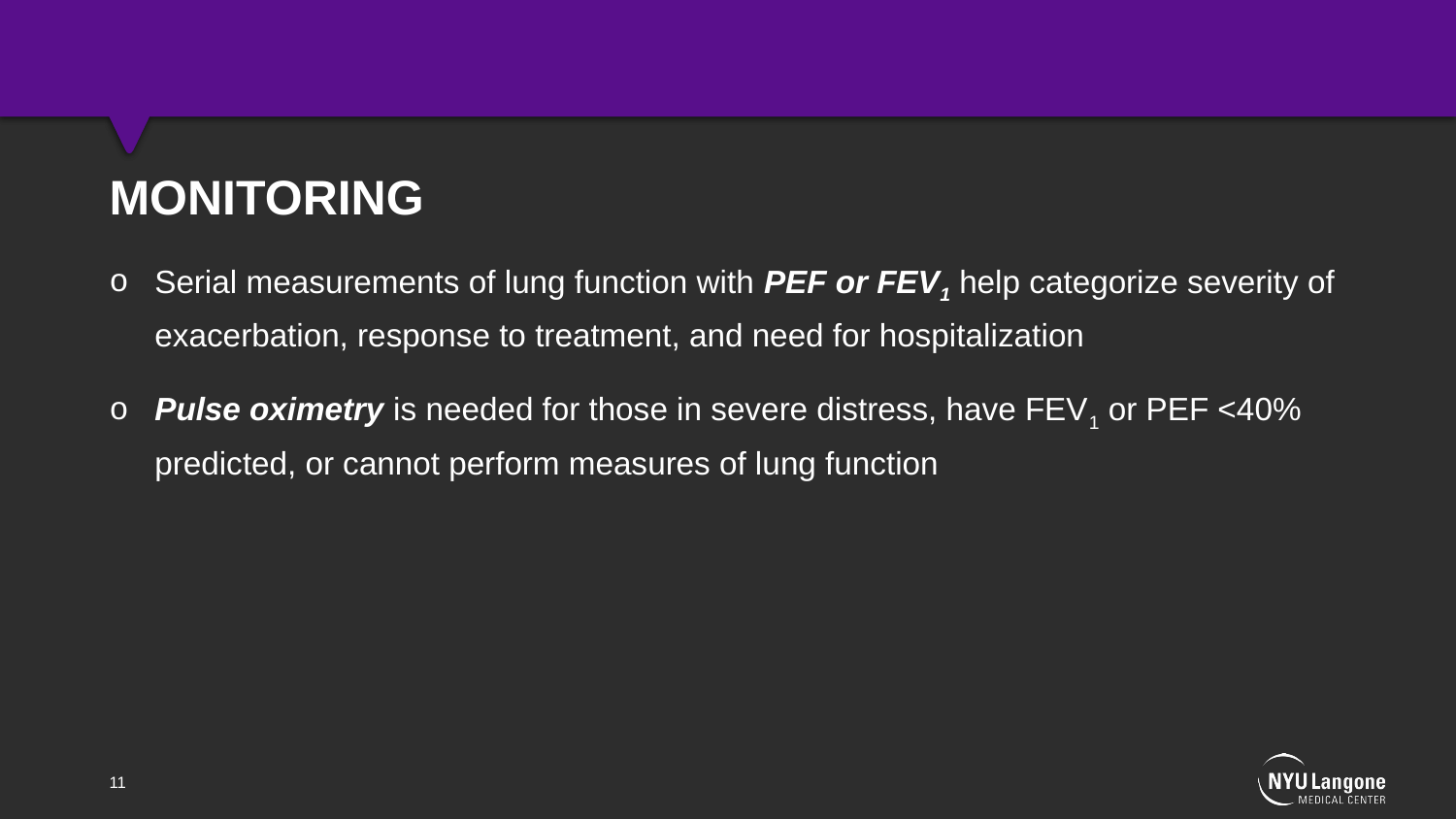

# MONITORING
Serial measurements of lung function with PEF or FEV1 help categorize severity of exacerbation, response to treatment, and need for hospitalization
Pulse oximetry is needed for those in severe distress, have FEV1 or PEF <40% predicted, or cannot perform measures of lung function
11

## Slide 12
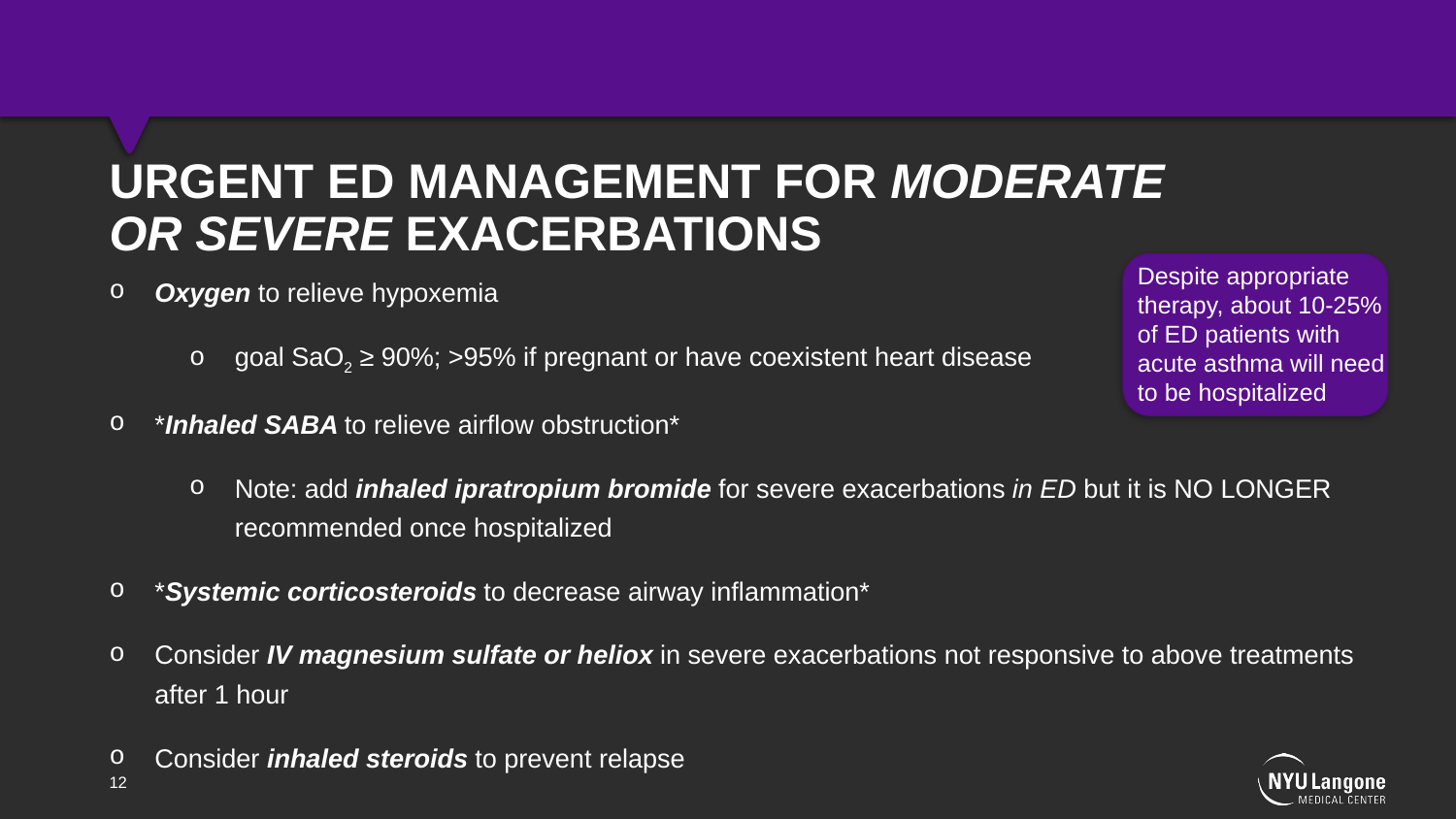

# URGENT ED MANAGEMENT FOR MODERATE OR SEVERE EXACERBATIONS
Despite appropriate therapy, about 10-25% of ED patients with acute asthma will need to be hospitalized
Oxygen to relieve hypoxemia
goal SaO2 ≥ 90%; >95% if pregnant or have coexistent heart disease
*Inhaled SABA to relieve airflow obstruction*
Note: add inhaled ipratropium bromide for severe exacerbations in ED but it is NO LONGER recommended once hospitalized
*Systemic corticosteroids to decrease airway inflammation*
Consider IV magnesium sulfate or heliox in severe exacerbations not responsive to above treatments after 1 hour
Consider inhaled steroids to prevent relapse
12

## Slide 13
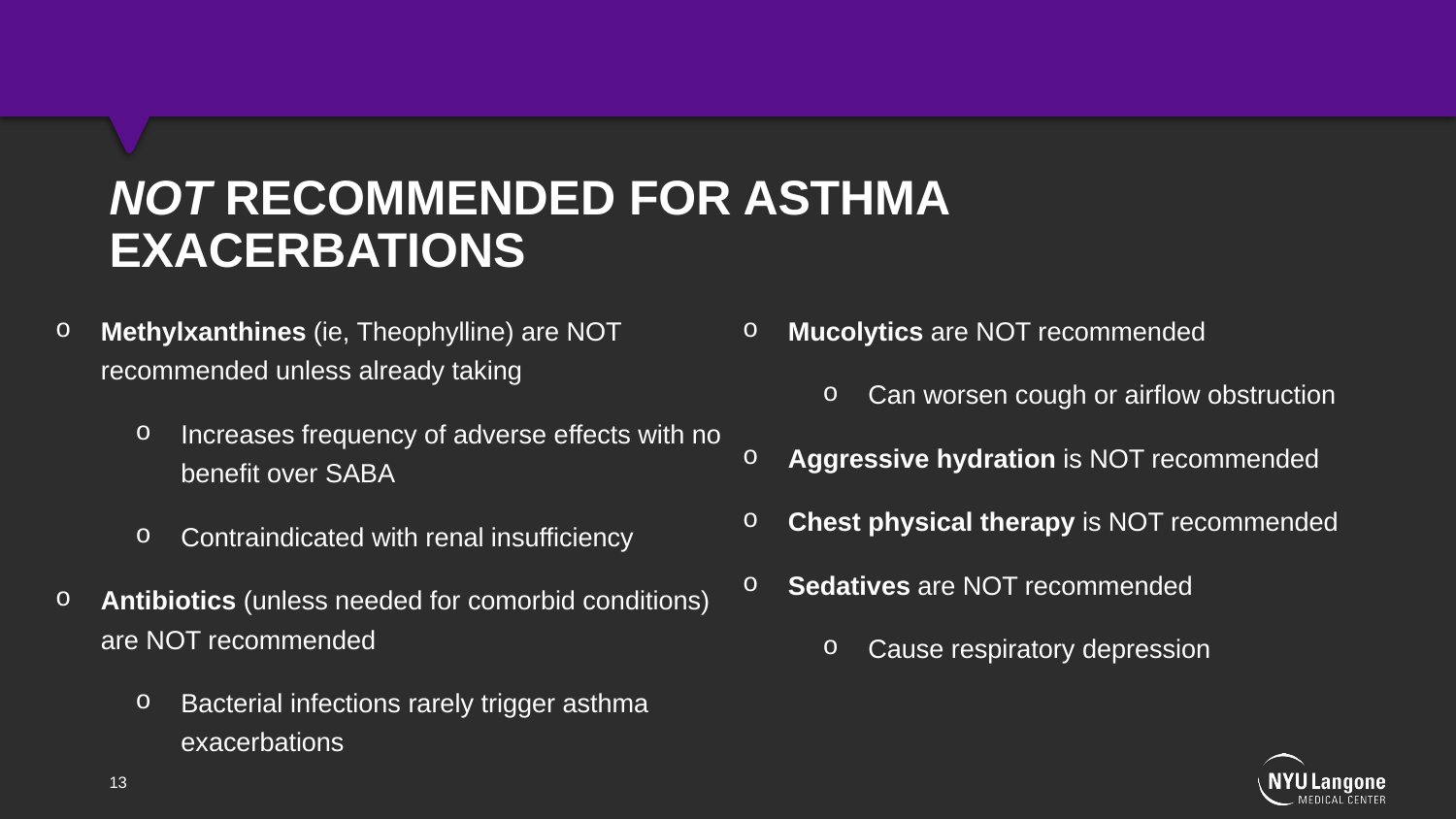

# NOT RECOMMENDED FOR ASTHMA EXACERBATIONS
Methylxanthines (ie, Theophylline) are NOT recommended unless already taking
Increases frequency of adverse effects with no benefit over SABA
Contraindicated with renal insufficiency
Antibiotics (unless needed for comorbid conditions) are NOT recommended
Bacterial infections rarely trigger asthma exacerbations
Mucolytics are NOT recommended
Can worsen cough or airflow obstruction
Aggressive hydration is NOT recommended
Chest physical therapy is NOT recommended
Sedatives are NOT recommended
Cause respiratory depression
13

## Slide 14
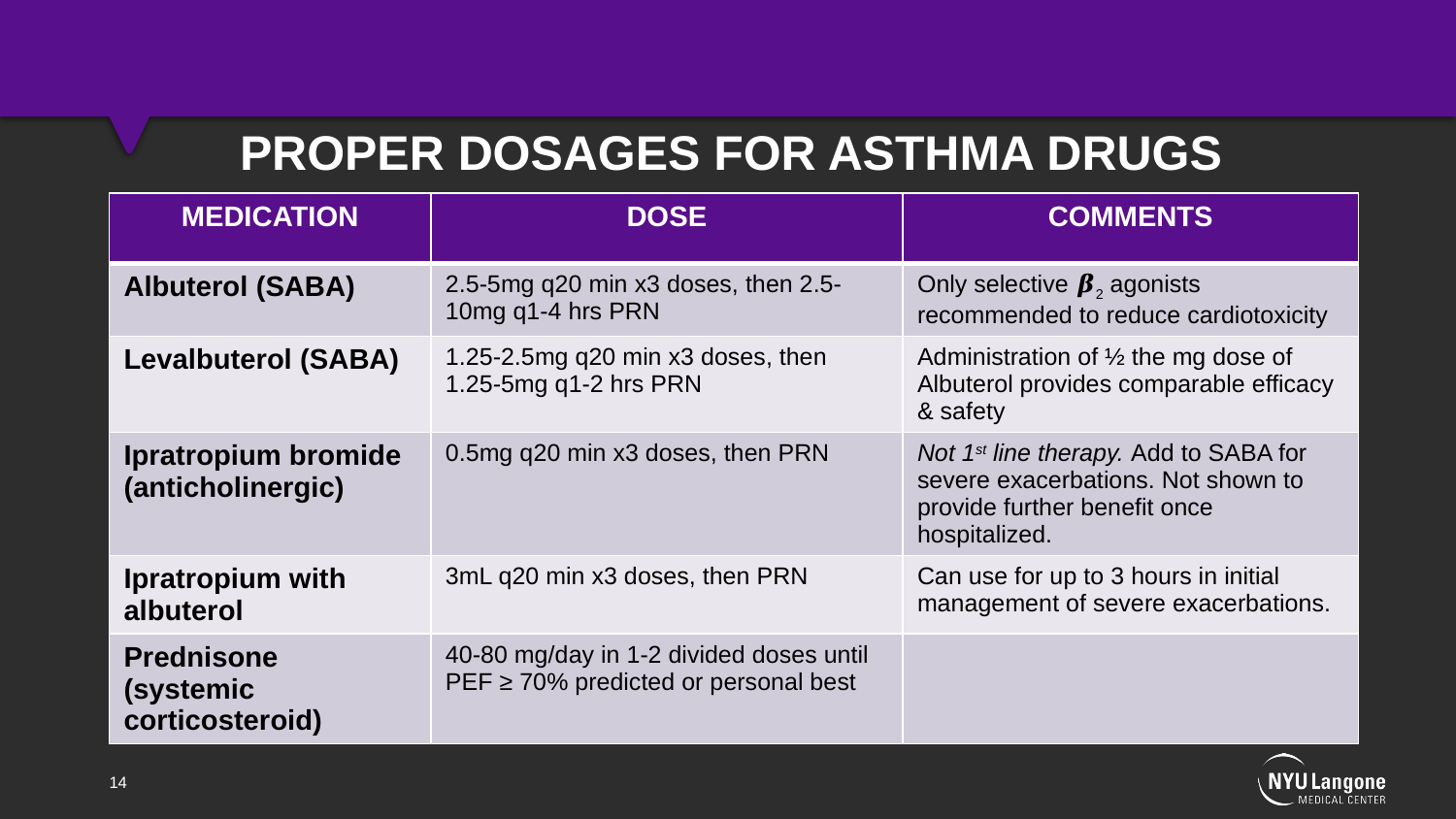

# PROPER DOSAGES FOR ASTHMA DRUGS
| MEDICATION | DOSE | COMMENTS |
| --- | --- | --- |
| Albuterol (SABA) | 2.5-5mg q20 min x3 doses, then 2.5-10mg q1-4 hrs PRN | Only selective 𝜷2 agonists recommended to reduce cardiotoxicity |
| Levalbuterol (SABA) | 1.25-2.5mg q20 min x3 doses, then 1.25-5mg q1-2 hrs PRN | Administration of ½ the mg dose of Albuterol provides comparable efficacy & safety |
| Ipratropium bromide (anticholinergic) | 0.5mg q20 min x3 doses, then PRN | Not 1st line therapy. Add to SABA for severe exacerbations. Not shown to provide further benefit once hospitalized. |
| Ipratropium with albuterol | 3mL q20 min x3 doses, then PRN | Can use for up to 3 hours in initial management of severe exacerbations. |
| Prednisone (systemic corticosteroid) | 40-80 mg/day in 1-2 divided doses until PEF ≥ 70% predicted or personal best | |
14

## Slide 15
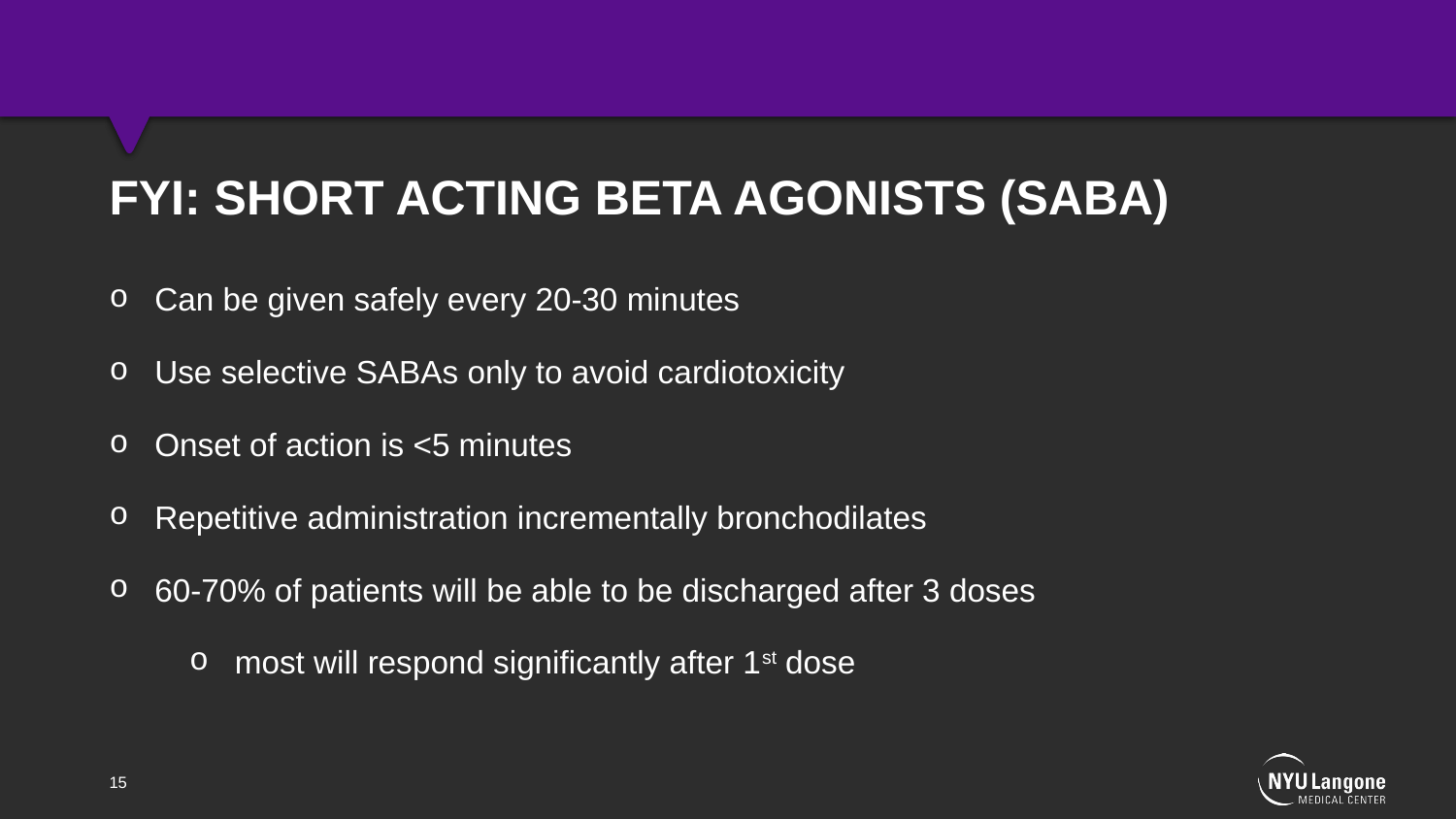

# FYI: SHORT ACTING BETA AGONISTS (SABA)
Can be given safely every 20-30 minutes
Use selective SABAs only to avoid cardiotoxicity
Onset of action is <5 minutes
Repetitive administration incrementally bronchodilates
60-70% of patients will be able to be discharged after 3 doses
most will respond significantly after 1st dose
15

## Slide 16
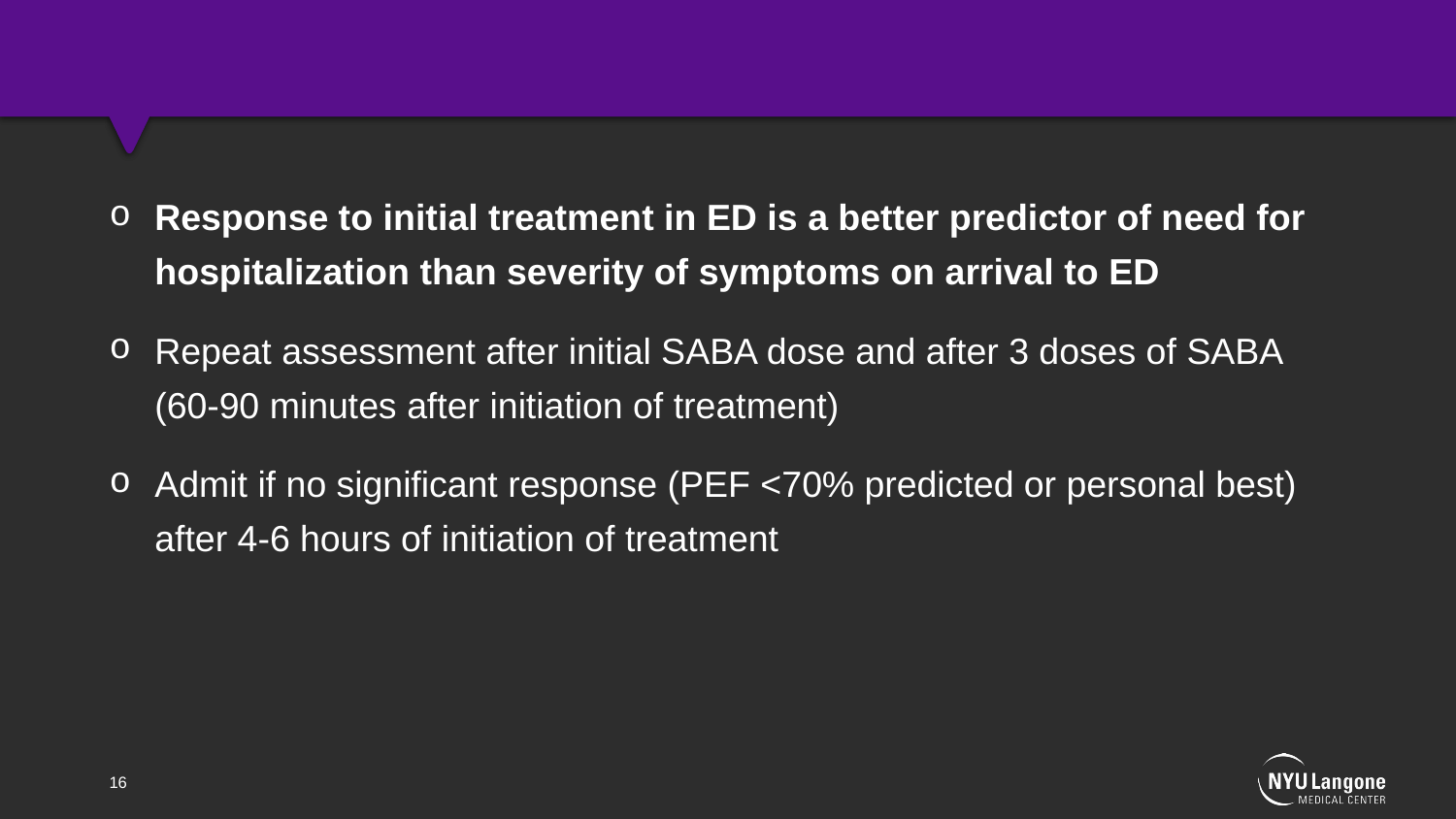

Response to initial treatment in ED is a better predictor of need for hospitalization than severity of symptoms on arrival to ED
Repeat assessment after initial SABA dose and after 3 doses of SABA (60-90 minutes after initiation of treatment)
Admit if no significant response (PEF <70% predicted or personal best) after 4-6 hours of initiation of treatment
16

## Slide 17
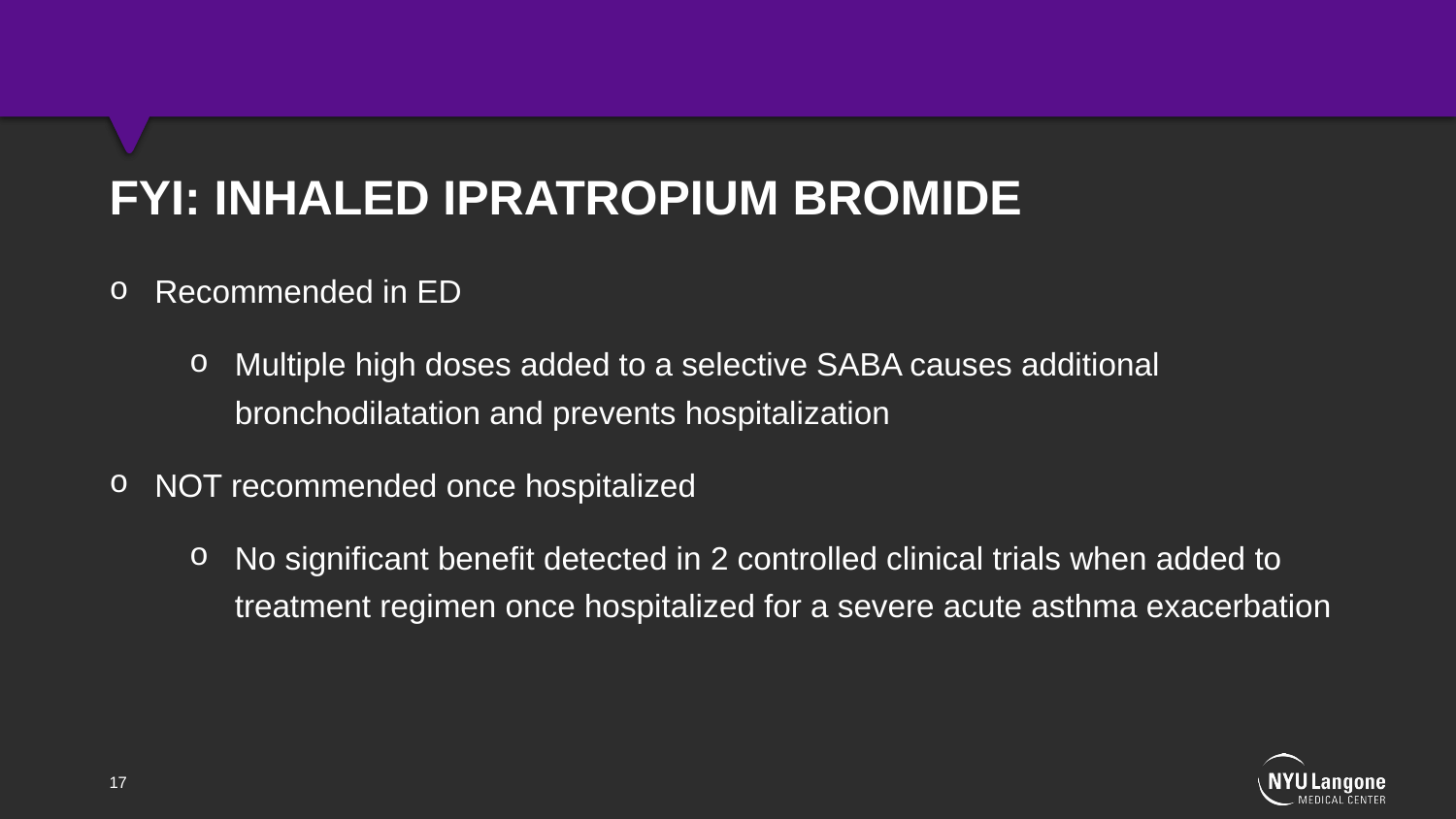

# FYI: INHALED IPRATROPIUM BROMIDE
Recommended in ED
Multiple high doses added to a selective SABA causes additional bronchodilatation and prevents hospitalization
NOT recommended once hospitalized
No significant benefit detected in 2 controlled clinical trials when added to treatment regimen once hospitalized for a severe acute asthma exacerbation
17

## Slide 18
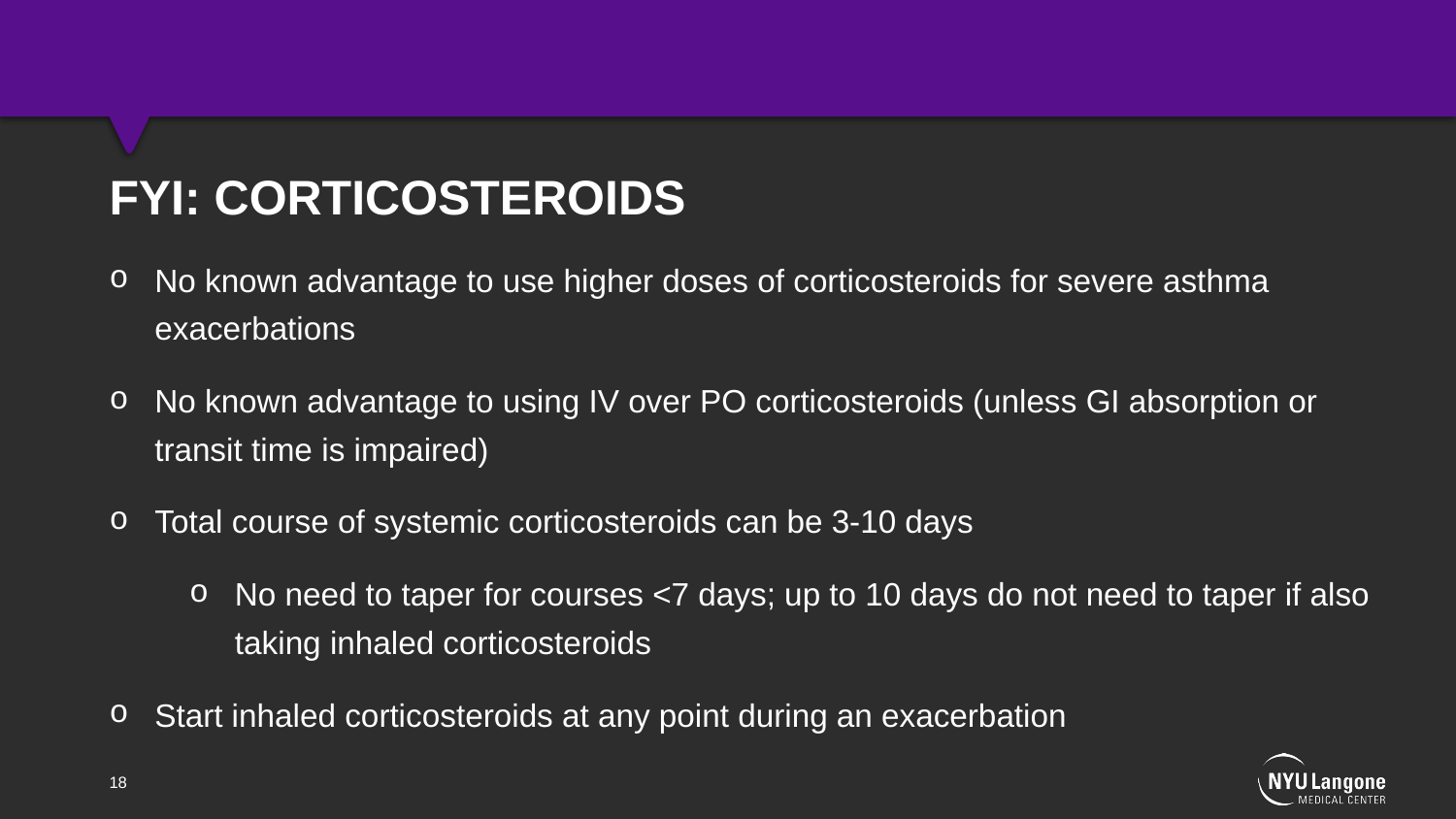

# FYI: CORTICOSTEROIDS
No known advantage to use higher doses of corticosteroids for severe asthma exacerbations
No known advantage to using IV over PO corticosteroids (unless GI absorption or transit time is impaired)
Total course of systemic corticosteroids can be 3-10 days
No need to taper for courses <7 days; up to 10 days do not need to taper if also taking inhaled corticosteroids
Start inhaled corticosteroids at any point during an exacerbation
18

## Slide 19
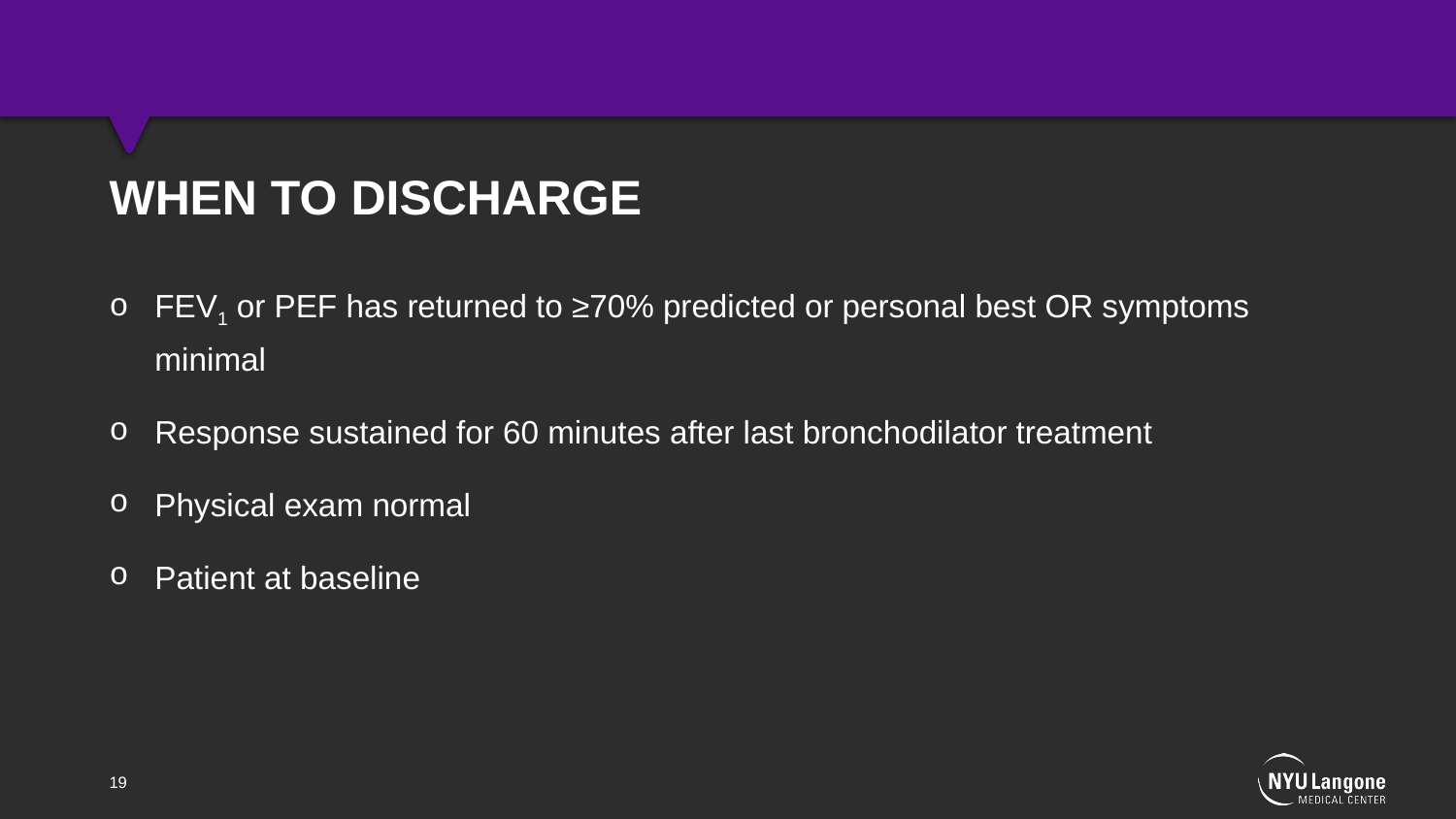

# WHEN TO DISCHARGE
FEV1 or PEF has returned to ≥70% predicted or personal best OR symptoms minimal
Response sustained for 60 minutes after last bronchodilator treatment
Physical exam normal
Patient at baseline
19

## Slide 20
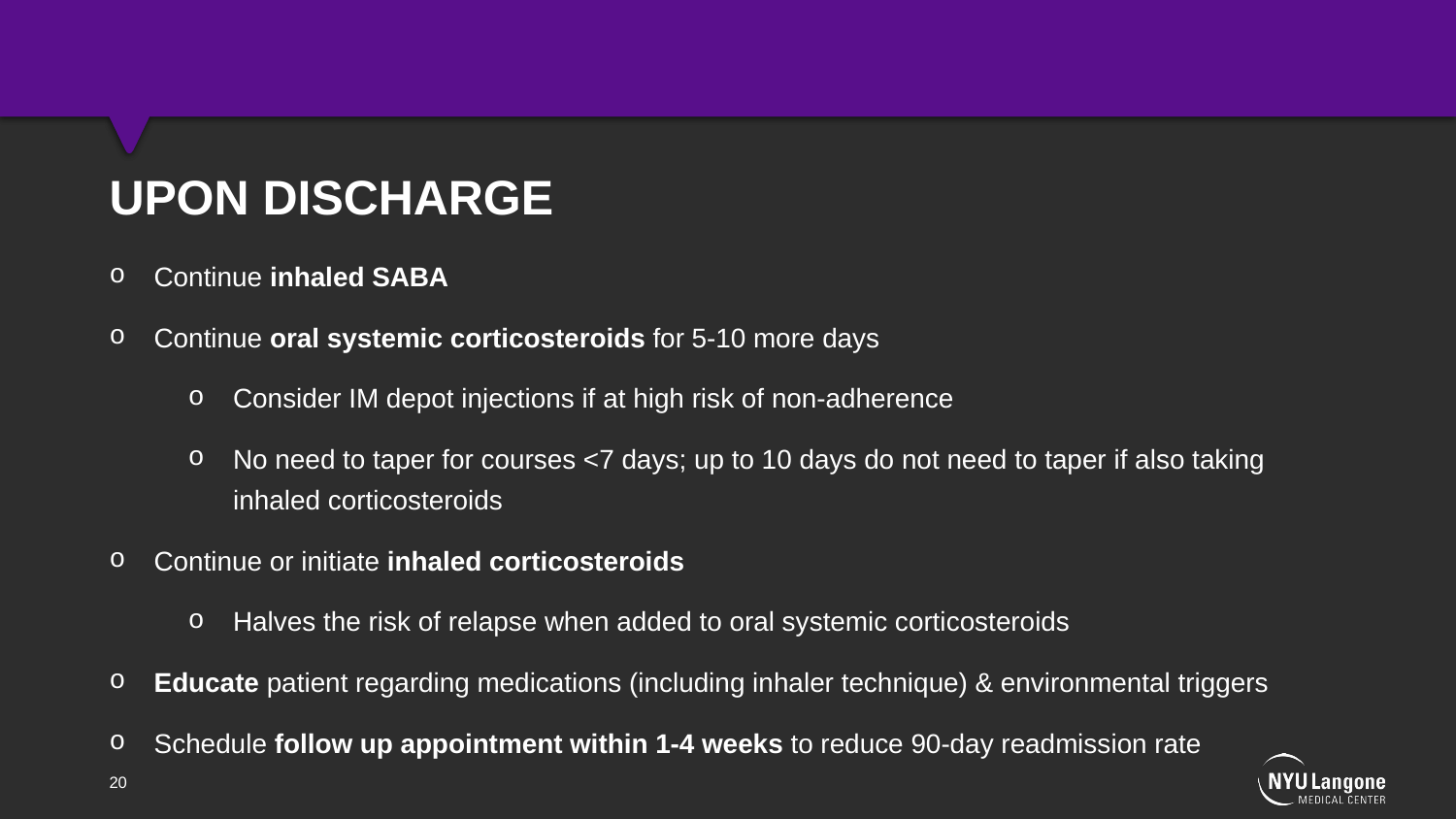

# UPON DISCHARGE
Continue inhaled SABA
Continue oral systemic corticosteroids for 5-10 more days
Consider IM depot injections if at high risk of non-adherence
No need to taper for courses <7 days; up to 10 days do not need to taper if also taking inhaled corticosteroids
Continue or initiate inhaled corticosteroids
Halves the risk of relapse when added to oral systemic corticosteroids
Educate patient regarding medications (including inhaler technique) & environmental triggers
Schedule follow up appointment within 1-4 weeks to reduce 90-day readmission rate
20

## Slide 21
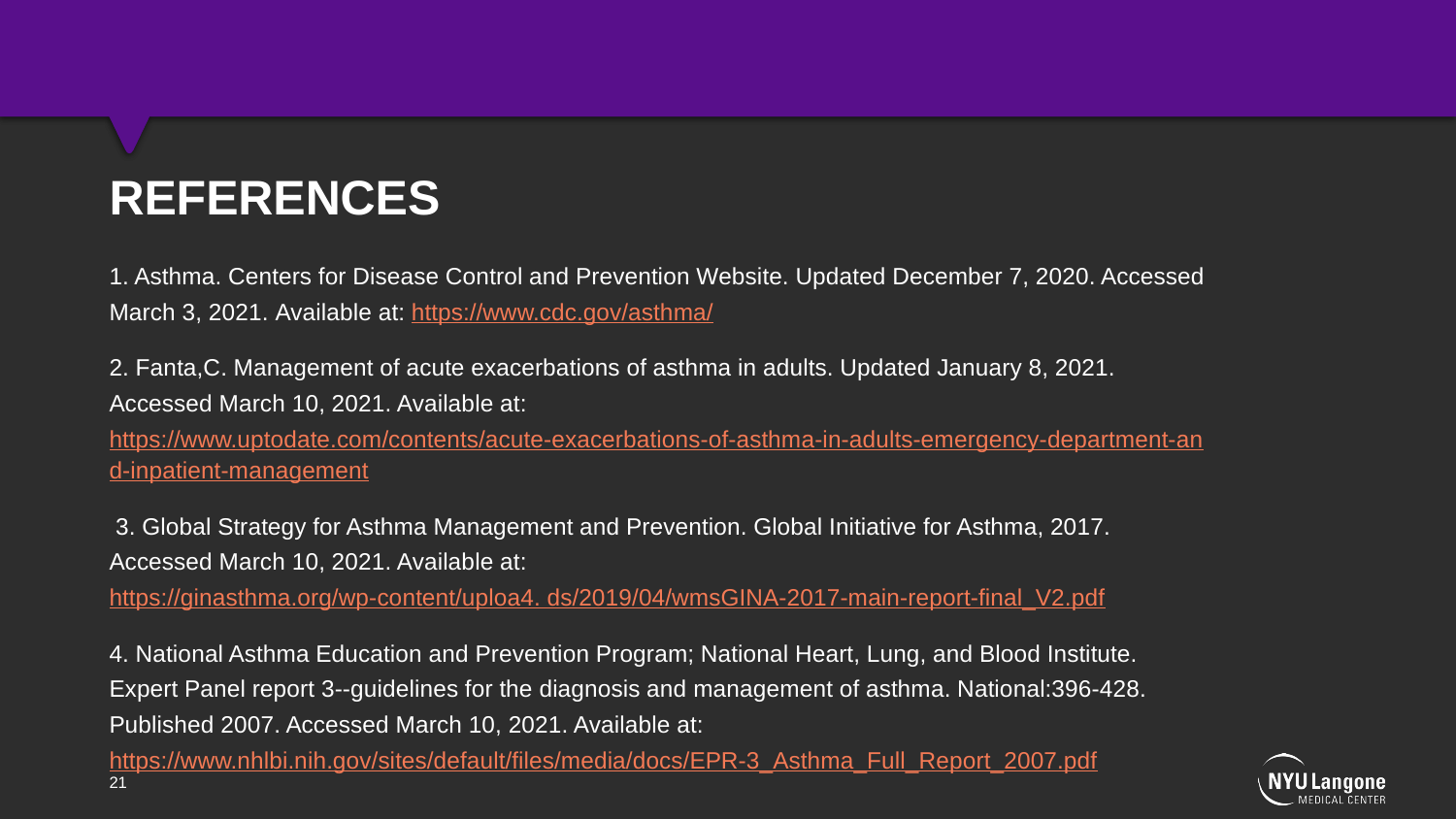

# REFERENCES
1. Asthma. Centers for Disease Control and Prevention Website. Updated December 7, 2020. Accessed March 3, 2021. Available at: https://www.cdc.gov/asthma/
2. Fanta,C. Management of acute exacerbations of asthma in adults. Updated January 8, 2021. Accessed March 10, 2021. Available at: https://www.uptodate.com/contents/acute-exacerbations-of-asthma-in-adults-emergency-department-and-inpatient-management
 3. Global Strategy for Asthma Management and Prevention. Global Initiative for Asthma, 2017. Accessed March 10, 2021. Available at: https://ginasthma.org/wp-content/uploa4. ds/2019/04/wmsGINA-2017-main-report-final_V2.pdf
4. National Asthma Education and Prevention Program; National Heart, Lung, and Blood Institute. Expert Panel report 3--guidelines for the diagnosis and management of asthma. National:396-428. Published 2007. Accessed March 10, 2021. Available at: https://www.nhlbi.nih.gov/sites/default/files/media/docs/EPR-3_Asthma_Full_Report_2007.pdf
21
