# Supplementary material for: An Observation Medicine Curriculum for Emergency Medicine Education
Source: J Educ Teach Emerg Med. 2021 Apr 19;6(2):C1–C72. doi: 10.21980/J87P92 (PMC10332786; doi:10.21980/J87P92)
Supplement: Supplementary file 8 — Please see associated PowerPoint file [file jetem-6-2-c1-supp8.pptx]

## Slide 1
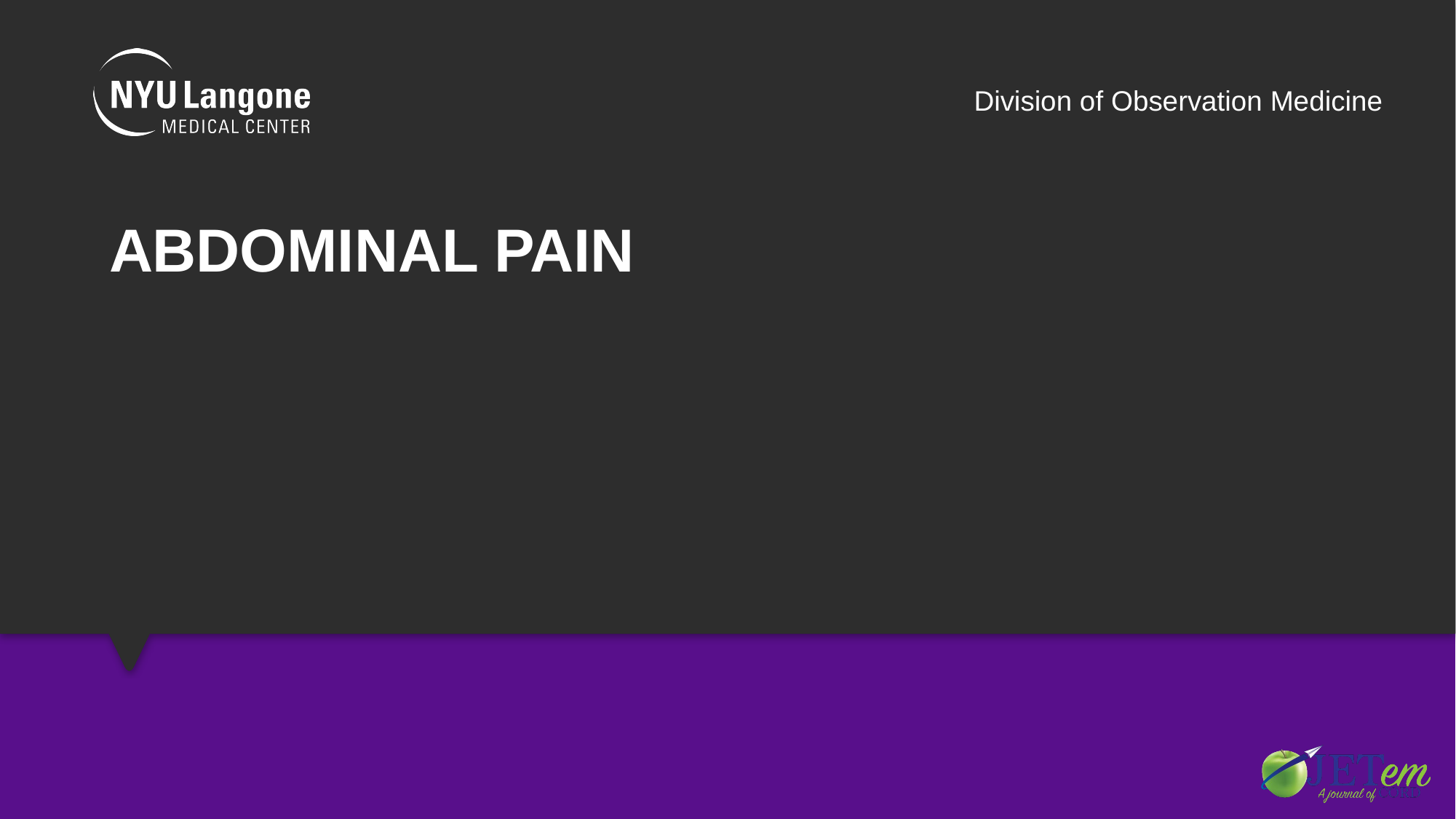

Division of Observation Medicine
# Abdominal Pain ​

## Slide 2
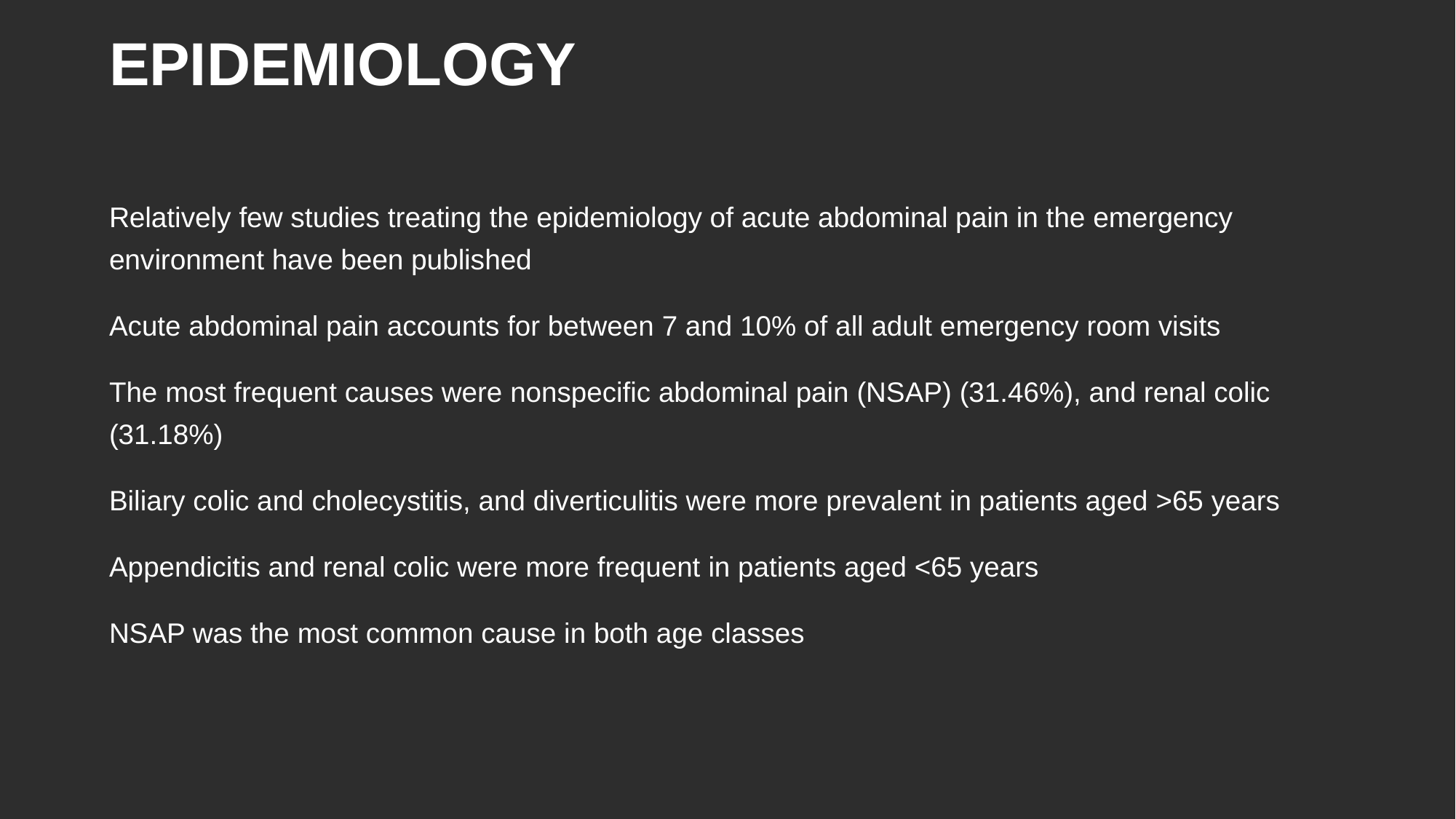

# Epidemiology
Relatively few studies treating the epidemiology of acute abdominal pain in the emergency environment have been published
Acute abdominal pain accounts for between 7 and 10% of all adult emergency room visits
The most frequent causes were nonspecific abdominal pain (NSAP) (31.46%), and renal colic (31.18%)
Biliary colic and cholecystitis, and diverticulitis were more prevalent in patients aged >65 years
Appendicitis and renal colic were more frequent in patients aged <65 years
NSAP was the most common cause in both age classes

## Slide 3
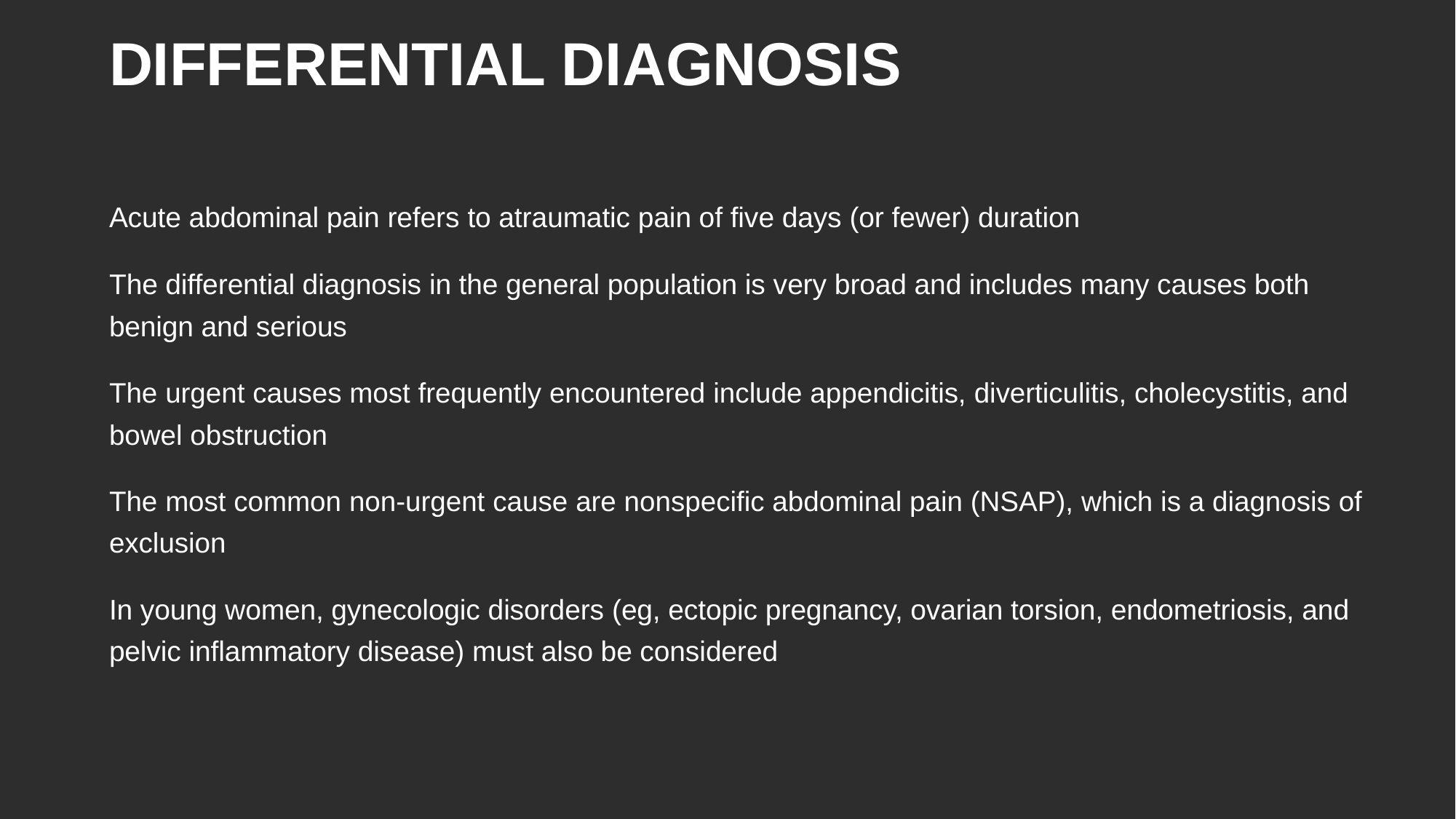

# Differential Diagnosis
Acute abdominal pain refers to atraumatic pain of five days (or fewer) duration
The differential diagnosis in the general population is very broad and includes many causes both benign and serious
The urgent causes most frequently encountered include appendicitis, diverticulitis, cholecystitis, and bowel obstruction
The most common non-urgent cause are nonspecific abdominal pain (NSAP), which is a diagnosis of exclusion
In young women, gynecologic disorders (eg, ectopic pregnancy, ovarian torsion, endometriosis, and pelvic inflammatory disease) must also be considered

## Slide 4
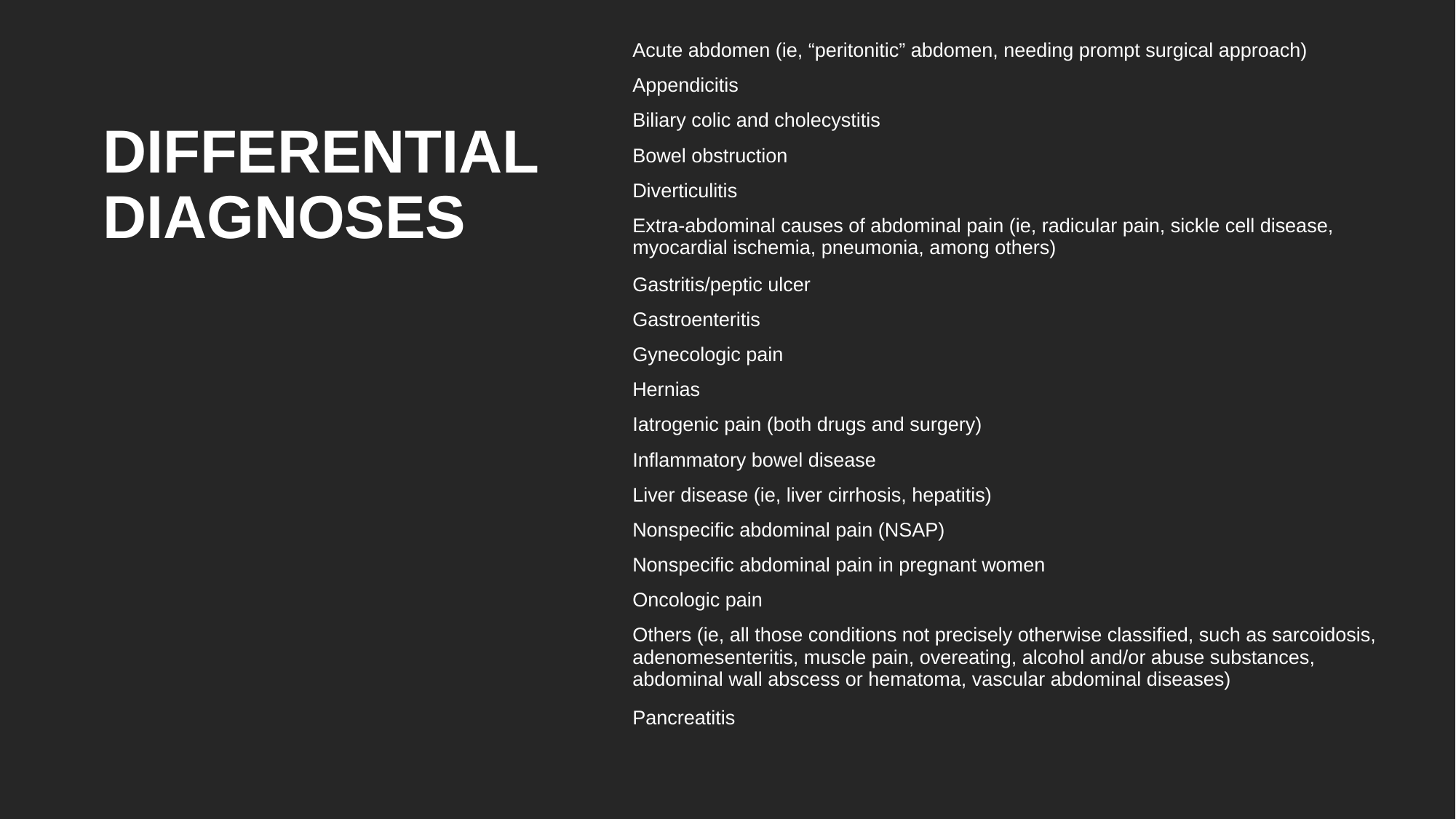

| Acute abdomen (ie, “peritonitic” abdomen, needing prompt surgical approach) |
| --- |
| Appendicitis |
| Biliary colic and cholecystitis |
| Bowel obstruction |
| Diverticulitis |
| Extra-abdominal causes of abdominal pain (ie, radicular pain, sickle cell disease, myocardial ischemia, pneumonia, among others) |
| Gastritis/peptic ulcer |
| Gastroenteritis |
| Gynecologic pain |
| Hernias |
| Iatrogenic pain (both drugs and surgery) |
| Inflammatory bowel disease |
| Liver disease (ie, liver cirrhosis, hepatitis) |
| Nonspecific abdominal pain (NSAP) |
| Nonspecific abdominal pain in pregnant women |
| Oncologic pain |
| Others (ie, all those conditions not precisely otherwise classified, such as sarcoidosis, adenomesenteritis, muscle pain, overeating, alcohol and/or abuse substances, abdominal wall abscess or hematoma, vascular abdominal diseases) |
| Pancreatitis |
# Differential Diagnoses

## Slide 5
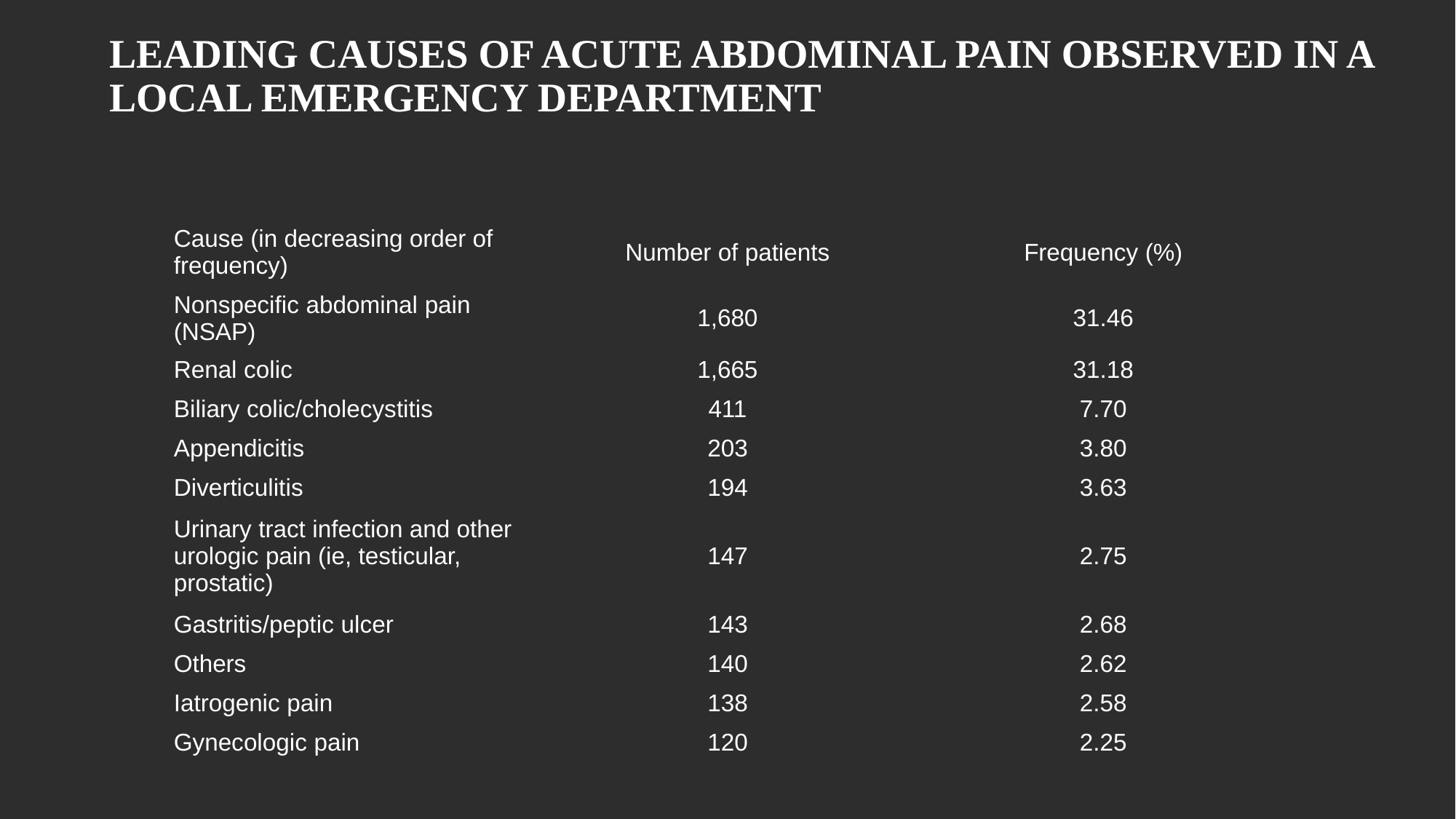

# Leading causes of acute abdominal pain observed in a local emergency department
| Cause (in decreasing order of frequency) | Number of patients | Frequency (%) |
| --- | --- | --- |
| Nonspecific abdominal pain (NSAP) | 1,680 | 31.46 |
| Renal colic | 1,665 | 31.18 |
| Biliary colic/cholecystitis | 411 | 7.70 |
| Appendicitis | 203 | 3.80 |
| Diverticulitis | 194 | 3.63 |
| Urinary tract infection and other urologic pain (ie, testicular, prostatic) | 147 | 2.75 |
| Gastritis/peptic ulcer | 143 | 2.68 |
| Others | 140 | 2.62 |
| Iatrogenic pain | 138 | 2.58 |
| Gynecologic pain | 120 | 2.25 |

## Slide 6
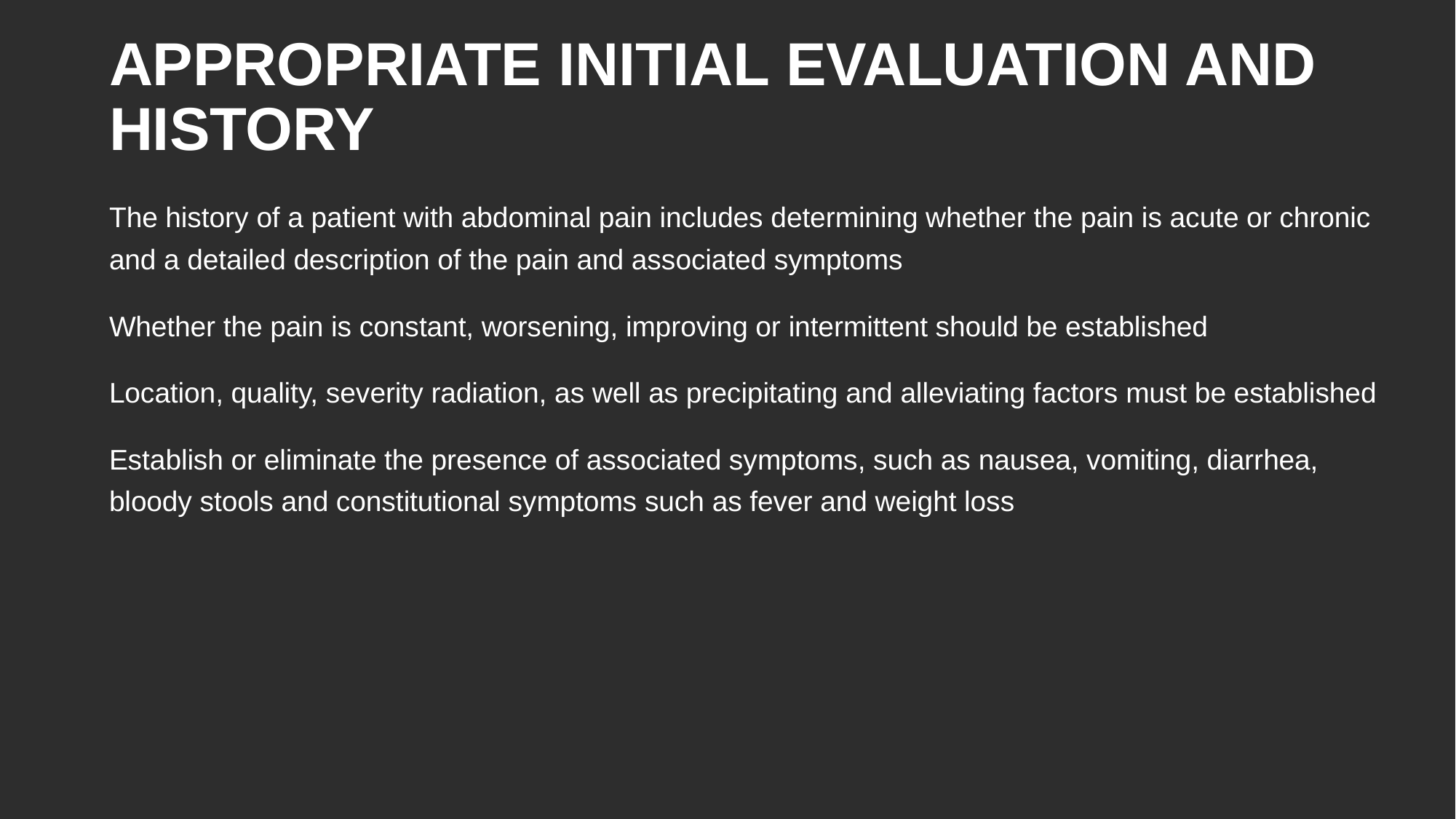

# Appropriate Initial Evaluation and History
The history of a patient with abdominal pain includes determining whether the pain is acute or chronic and a detailed description of the pain and associated symptoms
Whether the pain is constant, worsening, improving or intermittent should be established
Location, quality, severity radiation, as well as precipitating and alleviating factors must be established
Establish or eliminate the presence of associated symptoms, such as nausea, vomiting, diarrhea, bloody stools and constitutional symptoms such as fever and weight loss

## Slide 7
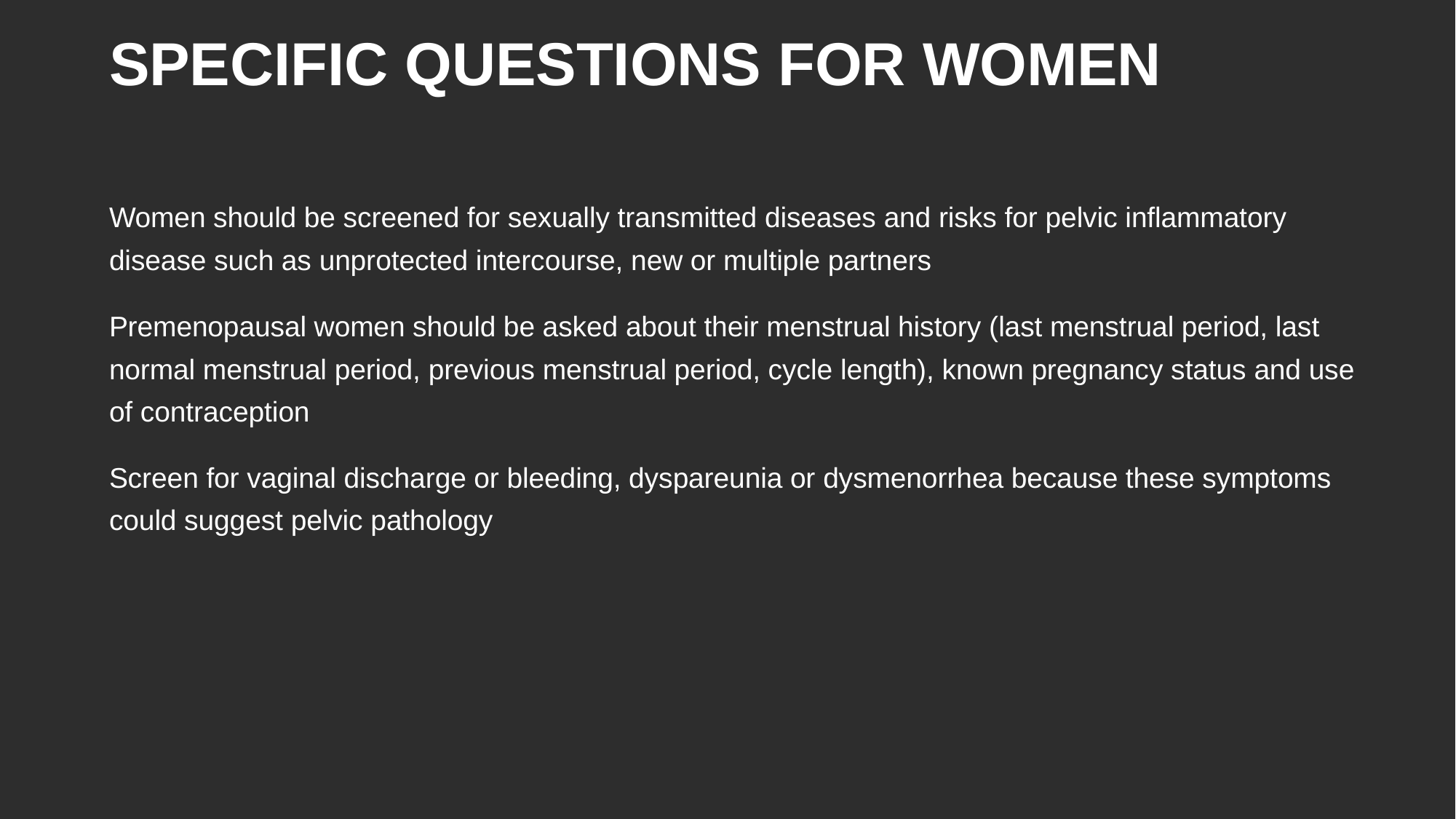

# Specific Questions for Women
Women should be screened for sexually transmitted diseases and risks for pelvic inflammatory disease such as unprotected intercourse, new or multiple partners
Premenopausal women should be asked about their menstrual history (last menstrual period, last normal menstrual period, previous menstrual period, cycle length), known pregnancy status and use of contraception
Screen for vaginal discharge or bleeding, dyspareunia or dysmenorrhea because these symptoms could suggest pelvic pathology

## Slide 8
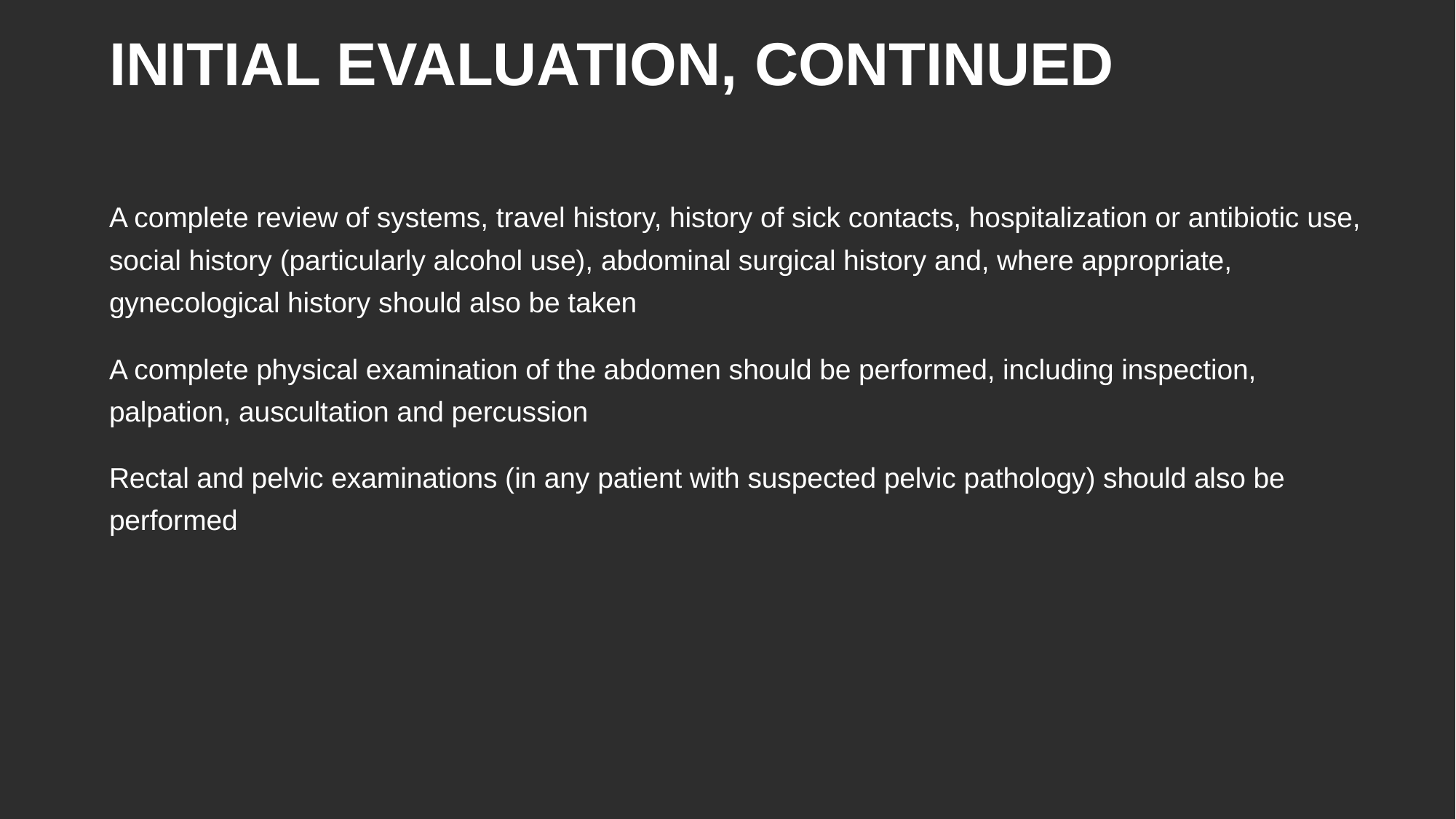

# Initial Evaluation, Continued
A complete review of systems, travel history, history of sick contacts, hospitalization or antibiotic use, social history (particularly alcohol use), abdominal surgical history and, where appropriate, gynecological history should also be taken
A complete physical examination of the abdomen should be performed, including inspection, palpation, auscultation and percussion
Rectal and pelvic examinations (in any patient with suspected pelvic pathology) should also be performed

## Slide 9
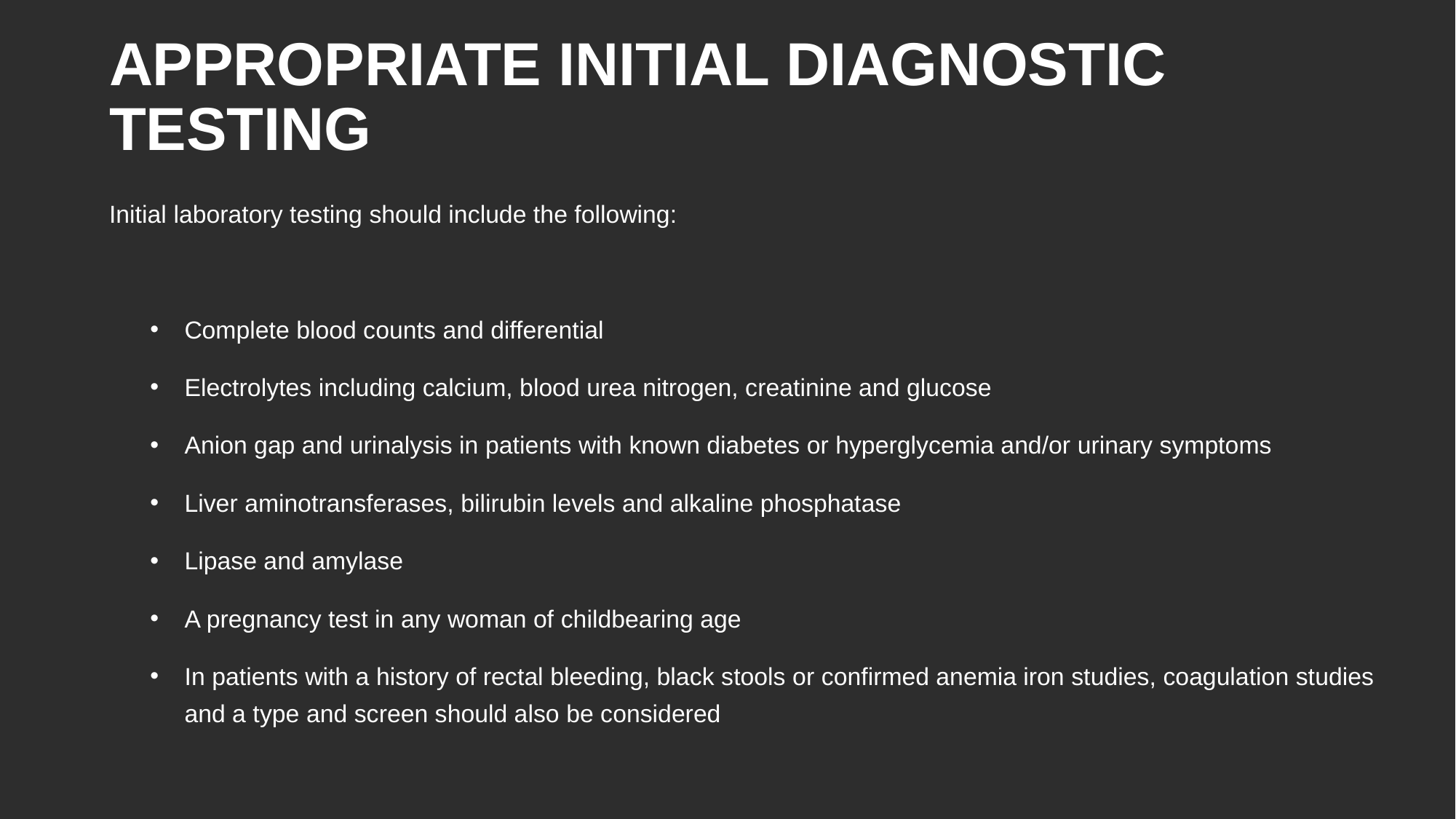

# Appropriate Initial Diagnostic Testing
Initial laboratory testing should include the following:
Complete blood counts and differential
Electrolytes including calcium, blood urea nitrogen, creatinine and glucose
Anion gap and urinalysis in patients with known diabetes or hyperglycemia and/or urinary symptoms
Liver aminotransferases, bilirubin levels and alkaline phosphatase
Lipase and amylase
A pregnancy test in any woman of childbearing age
In patients with a history of rectal bleeding, black stools or confirmed anemia iron studies, coagulation studies and a type and screen should also be considered

## Slide 10
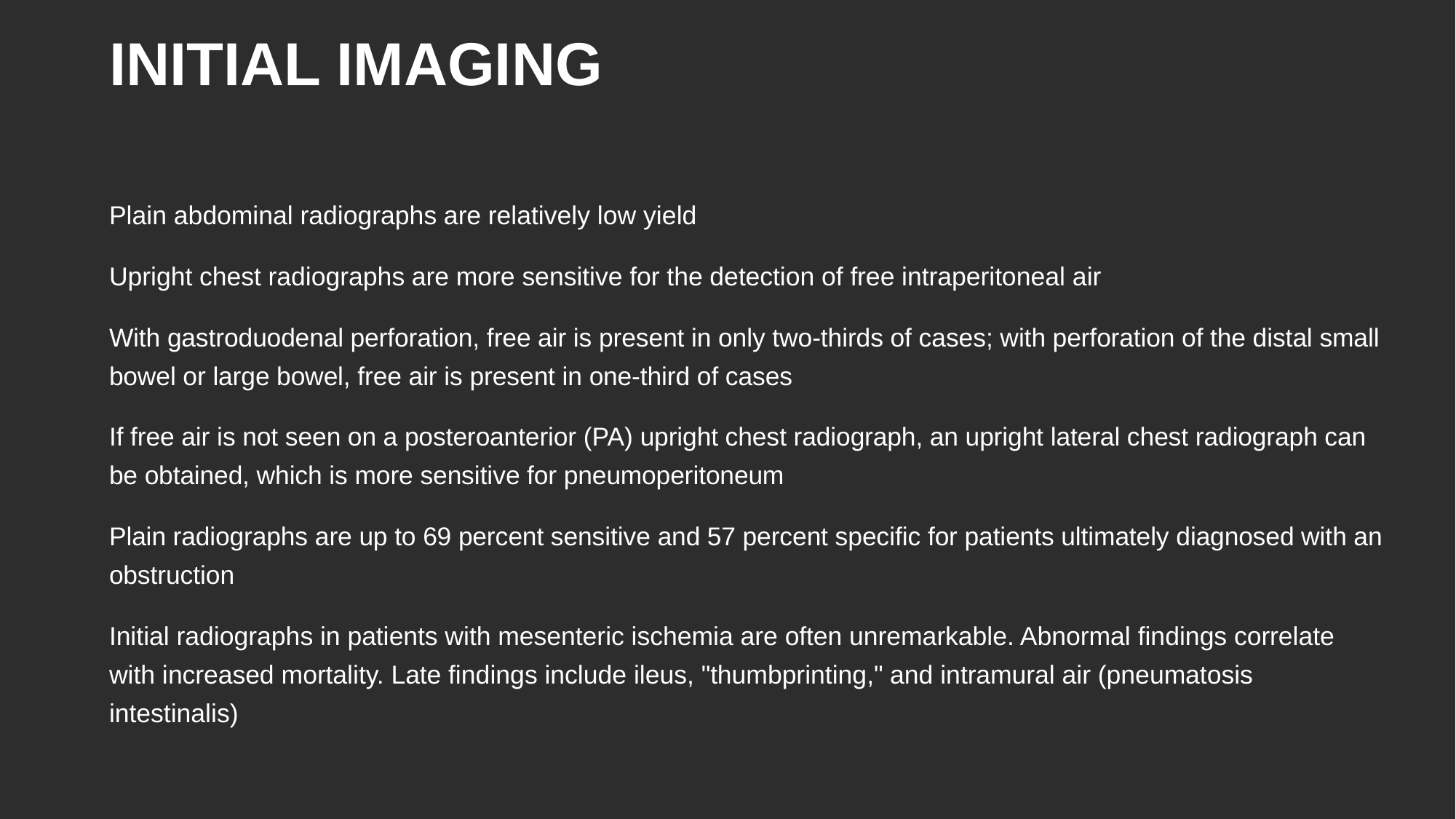

# Initial Imaging
Plain abdominal radiographs are relatively low yield
Upright chest radiographs are more sensitive for the detection of free intraperitoneal air
With gastroduodenal perforation, free air is present in only two-thirds of cases; with perforation of the distal small bowel or large bowel, free air is present in one-third of cases
If free air is not seen on a posteroanterior (PA) upright chest radiograph, an upright lateral chest radiograph can be obtained, which is more sensitive for pneumoperitoneum
Plain radiographs are up to 69 percent sensitive and 57 percent specific for patients ultimately diagnosed with an obstruction
Initial radiographs in patients with mesenteric ischemia are often unremarkable. Abnormal findings correlate with increased mortality. Late findings include ileus, "thumbprinting," and intramural air (pneumatosis intestinalis)

## Slide 11
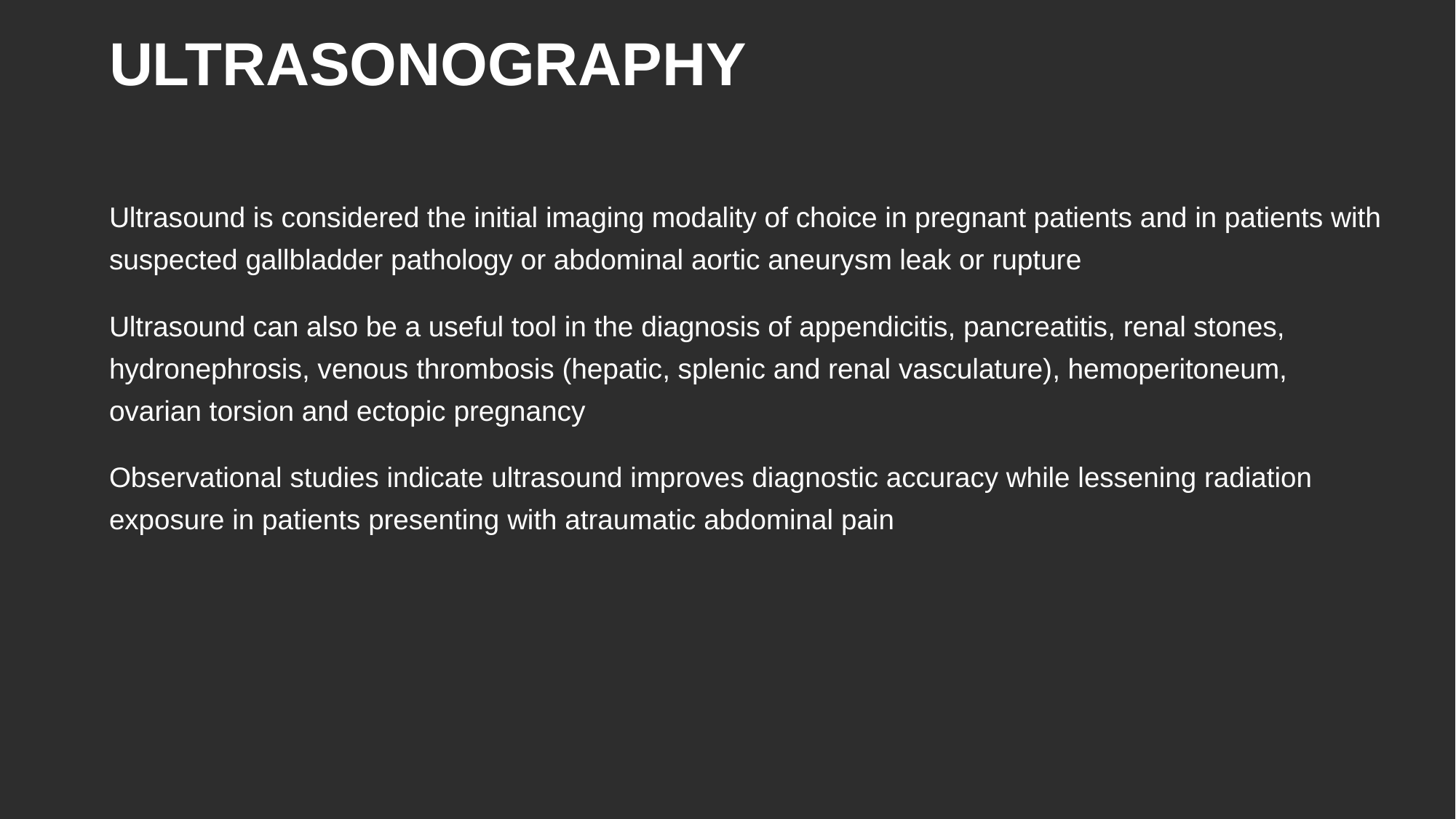

# Ultrasonography
Ultrasound is considered the initial imaging modality of choice in pregnant patients and in patients with suspected gallbladder pathology or abdominal aortic aneurysm leak or rupture
Ultrasound can also be a useful tool in the diagnosis of appendicitis, pancreatitis, renal stones, hydronephrosis, venous thrombosis (hepatic, splenic and renal vasculature), hemoperitoneum, ovarian torsion and ectopic pregnancy
Observational studies indicate ultrasound improves diagnostic accuracy while lessening radiation exposure in patients presenting with atraumatic abdominal pain

## Slide 12
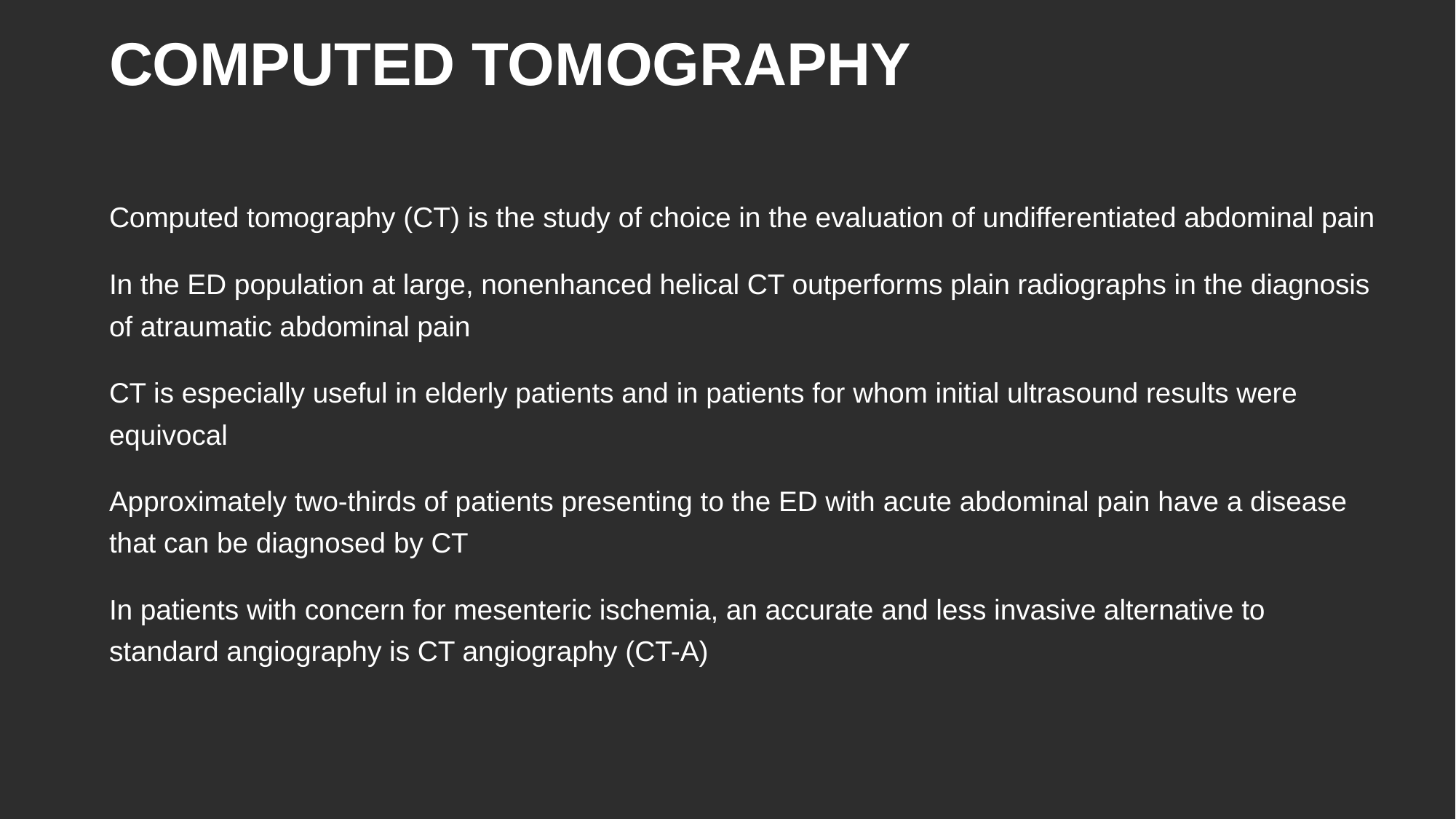

# Computed Tomography
Computed tomography (CT) is the study of choice in the evaluation of undifferentiated abdominal pain
In the ED population at large, nonenhanced helical CT outperforms plain radiographs in the diagnosis of atraumatic abdominal pain
CT is especially useful in elderly patients and in patients for whom initial ultrasound results were equivocal
Approximately two-thirds of patients presenting to the ED with acute abdominal pain have a disease that can be diagnosed by CT
In patients with concern for mesenteric ischemia, an accurate and less invasive alternative to standard angiography is CT angiography (CT-A)

## Slide 13
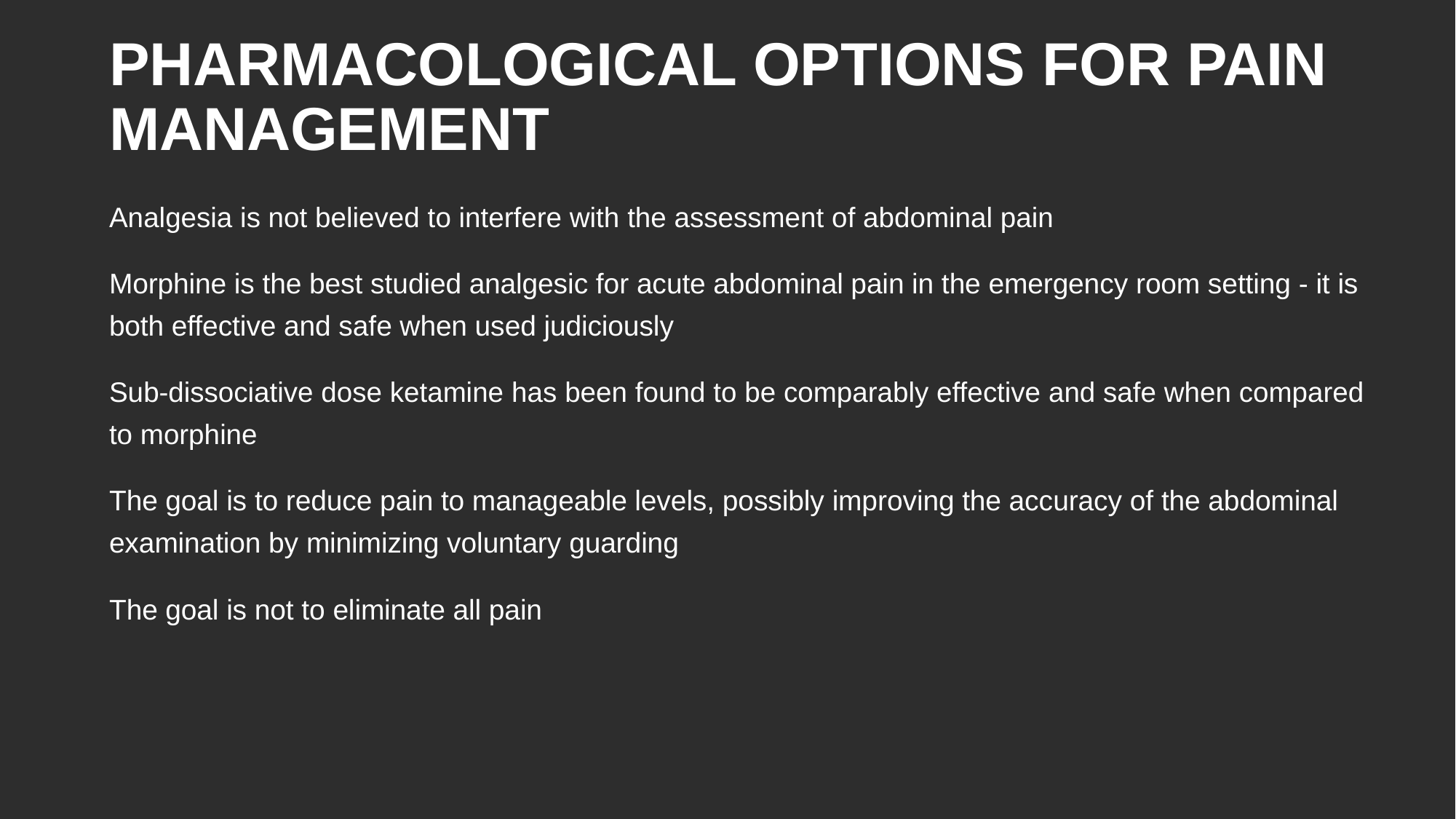

# Pharmacological Options for Pain Management
Analgesia is not believed to interfere with the assessment of abdominal pain
Morphine is the best studied analgesic for acute abdominal pain in the emergency room setting - it is both effective and safe when used judiciously
Sub-dissociative dose ketamine has been found to be comparably effective and safe when compared to morphine
The goal is to reduce pain to manageable levels, possibly improving the accuracy of the abdominal examination by minimizing voluntary guarding
The goal is not to eliminate all pain

## Slide 14
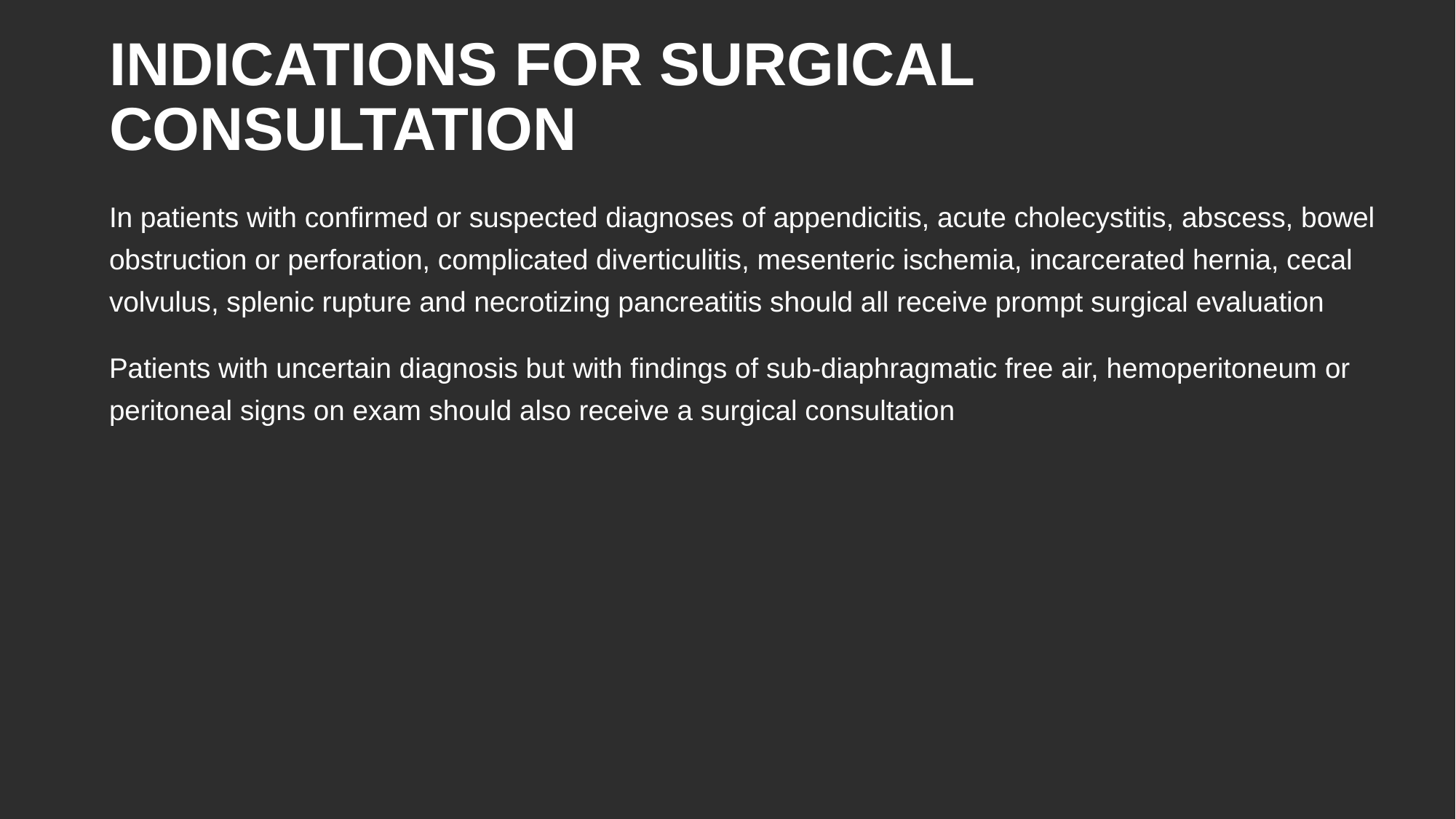

# Indications for Surgical Consultation
In patients with confirmed or suspected diagnoses of appendicitis, acute cholecystitis, abscess, bowel obstruction or perforation, complicated diverticulitis, mesenteric ischemia, incarcerated hernia, cecal volvulus, splenic rupture and necrotizing pancreatitis should all receive prompt surgical evaluation
Patients with uncertain diagnosis but with findings of sub-diaphragmatic free air, hemoperitoneum or peritoneal signs on exam should also receive a surgical consultation

## Slide 15
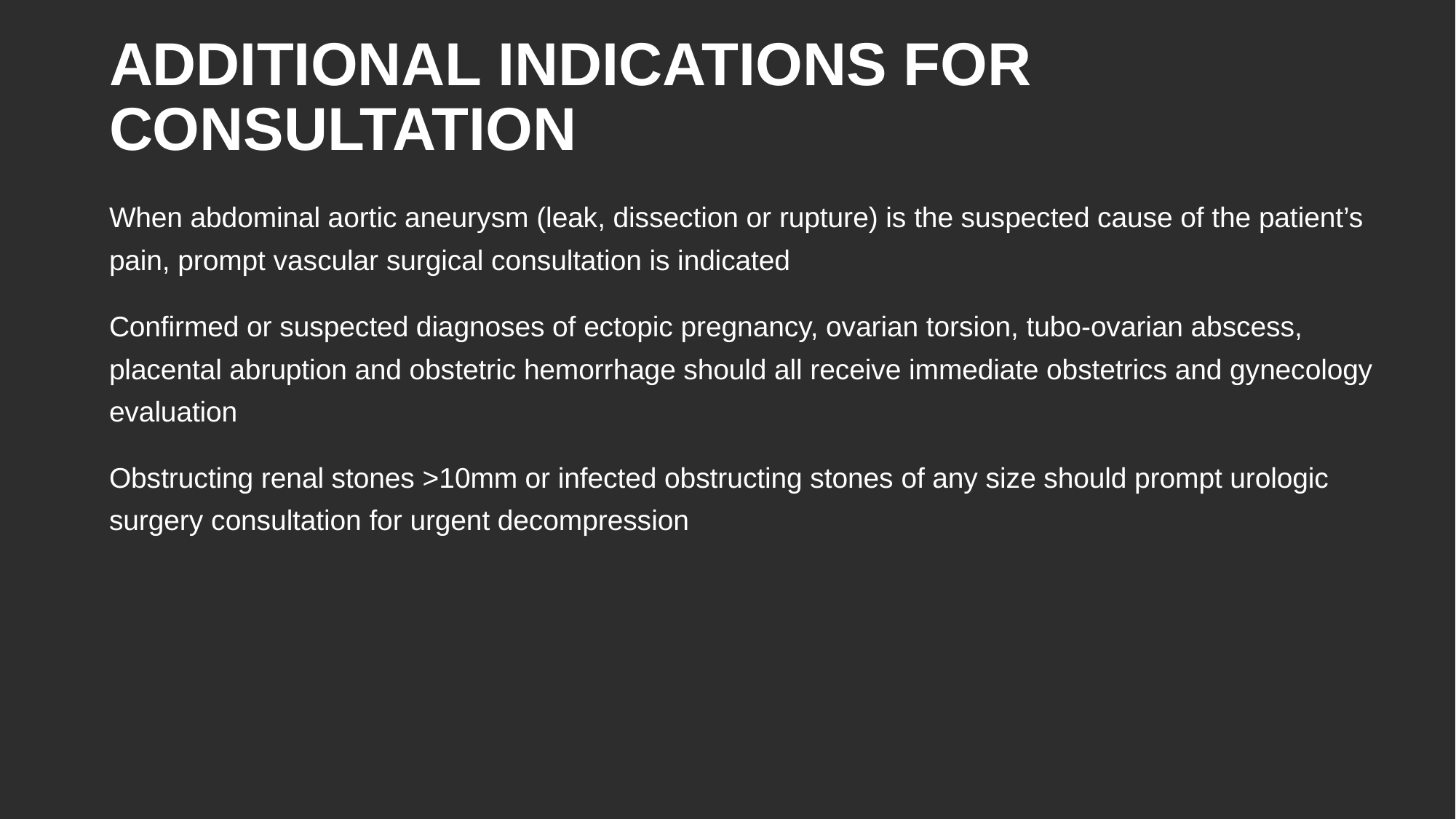

# Additional Indications for Consultation
When abdominal aortic aneurysm (leak, dissection or rupture) is the suspected cause of the patient’s pain, prompt vascular surgical consultation is indicated
Confirmed or suspected diagnoses of ectopic pregnancy, ovarian torsion, tubo-ovarian abscess, placental abruption and obstetric hemorrhage should all receive immediate obstetrics and gynecology evaluation
Obstructing renal stones >10mm or infected obstructing stones of any size should prompt urologic surgery consultation for urgent decompression

## Slide 16
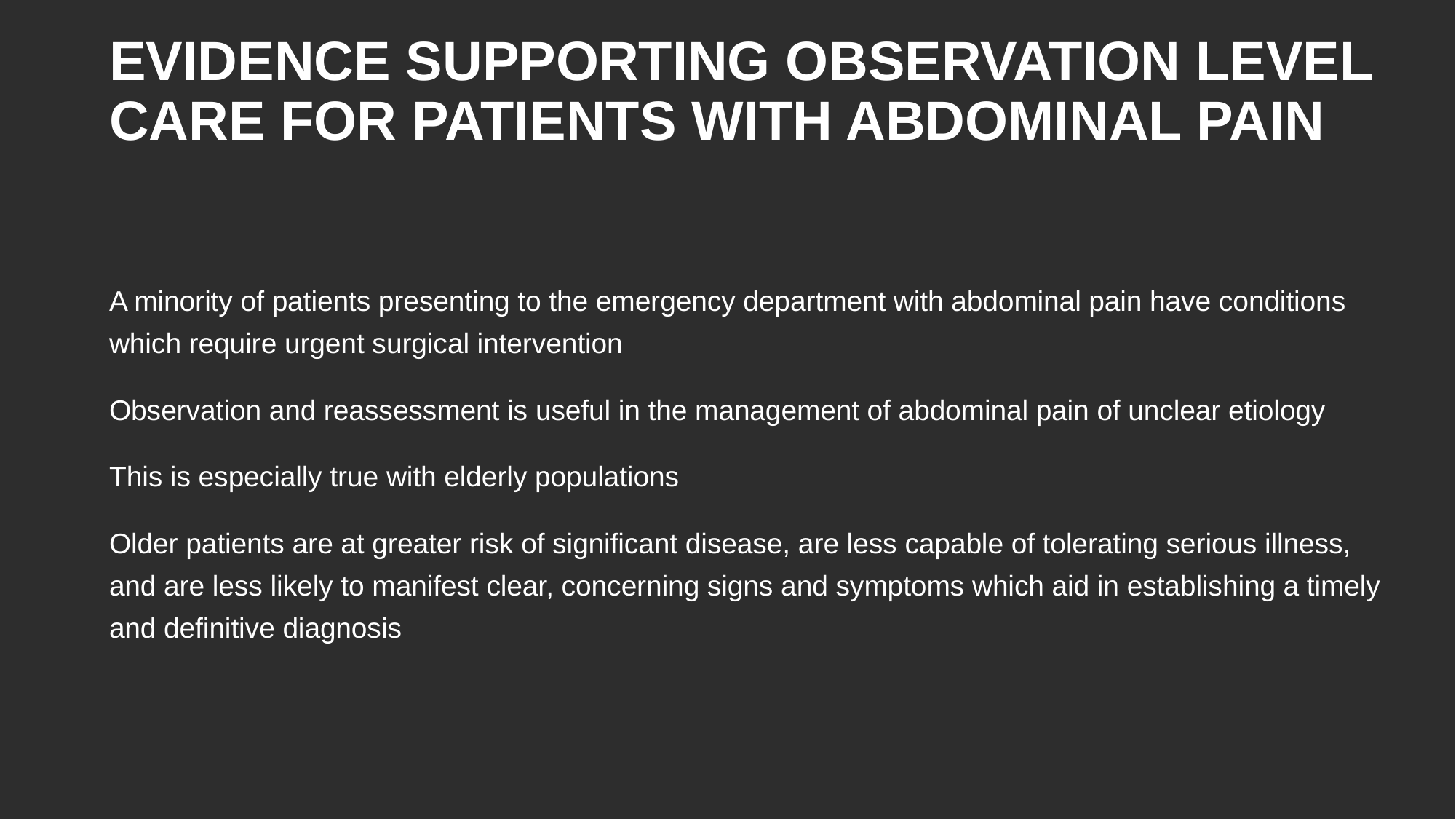

# Evidence Supporting Observation Level Care for Patients with Abdominal Pain
A minority of patients presenting to the emergency department with abdominal pain have conditions which require urgent surgical intervention
Observation and reassessment is useful in the management of abdominal pain of unclear etiology
This is especially true with elderly populations
Older patients are at greater risk of significant disease, are less capable of tolerating serious illness, and are less likely to manifest clear, concerning signs and symptoms which aid in establishing a timely and definitive diagnosis

## Slide 17
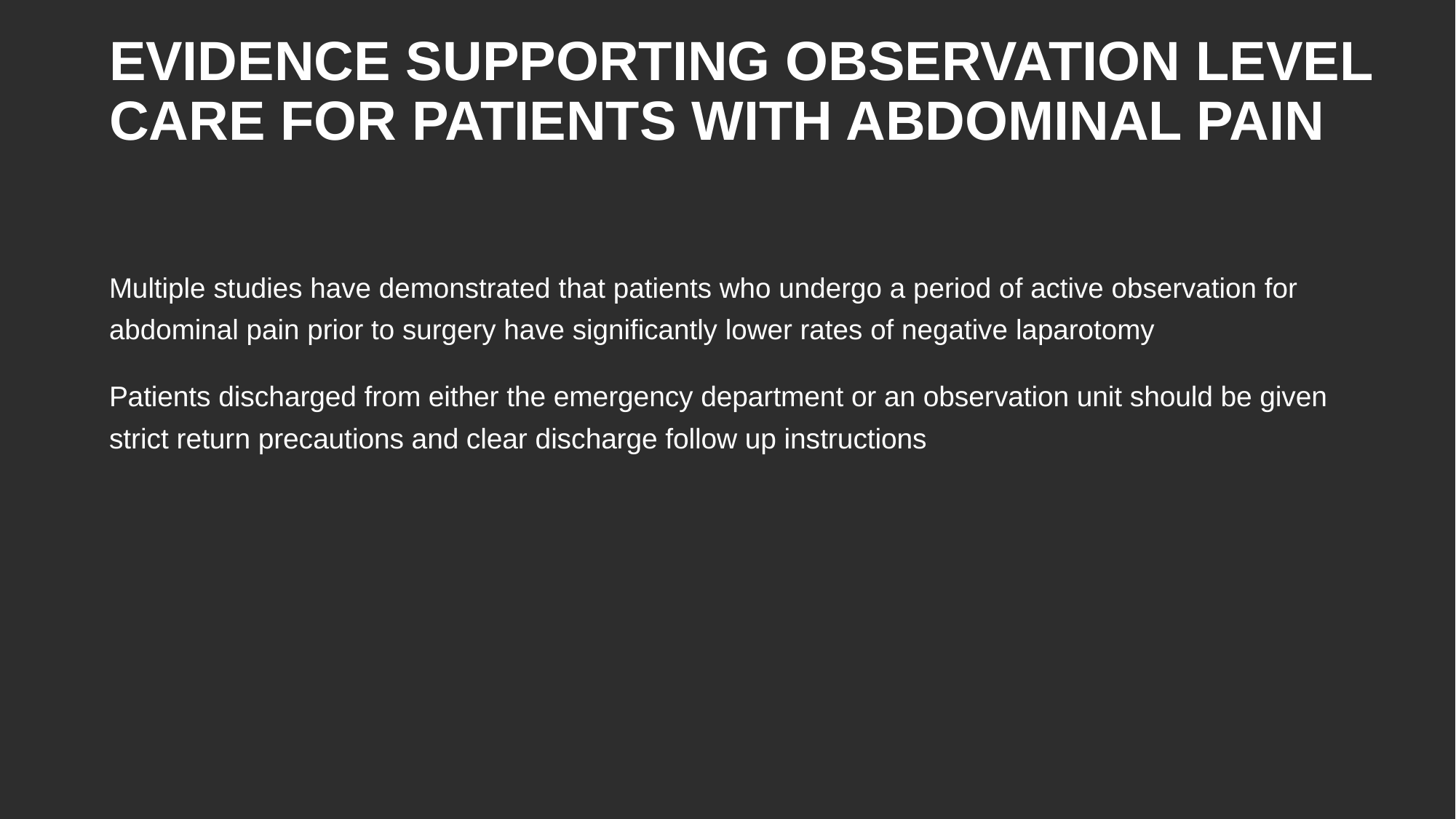

# Evidence Supporting Observation Level Care for Patients with Abdominal Pain
Multiple studies have demonstrated that patients who undergo a period of active observation for abdominal pain prior to surgery have significantly lower rates of negative laparotomy
Patients discharged from either the emergency department or an observation unit should be given strict return precautions and clear discharge follow up instructions

## Slide 18
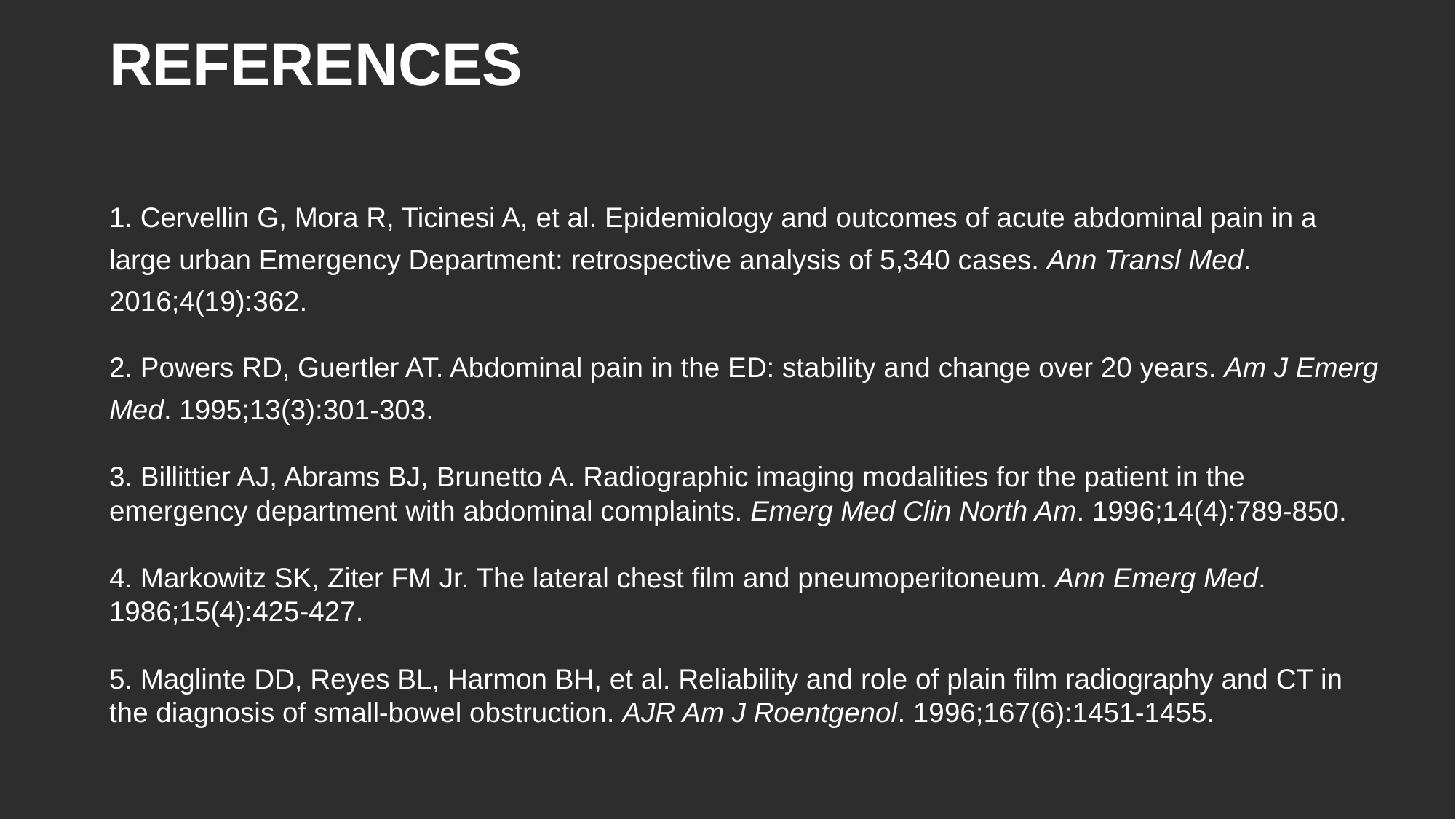

# references
1. Cervellin G, Mora R, Ticinesi A, et al. Epidemiology and outcomes of acute abdominal pain in a large urban Emergency Department: retrospective analysis of 5,340 cases. Ann Transl Med. 2016;4(19):362.
2. Powers RD, Guertler AT. Abdominal pain in the ED: stability and change over 20 years. Am J Emerg Med. 1995;13(3):301-303.
3. Billittier AJ, Abrams BJ, Brunetto A. Radiographic imaging modalities for the patient in the emergency department with abdominal complaints. Emerg Med Clin North Am. 1996;14(4):789-850.
4. Markowitz SK, Ziter FM Jr. The lateral chest film and pneumoperitoneum. Ann Emerg Med. 1986;15(4):425-427.
5. Maglinte DD, Reyes BL, Harmon BH, et al. Reliability and role of plain film radiography and CT in the diagnosis of small-bowel obstruction. AJR Am J Roentgenol. 1996;167(6):1451-1455.

## Slide 19
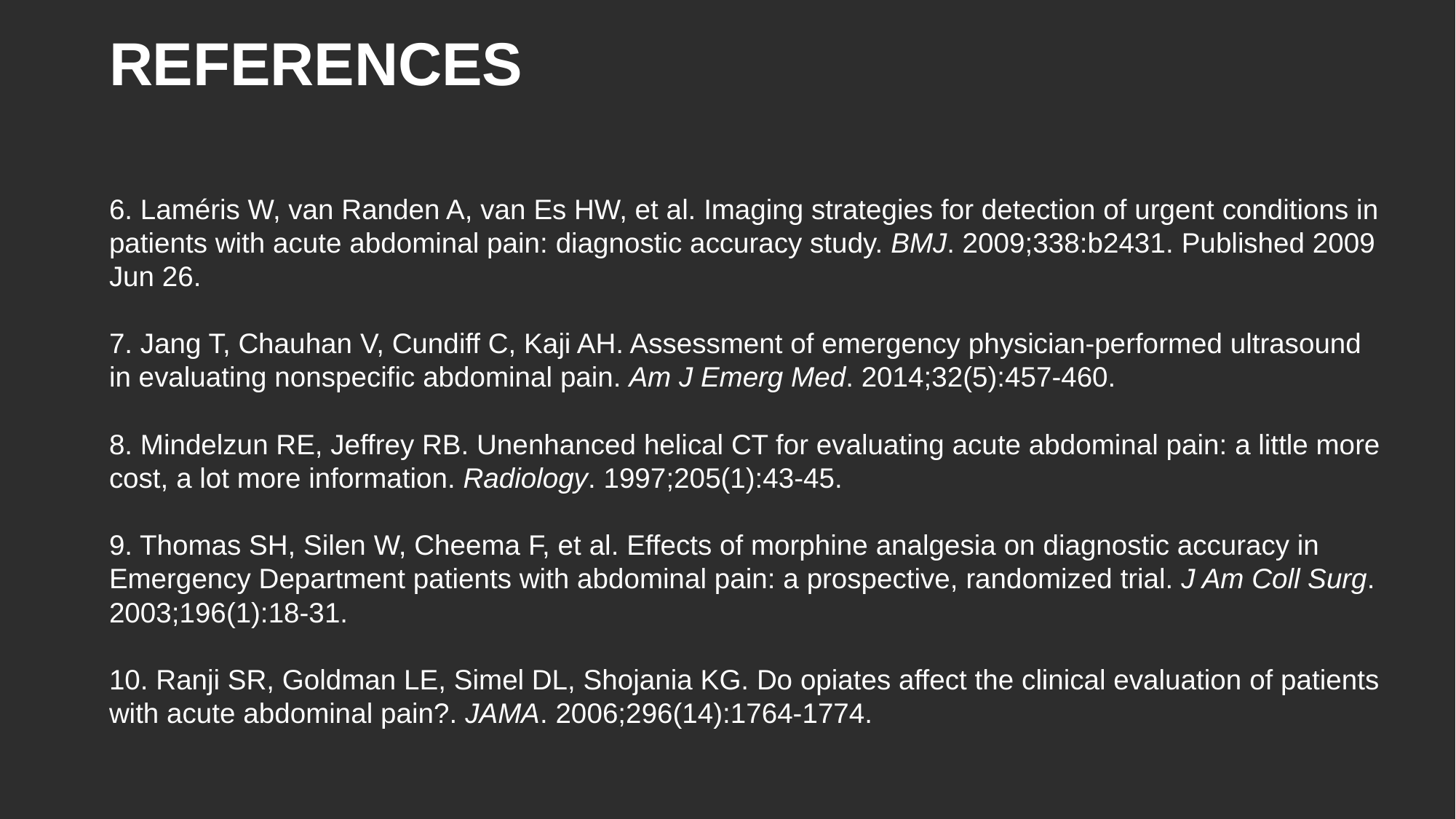

# References
6. Laméris W, van Randen A, van Es HW, et al. Imaging strategies for detection of urgent conditions in patients with acute abdominal pain: diagnostic accuracy study. BMJ. 2009;338:b2431. Published 2009 Jun 26.
7. Jang T, Chauhan V, Cundiff C, Kaji AH. Assessment of emergency physician-performed ultrasound in evaluating nonspecific abdominal pain. Am J Emerg Med. 2014;32(5):457-460.
8. Mindelzun RE, Jeffrey RB. Unenhanced helical CT for evaluating acute abdominal pain: a little more cost, a lot more information. Radiology. 1997;205(1):43-45.
9. Thomas SH, Silen W, Cheema F, et al. Effects of morphine analgesia on diagnostic accuracy in Emergency Department patients with abdominal pain: a prospective, randomized trial. J Am Coll Surg. 2003;196(1):18-31.
10. Ranji SR, Goldman LE, Simel DL, Shojania KG. Do opiates affect the clinical evaluation of patients with acute abdominal pain?. JAMA. 2006;296(14):1764-1774.

## Slide 20
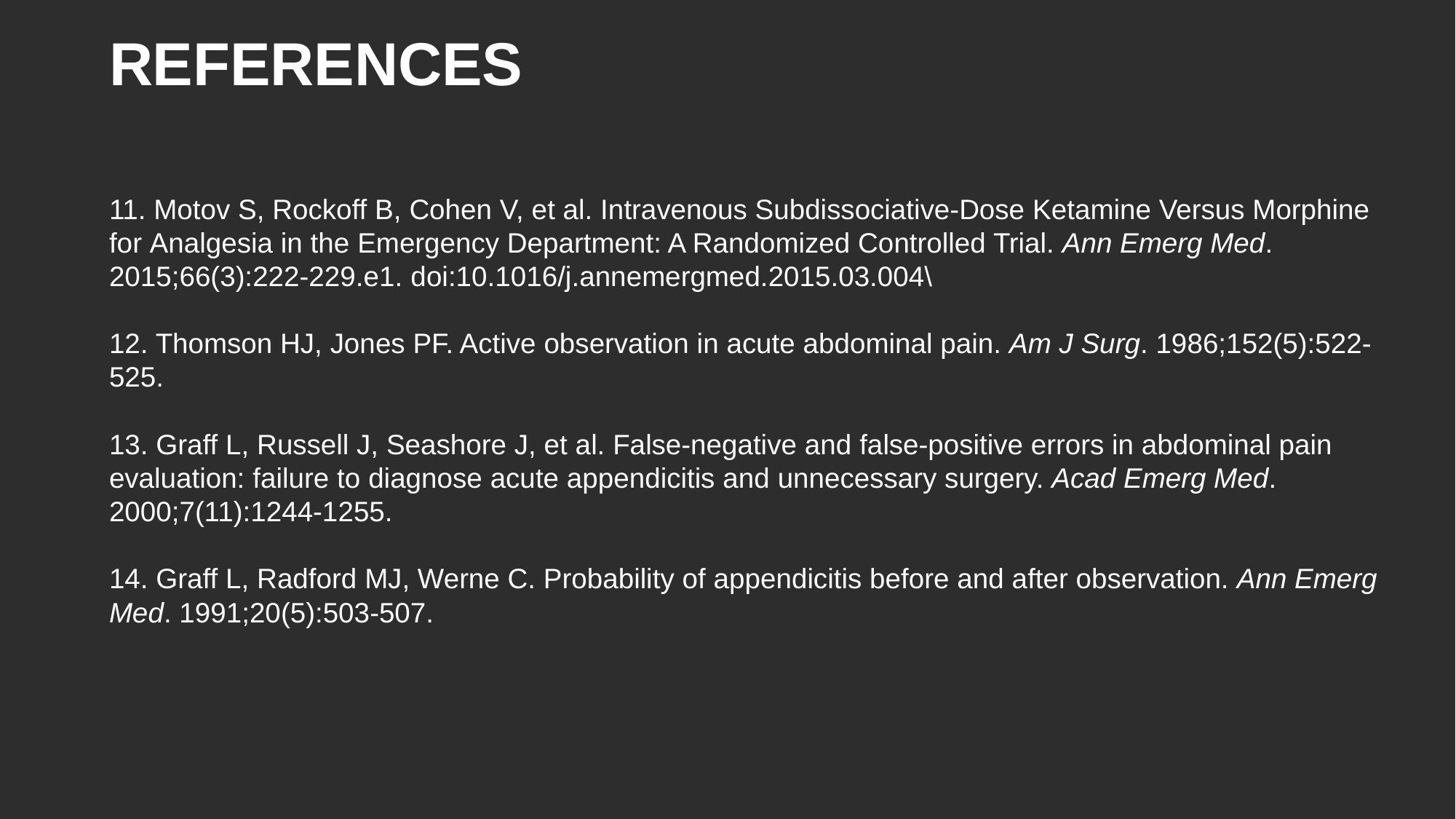

# References
11. Motov S, Rockoff B, Cohen V, et al. Intravenous Subdissociative-Dose Ketamine Versus Morphine for Analgesia in the Emergency Department: A Randomized Controlled Trial. Ann Emerg Med. 2015;66(3):222-229.e1. doi:10.1016/j.annemergmed.2015.03.004\
12. Thomson HJ, Jones PF. Active observation in acute abdominal pain. Am J Surg. 1986;152(5):522-525.
13. Graff L, Russell J, Seashore J, et al. False-negative and false-positive errors in abdominal pain evaluation: failure to diagnose acute appendicitis and unnecessary surgery. Acad Emerg Med. 2000;7(11):1244-1255.
14. Graff L, Radford MJ, Werne C. Probability of appendicitis before and after observation. Ann Emerg Med. 1991;20(5):503-507.
