# Supplementary material for: An Observation Medicine Curriculum for Emergency Medicine Education
Source: J Educ Teach Emerg Med. 2021 Apr 19;6(2):C1–C72. doi: 10.21980/J87P92 (PMC10332786; doi:10.21980/J87P92)
Supplement: Supplementary file 9 — Please see associated PowerPoint file [file jetem-6-2-c1-supp9.pptx]

## Slide 1
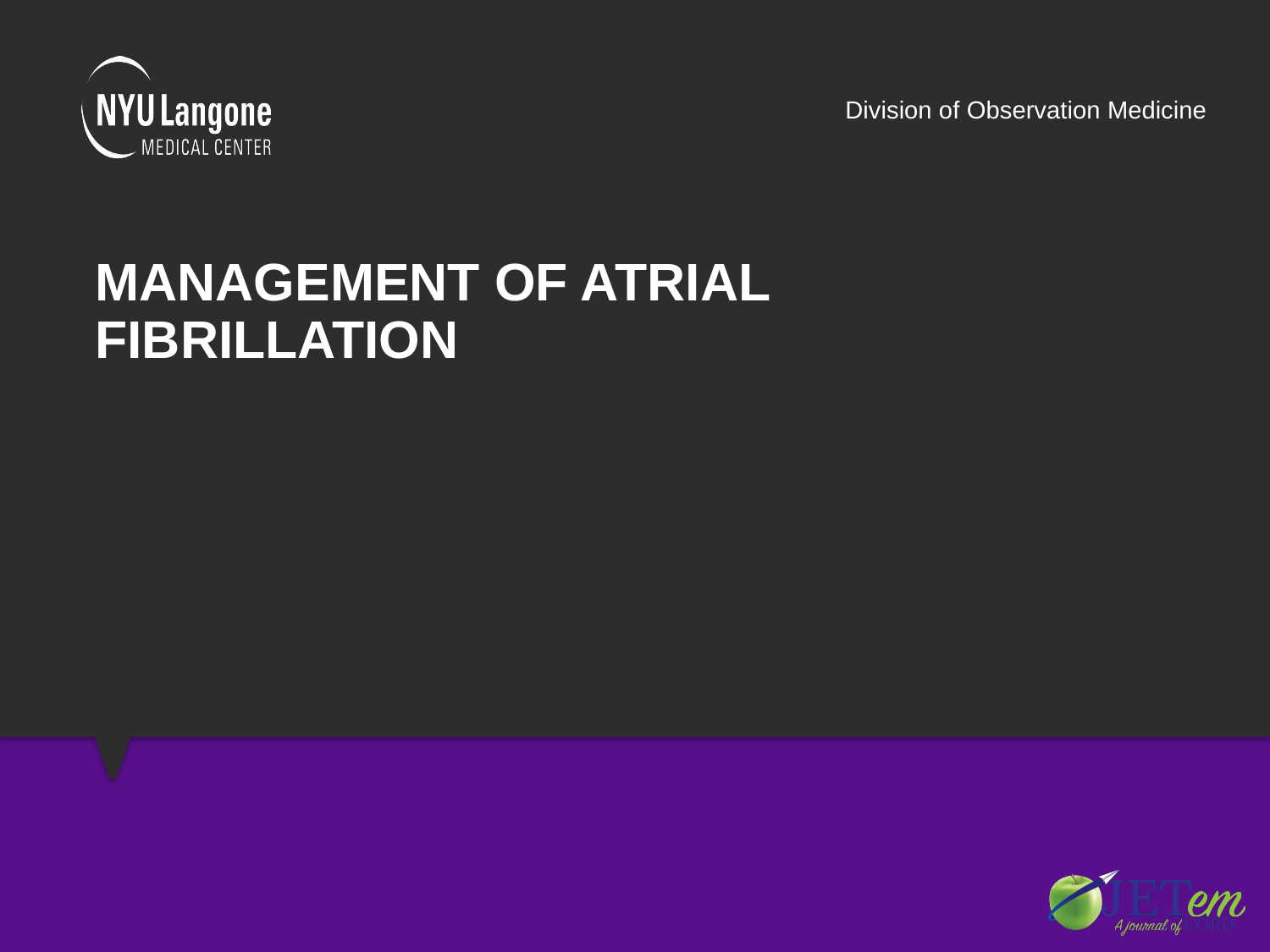

Division of Observation Medicine
# Management of Atrial Fibrillation​

## Slide 2
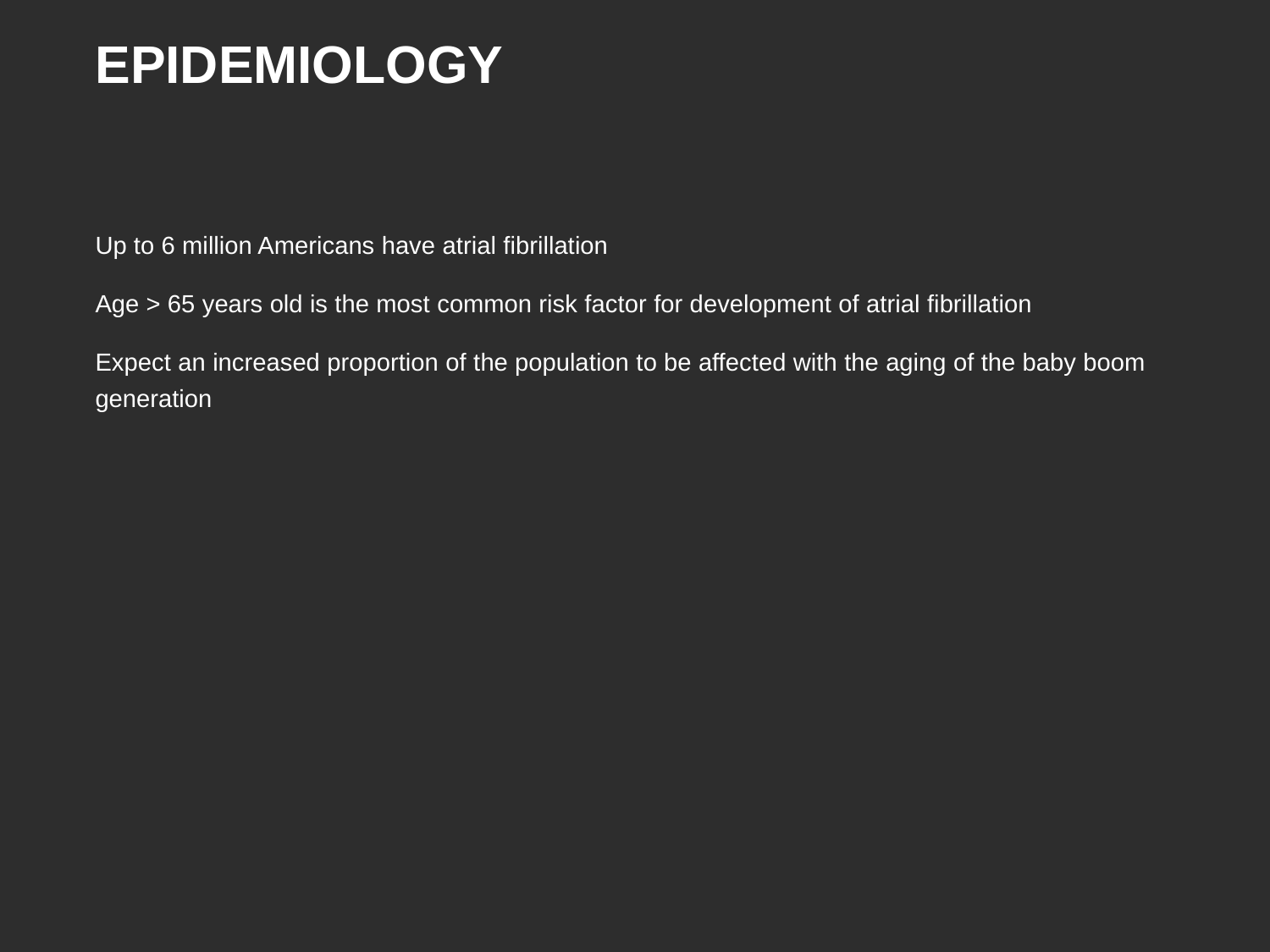

# Epidemiology
Up to 6 million Americans have atrial fibrillation
Age > 65 years old is the most common risk factor for development of atrial fibrillation
Expect an increased proportion of the population to be affected with the aging of the baby boom generation

## Slide 3
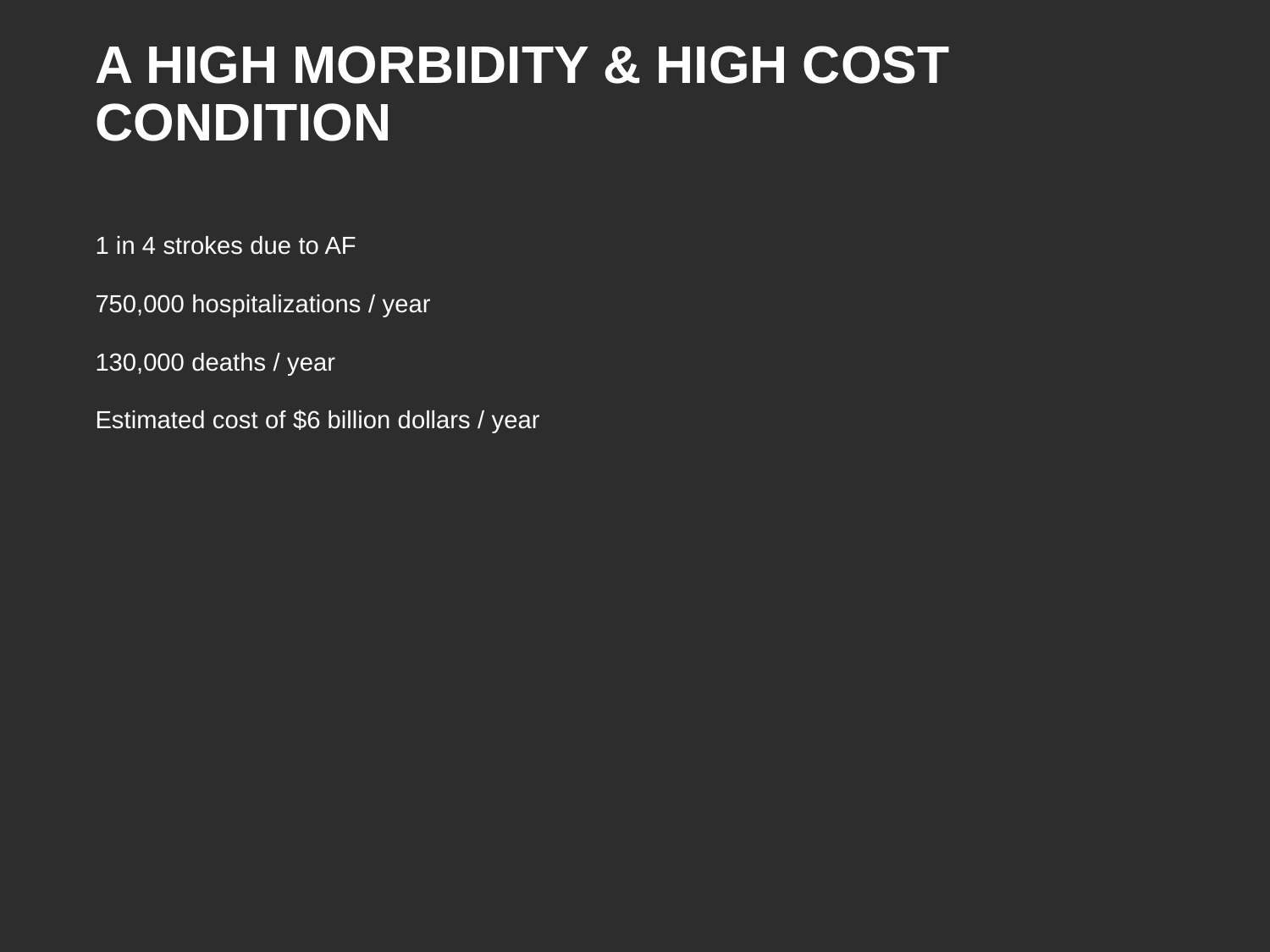

# A High Morbidity & High Cost Condition
1 in 4 strokes due to AF
750,000 hospitalizations / year
130,000 deaths / year
Estimated cost of $6 billion dollars / year

## Slide 4
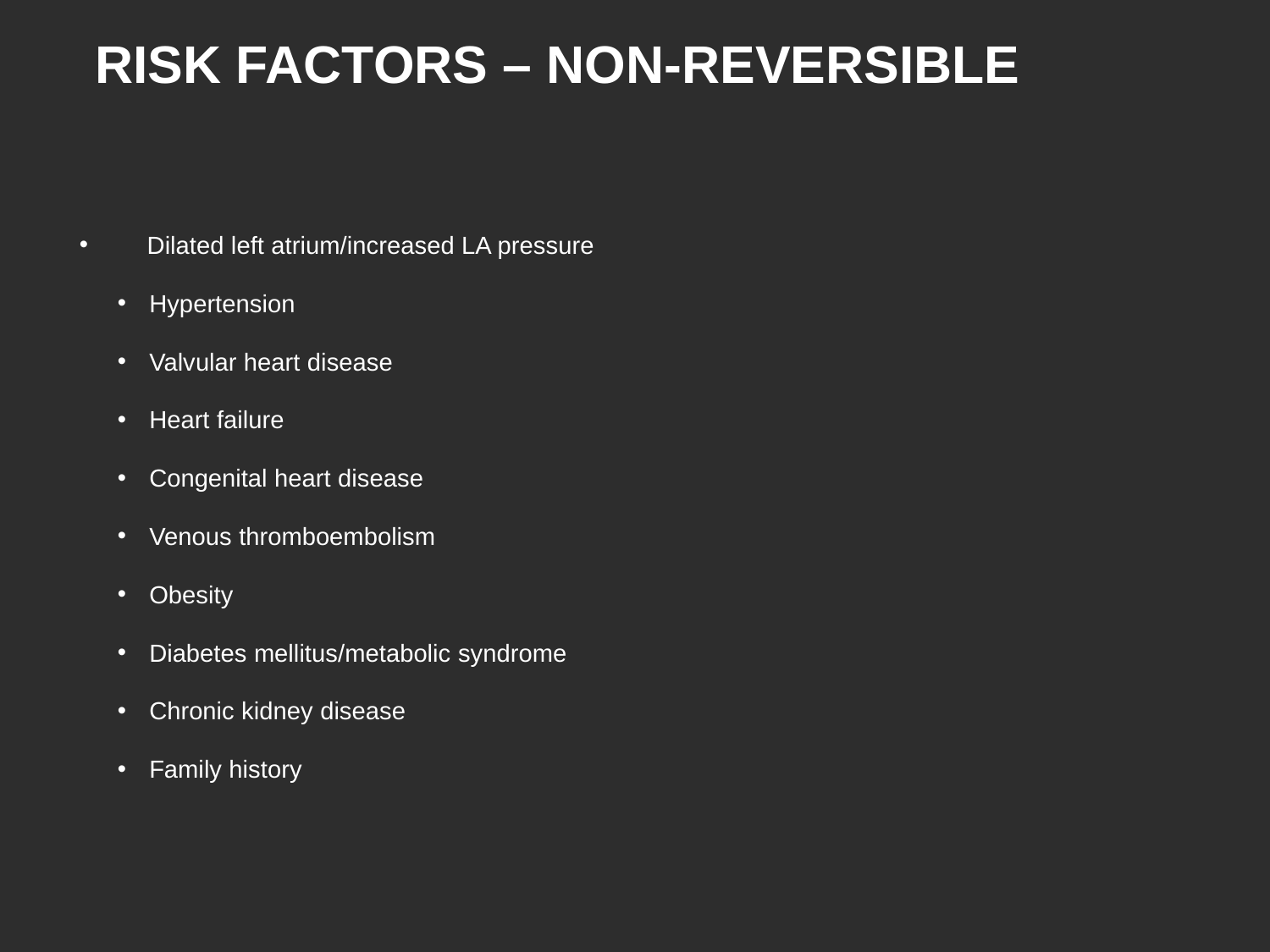

# Risk Factors – Non-Reversible
 Dilated left atrium/increased LA pressure
Hypertension
Valvular heart disease
Heart failure
Congenital heart disease
Venous thromboembolism
Obesity
Diabetes mellitus/metabolic syndrome
Chronic kidney disease
Family history

## Slide 5
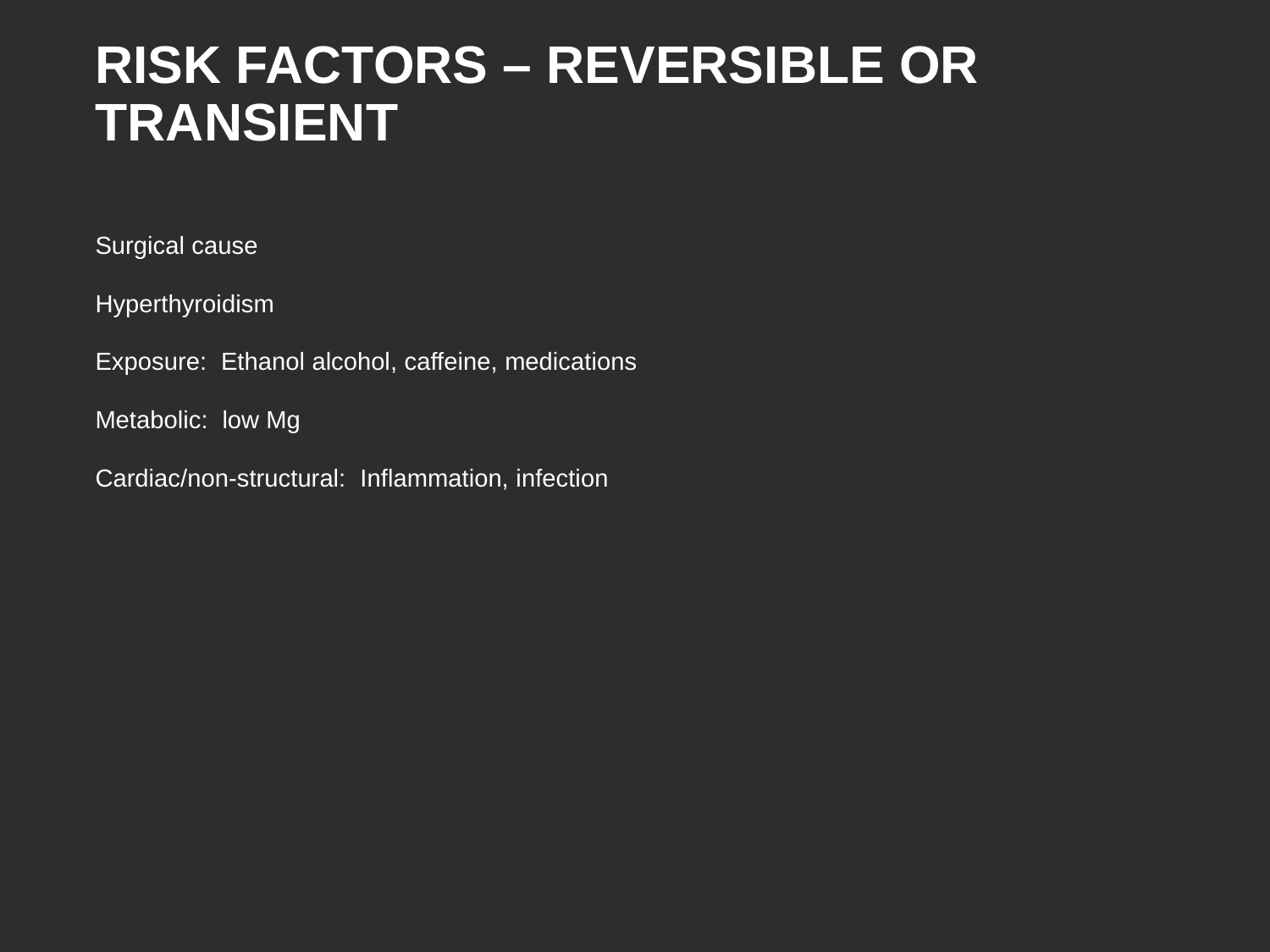

# Risk Factors – Reversible or Transient
Surgical cause
Hyperthyroidism
Exposure: Ethanol alcohol, caffeine, medications
Metabolic: low Mg
Cardiac/non-structural: Inflammation, infection

## Slide 6
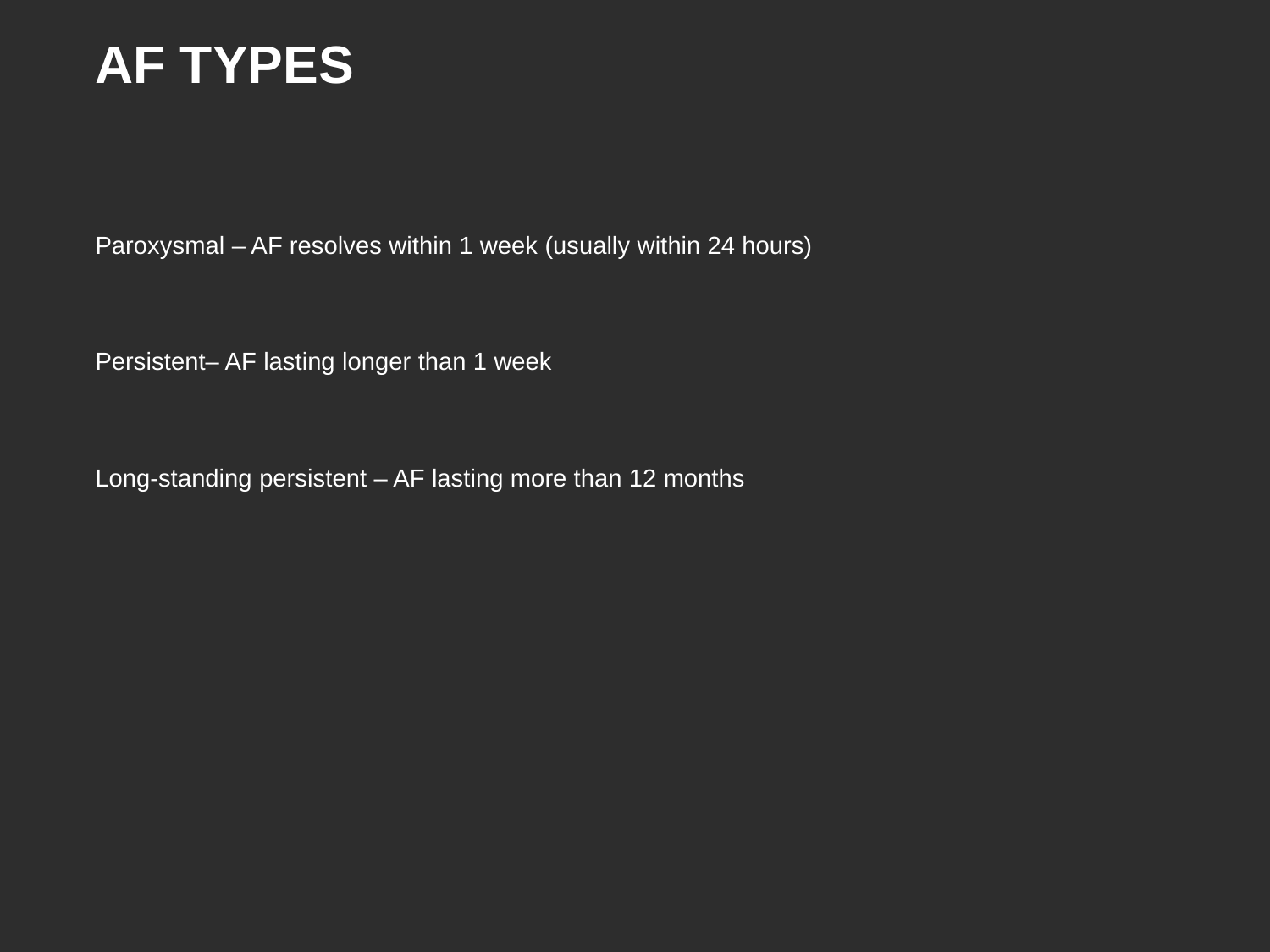

# AF Types
Paroxysmal – AF resolves within 1 week (usually within 24 hours)
Persistent– AF lasting longer than 1 week
Long-standing persistent – AF lasting more than 12 months

## Slide 7
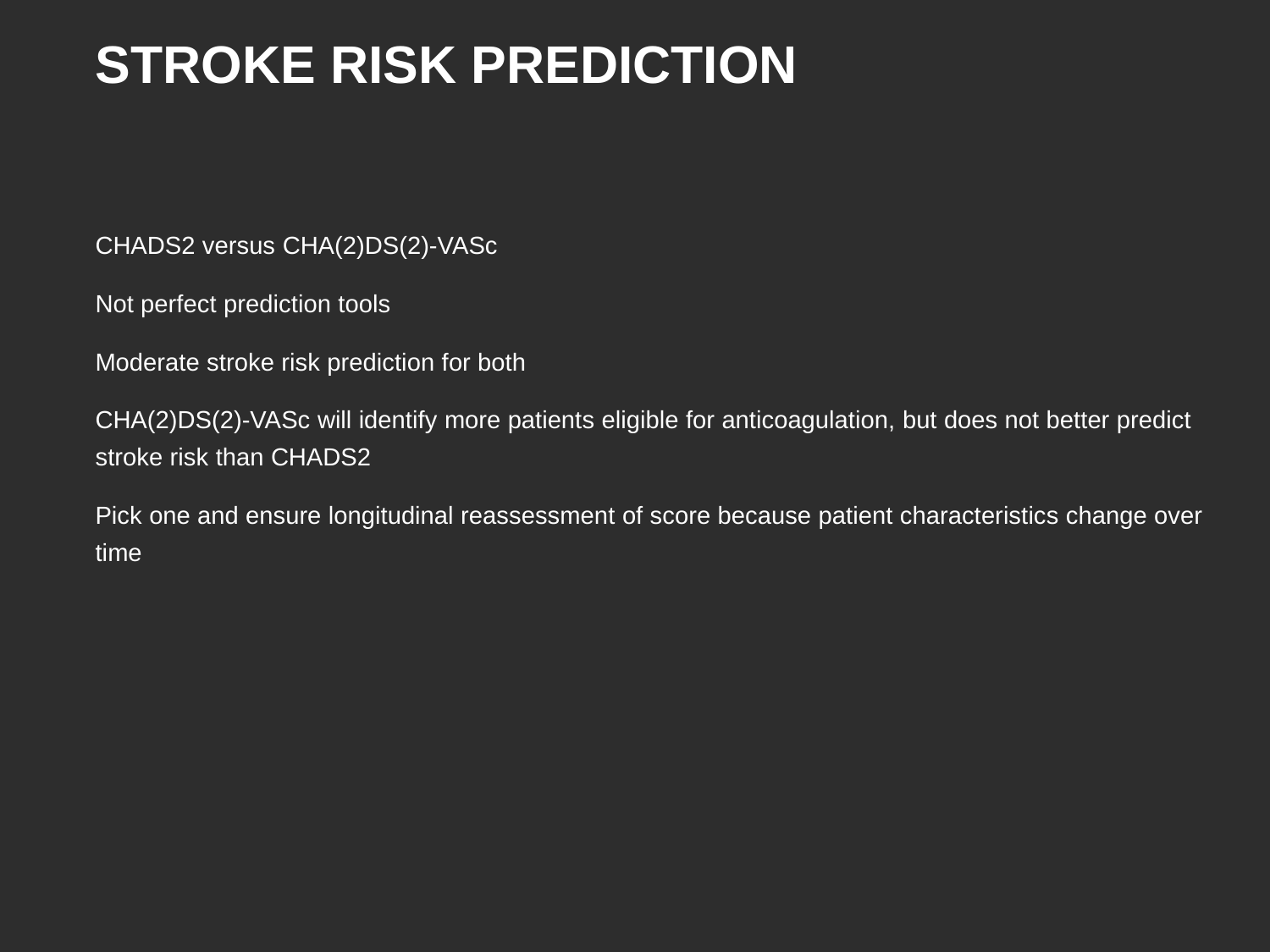

# Stroke Risk Prediction
CHADS2 versus CHA(2)DS(2)-VASc
Not perfect prediction tools
Moderate stroke risk prediction for both
CHA(2)DS(2)-VASc will identify more patients eligible for anticoagulation, but does not better predict stroke risk than CHADS2
Pick one and ensure longitudinal reassessment of score because patient characteristics change over time

## Slide 8
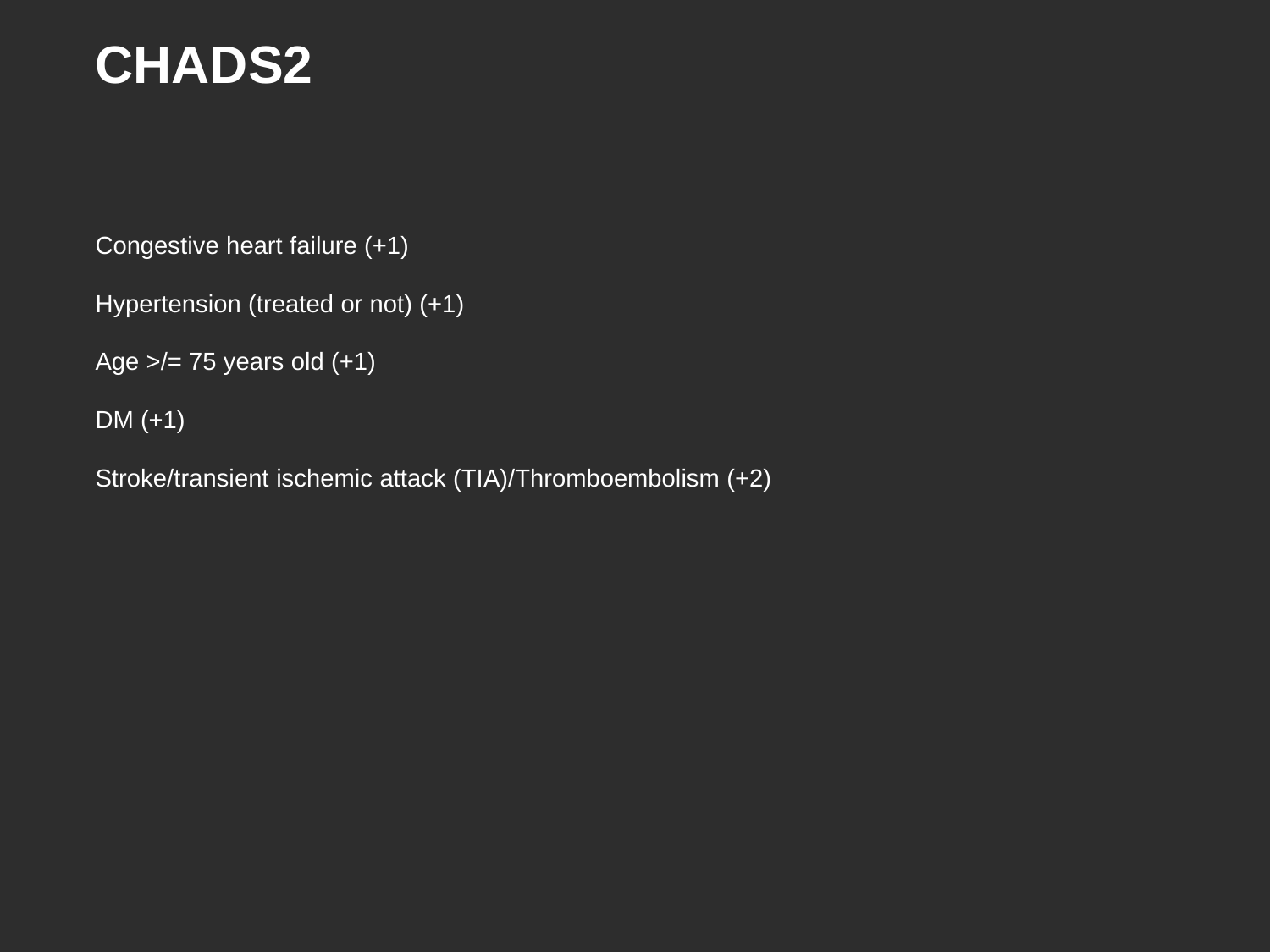

# CHADS2
Congestive heart failure (+1)
Hypertension (treated or not) (+1)
Age >/= 75 years old (+1)
DM (+1)
Stroke/transient ischemic attack (TIA)/Thromboembolism (+2)

## Slide 9
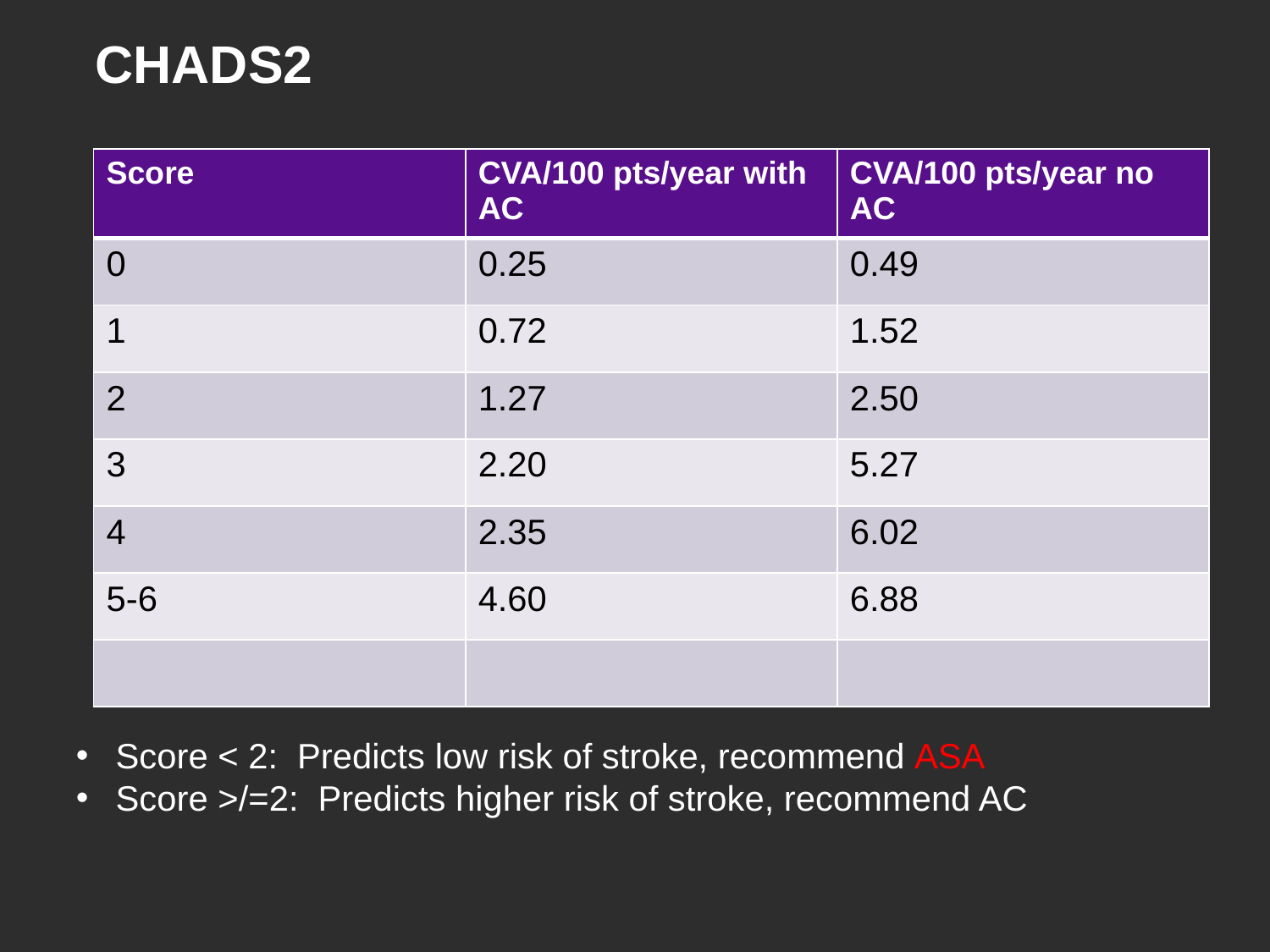

# CHADS2
| Score | CVA/100 pts/year with AC | CVA/100 pts/year no AC |
| --- | --- | --- |
| 0 | 0.25 | 0.49 |
| 1 | 0.72 | 1.52 |
| 2 | 1.27 | 2.50 |
| 3 | 2.20 | 5.27 |
| 4 | 2.35 | 6.02 |
| 5-6 | 4.60 | 6.88 |
| | | |
Score < 2:  Predicts low risk of stroke, recommend ASA
Score >/=2:  Predicts higher risk of stroke, recommend AC

## Slide 10
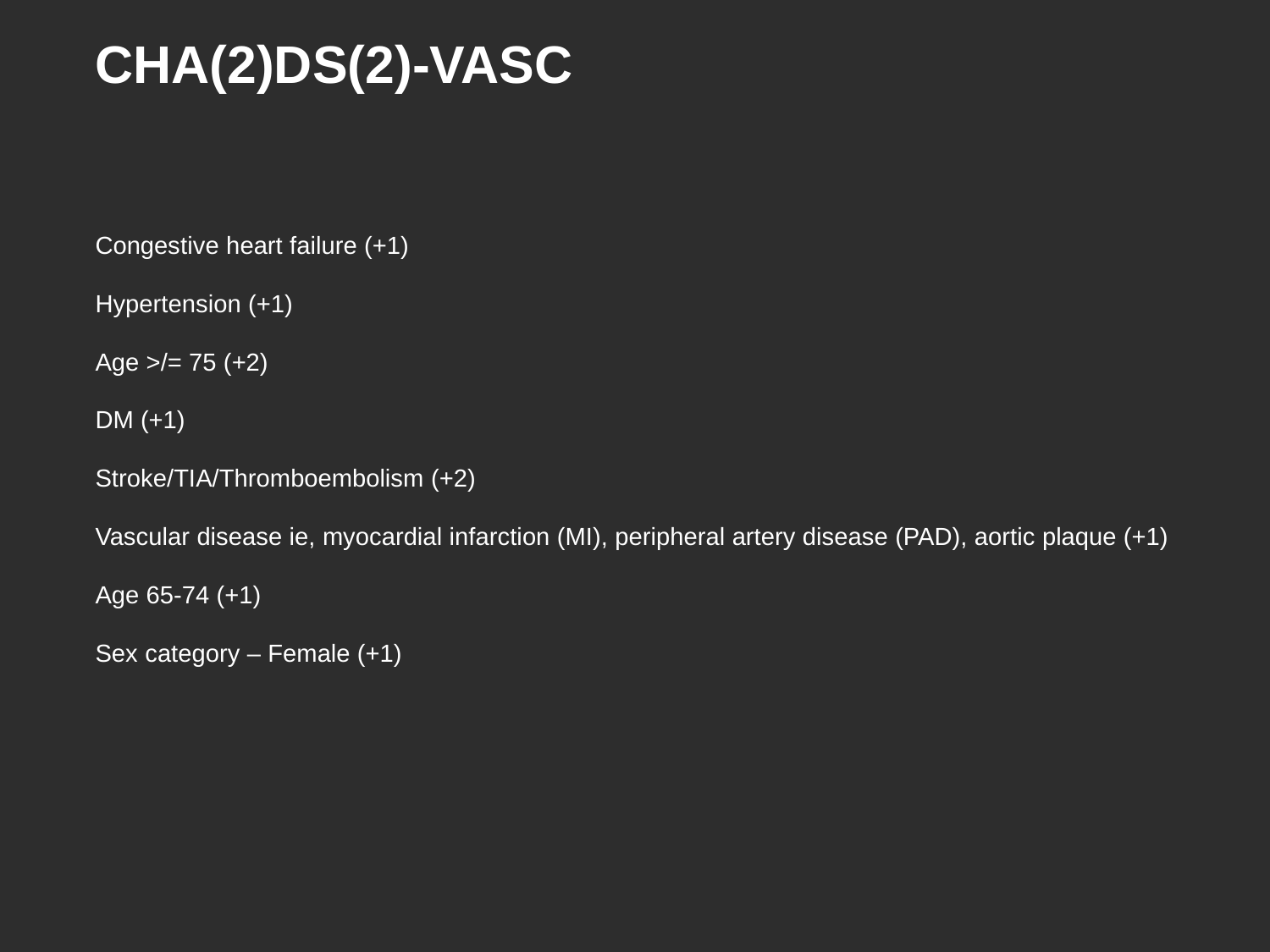

# CHA(2)DS(2)-VASc
Congestive heart failure (+1)
Hypertension (+1)
Age >/= 75 (+2)
DM (+1)
Stroke/TIA/Thromboembolism (+2)
Vascular disease ie, myocardial infarction (MI), peripheral artery disease (PAD), aortic plaque (+1)
Age 65-74 (+1)
Sex category – Female (+1)

## Slide 11
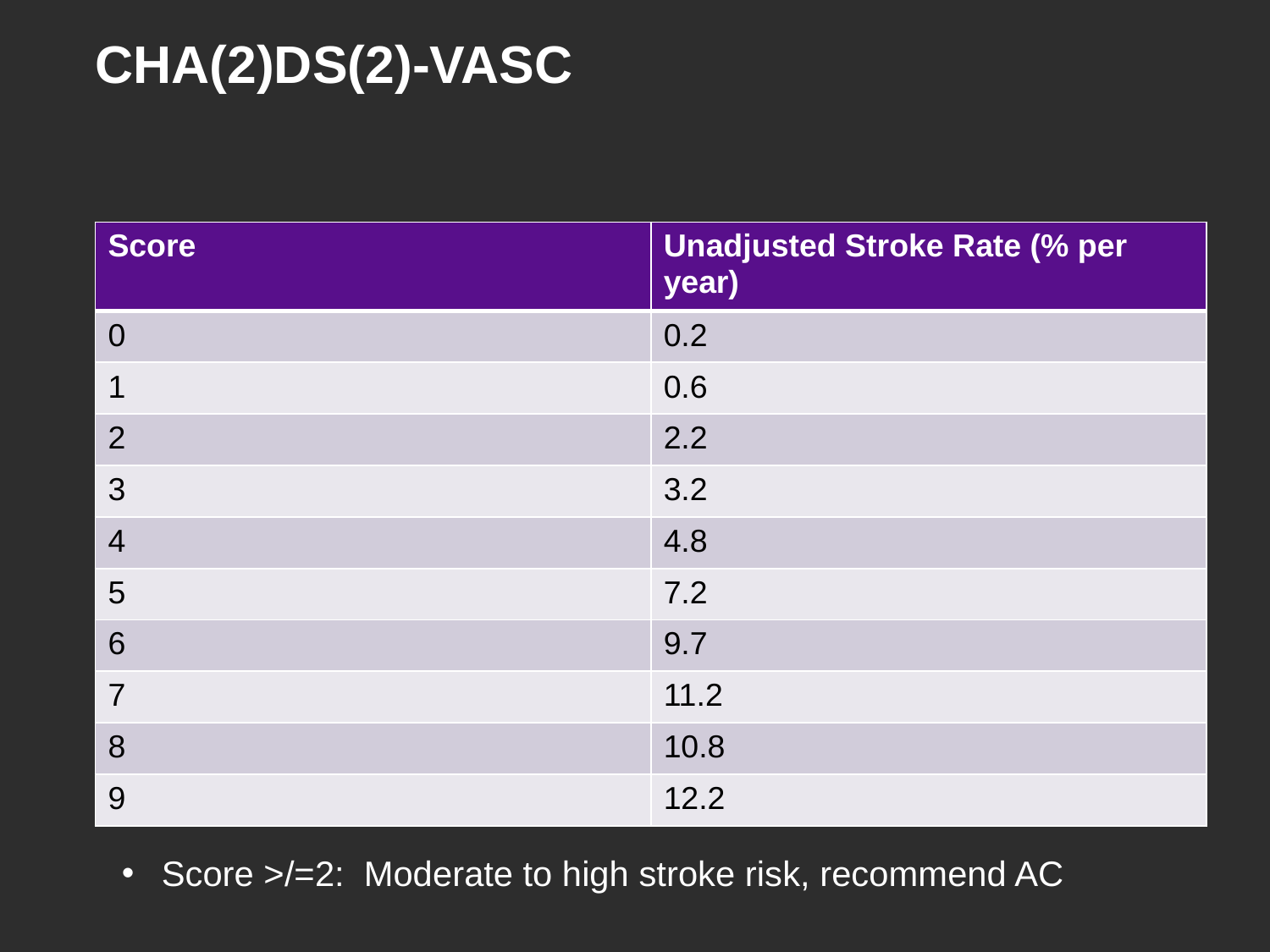

# CHA(2)DS(2)-VASc
| Score | Unadjusted Stroke Rate (% per year) |
| --- | --- |
| 0 | 0.2 |
| 1 | 0.6 |
| 2 | 2.2 |
| 3 | 3.2 |
| 4 | 4.8 |
| 5 | 7.2 |
| 6 | 9.7 |
| 7 | 11.2 |
| 8 | 10.8 |
| 9 | 12.2 |
Score >/=2:  Moderate to high stroke risk, recommend AC

## Slide 12
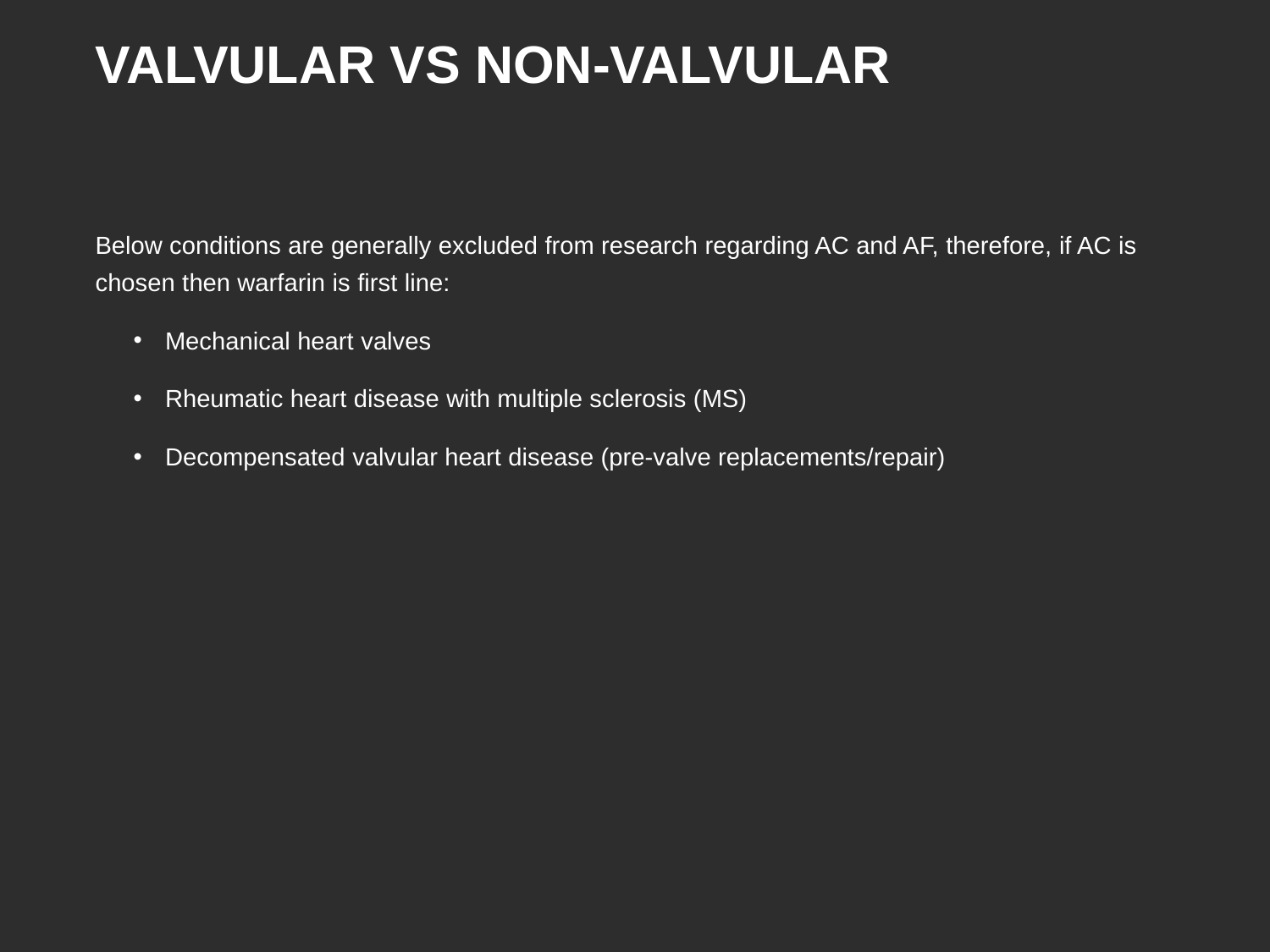

# Valvular vs Non-Valvular
Below conditions are generally excluded from research regarding AC and AF, therefore, if AC is chosen then warfarin is first line:
Mechanical heart valves
Rheumatic heart disease with multiple sclerosis (MS)
Decompensated valvular heart disease (pre-valve replacements/repair)

## Slide 13
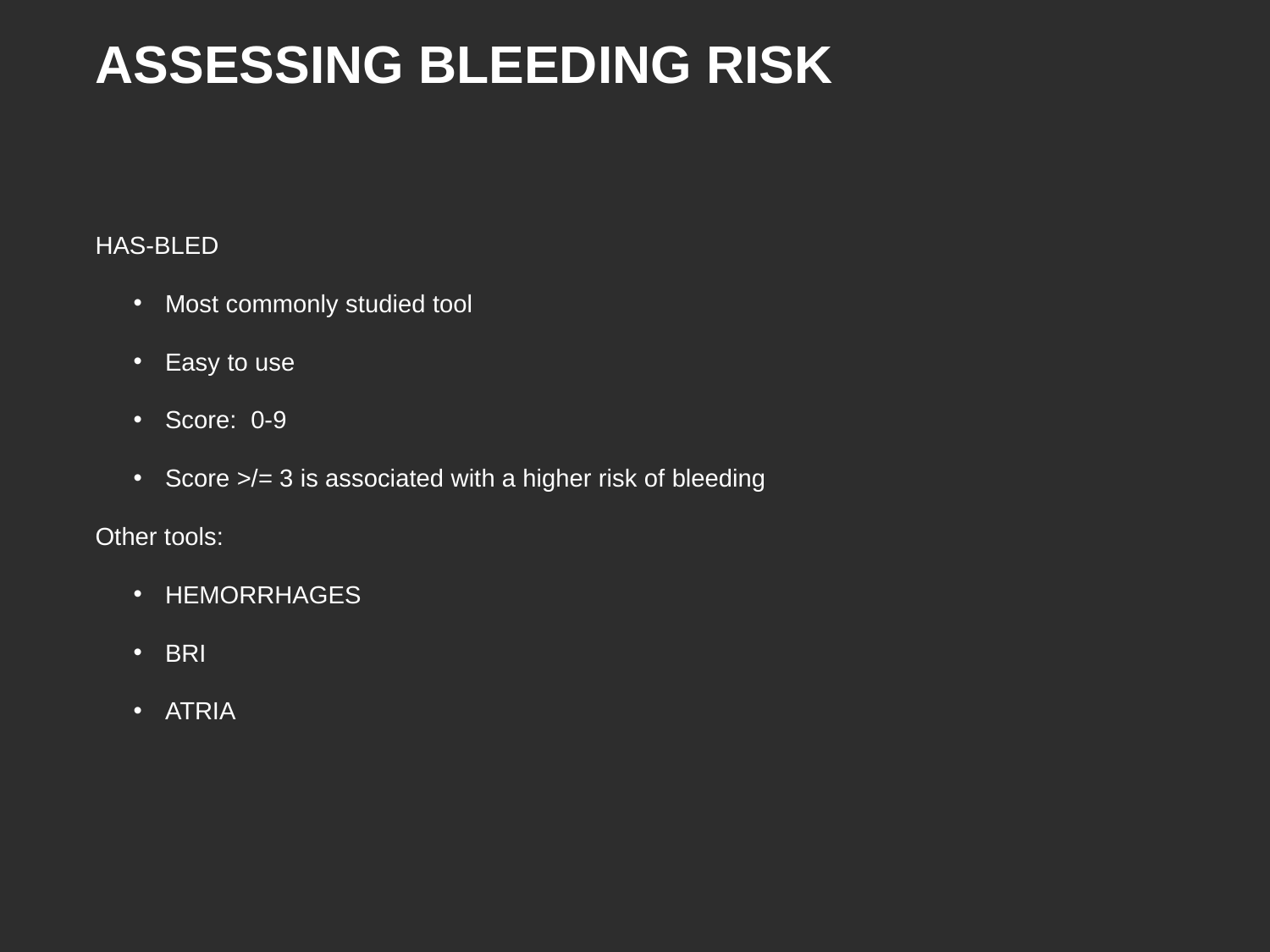

# Assessing Bleeding Risk
HAS-BLED
Most commonly studied tool
Easy to use
Score: 0-9
Score >/= 3 is associated with a higher risk of bleeding
Other tools:
HEMORRHAGES
BRI
ATRIA

## Slide 14
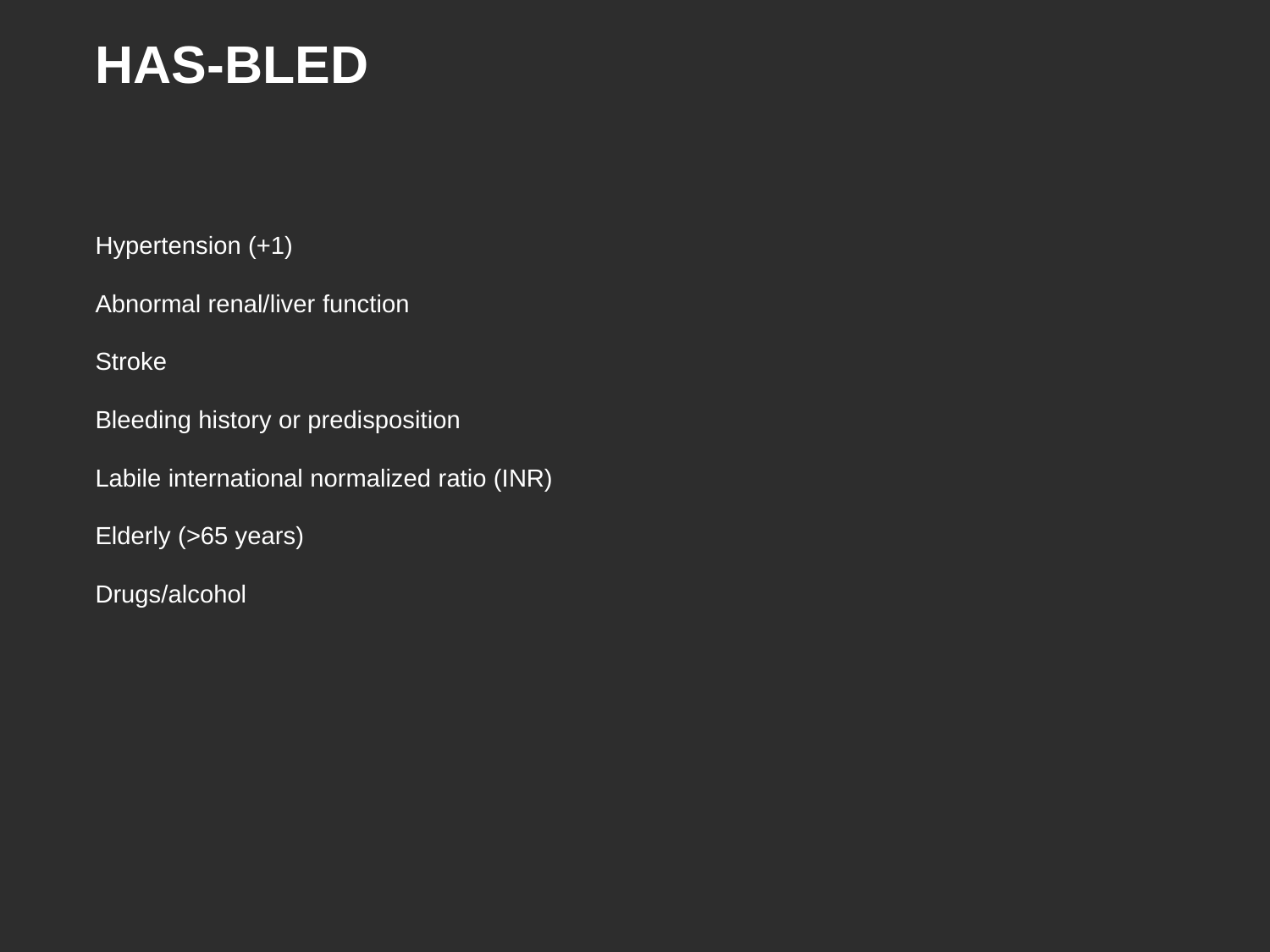

# HAS-BLED
Hypertension (+1)
Abnormal renal/liver function
Stroke
Bleeding history or predisposition
Labile international normalized ratio (INR)
Elderly (>65 years)
Drugs/alcohol

## Slide 15
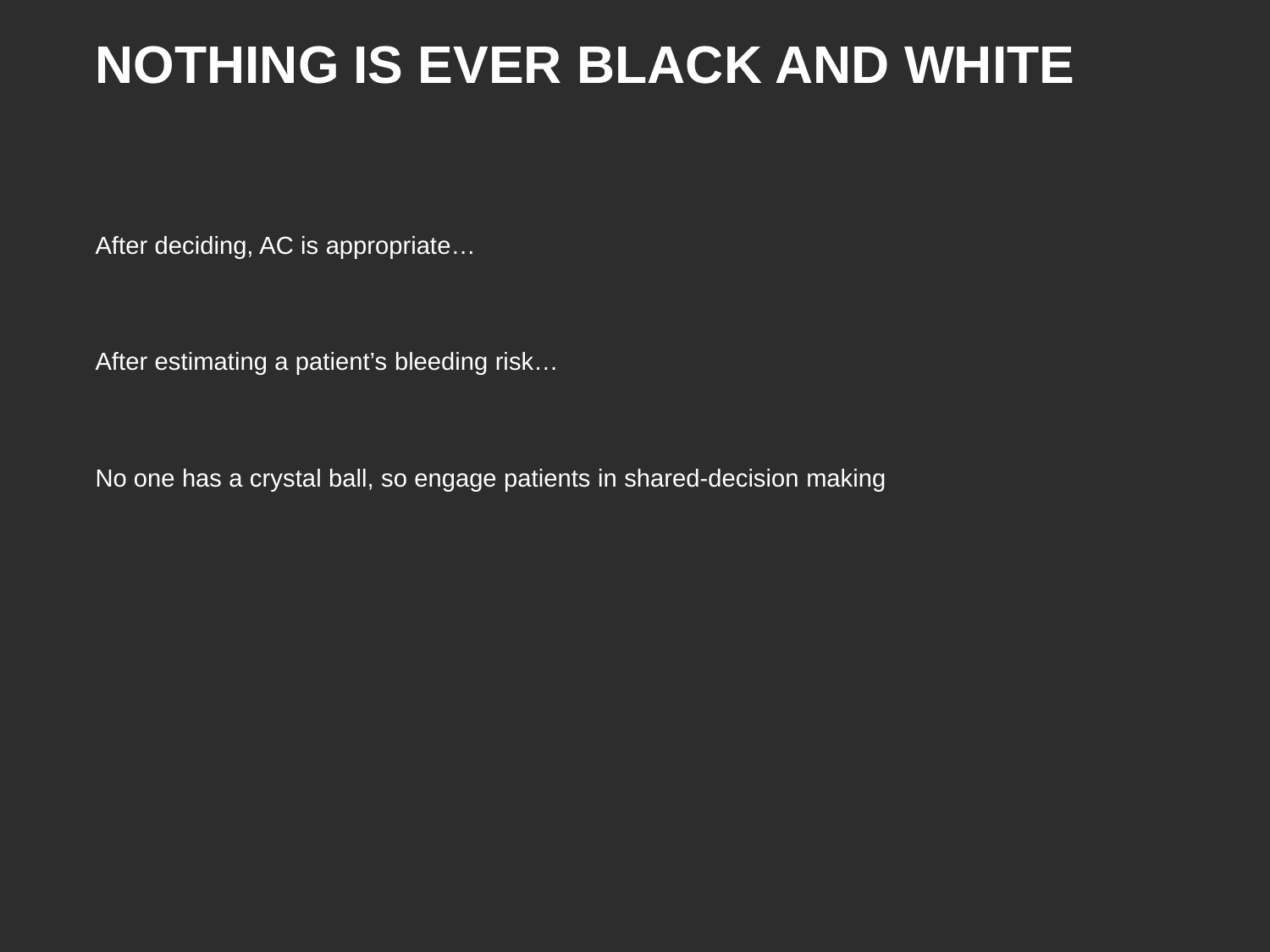

# Nothing is ever black and white
After deciding, AC is appropriate…
After estimating a patient’s bleeding risk…
No one has a crystal ball, so engage patients in shared-decision making

## Slide 16
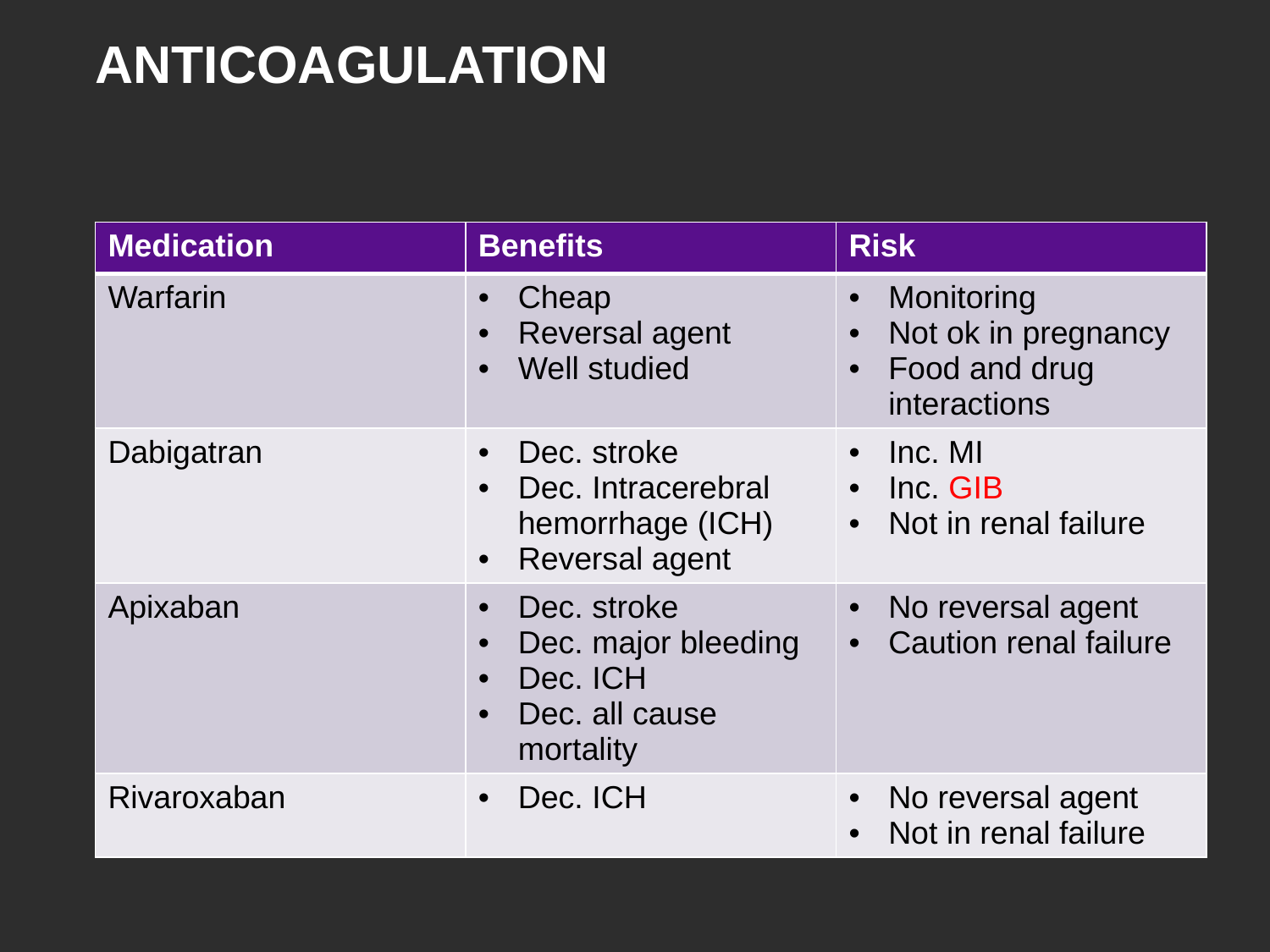

# Anticoagulation
| Medication | Benefits | Risk |
| --- | --- | --- |
| Warfarin | Cheap Reversal agent Well studied | Monitoring Not ok in pregnancy Food and drug interactions |
| Dabigatran | Dec. stroke Dec. Intracerebral hemorrhage (ICH) Reversal agent | Inc. MI Inc. GIB Not in renal failure |
| Apixaban | Dec. stroke Dec. major bleeding Dec. ICH Dec. all cause mortality | No reversal agent Caution renal failure |
| Rivaroxaban | Dec. ICH | No reversal agent Not in renal failure |

## Slide 17
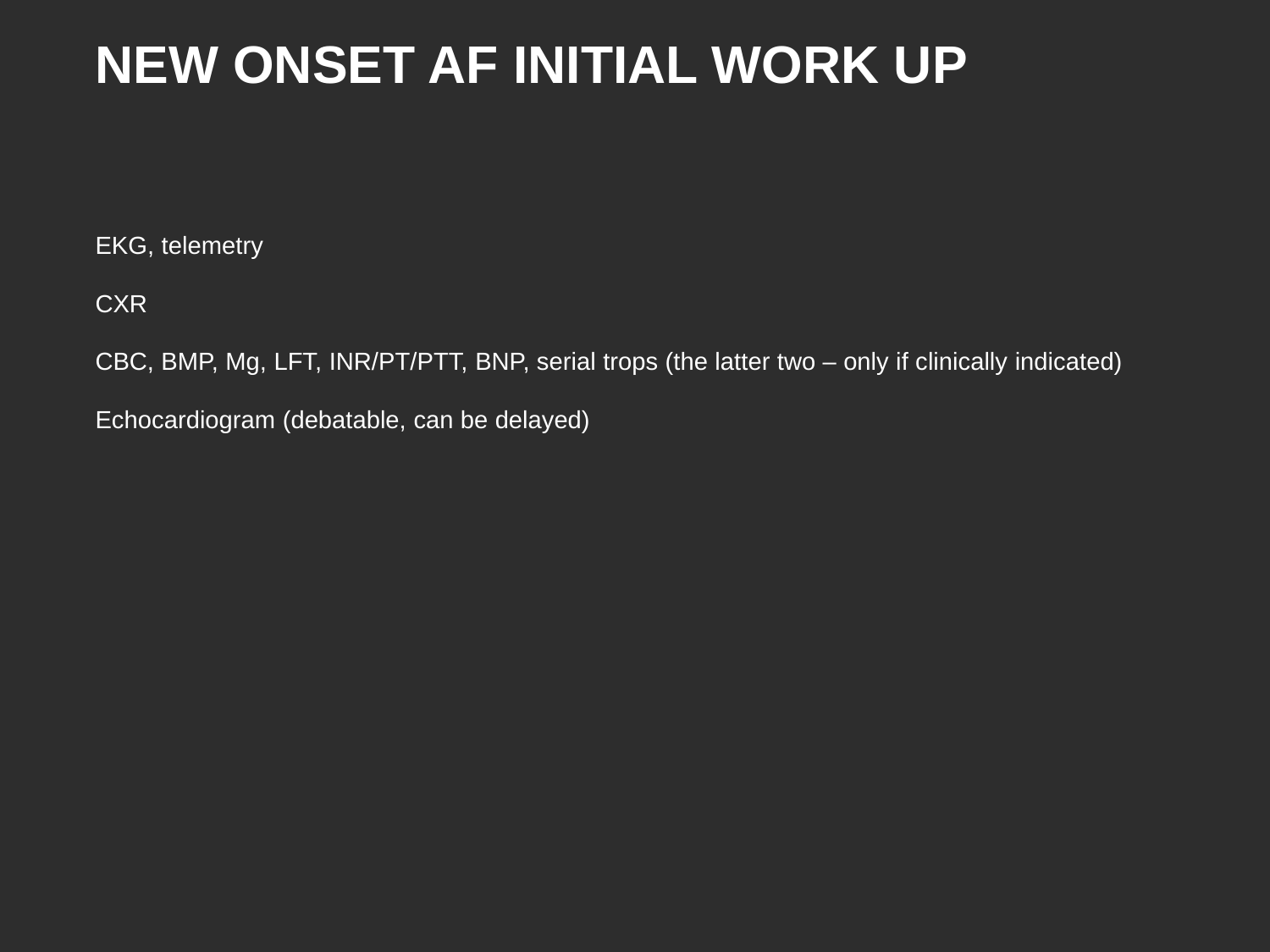

# New Onset AF Initial Work Up
EKG, telemetry
CXR
CBC, BMP, Mg, LFT, INR/PT/PTT, BNP, serial trops (the latter two – only if clinically indicated)
Echocardiogram (debatable, can be delayed)

## Slide 18
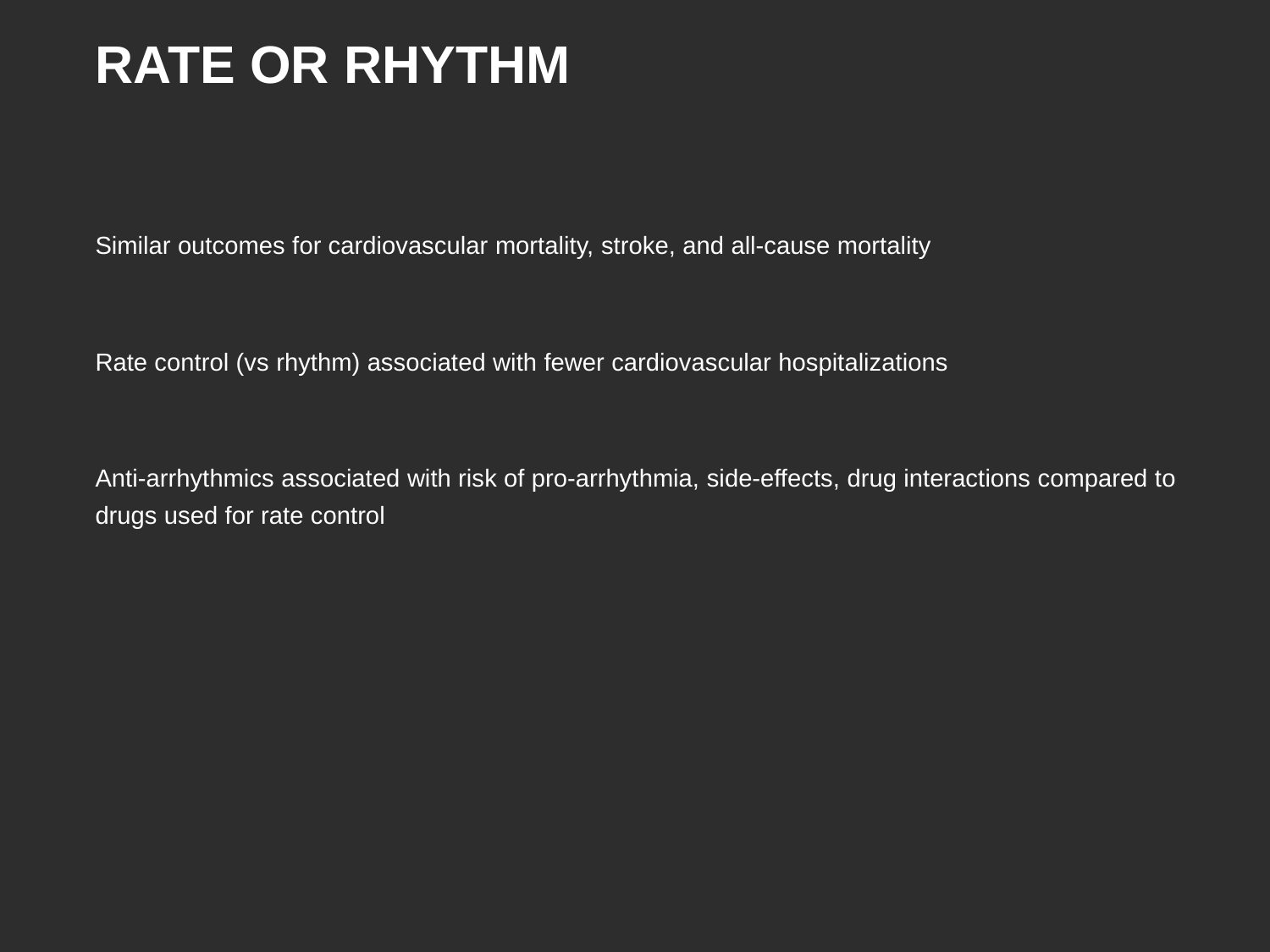

# Rate or Rhythm
Similar outcomes for cardiovascular mortality, stroke, and all-cause mortality
Rate control (vs rhythm) associated with fewer cardiovascular hospitalizations
Anti-arrhythmics associated with risk of pro-arrhythmia, side-effects, drug interactions compared to drugs used for rate control

## Slide 19
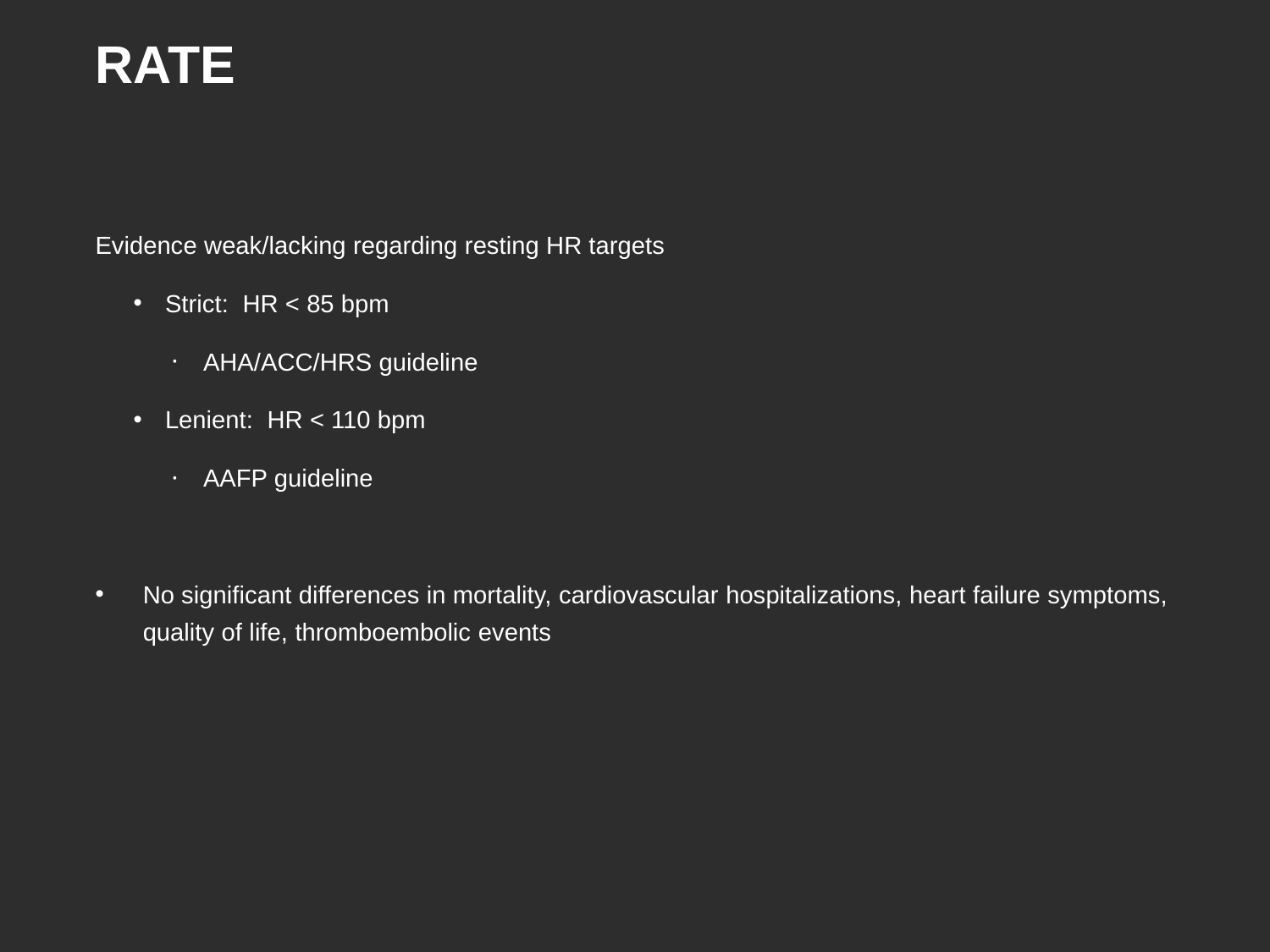

# Rate
Evidence weak/lacking regarding resting HR targets
Strict: HR < 85 bpm
AHA/ACC/HRS guideline
Lenient: HR < 110 bpm
AAFP guideline
No significant differences in mortality, cardiovascular hospitalizations, heart failure symptoms, quality of life, thromboembolic events

## Slide 20
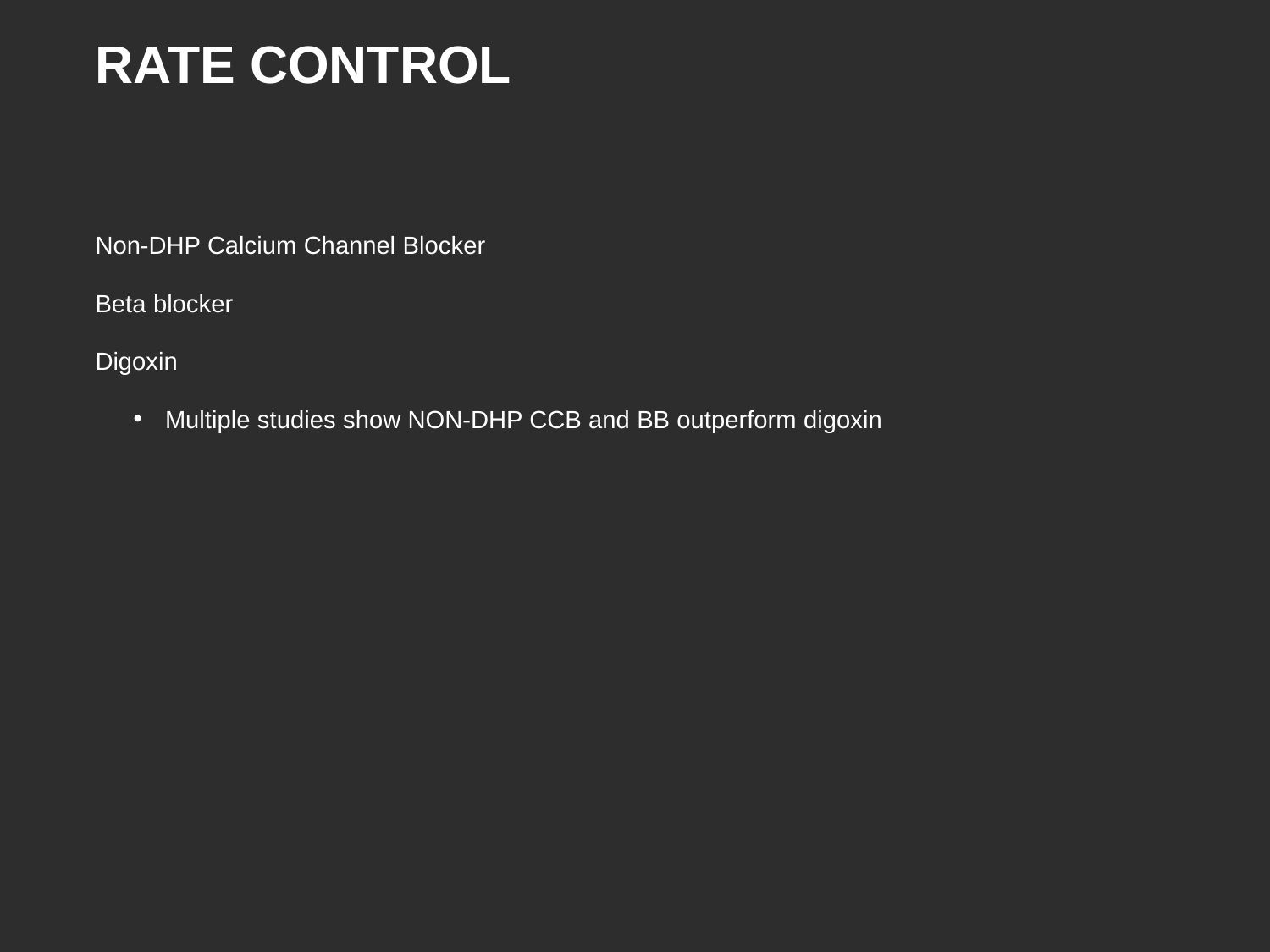

# Rate Control
Non-DHP Calcium Channel Blocker
Beta blocker
Digoxin
Multiple studies show NON-DHP CCB and BB outperform digoxin

## Slide 21
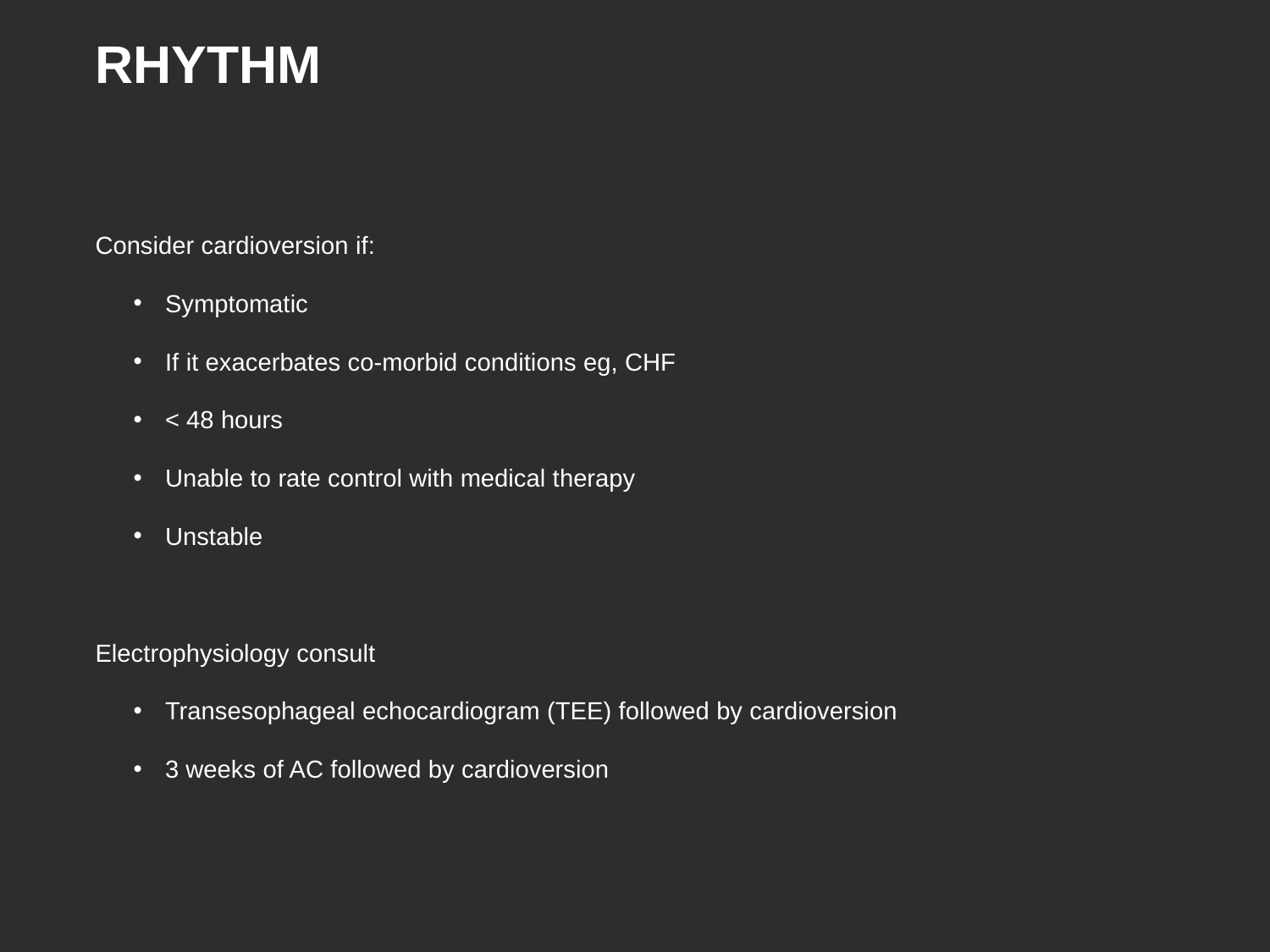

# Rhythm
Consider cardioversion if:
Symptomatic
If it exacerbates co-morbid conditions eg, CHF
< 48 hours
Unable to rate control with medical therapy
Unstable
Electrophysiology consult
Transesophageal echocardiogram (TEE) followed by cardioversion
3 weeks of AC followed by cardioversion

## Slide 22
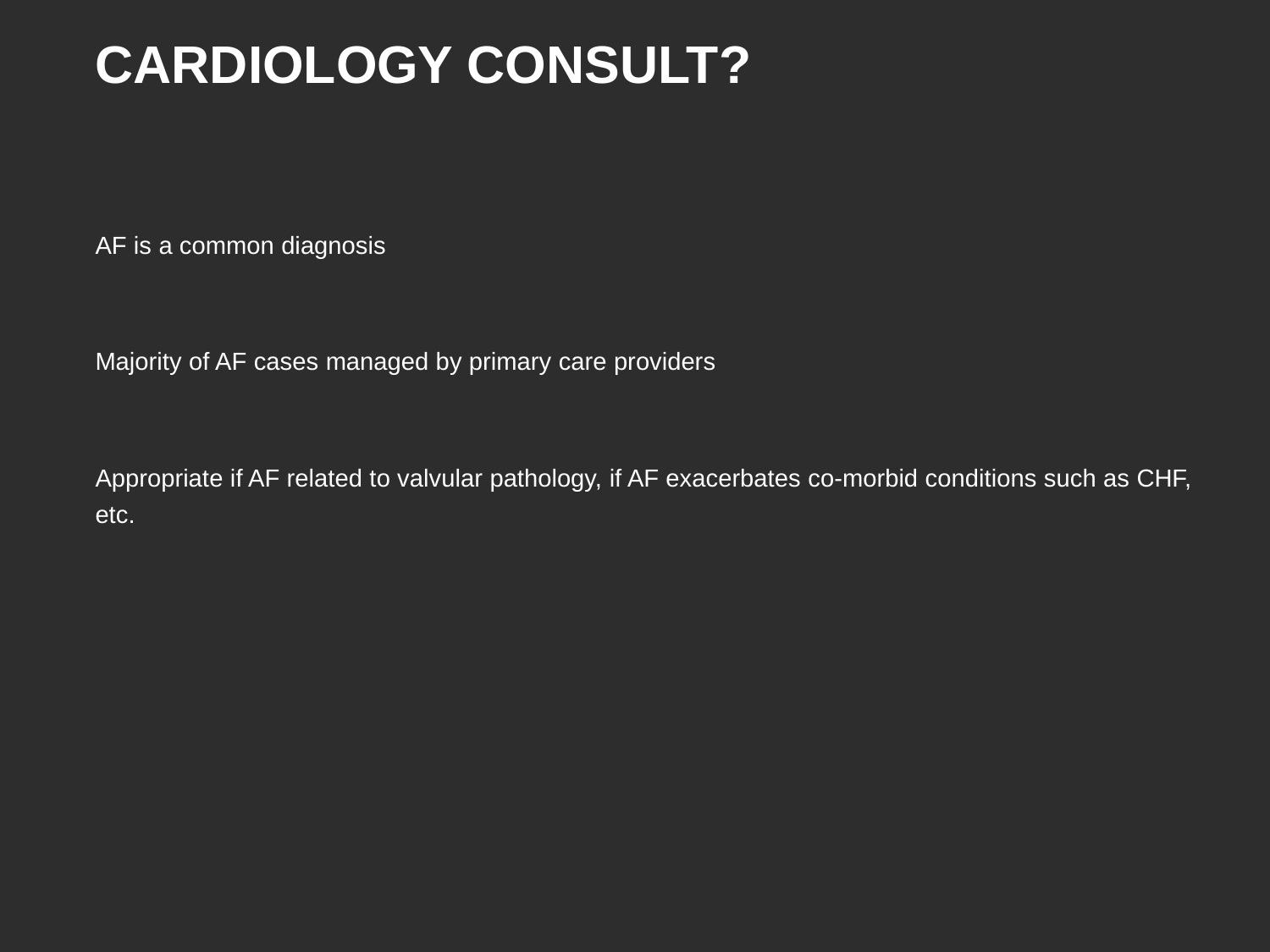

# Cardiology consult?
AF is a common diagnosis
Majority of AF cases managed by primary care providers
Appropriate if AF related to valvular pathology, if AF exacerbates co-morbid conditions such as CHF, etc.

## Slide 23
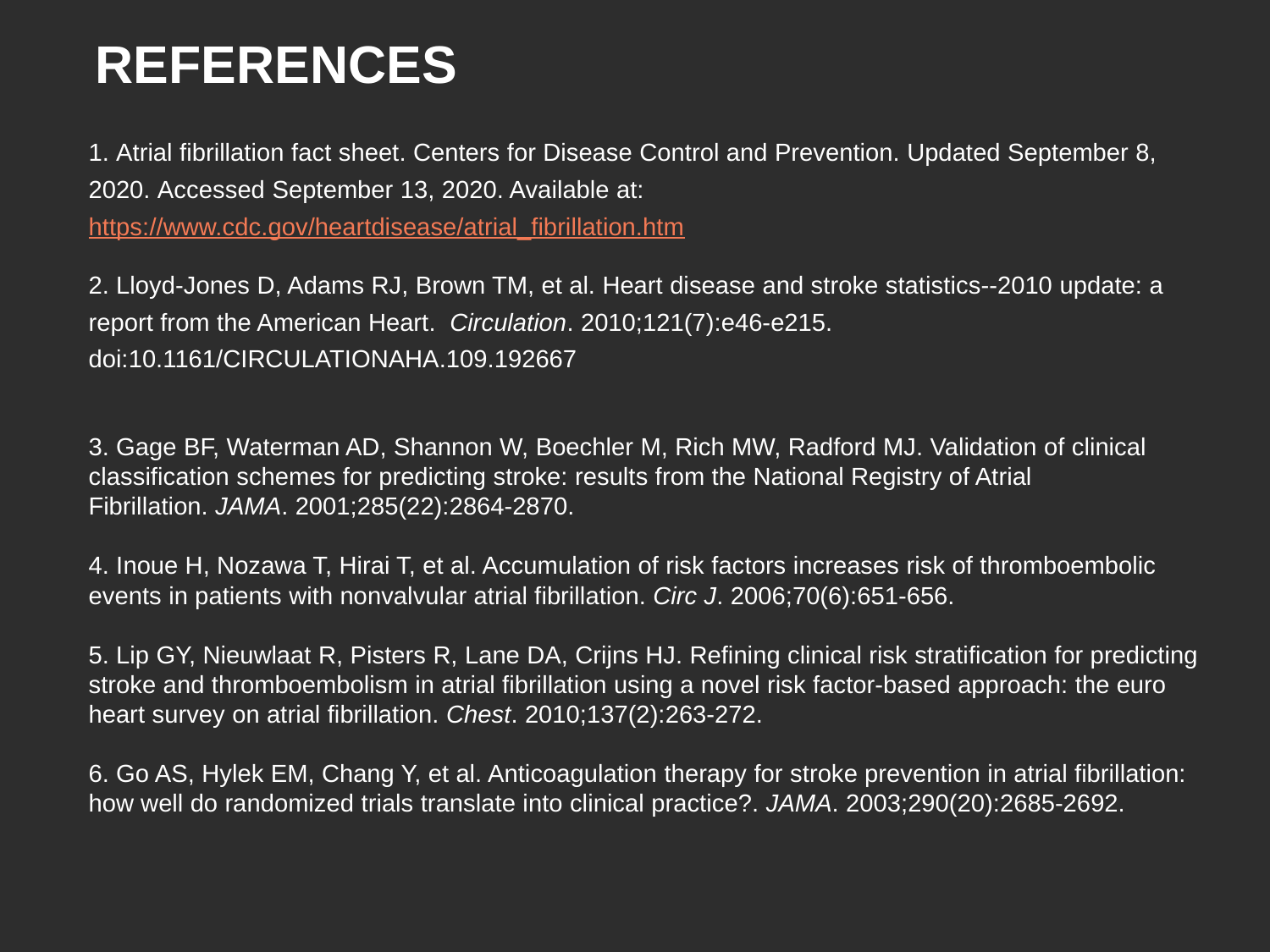

# References
1. Atrial fibrillation fact sheet. Centers for Disease Control and Prevention. Updated September 8, 2020. Accessed September 13, 2020. Available at: https://www.cdc.gov/heartdisease/atrial_fibrillation.htm
2. Lloyd-Jones D, Adams RJ, Brown TM, et al. Heart disease and stroke statistics--2010 update: a report from the American Heart. Circulation. 2010;121(7):e46-e215. doi:10.1161/CIRCULATIONAHA.109.192667
3. Gage BF, Waterman AD, Shannon W, Boechler M, Rich MW, Radford MJ. Validation of clinical classification schemes for predicting stroke: results from the National Registry of Atrial Fibrillation. JAMA. 2001;285(22):2864-2870.
4. Inoue H, Nozawa T, Hirai T, et al. Accumulation of risk factors increases risk of thromboembolic events in patients with nonvalvular atrial fibrillation. Circ J. 2006;70(6):651-656.
5. Lip GY, Nieuwlaat R, Pisters R, Lane DA, Crijns HJ. Refining clinical risk stratification for predicting stroke and thromboembolism in atrial fibrillation using a novel risk factor-based approach: the euro heart survey on atrial fibrillation. Chest. 2010;137(2):263-272.
6. Go AS, Hylek EM, Chang Y, et al. Anticoagulation therapy for stroke prevention in atrial fibrillation: how well do randomized trials translate into clinical practice?. JAMA. 2003;290(20):2685-2692.

## Slide 24
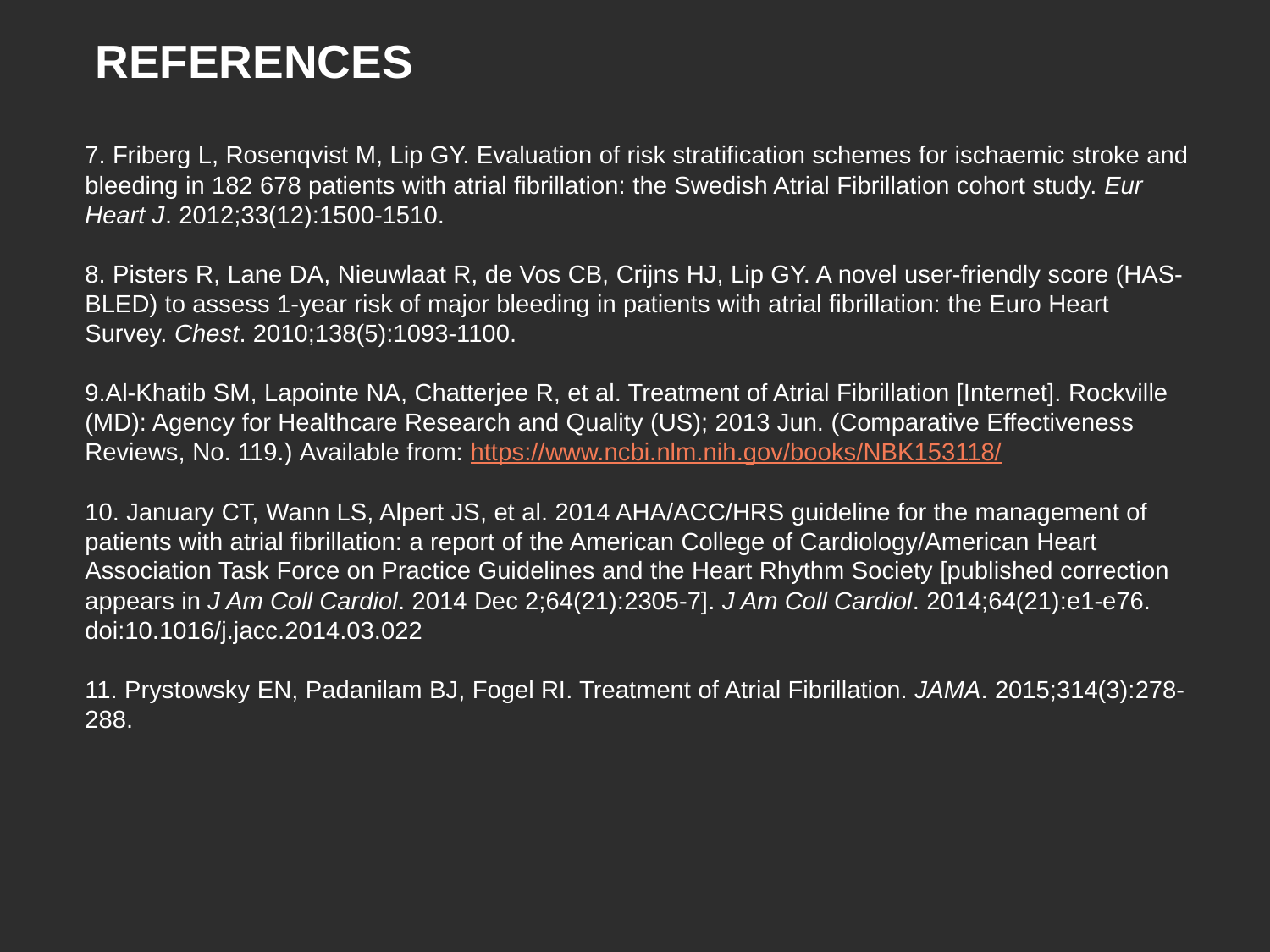

# References
7. Friberg L, Rosenqvist M, Lip GY. Evaluation of risk stratification schemes for ischaemic stroke and bleeding in 182 678 patients with atrial fibrillation: the Swedish Atrial Fibrillation cohort study. Eur Heart J. 2012;33(12):1500-1510.
8. Pisters R, Lane DA, Nieuwlaat R, de Vos CB, Crijns HJ, Lip GY. A novel user-friendly score (HAS-BLED) to assess 1-year risk of major bleeding in patients with atrial fibrillation: the Euro Heart Survey. Chest. 2010;138(5):1093-1100.
9.Al-Khatib SM, Lapointe NA, Chatterjee R, et al. Treatment of Atrial Fibrillation [Internet]. Rockville (MD): Agency for Healthcare Research and Quality (US); 2013 Jun. (Comparative Effectiveness Reviews, No. 119.) Available from: https://www.ncbi.nlm.nih.gov/books/NBK153118/
10. January CT, Wann LS, Alpert JS, et al. 2014 AHA/ACC/HRS guideline for the management of patients with atrial fibrillation: a report of the American College of Cardiology/American Heart Association Task Force on Practice Guidelines and the Heart Rhythm Society [published correction appears in J Am Coll Cardiol. 2014 Dec 2;64(21):2305-7]. J Am Coll Cardiol. 2014;64(21):e1-e76. doi:10.1016/j.jacc.2014.03.022
11. Prystowsky EN, Padanilam BJ, Fogel RI. Treatment of Atrial Fibrillation. JAMA. 2015;314(3):278-288.
