# Supplementary material for: An Observation Medicine Curriculum for Emergency Medicine Education
Source: J Educ Teach Emerg Med. 2021 Apr 19;6(2):C1–C72. doi: 10.21980/J87P92 (PMC10332786; doi:10.21980/J87P92)
Supplement: Supplementary file 10 — Please see associated PowerPoint file [file jetem-6-2-c1-supp10.pptx]

## Slide 1
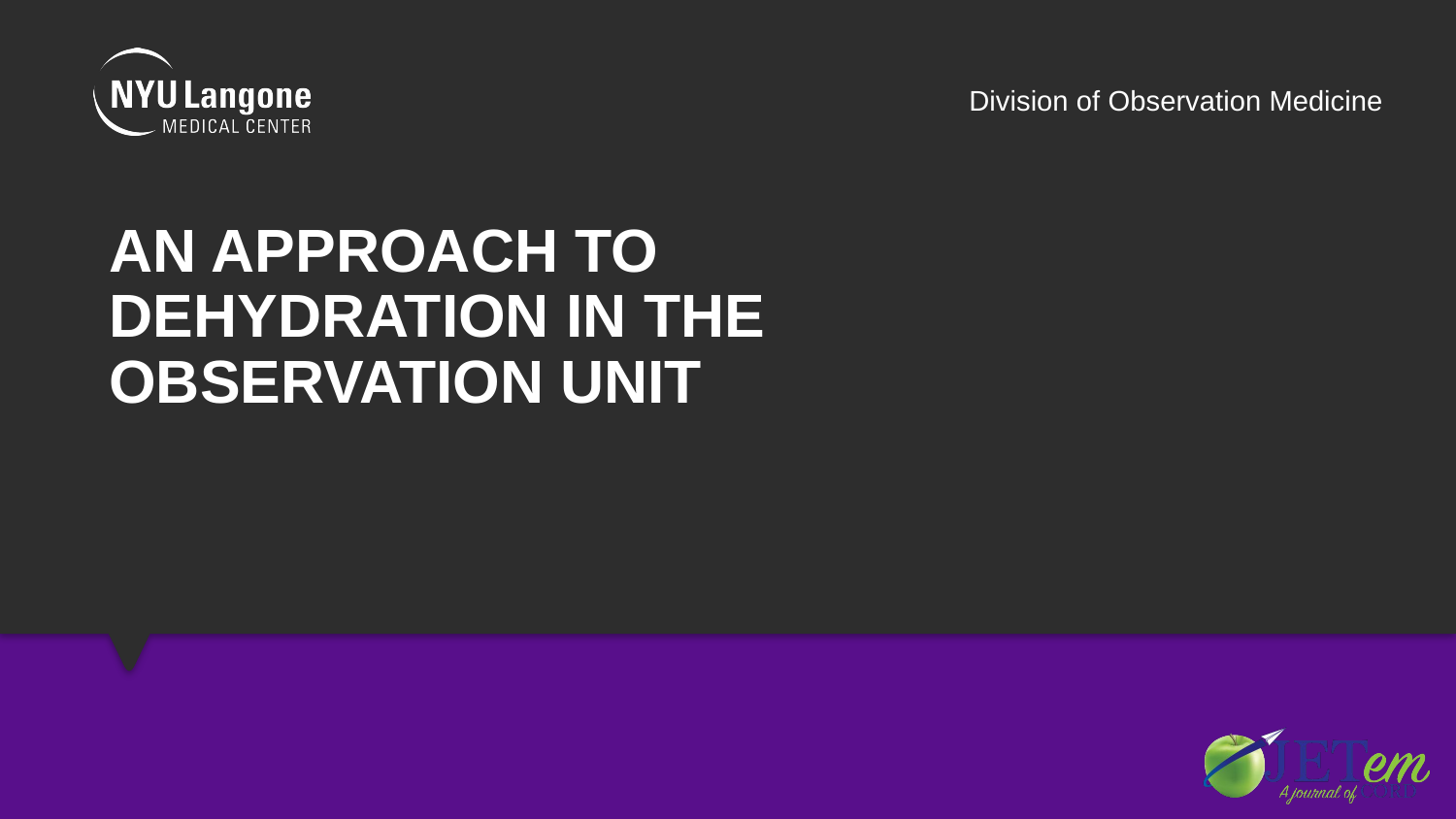

Division of Observation Medicine
# AN APPROACH TO DEHYDRATION in the observation unit

## Slide 2
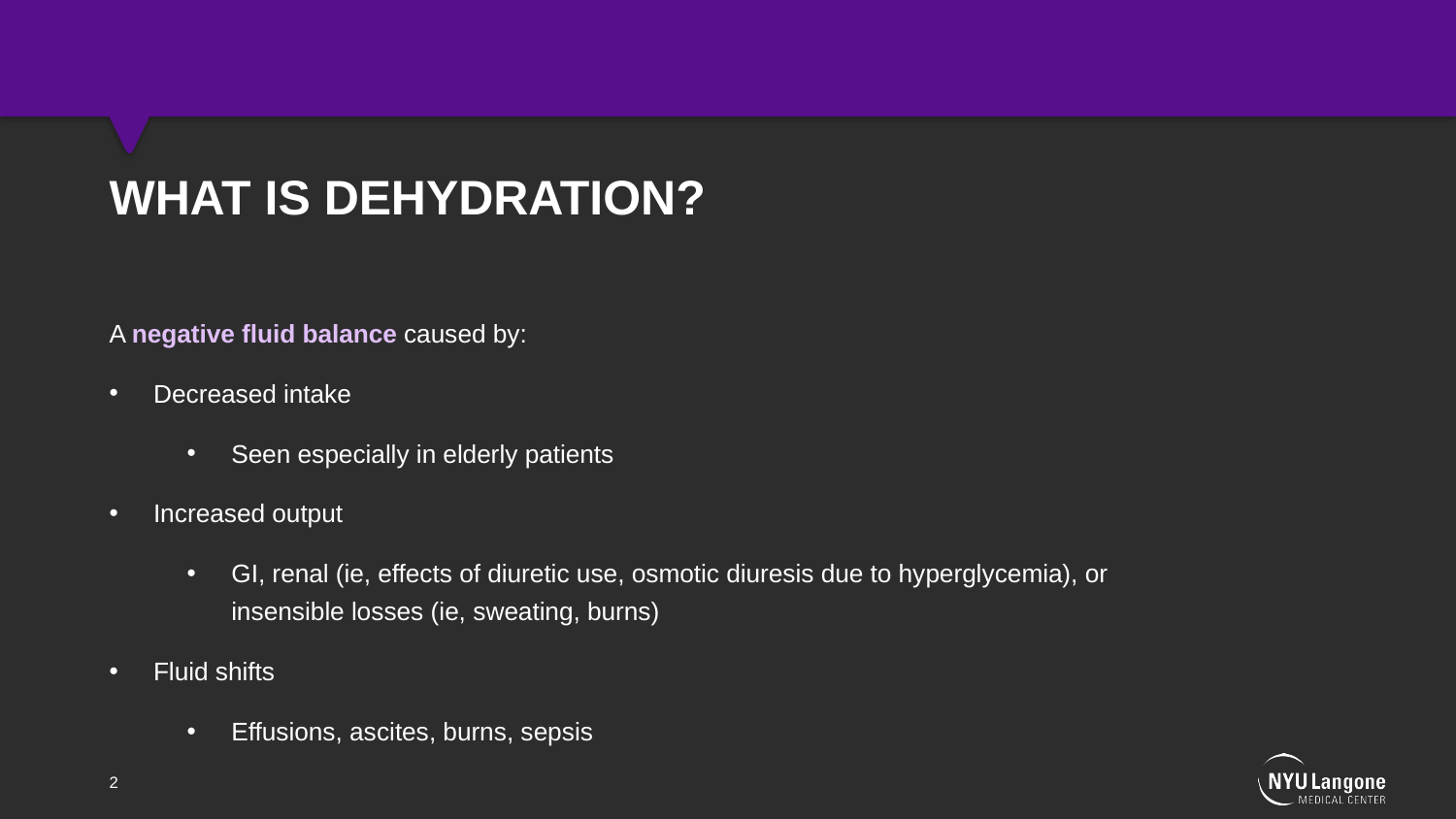

# WHAT IS DEHYDRATION?
A negative fluid balance caused by:
Decreased intake
Seen especially in elderly patients
Increased output
GI, renal (ie, effects of diuretic use, osmotic diuresis due to hyperglycemia), or insensible losses (ie, sweating, burns)
Fluid shifts
Effusions, ascites, burns, sepsis
2

## Slide 3
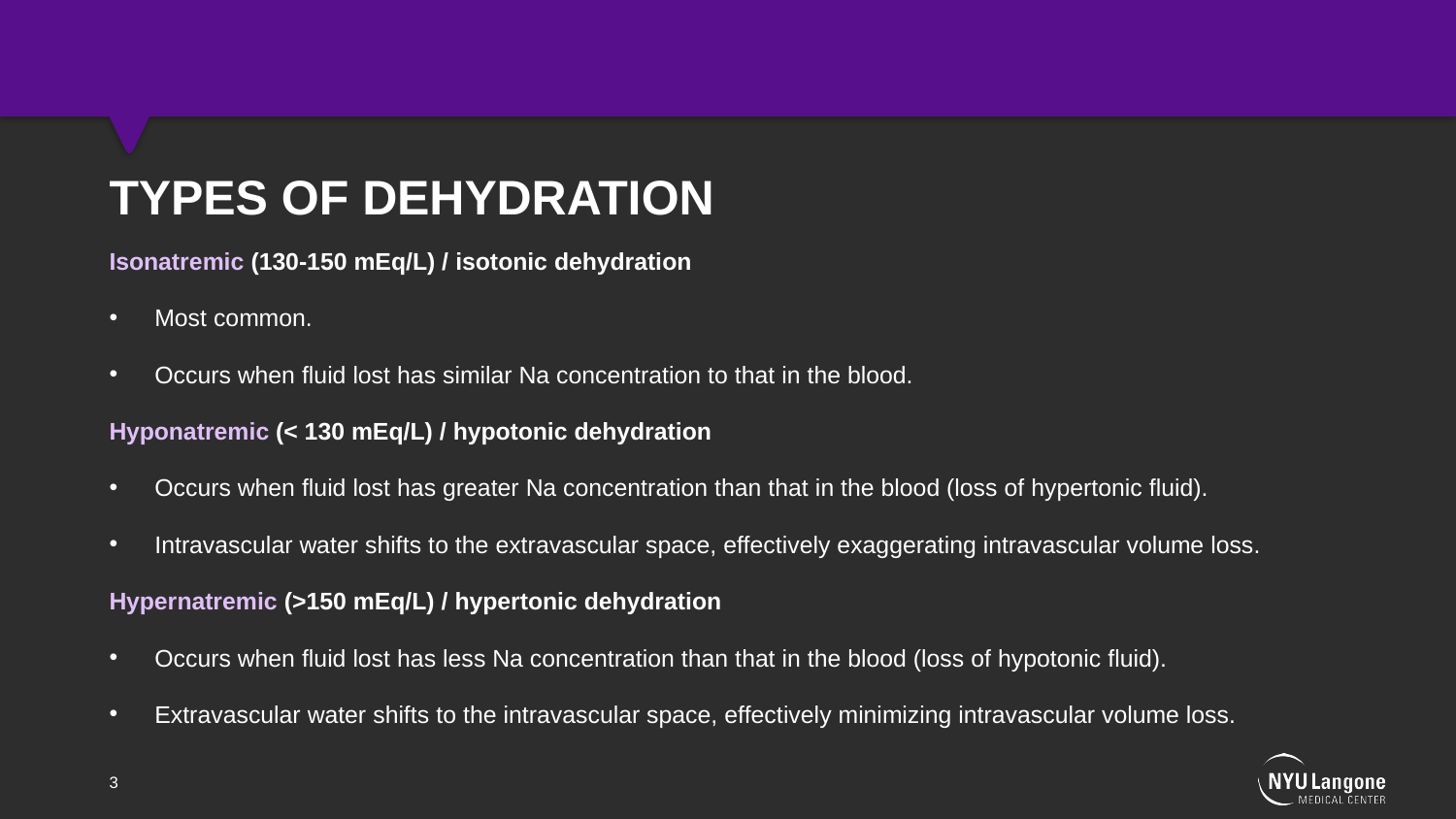

# TYPES OF DEHYDRATION
Isonatremic (130-150 mEq/L) / isotonic dehydration
Most common.
Occurs when fluid lost has similar Na concentration to that in the blood.
Hyponatremic (< 130 mEq/L) / hypotonic dehydration
Occurs when fluid lost has greater Na concentration than that in the blood (loss of hypertonic fluid).
Intravascular water shifts to the extravascular space, effectively exaggerating intravascular volume loss.
Hypernatremic (>150 mEq/L) / hypertonic dehydration
Occurs when fluid lost has less Na concentration than that in the blood (loss of hypotonic fluid).
Extravascular water shifts to the intravascular space, effectively minimizing intravascular volume loss.
3

## Slide 4
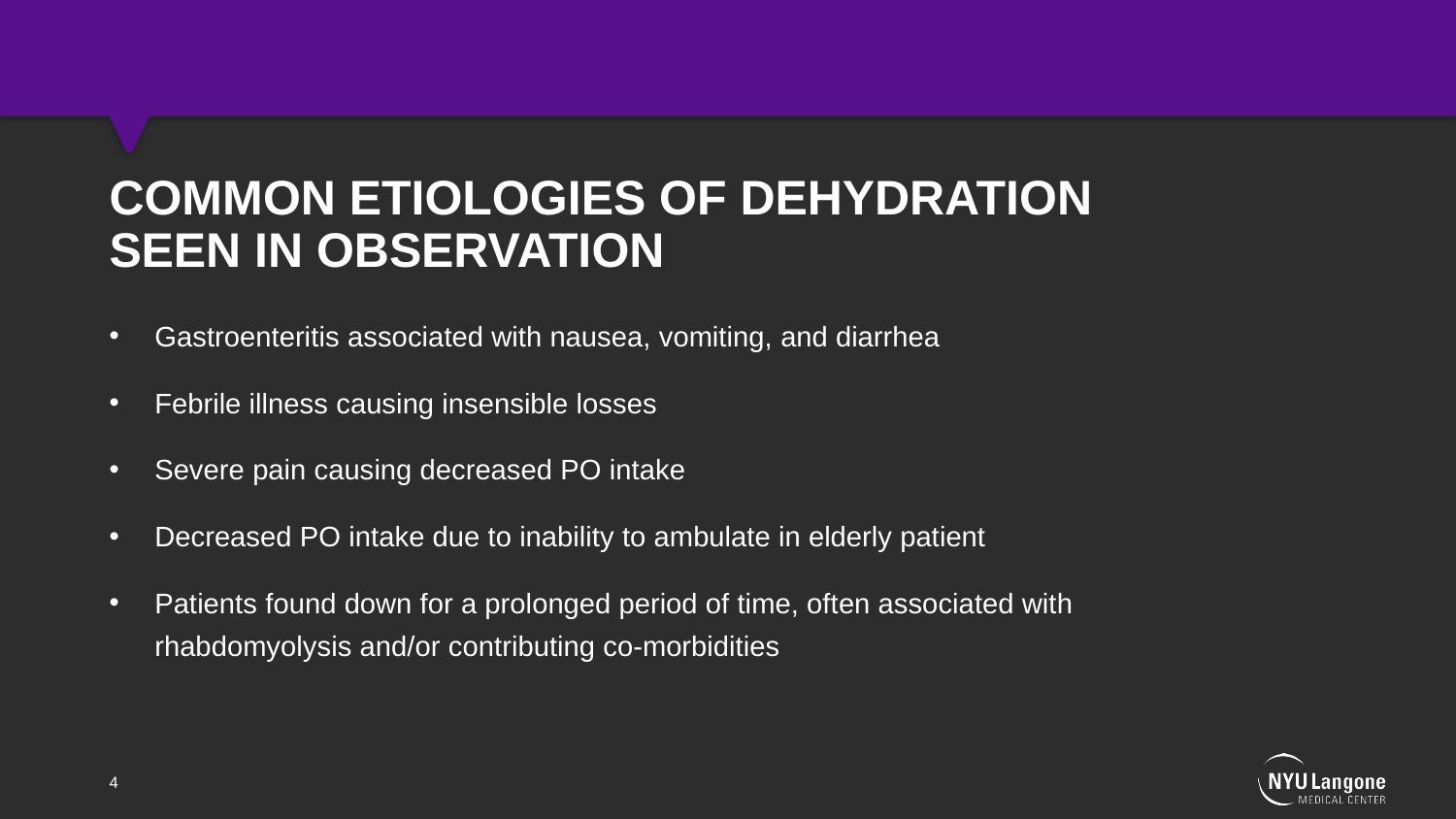

# COMMON ETIOLOGIES OF DEHYDRATION SEEN IN OBSERVATION
Gastroenteritis associated with nausea, vomiting, and diarrhea
Febrile illness causing insensible losses
Severe pain causing decreased PO intake
Decreased PO intake due to inability to ambulate in elderly patient
Patients found down for a prolonged period of time, often associated with rhabdomyolysis and/or contributing co-morbidities
4

## Slide 5
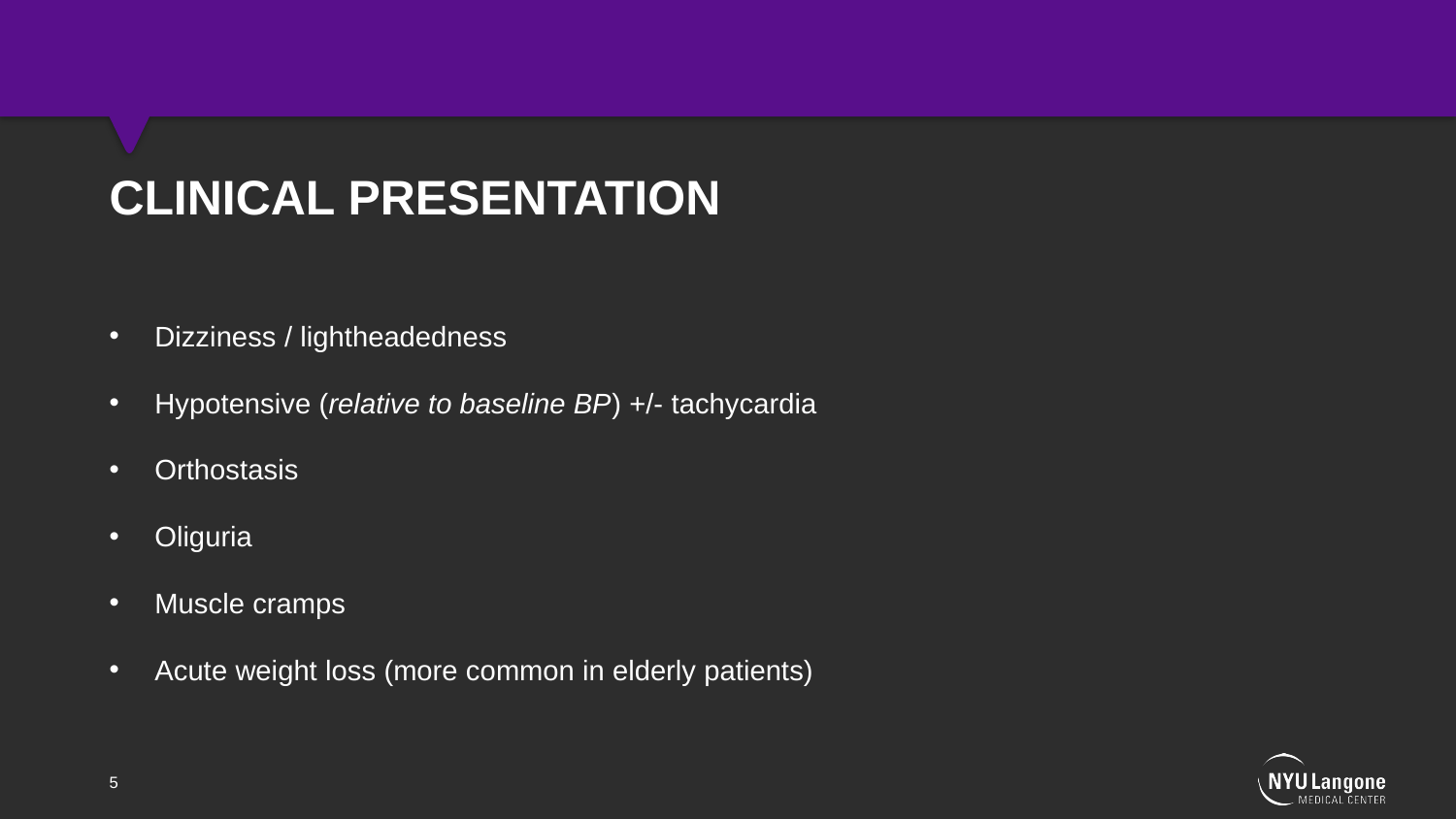

# CLINICAL PRESENTATION
Dizziness / lightheadedness
Hypotensive (relative to baseline BP) +/- tachycardia
Orthostasis
Oliguria
Muscle cramps
Acute weight loss (more common in elderly patients)
5

## Slide 6
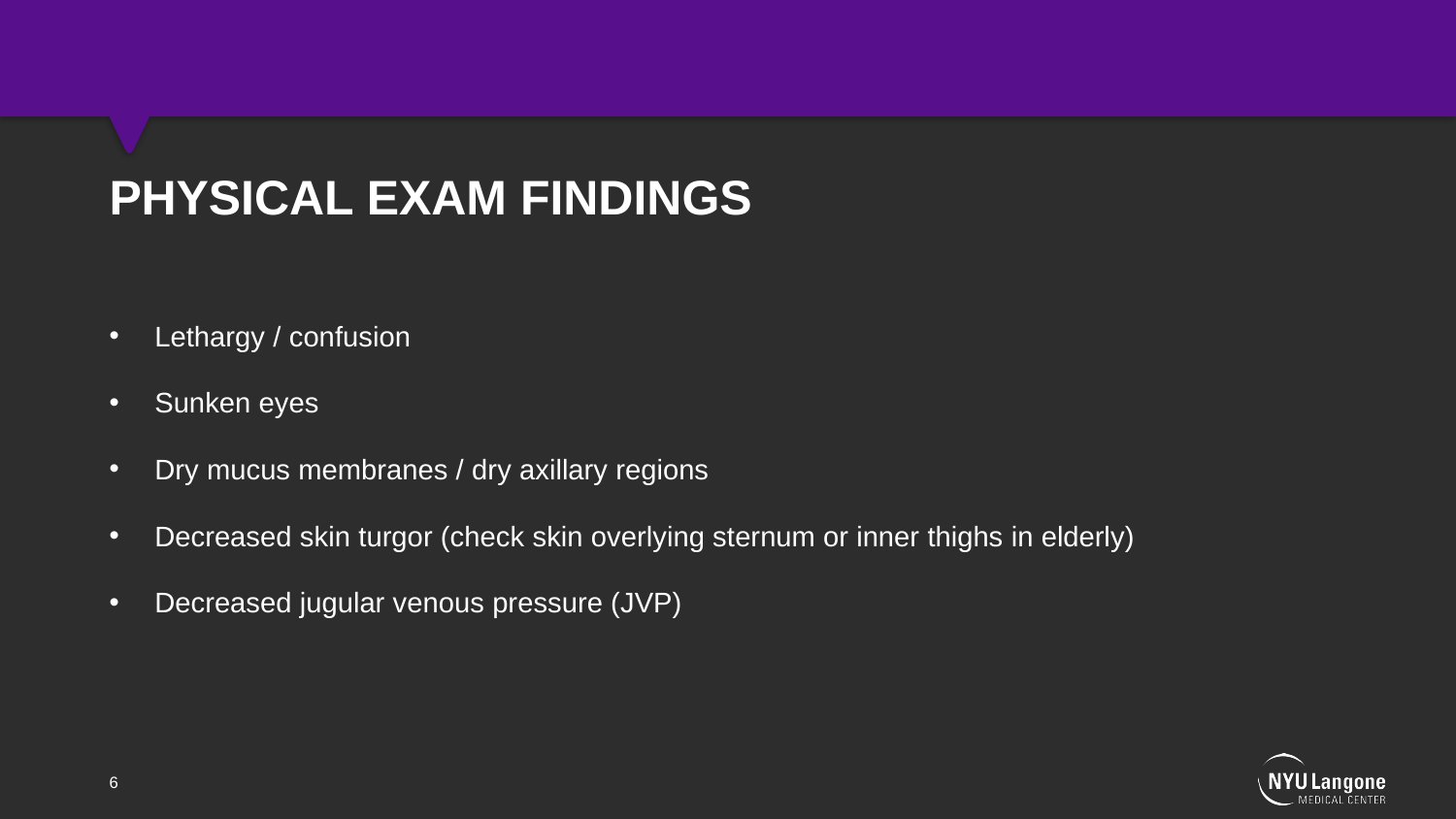

# PHYSICAL EXAM FINDINGS
Lethargy / confusion
Sunken eyes
Dry mucus membranes / dry axillary regions
Decreased skin turgor (check skin overlying sternum or inner thighs in elderly)
Decreased jugular venous pressure (JVP)
6

## Slide 7
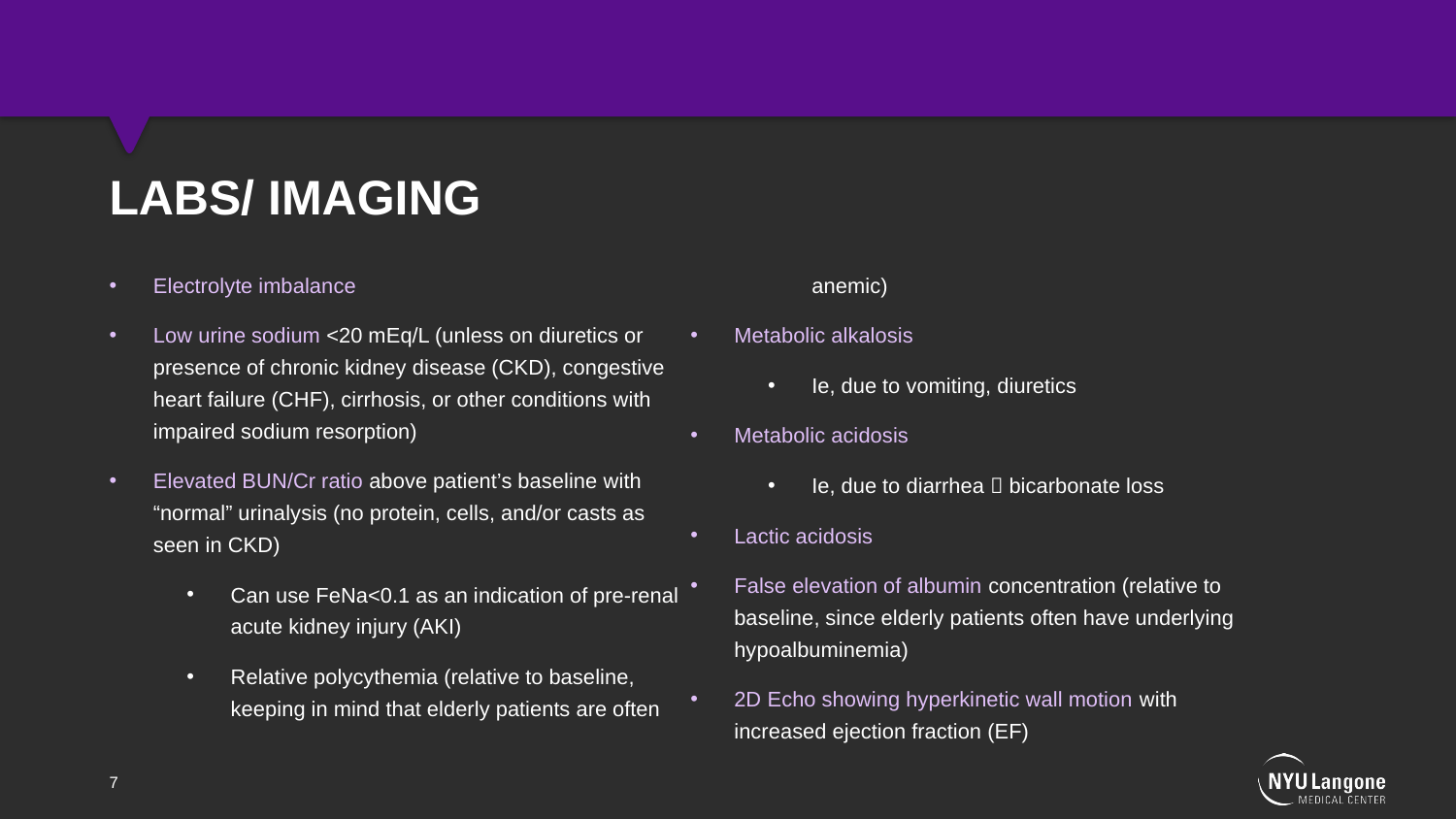

# LABS/ IMAGING
Electrolyte imbalance
Low urine sodium <20 mEq/L (unless on diuretics or presence of chronic kidney disease (CKD), congestive heart failure (CHF), cirrhosis, or other conditions with impaired sodium resorption)
Elevated BUN/Cr ratio above patient’s baseline with “normal” urinalysis (no protein, cells, and/or casts as seen in CKD)
Can use FeNa<0.1 as an indication of pre-renal acute kidney injury (AKI)
Relative polycythemia (relative to baseline, keeping in mind that elderly patients are often anemic)
Metabolic alkalosis
Ie, due to vomiting, diuretics
Metabolic acidosis
Ie, due to diarrhea  bicarbonate loss
Lactic acidosis
False elevation of albumin concentration (relative to baseline, since elderly patients often have underlying hypoalbuminemia)
2D Echo showing hyperkinetic wall motion with increased ejection fraction (EF)
7

## Slide 8
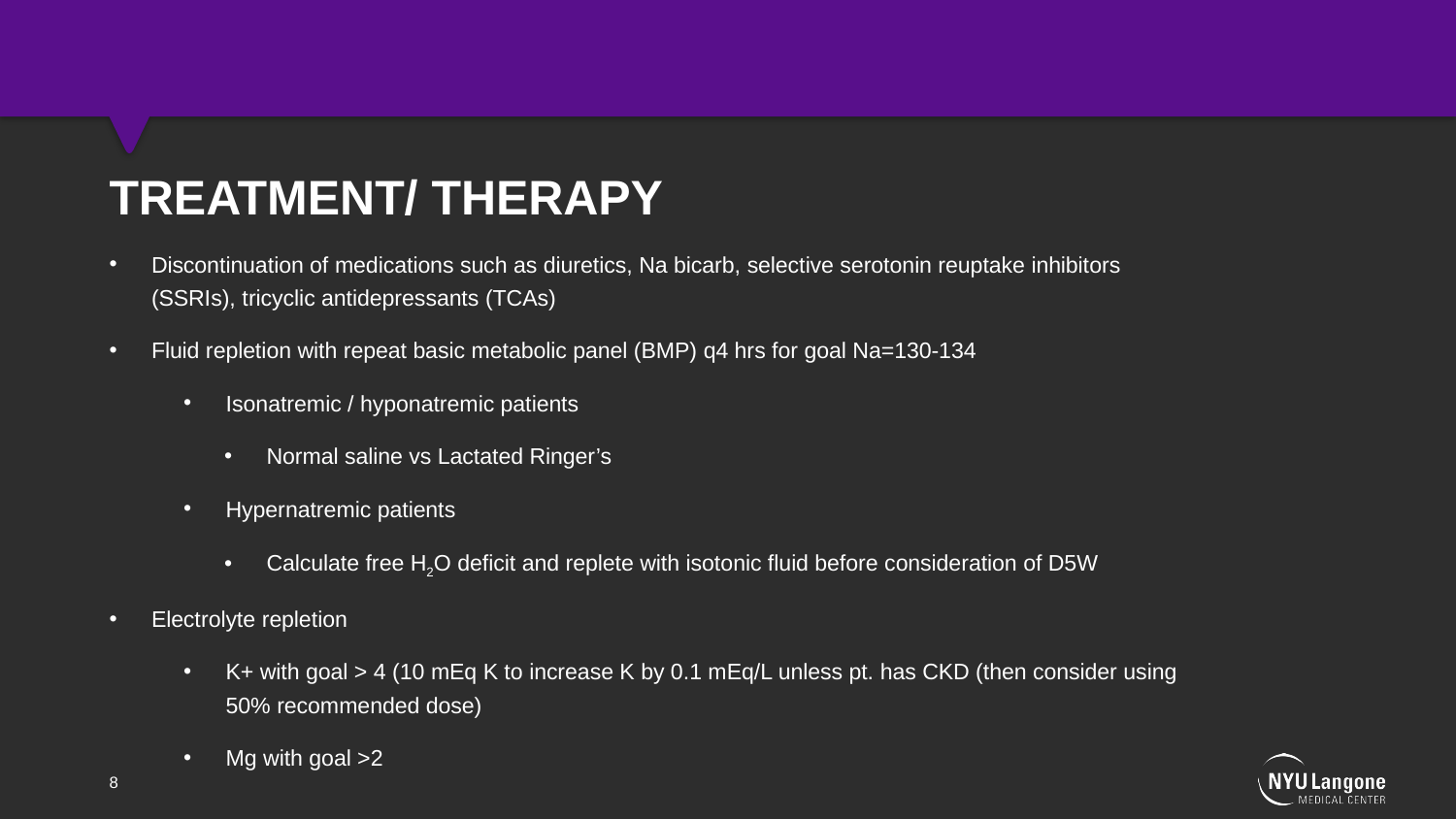

# TREATMENT/ THERAPY
Discontinuation of medications such as diuretics, Na bicarb, selective serotonin reuptake inhibitors (SSRIs), tricyclic antidepressants (TCAs)
Fluid repletion with repeat basic metabolic panel (BMP) q4 hrs for goal Na=130-134
Isonatremic / hyponatremic patients
Normal saline vs Lactated Ringer’s
Hypernatremic patients
Calculate free H2O deficit and replete with isotonic fluid before consideration of D5W
Electrolyte repletion
K+ with goal > 4 (10 mEq K to increase K by 0.1 mEq/L unless pt. has CKD (then consider using 50% recommended dose)
Mg with goal >2
8

## Slide 9
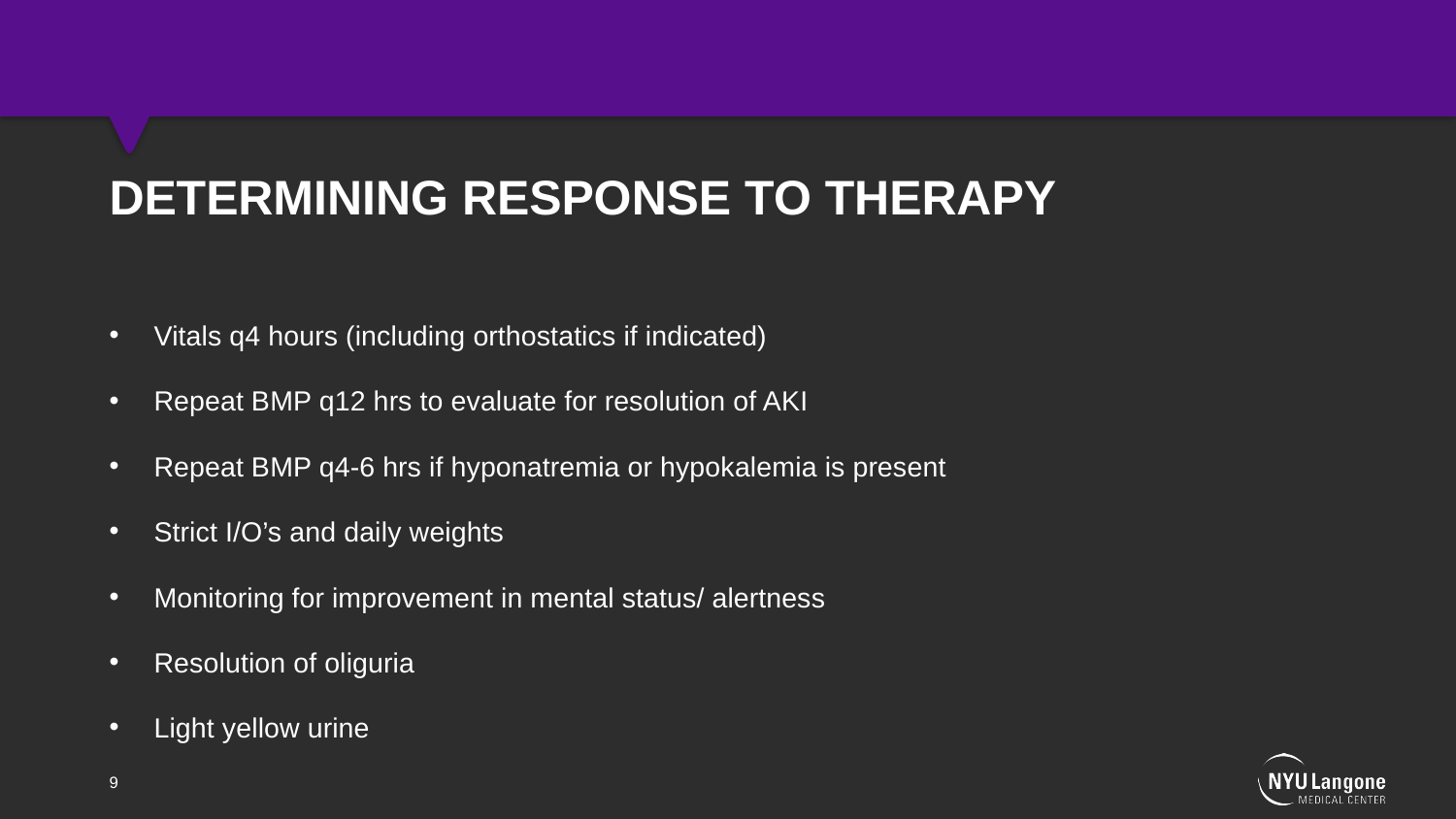

# DETERMINING RESPONSE TO THERAPY
Vitals q4 hours (including orthostatics if indicated)
Repeat BMP q12 hrs to evaluate for resolution of AKI
Repeat BMP q4-6 hrs if hyponatremia or hypokalemia is present
Strict I/O’s and daily weights
Monitoring for improvement in mental status/ alertness
Resolution of oliguria
Light yellow urine
9

## Slide 10
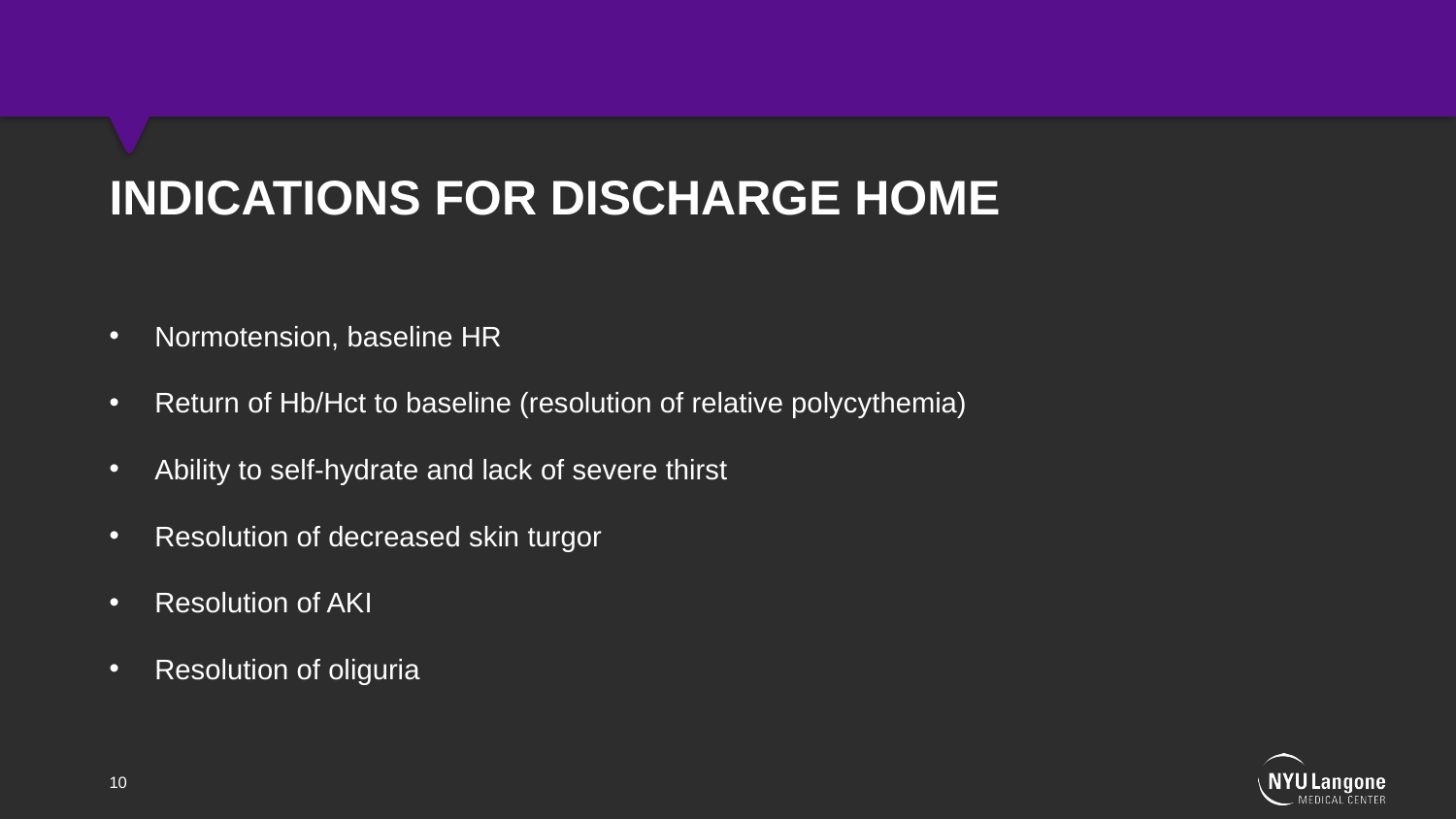

# INDICATIONS FOR DISCHARGE HOME
Normotension, baseline HR
Return of Hb/Hct to baseline (resolution of relative polycythemia)
Ability to self-hydrate and lack of severe thirst
Resolution of decreased skin turgor
Resolution of AKI
Resolution of oliguria
10

## Slide 11
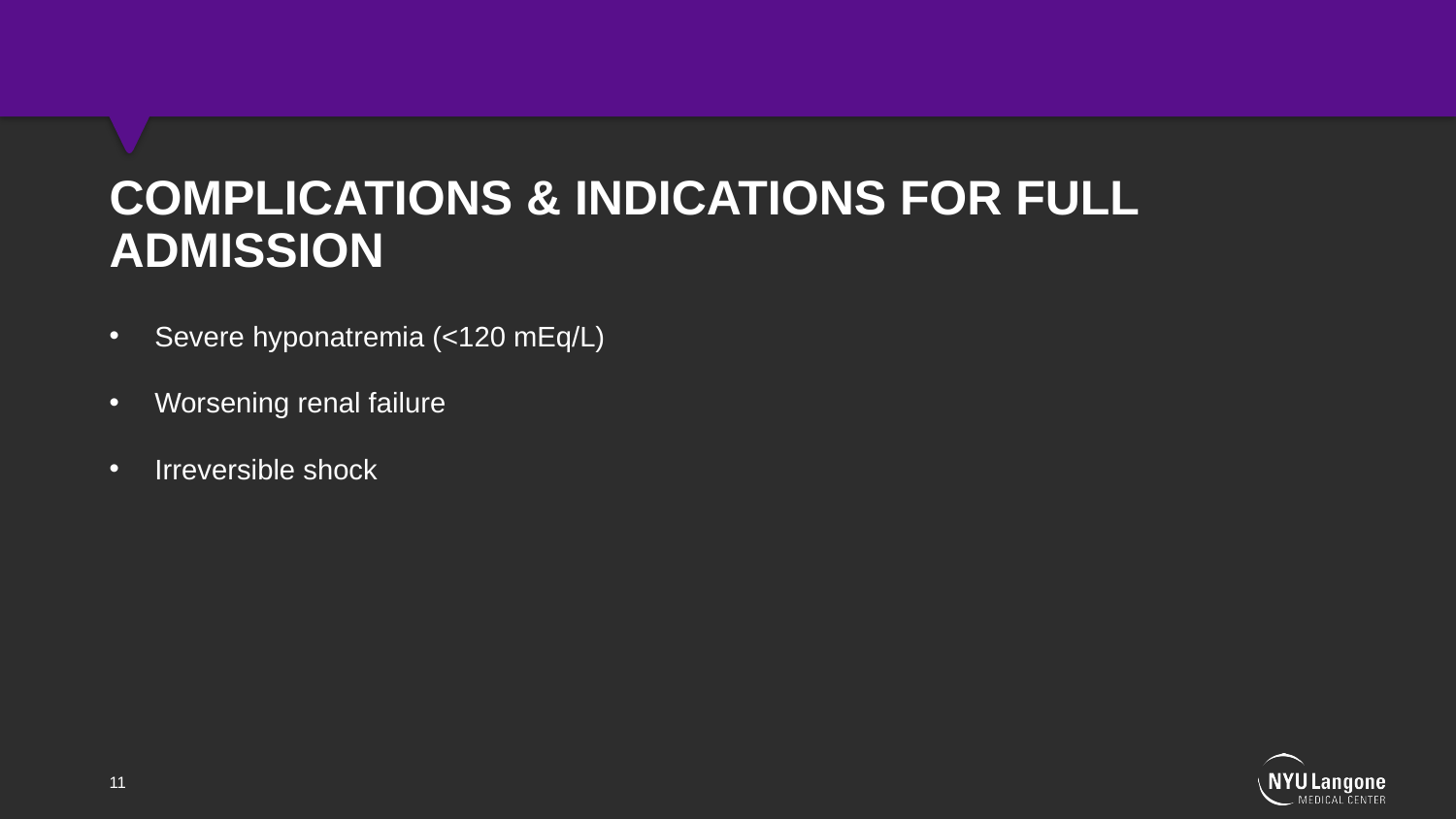

# COMPLICATIONS & INDICATIONS FOR FULL ADMISSION
Severe hyponatremia (<120 mEq/L)
Worsening renal failure
Irreversible shock
11

## Slide 12
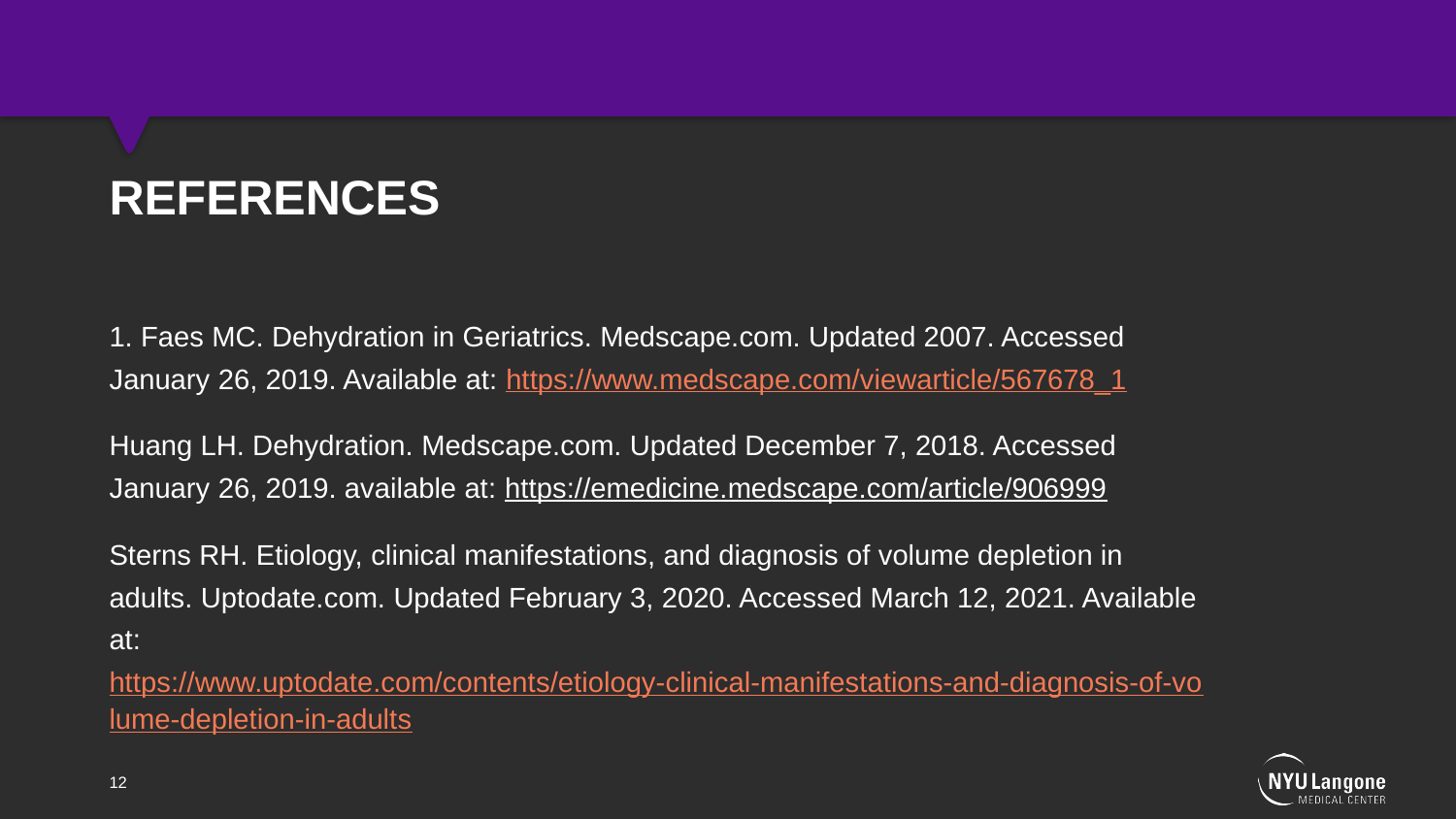

# REFERENCES
1. Faes MC. Dehydration in Geriatrics. Medscape.com. Updated 2007. Accessed January 26, 2019. Available at: https://www.medscape.com/viewarticle/567678_1
Huang LH. Dehydration. Medscape.com. Updated December 7, 2018. Accessed January 26, 2019. available at: https://emedicine.medscape.com/article/906999
Sterns RH. Etiology, clinical manifestations, and diagnosis of volume depletion in adults. Uptodate.com. Updated February 3, 2020. Accessed March 12, 2021. Available at: https://www.uptodate.com/contents/etiology-clinical-manifestations-and-diagnosis-of-volume-depletion-in-adults
12
