# Supplementary material for: An Observation Medicine Curriculum for Emergency Medicine Education
Source: J Educ Teach Emerg Med. 2021 Apr 19;6(2):C1–C72. doi: 10.21980/J87P92 (PMC10332786; doi:10.21980/J87P92)
Supplement: Supplementary file 11 — Please see associated PowerPoint file [file jetem-6-2-c1-supp11.pptx]

## Slide 1
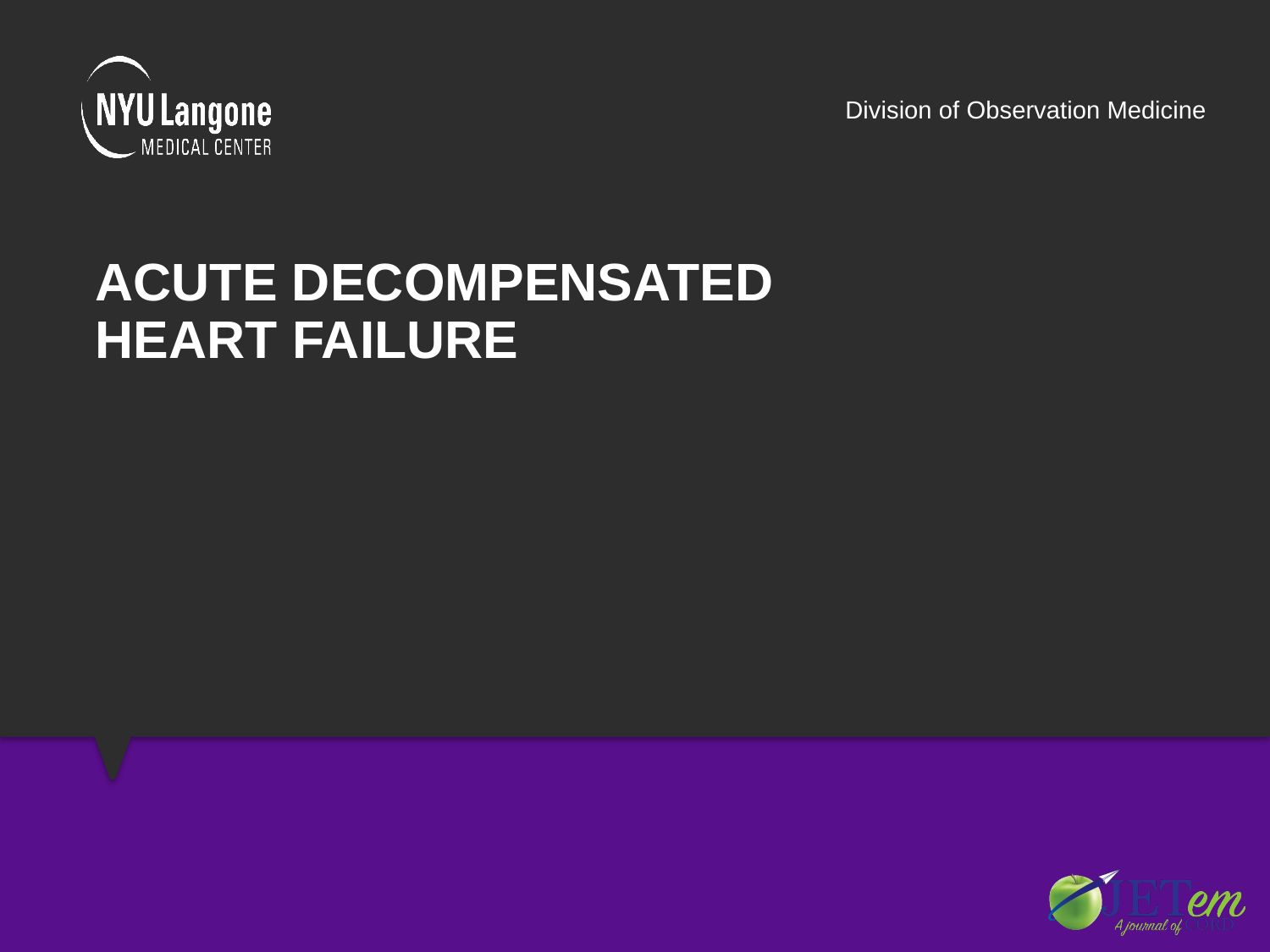

Division of Observation Medicine
# Acute Decompensated HEART FAILURE

## Slide 2
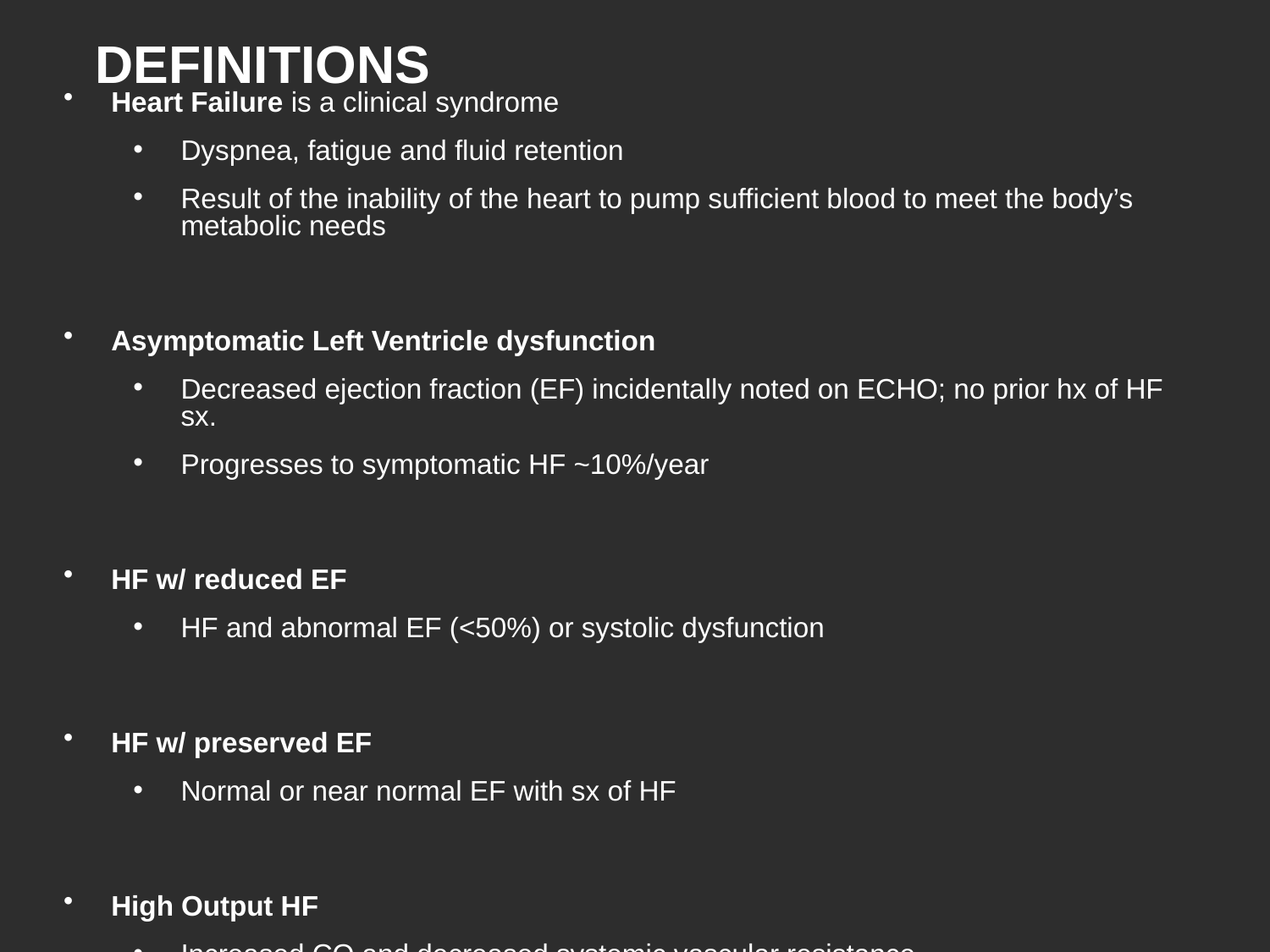

# Definitions
Heart Failure is a clinical syndrome
Dyspnea, fatigue and fluid retention
Result of the inability of the heart to pump sufficient blood to meet the body’s metabolic needs
Asymptomatic Left Ventricle dysfunction
Decreased ejection fraction (EF) incidentally noted on ECHO; no prior hx of HF sx.
Progresses to symptomatic HF ~10%/year
HF w/ reduced EF
HF and abnormal EF (<50%) or systolic dysfunction
HF w/ preserved EF
Normal or near normal EF with sx of HF
High Output HF
Increased CO and decreased systemic vascular resistance

## Slide 3
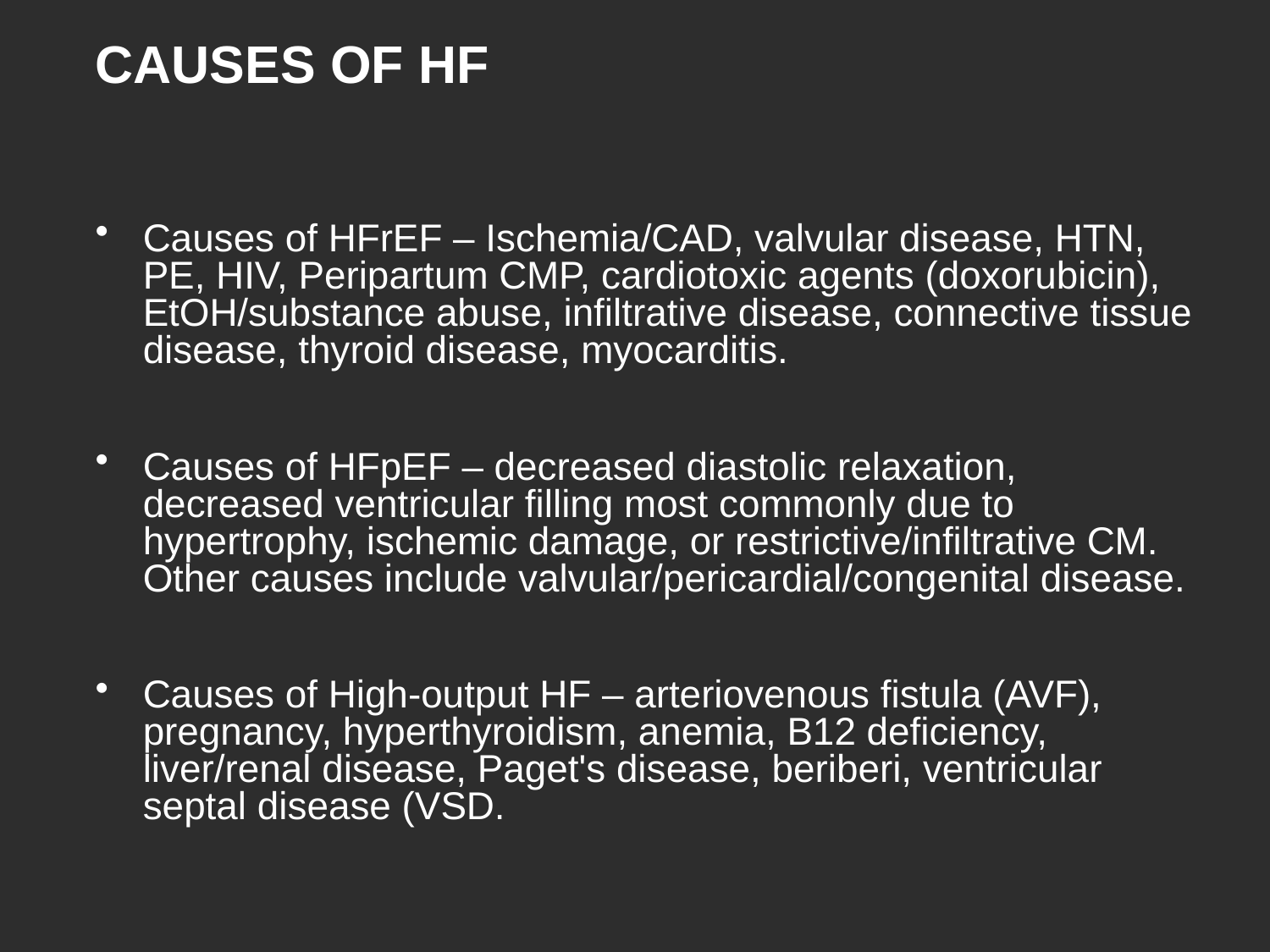

# Causes of HF
Causes of HFrEF – Ischemia/CAD, valvular disease, HTN, PE, HIV, Peripartum CMP, cardiotoxic agents (doxorubicin), EtOH/substance abuse, infiltrative disease, connective tissue disease, thyroid disease, myocarditis.
Causes of HFpEF – decreased diastolic relaxation, decreased ventricular filling most commonly due to hypertrophy, ischemic damage, or restrictive/infiltrative CM. Other causes include valvular/pericardial/congenital disease.
Causes of High-output HF – arteriovenous fistula (AVF), pregnancy, hyperthyroidism, anemia, B12 deficiency, liver/renal disease, Paget's disease, beriberi, ventricular septal disease (VSD.

## Slide 4
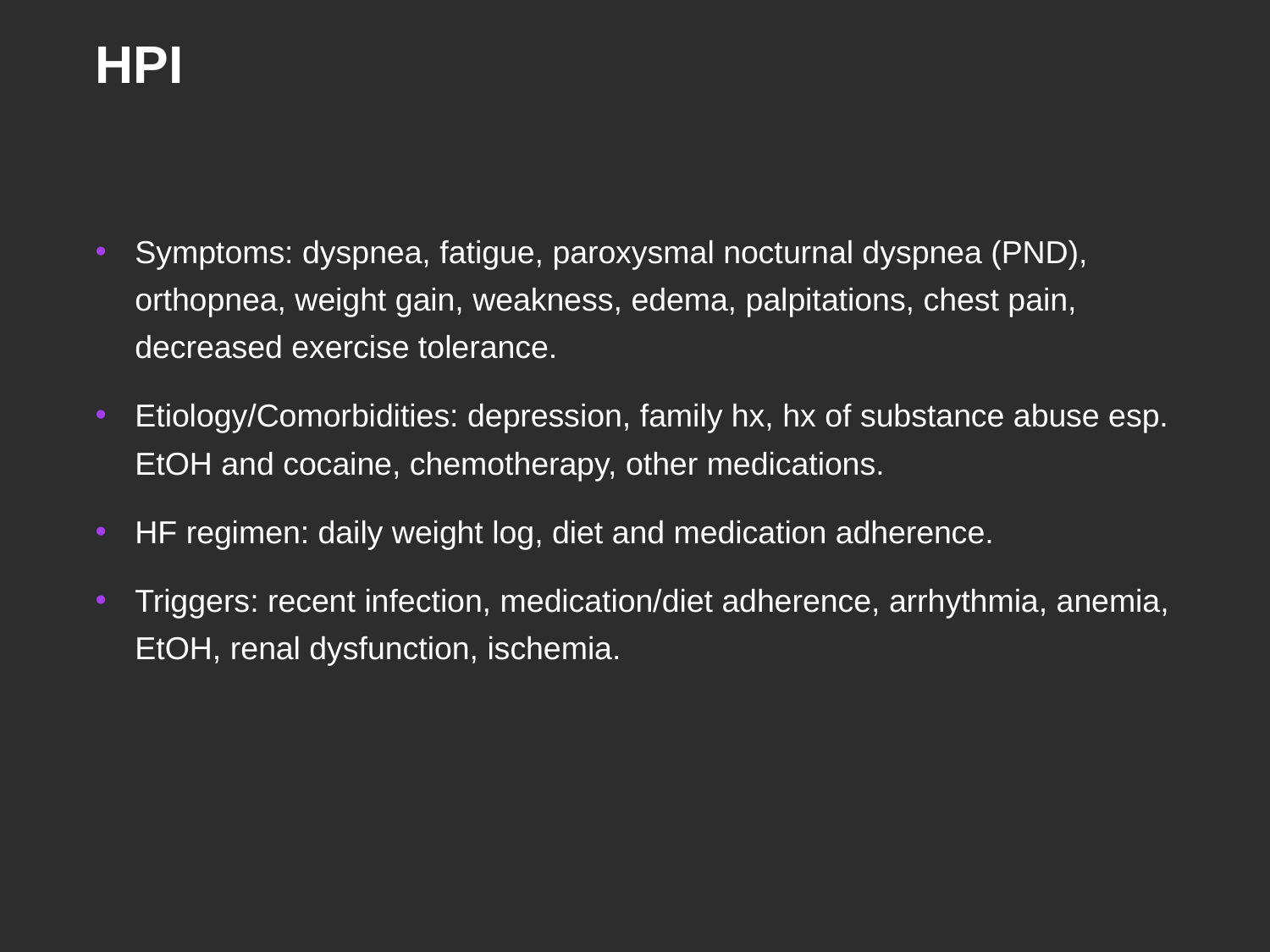

# HPI
Symptoms: dyspnea, fatigue, paroxysmal nocturnal dyspnea (PND), orthopnea, weight gain, weakness, edema, palpitations, chest pain, decreased exercise tolerance.
Etiology/Comorbidities: depression, family hx, hx of substance abuse esp. EtOH and cocaine, chemotherapy, other medications.
HF regimen: daily weight log, diet and medication adherence.
Triggers: recent infection, medication/diet adherence, arrhythmia, anemia, EtOH, renal dysfunction, ischemia.

## Slide 5
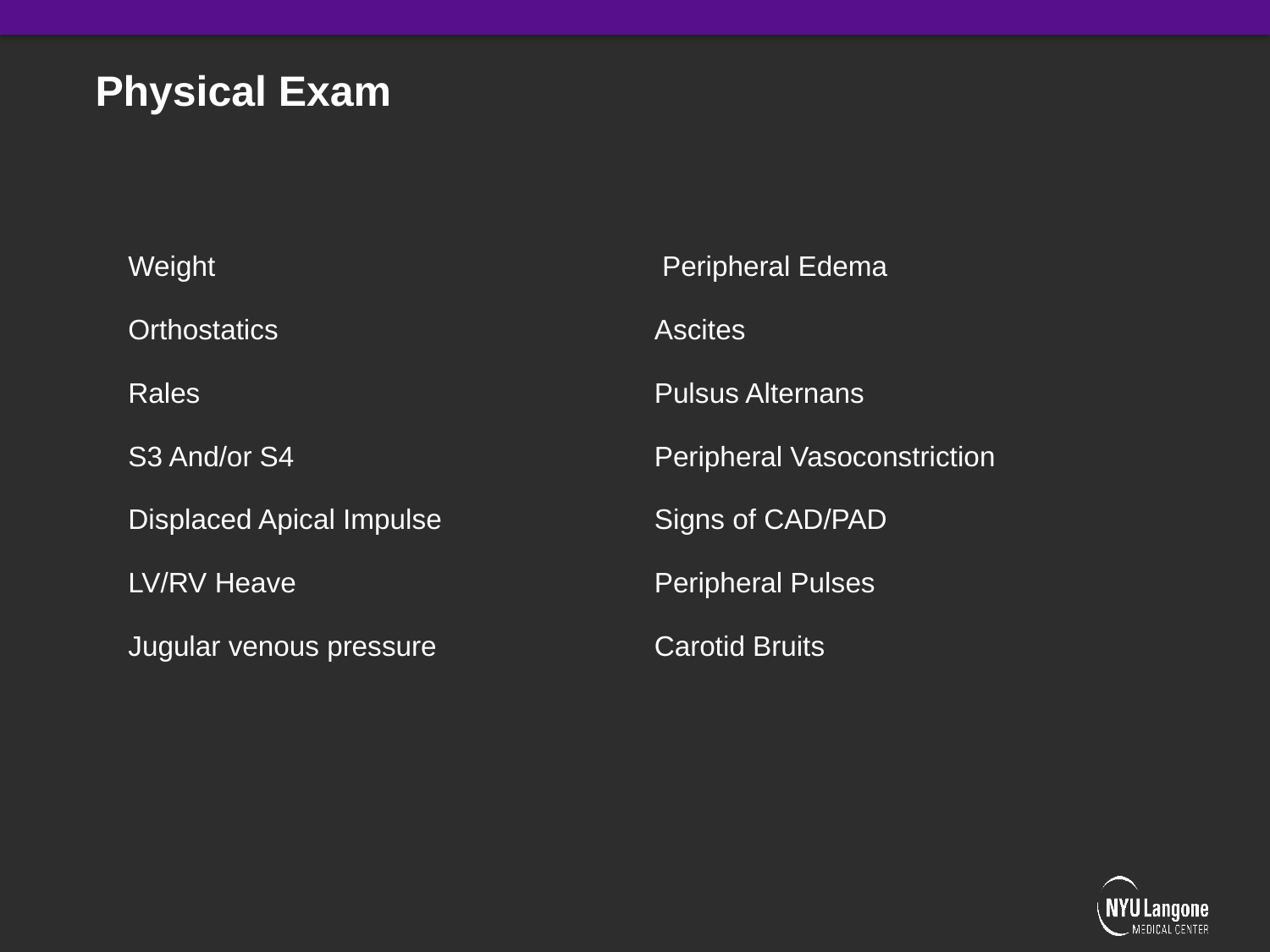

# Physical Exam
Weight
Orthostatics
Rales
S3 And/or S4
Displaced Apical Impulse
LV/RV Heave
Jugular venous pressure
 Peripheral Edema
Ascites
Pulsus Alternans
Peripheral Vasoconstriction
Signs of CAD/PAD
Peripheral Pulses
Carotid Bruits

## Slide 6
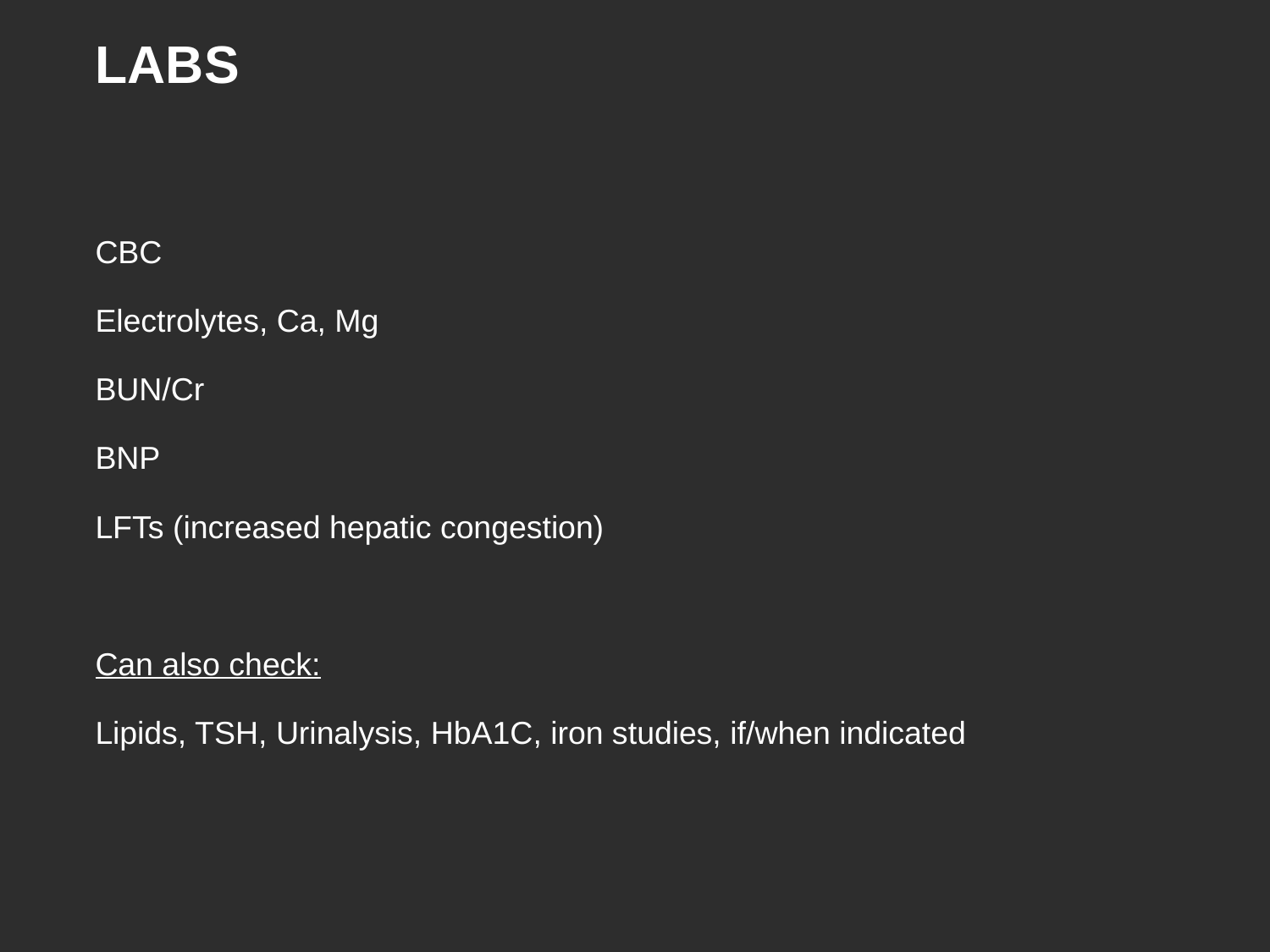

# Labs
CBC
Electrolytes, Ca, Mg
BUN/Cr
BNP
LFTs (increased hepatic congestion)
Can also check:
Lipids, TSH, Urinalysis, HbA1C, iron studies, if/when indicated

## Slide 7
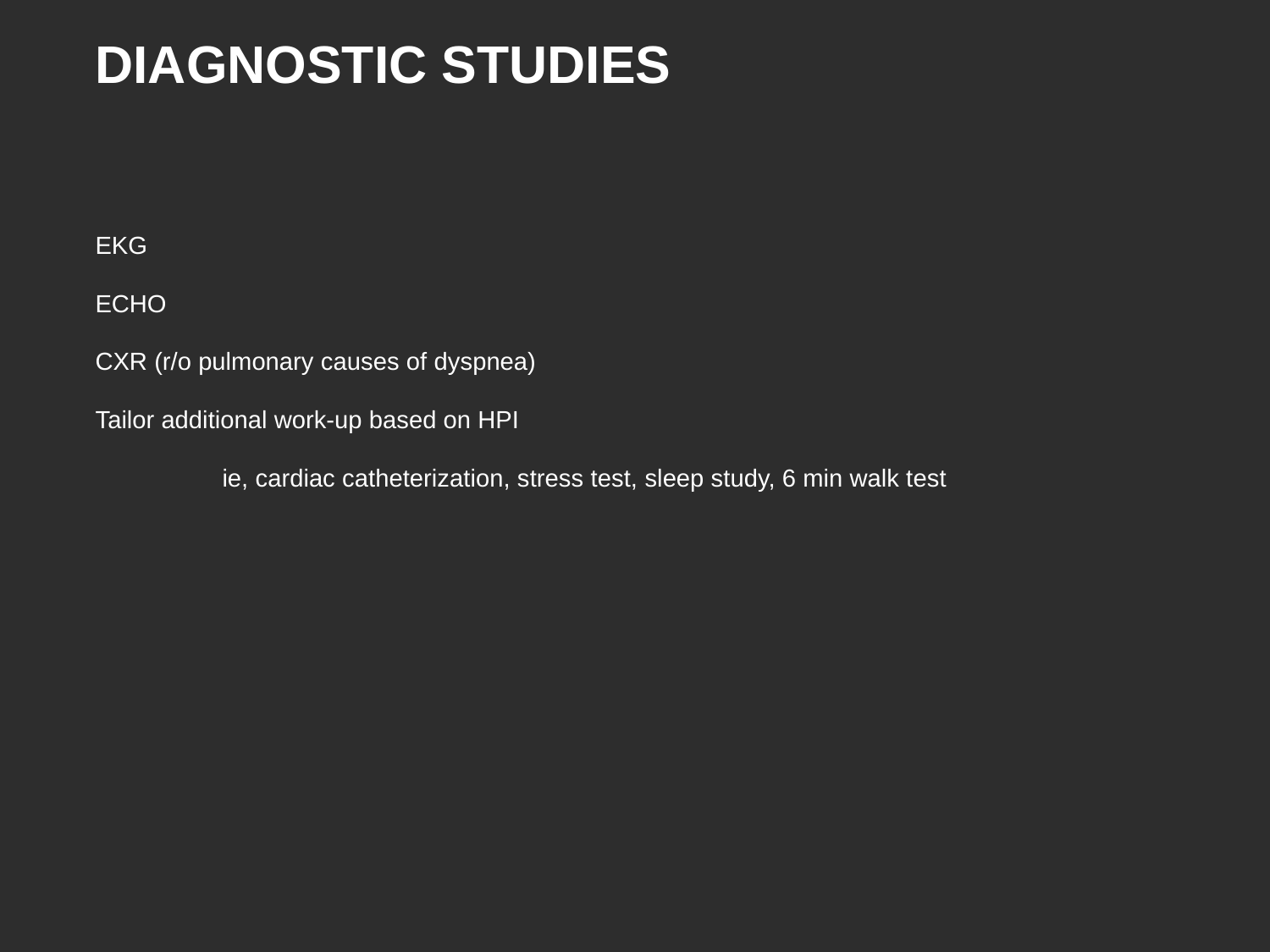

# Diagnostic Studies
EKG
ECHO
CXR (r/o pulmonary causes of dyspnea)
Tailor additional work-up based on HPI
	ie, cardiac catheterization, stress test, sleep study, 6 min walk test

## Slide 8
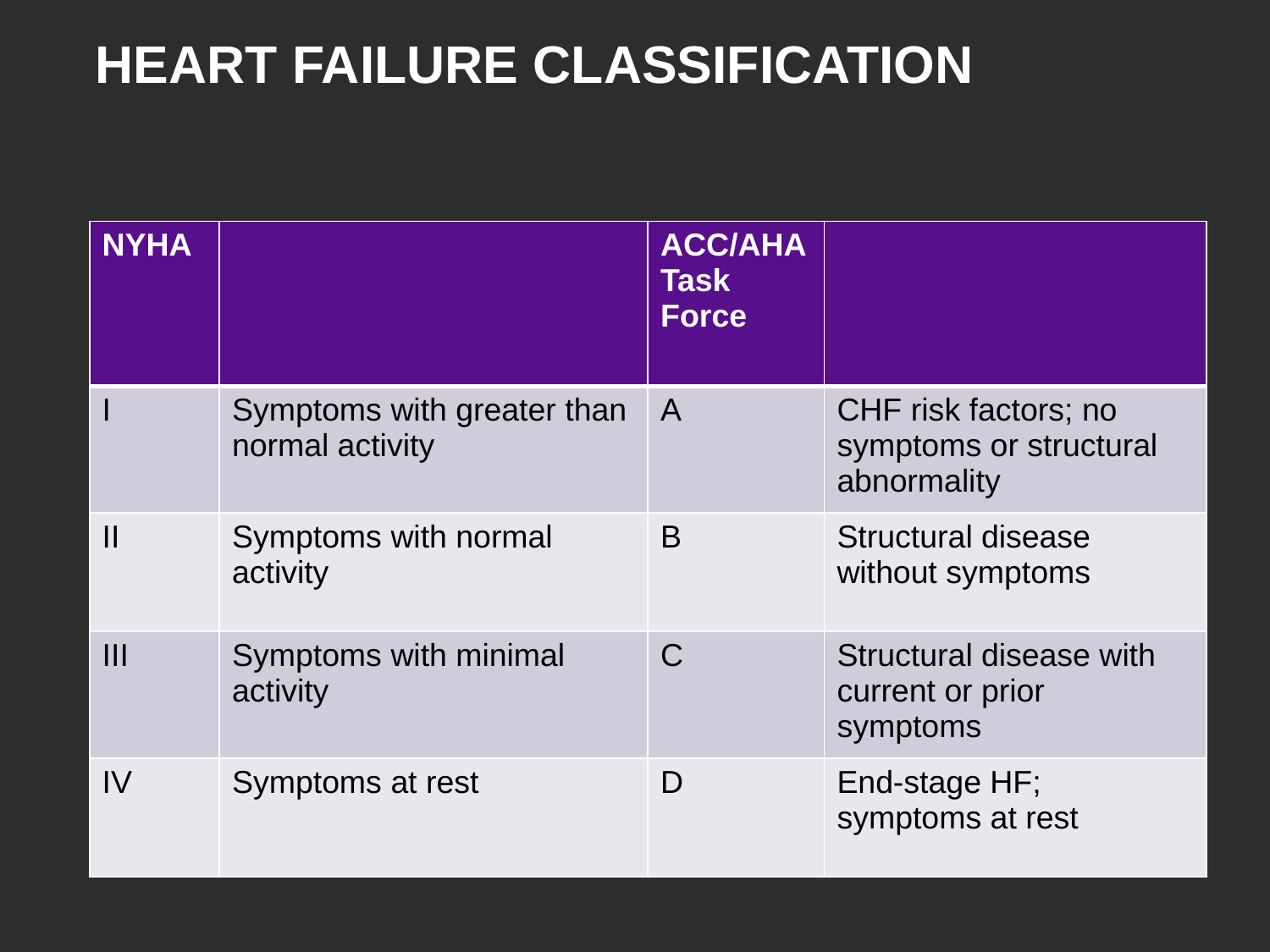

# Heart Failure Classification
| NYHA | | ACC/AHA Task Force | |
| --- | --- | --- | --- |
| I | Symptoms with greater than normal activity | A | CHF risk factors; no symptoms or structural abnormality |
| II | Symptoms with normal activity | B | Structural disease without symptoms |
| III | Symptoms with minimal activity | C | Structural disease with current or prior symptoms |
| IV | Symptoms at rest | D | End-stage HF; symptoms at rest |

## Slide 9
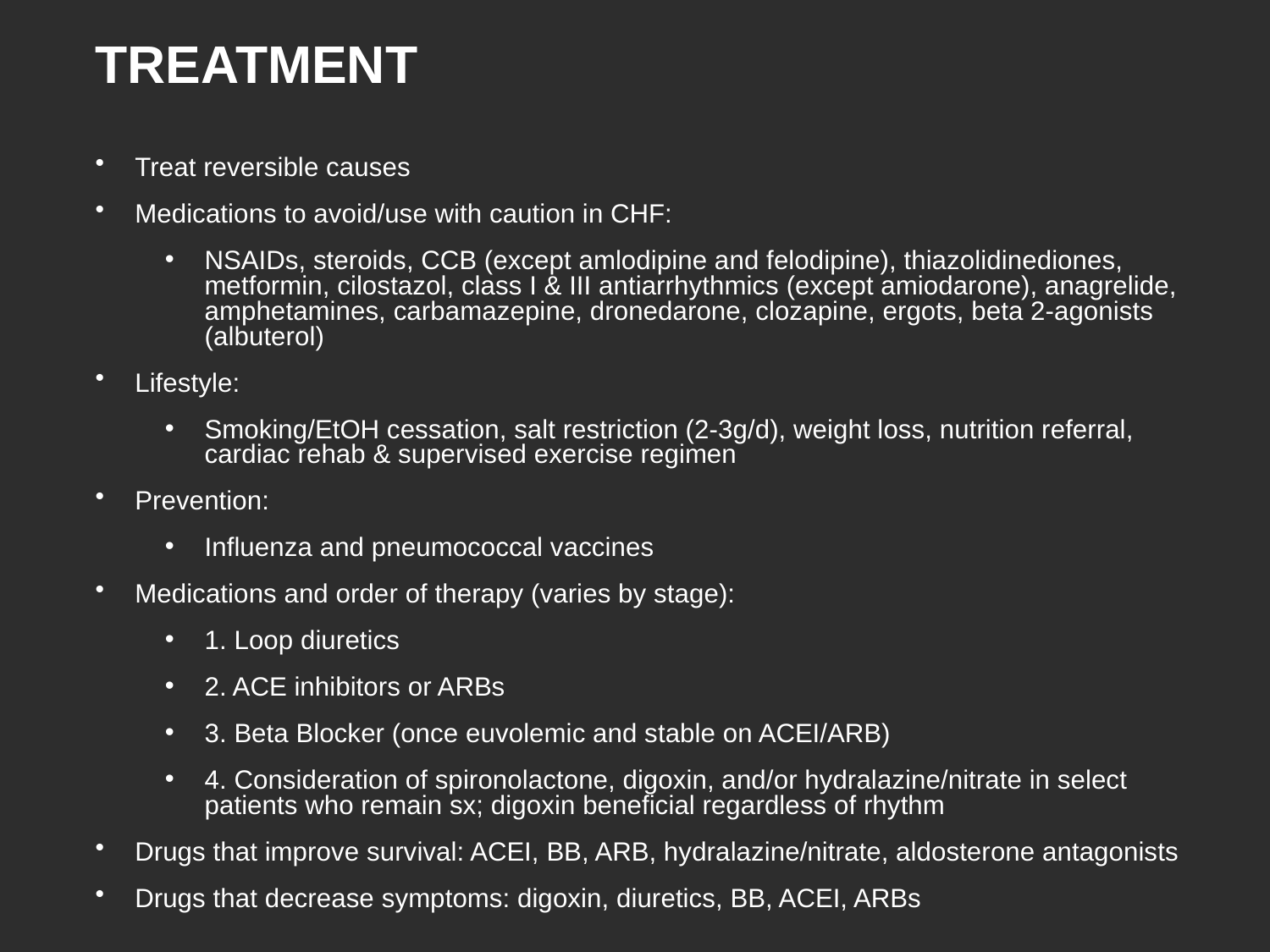

# Treatment
Treat reversible causes
Medications to avoid/use with caution in CHF:
NSAIDs, steroids, CCB (except amlodipine and felodipine), thiazolidinediones, metformin, cilostazol, class I & III antiarrhythmics (except amiodarone), anagrelide, amphetamines, carbamazepine, dronedarone, clozapine, ergots, beta 2-agonists (albuterol)
Lifestyle:
Smoking/EtOH cessation, salt restriction (2-3g/d), weight loss, nutrition referral, cardiac rehab & supervised exercise regimen
Prevention:
Influenza and pneumococcal vaccines
Medications and order of therapy (varies by stage):
1. Loop diuretics
2. ACE inhibitors or ARBs
3. Beta Blocker (once euvolemic and stable on ACEI/ARB)
4. Consideration of spironolactone, digoxin, and/or hydralazine/nitrate in select patients who remain sx; digoxin beneficial regardless of rhythm
Drugs that improve survival: ACEI, BB, ARB, hydralazine/nitrate, aldosterone antagonists
Drugs that decrease symptoms: digoxin, diuretics, BB, ACEI, ARBs

## Slide 10
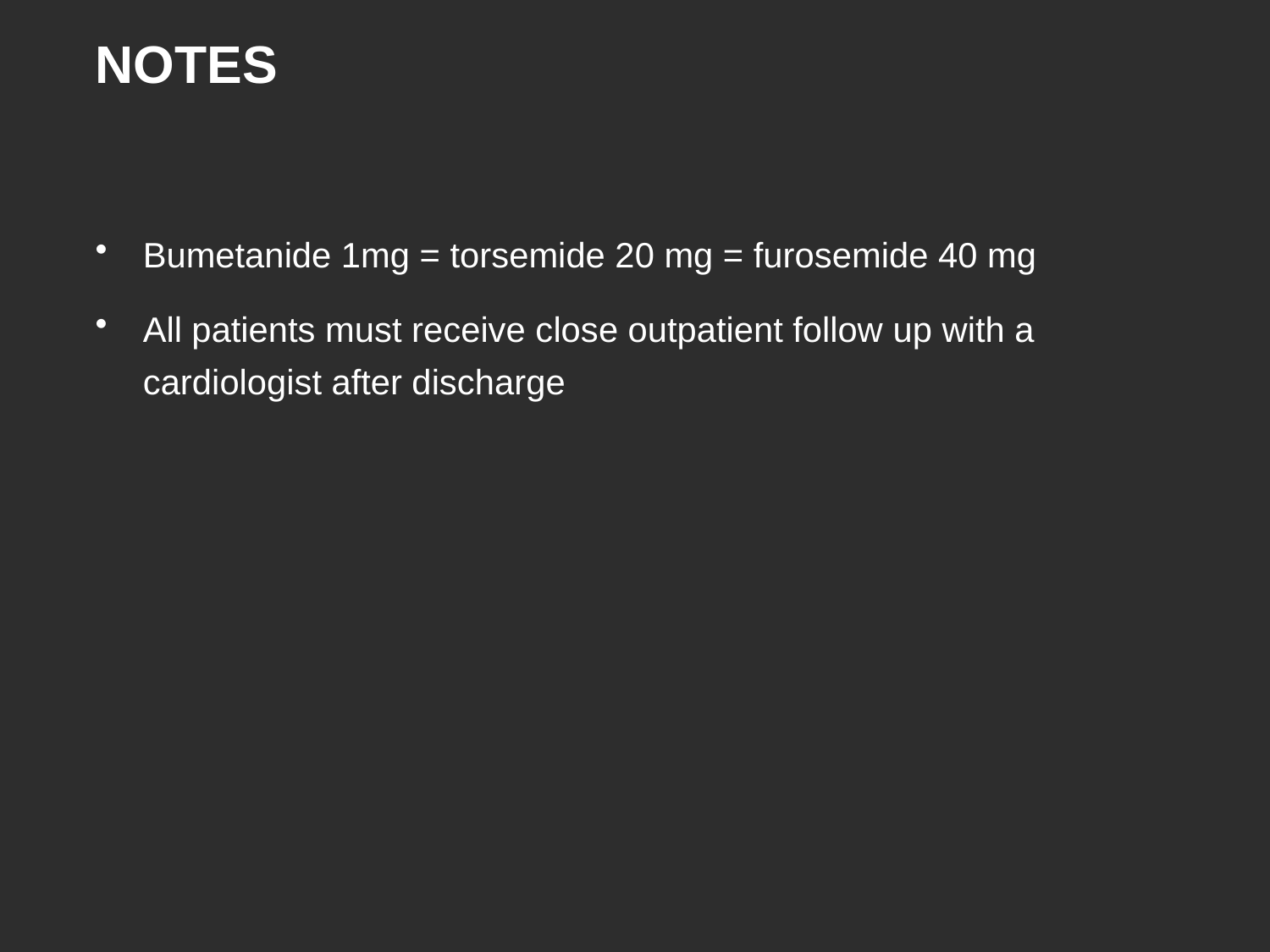

# Notes
Bumetanide 1mg = torsemide 20 mg = furosemide 40 mg
All patients must receive close outpatient follow up with a cardiologist after discharge

## Slide 11
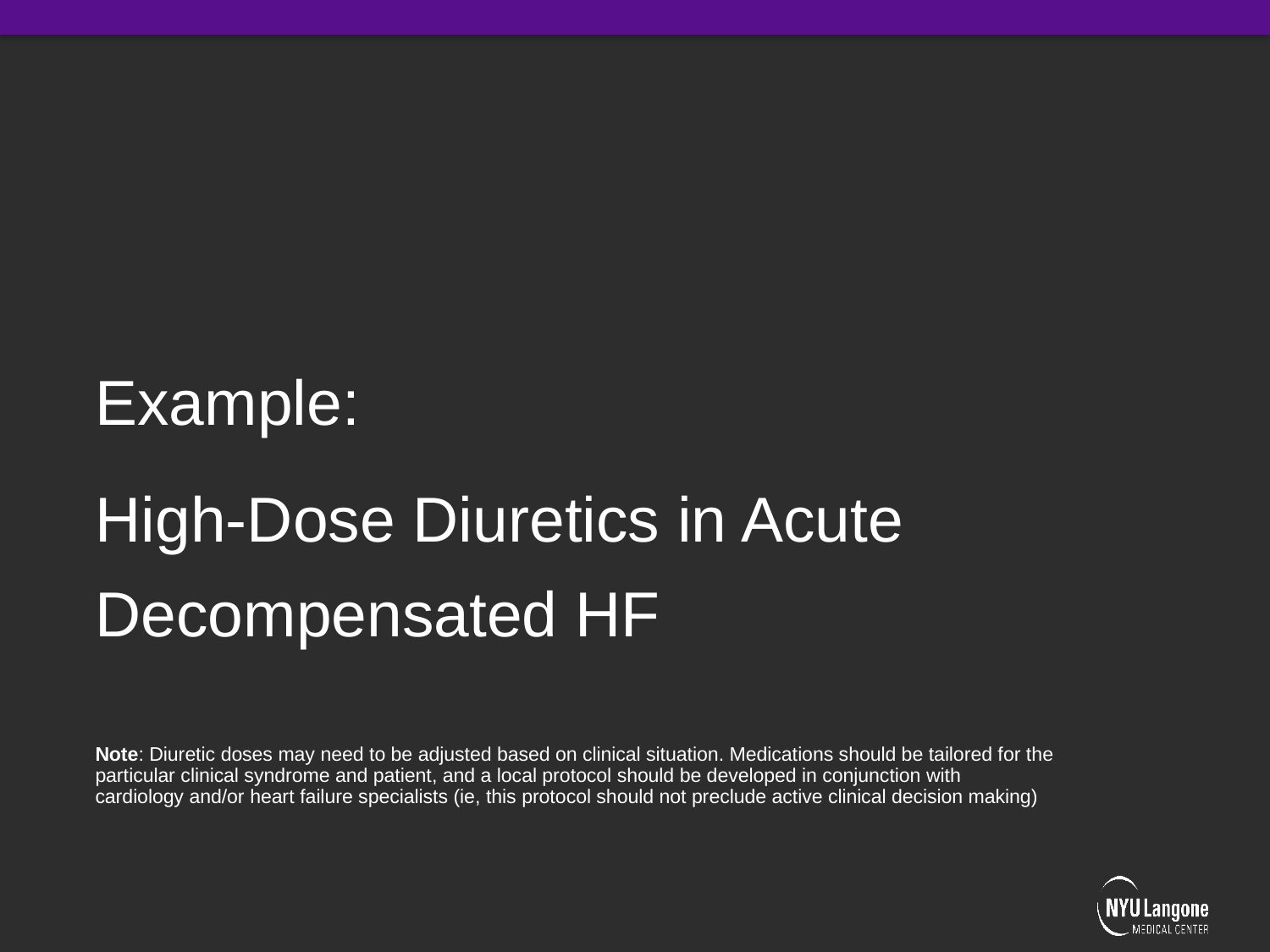

Example:
High-Dose Diuretics in Acute Decompensated HF
# Note: Diuretic doses may need to be adjusted based on clinical situation. Medications should be tailored for the particular clinical syndrome and patient, and a local protocol should be developed in conjunction with cardiology and/or heart failure specialists (ie, this protocol should not preclude active clinical decision making)

## Slide 12
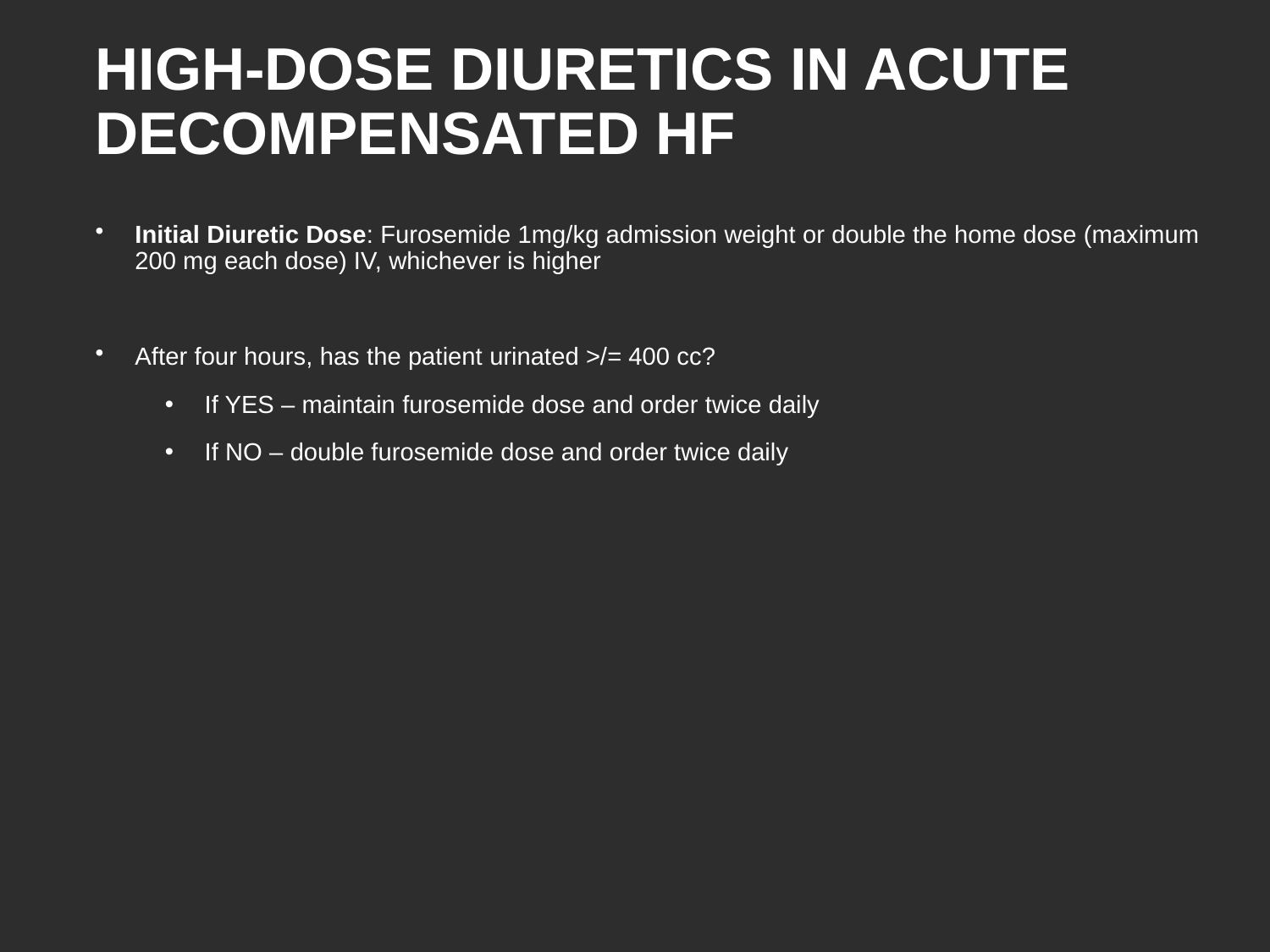

# High-Dose Diuretics in Acute Decompensated HF
Initial Diuretic Dose: Furosemide 1mg/kg admission weight or double the home dose (maximum 200 mg each dose) IV, whichever is higher
After four hours, has the patient urinated >/= 400 cc?
If YES – maintain furosemide dose and order twice daily
If NO – double furosemide dose and order twice daily

## Slide 13
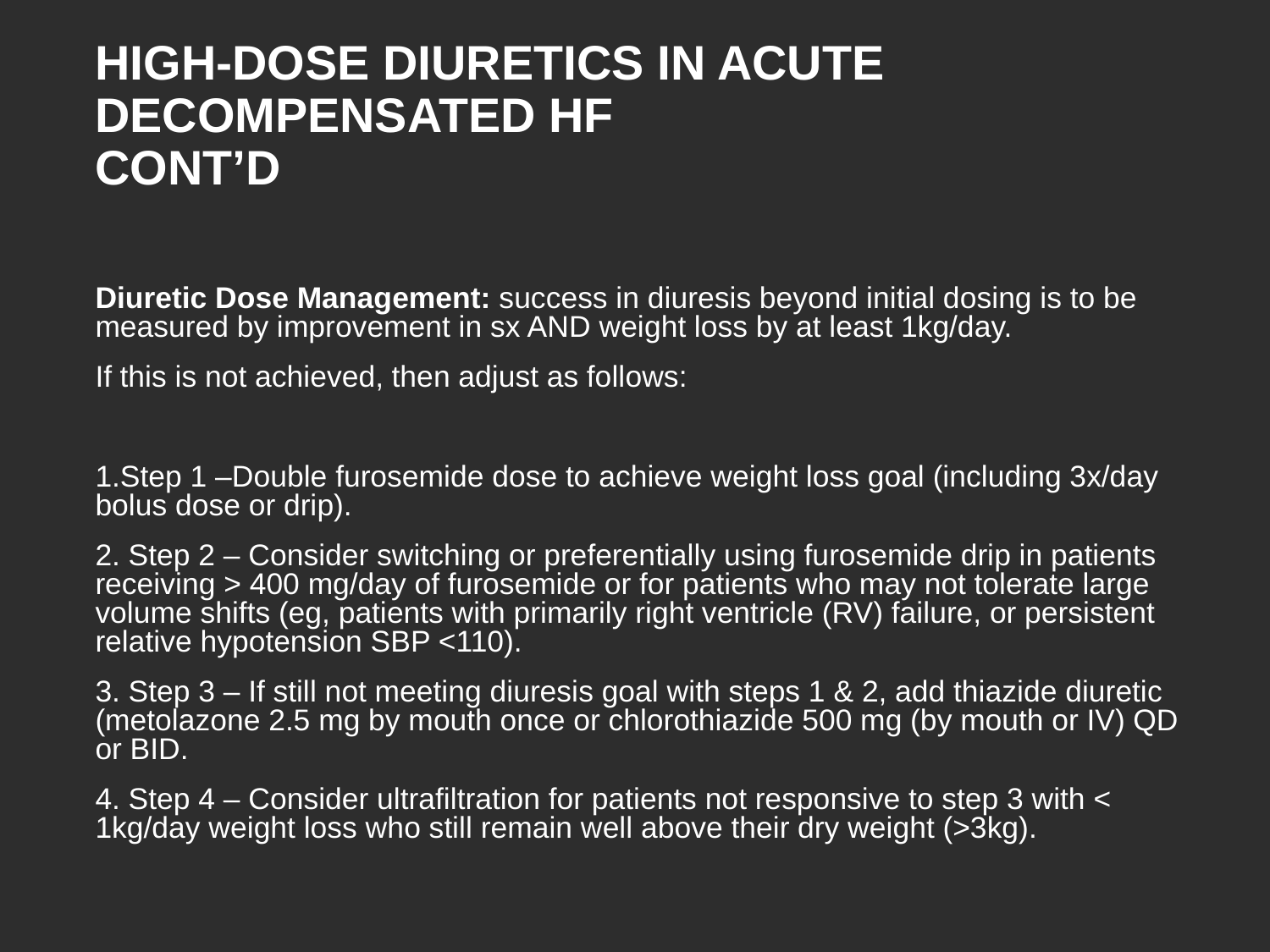

# High-Dose Diuretics in Acute Decompensated HF cont’d
Diuretic Dose Management: success in diuresis beyond initial dosing is to be measured by improvement in sx AND weight loss by at least 1kg/day.
If this is not achieved, then adjust as follows:
1.Step 1 –Double furosemide dose to achieve weight loss goal (including 3x/day bolus dose or drip).
2. Step 2 – Consider switching or preferentially using furosemide drip in patients receiving > 400 mg/day of furosemide or for patients who may not tolerate large volume shifts (eg, patients with primarily right ventricle (RV) failure, or persistent relative hypotension SBP <110).
3. Step 3 – If still not meeting diuresis goal with steps 1 & 2, add thiazide diuretic (metolazone 2.5 mg by mouth once or chlorothiazide 500 mg (by mouth or IV) QD or BID.
4. Step 4 – Consider ultrafiltration for patients not responsive to step 3 with < 1kg/day weight loss who still remain well above their dry weight (>3kg).

## Slide 14
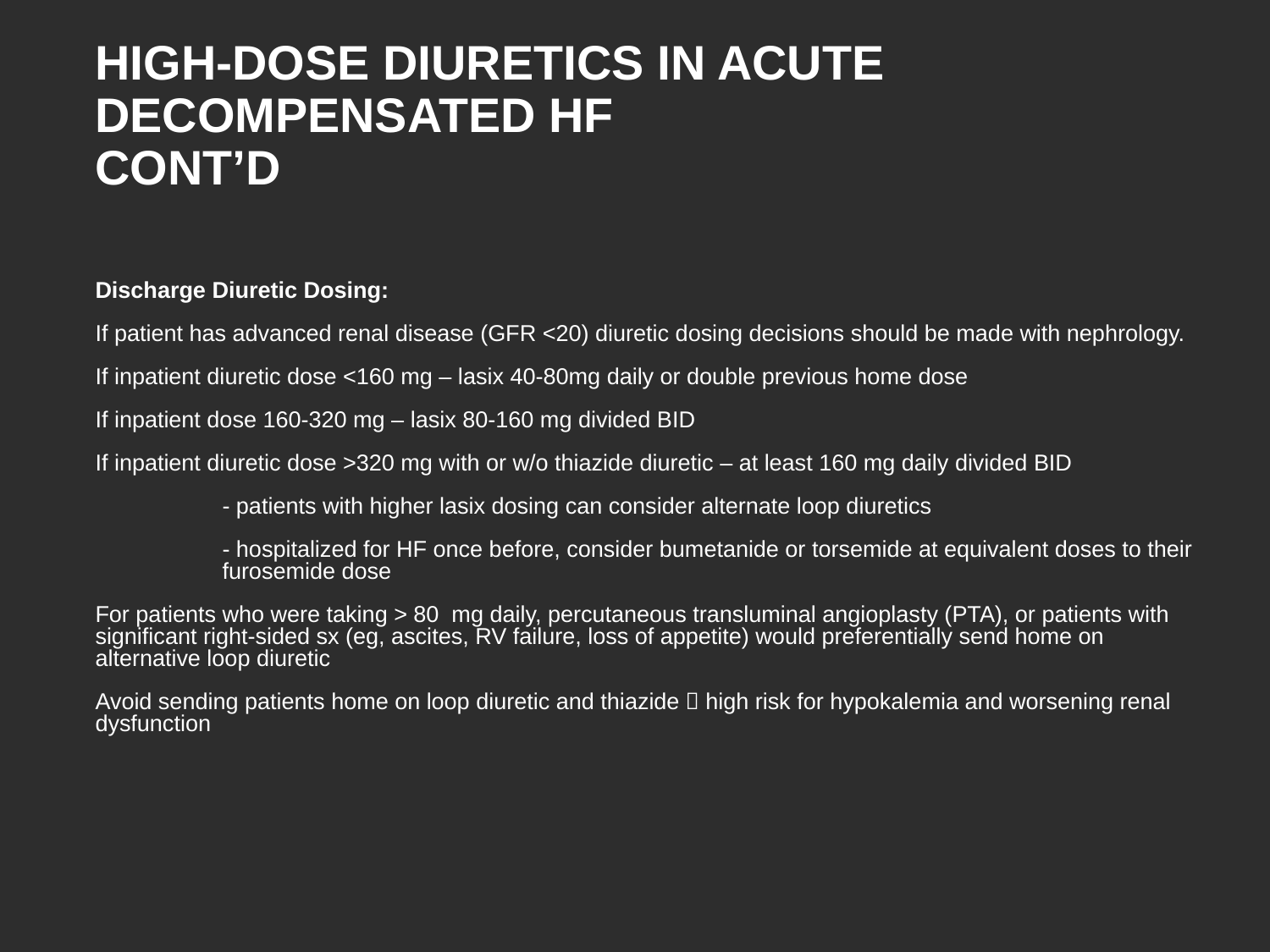

# High-Dose Diuretics in Acute Decompensated HF cont’d
Discharge Diuretic Dosing:
If patient has advanced renal disease (GFR <20) diuretic dosing decisions should be made with nephrology.
If inpatient diuretic dose <160 mg – lasix 40-80mg daily or double previous home dose
If inpatient dose 160-320 mg – lasix 80-160 mg divided BID
If inpatient diuretic dose >320 mg with or w/o thiazide diuretic – at least 160 mg daily divided BID
	- patients with higher lasix dosing can consider alternate loop diuretics
	- hospitalized for HF once before, consider bumetanide or torsemide at equivalent doses to their 	furosemide dose
For patients who were taking > 80 mg daily, percutaneous transluminal angioplasty (PTA), or patients with significant right-sided sx (eg, ascites, RV failure, loss of appetite) would preferentially send home on alternative loop diuretic
Avoid sending patients home on loop diuretic and thiazide  high risk for hypokalemia and worsening renal dysfunction

## Slide 15
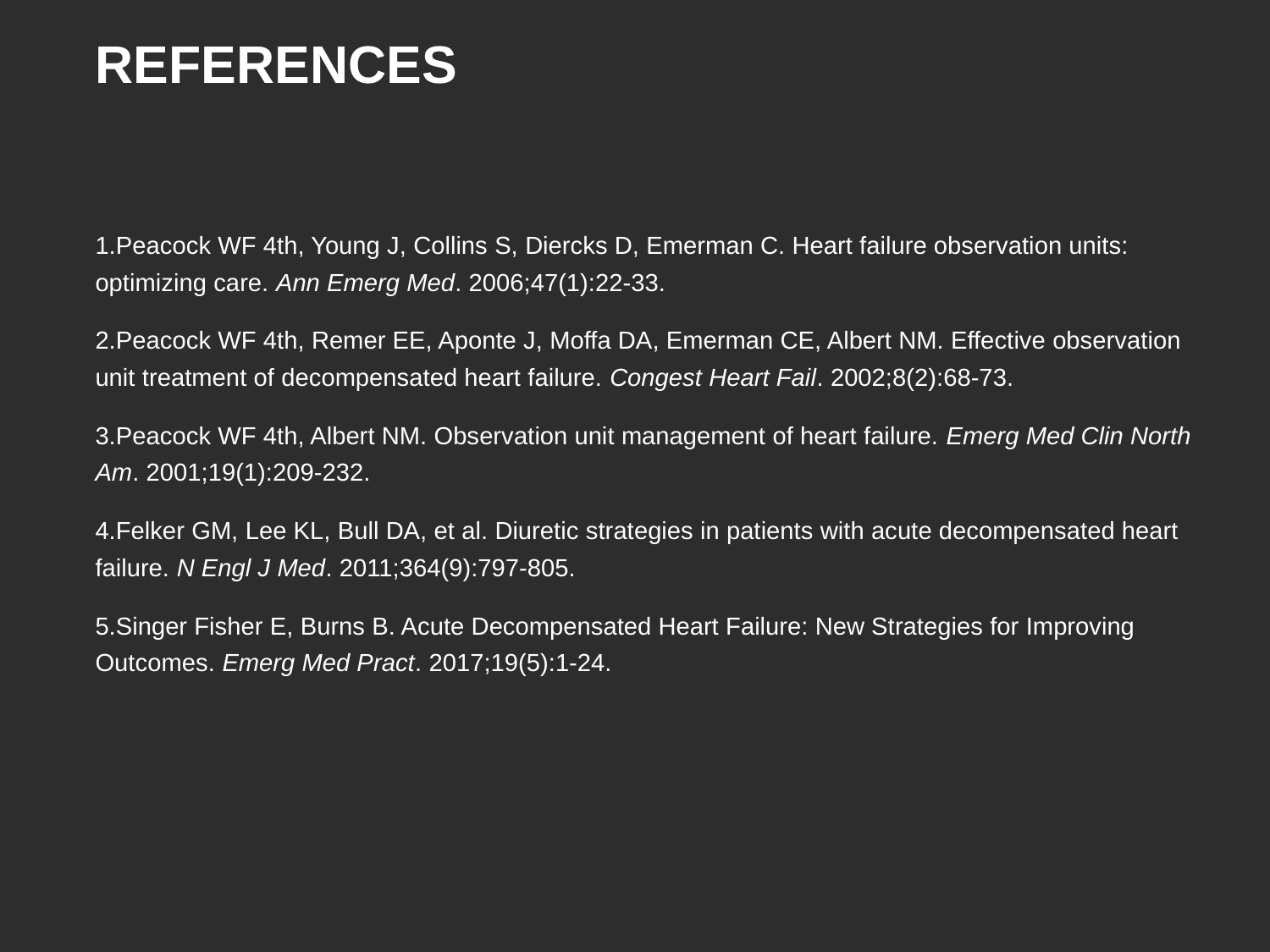

# references
1.Peacock WF 4th, Young J, Collins S, Diercks D, Emerman C. Heart failure observation units: optimizing care. Ann Emerg Med. 2006;47(1):22-33.
2.Peacock WF 4th, Remer EE, Aponte J, Moffa DA, Emerman CE, Albert NM. Effective observation unit treatment of decompensated heart failure. Congest Heart Fail. 2002;8(2):68-73.
3.Peacock WF 4th, Albert NM. Observation unit management of heart failure. Emerg Med Clin North Am. 2001;19(1):209-232.
4.Felker GM, Lee KL, Bull DA, et al. Diuretic strategies in patients with acute decompensated heart failure. N Engl J Med. 2011;364(9):797-805.
5.Singer Fisher E, Burns B. Acute Decompensated Heart Failure: New Strategies for Improving Outcomes. Emerg Med Pract. 2017;19(5):1-24.
