# Supplementary material for: An Observation Medicine Curriculum for Emergency Medicine Education
Source: J Educ Teach Emerg Med. 2021 Apr 19;6(2):C1–C72. doi: 10.21980/J87P92 (PMC10332786; doi:10.21980/J87P92)
Supplement: Supplementary file 12 — Please see associated PowerPoint file [file jetem-6-2-c1-supp12.pptx]

## Slide 1
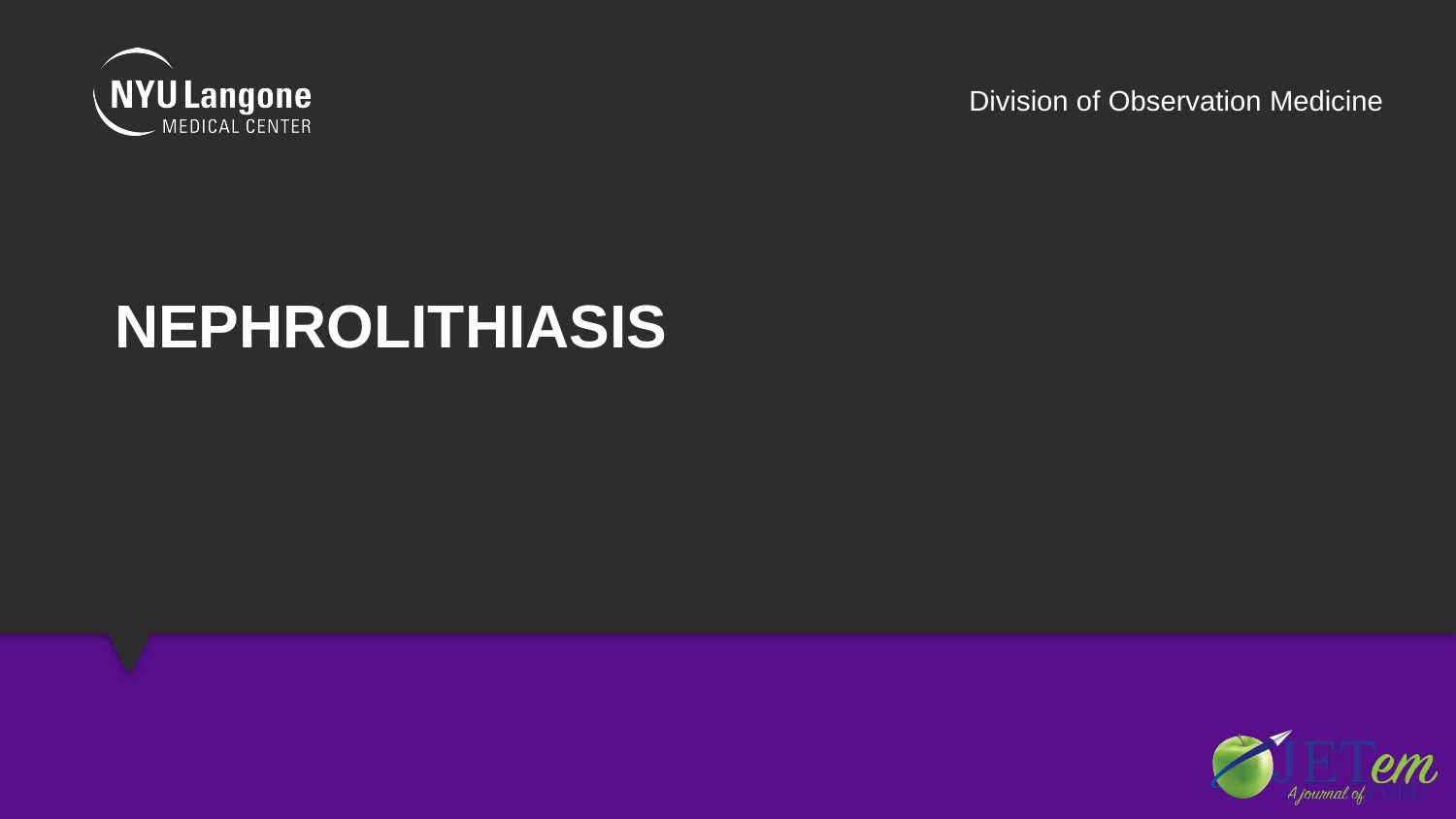

Division of Observation Medicine
# Nephrolithiasis​

## Slide 2
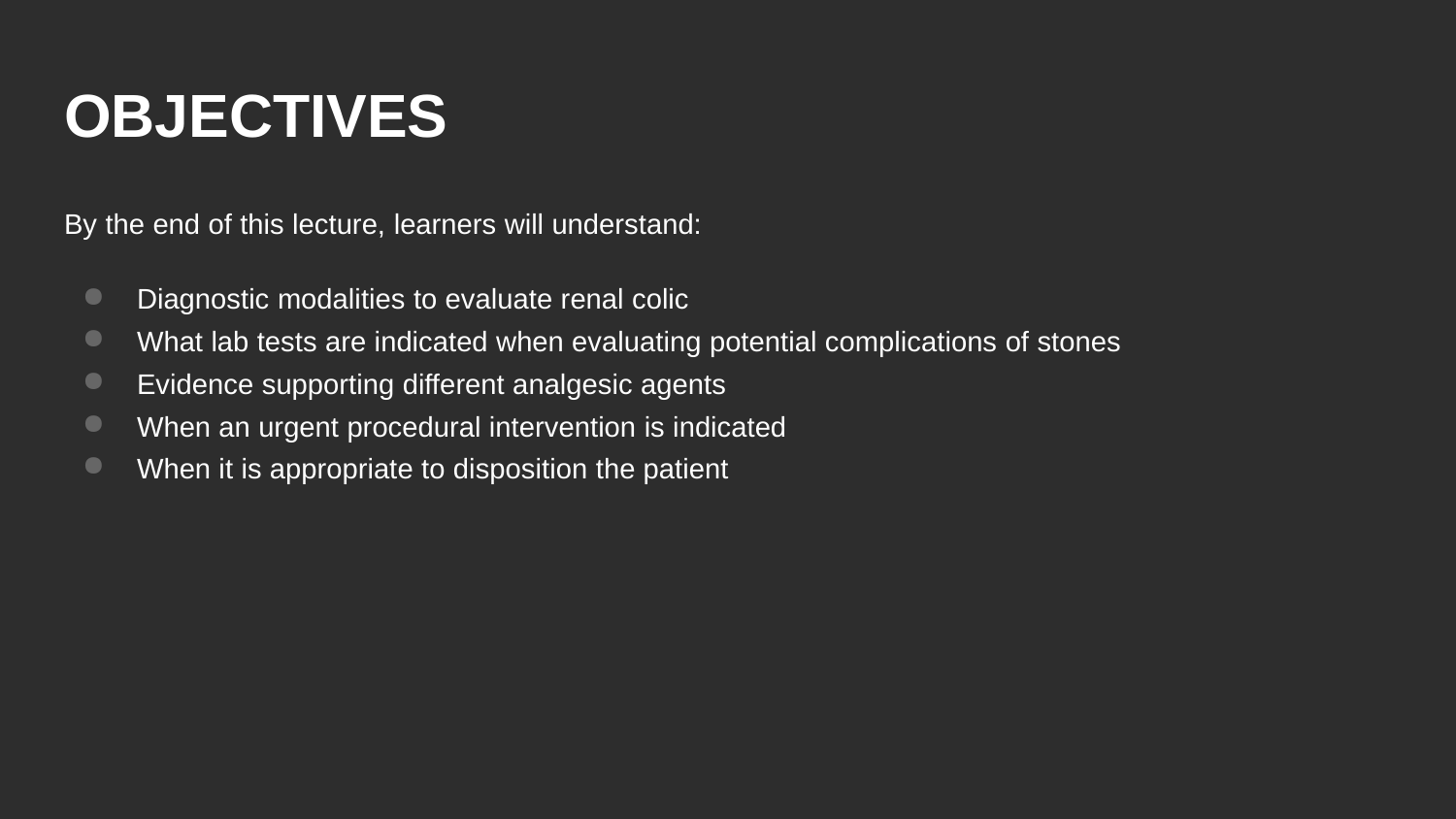

# Objectives
By the end of this lecture, learners will understand:
Diagnostic modalities to evaluate renal colic
What lab tests are indicated when evaluating potential complications of stones
Evidence supporting different analgesic agents
When an urgent procedural intervention is indicated
When it is appropriate to disposition the patient

## Slide 3
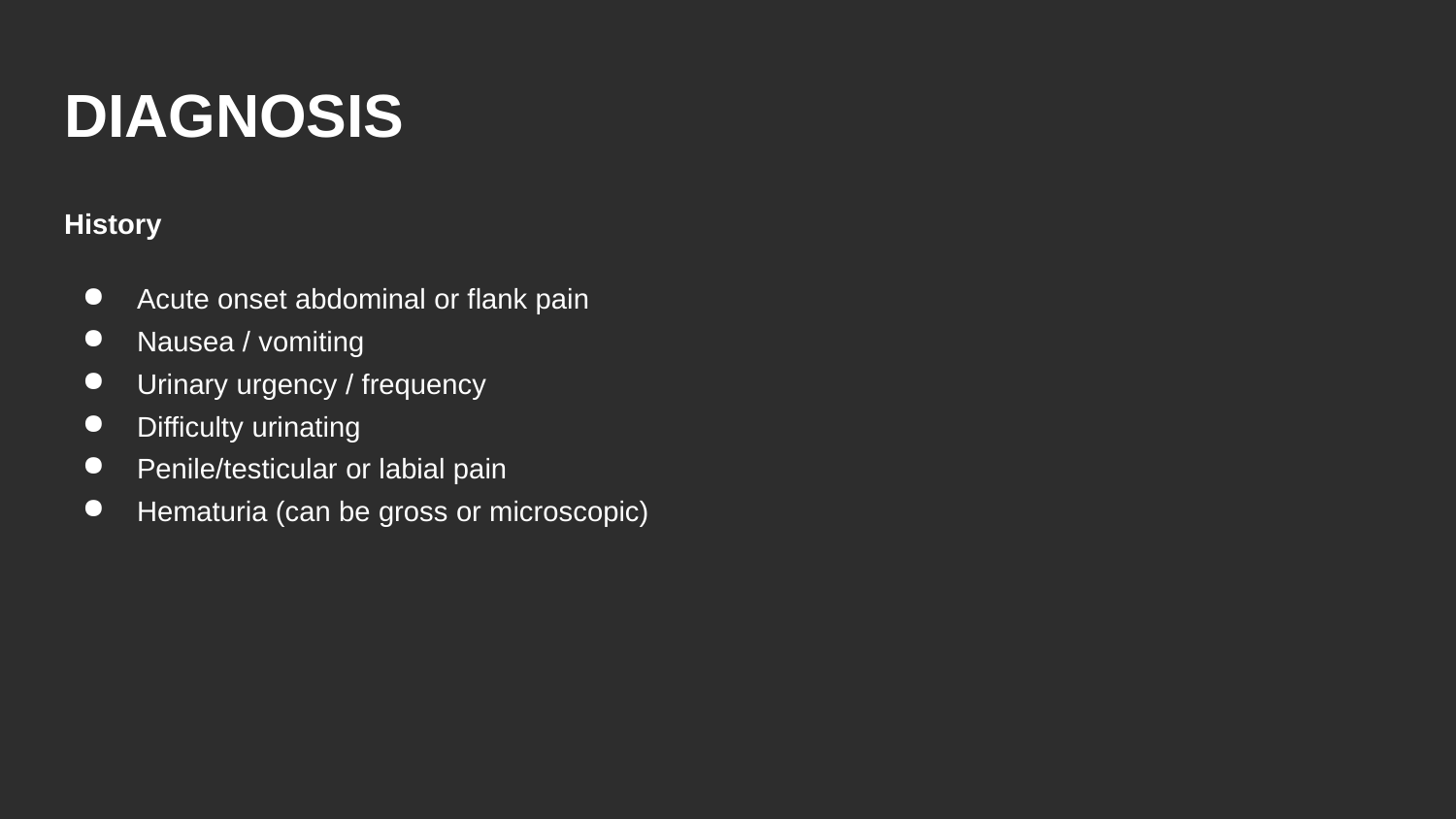

# Diagnosis
History
Acute onset abdominal or flank pain
Nausea / vomiting
Urinary urgency / frequency
Difficulty urinating
Penile/testicular or labial pain
Hematuria (can be gross or microscopic)

## Slide 4
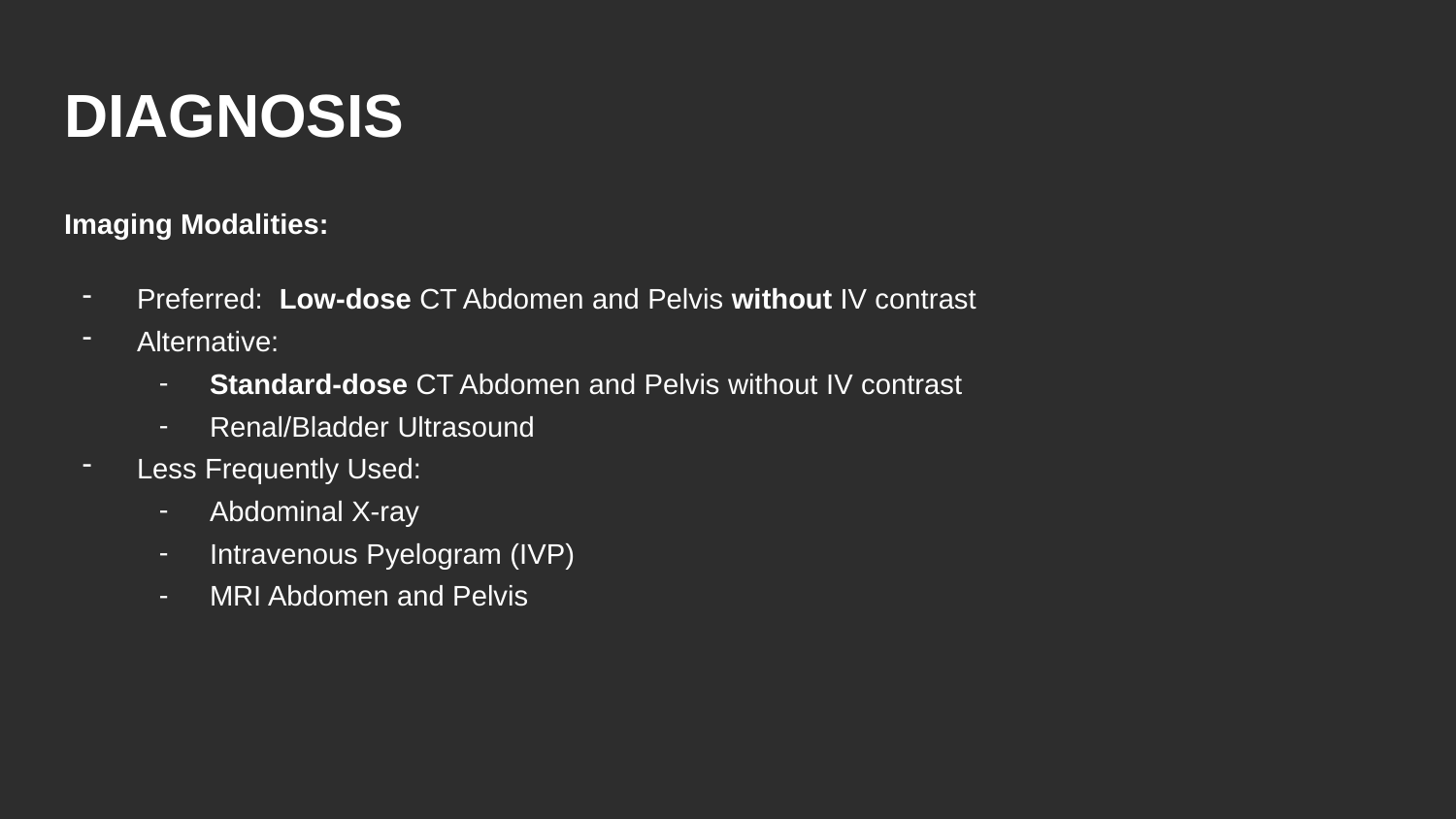

# Diagnosis
Imaging Modalities:
Preferred: Low-dose CT Abdomen and Pelvis without IV contrast
Alternative:
Standard-dose CT Abdomen and Pelvis without IV contrast
Renal/Bladder Ultrasound
Less Frequently Used:
Abdominal X-ray
Intravenous Pyelogram (IVP)
MRI Abdomen and Pelvis

## Slide 5
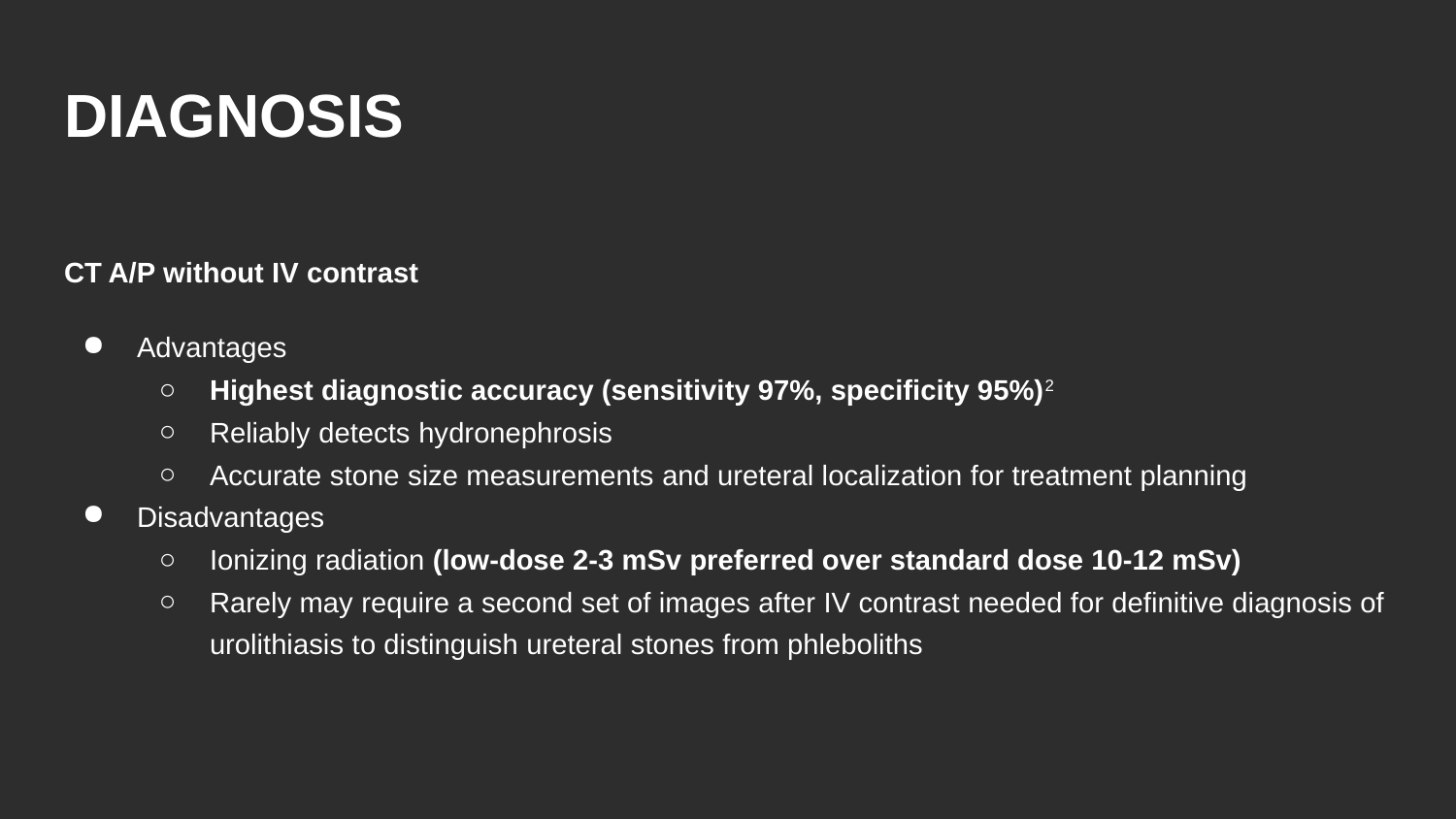

# Diagnosis
CT A/P without IV contrast
Advantages
Highest diagnostic accuracy (sensitivity 97%, specificity 95%)2
Reliably detects hydronephrosis
Accurate stone size measurements and ureteral localization for treatment planning
Disadvantages
Ionizing radiation (low-dose 2-3 mSv preferred over standard dose 10-12 mSv)
Rarely may require a second set of images after IV contrast needed for definitive diagnosis of urolithiasis to distinguish ureteral stones from phleboliths

## Slide 6
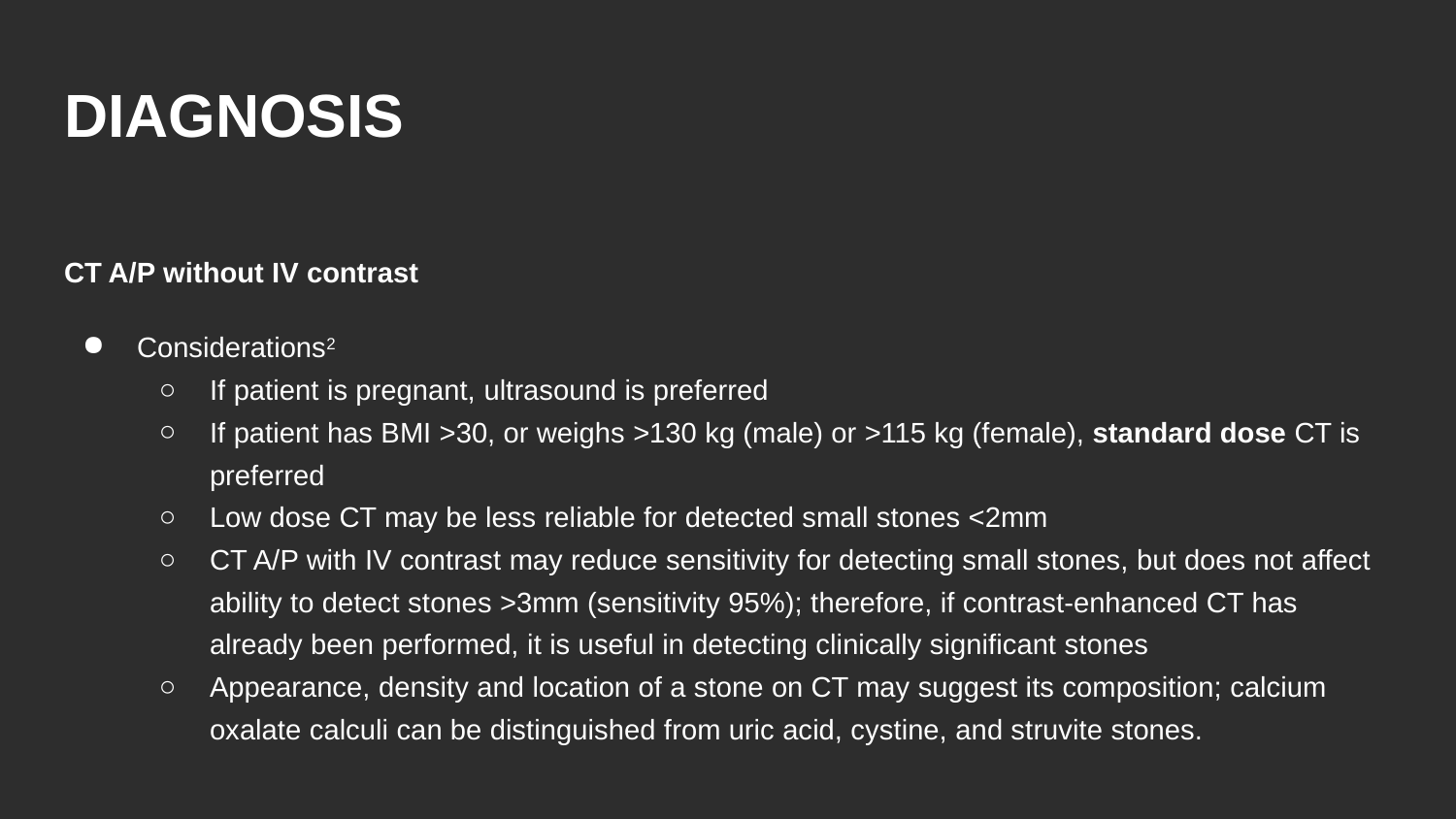

# Diagnosis
CT A/P without IV contrast
Considerations2
If patient is pregnant, ultrasound is preferred
If patient has BMI >30, or weighs >130 kg (male) or >115 kg (female), standard dose CT is preferred
Low dose CT may be less reliable for detected small stones <2mm
CT A/P with IV contrast may reduce sensitivity for detecting small stones, but does not affect ability to detect stones >3mm (sensitivity 95%); therefore, if contrast-enhanced CT has already been performed, it is useful in detecting clinically significant stones
Appearance, density and location of a stone on CT may suggest its composition; calcium oxalate calculi can be distinguished from uric acid, cystine, and struvite stones.

## Slide 7
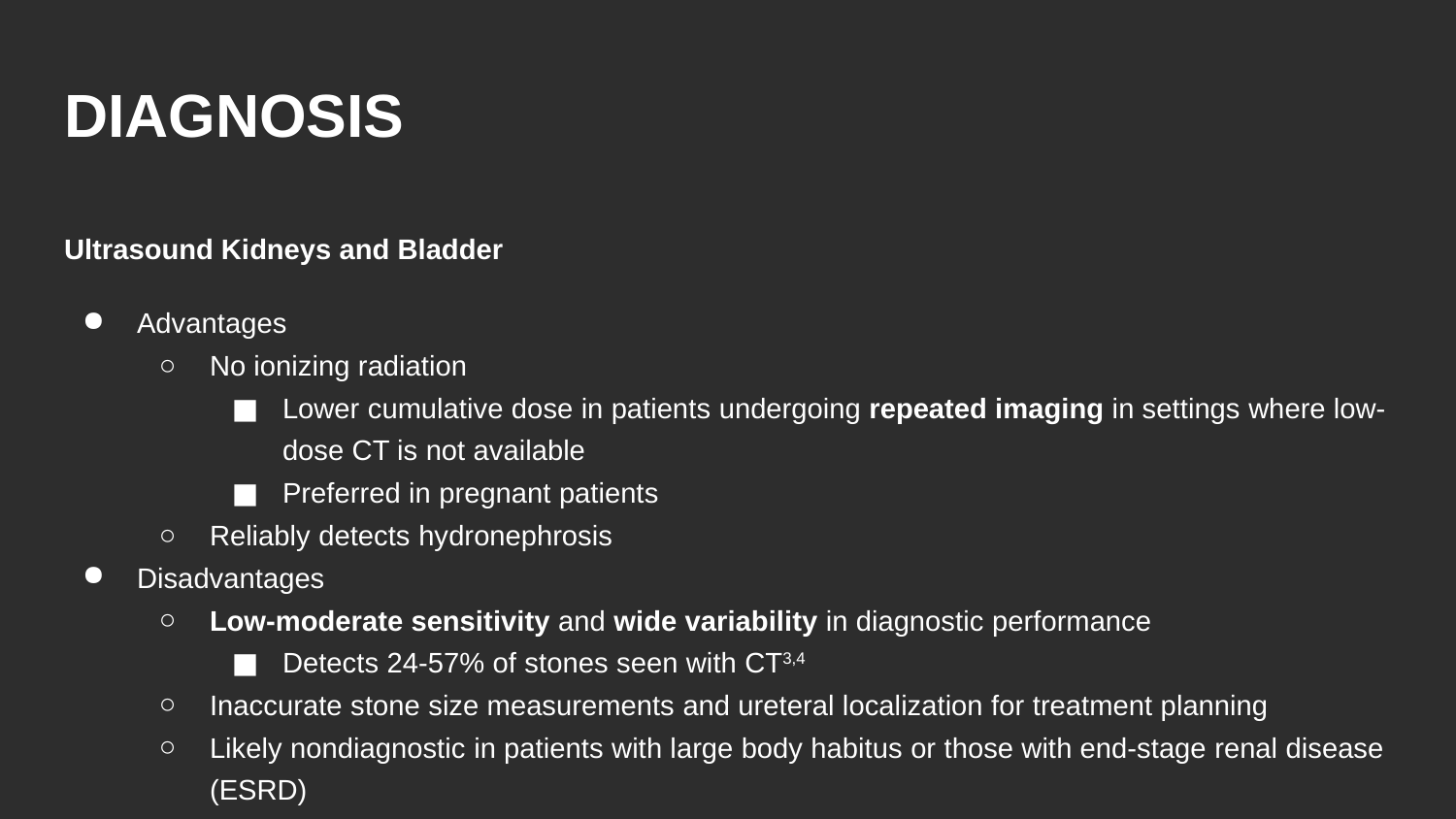

# Diagnosis
Ultrasound Kidneys and Bladder
Advantages
No ionizing radiation
Lower cumulative dose in patients undergoing repeated imaging in settings where low-dose CT is not available
Preferred in pregnant patients
Reliably detects hydronephrosis
Disadvantages
Low-moderate sensitivity and wide variability in diagnostic performance
Detects 24-57% of stones seen with CT3,4
Inaccurate stone size measurements and ureteral localization for treatment planning
Likely nondiagnostic in patients with large body habitus or those with end-stage renal disease (ESRD)

## Slide 8
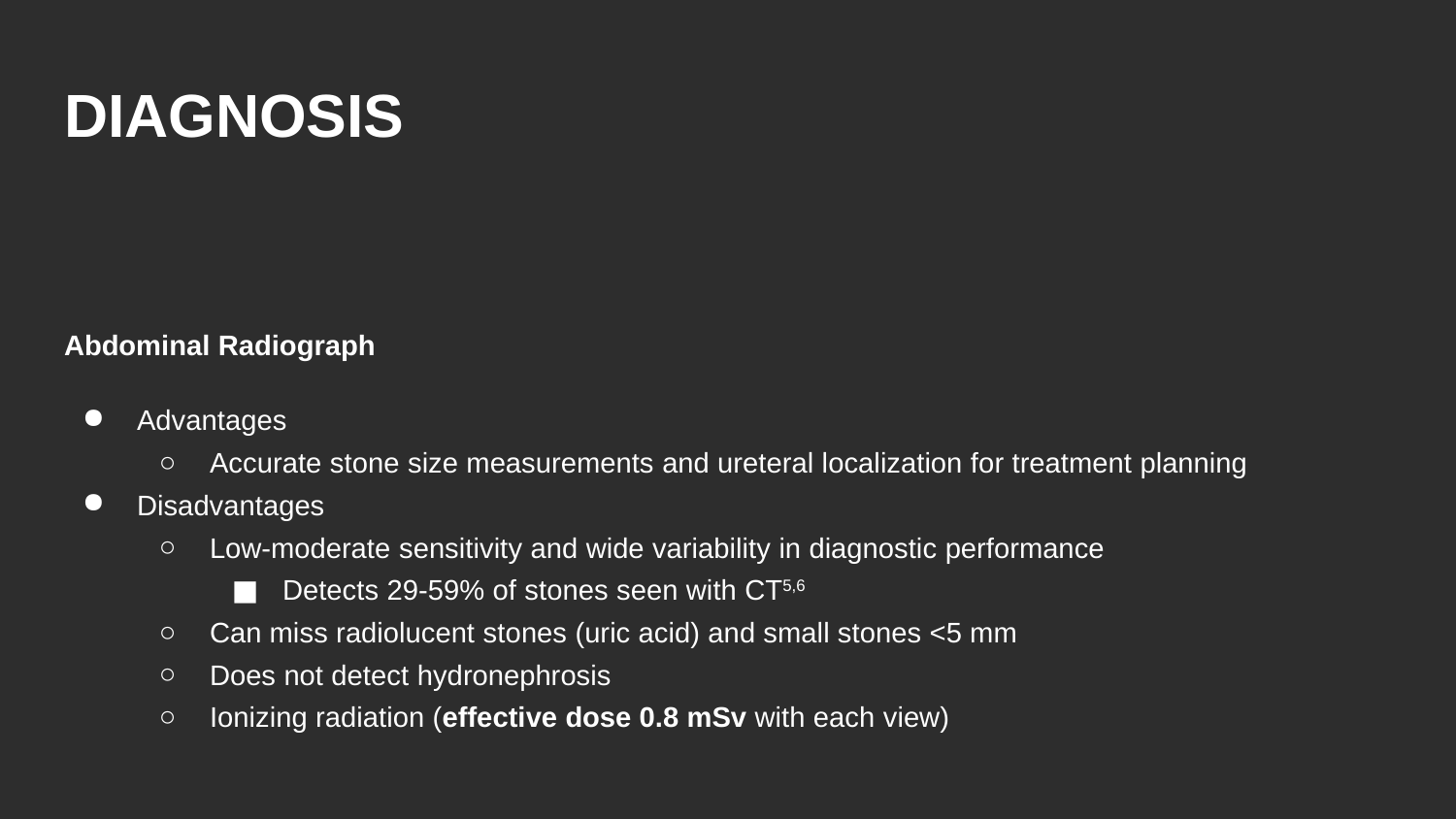

# Diagnosis
Abdominal Radiograph
Advantages
Accurate stone size measurements and ureteral localization for treatment planning
Disadvantages
Low-moderate sensitivity and wide variability in diagnostic performance
Detects 29-59% of stones seen with CT5,6
Can miss radiolucent stones (uric acid) and small stones <5 mm
Does not detect hydronephrosis
Ionizing radiation (effective dose 0.8 mSv with each view)

## Slide 9
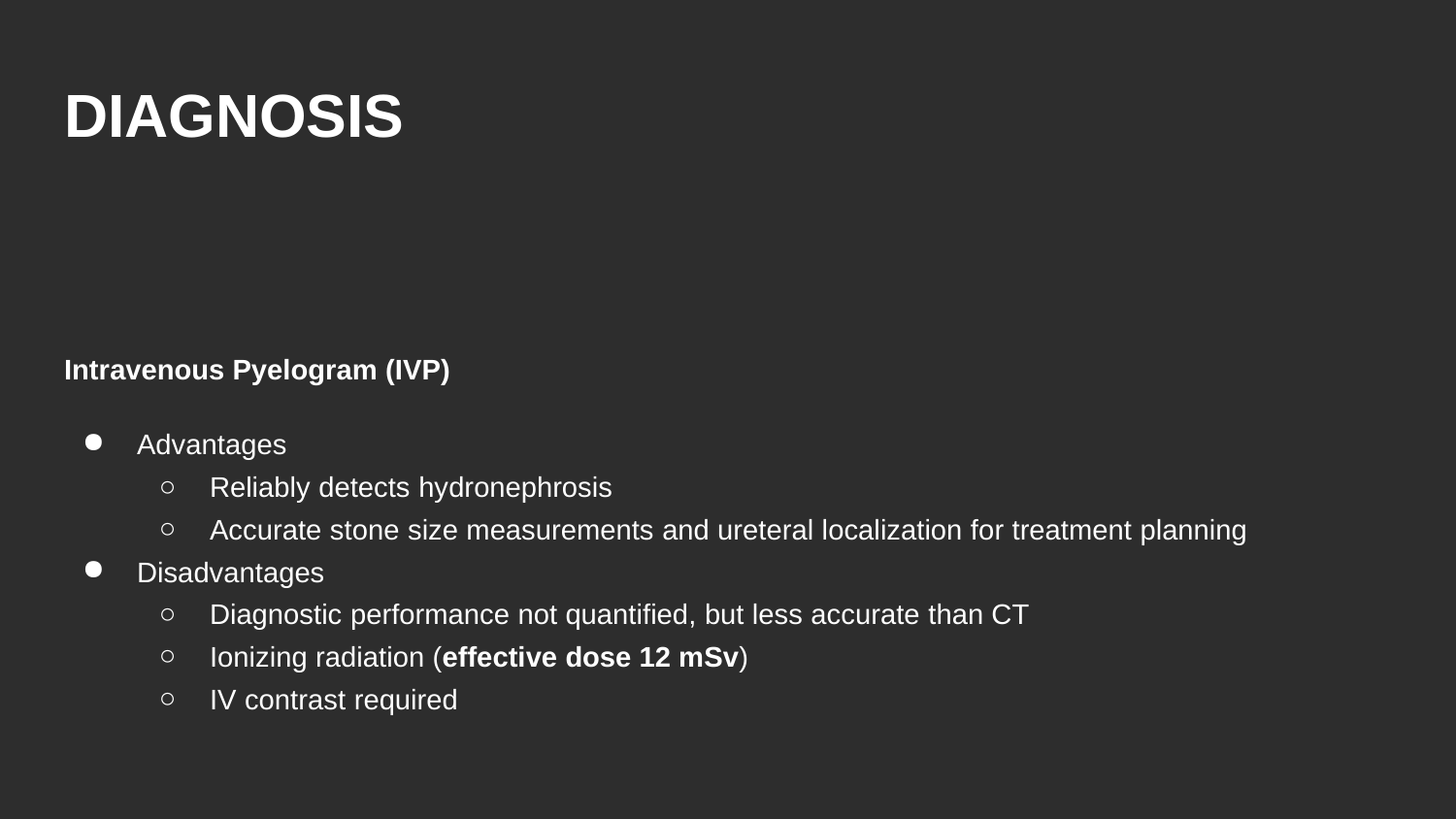

# Diagnosis
Intravenous Pyelogram (IVP)
Advantages
Reliably detects hydronephrosis
Accurate stone size measurements and ureteral localization for treatment planning
Disadvantages
Diagnostic performance not quantified, but less accurate than CT
Ionizing radiation (effective dose 12 mSv)
IV contrast required

## Slide 10
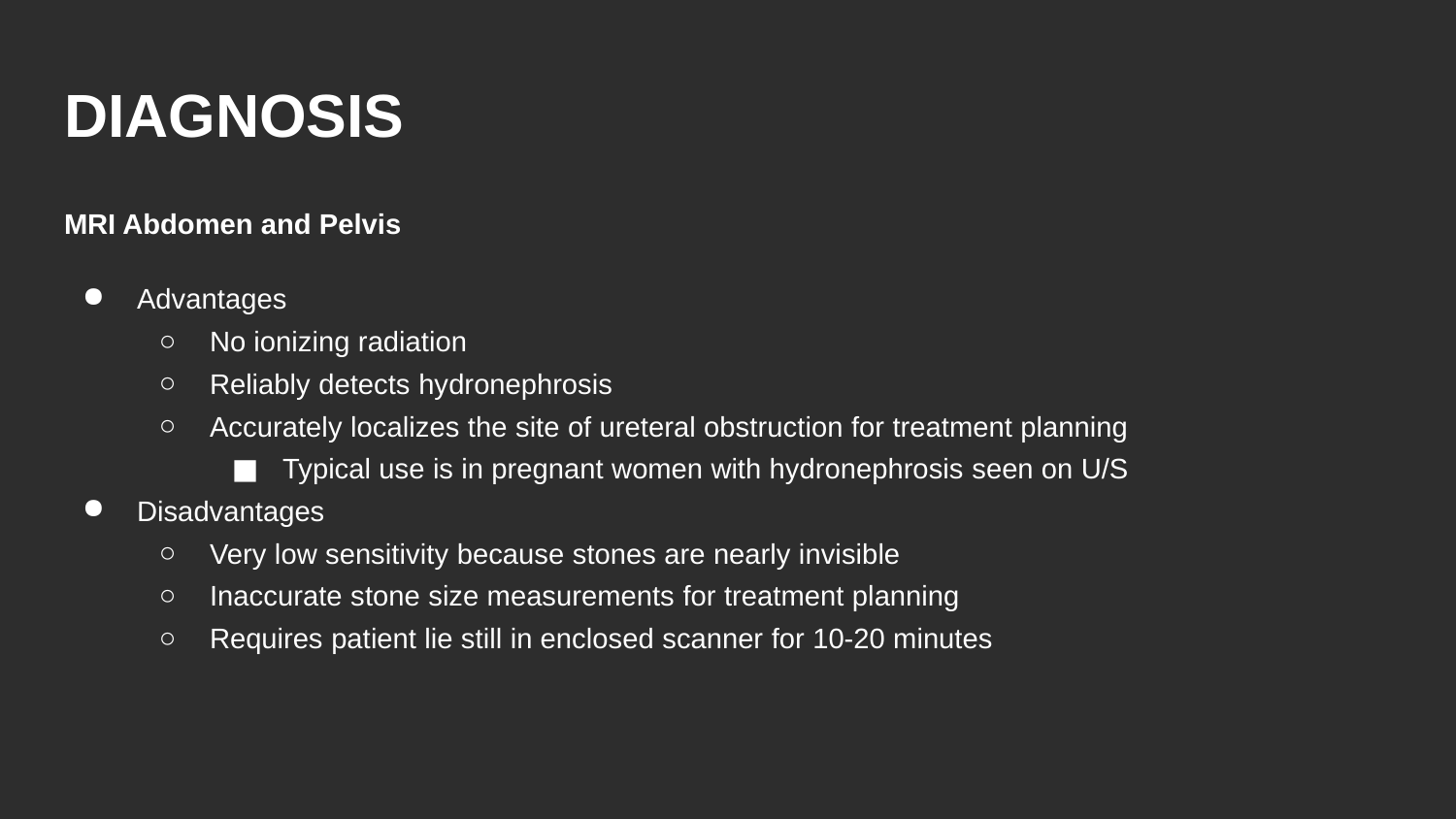

# Diagnosis
MRI Abdomen and Pelvis
Advantages
No ionizing radiation
Reliably detects hydronephrosis
Accurately localizes the site of ureteral obstruction for treatment planning
Typical use is in pregnant women with hydronephrosis seen on U/S
Disadvantages
Very low sensitivity because stones are nearly invisible
Inaccurate stone size measurements for treatment planning
Requires patient lie still in enclosed scanner for 10-20 minutes

## Slide 11
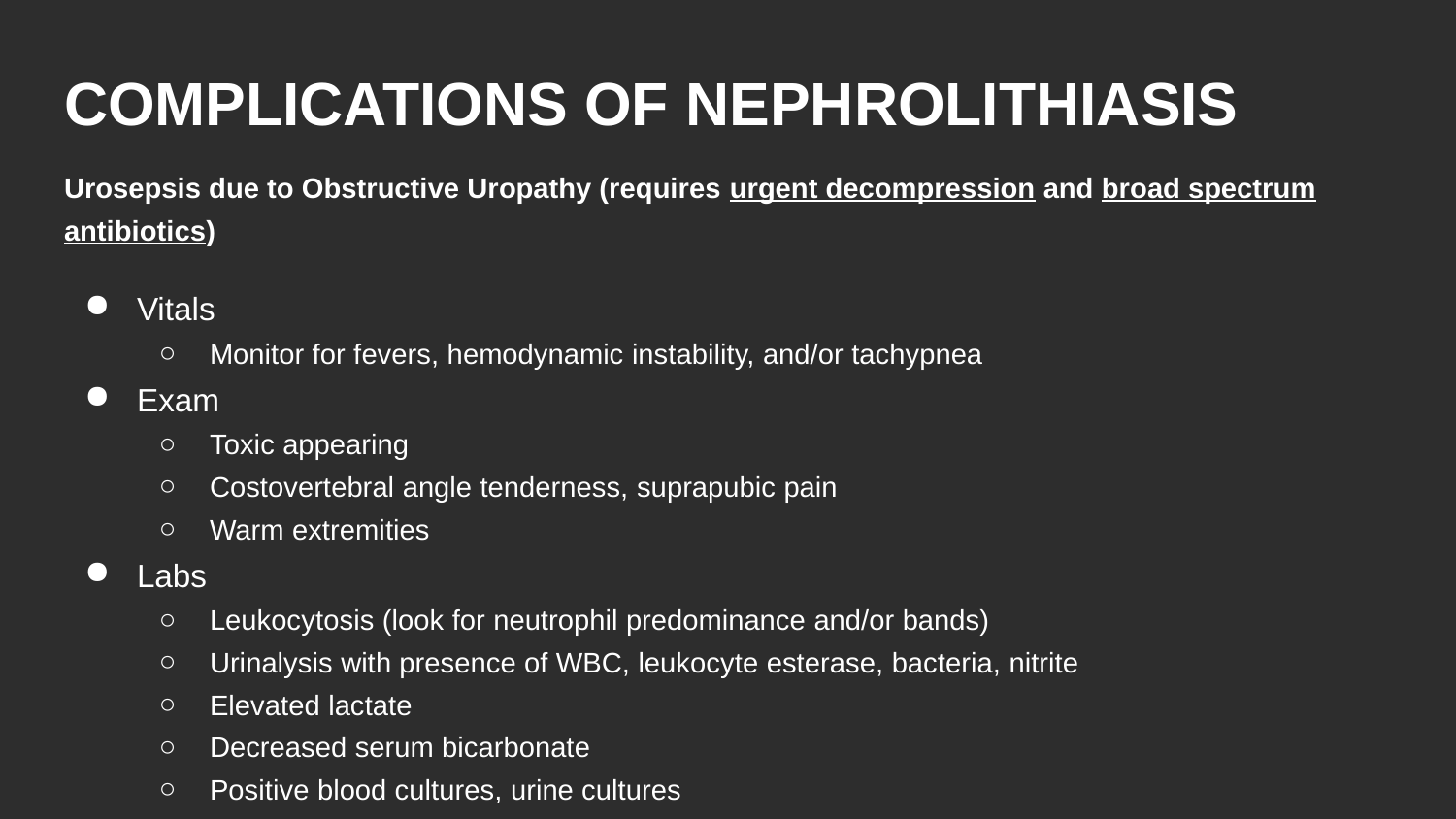

# Complications of Nephrolithiasis
Urosepsis due to Obstructive Uropathy (requires urgent decompression and broad spectrum antibiotics)
Vitals
Monitor for fevers, hemodynamic instability, and/or tachypnea
Exam
Toxic appearing
Costovertebral angle tenderness, suprapubic pain
Warm extremities
Labs
Leukocytosis (look for neutrophil predominance and/or bands)
Urinalysis with presence of WBC, leukocyte esterase, bacteria, nitrite
Elevated lactate
Decreased serum bicarbonate
Positive blood cultures, urine cultures

## Slide 12
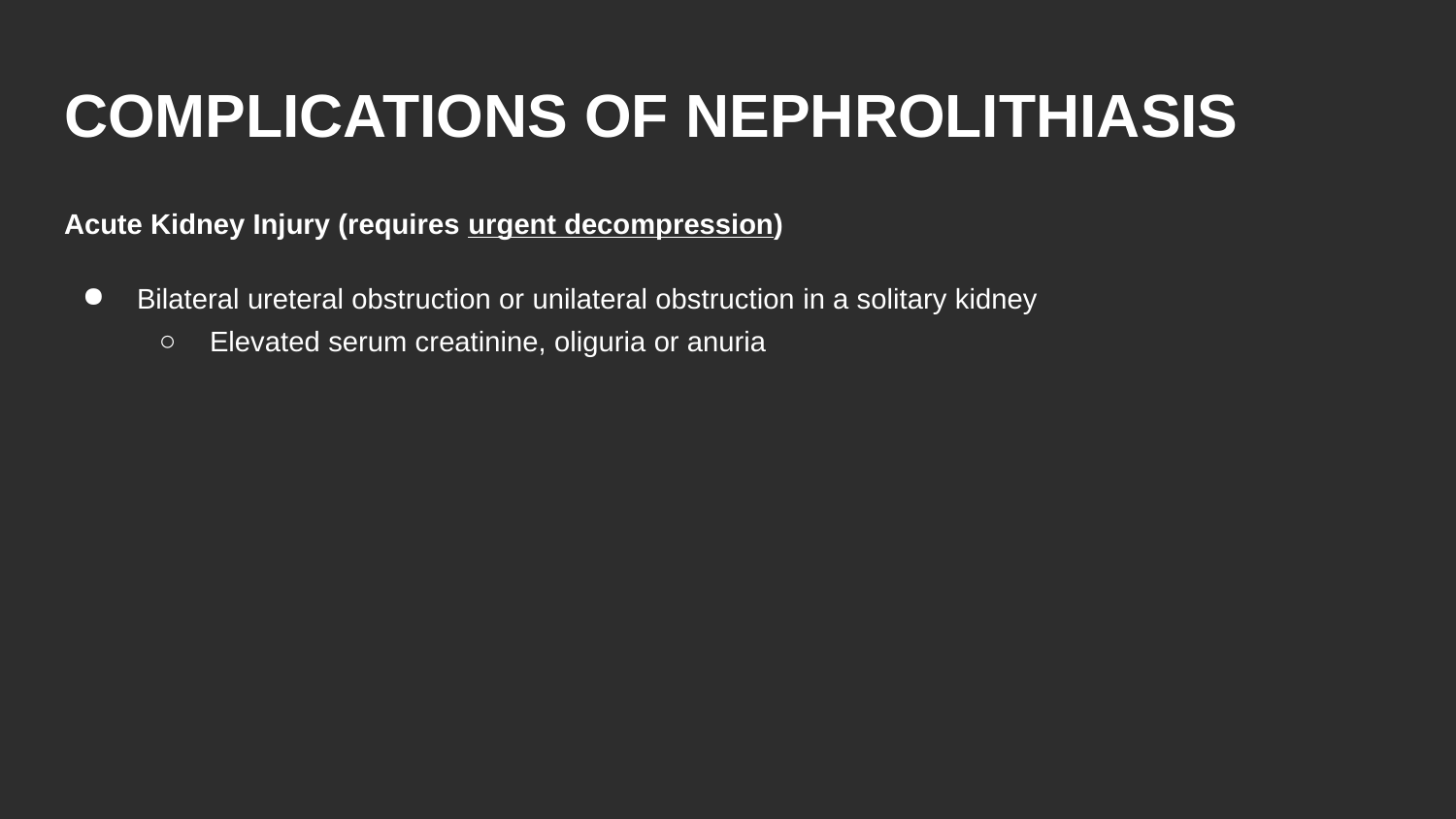

# Complications of Nephrolithiasis
Acute Kidney Injury (requires urgent decompression)
Bilateral ureteral obstruction or unilateral obstruction in a solitary kidney
Elevated serum creatinine, oliguria or anuria

## Slide 13
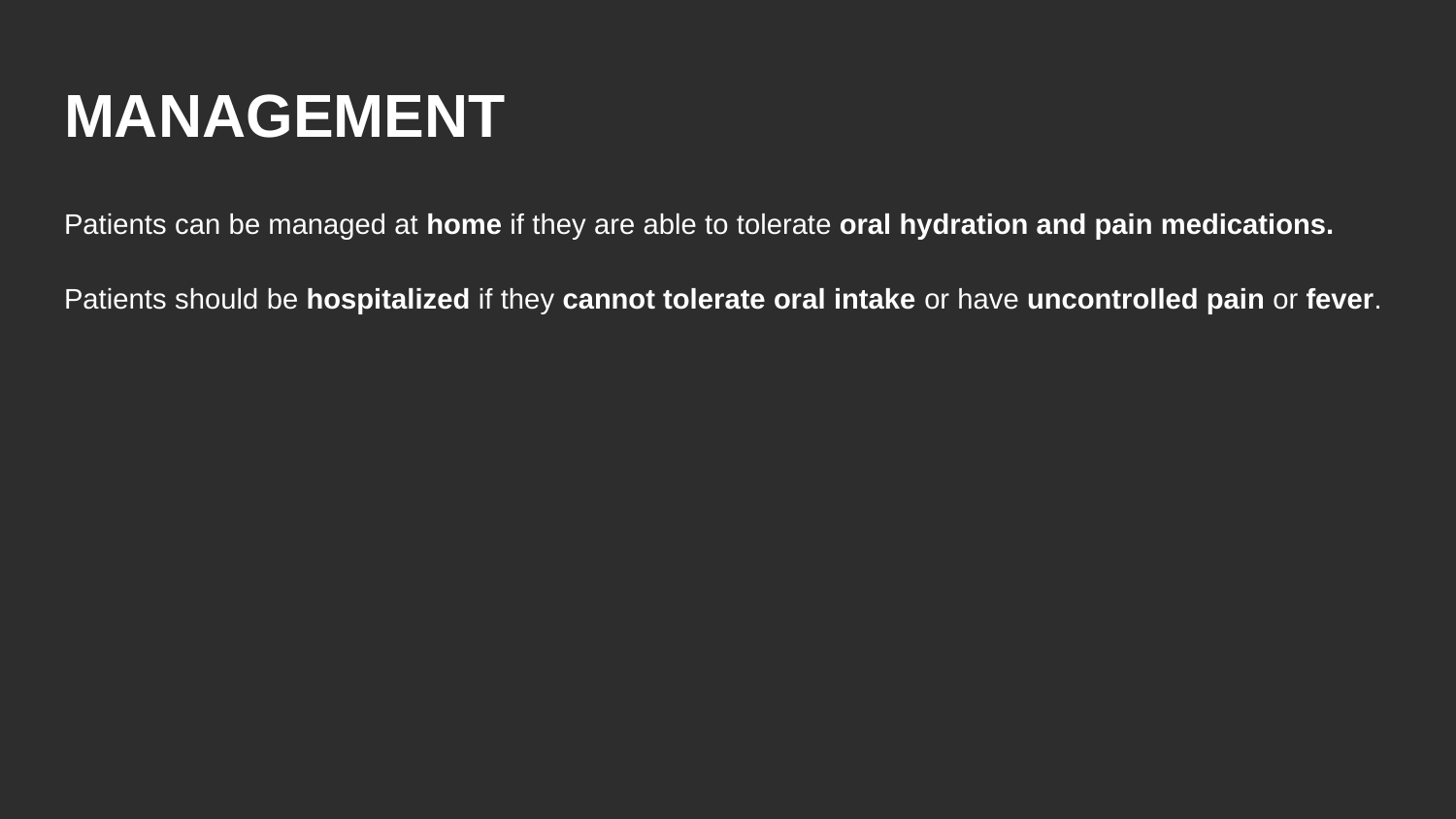

# Management
Patients can be managed at home if they are able to tolerate oral hydration and pain medications.
Patients should be hospitalized if they cannot tolerate oral intake or have uncontrolled pain or fever.

## Slide 14
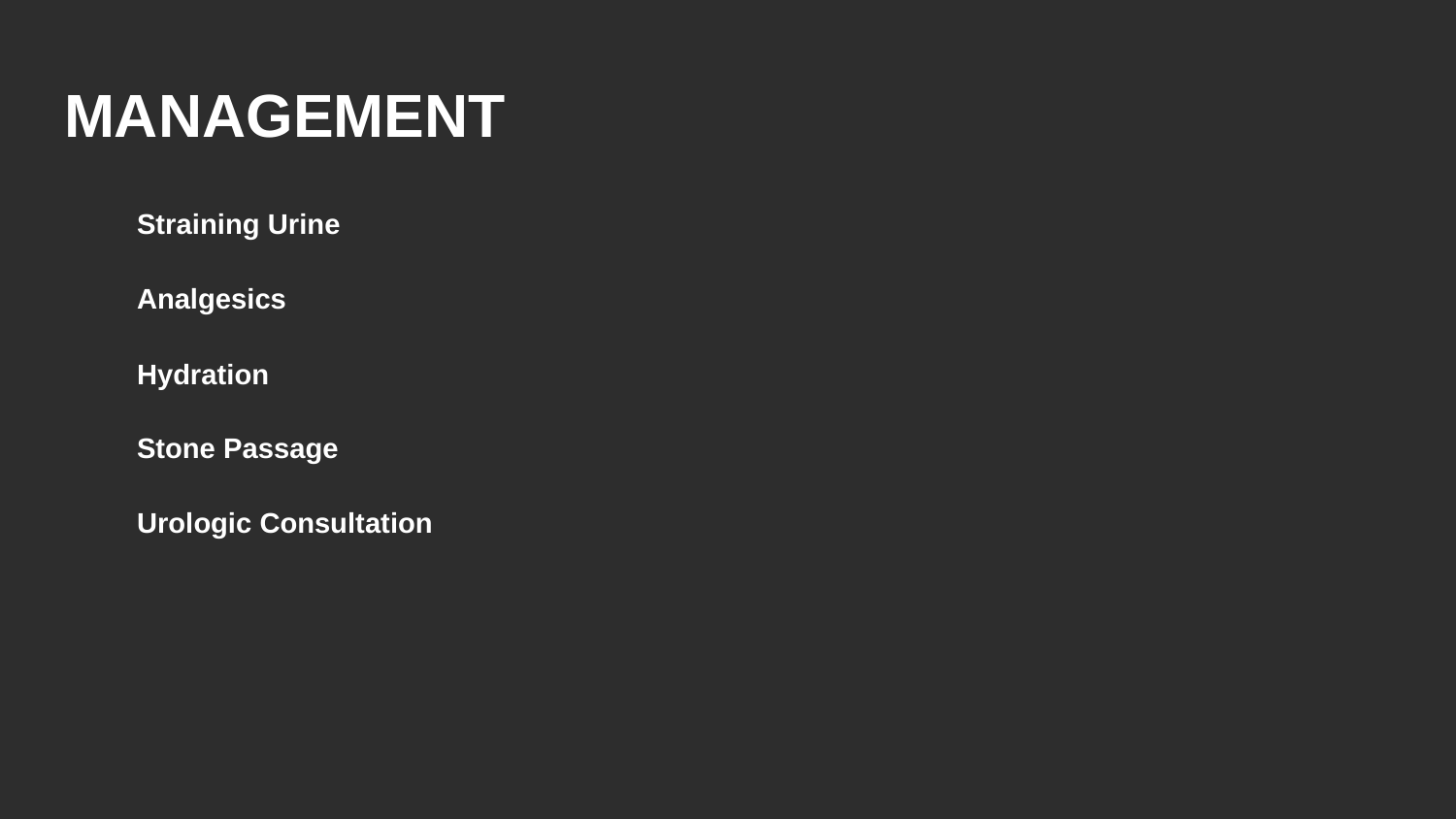

# Management
Straining Urine
Analgesics
Hydration
Stone Passage
Urologic Consultation

## Slide 15
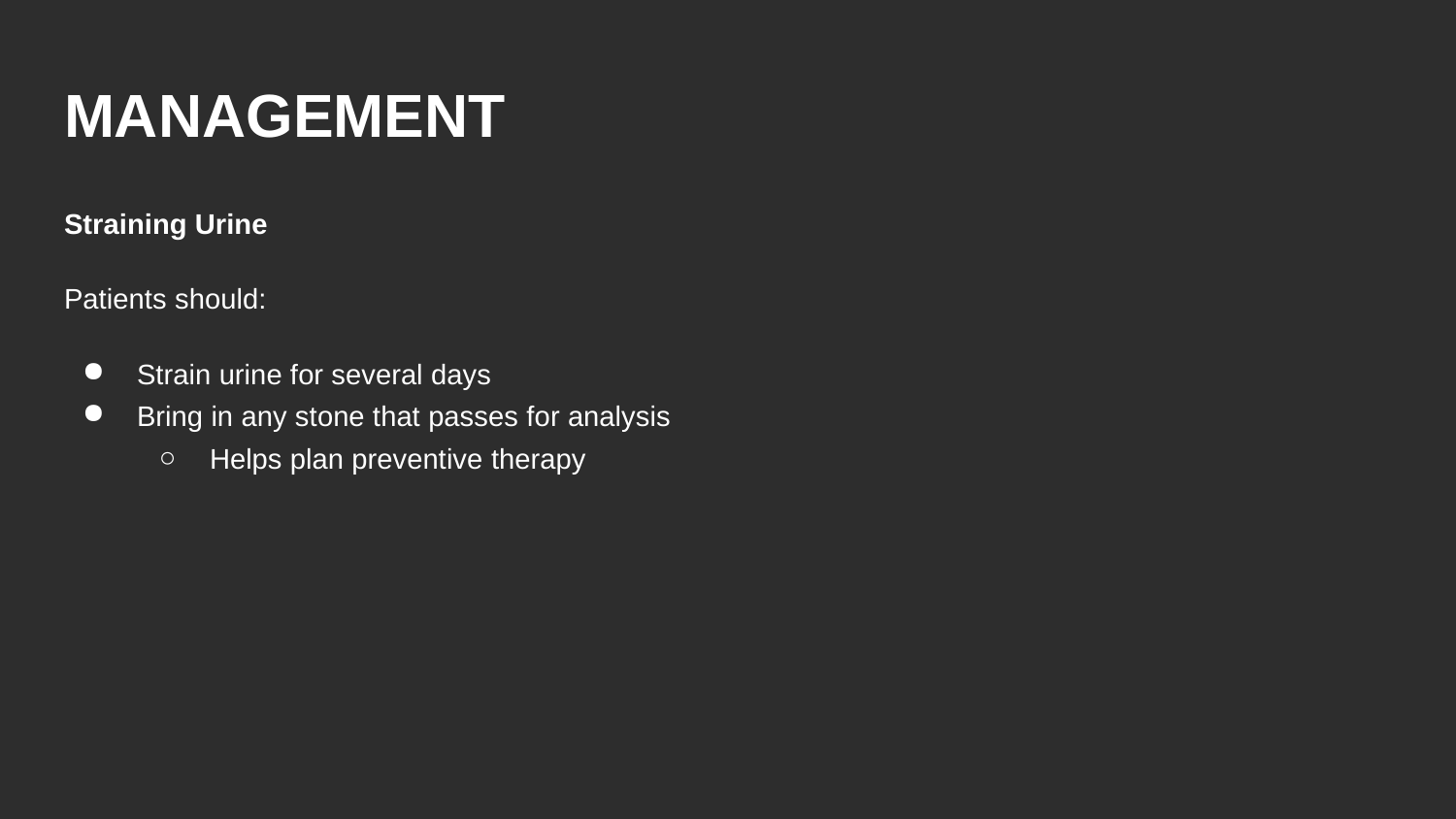

# Management
Straining Urine
Patients should:
Strain urine for several days
Bring in any stone that passes for analysis
Helps plan preventive therapy

## Slide 16
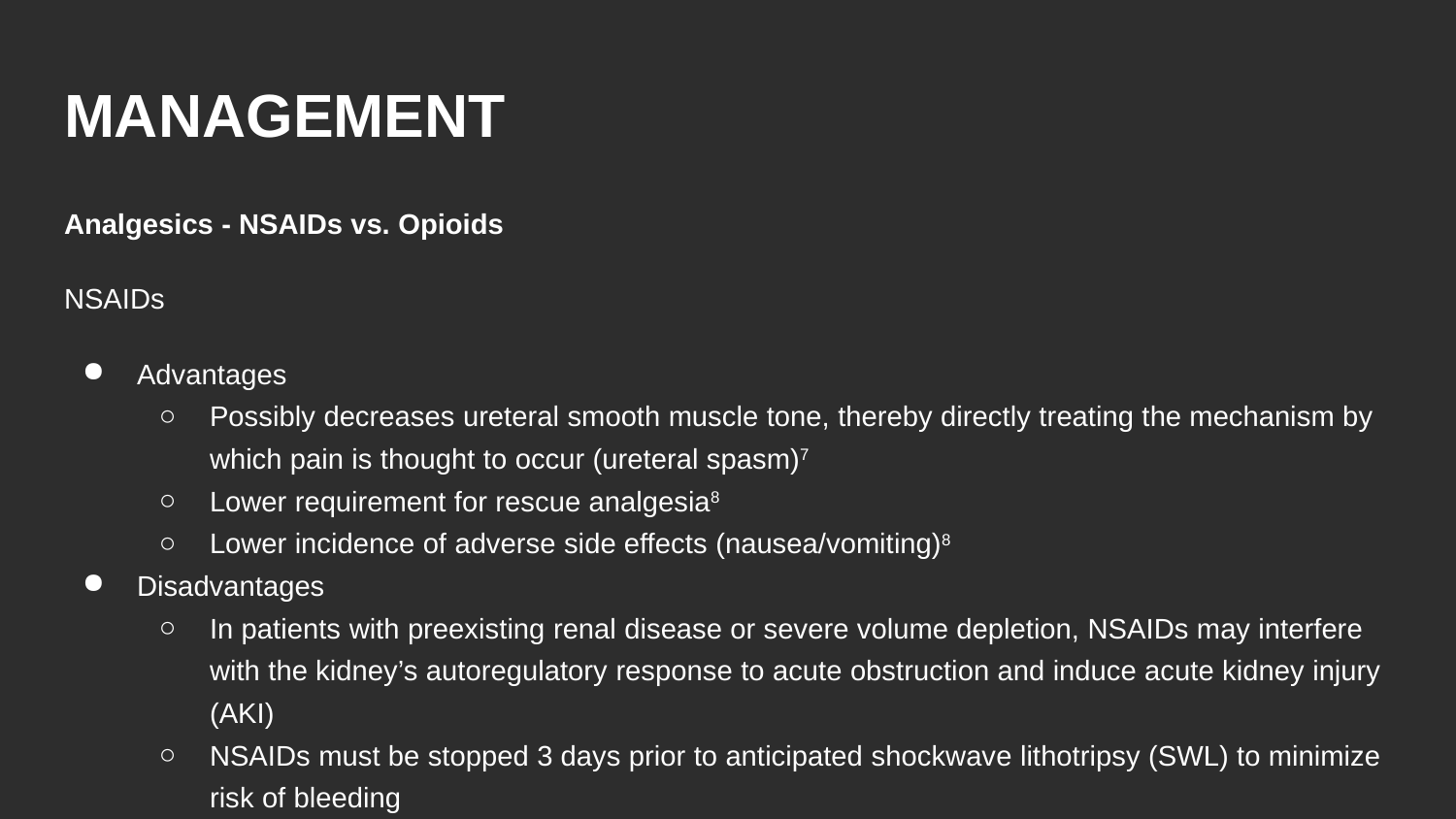

# Management
Analgesics - NSAIDs vs. Opioids
NSAIDs
Advantages
Possibly decreases ureteral smooth muscle tone, thereby directly treating the mechanism by which pain is thought to occur (ureteral spasm)7
Lower requirement for rescue analgesia8
Lower incidence of adverse side effects (nausea/vomiting)8
Disadvantages
In patients with preexisting renal disease or severe volume depletion, NSAIDs may interfere with the kidney’s autoregulatory response to acute obstruction and induce acute kidney injury (AKI)
NSAIDs must be stopped 3 days prior to anticipated shockwave lithotripsy (SWL) to minimize risk of bleeding

## Slide 17
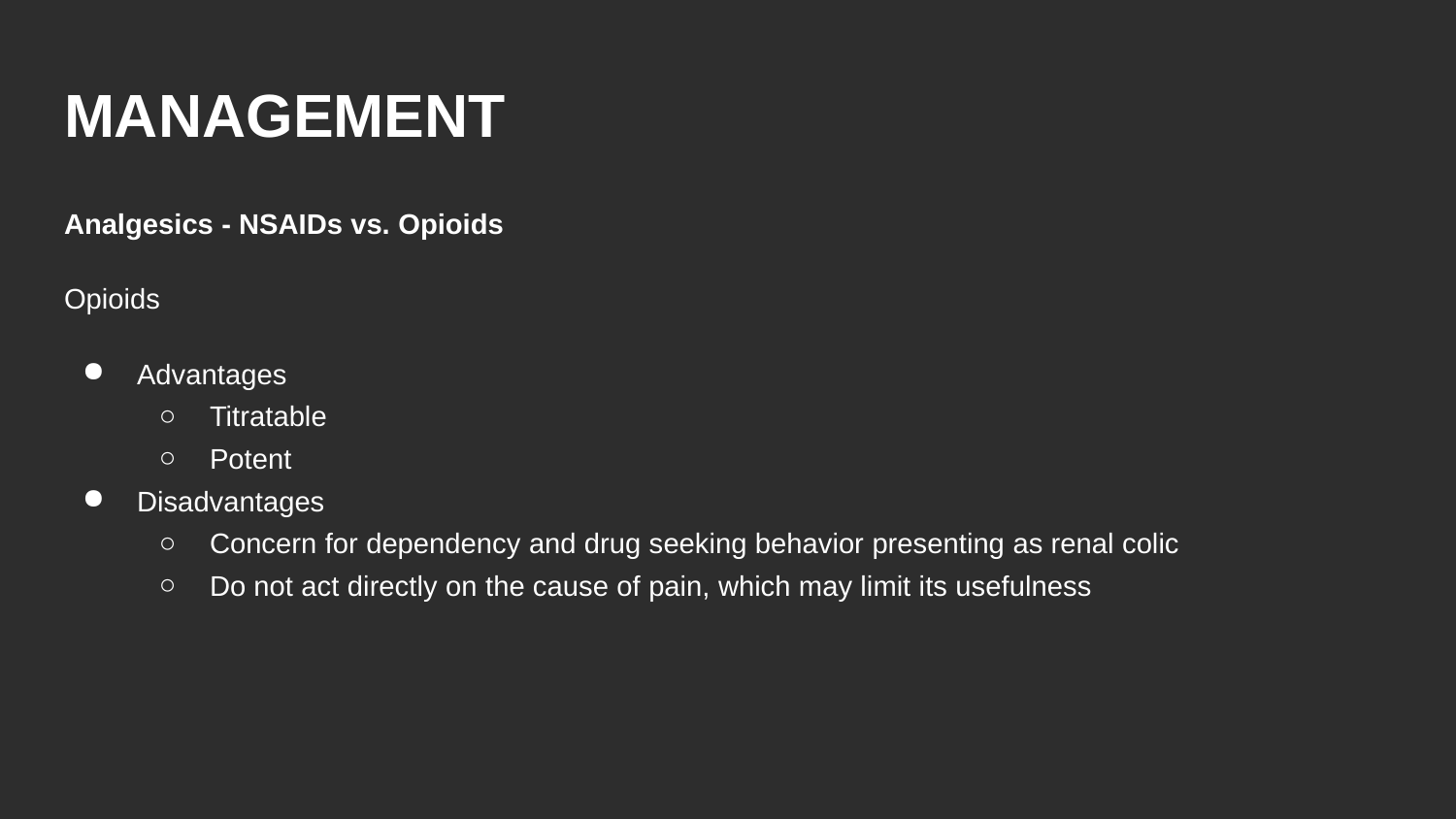

# Management
Analgesics - NSAIDs vs. Opioids
Opioids
Advantages
Titratable
Potent
Disadvantages
Concern for dependency and drug seeking behavior presenting as renal colic
Do not act directly on the cause of pain, which may limit its usefulness

## Slide 18
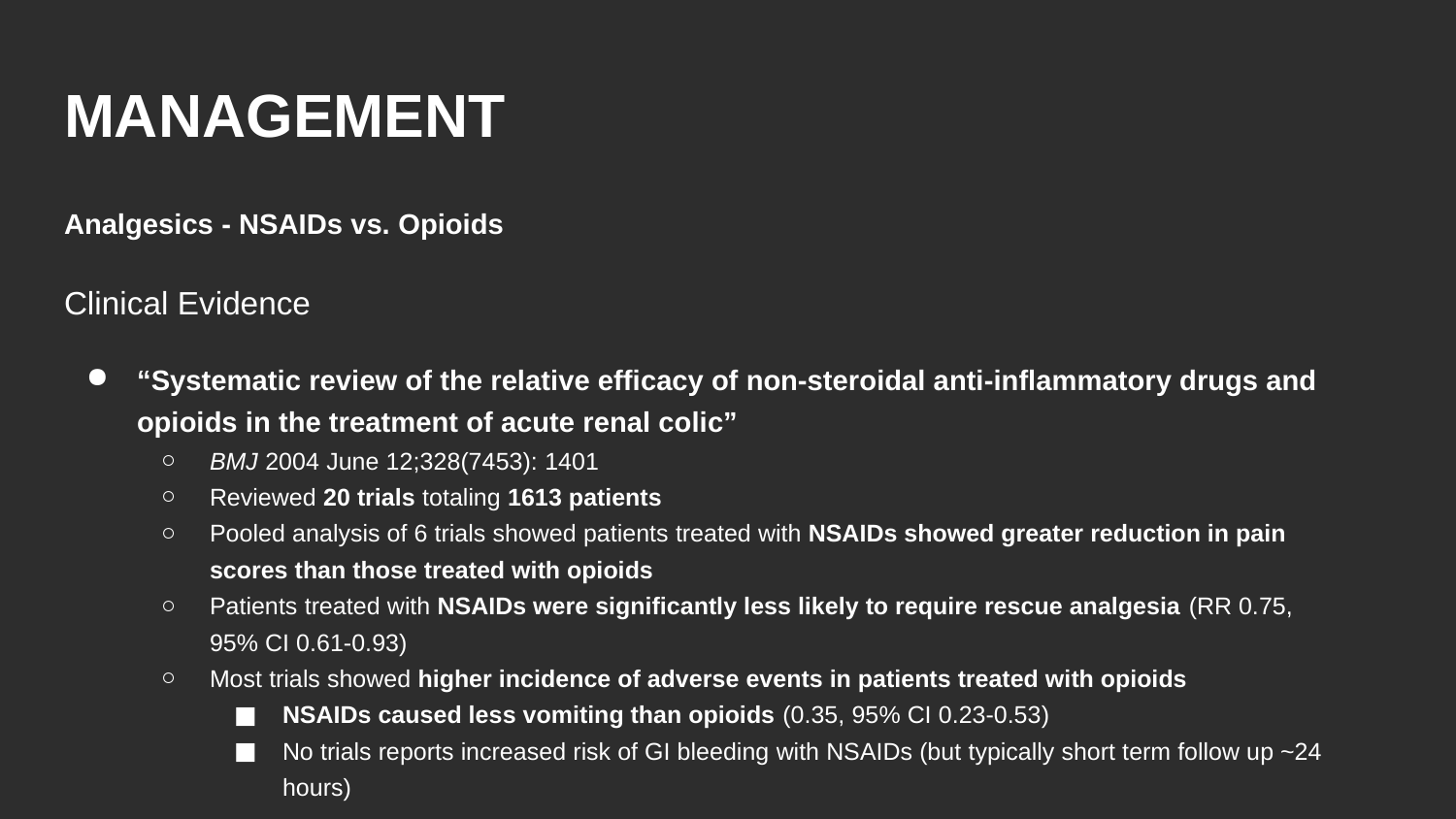

# Management
Analgesics - NSAIDs vs. Opioids
Clinical Evidence
“Systematic review of the relative efficacy of non-steroidal anti-inflammatory drugs and opioids in the treatment of acute renal colic”
BMJ 2004 June 12;328(7453): 1401
Reviewed 20 trials totaling 1613 patients
Pooled analysis of 6 trials showed patients treated with NSAIDs showed greater reduction in pain scores than those treated with opioids
Patients treated with NSAIDs were significantly less likely to require rescue analgesia (RR 0.75, 95% CI 0.61-0.93)
Most trials showed higher incidence of adverse events in patients treated with opioids
NSAIDs caused less vomiting than opioids (0.35, 95% CI 0.23-0.53)
No trials reports increased risk of GI bleeding with NSAIDs (but typically short term follow up ~24 hours)

## Slide 19
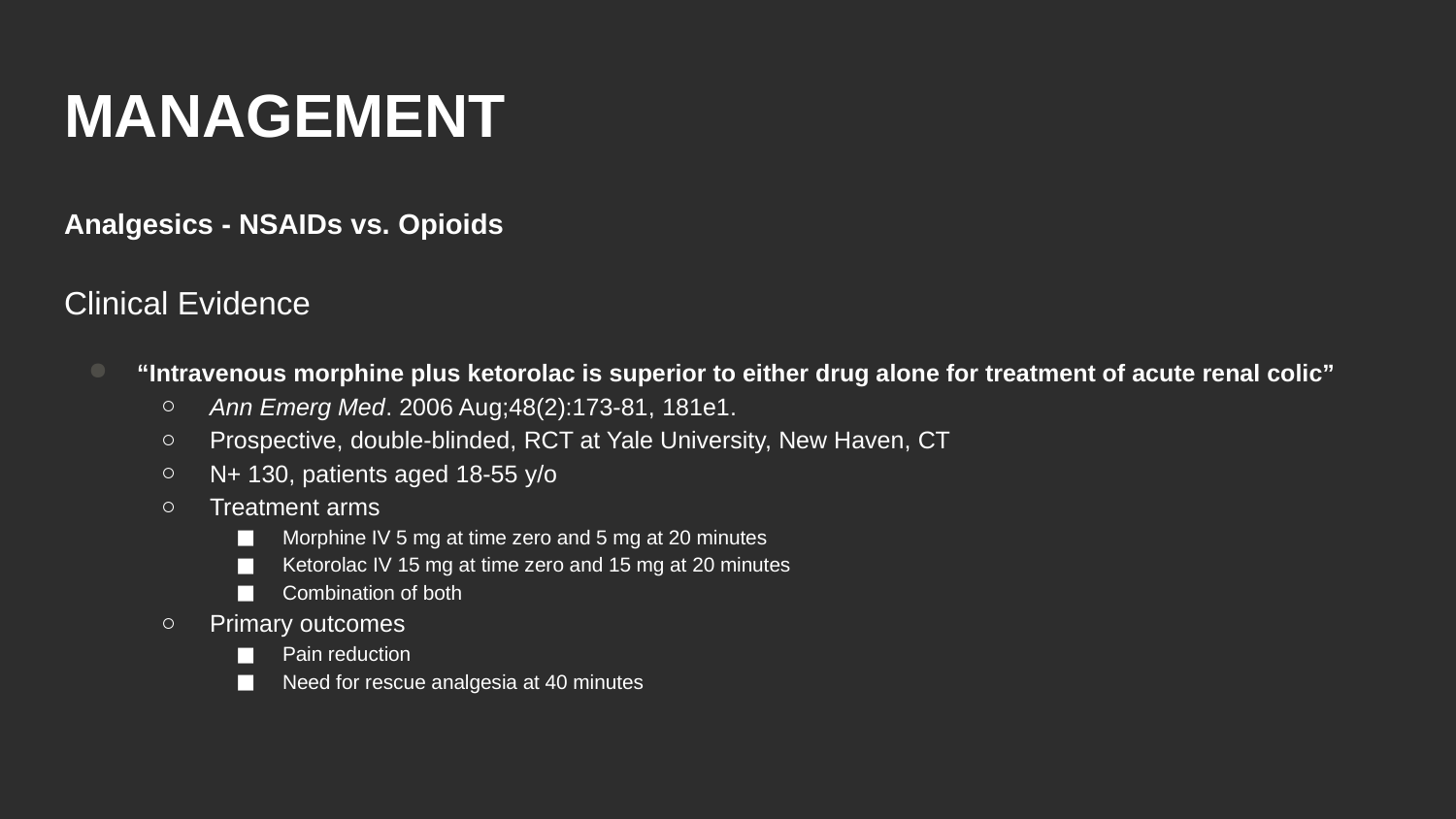

# Management
Analgesics - NSAIDs vs. Opioids
Clinical Evidence
“Intravenous morphine plus ketorolac is superior to either drug alone for treatment of acute renal colic”
Ann Emerg Med. 2006 Aug;48(2):173-81, 181e1.
Prospective, double-blinded, RCT at Yale University, New Haven, CT
N+ 130, patients aged 18-55 y/o
Treatment arms
Morphine IV 5 mg at time zero and 5 mg at 20 minutes
Ketorolac IV 15 mg at time zero and 15 mg at 20 minutes
Combination of both
Primary outcomes
Pain reduction
Need for rescue analgesia at 40 minutes

## Slide 20
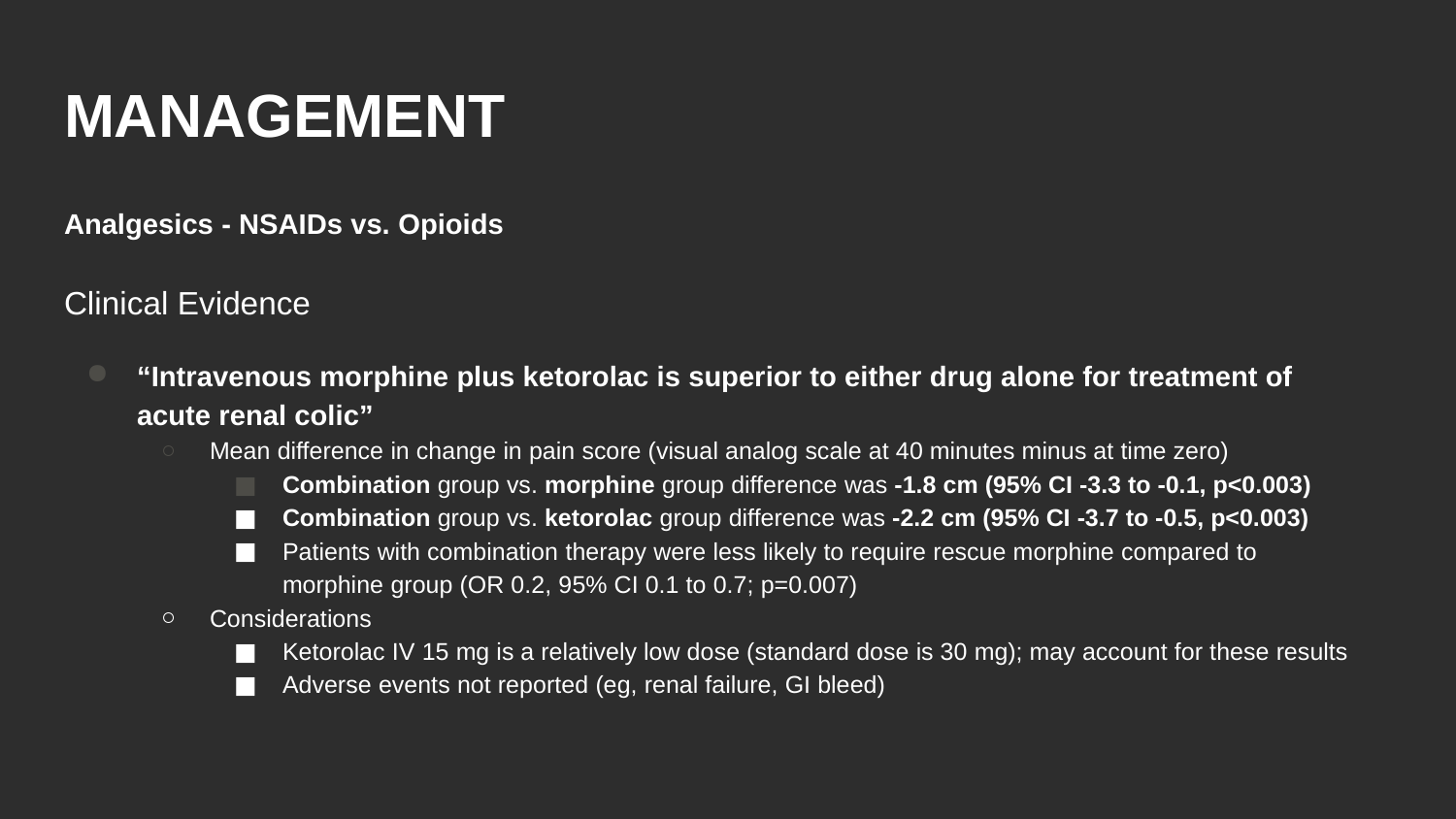

# Management
Analgesics - NSAIDs vs. Opioids
Clinical Evidence
“Intravenous morphine plus ketorolac is superior to either drug alone for treatment of acute renal colic”
Mean difference in change in pain score (visual analog scale at 40 minutes minus at time zero)
Combination group vs. morphine group difference was -1.8 cm (95% CI -3.3 to -0.1, p<0.003)
Combination group vs. ketorolac group difference was -2.2 cm (95% CI -3.7 to -0.5, p<0.003)
Patients with combination therapy were less likely to require rescue morphine compared to morphine group (OR 0.2, 95% CI 0.1 to 0.7; p=0.007)
Considerations
Ketorolac IV 15 mg is a relatively low dose (standard dose is 30 mg); may account for these results
Adverse events not reported (eg, renal failure, GI bleed)

## Slide 21
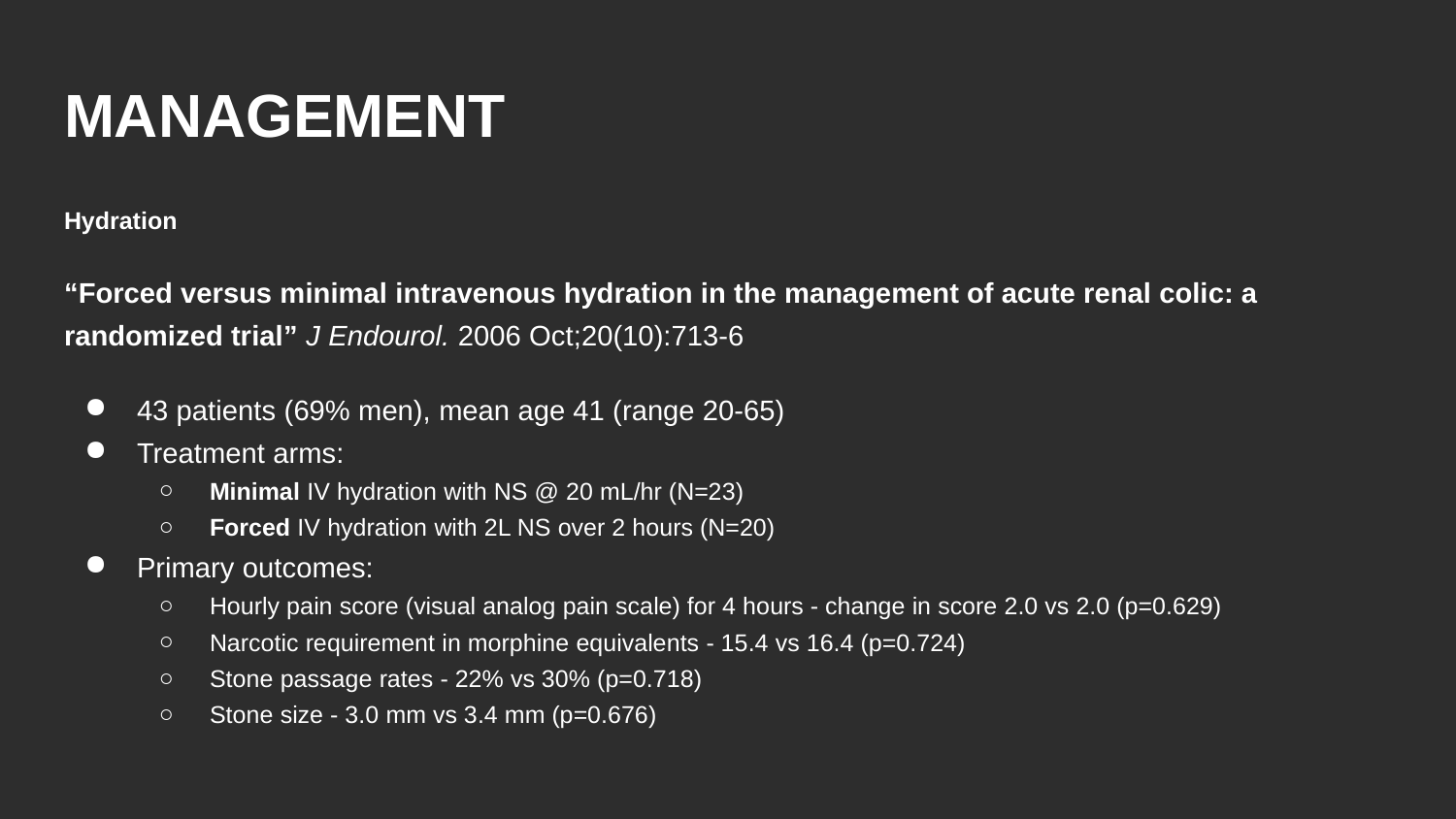

# Management
Hydration
“Forced versus minimal intravenous hydration in the management of acute renal colic: a randomized trial” J Endourol. 2006 Oct;20(10):713-6
43 patients (69% men), mean age 41 (range 20-65)
Treatment arms:
Minimal IV hydration with NS @ 20 mL/hr (N=23)
Forced IV hydration with 2L NS over 2 hours (N=20)
Primary outcomes:
Hourly pain score (visual analog pain scale) for 4 hours - change in score 2.0 vs 2.0 (p=0.629)
Narcotic requirement in morphine equivalents - 15.4 vs 16.4 (p=0.724)
Stone passage rates - 22% vs 30% (p=0.718)
Stone size - 3.0 mm vs 3.4 mm (p=0.676)

## Slide 22
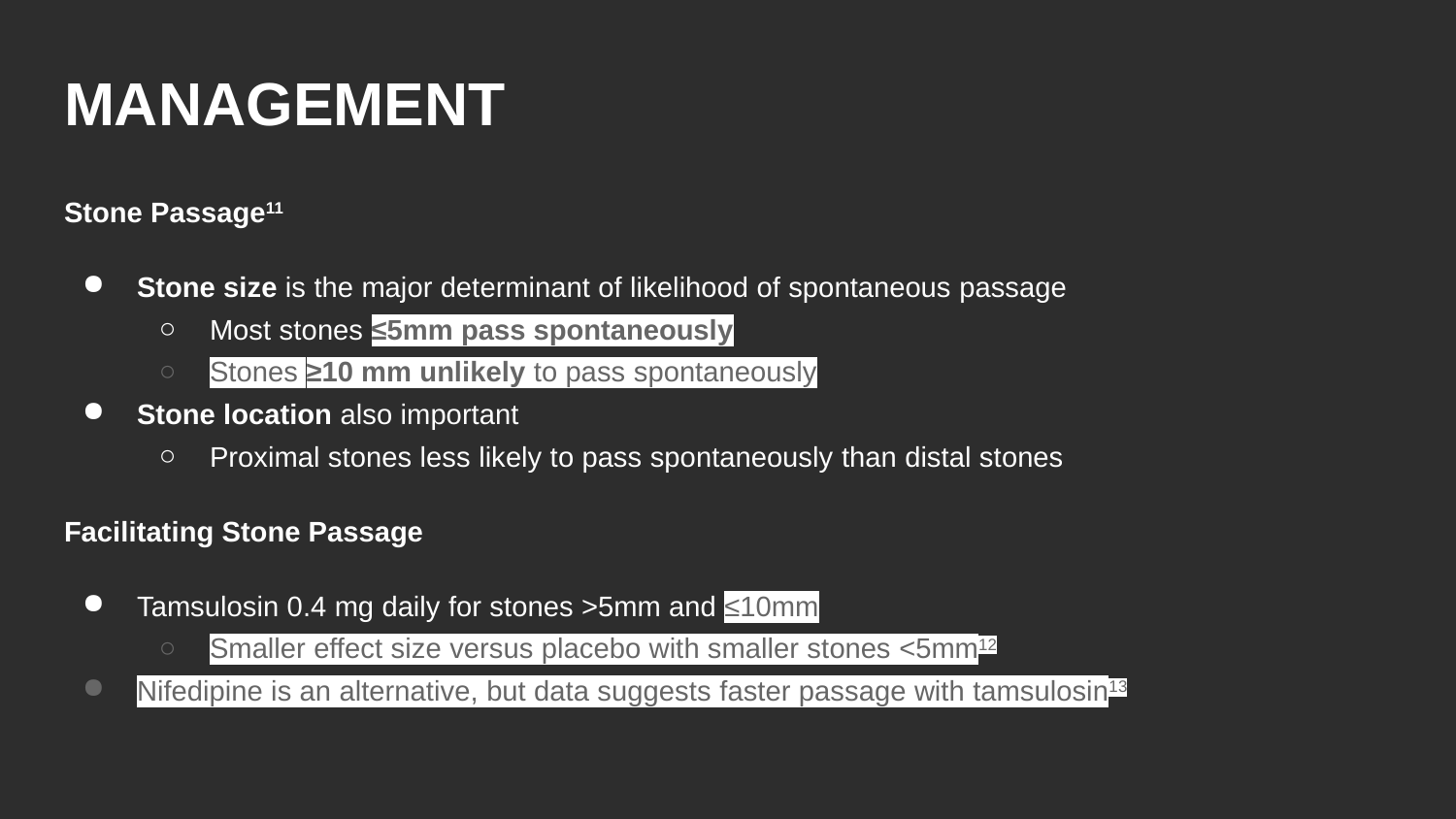

# Management
Stone Passage11
Stone size is the major determinant of likelihood of spontaneous passage
Most stones ≤5mm pass spontaneously
Stones ≥10 mm unlikely to pass spontaneously
Stone location also important
Proximal stones less likely to pass spontaneously than distal stones
Facilitating Stone Passage
Tamsulosin 0.4 mg daily for stones >5mm and ≤10mm
Smaller effect size versus placebo with smaller stones <5mm12
Nifedipine is an alternative, but data suggests faster passage with tamsulosin13

## Slide 23
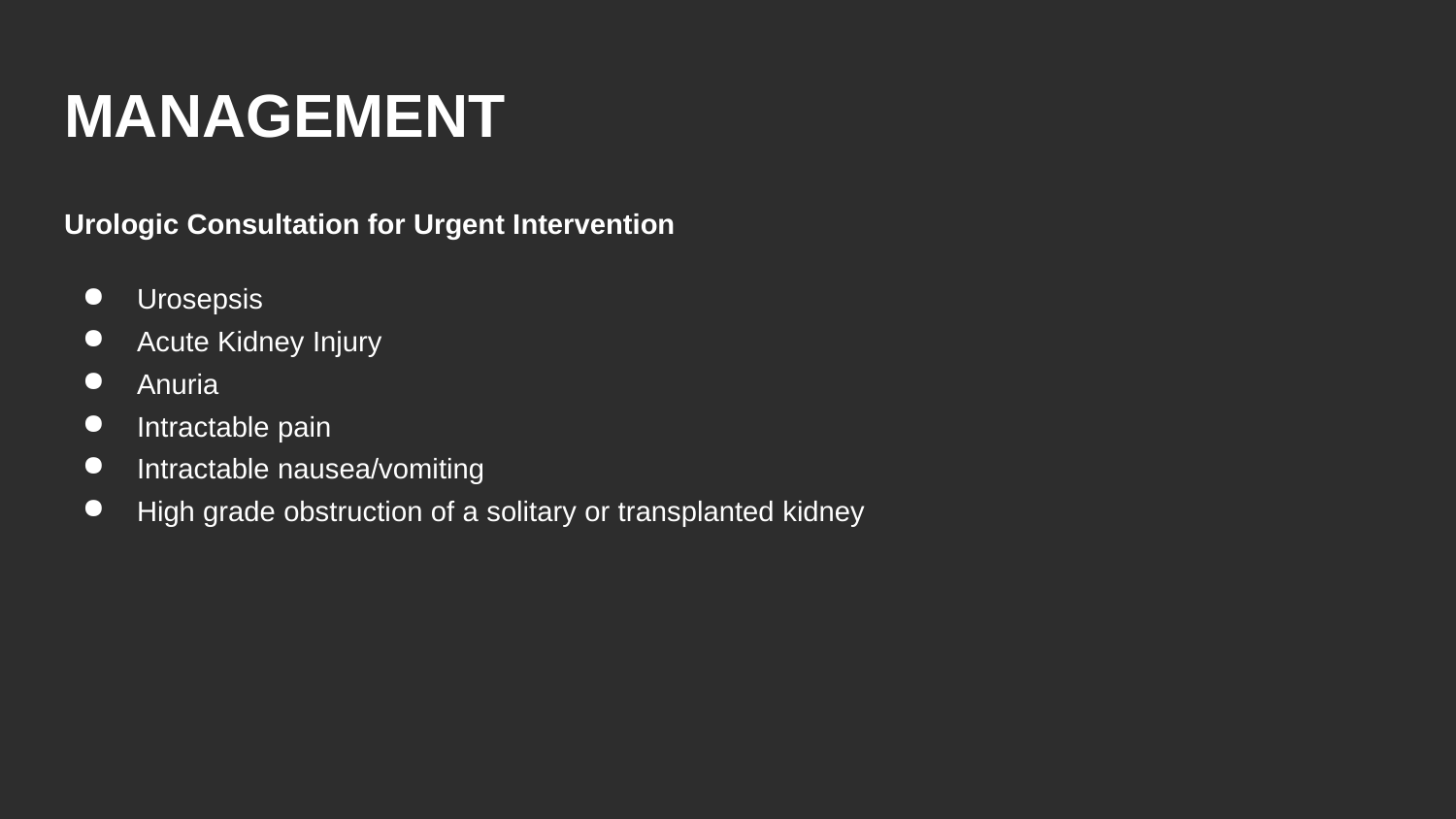

# Management
Urologic Consultation for Urgent Intervention
Urosepsis
Acute Kidney Injury
Anuria
Intractable pain
Intractable nausea/vomiting
High grade obstruction of a solitary or transplanted kidney

## Slide 24
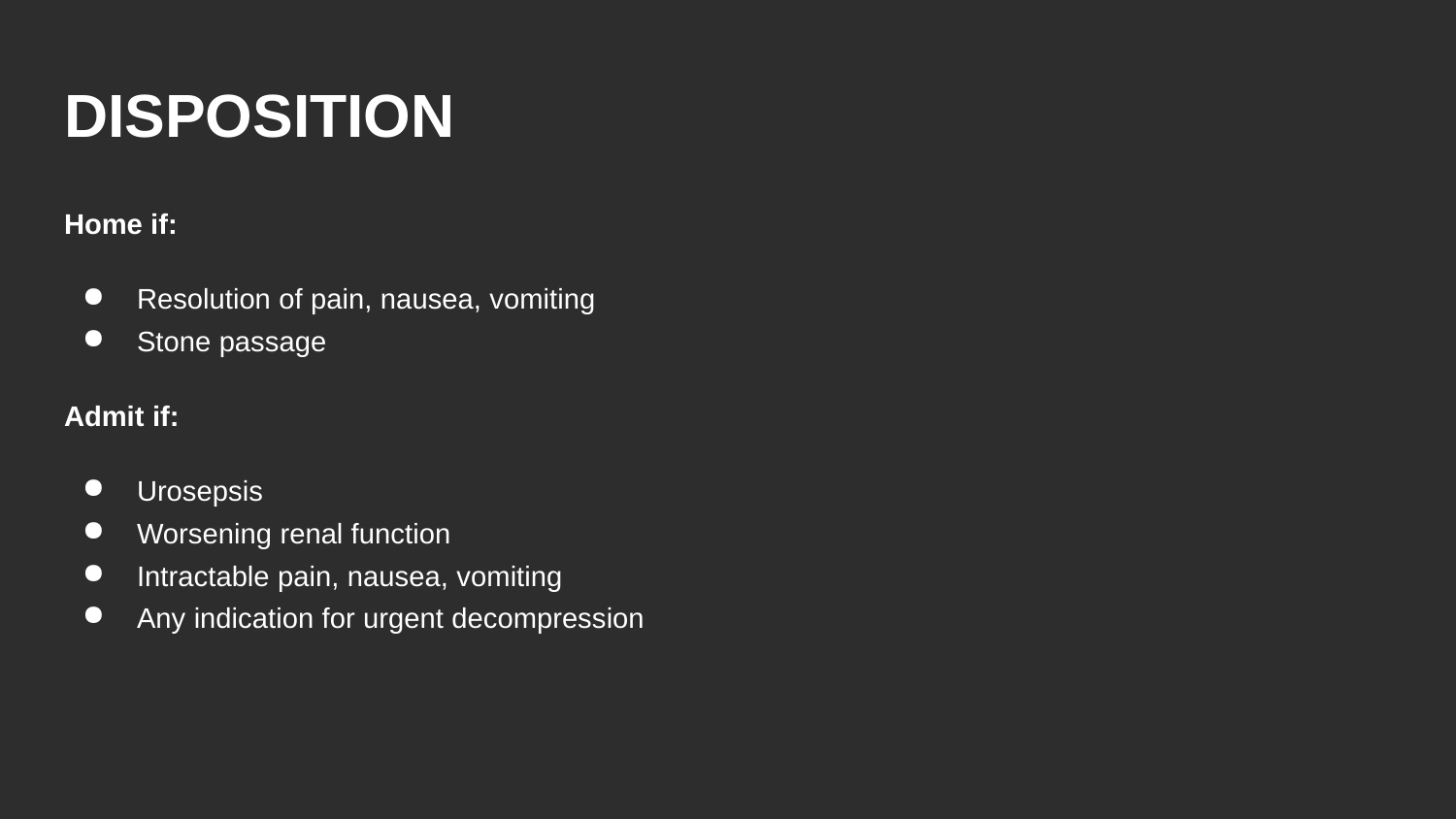

# Disposition
Home if:
Resolution of pain, nausea, vomiting
Stone passage
Admit if:
Urosepsis
Worsening renal function
Intractable pain, nausea, vomiting
Any indication for urgent decompression

## Slide 25
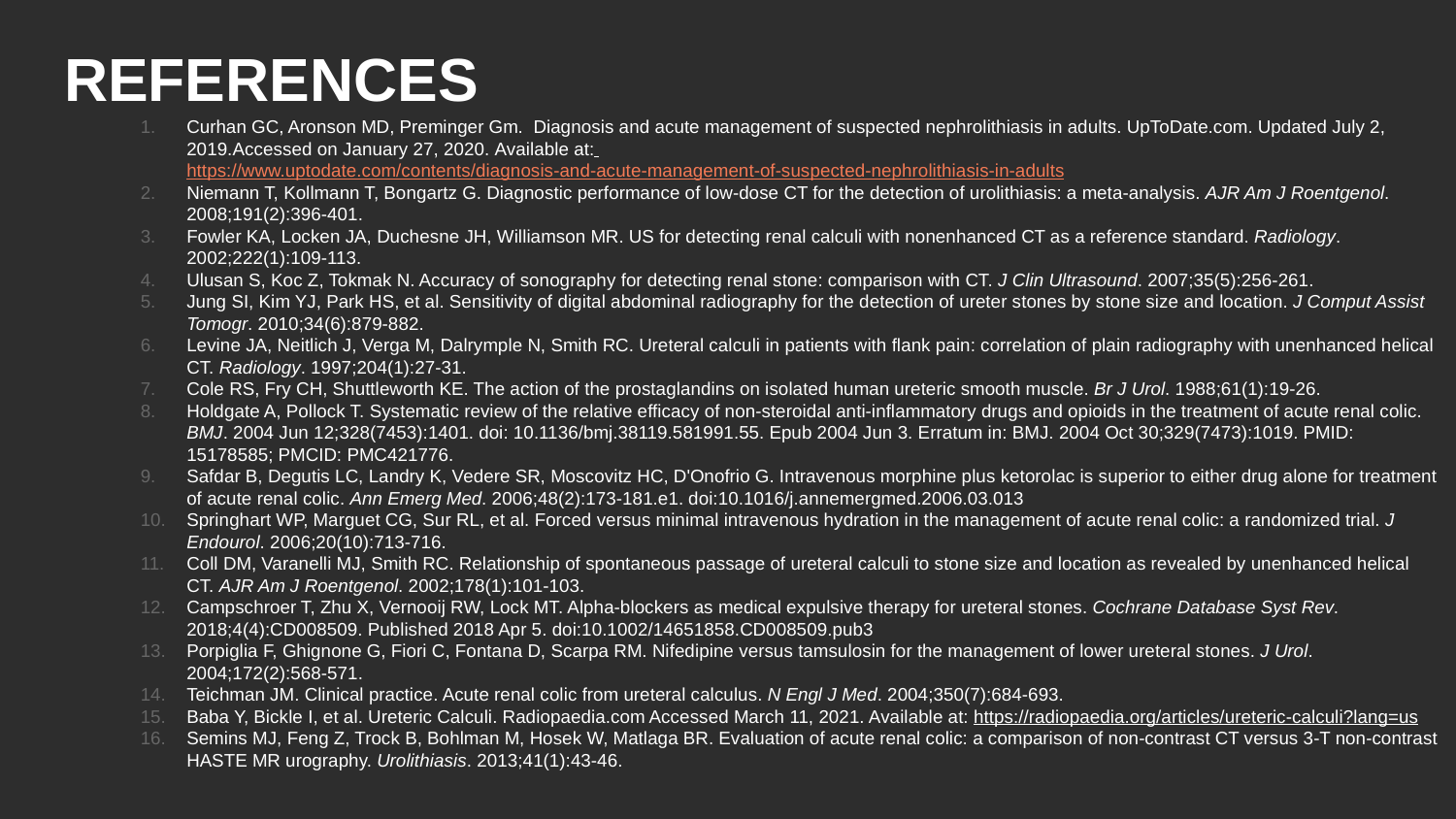

# References
Curhan GC, Aronson MD, Preminger Gm. Diagnosis and acute management of suspected nephrolithiasis in adults. UpToDate.com. Updated July 2, 2019.Accessed on January 27, 2020. Available at: https://www.uptodate.com/contents/diagnosis-and-acute-management-of-suspected-nephrolithiasis-in-adults
Niemann T, Kollmann T, Bongartz G. Diagnostic performance of low-dose CT for the detection of urolithiasis: a meta-analysis. AJR Am J Roentgenol. 2008;191(2):396-401.
Fowler KA, Locken JA, Duchesne JH, Williamson MR. US for detecting renal calculi with nonenhanced CT as a reference standard. Radiology. 2002;222(1):109-113.
Ulusan S, Koc Z, Tokmak N. Accuracy of sonography for detecting renal stone: comparison with CT. J Clin Ultrasound. 2007;35(5):256-261.
Jung SI, Kim YJ, Park HS, et al. Sensitivity of digital abdominal radiography for the detection of ureter stones by stone size and location. J Comput Assist Tomogr. 2010;34(6):879-882.
Levine JA, Neitlich J, Verga M, Dalrymple N, Smith RC. Ureteral calculi in patients with flank pain: correlation of plain radiography with unenhanced helical CT. Radiology. 1997;204(1):27-31.
Cole RS, Fry CH, Shuttleworth KE. The action of the prostaglandins on isolated human ureteric smooth muscle. Br J Urol. 1988;61(1):19-26.
Holdgate A, Pollock T. Systematic review of the relative efficacy of non-steroidal anti-inflammatory drugs and opioids in the treatment of acute renal colic. BMJ. 2004 Jun 12;328(7453):1401. doi: 10.1136/bmj.38119.581991.55. Epub 2004 Jun 3. Erratum in: BMJ. 2004 Oct 30;329(7473):1019. PMID: 15178585; PMCID: PMC421776.
Safdar B, Degutis LC, Landry K, Vedere SR, Moscovitz HC, D'Onofrio G. Intravenous morphine plus ketorolac is superior to either drug alone for treatment of acute renal colic. Ann Emerg Med. 2006;48(2):173-181.e1. doi:10.1016/j.annemergmed.2006.03.013
Springhart WP, Marguet CG, Sur RL, et al. Forced versus minimal intravenous hydration in the management of acute renal colic: a randomized trial. J Endourol. 2006;20(10):713-716.
Coll DM, Varanelli MJ, Smith RC. Relationship of spontaneous passage of ureteral calculi to stone size and location as revealed by unenhanced helical CT. AJR Am J Roentgenol. 2002;178(1):101-103.
Campschroer T, Zhu X, Vernooij RW, Lock MT. Alpha-blockers as medical expulsive therapy for ureteral stones. Cochrane Database Syst Rev. 2018;4(4):CD008509. Published 2018 Apr 5. doi:10.1002/14651858.CD008509.pub3
Porpiglia F, Ghignone G, Fiori C, Fontana D, Scarpa RM. Nifedipine versus tamsulosin for the management of lower ureteral stones. J Urol. 2004;172(2):568-571.
Teichman JM. Clinical practice. Acute renal colic from ureteral calculus. N Engl J Med. 2004;350(7):684-693.
Baba Y, Bickle I, et al. Ureteric Calculi. Radiopaedia.com Accessed March 11, 2021. Available at: https://radiopaedia.org/articles/ureteric-calculi?lang=us
Semins MJ, Feng Z, Trock B, Bohlman M, Hosek W, Matlaga BR. Evaluation of acute renal colic: a comparison of non-contrast CT versus 3-T non-contrast HASTE MR urography. Urolithiasis. 2013;41(1):43-46.
