# Supplementary material for: An Observation Medicine Curriculum for Emergency Medicine Education
Source: J Educ Teach Emerg Med. 2021 Apr 19;6(2):C1–C72. doi: 10.21980/J87P92 (PMC10332786; doi:10.21980/J87P92)
Supplement: Supplementary file 13 — Please see associated PowerPoint file [file jetem-6-2-c1-supp13.pptx]

## Slide 1
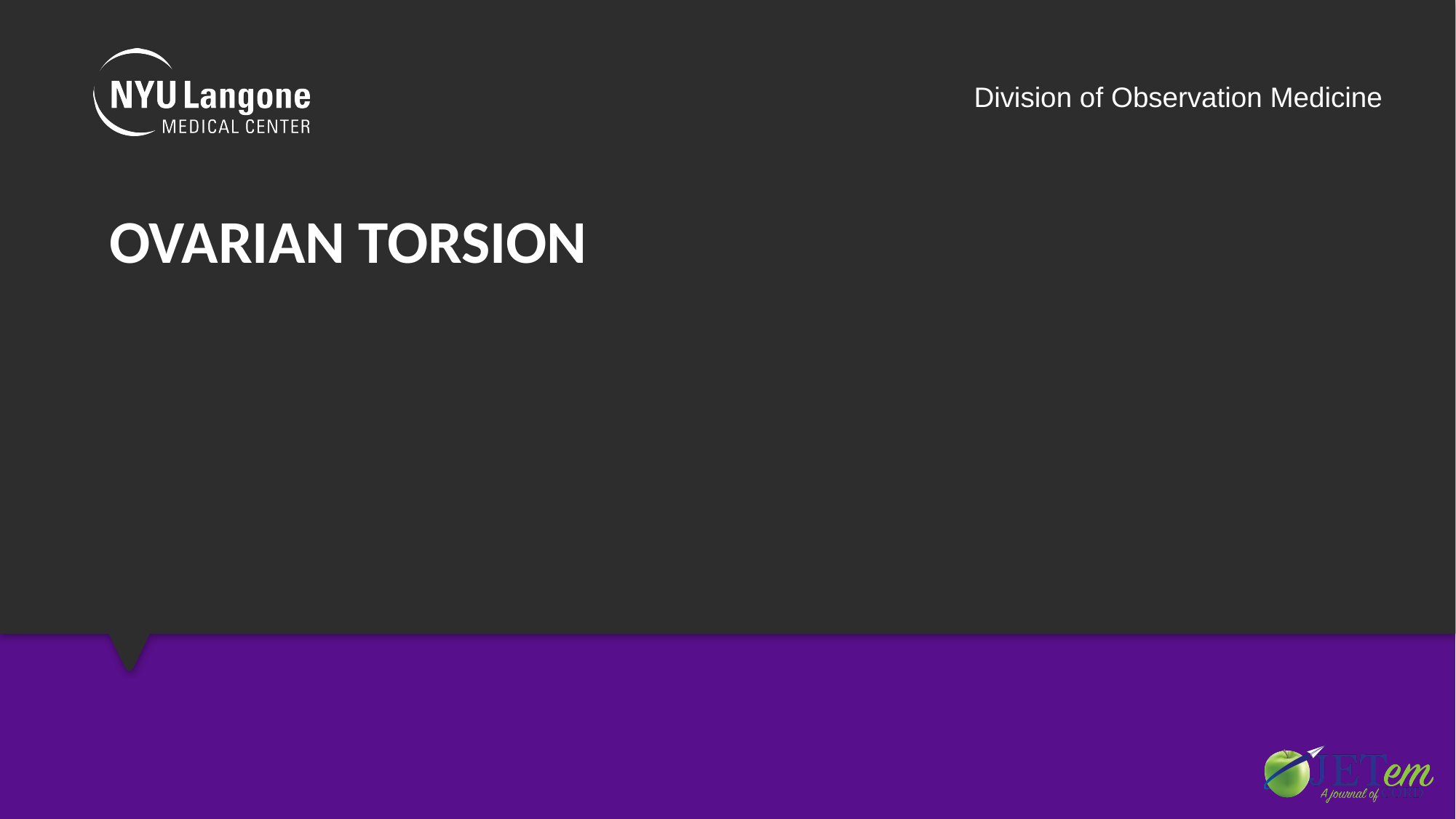

Division of Observation Medicine
# Ovarian Torsion

## Slide 2
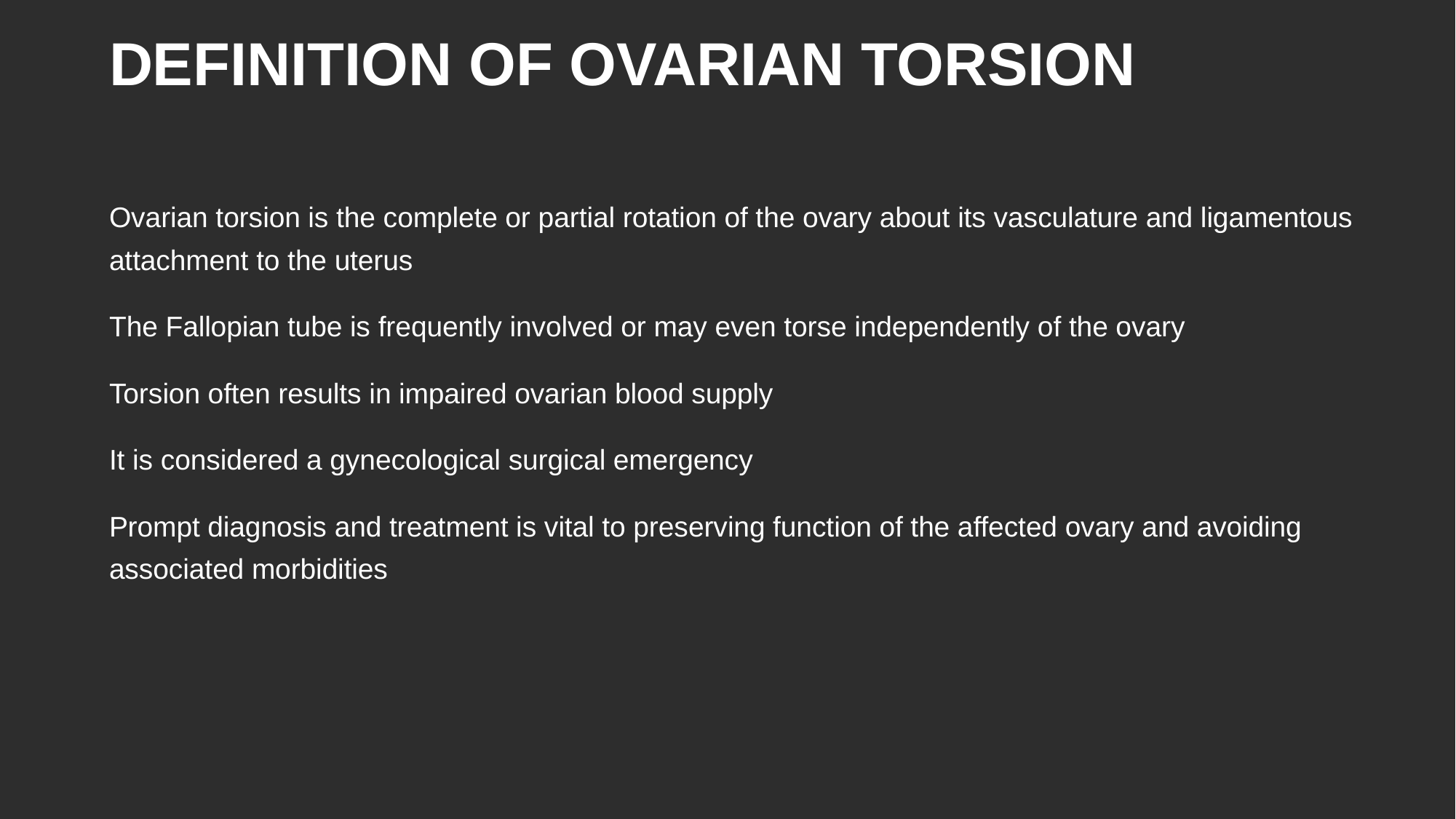

# Definition of Ovarian Torsion
Ovarian torsion is the complete or partial rotation of the ovary about its vasculature and ligamentous attachment to the uterus
The Fallopian tube is frequently involved or may even torse independently of the ovary
Torsion often results in impaired ovarian blood supply
It is considered a gynecological surgical emergency
Prompt diagnosis and treatment is vital to preserving function of the affected ovary and avoiding associated morbidities

## Slide 3
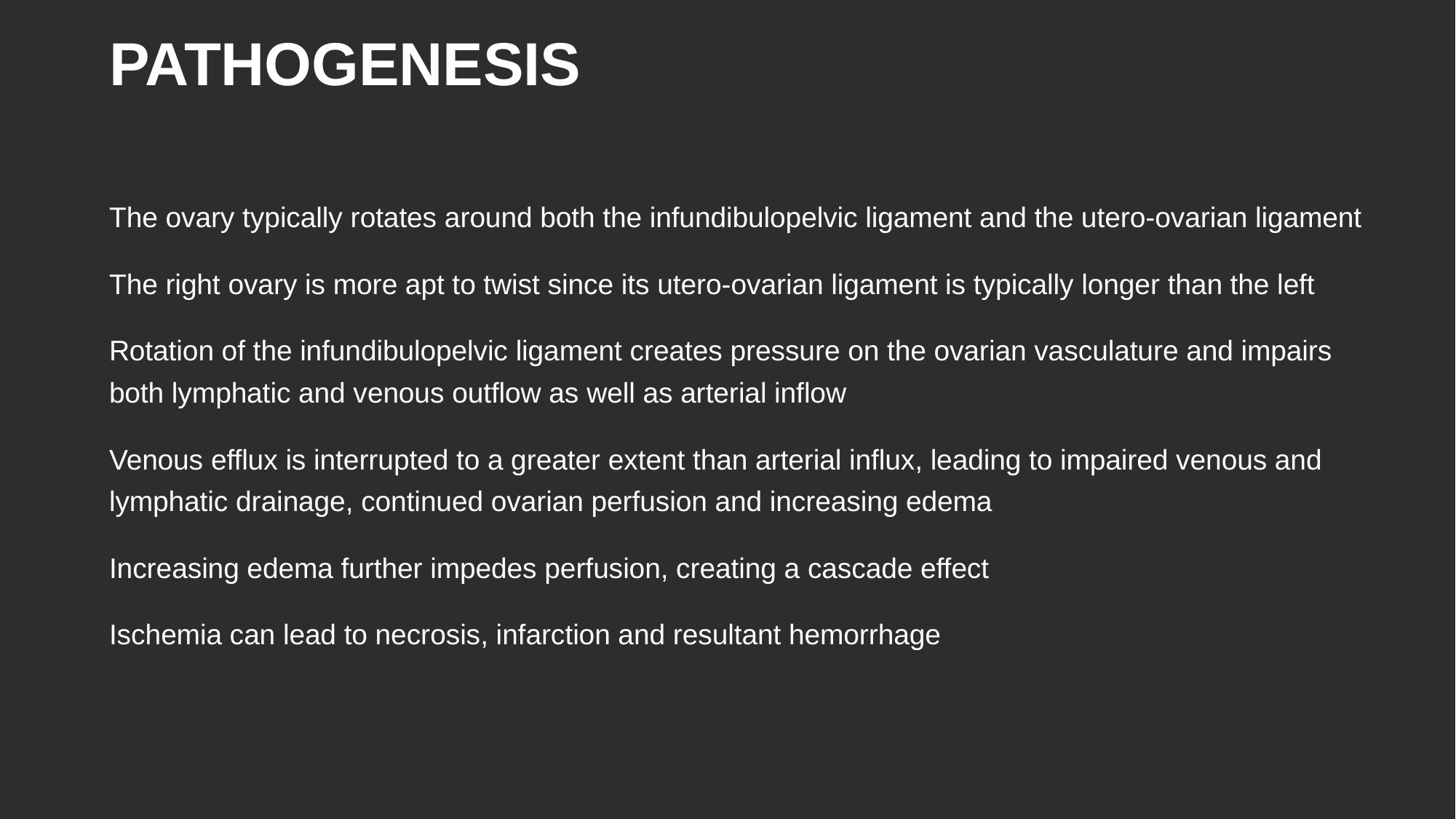

# Pathogenesis
The ovary typically rotates around both the infundibulopelvic ligament and the utero-ovarian ligament
The right ovary is more apt to twist since its utero-ovarian ligament is typically longer than the left
Rotation of the infundibulopelvic ligament creates pressure on the ovarian vasculature and impairs both lymphatic and venous outflow as well as arterial inflow
Venous efflux is interrupted to a greater extent than arterial influx, leading to impaired venous and lymphatic drainage, continued ovarian perfusion and increasing edema
Increasing edema further impedes perfusion, creating a cascade effect
Ischemia can lead to necrosis, infarction and resultant hemorrhage

## Slide 4
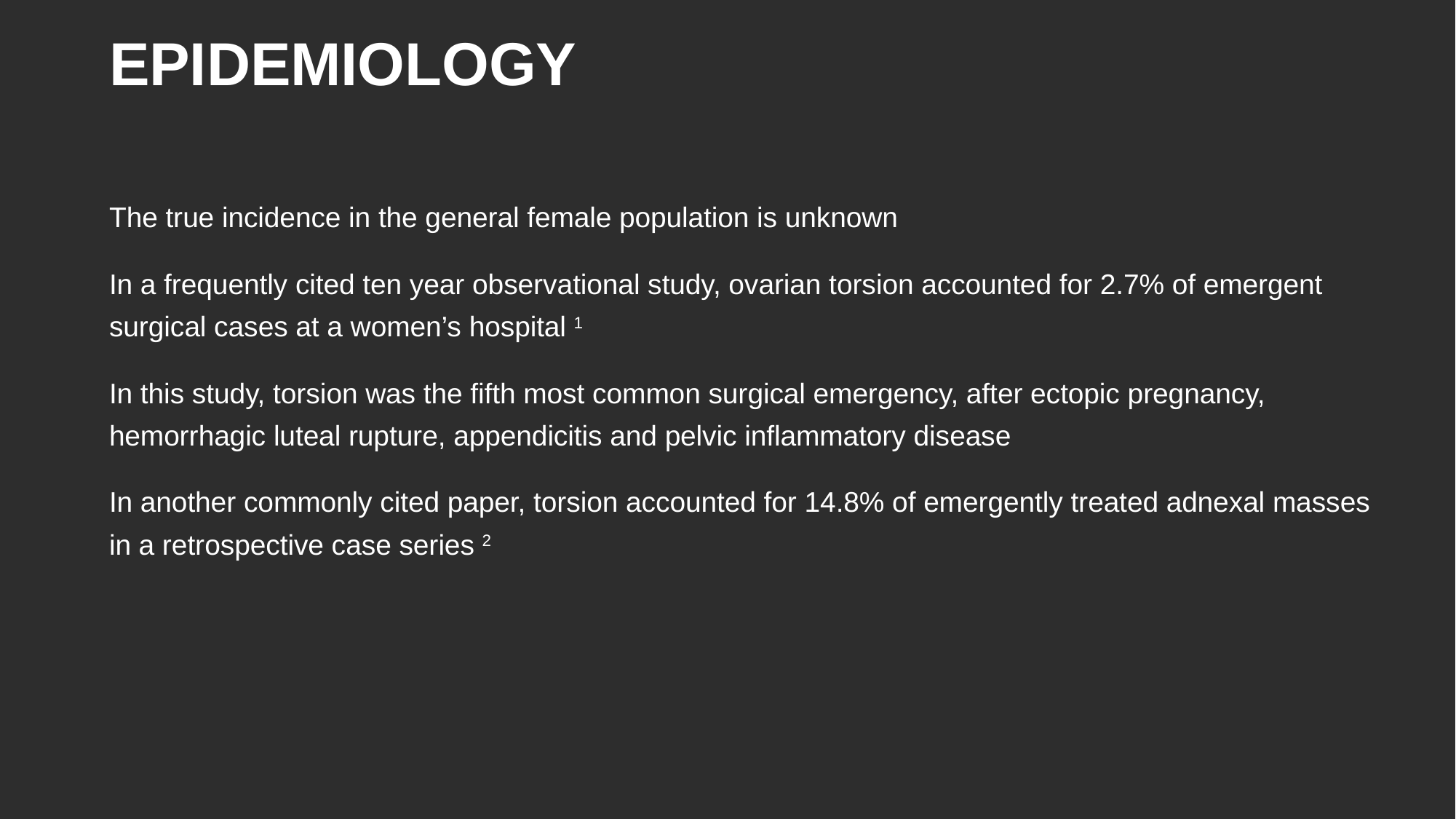

# Epidemiology
The true incidence in the general female population is unknown
In a frequently cited ten year observational study, ovarian torsion accounted for 2.7% of emergent surgical cases at a women’s hospital 1
In this study, torsion was the fifth most common surgical emergency, after ectopic pregnancy, hemorrhagic luteal rupture, appendicitis and pelvic inflammatory disease
In another commonly cited paper, torsion accounted for 14.8% of emergently treated adnexal masses in a retrospective case series 2

## Slide 5
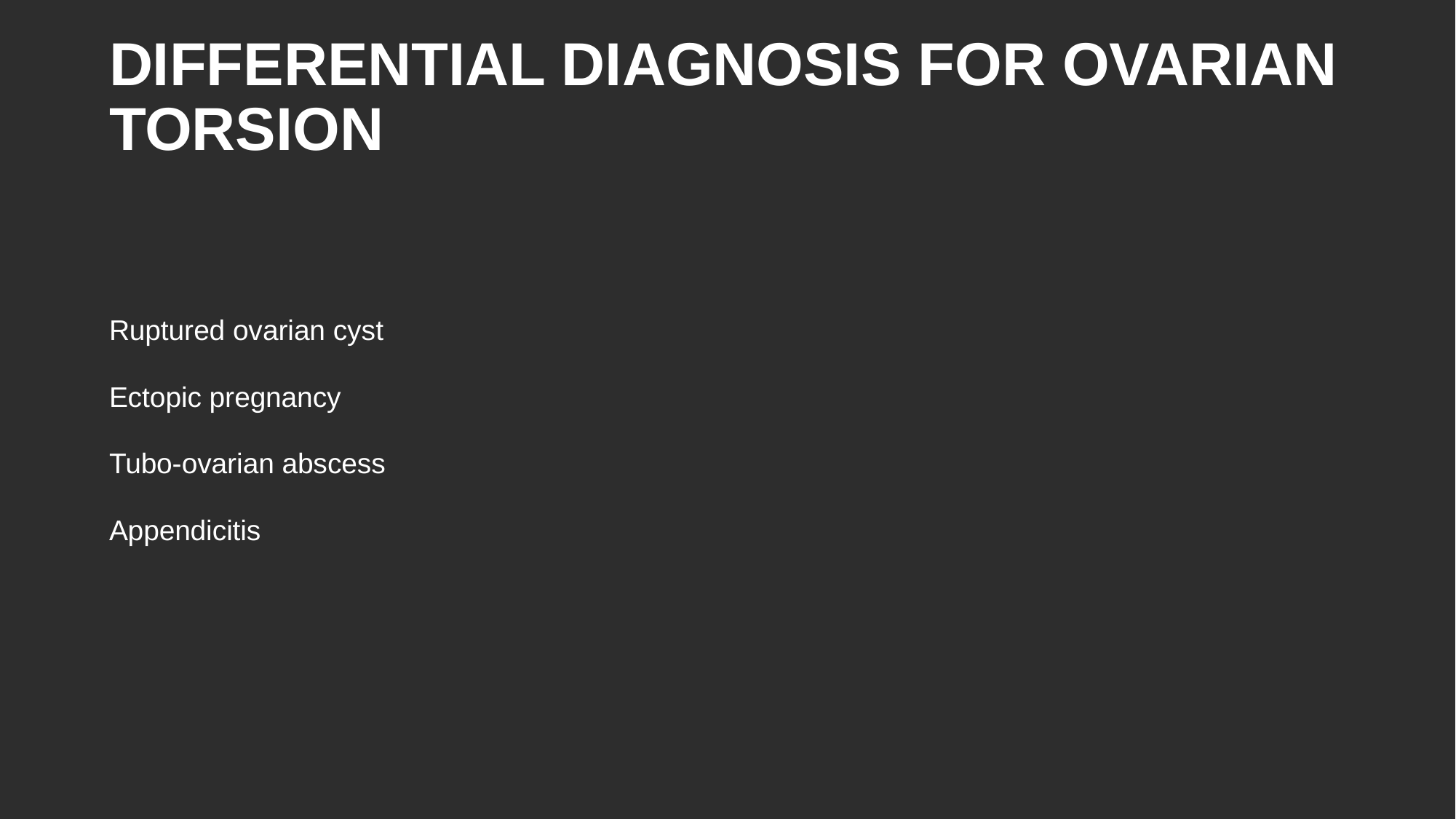

# Differential Diagnosis for Ovarian Torsion
Ruptured ovarian cyst
Ectopic pregnancy
Tubo-ovarian abscess
Appendicitis

## Slide 6
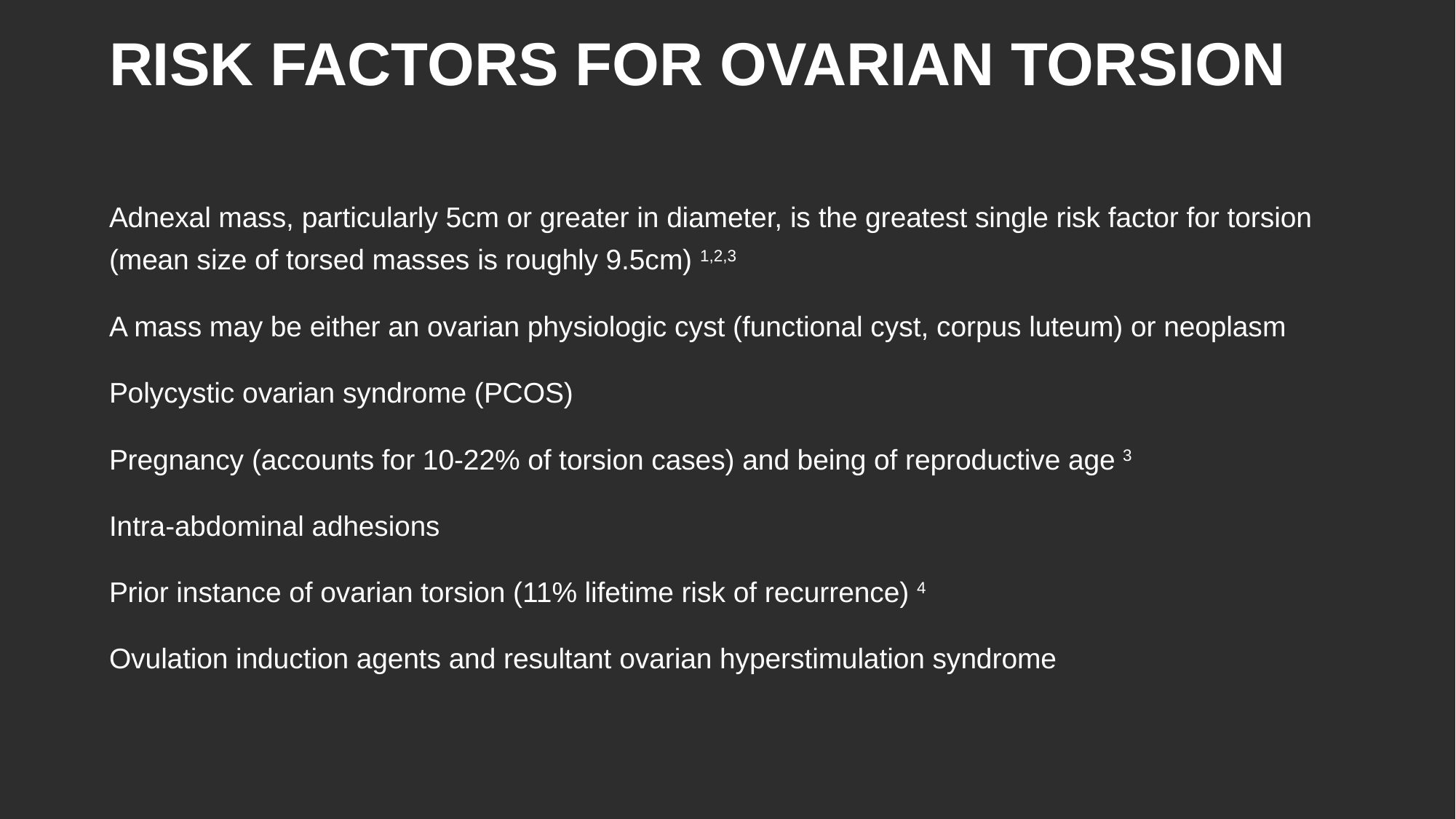

# Risk Factors for Ovarian Torsion
Adnexal mass, particularly 5cm or greater in diameter, is the greatest single risk factor for torsion (mean size of torsed masses is roughly 9.5cm) 1,2,3
A mass may be either an ovarian physiologic cyst (functional cyst, corpus luteum) or neoplasm
Polycystic ovarian syndrome (PCOS)
Pregnancy (accounts for 10-22% of torsion cases) and being of reproductive age 3
Intra-abdominal adhesions
Prior instance of ovarian torsion (11% lifetime risk of recurrence) 4
Ovulation induction agents and resultant ovarian hyperstimulation syndrome

## Slide 7
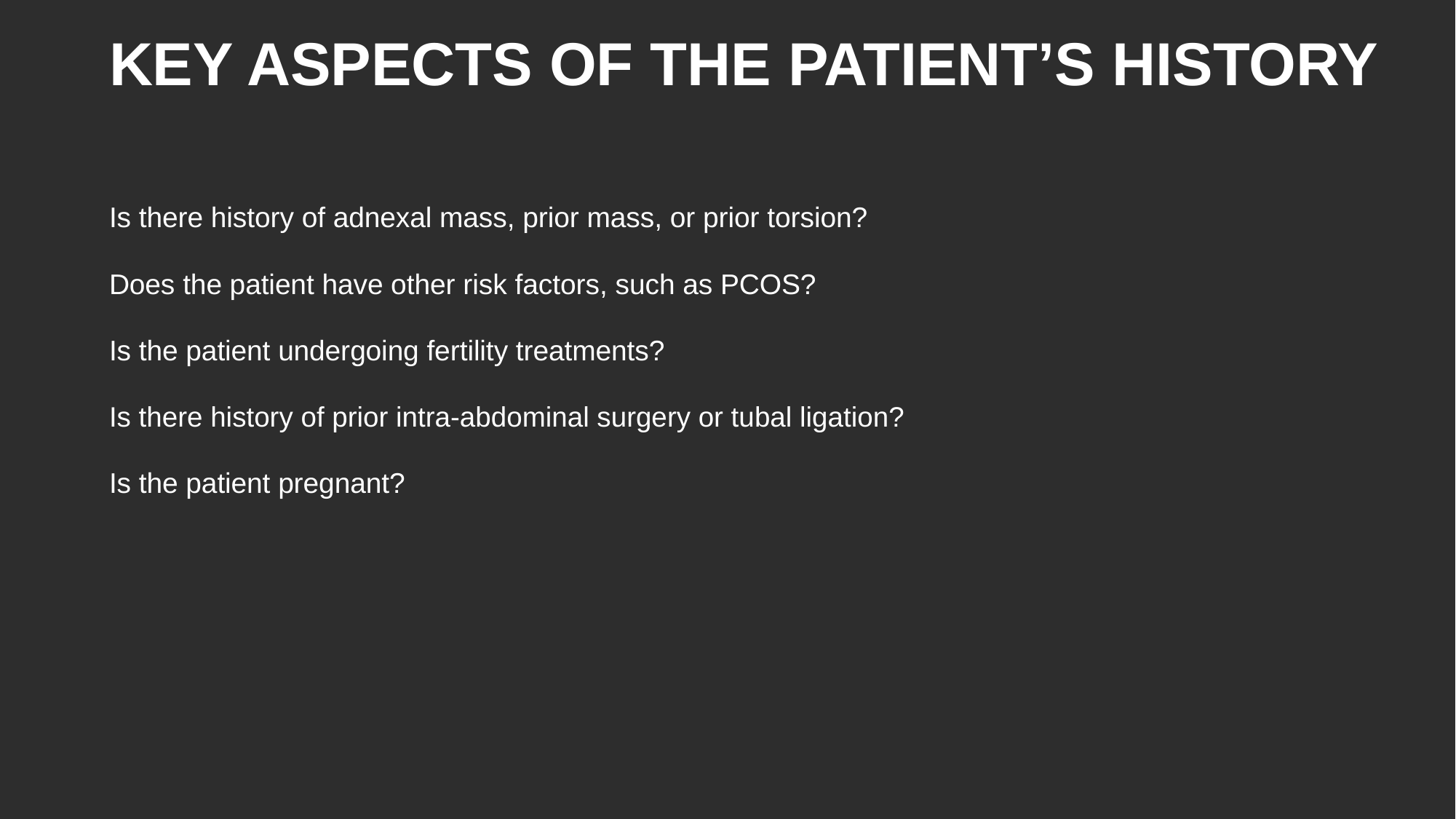

# Key Aspects of the Patient’s History
Is there history of adnexal mass, prior mass, or prior torsion?
Does the patient have other risk factors, such as PCOS?
Is the patient undergoing fertility treatments?
Is there history of prior intra-abdominal surgery or tubal ligation?
Is the patient pregnant?

## Slide 8
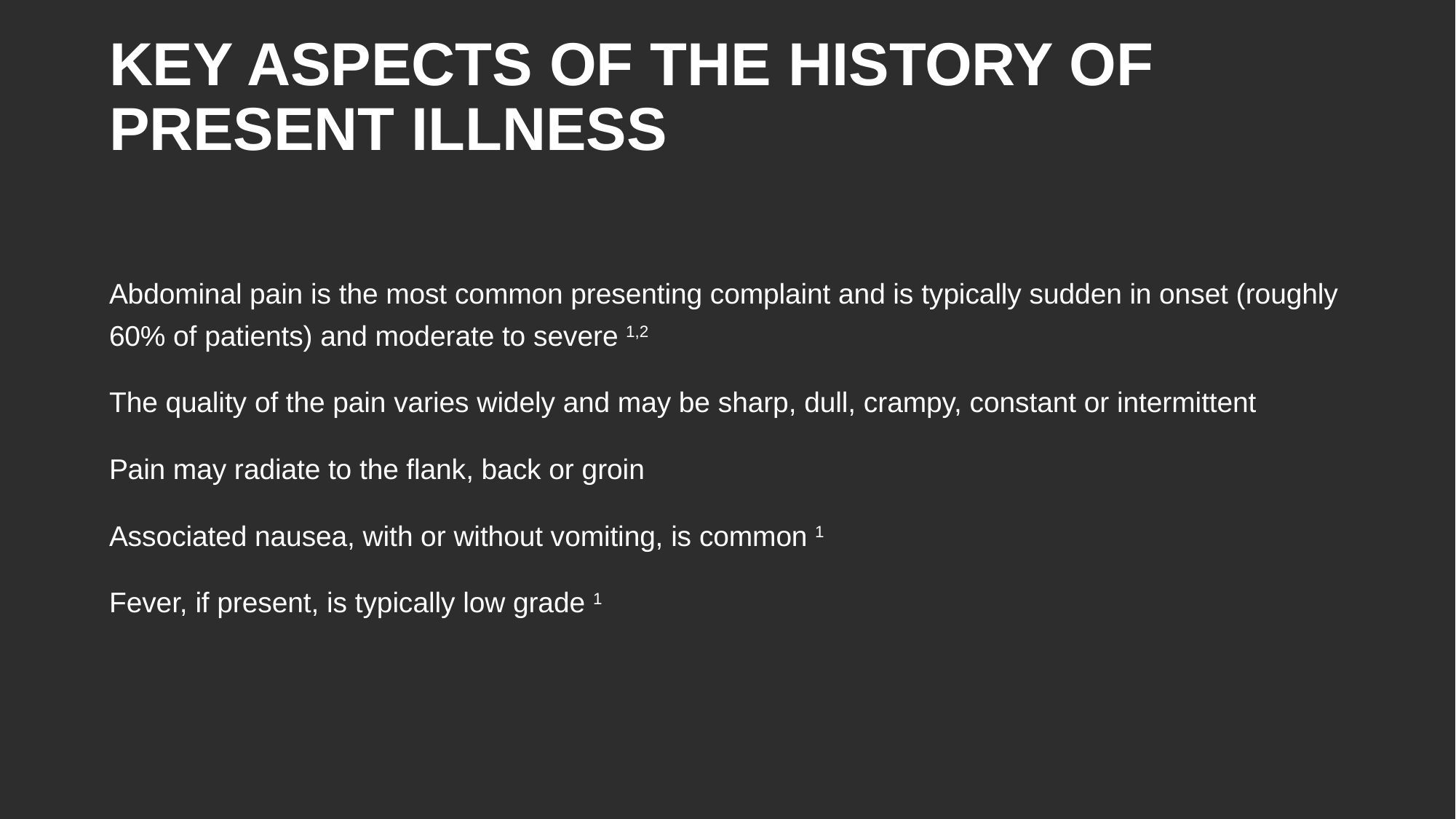

# Key Aspects of the History of Present Illness
Abdominal pain is the most common presenting complaint and is typically sudden in onset (roughly 60% of patients) and moderate to severe 1,2
The quality of the pain varies widely and may be sharp, dull, crampy, constant or intermittent
Pain may radiate to the flank, back or groin
Associated nausea, with or without vomiting, is common 1
Fever, if present, is typically low grade 1

## Slide 9
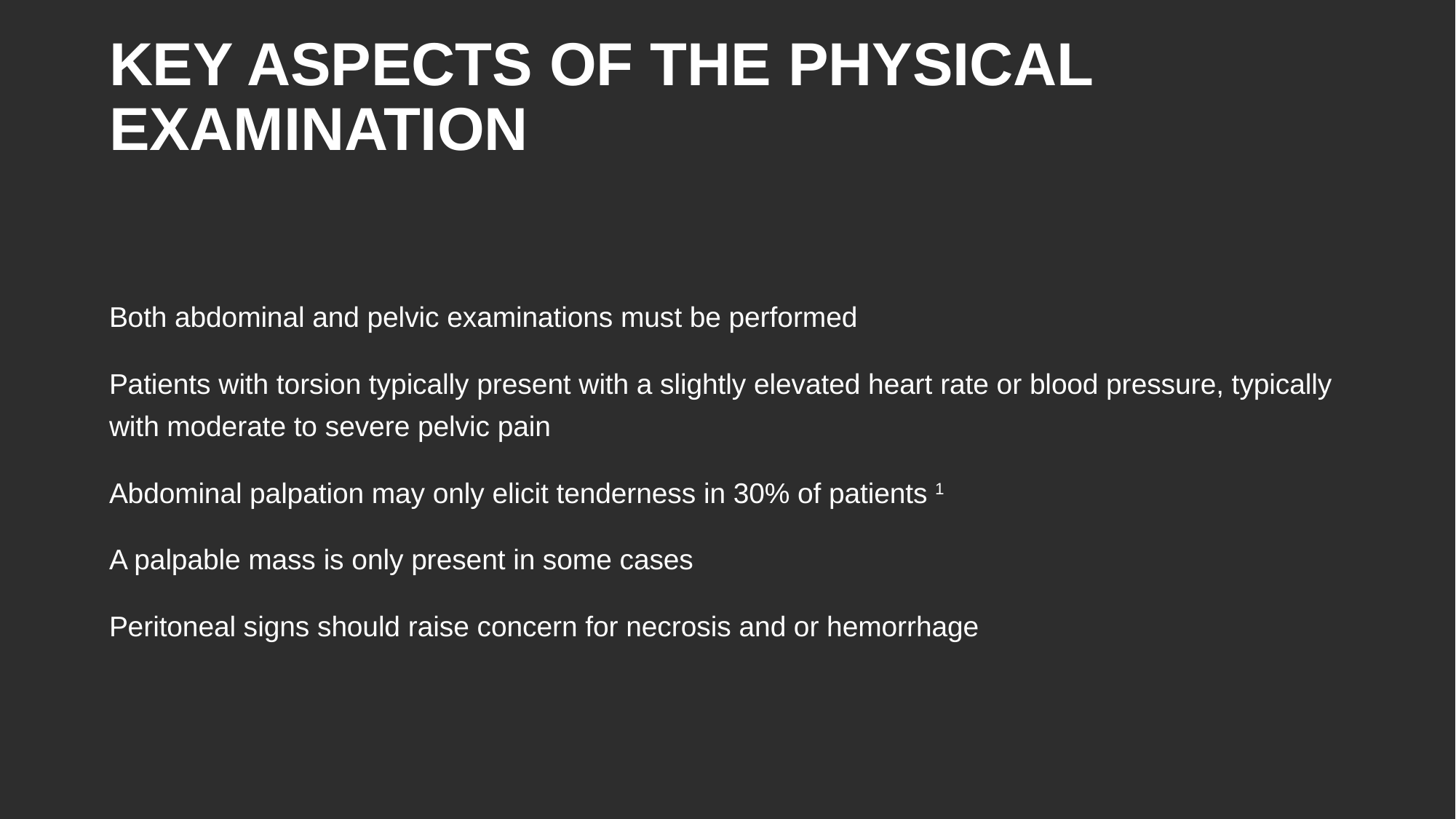

# Key Aspects of the Physical Examination
Both abdominal and pelvic examinations must be performed
Patients with torsion typically present with a slightly elevated heart rate or blood pressure, typically with moderate to severe pelvic pain
Abdominal palpation may only elicit tenderness in 30% of patients 1
A palpable mass is only present in some cases
Peritoneal signs should raise concern for necrosis and or hemorrhage

## Slide 10
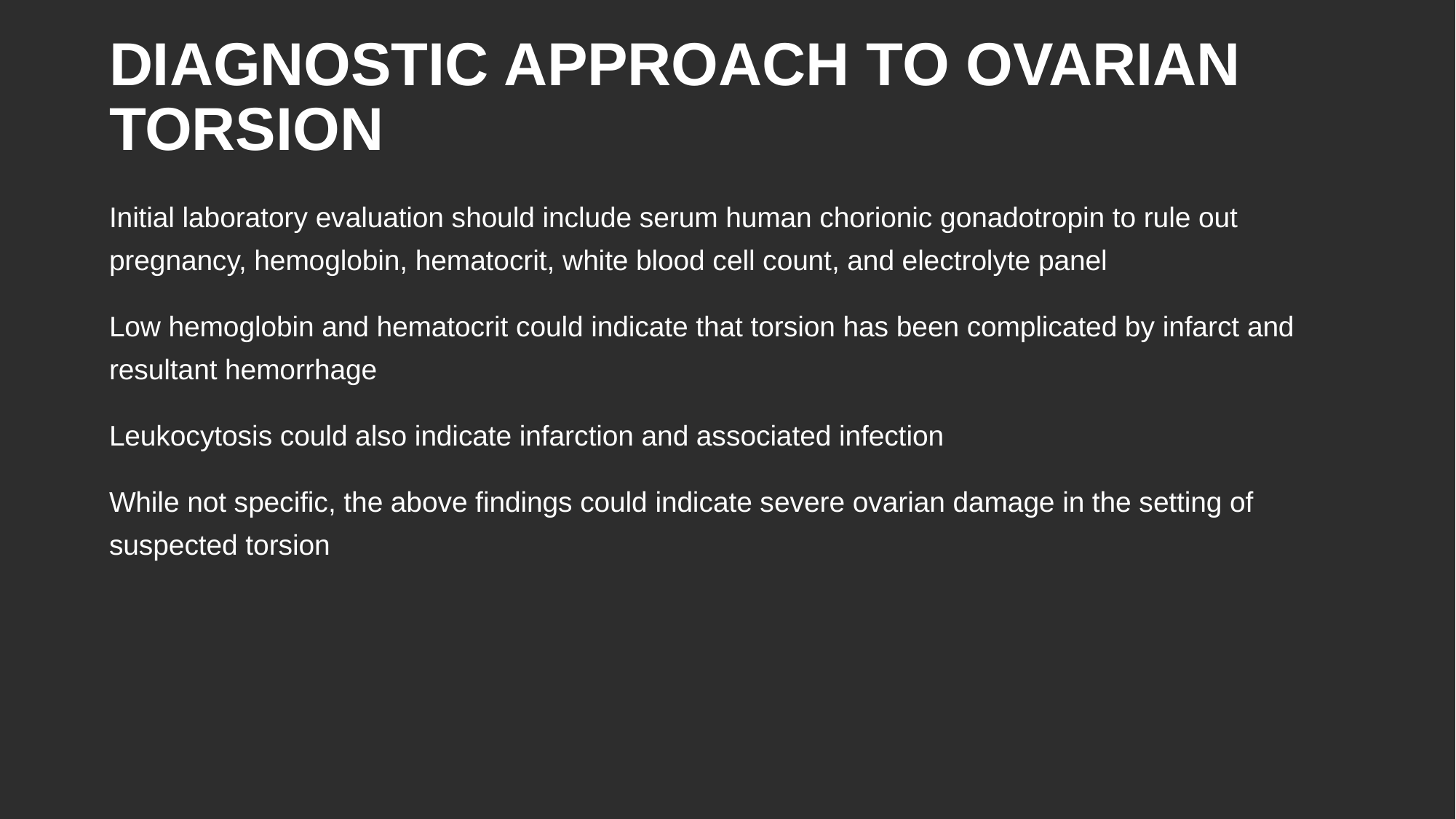

# Diagnostic Approach to Ovarian Torsion
Initial laboratory evaluation should include serum human chorionic gonadotropin to rule out pregnancy, hemoglobin, hematocrit, white blood cell count, and electrolyte panel
Low hemoglobin and hematocrit could indicate that torsion has been complicated by infarct and resultant hemorrhage
Leukocytosis could also indicate infarction and associated infection
While not specific, the above findings could indicate severe ovarian damage in the setting of suspected torsion

## Slide 11
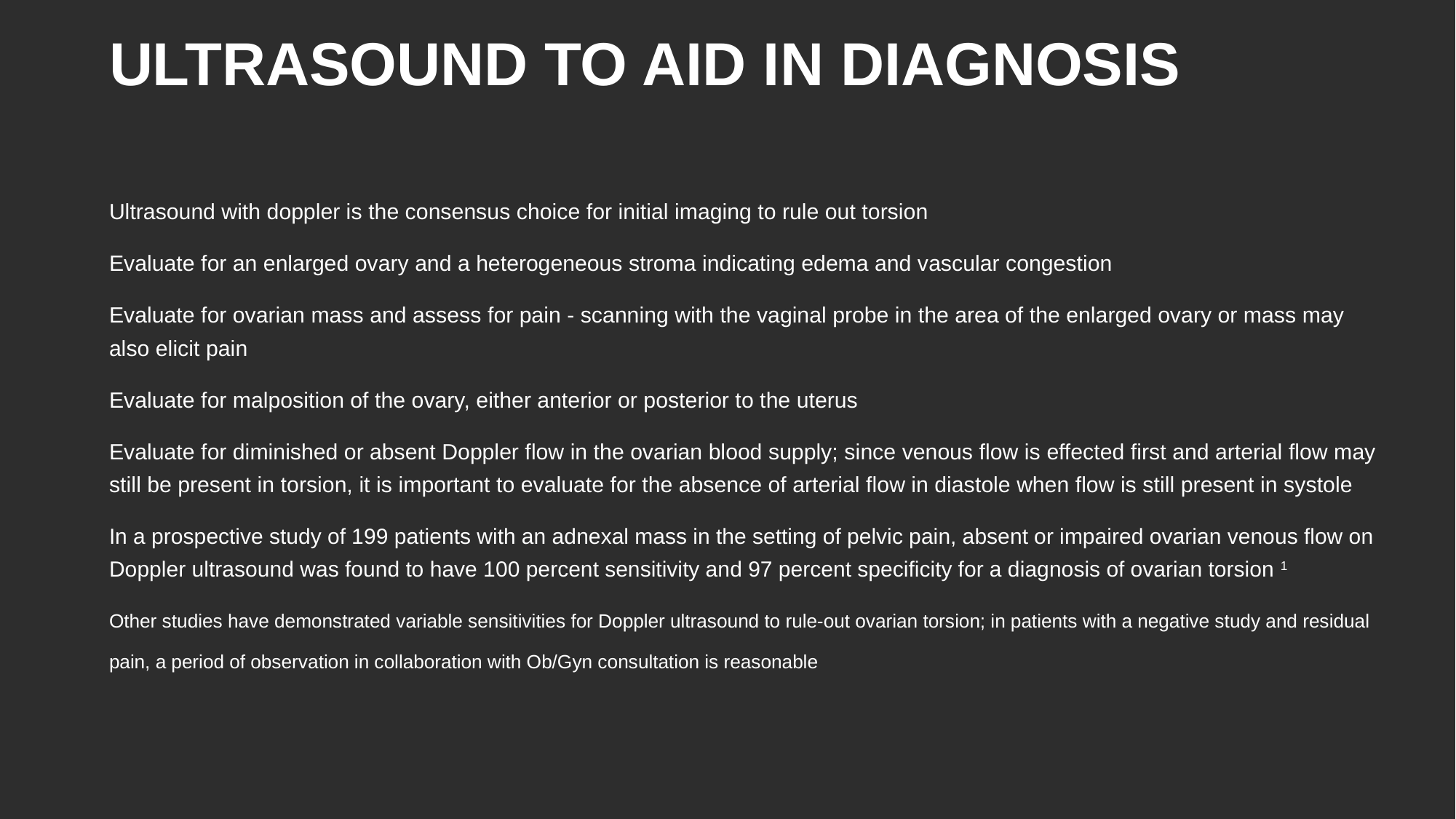

# Ultrasound to Aid in Diagnosis
Ultrasound with doppler is the consensus choice for initial imaging to rule out torsion
Evaluate for an enlarged ovary and a heterogeneous stroma indicating edema and vascular congestion
Evaluate for ovarian mass and assess for pain - scanning with the vaginal probe in the area of the enlarged ovary or mass may also elicit pain
Evaluate for malposition of the ovary, either anterior or posterior to the uterus
Evaluate for diminished or absent Doppler flow in the ovarian blood supply; since venous flow is effected first and arterial flow may still be present in torsion, it is important to evaluate for the absence of arterial flow in diastole when flow is still present in systole
In a prospective study of 199 patients with an adnexal mass in the setting of pelvic pain, absent or impaired ovarian venous flow on Doppler ultrasound was found to have 100 percent sensitivity and 97 percent specificity for a diagnosis of ovarian torsion 1
Other studies have demonstrated variable sensitivities for Doppler ultrasound to rule-out ovarian torsion; in patients with a negative study and residual pain, a period of observation in collaboration with Ob/Gyn consultation is reasonable

## Slide 12
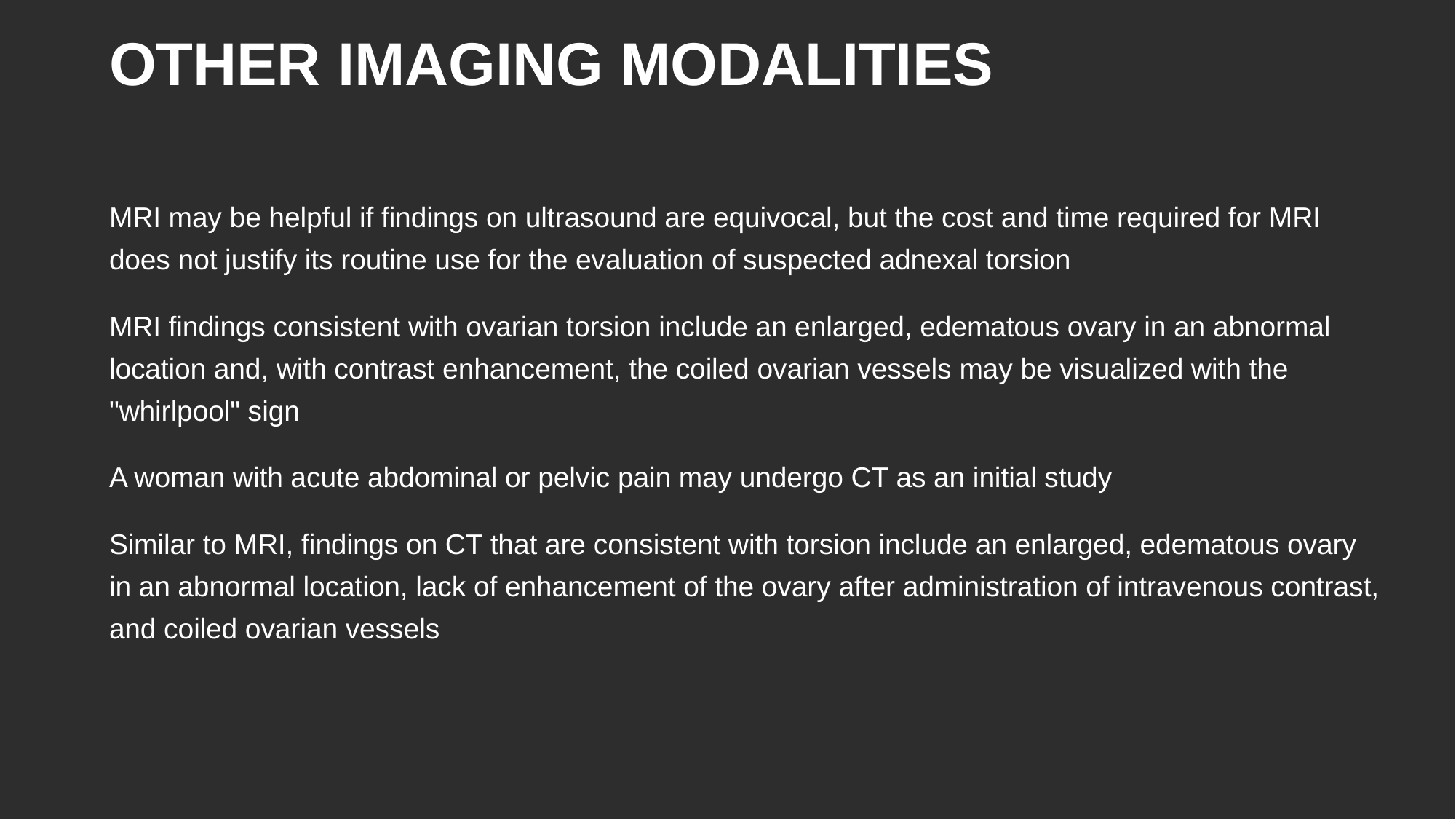

# Other Imaging Modalities
MRI may be helpful if findings on ultrasound are equivocal, but the cost and time required for MRI does not justify its routine use for the evaluation of suspected adnexal torsion
MRI findings consistent with ovarian torsion include an enlarged, edematous ovary in an abnormal location and, with contrast enhancement, the coiled ovarian vessels may be visualized with the "whirlpool" sign
A woman with acute abdominal or pelvic pain may undergo CT as an initial study
Similar to MRI, findings on CT that are consistent with torsion include an enlarged, edematous ovary in an abnormal location, lack of enhancement of the ovary after administration of intravenous contrast, and coiled ovarian vessels

## Slide 13
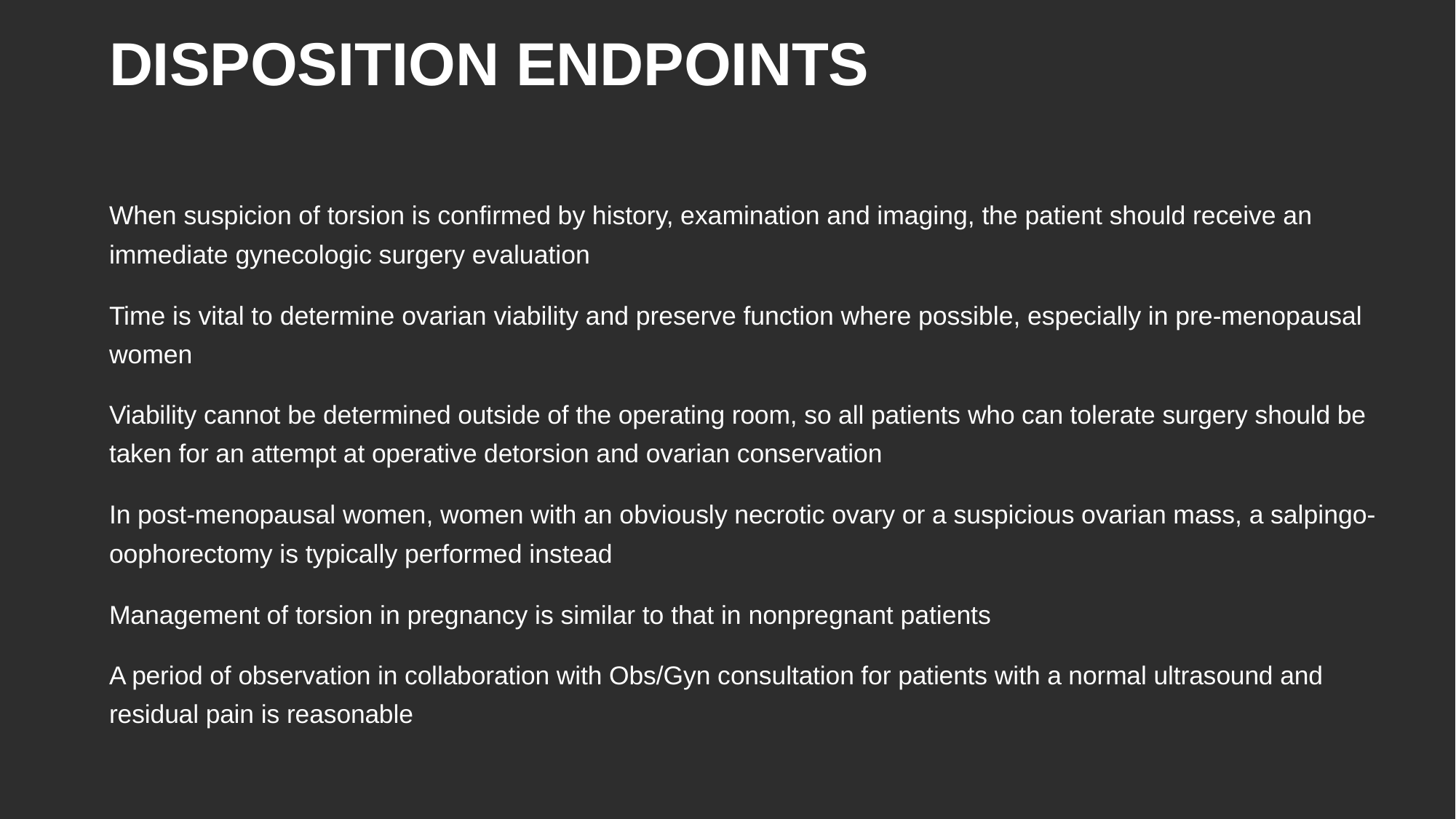

# Disposition Endpoints
When suspicion of torsion is confirmed by history, examination and imaging, the patient should receive an immediate gynecologic surgery evaluation
Time is vital to determine ovarian viability and preserve function where possible, especially in pre-menopausal women
Viability cannot be determined outside of the operating room, so all patients who can tolerate surgery should be taken for an attempt at operative detorsion and ovarian conservation
In post-menopausal women, women with an obviously necrotic ovary or a suspicious ovarian mass, a salpingo-oophorectomy is typically performed instead
Management of torsion in pregnancy is similar to that in nonpregnant patients
A period of observation in collaboration with Obs/Gyn consultation for patients with a normal ultrasound and residual pain is reasonable

## Slide 14
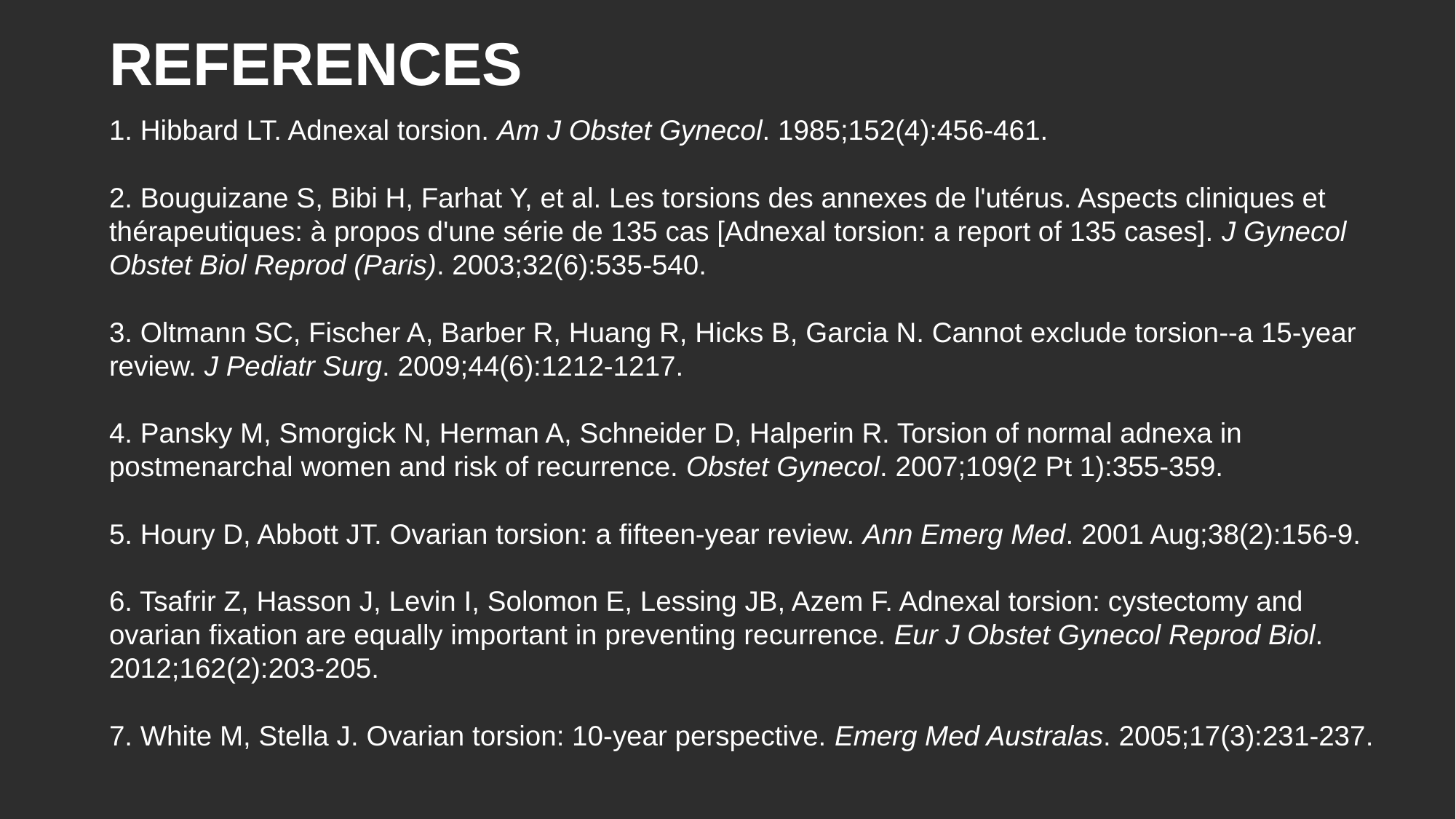

# References
1. Hibbard LT. Adnexal torsion. Am J Obstet Gynecol. 1985;152(4):456-461.
2. Bouguizane S, Bibi H, Farhat Y, et al. Les torsions des annexes de l'utérus. Aspects cliniques et thérapeutiques: à propos d'une série de 135 cas [Adnexal torsion: a report of 135 cases]. J Gynecol Obstet Biol Reprod (Paris). 2003;32(6):535-540.
3. Oltmann SC, Fischer A, Barber R, Huang R, Hicks B, Garcia N. Cannot exclude torsion--a 15-year review. J Pediatr Surg. 2009;44(6):1212-1217.
4. Pansky M, Smorgick N, Herman A, Schneider D, Halperin R. Torsion of normal adnexa in postmenarchal women and risk of recurrence. Obstet Gynecol. 2007;109(2 Pt 1):355-359.
5. Houry D, Abbott JT. Ovarian torsion: a fifteen-year review. Ann Emerg Med. 2001 Aug;38(2):156-9.
6. Tsafrir Z, Hasson J, Levin I, Solomon E, Lessing JB, Azem F. Adnexal torsion: cystectomy and ovarian fixation are equally important in preventing recurrence. Eur J Obstet Gynecol Reprod Biol. 2012;162(2):203-205.
7. White M, Stella J. Ovarian torsion: 10-year perspective. Emerg Med Australas. 2005;17(3):231-237.
