# Supplementary material for: An Observation Medicine Curriculum for Emergency Medicine Education
Source: J Educ Teach Emerg Med. 2021 Apr 19;6(2):C1–C72. doi: 10.21980/J87P92 (PMC10332786; doi:10.21980/J87P92)
Supplement: Supplementary file 14 — Please see associated PowerPoint file [file jetem-6-2-c1-supp14.pptx]

## Slide 1
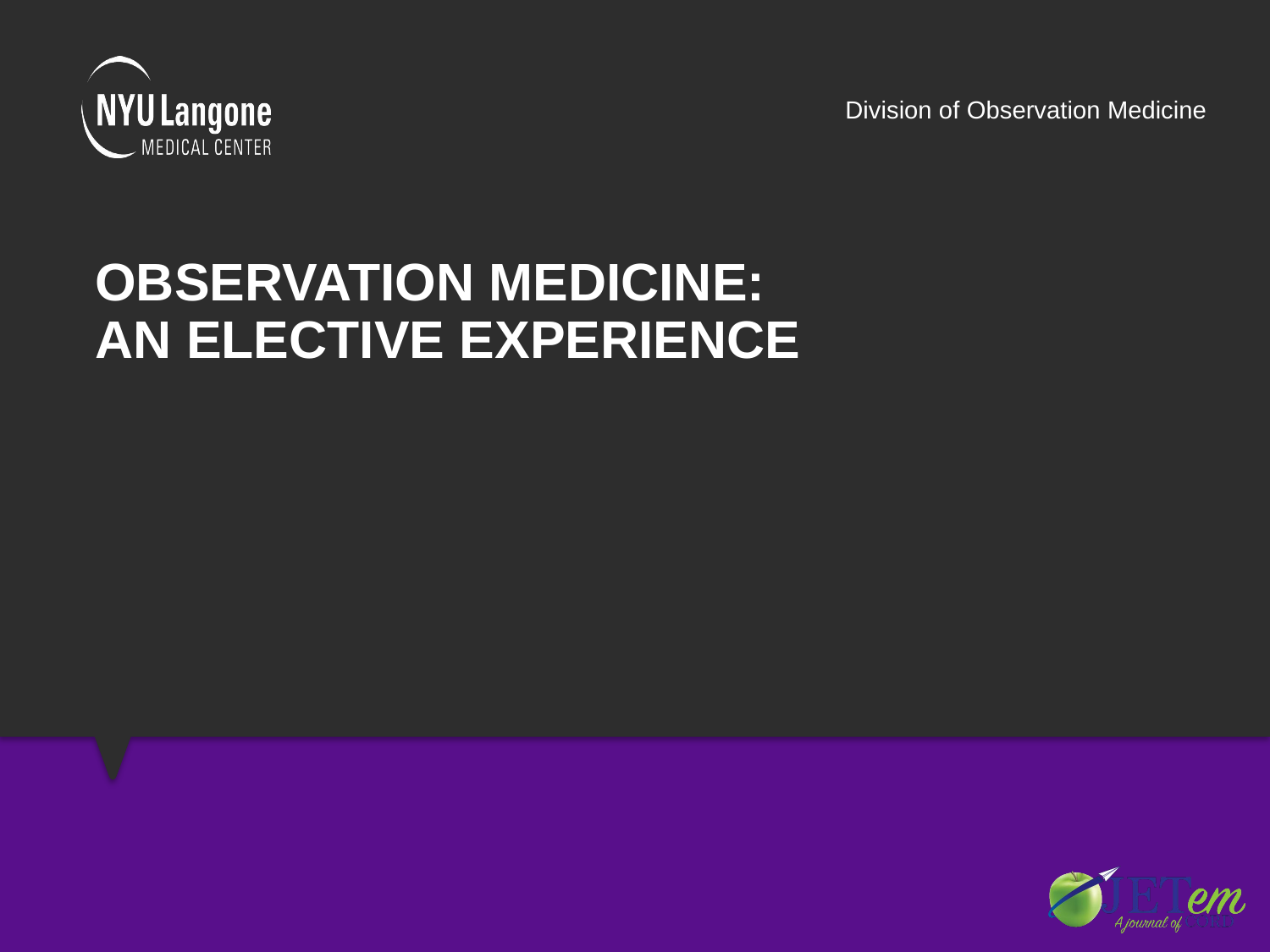

Division of Observation Medicine
# Observation Medicine:​ An elective experience​

## Slide 2
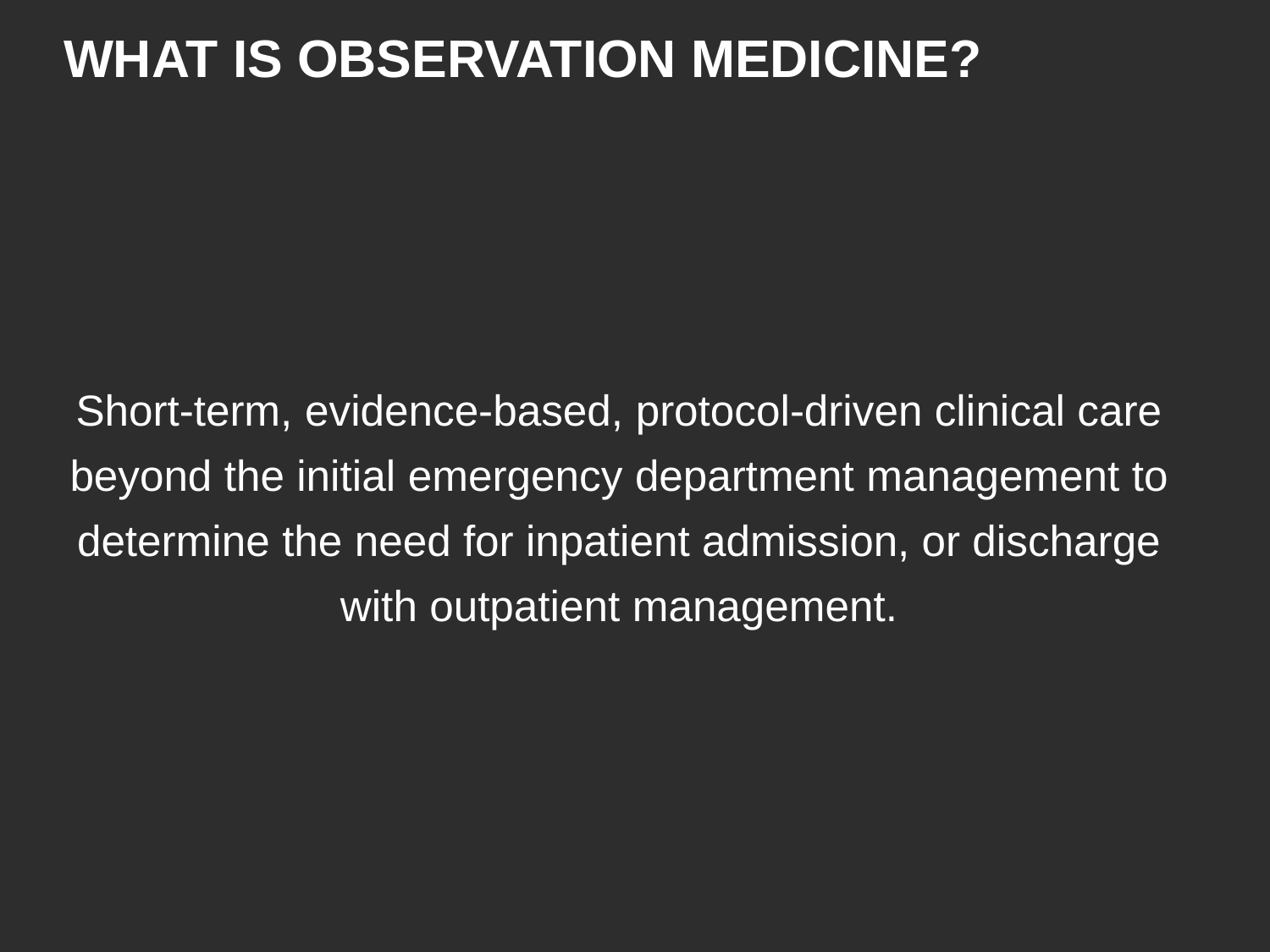

# What is Observation Medicine?
Short-term, evidence-based, protocol-driven clinical care beyond the initial emergency department management to determine the need for inpatient admission, or discharge with outpatient management.

## Slide 3
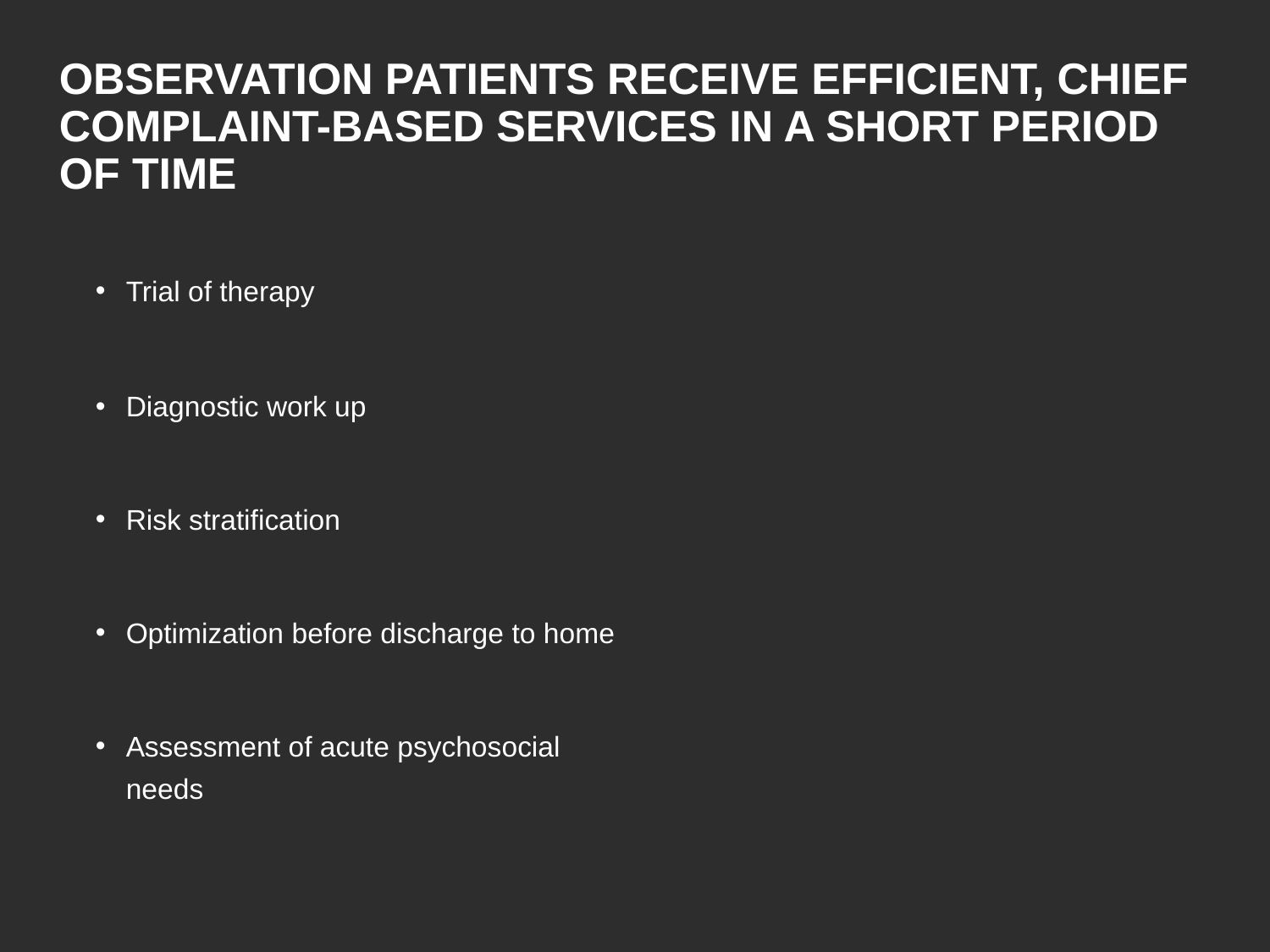

# Observation patients receive efficient, chief complaint-based services in a short period of time
Trial of therapy
Diagnostic work up
Risk stratification
Optimization before discharge to home
Assessment of acute psychosocial needs

## Slide 4
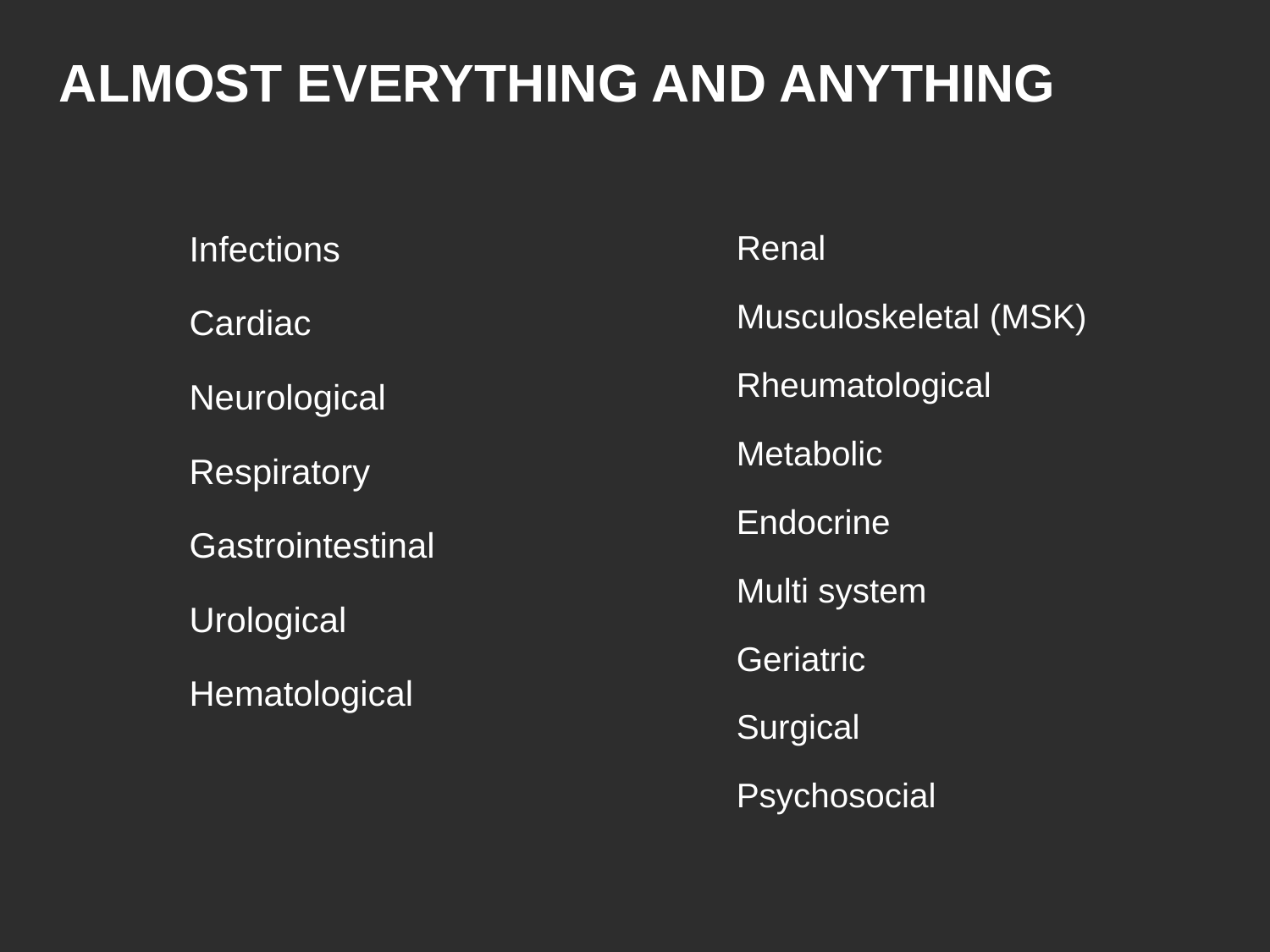

# Almost everything and anything
Infections
Cardiac
Neurological
Respiratory
Gastrointestinal
Urological
Hematological
Renal
Musculoskeletal (MSK)
Rheumatological
Metabolic
Endocrine
Multi system
Geriatric
Surgical
Psychosocial

## Slide 5
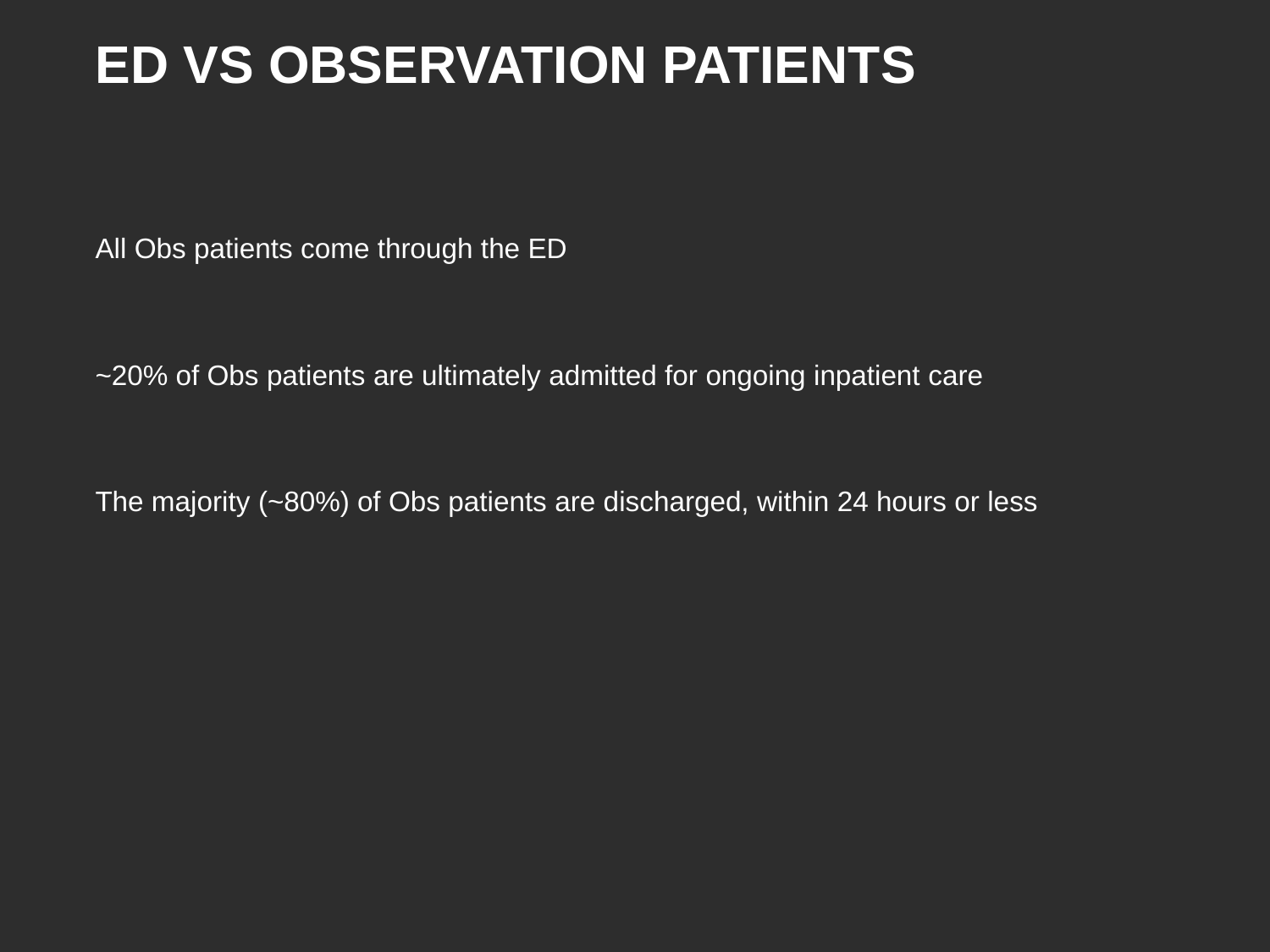

# ED vs Observation patients
All Obs patients come through the ED
~20% of Obs patients are ultimately admitted for ongoing inpatient care
The majority (~80%) of Obs patients are discharged, within 24 hours or less

## Slide 6
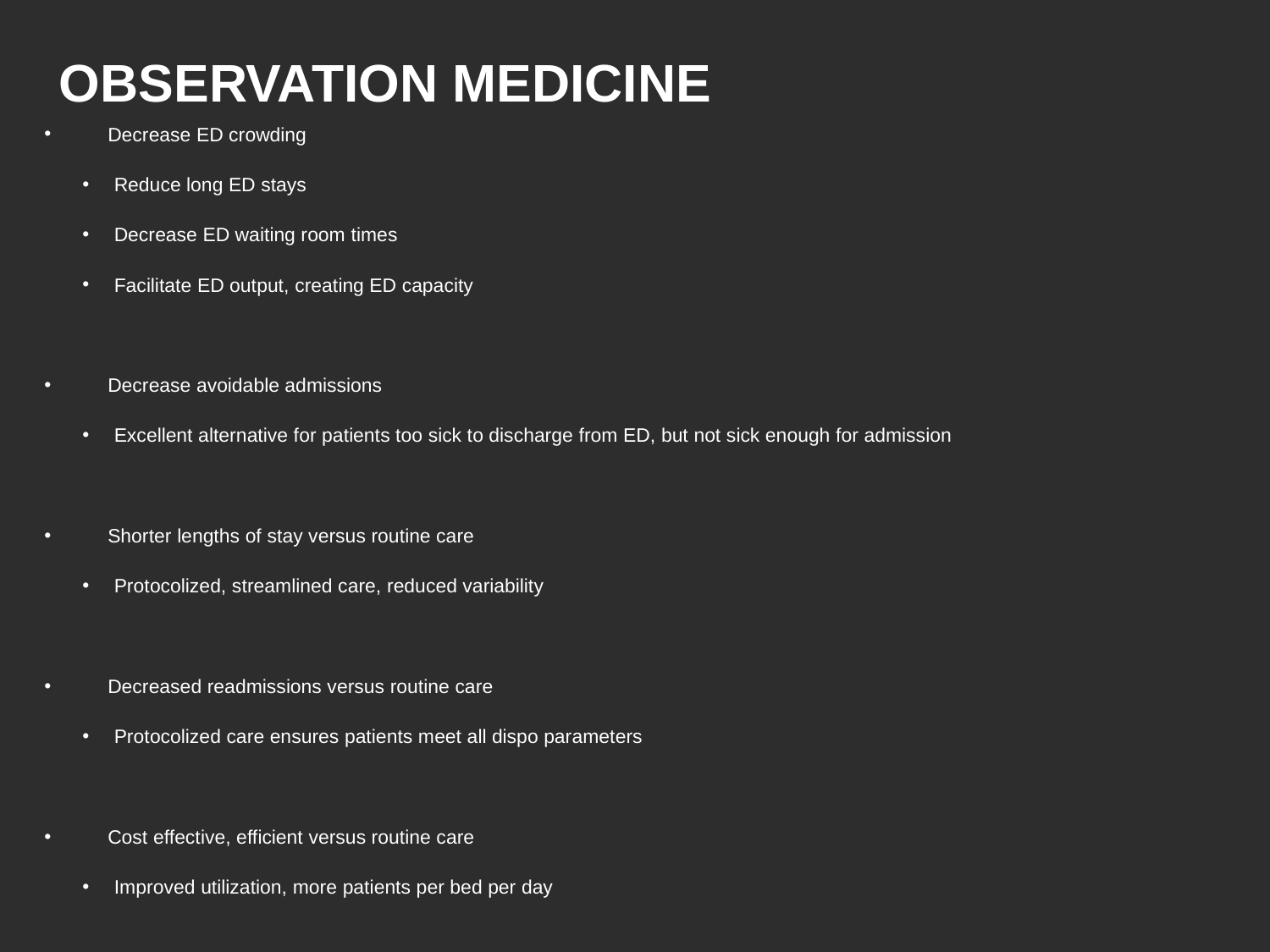

# Observation Medicine
Decrease ED crowding
Reduce long ED stays
Decrease ED waiting room times
Facilitate ED output, creating ED capacity
Decrease avoidable admissions
Excellent alternative for patients too sick to discharge from ED, but not sick enough for admission
Shorter lengths of stay versus routine care
Protocolized, streamlined care, reduced variability
Decreased readmissions versus routine care
Protocolized care ensures patients meet all dispo parameters
Cost effective, efficient versus routine care
Improved utilization, more patients per bed per day

## Slide 7
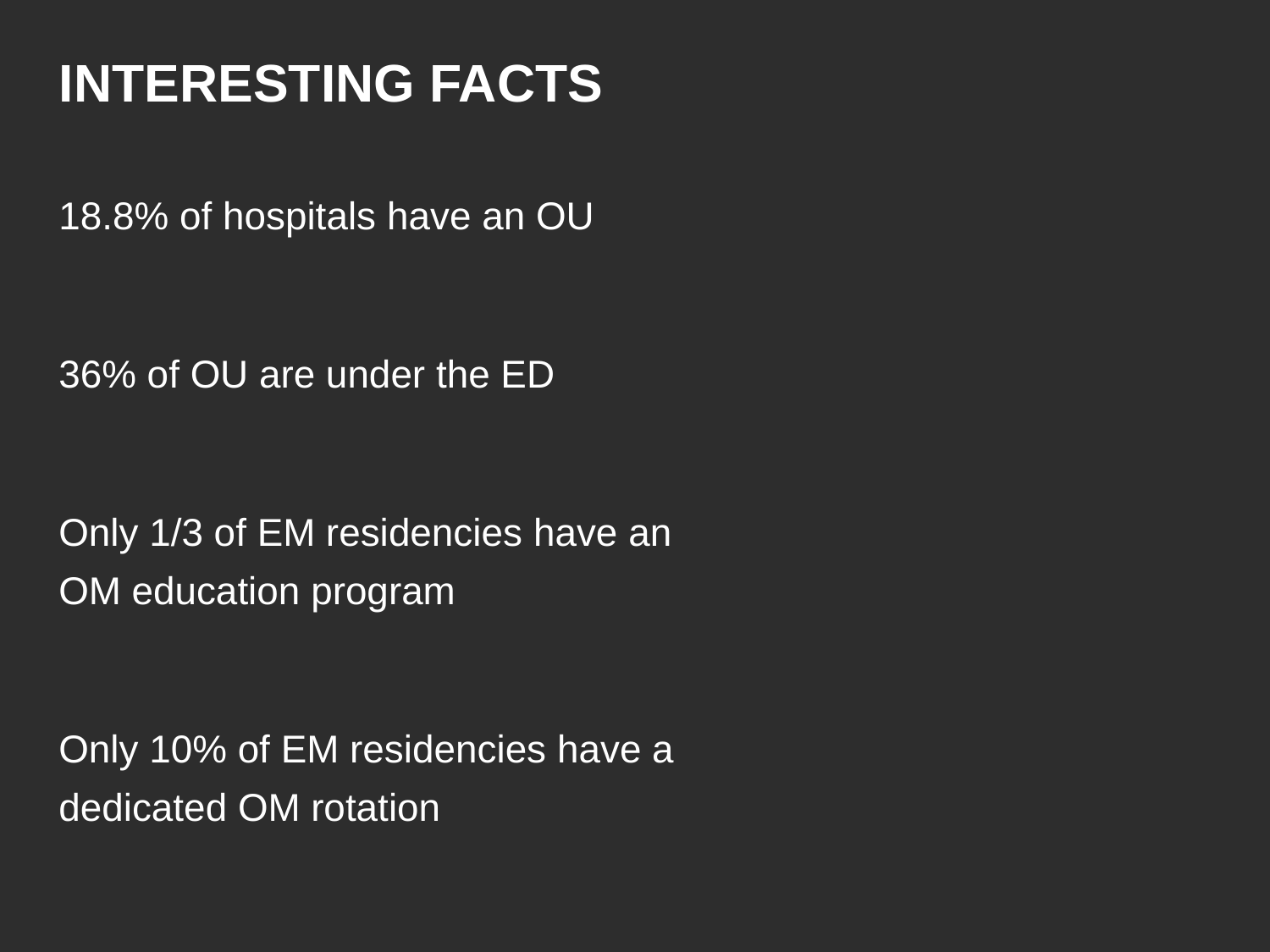

# Interesting Facts
18.8% of hospitals have an OU
36% of OU are under the ED
Only 1/3 of EM residencies have an OM education program
Only 10% of EM residencies have a dedicated OM rotation

## Slide 8
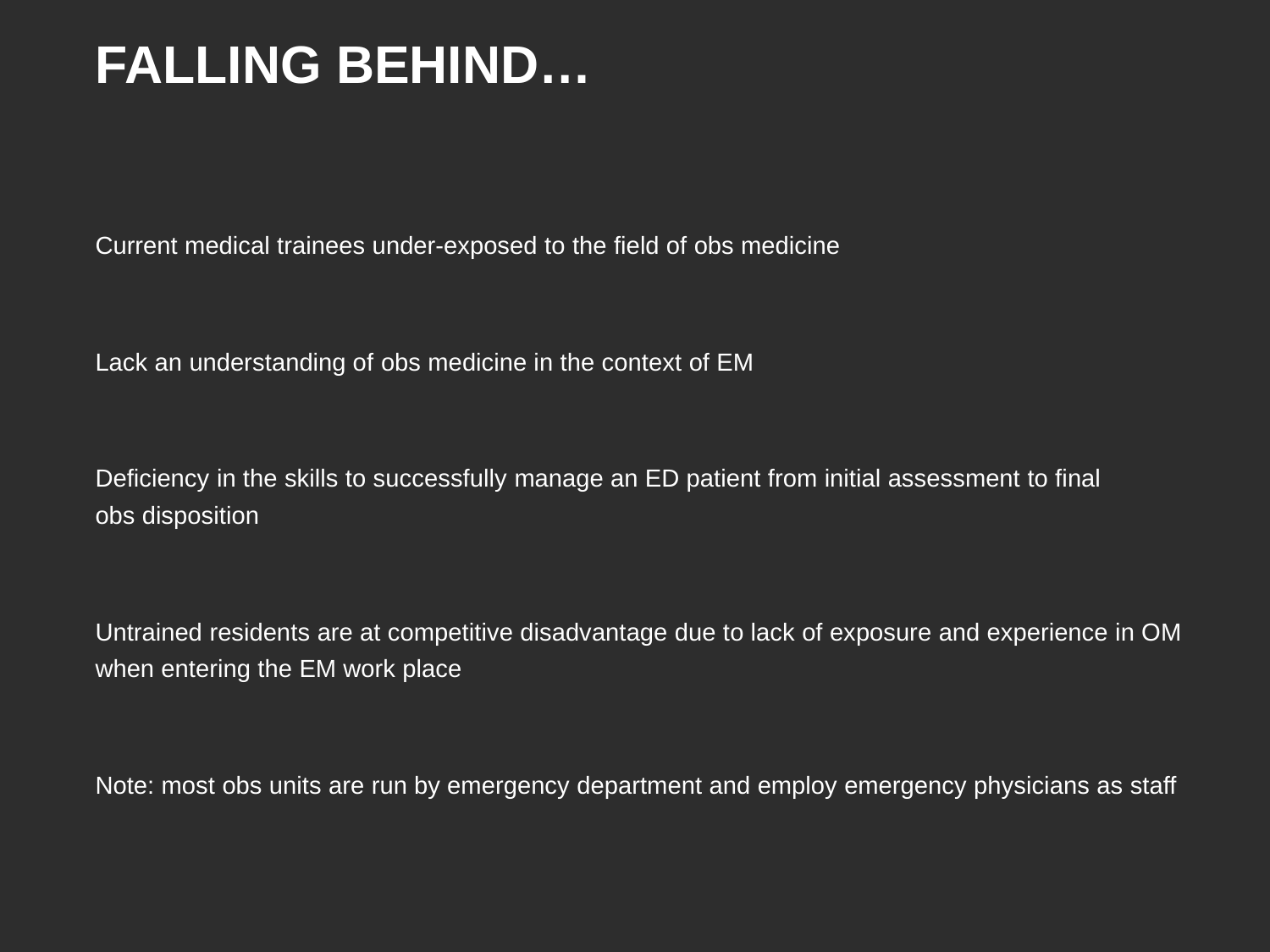

# Falling Behind…
Current medical trainees under-exposed to the field of obs medicine
Lack an understanding of obs medicine in the context of EM
Deficiency in the skills to successfully manage an ED patient from initial assessment to final obs disposition
Untrained residents are at competitive disadvantage due to lack of exposure and experience in OM when entering the EM work place
Note: most obs units are run by emergency department and employ emergency physicians as staff

## Slide 9
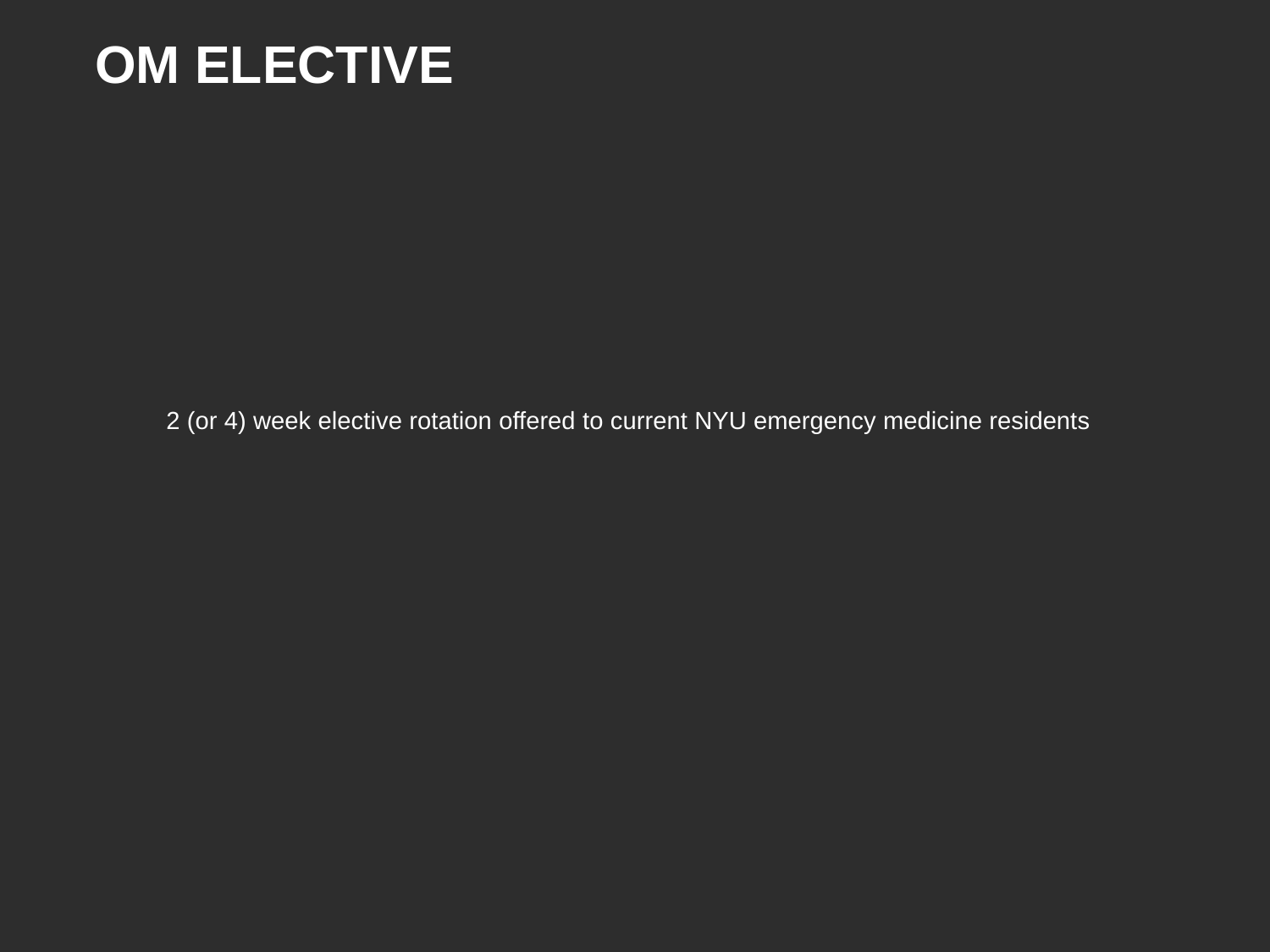

# OM Elective
2 (or 4) week elective rotation offered to current NYU emergency medicine residents

## Slide 10
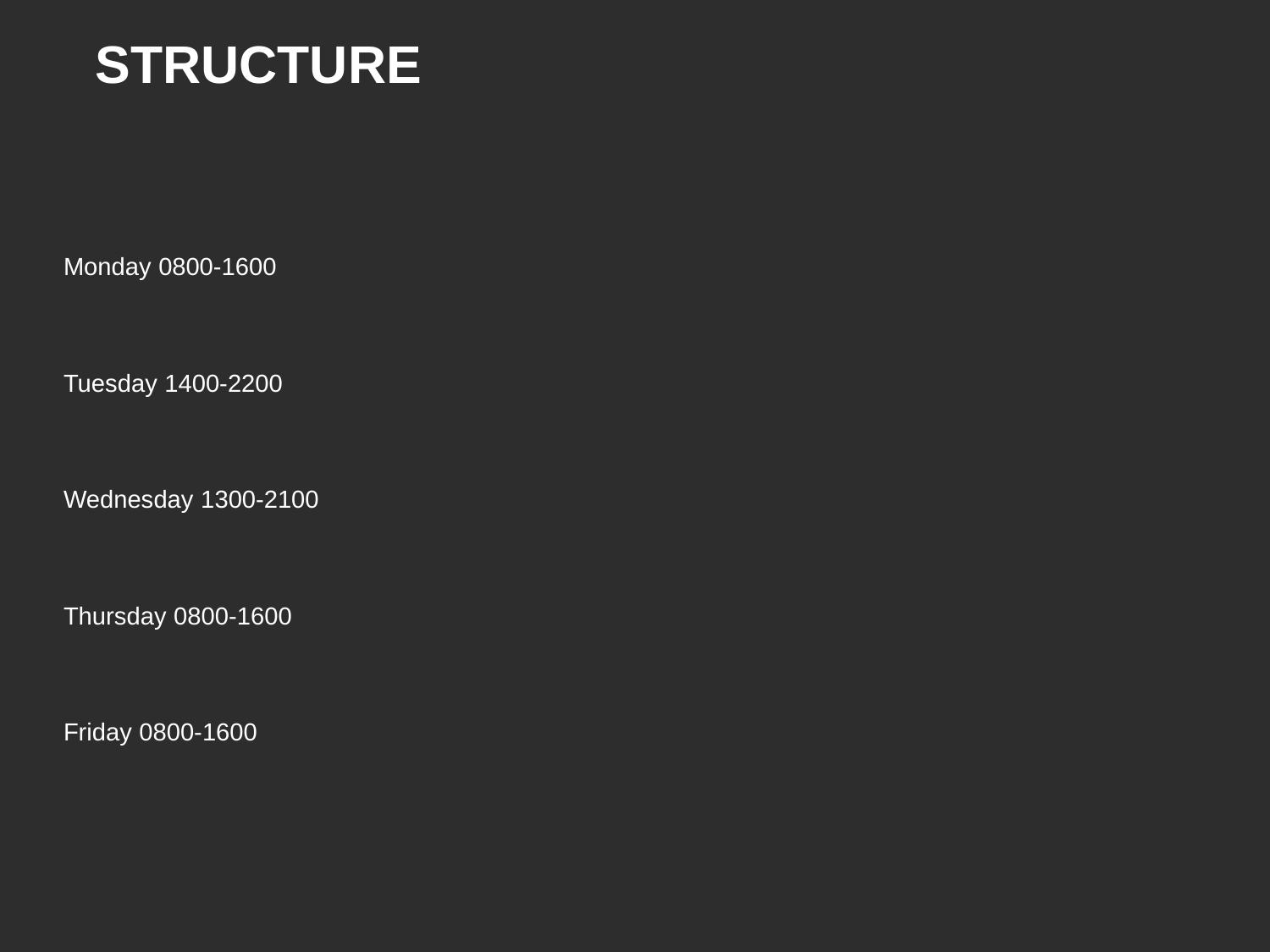

# Structure
Monday 0800-1600
Tuesday 1400-2200
Wednesday 1300-2100
Thursday 0800-1600
Friday 0800-1600

## Slide 11
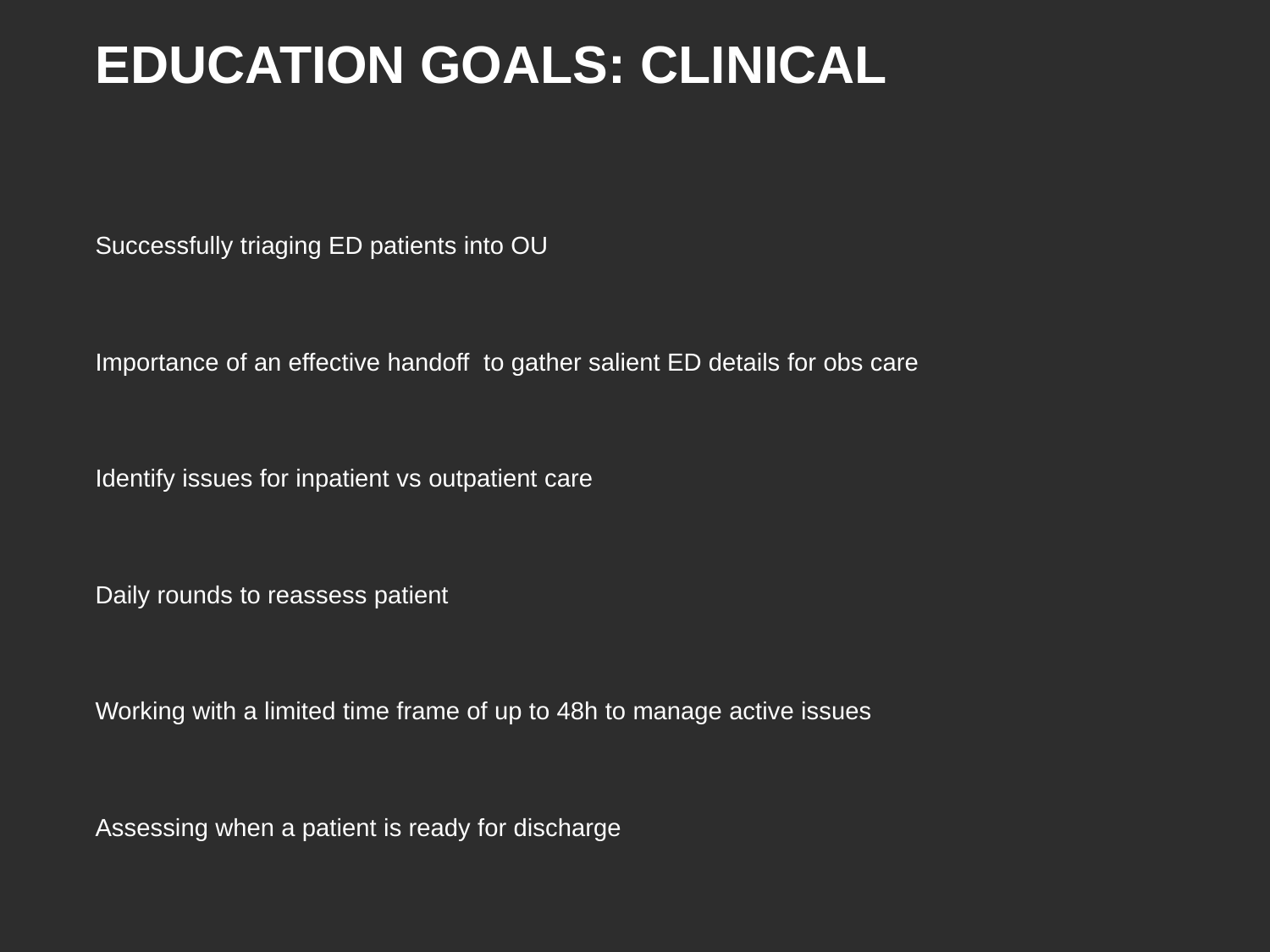

# Education Goals: Clinical
Successfully triaging ED patients into OU
Importance of an effective handoff  to gather salient ED details for obs care
Identify issues for inpatient vs outpatient care
Daily rounds to reassess patient
Working with a limited time frame of up to 48h to manage active issues
Assessing when a patient is ready for discharge

## Slide 12
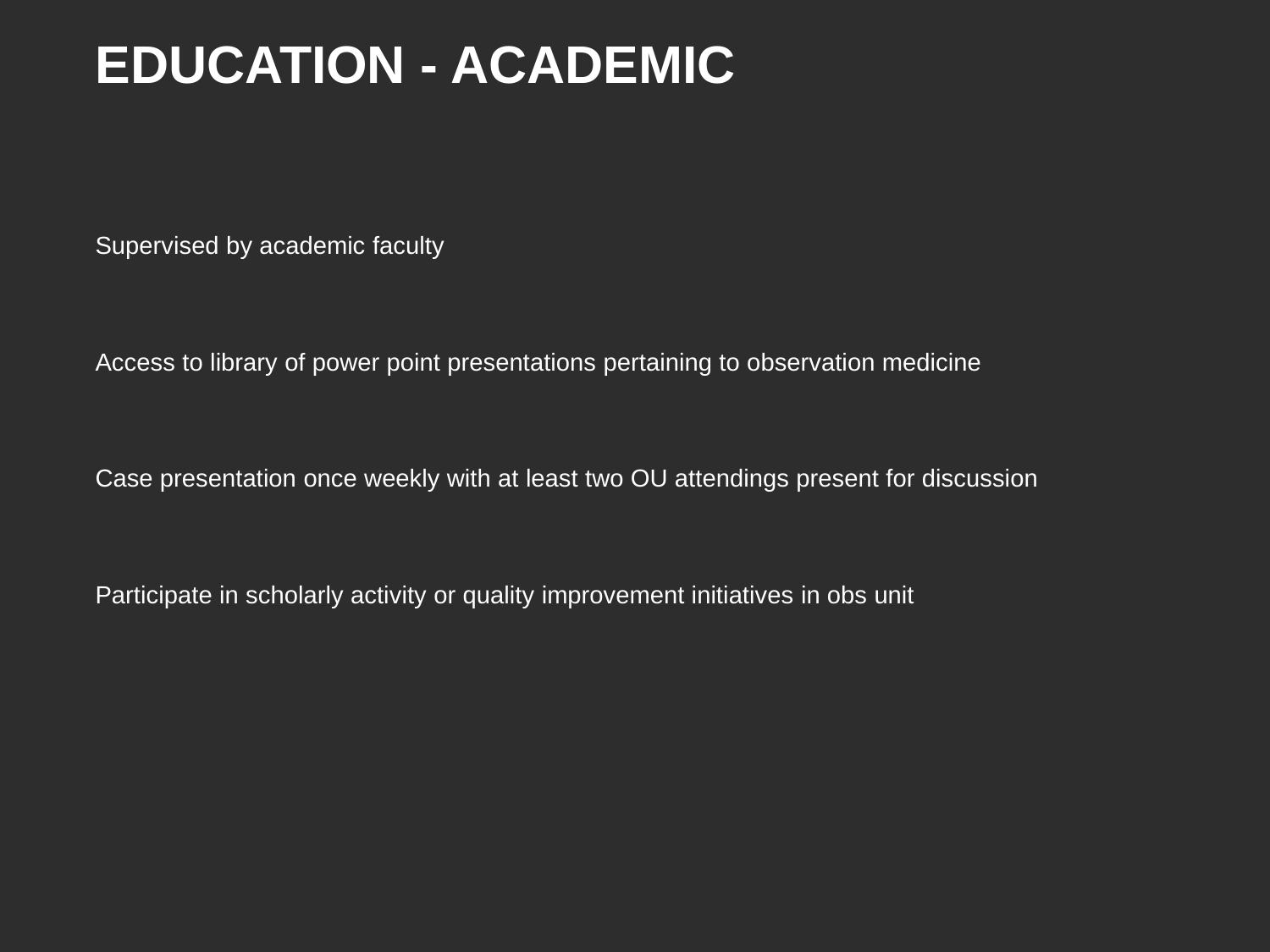

# Education - ACADEMIC
Supervised by academic faculty
Access to library of power point presentations pertaining to observation medicine
Case presentation once weekly with at least two OU attendings present for discussion
Participate in scholarly activity or quality improvement initiatives in obs unit

## Slide 13
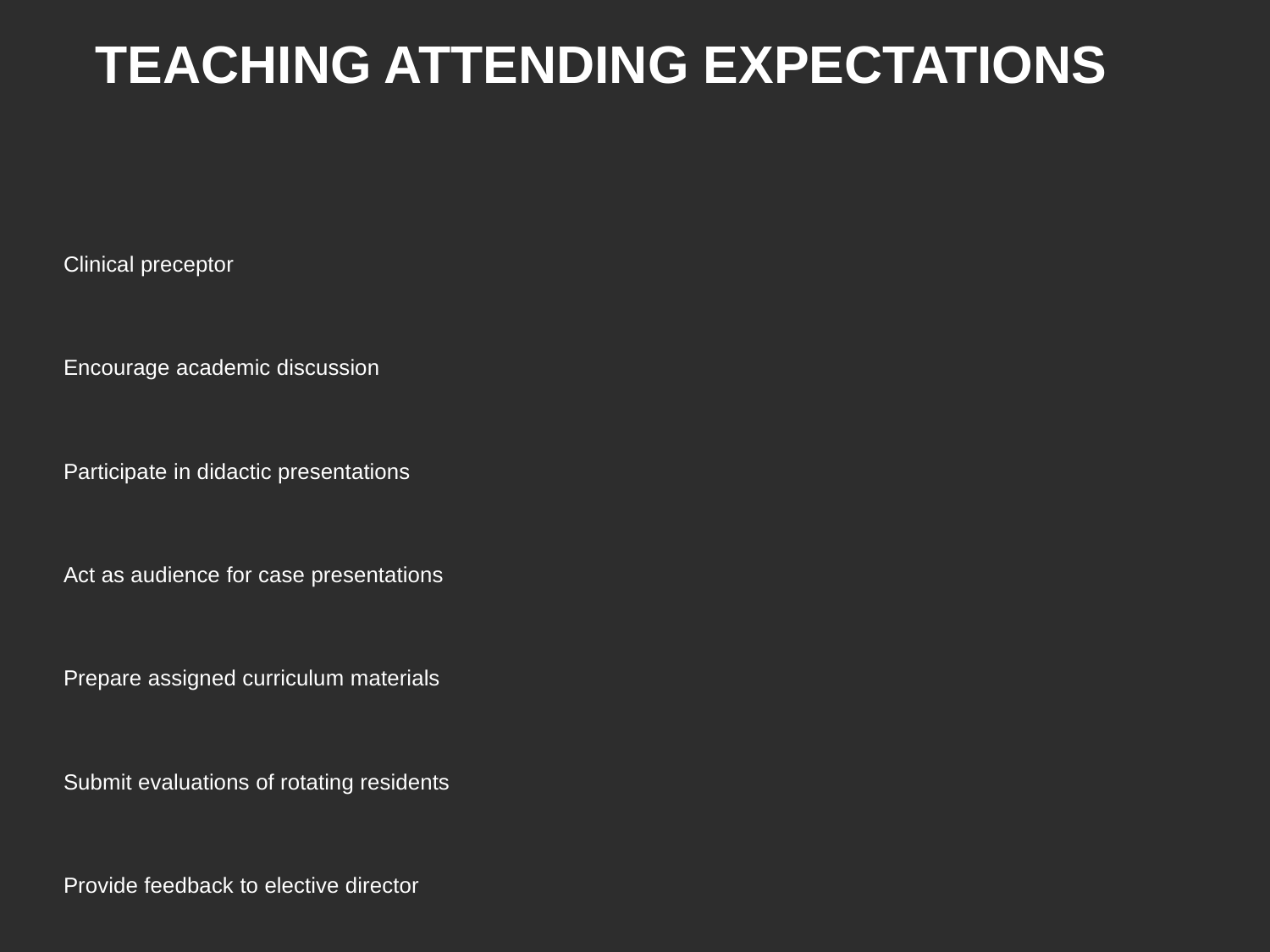

# Teaching Attending Expectations
Clinical preceptor
Encourage academic discussion
Participate in didactic presentations
Act as audience for case presentations
Prepare assigned curriculum materials
Submit evaluations of rotating residents
Provide feedback to elective director

## Slide 14
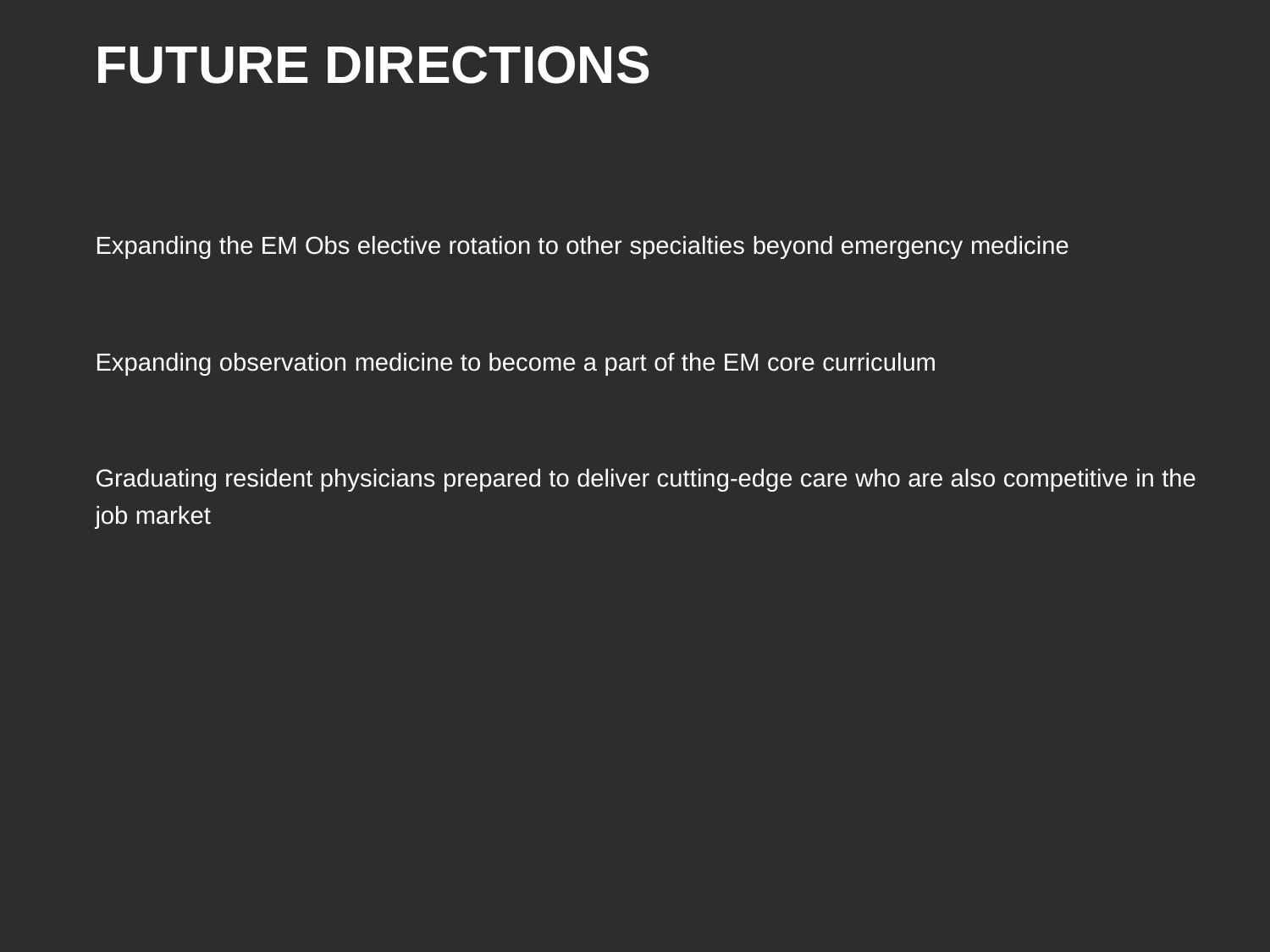

# Future directions
Expanding the EM Obs elective rotation to other specialties beyond emergency medicine
Expanding observation medicine to become a part of the EM core curriculum
Graduating resident physicians prepared to deliver cutting-edge care who are also competitive in the job market

## Slide 15
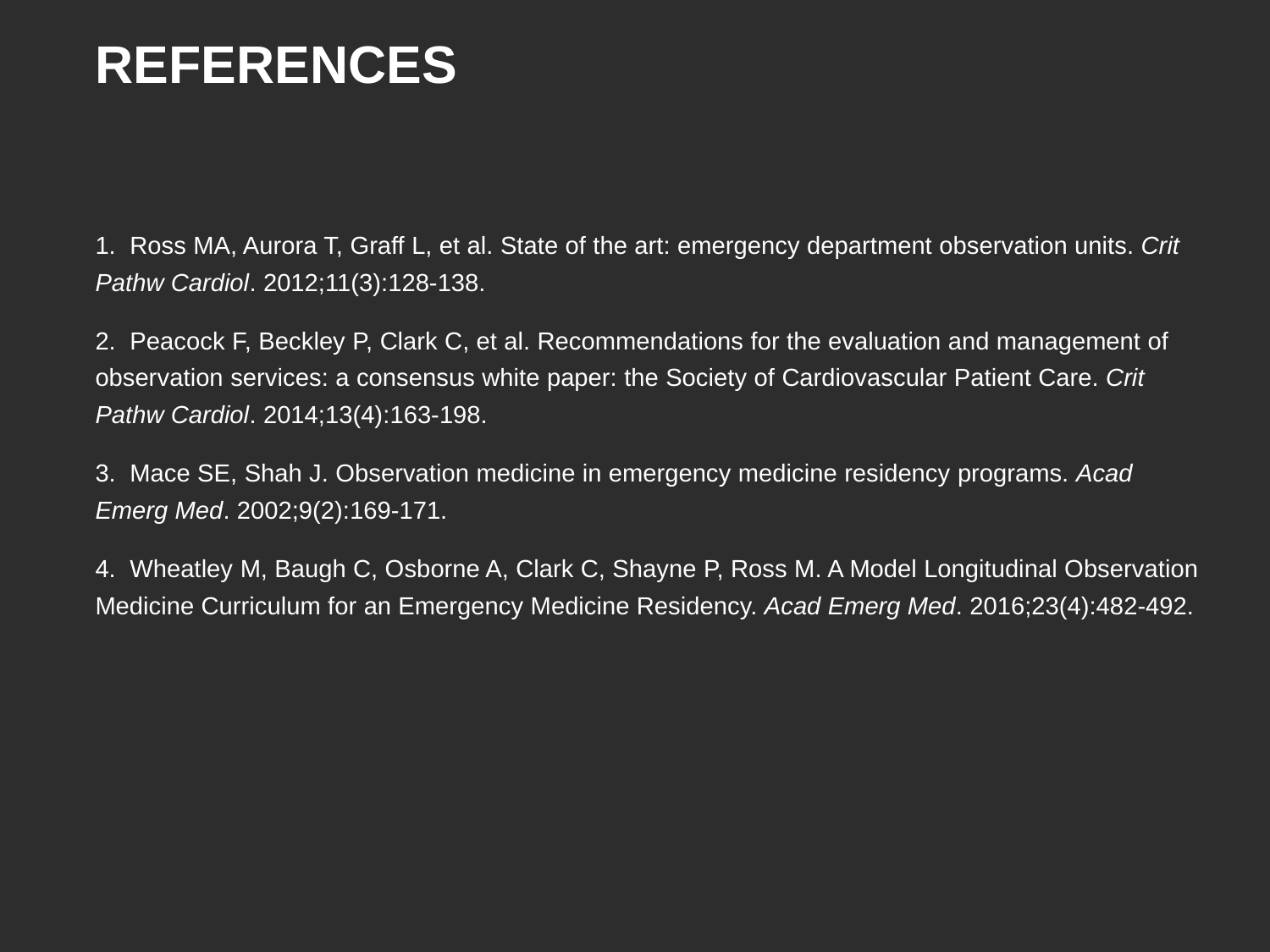

# references
1. Ross MA, Aurora T, Graff L, et al. State of the art: emergency department observation units. Crit Pathw Cardiol. 2012;11(3):128-138.
2. Peacock F, Beckley P, Clark C, et al. Recommendations for the evaluation and management of observation services: a consensus white paper: the Society of Cardiovascular Patient Care. Crit Pathw Cardiol. 2014;13(4):163-198.
3. Mace SE, Shah J. Observation medicine in emergency medicine residency programs. Acad Emerg Med. 2002;9(2):169-171.
4. Wheatley M, Baugh C, Osborne A, Clark C, Shayne P, Ross M. A Model Longitudinal Observation Medicine Curriculum for an Emergency Medicine Residency. Acad Emerg Med. 2016;23(4):482-492.
