# Supplementary material for: An Observation Medicine Curriculum for Emergency Medicine Education
Source: J Educ Teach Emerg Med. 2021 Apr 19;6(2):C1–C72. doi: 10.21980/J87P92 (PMC10332786; doi:10.21980/J87P92)
Supplement: Supplementary file 15 — Please see associated PowerPoint file [file jetem-6-2-c1-supp15.pptx]

## Slide 1
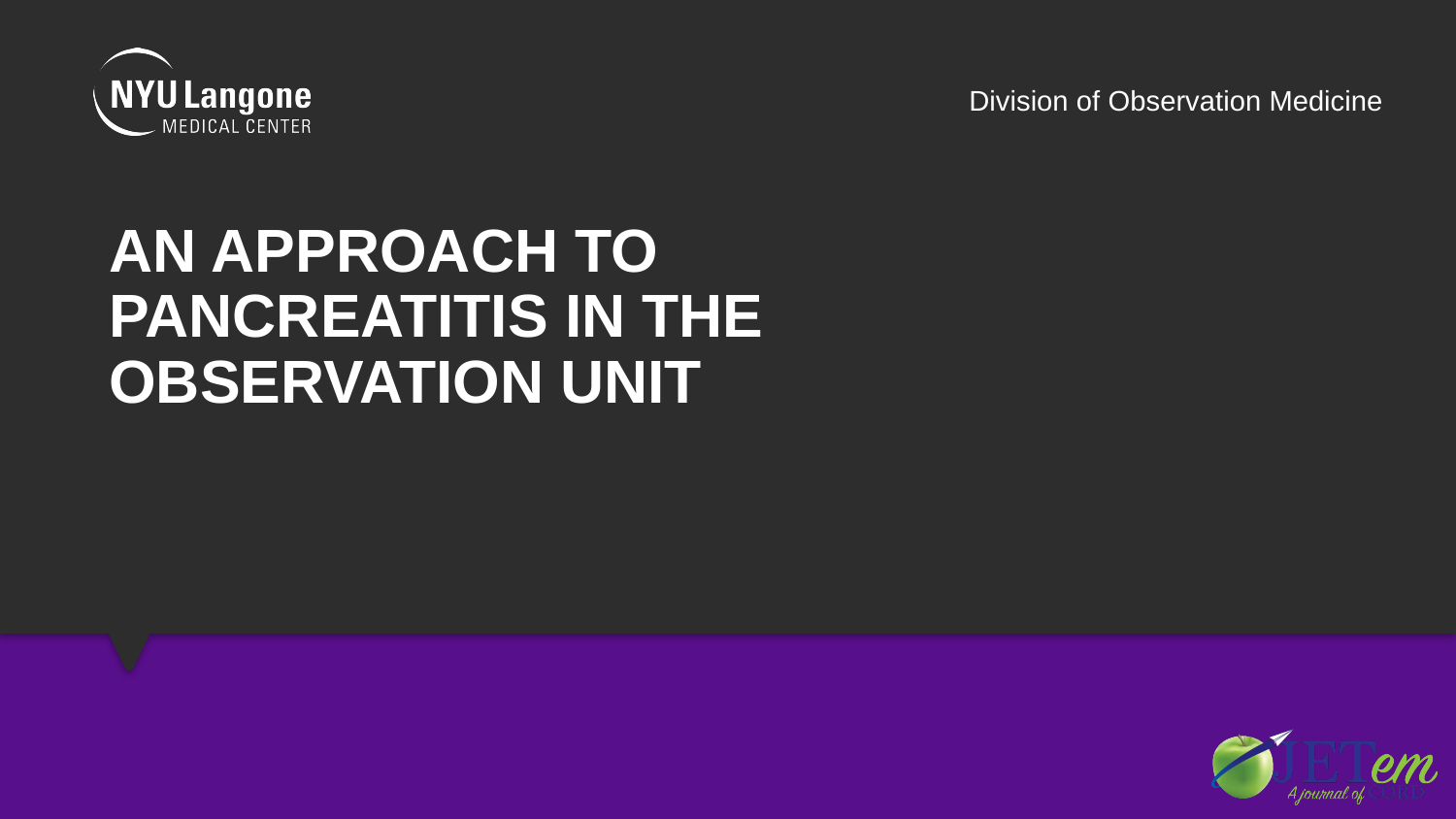

Division of Observation Medicine
# AN APPROACH TO pancreatitis in the observation unit

## Slide 2
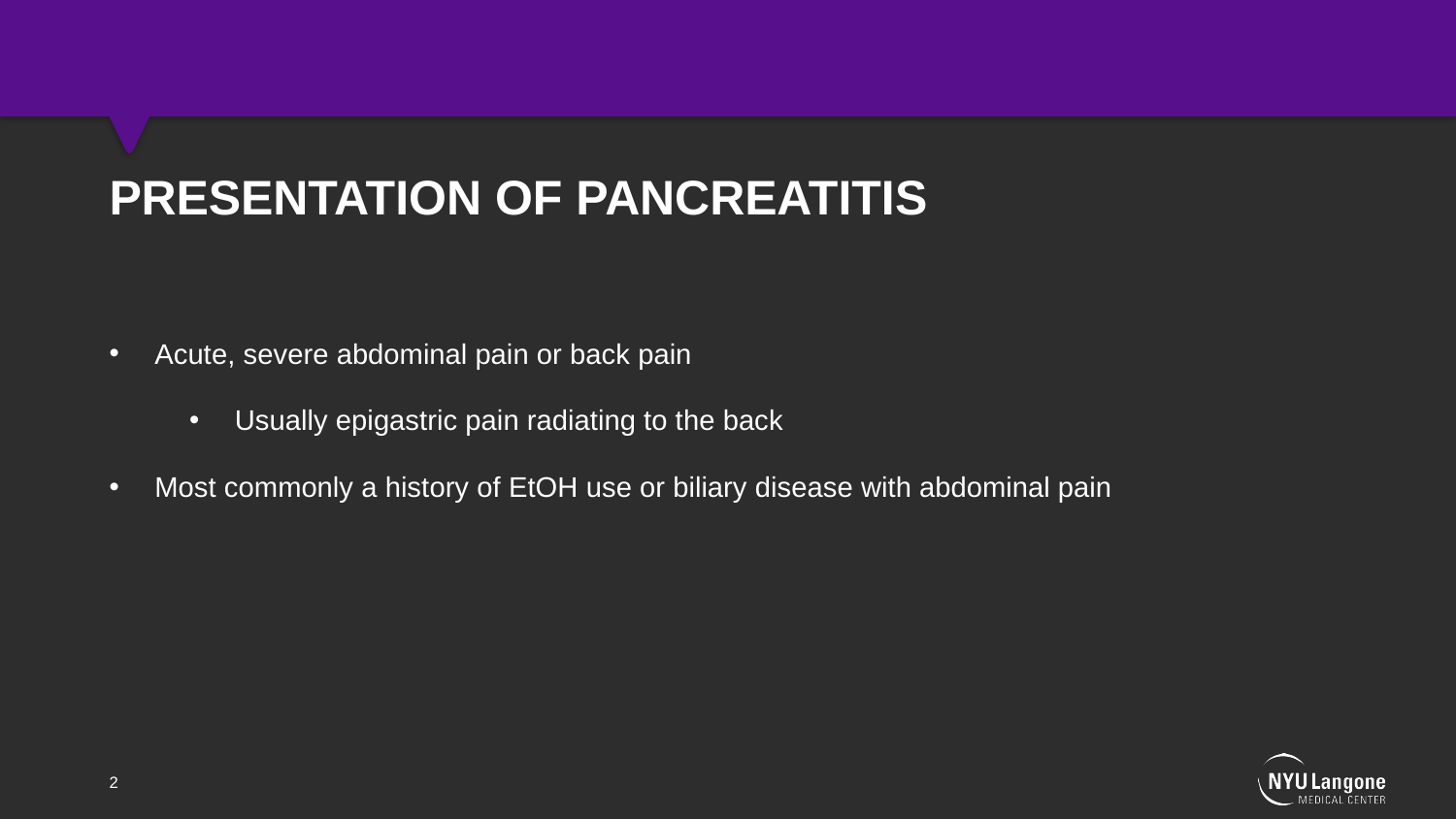

# PRESENTATION OF PANCREATITIS
Acute, severe abdominal pain or back pain
Usually epigastric pain radiating to the back
Most commonly a history of EtOH use or biliary disease with abdominal pain
2

## Slide 3
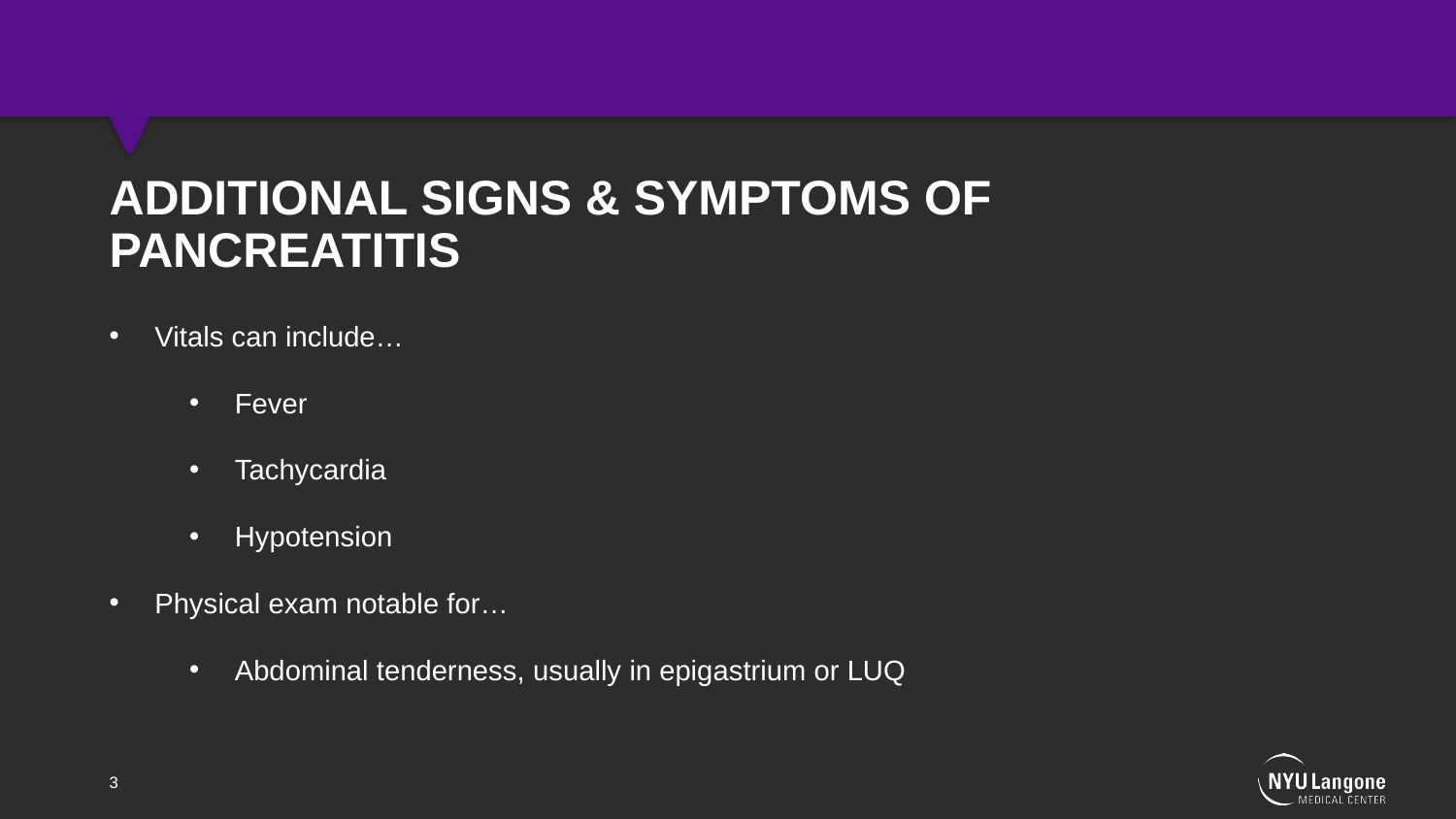

# ADDITIONAL SIGNS & SYMPTOMS OF PANCREATITIS
Vitals can include…
Fever
Tachycardia
Hypotension
Physical exam notable for…
Abdominal tenderness, usually in epigastrium or LUQ
3

## Slide 4
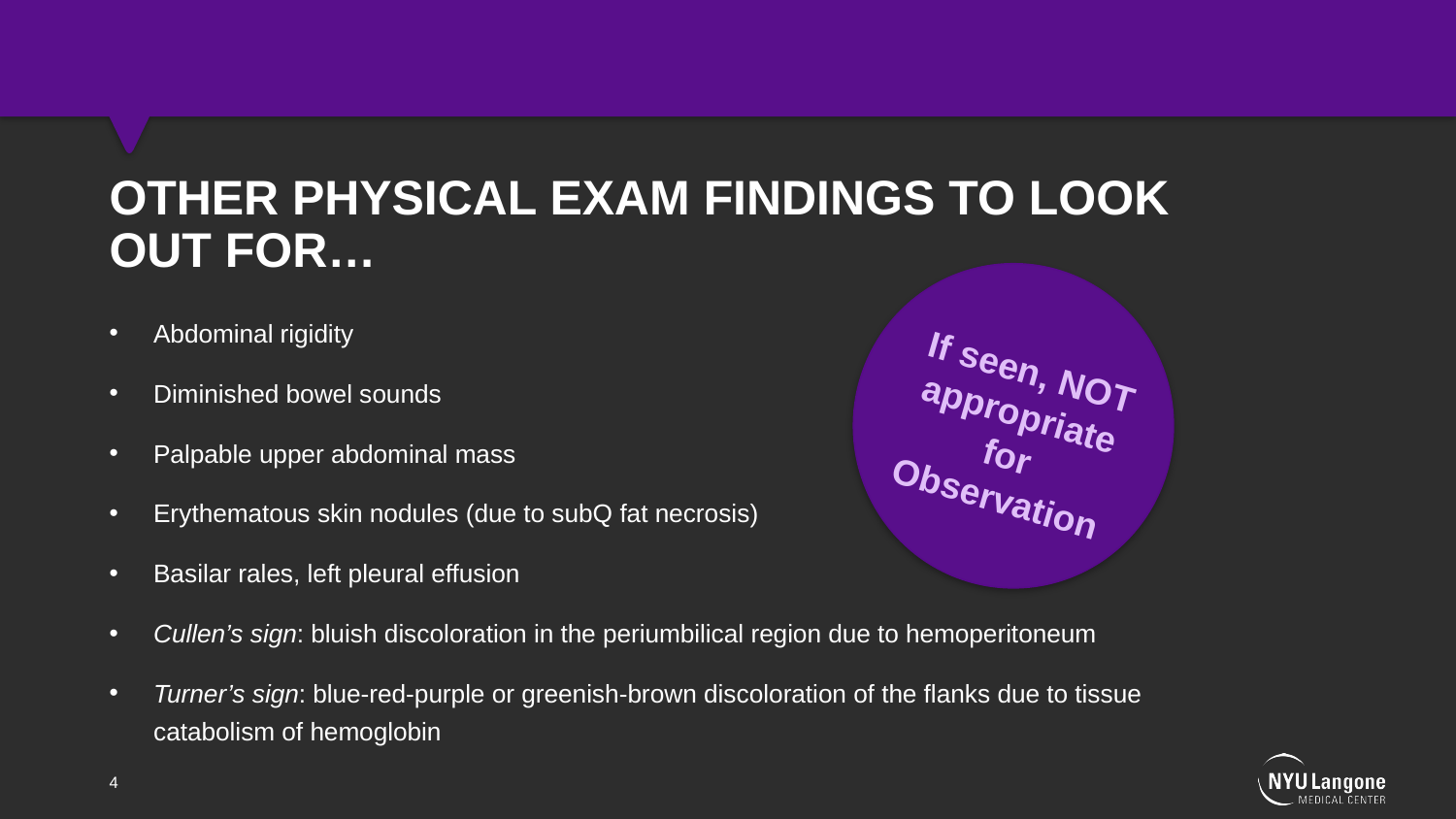

# OTHER PHYSICAL EXAM FINDINGS TO LOOK OUT FOR…
Abdominal rigidity
Diminished bowel sounds
Palpable upper abdominal mass
Erythematous skin nodules (due to subQ fat necrosis)
Basilar rales, left pleural effusion
Cullen’s sign: bluish discoloration in the periumbilical region due to hemoperitoneum
Turner’s sign: blue-red-purple or greenish-brown discoloration of the flanks due to tissue catabolism of hemoglobin
If seen, NOT
appropriate
for
Observation
4

## Slide 5
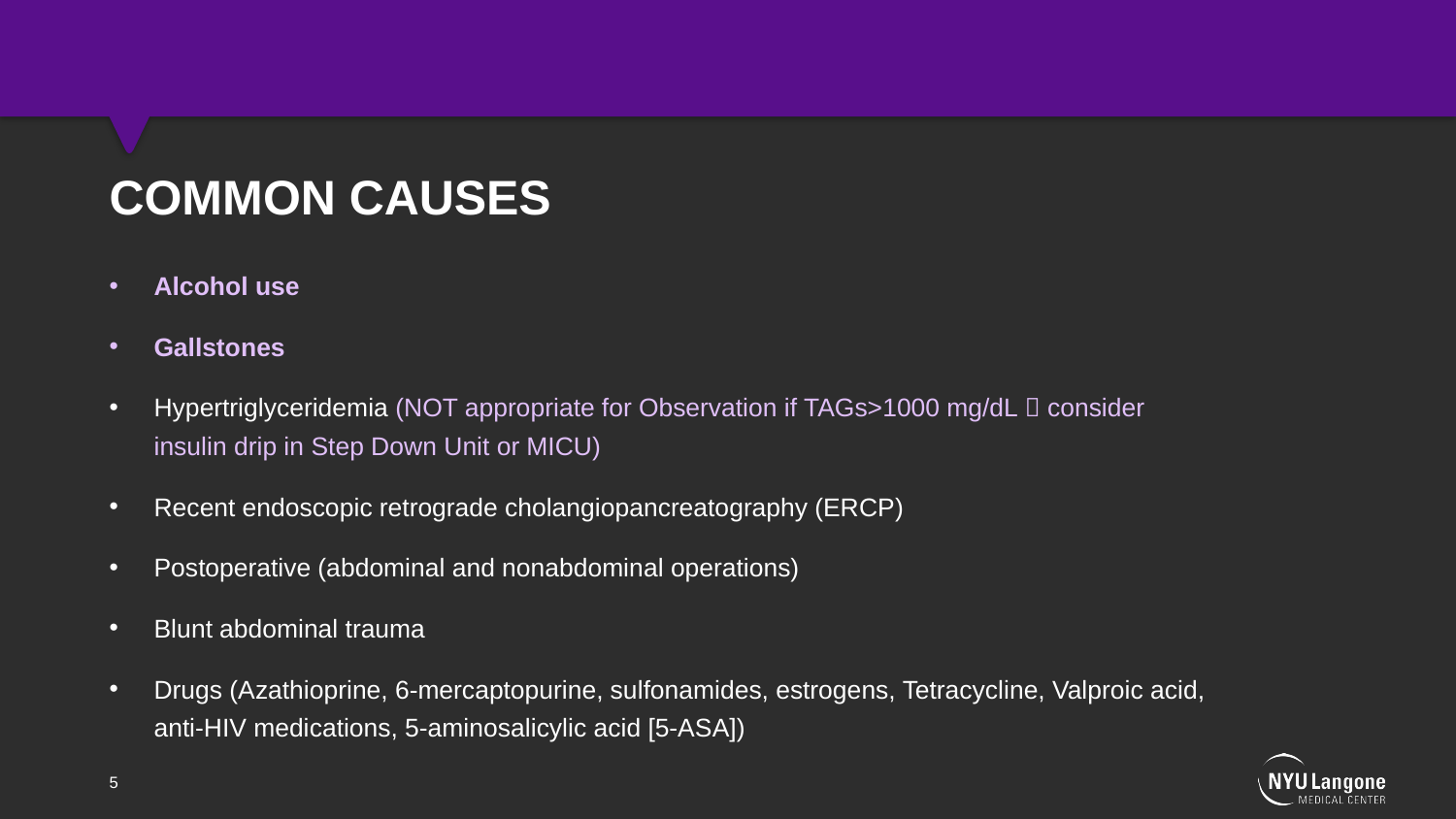

# COMMON CAUSES
Alcohol use
Gallstones
Hypertriglyceridemia (NOT appropriate for Observation if TAGs>1000 mg/dL  consider insulin drip in Step Down Unit or MICU)
Recent endoscopic retrograde cholangiopancreatography (ERCP)
Postoperative (abdominal and nonabdominal operations)
Blunt abdominal trauma
Drugs (Azathioprine, 6-mercaptopurine, sulfonamides, estrogens, Tetracycline, Valproic acid, anti-HIV medications, 5-aminosalicylic acid [5-ASA])
5

## Slide 6
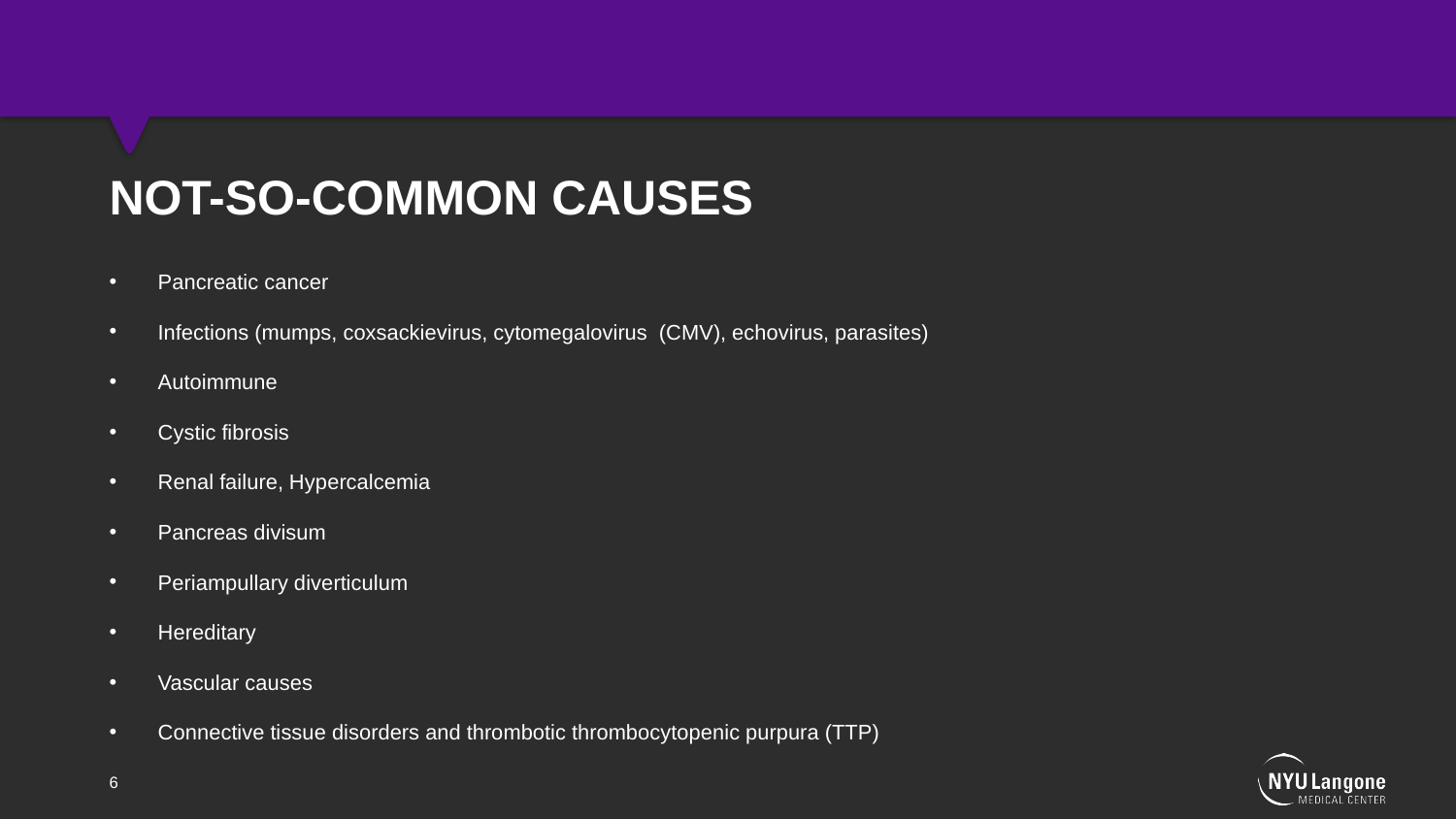

# NOT-SO-COMMON CAUSES
Pancreatic cancer
Infections (mumps, coxsackievirus, cytomegalovirus (CMV), echovirus, parasites)
Autoimmune
Cystic fibrosis
Renal failure, Hypercalcemia
Pancreas divisum
Periampullary diverticulum
Hereditary
Vascular causes
Connective tissue disorders and thrombotic thrombocytopenic purpura (TTP)
6

## Slide 7
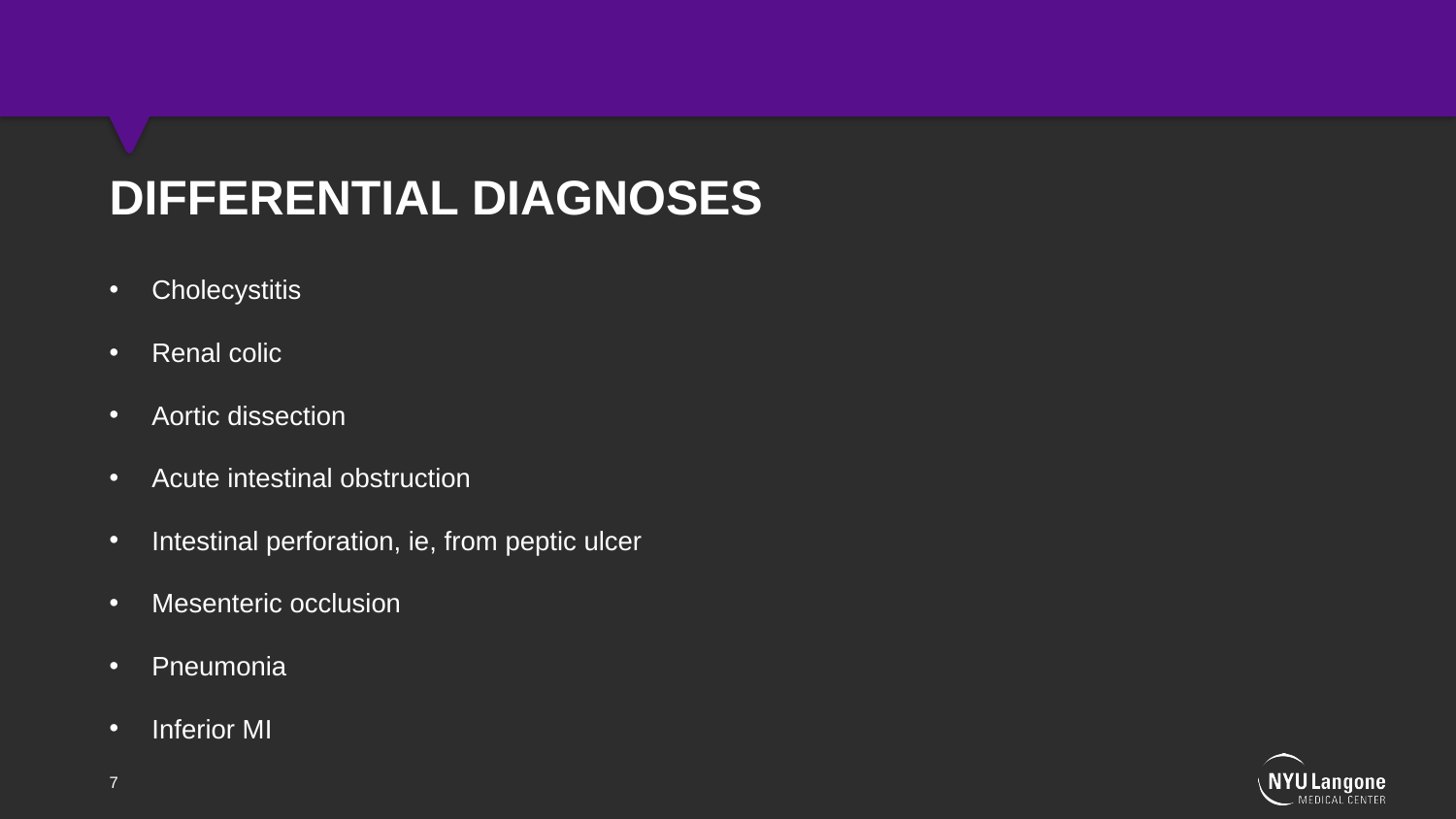

# DIFFERENTIAL DIAGNOSES
Cholecystitis
Renal colic
Aortic dissection
Acute intestinal obstruction
Intestinal perforation, ie, from peptic ulcer
Mesenteric occlusion
Pneumonia
Inferior MI
7

## Slide 8
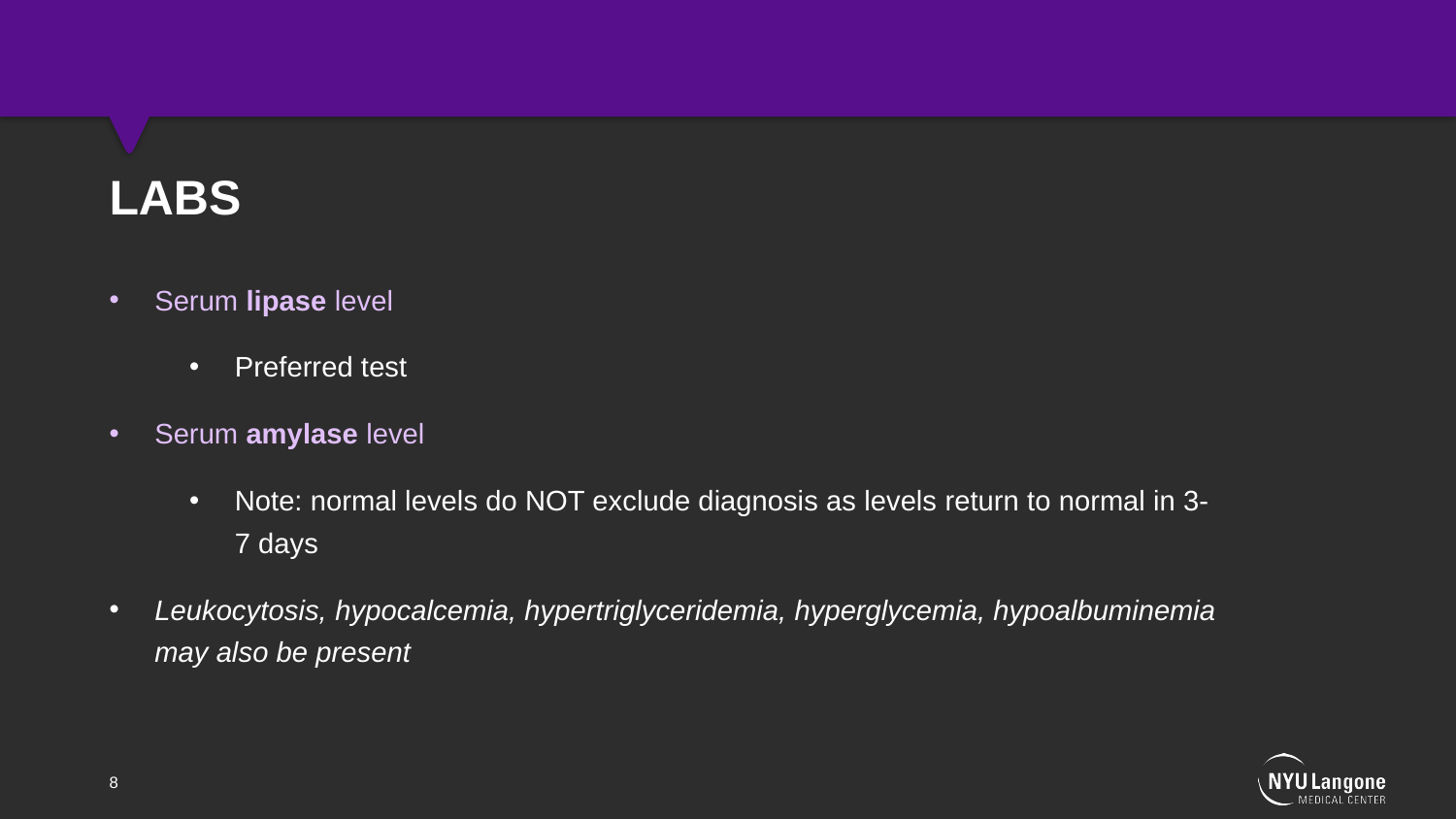

# LABS
Serum lipase level
Preferred test
Serum amylase level
Note: normal levels do NOT exclude diagnosis as levels return to normal in 3-7 days
Leukocytosis, hypocalcemia, hypertriglyceridemia, hyperglycemia, hypoalbuminemia may also be present
8

## Slide 9
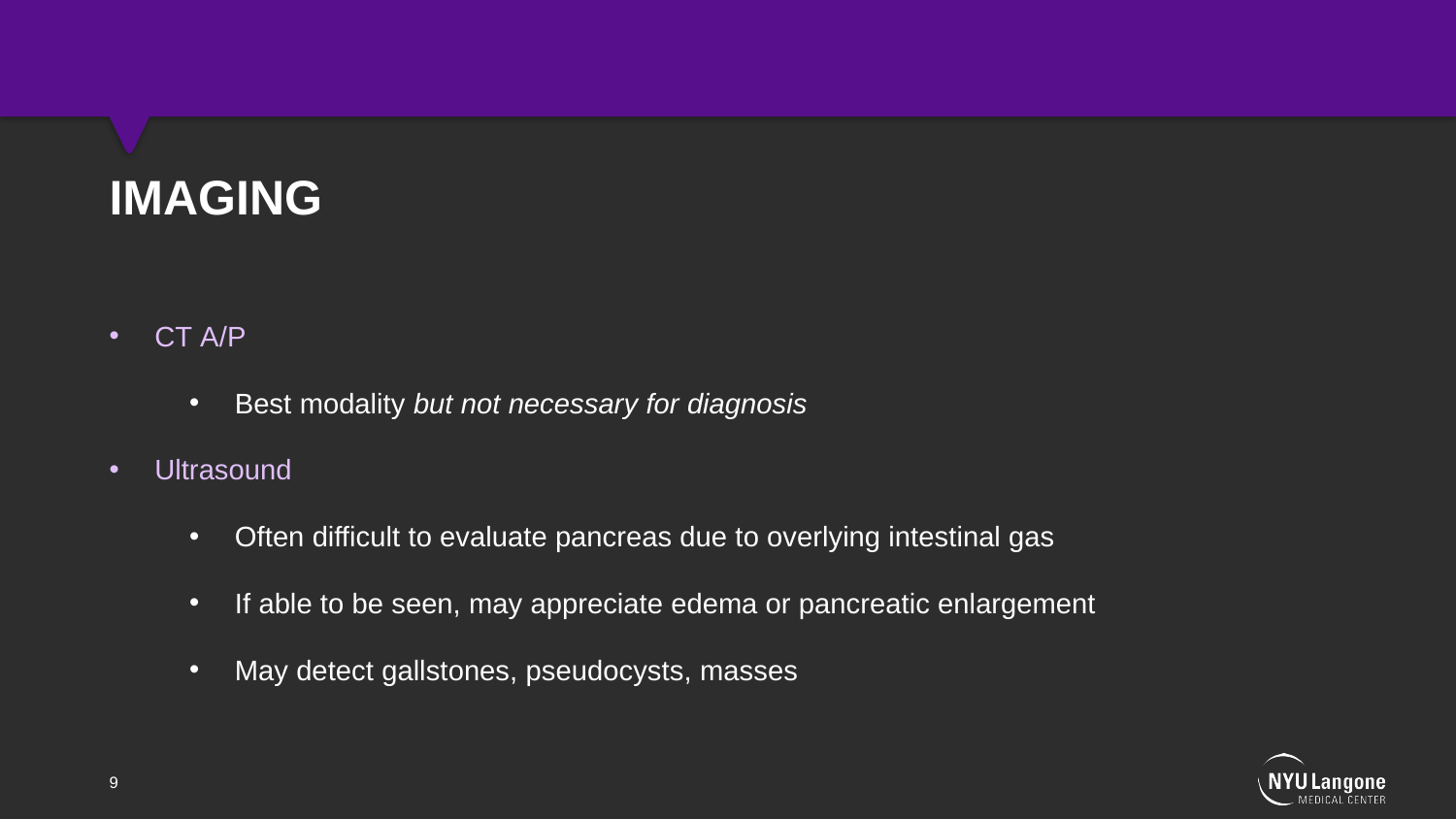

# IMAGING
CT A/P
Best modality but not necessary for diagnosis
Ultrasound
Often difficult to evaluate pancreas due to overlying intestinal gas
If able to be seen, may appreciate edema or pancreatic enlargement
May detect gallstones, pseudocysts, masses
9

## Slide 10
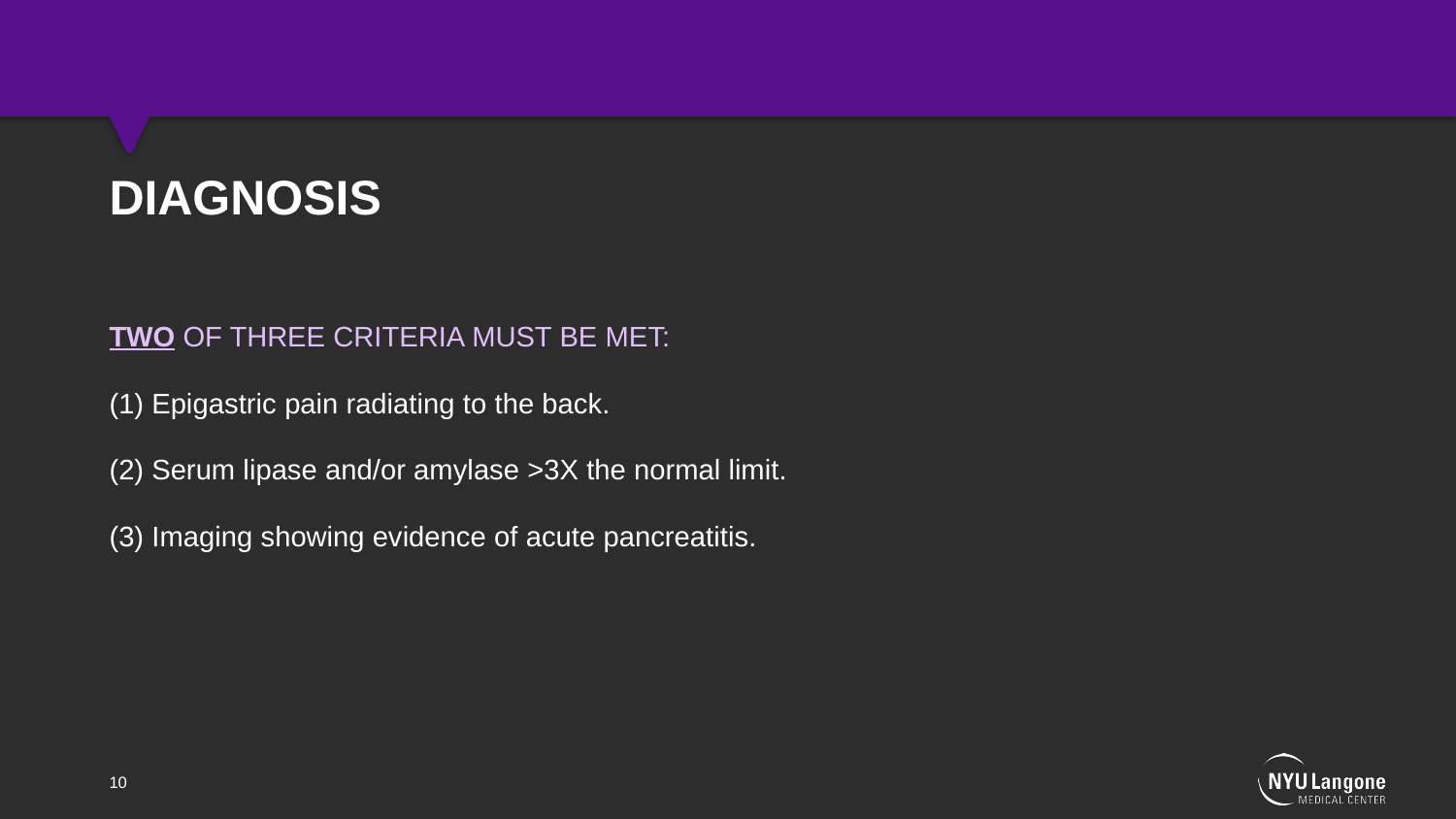

# DIAGNOSIS
TWO OF THREE CRITERIA MUST BE MET:
(1) Epigastric pain radiating to the back.
(2) Serum lipase and/or amylase >3X the normal limit.
(3) Imaging showing evidence of acute pancreatitis.
10

## Slide 11
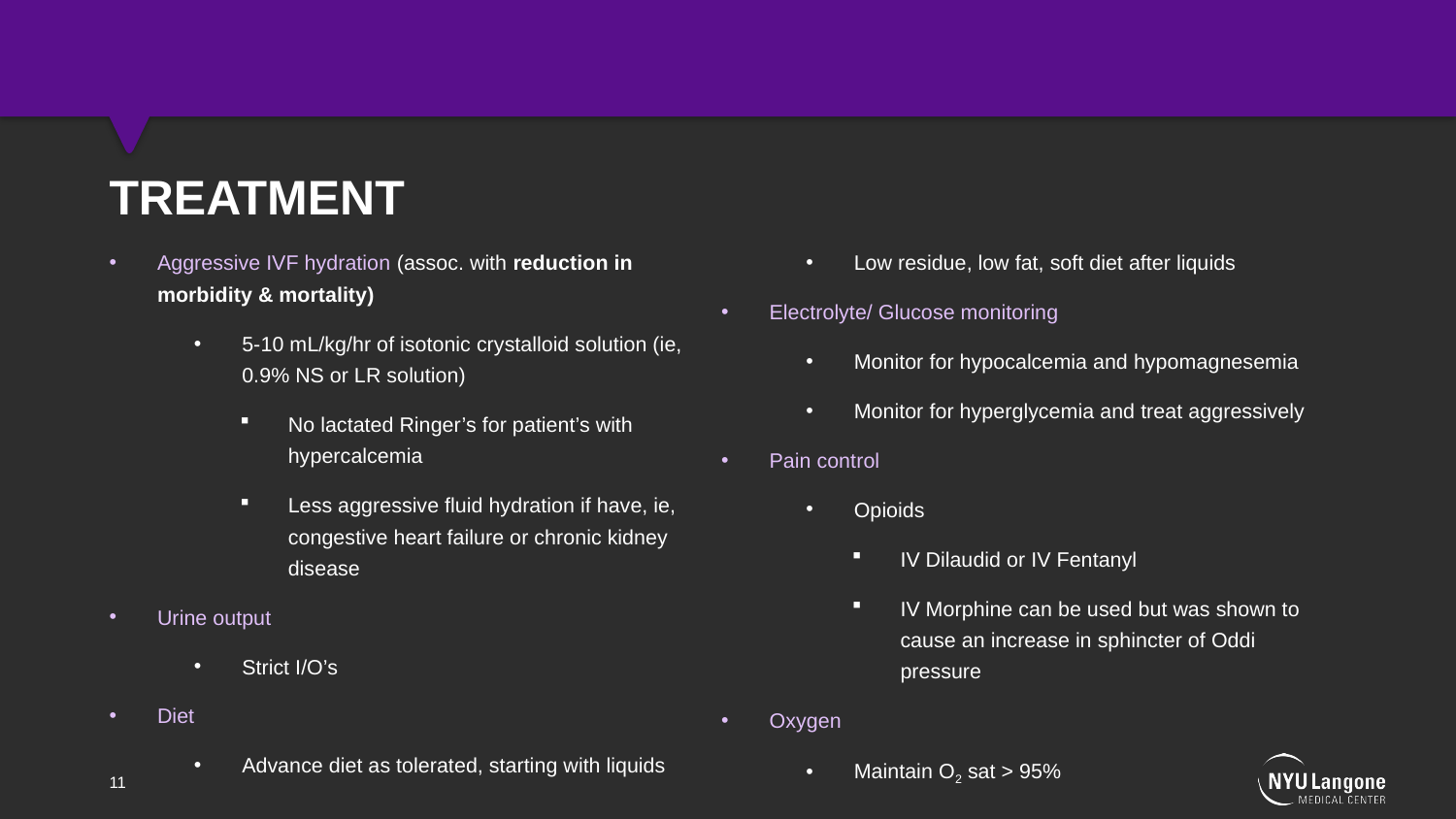

# TREATMENT
Aggressive IVF hydration (assoc. with reduction in morbidity & mortality)
5-10 mL/kg/hr of isotonic crystalloid solution (ie, 0.9% NS or LR solution)
No lactated Ringer’s for patient’s with hypercalcemia
Less aggressive fluid hydration if have, ie, congestive heart failure or chronic kidney disease
Urine output
Strict I/O’s
Diet
Advance diet as tolerated, starting with liquids
Low residue, low fat, soft diet after liquids
Electrolyte/ Glucose monitoring
Monitor for hypocalcemia and hypomagnesemia
Monitor for hyperglycemia and treat aggressively
Pain control
Opioids
IV Dilaudid or IV Fentanyl
IV Morphine can be used but was shown to cause an increase in sphincter of Oddi pressure
Oxygen
Maintain O2 sat > 95%
11

## Slide 12
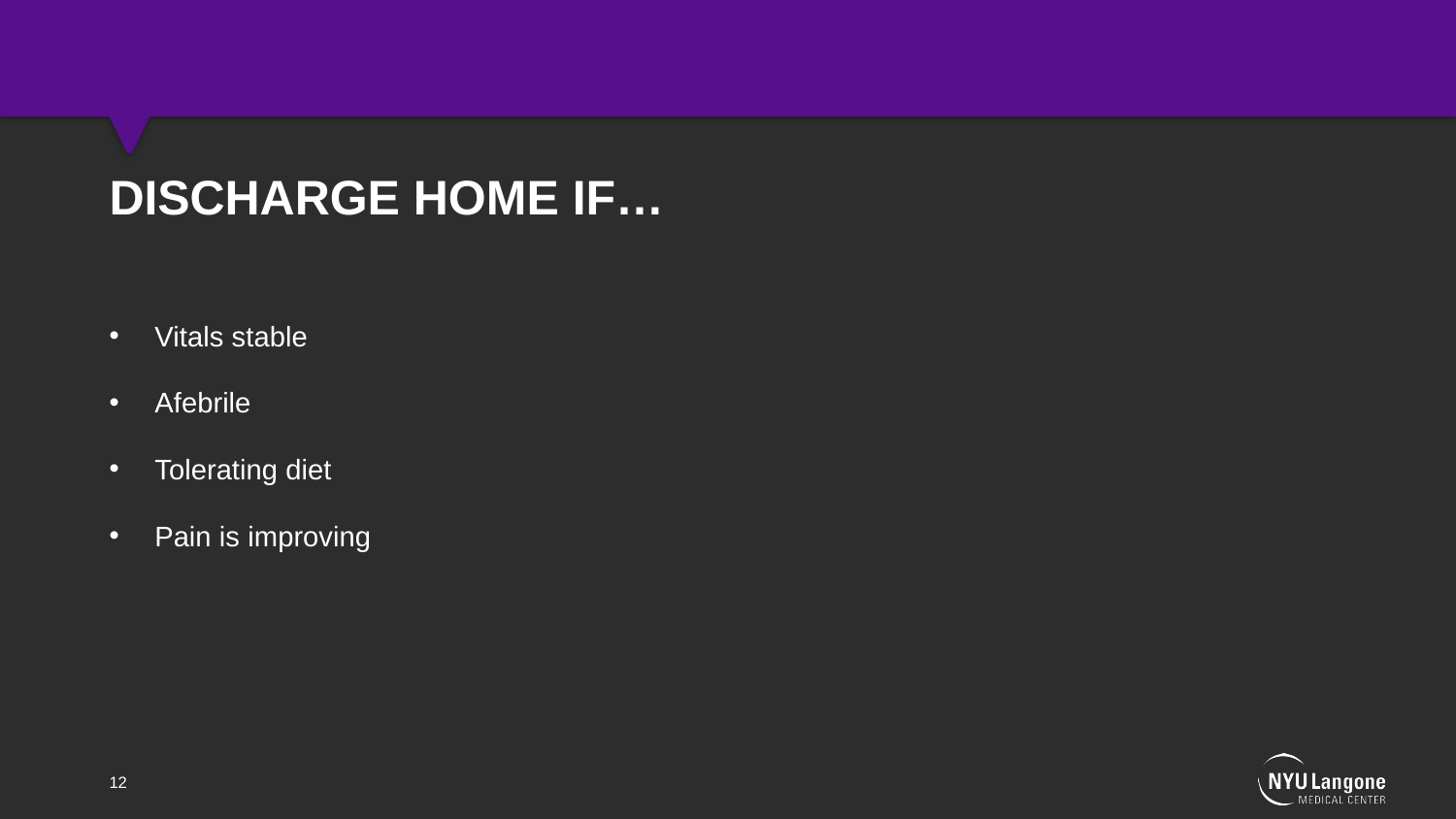

# DISCHARGE HOME IF…
Vitals stable
Afebrile
Tolerating diet
Pain is improving
12

## Slide 13
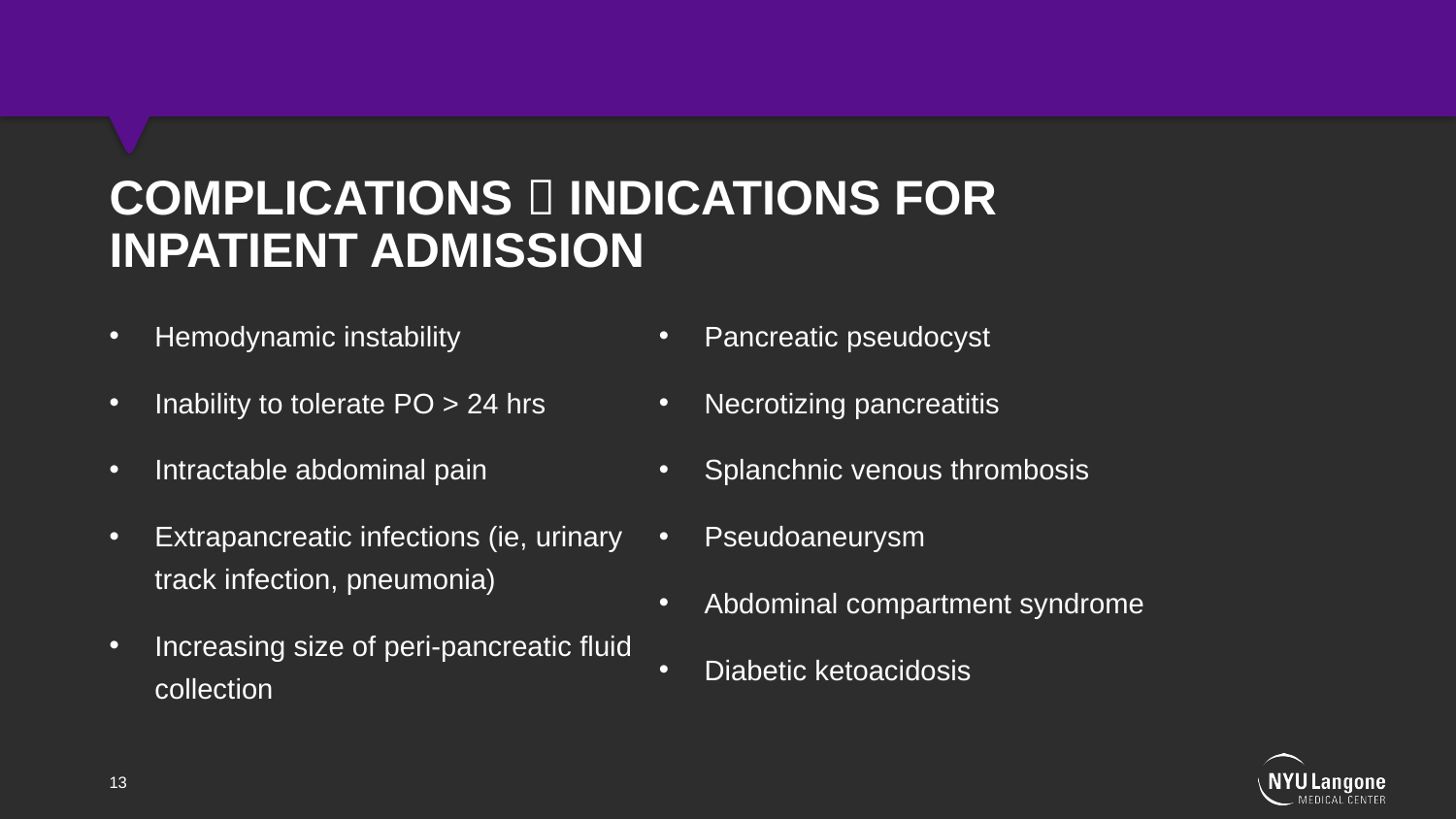

# COMPLICATIONS  INDICATIONS FOR INPATIENT ADMISSION
Hemodynamic instability
Inability to tolerate PO > 24 hrs
Intractable abdominal pain
Extrapancreatic infections (ie, urinary track infection, pneumonia)
Increasing size of peri-pancreatic fluid collection
Pancreatic pseudocyst
Necrotizing pancreatitis
Splanchnic venous thrombosis
Pseudoaneurysm
Abdominal compartment syndrome
Diabetic ketoacidosis
13

## Slide 14
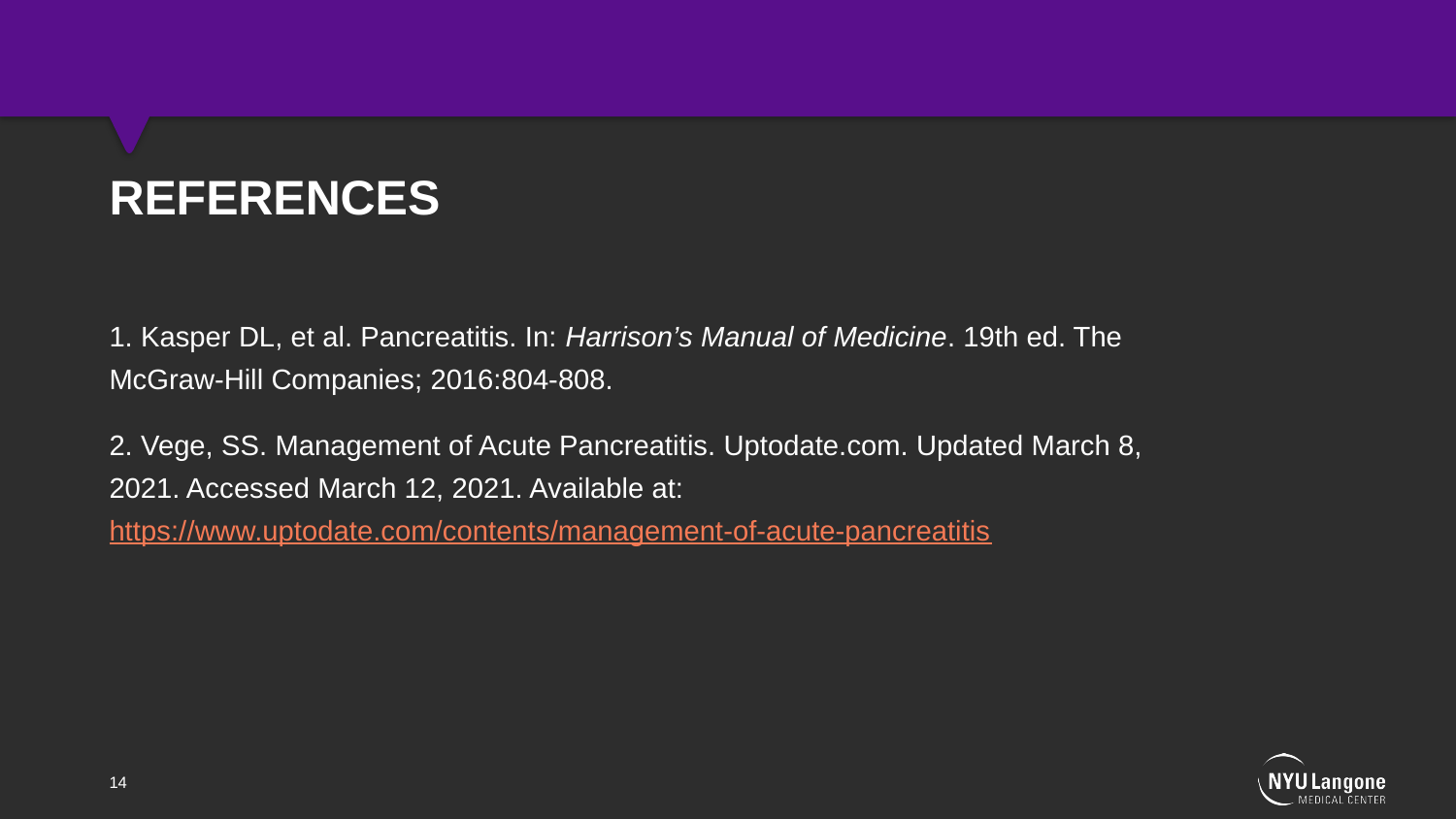

# REFERENCES
1. Kasper DL, et al. Pancreatitis. In: Harrison’s Manual of Medicine. 19th ed. The McGraw-Hill Companies; 2016:804-808.
2. Vege, SS. Management of Acute Pancreatitis. Uptodate.com. Updated March 8, 2021. Accessed March 12, 2021. Available at: https://www.uptodate.com/contents/management-of-acute-pancreatitis
14
