# Supplementary material for: An Observation Medicine Curriculum for Emergency Medicine Education
Source: J Educ Teach Emerg Med. 2021 Apr 19;6(2):C1–C72. doi: 10.21980/J87P92 (PMC10332786; doi:10.21980/J87P92)
Supplement: Supplementary file 16 — Please see associated PowerPoint file [file jetem-6-2-c1-supp16.pptx]

## Slide 1
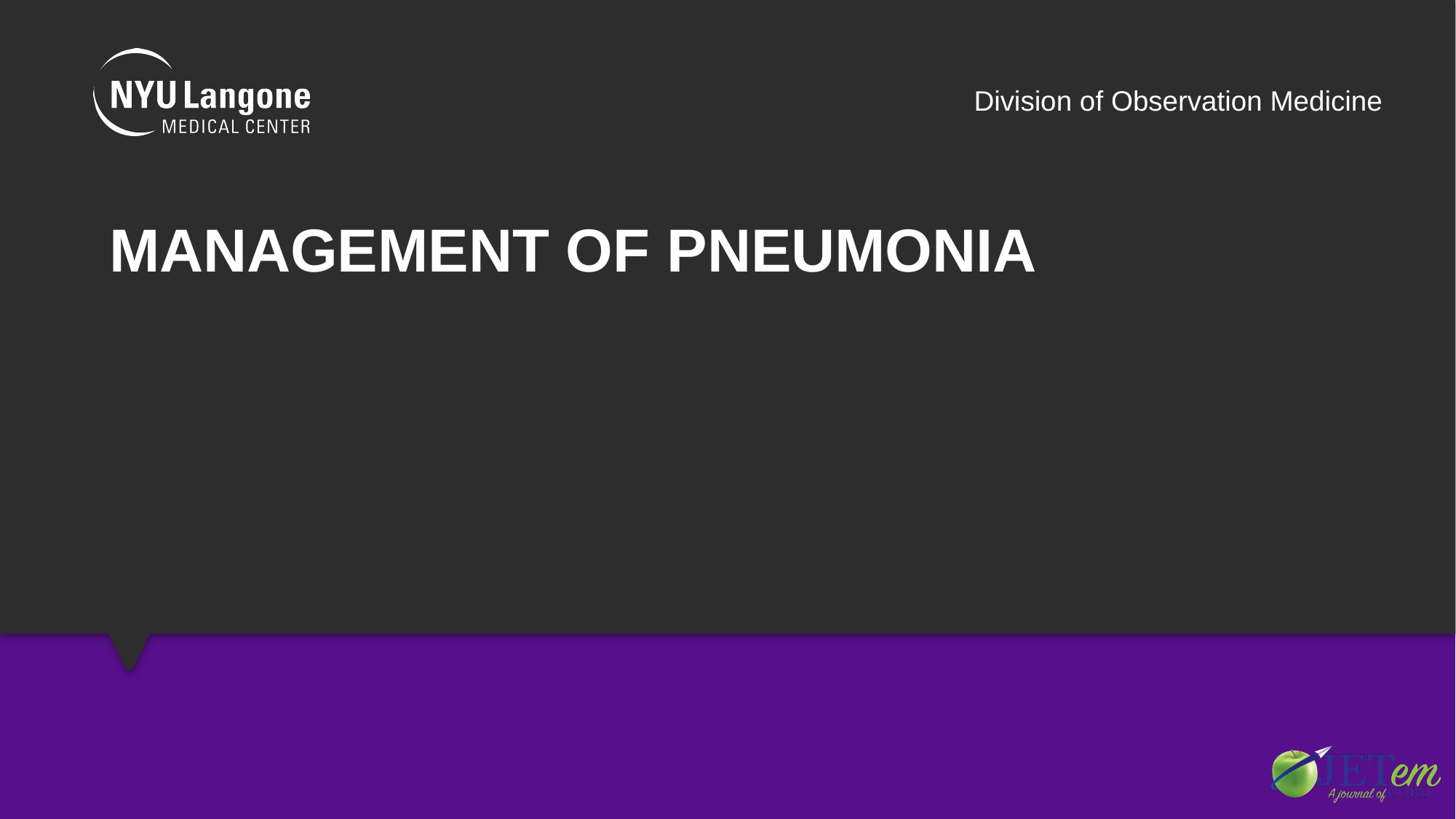

Division of Observation Medicine
# Management of Pneumonia

## Slide 2
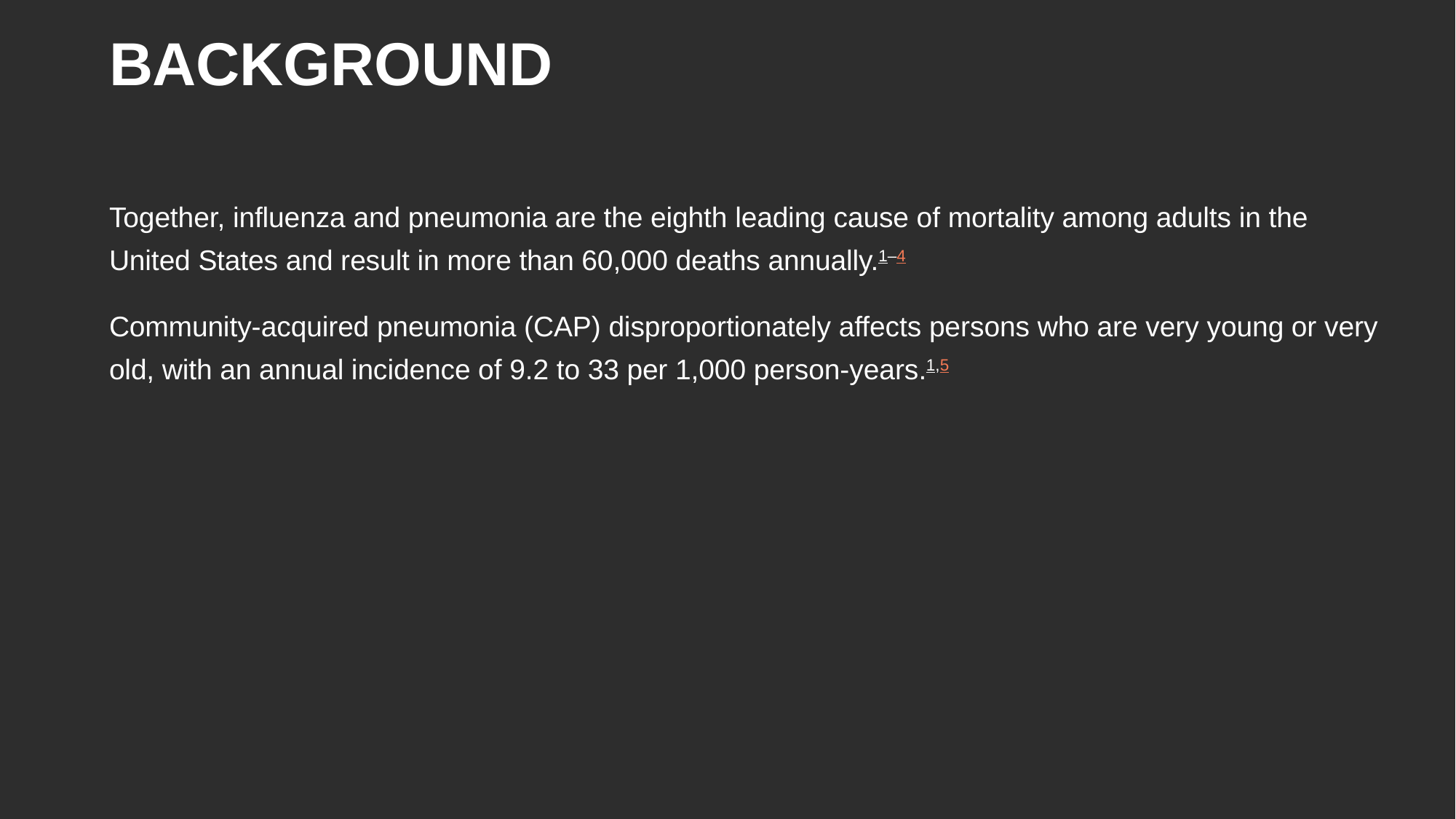

# BAckground
Together, influenza and pneumonia are the eighth leading cause of mortality among adults in the United States and result in more than 60,000 deaths annually.1–4
Community-acquired pneumonia (CAP) disproportionately affects persons who are very young or very old, with an annual incidence of 9.2 to 33 per 1,000 person-years.1,5

## Slide 3
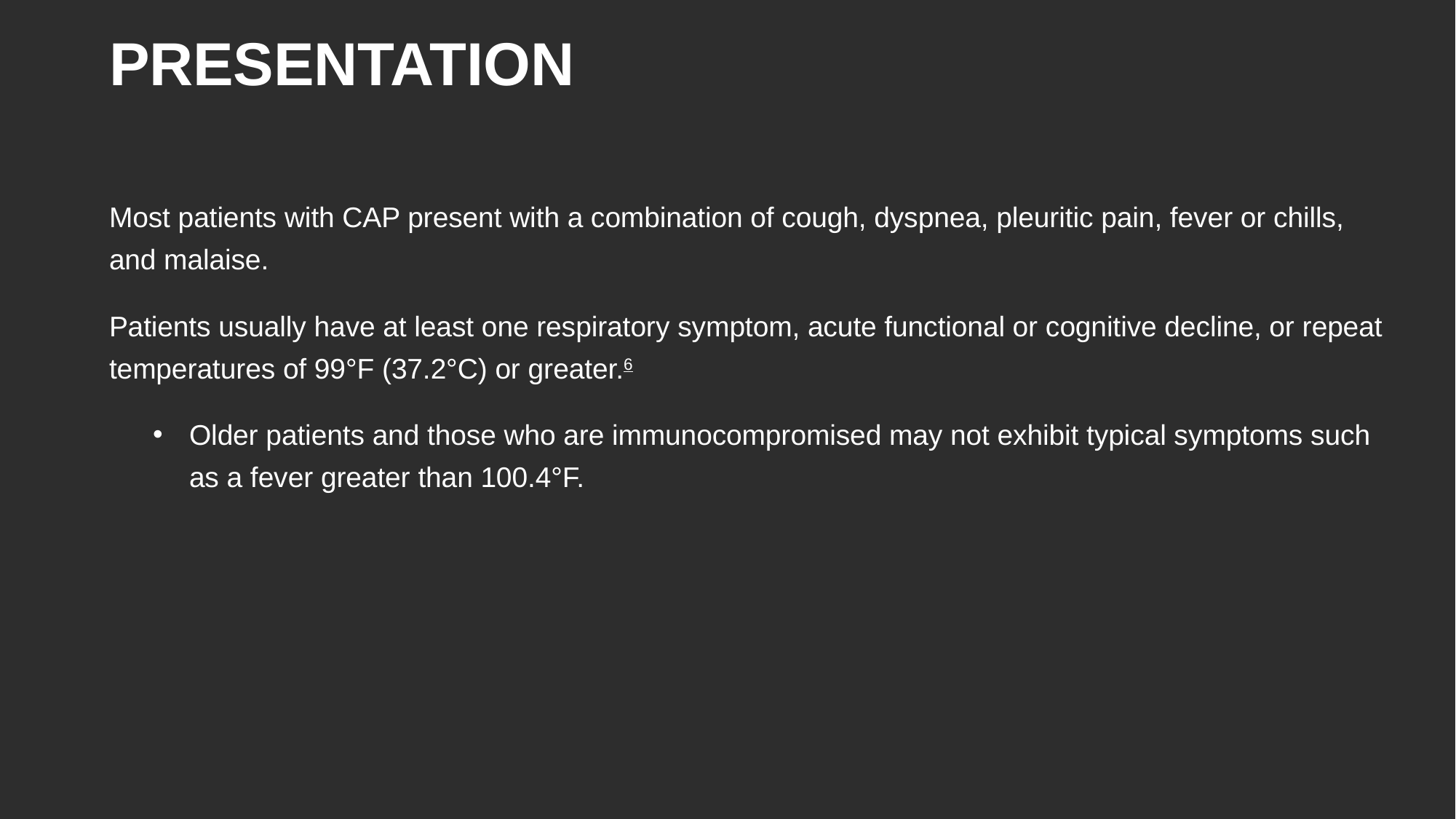

# Presentation
Most patients with CAP present with a combination of cough, dyspnea, pleuritic pain, fever or chills, and malaise.
Patients usually have at least one respiratory symptom, acute functional or cognitive decline, or repeat temperatures of 99°F (37.2°C) or greater.6
Older patients and those who are immunocompromised may not exhibit typical symptoms such as a fever greater than 100.4°F.

## Slide 4
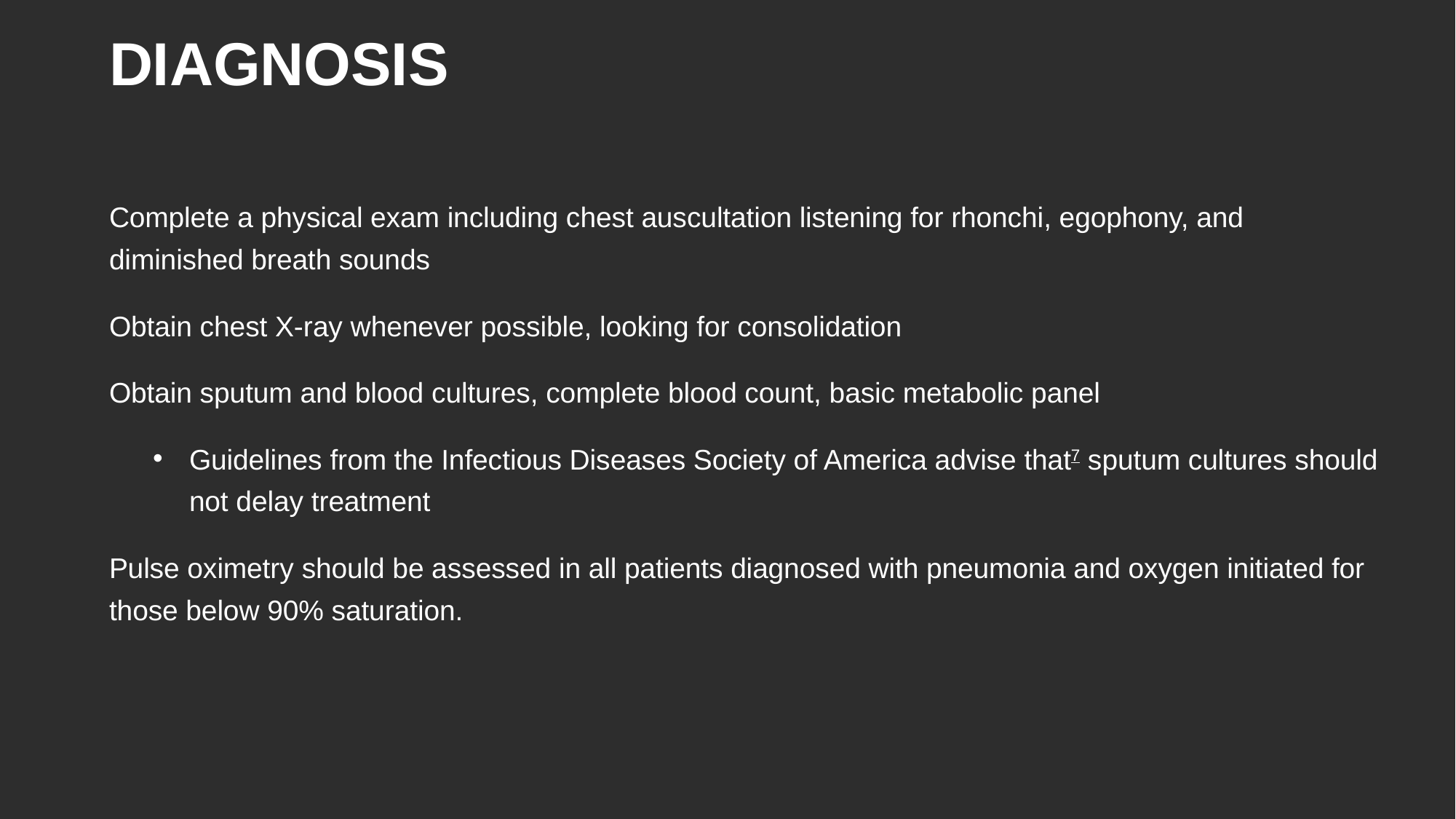

# Diagnosis
Complete a physical exam including chest auscultation listening for rhonchi, egophony, and diminished breath sounds
Obtain chest X-ray whenever possible, looking for consolidation
Obtain sputum and blood cultures, complete blood count, basic metabolic panel
Guidelines from the Infectious Diseases Society of America advise that7 sputum cultures should not delay treatment
Pulse oximetry should be assessed in all patients diagnosed with pneumonia and oxygen initiated for those below 90% saturation.

## Slide 5
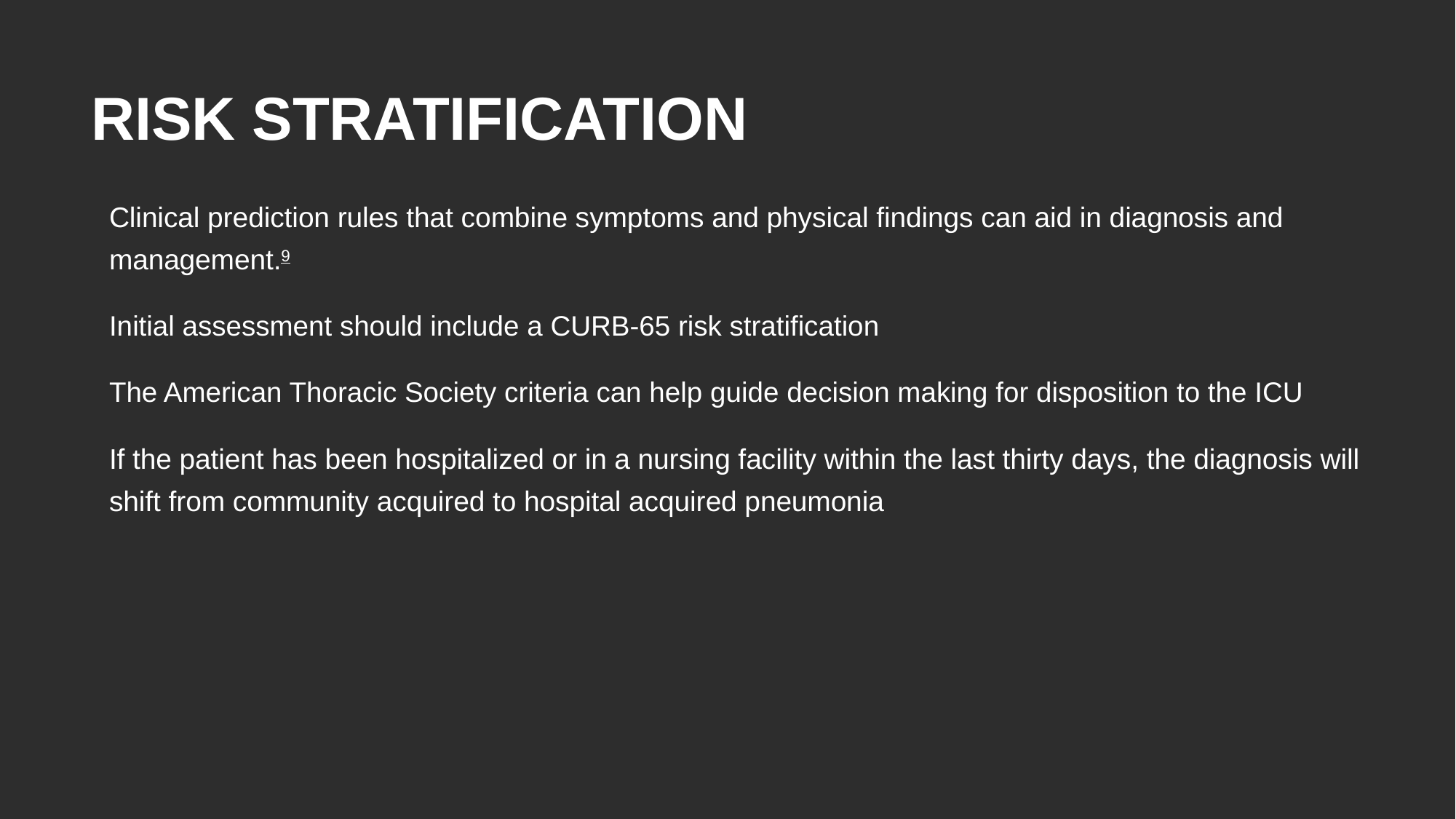

# Risk Stratification
Clinical prediction rules that combine symptoms and physical findings can aid in diagnosis and management.9
Initial assessment should include a CURB-65 risk stratification
The American Thoracic Society criteria can help guide decision making for disposition to the ICU
If the patient has been hospitalized or in a nursing facility within the last thirty days, the diagnosis will shift from community acquired to hospital acquired pneumonia

## Slide 6
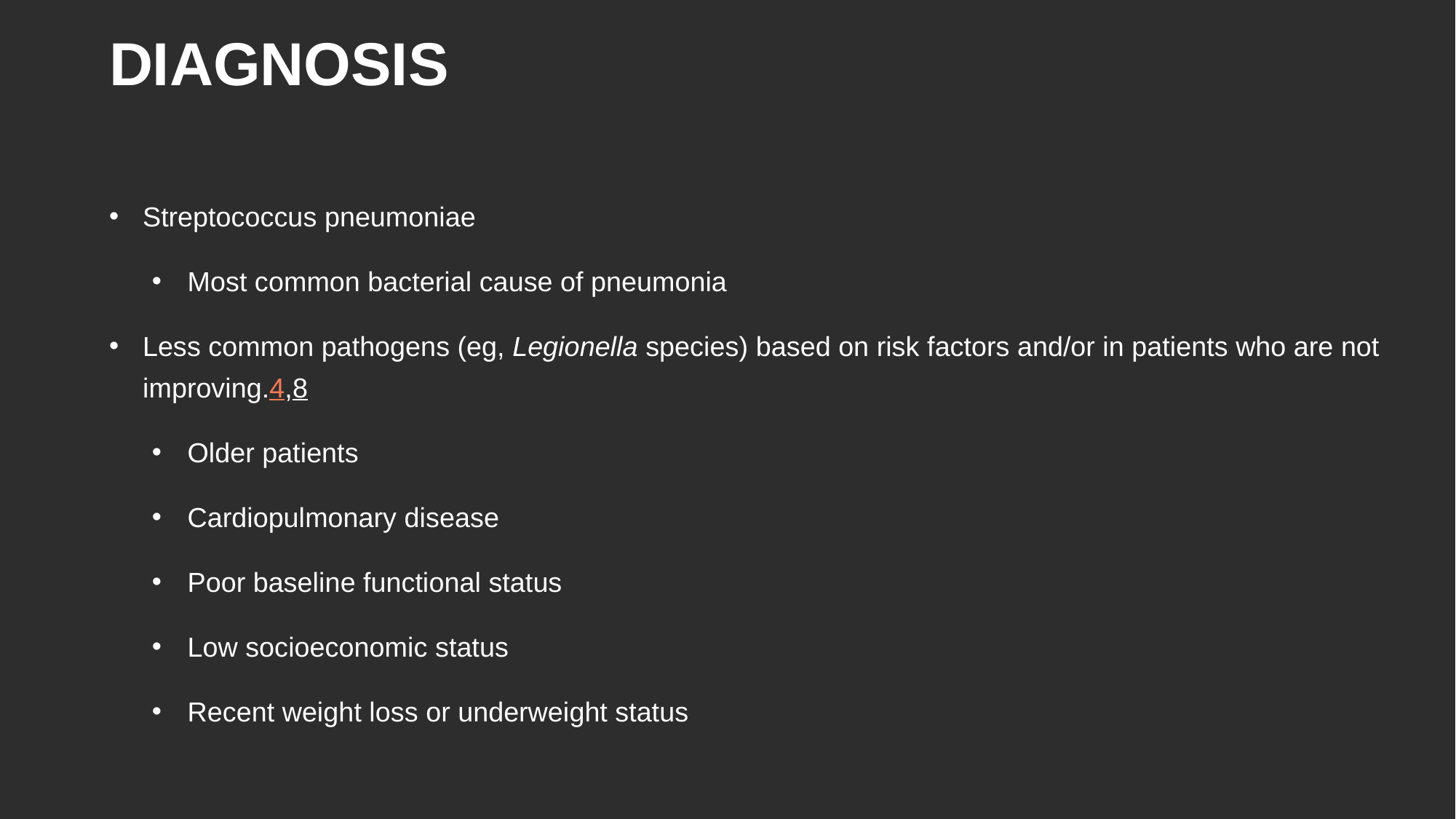

# Diagnosis
Streptococcus pneumoniae
Most common bacterial cause of pneumonia
Less common pathogens (eg, Legionella species) based on risk factors and/or in patients who are not improving.4,8
Older patients
Cardiopulmonary disease
Poor baseline functional status
Low socioeconomic status
Recent weight loss or underweight status

## Slide 7
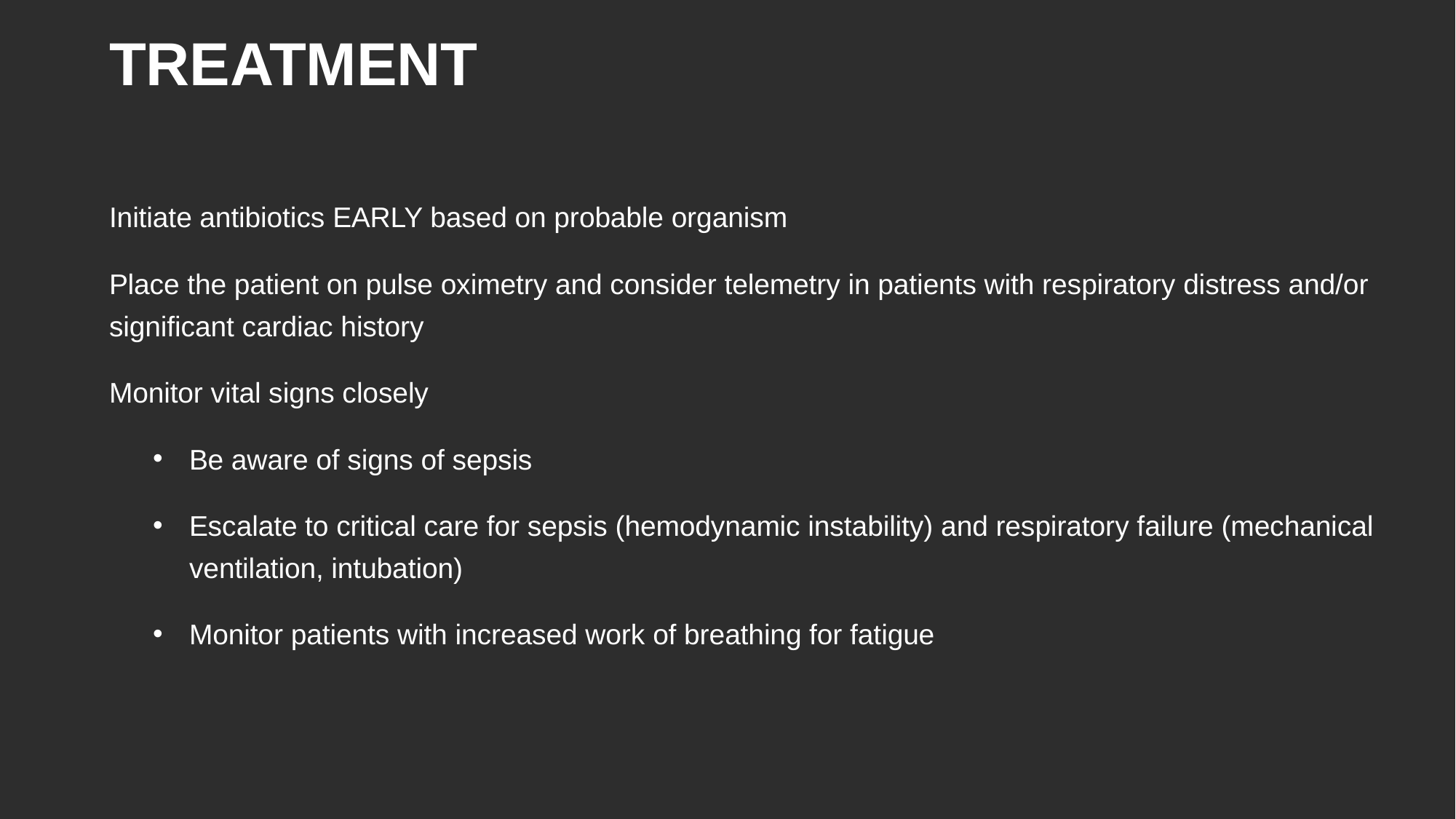

# Treatment
Initiate antibiotics EARLY based on probable organism
Place the patient on pulse oximetry and consider telemetry in patients with respiratory distress and/or significant cardiac history
Monitor vital signs closely
Be aware of signs of sepsis
Escalate to critical care for sepsis (hemodynamic instability) and respiratory failure (mechanical ventilation, intubation)
Monitor patients with increased work of breathing for fatigue

## Slide 8
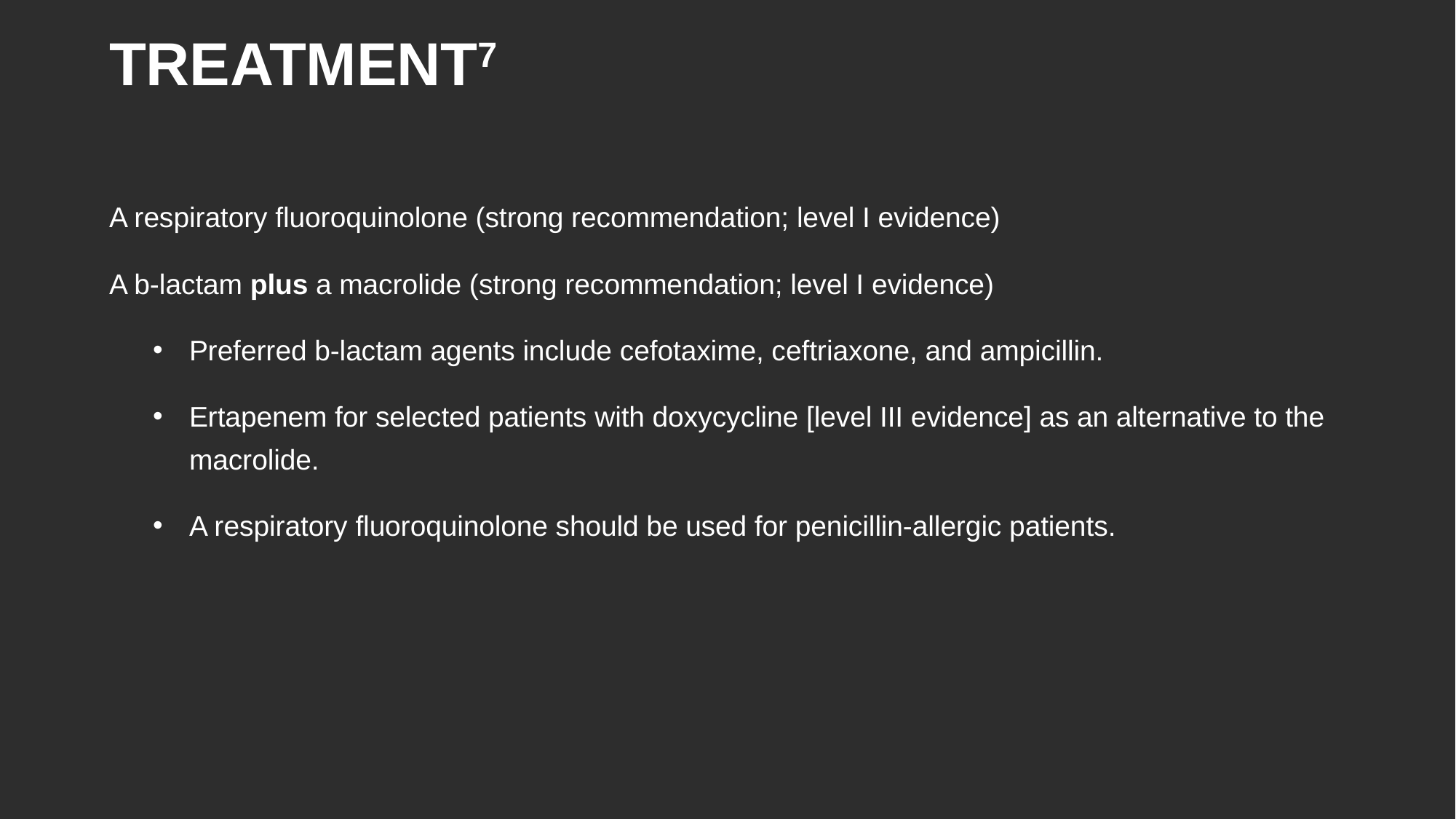

# Treatment7
A respiratory fluoroquinolone (strong recommendation; level I evidence)
A b-lactam plus a macrolide (strong recommendation; level I evidence)
Preferred b-lactam agents include cefotaxime, ceftriaxone, and ampicillin.
Ertapenem for selected patients with doxycycline [level III evidence] as an alternative to the macrolide.
A respiratory fluoroquinolone should be used for penicillin-allergic patients.

## Slide 9
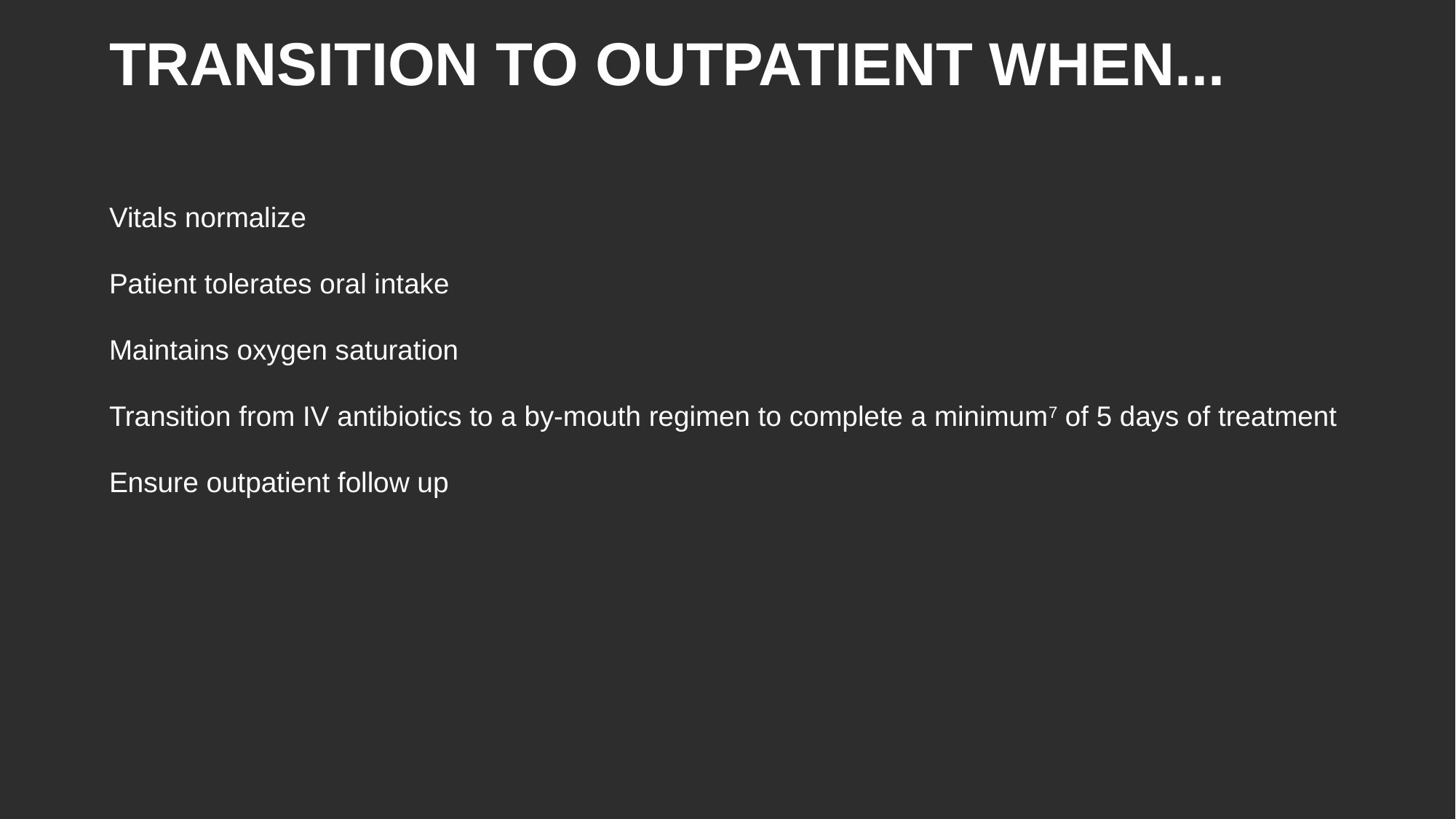

# Transition to outpatient When...
Vitals normalize
Patient tolerates oral intake
Maintains oxygen saturation
Transition from IV antibiotics to a by-mouth regimen to complete a minimum7 of 5 days of treatment
Ensure outpatient follow up

## Slide 10
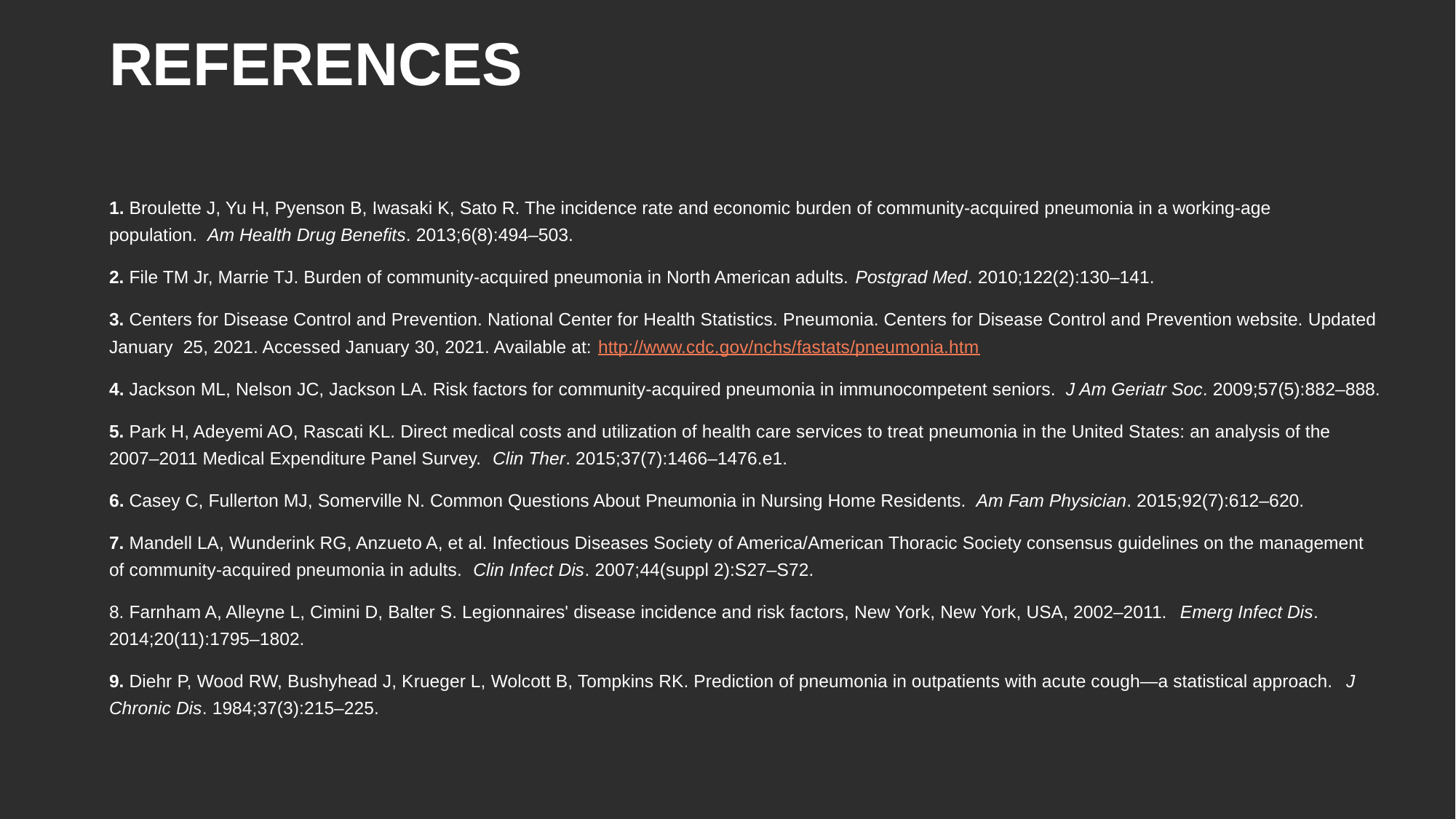

# References
1. Broulette J, Yu H, Pyenson B, Iwasaki K, Sato R. The incidence rate and economic burden of community-acquired pneumonia in a working-age population.  Am Health Drug Benefits. 2013;6(8):494–503.
2. File TM Jr, Marrie TJ. Burden of community-acquired pneumonia in North American adults. Postgrad Med. 2010;122(2):130–141.
3. Centers for Disease Control and Prevention. National Center for Health Statistics. Pneumonia. Centers for Disease Control and Prevention website. Updated January  25, 2021. Accessed January 30, 2021. Available at: http://www.cdc.gov/nchs/fastats/pneumonia.htm
4. Jackson ML, Nelson JC, Jackson LA. Risk factors for community-acquired pneumonia in immunocompetent seniors.  J Am Geriatr Soc. 2009;57(5):882–888.
5. Park H, Adeyemi AO, Rascati KL. Direct medical costs and utilization of health care services to treat pneumonia in the United States: an analysis of the 2007–2011 Medical Expenditure Panel Survey.  Clin Ther. 2015;37(7):1466–1476.e1.
6. Casey C, Fullerton MJ, Somerville N. Common Questions About Pneumonia in Nursing Home Residents.  Am Fam Physician. 2015;92(7):612–620.
7. Mandell LA, Wunderink RG, Anzueto A, et al. Infectious Diseases Society of America/American Thoracic Society consensus guidelines on the management of community-acquired pneumonia in adults.  Clin Infect Dis. 2007;44(suppl 2):S27–S72.
8. Farnham A, Alleyne L, Cimini D, Balter S. Legionnaires' disease incidence and risk factors, New York, New York, USA, 2002–2011.  Emerg Infect Dis. 2014;20(11):1795–1802.
9. Diehr P, Wood RW, Bushyhead J, Krueger L, Wolcott B, Tompkins RK. Prediction of pneumonia in outpatients with acute cough—a statistical approach.  J Chronic Dis. 1984;37(3):215–225.
