# Supplementary material for: An Observation Medicine Curriculum for Emergency Medicine Education
Source: J Educ Teach Emerg Med. 2021 Apr 19;6(2):C1–C72. doi: 10.21980/J87P92 (PMC10332786; doi:10.21980/J87P92)
Supplement: Supplementary file 17 — Please see associated PowerPoint file [file jetem-6-2-c1-supp17.pptx]

## Slide 1
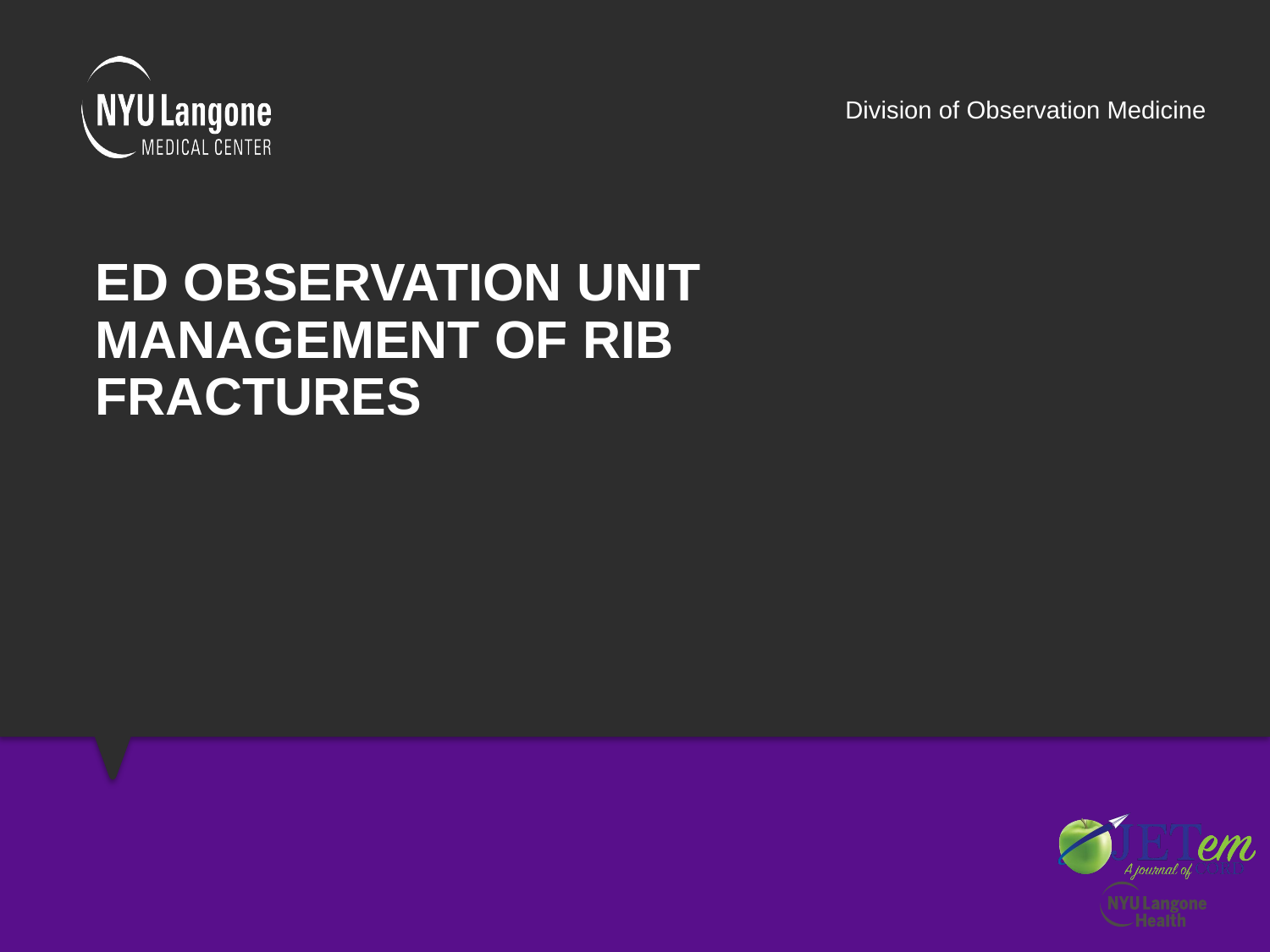

Division of Observation Medicine
# ED Observation Unit Management of Rib fractures

## Slide 2
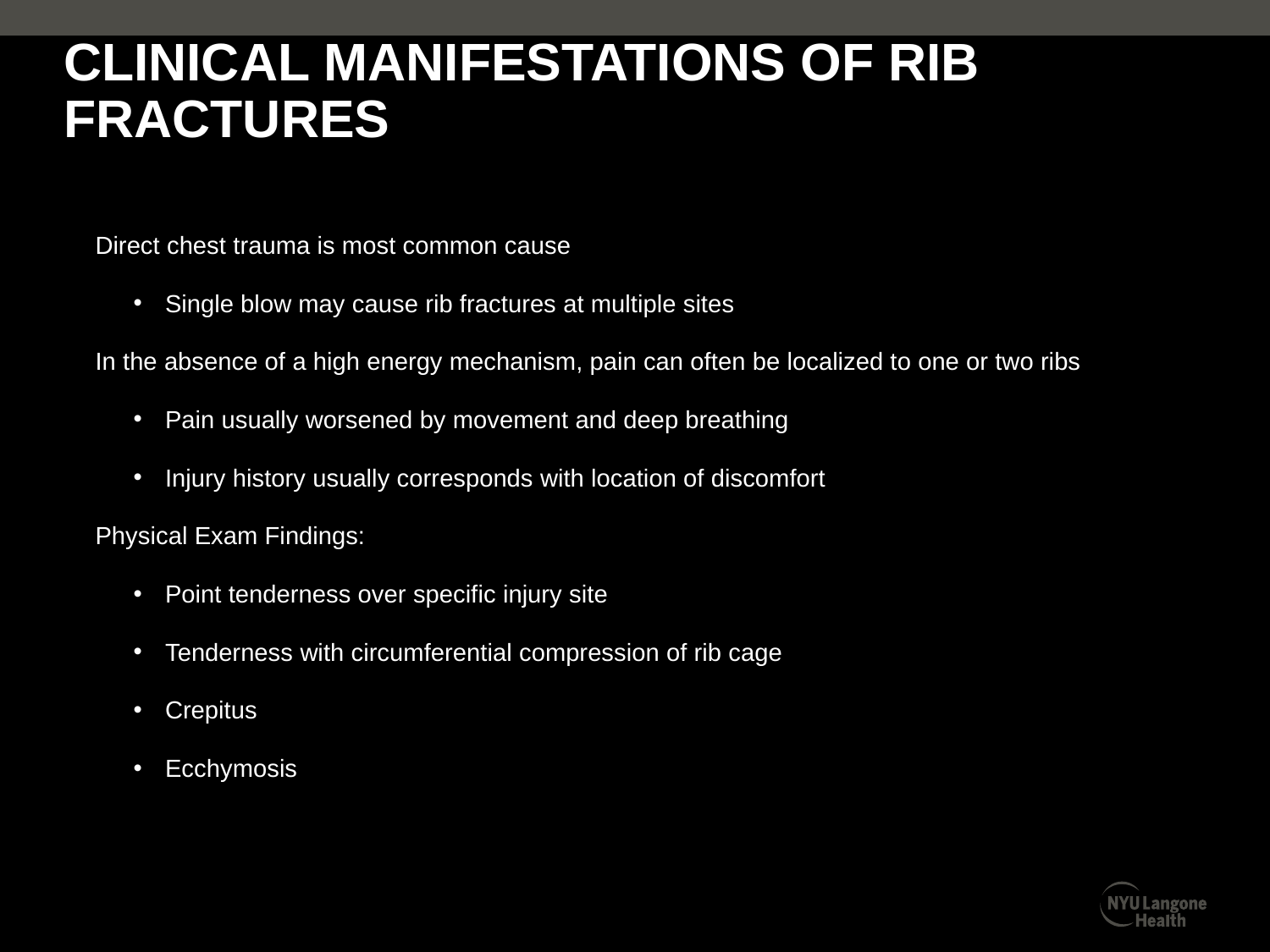

# Clinical Manifestations of Rib Fractures
Direct chest trauma is most common cause
Single blow may cause rib fractures at multiple sites
In the absence of a high energy mechanism, pain can often be localized to one or two ribs
Pain usually worsened by movement and deep breathing
Injury history usually corresponds with location of discomfort
Physical Exam Findings:
Point tenderness over specific injury site
Tenderness with circumferential compression of rib cage
Crepitus
Ecchymosis
2
NYU Perelman Department of Emergency Medicine

## Slide 3
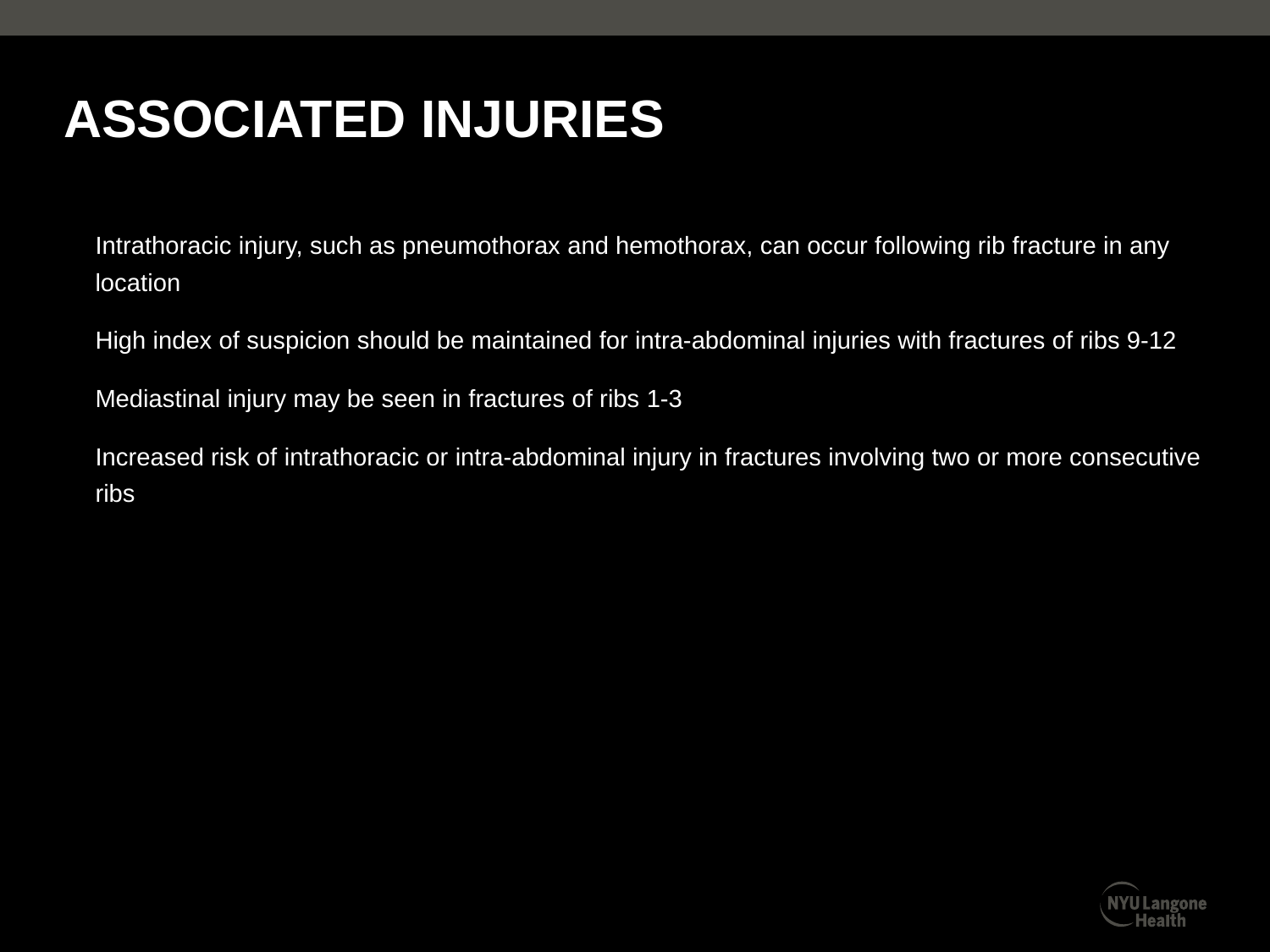

# Associated Injuries
Intrathoracic injury, such as pneumothorax and hemothorax, can occur following rib fracture in any location
High index of suspicion should be maintained for intra-abdominal injuries with fractures of ribs 9-12
Mediastinal injury may be seen in fractures of ribs 1-3
Increased risk of intrathoracic or intra-abdominal injury in fractures involving two or more consecutive ribs
3
NYU Perelman Department of Emergency Medicine

## Slide 4
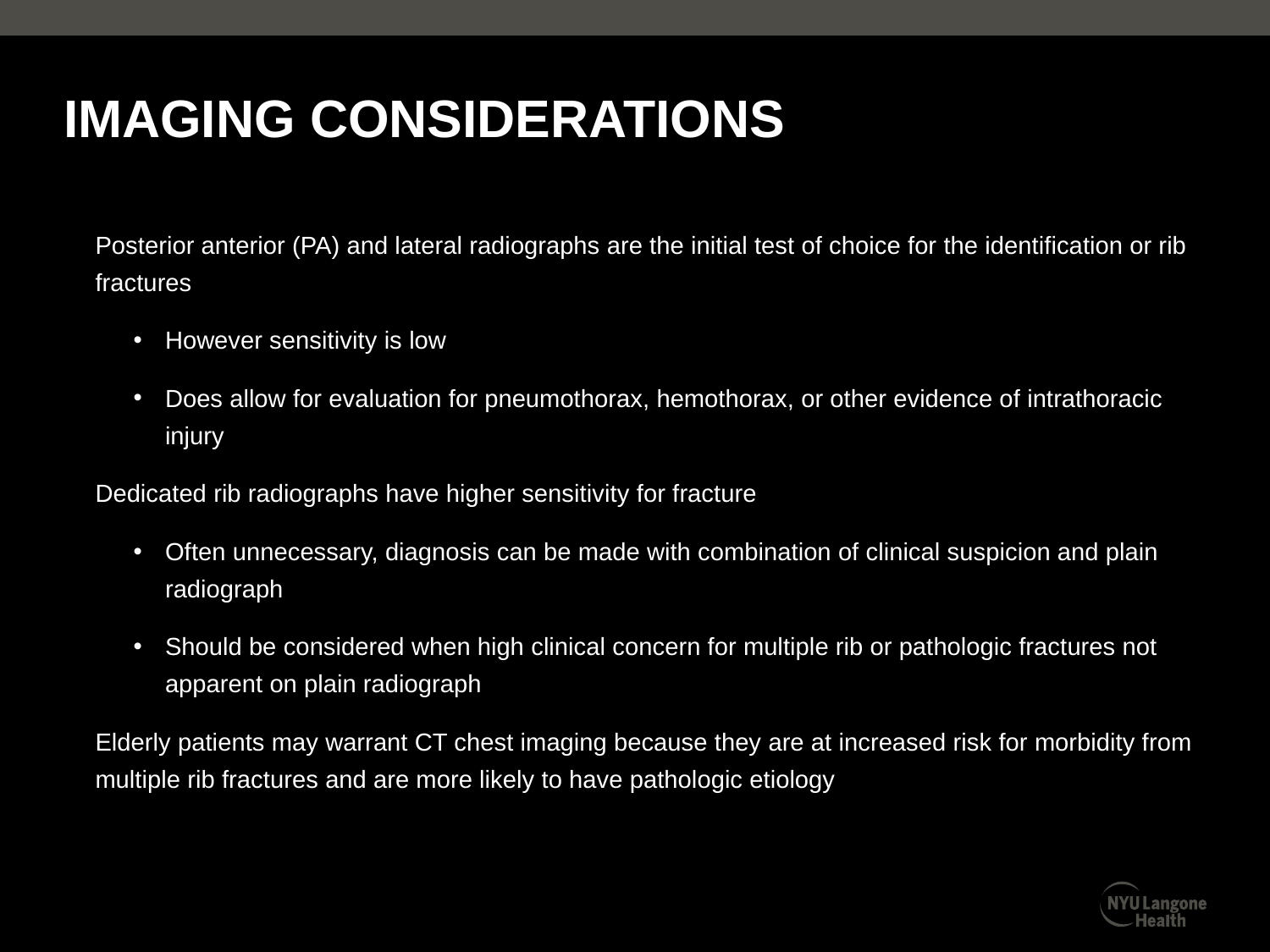

# Imaging Considerations
Posterior anterior (PA) and lateral radiographs are the initial test of choice for the identification or rib fractures
However sensitivity is low
Does allow for evaluation for pneumothorax, hemothorax, or other evidence of intrathoracic injury
Dedicated rib radiographs have higher sensitivity for fracture
Often unnecessary, diagnosis can be made with combination of clinical suspicion and plain radiograph
Should be considered when high clinical concern for multiple rib or pathologic fractures not apparent on plain radiograph
Elderly patients may warrant CT chest imaging because they are at increased risk for morbidity from multiple rib fractures and are more likely to have pathologic etiology
4
NYU Perelman Department of Emergency Medicine

## Slide 5
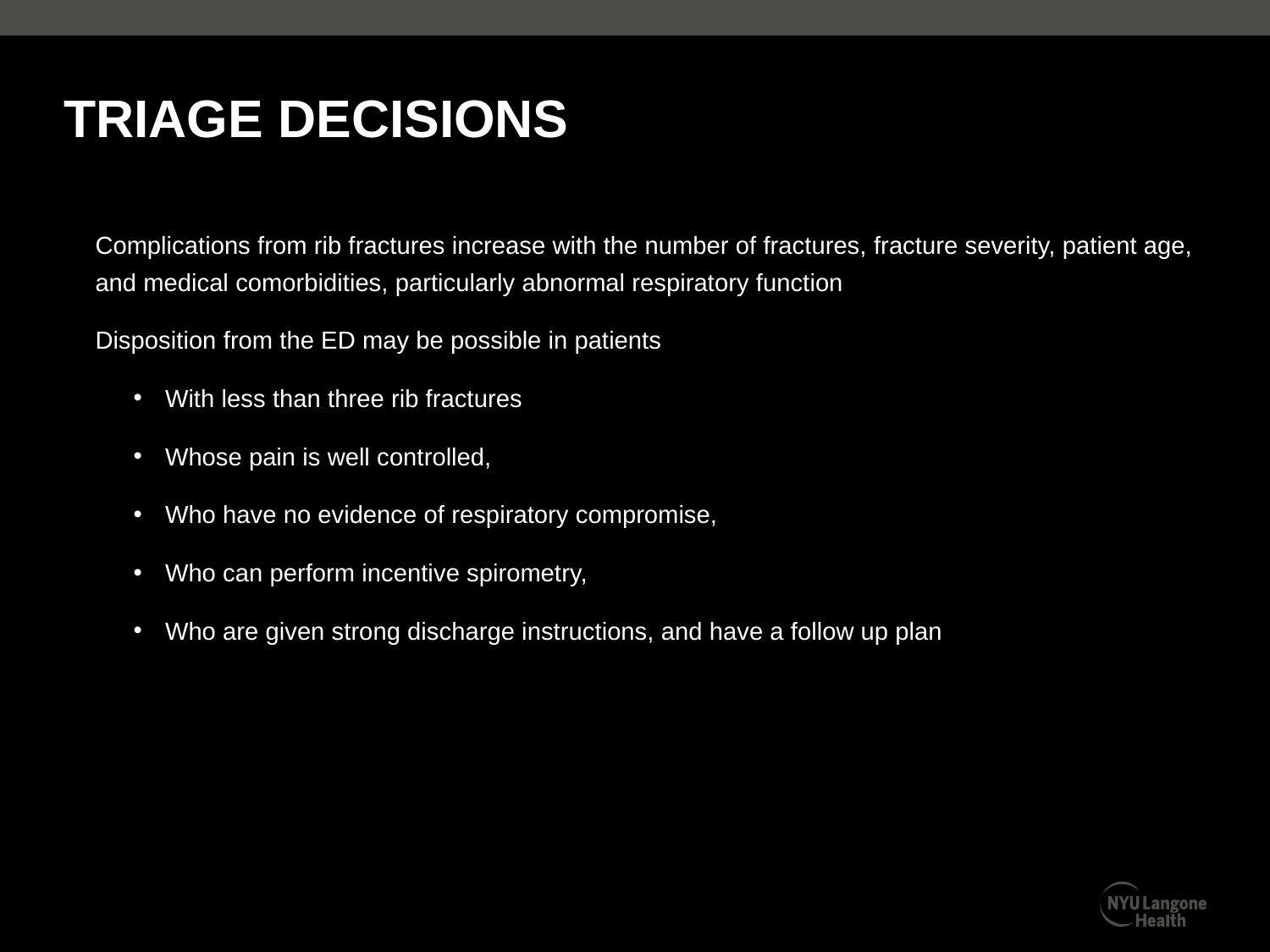

# Triage Decisions
Complications from rib fractures increase with the number of fractures, fracture severity, patient age, and medical comorbidities, particularly abnormal respiratory function
Disposition from the ED may be possible in patients
With less than three rib fractures
Whose pain is well controlled,
Who have no evidence of respiratory compromise,
Who can perform incentive spirometry,
Who are given strong discharge instructions, and have a follow up plan
5
NYU Perelman Department of Emergency Medicine

## Slide 6
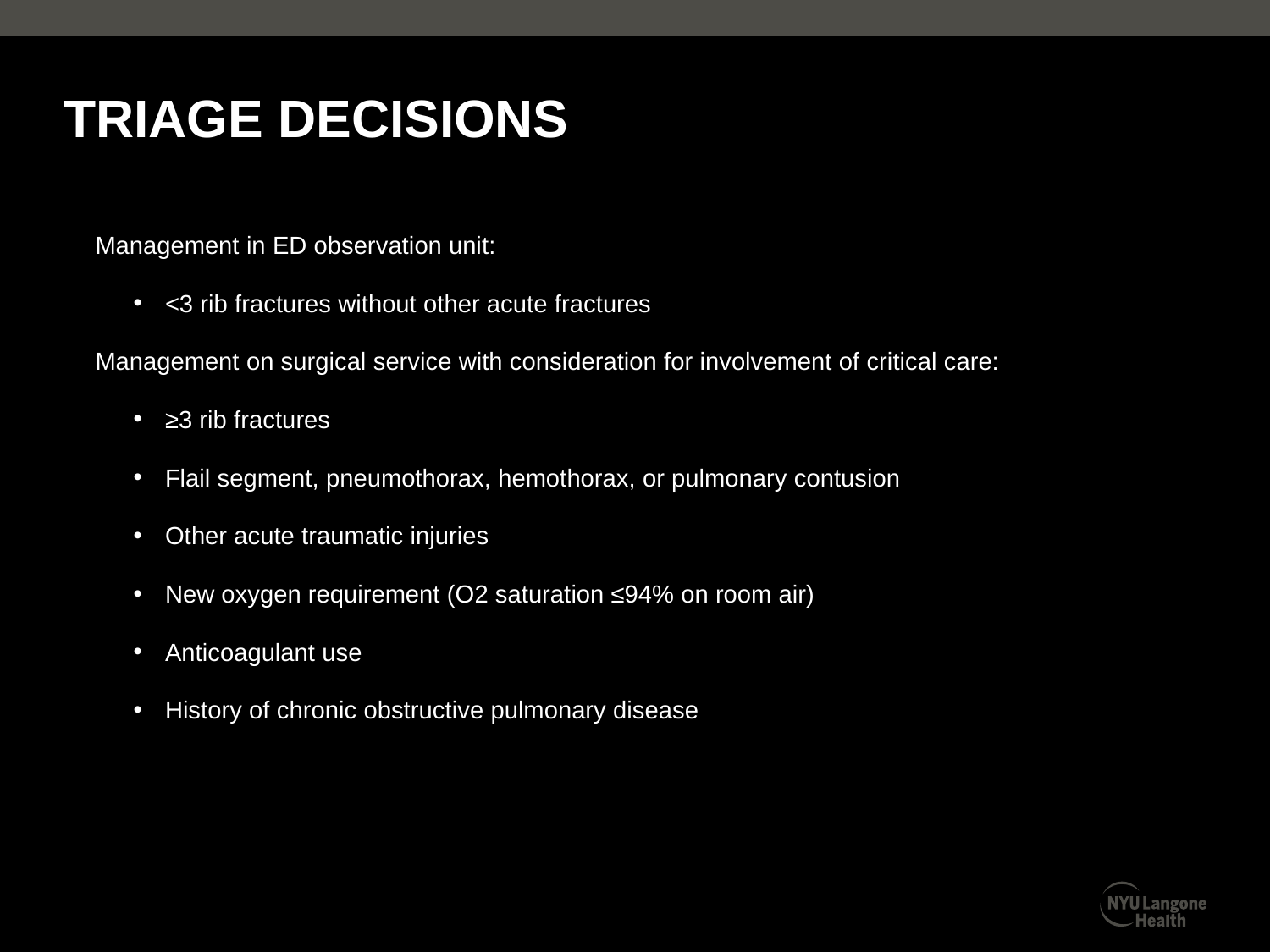

# Triage Decisions
Management in ED observation unit:
<3 rib fractures without other acute fractures
Management on surgical service with consideration for involvement of critical care:
≥3 rib fractures
Flail segment, pneumothorax, hemothorax, or pulmonary contusion
Other acute traumatic injuries
New oxygen requirement (O2 saturation ≤94% on room air)
Anticoagulant use
History of chronic obstructive pulmonary disease
6
NYU Perelman Department of Emergency Medicine

## Slide 7
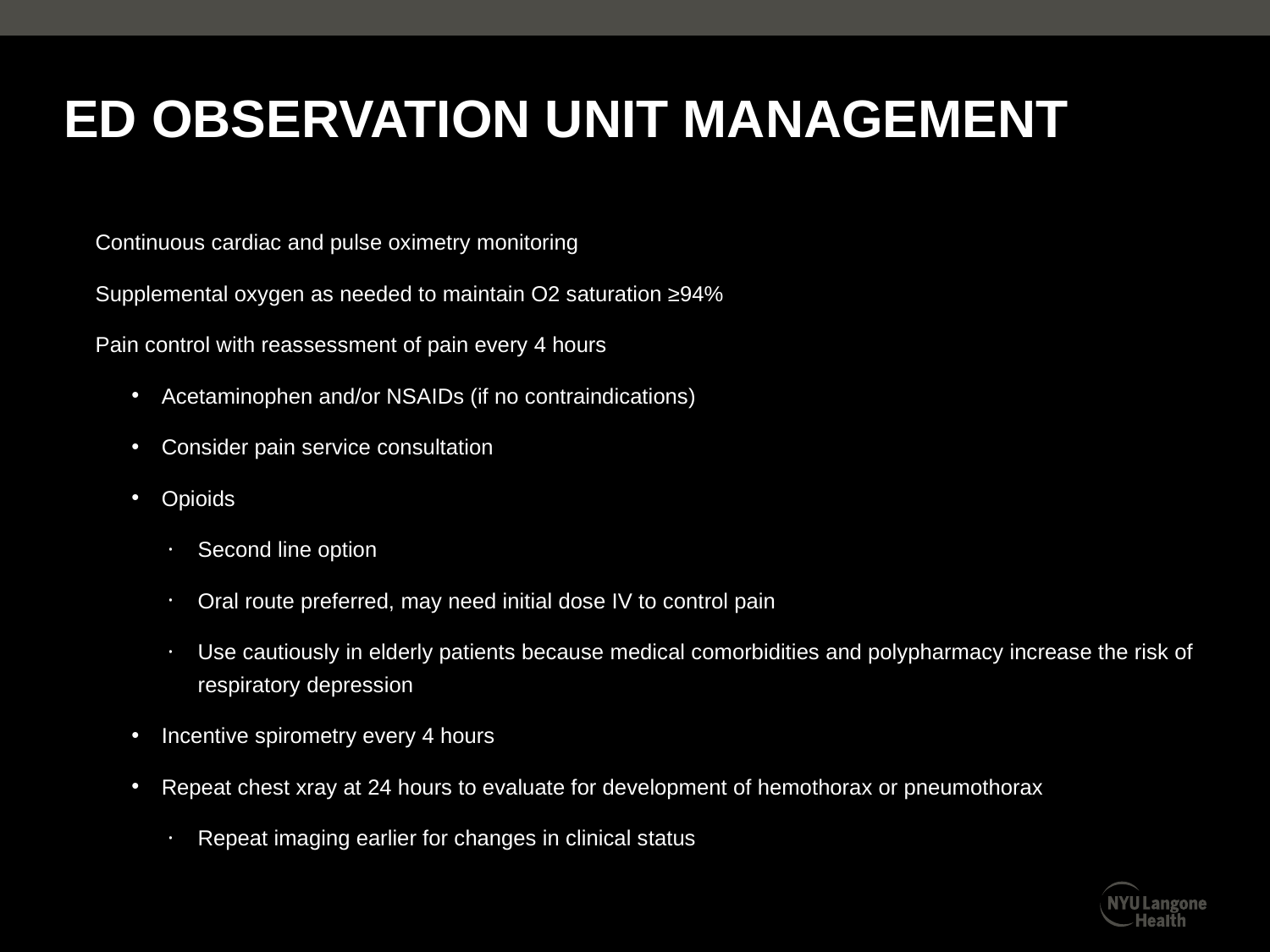

# ED Observation Unit Management
Continuous cardiac and pulse oximetry monitoring
Supplemental oxygen as needed to maintain O2 saturation ≥94%
Pain control with reassessment of pain every 4 hours
Acetaminophen and/or NSAIDs (if no contraindications)
Consider pain service consultation
Opioids
Second line option
Oral route preferred, may need initial dose IV to control pain
Use cautiously in elderly patients because medical comorbidities and polypharmacy increase the risk of respiratory depression
Incentive spirometry every 4 hours
Repeat chest xray at 24 hours to evaluate for development of hemothorax or pneumothorax
Repeat imaging earlier for changes in clinical status
7
NYU Perelman Department of Emergency Medicine

## Slide 8
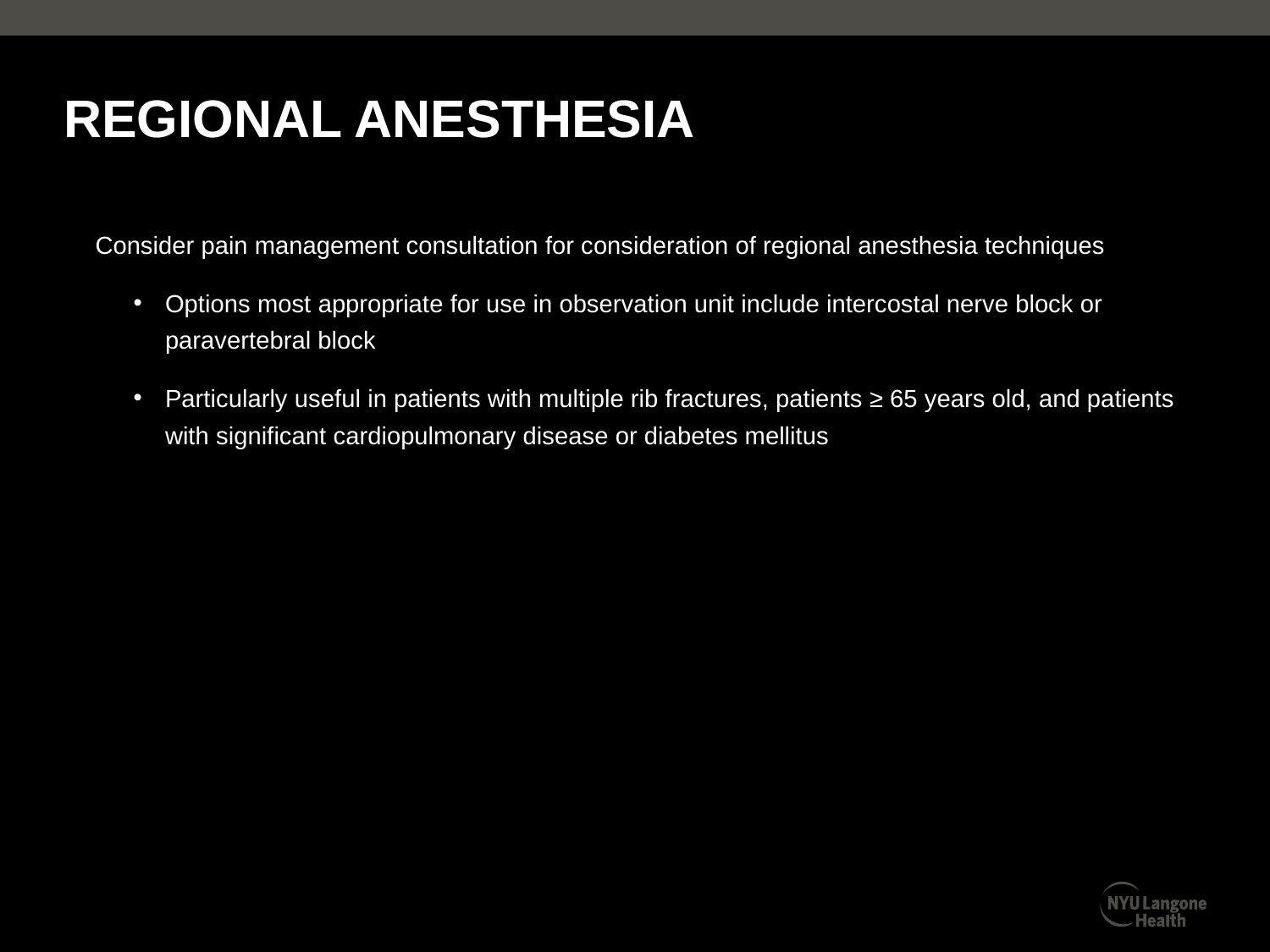

# Regional Anesthesia
Consider pain management consultation for consideration of regional anesthesia techniques
Options most appropriate for use in observation unit include intercostal nerve block or paravertebral block
Particularly useful in patients with multiple rib fractures, patients ≥ 65 years old, and patients with significant cardiopulmonary disease or diabetes mellitus
8
NYU Perelman Department of Emergency Medicine

## Slide 9
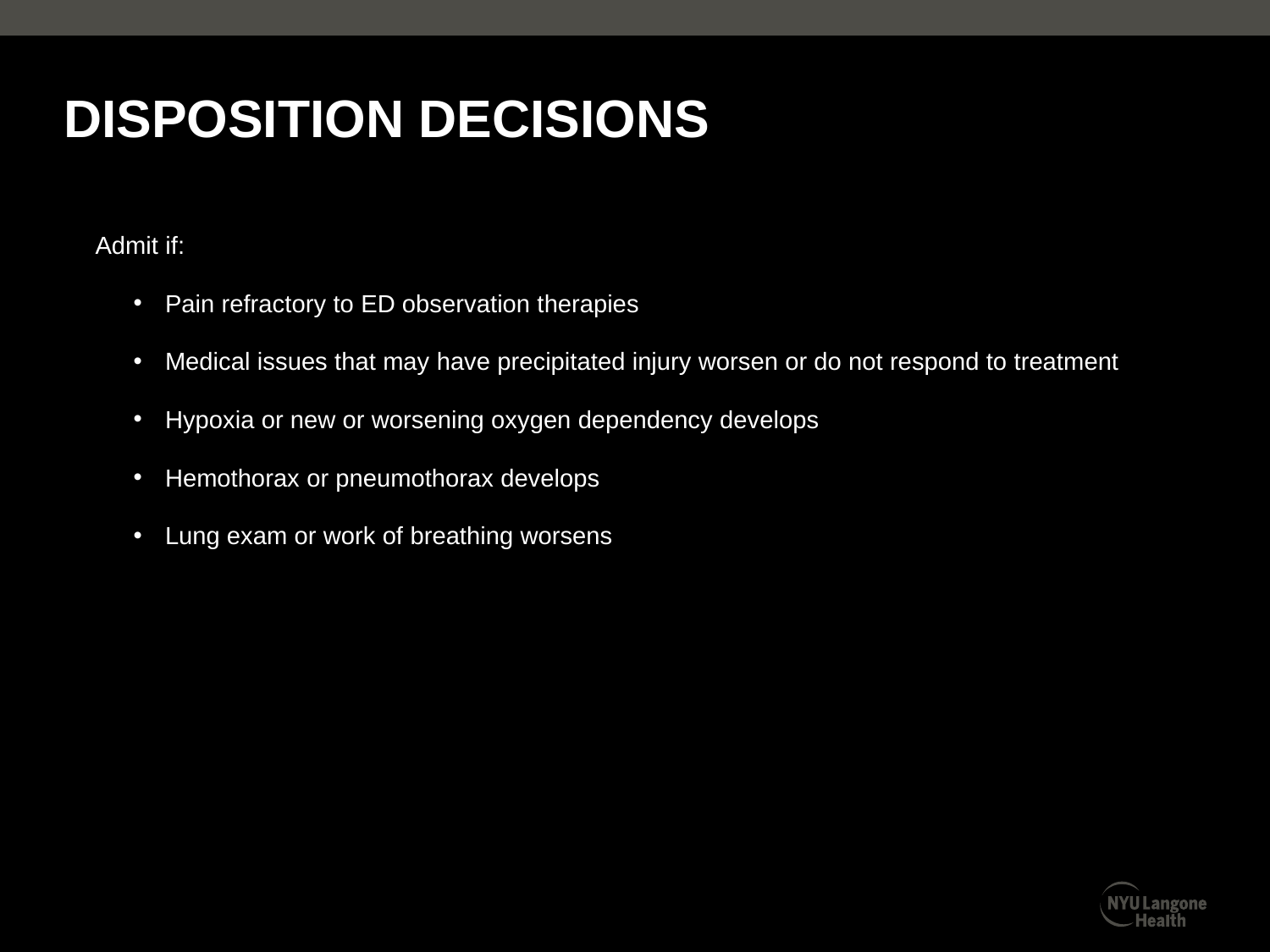

# Disposition Decisions
Admit if:
Pain refractory to ED observation therapies
Medical issues that may have precipitated injury worsen or do not respond to treatment
Hypoxia or new or worsening oxygen dependency develops
Hemothorax or pneumothorax develops
Lung exam or work of breathing worsens
9
NYU Perelman Department of Emergency Medicine

## Slide 10
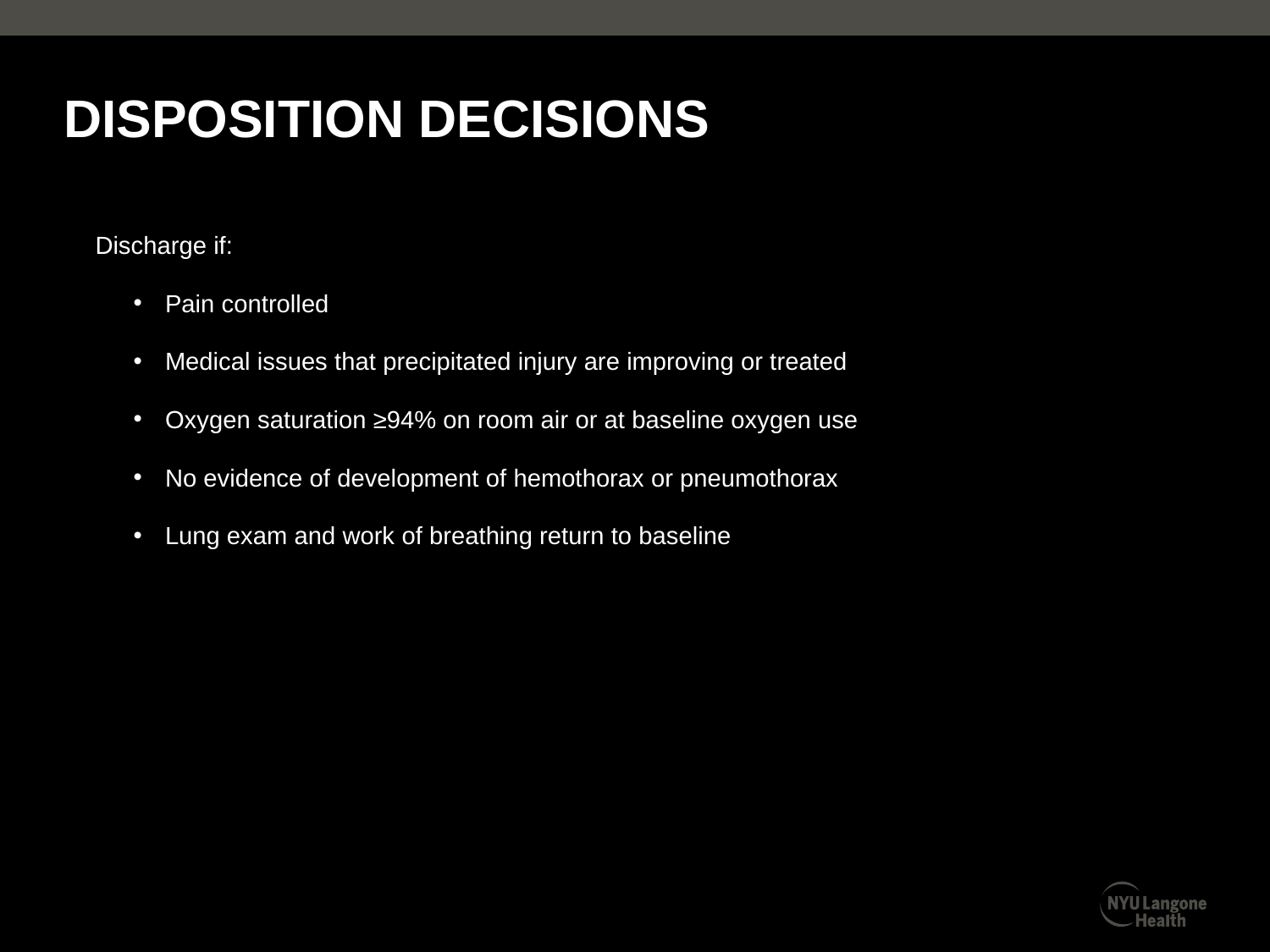

# Disposition Decisions
Discharge if:
Pain controlled
Medical issues that precipitated injury are improving or treated
Oxygen saturation ≥94% on room air or at baseline oxygen use
No evidence of development of hemothorax or pneumothorax
Lung exam and work of breathing return to baseline
10
NYU Perelman Department of Emergency Medicine

## Slide 11
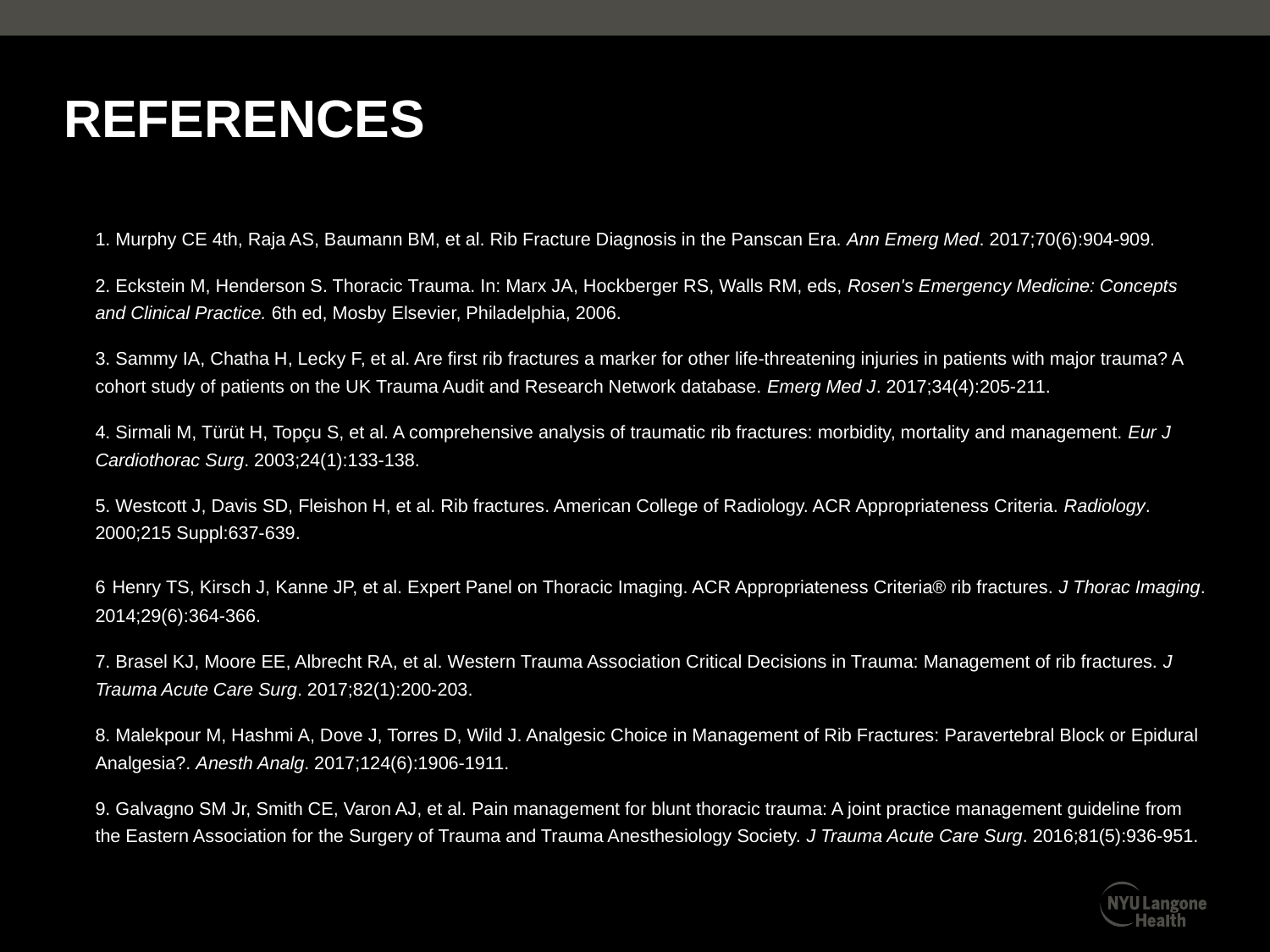

# References
1. Murphy CE 4th, Raja AS, Baumann BM, et al. Rib Fracture Diagnosis in the Panscan Era. Ann Emerg Med. 2017;70(6):904-909.
2. Eckstein M, Henderson S. Thoracic Trauma. In: Marx JA, Hockberger RS, Walls RM, eds, Rosen's Emergency Medicine: Concepts and Clinical Practice. 6th ed, Mosby Elsevier, Philadelphia, 2006.
3. Sammy IA, Chatha H, Lecky F, et al. Are first rib fractures a marker for other life-threatening injuries in patients with major trauma? A cohort study of patients on the UK Trauma Audit and Research Network database. Emerg Med J. 2017;34(4):205-211.
4. Sirmali M, Türüt H, Topçu S, et al. A comprehensive analysis of traumatic rib fractures: morbidity, mortality and management. Eur J Cardiothorac Surg. 2003;24(1):133-138.
5. Westcott J, Davis SD, Fleishon H, et al. Rib fractures. American College of Radiology. ACR Appropriateness Criteria. Radiology. 2000;215 Suppl:637-639.
6 Henry TS, Kirsch J, Kanne JP, et al. Expert Panel on Thoracic Imaging. ACR Appropriateness Criteria® rib fractures. J Thorac Imaging. 2014;29(6):364-366.
7. Brasel KJ, Moore EE, Albrecht RA, et al. Western Trauma Association Critical Decisions in Trauma: Management of rib fractures. J Trauma Acute Care Surg. 2017;82(1):200-203.
8. Malekpour M, Hashmi A, Dove J, Torres D, Wild J. Analgesic Choice in Management of Rib Fractures: Paravertebral Block or Epidural Analgesia?. Anesth Analg. 2017;124(6):1906-1911.
9. Galvagno SM Jr, Smith CE, Varon AJ, et al. Pain management for blunt thoracic trauma: A joint practice management guideline from the Eastern Association for the Surgery of Trauma and Trauma Anesthesiology Society. J Trauma Acute Care Surg. 2016;81(5):936-951.
11
NYU Perelman Department of Emergency Medicine
