# Supplementary material for: An Observation Medicine Curriculum for Emergency Medicine Education
Source: J Educ Teach Emerg Med. 2021 Apr 19;6(2):C1–C72. doi: 10.21980/J87P92 (PMC10332786; doi:10.21980/J87P92)
Supplement: Supplementary file 18 — Please see associated PowerPoint file [file jetem-6-2-c1-supp18.pptx]

## Slide 1
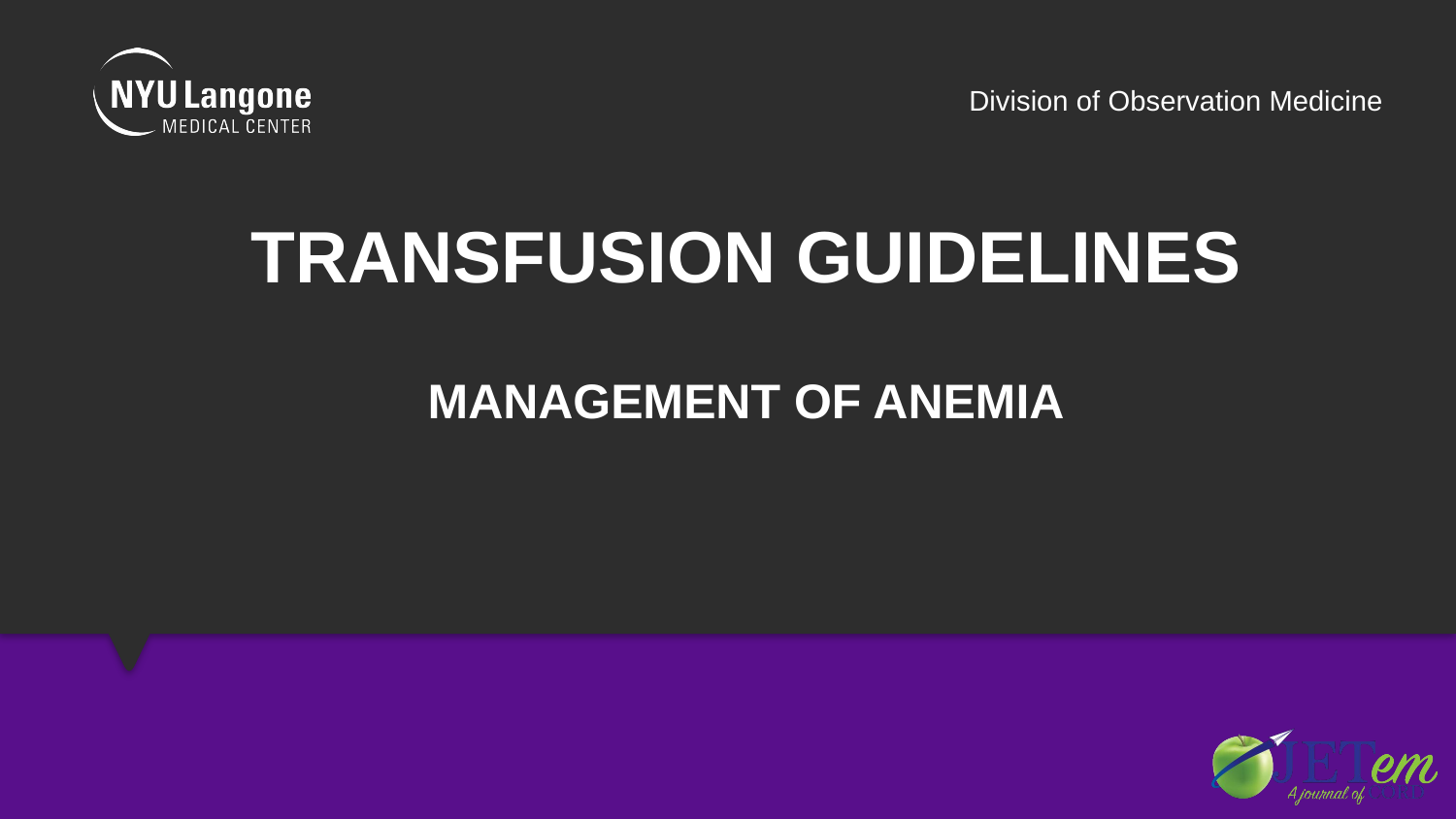

Division of Observation Medicine
# TRANSFUSION GUIDELINES MANAGEMENT OF ANEMIA

## Slide 2
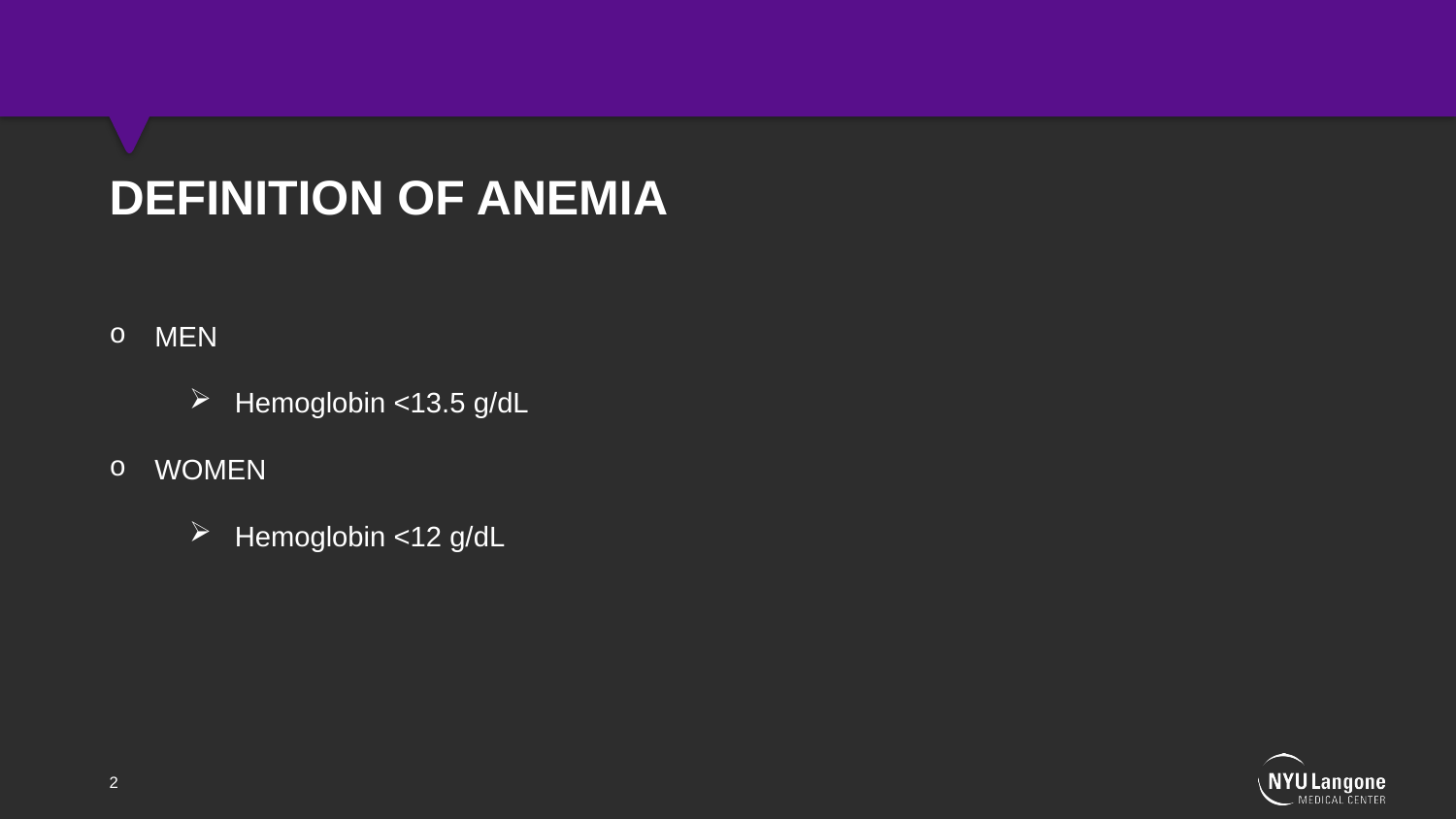

# DEFINITION OF ANEMIA
MEN
Hemoglobin <13.5 g/dL
WOMEN
Hemoglobin <12 g/dL
2

## Slide 3
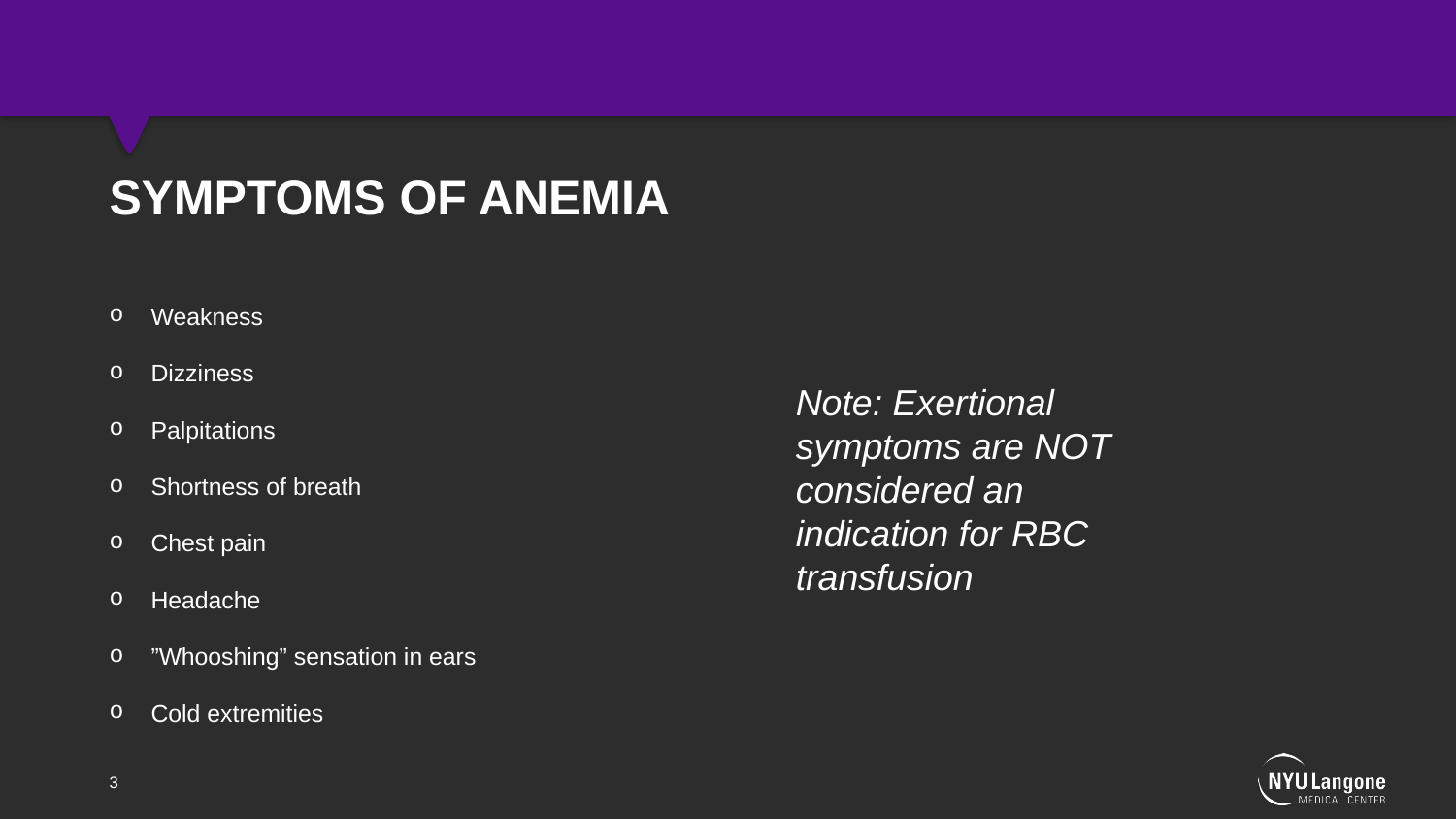

# SYMPTOMS OF ANEMIA
Weakness
Dizziness
Palpitations
Shortness of breath
Chest pain
Headache
”Whooshing” sensation in ears
Cold extremities
Note: Exertional symptoms are NOT considered an indication for RBC transfusion
3

## Slide 4
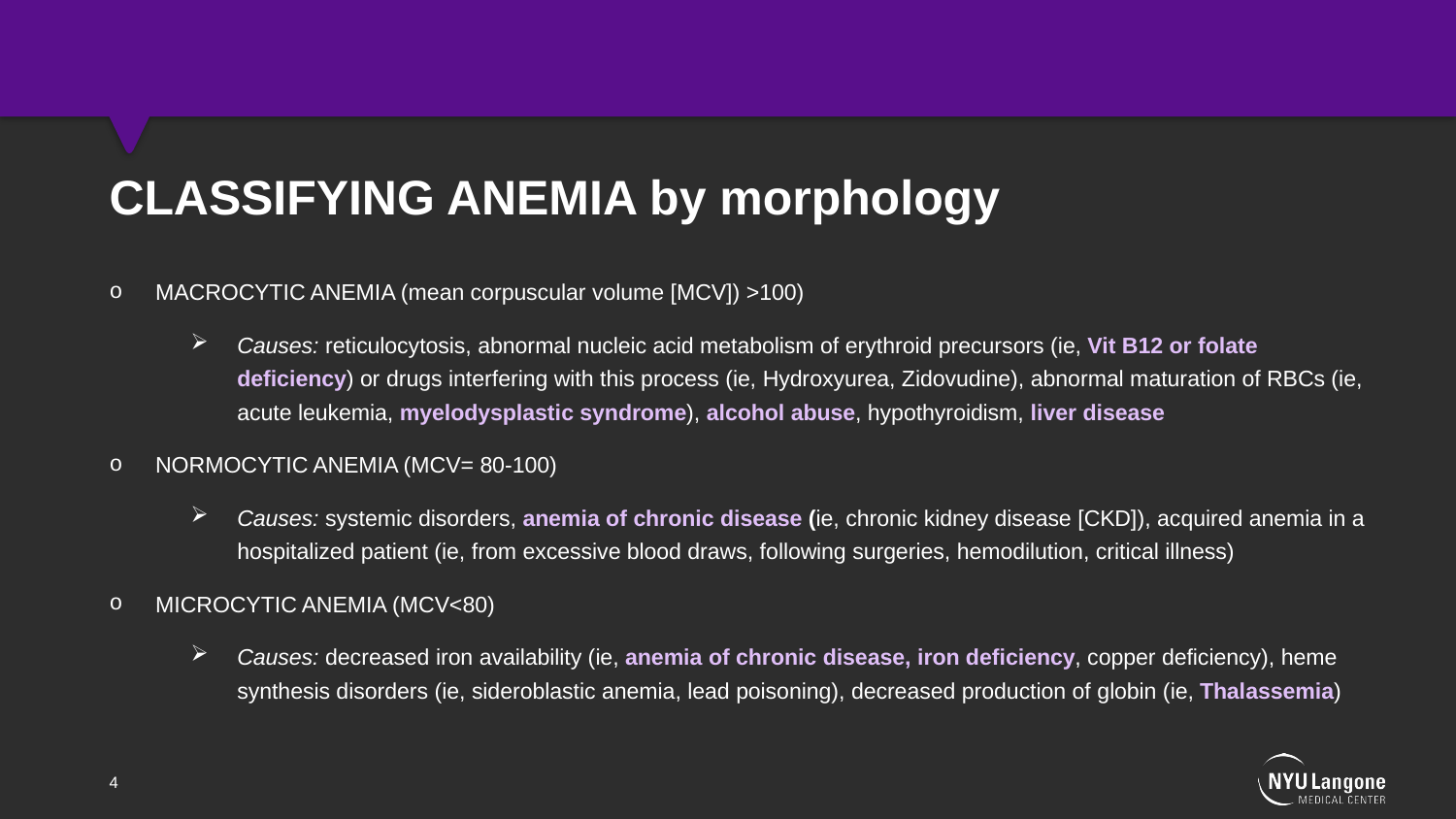

# CLASSIFYING ANEMIA by morphology
MACROCYTIC ANEMIA (mean corpuscular volume [MCV]) >100)
Causes: reticulocytosis, abnormal nucleic acid metabolism of erythroid precursors (ie, Vit B12 or folate deficiency) or drugs interfering with this process (ie, Hydroxyurea, Zidovudine), abnormal maturation of RBCs (ie, acute leukemia, myelodysplastic syndrome), alcohol abuse, hypothyroidism, liver disease
NORMOCYTIC ANEMIA (MCV= 80-100)
Causes: systemic disorders, anemia of chronic disease (ie, chronic kidney disease [CKD]), acquired anemia in a hospitalized patient (ie, from excessive blood draws, following surgeries, hemodilution, critical illness)
MICROCYTIC ANEMIA (MCV<80)
Causes: decreased iron availability (ie, anemia of chronic disease, iron deficiency, copper deficiency), heme synthesis disorders (ie, sideroblastic anemia, lead poisoning), decreased production of globin (ie, Thalassemia)
4

## Slide 5
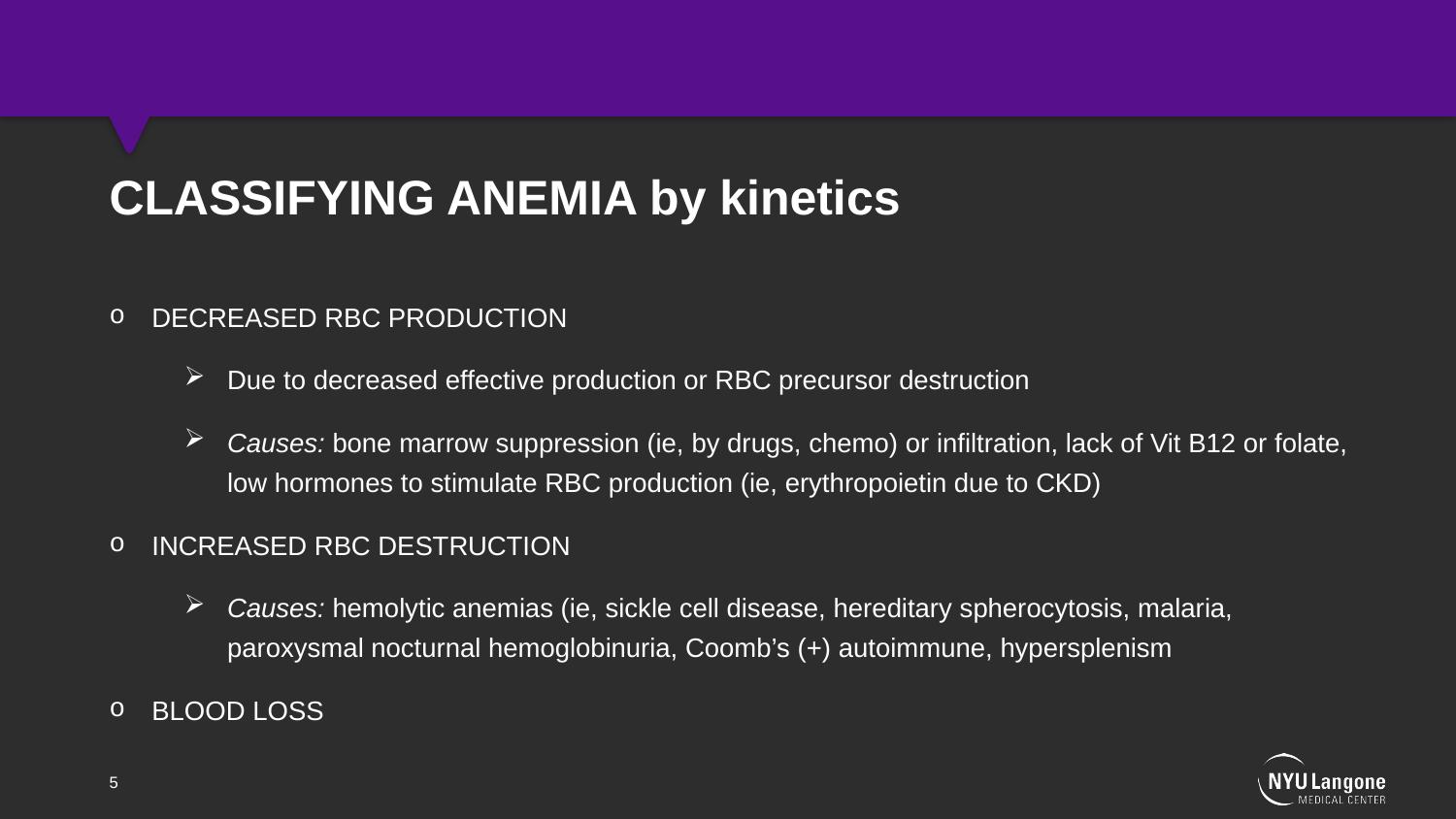

# CLASSIFYING ANEMIA by kinetics
DECREASED RBC PRODUCTION
Due to decreased effective production or RBC precursor destruction
Causes: bone marrow suppression (ie, by drugs, chemo) or infiltration, lack of Vit B12 or folate, low hormones to stimulate RBC production (ie, erythropoietin due to CKD)
INCREASED RBC DESTRUCTION
Causes: hemolytic anemias (ie, sickle cell disease, hereditary spherocytosis, malaria, paroxysmal nocturnal hemoglobinuria, Coomb’s (+) autoimmune, hypersplenism
BLOOD LOSS
5

## Slide 6
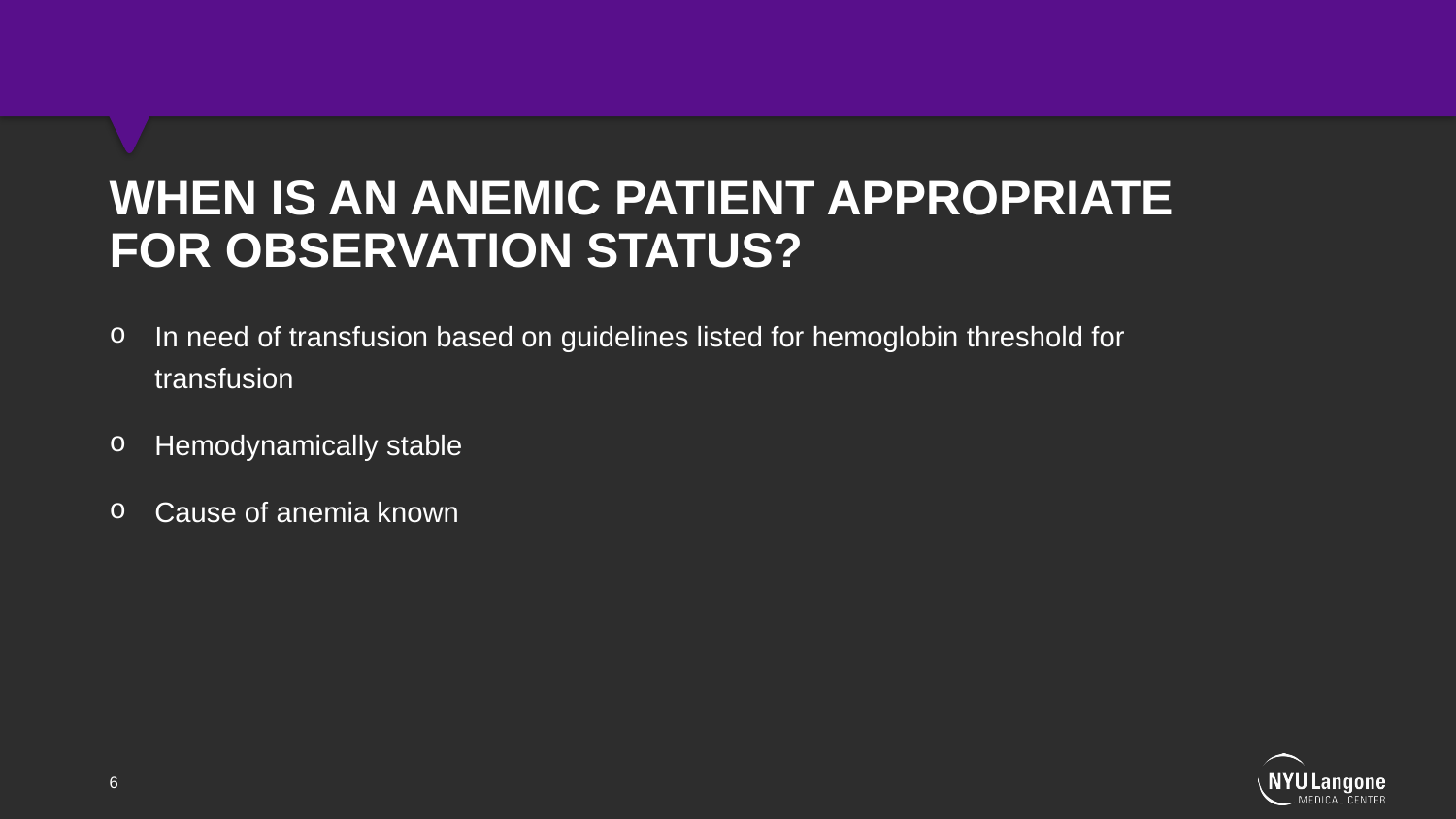

# WHEN IS AN ANEMIC PATIENT APPROPRIATE FOR OBSERVATION STATUS?
In need of transfusion based on guidelines listed for hemoglobin threshold for transfusion
Hemodynamically stable
Cause of anemia known
6

## Slide 7
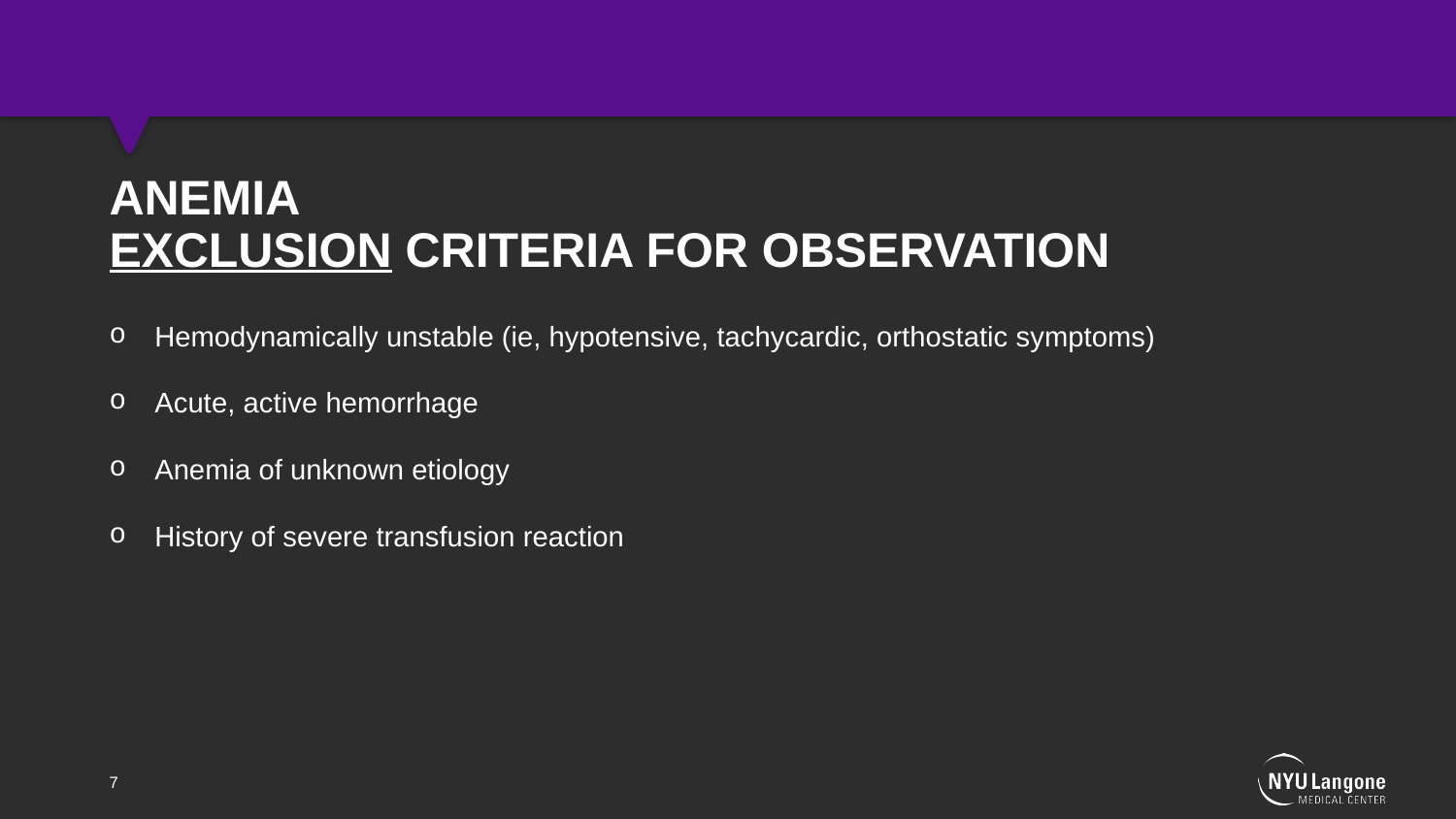

# ANEMIA EXCLUSION CRITERIA FOR OBSERVATION
Hemodynamically unstable (ie, hypotensive, tachycardic, orthostatic symptoms)
Acute, active hemorrhage
Anemia of unknown etiology
History of severe transfusion reaction
7

## Slide 8
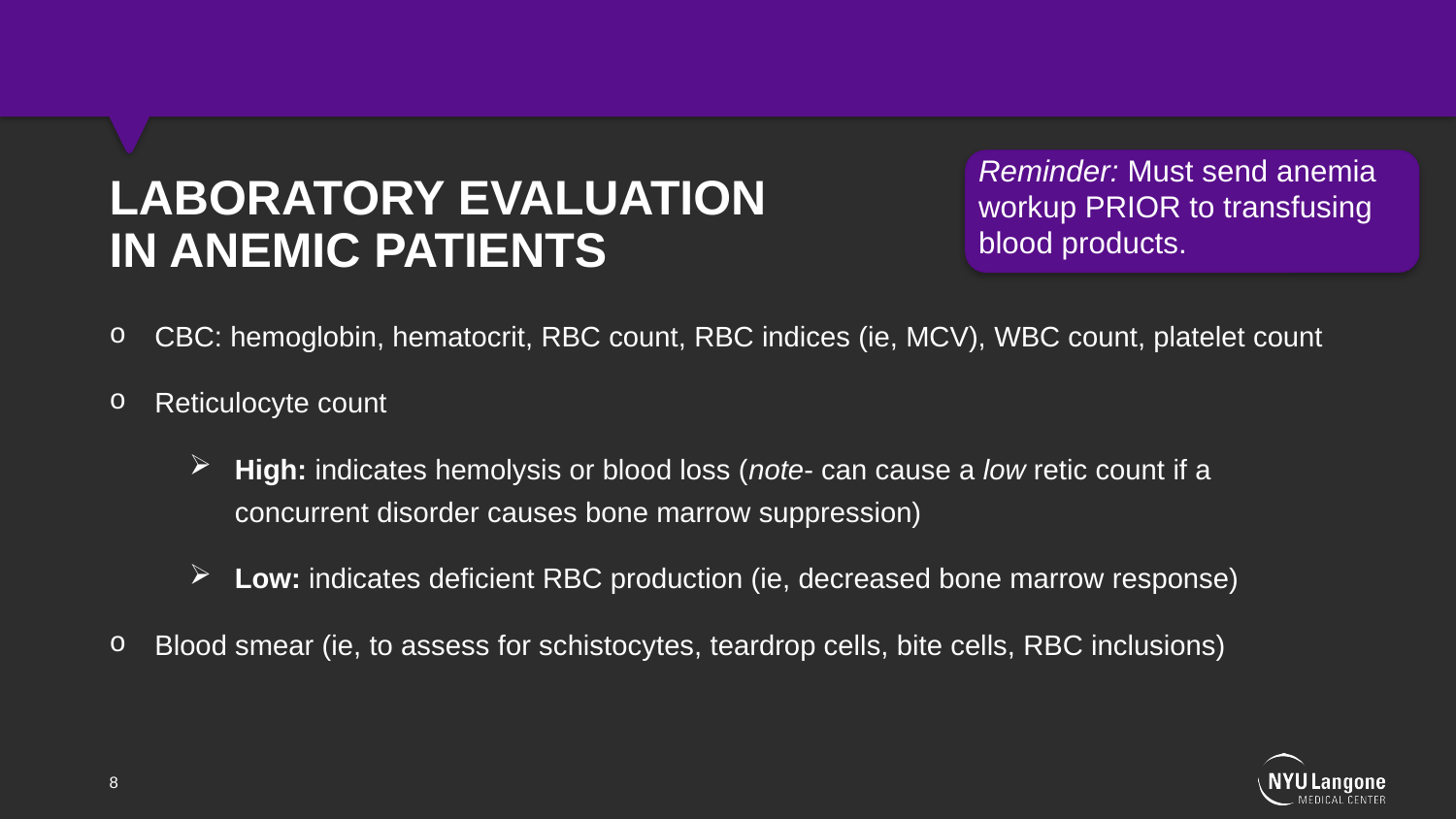

Reminder: Must send anemia workup PRIOR to transfusing blood products.
# LABORATORY EVALUATION IN ANEMIC PATIENTS
CBC: hemoglobin, hematocrit, RBC count, RBC indices (ie, MCV), WBC count, platelet count
Reticulocyte count
High: indicates hemolysis or blood loss (note- can cause a low retic count if a concurrent disorder causes bone marrow suppression)
Low: indicates deficient RBC production (ie, decreased bone marrow response)
Blood smear (ie, to assess for schistocytes, teardrop cells, bite cells, RBC inclusions)
8

## Slide 9
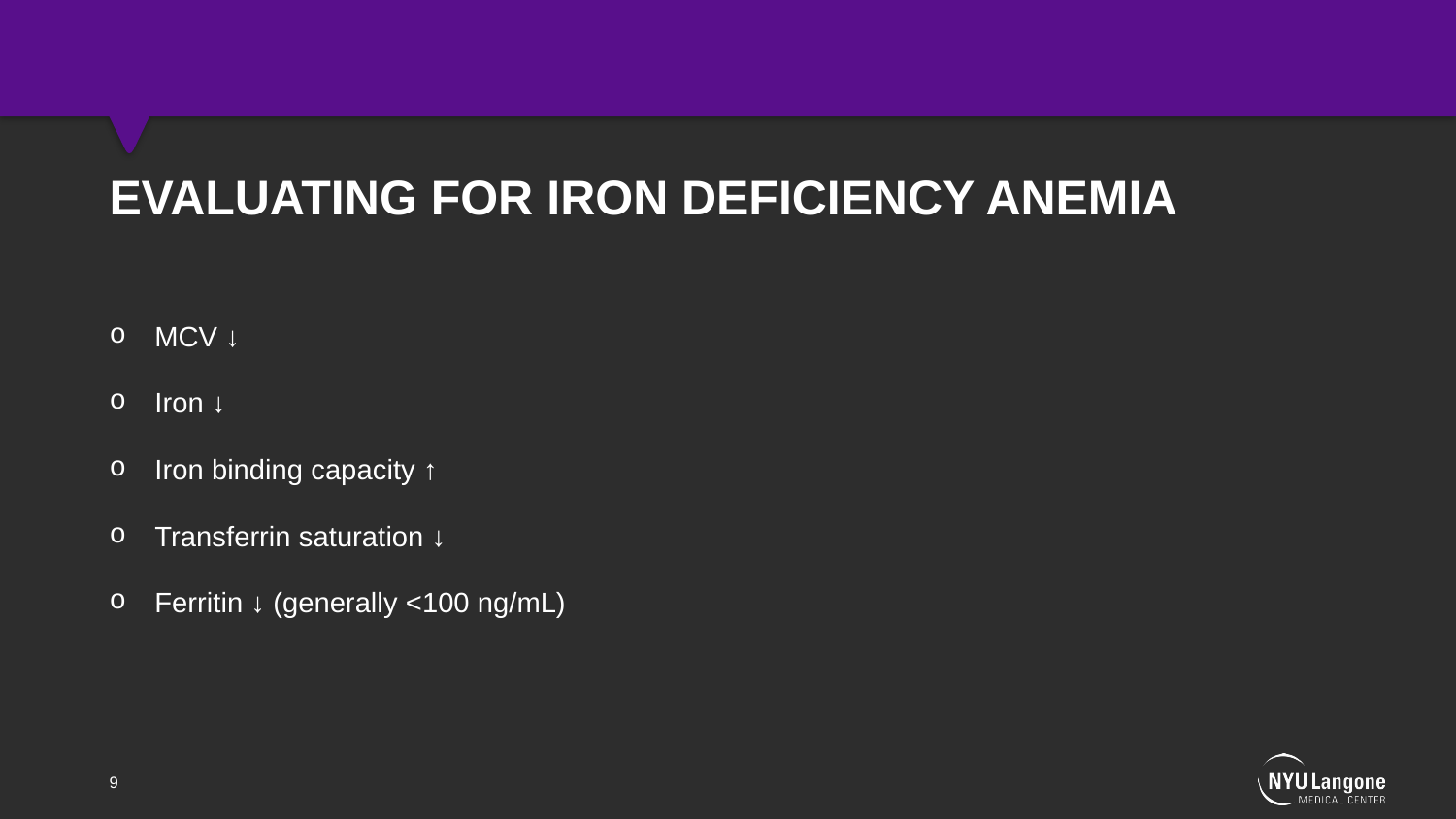

# EVALUATING FOR IRON DEFICIENCY ANEMIA
MCV ↓
Iron ↓
Iron binding capacity ↑
Transferrin saturation ↓
Ferritin ↓ (generally <100 ng/mL)
9

## Slide 10
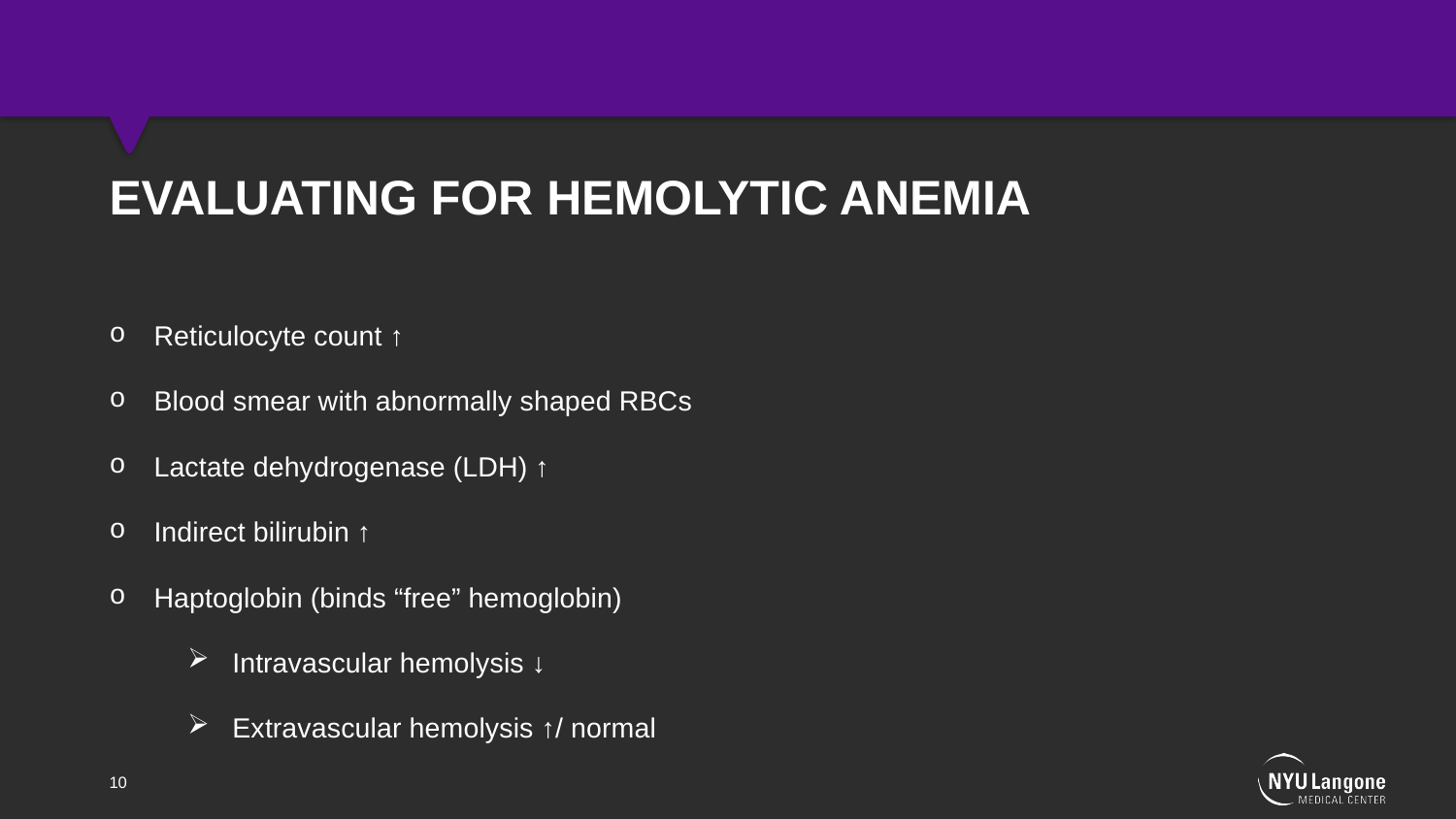

# EVALUATING FOR HEMOLYTIC ANEMIA
Reticulocyte count ↑
Blood smear with abnormally shaped RBCs
Lactate dehydrogenase (LDH) ↑
Indirect bilirubin ↑
Haptoglobin (binds “free” hemoglobin)
Intravascular hemolysis ↓
Extravascular hemolysis ↑/ normal
10

## Slide 11
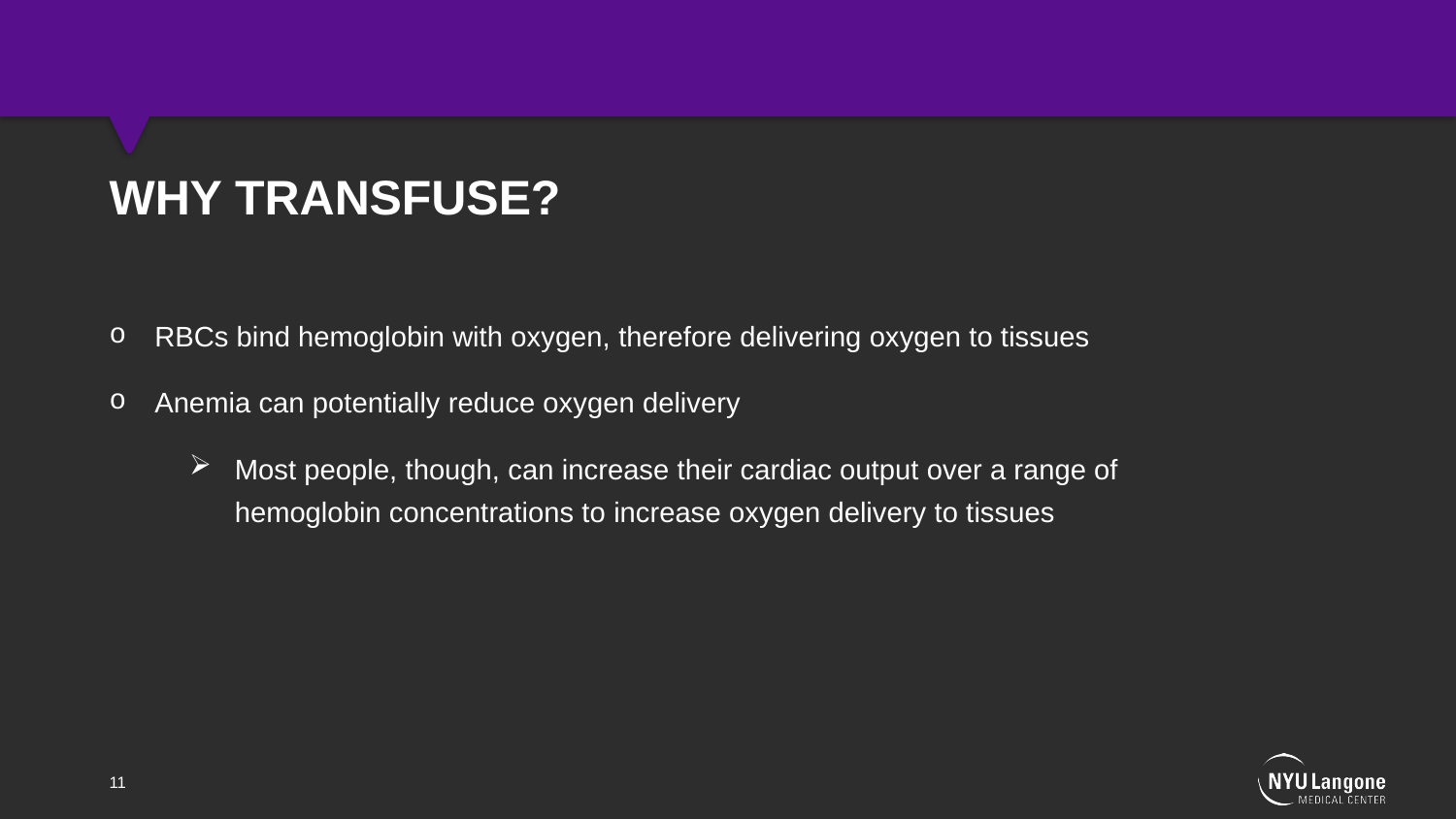

# WHY TRANSFUSE?
RBCs bind hemoglobin with oxygen, therefore delivering oxygen to tissues
Anemia can potentially reduce oxygen delivery
Most people, though, can increase their cardiac output over a range of hemoglobin concentrations to increase oxygen delivery to tissues
11

## Slide 12
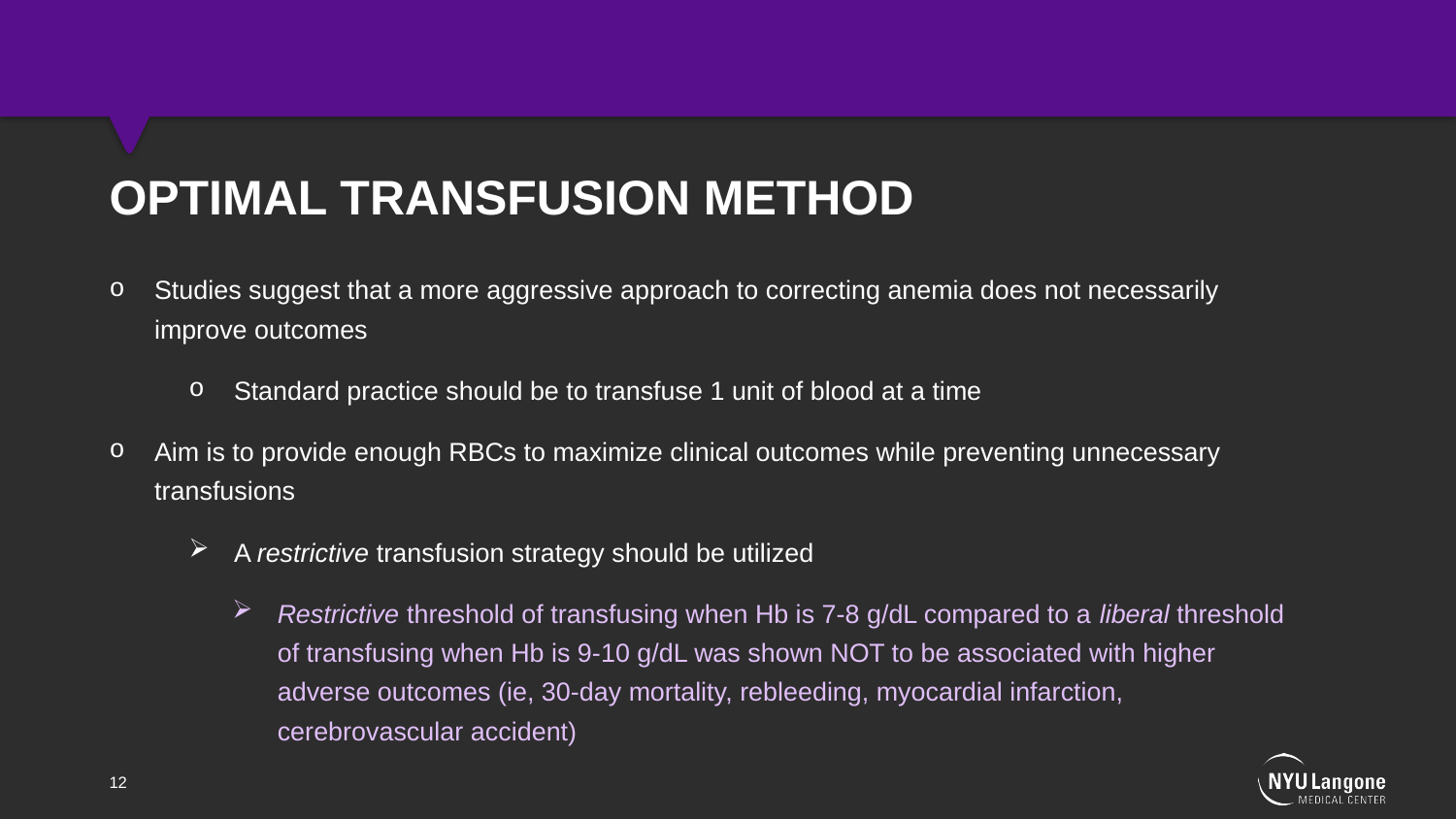

# OPTIMAL TRANSFUSION METHOD
Studies suggest that a more aggressive approach to correcting anemia does not necessarily improve outcomes
Standard practice should be to transfuse 1 unit of blood at a time
Aim is to provide enough RBCs to maximize clinical outcomes while preventing unnecessary transfusions
A restrictive transfusion strategy should be utilized
Restrictive threshold of transfusing when Hb is 7-8 g/dL compared to a liberal threshold of transfusing when Hb is 9-10 g/dL was shown NOT to be associated with higher adverse outcomes (ie, 30-day mortality, rebleeding, myocardial infarction, cerebrovascular accident)
12

## Slide 13
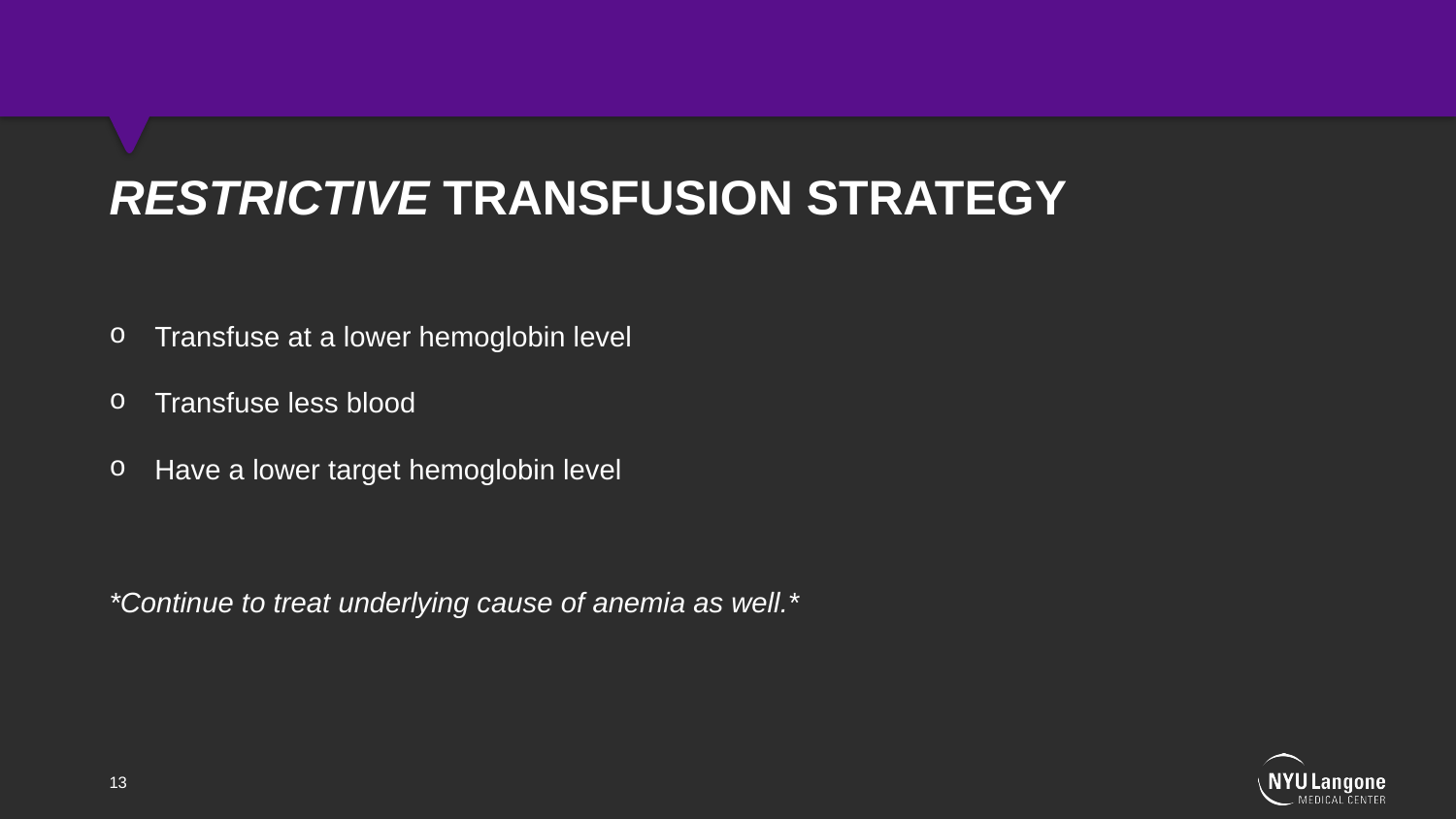

# RESTRICTIVE TRANSFUSION STRATEGY
Transfuse at a lower hemoglobin level
Transfuse less blood
Have a lower target hemoglobin level
*Continue to treat underlying cause of anemia as well.*
13

## Slide 14
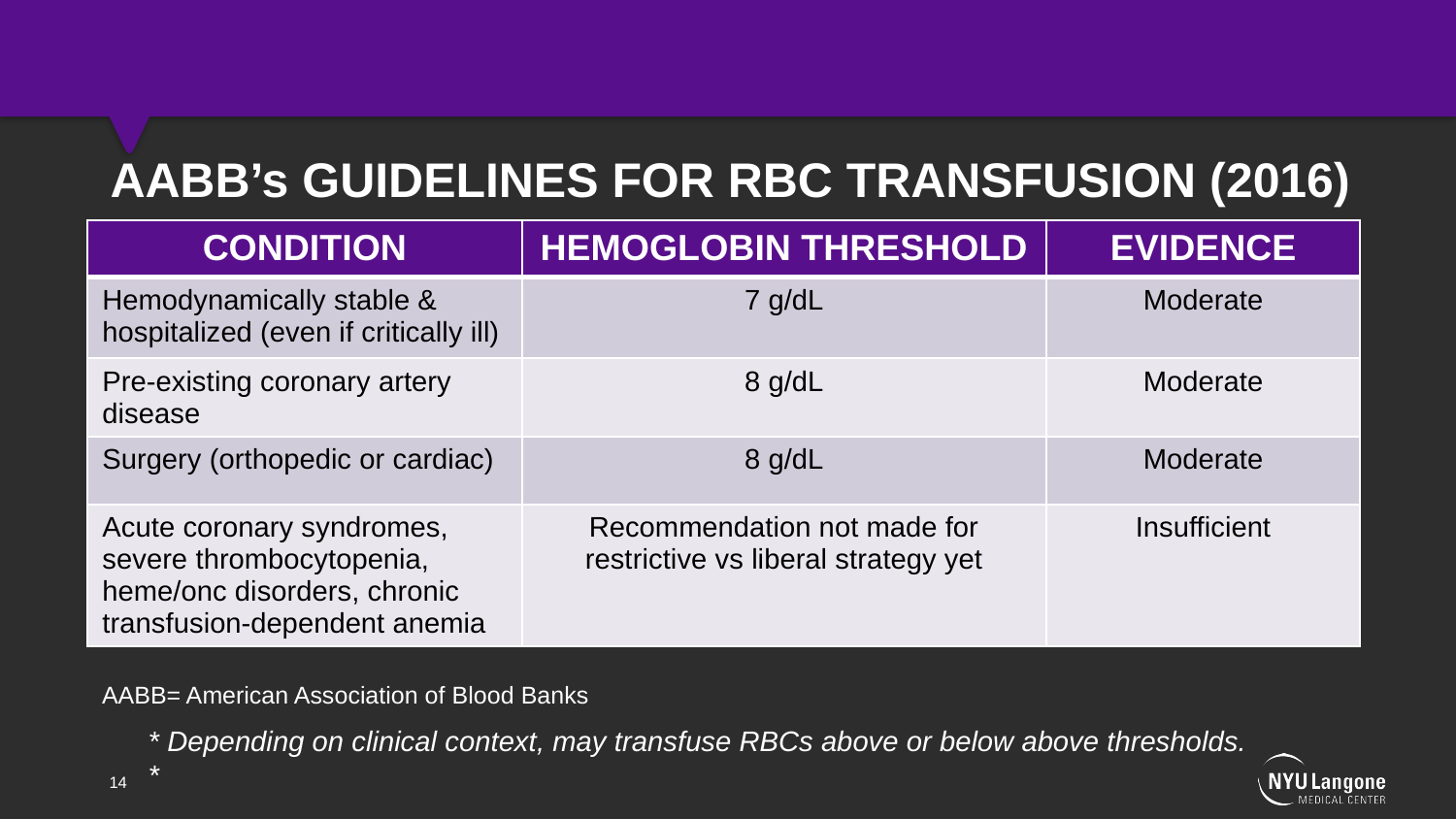

# AABB’s GUIDELINES FOR RBC TRANSFUSION (2016)
| CONDITION | HEMOGLOBIN THRESHOLD | EVIDENCE |
| --- | --- | --- |
| Hemodynamically stable & hospitalized (even if critically ill) | 7 g/dL | Moderate |
| Pre-existing coronary artery disease | 8 g/dL | Moderate |
| Surgery (orthopedic or cardiac) | 8 g/dL | Moderate |
| Acute coronary syndromes, severe thrombocytopenia, heme/onc disorders, chronic transfusion-dependent anemia | Recommendation not made for restrictive vs liberal strategy yet | Insufficient |
AABB= American Association of Blood Banks
* Depending on clinical context, may transfuse RBCs above or below above thresholds. *
14

## Slide 15
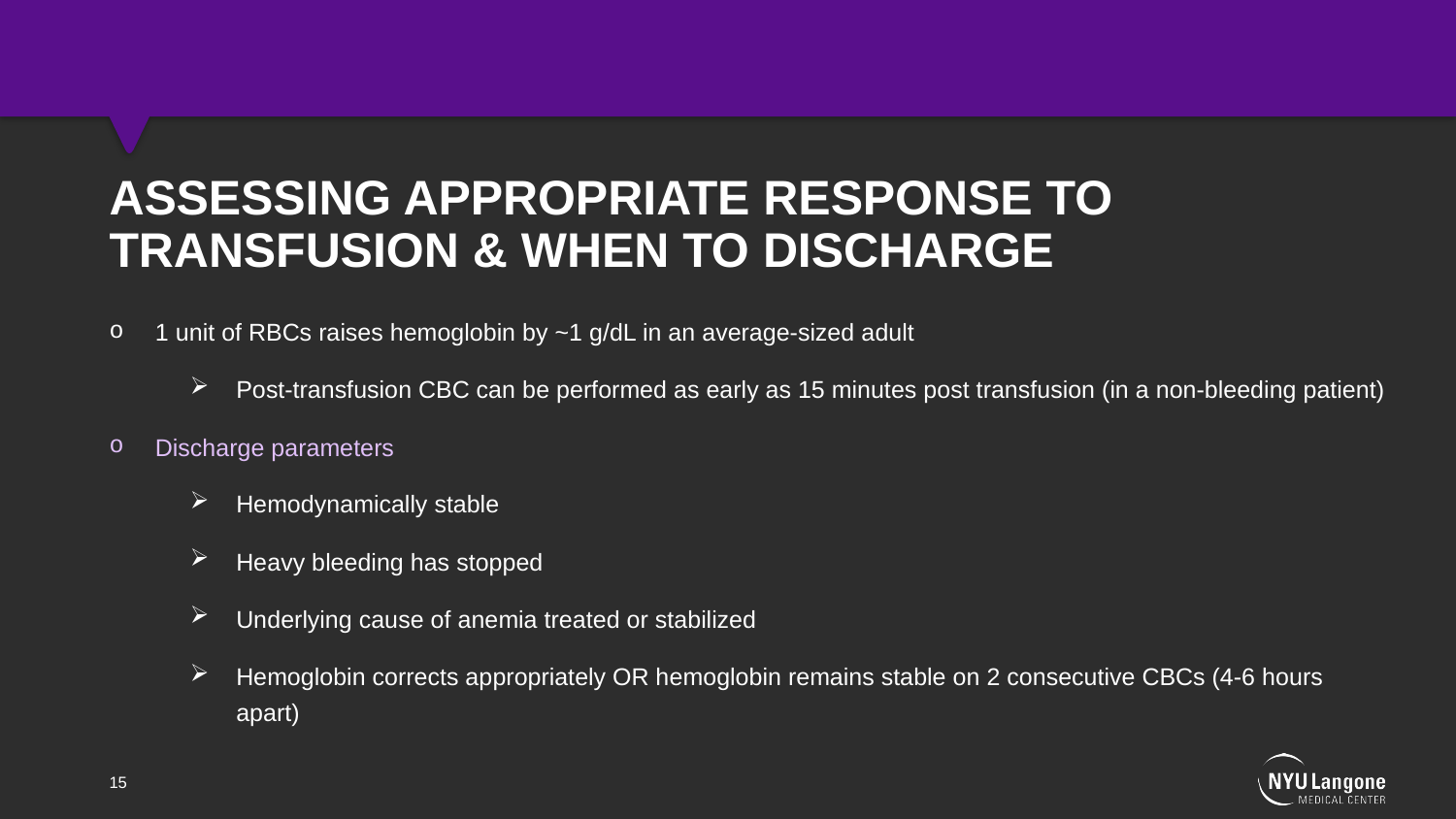

# ASSESSING APPROPRIATE RESPONSE TO TRANSFUSION & WHEN TO DISCHARGE
1 unit of RBCs raises hemoglobin by ~1 g/dL in an average-sized adult
Post-transfusion CBC can be performed as early as 15 minutes post transfusion (in a non-bleeding patient)
Discharge parameters
Hemodynamically stable
Heavy bleeding has stopped
Underlying cause of anemia treated or stabilized
Hemoglobin corrects appropriately OR hemoglobin remains stable on 2 consecutive CBCs (4-6 hours apart)
15

## Slide 16
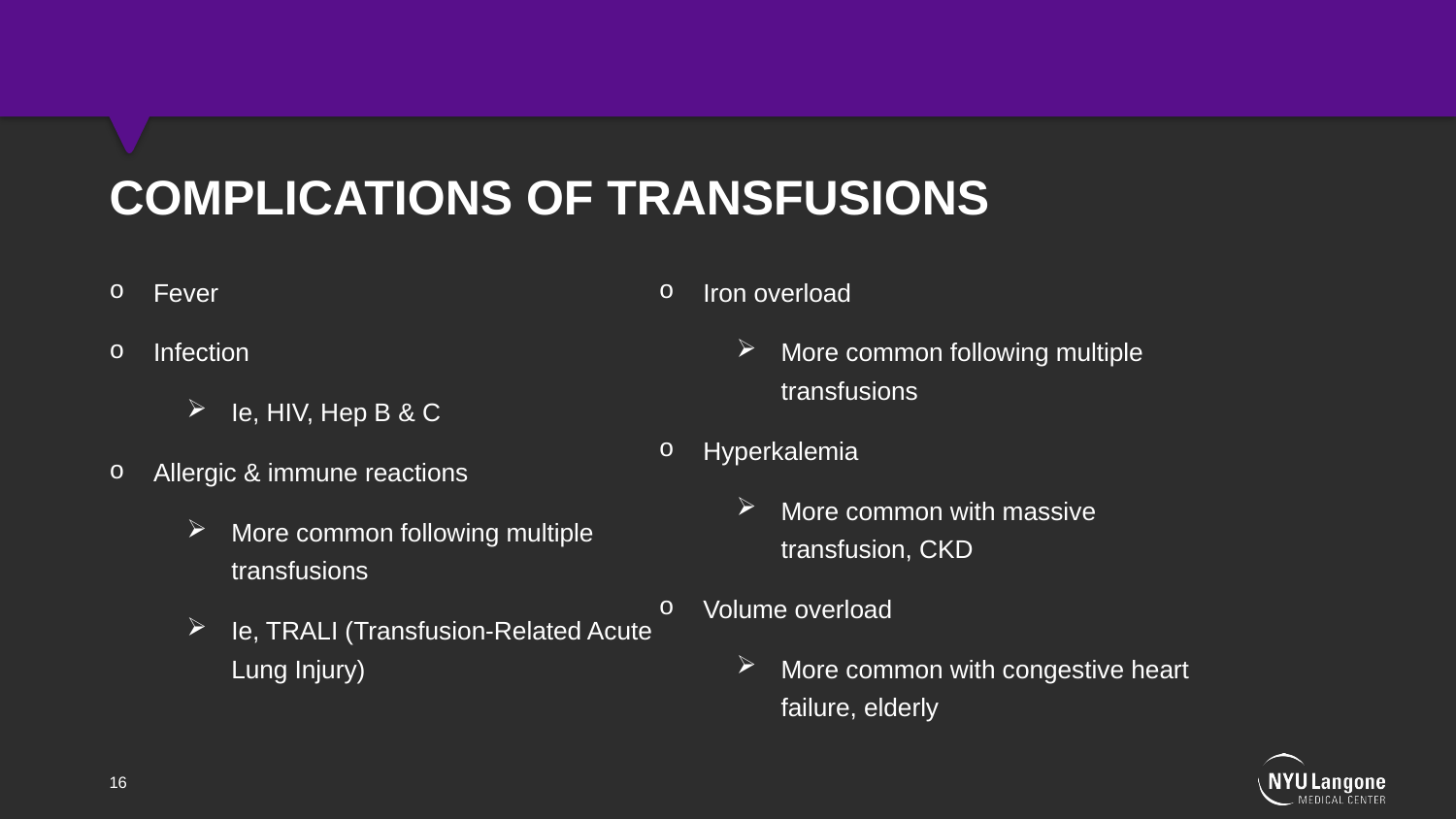

# COMPLICATIONS OF TRANSFUSIONS
Fever
Infection
Ie, HIV, Hep B & C
Allergic & immune reactions
More common following multiple transfusions
Ie, TRALI (Transfusion-Related Acute Lung Injury)
Iron overload
More common following multiple transfusions
Hyperkalemia
More common with massive transfusion, CKD
Volume overload
More common with congestive heart failure, elderly
16

## Slide 17
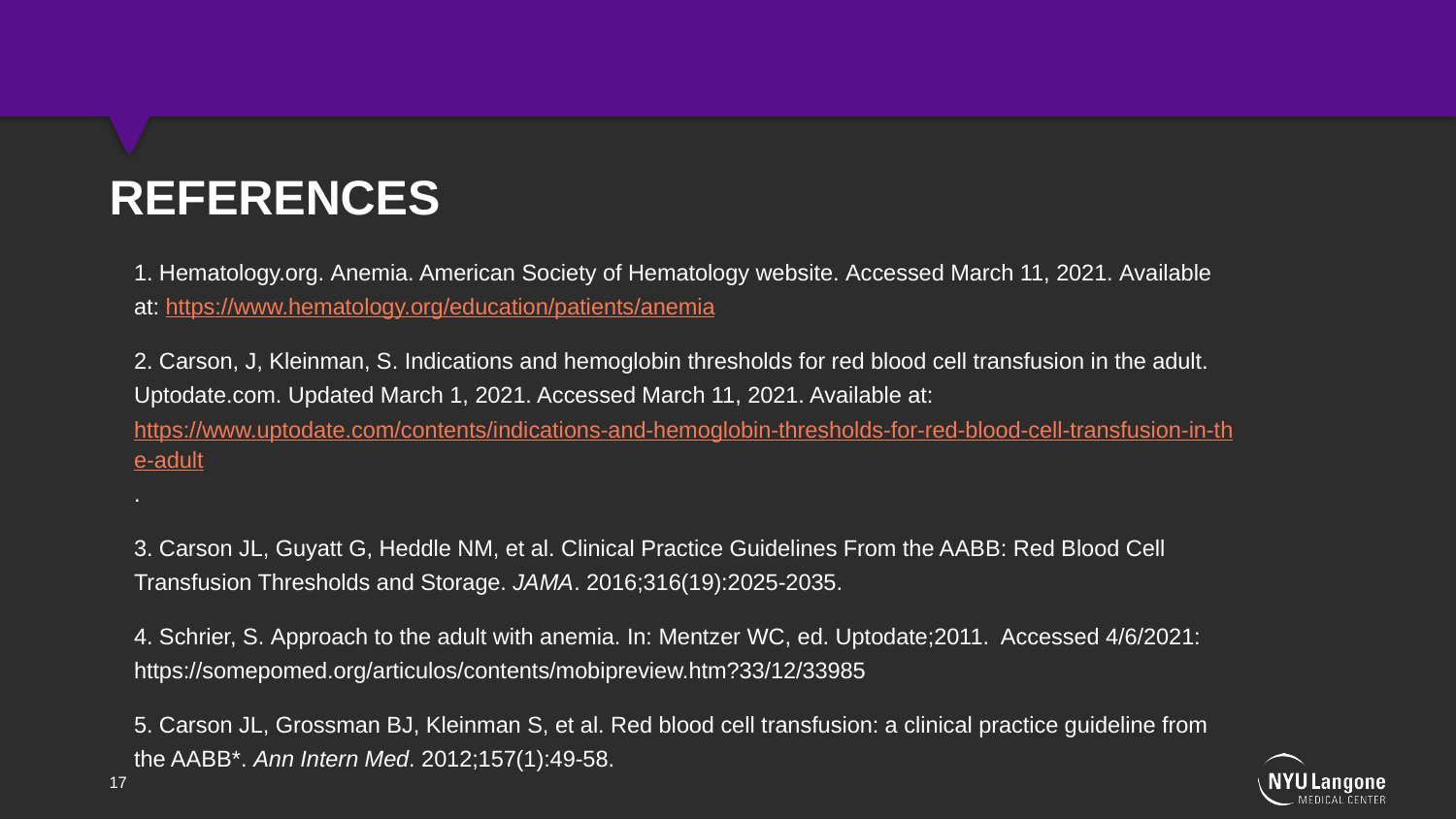

# REFERENCES
1. Hematology.org. Anemia. American Society of Hematology website. Accessed March 11, 2021. Available at: https://www.hematology.org/education/patients/anemia
2. Carson, J, Kleinman, S. Indications and hemoglobin thresholds for red blood cell transfusion in the adult.  Uptodate.com. Updated March 1, 2021. Accessed March 11, 2021. Available at: https://www.uptodate.com/contents/indications-and-hemoglobin-thresholds-for-red-blood-cell-transfusion-in-the-adult.
3. Carson JL, Guyatt G, Heddle NM, et al. Clinical Practice Guidelines From the AABB: Red Blood Cell Transfusion Thresholds and Storage. JAMA. 2016;316(19):2025-2035.
4. Schrier, S. Approach to the adult with anemia. In: Mentzer WC, ed. Uptodate;2011. Accessed 4/6/2021: https://somepomed.org/articulos/contents/mobipreview.htm?33/12/33985
5. Carson JL, Grossman BJ, Kleinman S, et al. Red blood cell transfusion: a clinical practice guideline from the AABB*. Ann Intern Med. 2012;157(1):49-58.
17
