# Supplementary material for: An Observation Medicine Curriculum for Emergency Medicine Education
Source: J Educ Teach Emerg Med. 2021 Apr 19;6(2):C1–C72. doi: 10.21980/J87P92 (PMC10332786; doi:10.21980/J87P92)
Supplement: Supplementary file 19 — Please see associated PowerPoint file [file jetem-6-2-c1-supp19.pptx]

## Slide 1
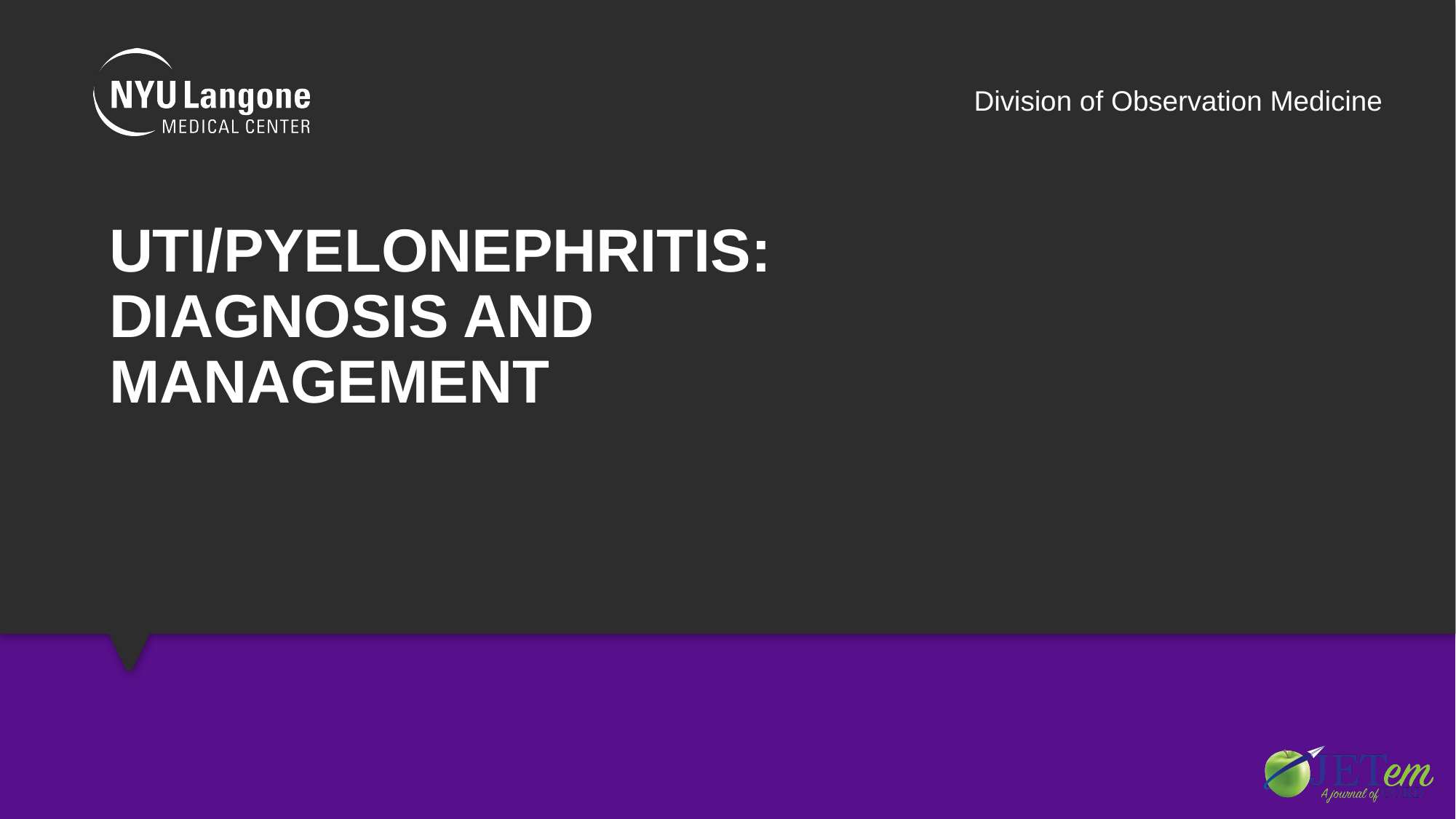

Division of Observation Medicine
# UTI/Pyelonephritis: Diagnosis and Management

## Slide 2
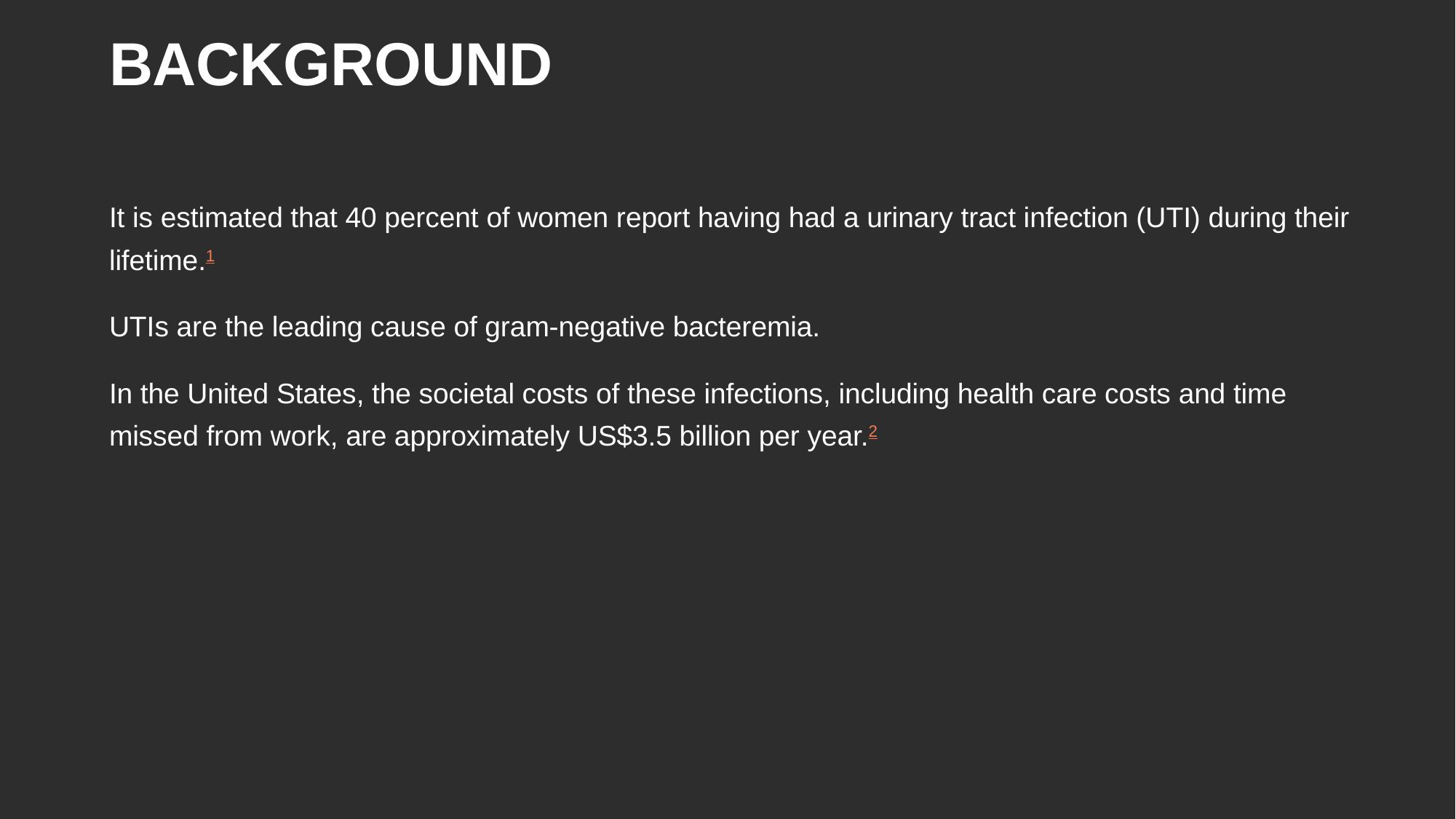

# Background
It is estimated that 40 percent of women report having had a urinary tract infection (UTI) during their lifetime.1
UTIs are the leading cause of gram-negative bacteremia.
In the United States, the societal costs of these infections, including health care costs and time missed from work, are approximately US$3.5 billion per year.2

## Slide 3
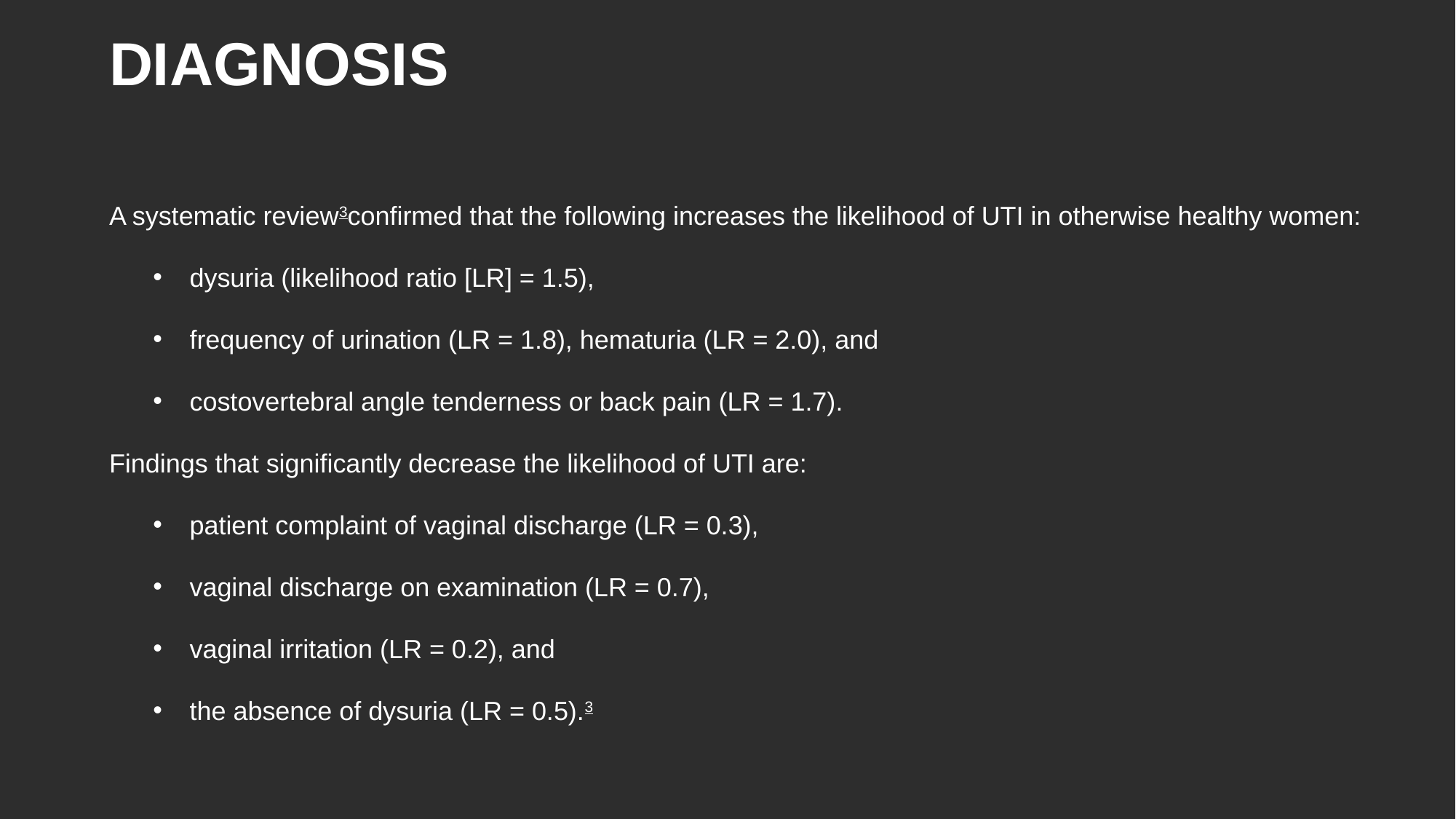

# Diagnosis
A systematic review3confirmed that the following increases the likelihood of UTI in otherwise healthy women:
dysuria (likelihood ratio [LR] = 1.5),
frequency of urination (LR = 1.8), hematuria (LR = 2.0), and
costovertebral angle tenderness or back pain (LR = 1.7).
Findings that significantly decrease the likelihood of UTI are:
patient complaint of vaginal discharge (LR = 0.3),
vaginal discharge on examination (LR = 0.7),
vaginal irritation (LR = 0.2), and
the absence of dysuria (LR = 0.5).3

## Slide 4
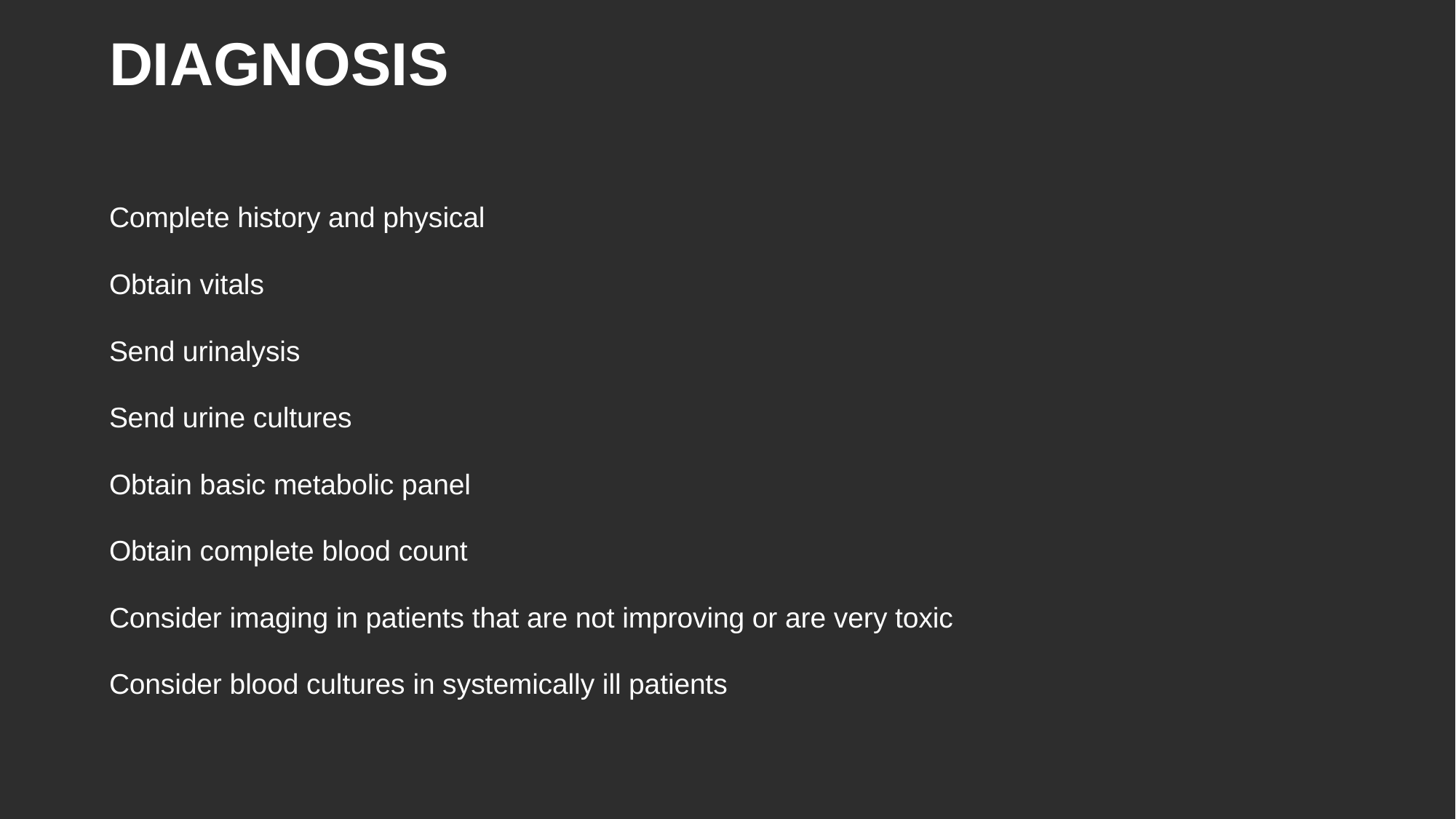

# Diagnosis
Complete history and physical
Obtain vitals
Send urinalysis
Send urine cultures
Obtain basic metabolic panel
Obtain complete blood count
Consider imaging in patients that are not improving or are very toxic
Consider blood cultures in systemically ill patients

## Slide 5
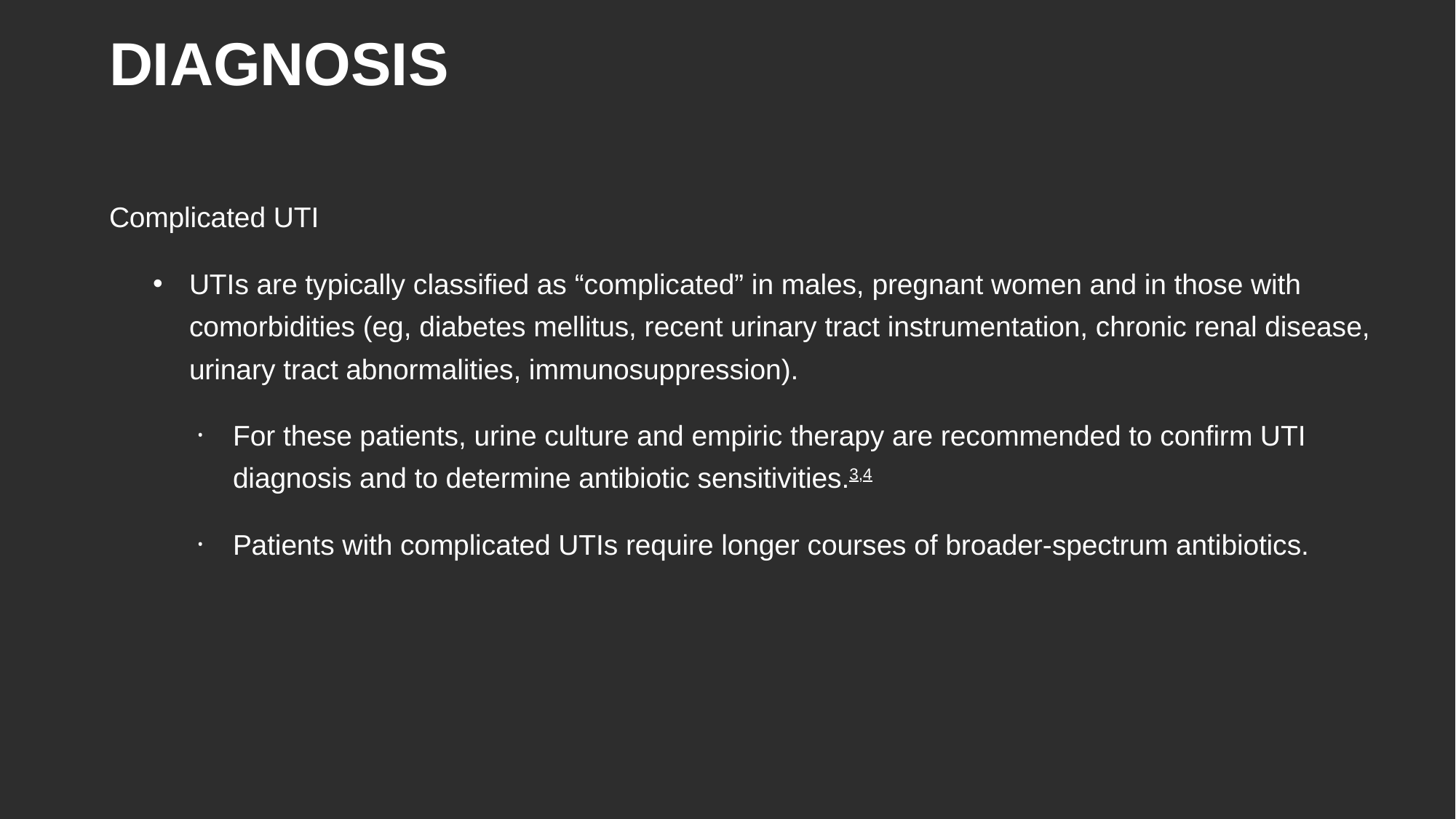

# Diagnosis
Complicated UTI
UTIs are typically classified as “complicated” in males, pregnant women and in those with comorbidities (eg, diabetes mellitus, recent urinary tract instrumentation, chronic renal disease, urinary tract abnormalities, immunosuppression).
For these patients, urine culture and empiric therapy are recommended to confirm UTI diagnosis and to determine antibiotic sensitivities.3,4
Patients with complicated UTIs require longer courses of broader-spectrum antibiotics.

## Slide 6
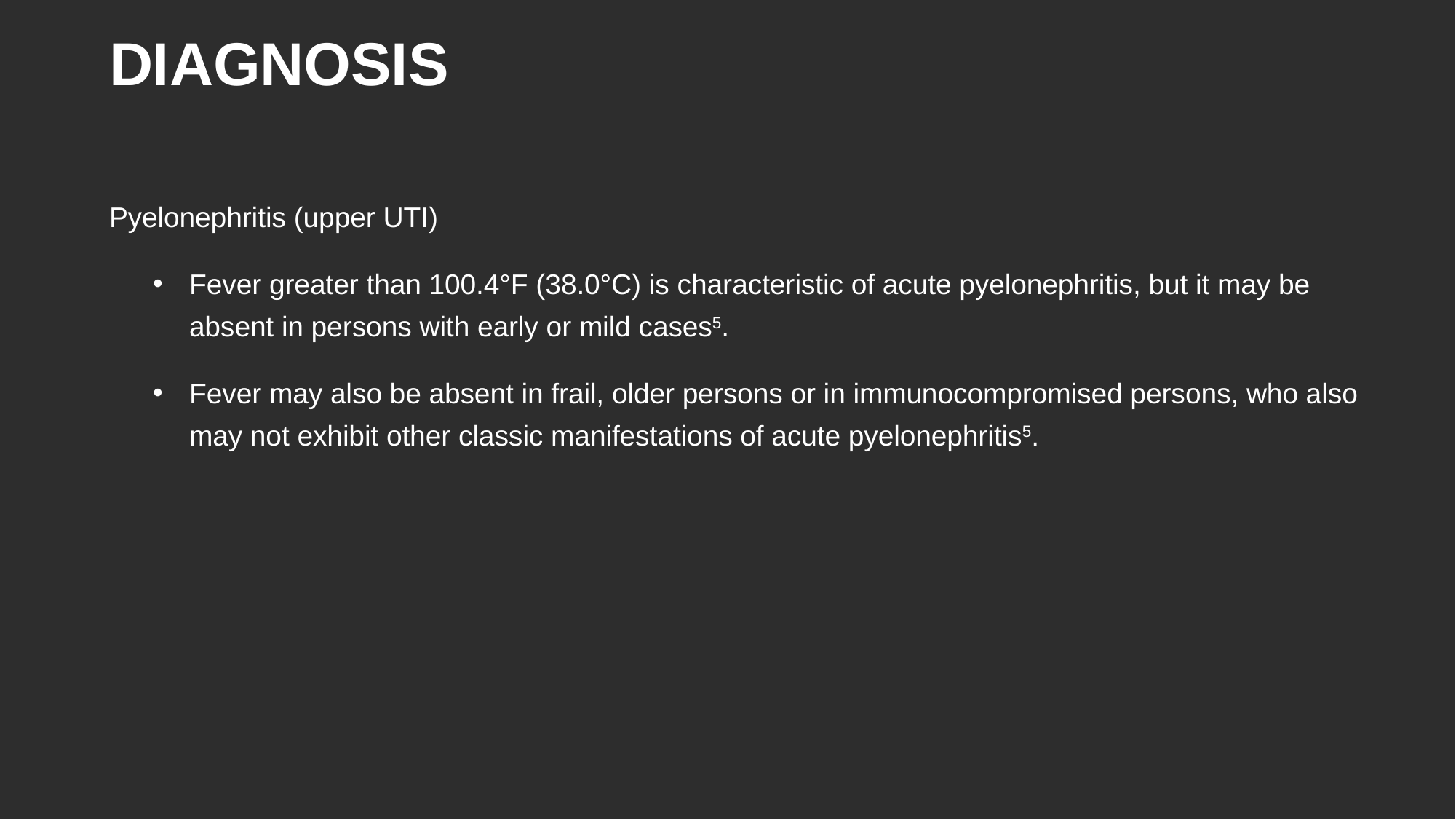

# Diagnosis
Pyelonephritis (upper UTI)
Fever greater than 100.4°F (38.0°C) is characteristic of acute pyelonephritis, but it may be absent in persons with early or mild cases5.
Fever may also be absent in frail, older persons or in immunocompromised persons, who also may not exhibit other classic manifestations of acute pyelonephritis5.

## Slide 7
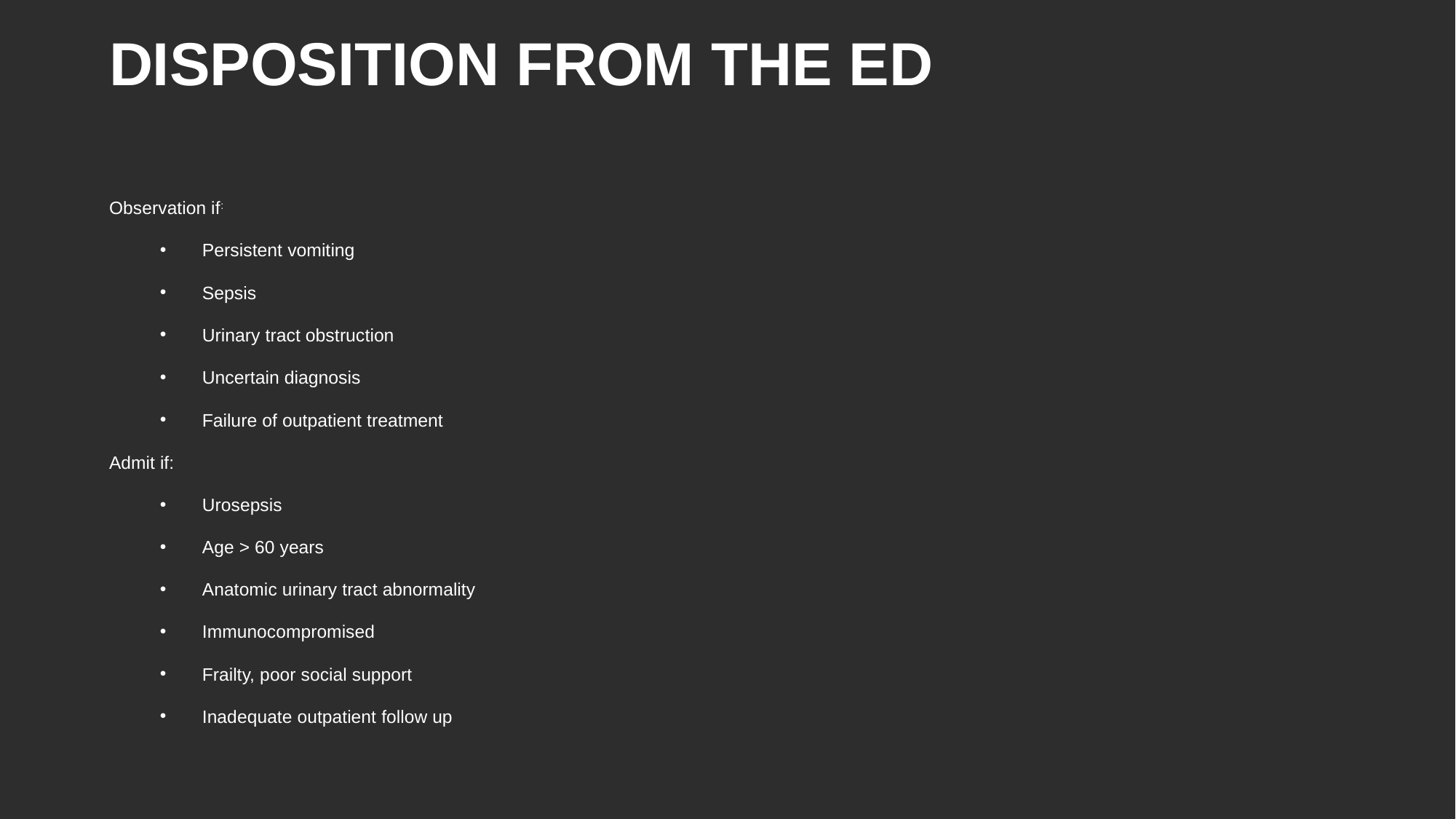

# Disposition from the ED
Observation if:
Persistent vomiting
Sepsis
Urinary tract obstruction
Uncertain diagnosis
Failure of outpatient treatment
Admit if:
Urosepsis
Age > 60 years
Anatomic urinary tract abnormality
Immunocompromised
Frailty, poor social support
Inadequate outpatient follow up

## Slide 8
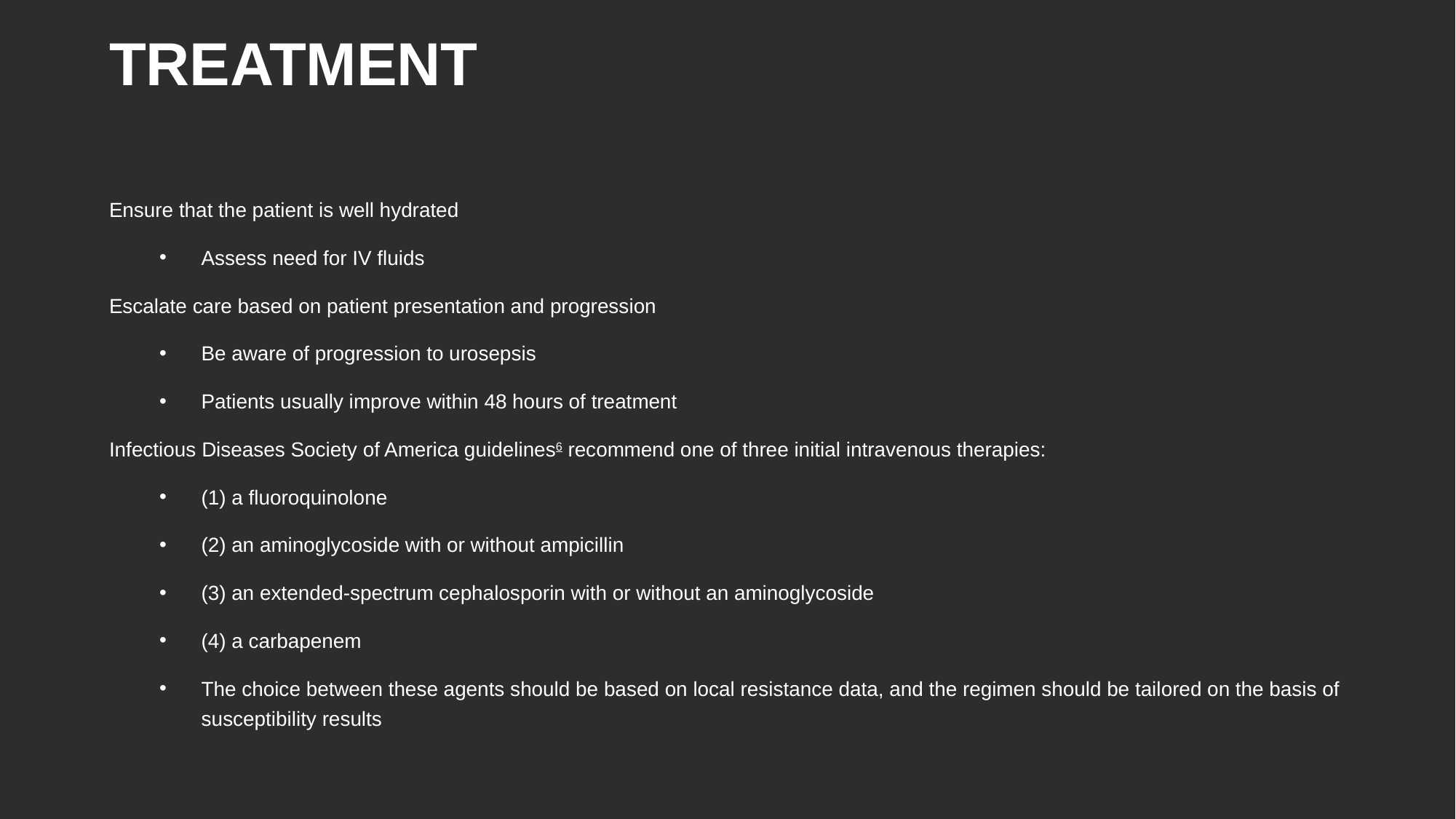

# Treatment
Ensure that the patient is well hydrated
Assess need for IV fluids
Escalate care based on patient presentation and progression
Be aware of progression to urosepsis
Patients usually improve within 48 hours of treatment
Infectious Diseases Society of America guidelines6 recommend one of three initial intravenous therapies:
(1) a fluoroquinolone
(2) an aminoglycoside with or without ampicillin
(3) an extended-spectrum cephalosporin with or without an aminoglycoside
(4) a carbapenem
The choice between these agents should be based on local resistance data, and the regimen should be tailored on the basis of susceptibility results

## Slide 9
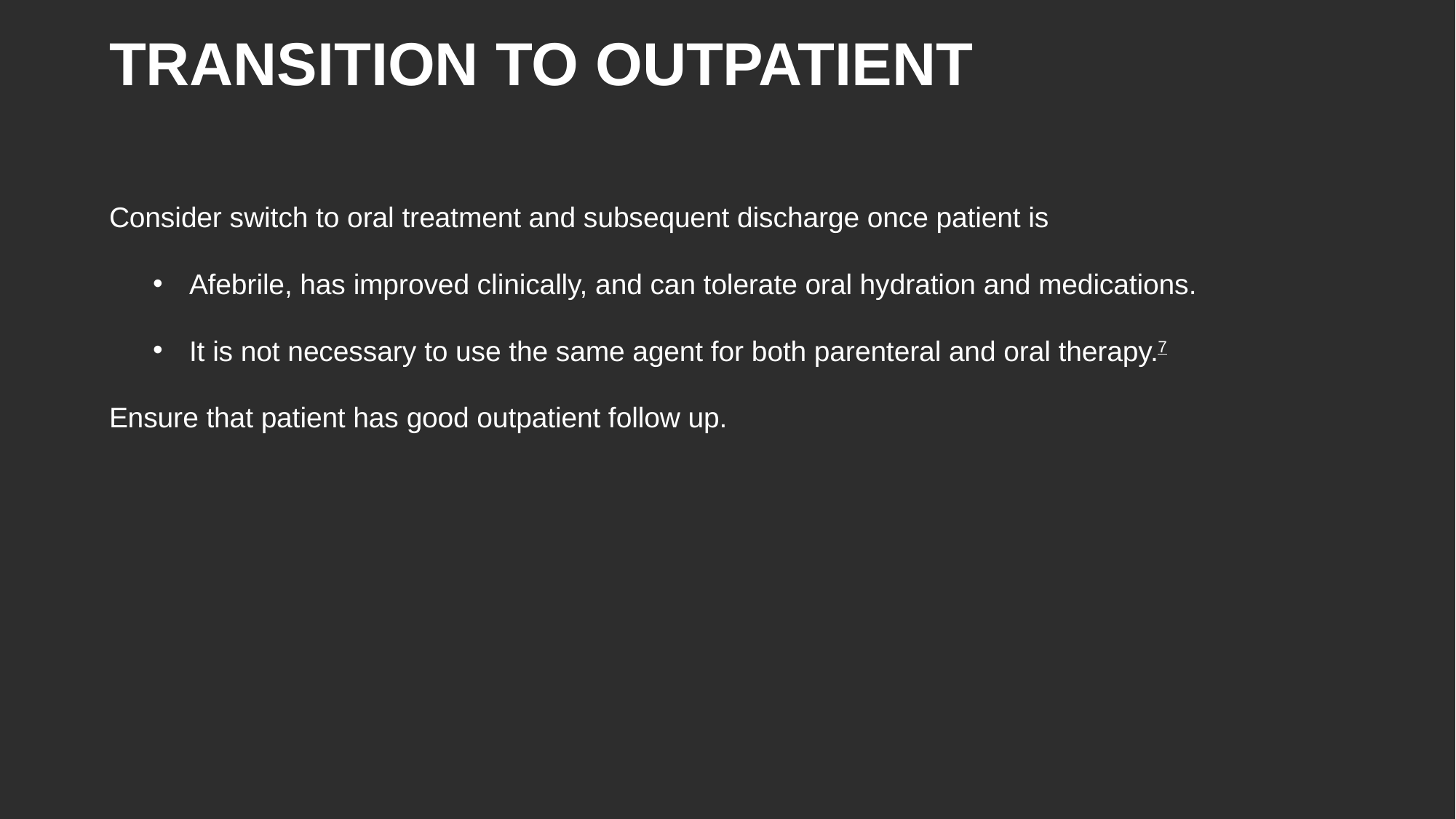

# Transition to outpatient
Consider switch to oral treatment and subsequent discharge once patient is
Afebrile, has improved clinically, and can tolerate oral hydration and medications.
It is not necessary to use the same agent for both parenteral and oral therapy.7
Ensure that patient has good outpatient follow up.

## Slide 10
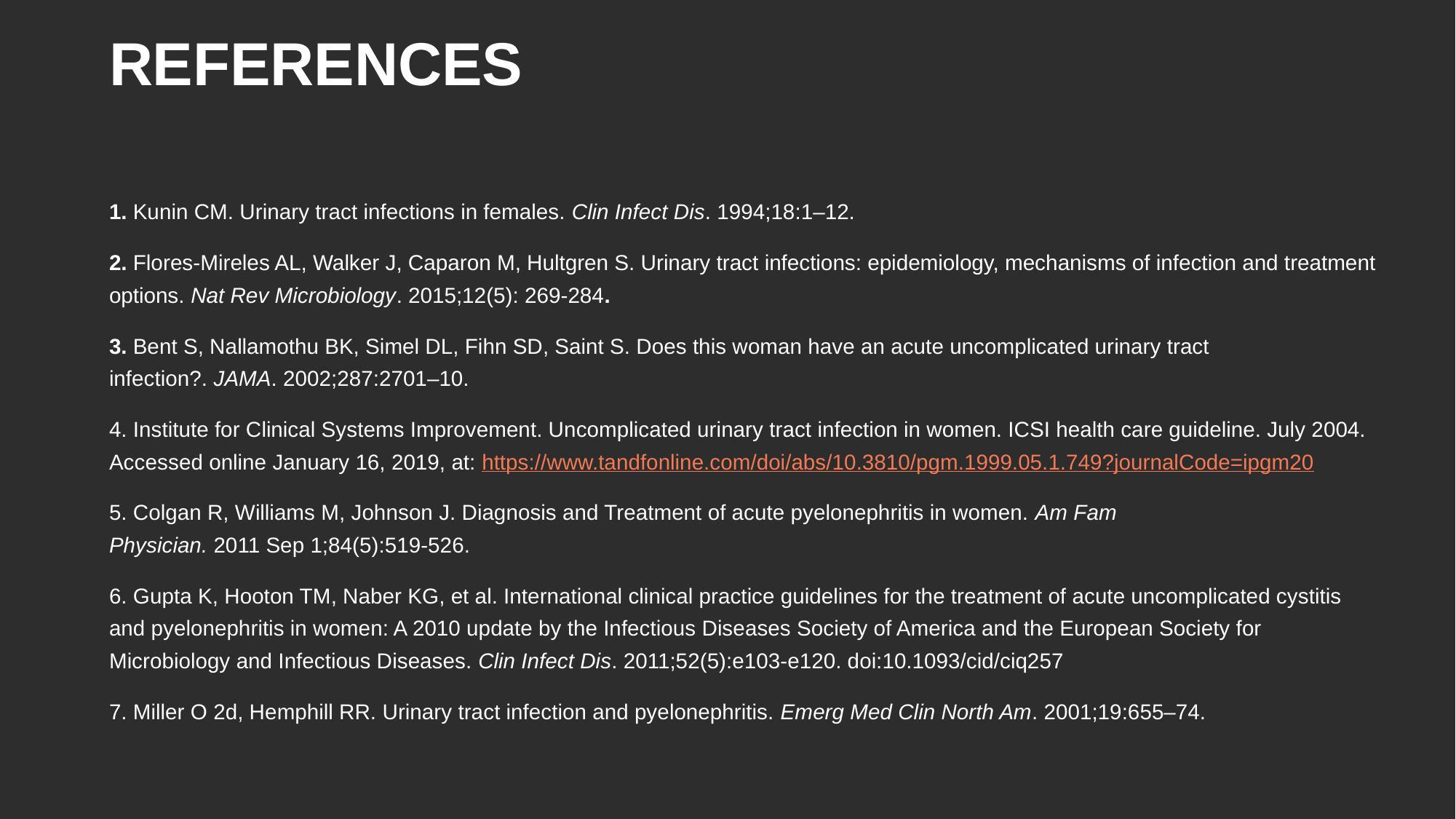

# References
1. Kunin CM. Urinary tract infections in females. Clin Infect Dis. 1994;18:1–12.
2. Flores-Mireles AL, Walker J, Caparon M, Hultgren S. Urinary tract infections: epidemiology, mechanisms of infection and treatment options. Nat Rev Microbiology. 2015;12(5): 269-284.
3. Bent S, Nallamothu BK, Simel DL, Fihn SD, Saint S. Does this woman have an acute uncomplicated urinary tract infection?. JAMA. 2002;287:2701–10.
4. Institute for Clinical Systems Improvement. Uncomplicated urinary tract infection in women. ICSI health care guideline. July 2004. Accessed online January 16, 2019, at: https://www.tandfonline.com/doi/abs/10.3810/pgm.1999.05.1.749?journalCode=ipgm20
5. Colgan R, Williams M, Johnson J. Diagnosis and Treatment of acute pyelonephritis in women. Am Fam Physician. 2011 Sep 1;84(5):519-526.
6. Gupta K, Hooton TM, Naber KG, et al. International clinical practice guidelines for the treatment of acute uncomplicated cystitis and pyelonephritis in women: A 2010 update by the Infectious Diseases Society of America and the European Society for Microbiology and Infectious Diseases. Clin Infect Dis. 2011;52(5):e103-e120. doi:10.1093/cid/ciq257
7. Miller O 2d, Hemphill RR. Urinary tract infection and pyelonephritis. Emerg Med Clin North Am. 2001;19:655–74.
