# Supplementary material for: An Observation Medicine Curriculum for Emergency Medicine Education
Source: J Educ Teach Emerg Med. 2021 Apr 19;6(2):C1–C72. doi: 10.21980/J87P92 (PMC10332786; doi:10.21980/J87P92)
Supplement: Supplementary file 20 — Please see associated PowerPoint file [file jetem-6-2-c1-supp20.pptx]

## Slide 1
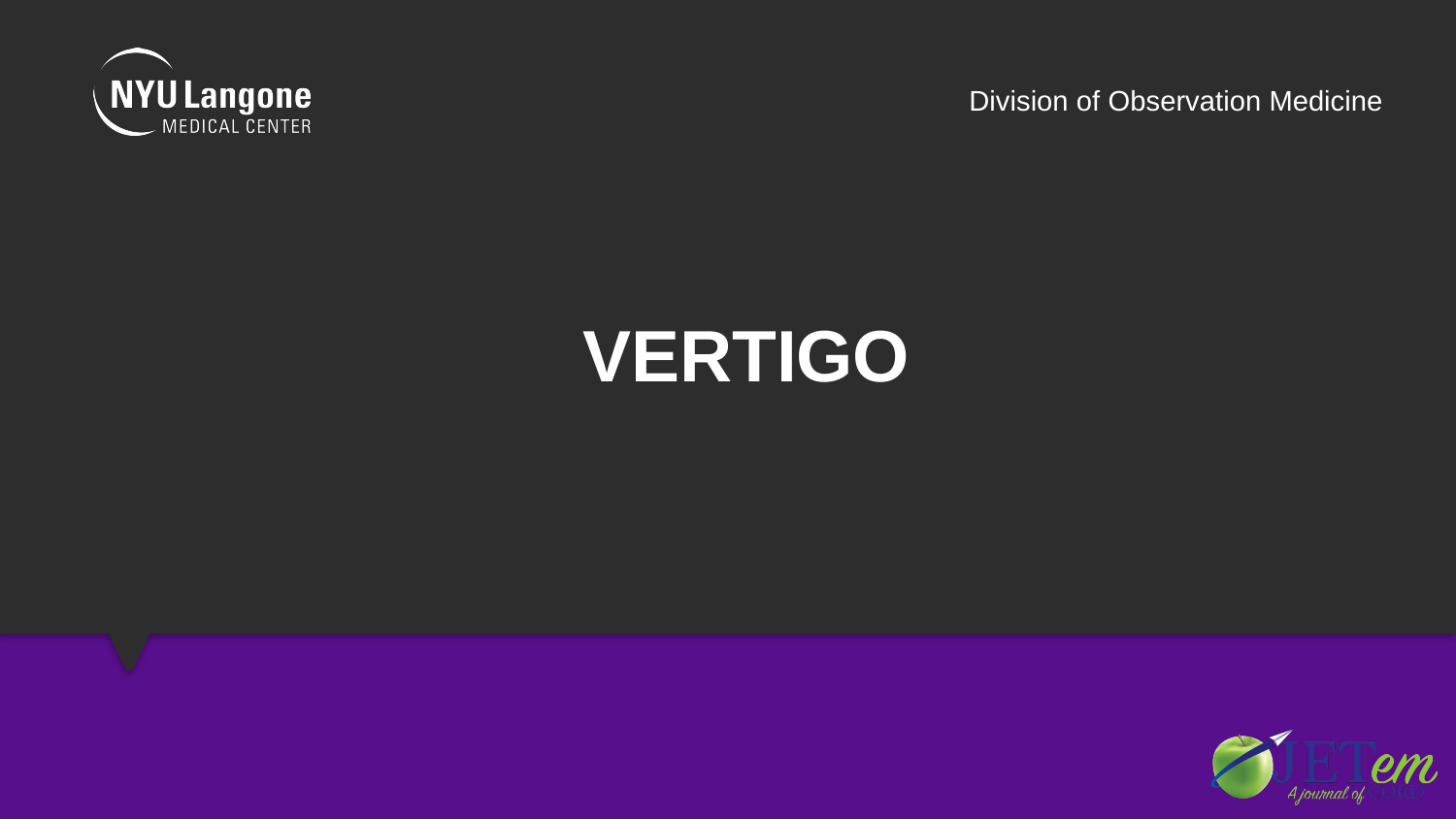

Division of Observation Medicine
# VERTIGO

## Slide 2
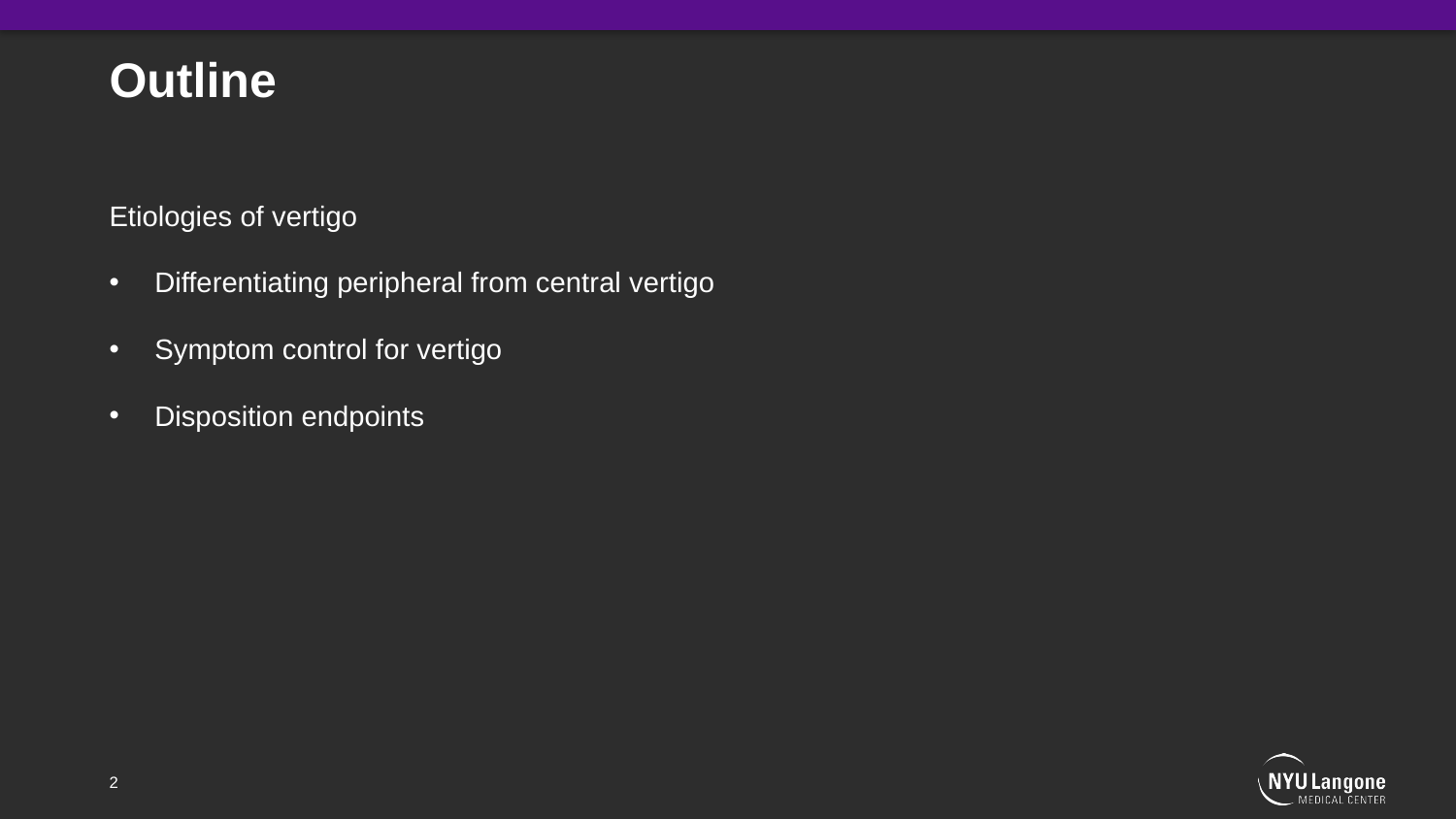

# Outline
Etiologies of vertigo
Differentiating peripheral from central vertigo
Symptom control for vertigo
Disposition endpoints
2

## Slide 3
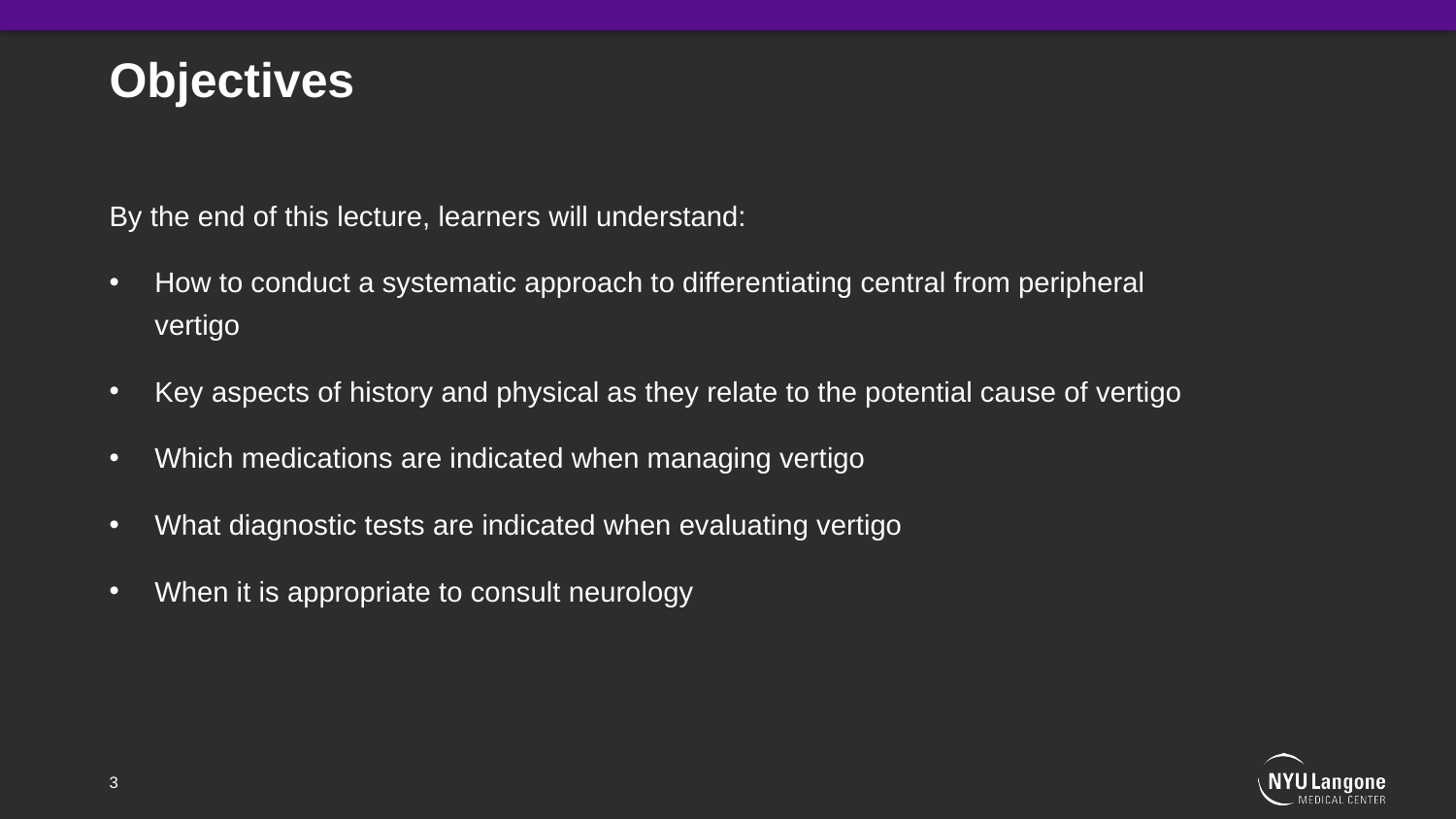

# Objectives
By the end of this lecture, learners will understand:
How to conduct a systematic approach to differentiating central from peripheral vertigo
Key aspects of history and physical as they relate to the potential cause of vertigo
Which medications are indicated when managing vertigo
What diagnostic tests are indicated when evaluating vertigo
When it is appropriate to consult neurology
3

## Slide 4
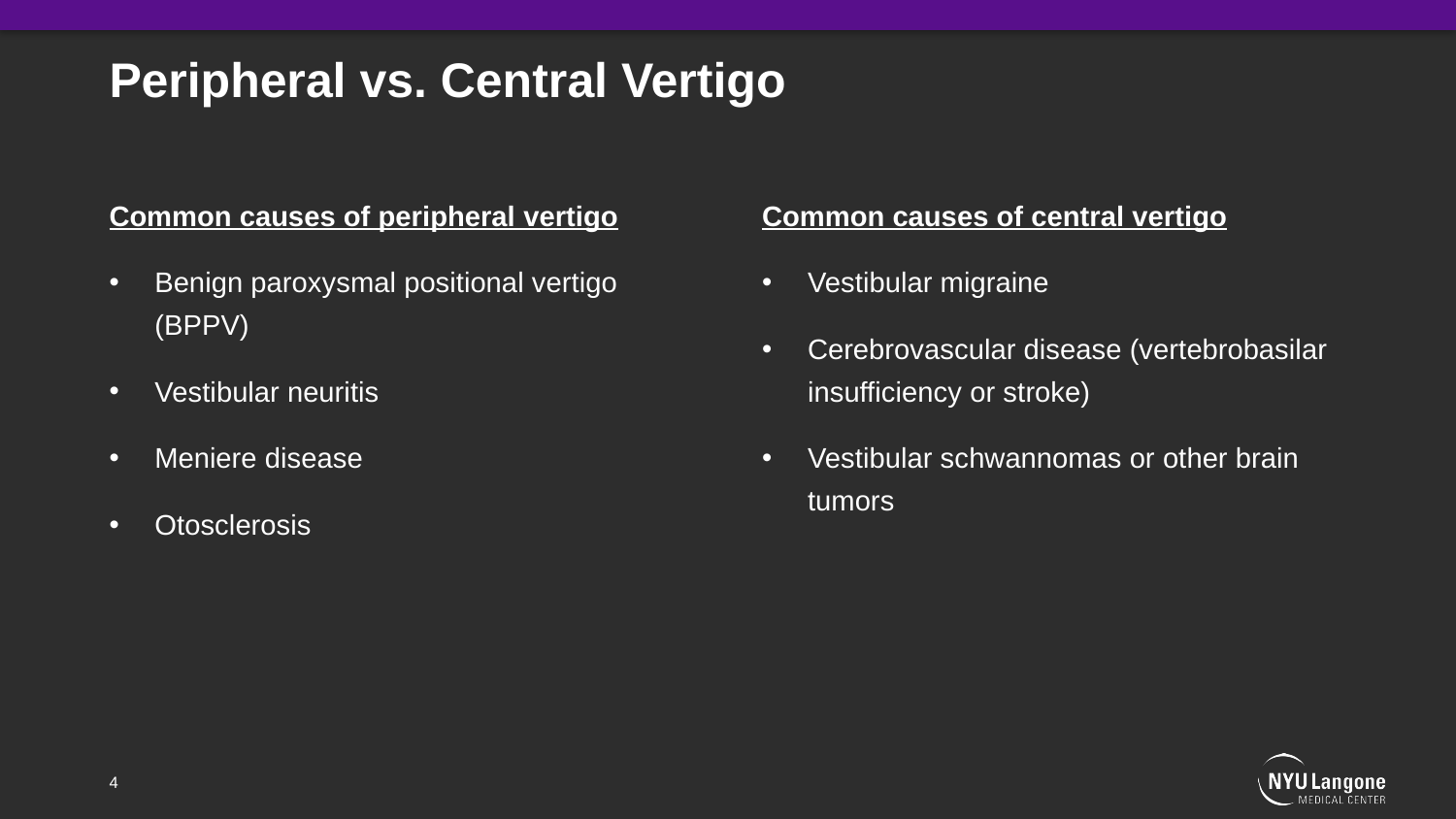

# Peripheral vs. Central Vertigo
Common causes of peripheral vertigo
Benign paroxysmal positional vertigo (BPPV)
Vestibular neuritis
Meniere disease
Otosclerosis
Common causes of central vertigo
Vestibular migraine
Cerebrovascular disease (vertebrobasilar insufficiency or stroke)
Vestibular schwannomas or other brain tumors
4

## Slide 5
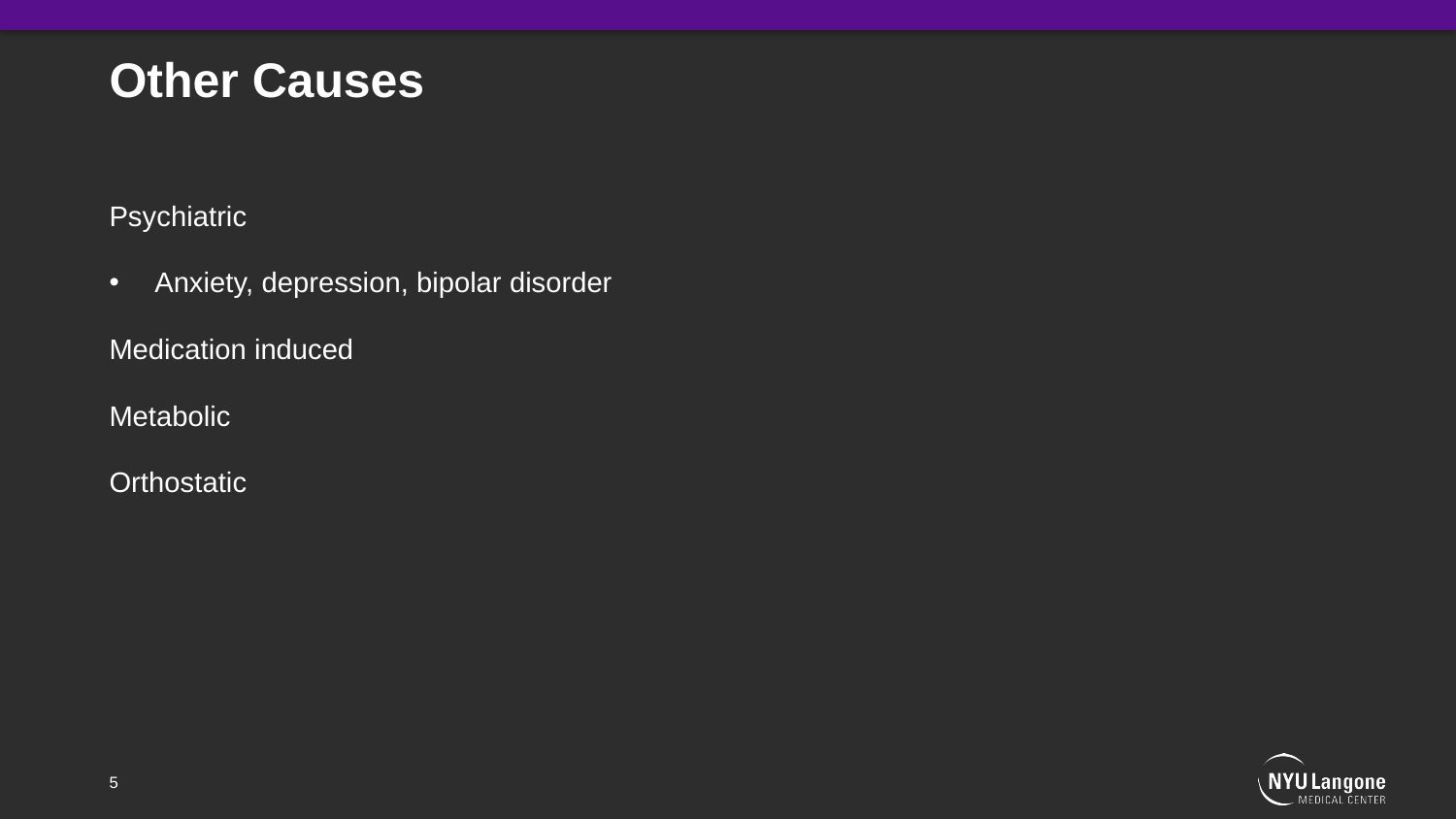

# Other Causes
Psychiatric
Anxiety, depression, bipolar disorder
Medication induced
Metabolic
Orthostatic
5

## Slide 6
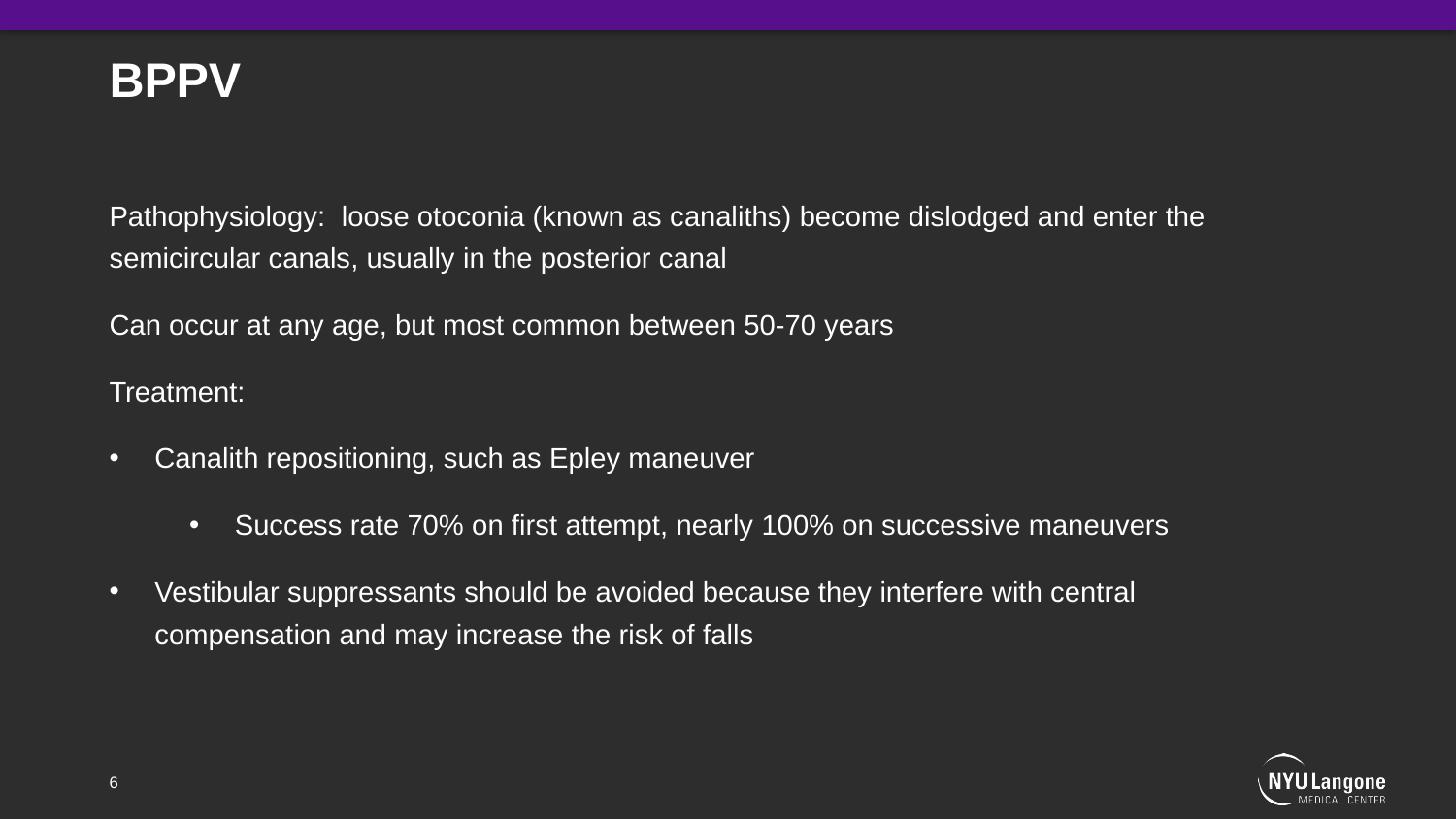

# BPPV
Pathophysiology: loose otoconia (known as canaliths) become dislodged and enter the semicircular canals, usually in the posterior canal
Can occur at any age, but most common between 50-70 years
Treatment:
Canalith repositioning, such as Epley maneuver
Success rate 70% on first attempt, nearly 100% on successive maneuvers
Vestibular suppressants should be avoided because they interfere with central compensation and may increase the risk of falls
6

## Slide 7
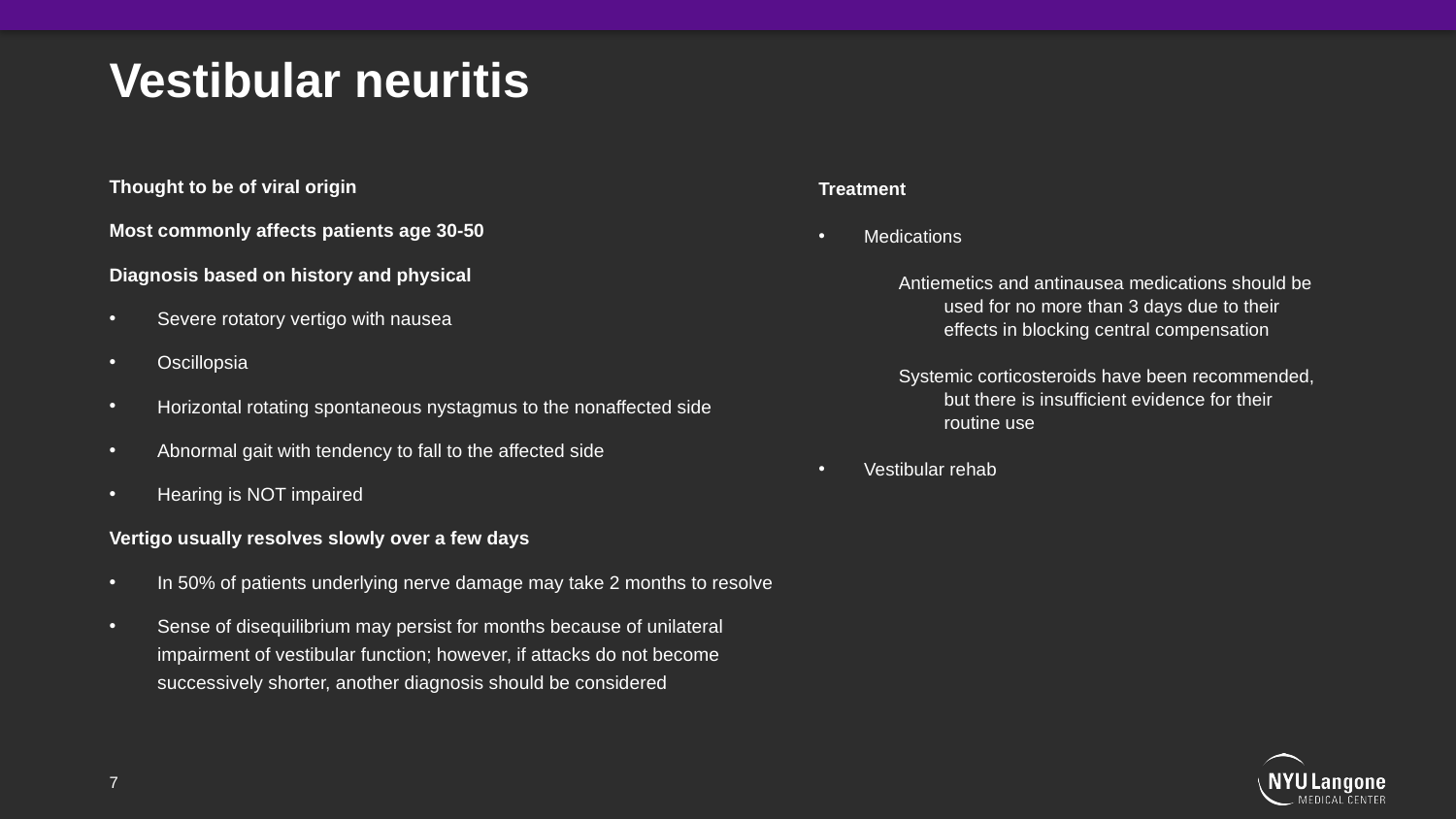

# Vestibular neuritis
Thought to be of viral origin
Most commonly affects patients age 30-50
Diagnosis based on history and physical
Severe rotatory vertigo with nausea
Oscillopsia
Horizontal rotating spontaneous nystagmus to the nonaffected side
Abnormal gait with tendency to fall to the affected side
Hearing is NOT impaired
Vertigo usually resolves slowly over a few days
In 50% of patients underlying nerve damage may take 2 months to resolve
Sense of disequilibrium may persist for months because of unilateral impairment of vestibular function; however, if attacks do not become successively shorter, another diagnosis should be considered
Treatment
Medications
Antiemetics and antinausea medications should be used for no more than 3 days due to their effects in blocking central compensation
Systemic corticosteroids have been recommended, but there is insufficient evidence for their routine use
Vestibular rehab
7

## Slide 8
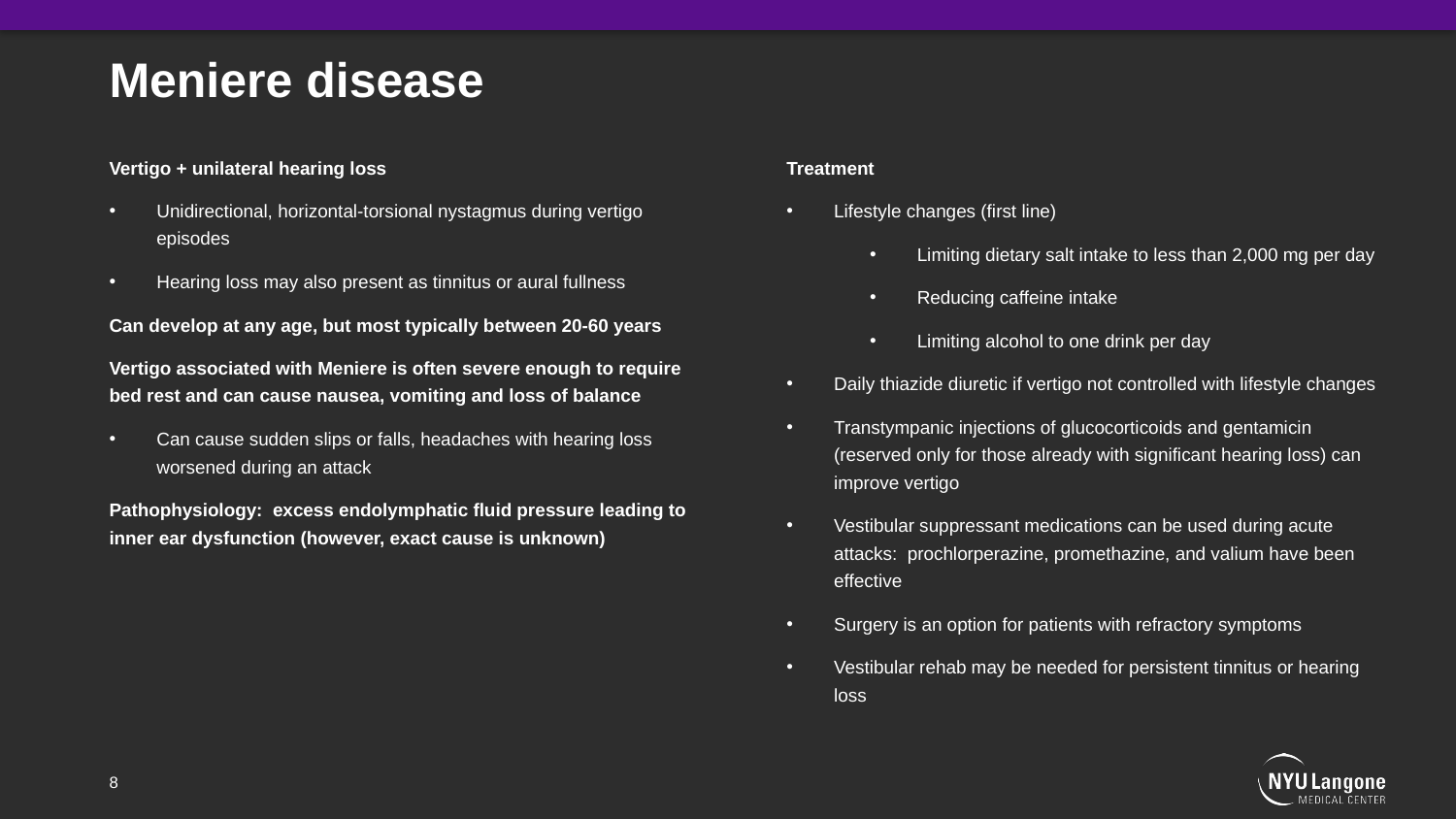

# Meniere disease
Vertigo + unilateral hearing loss
Unidirectional, horizontal-torsional nystagmus during vertigo episodes
Hearing loss may also present as tinnitus or aural fullness
Can develop at any age, but most typically between 20-60 years
Vertigo associated with Meniere is often severe enough to require bed rest and can cause nausea, vomiting and loss of balance
Can cause sudden slips or falls, headaches with hearing loss worsened during an attack
Pathophysiology: excess endolymphatic fluid pressure leading to inner ear dysfunction (however, exact cause is unknown)
Treatment
Lifestyle changes (first line)
Limiting dietary salt intake to less than 2,000 mg per day
Reducing caffeine intake
Limiting alcohol to one drink per day
Daily thiazide diuretic if vertigo not controlled with lifestyle changes
Transtympanic injections of glucocorticoids and gentamicin (reserved only for those already with significant hearing loss) can improve vertigo
Vestibular suppressant medications can be used during acute attacks: prochlorperazine, promethazine, and valium have been effective
Surgery is an option for patients with refractory symptoms
Vestibular rehab may be needed for persistent tinnitus or hearing loss
8

## Slide 9
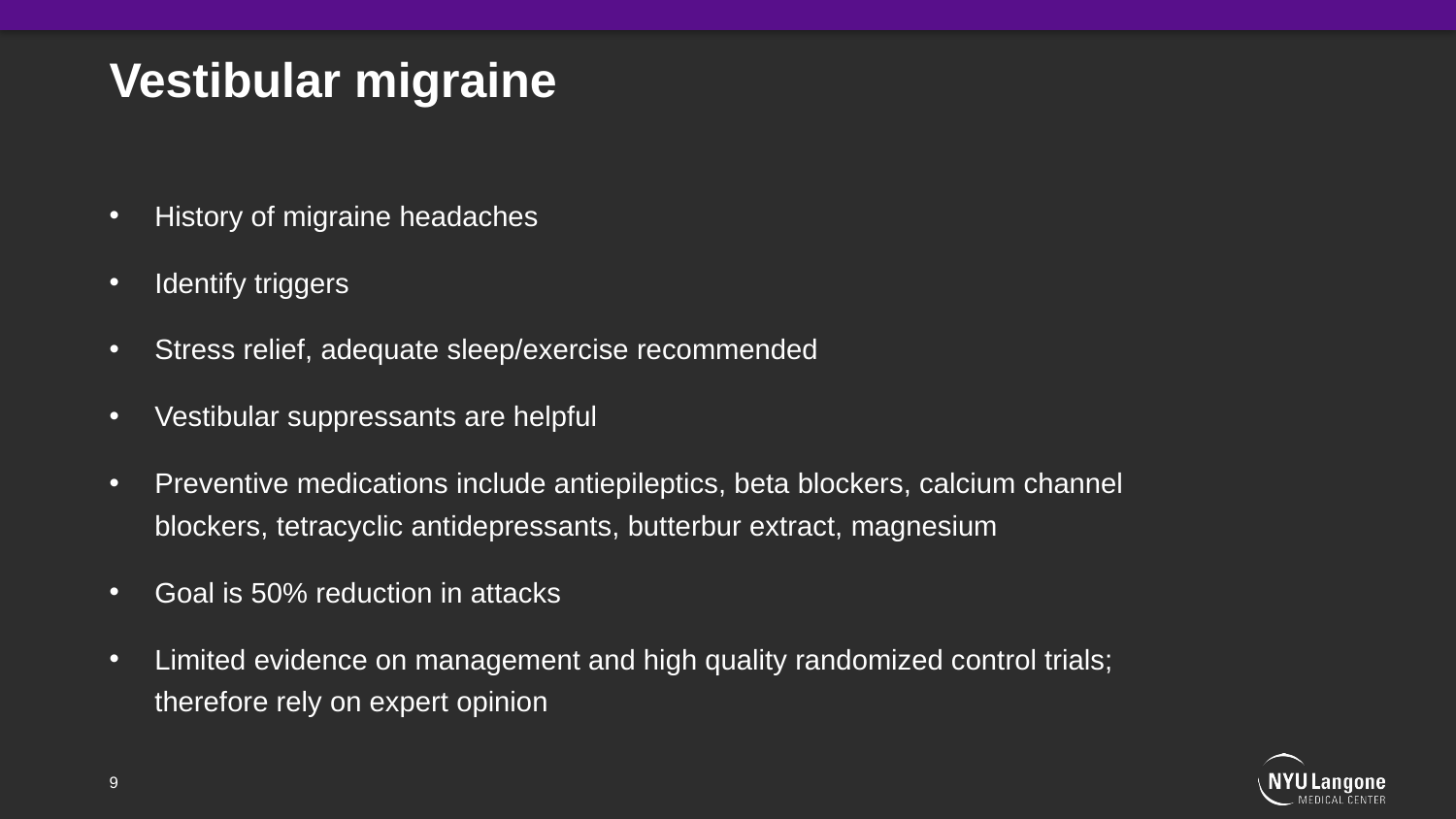

# Vestibular migraine
History of migraine headaches
Identify triggers
Stress relief, adequate sleep/exercise recommended
Vestibular suppressants are helpful
Preventive medications include antiepileptics, beta blockers, calcium channel blockers, tetracyclic antidepressants, butterbur extract, magnesium
Goal is 50% reduction in attacks
Limited evidence on management and high quality randomized control trials; therefore rely on expert opinion
9

## Slide 10
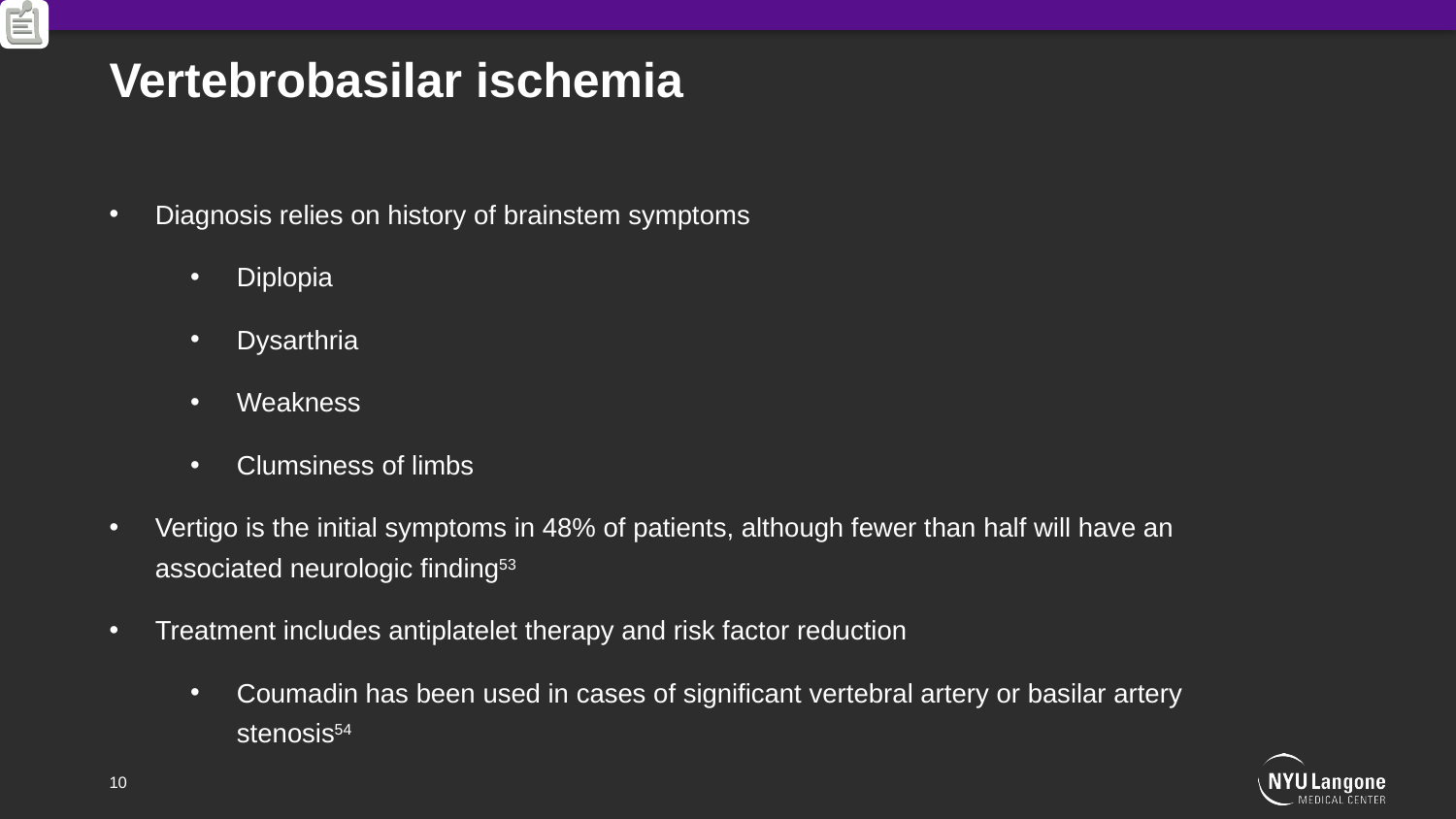

# Vertebrobasilar ischemia
Diagnosis relies on history of brainstem symptoms
Diplopia
Dysarthria
Weakness
Clumsiness of limbs
Vertigo is the initial symptoms in 48% of patients, although fewer than half will have an associated neurologic finding53
Treatment includes antiplatelet therapy and risk factor reduction
Coumadin has been used in cases of significant vertebral artery or basilar artery stenosis54
10

## Slide 11
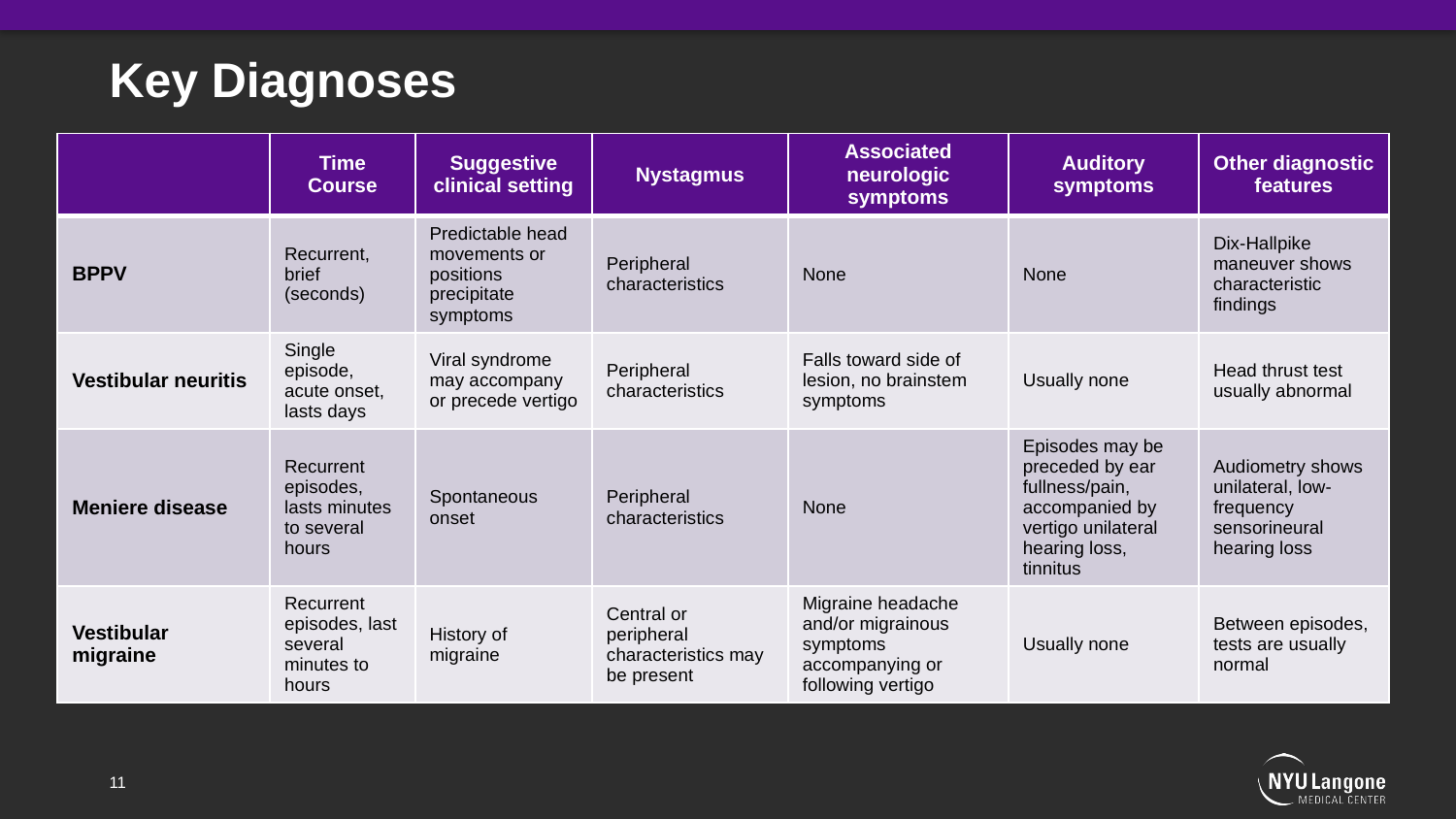

# Key Diagnoses
| | Time Course | Suggestive clinical setting | Nystagmus | Associated neurologic symptoms | Auditory symptoms | Other diagnostic features |
| --- | --- | --- | --- | --- | --- | --- |
| BPPV | Recurrent, brief (seconds) | Predictable head movements or positions precipitate symptoms | Peripheral characteristics | None | None | Dix-Hallpike maneuver shows characteristic findings |
| Vestibular neuritis | Single episode, acute onset, lasts days | Viral syndrome may accompany or precede vertigo | Peripheral characteristics | Falls toward side of lesion, no brainstem symptoms | Usually none | Head thrust test usually abnormal |
| Meniere disease | Recurrent episodes, lasts minutes to several hours | Spontaneous onset | Peripheral characteristics | None | Episodes may be preceded by ear fullness/pain, accompanied by vertigo unilateral hearing loss, tinnitus | Audiometry shows unilateral, low-frequency sensorineural hearing loss |
| Vestibular migraine | Recurrent episodes, last several minutes to hours | History of migraine | Central or peripheral characteristics may be present | Migraine headache and/or migrainous symptoms accompanying or following vertigo | Usually none | Between episodes, tests are usually normal |
11

## Slide 12
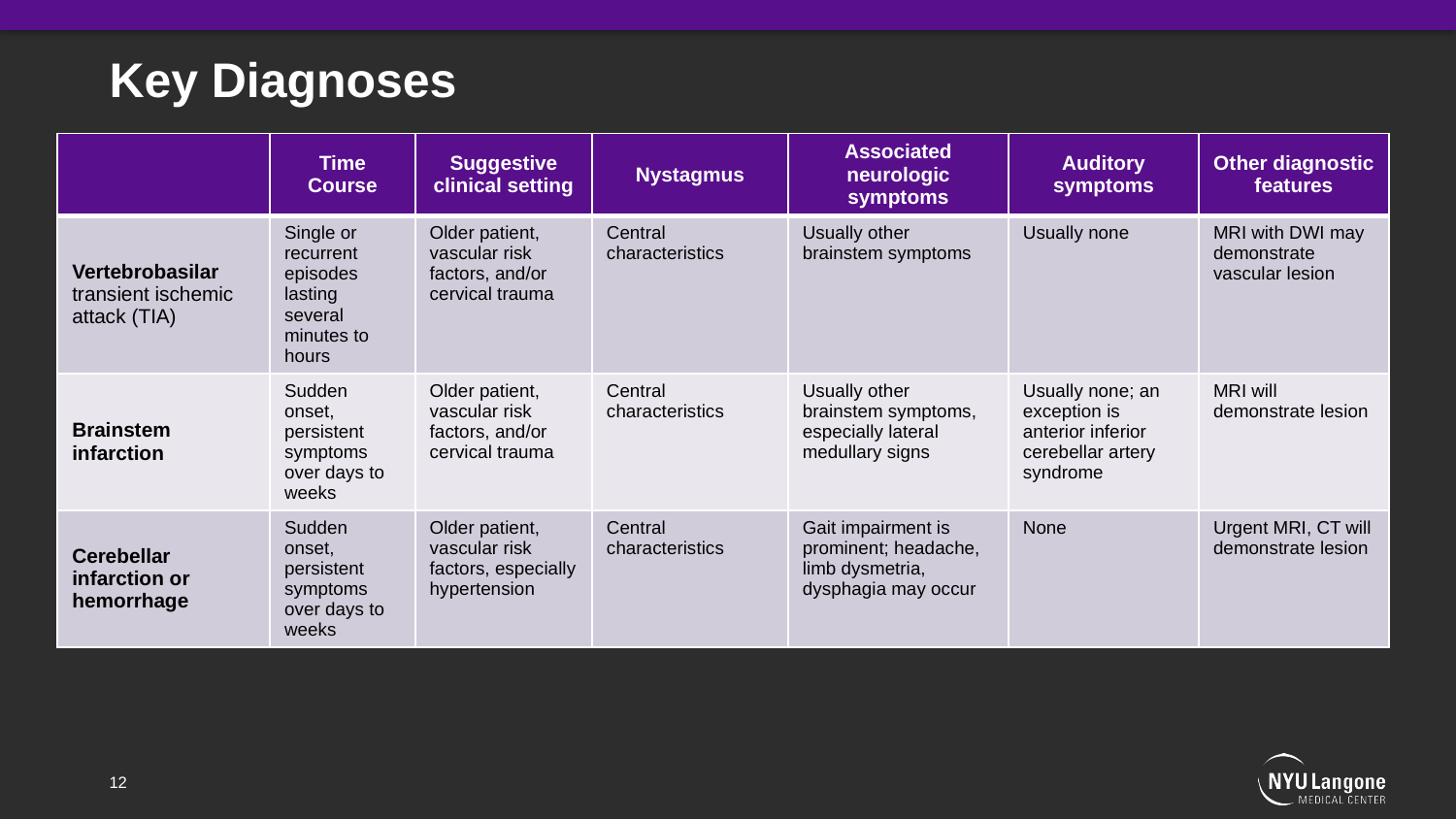

# Key Diagnoses
| | Time Course | Suggestive clinical setting | Nystagmus | Associated neurologic symptoms | Auditory symptoms | Other diagnostic features |
| --- | --- | --- | --- | --- | --- | --- |
| Vertebrobasilar transient ischemic attack (TIA) | Single or recurrent episodes lasting several minutes to hours | Older patient, vascular risk factors, and/or cervical trauma | Central characteristics | Usually other brainstem symptoms | Usually none | MRI with DWI may demonstrate vascular lesion |
| Brainstem infarction | Sudden onset, persistent symptoms over days to weeks | Older patient, vascular risk factors, and/or cervical trauma | Central characteristics | Usually other brainstem symptoms, especially lateral medullary signs | Usually none; an exception is anterior inferior cerebellar artery syndrome | MRI will demonstrate lesion |
| Cerebellar infarction or hemorrhage | Sudden onset, persistent symptoms over days to weeks | Older patient, vascular risk factors, especially hypertension | Central characteristics | Gait impairment is prominent; headache, limb dysmetria, dysphagia may occur | None | Urgent MRI, CT will demonstrate lesion |
12

## Slide 13
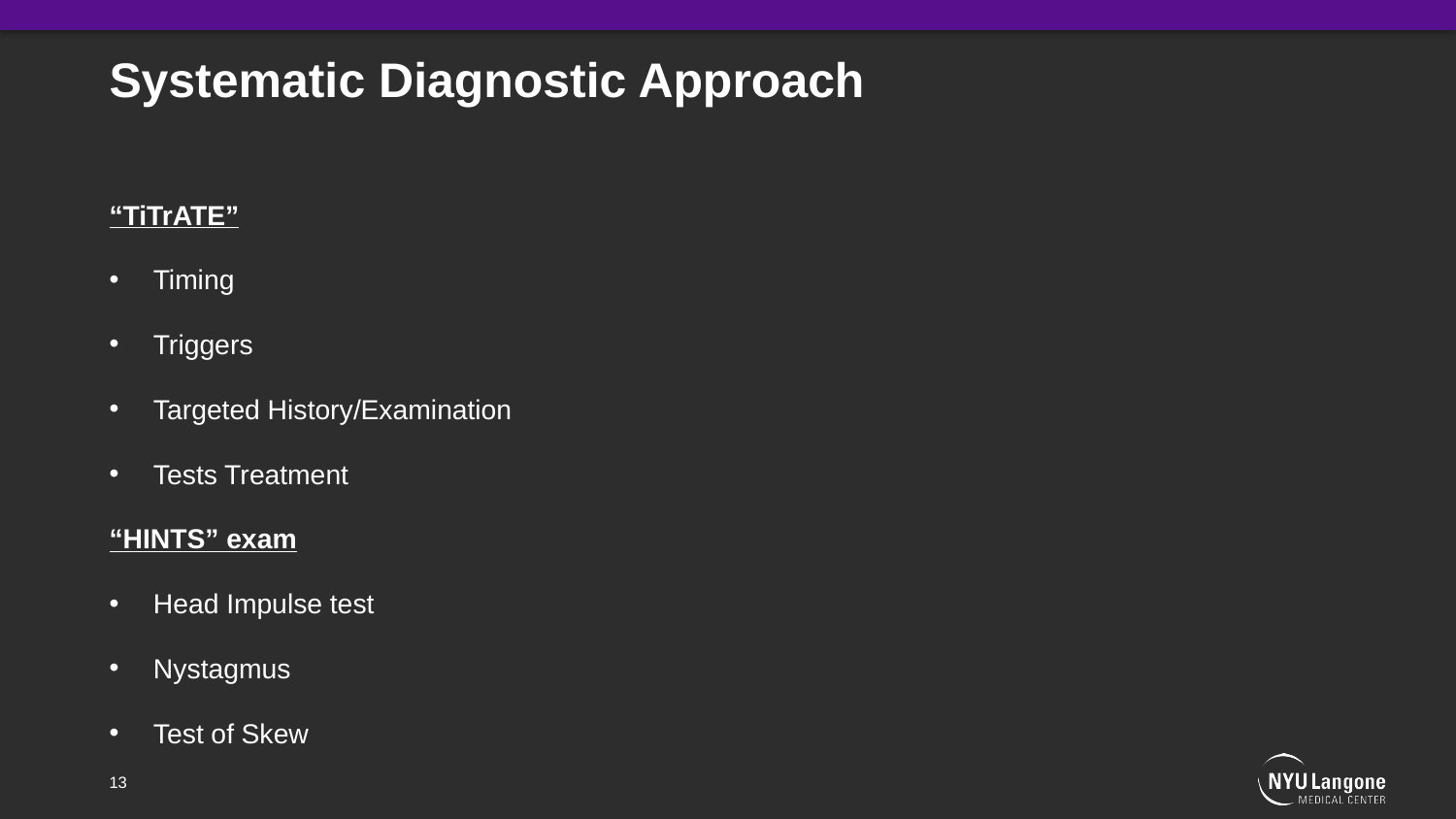

# Systematic Diagnostic Approach
“TiTrATE”
Timing
Triggers
Targeted History/Examination
Tests Treatment
“HINTS” exam
Head Impulse test
Nystagmus
Test of Skew
13

## Slide 14
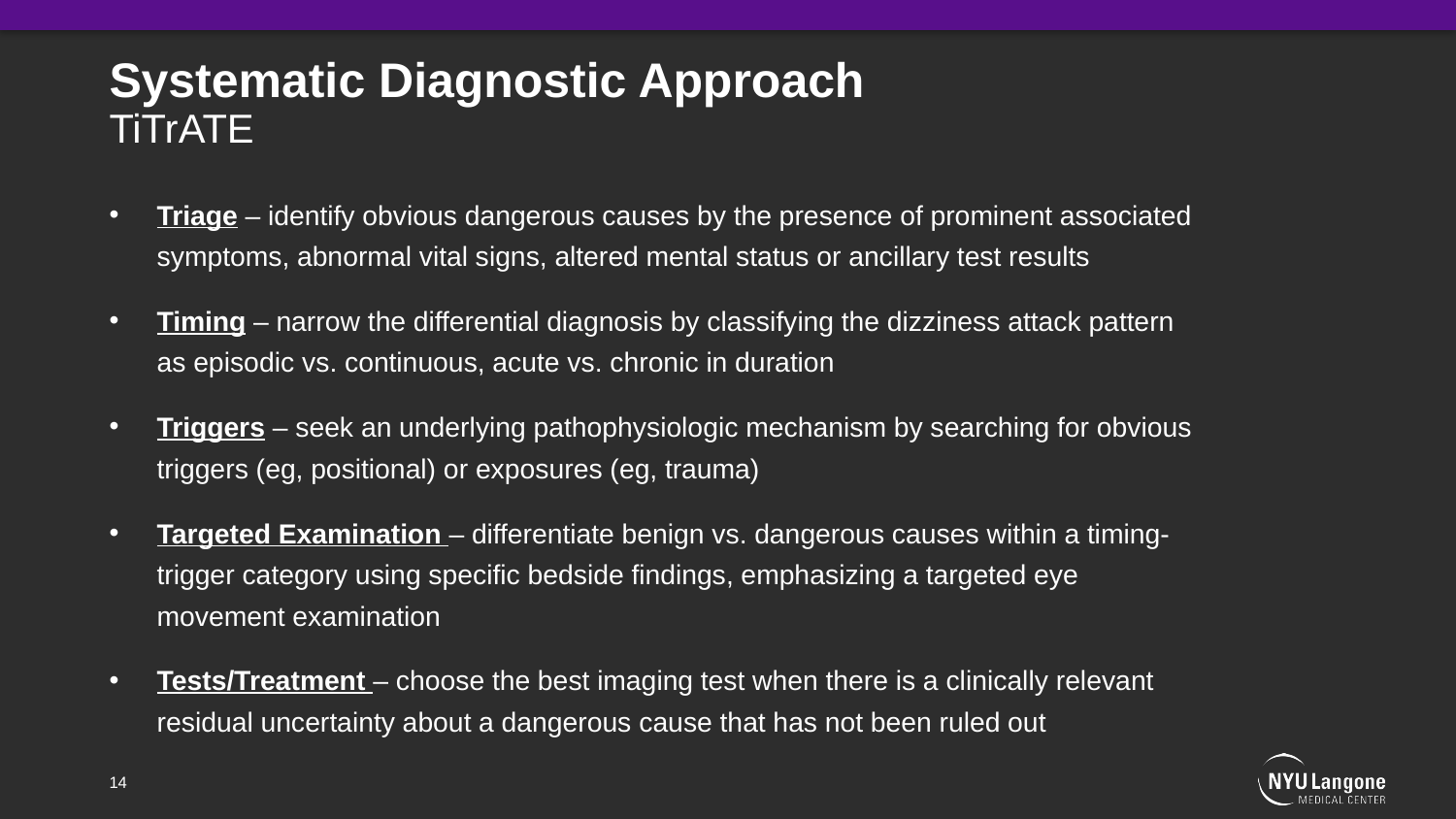

# Systematic Diagnostic Approach TiTrATE
Triage – identify obvious dangerous causes by the presence of prominent associated symptoms, abnormal vital signs, altered mental status or ancillary test results
Timing – narrow the differential diagnosis by classifying the dizziness attack pattern as episodic vs. continuous, acute vs. chronic in duration
Triggers – seek an underlying pathophysiologic mechanism by searching for obvious triggers (eg, positional) or exposures (eg, trauma)
Targeted Examination – differentiate benign vs. dangerous causes within a timing-trigger category using specific bedside findings, emphasizing a targeted eye movement examination
Tests/Treatment – choose the best imaging test when there is a clinically relevant residual uncertainty about a dangerous cause that has not been ruled out
14

## Slide 15
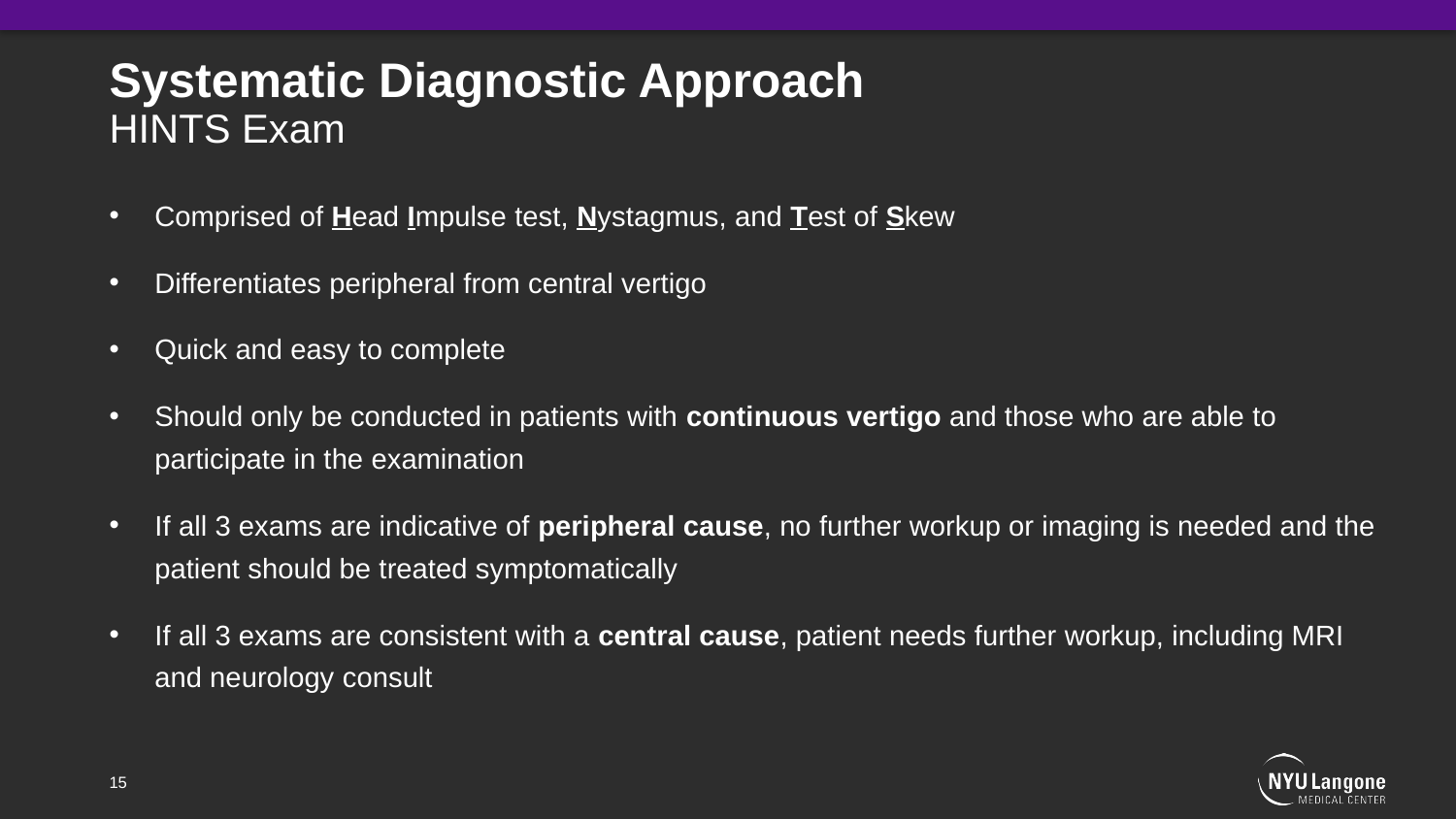

# Systematic Diagnostic Approach HINTS Exam
Comprised of Head Impulse test, Nystagmus, and Test of Skew
Differentiates peripheral from central vertigo
Quick and easy to complete
Should only be conducted in patients with continuous vertigo and those who are able to participate in the examination
If all 3 exams are indicative of peripheral cause, no further workup or imaging is needed and the patient should be treated symptomatically
If all 3 exams are consistent with a central cause, patient needs further workup, including MRI and neurology consult
15

## Slide 16
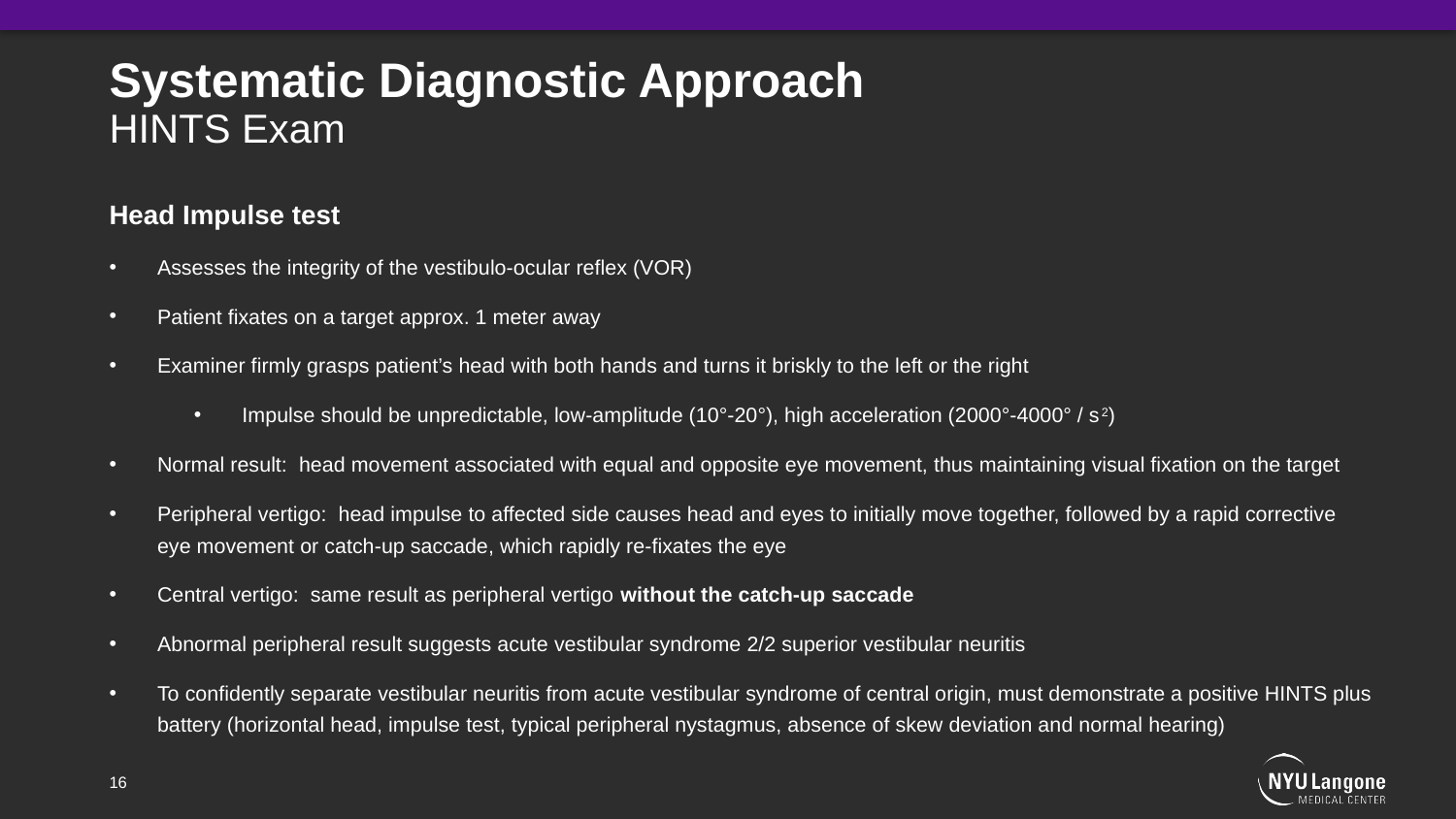

# Systematic Diagnostic Approach HINTS Exam
Head Impulse test
Assesses the integrity of the vestibulo-ocular reflex (VOR)
Patient fixates on a target approx. 1 meter away
Examiner firmly grasps patient’s head with both hands and turns it briskly to the left or the right
Impulse should be unpredictable, low-amplitude (10°-20°), high acceleration (2000°-4000° / s2)
Normal result: head movement associated with equal and opposite eye movement, thus maintaining visual fixation on the target
Peripheral vertigo: head impulse to affected side causes head and eyes to initially move together, followed by a rapid corrective eye movement or catch-up saccade, which rapidly re-fixates the eye
Central vertigo: same result as peripheral vertigo without the catch-up saccade
Abnormal peripheral result suggests acute vestibular syndrome 2/2 superior vestibular neuritis
To confidently separate vestibular neuritis from acute vestibular syndrome of central origin, must demonstrate a positive HINTS plus battery (horizontal head, impulse test, typical peripheral nystagmus, absence of skew deviation and normal hearing)
16

## Slide 17
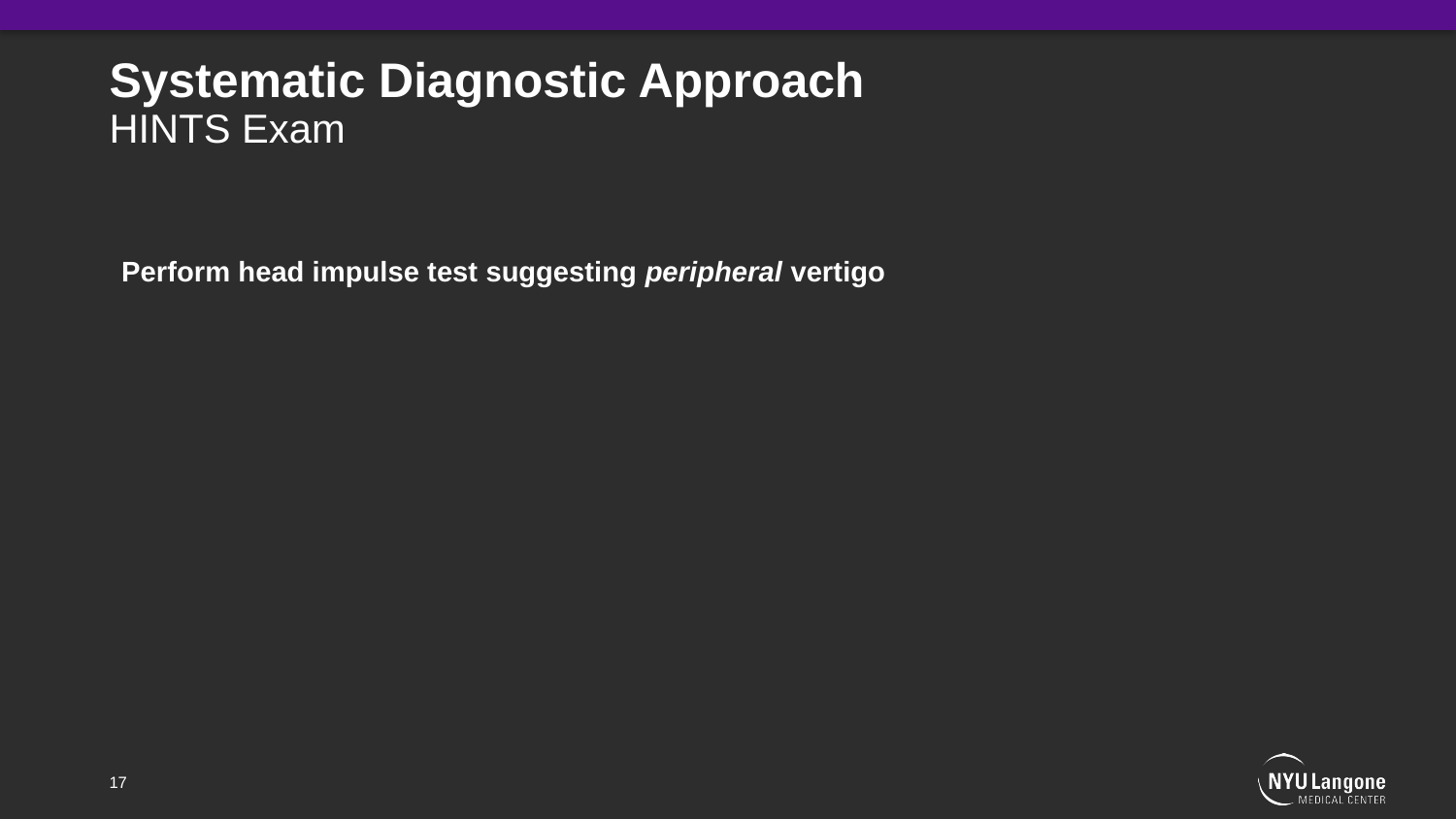

# Systematic Diagnostic Approach HINTS Exam
Perform head impulse test suggesting peripheral vertigo
17

## Slide 18
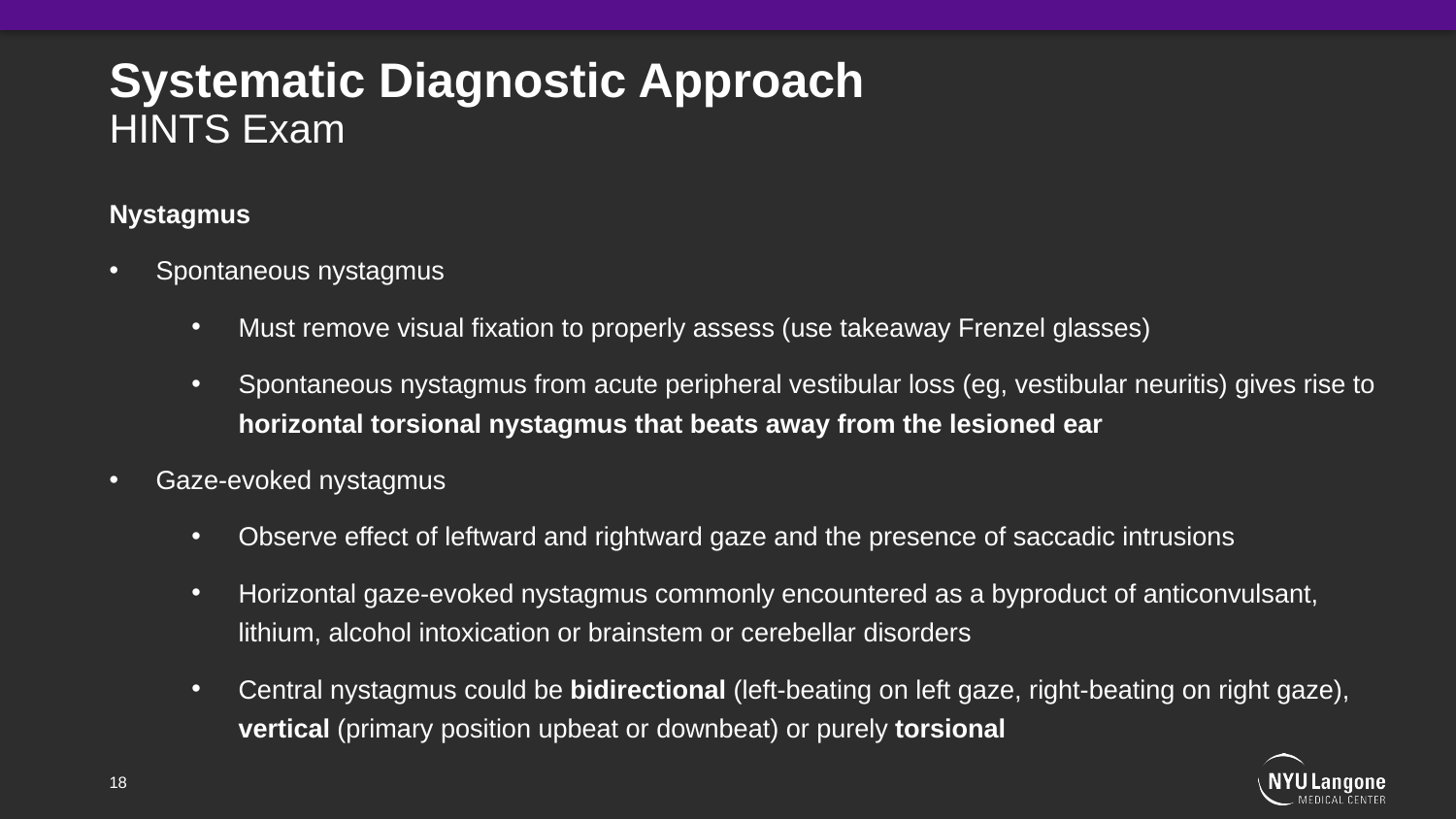

# Systematic Diagnostic Approach HINTS Exam
Nystagmus
Spontaneous nystagmus
Must remove visual fixation to properly assess (use takeaway Frenzel glasses)
Spontaneous nystagmus from acute peripheral vestibular loss (eg, vestibular neuritis) gives rise to horizontal torsional nystagmus that beats away from the lesioned ear
Gaze-evoked nystagmus
Observe effect of leftward and rightward gaze and the presence of saccadic intrusions
Horizontal gaze-evoked nystagmus commonly encountered as a byproduct of anticonvulsant, lithium, alcohol intoxication or brainstem or cerebellar disorders
Central nystagmus could be bidirectional (left-beating on left gaze, right-beating on right gaze), vertical (primary position upbeat or downbeat) or purely torsional
18

## Slide 19
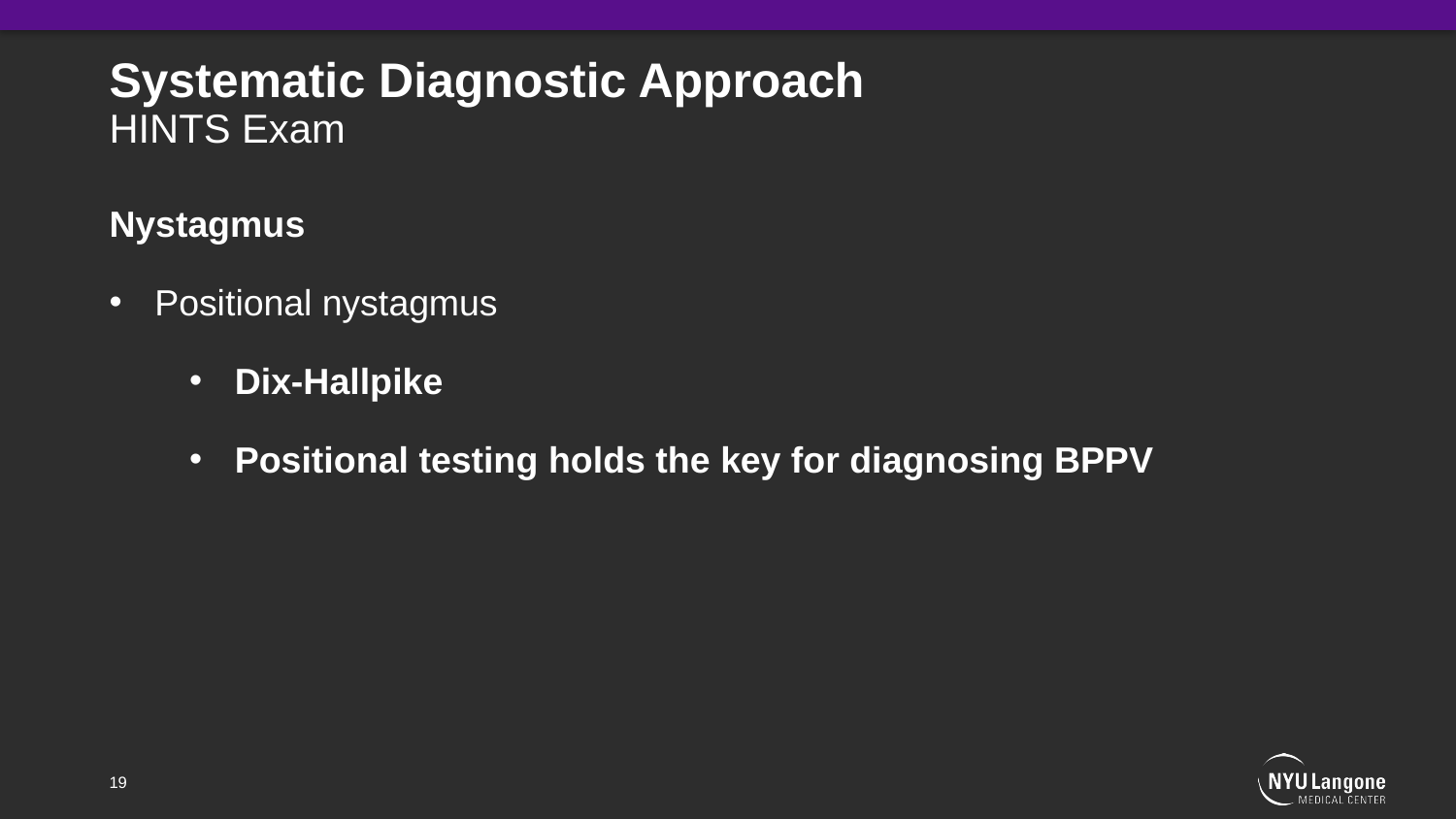

# Systematic Diagnostic Approach HINTS Exam
Nystagmus
Positional nystagmus
Dix-Hallpike
Positional testing holds the key for diagnosing BPPV
19

## Slide 20
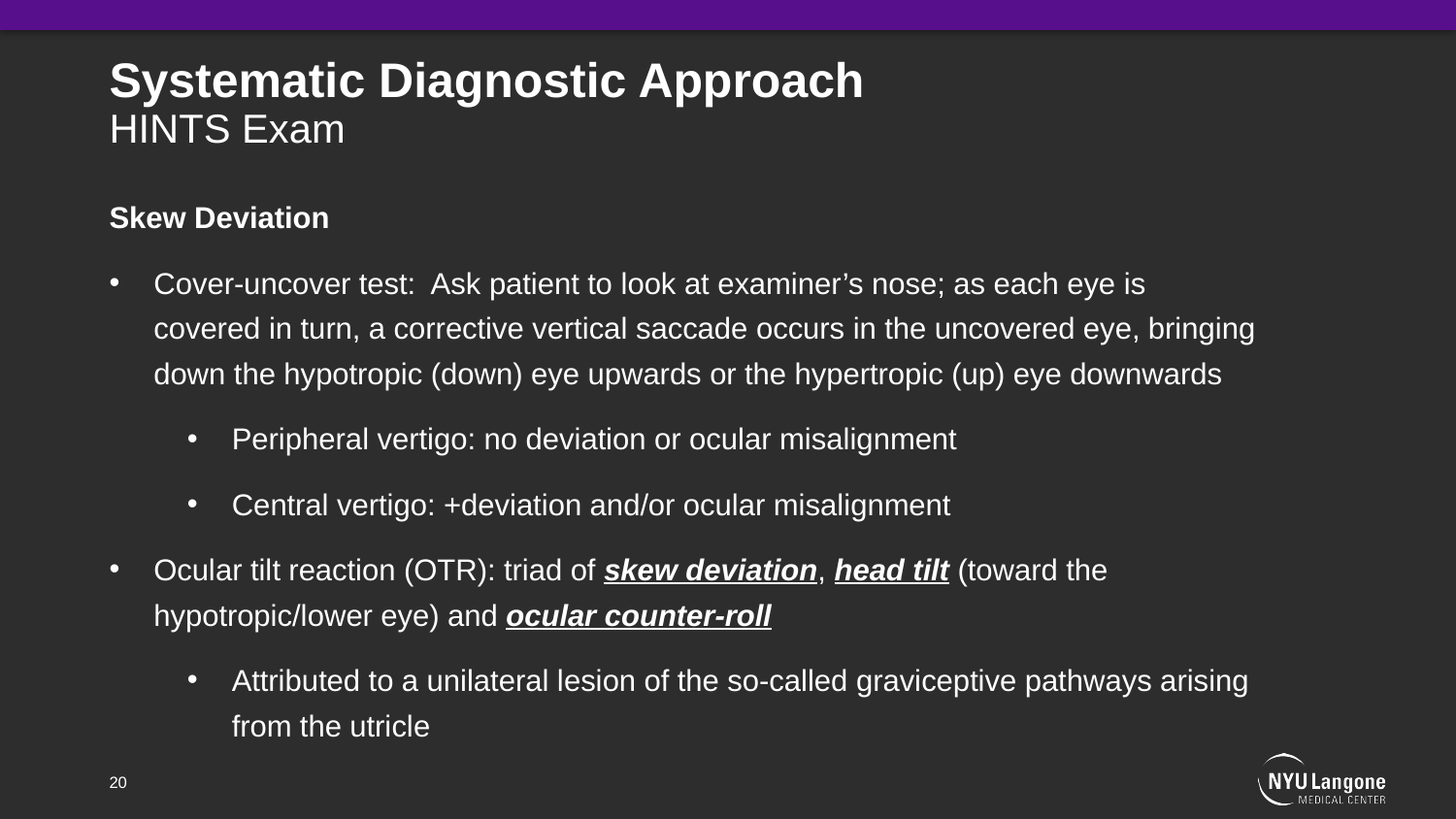

# Systematic Diagnostic Approach HINTS Exam
Skew Deviation
Cover-uncover test: Ask patient to look at examiner’s nose; as each eye is covered in turn, a corrective vertical saccade occurs in the uncovered eye, bringing down the hypotropic (down) eye upwards or the hypertropic (up) eye downwards
Peripheral vertigo: no deviation or ocular misalignment
Central vertigo: +deviation and/or ocular misalignment
Ocular tilt reaction (OTR): triad of skew deviation, head tilt (toward the hypotropic/lower eye) and ocular counter-roll
Attributed to a unilateral lesion of the so-called graviceptive pathways arising from the utricle
20

## Slide 21
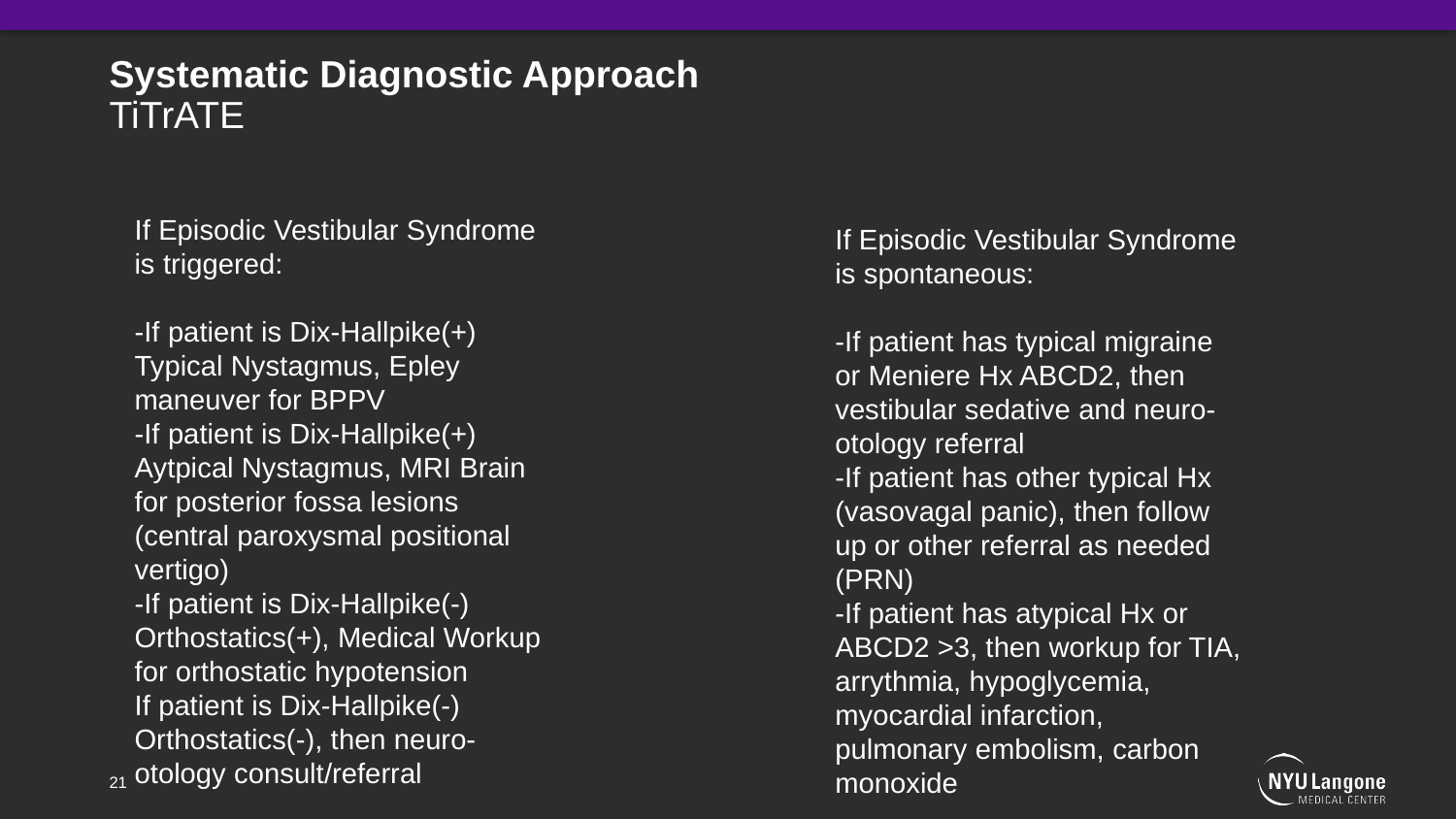

# Systematic Diagnostic Approach TiTrATE
If Episodic Vestibular Syndrome is triggered:
-If patient is Dix-Hallpike(+) Typical Nystagmus, Epley maneuver for BPPV
-If patient is Dix-Hallpike(+) Aytpical Nystagmus, MRI Brain for posterior fossa lesions (central paroxysmal positional vertigo)
-If patient is Dix-Hallpike(-) Orthostatics(+), Medical Workup for orthostatic hypotension
If patient is Dix-Hallpike(-) Orthostatics(-), then neuro-otology consult/referral
If Episodic Vestibular Syndrome is spontaneous:
-If patient has typical migraine or Meniere Hx ABCD2, then vestibular sedative and neuro-otology referral
-If patient has other typical Hx  (vasovagal panic), then follow up or other referral as needed (PRN)
-If patient has atypical Hx or ABCD2 >3, then workup for TIA, arrythmia, hypoglycemia, myocardial infarction, pulmonary embolism, carbon monoxide
21

## Slide 22
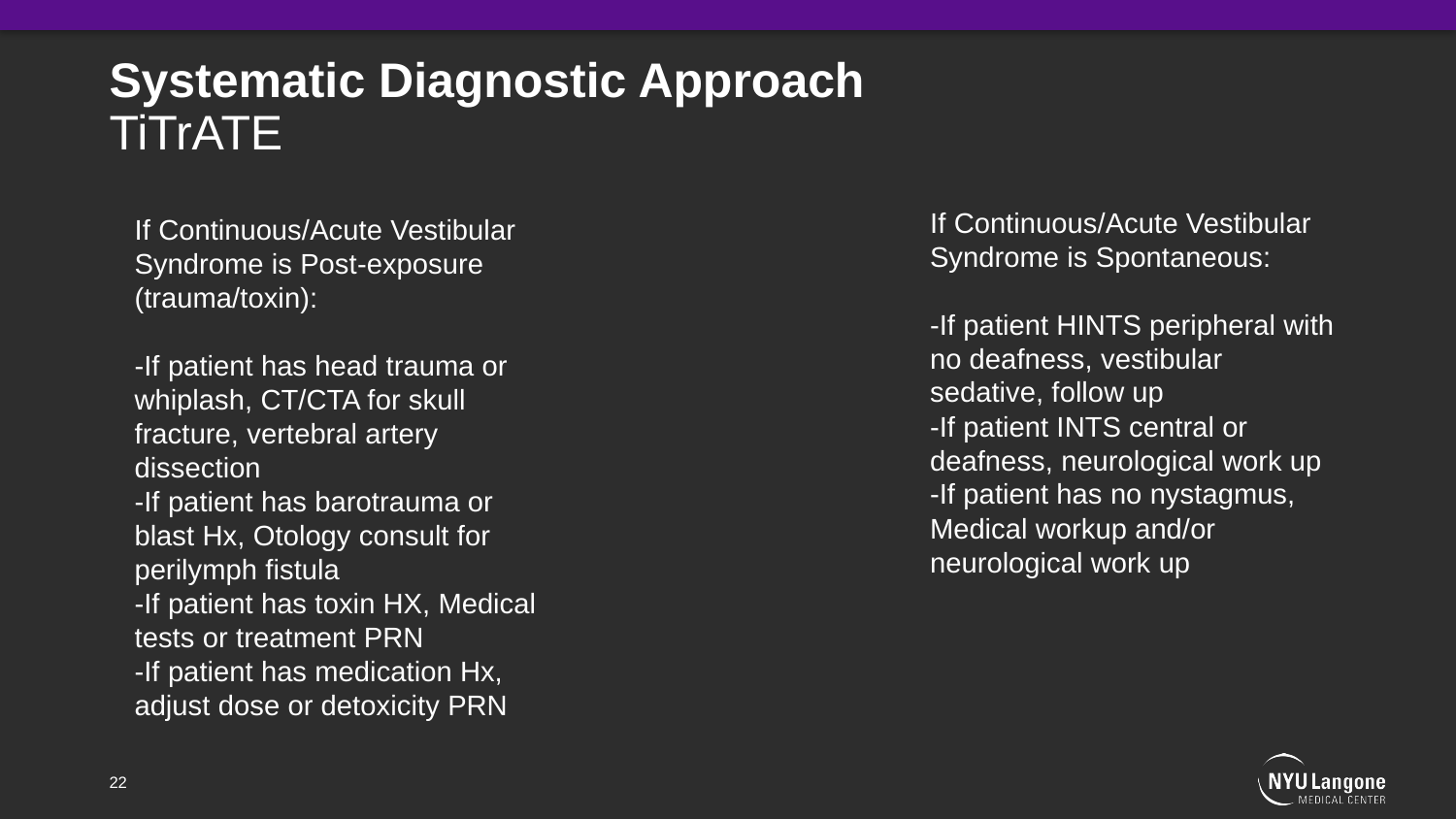

# Systematic Diagnostic Approach TiTrATE
If Continuous/Acute Vestibular Syndrome is Spontaneous:
-If patient HINTS peripheral with no deafness, vestibular sedative, follow up
-If patient INTS central or deafness, neurological work up
-If patient has no nystagmus, Medical workup and/or neurological work up
If Continuous/Acute Vestibular Syndrome is Post-exposure (trauma/toxin):
-If patient has head trauma or whiplash, CT/CTA for skull fracture, vertebral artery dissection
-If patient has barotrauma or blast Hx, Otology consult for perilymph fistula
-If patient has toxin HX, Medical tests or treatment PRN
-If patient has medication Hx, adjust dose or detoxicity PRN
22

## Slide 23
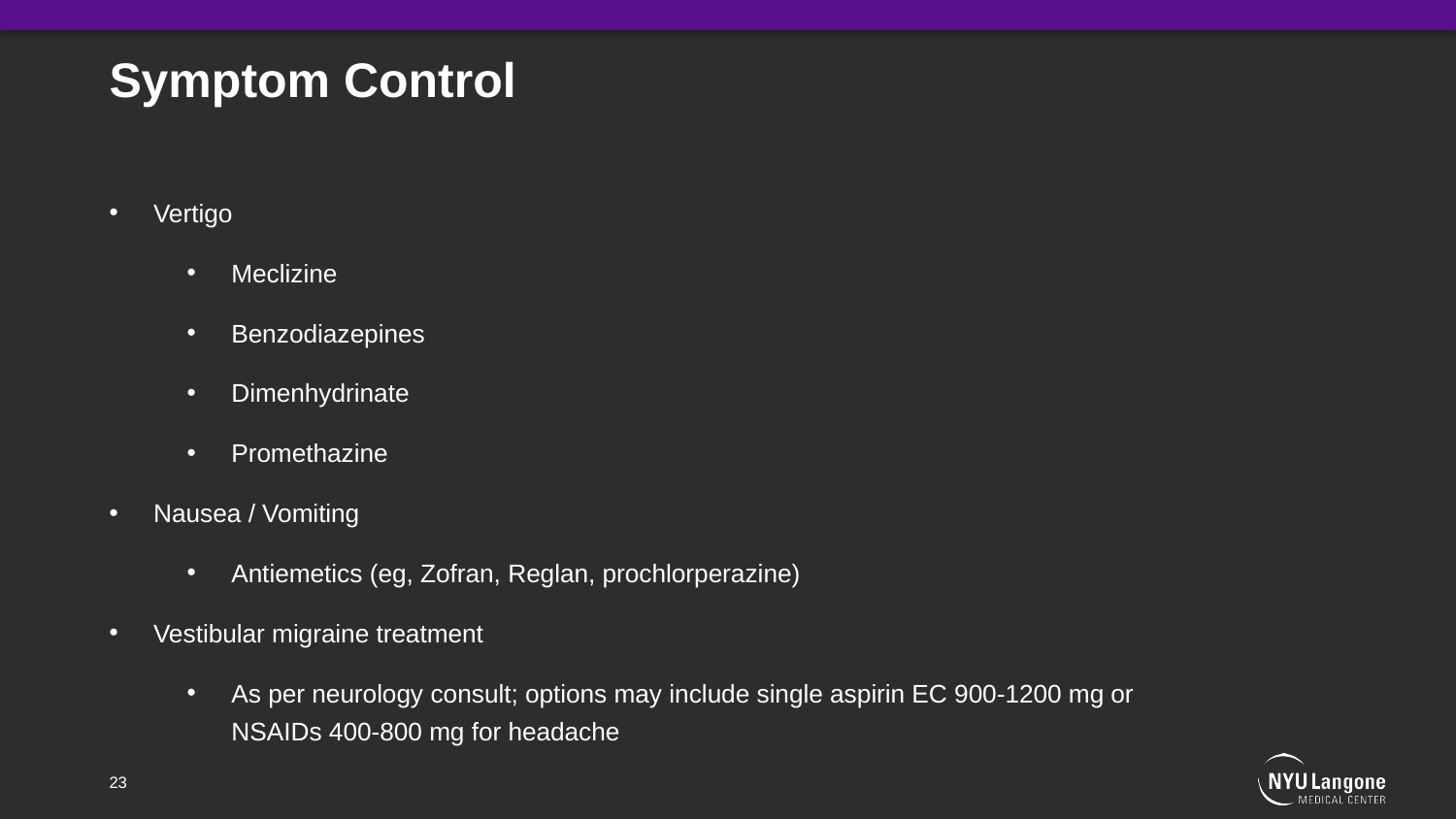

# Symptom Control
Vertigo
Meclizine
Benzodiazepines
Dimenhydrinate
Promethazine
Nausea / Vomiting
Antiemetics (eg, Zofran, Reglan, prochlorperazine)
Vestibular migraine treatment
As per neurology consult; options may include single aspirin EC 900-1200 mg or NSAIDs 400-800 mg for headache
23

## Slide 24
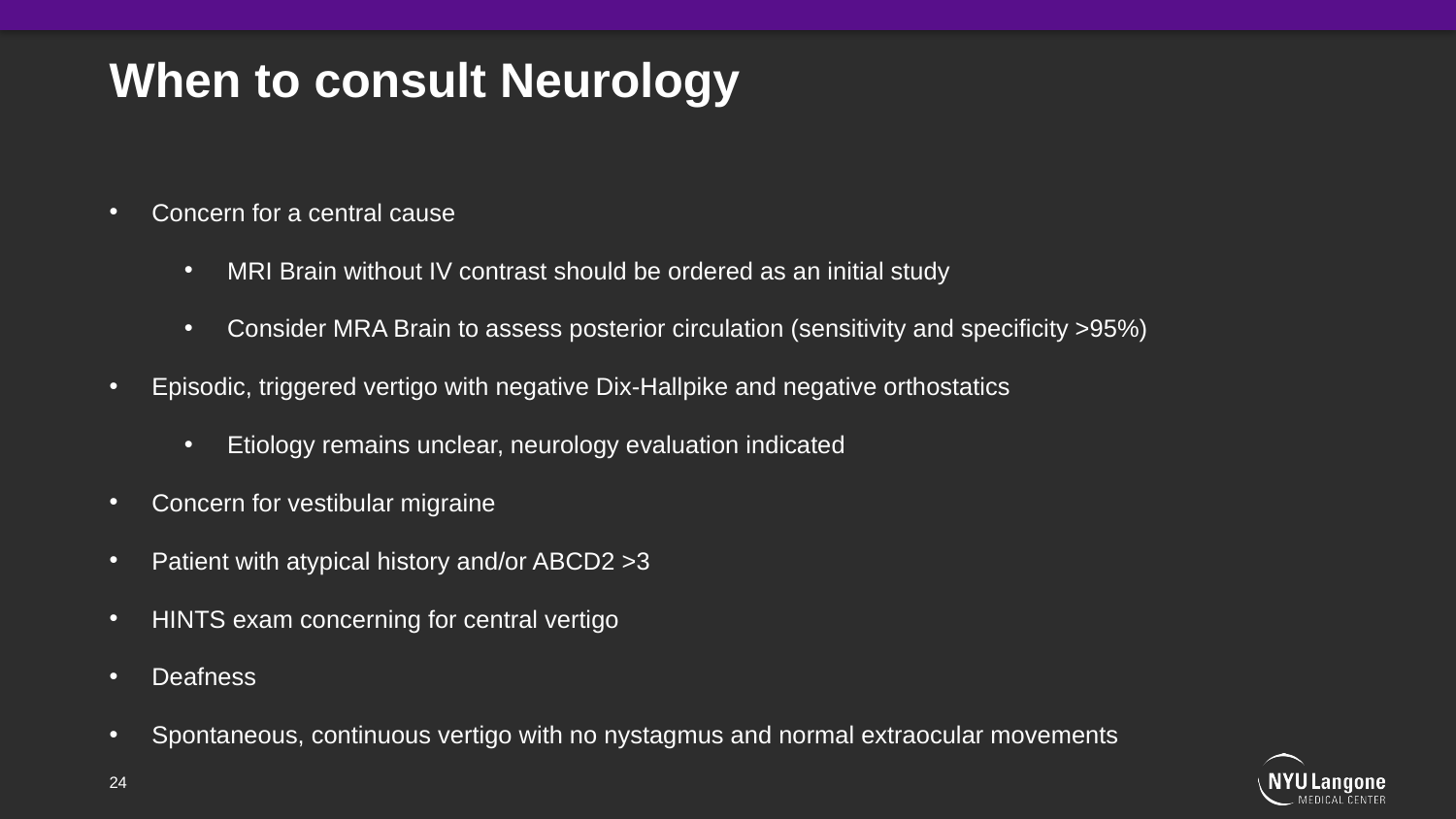

# When to consult Neurology
Concern for a central cause
MRI Brain without IV contrast should be ordered as an initial study
Consider MRA Brain to assess posterior circulation (sensitivity and specificity >95%)
Episodic, triggered vertigo with negative Dix-Hallpike and negative orthostatics
Etiology remains unclear, neurology evaluation indicated
Concern for vestibular migraine
Patient with atypical history and/or ABCD2 >3
HINTS exam concerning for central vertigo
Deafness
Spontaneous, continuous vertigo with no nystagmus and normal extraocular movements
24

## Slide 25
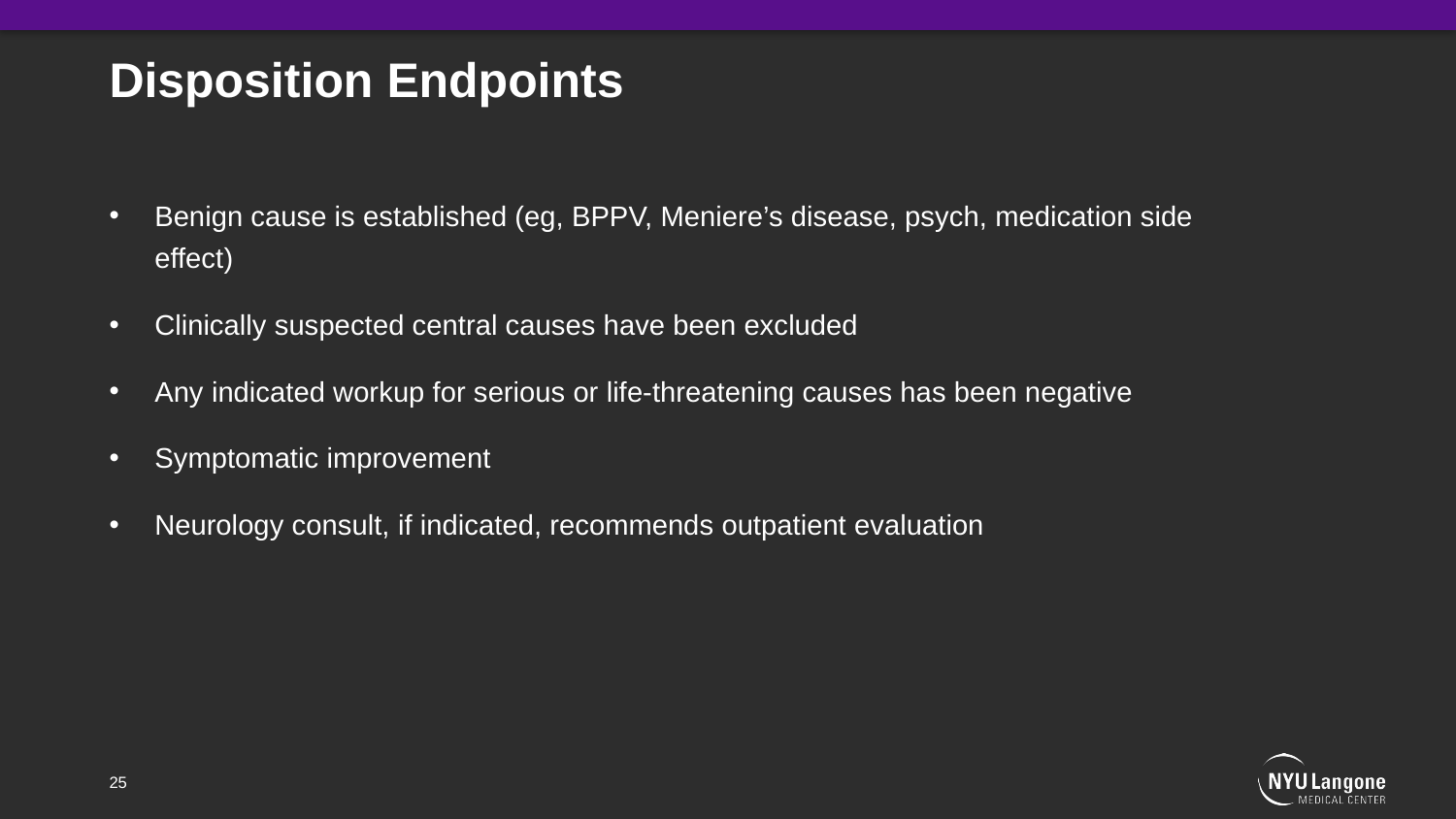

# Disposition Endpoints
Benign cause is established (eg, BPPV, Meniere’s disease, psych, medication side effect)
Clinically suspected central causes have been excluded
Any indicated workup for serious or life-threatening causes has been negative
Symptomatic improvement
Neurology consult, if indicated, recommends outpatient evaluation
25

## Slide 26
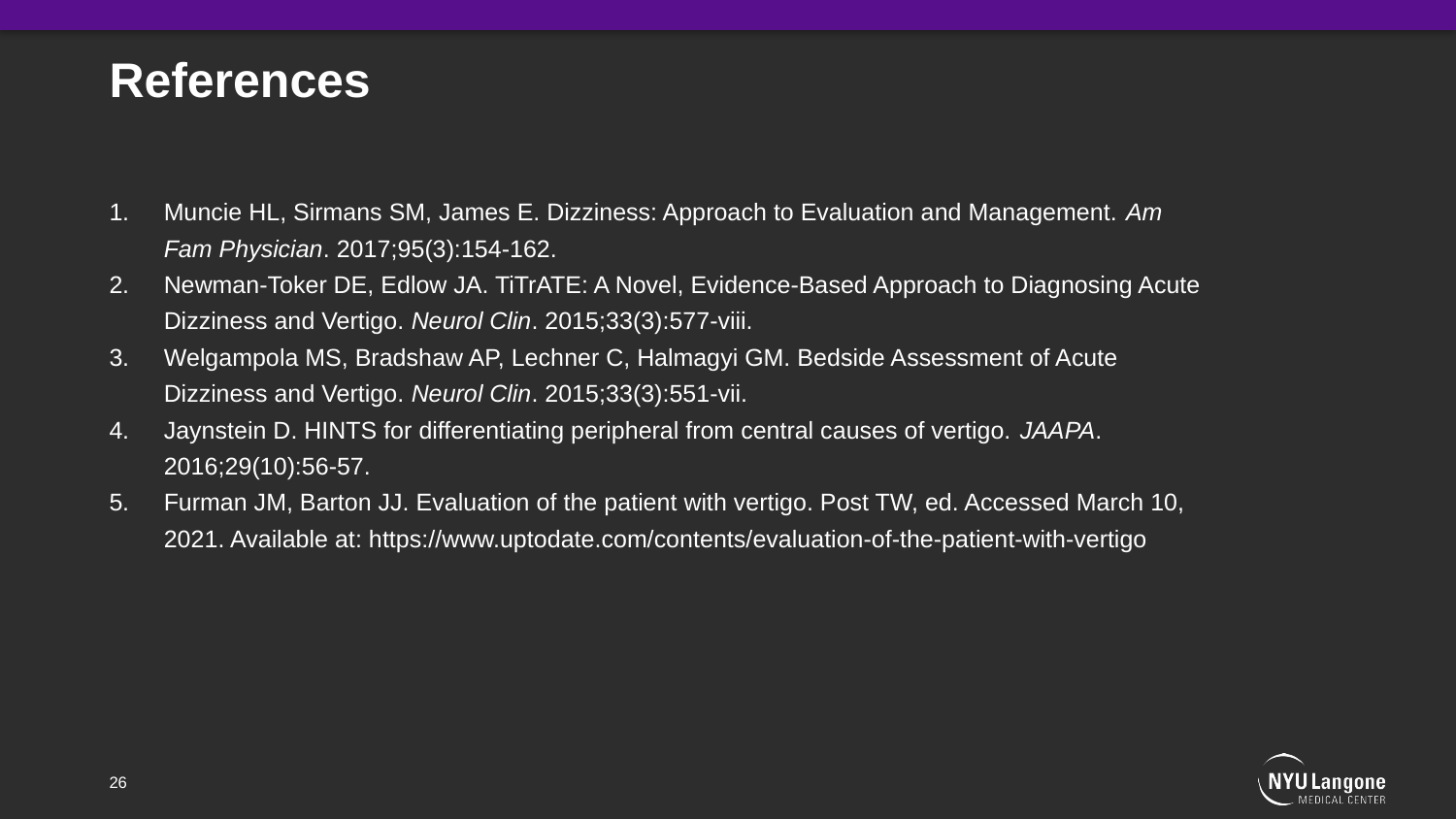

# References
Muncie HL, Sirmans SM, James E. Dizziness: Approach to Evaluation and Management. Am Fam Physician. 2017;95(3):154-162.
Newman-Toker DE, Edlow JA. TiTrATE: A Novel, Evidence-Based Approach to Diagnosing Acute Dizziness and Vertigo. Neurol Clin. 2015;33(3):577-viii.
Welgampola MS, Bradshaw AP, Lechner C, Halmagyi GM. Bedside Assessment of Acute Dizziness and Vertigo. Neurol Clin. 2015;33(3):551-vii.
Jaynstein D. HINTS for differentiating peripheral from central causes of vertigo. JAAPA. 2016;29(10):56-57.
Furman JM, Barton JJ. Evaluation of the patient with vertigo. Post TW, ed. Accessed March 10, 2021. Available at: https://www.uptodate.com/contents/evaluation-of-the-patient-with-vertigo
26
