# Supplementary material for: An Observation Medicine Curriculum for Emergency Medicine Education
Source: J Educ Teach Emerg Med. 2021 Apr 19;6(2):C1–C72. doi: 10.21980/J87P92 (PMC10332786; doi:10.21980/J87P92)
Supplement: Supplementary file 22 — Please see associated PowerPoint file [file jetem-6-2-c1-supp22.pptx]

## Slide 1
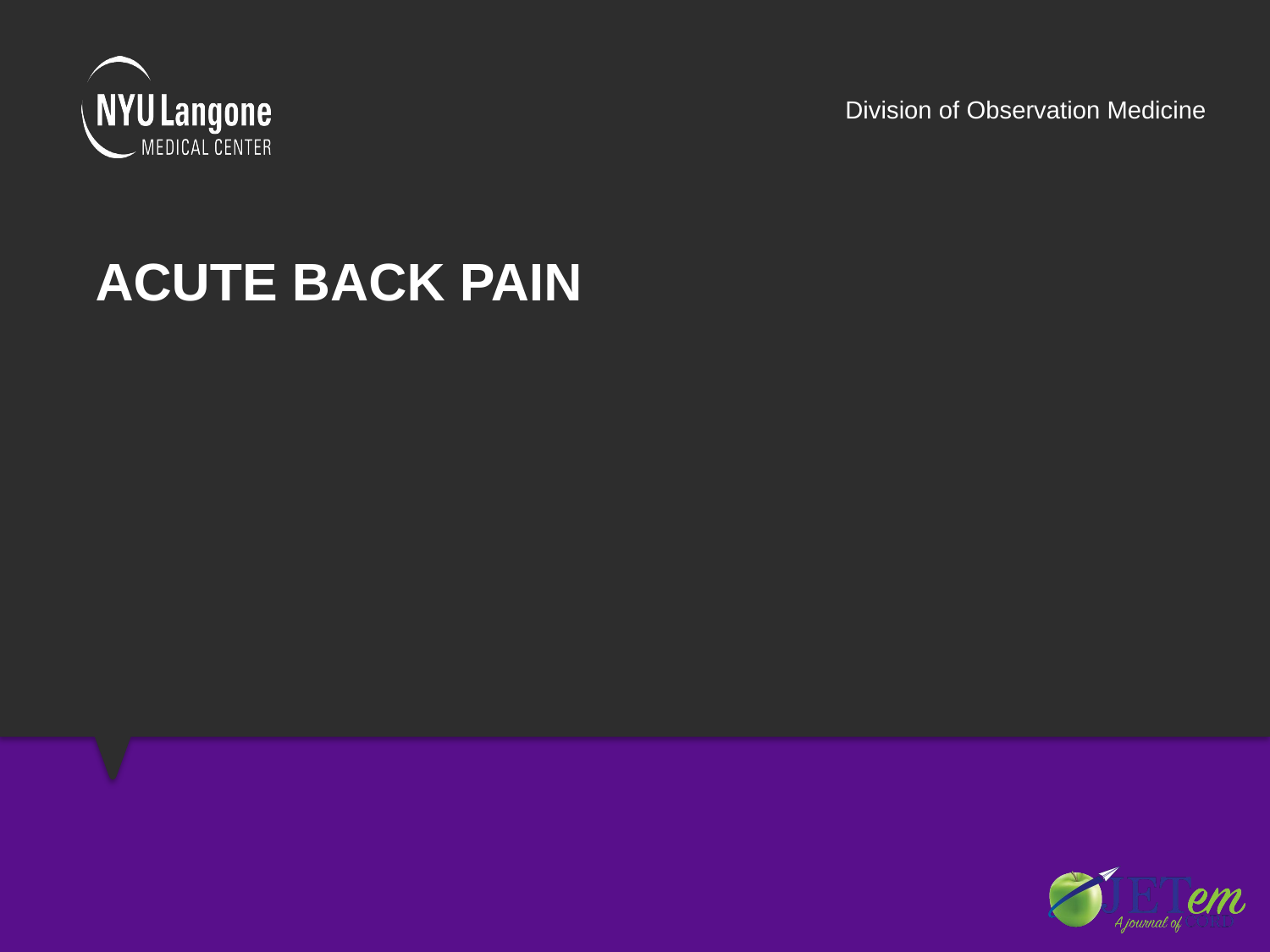

Division of Observation Medicine
# Acute back pain

## Slide 2
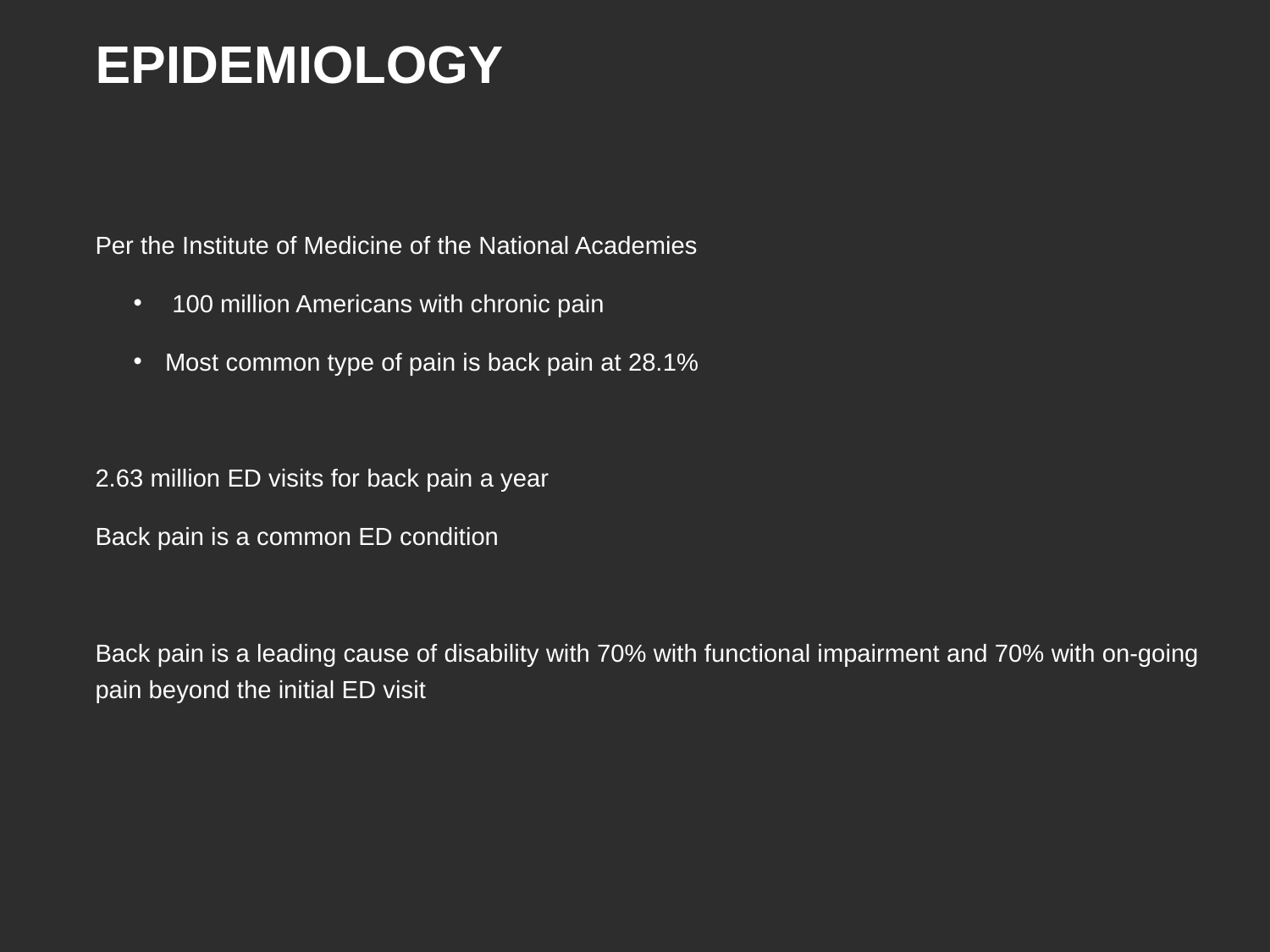

# Epidemiology
Per the Institute of Medicine of the National Academies
 100 million Americans with chronic pain
Most common type of pain is back pain at 28.1%
2.63 million ED visits for back pain a year
Back pain is a common ED condition
Back pain is a leading cause of disability with 70% with functional impairment and 70% with on-going pain beyond the initial ED visit

## Slide 3
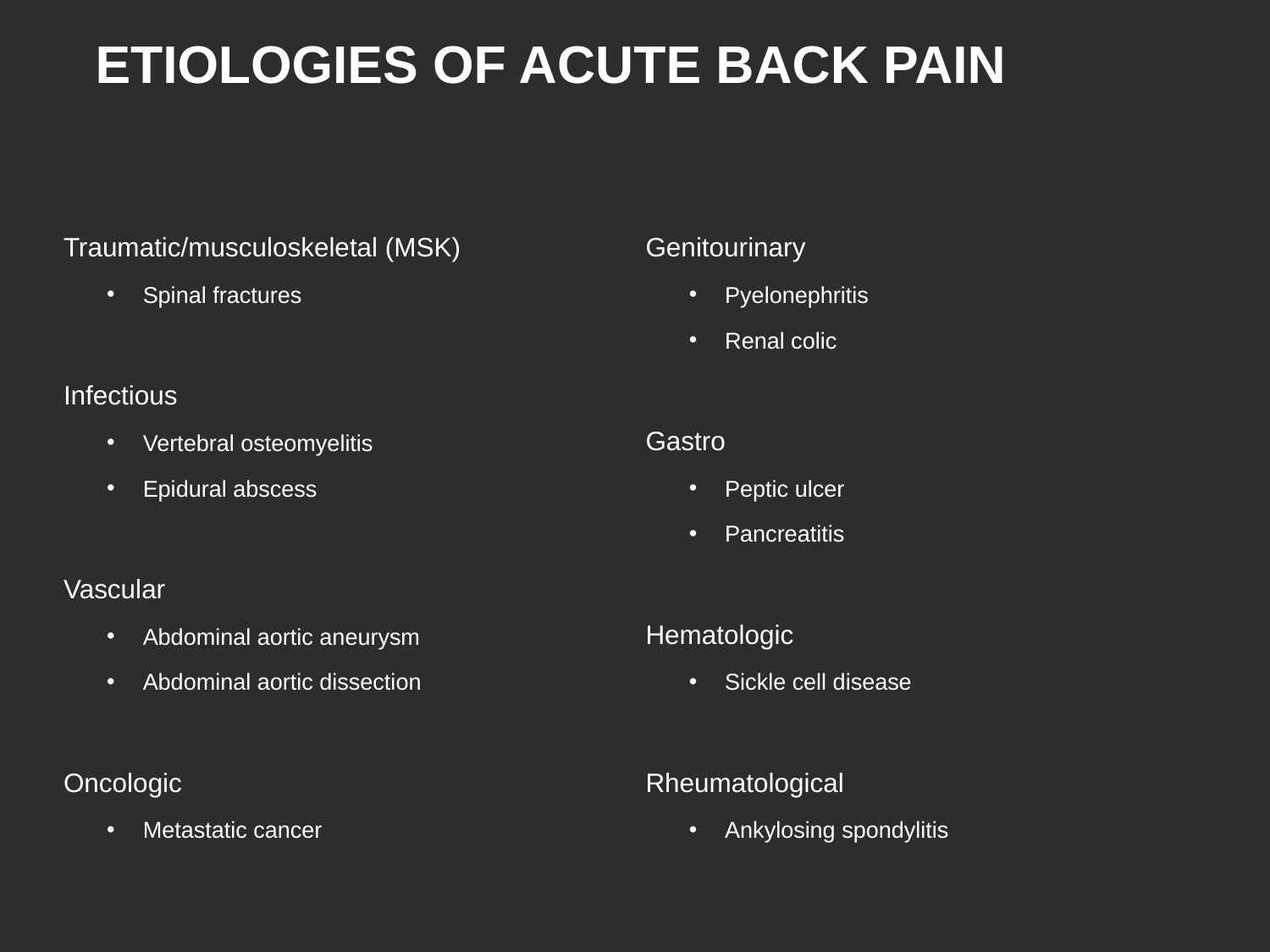

# Etiologies of Acute Back Pain
Traumatic/musculoskeletal (MSK)
Spinal fractures
Infectious
Vertebral osteomyelitis
Epidural abscess
Vascular
Abdominal aortic aneurysm
Abdominal aortic dissection
Oncologic
Metastatic cancer
Genitourinary
Pyelonephritis
Renal colic
Gastro
Peptic ulcer
Pancreatitis
Hematologic
Sickle cell disease
Rheumatological
Ankylosing spondylitis

## Slide 4
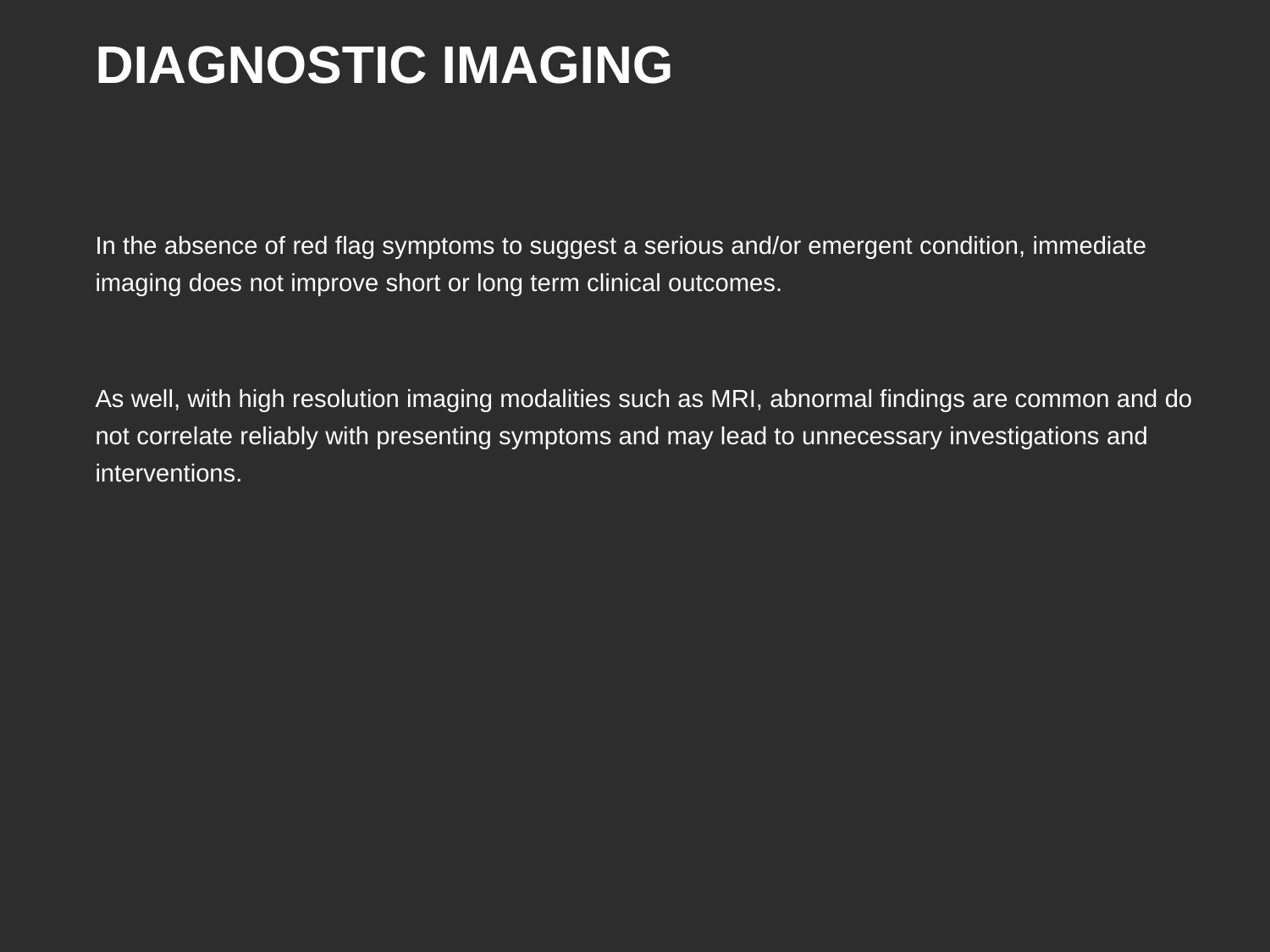

# Diagnostic Imaging
In the absence of red flag symptoms to suggest a serious and/or emergent condition, immediate imaging does not improve short or long term clinical outcomes.
As well, with high resolution imaging modalities such as MRI, abnormal findings are common and do not correlate reliably with presenting symptoms and may lead to unnecessary investigations and interventions.

## Slide 5
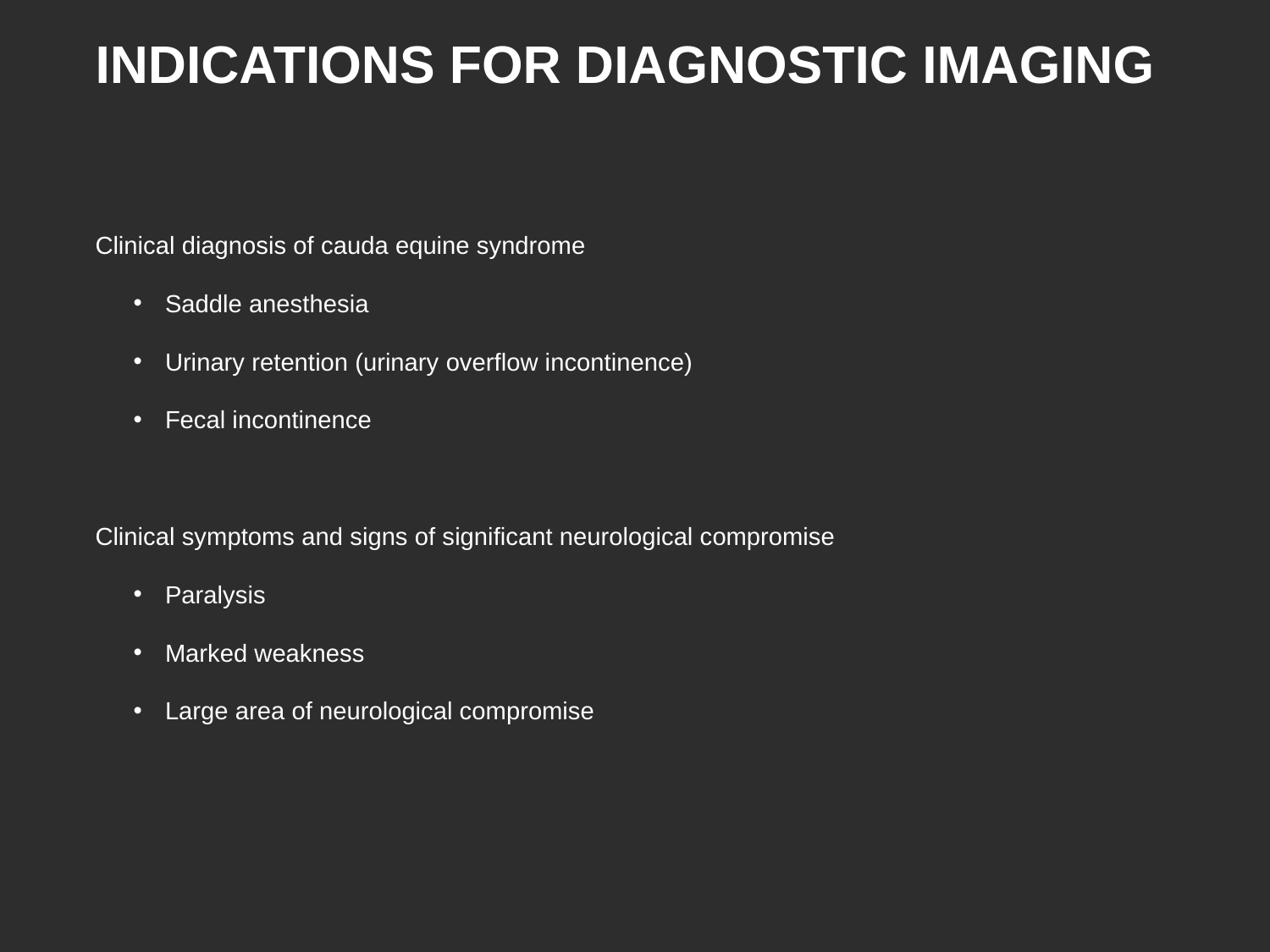

# Indications for Diagnostic Imaging
Clinical diagnosis of cauda equine syndrome
Saddle anesthesia
Urinary retention (urinary overflow incontinence)
Fecal incontinence
Clinical symptoms and signs of significant neurological compromise
Paralysis
Marked weakness
Large area of neurological compromise

## Slide 6
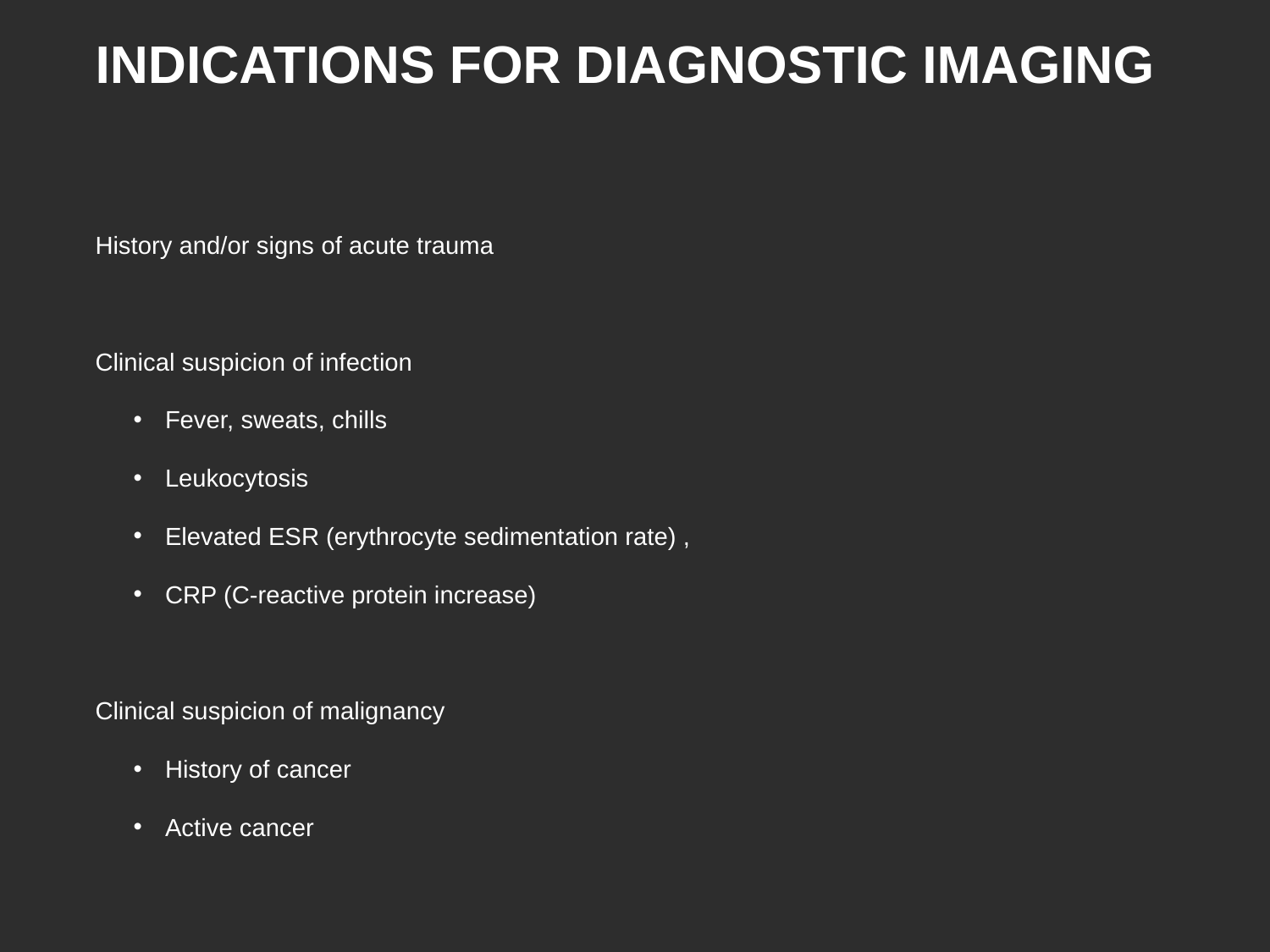

# Indications for Diagnostic Imaging
History and/or signs of acute trauma
Clinical suspicion of infection
Fever, sweats, chills
Leukocytosis
Elevated ESR (erythrocyte sedimentation rate) ,
CRP (C-reactive protein increase)
Clinical suspicion of malignancy
History of cancer
Active cancer

## Slide 7
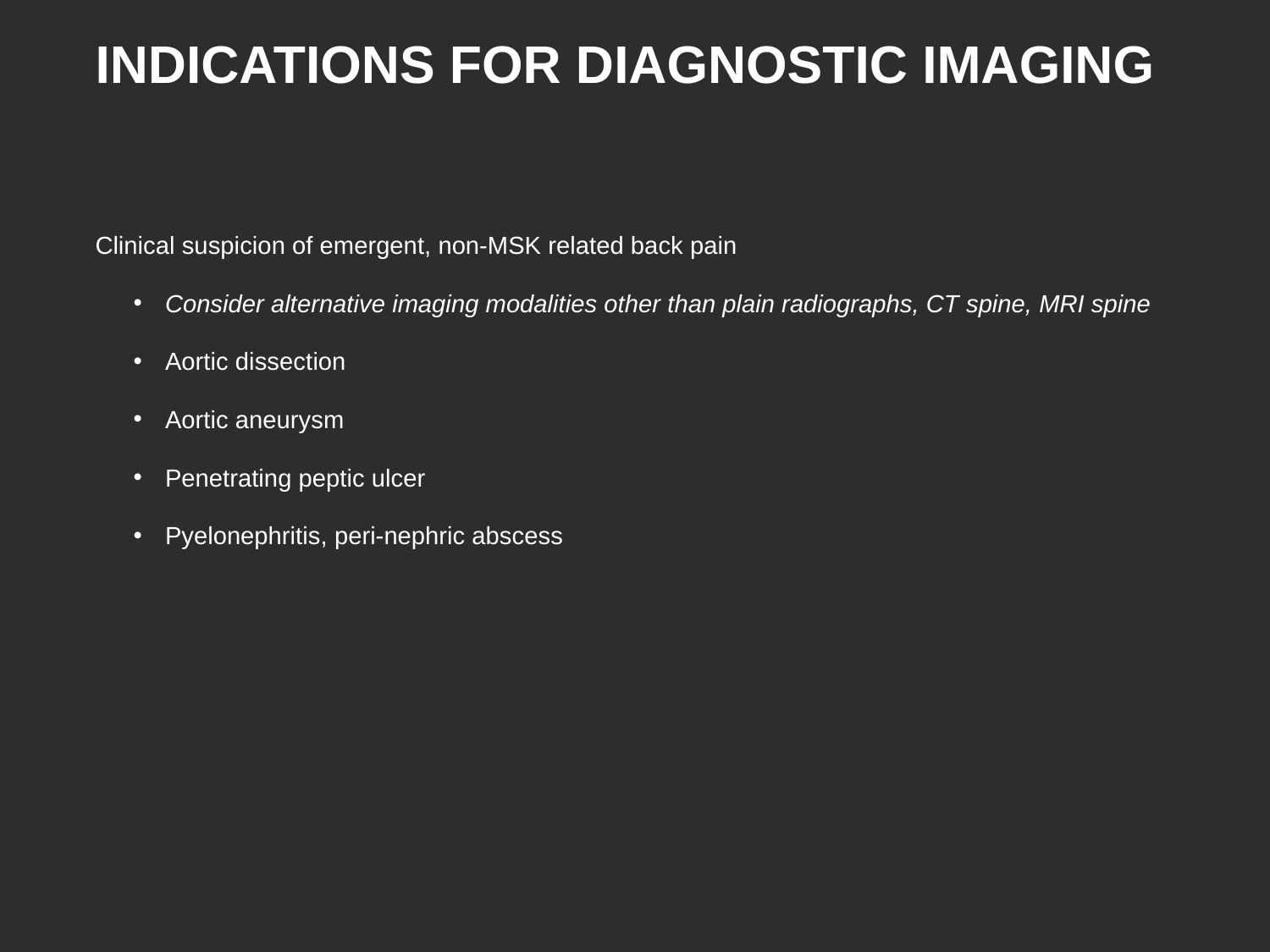

# Indications for Diagnostic Imaging
Clinical suspicion of emergent, non-MSK related back pain
Consider alternative imaging modalities other than plain radiographs, CT spine, MRI spine
Aortic dissection
Aortic aneurysm
Penetrating peptic ulcer
Pyelonephritis, peri-nephric abscess

## Slide 8
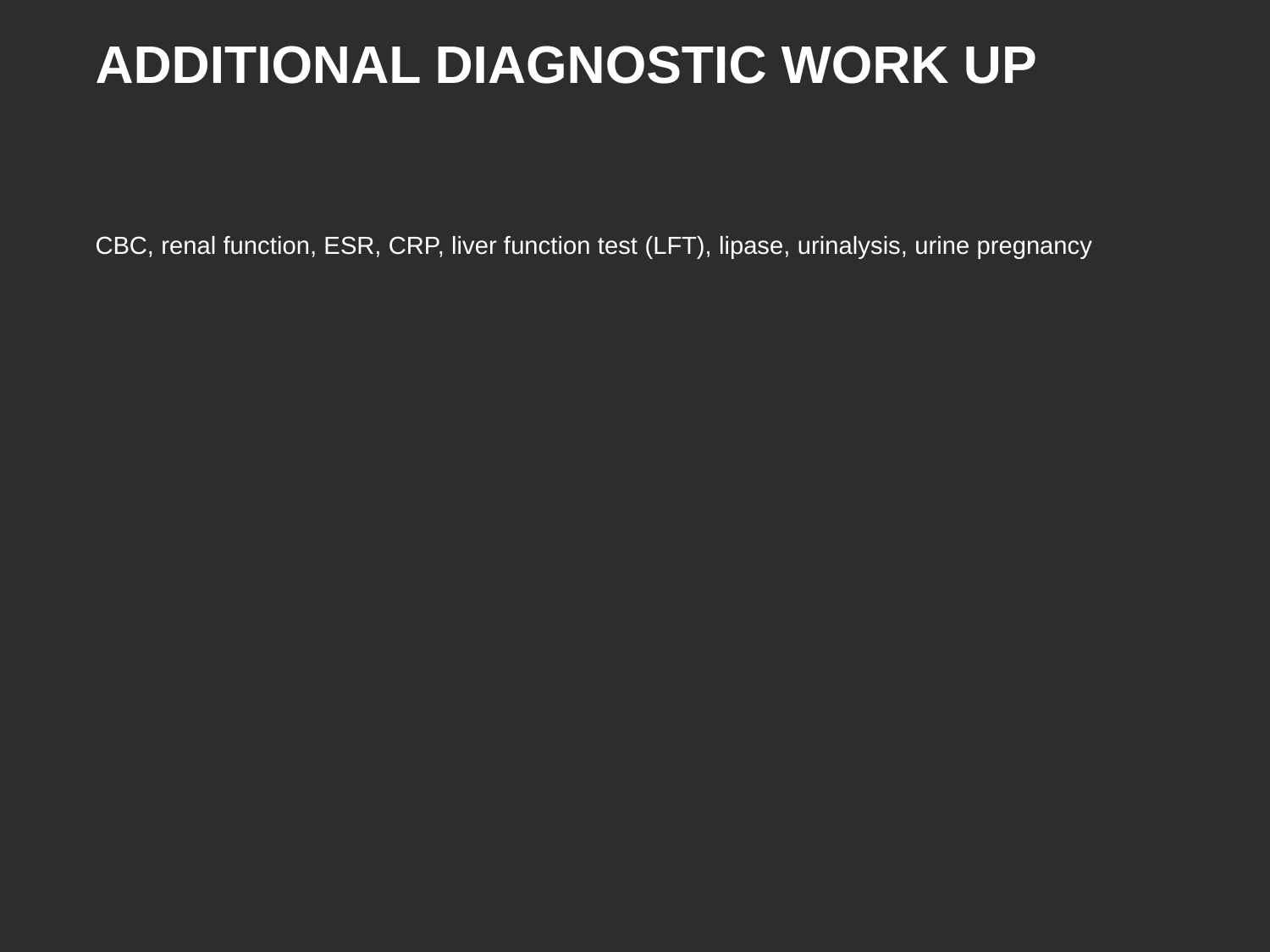

# Additional Diagnostic Work Up
CBC, renal function, ESR, CRP, liver function test (LFT), lipase, urinalysis, urine pregnancy

## Slide 9
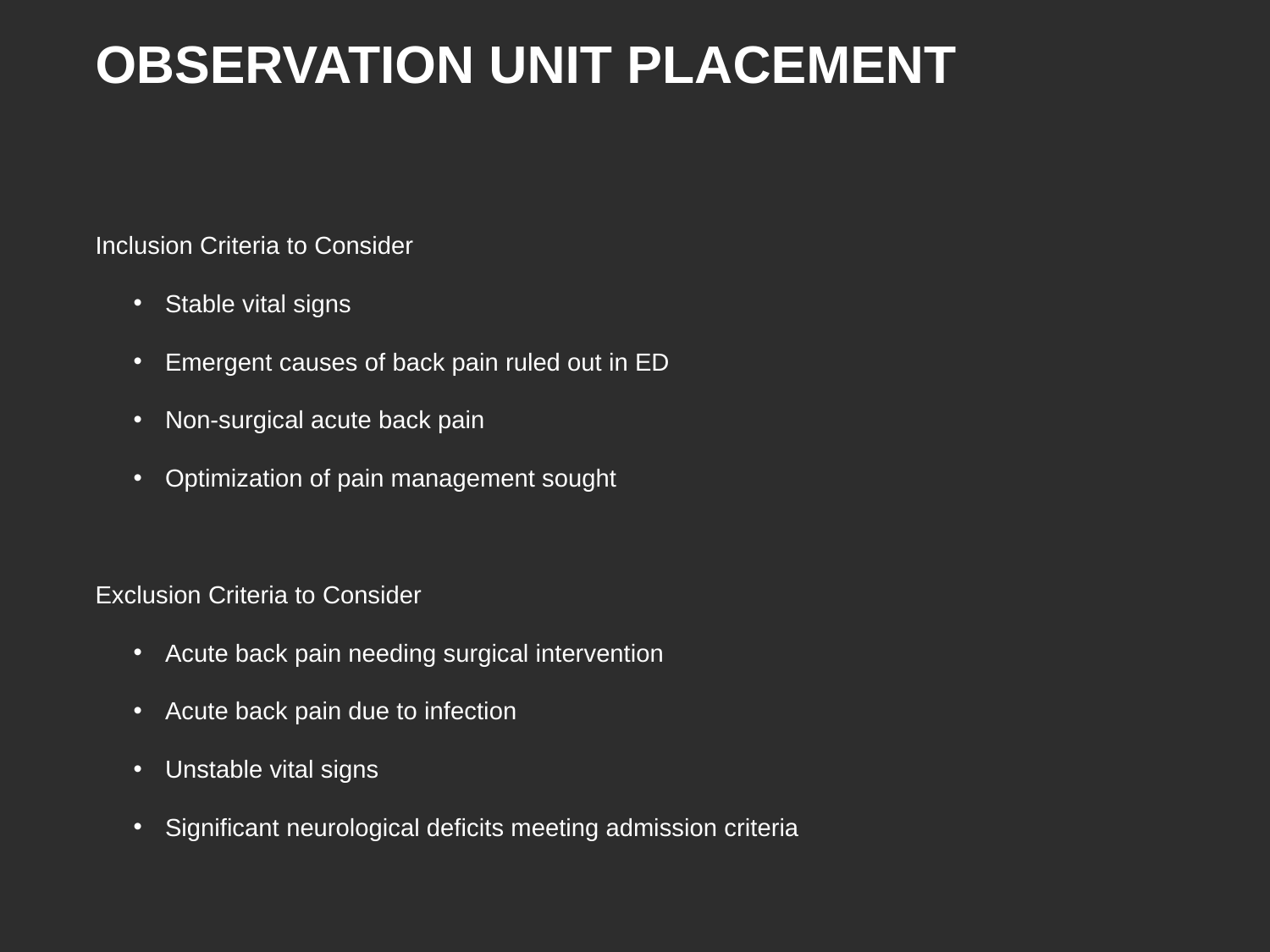

# Observation Unit Placement
Inclusion Criteria to Consider
Stable vital signs
Emergent causes of back pain ruled out in ED
Non-surgical acute back pain
Optimization of pain management sought
Exclusion Criteria to Consider
Acute back pain needing surgical intervention
Acute back pain due to infection
Unstable vital signs
Significant neurological deficits meeting admission criteria

## Slide 10
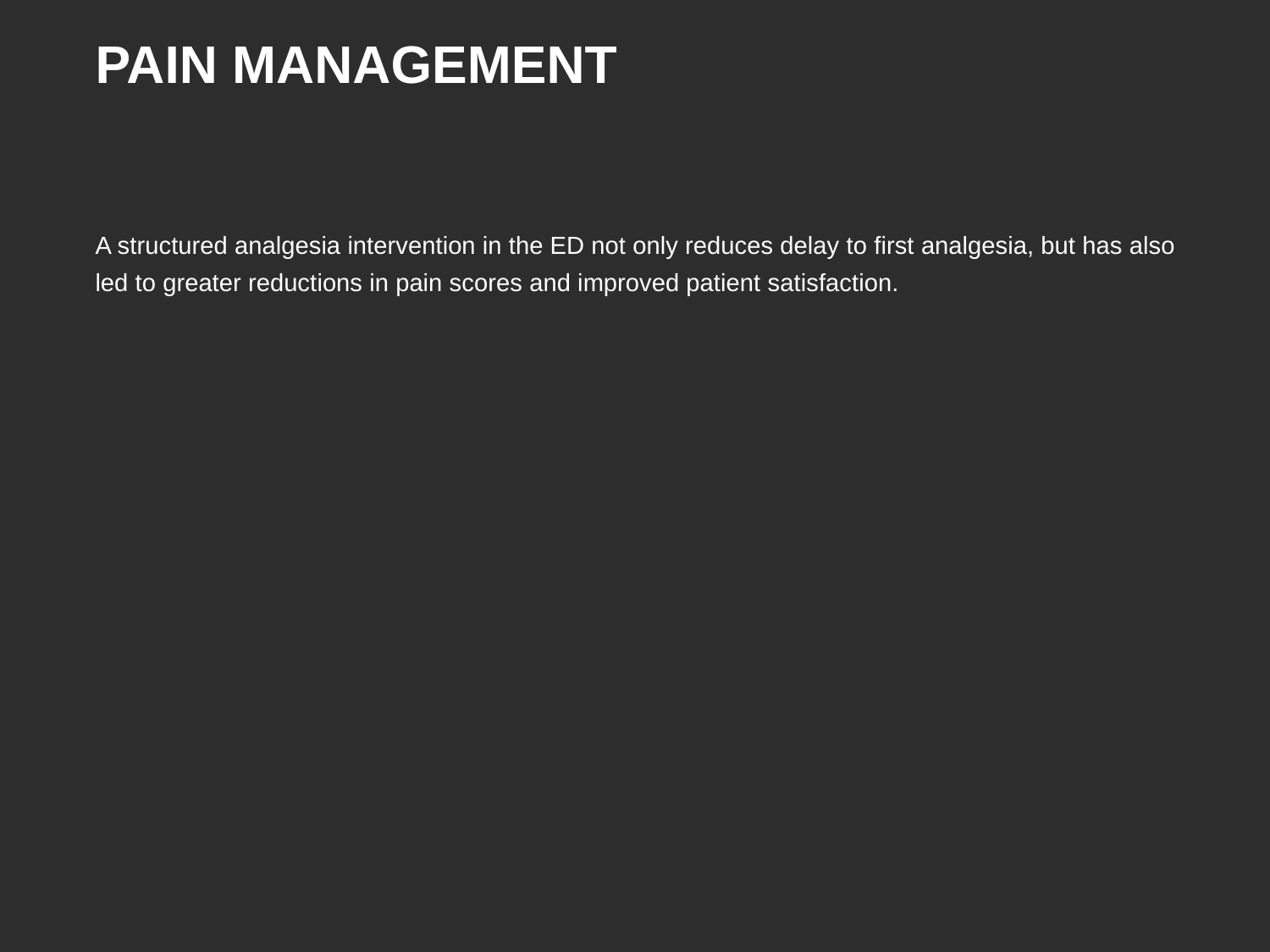

# Pain Management
A structured analgesia intervention in the ED not only reduces delay to first analgesia, but has also led to greater reductions in pain scores and improved patient satisfaction.

## Slide 11
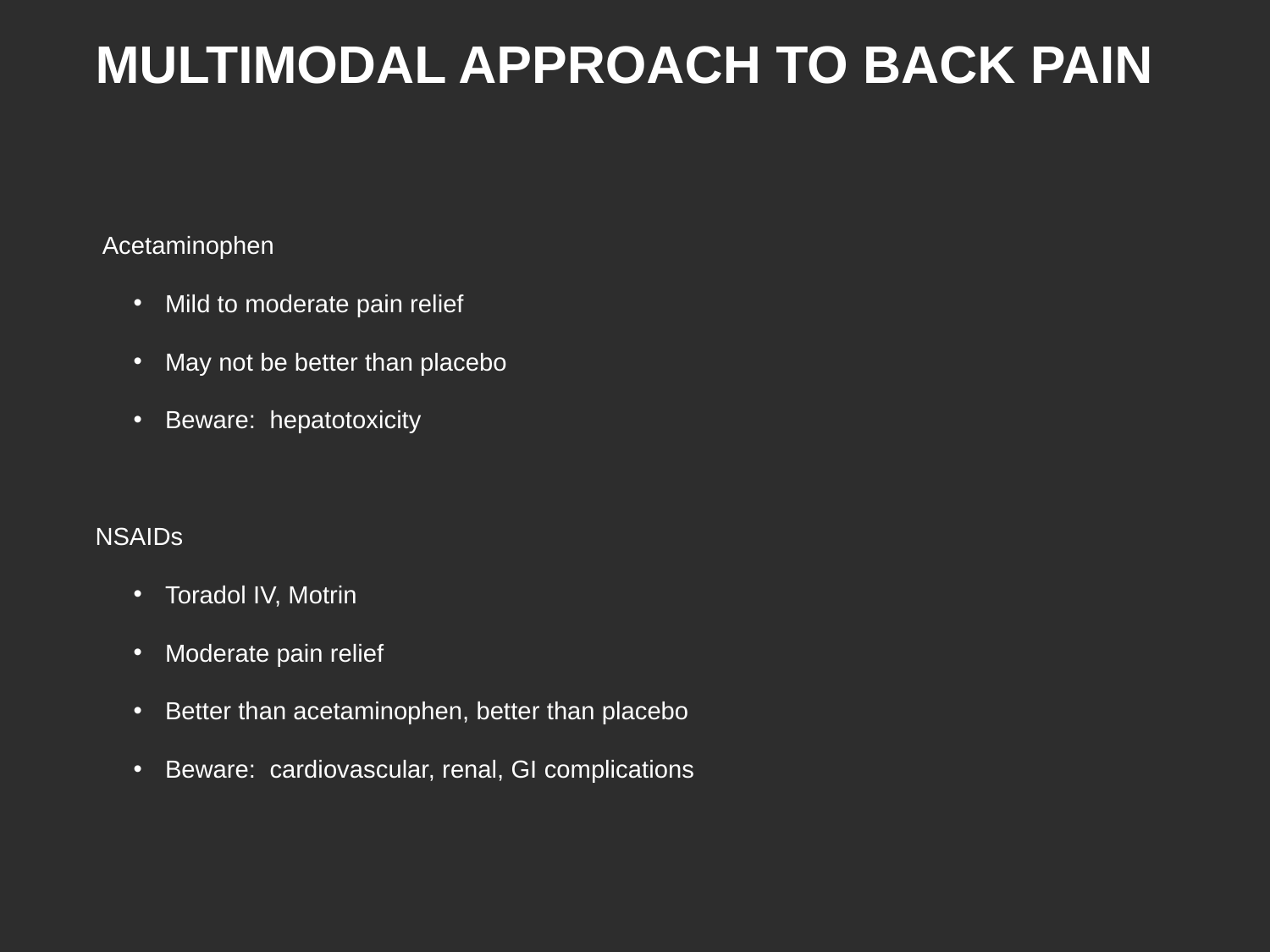

# Multimodal Approach to Back Pain
 Acetaminophen
Mild to moderate pain relief
May not be better than placebo
Beware: hepatotoxicity
NSAIDs
Toradol IV, Motrin
Moderate pain relief
Better than acetaminophen, better than placebo
Beware: cardiovascular, renal, GI complications

## Slide 12
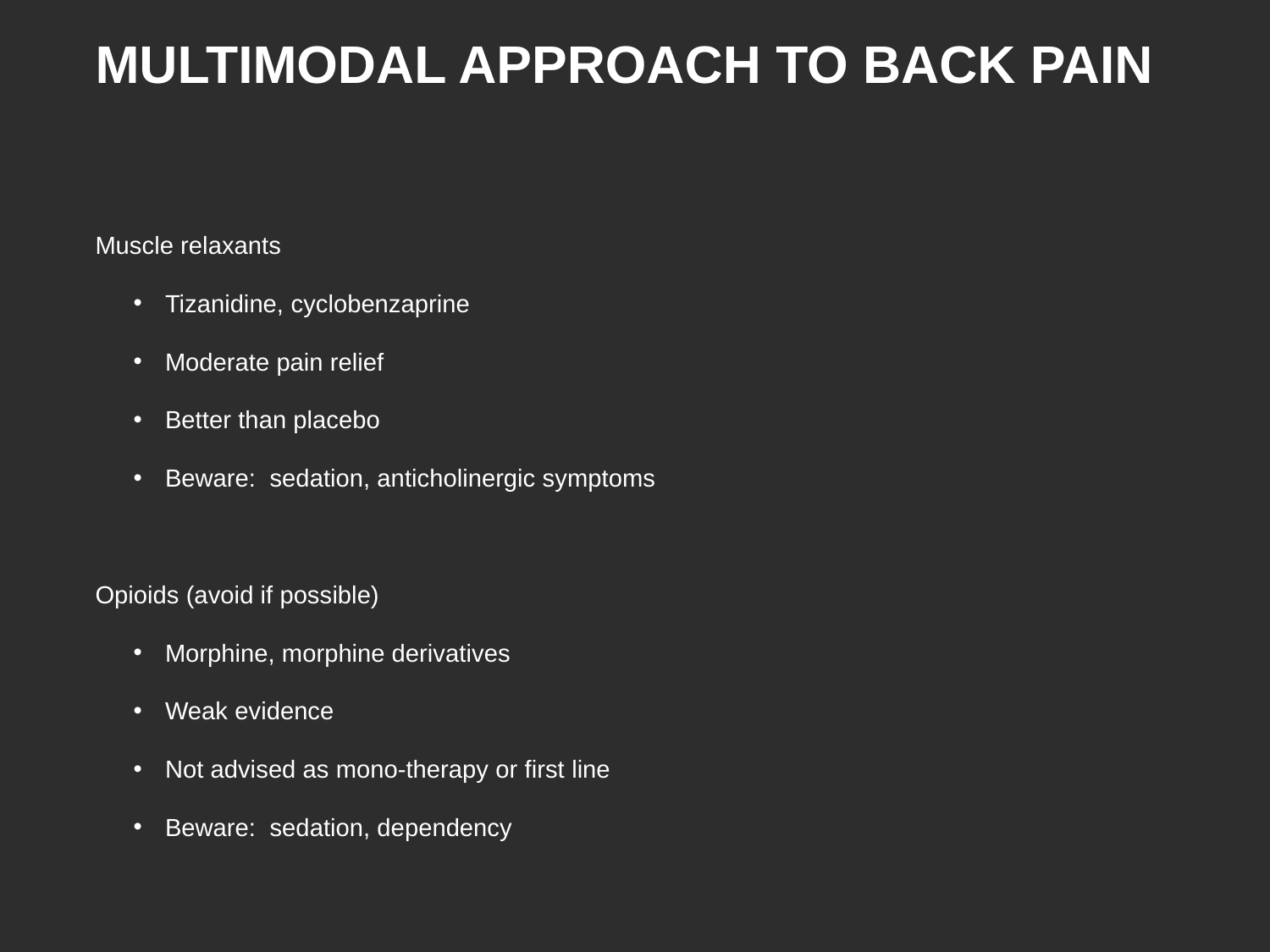

# Multimodal Approach to Back Pain
Muscle relaxants
Tizanidine, cyclobenzaprine
Moderate pain relief
Better than placebo
Beware: sedation, anticholinergic symptoms
Opioids (avoid if possible)
Morphine, morphine derivatives
Weak evidence
Not advised as mono-therapy or first line
Beware: sedation, dependency

## Slide 13
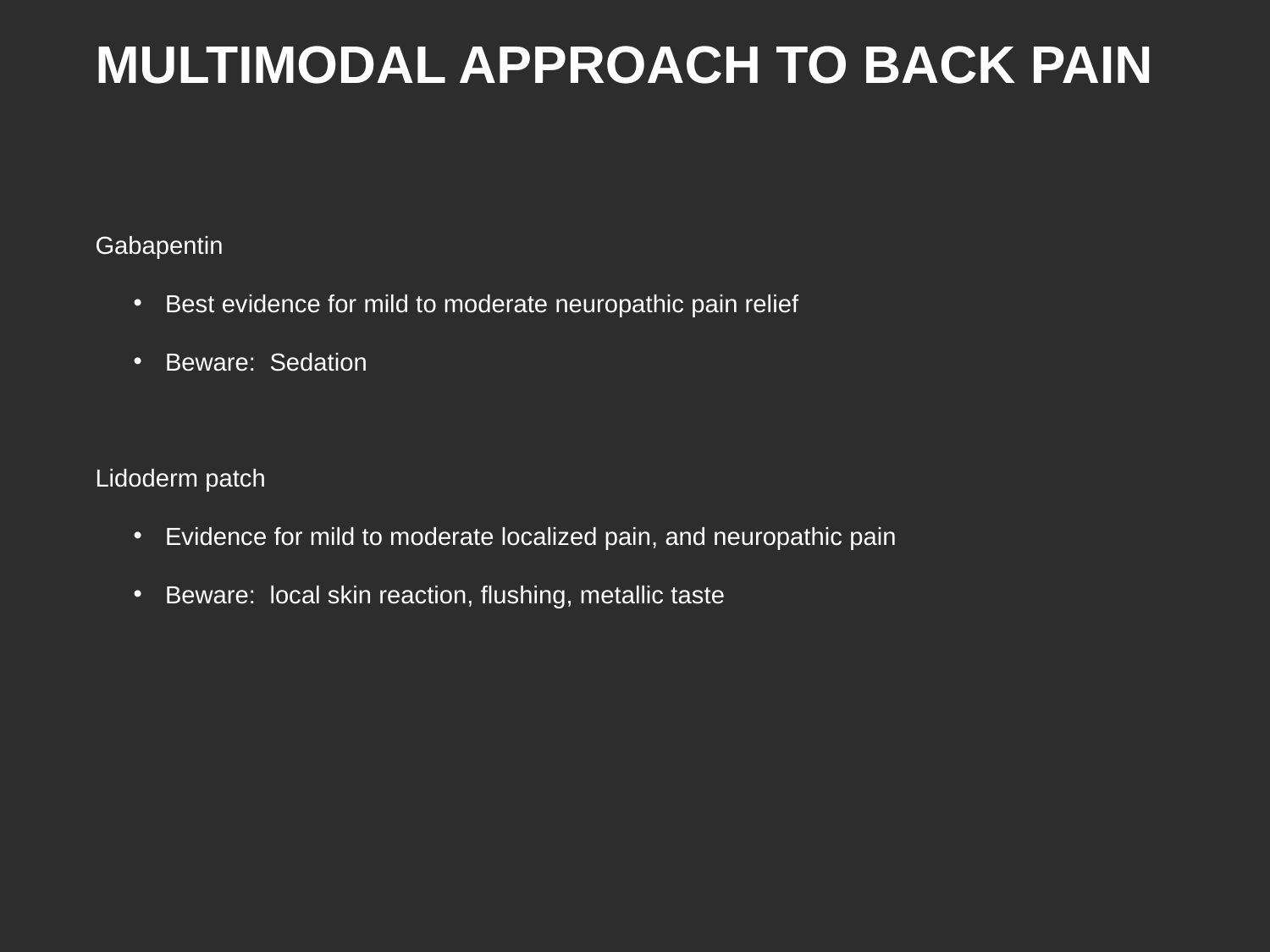

# Multimodal Approach to Back Pain
Gabapentin
Best evidence for mild to moderate neuropathic pain relief
Beware: Sedation
Lidoderm patch
Evidence for mild to moderate localized pain, and neuropathic pain
Beware: local skin reaction, flushing, metallic taste

## Slide 14
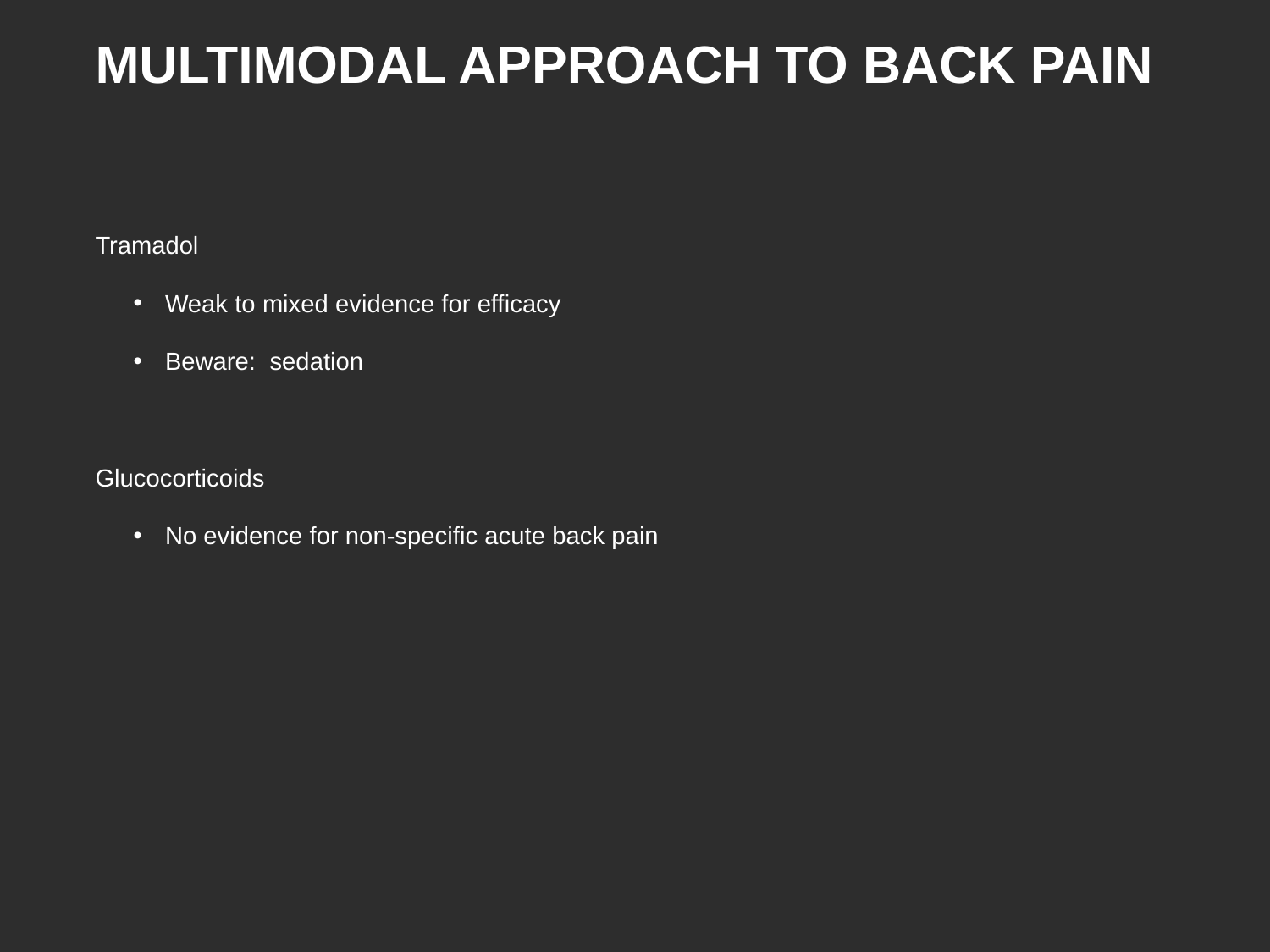

# Multimodal Approach to Back Pain
Tramadol
Weak to mixed evidence for efficacy
Beware: sedation
Glucocorticoids
No evidence for non-specific acute back pain

## Slide 15
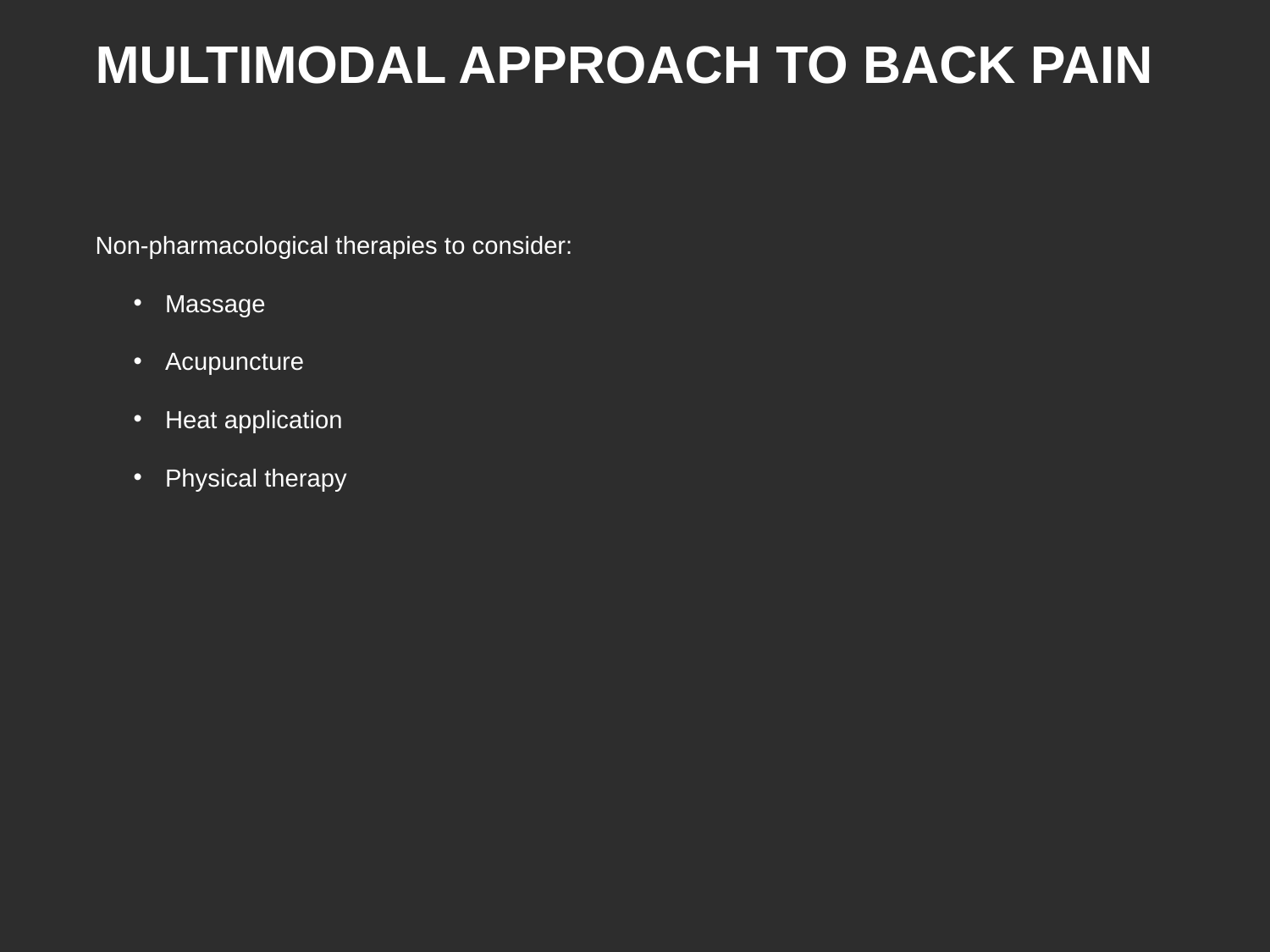

# Multimodal Approach to Back Pain
Non-pharmacological therapies to consider:
Massage
Acupuncture
Heat application
Physical therapy

## Slide 16
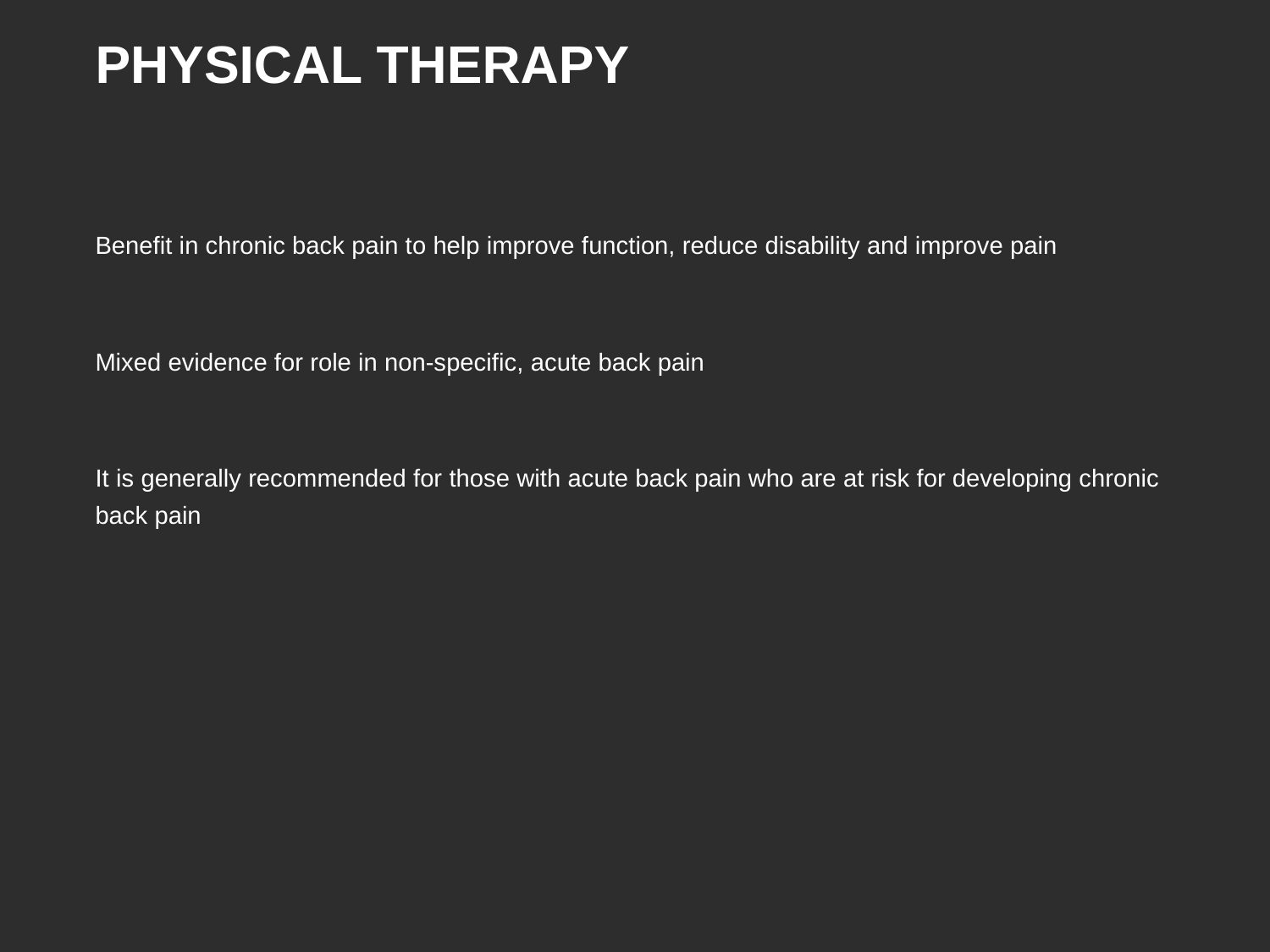

# Physical Therapy
Benefit in chronic back pain to help improve function, reduce disability and improve pain
Mixed evidence for role in non-specific, acute back pain
It is generally recommended for those with acute back pain who are at risk for developing chronic back pain

## Slide 17
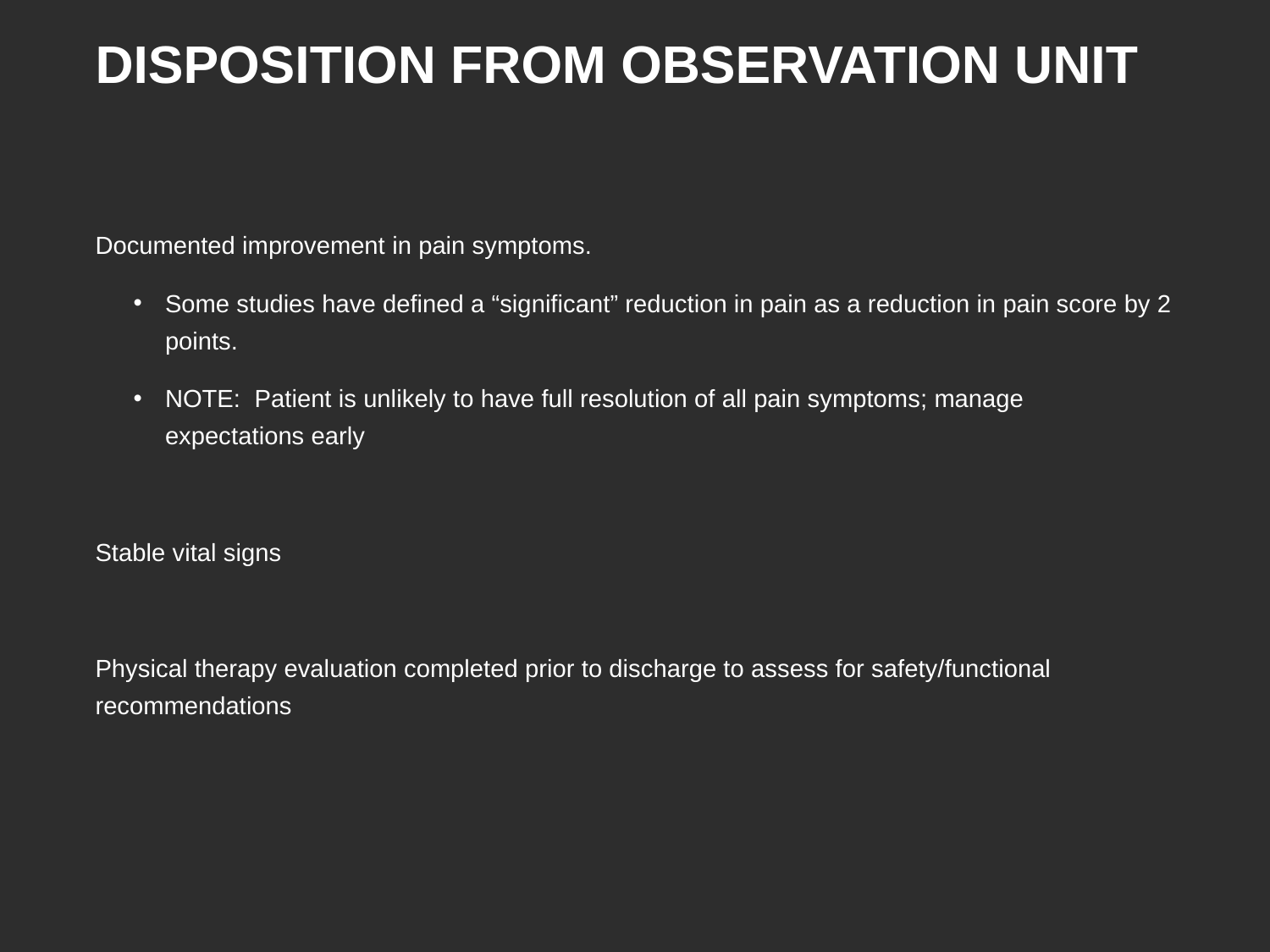

# Disposition from Observation Unit
Documented improvement in pain symptoms.
Some studies have defined a “significant” reduction in pain as a reduction in pain score by 2 points.
NOTE:  Patient is unlikely to have full resolution of all pain symptoms; manage expectations early
Stable vital signs
Physical therapy evaluation completed prior to discharge to assess for safety/functional recommendations

## Slide 18
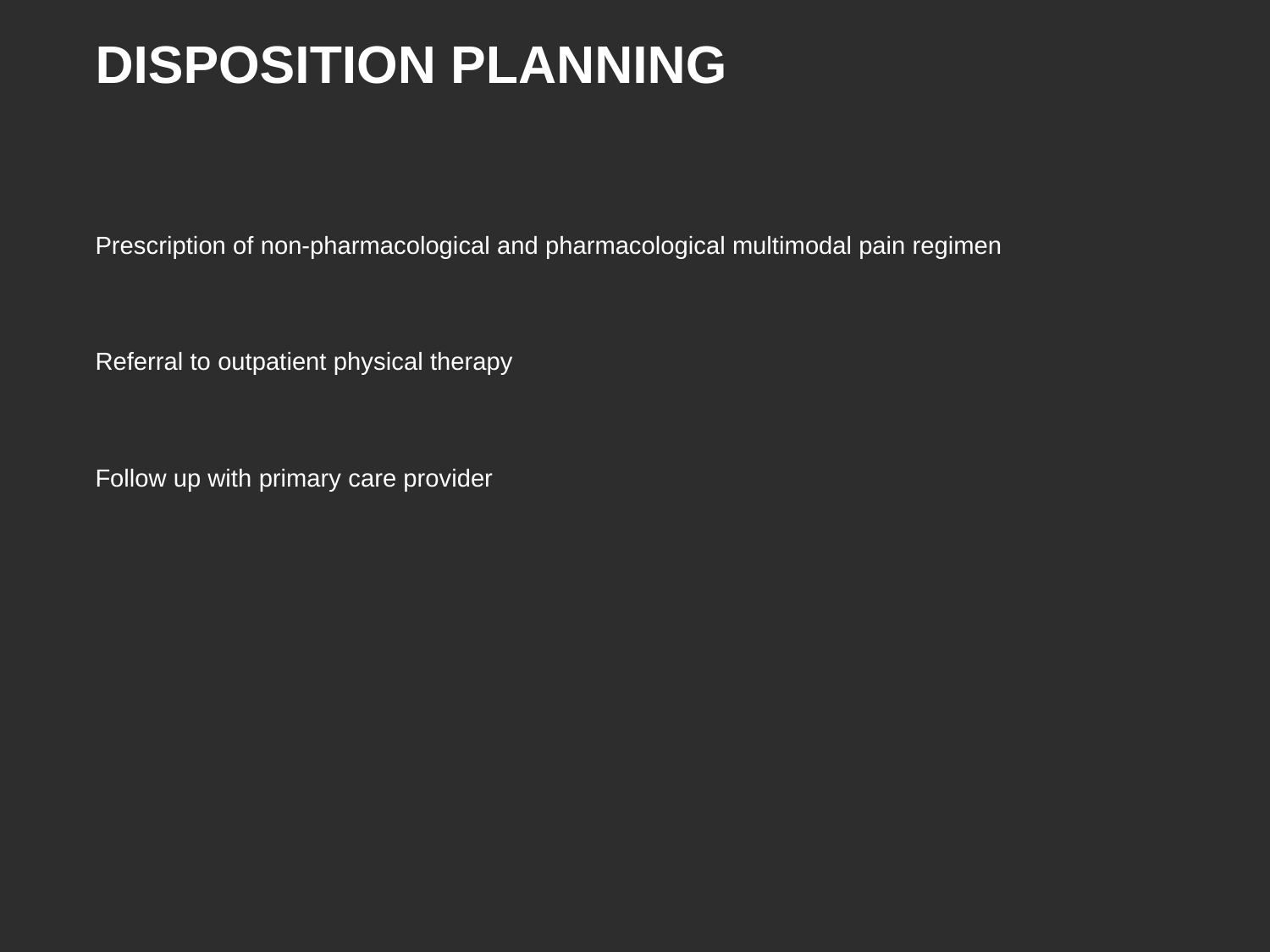

# Disposition Planning
Prescription of non-pharmacological and pharmacological multimodal pain regimen
Referral to outpatient physical therapy
Follow up with primary care provider

## Slide 19
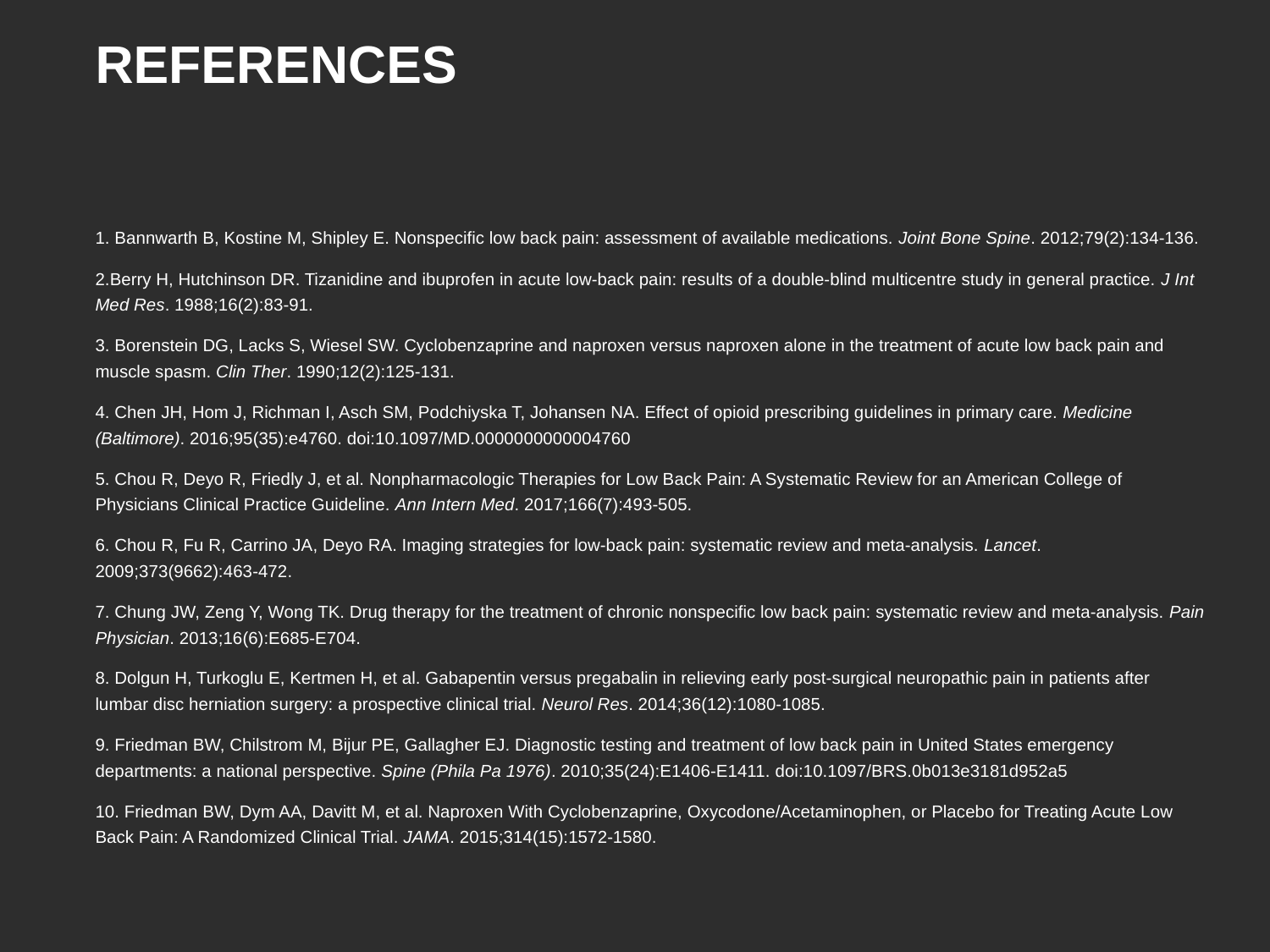

# References
1. Bannwarth B, Kostine M, Shipley E. Nonspecific low back pain: assessment of available medications. Joint Bone Spine. 2012;79(2):134-136.
2.Berry H, Hutchinson DR. Tizanidine and ibuprofen in acute low-back pain: results of a double-blind multicentre study in general practice. J Int Med Res. 1988;16(2):83-91.
3. Borenstein DG, Lacks S, Wiesel SW. Cyclobenzaprine and naproxen versus naproxen alone in the treatment of acute low back pain and muscle spasm. Clin Ther. 1990;12(2):125-131.
4. Chen JH, Hom J, Richman I, Asch SM, Podchiyska T, Johansen NA. Effect of opioid prescribing guidelines in primary care. Medicine (Baltimore). 2016;95(35):e4760. doi:10.1097/MD.0000000000004760
5. Chou R, Deyo R, Friedly J, et al. Nonpharmacologic Therapies for Low Back Pain: A Systematic Review for an American College of Physicians Clinical Practice Guideline. Ann Intern Med. 2017;166(7):493-505.
6. Chou R, Fu R, Carrino JA, Deyo RA. Imaging strategies for low-back pain: systematic review and meta-analysis. Lancet. 2009;373(9662):463-472.
7. Chung JW, Zeng Y, Wong TK. Drug therapy for the treatment of chronic nonspecific low back pain: systematic review and meta-analysis. Pain Physician. 2013;16(6):E685-E704.
8. Dolgun H, Turkoglu E, Kertmen H, et al. Gabapentin versus pregabalin in relieving early post-surgical neuropathic pain in patients after lumbar disc herniation surgery: a prospective clinical trial. Neurol Res. 2014;36(12):1080-1085.
9. Friedman BW, Chilstrom M, Bijur PE, Gallagher EJ. Diagnostic testing and treatment of low back pain in United States emergency departments: a national perspective. Spine (Phila Pa 1976). 2010;35(24):E1406-E1411. doi:10.1097/BRS.0b013e3181d952a5
10. Friedman BW, Dym AA, Davitt M, et al. Naproxen With Cyclobenzaprine, Oxycodone/Acetaminophen, or Placebo for Treating Acute Low Back Pain: A Randomized Clinical Trial. JAMA. 2015;314(15):1572-1580.

## Slide 20
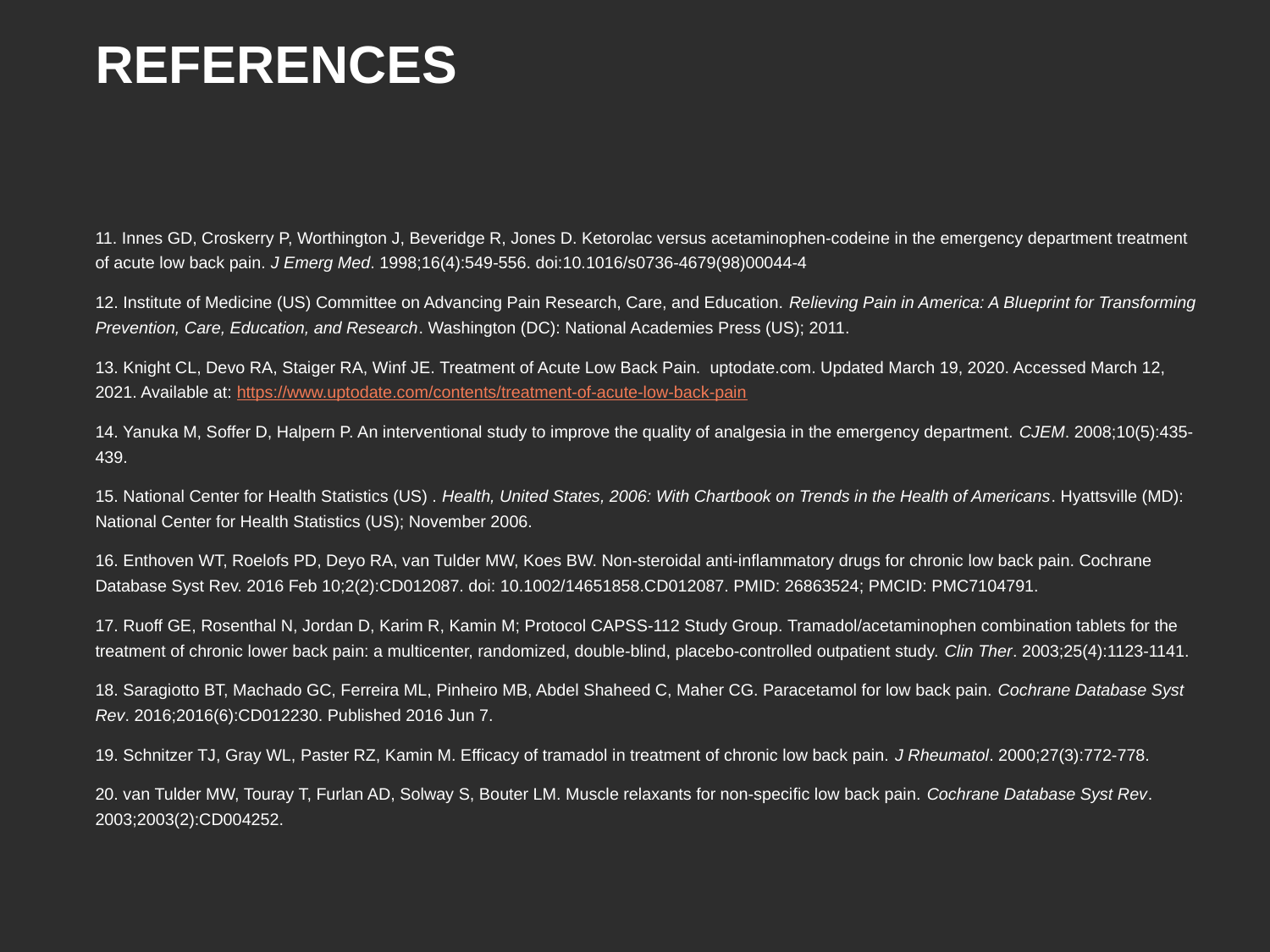

# References
11. Innes GD, Croskerry P, Worthington J, Beveridge R, Jones D. Ketorolac versus acetaminophen-codeine in the emergency department treatment of acute low back pain. J Emerg Med. 1998;16(4):549-556. doi:10.1016/s0736-4679(98)00044-4
12. Institute of Medicine (US) Committee on Advancing Pain Research, Care, and Education. Relieving Pain in America: A Blueprint for Transforming Prevention, Care, Education, and Research. Washington (DC): National Academies Press (US); 2011.
13. Knight CL, Devo RA, Staiger RA, Winf JE. Treatment of Acute Low Back Pain.  uptodate.com. Updated March 19, 2020. Accessed March 12, 2021. Available at: https://www.uptodate.com/contents/treatment-of-acute-low-back-pain
14. Yanuka M, Soffer D, Halpern P. An interventional study to improve the quality of analgesia in the emergency department. CJEM. 2008;10(5):435-439.
15. National Center for Health Statistics (US) . Health, United States, 2006: With Chartbook on Trends in the Health of Americans. Hyattsville (MD): National Center for Health Statistics (US); November 2006.
16. Enthoven WT, Roelofs PD, Deyo RA, van Tulder MW, Koes BW. Non-steroidal anti-inflammatory drugs for chronic low back pain. Cochrane Database Syst Rev. 2016 Feb 10;2(2):CD012087. doi: 10.1002/14651858.CD012087. PMID: 26863524; PMCID: PMC7104791.
17. Ruoff GE, Rosenthal N, Jordan D, Karim R, Kamin M; Protocol CAPSS-112 Study Group. Tramadol/acetaminophen combination tablets for the treatment of chronic lower back pain: a multicenter, randomized, double-blind, placebo-controlled outpatient study. Clin Ther. 2003;25(4):1123-1141.
18. Saragiotto BT, Machado GC, Ferreira ML, Pinheiro MB, Abdel Shaheed C, Maher CG. Paracetamol for low back pain. Cochrane Database Syst Rev. 2016;2016(6):CD012230. Published 2016 Jun 7.
19. Schnitzer TJ, Gray WL, Paster RZ, Kamin M. Efficacy of tramadol in treatment of chronic low back pain. J Rheumatol. 2000;27(3):772-778.
20. van Tulder MW, Touray T, Furlan AD, Solway S, Bouter LM. Muscle relaxants for non-specific low back pain. Cochrane Database Syst Rev. 2003;2003(2):CD004252.
