# Supplementary material for: An Observation Medicine Curriculum for Emergency Medicine Education
Source: J Educ Teach Emerg Med. 2021 Apr 19;6(2):C1–C72. doi: 10.21980/J87P92 (PMC10332786; doi:10.21980/J87P92)
Supplement: Supplementary file 23 — Please see associated PowerPoint file [file jetem-6-2-c1-supp23.pptx]

## Slide 1
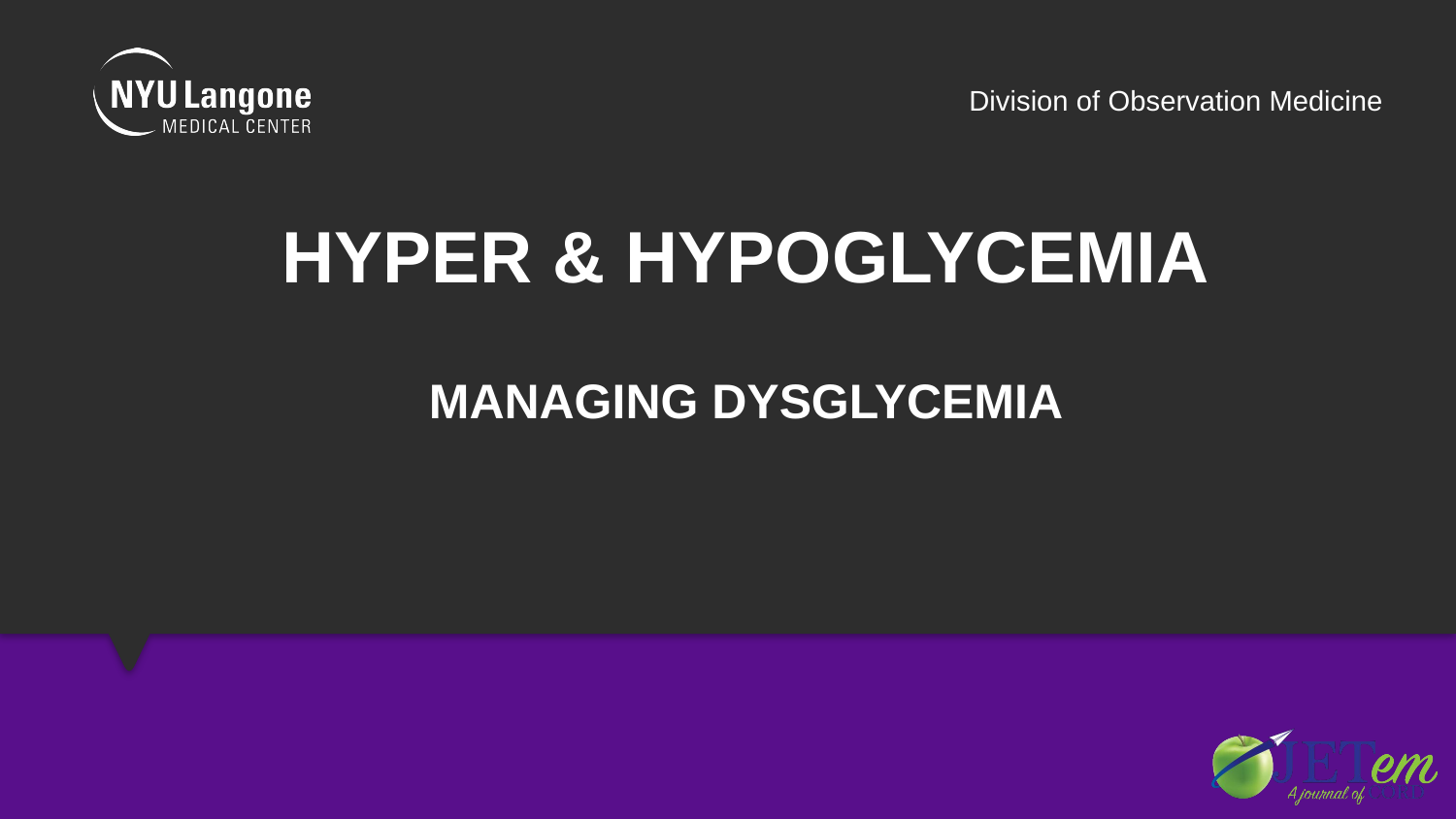

Division of Observation Medicine
# HYPER & HYPOGLYCEMIA Managing DYSGLYCEMIA

## Slide 2
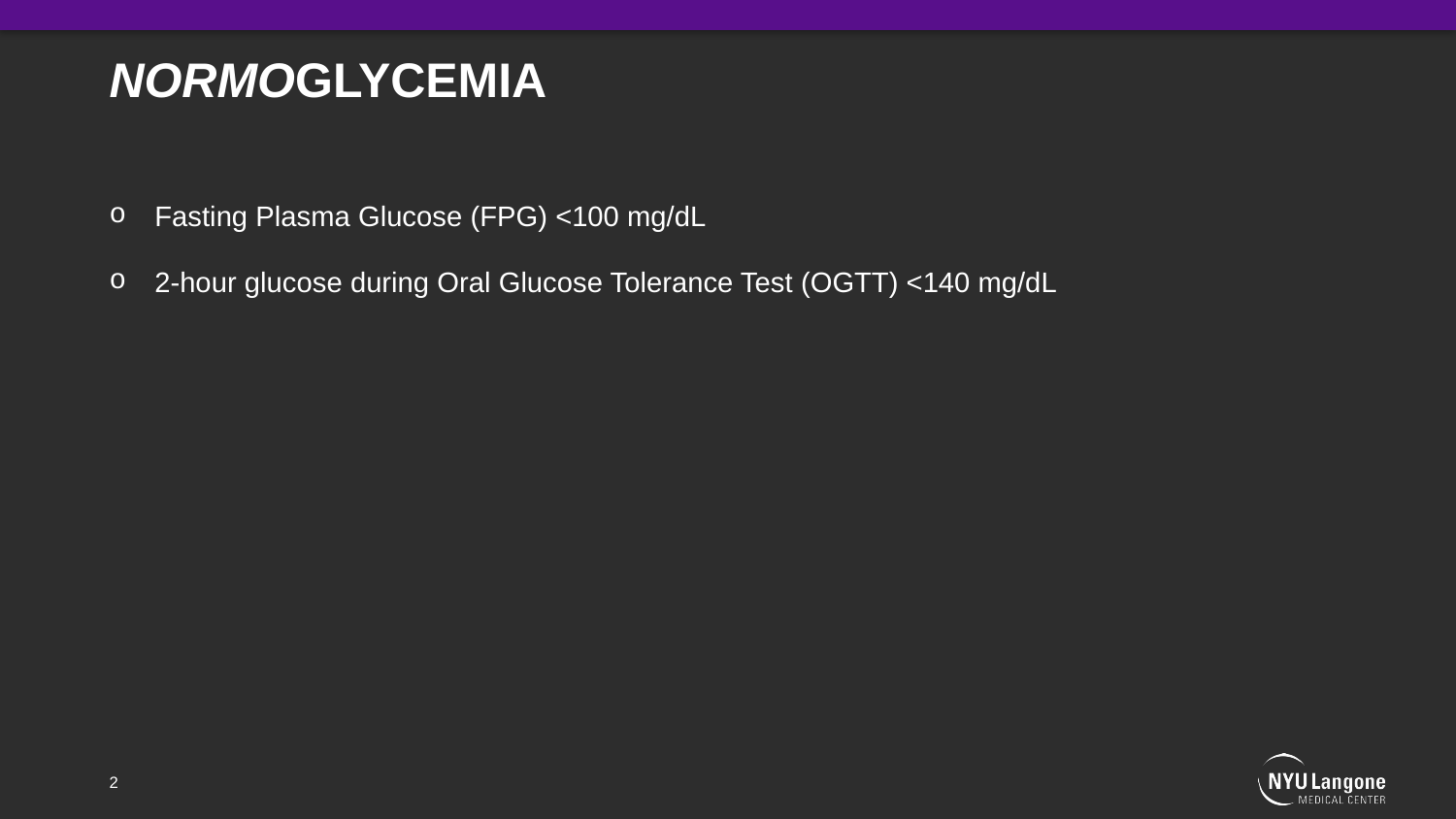

# NORMOGLYCEMIA
Fasting Plasma Glucose (FPG) <100 mg/dL
2-hour glucose during Oral Glucose Tolerance Test (OGTT) <140 mg/dL
2

## Slide 3
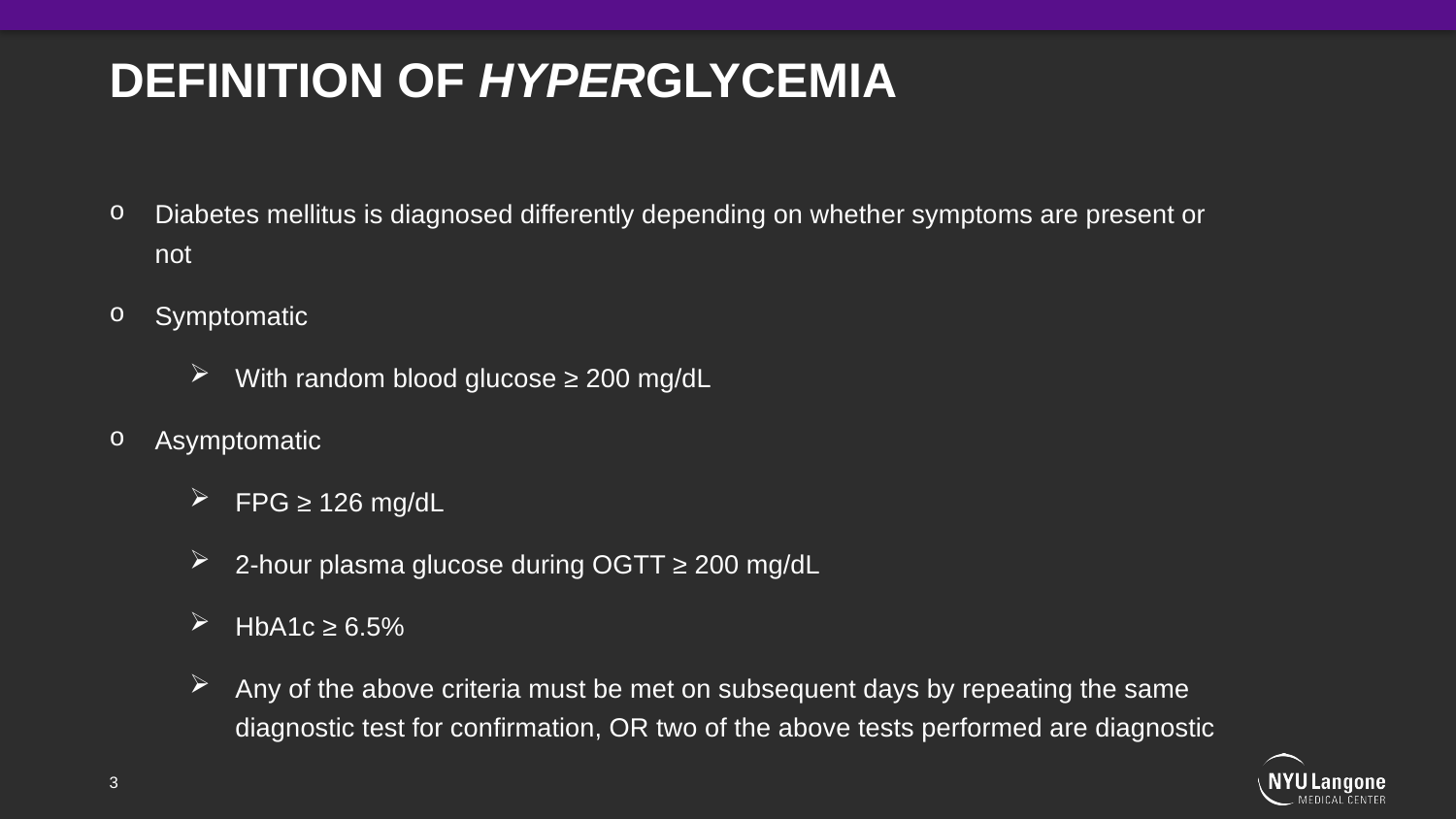

# DEFINITION OF HYPERGLYCEMIA
Diabetes mellitus is diagnosed differently depending on whether symptoms are present or not
Symptomatic
With random blood glucose ≥ 200 mg/dL
Asymptomatic
FPG ≥ 126 mg/dL
2-hour plasma glucose during OGTT ≥ 200 mg/dL
HbA1c ≥ 6.5%
Any of the above criteria must be met on subsequent days by repeating the same diagnostic test for confirmation, OR two of the above tests performed are diagnostic
3

## Slide 4
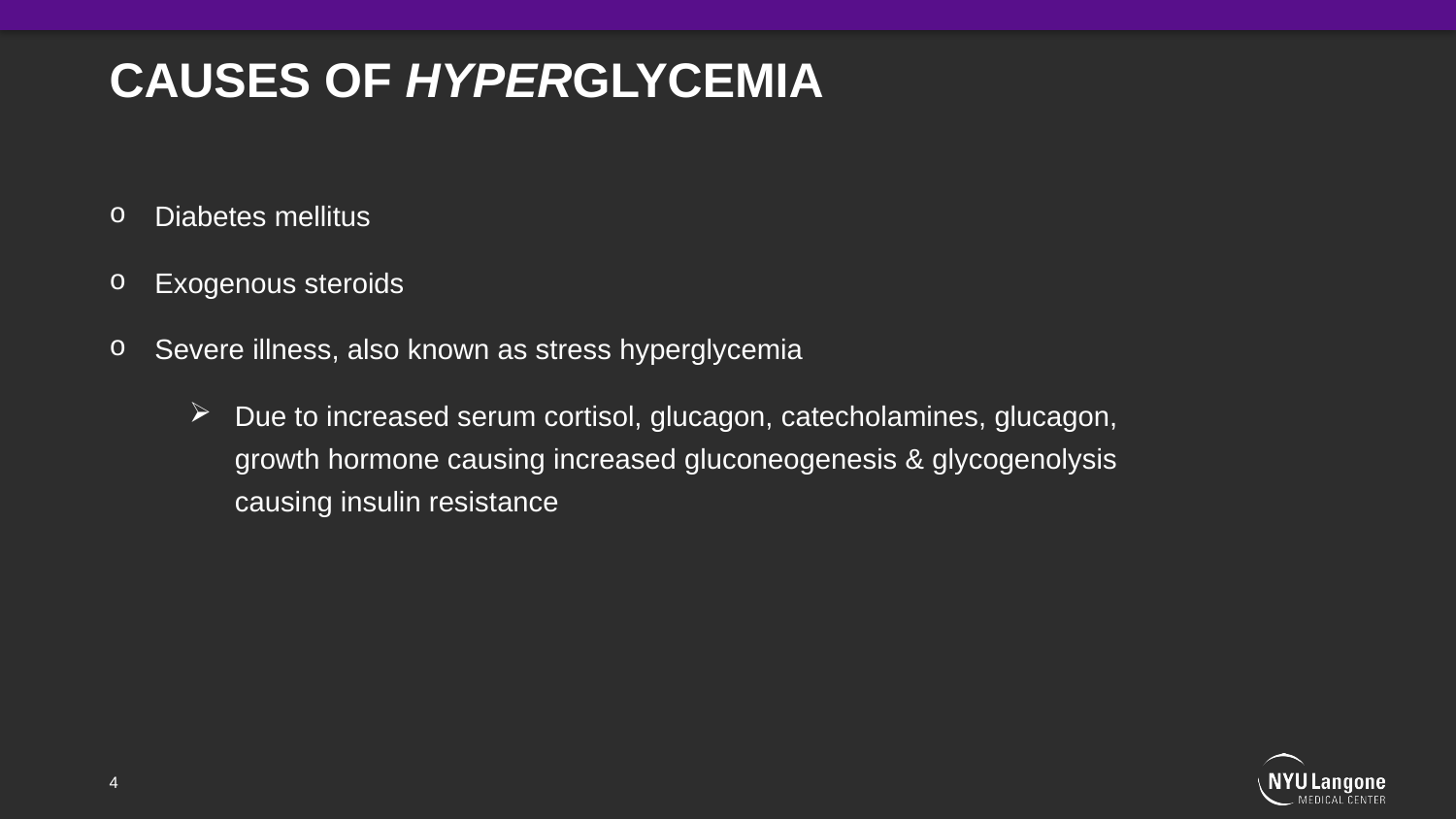

# CAUSES OF HYPERGLYCEMIA
Diabetes mellitus
Exogenous steroids
Severe illness, also known as stress hyperglycemia
Due to increased serum cortisol, glucagon, catecholamines, glucagon, growth hormone causing increased gluconeogenesis & glycogenolysis causing insulin resistance
4

## Slide 5
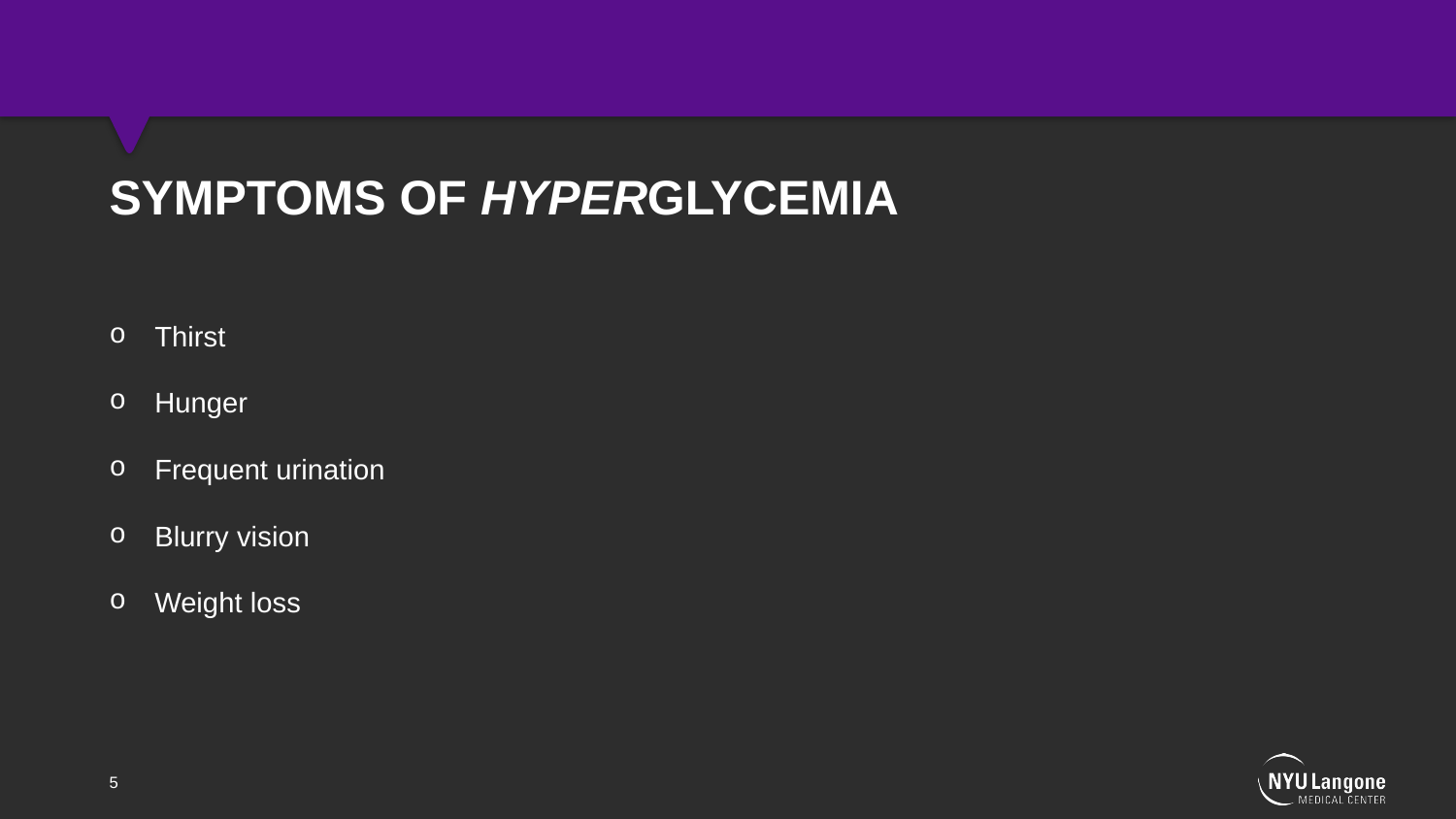

# SYMPTOMS OF HYPERGLYCEMIA
Thirst
Hunger
Frequent urination
Blurry vision
Weight loss
5

## Slide 6
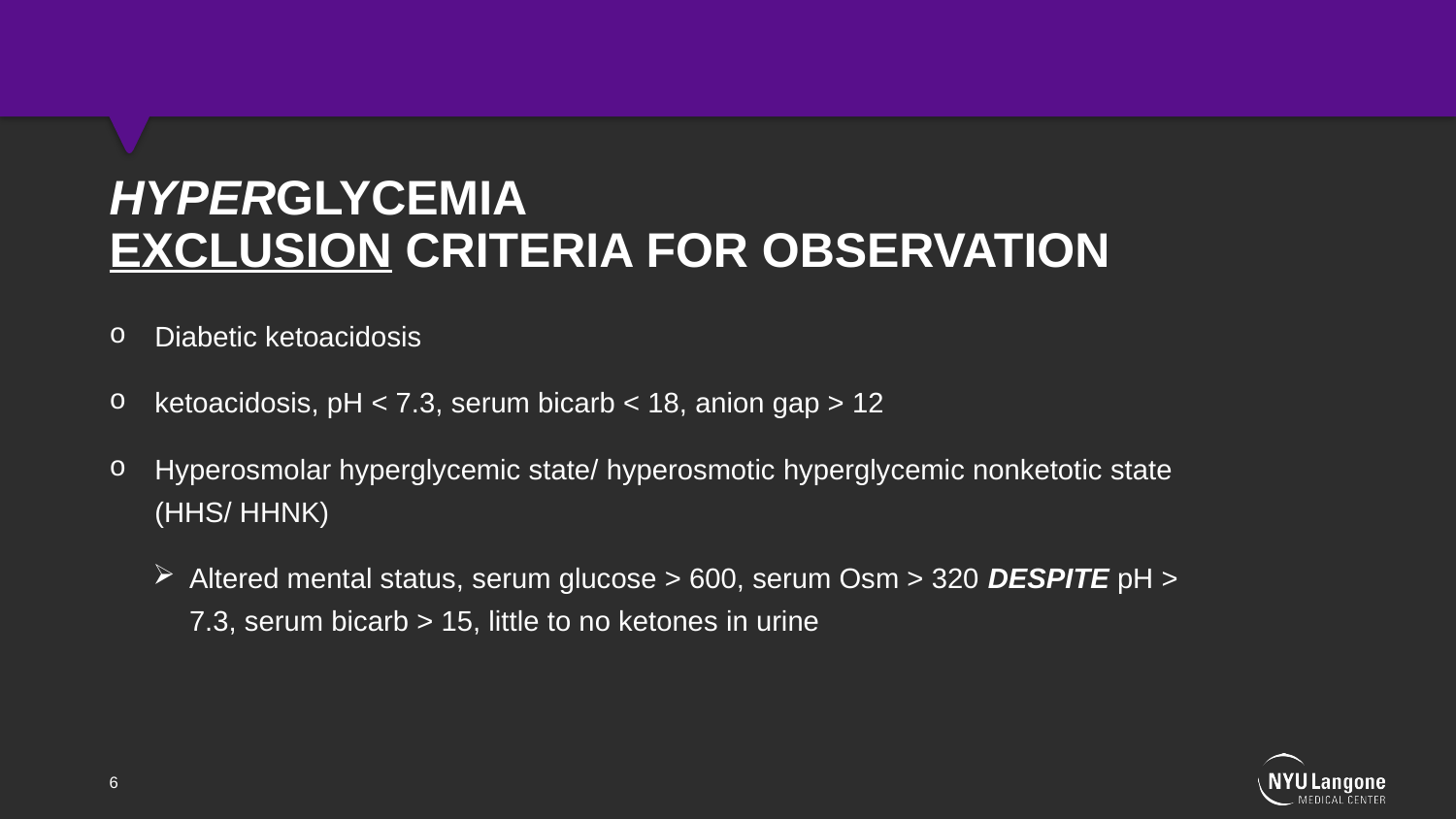

# HYPERGLYCEMIA EXCLUSION CRITERIA FOR OBSERVATION
Diabetic ketoacidosis
ketoacidosis, pH < 7.3, serum bicarb < 18, anion gap > 12
Hyperosmolar hyperglycemic state/ hyperosmotic hyperglycemic nonketotic state (HHS/ HHNK)
Altered mental status, serum glucose > 600, serum Osm > 320 DESPITE pH > 7.3, serum bicarb > 15, little to no ketones in urine
6

## Slide 7
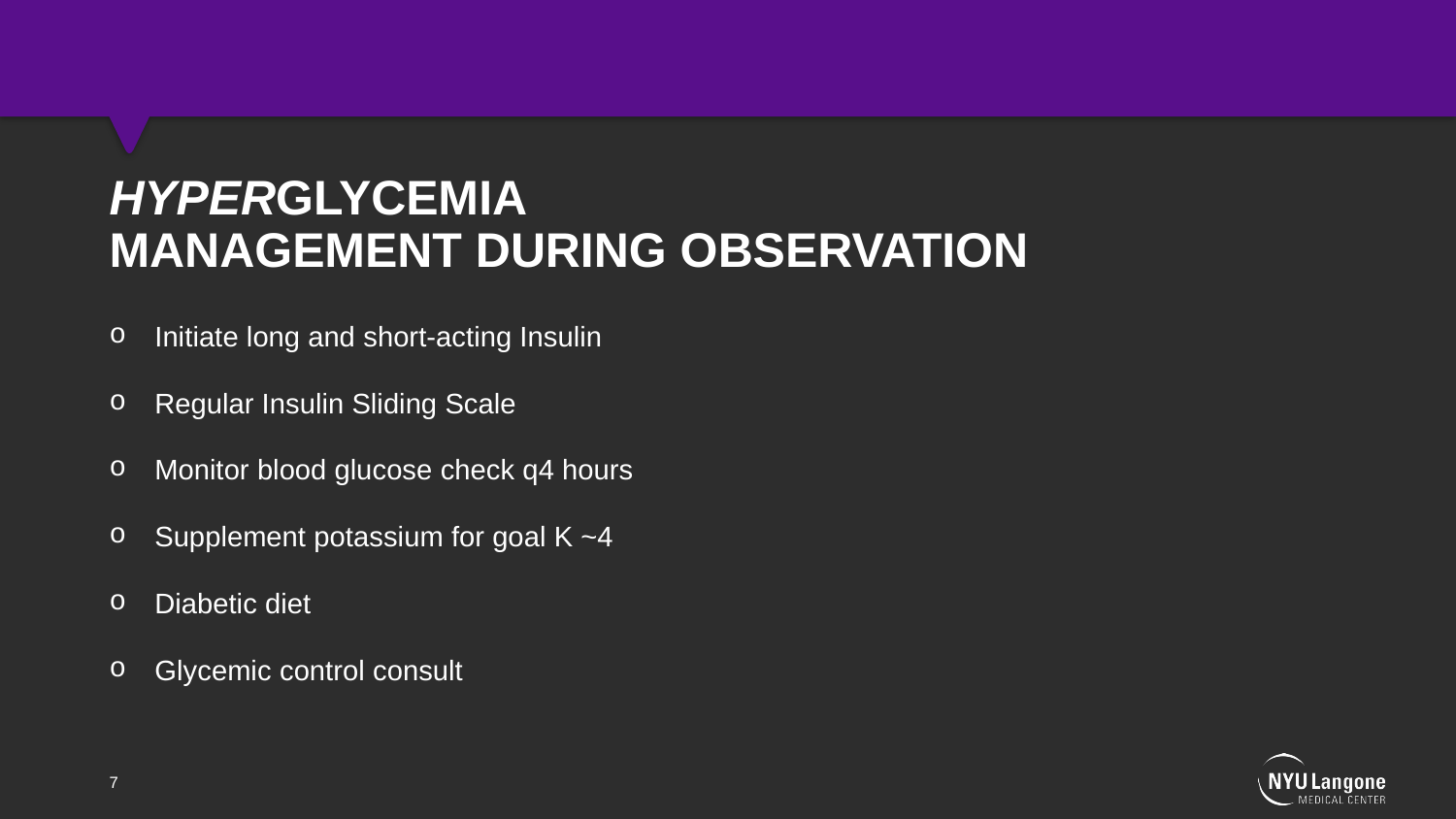

# HYPERGLYCEMIA MANAGEMENT DURING OBSERVATION
Initiate long and short-acting Insulin
Regular Insulin Sliding Scale
Monitor blood glucose check q4 hours
Supplement potassium for goal K ~4
Diabetic diet
Glycemic control consult
7

## Slide 8
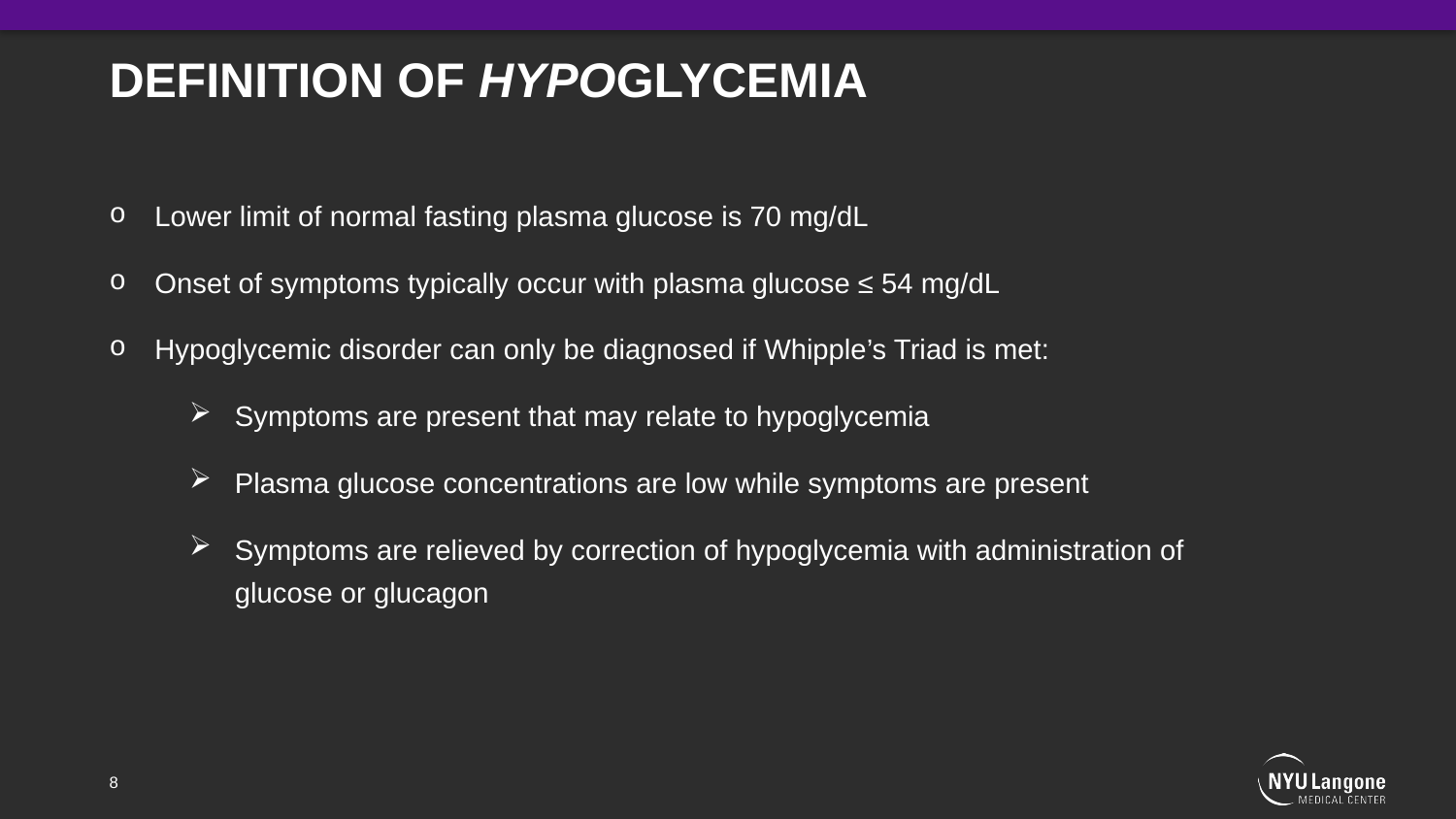

# DEFINITION OF HYPOGLYCEMIA
Lower limit of normal fasting plasma glucose is 70 mg/dL
Onset of symptoms typically occur with plasma glucose ≤ 54 mg/dL
Hypoglycemic disorder can only be diagnosed if Whipple’s Triad is met:
Symptoms are present that may relate to hypoglycemia
Plasma glucose concentrations are low while symptoms are present
Symptoms are relieved by correction of hypoglycemia with administration of glucose or glucagon
8

## Slide 9
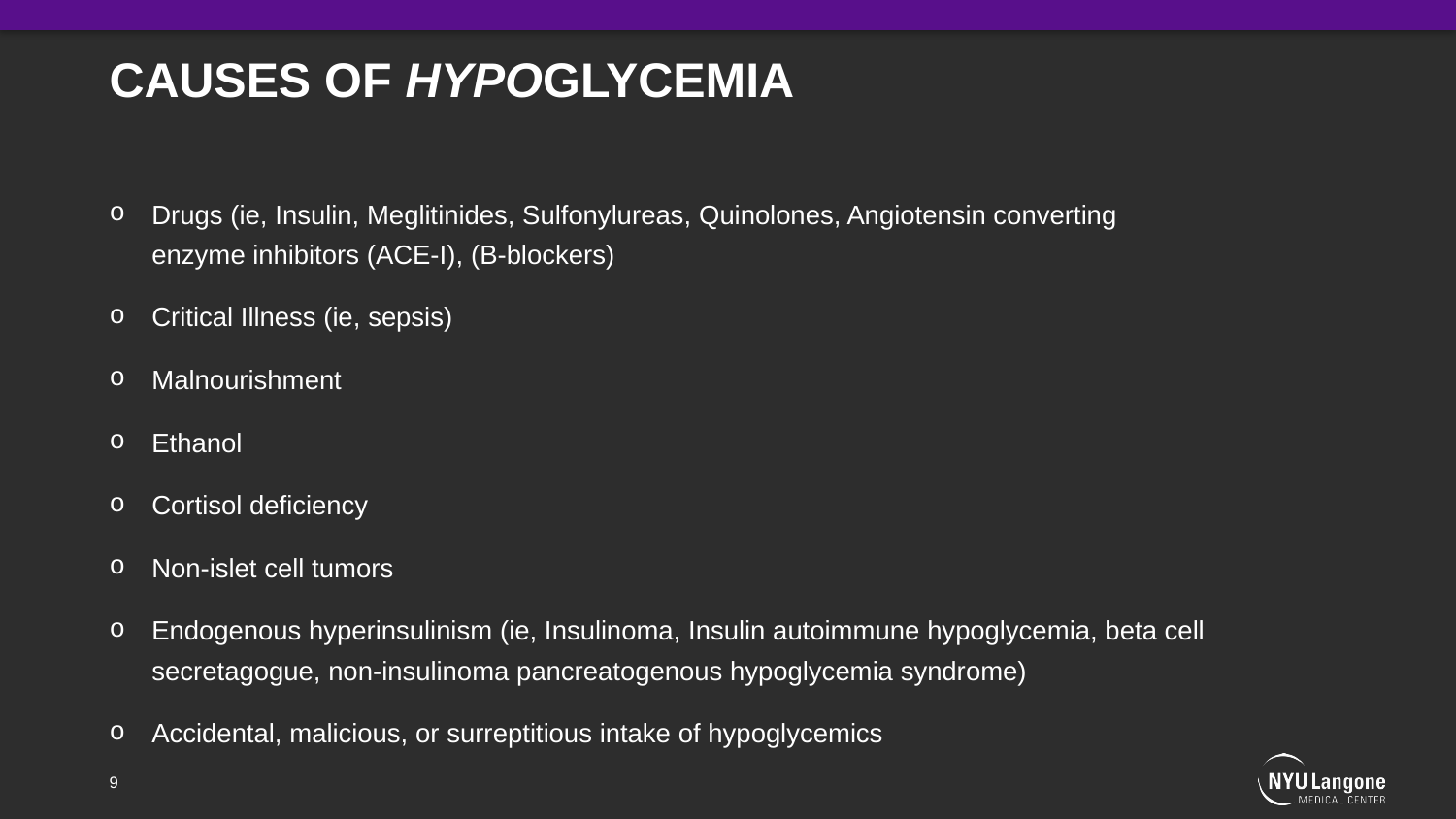

# CAUSES OF HYPOGLYCEMIA
Drugs (ie, Insulin, Meglitinides, Sulfonylureas, Quinolones, Angiotensin converting enzyme inhibitors (ACE-I), (B-blockers)
Critical Illness (ie, sepsis)
Malnourishment
Ethanol
Cortisol deficiency
Non-islet cell tumors
Endogenous hyperinsulinism (ie, Insulinoma, Insulin autoimmune hypoglycemia, beta cell secretagogue, non-insulinoma pancreatogenous hypoglycemia syndrome)
Accidental, malicious, or surreptitious intake of hypoglycemics
9

## Slide 10
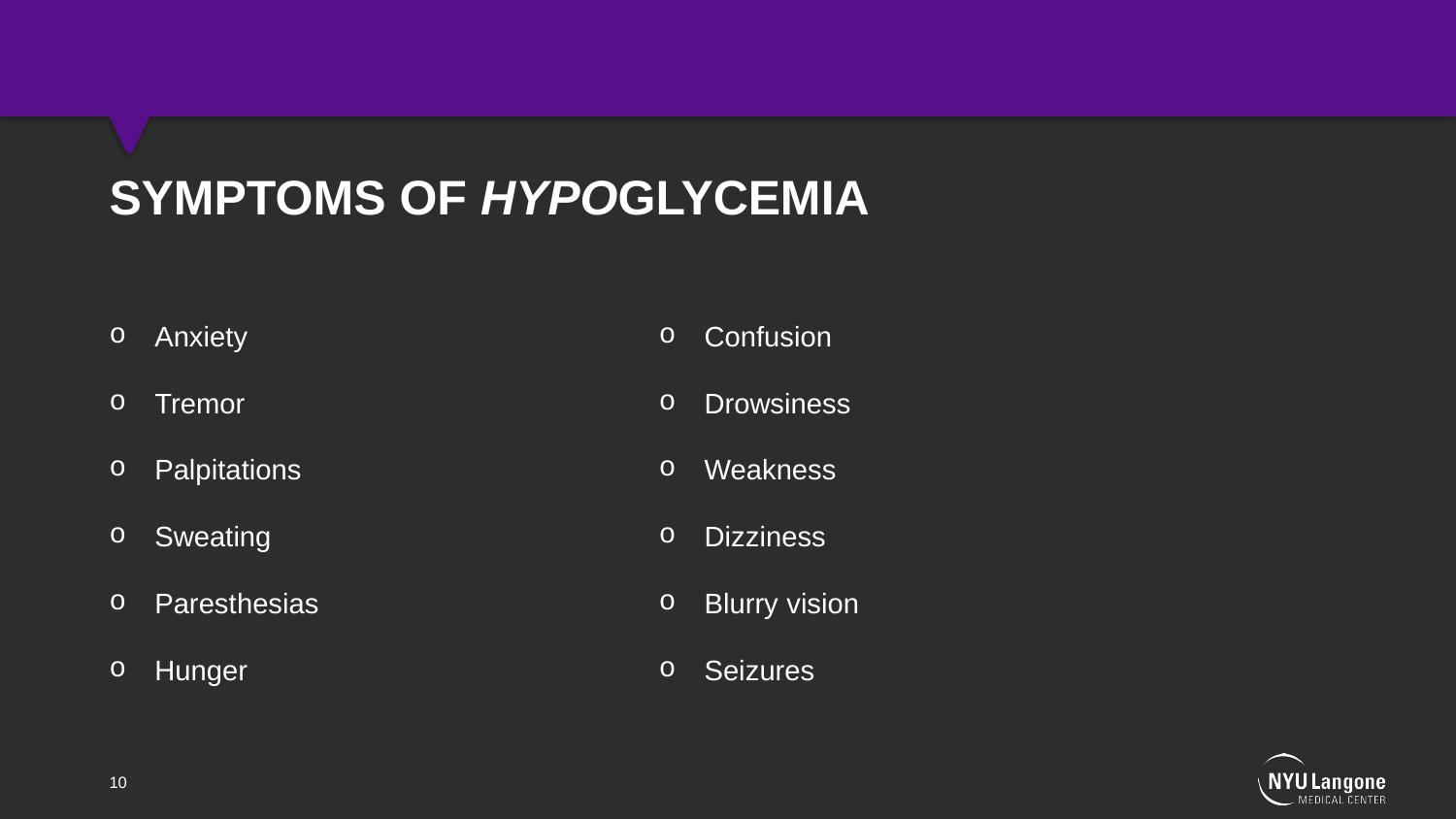

# SYMPTOMS OF HYPOGLYCEMIA
Anxiety
Tremor
Palpitations
Sweating
Paresthesias
Hunger
Confusion
Drowsiness
Weakness
Dizziness
Blurry vision
Seizures
10

## Slide 11
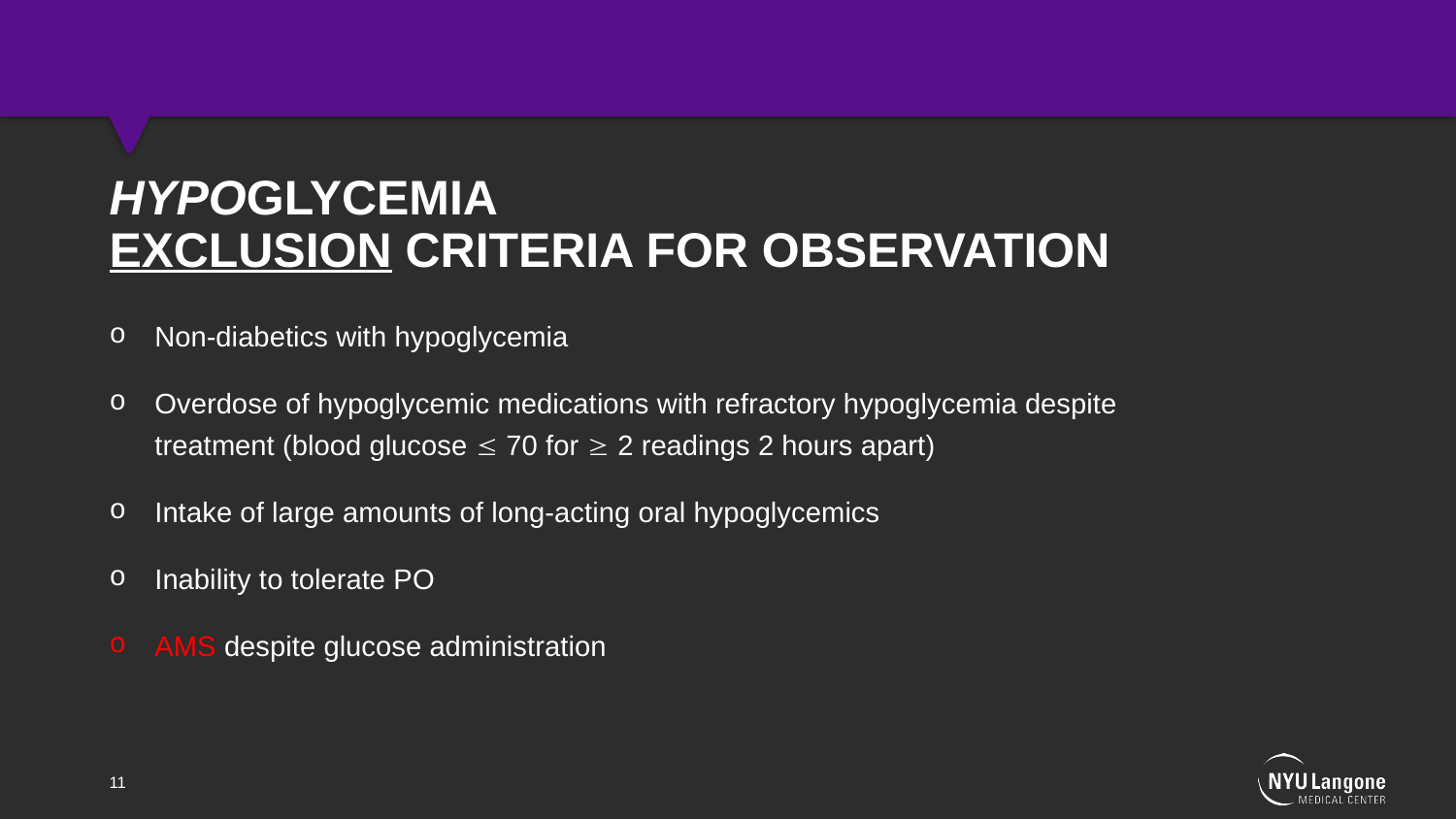

# HYPOGLYCEMIA EXCLUSION CRITERIA FOR OBSERVATION
Non-diabetics with hypoglycemia
Overdose of hypoglycemic medications with refractory hypoglycemia despite treatment (blood glucose ≤ 70 for ≥ 2 readings 2 hours apart)
Intake of large amounts of long-acting oral hypoglycemics
Inability to tolerate PO
AMS despite glucose administration
11

## Slide 12
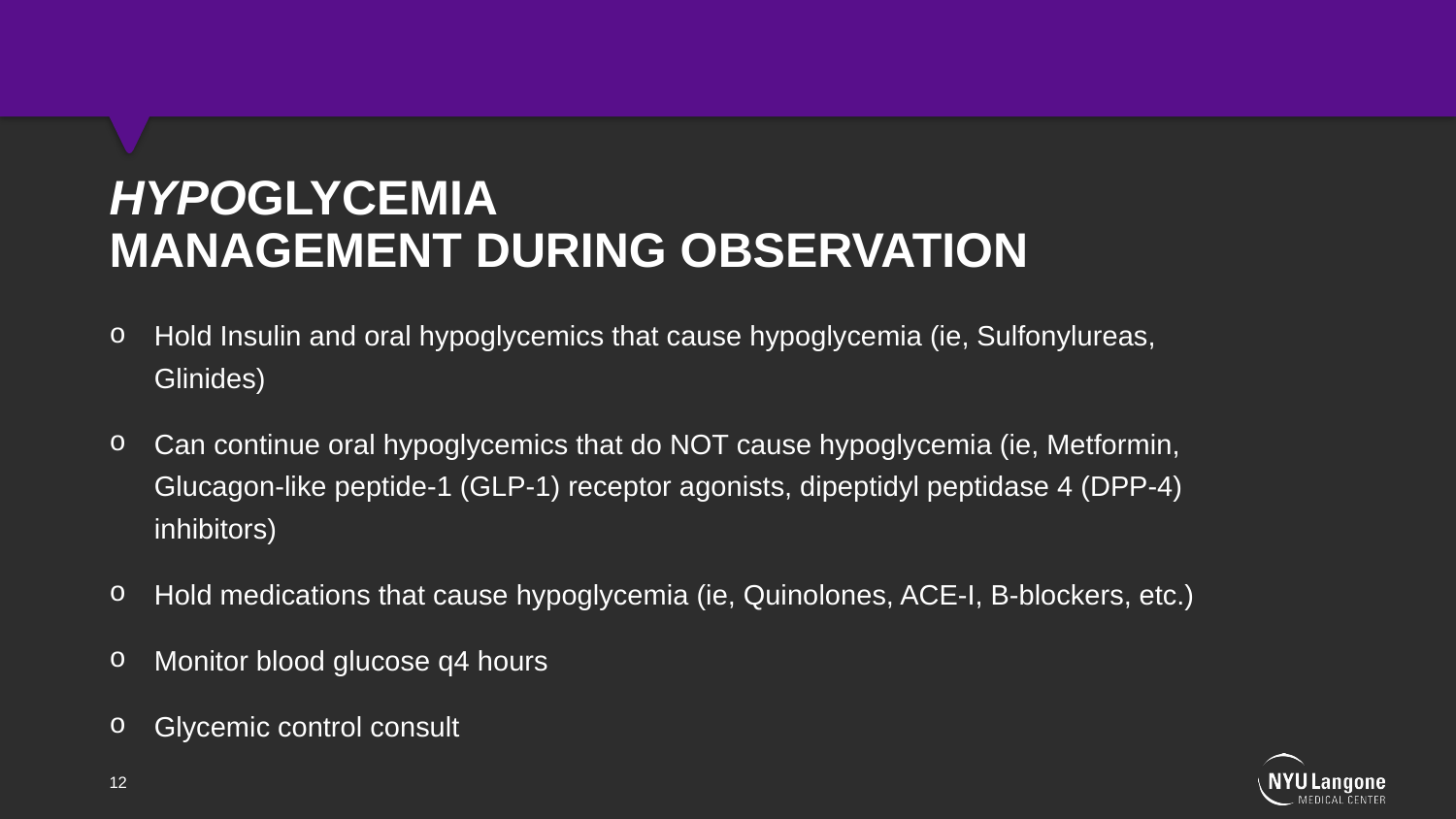

# HYPOGLYCEMIA MANAGEMENT DURING OBSERVATION
Hold Insulin and oral hypoglycemics that cause hypoglycemia (ie, Sulfonylureas, Glinides)
Can continue oral hypoglycemics that do NOT cause hypoglycemia (ie, Metformin, Glucagon-like peptide-1 (GLP-1) receptor agonists, dipeptidyl peptidase 4 (DPP-4) inhibitors)
Hold medications that cause hypoglycemia (ie, Quinolones, ACE-I, B-blockers, etc.)
Monitor blood glucose q4 hours
Glycemic control consult
12

## Slide 13
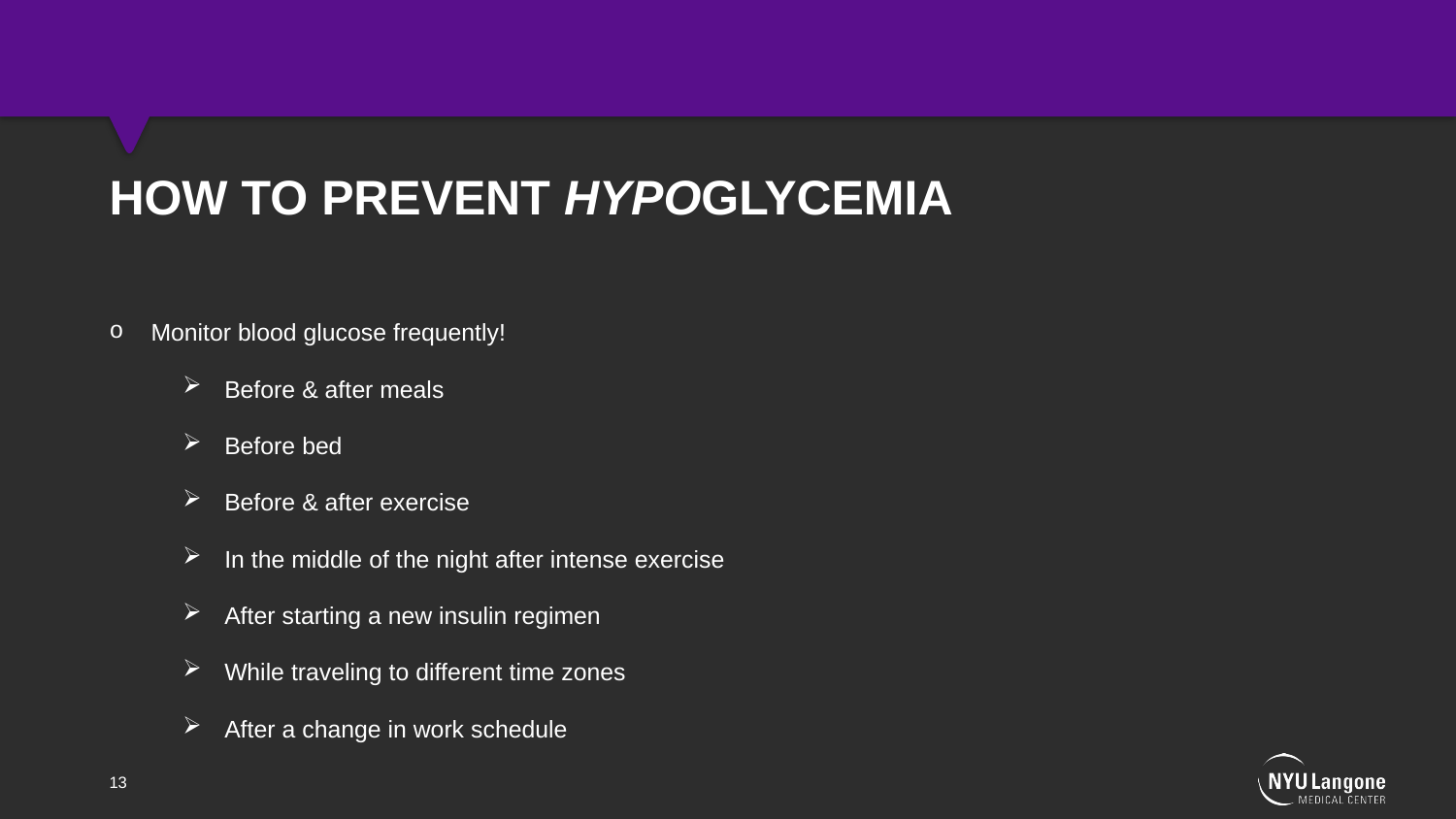

# HOW TO PREVENT HYPOGLYCEMIA
Monitor blood glucose frequently!
Before & after meals
Before bed
Before & after exercise
In the middle of the night after intense exercise
After starting a new insulin regimen
While traveling to different time zones
After a change in work schedule
13

## Slide 14
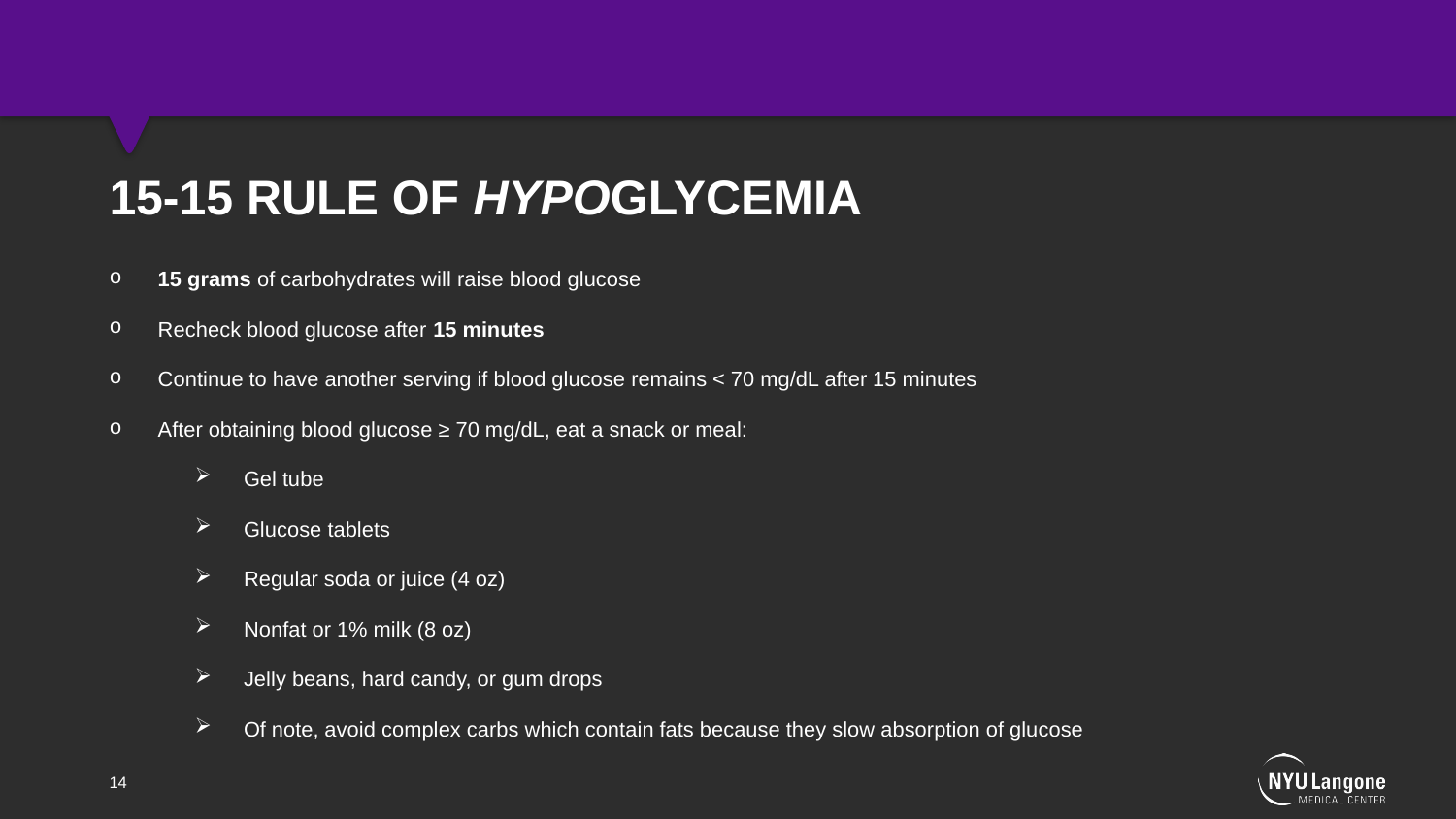

# 15-15 RULE OF HYPOGLYCEMIA
15 grams of carbohydrates will raise blood glucose
Recheck blood glucose after 15 minutes
Continue to have another serving if blood glucose remains < 70 mg/dL after 15 minutes
After obtaining blood glucose ≥ 70 mg/dL, eat a snack or meal:
Gel tube
Glucose tablets
Regular soda or juice (4 oz)
Nonfat or 1% milk (8 oz)
Jelly beans, hard candy, or gum drops
Of note, avoid complex carbs which contain fats because they slow absorption of glucose
14

## Slide 15
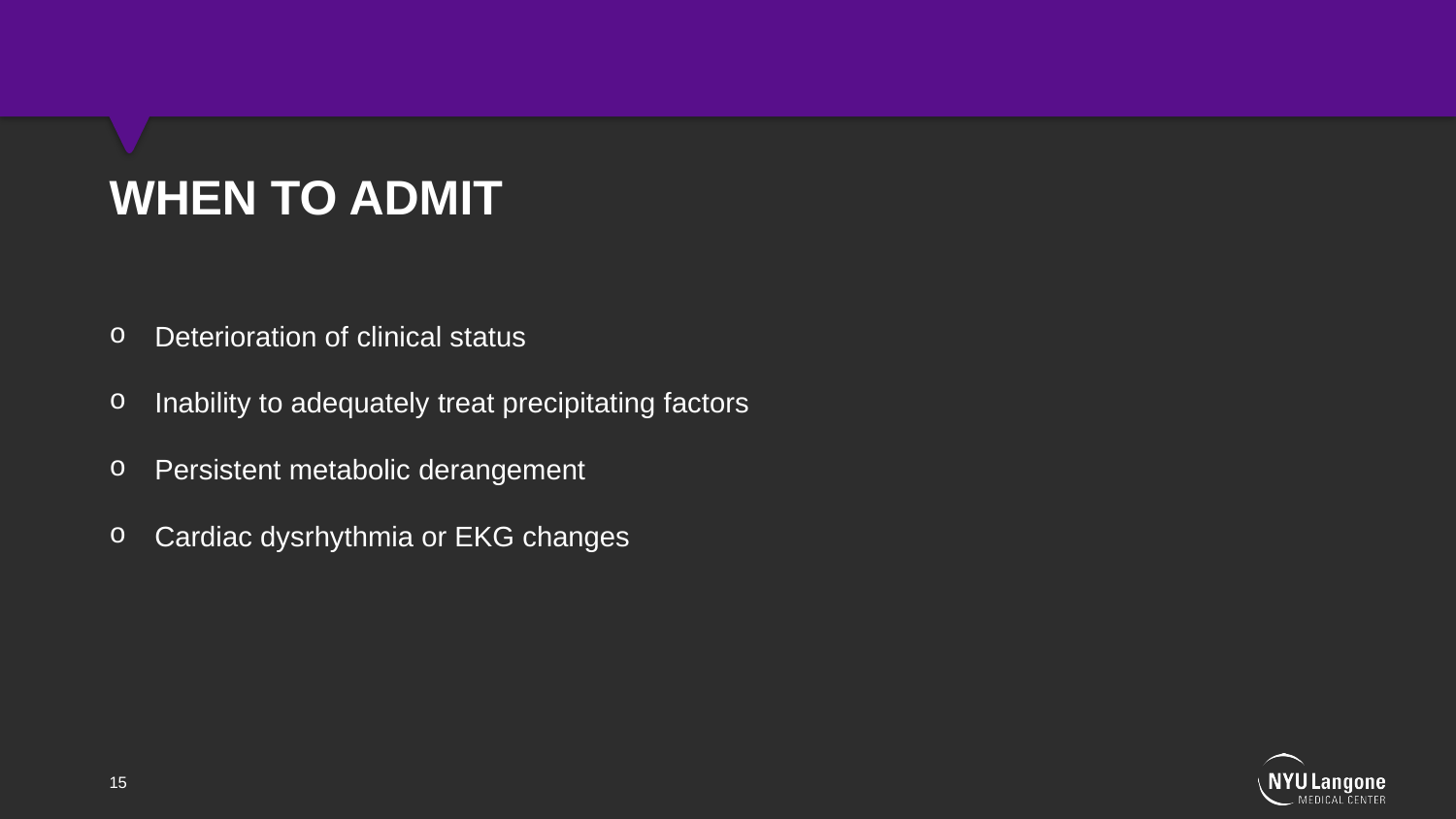

# WHEN TO ADMIT
Deterioration of clinical status
Inability to adequately treat precipitating factors
Persistent metabolic derangement
Cardiac dysrhythmia or EKG changes
15

## Slide 16
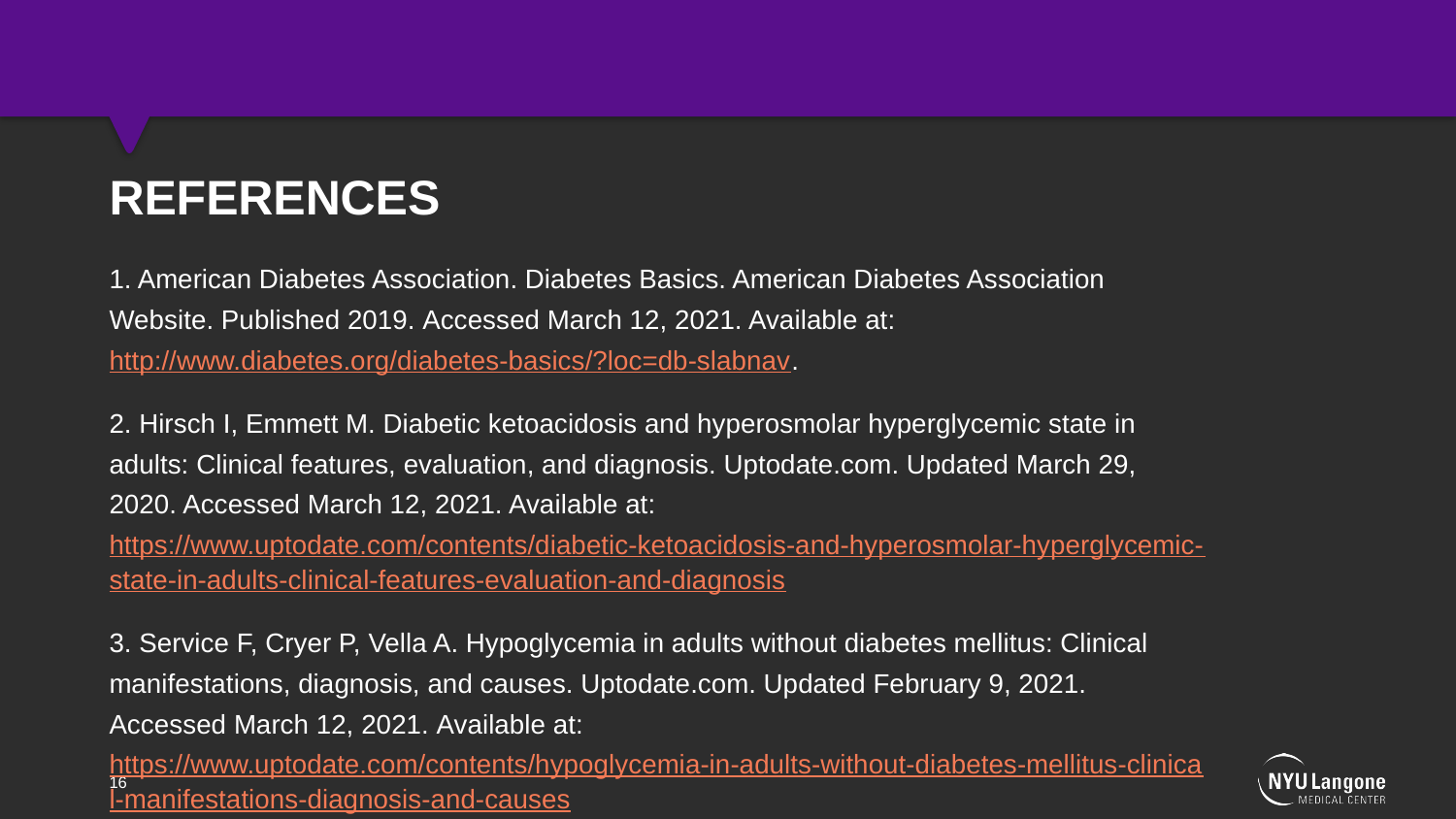

# REFERENCES
1. American Diabetes Association. Diabetes Basics. American Diabetes Association Website. Published 2019. Accessed March 12, 2021. Available at: http://www.diabetes.org/diabetes-basics/?loc=db-slabnav.
2. Hirsch I, Emmett M. Diabetic ketoacidosis and hyperosmolar hyperglycemic state in adults: Clinical features, evaluation, and diagnosis. Uptodate.com. Updated March 29, 2020. Accessed March 12, 2021. Available at: https://www.uptodate.com/contents/diabetic-ketoacidosis-and-hyperosmolar-hyperglycemic-state-in-adults-clinical-features-evaluation-and-diagnosis
3. Service F, Cryer P, Vella A. Hypoglycemia in adults without diabetes mellitus: Clinical manifestations, diagnosis, and causes. Uptodate.com. Updated February 9, 2021. Accessed March 12, 2021. Available at: https://www.uptodate.com/contents/hypoglycemia-in-adults-without-diabetes-mellitus-clinical-manifestations-diagnosis-and-causes
16
