# Supplementary material for: An Observation Medicine Curriculum for Emergency Medicine Education
Source: J Educ Teach Emerg Med. 2021 Apr 19;6(2):C1–C72. doi: 10.21980/J87P92 (PMC10332786; doi:10.21980/J87P92)
Supplement: Supplementary file 24 — Please see associated PowerPoint file [file jetem-6-2-c1-supp24.pptx]

## Slide 1
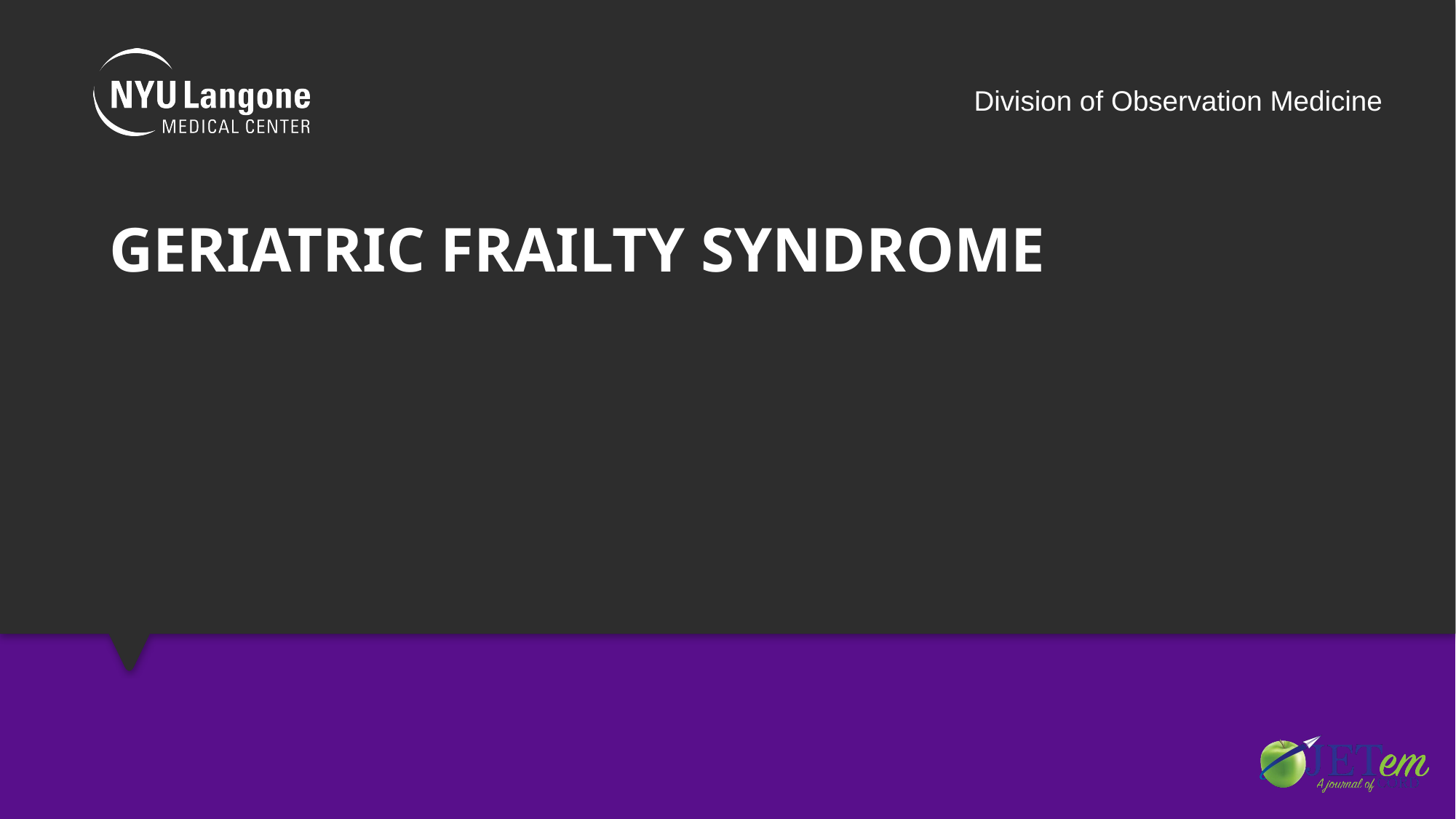

Division of Observation Medicine
# Geriatric Frailty Syndrome​

## Slide 2
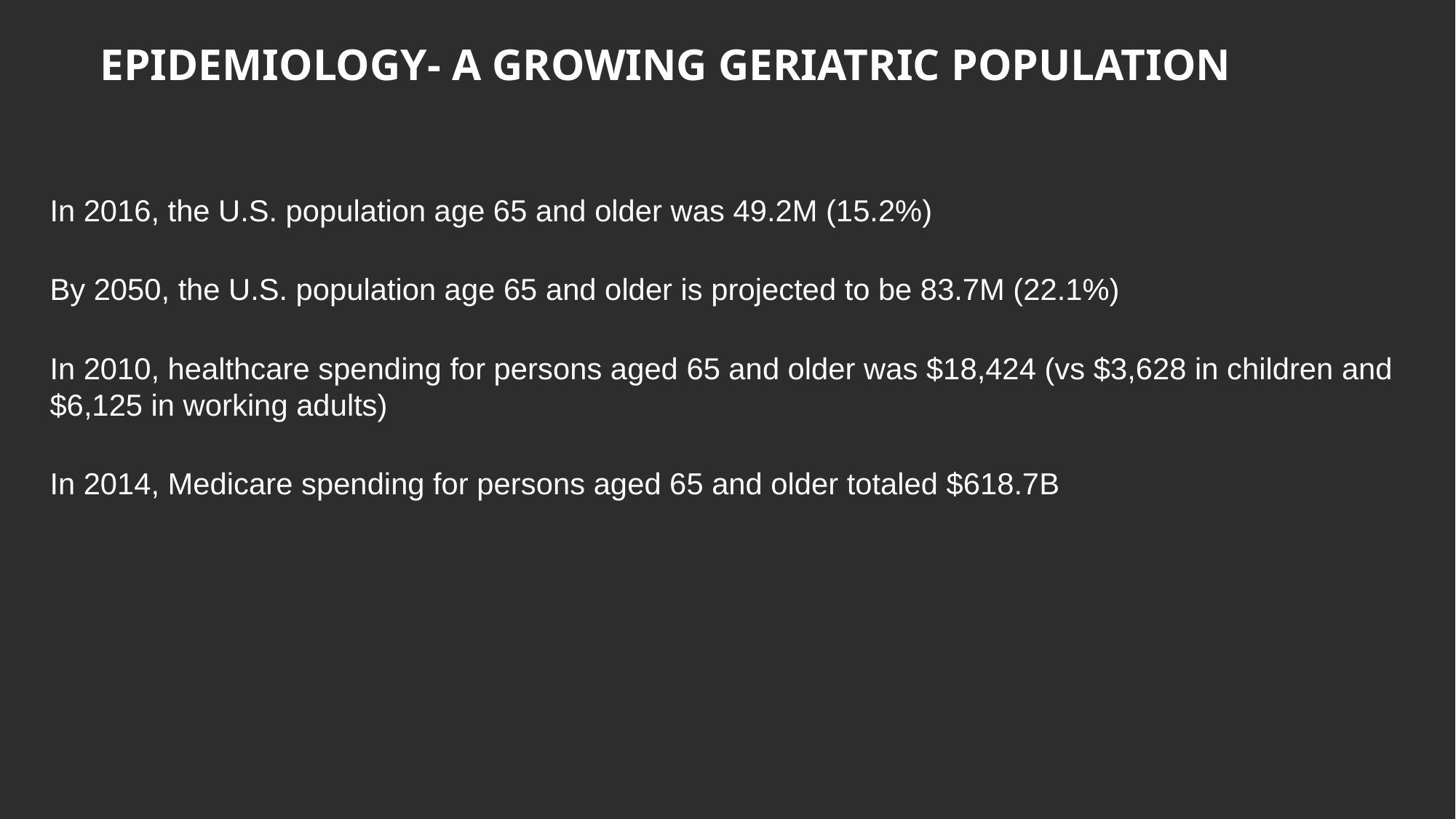

# Epidemiology- A Growing Geriatric Population
In 2016, the U.S. population age 65 and older was 49.2M (15.2%)
By 2050, the U.S. population age 65 and older is projected to be 83.7M (22.1%)
In 2010, healthcare spending for persons aged 65 and older was $18,424 (vs $3,628 in children and $6,125 in working adults)
In 2014, Medicare spending for persons aged 65 and older totaled $618.7B

## Slide 3
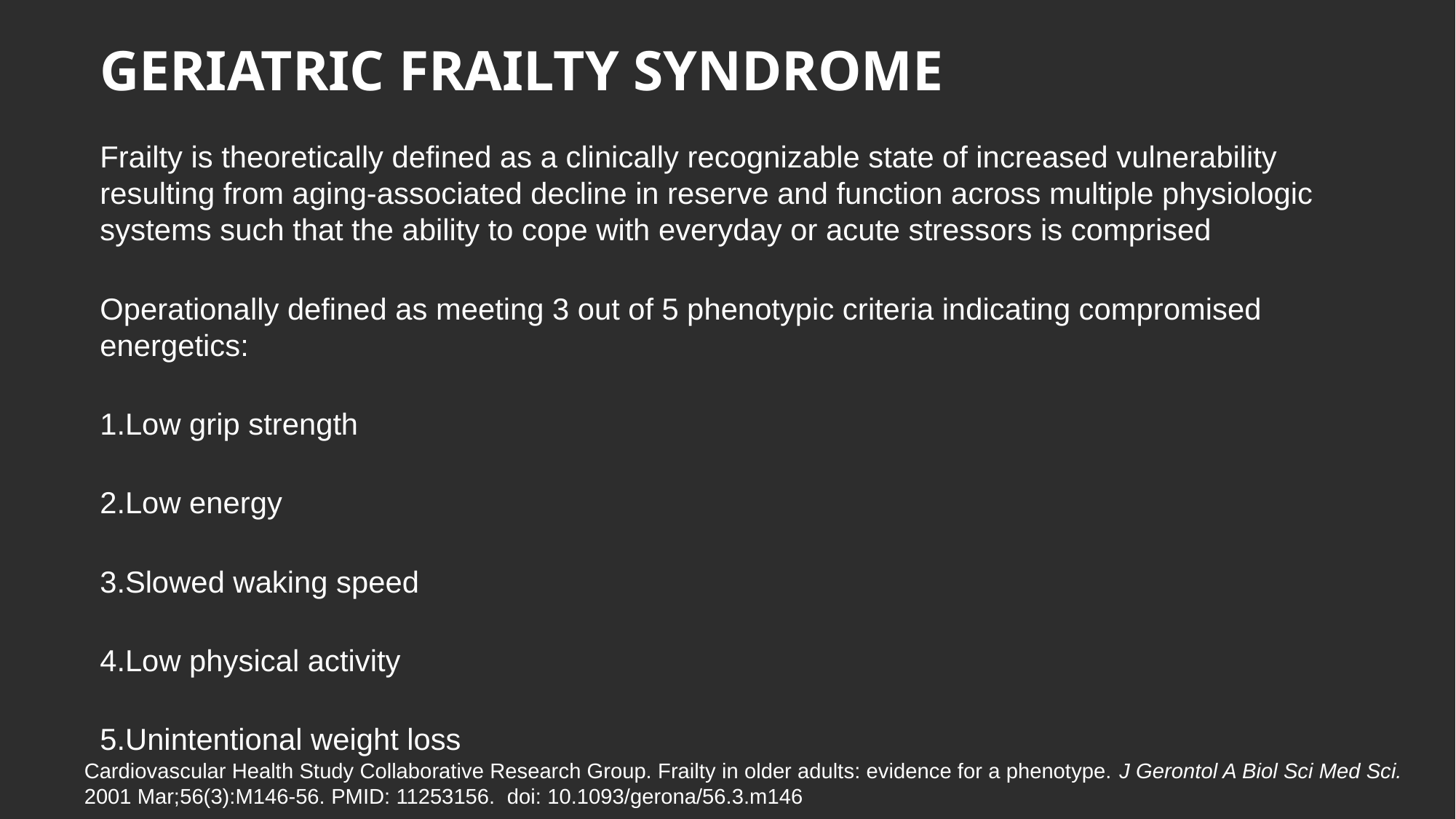

# Geriatric Frailty Syndrome
Frailty is theoretically defined as a clinically recognizable state of increased vulnerability resulting from aging-associated decline in reserve and function across multiple physiologic systems such that the ability to cope with everyday or acute stressors is comprised
Operationally defined as meeting 3 out of 5 phenotypic criteria indicating compromised energetics:
Low grip strength
Low energy
Slowed waking speed
Low physical activity
Unintentional weight loss
Cardiovascular Health Study Collaborative Research Group. Frailty in older adults: evidence for a phenotype. J Gerontol A Biol Sci Med Sci. 2001 Mar;56(3):M146-56. PMID: 11253156. doi: 10.1093/gerona/56.3.m146

## Slide 4
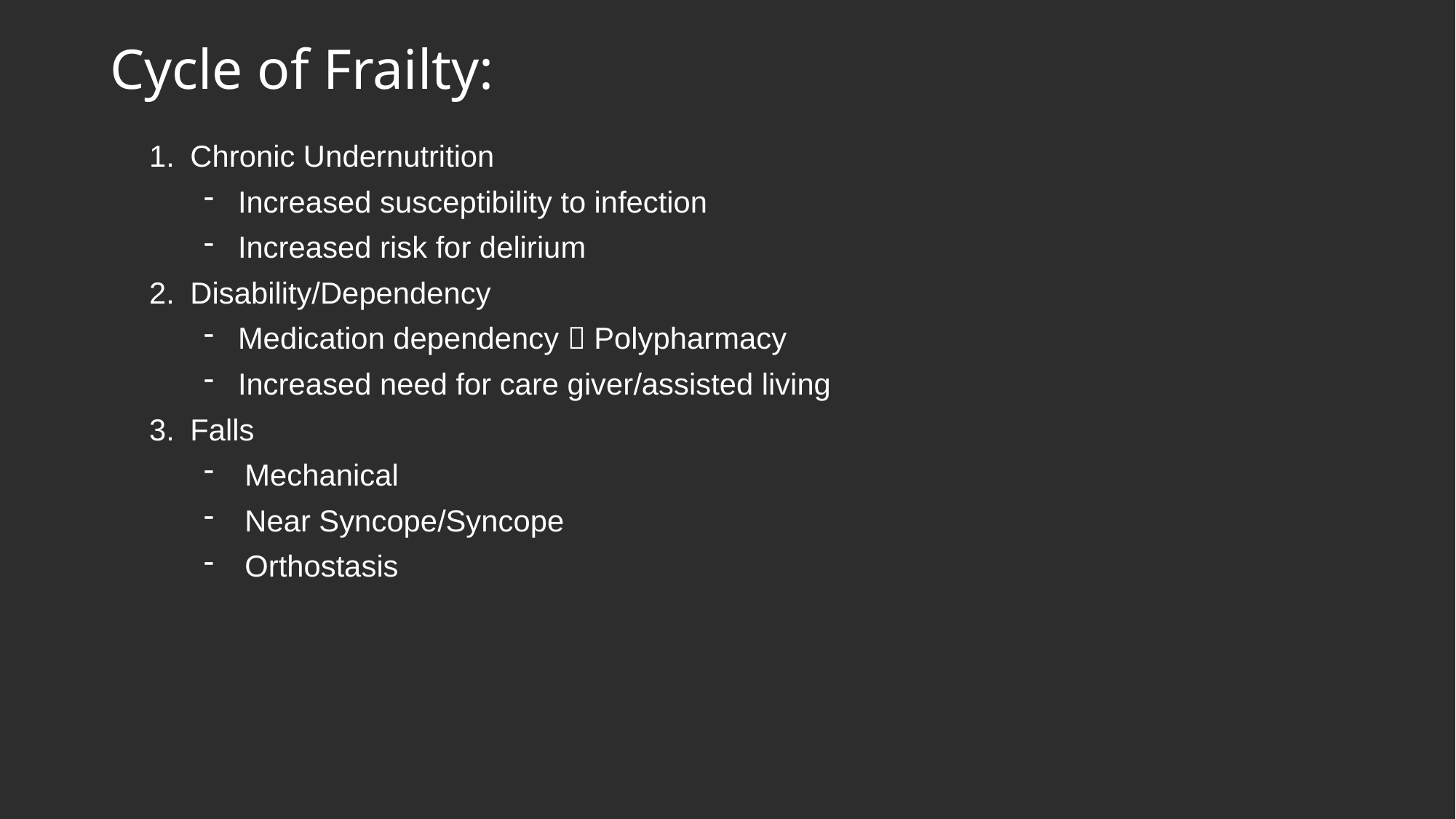

Cycle of Frailty:
Chronic Undernutrition
Increased susceptibility to infection
Increased risk for delirium
Disability/Dependency
Medication dependency  Polypharmacy
Increased need for care giver/assisted living
Falls
Mechanical
Near Syncope/Syncope
Orthostasis

## Slide 5
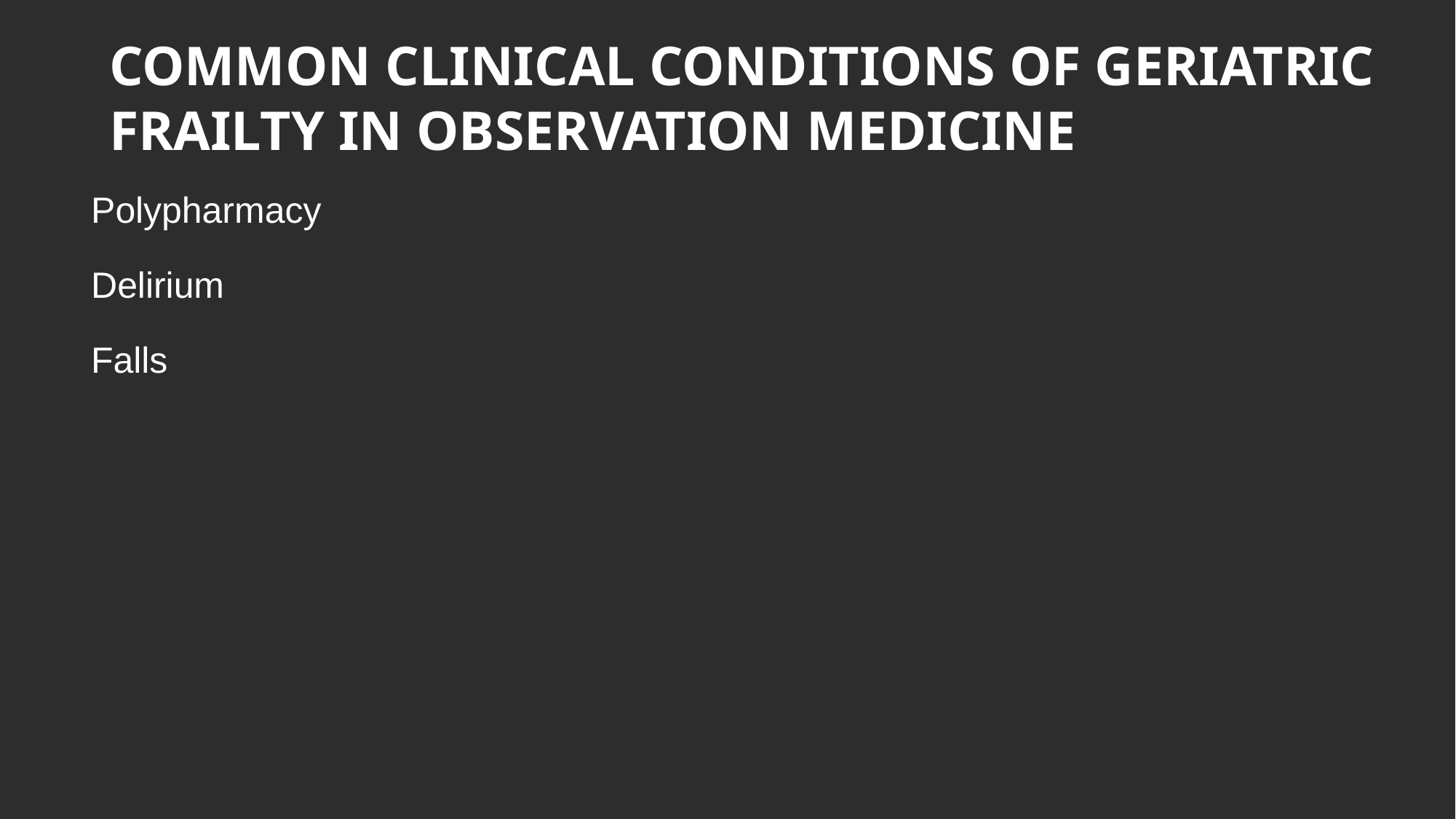

# Common Clinical Conditions of Geriatric Frailty in Observation Medicine
Polypharmacy
Delirium
Falls

## Slide 6
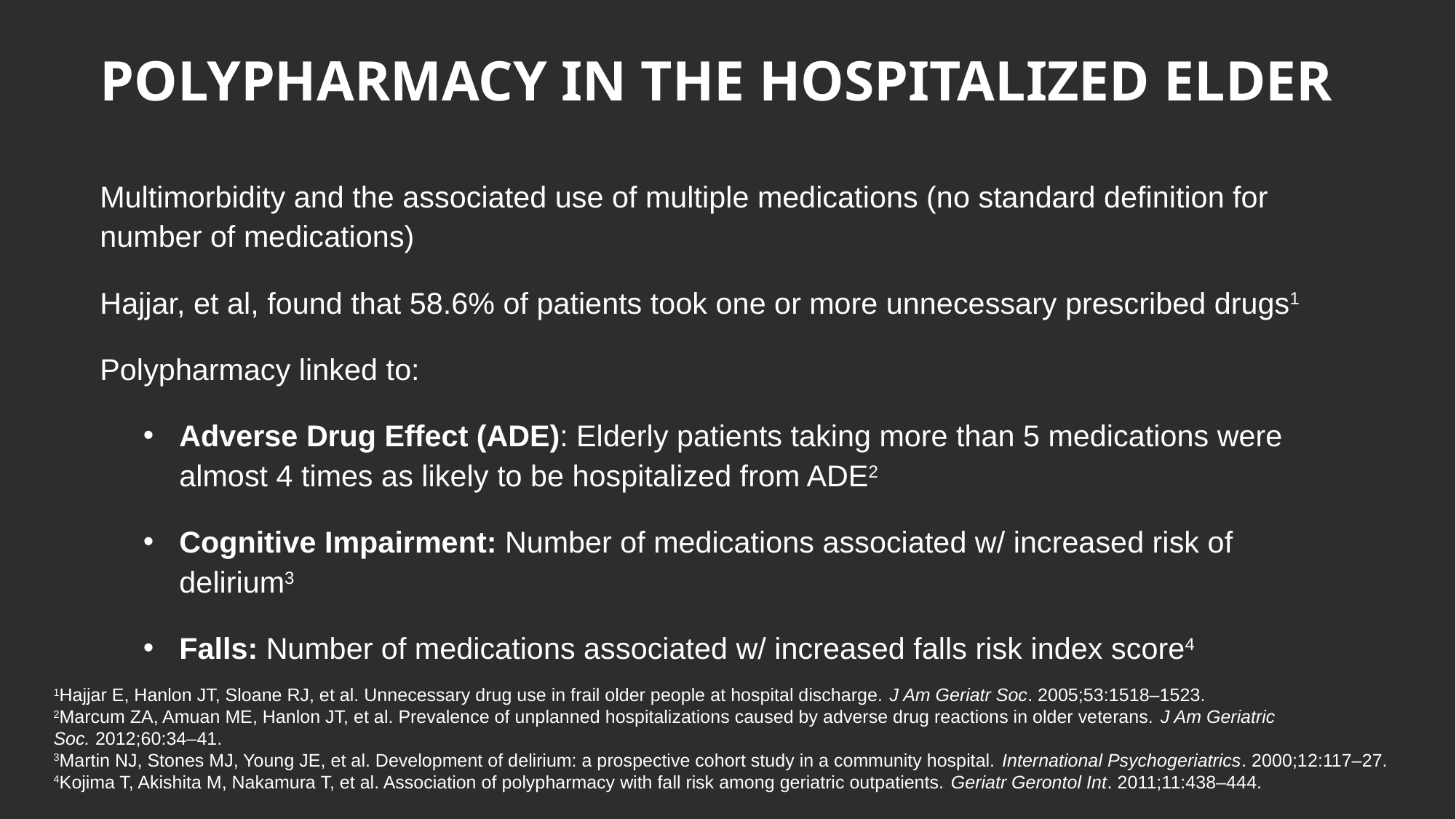

# Polypharmacy in the Hospitalized Elder
Multimorbidity and the associated use of multiple medications (no standard definition for number of medications)
Hajjar, et al, found that 58.6% of patients took one or more unnecessary prescribed drugs1
Polypharmacy linked to:
Adverse Drug Effect (ADE): Elderly patients taking more than 5 medications were almost 4 times as likely to be hospitalized from ADE2
Cognitive Impairment: Number of medications associated w/ increased risk of delirium3
Falls: Number of medications associated w/ increased falls risk index score4
1Hajjar E, Hanlon JT, Sloane RJ, et al. Unnecessary drug use in frail older people at hospital discharge. J Am Geriatr Soc. 2005;53:1518–1523.
2Marcum ZA, Amuan ME, Hanlon JT, et al. Prevalence of unplanned hospitalizations caused by adverse drug reactions in older veterans. J Am Geriatric Soc. 2012;60:34–41.
3Martin NJ, Stones MJ, Young JE, et al. Development of delirium: a prospective cohort study in a community hospital. International Psychogeriatrics. 2000;12:117–27.
4Kojima T, Akishita M, Nakamura T, et al. Association of polypharmacy with fall risk among geriatric outpatients. Geriatr Gerontol Int. 2011;11:438–444.

## Slide 7
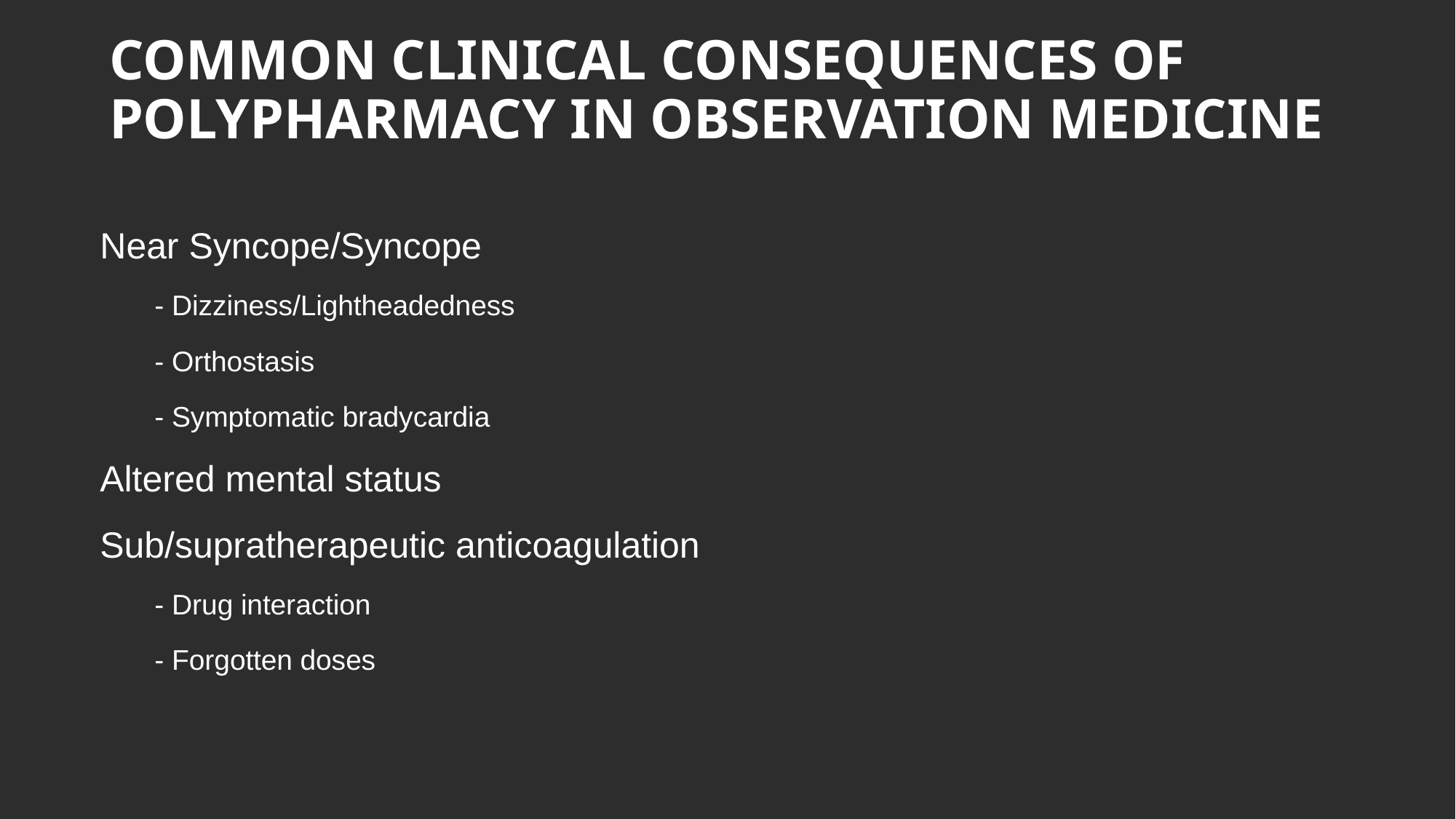

# Common Clinical Consequences of Polypharmacy in Observation Medicine
Near Syncope/Syncope
- Dizziness/Lightheadedness
- Orthostasis
- Symptomatic bradycardia
Altered mental status
Sub/supratherapeutic anticoagulation
- Drug interaction
- Forgotten doses

## Slide 8
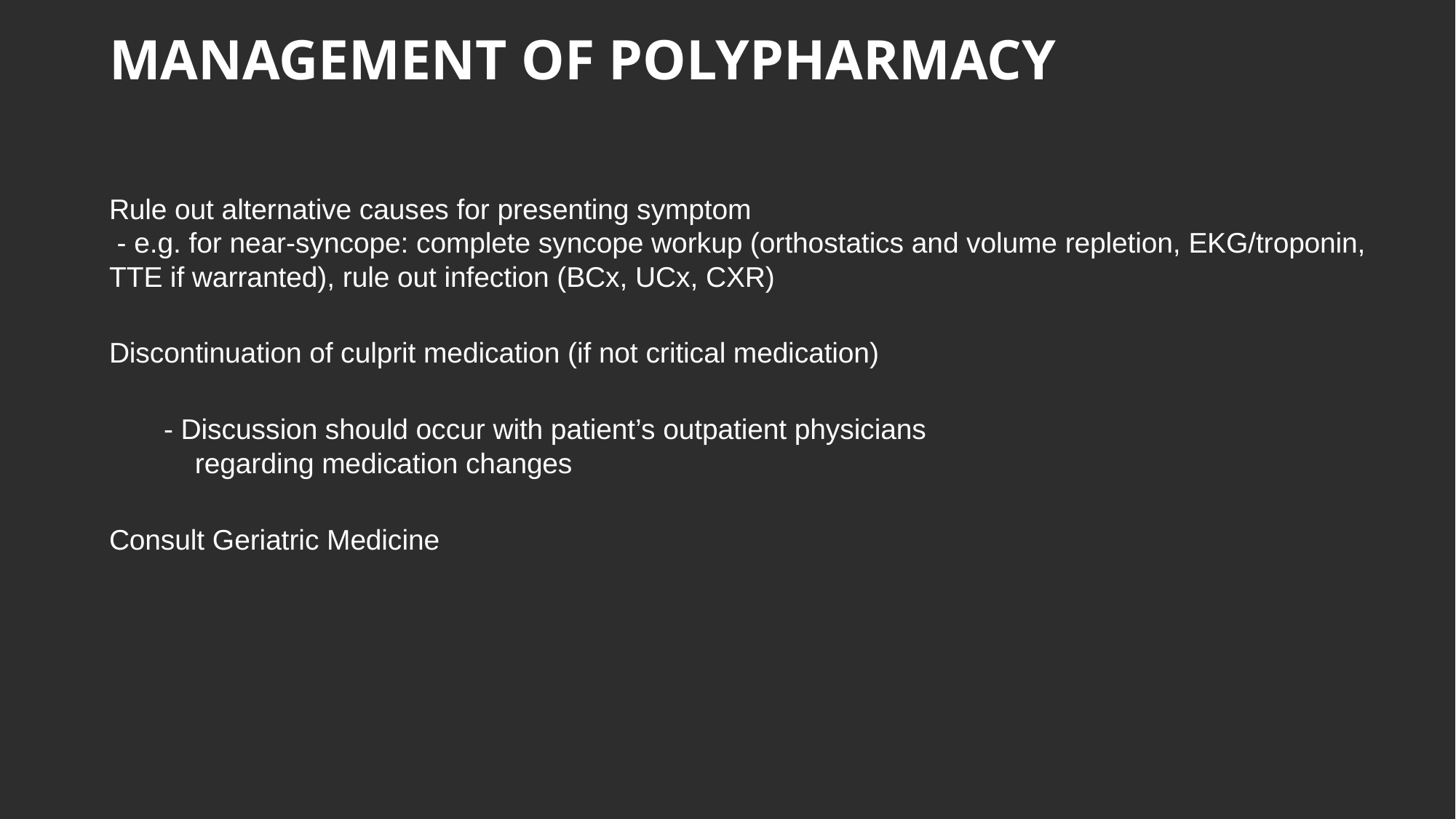

# Management of Polypharmacy
Rule out alternative causes for presenting symptom
 - e.g. for near-syncope: complete syncope workup (orthostatics and volume repletion, EKG/troponin, TTE if warranted), rule out infection (BCx, UCx, CXR)
Discontinuation of culprit medication (if not critical medication)
- Discussion should occur with patient’s outpatient physicians
 regarding medication changes
Consult Geriatric Medicine

## Slide 9
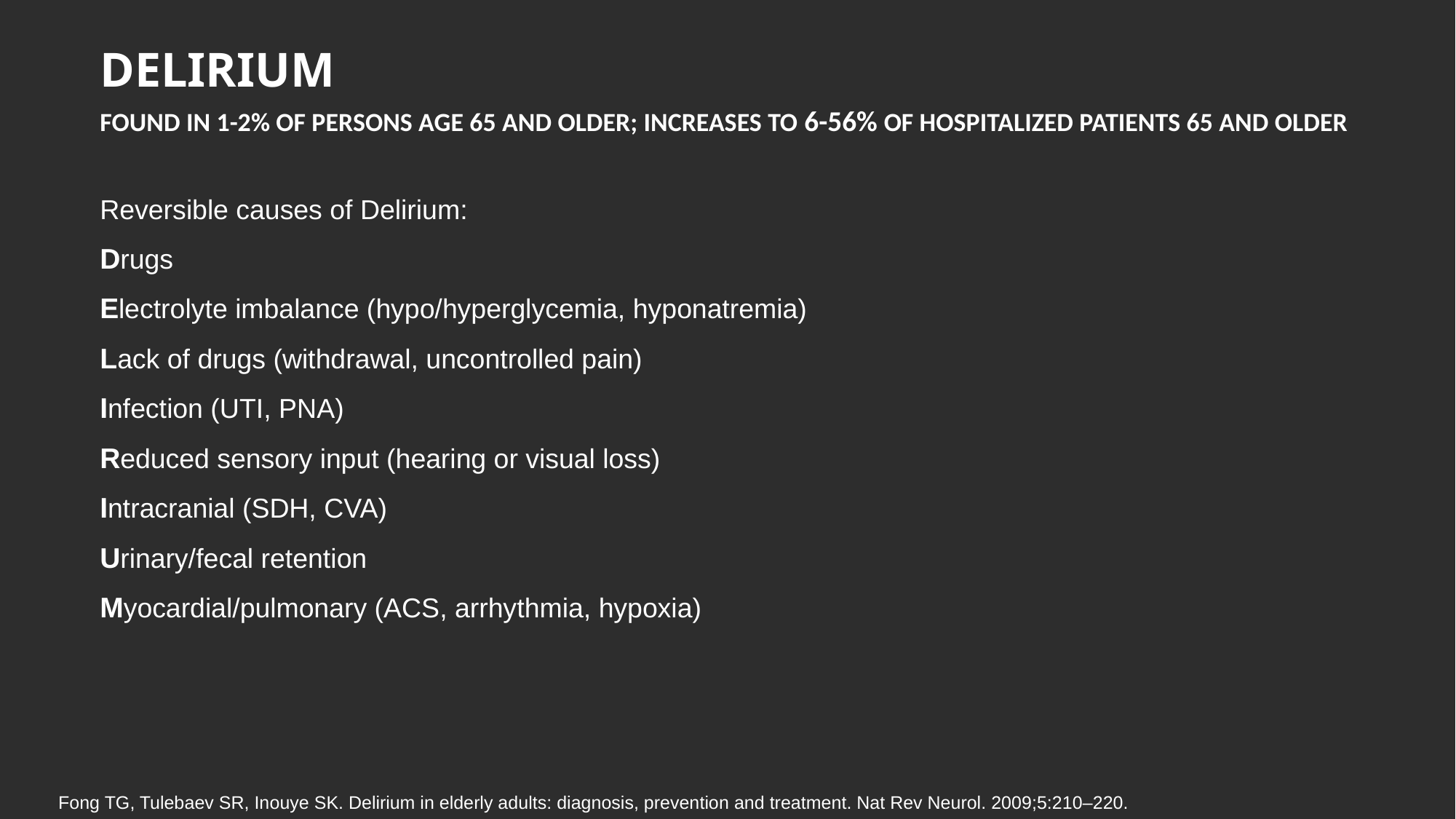

# Delirium Found in 1-2% of persons age 65 and older; increases to 6-56% of hospitalized patients 65 and older
Reversible causes of Delirium:
Drugs
Electrolyte imbalance (hypo/hyperglycemia, hyponatremia)
Lack of drugs (withdrawal, uncontrolled pain)
Infection (UTI, PNA)
Reduced sensory input (hearing or visual loss)
Intracranial (SDH, CVA)
Urinary/fecal retention
Myocardial/pulmonary (ACS, arrhythmia, hypoxia)
Fong TG, Tulebaev SR, Inouye SK. Delirium in elderly adults: diagnosis, prevention and treatment. Nat Rev Neurol. 2009;5:210–220.

## Slide 10
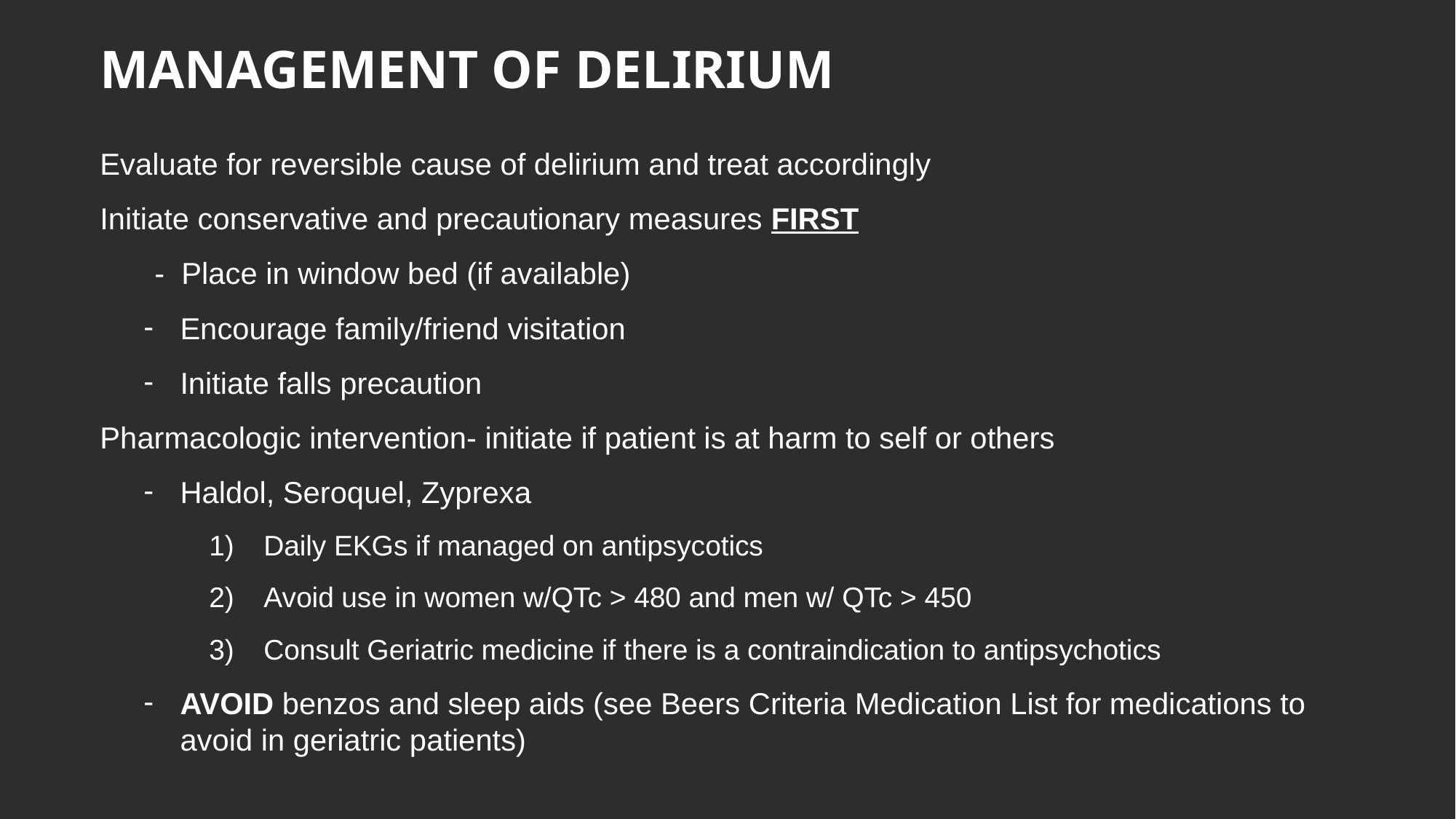

# Management of Delirium
Evaluate for reversible cause of delirium and treat accordingly
Initiate conservative and precautionary measures FIRST
- Place in window bed (if available)
Encourage family/friend visitation
Initiate falls precaution
Pharmacologic intervention- initiate if patient is at harm to self or others
Haldol, Seroquel, Zyprexa
Daily EKGs if managed on antipsycotics
Avoid use in women w/QTc > 480 and men w/ QTc > 450
Consult Geriatric medicine if there is a contraindication to antipsychotics
AVOID benzos and sleep aids (see Beers Criteria Medication List for medications to avoid in geriatric patients)

## Slide 11
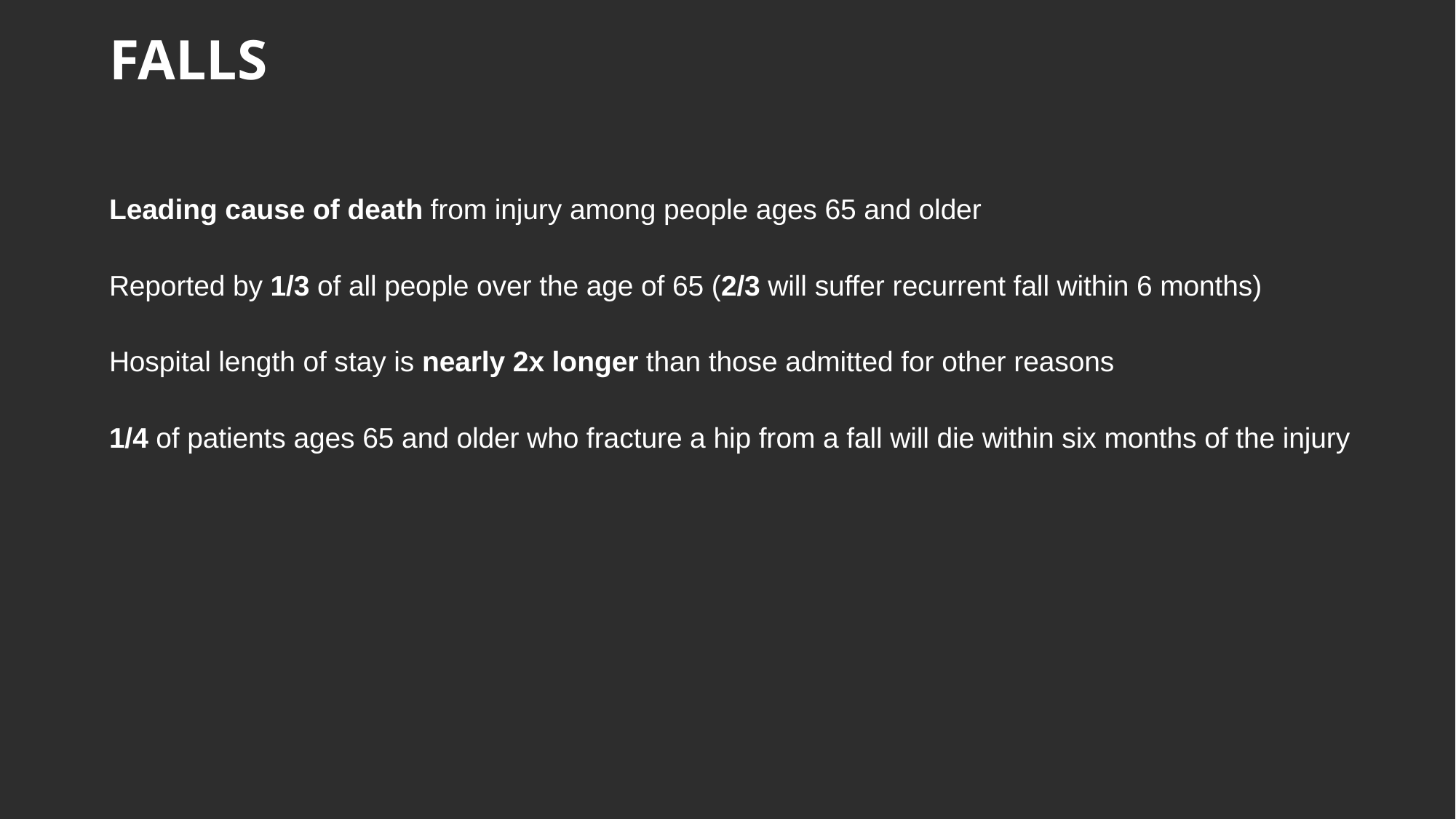

# Falls
Leading cause of death from injury among people ages 65 and older
Reported by 1/3 of all people over the age of 65 (2/3 will suffer recurrent fall within 6 months)
Hospital length of stay is nearly 2x longer than those admitted for other reasons
1/4 of patients ages 65 and older who fracture a hip from a fall will die within six months of the injury

## Slide 12
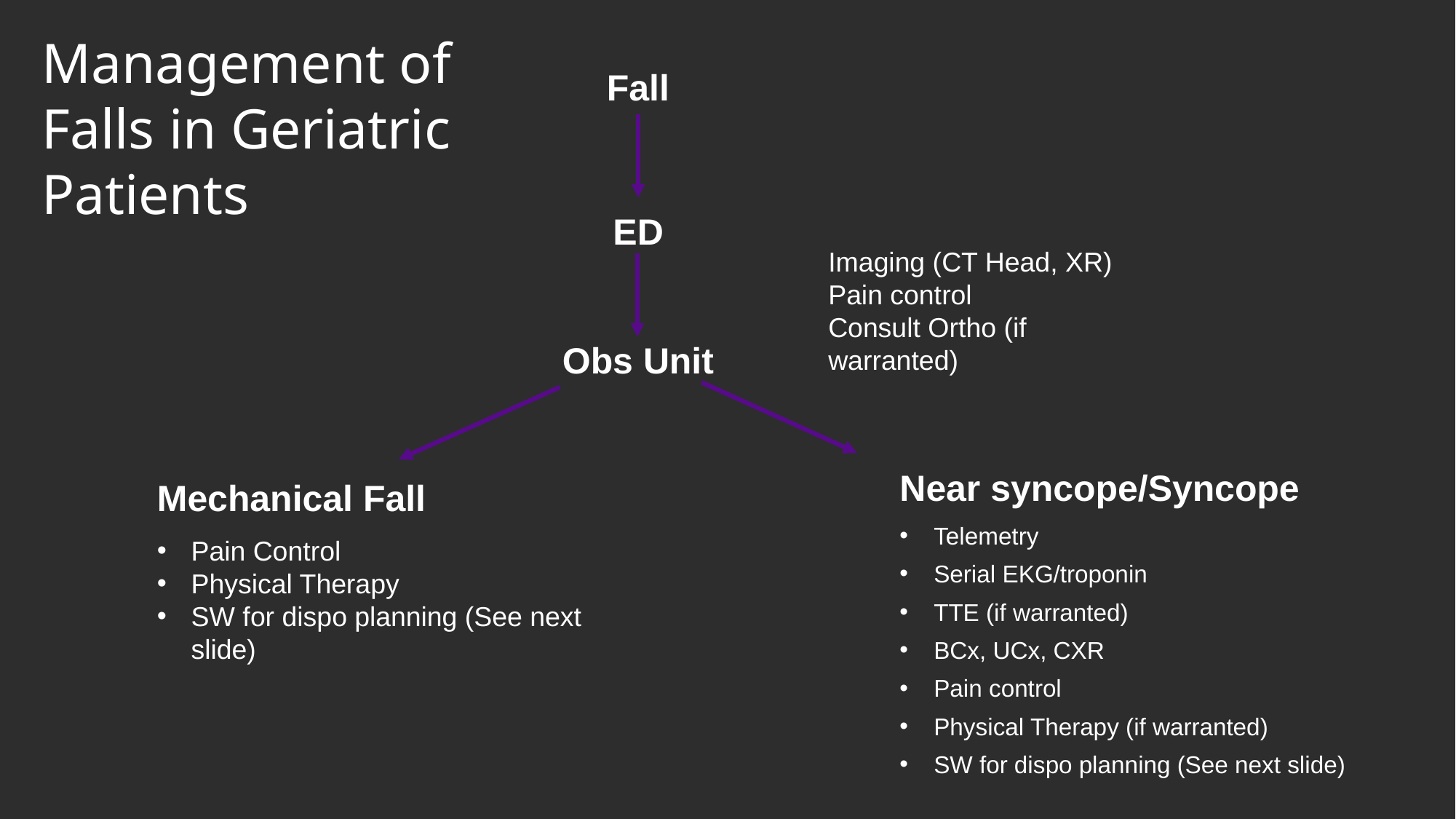

Management of Falls in Geriatric Patients
Fall
ED
Imaging (CT Head, XR)
Pain control
Consult Ortho (if warranted)
Obs Unit
Near syncope/Syncope
Mechanical Fall
Telemetry
Serial EKG/troponin
TTE (if warranted)
BCx, UCx, CXR
Pain control
Physical Therapy (if warranted)
SW for dispo planning (See next slide)
Pain Control
Physical Therapy
SW for dispo planning (See next slide)

## Slide 13
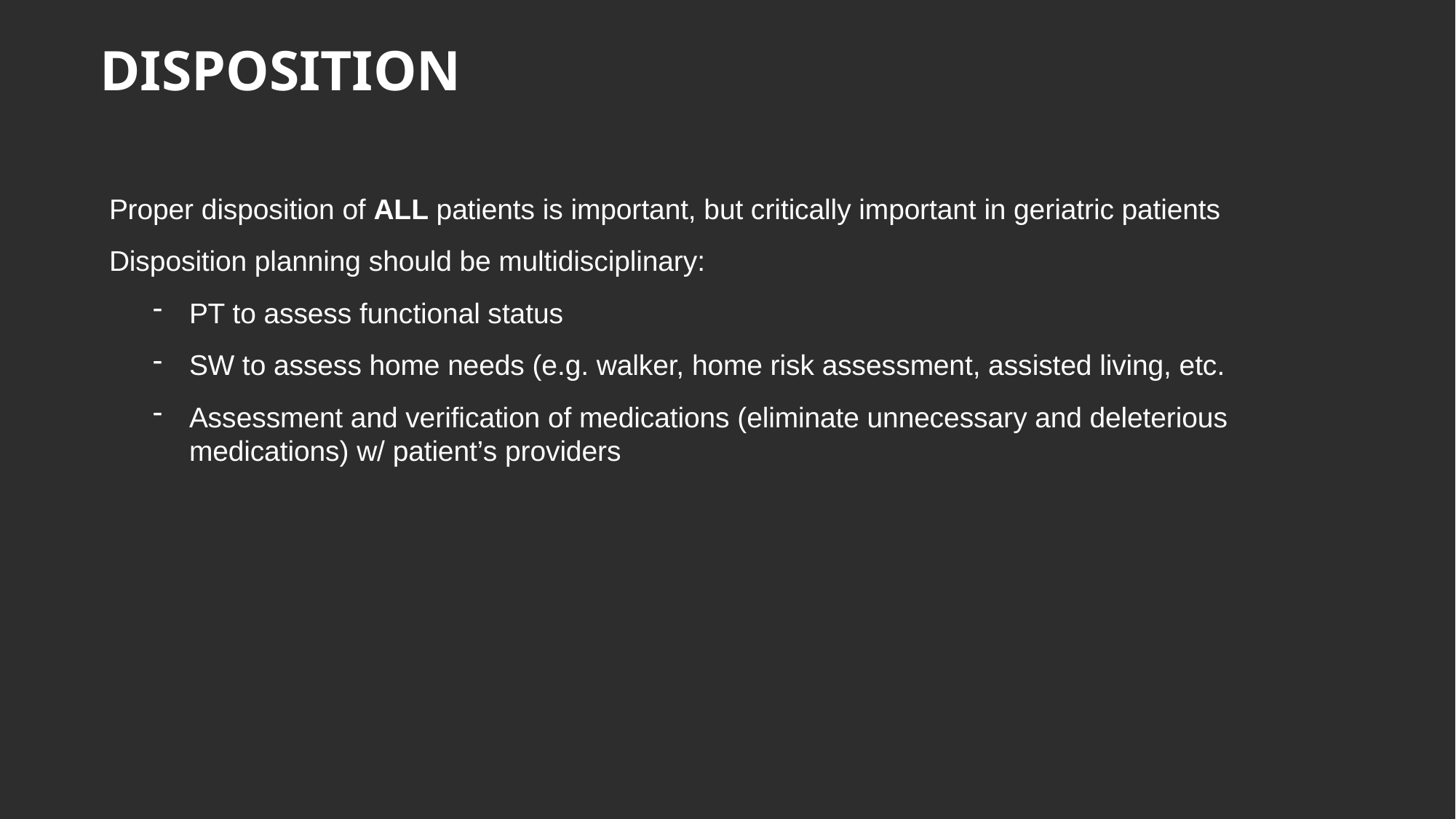

# Disposition
Proper disposition of ALL patients is important, but critically important in geriatric patients
Disposition planning should be multidisciplinary:
PT to assess functional status
SW to assess home needs (e.g. walker, home risk assessment, assisted living, etc.
Assessment and verification of medications (eliminate unnecessary and deleterious medications) w/ patient’s providers

## Slide 14
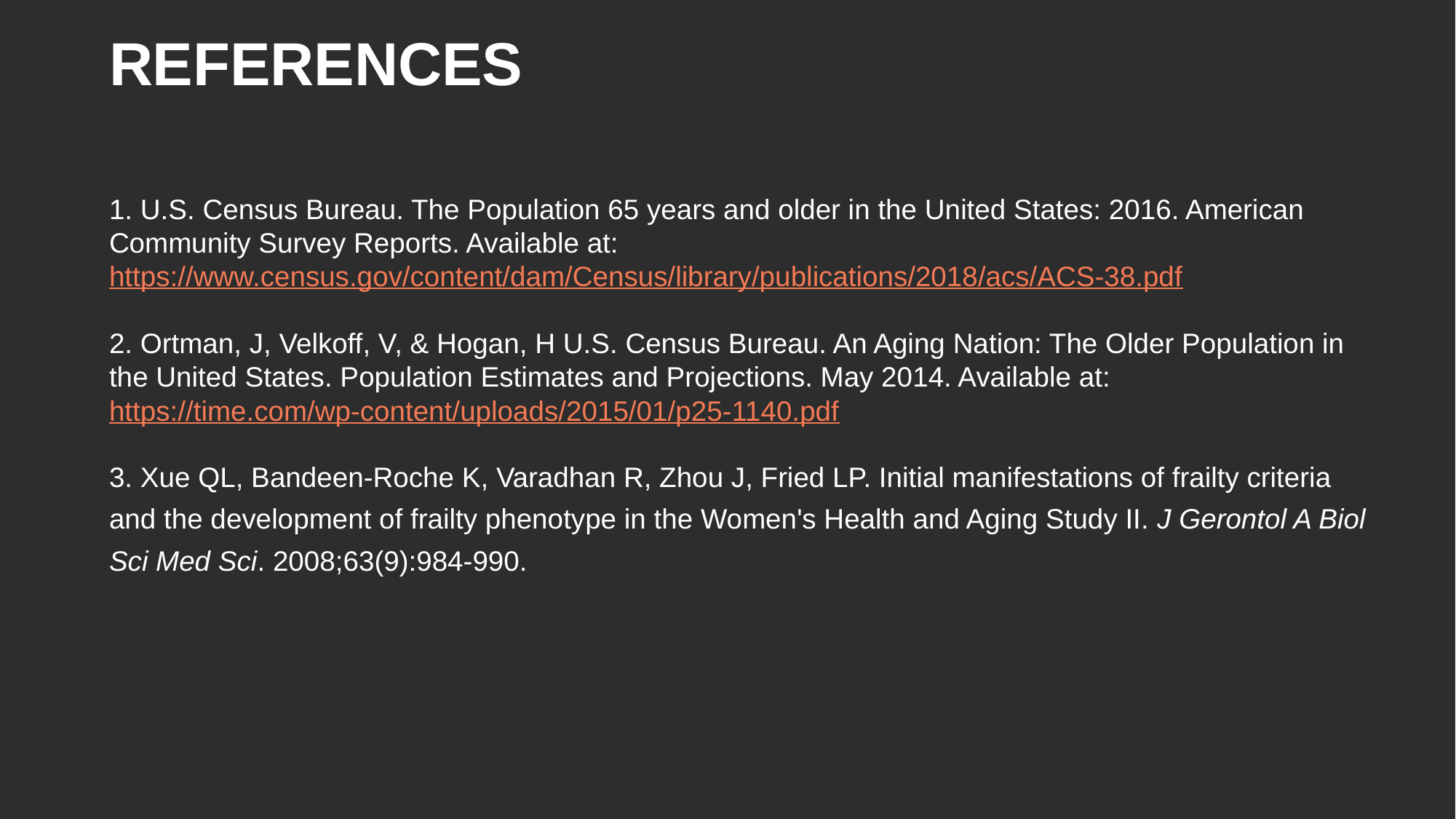

# references
1. U.S. Census Bureau. The Population 65 years and older in the United States: 2016. American Community Survey Reports. Available at: https://www.census.gov/content/dam/Census/library/publications/2018/acs/ACS-38.pdf
2. Ortman, J, Velkoff, V, & Hogan, H U.S. Census Bureau. An Aging Nation: The Older Population in the United States. Population Estimates and Projections. May 2014. Available at: https://time.com/wp-content/uploads/2015/01/p25-1140.pdf
3. Xue QL, Bandeen-Roche K, Varadhan R, Zhou J, Fried LP. Initial manifestations of frailty criteria and the development of frailty phenotype in the Women's Health and Aging Study II. J Gerontol A Biol Sci Med Sci. 2008;63(9):984-990.
