# Supplementary material for: An Observation Medicine Curriculum for Emergency Medicine Education
Source: J Educ Teach Emerg Med. 2021 Apr 19;6(2):C1–C72. doi: 10.21980/J87P92 (PMC10332786; doi:10.21980/J87P92)
Supplement: Supplementary file 25 — Please see associated PowerPoint file [file jetem-6-2-c1-supp25.pptx]

## Slide 1
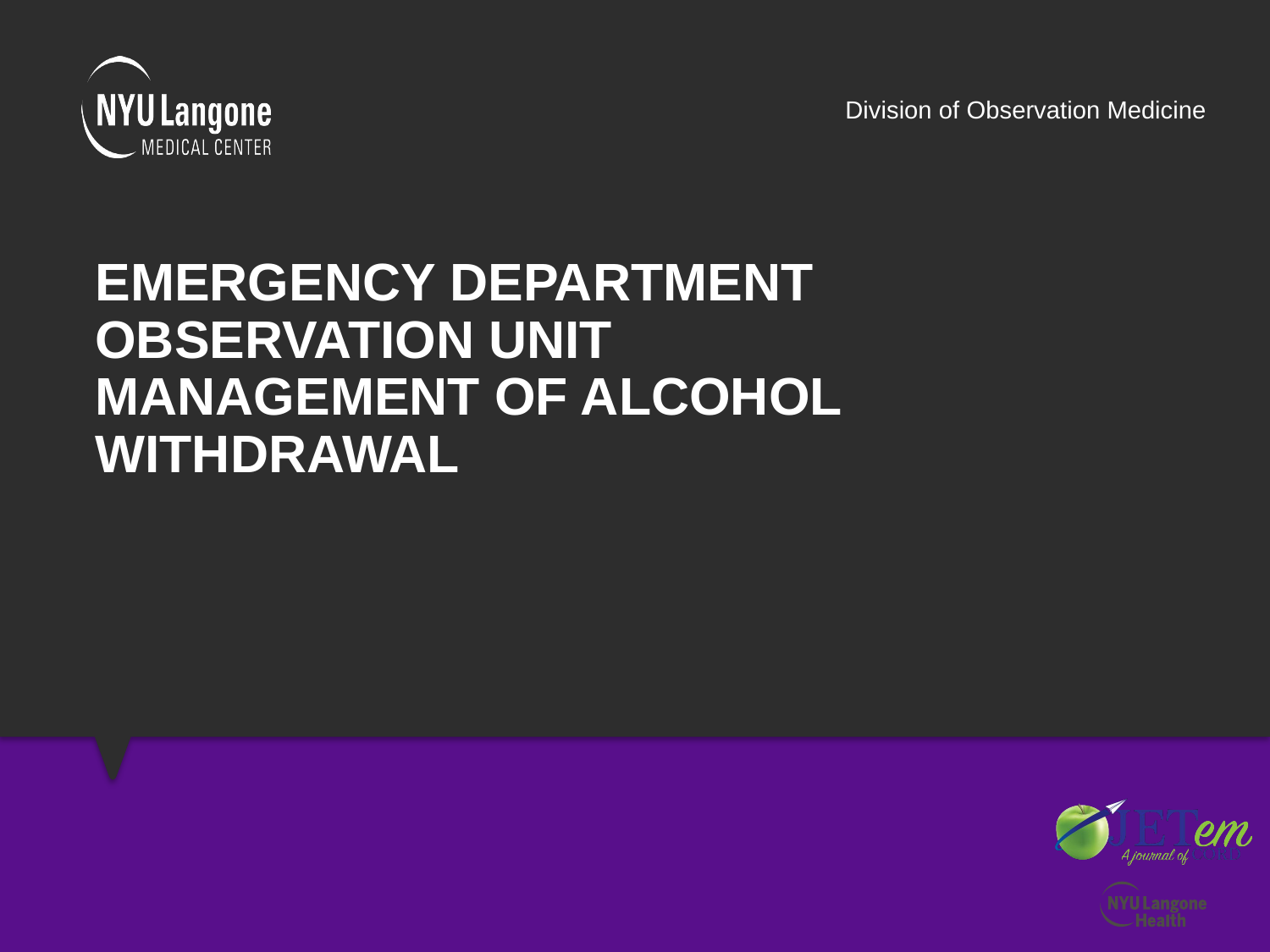

Division of Observation Medicine
# Emergency Department Observation Unit Management of Alcohol Withdrawal

## Slide 2
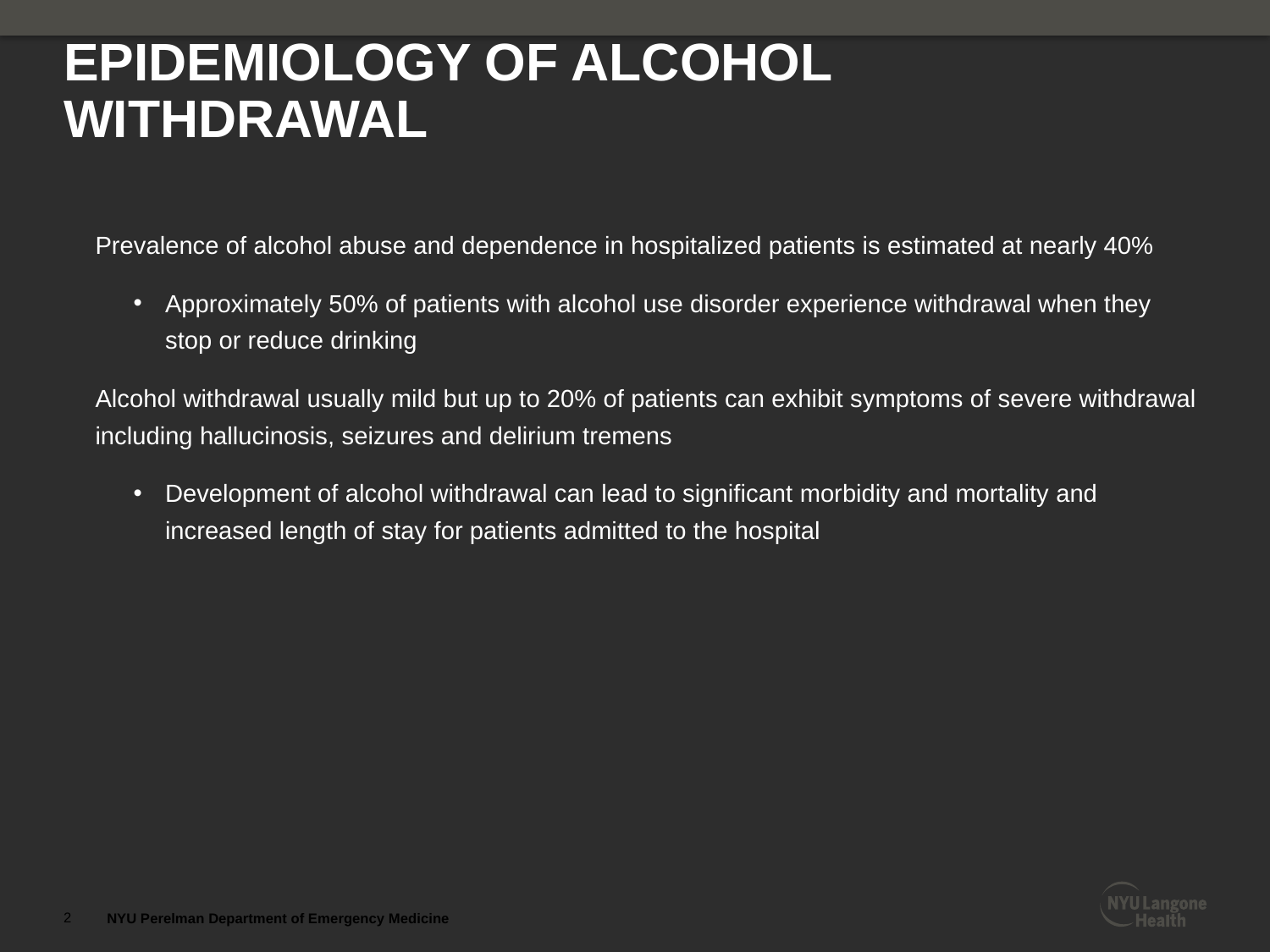

# Epidemiology of Alcohol Withdrawal
Prevalence of alcohol abuse and dependence in hospitalized patients is estimated at nearly 40%
Approximately 50% of patients with alcohol use disorder experience withdrawal when they stop or reduce drinking
Alcohol withdrawal usually mild but up to 20% of patients can exhibit symptoms of severe withdrawal including hallucinosis, seizures and delirium tremens
Development of alcohol withdrawal can lead to significant morbidity and mortality and increased length of stay for patients admitted to the hospital
2
NYU Perelman Department of Emergency Medicine

## Slide 3
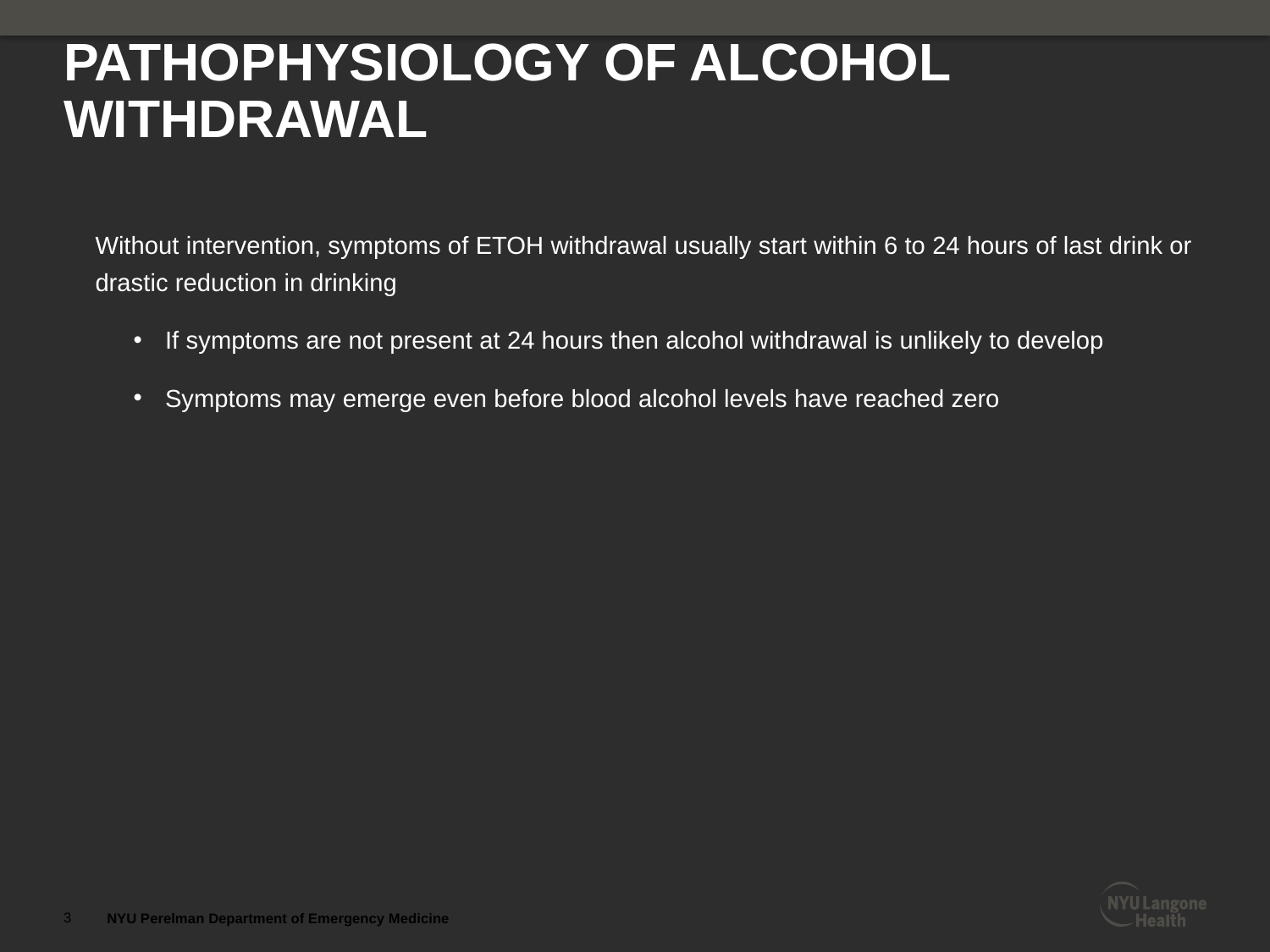

# Pathophysiology of Alcohol Withdrawal
Without intervention, symptoms of ETOH withdrawal usually start within 6 to 24 hours of last drink or drastic reduction in drinking
If symptoms are not present at 24 hours then alcohol withdrawal is unlikely to develop
Symptoms may emerge even before blood alcohol levels have reached zero
3
NYU Perelman Department of Emergency Medicine

## Slide 4
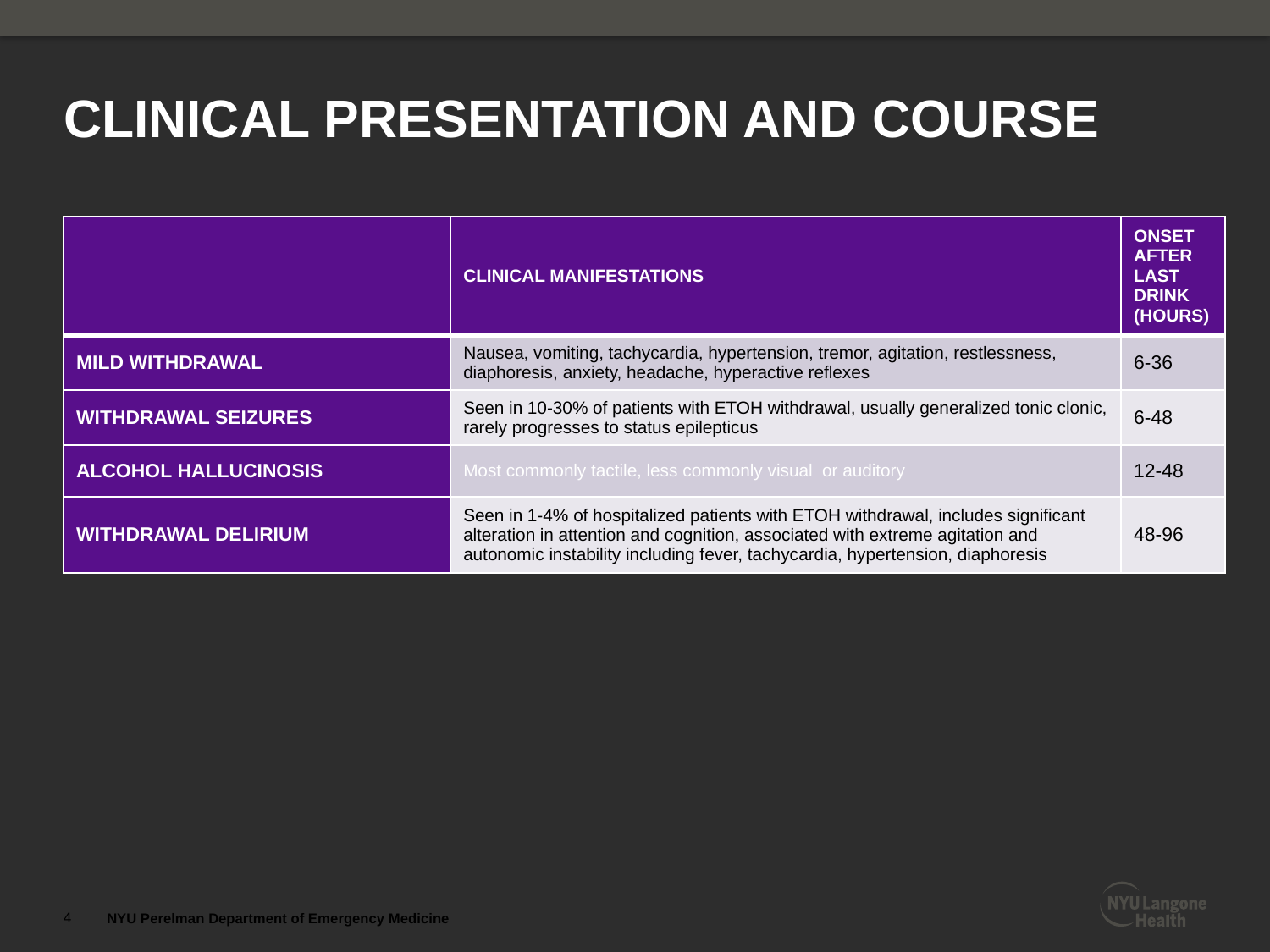

# Clinical Presentation and Course
| | Clinical Manifestations | Onset After Last Drink (Hours) |
| --- | --- | --- |
| Mild Withdrawal | Nausea, vomiting, tachycardia, hypertension, tremor, agitation, restlessness, diaphoresis, anxiety, headache, hyperactive reflexes | 6-36 |
| Withdrawal Seizures | Seen in 10-30% of patients with ETOH withdrawal, usually generalized tonic clonic, rarely progresses to status epilepticus | 6-48 |
| Alcohol Hallucinosis | Most commonly tactile, less commonly visual or auditory | 12-48 |
| Withdrawal Delirium | Seen in 1-4% of hospitalized patients with ETOH withdrawal, includes significant alteration in attention and cognition, associated with extreme agitation and autonomic instability including fever, tachycardia, hypertension, diaphoresis | 48-96 |
4
NYU Perelman Department of Emergency Medicine

## Slide 5
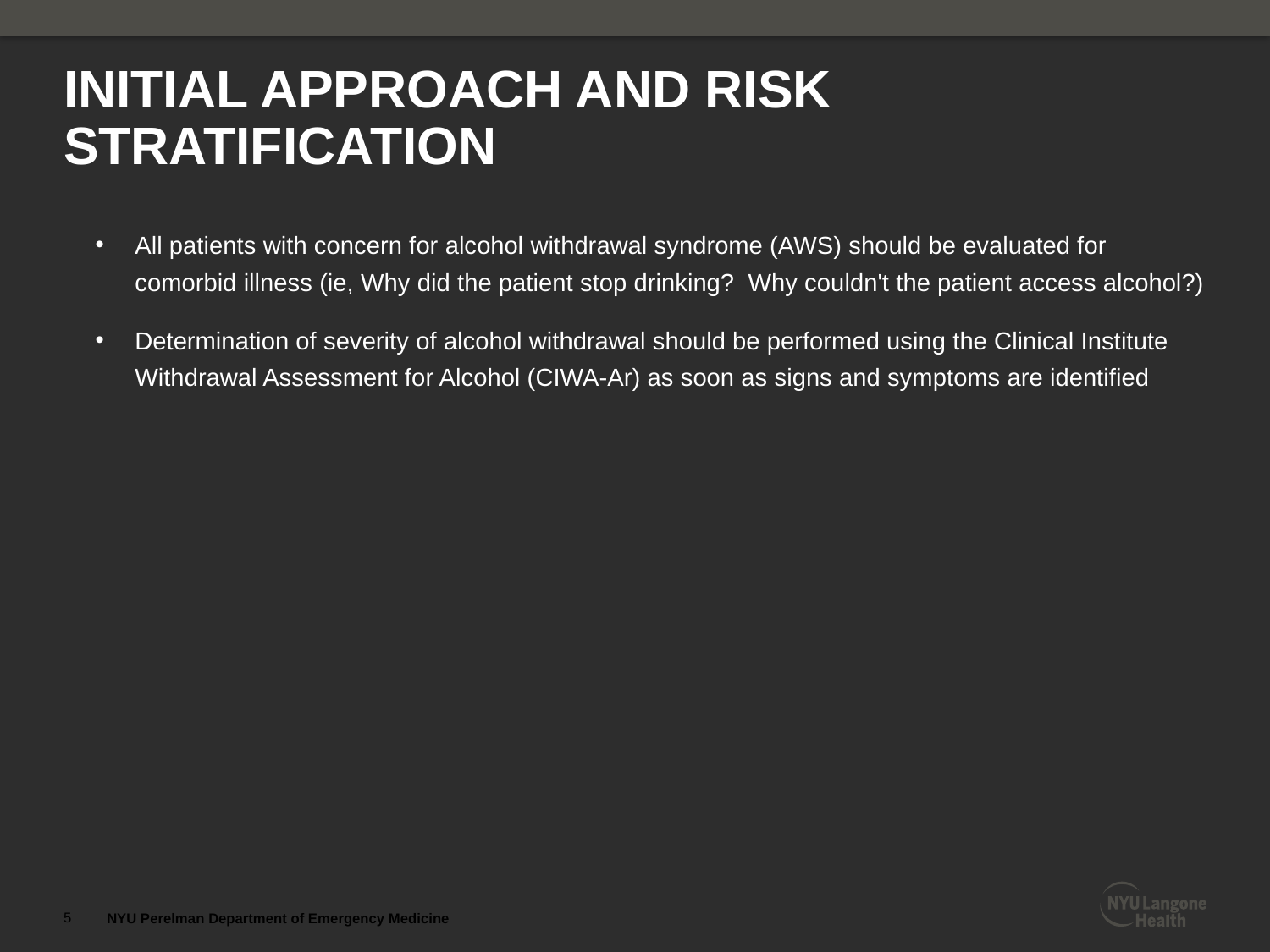

# Initial Approach and Risk Stratification
All patients with concern for alcohol withdrawal syndrome (AWS) should be evaluated for comorbid illness (ie, Why did the patient stop drinking?  Why couldn't the patient access alcohol?)
Determination of severity of alcohol withdrawal should be performed using the Clinical Institute Withdrawal Assessment for Alcohol (CIWA-Ar) as soon as signs and symptoms are identified
5
NYU Perelman Department of Emergency Medicine

## Slide 6
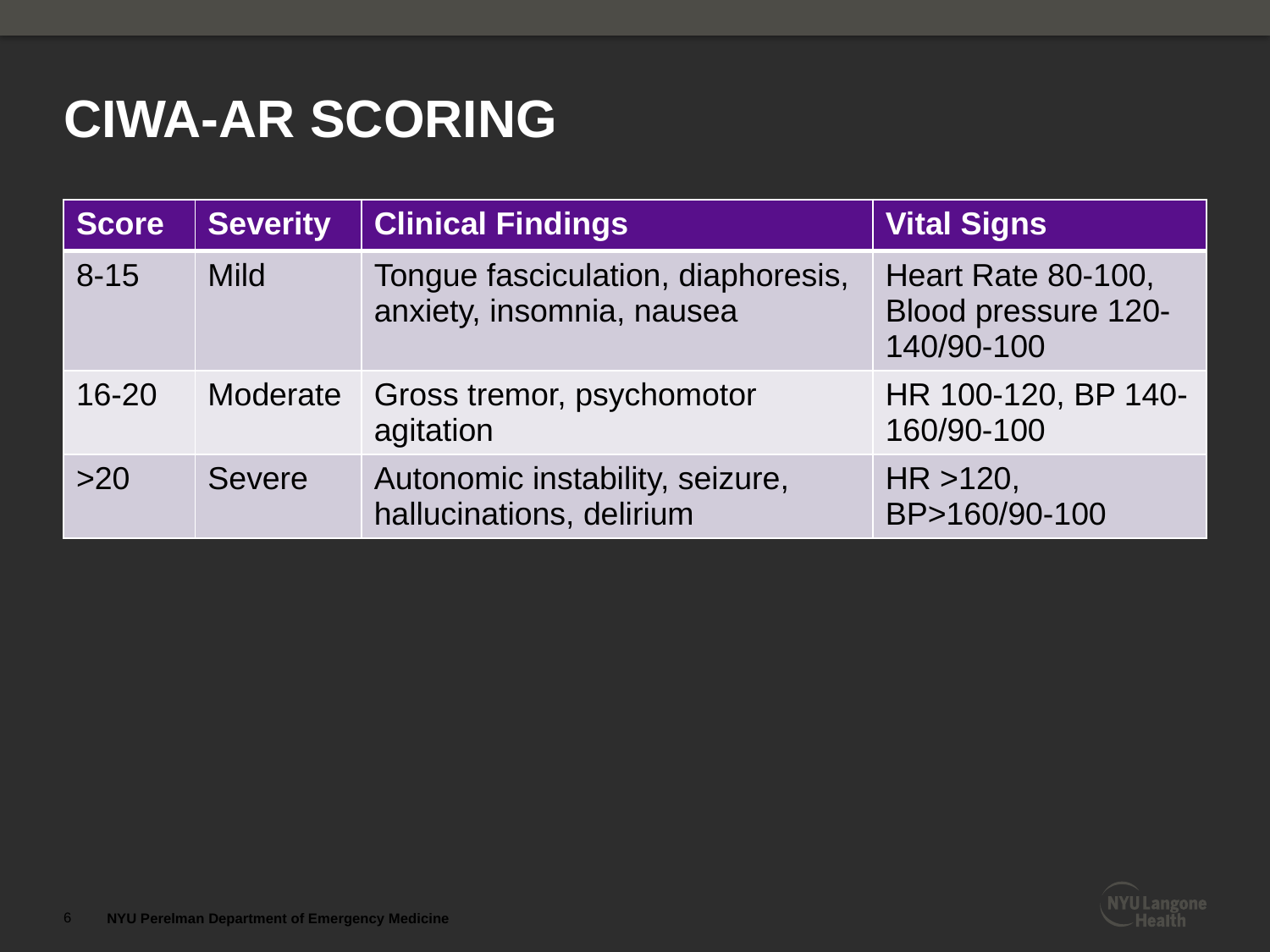

# CIWA-Ar Scoring
| Score | Severity | Clinical Findings | Vital Signs |
| --- | --- | --- | --- |
| 8-15 | Mild | Tongue fasciculation, diaphoresis, anxiety, insomnia, nausea | Heart Rate 80-100, Blood pressure 120-140/90-100 |
| 16-20 | Moderate | Gross tremor, psychomotor agitation | HR 100-120, BP 140-160/90-100 |
| >20 | Severe | Autonomic instability, seizure, hallucinations, delirium | HR >120, BP>160/90-100 |
6
NYU Perelman Department of Emergency Medicine

## Slide 7
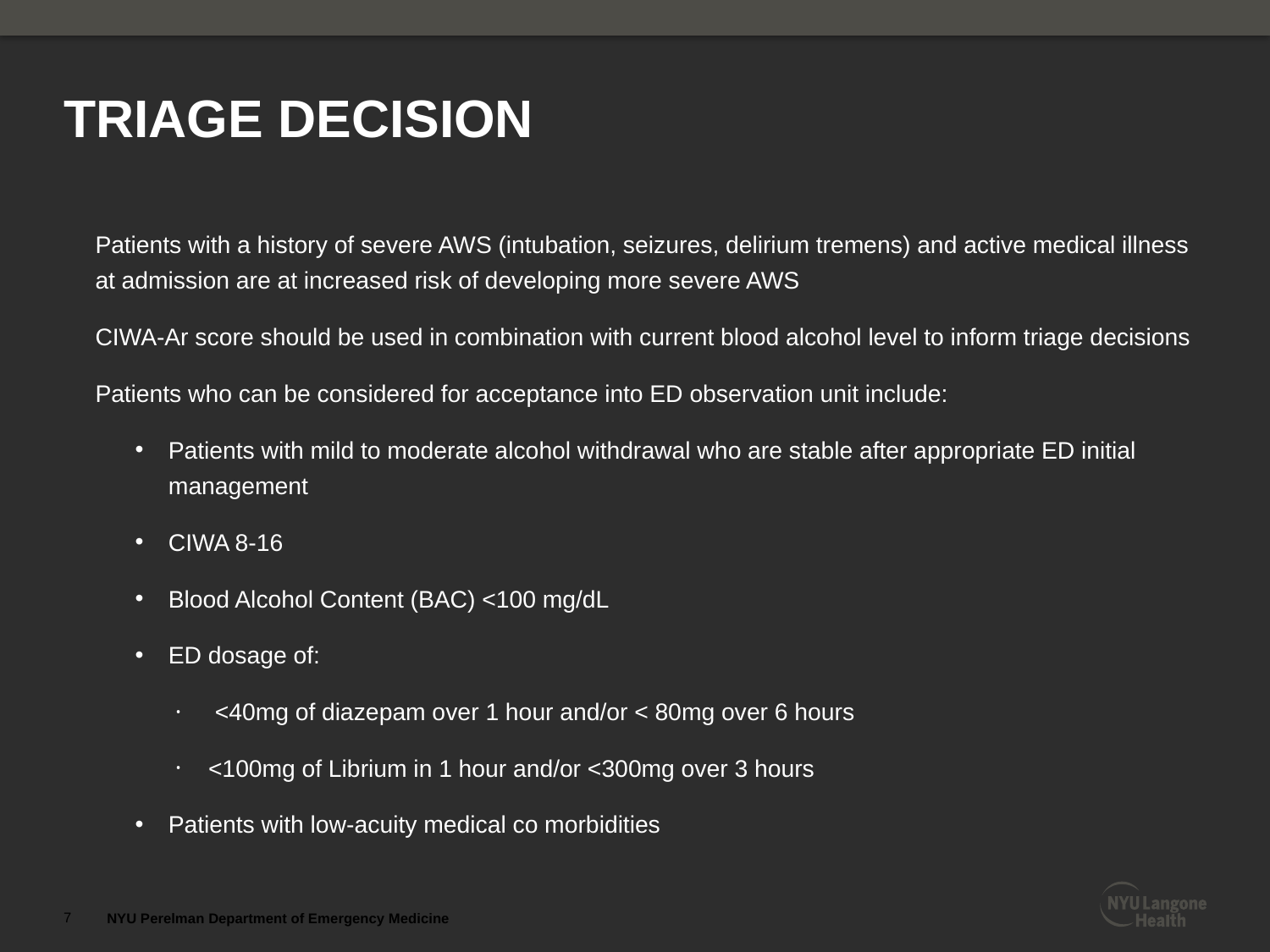

# Triage Decision
Patients with a history of severe AWS (intubation, seizures, delirium tremens) and active medical illness at admission are at increased risk of developing more severe AWS
CIWA-Ar score should be used in combination with current blood alcohol level to inform triage decisions
Patients who can be considered for acceptance into ED observation unit include:
Patients with mild to moderate alcohol withdrawal who are stable after appropriate ED initial management
CIWA 8-16
Blood Alcohol Content (BAC) <100 mg/dL
ED dosage of:
 <40mg of diazepam over 1 hour and/or < 80mg over 6 hours
<100mg of Librium in 1 hour and/or <300mg over 3 hours
Patients with low-acuity medical co morbidities
7
NYU Perelman Department of Emergency Medicine

## Slide 8
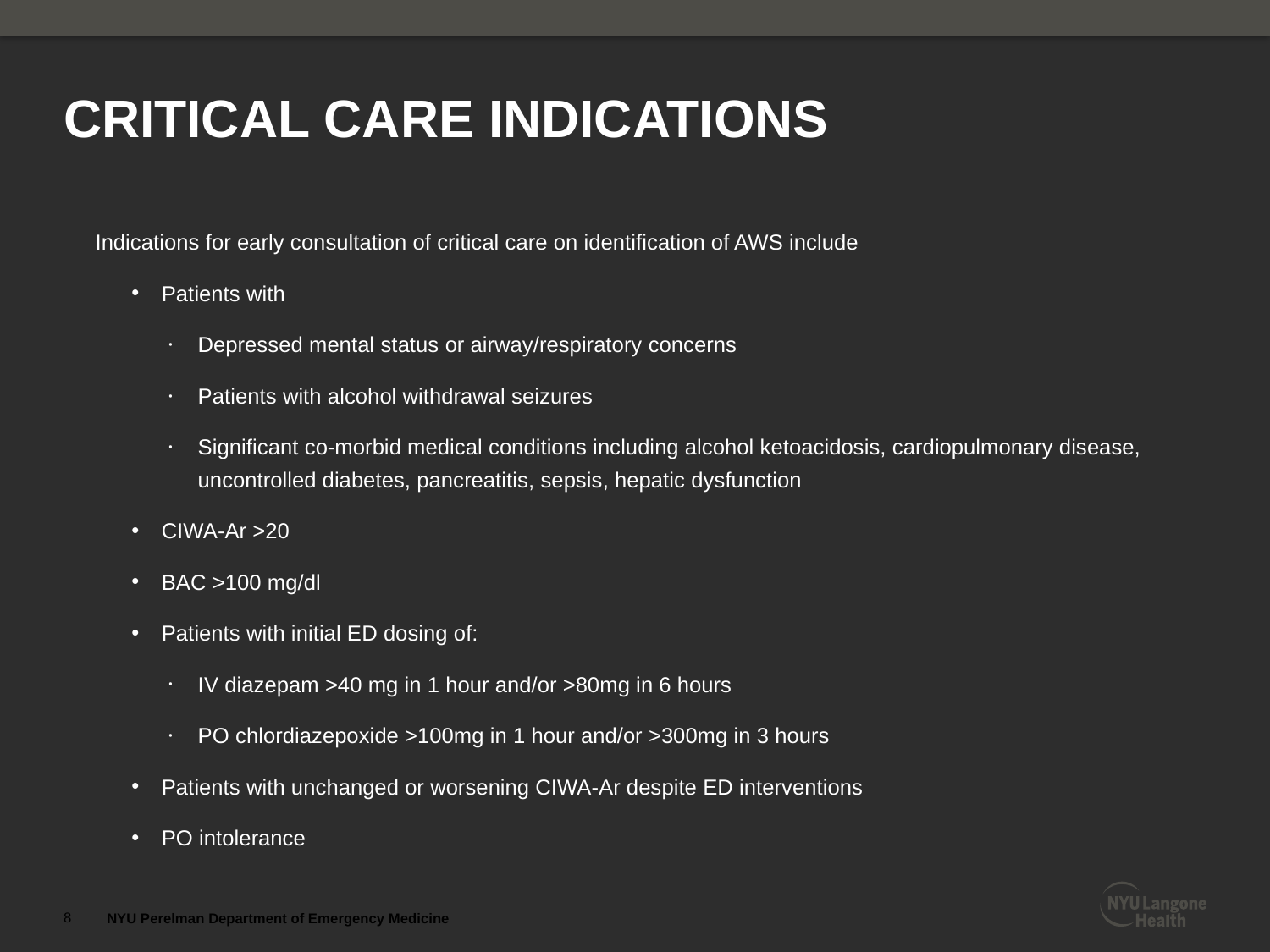

# Critical Care Indications
Indications for early consultation of critical care on identification of AWS include
Patients with
Depressed mental status or airway/respiratory concerns
Patients with alcohol withdrawal seizures
Significant co-morbid medical conditions including alcohol ketoacidosis, cardiopulmonary disease, uncontrolled diabetes, pancreatitis, sepsis, hepatic dysfunction
CIWA-Ar >20
BAC >100 mg/dl
Patients with initial ED dosing of:
IV diazepam >40 mg in 1 hour and/or >80mg in 6 hours
PO chlordiazepoxide >100mg in 1 hour and/or >300mg in 3 hours
Patients with unchanged or worsening CIWA-Ar despite ED interventions
PO intolerance
8
NYU Perelman Department of Emergency Medicine

## Slide 9
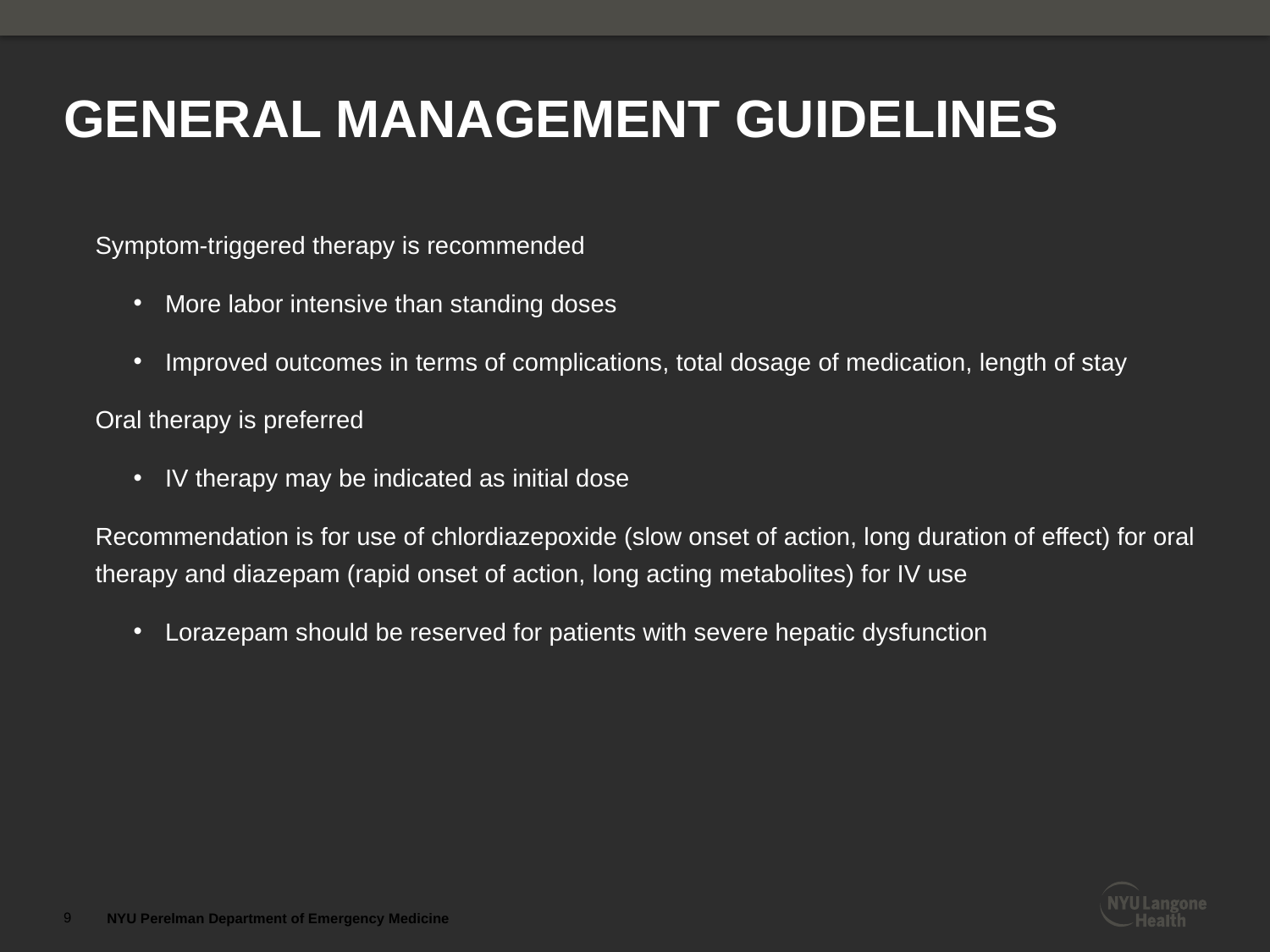

# General Management Guidelines
Symptom-triggered therapy is recommended
More labor intensive than standing doses
Improved outcomes in terms of complications, total dosage of medication, length of stay
Oral therapy is preferred
IV therapy may be indicated as initial dose
Recommendation is for use of chlordiazepoxide (slow onset of action, long duration of effect) for oral therapy and diazepam (rapid onset of action, long acting metabolites) for IV use
Lorazepam should be reserved for patients with severe hepatic dysfunction
9
NYU Perelman Department of Emergency Medicine

## Slide 10
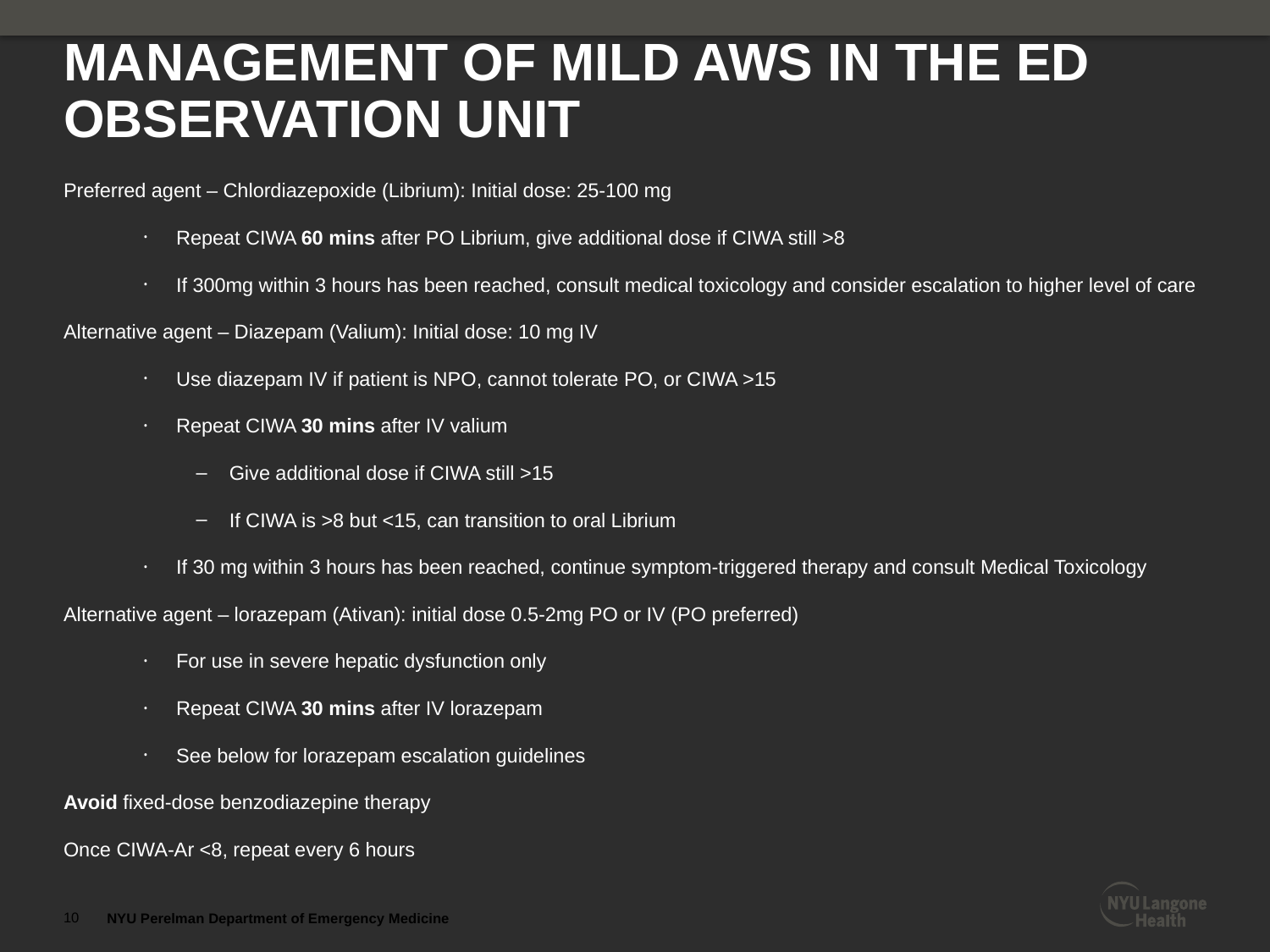

# Management of Mild AWS in the ED Observation Unit
Preferred agent – Chlordiazepoxide (Librium): Initial dose: 25-100 mg
Repeat CIWA 60 mins after PO Librium, give additional dose if CIWA still >8
If 300mg within 3 hours has been reached, consult medical toxicology and consider escalation to higher level of care
Alternative agent – Diazepam (Valium): Initial dose: 10 mg IV
Use diazepam IV if patient is NPO, cannot tolerate PO, or CIWA >15
Repeat CIWA 30 mins after IV valium
Give additional dose if CIWA still >15
If CIWA is >8 but <15, can transition to oral Librium
If 30 mg within 3 hours has been reached, continue symptom-triggered therapy and consult Medical Toxicology
Alternative agent – lorazepam (Ativan): initial dose 0.5-2mg PO or IV (PO preferred)
For use in severe hepatic dysfunction only
Repeat CIWA 30 mins after IV lorazepam
See below for lorazepam escalation guidelines
Avoid fixed-dose benzodiazepine therapy
Once CIWA-Ar <8, repeat every 6 hours
10
NYU Perelman Department of Emergency Medicine

## Slide 11
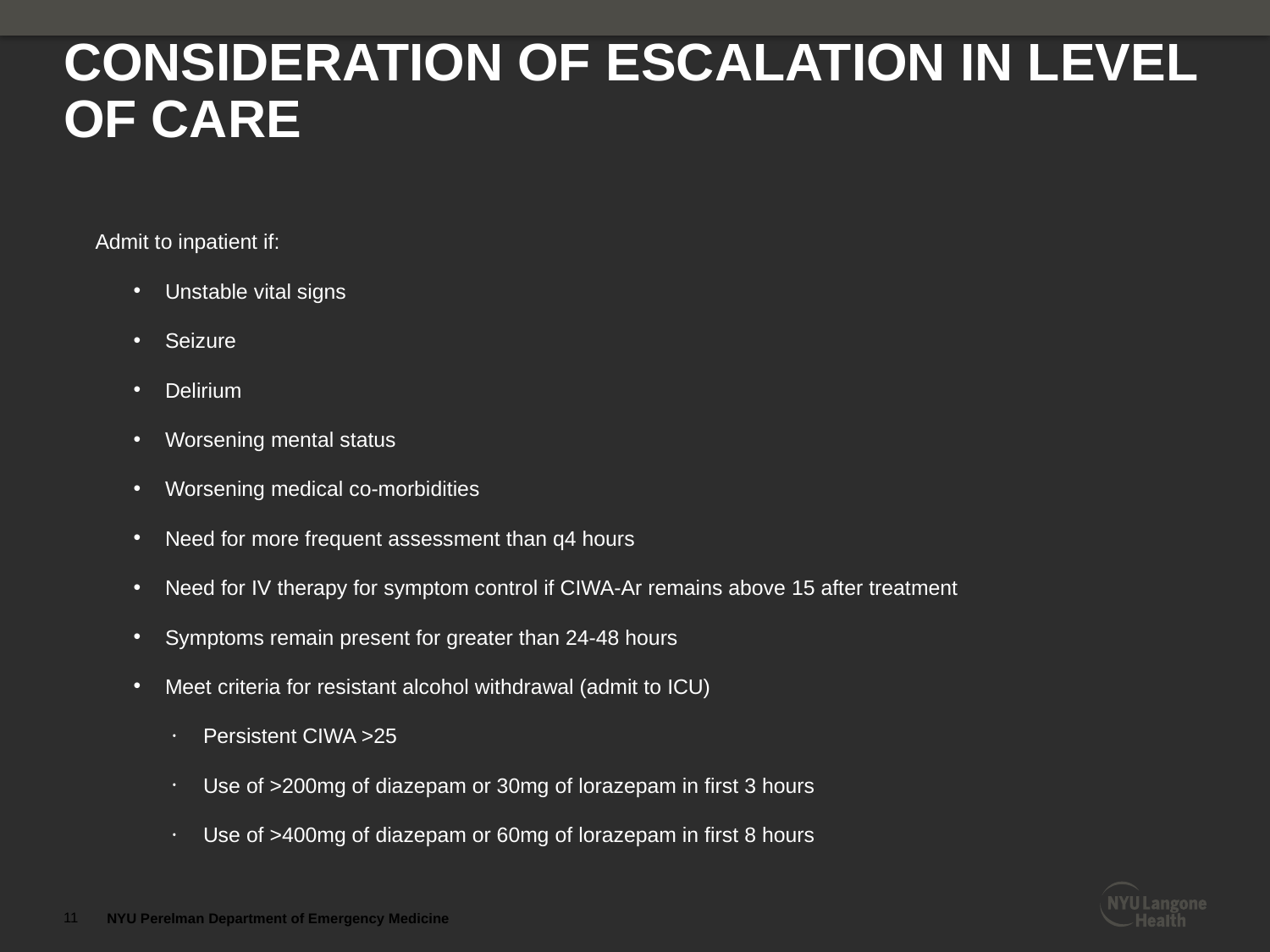

# Consideration of Escalation in Level of Care
Admit to inpatient if:
Unstable vital signs
Seizure
Delirium
Worsening mental status
Worsening medical co-morbidities
Need for more frequent assessment than q4 hours
Need for IV therapy for symptom control if CIWA-Ar remains above 15 after treatment
Symptoms remain present for greater than 24-48 hours
Meet criteria for resistant alcohol withdrawal (admit to ICU)
Persistent CIWA >25
Use of >200mg of diazepam or 30mg of lorazepam in first 3 hours
Use of >400mg of diazepam or 60mg of lorazepam in first 8 hours
11
NYU Perelman Department of Emergency Medicine

## Slide 12
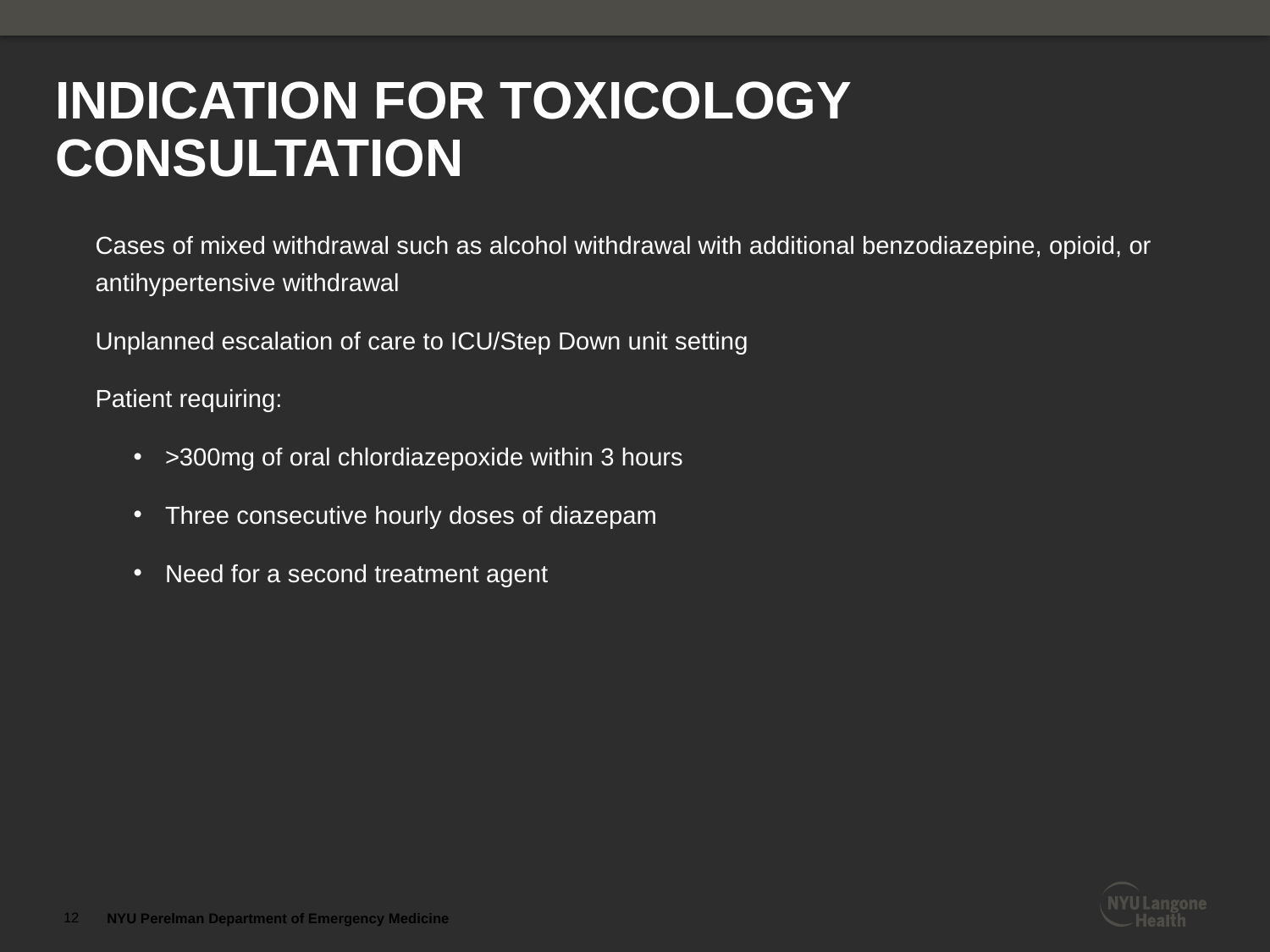

# Indication for Toxicology Consultation
Cases of mixed withdrawal such as alcohol withdrawal with additional benzodiazepine, opioid, or antihypertensive withdrawal
Unplanned escalation of care to ICU/Step Down unit setting
Patient requiring:
>300mg of oral chlordiazepoxide within 3 hours
Three consecutive hourly doses of diazepam
Need for a second treatment agent
12
NYU Perelman Department of Emergency Medicine

## Slide 13
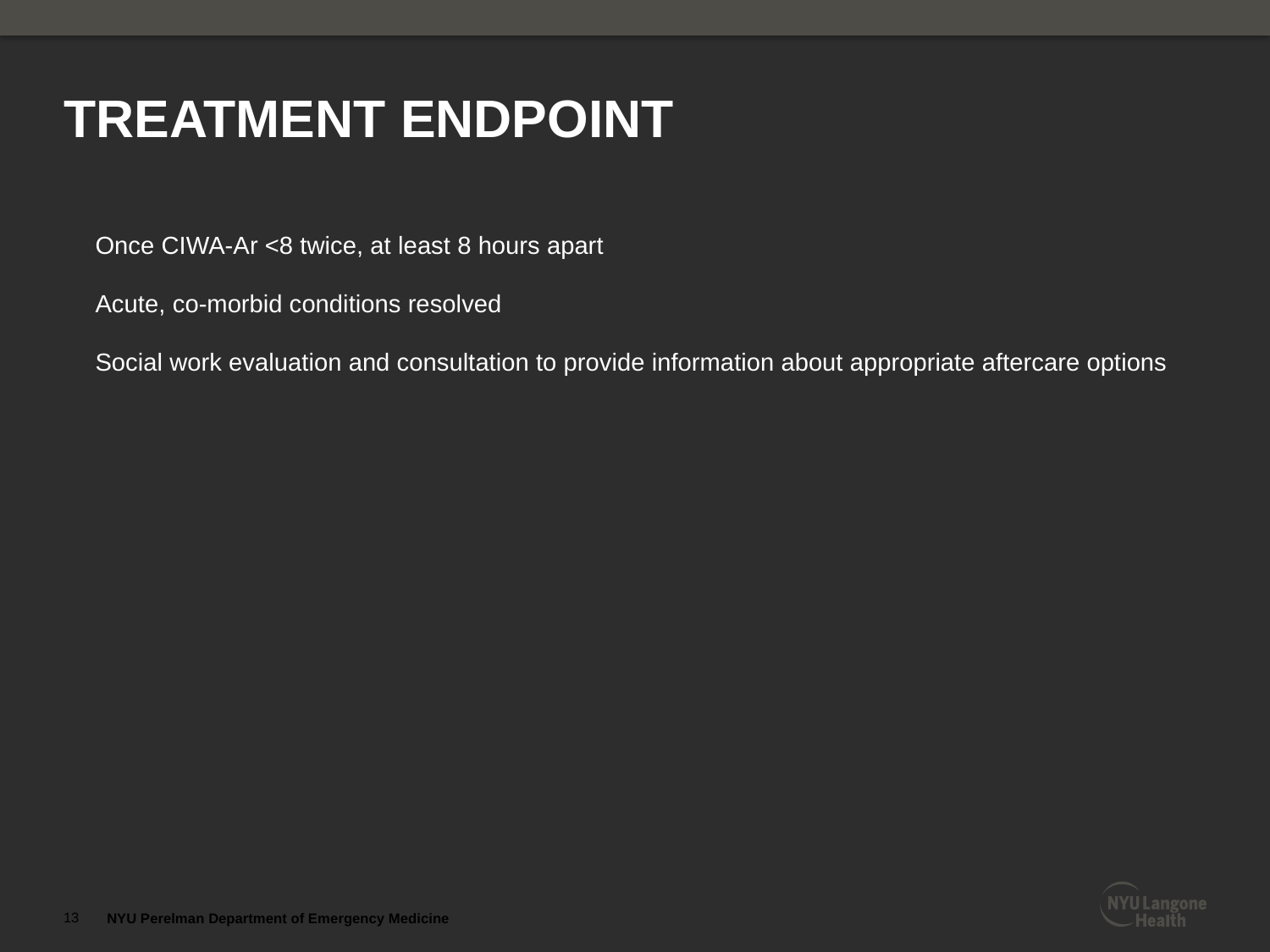

# Treatment Endpoint
Once CIWA-Ar <8 twice, at least 8 hours apart
Acute, co-morbid conditions resolved
Social work evaluation and consultation to provide information about appropriate aftercare options
13
NYU Perelman Department of Emergency Medicine

## Slide 14
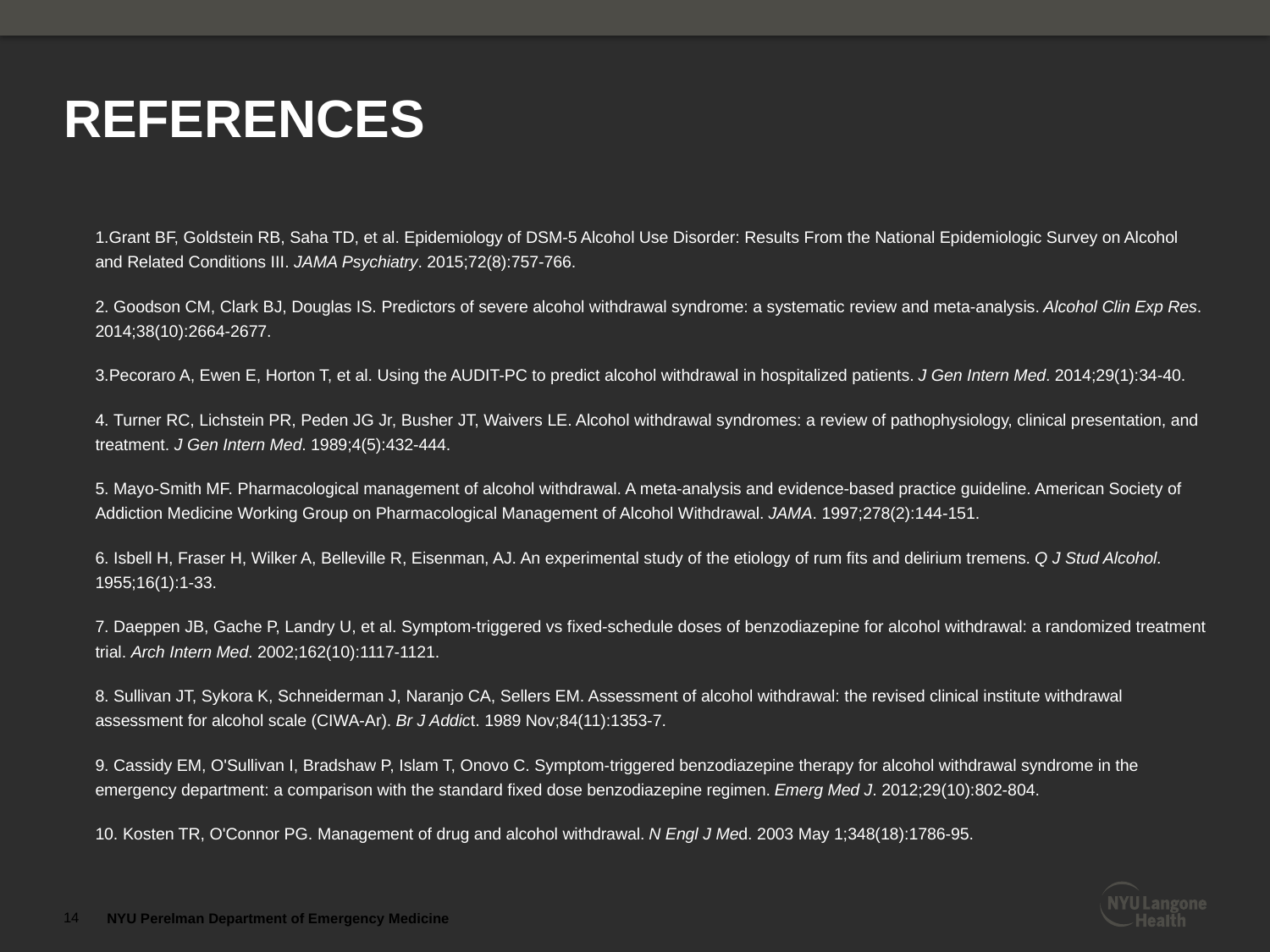

# References
1.Grant BF, Goldstein RB, Saha TD, et al. Epidemiology of DSM-5 Alcohol Use Disorder: Results From the National Epidemiologic Survey on Alcohol and Related Conditions III. JAMA Psychiatry. 2015;72(8):757-766.
2. Goodson CM, Clark BJ, Douglas IS. Predictors of severe alcohol withdrawal syndrome: a systematic review and meta-analysis. Alcohol Clin Exp Res. 2014;38(10):2664-2677.
3.Pecoraro A, Ewen E, Horton T, et al. Using the AUDIT-PC to predict alcohol withdrawal in hospitalized patients. J Gen Intern Med. 2014;29(1):34-40.
4. Turner RC, Lichstein PR, Peden JG Jr, Busher JT, Waivers LE. Alcohol withdrawal syndromes: a review of pathophysiology, clinical presentation, and treatment. J Gen Intern Med. 1989;4(5):432-444.
5. Mayo-Smith MF. Pharmacological management of alcohol withdrawal. A meta-analysis and evidence-based practice guideline. American Society of Addiction Medicine Working Group on Pharmacological Management of Alcohol Withdrawal. JAMA. 1997;278(2):144-151.
6. Isbell H, Fraser H, Wilker A, Belleville R, Eisenman, AJ. An experimental study of the etiology of rum fits and delirium tremens. Q J Stud Alcohol. 1955;16(1):1-33.
7. Daeppen JB, Gache P, Landry U, et al. Symptom-triggered vs fixed-schedule doses of benzodiazepine for alcohol withdrawal: a randomized treatment trial. Arch Intern Med. 2002;162(10):1117-1121.
8. Sullivan JT, Sykora K, Schneiderman J, Naranjo CA, Sellers EM. Assessment of alcohol withdrawal: the revised clinical institute withdrawal assessment for alcohol scale (CIWA-Ar). Br J Addict. 1989 Nov;84(11):1353-7.
9. Cassidy EM, O'Sullivan I, Bradshaw P, Islam T, Onovo C. Symptom-triggered benzodiazepine therapy for alcohol withdrawal syndrome in the emergency department: a comparison with the standard fixed dose benzodiazepine regimen. Emerg Med J. 2012;29(10):802-804.
10. Kosten TR, O'Connor PG. Management of drug and alcohol withdrawal. N Engl J Med. 2003 May 1;348(18):1786-95.
14
NYU Perelman Department of Emergency Medicine
