# Supplementary material for: An Observation Medicine Curriculum for Emergency Medicine Education
Source: J Educ Teach Emerg Med. 2021 Apr 19;6(2):C1–C72. doi: 10.21980/J87P92 (PMC10332786; doi:10.21980/J87P92)
Supplement: Supplementary file 26 — Please see associated PowerPoint file [file jetem-6-2-c1-supp26.pptx]

## Slide 1
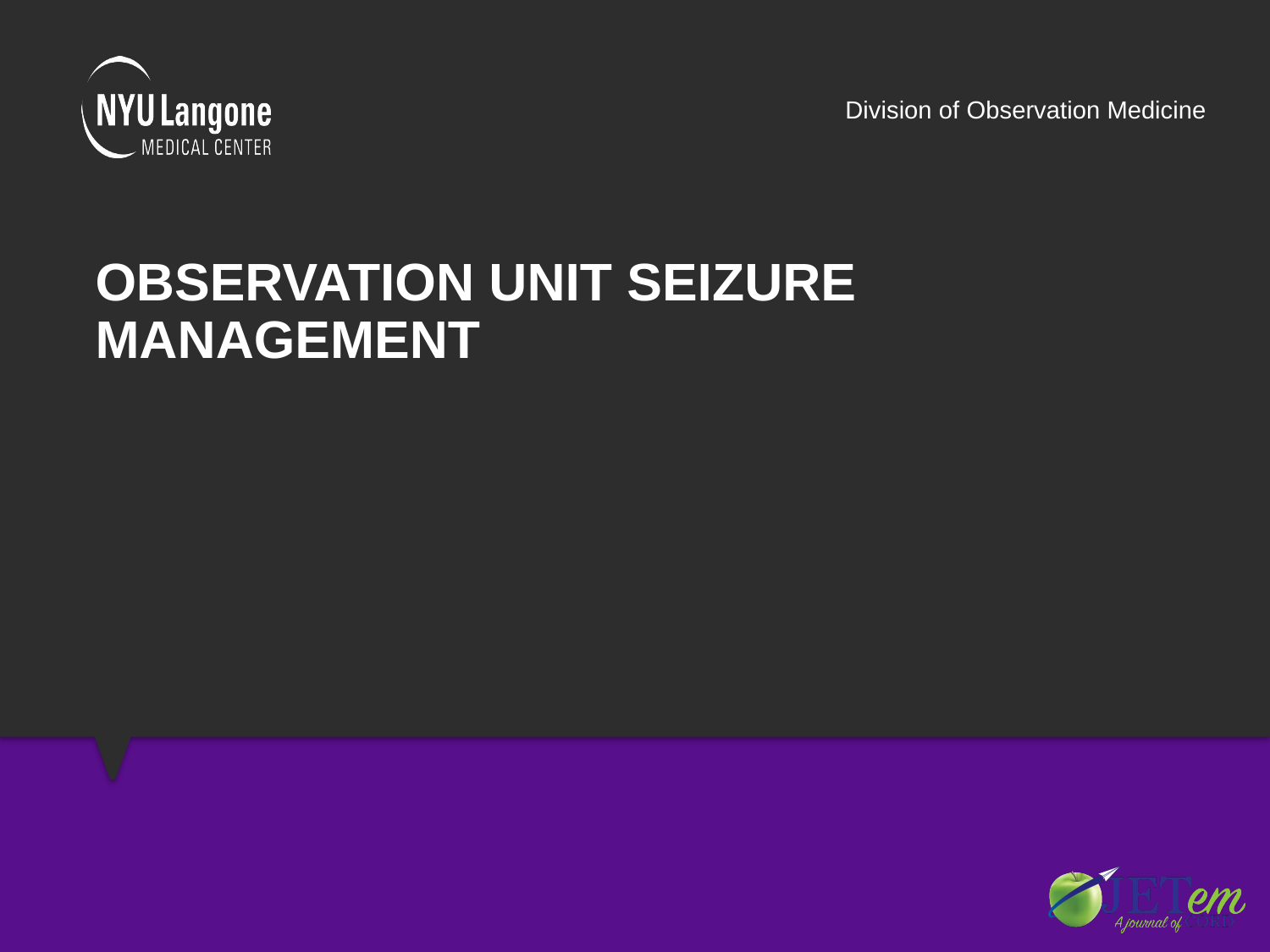

Division of Observation Medicine
# Observation unit Seizure management

## Slide 2
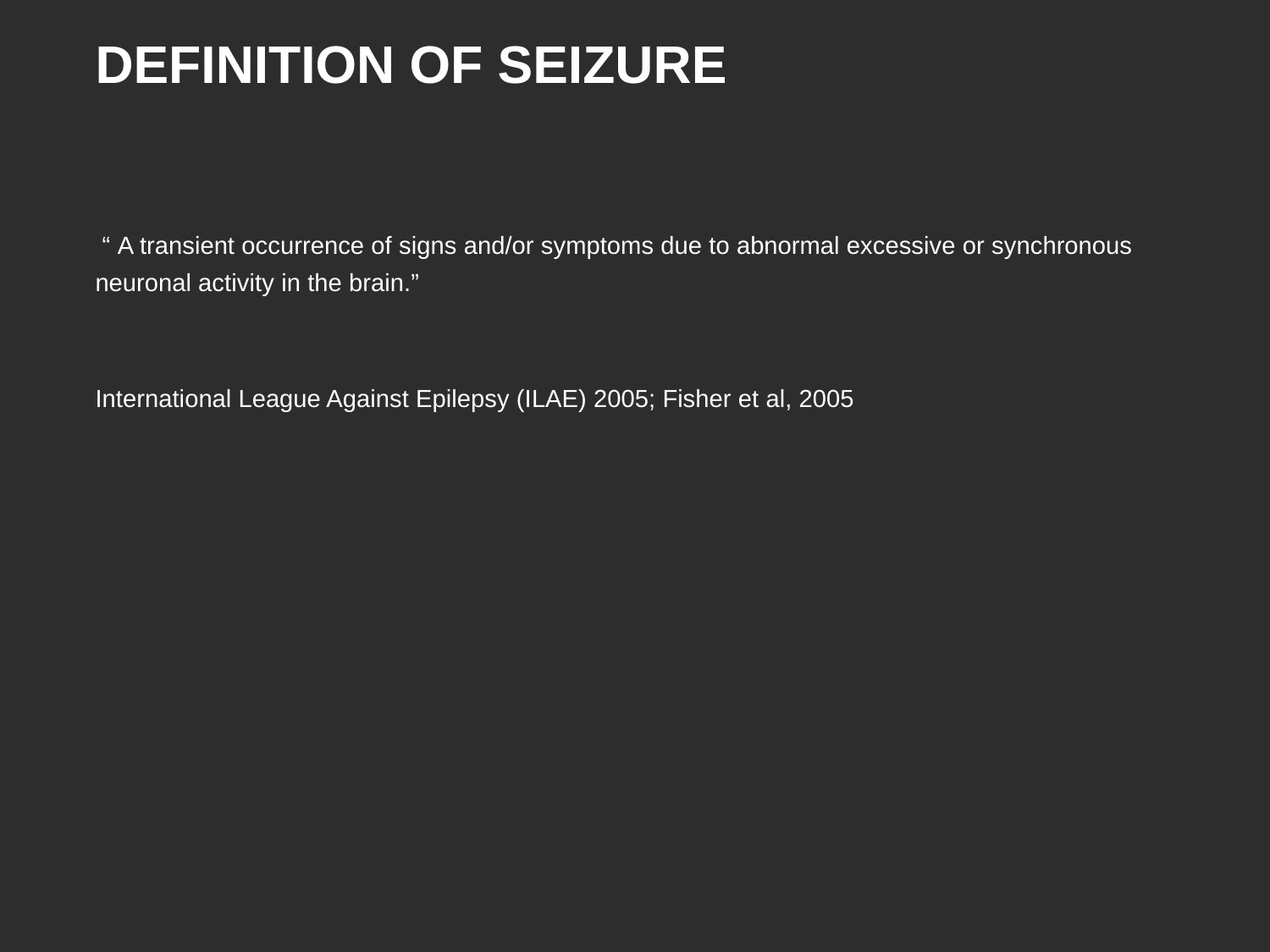

# Definition of Seizure
 “ A transient occurrence of signs and/or symptoms due to abnormal excessive or synchronous neuronal activity in the brain.”
International League Against Epilepsy (ILAE) 2005; Fisher et al, 2005

## Slide 3
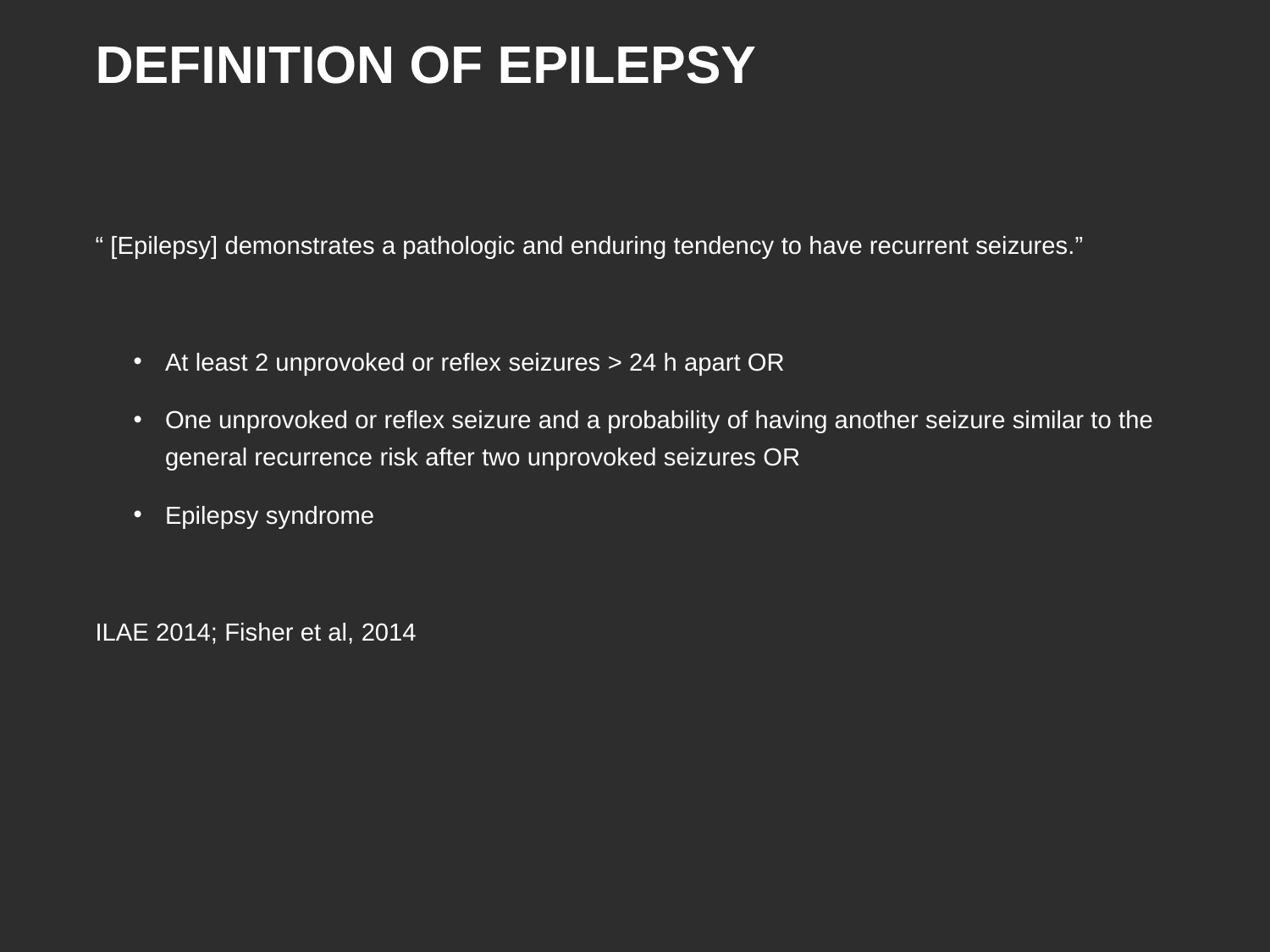

# Definition of Epilepsy
“ [Epilepsy] demonstrates a pathologic and enduring tendency to have recurrent seizures.”
At least 2 unprovoked or reflex seizures > 24 h apart OR
One unprovoked or reflex seizure and a probability of having another seizure similar to the general recurrence risk after two unprovoked seizures OR
Epilepsy syndrome
ILAE 2014; Fisher et al, 2014

## Slide 4
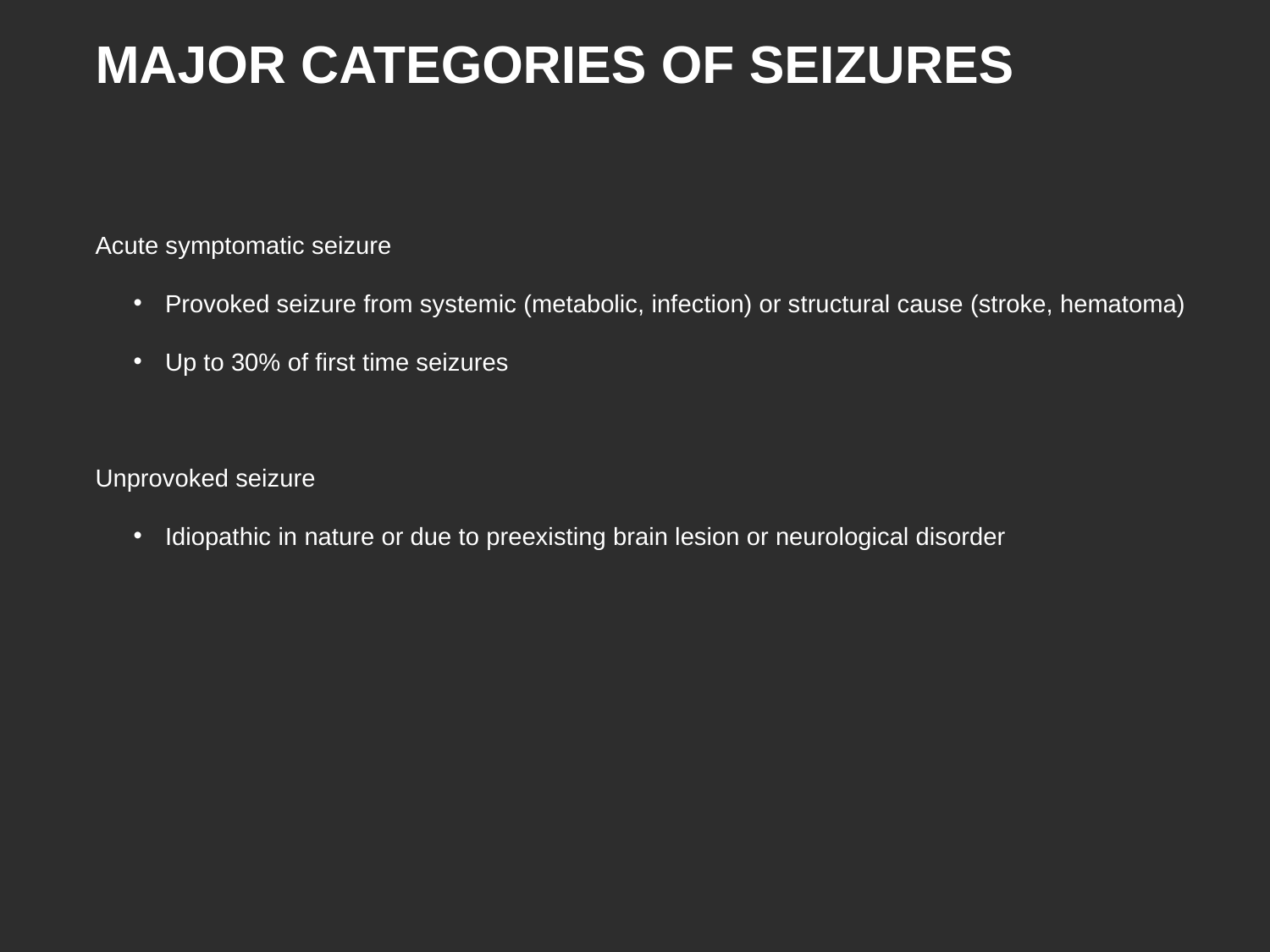

# Major Categories of Seizures
Acute symptomatic seizure
Provoked seizure from systemic (metabolic, infection) or structural cause (stroke, hematoma)
Up to 30% of first time seizures
Unprovoked seizure
Idiopathic in nature or due to preexisting brain lesion or neurological disorder

## Slide 5
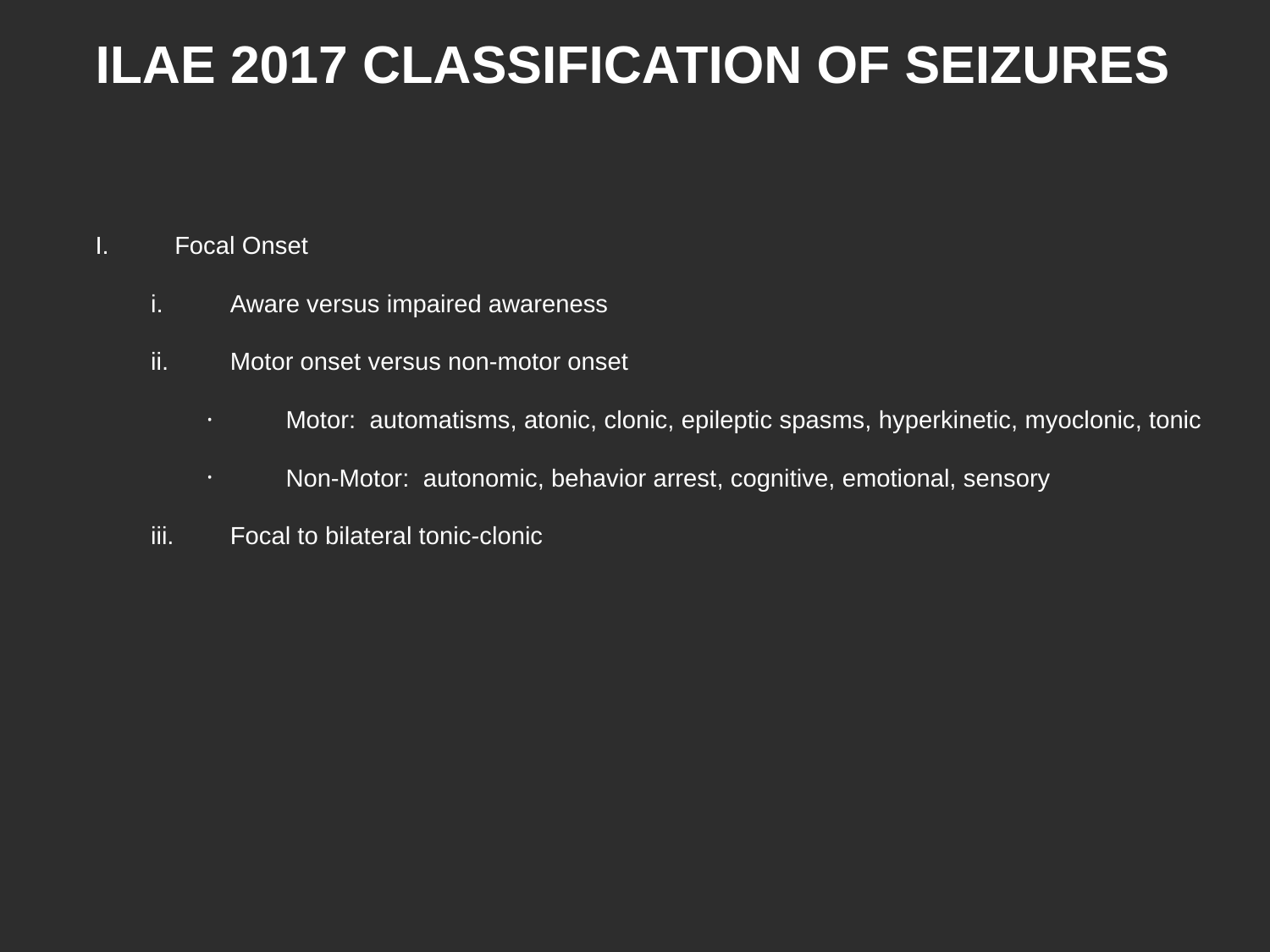

# ILAE 2017 Classification of Seizures
Focal Onset
Aware versus impaired awareness
Motor onset versus non-motor onset
Motor: automatisms, atonic, clonic, epileptic spasms, hyperkinetic, myoclonic, tonic
Non-Motor: autonomic, behavior arrest, cognitive, emotional, sensory
Focal to bilateral tonic-clonic

## Slide 6
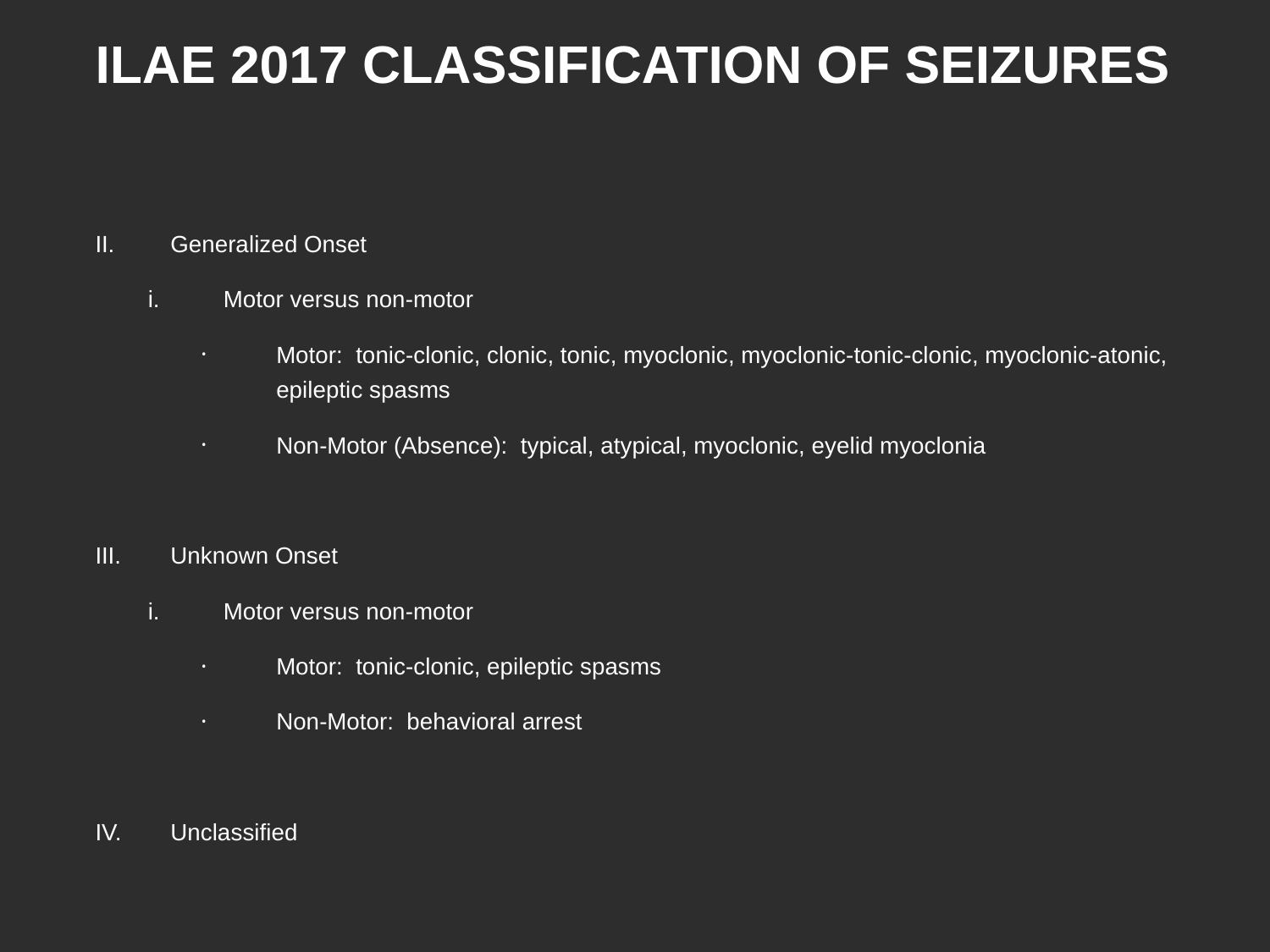

# ILAE 2017 Classification of Seizures
Generalized Onset
Motor versus non-motor
Motor: tonic-clonic, clonic, tonic, myoclonic, myoclonic-tonic-clonic, myoclonic-atonic, epileptic spasms
Non-Motor (Absence): typical, atypical, myoclonic, eyelid myoclonia
Unknown Onset
Motor versus non-motor
Motor: tonic-clonic, epileptic spasms
Non-Motor: behavioral arrest
Unclassified

## Slide 7
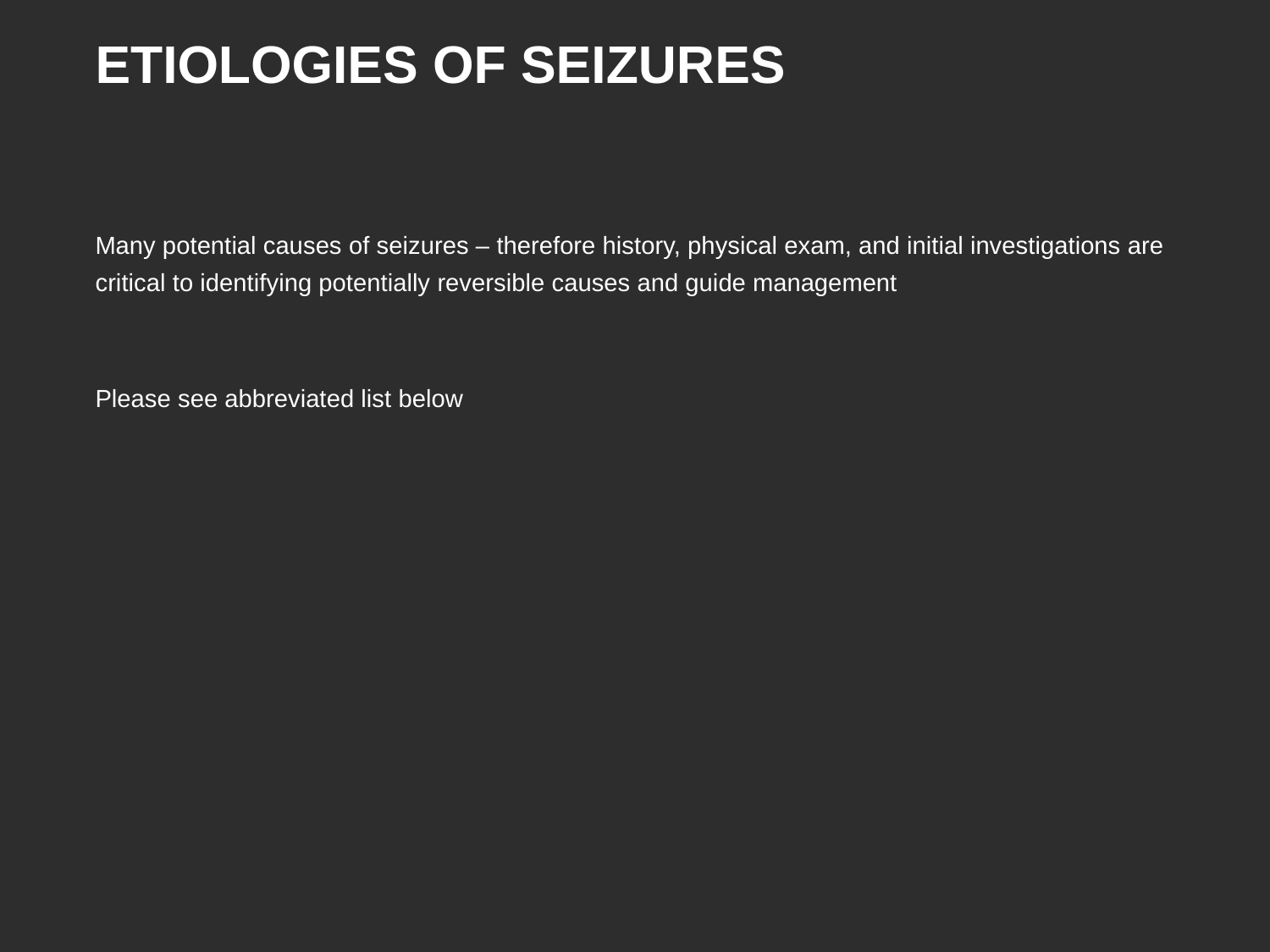

# Etiologies of Seizures
Many potential causes of seizures – therefore history, physical exam, and initial investigations are critical to identifying potentially reversible causes and guide management
Please see abbreviated list below

## Slide 8
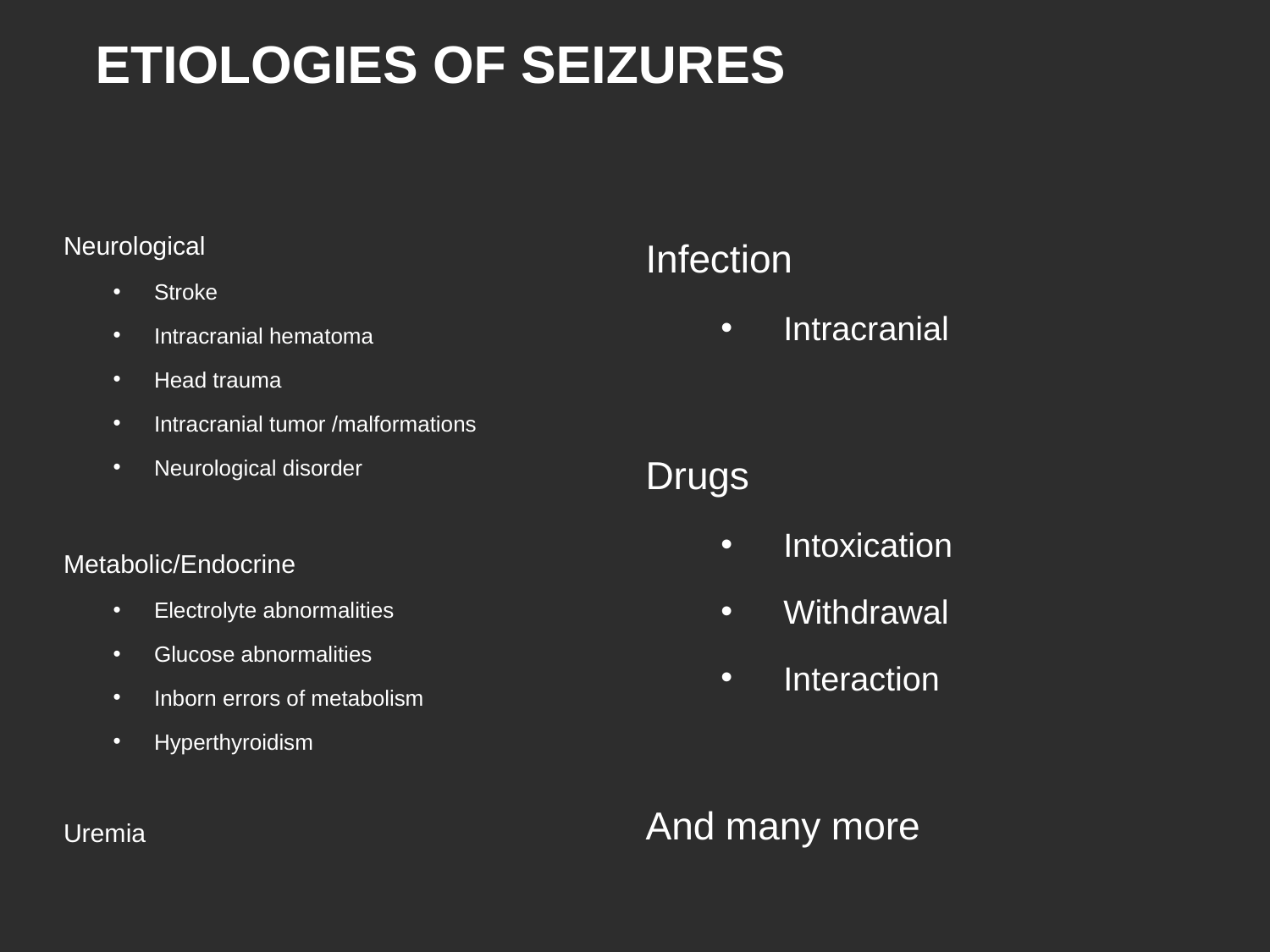

# Etiologies of Seizures
Neurological
Stroke
Intracranial hematoma
Head trauma
Intracranial tumor /malformations
Neurological disorder
Metabolic/Endocrine
Electrolyte abnormalities
Glucose abnormalities
Inborn errors of metabolism
Hyperthyroidism
Uremia
Infection
Intracranial
Drugs
Intoxication
Withdrawal
Interaction
And many more

## Slide 9
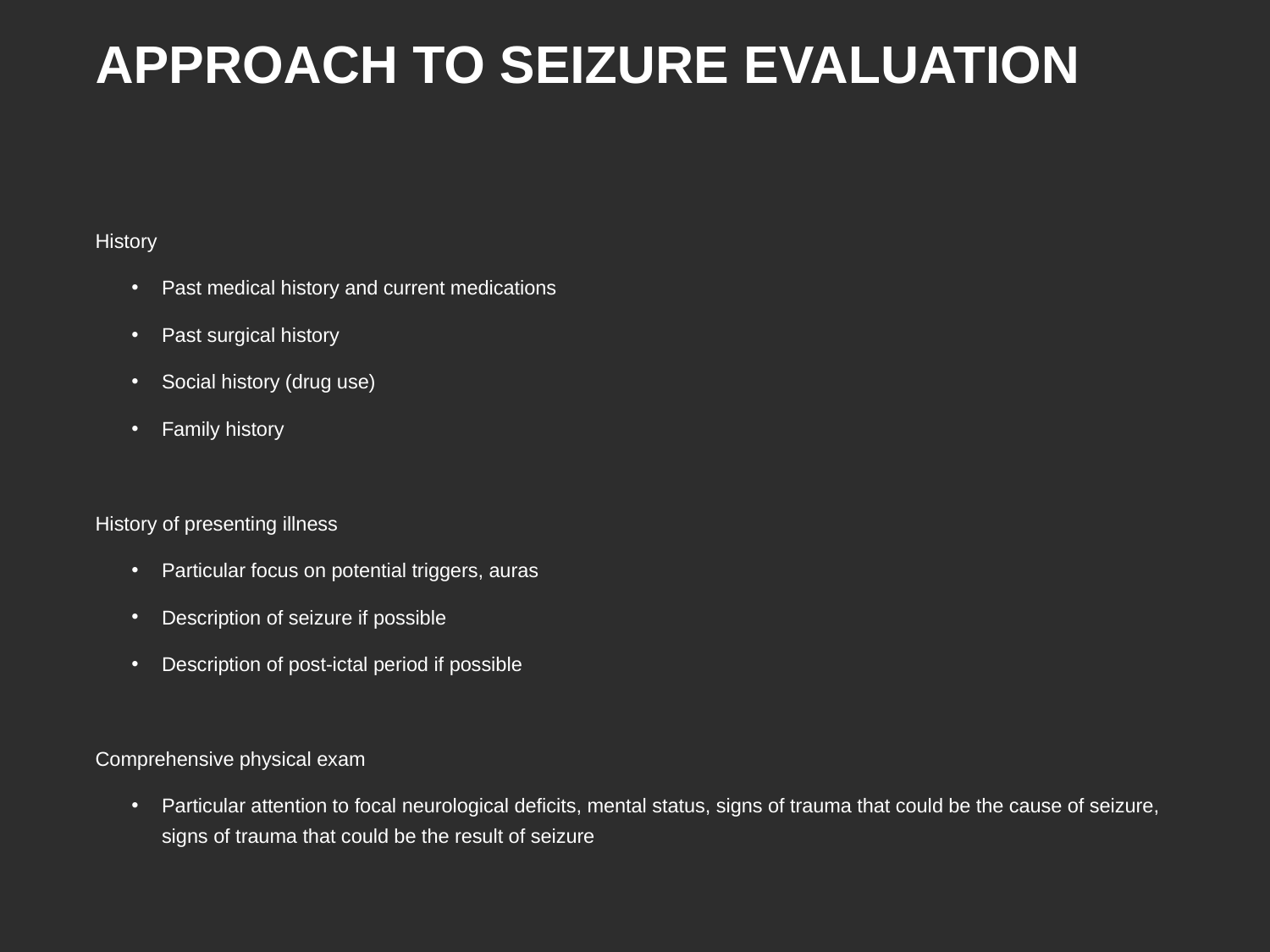

# Approach to Seizure Evaluation
History
Past medical history and current medications
Past surgical history
Social history (drug use)
Family history
History of presenting illness
Particular focus on potential triggers, auras
Description of seizure if possible
Description of post-ictal period if possible
Comprehensive physical exam
Particular attention to focal neurological deficits, mental status, signs of trauma that could be the cause of seizure, signs of trauma that could be the result of seizure

## Slide 10
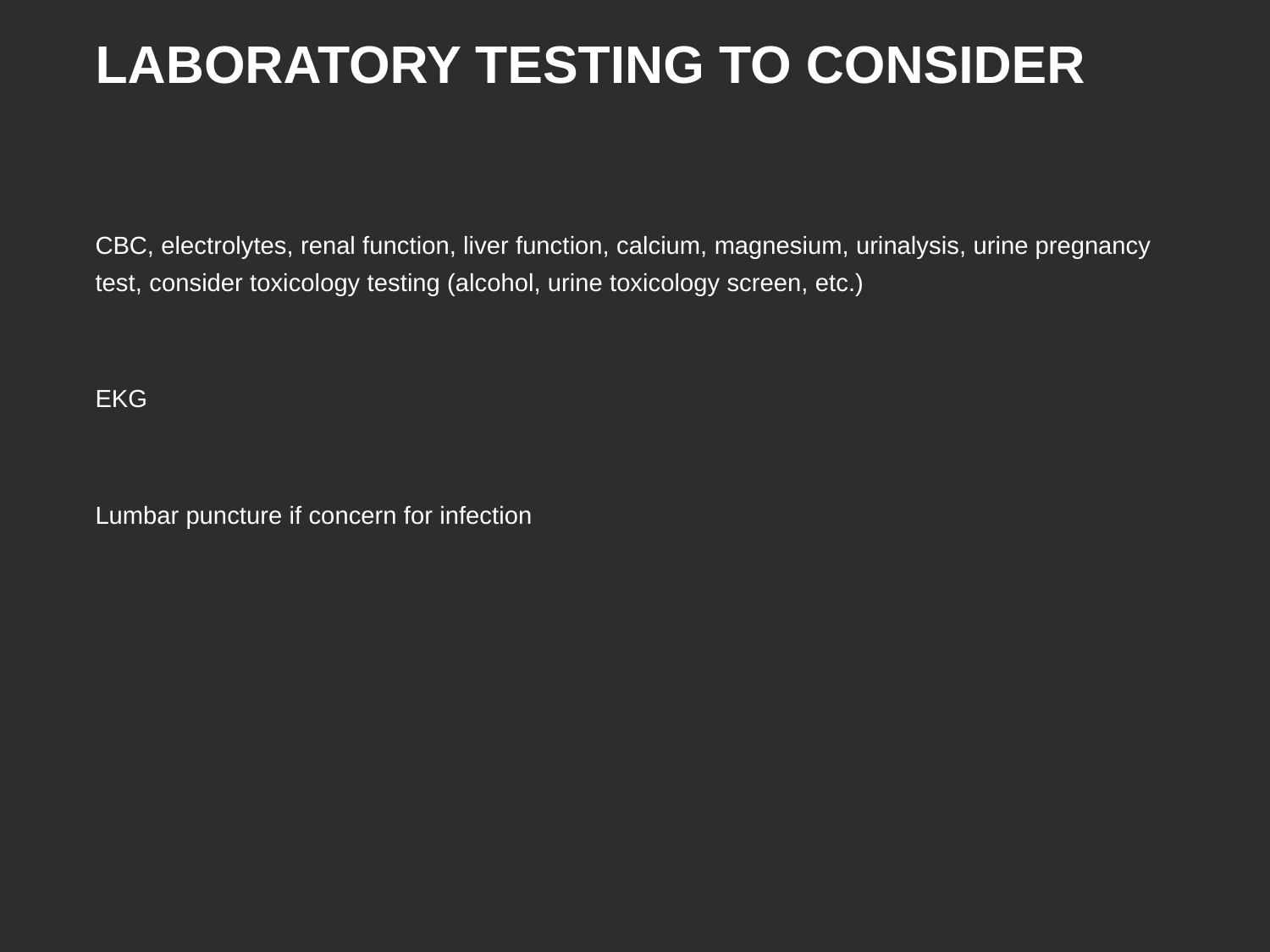

# Laboratory Testing to Consider
CBC, electrolytes, renal function, liver function, calcium, magnesium, urinalysis, urine pregnancy test, consider toxicology testing (alcohol, urine toxicology screen, etc.)
EKG
Lumbar puncture if concern for infection

## Slide 11
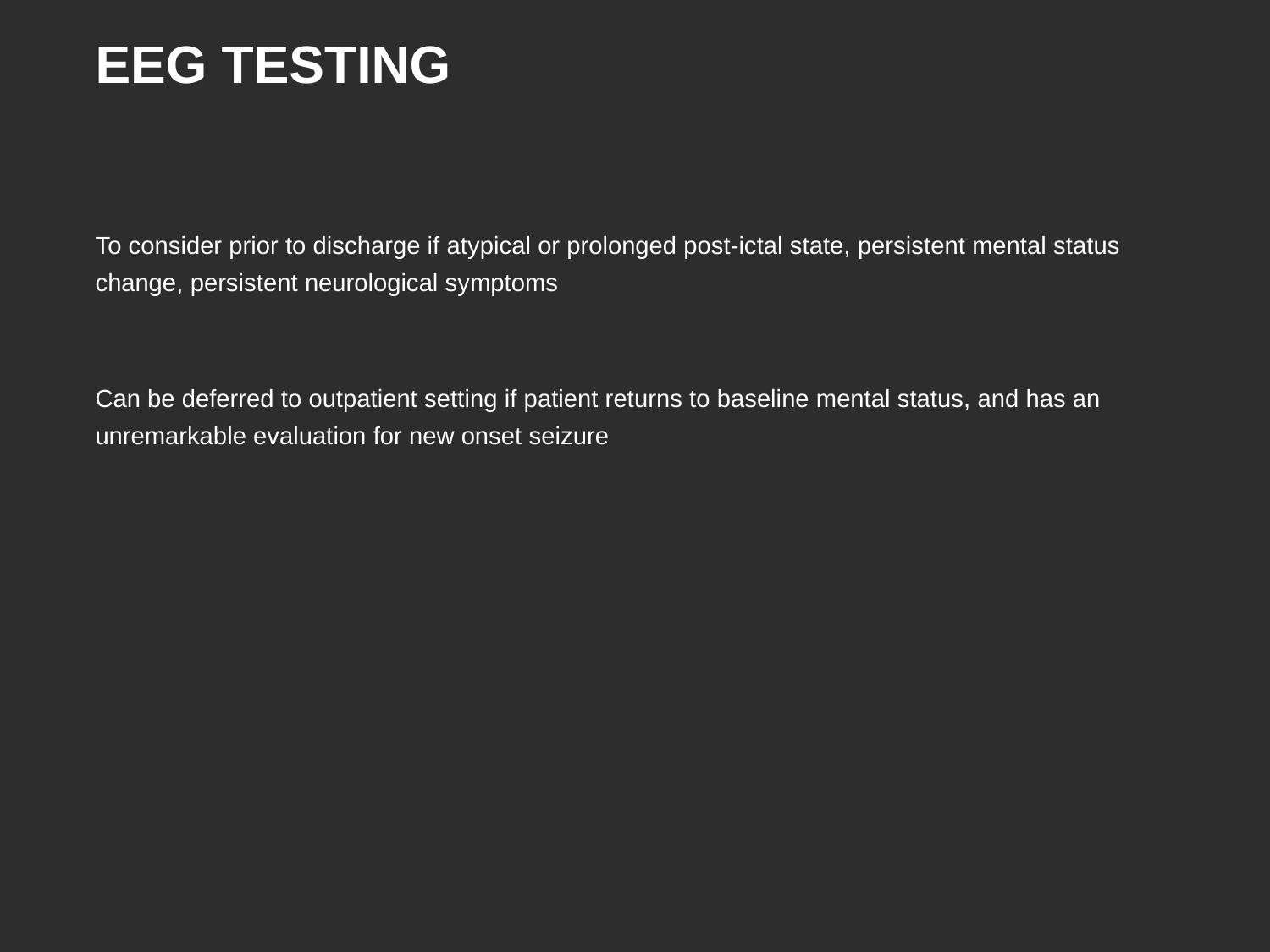

# EEG Testing
To consider prior to discharge if atypical or prolonged post-ictal state, persistent mental status change, persistent neurological symptoms
Can be deferred to outpatient setting if patient returns to baseline mental status, and has an unremarkable evaluation for new onset seizure

## Slide 12
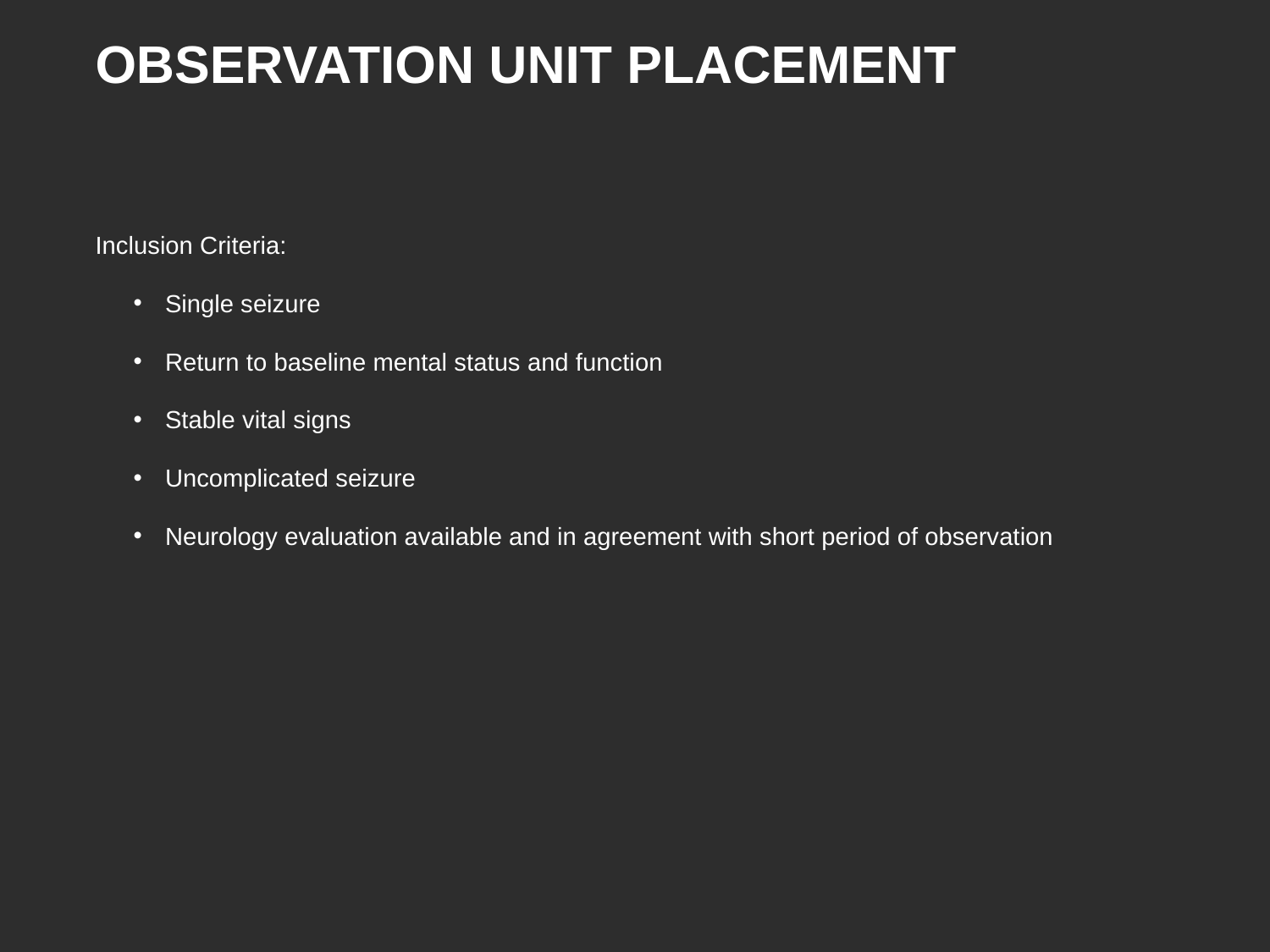

# Observation Unit Placement
Inclusion Criteria:
Single seizure
Return to baseline mental status and function
Stable vital signs
Uncomplicated seizure
Neurology evaluation available and in agreement with short period of observation

## Slide 13
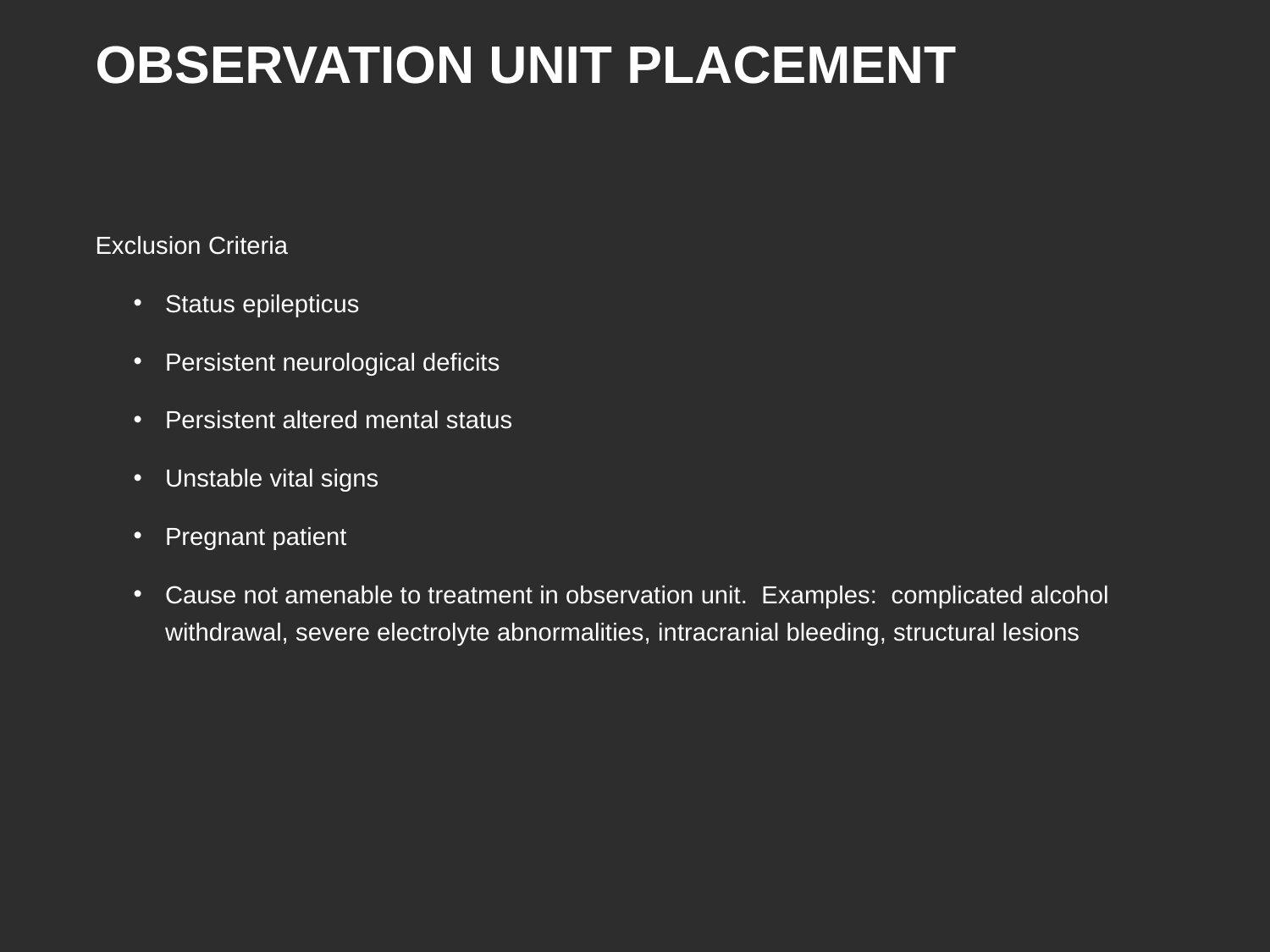

# Observation Unit Placement
Exclusion Criteria
Status epilepticus
Persistent neurological deficits
Persistent altered mental status
Unstable vital signs
Pregnant patient
Cause not amenable to treatment in observation unit.  Examples:  complicated alcohol withdrawal, severe electrolyte abnormalities, intracranial bleeding, structural lesions

## Slide 14
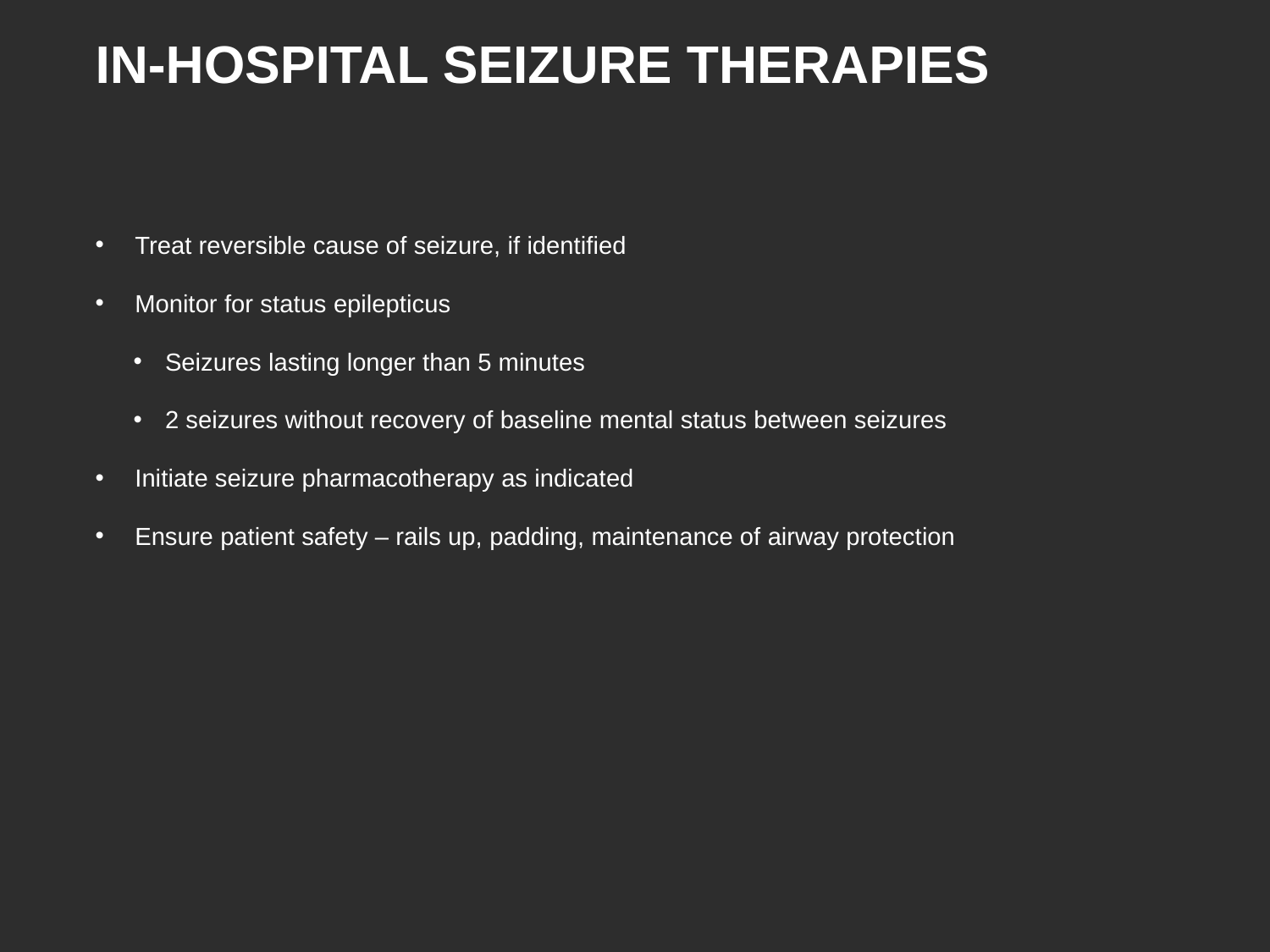

# In-Hospital Seizure Therapies
Treat reversible cause of seizure, if identified
Monitor for status epilepticus
Seizures lasting longer than 5 minutes
2 seizures without recovery of baseline mental status between seizures
Initiate seizure pharmacotherapy as indicated
Ensure patient safety – rails up, padding, maintenance of airway protection

## Slide 15
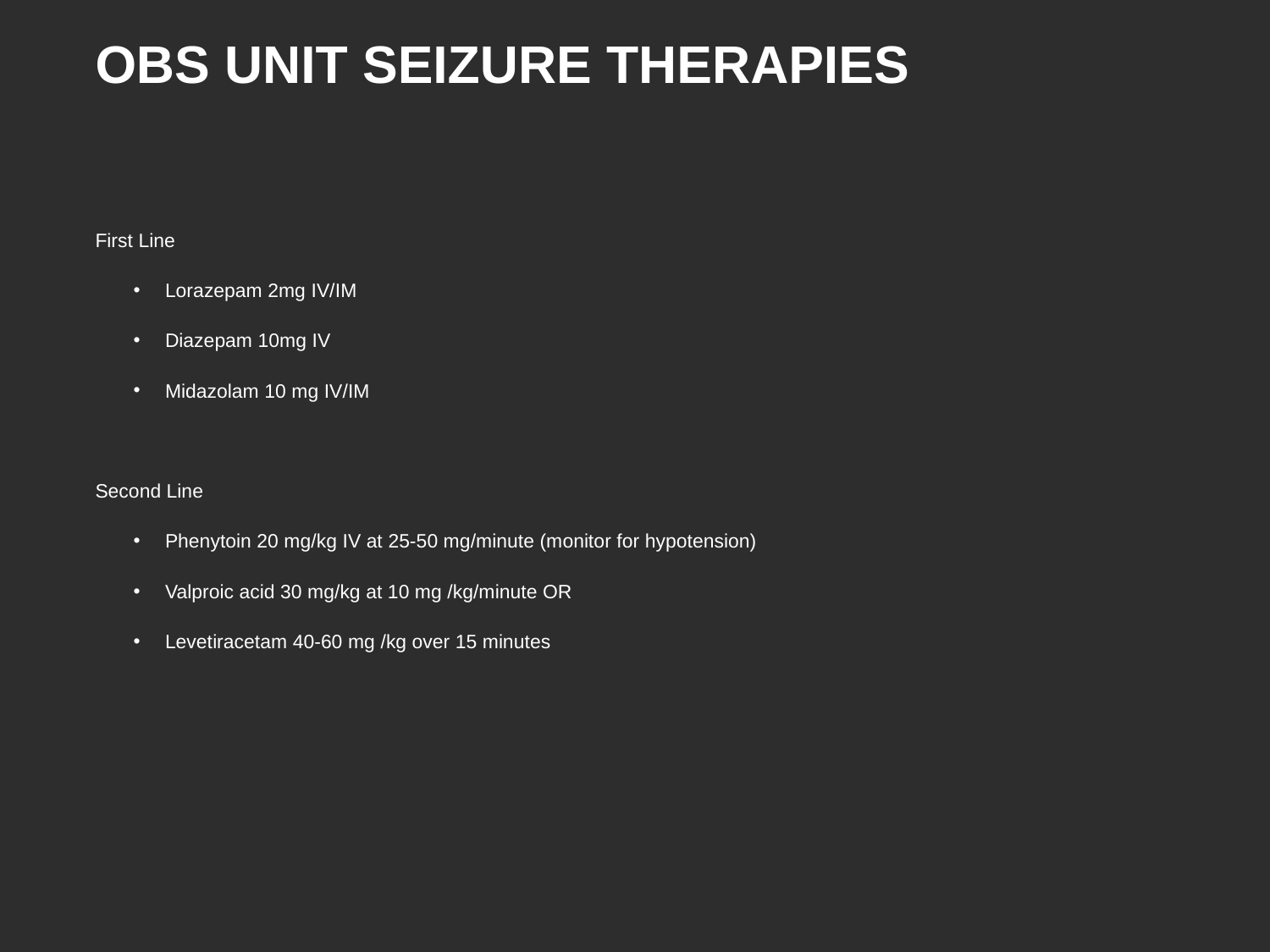

# Obs Unit Seizure Therapies
First Line
Lorazepam 2mg IV/IM
Diazepam 10mg IV
Midazolam 10 mg IV/IM
Second Line
Phenytoin 20 mg/kg IV at 25-50 mg/minute (monitor for hypotension)
Valproic acid 30 mg/kg at 10 mg /kg/minute OR
Levetiracetam 40-60 mg /kg over 15 minutes

## Slide 16
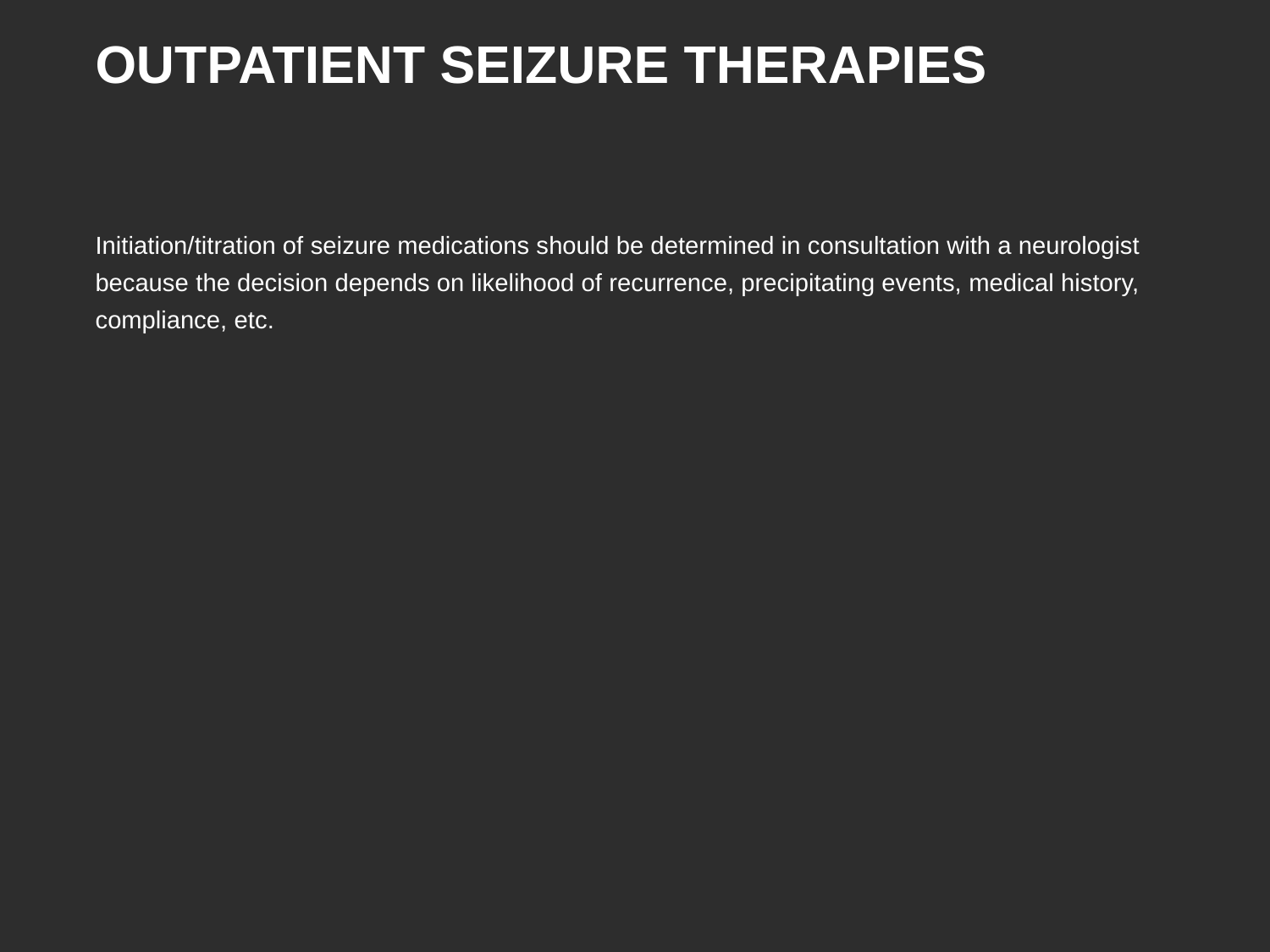

# Outpatient Seizure Therapies
Initiation/titration of seizure medications should be determined in consultation with a neurologist because the decision depends on likelihood of recurrence, precipitating events, medical history, compliance, etc.

## Slide 17
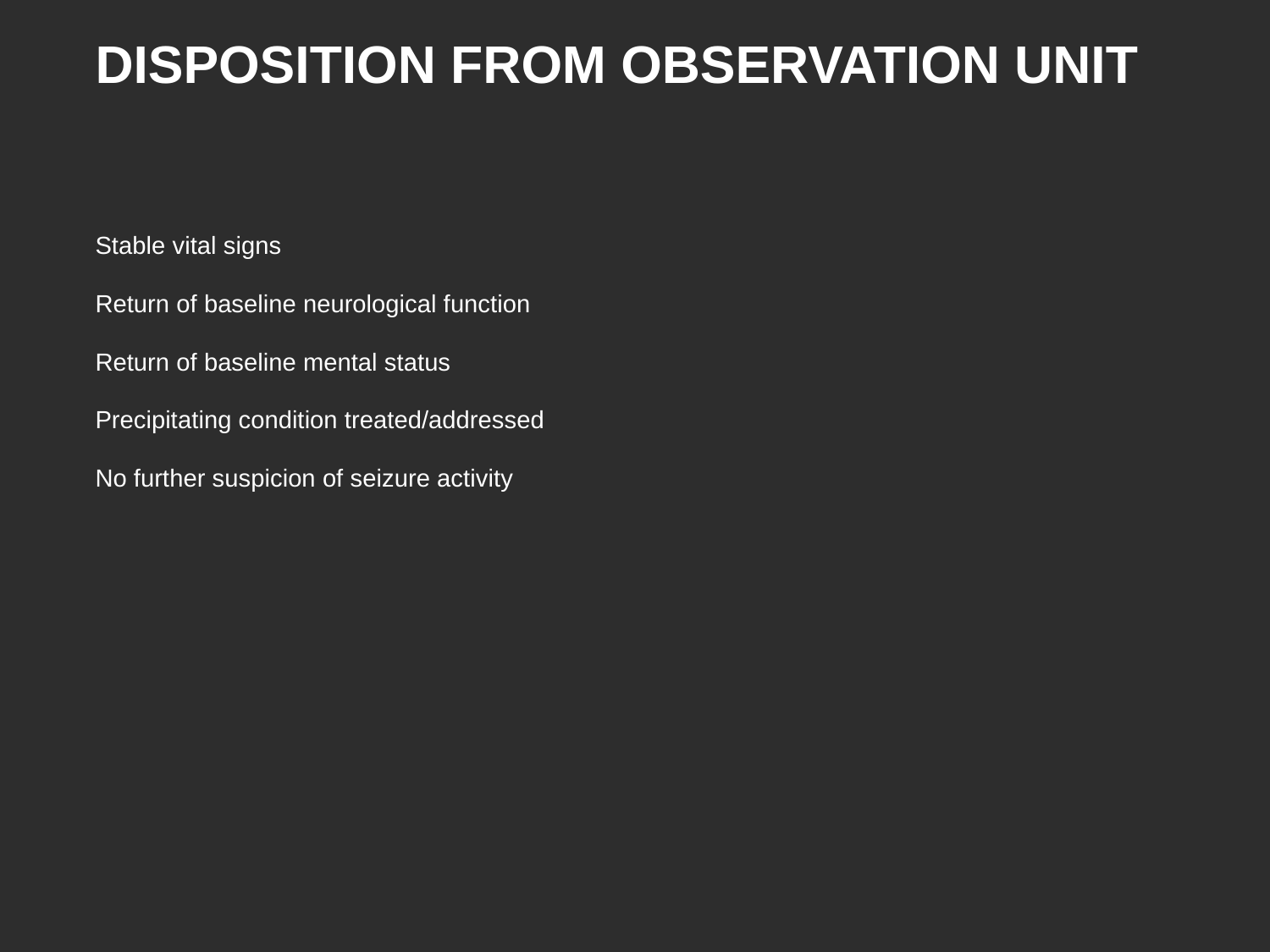

# Disposition from Observation Unit
Stable vital signs
Return of baseline neurological function
Return of baseline mental status
Precipitating condition treated/addressed
No further suspicion of seizure activity

## Slide 18
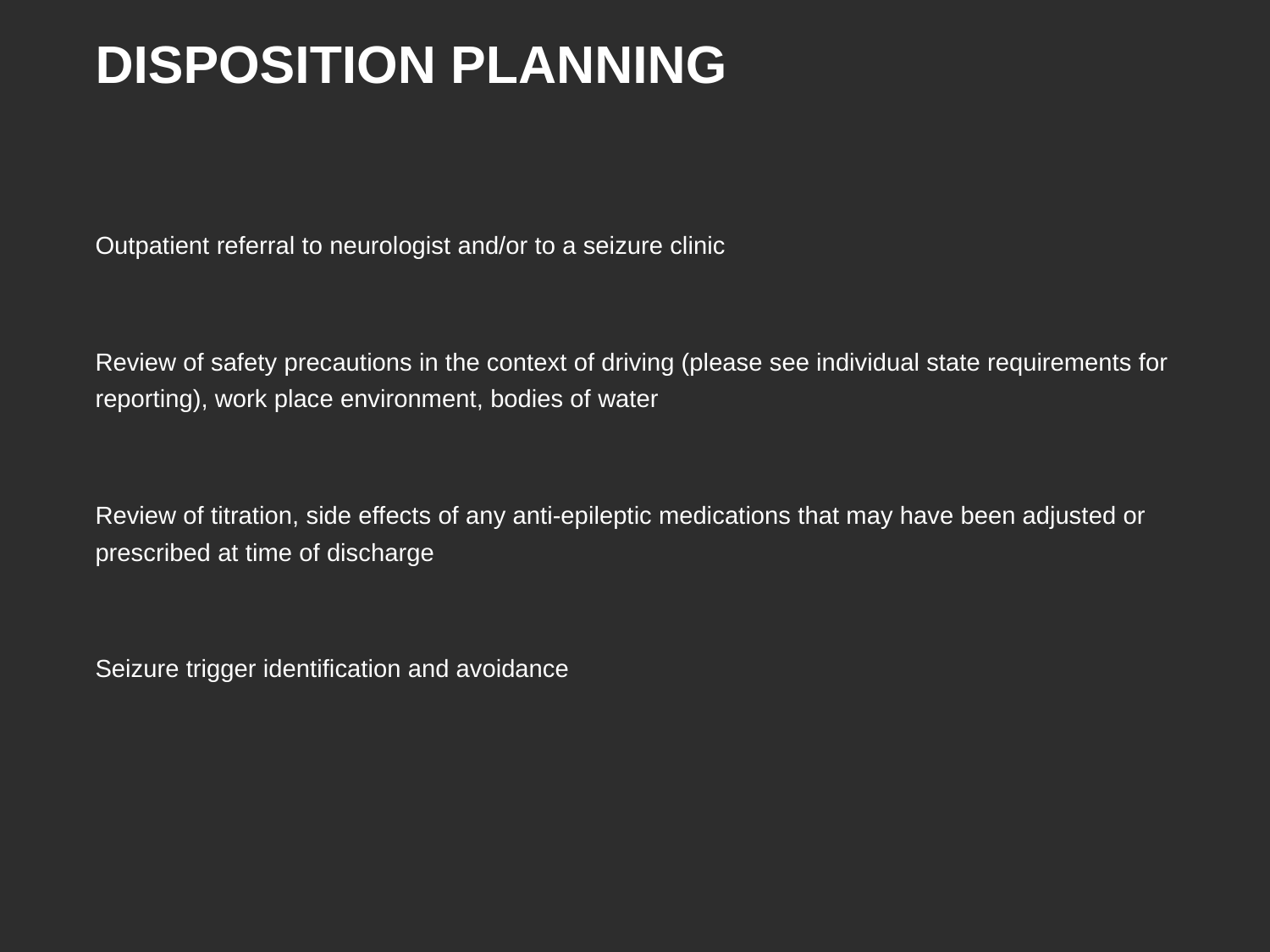

# Disposition Planning
Outpatient referral to neurologist and/or to a seizure clinic
Review of safety precautions in the context of driving (please see individual state requirements for reporting), work place environment, bodies of water
Review of titration, side effects of any anti-epileptic medications that may have been adjusted or prescribed at time of discharge
Seizure trigger identification and avoidance

## Slide 19
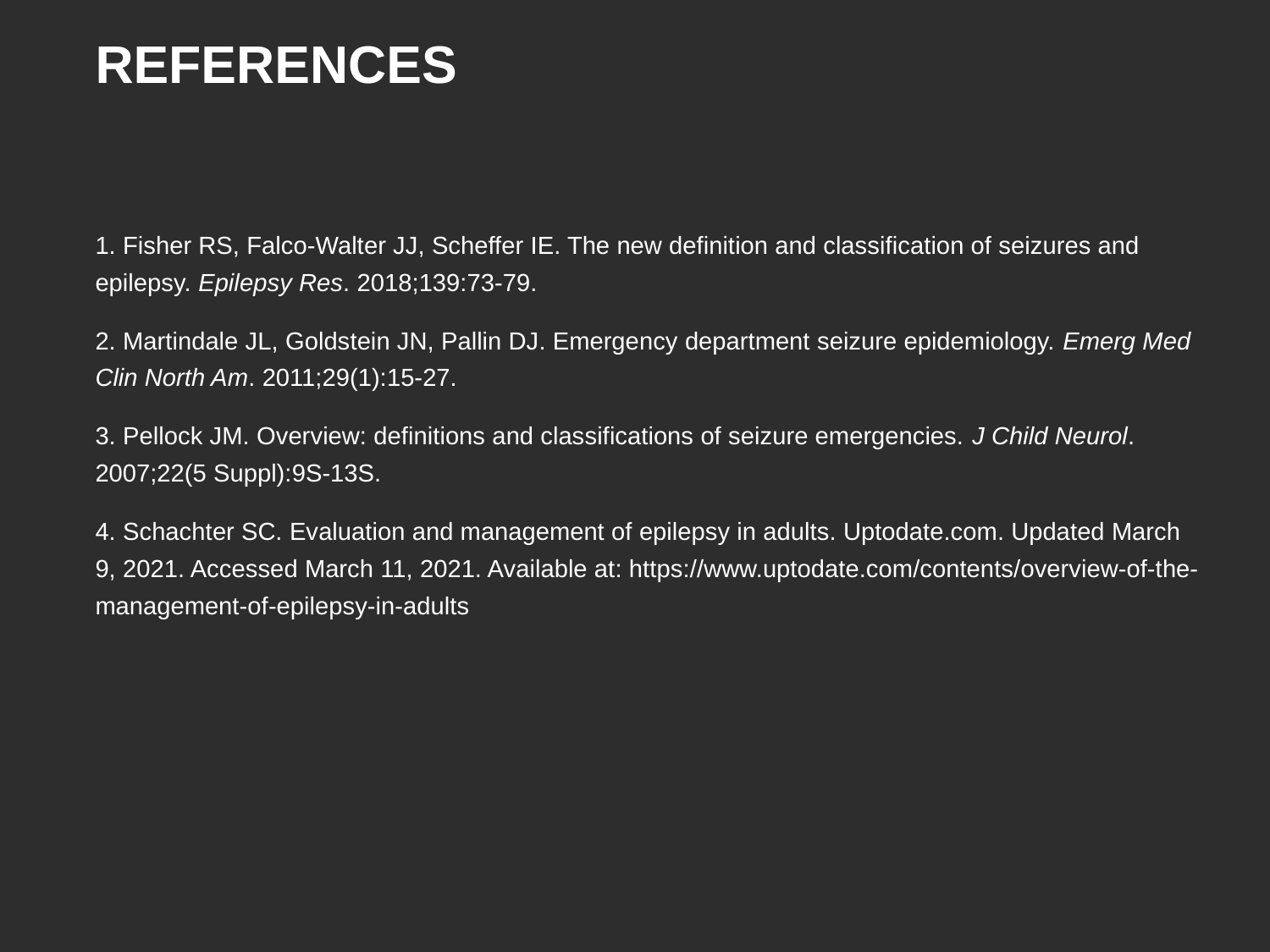

# References
1. Fisher RS, Falco-Walter JJ, Scheffer IE. The new definition and classification of seizures and epilepsy. Epilepsy Res. 2018;139:73-79.
2. Martindale JL, Goldstein JN, Pallin DJ. Emergency department seizure epidemiology. Emerg Med Clin North Am. 2011;29(1):15-27.
3. Pellock JM. Overview: definitions and classifications of seizure emergencies. J Child Neurol. 2007;22(5 Suppl):9S-13S.
4. Schachter SC. Evaluation and management of epilepsy in adults. Uptodate.com. Updated March 9, 2021. Accessed March 11, 2021. Available at: https://www.uptodate.com/contents/overview-of-the-management-of-epilepsy-in-adults
