# Supplementary material for: An Observation Medicine Curriculum for Emergency Medicine Education
Source: J Educ Teach Emerg Med. 2021 Apr 19;6(2):C1–C72. doi: 10.21980/J87P92 (PMC10332786; doi:10.21980/J87P92)
Supplement: Supplementary file 27 — Please see associated PowerPoint file [file jetem-6-2-c1-supp27.pptx]

## Slide 1
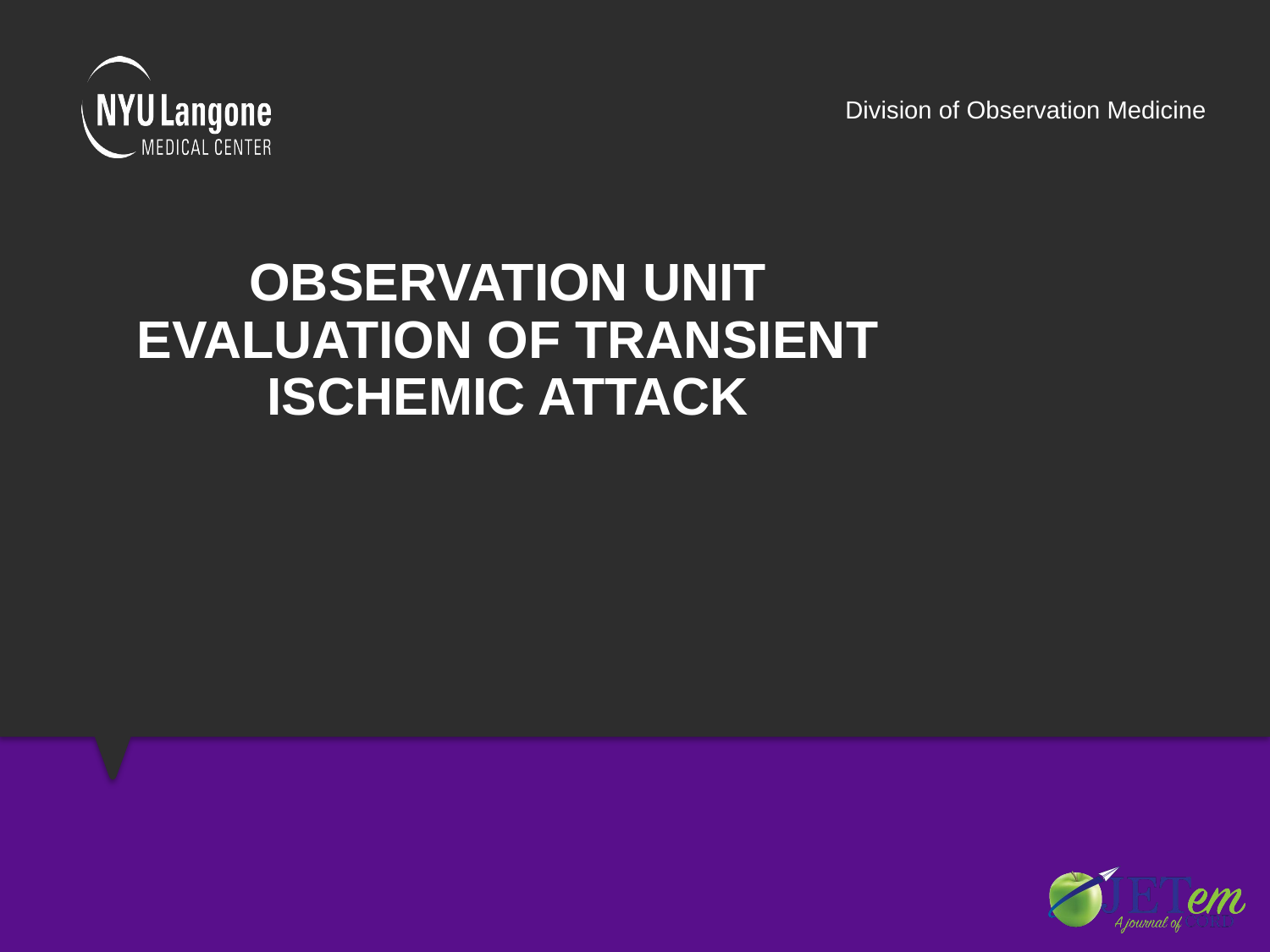

Division of Observation Medicine
# Observation Unit Evaluation of Transient Ischemic Attack

## Slide 2
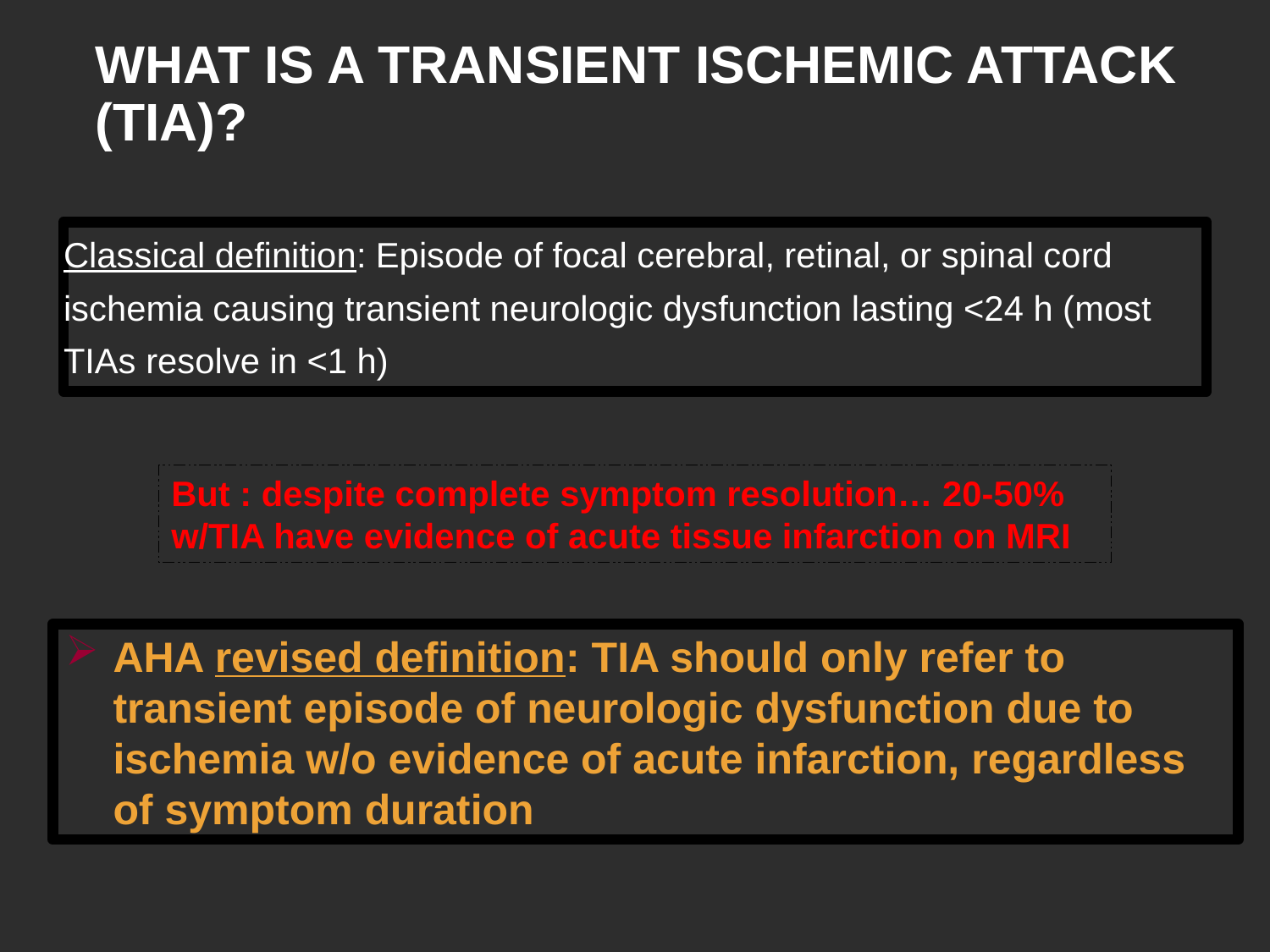

# What is a transient ischemic attack (TIA)?
Classical definition: Episode of focal cerebral, retinal, or spinal cord ischemia causing transient neurologic dysfunction lasting <24 h (most TIAs resolve in <1 h)
But : despite complete symptom resolution… 20-50% w/TIA have evidence of acute tissue infarction on MRI
AHA revised definition: TIA should only refer to transient episode of neurologic dysfunction due to ischemia w/o evidence of acute infarction, regardless of symptom duration

## Slide 3
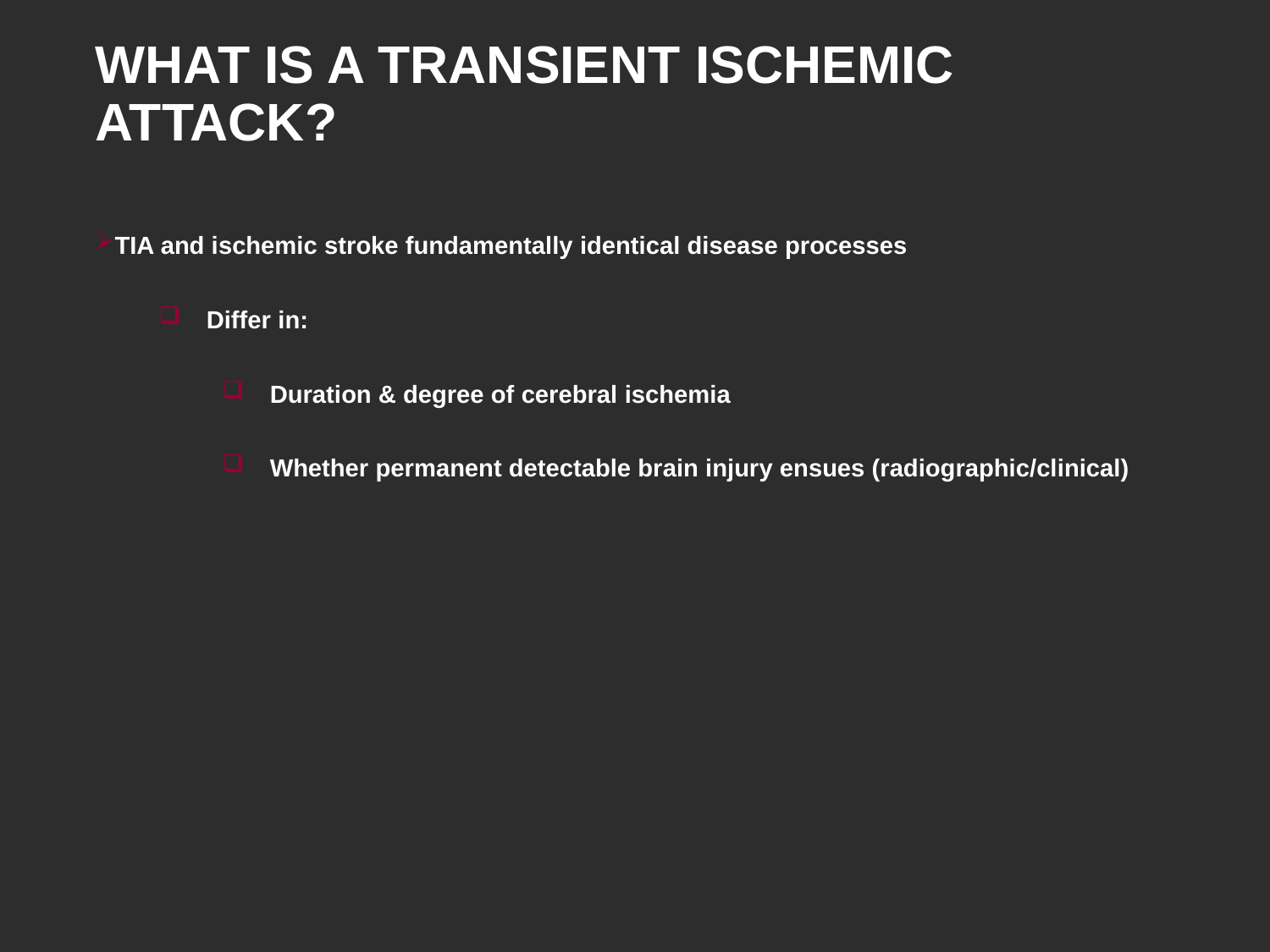

# What is a transient ischemic attack?
TIA and ischemic stroke fundamentally identical disease processes
Differ in:
Duration & degree of cerebral ischemia
Whether permanent detectable brain injury ensues (radiographic/clinical)

## Slide 4
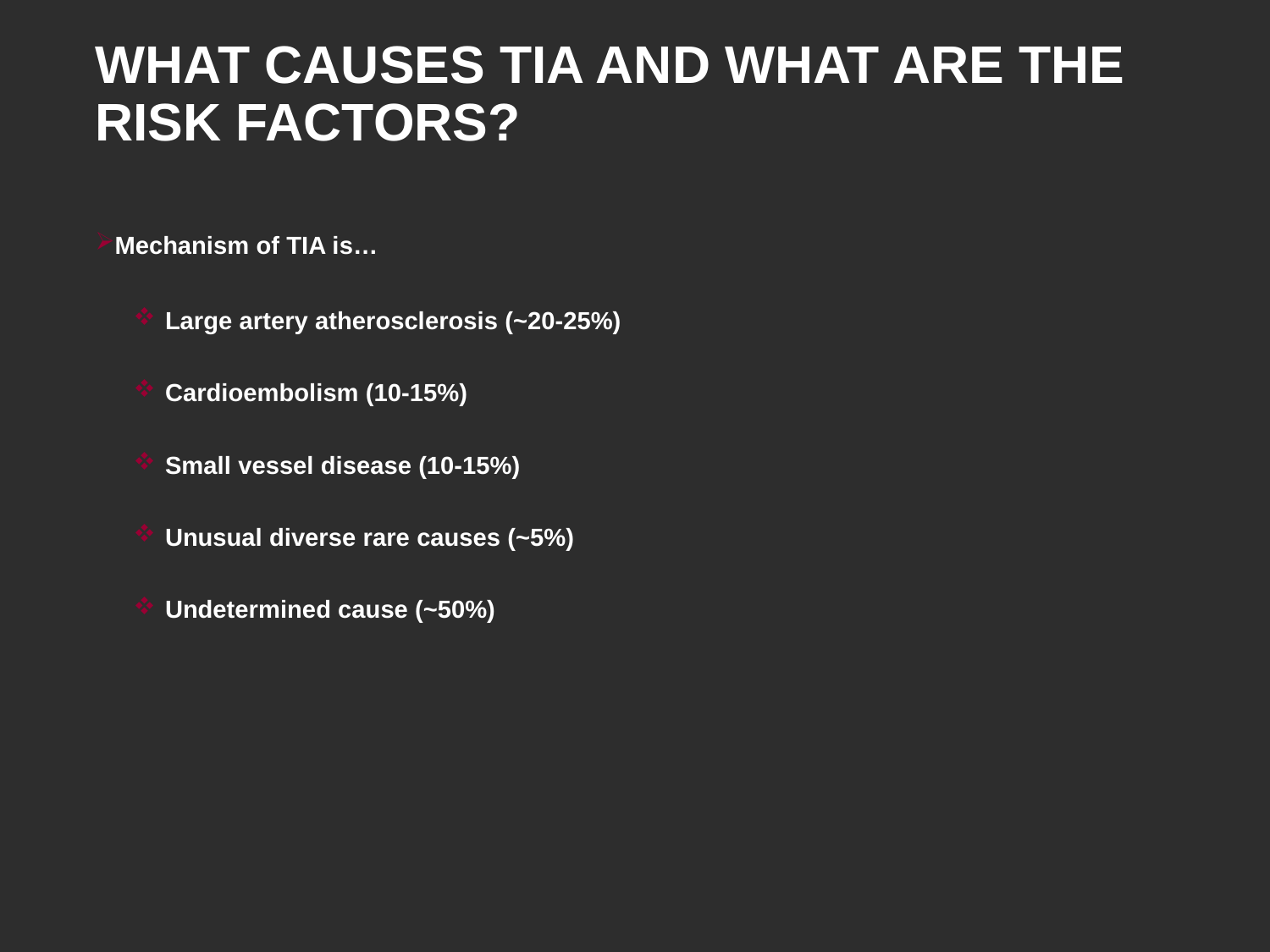

# What causes TIA and what are the risk factors?
Mechanism of TIA is…
Large artery atherosclerosis (~20-25%)
Cardioembolism (10-15%)
Small vessel disease (10-15%)
Unusual diverse rare causes (~5%)
Undetermined cause (~50%)

## Slide 5
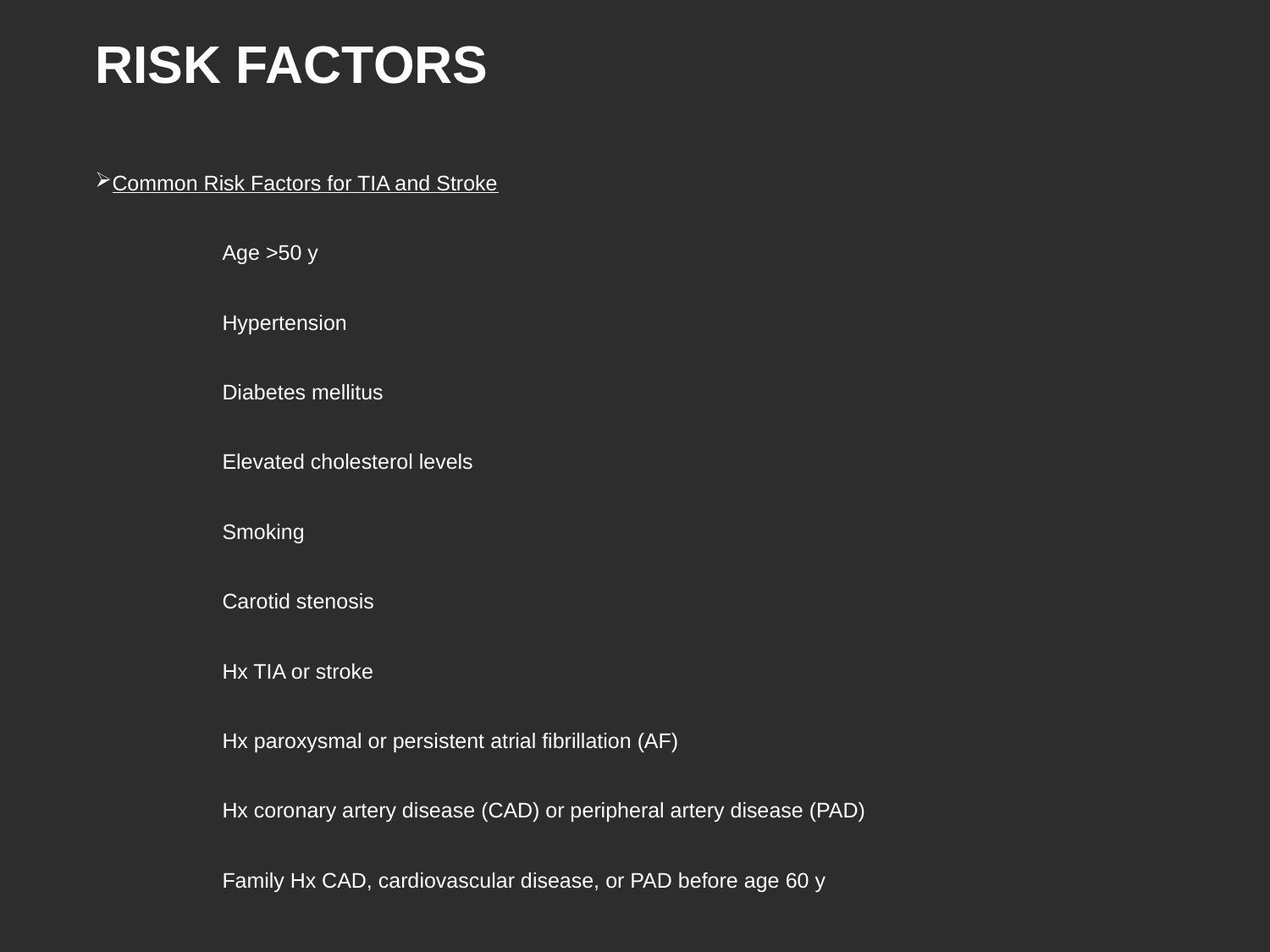

# Risk factors
Common Risk Factors for TIA and Stroke
Age >50 y
Hypertension
Diabetes mellitus
Elevated cholesterol levels
Smoking
Carotid stenosis
Hx TIA or stroke
Hx paroxysmal or persistent atrial fibrillation (AF)
Hx coronary artery disease (CAD) or peripheral artery disease (PAD)
Family Hx CAD, cardiovascular disease, or PAD before age 60 y

## Slide 6
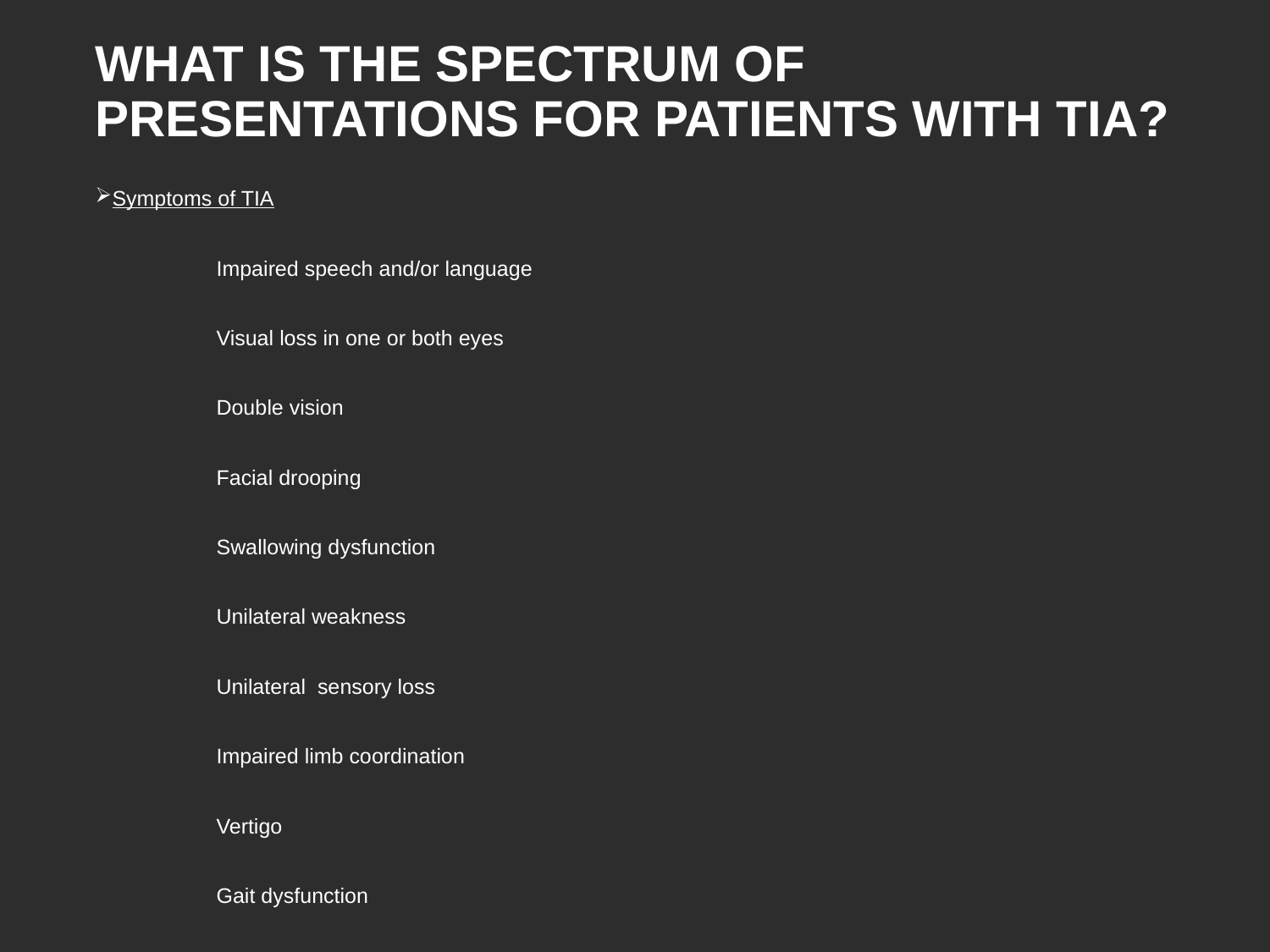

# What is the spectrum of presentations for patients with TIA?
Symptoms of TIA
Impaired speech and/or language
Visual loss in one or both eyes
Double vision
Facial drooping
Swallowing dysfunction
Unilateral weakness
Unilateral sensory loss
Impaired limb coordination
Vertigo
Gait dysfunction

## Slide 7
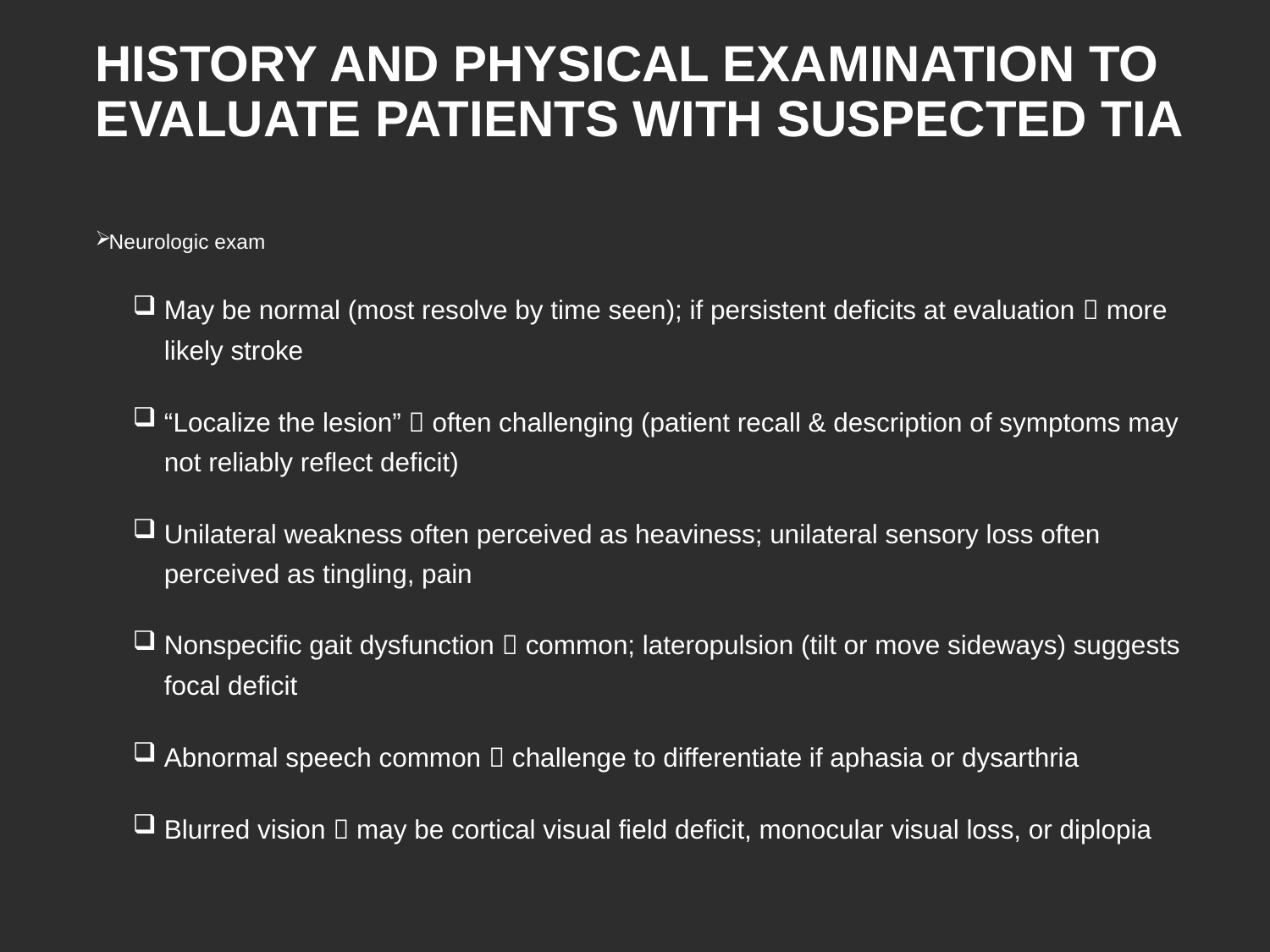

# History and physical examination to evaluate patients with suspected TIA
Neurologic exam
May be normal (most resolve by time seen); if persistent deficits at evaluation  more likely stroke
“Localize the lesion”  often challenging (patient recall & description of symptoms may not reliably reflect deficit)
Unilateral weakness often perceived as heaviness; unilateral sensory loss often perceived as tingling, pain
Nonspecific gait dysfunction  common; lateropulsion (tilt or move sideways) suggests focal deficit
Abnormal speech common  challenge to differentiate if aphasia or dysarthria
Blurred vision  may be cortical visual field deficit, monocular visual loss, or diplopia

## Slide 8
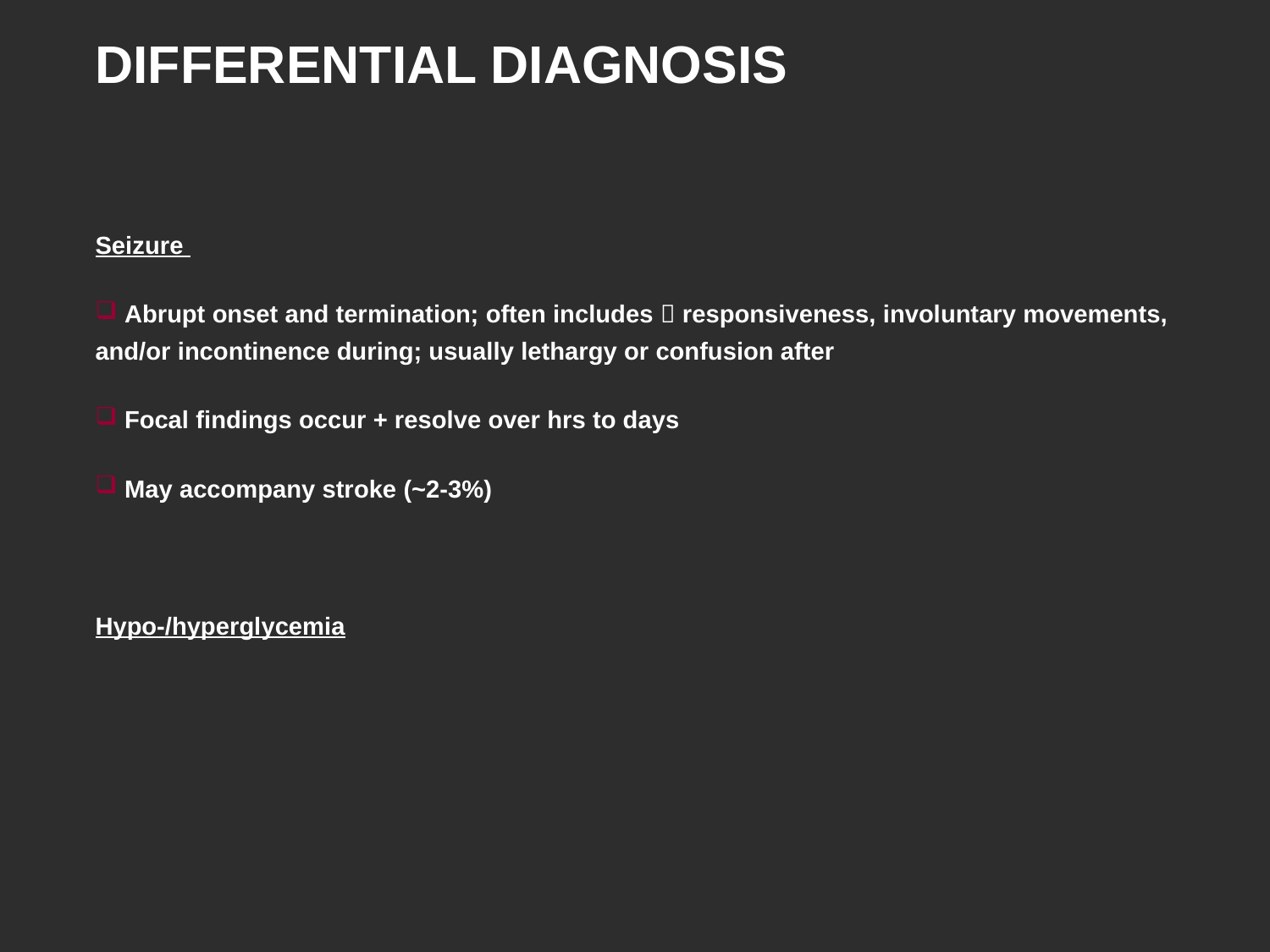

# Differential Diagnosis
Seizure
 Abrupt onset and termination; often includes  responsiveness, involuntary movements, and/or incontinence during; usually lethargy or confusion after
 Focal findings occur + resolve over hrs to days
 May accompany stroke (~2-3%)
Hypo-/hyperglycemia

## Slide 9
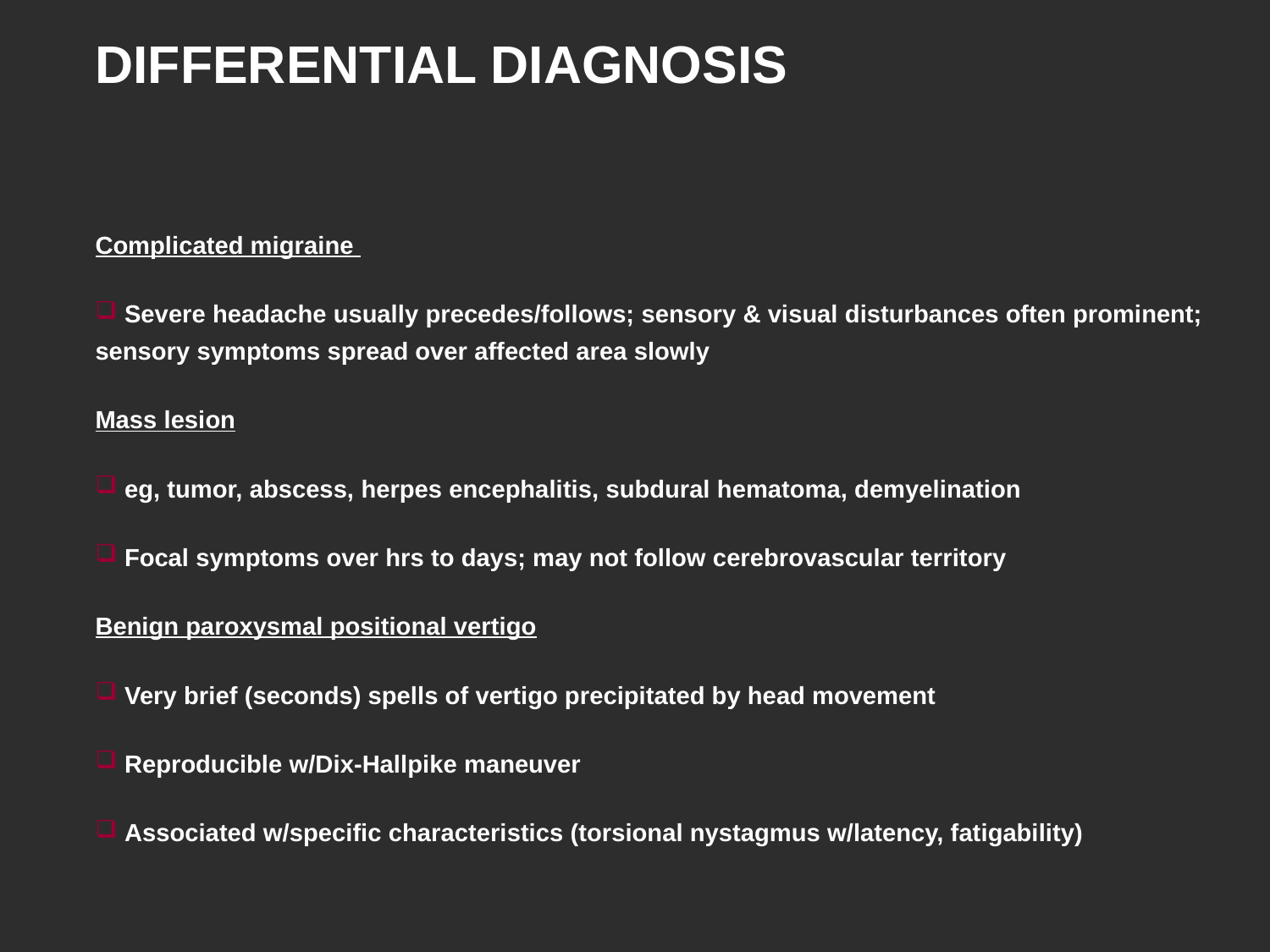

# Differential Diagnosis
Complicated migraine
 Severe headache usually precedes/follows; sensory & visual disturbances often prominent; sensory symptoms spread over affected area slowly
Mass lesion
 eg, tumor, abscess, herpes encephalitis, subdural hematoma, demyelination
 Focal symptoms over hrs to days; may not follow cerebrovascular territory
Benign paroxysmal positional vertigo
 Very brief (seconds) spells of vertigo precipitated by head movement
 Reproducible w/Dix-Hallpike maneuver
 Associated w/specific characteristics (torsional nystagmus w/latency, fatigability)

## Slide 10
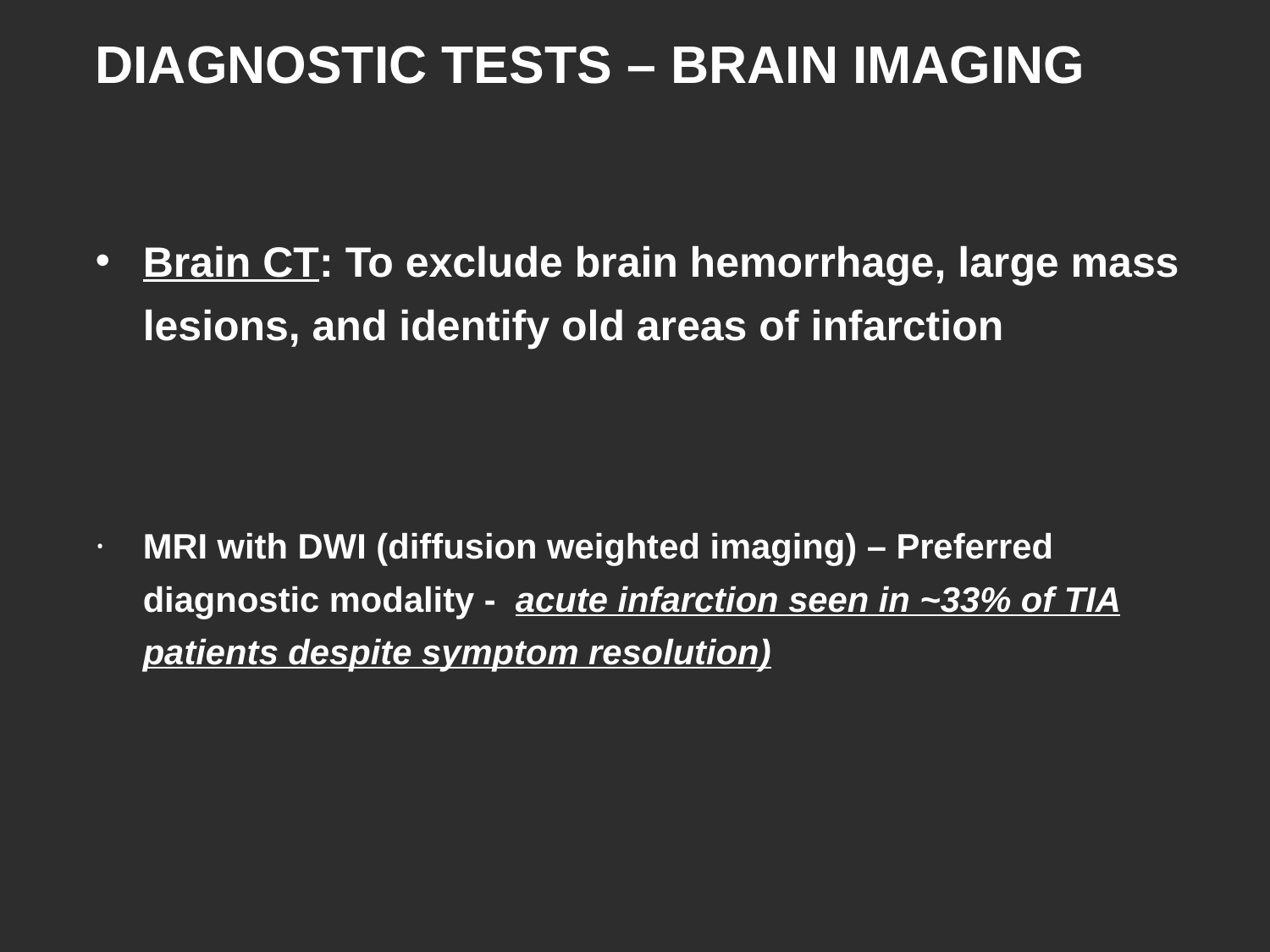

# Diagnostic Tests – Brain imaging
Brain CT: To exclude brain hemorrhage, large mass lesions, and identify old areas of infarction
MRI with DWI (diffusion weighted imaging) – Preferred diagnostic modality -  acute infarction seen in ~33% of TIA patients despite symptom resolution)

## Slide 11
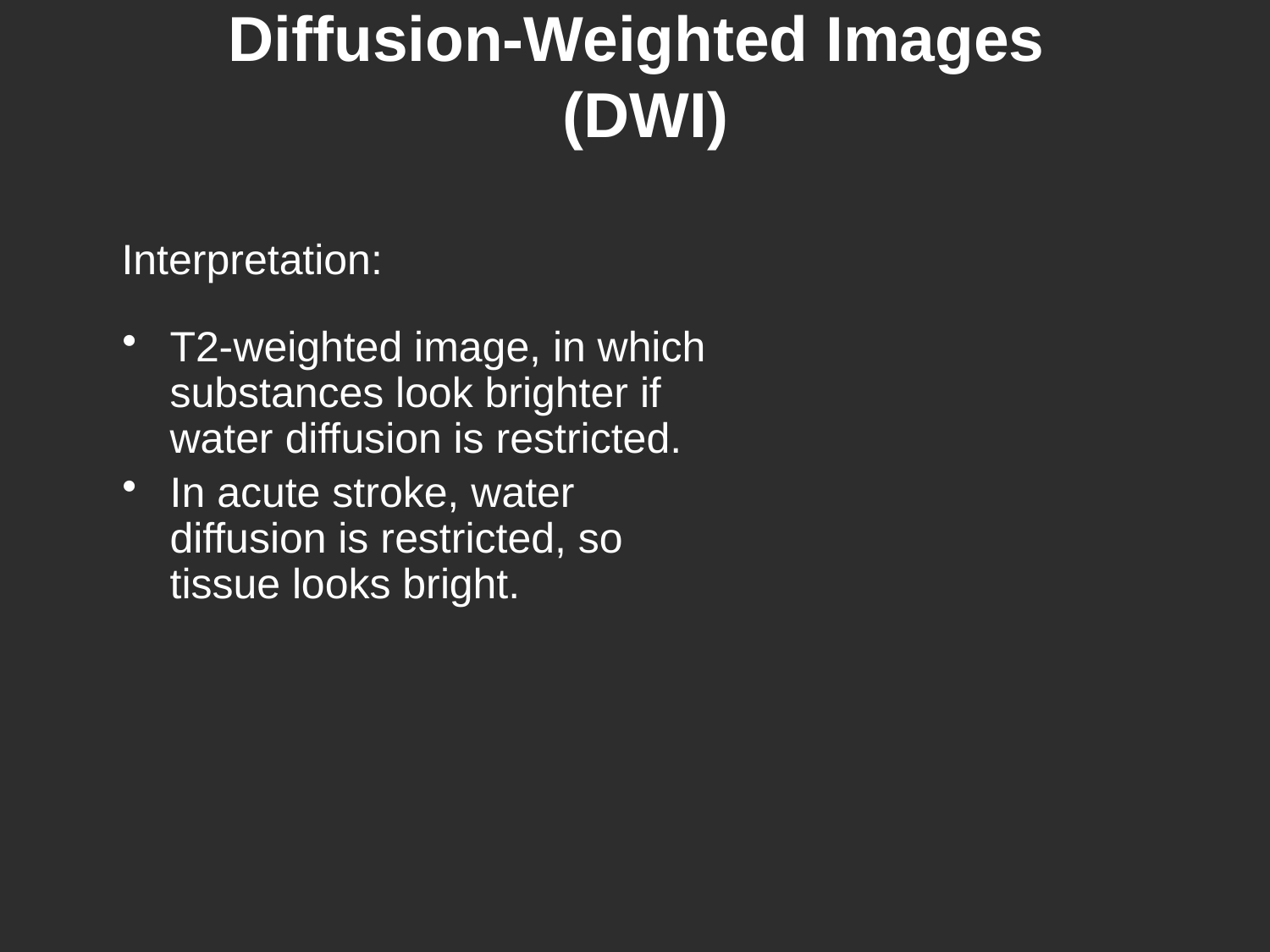

Diffusion-Weighted Images (DWI)
Interpretation:
T2-weighted image, in which substances look brighter if water diffusion is restricted.
In acute stroke, water diffusion is restricted, so tissue looks bright.

## Slide 12
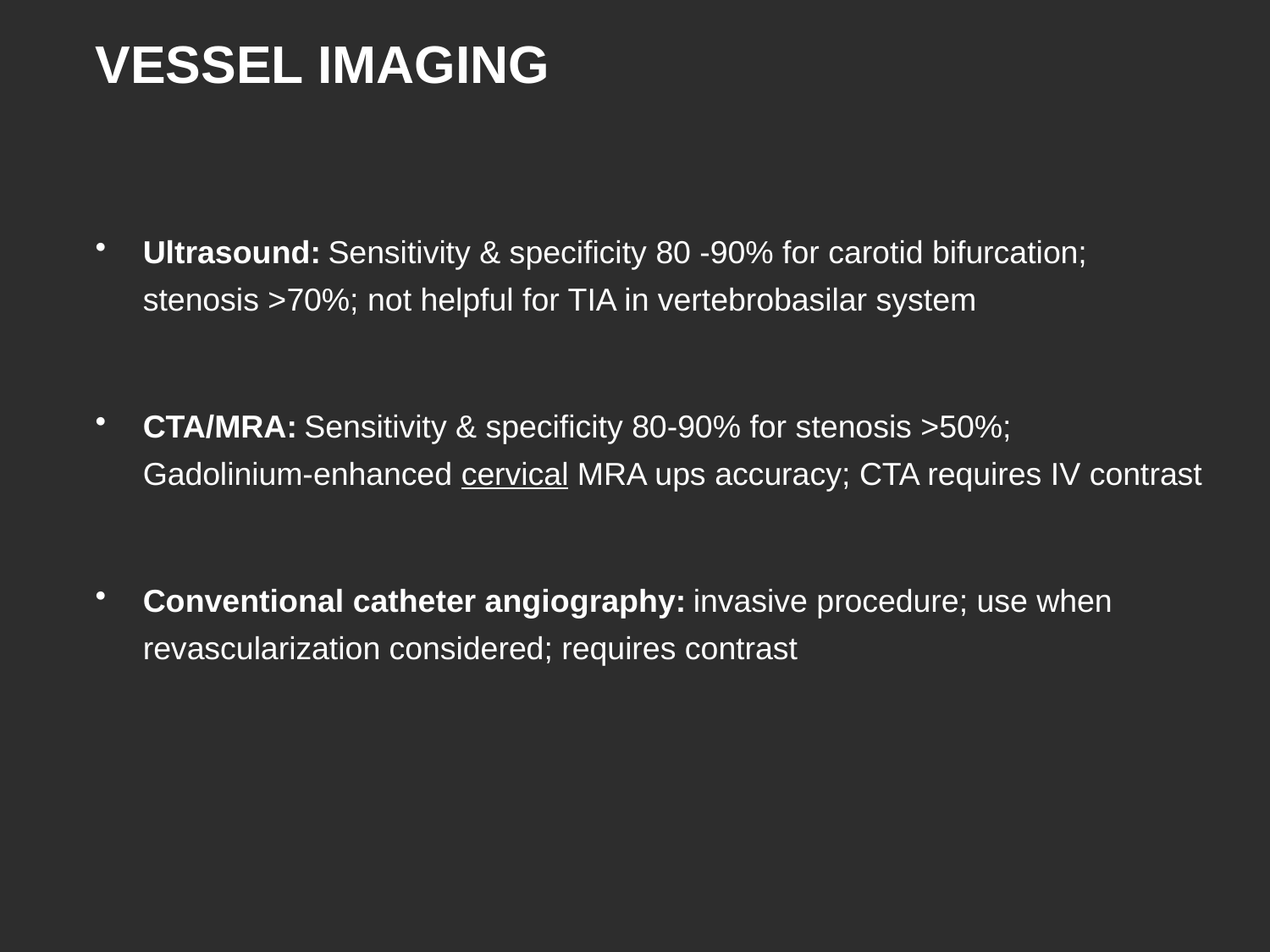

# Vessel imaging
Ultrasound: Sensitivity & specificity 80 -90% for carotid bifurcation; stenosis >70%; not helpful for TIA in vertebrobasilar system
CTA/MRA: Sensitivity & specificity 80-90% for stenosis >50%;
Gadolinium-enhanced cervical MRA ups accuracy; CTA requires IV contrast
Conventional catheter angiography: invasive procedure; use when revascularization considered; requires contrast

## Slide 13
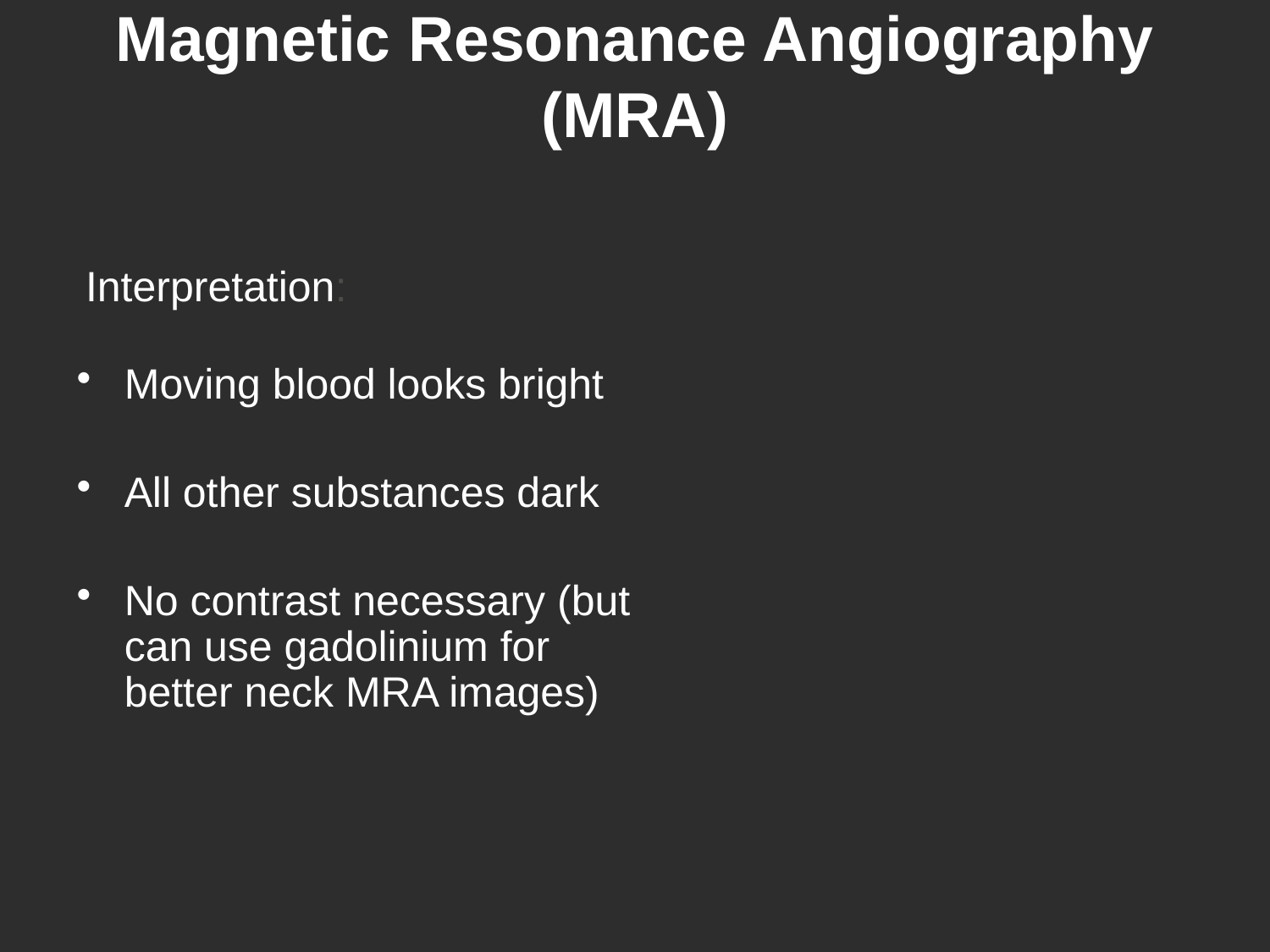

Magnetic Resonance Angiography (MRA)
Interpretation:
Moving blood looks bright
All other substances dark
No contrast necessary (but can use gadolinium for better neck MRA images)

## Slide 14
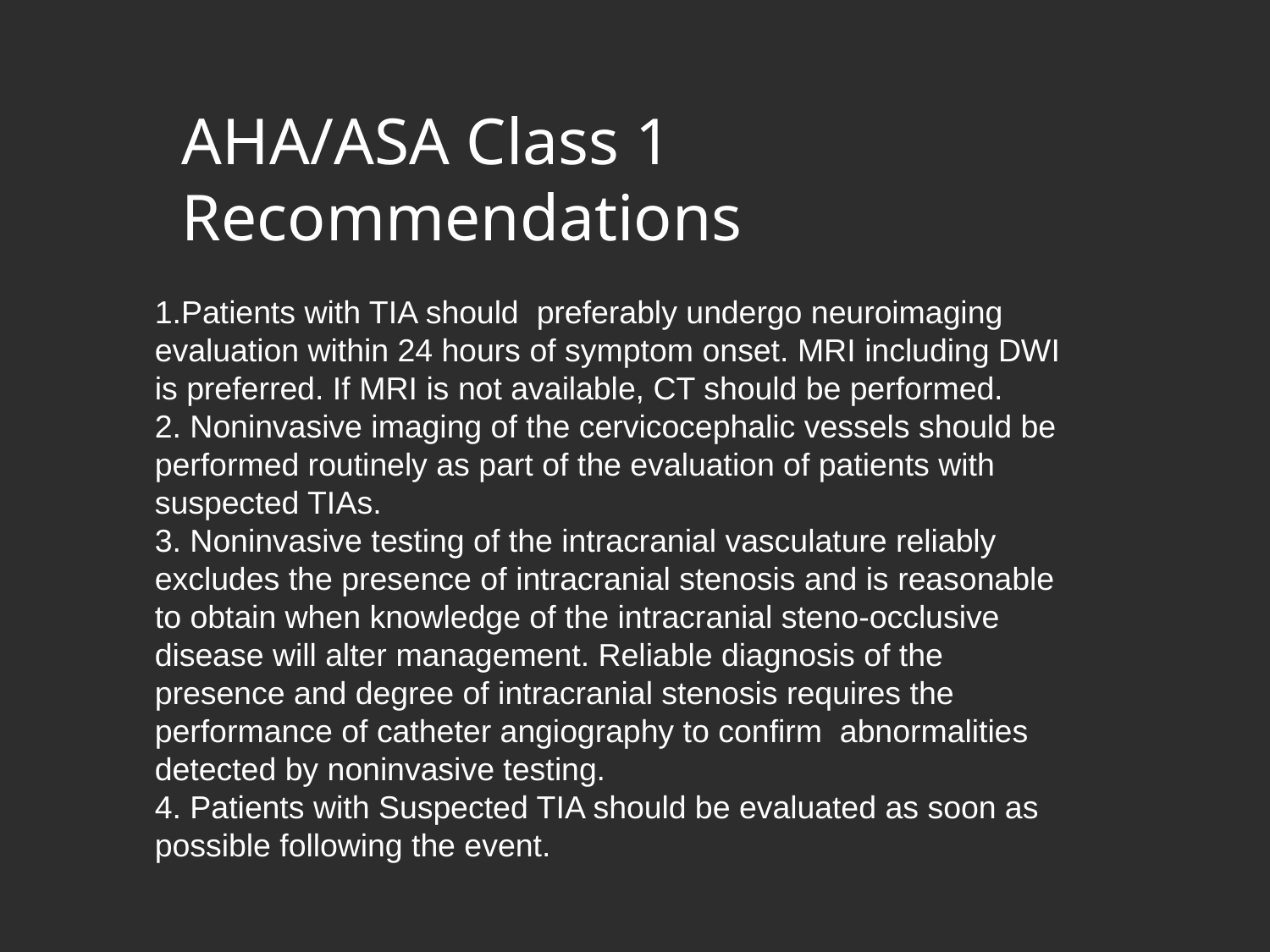

AHA/ASA Class 1 Recommendations
1.Patients with TIA should  preferably undergo neuroimaging evaluation within 24 hours of symptom onset. MRI including DWI is preferred. If MRI is not available, CT should be performed.
2. Noninvasive imaging of the cervicocephalic vessels should be performed routinely as part of the evaluation of patients with suspected TIAs.
3. Noninvasive testing of the intracranial vasculature reliably excludes the presence of intracranial stenosis and is reasonable to obtain when knowledge of the intracranial steno-occlusive disease will alter management. Reliable diagnosis of the presence and degree of intracranial stenosis requires the performance of catheter angiography to confirm  abnormalities detected by noninvasive testing.
4. Patients with Suspected TIA should be evaluated as soon as possible following the event.

## Slide 15
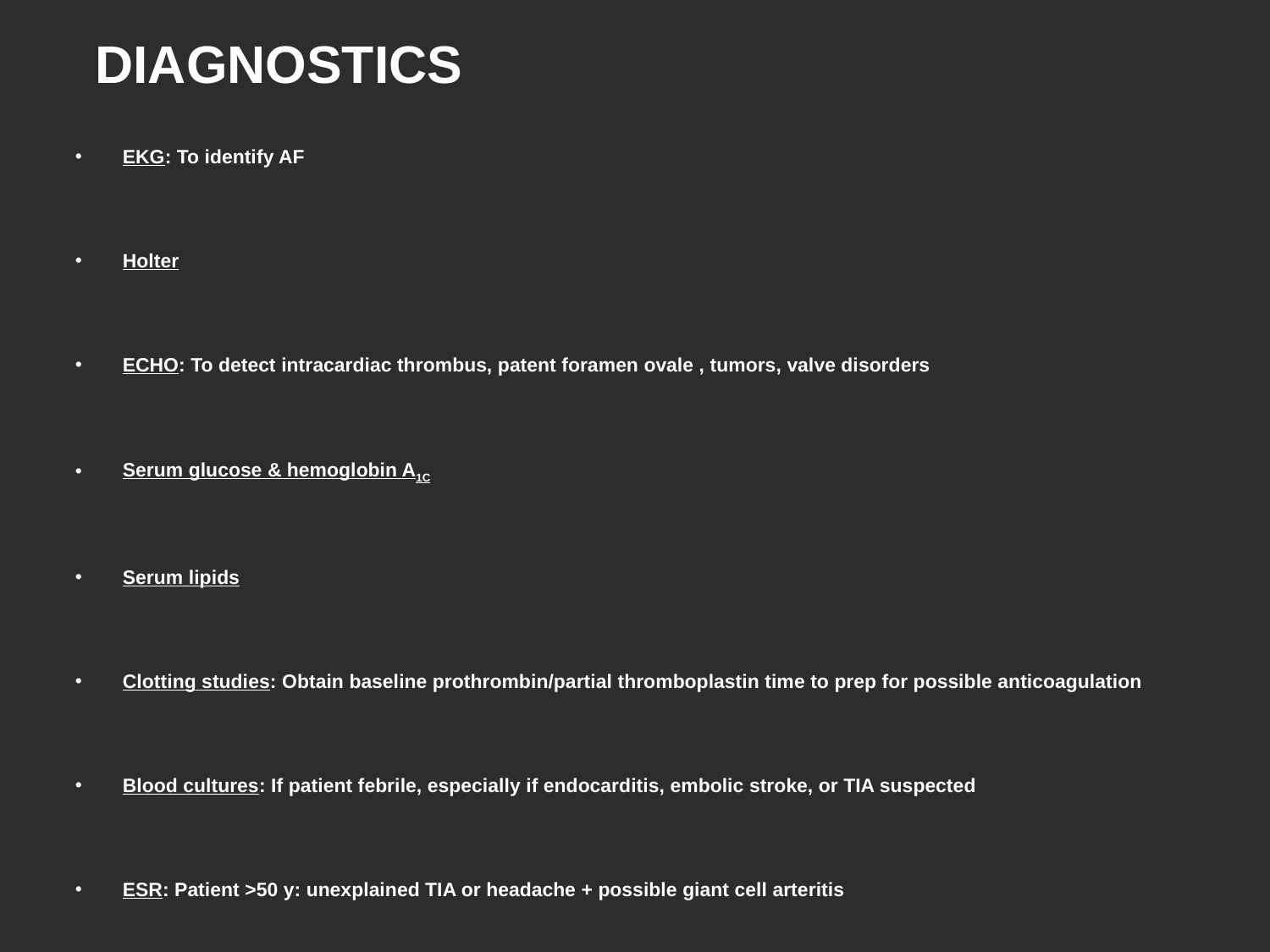

# Diagnostics
EKG: To identify AF
Holter
ECHO: To detect intracardiac thrombus, patent foramen ovale , tumors, valve disorders
Serum glucose & hemoglobin A1C
Serum lipids
Clotting studies: Obtain baseline prothrombin/partial thromboplastin time to prep for possible anticoagulation
Blood cultures: If patient febrile, especially if endocarditis, embolic stroke, or TIA suspected
ESR: Patient >50 y: unexplained TIA or headache + possible giant cell arteritis

## Slide 16
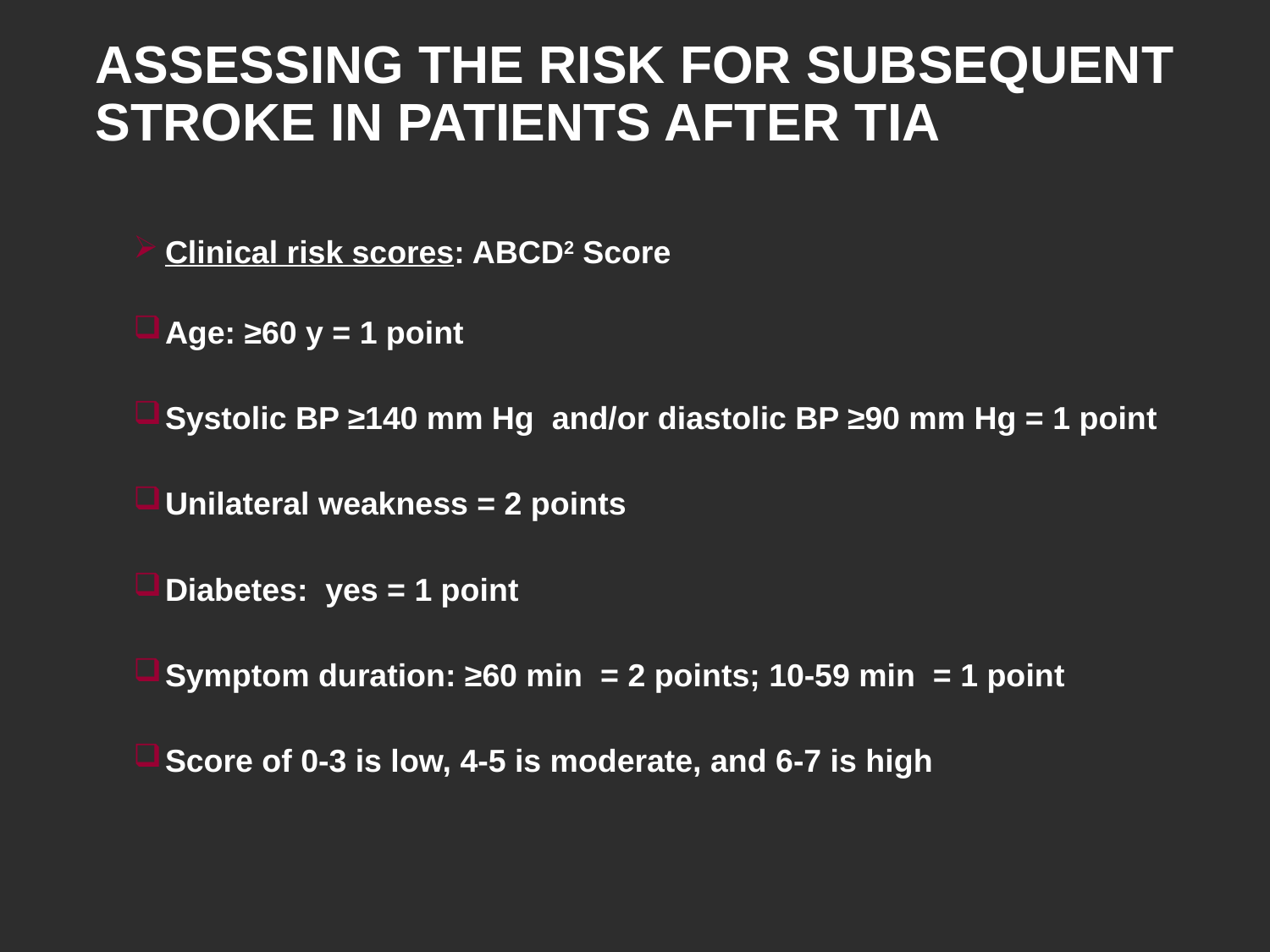

# Assessing the risk for subsequent stroke in patients after TIA
Clinical risk scores: ABCD2 Score
Age: ≥60 y = 1 point
Systolic BP ≥140 mm Hg and/or diastolic BP ≥90 mm Hg = 1 point
Unilateral weakness = 2 points
Diabetes: yes = 1 point
Symptom duration: ≥60 min = 2 points; 10-59 min = 1 point
Score of 0-3 is low, 4-5 is moderate, and 6-7 is high

## Slide 17
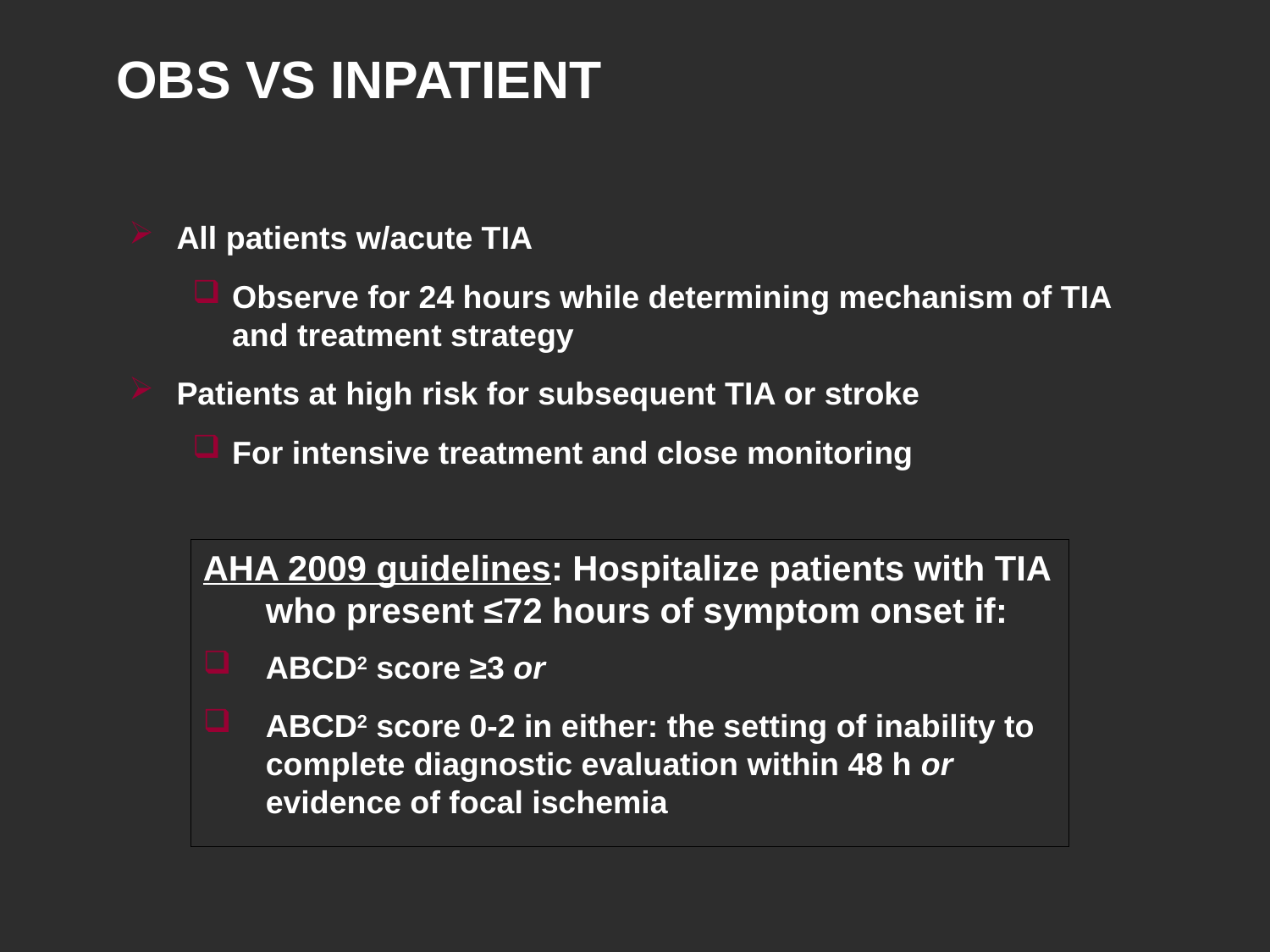

# OBS vs Inpatient
All patients w/acute TIA
Observe for 24 hours while determining mechanism of TIA and treatment strategy
Patients at high risk for subsequent TIA or stroke
For intensive treatment and close monitoring
AHA 2009 guidelines: Hospitalize patients with TIA who present ≤72 hours of symptom onset if:
ABCD2 score ≥3 or
ABCD2 score 0-2 in either: the setting of inability to complete diagnostic evaluation within 48 h or evidence of focal ischemia

## Slide 18
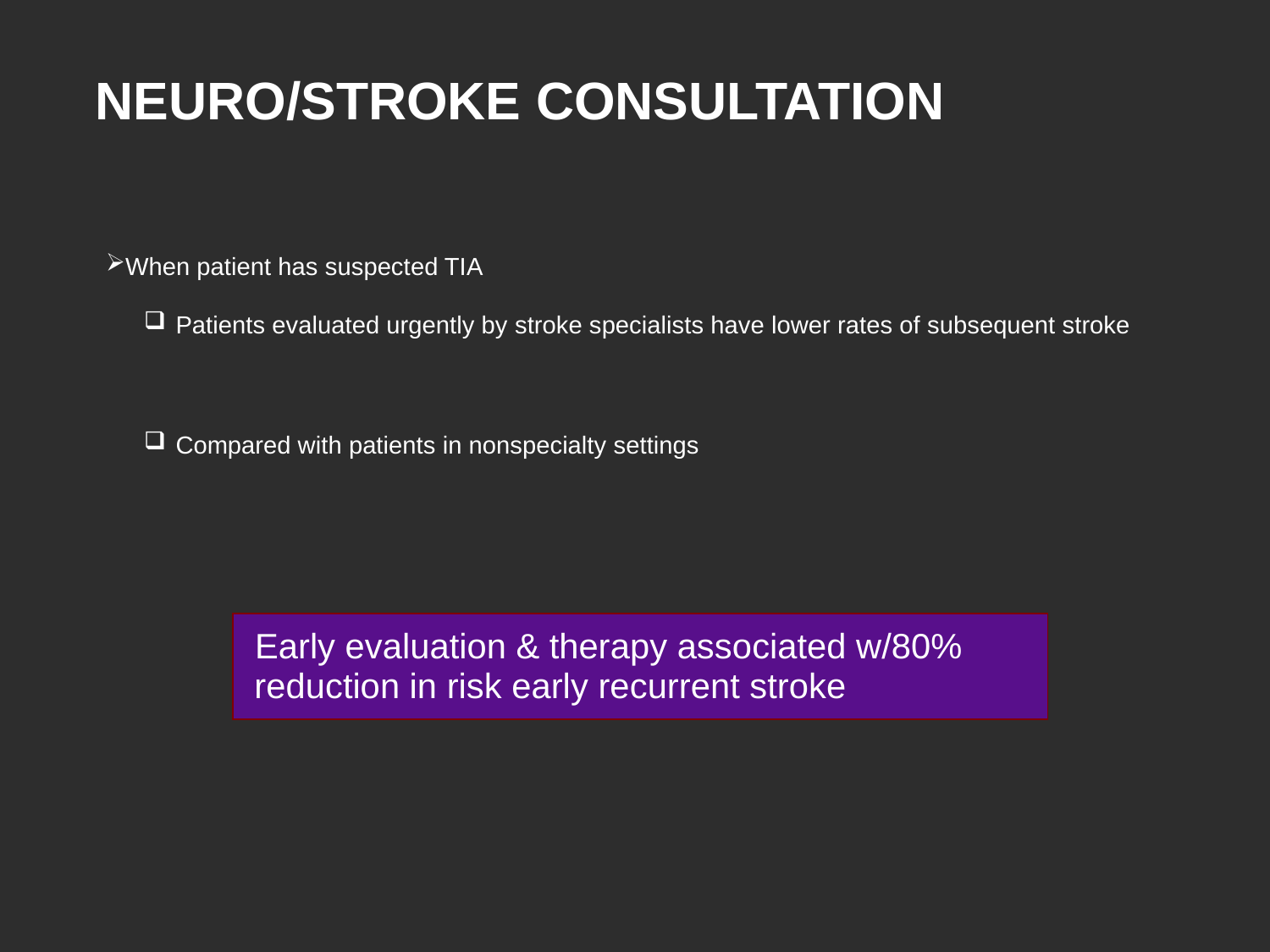

# Neuro/Stroke Consultation
When patient has suspected TIA
Patients evaluated urgently by stroke specialists have lower rates of subsequent stroke
Compared with patients in nonspecialty settings
| Early evaluation & therapy associated w/80% reduction in risk early recurrent stroke |
| --- |

## Slide 19
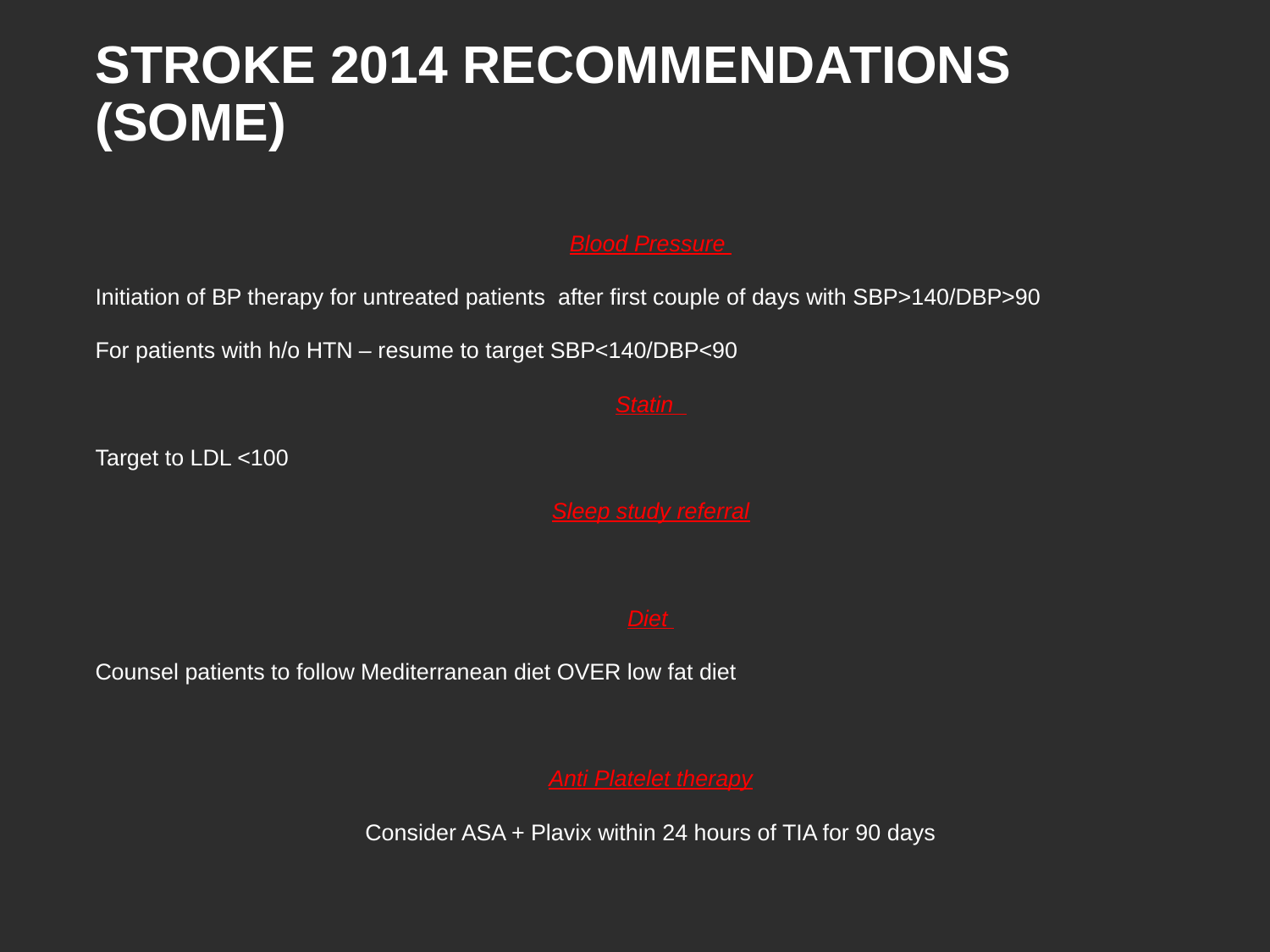

# STROKE 2014 recommendations (some)
Blood Pressure
Initiation of BP therapy for untreated patients after first couple of days with SBP>140/DBP>90
For patients with h/o HTN – resume to target SBP<140/DBP<90
Statin
Target to LDL <100
Sleep study referral
Diet
Counsel patients to follow Mediterranean diet OVER low fat diet
Anti Platelet therapy
Consider ASA + Plavix within 24 hours of TIA for 90 days

## Slide 20
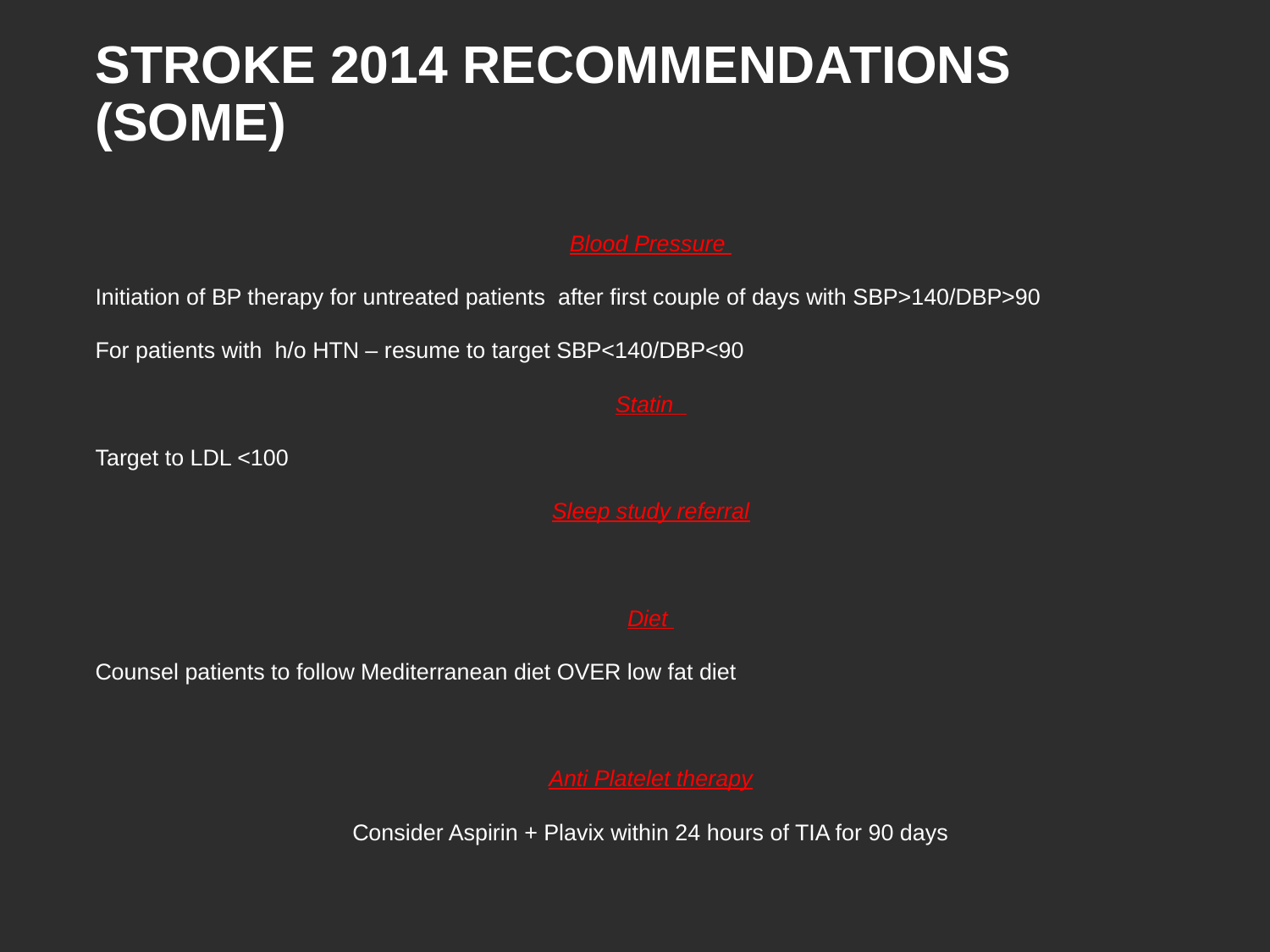

# STROKE 2014 recommendations (some)
Blood Pressure
Initiation of BP therapy for untreated patients after first couple of days with SBP>140/DBP>90
For patients with h/o HTN – resume to target SBP<140/DBP<90
Statin
Target to LDL <100
Sleep study referral
Diet
Counsel patients to follow Mediterranean diet OVER low fat diet
Anti Platelet therapy
Consider Aspirin + Plavix within 24 hours of TIA for 90 days

## Slide 21
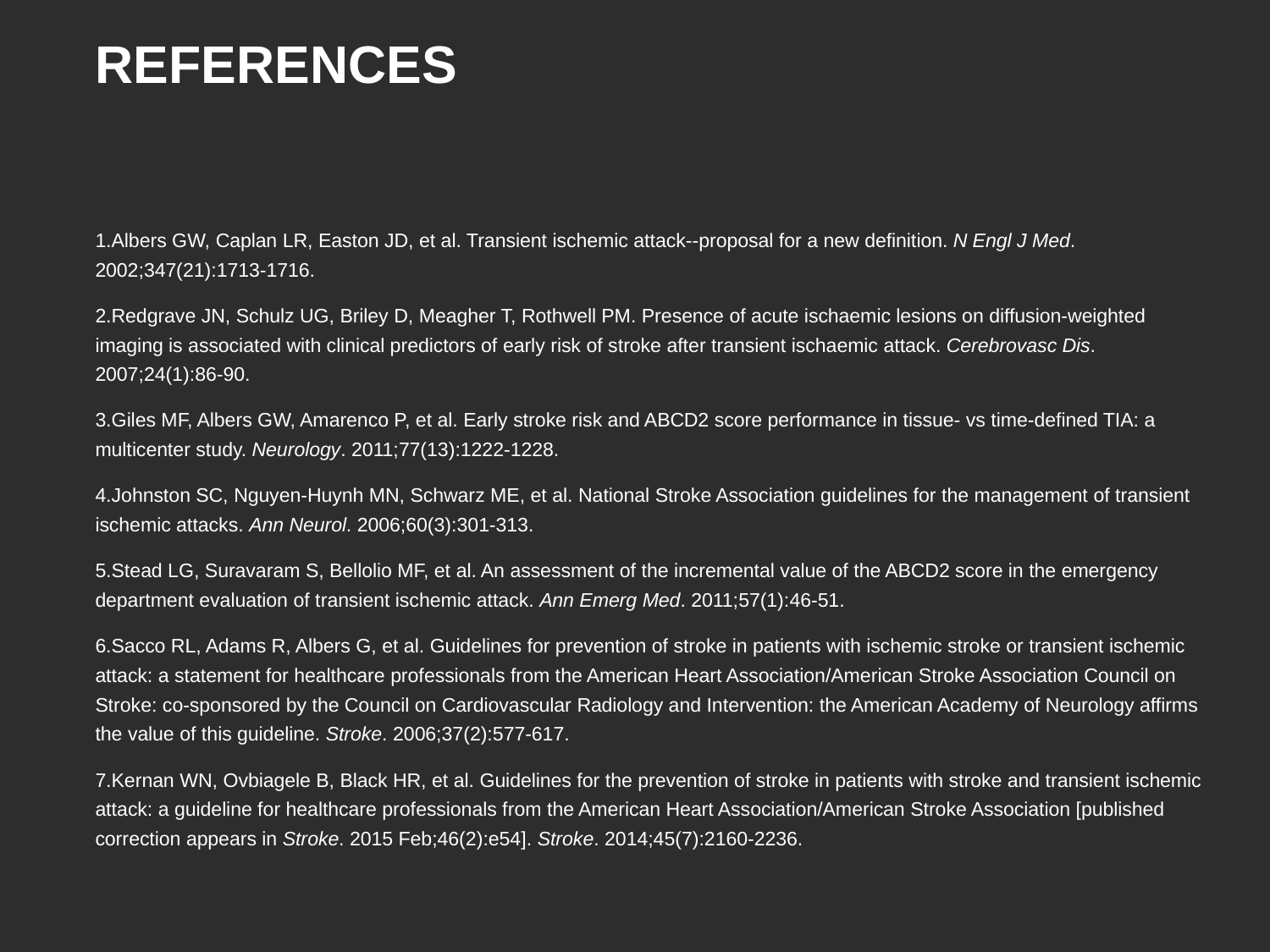

# references
1.Albers GW, Caplan LR, Easton JD, et al. Transient ischemic attack--proposal for a new definition. N Engl J Med. 2002;347(21):1713-1716.
2.Redgrave JN, Schulz UG, Briley D, Meagher T, Rothwell PM. Presence of acute ischaemic lesions on diffusion-weighted imaging is associated with clinical predictors of early risk of stroke after transient ischaemic attack. Cerebrovasc Dis. 2007;24(1):86-90.
3.Giles MF, Albers GW, Amarenco P, et al. Early stroke risk and ABCD2 score performance in tissue- vs time-defined TIA: a multicenter study. Neurology. 2011;77(13):1222-1228.
4.Johnston SC, Nguyen-Huynh MN, Schwarz ME, et al. National Stroke Association guidelines for the management of transient ischemic attacks. Ann Neurol. 2006;60(3):301-313.
5.Stead LG, Suravaram S, Bellolio MF, et al. An assessment of the incremental value of the ABCD2 score in the emergency department evaluation of transient ischemic attack. Ann Emerg Med. 2011;57(1):46-51.
6.Sacco RL, Adams R, Albers G, et al. Guidelines for prevention of stroke in patients with ischemic stroke or transient ischemic attack: a statement for healthcare professionals from the American Heart Association/American Stroke Association Council on Stroke: co-sponsored by the Council on Cardiovascular Radiology and Intervention: the American Academy of Neurology affirms the value of this guideline. Stroke. 2006;37(2):577-617.
7.Kernan WN, Ovbiagele B, Black HR, et al. Guidelines for the prevention of stroke in patients with stroke and transient ischemic attack: a guideline for healthcare professionals from the American Heart Association/American Stroke Association [published correction appears in Stroke. 2015 Feb;46(2):e54]. Stroke. 2014;45(7):2160-2236.
